# Identification and Functional Prediction of Circular RNAs Related to Growth Traits and Skeletal Muscle Development in Duroc pigs

**DOI:** 10.3389/fgene.2022.858763

**Published:** 2022-09-02

**Authors:** Lixia Ma, Wei Chen, Shiyin Li, Ming Qin, Yongqing Zeng

**Affiliations:** ^1^ Shandong Provincial Key Laboratory of Animal Biotechnology and Disease Control and Prevention, College of Animal Science and Technology, Shandong Agricultural University, Tai’an City, China; ^2^ Institute of Animal Science and Veterinary Medicine, Yantai Academy of Agricultural Sciences, Yantai City, China

**Keywords:** circRNAs, skeletal muscle, meat quality, growth traits, Duroc pigs

## Abstract

Porcine skeletal muscle is a highly heterogeneous tissue type, and the Longissimus Dorsi muscle (LDM), as the most economical and physiologically metabolized skeletal muscle in pigs, has always been the focus of research and improvement in pig molecular breeding. Circular RNA, as an important new member of regulatory non-coding RNA after microRNA, has become a frontier hot spot in life science research. This study aims to explore candidate circRNAs related to growth, meat quality, and skeletal muscle development among Duroc pigs with different average daily gain (ADG). Eight pigs were selected and divided into two groups: H group (high-ADG) and L group (low-ADG), followed by RNA-Seq analysis to identify circRNAs. The results showed that backfat at 6-7 rib (BF) and Intramuscular fat (IMF) content in the H group was significantly lower than L group, but ribeye area (REA) in the H group was higher than in the L group. In RNA-seq, 296 Differentially expressed (DE) circRNAs (157 upregulated and 139 downregulated) were identified and exons flanking long introns are easier to circularize to produce circRNAs. Most of the DE circRNAs were enriched in Quantitative trait locus (QTL) regions related to meat quality and growth traits. In addition, a gene can produce one or more circRNA transcripts. It was also found that the source genes of DE circRNAs were enriched in MAPK, FoXO, mTOR, PI3K-Akt, and Wnt signaling pathways. The results showed that different ADG, carcass, and meat quality traits among half-sibling Duroc pigs with the same diet may be due to the DE circRNAs related to skeletal muscle growth and development.

## 1 Introduction

Skeletal muscle is an important tissue that plays a key role in metabolism (Das et al., 2020), abnormal growth of skeletal muscle can cause many diseases, such as Duchenne muscular dystrophy (DMD) and Facioscapulohumeral dystrophy (FSHD) (Birnkrant et al., 2018; Campbell et al., 2018), and it is directly related to growth and meat quality of livestock and poultry (Xu et al., 2017). In recent years, more and more reports have shown that non-coding RNA can regulate skeletal muscle growth and development, including microRNAs (miRNAs), long non-coding RNAs (lncRNAs) and circular RNAs (circRNAs) (Legnini et al., 2017; Li Y. et al., 2018; Yin et al., 2020). In 1976, circRNAs were first discovered in potato tuber (Sanger et al., 1976). Firstly, circRNAs have been regarded as abnormal products because of their low expression abundance, and the fact that it exists only in a few pathogenic bacteria and does not have biological functions (Sanger et al., 1976; Kos et al., 1986). With the rapid development of high-throughput sequencing technology and bioinformatics, more and more studies have found that circRNAs are widely spread in organisms, and their functions are gradually being discovered. Compared with lncRNAs and miRNAs, circRNAs have a closed loop structure without 5′ end caps and 3′ end poly(A) tails, resisting the degradation of RNaseRand, therefore, presenting higher conservation and stability (Hansen et al., 2013; Chen et al., 2015).

Regarding the regulation of circRNAs in muscle development is still in its infancy, current researches on circRNAs in skeletal muscle include identification of circRNAs, analysis of expression patterns, and researches on the regulation mechanism of circRNAs in skeletal muscle development. The current reports found that circRNAs mainly act as molecular “sponges” of miRNAs and participate in the regulation of muscle development. The *circLMO7* regulates myoblasts differentiation and survival by sponging *miR-378a-3p* (Wei et al., 2017). The circRNA *circHIPK3* produced by the third exon of the chicken HIPK3 gene, has the highest expression level compared to other circular RNAs generated from the *HIPK3* gene. It was also differentially expressed in different stages of skeletal development. The *circHIPK3* can act as a sponge of *miR-30a-3p*, and promote cell proliferation and differentiation, binding to *MEF2C*, while *miR-3p* could inhibit the proliferation of Chicken primary myoblasts (CPMs) and repress the differentiation of CPMs by decreasing the expression of *MEF2C* (Chen et al., 2019). In addition, the *circFGFR2* can promote myoblast proliferation by sponging *miR-29b-1-5p* and *miR-133a-5p* (Chen X. et al., 2018). The *circFUT10* can act as a molecular sponge of *miR-133a* and promote cell differentiation, apoptosis and inhibit cell proliferation by upregulating the expression of *miR-133a* target genes (Li H. et al., 2018).

At present, the researches on circRNAs in the muscles of different animal species mainly focus on the comparison between animals with different genetic backgrounds, and there are relatively few comparative studies between half-siblings. This study aims to explore candidate circRNAs related to skeletal muscle growth and development among Duroc pigs with different average daily gain (ADG).

## 2 Materials and Methods

### 2.1 Ethics Statement

All animal care and treatment procedures were conducted in strict accordance with the Animal Ethics Committee of Shandong Agricultural University, China, and performed in accordance with the Committee’s guidelines and regulations (Approval No.: 2004006).

### 2.2 Animals

Duroc pigs come from a core breeding farm. Combined with the existing measurement data, we selected the top 30% (at least 200) of the excellent individuals from the 30–110 kg pigs, and continue the performance measurements until the animals reached 130 kg. According to the average daily gain, eight pigs were selected and divided into two groups: high-average daily gain group (774.89 g) and low-average daily gain group (658.77 g). The Longissimus Dorsi muscle (LDM) tissues were sampled and snap-frozen in liquid nitrogen for extraction of total RNA.

### 2.3 RNA Extraction, Strand-Specific Library Construction and Sequencing

Total RNA was extracted from LDM tissues with Trizol reagent kit (Invitrogen, Carlsbad, CA, United States) according to the instructions. RNA quality was detected on an Agilent 2100 Bioanalyzer (Agilent Technologies, Palo Alto, CA, United States), and checked with RNase free agarose gel electrophoresis. Then total RNA was treated with RNase R to degrade the linear RNAs, and purified with the RNeasy MinElute Cleanup kit (Qiagen, Venlo, Netherlands). Strand-specific library was constructed with VAHTS Total RNAseq (H/M/R) Library Prep Kit (Vazyme, Nanjing, China) for Illumina. In a word, ribosome RNAs were removed to retain circRNAs.

The enriched circRNAs were fragmented into short fragments with fragmentation buffer and transcribed into cDNA with random primers. Second-strand were synthesized with DNA polymerase I, RNase H, dUTP and buffer. And then, the cDNA fragments were purified with VAHTSTM DNA Clean Beads (Vazyme, Nanjing, China), end repaired, poly(A) added, and ligated to Illumina sequencing adapters. Then Uracil-N-Glycosylase (UNG) was used to digest the second-strand cDNA. The digested products were purified with VAHTSTM DNA Clean Beads, PCR amplified, and sequenced by Illumina Novaseq60000 by Gene *Denovo* Biotechnology Co. (Guangzhou, China).

### 2.4 Quality Control for Raw Reads (Raw Datas) and Mapping

Raw reads contained adapters or low-quality reads. Thus, raw reads were further filtered by fastp (Chen S. et al., 2018) (version 0.18.0, parameter settings: -a AGATCGGAAGAGC -q 20 -u 50 -n 15 -l 50 -w 1) to get high quality clean reads. The quality control standards were as follows: 1) removing adapters reads; 2) removing reads containing more than 10% of unknown nucleotides (N); 3) removing low quality reads containing more than 50% of low quality (Q-value≤20) bases. Bowtie2 (Langmead and Salzberg., 2012) (version 2.2.8, parameter settings: -local -p 4 --mm) software was used for mapping reads to ribosome RNA (rRNA) database. The rRNA removed reads of each sample were then mapped to reference genome by TopHat2 (Kim et al., 2015) (version 2.1.1, parameter settings: -rna- strandness RF -p 4 -q -t --dta --new-summary --mm), respectively.

### 2.5 Identification of circRNAs and circRNAs Statistics

The 20mers from both ends of the unmapped reads were extracted and aligned to the reference genome to find unique anchor positions within splice site. Anchor reads that aligned in the reversed orientation (head-to-tail) indicated circRNA splicing and then were subjected to find_circ (Memczak et al., 2013) (version 1, parameter settings: -r <sample.txt> -G <genome.fa> -p novel_ -s <stats.file>) to identify circRNAs. The anchor alignments were then extended such that the complete read aligns and the breakpoints were flanked by GU/AG splice sites. A candidate circRNA was called if it was supported by at least two unique back spliced reads at least in one sample.

The identified circRNAs were analyzed including type, chromosome distribution and length distribution by find_circ (version 1) and the annotation information of the reference genome.

### 2.6 Analysis of differentially expressed circRNAs

Differentially expressed (DE) circRNAs across group were identified by edgeR package (version 3.12.1) (http://www.r-project.org/) (Robinson et al., 2010). Identified DE circRNAs with an absolute fold change ≥1.5 and a *p* value < 0.05 were considered significant DE circRNAs.

### 2.7 Functional Enrichment Analysis of Source Gene

Source gene is the origin gene of a circRNA, and the functional analysis of source genes were studied to know about the main functions of circRNAs source genes.

#### 2.7.1 GO Enrichment Analysis

Gene Ontology (GO) is an international standardized gene functional classification system, which includes three parts: cellular component, biological process and molecular function. On the one hand, the GO function analysis includes the GO function classification annotation of genes; on the other hand, it includes GO functional significant enrichment analysis of genes. Firstly, all source genes were mapped to GO terms in the Gene Ontology database (http://www.geneontology.org/), gene numbers were calculated for every term, significantly enriched GO terms in source genes comparing to the genome background were defined by hypergeometric test. The calculating formula of *p*-value is as follows:

$$P=1-\sum_{i=0}^{m-1}\frac{\left(\begin{array}{c} M\\ i \end{array}\right)\left(\begin{array}{c} N-M\\ n-i \end{array}\right)}{\left(\begin{array}{c} N\\ n \end{array}\right)}$$

N: the number of all genes with GO annotation; n: the number of source genes in N; M: the number of all genes that are annotated to the certain GO terms; m: the number of source genes in M. The calculated *p*-value were gone through FDR Correction, taking FDR ≤ 0.05 as a threshold. GO terms meeting this condition were defined as significantly enriched GO terms in source genes.

#### 2.7.2 KEGG enrichment analysis

The analysis of Pathway helps to further know about the biological functions of genes. KEGG is the main public database about Pathway (Kanehisa et al., 2008). Pathway enrichment analysis identified significantly enriched metabolic pathways or signal transduction pathways in source genes comparing with the whole genome background. The calculating formula is the same as that in GO analysis:

$$P=1-\sum_{i=0}^{m-1}\frac{\left(\begin{array}{c} M\\ i \end{array}\right)\left(\begin{array}{c} N-M\\ n-i \end{array}\right)}{\left(\begin{array}{c} N\\ n \end{array}\right)}$$

N: the number of all genes that with KEGG annotation; n: the nuber of source genes in N; M: the number all genes annotated to specific pathways; m: the number of source genes in M. The calculated *p*-value was gone through FDR Correction, taking FDR ≤ 0.05 as a threshold. Pathways meeting this condition were defined as significantly enriched pathways in source genes.

### 2.8 Quantitative trait locus analysis

Basic Local Alignment Search Tool (blast) comparison was performed on the QTL locus (<2Mb) with high confidence related to pig growth and meat quality traits. If the transcript or QTL interval is more than 50% duplicated, the transcript is located on this QTL. Genome information can be downloaded from AnimalQTLdb (PigQTLdb: http://www.animalgenome.org-/QTLdb/pig.html) Database.

### 2.9 Target Relationship Prediction

For the circRNAs included in the circBase database, the StarBase (Li et al., 2014) database provides the targeting relationship between circRNAs and miRNAs. Therefore, the target relationship between existing circRNAs and miRNAs can be found through the StarBase database. There may be some unreported targeting relationships between all circRNAs and all miRNAs of pigs. Therefore, the analysis will also predict the target relationship between all circRNAs and miRNAs of pigs. RNAhybrid + svm_light, Miranda, and TargetScan were used to predict the target relationship (Qiao et al., 2016).

### 2.10 Quantitative real-time PCR analysis

The total RNA was reverse-transcribed into cDNA by PrimeScript RT reagent kit (TaKaRa, Dalian, China), and the analyzed by SYBR® Green Pro Taq HS Premix (Accurate Biotechnology (Hunan) Co., Ltd., Changsha, China). Primers were compounded from Sangon Biotech (Shanghai, China), and sequences of primers are shown in Table 1. β-actin was used as a housekeeping gene. The fold change in expression was the obtained by 2^−ΔΔCT^ method, ΔΔCT= (CT _Target gene_ - CT_β-actin_) _H group_ - (CT _Target gene_ - CT_β-actin_) _L group_.

**TABLE 1:** Primers sequences.

Gene	Sequence (5′-3′)
novel_circ_008472-F	CAC​CAG​ATG​CCT​ACT​CTG​TTA​CTT
novel_circ_008472-R	GCC​TTT​GTT​GCT​CCT​TCT​ATG​ATC
novel_circ_010066-F	GGA​TCA​AAC​CCC​ACT​GGA​CAT
novel_circ_010066-R	AGT​ACA​TTG​TGC​CTG​GTA​GAT​TCA
novel_circ_008433-F	AGA​CAT​GCA​CAT​CCA​GAT​CAC​AGA
novel_circ_008433-R	CAG​GAA​CAC​AAC​ACC​ACG​CTG
novel_circ_005672-F	GTG​TCG​GGA​AGG​TGA​ACT​TGT
novel_circ_005672-R	TCT​GAG​GTT​TCT​TGT​GCT​CTT​GG
novel_circ_012322-F	TGC​CAG​AAG​GTC​TTG​CCA​TAG
novel_circ_012322-R	GGC​TAC​ATT​CAG​ACA​CAT​TGC​TT

### 2.11 Meat Quality and Carcass Traits Description

Backfat at 6-7 rib (BF): hang the right carcass upside down and measure the subcutaneous fat thickness at the 6-7 rib of the carcass with a vernier caliper. unit: mm.

Ribeye area (REA): The cross-sectional area of the Longissimus Dorsi at the junction of the thoracic and lumbar vertebrae. The carcass was laid flat and the height and width of the cross-section area were measured by vernier calipers. The formula was as follows:

$$\mathrm{REA}\;\left(cm^{2}\right)\;=\;\mathrm{height}\;\left(\mathrm{cm}\right)\;\times \;\mathrm{width}\;\left(\mathrm{cm}\right)\;\times \;0.7$$

Detailed experimental methods for Intramuscular Fat (IMF) analysis are described in-depth by Holman et al. (Holman et al., 2019).

## 3 Results

### 3.1 Meat Quality and Carcass Traits Description

As shown in Table 2, there was no difference in carcass weight concerning the two groups of AVG (H and L). For BF, the H group was significantly lower than the L group (*p* < 0.05), and for REA, the H group was higher than the L group (*p* < 0.05). In addition, IMF content in H group was significantly lower than L group.

**TABLE 2:** Meat quality and carcass traits description.

Traits	H group	L group
Carcass weight/ kg	97.57 ± 1.33	96.45 ± 2.47
BF/ mm	30.66 ± 3.22^b^	35.68 ± 4.23^a^
REA/ cm^2^	59.86 ± 3.61^a^	50.42 ± 1.61^b^
IMF content/%	4.30 ± 0.27 ^b^	4.80 ± 0.08^a^

Note: ^a, b^ Those with the different letters in each line showed significant difference (*p* < 0.05). H group represents High-average daily gain (ADG) group, L group represents L-average daily gain (ADG) group. BF, represents backfat at 6-7 rib, REA, represents ribeye area, and IMF, represents Intramuscular fat.

### 3.2 Overview of Sequencing Data and Quality Assessment

The eight libraries in this study were filtered to remove the reads containing adapters (about 0.19%) and low-quality reads (about 1.13%). Finally, about 98.68% of the raw data reached the quality control standard as clean data (Supplementary Table S1). Through ribosome alignment, it was found that only 52.41% (49.75%–55.05%) of the data in the eight samples were available for subsequent transcriptome analysis (Supplementary Table S2). With the alignment in the reference Sus scrofa genome (Ensembl-release104), on average about 84.10% of the clean reads were mapped to the reference, about 77.60% of them were considered Unique Mapped reads, and only 6.5% were Multiple Mapped reads (Supplementary Table S3).

### 3.3 Identification of circRNAs

**FIGURE 1:**
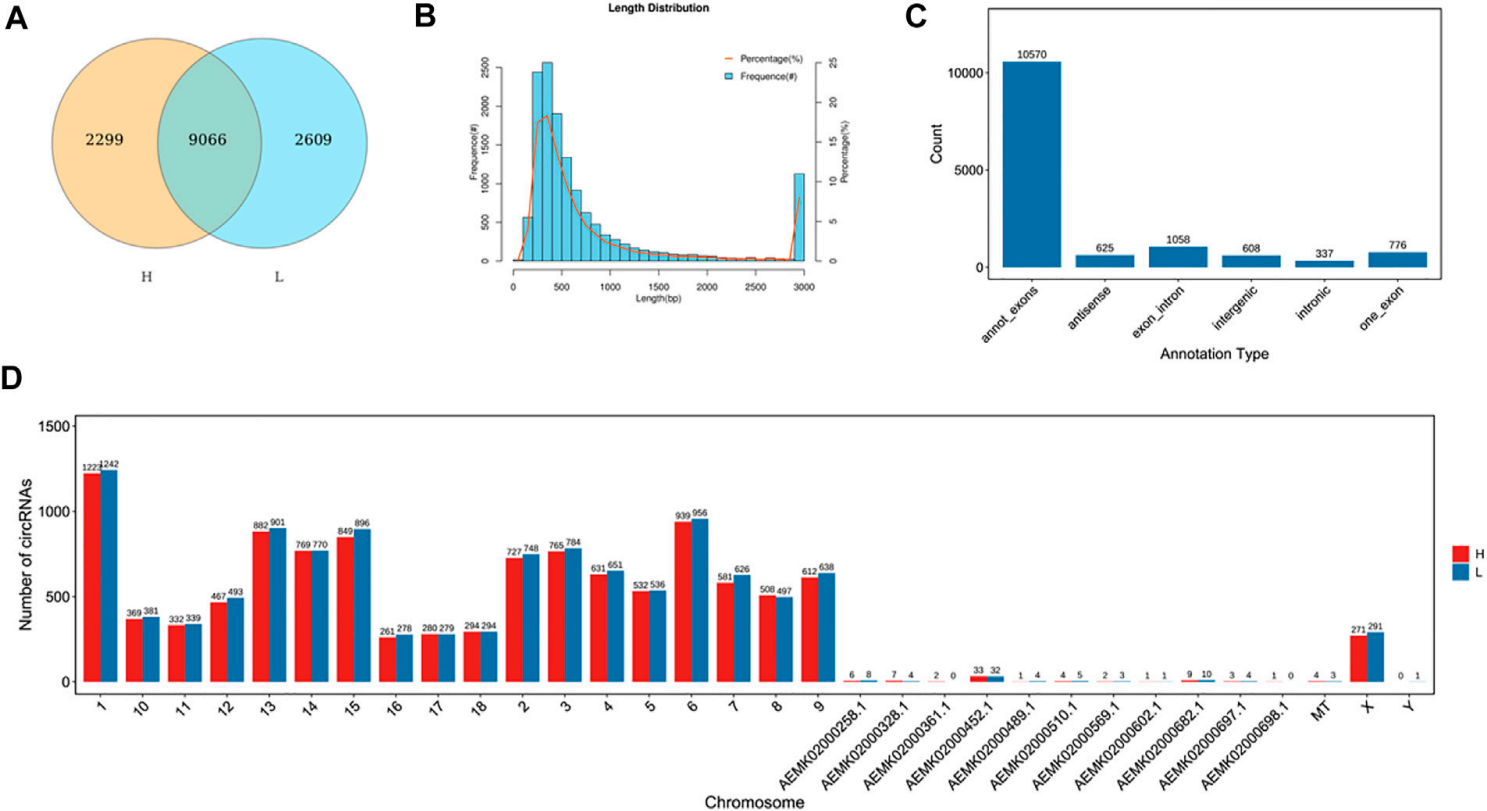
Identification of circRNAs. **(A)** The number of identified circRNAs. Yellow represents H group, blue represents L group. The overlap represents the number of genes shared between the two groups, and non-overlap represents genes unique to each group. **(B)** The length of circRNAs. The abscissa represents the length of circRNAs, the ordinate represents frequency. **(C)** The location distribution of circRNAs. The abscissa represents the annotation type, the ordinate represents the number of circRNAs. **(D)** The chromosome distribution of identified lncRNAs. The abscissa represents the chromosome, the ordinate represents the number of circRNAs. Red represents H group, blue represents L group.

To identify circRNAs related to skeletal muscle development, we identified the expression of circRNAs by removing the ribosomal RNA (rRNA) RNA-seq. A total of 13974 circRNAs were identified (Supplementary Table S10), and 9066 circRNAs were co-expressed in the two groups (Figure 1A). The length of circRNAs was less than 1600bp and most of the circRNAs were from exon regions (Figures 1B,C), in addition, circRNAs were distributed on every chromosome (Figure 1D).

### 3.4 Analysis of Genomic Characteristics on circRNAs

**FIGURE 2:**
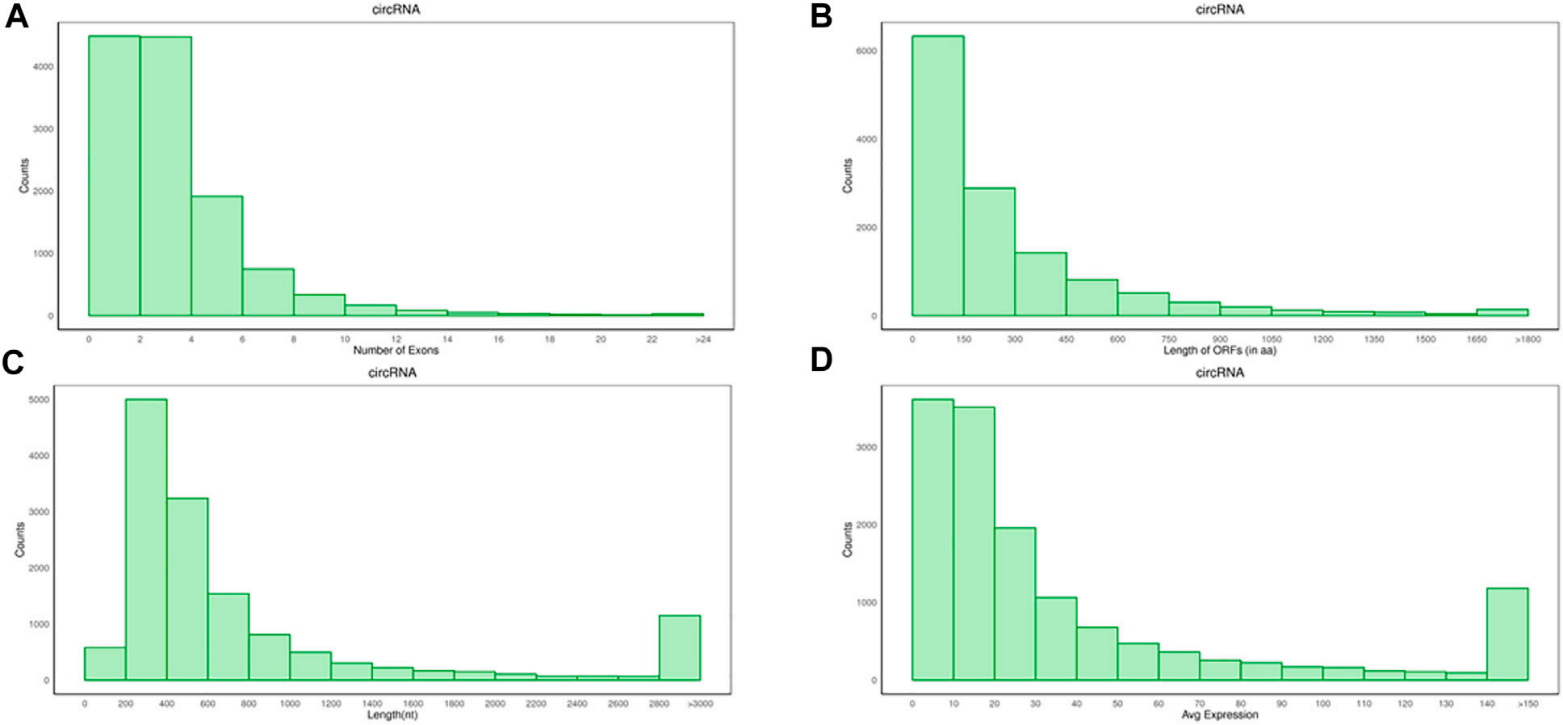
Analysis of genomic characteristics on circRNAs. **(A)** Number of circRNAs exons. **(B)** Length of circRNAs ORF. **(C)** Transcript length of circRNAs. **(D)** The expression levels of circRNAs.

The average number of circRNAs exon was 3.91, the average length of the open reading frame (ORF) was 23 amino acids (aa), and the length of the transcript was 1665.73nt (Figures 2A–C), which were lower than mRNA, but the expression levels of circRNAs were higher than mRNAs.

### 3.5 Analysis of Source Genes and Molecular Characteristics of circRNAs

**FIGURE 3:**
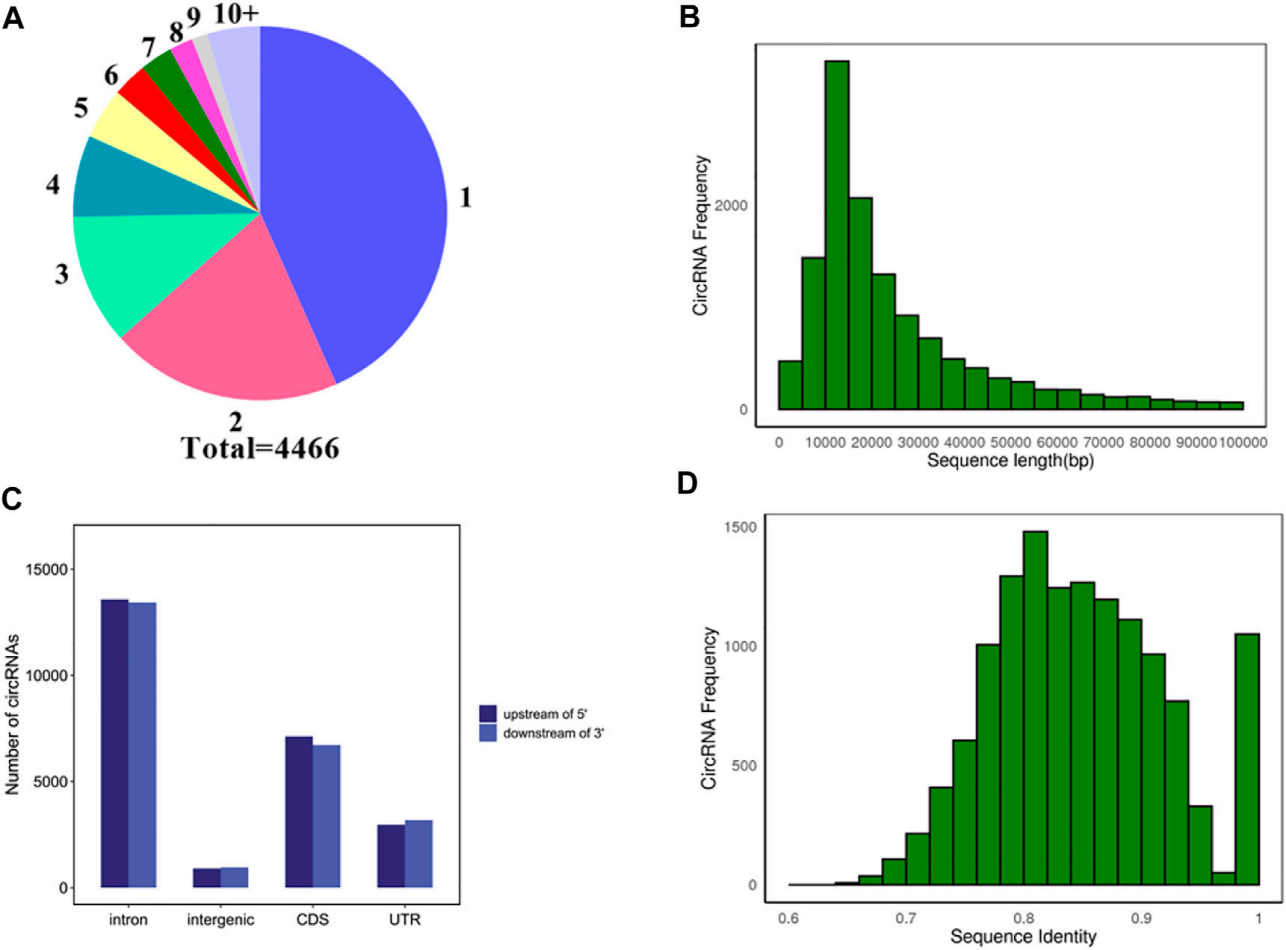
Analysis of source genes and molecular characteristics of circRNAs. **(A)** Statistics of source genes. Different numbers represent different numbers of circRNA transcripts. 1, 2, 3…10+ represent one, two, three…and 10+ circRNA transcripts, respectively. Different colors represent the different number of source genes. For instance, blue represents the number of source genes that produce one circRNA transcript. Total 4,466 represents a total of 4,466 source genes. **(B)** Distribution of flanking intron sequences lengths of circRNAs. **(C)** Flanking sequences of a circRNA 5′ end upstream and 3′ end downstream. **(D)** Distribution of sequences identified on each intron pair that are flanking cricRNAs.

To explore the source of circRNA transcription, the source genes of 13974 circRNAs transcripts were analyzed. As shown in Figure 3A, a total of 608 circRNAs did not detect their source genes, and the remaining 13366 circRNAs transcripts were generated from 4,466 genes. The majority of genes 1935) produce one circRNA transcript, and only 265 genes could produce ten or more circRNA transcripts.

To explore the molecular characteristics of circRNAs, the 5 kb flanking sequence of pig circRNAs upstream and downstream was analyzed. The results showed that most of the flanking sequences of circularized exons were introns. About 55.25% of 5′ sequences upstream of circRNAs contained introns, and 55.28% of 3′ sequences downstream of circRNAs contained introns. In addition, 29% of 5′ flanking sequences and 27.66% of the 3′ flanking sequences contained CDS (Coding Sequence), respectively, and the remaining flanking sequences were intergenic and UTR (Untranslated Region) (Figure 3C). Statistics on the length of introns in the flanking sequences of circRNA found that most of the introns in the flanking sequence were longer than 5000 bp, indicating that the flanking sequence of pig circRNAs contains longer introns (Figure 3B). The basic Local Alignment Search Tool (BLAST) was used to align the introns of each intron pair that flanked circRNAs. Most of circRNAs shared reverse complementary matches (RCMs) of at least 200 bp (Figure 3D).

**FIGURE 4:**
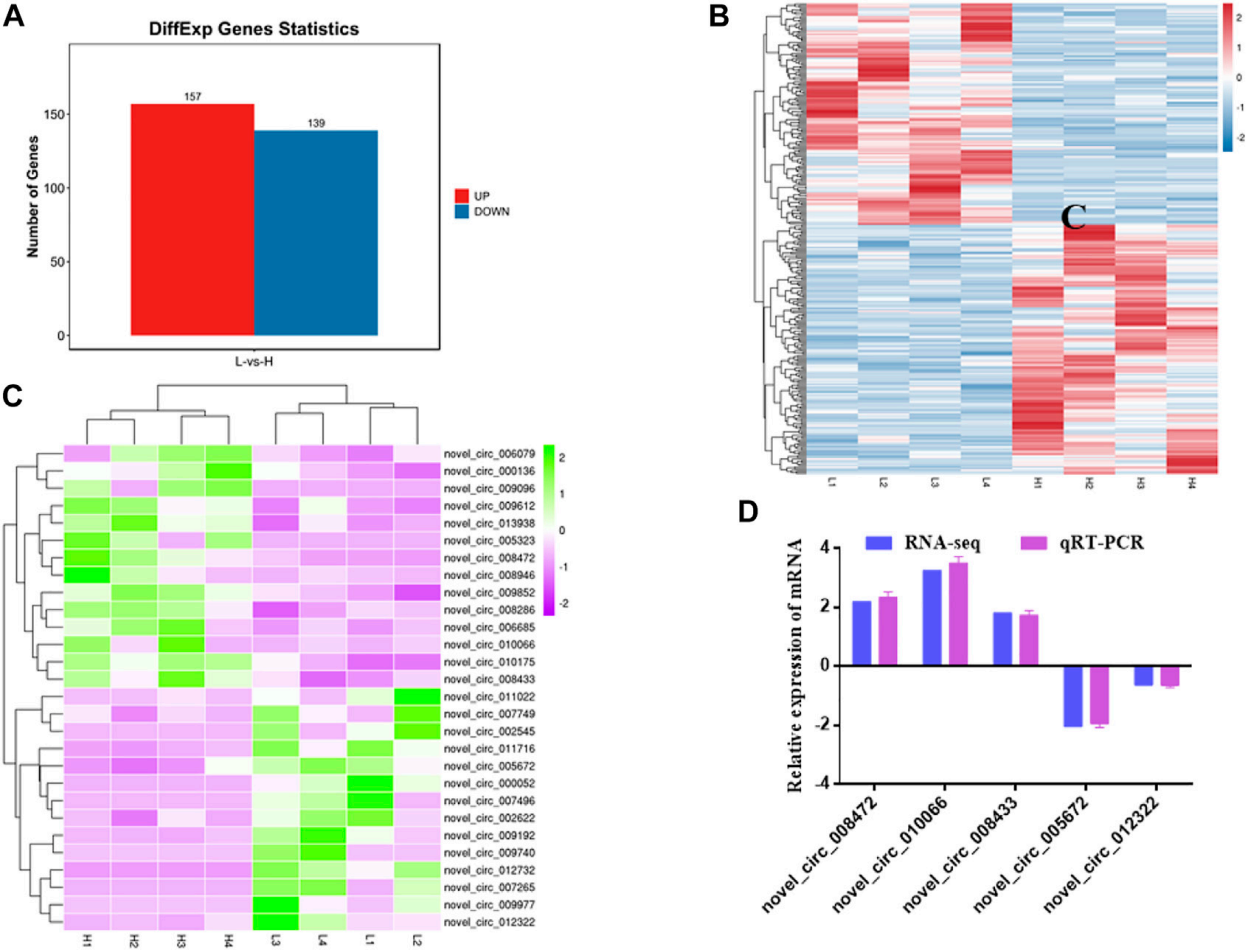
Statistics and heat map analyses of DE circRNAs. **(A)** Statistics of DE circRNAs. Red represents up-regulation, blue represents down-regulation. **(B)** Heat map analysis of DE circRNAs. Each column represents a sample and each row represents a circRNA. Red and blue gradients indicate increase and decrease in gene expression abundance, respectively. **(C)** Heat map analysis of DE circRNAs related to skeletal muscle development. Each column represents a sample and each row represents a circRNA. Purple and green gradients indicate increase and decrease in gene expression abundance, respectively. **(D)** Validation of DE circRNAs. Blue represents RNA-seq result, purple represents qRT-PCR result.

In addition, 296 DE circRNAs were identified, including 157 upregulated circRNAs and 139 downregulated circRNAs (Figures 4A,B, Supplementary Table S4). Among them, 28 circRNAs related to muscle development were identified (14 upregulated and 14 downregulated) (Figure 4C). Hierarchical clustering showed that the expressions of circRNAs were distinguishable between H group and L group, indicating a significant difference between H group and L group.

To verify RNA-seq results, five DE circRNAs were selected to perform by qRT-PCR. The results showed that qRT-PCR results were in agreement with those in RNA-seq (Figure 4D). The results indicated that circRNAs identified from RNA-seq were reliable.

### 3.6 GO and KEGG Analysis of Source Genes of DE circRNAs

**FIGURE 5:**
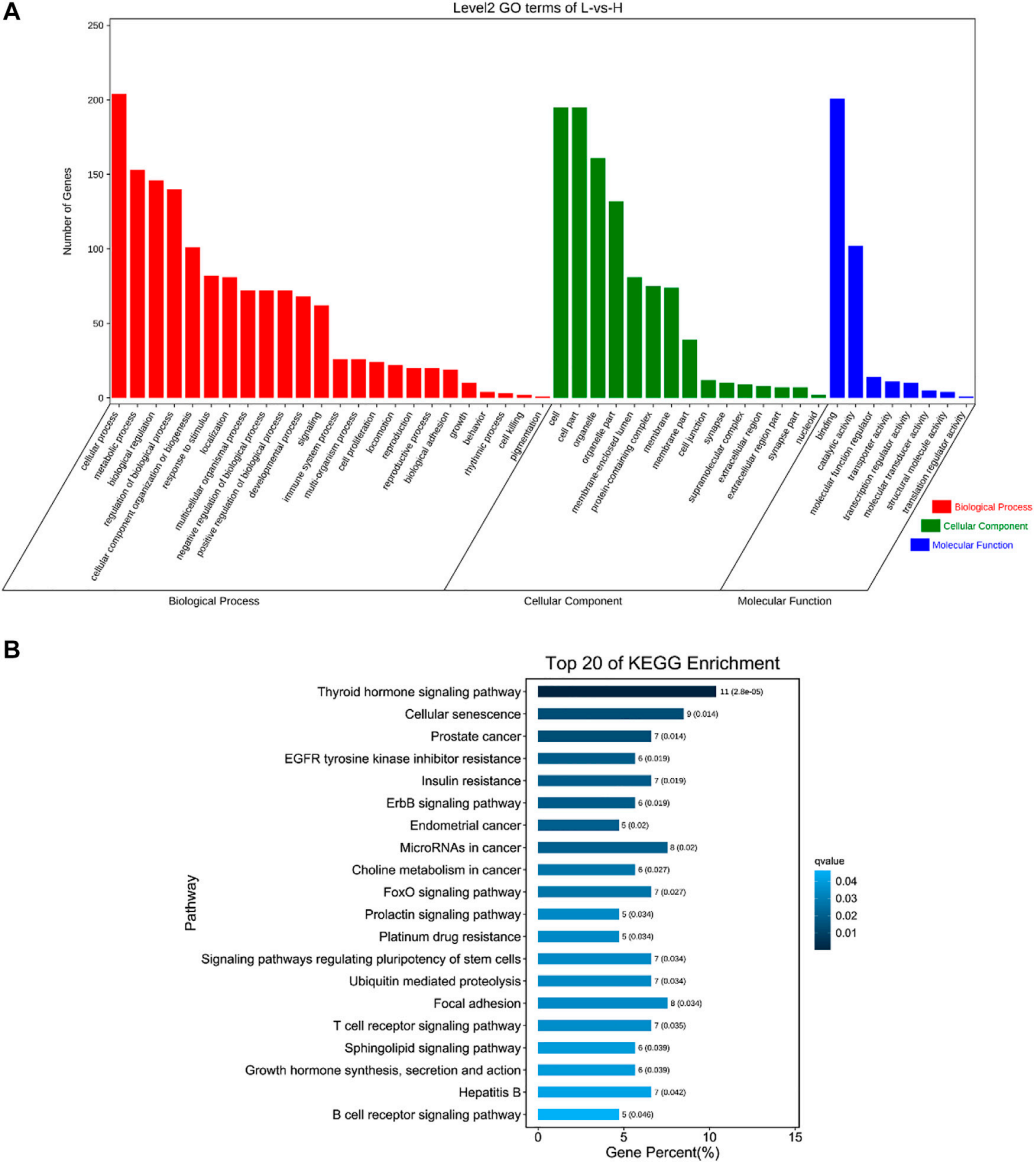
GO and KEGG analysis of source genes of DE circRNAs. **(A)** GO enrichment. The abscissa is the secondary GO term, and the ordinate is the number of genes in this term. Different colors show different types of GO terms, red represents Biological Process, green represents Cellular Component, blue represents Molecular Function. **(B)** KEGG enrichment. Using the top 20 pathways with the smallest Q value to make a map, the ordinate is the pathway, the abscissa is the enrichment factor, the size of the column indicates the number of genes, the bluer the color, the smaller the Q value.

To understand the functions of DE circRNAs, GO and KEGG analysis were performed. The results showed that the source genes of DE circRNAs were significantly enriched in regulation of DNA recombination, blood vessel development, response to oxidative stress, mitotic spindle organization, programmed cell death and other GO terms (Figure 5A, Supplementary Table S5). The source genes of DE circRNAs were significantly enriched in pathways including MAPK, FoXO, mTOR, PI3K-Akt and Wnt signaling pathways (Figure 5B, Supplementary Table S6).

**FIGURE 6:**
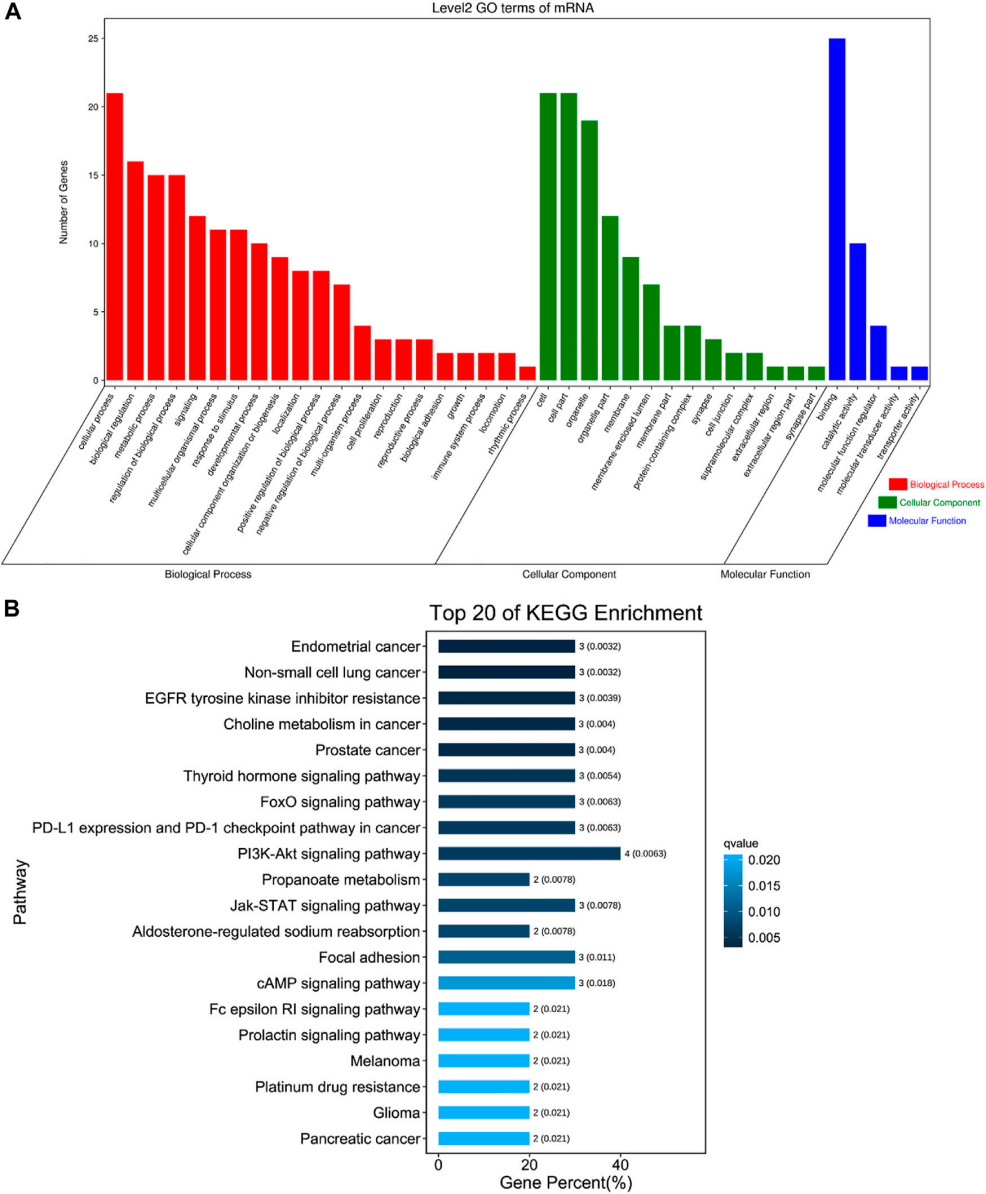
The first 20 GO terms and KEGG. **(A)** The first 20 GO terms of DE circRNAs related to skeletal muscle. The abscissa is the secondary GO term, and the ordinate is the number of genes in this term. Different colors show different types of GO terms, red represents Biological Process, green represents Cellular Component, blue represents Molecular Function. **(B)** The first 20 KEGG of DE circRNAs related to skeletal muscle. Using the top 20 pathways with the smallest Q value to make a map, the ordinate is the pathway, the abscissa is the enrichment factor, the size of the column indicates the number of genes, the bluer the color, the smaller the Q value.

The DE circRNAs related to muscle development were significantly enriched in regulation of endothelial cell proliferation, calcium ion transport, regulation of skeletal muscle contraction by calcium ion signaling, transition between fast and slow fiber, myofibril assembly, muscle cell differentiation and other GO terms (Figure 6A, Supplementary Table S7), and they were also enriched in Pathways in cancer, EGFR tyrosine kinase inhibitor resistance, PPAR signaling pathway, Insulin signaling pathway, cAMP signaling pathway, FoxO signaling pathway, PI3K-Akt signaling pathway, AMPK signaling pathway, Jak-STAT signaling pathway and other pathways (Figure 6B, Supplementary Table S8).

### 3.7 Expression Analysis of Growth Traits and Meat Quality-Relevant QTLs

According to the QTL mapping analysis, 256 DE circRNAs source genes were enriched in QTLs related to growth and meat quality in pigs (Table 3, Supplementary Table S9). The results showed that DE circRNAs were closely related to the growth traits and meat quality. For example, circRNA ENSSSCG00000049158 was enriched in Drip loss QTL, Water holding capacity QTL, Average daily gain QTL and other QTLs. circRNA ENSSSCG00000004203 was enriched in Backfat at last rib QTL, Shoulder subcutaneous fat thickness QTL, Backfat at last rib QTL, Percentage type IIa fibers QTL, PH for Longissimus Dorsi QTL and Leaf fat weight QTL.

**TABLE 3:** Target gene of DE circRNAs distribution in chromosome and QTLs region.

Chromosome/Mitochondrion	DE gene number	DE gene number in QTL region	QTL region length(Mb)
1	17	16	272.538
2	17	17	152.419
3	14	13	129.972
4	12	11	126.574
5	14	13	98.508
6	20	20	146.584
7	15	15	126.113
8	19	19	143.374
9	12	12	138.044
10	7	7	67.1919
11	6	5	77.1184
12	10	10	58.0079
13	32	32	208.259
14	22	22	154.672
15	16	16	137.024
16	6	6	66.4773
17	10	9	52.1699
18	8	8	58.1156
X	6	5	83.7497
Y	0	0	0
MT	0	0	0

Note: Chromosome/Mitochondrion represents Chromosome number. DE, gene number represents differentially expressed genes in this Chromosome; DE, gene number in QTL, region represents differentially expressed genes in QTL, region.

### 3.8 Analysis of Target Relationship Prediction

In this study, 252534 miRNA-circRNA pairs were identified between 453 miRNAs and 13836 circRNAs.

## 4 Discussion

Average daily gain (ADG) is considered an important growth trait, it can directly affect the economic benefits of the pig producers. Growing fast and having a high lean meat rate is the best ideal state. In this study, the results showed that H group not only grew faster than L group, but also had less BF and IMF content than L group. Therefore, pigs in H group will be preferred by more pig producers. To explore the phenotype differences between the H and L groups, RNA-seq was used to identify circRNAs.

Due to the imperfect methods of circRNAs research and identification, the functional identification of circRNAs is still in its infancy. In recent years, the development of sequencing technology and bioinformatics have provided technical support for the study of circRNAs from the whole genome.

Genome-wide methods for circRNAs identification mainly include gene chip technology and RNA-seq technology. RNA-seq is widely used in circRNAs research in both model organisms and non-model organisms. At present, RNA-seq has been used in many species, such as mice (Jakobi et al., 2016), cattle (Wei et al., 2017), pigs (Liang et al., 2017) and sheep (Li et al., 2017), with high reliability and feasibility.

Firstly, to deeply understand the genomic characteristics of circRNAs, 13974 circRNA transcripts were analyzed. A total of 13366 circRNA transcripts were generated from 4,466 genes, and most genes can produce multiple circRNA transcripts. In previous research, 5,934 circRNAs were identified on different muscles in pigs, and 4,928 circRNAs corresponded to 2358 source genes, in addition, 85.7% of the source genes were transcribed to generate two or more circRNA transcripts (Liang et al., 2017). According to the source gene location, 13950 circRNAs were distributed on all chromosomes, and most of circRNAs contained 1-3 exons (Tao et al., 2017). The genome characteristics of identified circRNAs in this study were consistent with other researches, which further illustrated accuracy and reliability of the identified circRNAs, and also showed that circRNAs had similarities among various species.

The conservation of circRNA sequences among species indicates that the formation mechanism of circRNAs may also have inter-species similarities, studies have shown that exons flanking long introns are easier to circularize to produce circRNAs, and the reverse complementary sequence of flanking introns promotes the reverse splicing of circRNAs (Ashwal-Fluss et al., 2014; Westholm et al., 2014). Our findings were consistent with literature reports.

At present, the functions of circRNAs are mainly annotated by GO and KEGG analysis of circRNA source genes. Through enrichment analysis of DE circRNA (including related to muscle development) source genes, we found that the source genes were significantly enriched in histone methylation, programmed cell death, developmental growth, cytoskeletal protein binding and other GO terms. The significantly enriched pathways included Apoptosis, Regulation of actin cytoskeleton, FoxO signaling pathway, mTOR signaling pathway, PI3K−Akt signaling pathway, Wnt signaling pathway, Hedgehog signaling pathway and other pathways related to growth traits and skeletal muscle growth and development. Previous studies have found that the source genes of DE circRNAs were enriched in pathways related to skeletal muscle development, such as JAK-STAT, PI3K-Akt, Wnt and the transition between fast and slow fibers signaling pathways (Wang J. et al., 2019; Huang et al., 2021). The *Map3k20* gene is one of the upstream factors regulating the JNK/MAPK signaling pathway. The *circFgfr2* regulated Map3k20 expression by sponging *miR-133*, *circFgfr2* can regulate skeletal muscle development via the JNK/MAPK signaling pathway (Yan et al., 2021). Mechanistically, *circCCDC91* could absorb *miR-15a*, *miR-15b-5p*, and *miR-15c-5p* to regulate the expression of Insulin receptor substrate1 (*IRS1*), as well as activate insulin-1ike growth factor 1-phosphatidylinositol 3-kinase/AKT (IGF1-PI3K/AKT) signaling pathway. In addition, *circCCDC91* could rescue skeletal muscle atrophy by activating IGF1-PI3K/AKT pathway (Zhao et al., 2022). The *circPPP1R13B* promotes chicken SMSC proliferation and differentiation by targeting miR-9-5p and activating IGF/PI3K/AKT signaling pathway. The results showed that the DE circRNAs play an important role in meat quality and skeletal muscle development, and can be used as candidate functional circRNAs related to skeletal muscle.

circRNAs are found in almost all cells and tissues. At the same time, circRNAs also have a certain degree of sequence conservation (higher sequence conservation than lncRNAs), and have the miRNA response elements (MREs), therefore, circRNAs can be competing endogenous RNAs (ceRNAs) for regulating miRNAs (Song and Li., 2018). In this study, it was found that circRNAs have one or more binding sites for miRNAs. Previous research showed that the *ciRS-7* has multiple binding site for *miR-7*, thereby inhibiting *miR-7* activity and leading to upregulation of *miR-7* target gene expression (Hansen et al., 2013). In addition, many reports have demonstrated that circRNAs can inhibit the binding of miRNAs to target genes, thereby regulating cell growth and development. For instance, the *circZfp609* acts as a sponge of *miR-194-5p* to increase the expression of *BCLAF1* and inhibit myoblast differentiation (Wang Y. et al, 2019). The *circSVIL* can promote cell proliferation and differentiation by sponging *miR-203* and promoting the expression of *MEF2C* and *c-JUN* in chicken (Ouyang et al., 2018a). The *circRBFOX2* interacts with *miR-206* to increase the expression of *cyclin D2* and thereby promotes cell proliferation in chickens (Ouyang et al., 2018b).

In recent years, due to the extensive development of QTL research, a large number of QTLs have been discovered in pigs. Taking the QTL mapping study of domestic pigs in the AnimalQTLdb database as an example, more than 20,000 QTLs have been identified, involving more than 600 different traits. The QTLs mapping studies of other livestock and poultry, such as cattle, chickens, and horses, are also very common (Legarra et al., 2015). How to integrate this large amount of information, compare and locate, and finally determine the genetic basis of candidate genes and traits is a key issue in applying these localization results. Therefore, the research on QTL has become a new hot issue.

Some studies have shown that muscle fibers were related to meat quality and growth development (Lee et al., 2010). In QTL analysis, it was found that most DE circRNAs were related to meat quality and growth traits. In other research, 54.01% of DE circRNAs were enriched in QTL regions related to meat quality and growth traits, such as the *circRNA227* was enriched in the Intramuscular fat content QTL and Loin muscle area QTL and *circRNA11553* was enriched in the Mean corpuscular hemoglobin content QTL (Shen et al., 2019). But compared with QTL, the number of circRNAs that control muscle traits in pigs is few. However, in the coming decades, the development of bioinformatics, genetics, and transgenic technology will greatly increase the number of circRNAs controlling muscle traits in pigs. This will facilitate the discovery of functional candidate circRNAs. The results of QTL analysis may provide some data support for the discovery of new functions of circRNAs.

circRNAs are involved in many physiological and pathological processes, including cancer, aging, and muscle development, but their specific functions are still unclear. Thus, our results could help to understand the genetic mechanisms in meat quality in livestock and poultry, and further studies are necessary for a better comprehension of circRNAs functions in skeletal muscle development.

## 5 Conclusion

In conclusion, 296 DE circRNAs were identified. Most DE circRNAs were enriched in QTLs related to meat quality and growth traits. In addition, the source genes of DE circRNAs were enriched in pathways related to cell growth and skeletal muscle development. Therefore, these DE circRNAs may play a key role in meat quality and growth traits. Researches on circRNAs in skeletal muscle development and disease are still in its infancy, and skeletal muscle plays a key role in body development. How to use these circRNAs and apply them to livestock breeding and disease treatment remains a challenge. Therefore, the understanding of the complex molecular mechanism of circRNAs requires further study.

## Data Availability

The datasets presented in this study can be found in online repositories. The names of the repository/repositories and accession number(s) can be found below: https://www.ncbi.nlm.nih.gov/bioproject/, PRJNA812354.

## Fundings

This study was supported financially by National Key R&D Program of China (No. 2021YFD1301200), the Agricultural Animal Breeding Project of Shandong Province (No. 2020LZGC012), Shandong Province Pig Industry Technology System Project (No. SDAIT-08–02), Shandong Provincial Natural Science Foundation (No. ZR2019MC053).
