# Supplementary material for: Identification and Functional Prediction of Circular RNAs Related to Growth Traits and Skeletal Muscle Development in Duroc pigs
Source: Front Genet. 2022 Sep 2;13:858763. doi: 10.3389/fgene.2022.858763 (PMC9478749; doi:10.3389/fgene.2022.858763)
Supplement: Supplementary file 1 [file Table1.DOCX]

Supplementary Table 1 Statistics for filtering reads

| Sample | Raw Reads^a^ | Clean Reads^b^ | Adapter^c^ | Low quality^d^ | polyA^e^ | N^f^ |
| --- | --- | --- | --- | --- | --- | --- |
| H1 | 115340660 | 113682224 | 212138 | 1446298 | 0 | 0 |
| H2 | 129492024 | 127710488 | 254268 | 1527268 | 0 | 0 |
| H3 | 112871496 | 111321746 | 204364 | 1345386 | 0 | 0 |
| H4 | 117494942 | 115842072 | 228258 | 1424612 | 0 | 0 |
| L1 | 123083610 | 121717410 | 222712 | 1143488 | 0 | 0 |
| L2 | 134650670 | 132916492 | 234886 | 1499292 | 0 | 0 |
| L3 | 116060728 | 114580756 | 212466 | 1267506 | 0 | 0 |
| L4 | 109274778 | 107891058 | 228686 | 1155034 | 0 | 0 |

^a^ Raw Reads: Reads obtained from the sequencing machines

^b^ Clean Reads: High quality reads

^c^ Adapter: Reads containing adapters

^d^ Low quality: Reads containing more than 50% of low quality (Q-value≤20) bases.

^e^ polyA: Reads containing polyA

^f^ N: Reads containing more than 10% of unknown nucleotides (N)

Supplementary Table 2 Statistics for ribosome alignment

| Sample | Clean Reads^a^ | Mapped Reads^b^ | Unmapped Reads^c^ |
| --- | --- | --- | --- |
| H1 | 113682224 | 53717464 | 59964760 |
| H2 | 127710488 | 64176864 | 63533624 |
| H3 | 111321746 | 54527636 | 56794110 |
| H4 | 115842072 | 55260014 | 60582058 |
| L1 | 121717410 | 57782028 | 63935382 |
| L2 | 132916492 | 63701294 | 69215198 |
| L3 | 114580756 | 51506486 | 63074270 |
| L4 | 107891058 | 49816534 | 58074524 |

^a^ Clean Reads: Reads that meet the quality control standard

^b^ Mapped Reads: Reads mapped on the ribosome database

^c^ Unmapped Read: Reads not mapped on the ribosome database

Supplementary Table 3 Statistics for mapping reads

^a^ Total: Total number of clean reads

^b^ Unmapped: Reads not aligned to the reference sequence

^c^ Unique_ Mapped: Reads aligned to only one position
^d^ Multiple_ Mapped: Reads aligned to two or more positions
^e^ Total_ mapped: Reads that completely aligned to the reference sequence

| Sample | Total^a^ | Unmapped^b^ | Unique_ Mapped^c^ | Multiple_ Mapped^d^ | Total_ Mapped^e^ |
| --- | --- | --- | --- | --- | --- |
| H1 | 59964760 | 9066482 | 47380876 | 3517402 | 50898278 |
| H2 | 63533624 | 10671923 | 48784652 | 4077049 | 52861701 |
| H3 | 56794110 | 9534115 | 43462525 | 3797470 | 47259995 |
| H4 | 60582058 | 9378794 | 46859700 | 4343564 | 51203264 |
| L1 | 63935382 | 10529588 | 49131821 | 4273973 | 53405794 |
| L2 | 69215198 | 11304511 | 53523484 | 4387203 | 57910687 |
| L3 | 63074270 | 9600083 | 49311578 | 4162609 | 53474187 |
| L4 | 58074524 | 8705438 | 45735733 | 3633353 | 49369086 |
