# Supplementary material for: Identification and Functional Prediction of Circular RNAs Related to Growth Traits and Skeletal Muscle Development in Duroc pigs
Source: Front Genet. 2022 Sep 2;13:858763. doi: 10.3389/fgene.2022.858763 (PMC9478749; doi:10.3389/fgene.2022.858763)
Supplement: Supplementary file 2 [file Table2.DOCX]

Supplementary Table 1 Statistics for filtering reads

| Sample | Raw Reads^a^ | Clean Reads^b^ | Adapter^c^ | Low quality^d^ | polyA^e^ | N^f^ | Q20(%) | Q30(%) |
| --- | --- | --- | --- | --- | --- | --- | --- | --- |
| H1 | 115340660 | 113682224 | 212138 | 1446298 | 0 | 0 | 96.02 | 91.73 |
| H2 | 129492024 | 127710488 | 254268 | 1527268 | 0 | 0 | 96.00 | 91.81 |
| H3 | 112871496 | 111321746 | 204364 | 1345386 | 0 | 0 | 96.05 | 91.81 |
| H4 | 117494942 | 115842072 | 228258 | 1424612 | 0 | 0 | 96.14 | 91.98 |
| L1 | 123083610 | 121717410 | 222712 | 1143488 | 0 | 0 | 96.18 | 91.78 |
| L2 | 134650670 | 132916492 | 234886 | 1499292 | 0 | 0 | 96.30 | 92.16 |
| L3 | 116060728 | 114580756 | 212466 | 1267506 | 0 | 0 | 96.38 | 92.30 |
| L4 | 109274778 | 107891058 | 228686 | 1155034 | 0 | 0 | 96.35 | 92.24 |

^a^ Raw Reads: Reads obtained from the sequencing machines

^b^ Clean Reads: High quality reads

^c^ Adapter: Reads containing adapters

^d^ Low quality: Reads containing more than 50% of low quality (Q-value≤20) bases.

^e^ polyA: Reads containing polyA

^f^ N: Reads containing more than 10% of unknown nucleotides (N)

Supplementary Table 2 Statistics for ribosome alignment

| Sample | Clean Reads^a^ | Mapped Reads^b^ | Unmapped Reads^c^ |
| --- | --- | --- | --- |
| H1 | 113682224 | 53717464 | 59964760 |
| H2 | 127710488 | 64176864 | 63533624 |
| H3 | 111321746 | 54527636 | 56794110 |
| H4 | 115842072 | 55260014 | 60582058 |
| L1 | 121717410 | 57782028 | 63935382 |
| L2 | 132916492 | 63701294 | 69215198 |
| L3 | 114580756 | 51506486 | 63074270 |
| L4 | 107891058 | 49816534 | 58074524 |

^a^ Clean Reads: Reads that meet the quality control standard

^b^ Mapped Reads: Reads mapped on the ribosome database

^c^ Unmapped Read: Reads not mapped on the ribosome database

Supplementary Table 3 Statistics for mapping reads

^a^ Total: Total number of clean reads

^b^ Unmapped: Reads not aligned to the reference sequence

^c^ Unique_ Mapped: Reads aligned to only one position
^d^ Multiple_ Mapped: Reads aligned to two or more positions
^e^ Total_ mapped: Reads that completely aligned to the reference sequence

| Sample | Total^a^ | Unmapped^b^ | Unique_ Mapped^c^ | Multiple_ Mapped^d^ | Total_ Mapped^e^ |
| --- | --- | --- | --- | --- | --- |
| H1 | 59964760 | 9066482 | 47380876 | 3517402 | 50898278 |
| H2 | 63533624 | 10671923 | 48784652 | 4077049 | 52861701 |
| H3 | 56794110 | 9534115 | 43462525 | 3797470 | 47259995 |
| H4 | 60582058 | 9378794 | 46859700 | 4343564 | 51203264 |
| L1 | 63935382 | 10529588 | 49131821 | 4273973 | 53405794 |
| L2 | 69215198 | 11304511 | 53523484 | 4387203 | 57910687 |
| L3 | 63074270 | 9600083 | 49311578 | 4162609 | 53474187 |
| L4 | 58074524 | 8705438 | 45735733 | 3633353 | 49369086 |

Supplementary Table 4 The specific information of DE circRNAs

| id | log2(fc) | P Value | source_gene |
| --- | --- | --- | --- |
| novel_circ_000052 | -15.7274251 | 0.01543337 | ENSSSCG00000015777 |
| novel_circ_000083 | -2.92820348 | 0.00991035 | ENSSSCG00000000530 |
| novel_circ_000136 | 1.354170453 | 0.04523208 | ENSSSCG00000009405 |
| novel_circ_000156 | 15.62523988 | 0.03658244 | ENSSSCG00000008826 |
| novel_circ_000348 | -3.514289316 | 0.01742797 | ENSSSCG00000025156 |
| novel_circ_000438 | 1.63352631 | 0.04305408 | ENSSSCG00000002285 |
| novel_circ_000636 | -2.560231891 | 0.04610706 | ENSSSCG00000000755 |
| novel_circ_000638 | 1.30958409 | 0.0093062 | ENSSSCG00000010360 |
| novel_circ_000688 | 0.843530422 | 0.00624105 | ENSSSCG00000031705 |
| novel_circ_000736 | 1.126186855 | 0.04859827 | ENSSSCG00000015428 |
| novel_circ_000796 | 0.680583182 | 0.03304006 | ENSSSCG00000024570 |
| novel_circ_000844 | -1.665236433 | 0.01615344 | ENSSSCG00000002755 |
| novel_circ_000888 | -1.02755408 | 0.03777698 | ENSSSCG00000006388 |
| novel_circ_000966 | -2.883831021 | 0.01747674 | ENSSSCG00000010579 |
| novel_circ_000984 | 15.79166382 | 0.0349461 | ENSSSCG00000009776 |
| novel_circ_001014 | -2.226958269 | 0.00495106 | ENSSSCG00000004203 |
| novel_circ_001137 | 0.875857388 | 0.03699175 | ENSSSCG00000003531 |
| novel_circ_001213 | -15.58517788 | 0.04909681 | ENSSSCG00000007026 |
| novel_circ_001237 | 1.44576533 | 0.03554933 | ENSSSCG00000015097 |
| novel_circ_001283 | -1.256699562 | 0.03112711 | ENSSSCG00000015867 |
| novel_circ_001312 | -16.73527809 | 0.00025321 | ENSSSCG00000008761 |
| novel_circ_001362 | -2.166810542 | 0.01464081 | ENSSSCG00000011131 |
| novel_circ_001488 | 15.6808644 | 0.04851022 | ENSSSCG00000010508 |
| novel_circ_001578 | 15.7541243 | 0.03637256 | ENSSSCG00000000970 |
| novel_circ_001622 | -15.75658662 | 0.03327359 | ENSSSCG00000026940 |
| novel_circ_001652 | 0.931910585 | 0.04077591 | ENSSSCG00000016113 |
| novel_circ_001702 | -1.734145758 | 0.00315872 | ENSSSCG00000038617 |
| novel_circ_001745 | -15.58187234 | 0.03927457 | ENSSSCG00000013402 |
| novel_circ_001762 | -15.86010518 | 0.02202215 | ENSSSCG00000004328 |
| novel_circ_001784 | 0.840239006 | 0.02501447 | ENSSSCG00000006267 |
| novel_circ_001865 | -2.441833592 | 0.02379101 | ENSSSCG00000029251 |
| novel_circ_002049 | 2.747972405 | 0.02216549 | ENSSSCG00000004276 |
| novel_circ_002090 | -16.5619132 | 0.00021637 | ENSSSCG00000018049 |
| novel_circ_002091 | -17.23117707 | 6.09E-06 | ENSSSCG00000002277 |
| novel_circ_002181 | 1.350983293 | 0.0493022 | ENSSSCG00000017513 |
| novel_circ_002254 | -3.860476499 | 0.01007386 | ENSSSCG00000004636 |
| novel_circ_002265 | -16.04086703 | 0.02069256 | ENSSSCG00000035581 |
| novel_circ_002272 | 2.498759348 | 0.022568 | ENSSSCG00000003700 |
| novel_circ_002301 | 3.602645418 | 0.02935025 | ENSSSCG00000005916 |
| novel_circ_002336 | 2.811675467 | 0.03073733 | ENSSSCG00000027722 |
| novel_circ_002418 | 3.490202951 | 0.005432 | ENSSSCG00000007951 |
| novel_circ_002545 | -16.19578588 | 0.00710245 | ENSSSCG00000031940 |
| novel_circ_002577 | -1.201371329 | 0.0499232 | ENSSSCG00000011103 |
| novel_circ_002622 | -1.890768549 | 0.04659844 | ENSSSCG00000006981 |
| novel_circ_002640 | -1.108848444 | 0.00613304 | ENSSSCG00000009652 |
| novel_circ_002663 | -1.660561016 | 0.04563223 | ENSSSCG00000016834 |
| novel_circ_002672 | 0.785079899 | 0.01788081 | ENSSSCG00000013054 |
| novel_circ_002677 | -15.50756778 | 0.02617961 | ENSSSCG00000017094 |
| novel_circ_002707 | 3.651120313 | 0.03897809 | ENSSSCG00000016510 |
| novel_circ_002881 | 15.81998144 | 0.02489127 | ENSSSCG00000004938 |
| novel_circ_003011 | 1.316621048 | 0.03115186 | ENSSSCG00000016888 |
| novel_circ_003068 | -2.920889359 | 0.03186631 | ENSSSCG00000011811 |
| novel_circ_003073 | 2.808825695 | 0.00010547 | ENSSSCG00000009300 |
| novel_circ_003086 | 2.976775652 | 0.00343054 | ENSSSCG00000009464 |
| novel_circ_003114 | 1.289748623 | 0.01021342 | ENSSSCG00000039885 |
| novel_circ_003173 | -3.325547958 | 0.00326419 | ENSSSCG00000016543 |
| novel_circ_003193 | 0.82745072 | 0.04839539 | ENSSSCG00000009968 |
| novel_circ_003239 | 1.583632729 | 0.03905854 | ENSSSCG00000013223 |
| novel_circ_003270 | -15.7336165 | 0.02731812 | ENSSSCG00000009007 |
| novel_circ_003296 | 16.22399235 | 0.01423401 | ENSSSCG00000011022 |
| novel_circ_003448 | 15.82366381 | 0.0099466 | ENSSSCG00000017812 |
| novel_circ_003457 | -2.695323741 | 0.0055461 | ENSSSCG00000015743 |
| novel_circ_003472 | 2.369506817 | 0.03394197 | ENSSSCG00000018049 |
| novel_circ_003596 | 2.686021694 | 0.01667707 | ENSSSCG00000024453 |
| novel_circ_003643 | 16.03896321 | 0.00635999 | ENSSSCG00000007046 |
| novel_circ_003778 | -0.887030657 | 0.02887474 | ENSSSCG00000011068 |
| novel_circ_003813 | -15.37619691 | 0.02676614 | ENSSSCG00000033954 |
| novel_circ_003822 | 1.584468219 | 0.02159689 | ENSSSCG00000036083 |
| novel_circ_003845 | 2.06396414 | 0.00443197 | ENSSSCG00000015569 |
| novel_circ_003927 | -2.947004896 | 0.02951939 | ENSSSCG00000007187 |
| novel_circ_003977 | -4.017063925 | 0.02257562 | ENSSSCG00000015908 |
| novel_circ_003999 | -15.68649808 | 0.03106554 | ENSSSCG00000017748 |
| novel_circ_004044 | -3.50902874 | 0.04904269 | ENSSSCG00000008540 |
| novel_circ_004047 | -15.784717 | 0.0248175 | ENSSSCG00000023533 |
| novel_circ_004154 | -2.389021525 | 0.01096936 | ENSSSCG00000050244 |
| novel_circ_004167 | 2.1436184 | 0.03444374 | ENSSSCG00000009090 |
| novel_circ_004375 | -1.955137875 | 0.01172706 | ENSSSCG00000002426 |
| novel_circ_004386 | -15.53767133 | 0.01266839 | ENSSSCG00000032549 |
| novel_circ_004395 | 2.418851575 | 0.0328454 | ENSSSCG00000000078 |
| novel_circ_004497 | -0.965736415 | 0.029457 | ENSSSCG00000028225 |
| novel_circ_004709 | -3.302585798 | 0.02332017 | ENSSSCG00000012884 |
| novel_circ_004720 | -15.96008798 | 0.00677866 | ENSSSCG00000033591 |
| novel_circ_004734 | -0.850448992 | 0.04814334 | ENSSSCG00000029249 |
| novel_circ_004735 | -15.74754593 | 0.02729723 | ENSSSCG00000011949 |
| novel_circ_004757 | 3.470006701 | 0.02903626 | ENSSSCG00000029119 |
| novel_circ_004782 | 15.82364816 | 0.0225924 | ENSSSCG00000012055 |
| novel_circ_004826 | -15.55928375 | 0.02002934 | ENSSSCG00000014364 |
| novel_circ_004868 | -3.197233506 | 0.0035561 | ENSSSCG00000007307 |
| novel_circ_004932 | 2.214191103 | 0.04488864 | ENSSSCG00000003828 |
| novel_circ_004988 | 15.79163182 | 0.02071236 | NA |
| novel_circ_005018 | 15.99866566 | 0.03539371 | ENSSSCG00000000492 |
| novel_circ_005085 | 16.02037813 | 0.01754762 | ENSSSCG00000000770 |
| novel_circ_005170 | -0.931620359 | 0.03717252 | ENSSSCG00000001852 |
| novel_circ_005211 | 3.680754288 | 0.04279983 | ENSSSCG00000007331 |
| novel_circ_005294 | 0.933096289 | 0.03043817 | ENSSSCG00000010652 |
| novel_circ_005315 | 1.483722235 | 0.03197994 | ENSSSCG00000023520 |
| novel_circ_005319 | 2.240351226 | 0.01994653 | ENSSSCG00000022830 |
| novel_circ_005323 | 15.55828155 | 0.04836545 | ENSSSCG00000008635 |
| novel_circ_005338 | -4.278539773 | 0.00125729 | ENSSSCG00000030396 |
| novel_circ_005349 | 2.030230432 | 0.02335915 | ENSSSCG00000027459 |
| novel_circ_005381 | -1.867083618 | 0.01149911 | ENSSSCG00000039085 |
| novel_circ_005416 | -3.397757574 | 0.03057924 | ENSSSCG00000017513 |
| novel_circ_005417 | 1.798671109 | 0.00818949 | ENSSSCG00000014828 |
| novel_circ_005471 | 0.769647874 | 0.00679006 | ENSSSCG00000024814 |
| novel_circ_005502 | 1.262768726 | 0.01132114 | ENSSSCG00000005090 |
| novel_circ_005539 | -16.41213261 | 0.04135793 | ENSSSCG00000008974 |
| novel_circ_005610 | 1.375688115 | 0.00553504 | ENSSSCG00000020663 |
| novel_circ_005672 | -2.037839351 | 0.00694082 | ENSSSCG00000040184 |
| novel_circ_005761 | -15.87090812 | 0.02055374 | ENSSSCG00000011845 |
| novel_circ_005789 | 0.639571807 | 0.04175163 | ENSSSCG00000032531 |
| novel_circ_005805 | 2.603211721 | 0.04227849 | ENSSSCG00000015052 |
| novel_circ_005888 | 2.597913179 | 0.01588389 | ENSSSCG00000010703 |
| novel_circ_006009 | 1.60710458 | 0.04630374 | ENSSSCG00000026268 |
| novel_circ_006022 | 15.60874532 | 0.04697025 | ENSSSCG00000016061 |
| novel_circ_006079 | 1.80588985 | 0.02653465 | ENSSSCG00000023759 |
| novel_circ_006098 | 16.52795525 | 0.02127046 | ENSSSCG00000006872 |
| novel_circ_006227 | -3.309185971 | 0.04844186 | ENSSSCG00000001903 |
| novel_circ_006263 | 15.79829522 | 0.02410205 | ENSSSCG00000022225 |
| novel_circ_006335 | 1.431575834 | 0.04549259 | ENSSSCG00000013984 |
| novel_circ_006388 | 15.77334143 | 0.03608259 | ENSSSCG00000037065 |
| novel_circ_006502 | 0.863883361 | 0.0463449 | ENSSSCG00000000900 |
| novel_circ_006520 | -1.064051376 | 0.0070608 | ENSSSCG00000034569 |
| novel_circ_006522 | -1.363543192 | 0.03717111 | ENSSSCG00000000801 |
| novel_circ_006608 | 1.455975409 | 0.03321729 | ENSSSCG00000008803 |
| novel_circ_006685 | 2.790783846 | 0.03906282 | ENSSSCG00000029311 |
| novel_circ_006741 | -1.373665247 | 0.013842 | ENSSSCG00000011394 |
| novel_circ_006754 | -15.97511263 | 0.00997085 | ENSSSCG00000034364 |
| novel_circ_006764 | 0.97845304 | 0.00029881 | ENSSSCG00000010719 |
| novel_circ_006787 | -1.345448729 | 0.03486846 | ENSSSCG00000002844 |
| novel_circ_006803 | -1.120202093 | 0.03828201 | ENSSSCG00000000784 |
| novel_circ_006870 | 1.457291314 | 0.02760796 | ENSSSCG00000011775 |
| novel_circ_006947 | -2.406354222 | 0.00646206 | ENSSSCG00000028293 |
| novel_circ_006958 | -2.88939812 | 0.01390475 | ENSSSCG00000011312 |
| novel_circ_006960 | 0.606383841 | 0.02789832 | ENSSSCG00000007478 |
| novel_circ_006997 | -16.27180413 | 0.00575792 | ENSSSCG00000023133 |
| novel_circ_007065 | 3.978876421 | 0.04081967 | ENSSSCG00000010243 |
| novel_circ_007067 | -2.524423013 | 0.02446814 | ENSSSCG00000011424 |
| novel_circ_007121 | 1.039976051 | 0.02165243 | ENSSSCG00000010485 |
| novel_circ_007139 | 1.414001609 | 0.01676838 | ENSSSCG00000024916 |
| novel_circ_007180 | -3.169269205 | 0.00357756 | ENSSSCG00000010299 |
| novel_circ_007226 | -3.836745646 | 0.00708554 | ENSSSCG00000009814 |
| novel_circ_007256 | 1.432277523 | 0.03443387 | ENSSSCG00000024814 |
| novel_circ_007261 | 3.095232238 | 0.02075875 | ENSSSCG00000014126 |
| novel_circ_007265 | -15.85029592 | 0.02272005 | ENSSSCG00000004979 |
| novel_circ_007285 | 3.453026993 | 0.04721011 | ENSSSCG00000011183 |
| novel_circ_007404 | -0.690073761 | 0.02351933 | ENSSSCG00000006487 |
| novel_circ_007493 | -1.283799474 | 0.03801159 | ENSSSCG00000004919 |
| novel_circ_007496 | -15.91934407 | 0.0205261 | ENSSSCG00000006867 |
| novel_circ_007555 | -3.382532507 | 0.03241616 | ENSSSCG00000011373 |
| novel_circ_007580 | -15.37619691 | 0.02677789 | ENSSSCG00000024517 |
| novel_circ_007586 | -1.08212421 | 0.04520613 | ENSSSCG00000028979 |
| novel_circ_007587 | 1.711014644 | 0.02078069 | ENSSSCG00000015589 |
| novel_circ_007640 | 1.854896129 | 0.04318637 | NA |
| novel_circ_007669 | -15.38067293 | 0.02709689 | ENSSSCG00000021814 |
| novel_circ_007686 | -1.876308419 | 0.01903217 | ENSSSCG00000010882 |
| novel_circ_007746 | 15.64547612 | 0.04704887 | ENSSSCG00000023112 |
| novel_circ_007749 | -1.382890968 | 0.04415133 | ENSSSCG00000003693 |
| novel_circ_007779 | -1.358060491 | 0.03335061 | ENSSSCG00000024373 |
| novel_circ_007849 | 1.298148494 | 0.0384743 | ENSSSCG00000029311 |
| novel_circ_007873 | 3.530899651 | 0.00439674 | ENSSSCG00000023865 |
| novel_circ_007943 | 15.70110967 | 0.02532791 | ENSSSCG00000032531 |
| novel_circ_007949 | -15.65937308 | 0.03435884 | ENSSSCG00000012743 |
| novel_circ_008004 | -3.659749957 | 0.02097649 | ENSSSCG00000022240 |
| novel_circ_008038 | 1.286038762 | 0.04080385 | ENSSSCG00000010485 |
| novel_circ_008042 | -1.124833018 | 0.01774592 | ENSSSCG00000000571 |
| novel_circ_008054 | -2.892109738 | 0.0374556 | ENSSSCG00000015840 |
| novel_circ_008059 | 2.272874123 | 0.01110327 | ENSSSCG00000038913 |
| novel_circ_008205 | 0.900282251 | 0.01257307 | ENSSSCG00000002703 |
| novel_circ_008220 | -2.404794822 | 0.01441609 | ENSSSCG00000038465 |
| novel_circ_008262 | -3.913499384 | 0.00139995 | ENSSSCG00000014416 |
| novel_circ_008268 | 16.01446904 | 0.01875082 | ENSSSCG00000016842 |
| novel_circ_008286 | 0.670663803 | 0.04878579 | ENSSSCG00000017198 |
| novel_circ_008287 | 2.475611743 | 0.00857041 | ENSSSCG00000006137 |
| novel_circ_008312 | -2.685708092 | 0.04285291 | ENSSSCG00000009029 |
| novel_circ_008356 | 15.60037418 | 0.04146641 | ENSSSCG00000003461 |
| novel_circ_008374 | 1.81295085 | 0.03154211 | ENSSSCG00000025194 |
| novel_circ_008433 | 1.814970332 | 0.0172591 | ENSSSCG00000037719 |
| novel_circ_008472 | 2.197156232 | 0.01261214 | ENSSSCG00000000866 |
| novel_circ_008483 | 3.715818941 | 0.03644696 | ENSSSCG00000026004 |
| novel_circ_008533 | -2.972764351 | 0.04196572 | ENSSSCG00000003531 |
| novel_circ_008611 | 15.74711362 | 0.0484693 | ENSSSCG00000006388 |
| novel_circ_008667 | -16.86878747 | 0.02066149 | ENSSSCG00000006701 |
| novel_circ_008800 | 1.283812142 | 0.04490399 | ENSSSCG00000021791 |
| novel_circ_008946 | 1.392863447 | 0.02840122 | ENSSSCG00000034191 |
| novel_circ_008975 | 1.494572384 | 0.01691846 | ENSSSCG00000026465 |
| novel_circ_009025 | 4.752859296 | 0.00030627 | ENSSSCG00000003627 |
| novel_circ_009034 | 16.03620631 | 0.03339606 | ENSSSCG00000011342 |
| novel_circ_009096 | 15.669331 | 0.03260969 | ENSSSCG00000012059 |
| novel_circ_009109 | 2.243948074 | 0.00349316 | ENSSSCG00000027667 |
| novel_circ_009189 | 16.05419406 | 0.01486433 | ENSSSCG00000017505 |
| novel_circ_009192 | -1.228427983 | 0.00800198 | ENSSSCG00000006390 |
| novel_circ_009244 | -15.58575444 | 0.0210128 | ENSSSCG00000046928 |
| novel_circ_009297 | 15.93059358 | 0.00896136 | ENSSSCG00000008943 |
| novel_circ_009331 | 2.940971167 | 0.00810489 | ENSSSCG00000049158 |
| novel_circ_009427 | 0.846385088 | 0.00557834 | ENSSSCG00000016543 |
| novel_circ_009585 | 15.87313096 | 0.01465439 | ENSSSCG00000011858 |
| novel_circ_009612 | 2.287248564 | 0.00571955 | ENSSSCG00000011064 |
| novel_circ_009669 | 1.445485368 | 0.04951757 | ENSSSCG00000024168 |
| novel_circ_009740 | -15.93205052 | 0.04897272 | ENSSSCG00000006911 |
| novel_circ_009756 | -15.72993847 | 0.02813879 | ENSSSCG00000009007 |
| novel_circ_009769 | 0.827011509 | 0.01177809 | ENSSSCG00000006179 |
| novel_circ_009843 | -3.80886683 | 0.02513533 | ENSSSCG00000038994 |
| novel_circ_009852 | 0.717592812 | 0.00515897 | ENSSSCG00000004029 |
| novel_circ_009872 | 3.543432753 | 0.04837484 | ENSSSCG00000002799 |
| novel_circ_009977 | -15.94706992 | 0.02787737 | ENSSSCG00000004725 |
| novel_circ_009983 | 2.890935567 | 0.03064388 | ENSSSCG00000001950 |
| novel_circ_010012 | -2.778588872 | 0.04743551 | ENSSSCG00000025323 |
| novel_circ_010066 | 3.259710621 | 0.02983922 | ENSSSCG00000005215 |
| novel_circ_010175 | 1.23899222 | 0.01059177 | ENSSSCG00000034386 |
| novel_circ_010385 | -1.454908023 | 0.04919283 | ENSSSCG00000020743 |
| novel_circ_010391 | -2.082011561 | 0.01665528 | ENSSSCG00000026606 |
| novel_circ_010412 | -15.86650196 | 0.00600091 | ENSSSCG00000009242 |
| novel_circ_010419 | 3.766388484 | 0.03493312 | ENSSSCG00000005444 |
| novel_circ_010454 | 2.071857201 | 0.04777816 | ENSSSCG00000012459 |
| novel_circ_010490 | 1.454722434 | 0.02727874 | ENSSSCG00000021042 |
| novel_circ_010509 | 15.62077132 | 0.03214069 | ENSSSCG00000002799 |
| novel_circ_010549 | -1.369728201 | 0.03223834 | ENSSSCG00000039157 |
| novel_circ_010594 | -3.400754504 | 0.04169569 | ENSSSCG00000026177 |
| novel_circ_010708 | -2.648022779 | 0.02462764 | ENSSSCG00000000078 |
| novel_circ_010766 | 1.51451118 | 0.00073565 | ENSSSCG00000021038 |
| novel_circ_010859 | -2.188610248 | 0.03470222 | ENSSSCG00000035854 |
| novel_circ_010896 | 16.22398049 | 0.00743232 | ENSSSCG00000020858 |
| novel_circ_011021 | -1.64534884 | 0.03394562 | ENSSSCG00000014949 |
| novel_circ_011022 | -3.730343986 | 0.0311523 | ENSSSCG00000040013 |
| novel_circ_011133 | 1.67484536 | 0.0048066 | ENSSSCG00000008144 |
| novel_circ_011134 | -1.925659102 | 0.02565394 | ENSSSCG00000003670 |
| novel_circ_011195 | 15.54494005 | 0.03056253 | ENSSSCG00000000959 |
| novel_circ_011212 | 16.19395835 | 0.01564302 | ENSSSCG00000006267 |
| novel_circ_011248 | 16.36569291 | 0.04913288 | ENSSSCG00000016430 |
| novel_circ_011278 | 1.831429251 | 0.02904541 | ENSSSCG00000007874 |
| novel_circ_011328 | -4.074506275 | 0.00651034 | ENSSSCG00000034086 |
| novel_circ_011388 | 3.443846709 | 0.01808777 | ENSSSCG00000009241 |
| novel_circ_011402 | -17.05706349 | 2.00E-05 | ENSSSCG00000010164 |
| novel_circ_011448 | 2.762647654 | 0.02289354 | ENSSSCG00000017668 |
| novel_circ_011509 | 1.104828937 | 0.03728634 | ENSSSCG00000016717 |
| novel_circ_011548 | -16.0029096 | 0.01006742 | ENSSSCG00000034200 |
| novel_circ_011579 | -1.651537547 | 0.01391024 | ENSSSCG00000011752 |
| novel_circ_011584 | -1.259183298 | 0.04065283 | ENSSSCG00000011765 |
| novel_circ_011649 | 3.689331885 | 0.03320598 | ENSSSCG00000011340 |
| novel_circ_011661 | 1.749192189 | 0.02664016 | ENSSSCG00000034191 |
| novel_circ_011712 | -3.675544643 | 0.00832296 | ENSSSCG00000030486 |
| novel_circ_011716 | -1.913768239 | 0.0016629 | ENSSSCG00000021885 |
| novel_circ_011764 | 2.446508772 | 0.0450469 | ENSSSCG00000001966 |
| novel_circ_011778 | -16.22846643 | 0.01301825 | ENSSSCG00000009435 |
| novel_circ_011783 | 1.242582676 | 0.04271561 | ENSSSCG00000036828 |
| novel_circ_011854 | 1.181245044 | 0.0053309 | ENSSSCG00000010243 |
| novel_circ_011981 | 2.749819384 | 0.02346905 | ENSSSCG00000033323 |
| novel_circ_012051 | 0.902710515 | 0.03715909 | ENSSSCG00000025606 |
| novel_circ_012119 | 1.680801524 | 0.0455154 | ENSSSCG00000008150 |
| novel_circ_012135 | 1.803966361 | 0.03188021 | ENSSSCG00000003630 |
| novel_circ_012137 | -17.96646511 | 0.00212188 | ENSSSCG00000007805 |
| novel_circ_012163 | -3.472764141 | 0.01571468 | ENSSSCG00000000571 |
| novel_circ_012165 | -1.112056601 | 0.02305258 | ENSSSCG00000030957 |
| novel_circ_012222 | -1.183368071 | 8.78E-05 | ENSSSCG00000015283 |
| novel_circ_012283 | -15.72824755 | 0.03347551 | ENSSSCG00000029464 |
| novel_circ_012322 | -0.63309369 | 0.03366208 | ENSSSCG00000009134 |
| novel_circ_012342 | -16.44012511 | 0.0051236 | ENSSSCG00000012884 |
| novel_circ_012347 | -2.080562357 | 0.03872363 | ENSSSCG00000016510 |
| novel_circ_012363 | -17.14442547 | 0.0117509 | ENSSSCG00000037775 |
| novel_circ_012387 | -1.526740397 | 0.02764801 | ENSSSCG00000010734 |
| novel_circ_012401 | -2.864979706 | 0.03712726 | ENSSSCG00000001646 |
| novel_circ_012519 | 2.99485872 | 0.046257 | ENSSSCG00000008761 |
| novel_circ_012551 | 15.73928674 | 0.01143599 | ENSSSCG00000017877 |
| novel_circ_012588 | -1.575198506 | 0.04568151 | ENSSSCG00000003461 |
| novel_circ_012653 | 0.912826844 | 0.01611842 | ENSSSCG00000014114 |
| novel_circ_012727 | 1.08702073 | 0.02075028 | ENSSSCG00000048636 |
| novel_circ_012728 | -15.77063766 | 0.04965515 | ENSSSCG00000006179 |
| novel_circ_012732 | -15.53365748 | 0.02478187 | ENSSSCG00000001862 |
| novel_circ_012743 | -15.48377541 | 0.04708632 | ENSSSCG00000021023 |
| novel_circ_012802 | -1.784456624 | 0.02492786 | ENSSSCG00000025703 |
| novel_circ_012830 | 15.87311584 | 0.0088658 | NA |
| novel_circ_012836 | 15.57949064 | 0.02682154 | ENSSSCG00000036350 |
| novel_circ_012954 | -3.186542996 | 0.02931202 | ENSSSCG00000024592 |
| novel_circ_012982 | 4.491553405 | 0.00309489 | ENSSSCG00000011775 |
| novel_circ_013009 | -1.949697265 | 0.02283278 | ENSSSCG00000003771 |
| novel_circ_013017 | 15.84495938 | 0.01038098 | ENSSSCG00000008105 |
| novel_circ_013087 | -1.596166308 | 0.0321901 | ENSSSCG00000045048 |
| novel_circ_013095 | -1.563551395 | 0.0366892 | ENSSSCG00000016374 |
| novel_circ_013133 | -3.421516965 | 0.02295878 | ENSSSCG00000016306 |
| novel_circ_013161 | 1.895069846 | 0.00643929 | ENSSSCG00000030610 |
| novel_circ_013237 | 16.50596893 | 0.02187235 | ENSSSCG00000011632 |
| novel_circ_013258 | -1.978327352 | 0.03234434 | ENSSSCG00000013284 |
| novel_circ_013259 | -2.126770803 | 0.0464501 | ENSSSCG00000011889 |
| novel_circ_013293 | 2.019488745 | 0.04952716 | ENSSSCG00000008821 |
| novel_circ_013362 | 2.768037888 | 0.03361614 | ENSSSCG00000023983 |
| novel_circ_013417 | 0.995467176 | 0.01758686 | ENSSSCG00000014406 |
| novel_circ_013460 | -15.67664578 | 0.0319782 | ENSSSCG00000015743 |
| novel_circ_013501 | -3.038976524 | 0.00064332 | ENSSSCG00000039952 |
| novel_circ_013583 | 0.965777942 | 0.02063453 | ENSSSCG00000038397 |
| novel_circ_013623 | 2.688771725 | 0.00110546 | ENSSSCG00000002875 |
| novel_circ_013659 | 3.966811057 | 0.00538234 | NA |
| novel_circ_013806 | -1.170436777 | 0.0460845 | ENSSSCG00000003401 |
| novel_circ_013831 | 1.435277243 | 0.01048645 | ENSSSCG00000009230 |
| novel_circ_013844 | 1.38719258 | 0.00925757 | ENSSSCG00000010485 |
| novel_circ_013938 | 2.0270089 | 0.0114372 | ENSSSCG00000030413 |
| novel_circ_013939 | 3.32529573 | 0.03238265 | ENSSSCG00000011317 |

Supplementary Table 5 GO analysis of source genes of DE circRNAs

| Type | Term | L-vs-H |
| --- | --- | --- |
| Biological Process | GO:0000003 reproduction | 0.020764873 |
| Biological Process | GO:0000018 regulation of DNA recombination | 0.027588216 |
| Biological Process | GO:0000027 ribosomal large subunit assembly | 0.020803498 |
| Biological Process | GO:0000186 activation of MAPKK activity | 0.004189952 |
| Biological Process | GO:0000271 polysaccharide biosynthetic process | 0.031759239 |
| Biological Process | GO:0000288 nuclear-transcribed mRNA catabolic process, deadenylation-dependent decay | 0.011076044 |
| Biological Process | GO:0000289 nuclear-transcribed mRNA poly(A) tail shortening | 0.013468928 |
| Biological Process | GO:0000723 telomere maintenance | 0.000411761 |
| Biological Process | GO:0000956 nuclear-transcribed mRNA catabolic process | 0.000101999 |
| Biological Process | GO:0001543 ovarian follicle rupture | 0.01504685 |
| Biological Process | GO:0001544 initiation of primordial ovarian follicle growth | 0.029868307 |
| Biological Process | GO:0001568 blood vessel development | 0.027764374 |
| Biological Process | GO:0001880 Mullerian duct regression | 0.029868307 |
| Biological Process | GO:0001935 endothelial cell proliferation | 0.005308351 |
| Biological Process | GO:0001936 regulation of endothelial cell proliferation | 0.002784882 |
| Biological Process | GO:0001938 positive regulation of endothelial cell proliferation | 0.009947245 |
| Biological Process | GO:0001944 vasculature development | 0.0399914 |
| Biological Process | GO:0001952 regulation of cell-matrix adhesion | 0.036472097 |
| Biological Process | GO:0002331 pre-B cell allelic exclusion | 0.04446773 |
| Biological Process | GO:0002444 myeloid leukocyte mediated immunity | 0.043331851 |
| Biological Process | GO:0002886 regulation of myeloid leukocyte mediated immunity | 0.013152448 |
| Biological Process | GO:0002888 positive regulation of myeloid leukocyte mediated immunity | 0.02943371 |
| Biological Process | GO:0003009 skeletal muscle contraction | 0.04635113 |
| Biological Process | GO:0003010 voluntary skeletal muscle contraction | 0.04446773 |
| Biological Process | GO:0003148 outflow tract septum morphogenesis | 0.035843078 |
| Biological Process | GO:0003171 atrioventricular valve development | 0.03257587 |
| Biological Process | GO:0003174 mitral valve development | 0.007568012 |
| Biological Process | GO:0003175 tricuspid valve development | 0.003248694 |
| Biological Process | GO:0003181 atrioventricular valve morphogenesis | 0.026421141 |
| Biological Process | GO:0003183 mitral valve morphogenesis | 0.005944903 |
| Biological Process | GO:0003186 tricuspid valve morphogenesis | 0.002187463 |
| Biological Process | GO:0003222 ventricular trabecula myocardium morphogenesis | 0.011335565 |
| Biological Process | GO:0003249 cell proliferation involved in heart valve morphogenesis | 0.04446773 |
| Biological Process | GO:0003250 regulation of cell proliferation involved in heart valve morphogenesis | 0.04446773 |
| Biological Process | GO:0003252 negative regulation of cell proliferation involved in heart valve morphogenesis | 0.029868307 |
| Biological Process | GO:0003292 cardiac septum cell differentiation | 0.04446773 |
| Biological Process | GO:0005976 polysaccharide metabolic process | 0.000427241 |
| Biological Process | GO:0005977 glycogen metabolic process | 0.00496449 |
| Biological Process | GO:0006073 cellular glucan metabolic process | 0.00496449 |
| Biological Process | GO:0006091 generation of precursor metabolites and energy | 0.018655056 |
| Biological Process | GO:0006102 isocitrate metabolic process | 0.029868307 |
| Biological Process | GO:0006112 energy reserve metabolic process | 0.006687386 |
| Biological Process | GO:0006139 nucleobase-containing compound metabolic process | 0.009765036 |
| Biological Process | GO:0006259 DNA metabolic process | 5.97E-05 |
| Biological Process | GO:0006260 DNA replication | 0.025962277 |
| Biological Process | GO:0006275 regulation of DNA replication | 0.022128476 |
| Biological Process | GO:0006278 RNA-dependent DNA biosynthetic process | 0.03731412 |
| Biological Process | GO:0006281 DNA repair | 0.004980463 |
| Biological Process | GO:0006302 double-strand break repair | 0.039674089 |
| Biological Process | GO:0006325 chromatin organization | 0.013763142 |
| Biological Process | GO:0006401 RNA catabolic process | 0.005746038 |
| Biological Process | GO:0006402 mRNA catabolic process | 0.001723418 |
| Biological Process | GO:0006403 RNA localization | 0.016838193 |
| Biological Process | GO:0006417 regulation of translation | 0.003208519 |
| Biological Process | GO:0006429 leucyl-tRNA aminoacylation | 0.029868307 |
| Biological Process | GO:0006446 regulation of translational initiation | 0.019254256 |
| Biological Process | GO:0006464 cellular protein modification process | 7.54E-05 |
| Biological Process | GO:0006474 N-terminal protein amino acid acetylation | 0.015761503 |
| Biological Process | GO:0006479 protein methylation | 0.018057702 |
| Biological Process | GO:0006482 protein demethylation | 0.006053953 |
| Biological Process | GO:0006511 ubiquitin-dependent protein catabolic process | 0.002323764 |
| Biological Process | GO:0006611 protein export from nucleus | 0.043716212 |
| Biological Process | GO:0006657 CDP-choline pathway | 0.029868307 |
| Biological Process | GO:0006702 androgen biosynthetic process | 0.029868307 |
| Biological Process | GO:0006725 cellular aromatic compound metabolic process | 0.01476534 |
| Biological Process | GO:0006777 Mo-molybdopterin cofactor biosynthetic process | 0.002187463 |
| Biological Process | GO:0006793 phosphorus metabolic process | 0.033857762 |
| Biological Process | GO:0006796 phosphate-containing compound metabolic process | 0.031271468 |
| Biological Process | GO:0006807 nitrogen compound metabolic process | 0.006295737 |
| Biological Process | GO:0006810 transport | 0.031978713 |
| Biological Process | GO:0006816 calcium ion transport | 0.012597091 |
| Biological Process | GO:0006885 regulation of pH | 0.04543907 |
| Biological Process | GO:0006887 exocytosis | 0.000402314 |
| Biological Process | GO:0006888 ER to Golgi vesicle-mediated transport | 0.012608487 |
| Biological Process | GO:0006890 retrograde vesicle-mediated transport, Golgi to ER | 0.006768609 |
| Biological Process | GO:0006892 post-Golgi vesicle-mediated transport | 0.001175601 |
| Biological Process | GO:0006903 vesicle targeting | 0.000492238 |
| Biological Process | GO:0006904 vesicle docking involved in exocytosis | 0.02943371 |
| Biological Process | GO:0006914 autophagy | 0.001296605 |
| Biological Process | GO:0006915 apoptotic process | 0.049676649 |
| Biological Process | GO:0006950 response to stress | 0.014883631 |
| Biological Process | GO:0006974 cellular response to DNA damage stimulus | 0.010866458 |
| Biological Process | GO:0006984 ER-nucleus signaling pathway | 0.015435969 |
| Biological Process | GO:0006996 organelle organization | 2.89E-10 |
| Biological Process | GO:0007004 telomere maintenance via telomerase | 0.033558954 |
| Biological Process | GO:0007010 cytoskeleton organization | 0.000361597 |
| Biological Process | GO:0007020 microtubule nucleation | 0.042734991 |
| Biological Process | GO:0007026 negative regulation of microtubule depolymerization | 0.02943371 |
| Biological Process | GO:0007029 endoplasmic reticulum organization | 0.04543907 |
| Biological Process | GO:0007030 Golgi organization | 0.005418343 |
| Biological Process | GO:0007049 cell cycle | 0.044448662 |
| Biological Process | GO:0007062 sister chromatid cohesion | 0.031759239 |
| Biological Process | GO:0007064 mitotic sister chromatid cohesion | 0.042734991 |
| Biological Process | GO:0007163 establishment or maintenance of cell polarity | 0.01288057 |
| Biological Process | GO:0007172 signal complex assembly | 0.029868307 |
| Biological Process | GO:0007249 I-kappaB kinase/NF-kappaB signaling | 0.03498131 |
| Biological Process | GO:0007405 neuroblast proliferation | 0.025084522 |
| Biological Process | GO:0007417 central nervous system development | 0.040766556 |
| Biological Process | GO:0007420 brain development | 0.019792244 |
| Biological Process | GO:0007498 mesoderm development | 0.048423112 |
| Biological Process | GO:0007565 female pregnancy | 0.005418343 |
| Biological Process | GO:0007566 embryo implantation | 0.016656127 |
| Biological Process | GO:0007568 aging | 0.005809579 |
| Biological Process | GO:0007569 cell aging | 0.019198955 |
| Biological Process | GO:0008104 protein localization | 0.022235341 |
| Biological Process | GO:0008152 metabolic process | 0.000175945 |
| Biological Process | GO:0008213 protein alkylation | 0.018057702 |
| Biological Process | GO:0008214 protein dealkylation | 0.006053953 |
| Biological Process | GO:0008219 cell death | 0.031993321 |
| Biological Process | GO:0008340 determination of adult lifespan | 0.020803498 |
| Biological Process | GO:0008360 regulation of cell shape | 0.001660201 |
| Biological Process | GO:0008630 intrinsic apoptotic signaling pathway in response to DNA damage | 0.024224968 |
| Biological Process | GO:0009056 catabolic process | 0.002027588 |
| Biological Process | GO:0009057 macromolecule catabolic process | 0.000569195 |
| Biological Process | GO:0009314 response to radiation | 0.019912161 |
| Biological Process | GO:0009411 response to UV | 0.006072755 |
| Biological Process | GO:0009628 response to abiotic stimulus | 0.039235616 |
| Biological Process | GO:0009725 response to hormone | 0.025431032 |
| Biological Process | GO:0009889 regulation of biosynthetic process | 0.047069969 |
| Biological Process | GO:0009892 negative regulation of metabolic process | 0.033596916 |
| Biological Process | GO:0009894 regulation of catabolic process | 0.043181261 |
| Biological Process | GO:0009896 positive regulation of catabolic process | 0.035379183 |
| Biological Process | GO:0009967 positive regulation of signal transduction | 0.03217823 |
| Biological Process | GO:0009987 cellular process | 0.00484777 |
| Biological Process | GO:0010212 response to ionizing radiation | 0.002360771 |
| Biological Process | GO:0010259 multicellular organism aging | 0.006768609 |
| Biological Process | GO:0010468 regulation of gene expression | 0.024567246 |
| Biological Process | GO:0010501 RNA secondary structure unwinding | 0.004503174 |
| Biological Process | GO:0010556 regulation of macromolecule biosynthetic process | 0.030299815 |
| Biological Process | GO:0010558 negative regulation of macromolecule biosynthetic process | 0.032786136 |
| Biological Process | GO:0010586 miRNA metabolic process | 0.042734991 |
| Biological Process | GO:0010594 regulation of endothelial cell migration | 0.038254788 |
| Biological Process | GO:0010605 negative regulation of macromolecule metabolic process | 0.013237716 |
| Biological Process | GO:0010606 positive regulation of cytoplasmic mRNA processing body assembly | 0.04446773 |
| Biological Process | GO:0010608 posttranscriptional regulation of gene expression | 0.001502551 |
| Biological Process | GO:0010629 negative regulation of gene expression | 0.011253628 |
| Biological Process | GO:0010631 epithelial cell migration | 0.042399379 |
| Biological Process | GO:0010632 regulation of epithelial cell migration | 0.012099345 |
| Biological Process | GO:0010638 positive regulation of organelle organization | 0.000874551 |
| Biological Process | GO:0010647 positive regulation of cell communication | 0.02700376 |
| Biological Process | GO:0010737 protein kinase A signaling | 0.02943371 |
| Biological Process | GO:0010762 regulation of fibroblast migration | 0.035843078 |
| Biological Process | GO:0010833 telomere maintenance via telomere lengthening | 0.001508872 |
| Biological Process | GO:0010927 cellular component assembly involved in morphogenesis | 0.04980314 |
| Biological Process | GO:0010956 negative regulation of calcidiol 1-monooxygenase activity | 0.04446773 |
| Biological Process | GO:0010959 regulation of metal ion transport | 0.033309499 |
| Biological Process | GO:0010961 cellular magnesium ion homeostasis | 0.029868307 |
| Biological Process | GO:0012501 programmed cell death | 0.039958862 |
| Biological Process | GO:0014721 twitch skeletal muscle contraction | 0.04446773 |
| Biological Process | GO:0014722 regulation of skeletal muscle contraction by calcium ion signaling | 0.04446773 |
| Biological Process | GO:0014724 regulation of twitch skeletal muscle contraction | 0.029868307 |
| Biological Process | GO:0014809 regulation of skeletal muscle contraction by regulation of release of sequestered calcium ion | 0.029868307 |
| Biological Process | GO:0014819 regulation of skeletal muscle contraction | 0.011335565 |
| Biological Process | GO:0014916 regulation of lung blood pressure | 0.029868307 |
| Biological Process | GO:0015914 phospholipid transport | 0.043331851 |
| Biological Process | GO:0015980 energy derivation by oxidation of organic compounds | 0.031497205 |
| Biological Process | GO:0016043 cellular component organization | 4.64E-09 |
| Biological Process | GO:0016050 vesicle organization | 0.03320957 |
| Biological Process | GO:0016071 mRNA metabolic process | 0.002116811 |
| Biological Process | GO:0016192 vesicle-mediated transport | 0.000144851 |
| Biological Process | GO:0016197 endosomal transport | 0.001205961 |
| Biological Process | GO:0016236 macroautophagy | 0.003154975 |
| Biological Process | GO:0016240 autophagosome membrane docking | 0.003248694 |
| Biological Process | GO:0016340 calcium-dependent cell-matrix adhesion | 0.01504685 |
| Biological Process | GO:0016441 posttranscriptional gene silencing | 0.002376843 |
| Biological Process | GO:0016458 gene silencing | 0.024416041 |
| Biological Process | GO:0016482 cytosolic transport | 0.001291715 |
| Biological Process | GO:0016556 mRNA modification | 0.04635113 |
| Biological Process | GO:0016567 protein ubiquitination | 0.017938245 |
| Biological Process | GO:0016569 covalent chromatin modification | 0.005979854 |
| Biological Process | GO:0016570 histone modification | 0.004752783 |
| Biological Process | GO:0016571 histone methylation | 0.026156381 |
| Biological Process | GO:0016577 histone demethylation | 0.004765527 |
| Biological Process | GO:0017148 negative regulation of translation | 2.65E-05 |
| Biological Process | GO:0017157 regulation of exocytosis | 0.002465331 |
| Biological Process | GO:0018076 N-terminal peptidyl-lysine acetylation | 0.04446773 |
| Biological Process | GO:0018105 peptidyl-serine phosphorylation | 0.008634729 |
| Biological Process | GO:0018107 peptidyl-threonine phosphorylation | 0.028768115 |
| Biological Process | GO:0018125 peptidyl-cysteine methylation | 0.029868307 |
| Biological Process | GO:0018193 peptidyl-amino acid modification | 0.009350595 |
| Biological Process | GO:0018209 peptidyl-serine modification | 0.013904611 |
| Biological Process | GO:0018210 peptidyl-threonine modification | 0.008697515 |
| Biological Process | GO:0018282 metal incorporation into metallo-sulfur cluster | 0.029868307 |
| Biological Process | GO:0018283 iron incorporation into metallo-sulfur cluster | 0.029868307 |
| Biological Process | GO:0019072 viral genome packaging | 0.029868307 |
| Biological Process | GO:0019074 viral RNA genome packaging | 0.029868307 |
| Biological Process | GO:0019219 regulation of nucleobase-containing compound metabolic process | 0.007733334 |
| Biological Process | GO:0019222 regulation of metabolic process | 0.025734159 |
| Biological Process | GO:0019538 protein metabolic process | 1.51E-05 |
| Biological Process | GO:0019720 Mo-molybdopterin cofactor metabolic process | 0.002187463 |
| Biological Process | GO:0019722 calcium-mediated signaling | 0.030848123 |
| Biological Process | GO:0019827 stem cell population maintenance | 0.005069984 |
| Biological Process | GO:0019941 modification-dependent protein catabolic process | 0.002732193 |
| Biological Process | GO:0021998 neural plate mediolateral regionalization | 0.01504685 |
| Biological Process | GO:0022406 membrane docking | 0.004227359 |
| Biological Process | GO:0022411 cellular component disassembly | 0.000458148 |
| Biological Process | GO:0022414 reproductive process | 0.020523011 |
| Biological Process | GO:0022603 regulation of anatomical structure morphogenesis | 0.0057031 |
| Biological Process | GO:0022604 regulation of cell morphogenesis | 0.00999465 |
| Biological Process | GO:0022607 cellular component assembly | 0.030014023 |
| Biological Process | GO:0022613 ribonucleoprotein complex biogenesis | 0.047442009 |
| Biological Process | GO:0022618 ribonucleoprotein complex assembly | 0.005692326 |
| Biological Process | GO:0022898 regulation of transmembrane transporter activity | 0.039394163 |
| Biological Process | GO:0023056 positive regulation of signaling | 0.028584792 |
| Biological Process | GO:0030001 metal ion transport | 0.03539381 |
| Biological Process | GO:0030004 cellular monovalent inorganic cation homeostasis | 0.030011756 |
| Biological Process | GO:0030029 actin filament-based process | 0.034424372 |
| Biological Process | GO:0030036 actin cytoskeleton organization | 0.017938245 |
| Biological Process | GO:0030155 regulation of cell adhesion | 0.017252879 |
| Biological Process | GO:0030163 protein catabolic process | 0.031798669 |
| Biological Process | GO:0030177 positive regulation of Wnt signaling pathway | 0.019649294 |
| Biological Process | GO:0030212 hyaluronan metabolic process | 0.02943371 |
| Biological Process | GO:0030213 hyaluronan biosynthetic process | 0.005944903 |
| Biological Process | GO:0030242 autophagy of peroxisome | 0.029868307 |
| Biological Process | GO:0030258 lipid modification | 0.023803112 |
| Biological Process | GO:0030334 regulation of cell migration | 0.03971404 |
| Biological Process | GO:0030423 targeting of mRNA for destruction involved in RNA interference | 0.04446773 |
| Biological Process | GO:0030641 regulation of cellular pH | 0.015435969 |
| Biological Process | GO:0030728 ovulation | 0.013468928 |
| Biological Process | GO:0030846 termination of RNA polymerase II transcription, poly(A)-coupled | 0.04446773 |
| Biological Process | GO:0030859 polarized epithelial cell differentiation | 0.03257587 |
| Biological Process | GO:0031047 gene silencing by RNA | 0.003127529 |
| Biological Process | GO:0031054 pre-miRNA processing | 0.011335565 |
| Biological Process | GO:0031056 regulation of histone modification | 0.028912419 |
| Biological Process | GO:0031063 regulation of histone deacetylation | 0.042734991 |
| Biological Process | GO:0031065 positive regulation of histone deacetylation | 0.023542815 |
| Biological Process | GO:0031086 nuclear-transcribed mRNA catabolic process, deadenylation-independent decay | 0.029868307 |
| Biological Process | GO:0031087 deadenylation-independent decapping of nuclear-transcribed mRNA | 0.029868307 |
| Biological Process | GO:0031112 positive regulation of microtubule polymerization or depolymerization | 0.042734991 |
| Biological Process | GO:0031114 regulation of microtubule depolymerization | 0.02943371 |
| Biological Process | GO:0031116 positive regulation of microtubule polymerization | 0.035843078 |
| Biological Process | GO:0031323 regulation of cellular metabolic process | 0.03917391 |
| Biological Process | GO:0031326 regulation of cellular biosynthetic process | 0.038125456 |
| Biological Process | GO:0031327 negative regulation of cellular biosynthetic process | 0.048219173 |
| Biological Process | GO:0031329 regulation of cellular catabolic process | 0.02812796 |
| Biological Process | GO:0031331 positive regulation of cellular catabolic process | 0.015230434 |
| Biological Process | GO:0031334 positive regulation of protein complex assembly | 0.019780438 |
| Biological Process | GO:0031365 N-terminal protein amino acid modification | 0.039230896 |
| Biological Process | GO:0031440 regulation of mRNA 3'-end processing | 0.04635113 |
| Biological Process | GO:0031443 fast-twitch skeletal muscle fiber contraction | 0.029868307 |
| Biological Process | GO:0031446 regulation of fast-twitch skeletal muscle fiber contraction | 0.029868307 |
| Biological Process | GO:0031448 positive regulation of fast-twitch skeletal muscle fiber contraction | 0.029868307 |
| Biological Process | GO:0031554 regulation of DNA-templated transcription, termination | 0.04446773 |
| Biological Process | GO:0031647 regulation of protein stability | 0.014274418 |
| Biological Process | GO:0032091 negative regulation of protein binding | 0.031216809 |
| Biological Process | GO:0032147 activation of protein kinase activity | 0.024508923 |
| Biological Process | GO:0032200 telomere organization | 0.000440464 |
| Biological Process | GO:0032204 regulation of telomere maintenance | 0.000251167 |
| Biological Process | GO:0032206 positive regulation of telomere maintenance | 0.002982079 |
| Biological Process | GO:0032210 regulation of telomere maintenance via telomerase | 0.022063605 |
| Biological Process | GO:0032212 positive regulation of telomere maintenance via telomerase | 0.008342776 |
| Biological Process | GO:0032259 methylation | 0.023262622 |
| Biological Process | GO:0032268 regulation of cellular protein metabolic process | 0.024923602 |
| Biological Process | GO:0032269 negative regulation of cellular protein metabolic process | 0.031409626 |
| Biological Process | GO:0032324 molybdopterin cofactor biosynthetic process | 0.029868307 |
| Biological Process | GO:0032392 DNA geometric change | 0.012088529 |
| Biological Process | GO:0032411 positive regulation of transporter activity | 0.04980314 |
| Biological Process | GO:0032412 regulation of ion transmembrane transporter activity | 0.03495916 |
| Biological Process | GO:0032414 positive regulation of ion transmembrane transporter activity | 0.039269018 |
| Biological Process | GO:0032434 regulation of proteasomal ubiquitin-dependent protein catabolic process | 0.046824157 |
| Biological Process | GO:0032446 protein modification by small protein conjugation | 0.036379819 |
| Biological Process | GO:0032456 endocytic recycling | 0.033558954 |
| Biological Process | GO:0032465 regulation of cytokinesis | 0.015686165 |
| Biological Process | GO:0032468 Golgi calcium ion homeostasis | 0.01504685 |
| Biological Process | GO:0032469 endoplasmic reticulum calcium ion homeostasis | 0.03257587 |
| Biological Process | GO:0032470 positive regulation of endoplasmic reticulum calcium ion concentration | 0.00066946 |
| Biological Process | GO:0032472 Golgi calcium ion transport | 0.029868307 |
| Biological Process | GO:0032870 cellular response to hormone stimulus | 0.023236898 |
| Biological Process | GO:0032879 regulation of localization | 0.009325748 |
| Biological Process | GO:0032881 regulation of polysaccharide metabolic process | 0.039230896 |
| Biological Process | GO:0032885 regulation of polysaccharide biosynthetic process | 0.03257587 |
| Biological Process | GO:0032886 regulation of microtubule-based process | 0.017728756 |
| Biological Process | GO:0032915 positive regulation of transforming growth factor beta2 production | 0.04446773 |
| Biological Process | GO:0032940 secretion by cell | 0.009413488 |
| Biological Process | GO:0032984 protein-containing complex disassembly | 6.06E-05 |
| Biological Process | GO:0033003 regulation of mast cell activation | 0.035843078 |
| Biological Process | GO:0033006 regulation of mast cell activation involved in immune response | 0.02943371 |
| Biological Process | GO:0033036 macromolecule localization | 0.005464875 |
| Biological Process | GO:0033043 regulation of organelle organization | 0.000664052 |
| Biological Process | GO:0033044 regulation of chromosome organization | 0.000509015 |
| Biological Process | GO:0033168 conversion of ds siRNA to ss siRNA involved in RNA interference | 0.01504685 |
| Biological Process | GO:0033169 histone H3-K9 demethylation | 0.009366768 |
| Biological Process | GO:0033365 protein localization to organelle | 0.046134881 |
| Biological Process | GO:0033540 fatty acid beta-oxidation using acyl-CoA oxidase | 0.04446773 |
| Biological Process | GO:0033554 cellular response to stress | 0.001931348 |
| Biological Process | GO:0033962 cytoplasmic mRNA processing body assembly | 0.015761503 |
| Biological Process | GO:0034067 protein localization to Golgi apparatus | 0.03257587 |
| Biological Process | GO:0034110 regulation of homotypic cell-cell adhesion | 0.035843078 |
| Biological Process | GO:0034204 lipid translocation | 0.023542815 |
| Biological Process | GO:0034248 regulation of cellular amide metabolic process | 0.01229354 |
| Biological Process | GO:0034249 negative regulation of cellular amide metabolic process | 0.000105986 |
| Biological Process | GO:0034394 protein localization to cell surface | 0.003888178 |
| Biological Process | GO:0034421 post-translational protein acetylation | 0.04446773 |
| Biological Process | GO:0034613 cellular protein localization | 0.020166415 |
| Biological Process | GO:0034622 cellular protein-containing complex assembly | 0.021295233 |
| Biological Process | GO:0034644 cellular response to UV | 0.002733564 |
| Biological Process | GO:0034655 nucleobase-containing compound catabolic process | 0.045212439 |
| Biological Process | GO:0034699 response to luteinizing hormone | 0.01504685 |
| Biological Process | GO:0034720 histone H3-K4 demethylation | 0.004503174 |
| Biological Process | GO:0034767 positive regulation of ion transmembrane transport | 0.035113375 |
| Biological Process | GO:0034968 histone lysine methylation | 0.045255178 |
| Biological Process | GO:0035087 siRNA loading onto RISC involved in RNA interference | 0.04446773 |
| Biological Process | GO:0035107 appendage morphogenesis | 0.03286346 |
| Biological Process | GO:0035108 limb morphogenesis | 0.03286346 |
| Biological Process | GO:0035194 posttranscriptional gene silencing by RNA | 0.002055234 |
| Biological Process | GO:0035195 gene silencing by miRNA | 0.001766613 |
| Biological Process | GO:0035261 external genitalia morphogenesis | 0.01504685 |
| Biological Process | GO:0035278 miRNA mediated inhibition of translation | 0.001082145 |
| Biological Process | GO:0035280 miRNA loading onto RISC involved in gene silencing by miRNA | 0.002187463 |
| Biological Process | GO:0035332 positive regulation of hippo signaling | 0.04446773 |
| Biological Process | GO:0035406 histone-tyrosine phosphorylation | 0.01504685 |
| Biological Process | GO:0035409 histone H3-Y41 phosphorylation | 0.01504685 |
| Biological Process | GO:0035556 intracellular signal transduction | 0.017939433 |
| Biological Process | GO:0035712 T-helper 2 cell activation | 0.01504685 |
| Biological Process | GO:0035722 interleukin-12-mediated signaling pathway | 0.029868307 |
| Biological Process | GO:0035855 megakaryocyte development | 0.026421141 |
| Biological Process | GO:0035964 COPI-coated vesicle budding | 0.04446773 |
| Biological Process | GO:0035994 response to muscle stretch | 0.001337738 |
| Biological Process | GO:0036092 phosphatidylinositol-3-phosphate biosynthetic process | 0.029868307 |
| Biological Process | GO:0036211 protein modification process | 7.54E-05 |
| Biological Process | GO:0036404 conversion of ds siRNA to ss siRNA | 0.01504685 |
| Biological Process | GO:0036506 maintenance of unfolded protein | 0.029868307 |
| Biological Process | GO:0038029 epidermal growth factor receptor signaling pathway via MAPK cascade | 0.01504685 |
| Biological Process | GO:0040012 regulation of locomotion | 0.033965689 |
| Biological Process | GO:0040029 regulation of gene expression, epigenetic | 0.023111076 |
| Biological Process | GO:0040033 negative regulation of translation, ncRNA-mediated | 0.001082145 |
| Biological Process | GO:0042126 nitrate metabolic process | 0.029868307 |
| Biological Process | GO:0042147 retrograde transport, endosome to Golgi | 0.000251167 |
| Biological Process | GO:0042148 strand invasion | 0.029868307 |
| Biological Process | GO:0042273 ribosomal large subunit biogenesis | 0.02063249 |
| Biological Process | GO:0042692 muscle cell differentiation | 0.04894852 |
| Biological Process | GO:0042996 regulation of Golgi to plasma membrane protein transport | 0.007568012 |
| Biological Process | GO:0042998 positive regulation of Golgi to plasma membrane protein transport | 0.002187463 |
| Biological Process | GO:0043085 positive regulation of catalytic activity | 0.042073648 |
| Biological Process | GO:0043170 macromolecule metabolic process | 0.000274665 |
| Biological Process | GO:0043242 negative regulation of protein complex disassembly | 0.006687386 |
| Biological Process | GO:0043244 regulation of protein complex disassembly | 0.004830472 |
| Biological Process | GO:0043266 regulation of potassium ion transport | 0.028316703 |
| Biological Process | GO:0043269 regulation of ion transport | 0.04753566 |
| Biological Process | GO:0043270 positive regulation of ion transport | 0.020478565 |
| Biological Process | GO:0043303 mast cell degranulation | 0.04635113 |
| Biological Process | GO:0043304 regulation of mast cell degranulation | 0.023542815 |
| Biological Process | GO:0043412 macromolecule modification | 9.68E-05 |
| Biological Process | GO:0043414 macromolecule methylation | 0.014274418 |
| Biological Process | GO:0043434 response to peptide hormone | 0.003154975 |
| Biological Process | GO:0043545 molybdopterin cofactor metabolic process | 0.002187463 |
| Biological Process | GO:0043624 cellular protein complex disassembly | 0.035113375 |
| Biological Process | GO:0043632 modification-dependent macromolecule catabolic process | 0.002924473 |
| Biological Process | GO:0043933 protein-containing complex subunit organization | 8.54E-05 |
| Biological Process | GO:0044028 DNA hypomethylation | 0.01504685 |
| Biological Process | GO:0044029 hypomethylation of CpG island | 0.01504685 |
| Biological Process | GO:0044030 regulation of DNA methylation | 0.015761503 |
| Biological Process | GO:0044042 glucan metabolic process | 0.00496449 |
| Biological Process | GO:0044057 regulation of system process | 0.035379183 |
| Biological Process | GO:0044093 positive regulation of molecular function | 0.019924543 |
| Biological Process | GO:0044237 cellular metabolic process | 4.54E-05 |
| Biological Process | GO:0044238 primary metabolic process | 0.000439951 |
| Biological Process | GO:0044248 cellular catabolic process | 0.000523682 |
| Biological Process | GO:0044257 cellular protein catabolic process | 0.024405942 |
| Biological Process | GO:0044260 cellular macromolecule metabolic process | 0.000500936 |
| Biological Process | GO:0044264 cellular polysaccharide metabolic process | 0.011904937 |
| Biological Process | GO:0044265 cellular macromolecule catabolic process | 0.00130066 |
| Biological Process | GO:0044267 cellular protein metabolic process | 3.93E-05 |
| Biological Process | GO:0044313 protein K6-linked deubiquitination | 0.04446773 |
| Biological Process | GO:0044571 [2Fe-2S] cluster assembly | 0.04446773 |
| Biological Process | GO:0044703 multi-organism reproductive process | 0.008388526 |
| Biological Process | GO:0044706 multi-multicellular organism process | 0.01051978 |
| Biological Process | GO:0044806 G-quadruplex DNA unwinding | 0.002187463 |
| Biological Process | GO:0045186 zonula adherens assembly | 0.01504685 |
| Biological Process | GO:0045332 phospholipid translocation | 0.018208063 |
| Biological Process | GO:0045913 positive regulation of carbohydrate metabolic process | 0.025084522 |
| Biological Process | GO:0045935 positive regulation of nucleobase-containing compound metabolic process | 0.038363141 |
| Biological Process | GO:0045947 negative regulation of translational initiation | 0.005944903 |
| Biological Process | GO:0045974 regulation of translation, ncRNA-mediated | 0.001082145 |
| Biological Process | GO:0045995 regulation of embryonic development | 0.016179763 |
| Biological Process | GO:0046039 GTP metabolic process | 0.042734991 |
| Biological Process | GO:0046483 heterocycle metabolic process | 0.011902162 |
| Biological Process | GO:0046604 positive regulation of mitotic centrosome separation | 0.04446773 |
| Biological Process | GO:0046785 microtubule polymerization | 0.047596306 |
| Biological Process | GO:0046834 lipid phosphorylation | 0.011076044 |
| Biological Process | GO:0046854 phosphatidylinositol phosphorylation | 0.006053953 |
| Biological Process | GO:0046903 secretion | 0.008388526 |
| Biological Process | GO:0046907 intracellular transport | 0.008806488 |
| Biological Process | GO:0046928 regulation of neurotransmitter secretion | 0.04980314 |
| Biological Process | GO:0047496 vesicle transport along microtubule | 0.04635113 |
| Biological Process | GO:0048193 Golgi vesicle transport | 3.04E-05 |
| Biological Process | GO:0048200 Golgi transport vesicle coating | 0.029868307 |
| Biological Process | GO:0048205 COPI coating of Golgi vesicle | 0.029868307 |
| Biological Process | GO:0048239 negative regulation of DNA recombination at telomere | 0.029868307 |
| Biological Process | GO:0048338 mesoderm structural organization | 0.01504685 |
| Biological Process | GO:0048339 paraxial mesoderm development | 0.02943371 |
| Biological Process | GO:0048340 paraxial mesoderm morphogenesis | 0.011335565 |
| Biological Process | GO:0048352 paraxial mesoderm structural organization | 0.01504685 |
| Biological Process | GO:0048369 lateral mesoderm morphogenesis | 0.029868307 |
| Biological Process | GO:0048370 lateral mesoderm formation | 0.029868307 |
| Biological Process | GO:0048371 lateral mesodermal cell differentiation | 0.029868307 |
| Biological Process | GO:0048372 lateral mesodermal cell fate commitment | 0.01504685 |
| Biological Process | GO:0048377 lateral mesodermal cell fate specification | 0.01504685 |
| Biological Process | GO:0048378 regulation of lateral mesodermal cell fate specification | 0.01504685 |
| Biological Process | GO:0048514 blood vessel morphogenesis | 0.042968852 |
| Biological Process | GO:0048519 negative regulation of biological process | 0.001994032 |
| Biological Process | GO:0048522 positive regulation of cellular process | 0.023930355 |
| Biological Process | GO:0048523 negative regulation of cellular process | 0.012095376 |
| Biological Process | GO:0048736 appendage development | 0.021116566 |
| Biological Process | GO:0048821 erythrocyte development | 0.042734991 |
| Biological Process | GO:0048822 enucleate erythrocyte development | 0.029868307 |
| Biological Process | GO:0048856 anatomical structure development | 0.042023533 |
| Biological Process | GO:0048869 cellular developmental process | 0.037976094 |
| Biological Process | GO:0050673 epithelial cell proliferation | 0.032257187 |
| Biological Process | GO:0050678 regulation of epithelial cell proliferation | 0.01113371 |
| Biological Process | GO:0050679 positive regulation of epithelial cell proliferation | 0.01155671 |
| Biological Process | GO:0051046 regulation of secretion | 0.035037686 |
| Biological Process | GO:0051049 regulation of transport | 0.039391034 |
| Biological Process | GO:0051050 positive regulation of transport | 0.039460469 |
| Biological Process | GO:0051052 regulation of DNA metabolic process | 3.00E-06 |
| Biological Process | GO:0051054 positive regulation of DNA metabolic process | 0.014507491 |
| Biological Process | GO:0051095 regulation of helicase activity | 0.007568012 |
| Biological Process | GO:0051097 negative regulation of helicase activity | 0.04446773 |
| Biological Process | GO:0051128 regulation of cellular component organization | 0.000350893 |
| Biological Process | GO:0051130 positive regulation of cellular component organization | 0.003467888 |
| Biological Process | GO:0051148 negative regulation of muscle cell differentiation | 0.022063605 |
| Biological Process | GO:0051171 regulation of nitrogen compound metabolic process | 0.0172847 |
| Biological Process | GO:0051172 negative regulation of nitrogen compound metabolic process | 0.037907783 |
| Biological Process | GO:0051179 localization | 0.005916185 |
| Biological Process | GO:0051189 prosthetic group metabolic process | 0.002187463 |
| Biological Process | GO:0051234 establishment of localization | 0.032671753 |
| Biological Process | GO:0051246 regulation of protein metabolic process | 0.036906276 |
| Biological Process | GO:0051248 negative regulation of protein metabolic process | 0.026744546 |
| Biological Process | GO:0051261 protein depolymerization | 0.018279067 |
| Biological Process | GO:0051270 regulation of cellular component movement | 0.032038682 |
| Biological Process | GO:0051271 negative regulation of cellular component movement | 0.034076581 |
| Biological Process | GO:0051276 chromosome organization | 0.000510826 |
| Biological Process | GO:0051301 cell division | 0.01119363 |
| Biological Process | GO:0051302 regulation of cell division | 0.014308039 |
| Biological Process | GO:0051345 positive regulation of hydrolase activity | 0.039337165 |
| Biological Process | GO:0051354 negative regulation of oxidoreductase activity | 0.020803498 |
| Biological Process | GO:0051453 regulation of intracellular pH | 0.012088529 |
| Biological Process | GO:0051570 regulation of histone H3-K9 methylation | 0.035843078 |
| Biological Process | GO:0051588 regulation of neurotransmitter transport | 0.023162256 |
| Biological Process | GO:0051599 response to hydrostatic pressure | 0.04446773 |
| Biological Process | GO:0051603 proteolysis involved in cellular protein catabolic process | 0.012237595 |
| Biological Process | GO:0051640 organelle localization | 1.18E-05 |
| Biological Process | GO:0051641 cellular localization | 0.000423016 |
| Biological Process | GO:0051648 vesicle localization | 0.000297315 |
| Biological Process | GO:0051649 establishment of localization in cell | 0.004443062 |
| Biological Process | GO:0051650 establishment of vesicle localization | 0.000192582 |
| Biological Process | GO:0051651 maintenance of location in cell | 0.04980314 |
| Biological Process | GO:0051656 establishment of organelle localization | 8.92E-05 |
| Biological Process | GO:0051657 maintenance of organelle location | 0.009366768 |
| Biological Process | GO:0051659 maintenance of mitochondrion location | 0.029868307 |
| Biological Process | GO:0051684 maintenance of Golgi location | 0.04446773 |
| Biological Process | GO:0051726 regulation of cell cycle | 0.024512841 |
| Biological Process | GO:0051891 positive regulation of cardioblast differentiation | 0.04446773 |
| Biological Process | GO:0060037 pharyngeal system development | 0.035843078 |
| Biological Process | GO:0060173 limb development | 0.021116566 |
| Biological Process | GO:0060211 regulation of nuclear-transcribed mRNA poly(A) tail shortening | 0.005944903 |
| Biological Process | GO:0060213 positive regulation of nuclear-transcribed mRNA poly(A) tail shortening | 0.005944903 |
| Biological Process | GO:0060255 regulation of macromolecule metabolic process | 0.010913028 |
| Biological Process | GO:0060260 regulation of transcription initiation from RNA polymerase II promoter | 0.042734991 |
| Biological Process | GO:0060261 positive regulation of transcription initiation from RNA polymerase II promoter | 0.026421141 |
| Biological Process | GO:0060306 regulation of membrane repolarization | 0.026421141 |
| Biological Process | GO:0060322 head development | 0.01568909 |
| Biological Process | GO:0060341 regulation of cellular localization | 0.033814918 |
| Biological Process | GO:0060389 pathway-restricted SMAD protein phosphorylation | 0.026674248 |
| Biological Process | GO:0060390 regulation of SMAD protein signal transduction | 0.042734991 |
| Biological Process | GO:0060391 positive regulation of SMAD protein signal transduction | 0.011335565 |
| Biological Process | GO:0060396 growth hormone receptor signaling pathway | 0.007568012 |
| Biological Process | GO:0060416 response to growth hormone | 0.009366768 |
| Biological Process | GO:0060485 mesenchyme development | 0.024992486 |
| Biological Process | GO:0060542 regulation of strand invasion | 0.01504685 |
| Biological Process | GO:0060567 negative regulation of DNA-templated transcription, termination | 0.029868307 |
| Biological Process | GO:0060627 regulation of vesicle-mediated transport | 0.003298154 |
| Biological Process | GO:0060819 inactivation of X chromosome by genetic imprinting | 0.029868307 |
| Biological Process | GO:0060922 atrioventricular node cell differentiation | 0.04446773 |
| Biological Process | GO:0060928 atrioventricular node cell development | 0.04446773 |
| Biological Process | GO:0061010 gall bladder development | 0.029868307 |
| Biological Process | GO:0061013 regulation of mRNA catabolic process | 0.048423112 |
| Biological Process | GO:0061014 positive regulation of mRNA catabolic process | 0.015435969 |
| Biological Process | GO:0061024 membrane organization | 0.030150336 |
| Biological Process | GO:0061091 regulation of phospholipid translocation | 0.04446773 |
| Biological Process | GO:0061092 positive regulation of phospholipid translocation | 0.04446773 |
| Biological Process | GO:0061136 regulation of proteasomal protein catabolic process | 0.049251691 |
| Biological Process | GO:0061162 establishment of monopolar cell polarity | 0.035843078 |
| Biological Process | GO:0061298 retina vasculature development in camera-type eye | 0.015761503 |
| Biological Process | GO:0061339 establishment or maintenance of monopolar cell polarity | 0.039230896 |
| Biological Process | GO:0061384 heart trabecula morphogenesis | 0.042734991 |
| Biological Process | GO:0061515 myeloid cell development | 0.041275043 |
| Biological Process | GO:0061626 pharyngeal arch artery morphogenesis | 0.003248694 |
| Biological Process | GO:0061919 process utilizing autophagic mechanism | 0.001296605 |
| Biological Process | GO:0065003 protein-containing complex assembly | 0.002615793 |
| Biological Process | GO:0065008 regulation of biological quality | 0.004080308 |
| Biological Process | GO:0065009 regulation of molecular function | 0.044437476 |
| Biological Process | GO:0070059 intrinsic apoptotic signaling pathway in response to endoplasmic reticulum stress | 0.004585964 |
| Biological Process | GO:0070076 histone lysine demethylation | 0.004189952 |
| Biological Process | GO:0070162 adiponectin secretion | 0.01504685 |
| Biological Process | GO:0070163 regulation of adiponectin secretion | 0.01504685 |
| Biological Process | GO:0070164 negative regulation of adiponectin secretion | 0.01504685 |
| Biological Process | GO:0070296 sarcoplasmic reticulum calcium ion transport | 0.03257587 |
| Biological Process | GO:0070318 positive regulation of G0 to G1 transition | 0.029868307 |
| Biological Process | GO:0070646 protein modification by small protein removal | 0.041734523 |
| Biological Process | GO:0070647 protein modification by small protein conjugation or removal | 0.004785416 |
| Biological Process | GO:0070727 cellular macromolecule localization | 0.021395725 |
| Biological Process | GO:0070838 divalent metal ion transport | 0.025048211 |
| Biological Process | GO:0070889 platelet alpha granule organization | 0.029868307 |
| Biological Process | GO:0070922 small RNA loading onto RISC | 0.004503174 |
| Biological Process | GO:0070936 protein K48-linked ubiquitination | 0.047596306 |
| Biological Process | GO:0070972 protein localization to endoplasmic reticulum | 0.023547622 |
| Biological Process | GO:0070973 protein localization to endoplasmic reticulum exit site | 0.002187463 |
| Biological Process | GO:0070988 demethylation | 0.033558954 |
| Biological Process | GO:0071109 superior temporal gyrus development | 0.01504685 |
| Biological Process | GO:0071166 ribonucleoprotein complex localization | 0.04543907 |
| Biological Process | GO:0071214 cellular response to abiotic stimulus | 0.002900032 |
| Biological Process | GO:0071287 cellular response to manganese ion | 0.029868307 |
| Biological Process | GO:0071349 cellular response to interleukin-12 | 0.04446773 |
| Biological Process | GO:0071375 cellular response to peptide hormone stimulus | 0.005072934 |
| Biological Process | GO:0071378 cellular response to growth hormone stimulus | 0.007568012 |
| Biological Process | GO:0071417 cellular response to organonitrogen compound | 0.0360277 |
| Biological Process | GO:0071426 ribonucleoprotein complex export from nucleus | 0.043331851 |
| Biological Process | GO:0071462 cellular response to water stimulus | 0.04446773 |
| Biological Process | GO:0071464 cellular response to hydrostatic pressure | 0.029868307 |
| Biological Process | GO:0071478 cellular response to radiation | 0.001591908 |
| Biological Process | GO:0071479 cellular response to ionizing radiation | 0.035410668 |
| Biological Process | GO:0071480 cellular response to gamma radiation | 0.035843078 |
| Biological Process | GO:0071482 cellular response to light stimulus | 0.004830472 |
| Biological Process | GO:0071514 genetic imprinting | 0.03257587 |
| Biological Process | GO:0071550 death-inducing signaling complex assembly | 0.01504685 |
| Biological Process | GO:0071704 organic substance metabolic process | 0.000706153 |
| Biological Process | GO:0071711 basement membrane organization | 0.02943371 |
| Biological Process | GO:0071826 ribonucleoprotein complex subunit organization | 0.008170178 |
| Biological Process | GO:0071840 cellular component organization or biogenesis | 2.12E-08 |
| Biological Process | GO:0071896 protein localization to adherens junction | 0.04446773 |
| Biological Process | GO:0071897 DNA biosynthetic process | 0.008164148 |
| Biological Process | GO:0072091 regulation of stem cell proliferation | 0.043331851 |
| Biological Process | GO:0072350 tricarboxylic acid metabolic process | 0.04635113 |
| Biological Process | GO:0072358 cardiovascular system development | 0.0399914 |
| Biological Process | GO:0072359 circulatory system development | 0.022224065 |
| Biological Process | GO:0072383 plus-end-directed vesicle transport along microtubule | 0.00066946 |
| Biological Process | GO:0072386 plus-end-directed organelle transport along microtubule | 0.00066946 |
| Biological Process | GO:0072511 divalent inorganic cation transport | 0.025048211 |
| Biological Process | GO:0072695 regulation of DNA recombination at telomere | 0.029868307 |
| Biological Process | GO:0080090 regulation of primary metabolic process | 0.029034339 |
| Biological Process | GO:0080134 regulation of response to stress | 0.03685164 |
| Biological Process | GO:0086036 regulation of cardiac muscle cell membrane potential | 0.029868307 |
| Biological Process | GO:0090075 relaxation of muscle | 0.02943371 |
| Biological Process | GO:0090076 relaxation of skeletal muscle | 0.04446773 |
| Biological Process | GO:0090100 positive regulation of transmembrane receptor protein serine/threonine kinase signaling pathway | 0.032485788 |
| Biological Process | GO:0090130 tissue migration | 0.046067582 |
| Biological Process | GO:0090132 epithelium migration | 0.044208891 |
| Biological Process | GO:0090263 positive regulation of canonical Wnt signaling pathway | 0.007106217 |
| Biological Process | GO:0090304 nucleic acid metabolic process | 0.008698911 |
| Biological Process | GO:0090312 positive regulation of protein deacetylation | 0.039230896 |
| Biological Process | GO:0090330 regulation of platelet aggregation | 0.023542815 |
| Biological Process | GO:0090370 negative regulation of cholesterol efflux | 0.04446773 |
| Biological Process | GO:0090522 vesicle tethering involved in exocytosis | 0.01504685 |
| Biological Process | GO:0097111 endoplasmic reticulum-Golgi intermediate compartment organization | 0.029868307 |
| Biological Process | GO:0097193 intrinsic apoptotic signaling pathway | 0.015035479 |
| Biological Process | GO:0097352 autophagosome maturation | 0.001005458 |
| Biological Process | GO:0097435 supramolecular fiber organization | 0.027764374 |
| Biological Process | GO:0097692 histone H3-K4 monomethylation | 0.002187463 |
| Biological Process | GO:0098530 positive regulation of strand invasion | 0.01504685 |
| Biological Process | GO:0098629 trans-Golgi network membrane organization | 0.029868307 |
| Biological Process | GO:0098660 inorganic ion transmembrane transport | 0.033013179 |
| Biological Process | GO:0098662 inorganic cation transmembrane transport | 0.020827578 |
| Biological Process | GO:0098727 maintenance of cell number | 0.00555483 |
| Biological Process | GO:0098885 modification of postsynaptic actin cytoskeleton | 0.04446773 |
| Biological Process | GO:0099022 vesicle tethering | 0.005944903 |
| Biological Process | GO:0099039 sphingolipid translocation | 0.01504685 |
| Biological Process | GO:0104004 cellular response to environmental stimulus | 0.002900032 |
| Biological Process | GO:0120040 regulation of macrophage proliferation | 0.04446773 |
| Biological Process | GO:0120041 positive regulation of macrophage proliferation | 0.04446773 |
| Biological Process | GO:0120191 negative regulation of termination of RNA polymerase II transcription | 0.029868307 |
| Biological Process | GO:0140029 exocytic process | 0.031759239 |
| Biological Process | GO:0140056 organelle localization by membrane tethering | 0.002014225 |
| Biological Process | GO:0140115 export across plasma membrane | 0.03257587 |
| Biological Process | GO:1900125 regulation of hyaluronan biosynthetic process | 0.002187463 |
| Biological Process | GO:1900127 positive regulation of hyaluronan biosynthetic process | 0.00066946 |
| Biological Process | GO:1900151 regulation of nuclear-transcribed mRNA catabolic process, deadenylation-dependent decay | 0.000668754 |
| Biological Process | GO:1900153 positive regulation of nuclear-transcribed mRNA catabolic process, deadenylation-dependent decay | 0.000668754 |
| Biological Process | GO:1900245 positive regulation of MDA-5 signaling pathway | 0.04446773 |
| Biological Process | GO:1901016 regulation of potassium ion transmembrane transporter activity | 0.00920367 |
| Biological Process | GO:1901021 positive regulation of calcium ion transmembrane transporter activity | 0.039230896 |
| Biological Process | GO:1901360 organic cyclic compound metabolic process | 0.020280077 |
| Biological Process | GO:1901379 regulation of potassium ion transmembrane transport | 0.022063605 |
| Biological Process | GO:1901532 regulation of hematopoietic progenitor cell differentiation | 0.04635113 |
| Biological Process | GO:1901534 positive regulation of hematopoietic progenitor cell differentiation | 0.04446773 |
| Biological Process | GO:1901564 organonitrogen compound metabolic process | 0.000115028 |
| Biological Process | GO:1901575 organic substance catabolic process | 0.016911407 |
| Biological Process | GO:1901652 response to peptide | 0.013186071 |
| Biological Process | GO:1901653 cellular response to peptide | 0.014084159 |
| Biological Process | GO:1901701 cellular response to oxygen-containing compound | 0.043693671 |
| Biological Process | GO:1901879 regulation of protein depolymerization | 0.035410668 |
| Biological Process | GO:1901880 negative regulation of protein depolymerization | 0.023547622 |
| Biological Process | GO:1901894 regulation of calcium-transporting ATPase activity | 0.04446773 |
| Biological Process | GO:1901896 positive regulation of calcium-transporting ATPase activity | 0.029868307 |
| Biological Process | GO:1902042 negative regulation of extrinsic apoptotic signaling pathway via death domain receptors | 0.035843078 |
| Biological Process | GO:1902064 regulation of transcription from RNA polymerase II promoter involved in spermatogenesis | 0.01504685 |
| Biological Process | GO:1902230 negative regulation of intrinsic apoptotic signaling pathway in response to DNA damage | 0.042734991 |
| Biological Process | GO:1902261 positive regulation of delayed rectifier potassium channel activity | 0.04446773 |
| Biological Process | GO:1902499 positive regulation of protein autoubiquitination | 0.04446773 |
| Biological Process | GO:1902692 regulation of neuroblast proliferation | 0.04635113 |
| Biological Process | GO:1902731 negative regulation of chondrocyte proliferation | 0.04446773 |
| Biological Process | GO:1902775 mitochondrial large ribosomal subunit assembly | 0.04446773 |
| Biological Process | GO:1902803 regulation of synaptic vesicle transport | 0.033558954 |
| Biological Process | GO:1903050 regulation of proteolysis involved in cellular protein catabolic process | 0.028248103 |
| Biological Process | GO:1903052 positive regulation of proteolysis involved in cellular protein catabolic process | 0.024224968 |
| Biological Process | GO:1903078 positive regulation of protein localization to plasma membrane | 0.026674248 |
| Biological Process | GO:1903123 regulation of thioredoxin peroxidase activity | 0.01504685 |
| Biological Process | GO:1903124 negative regulation of thioredoxin peroxidase activity | 0.01504685 |
| Biological Process | GO:1903125 negative regulation of thioredoxin peroxidase activity by peptidyl-threonine phosphorylation | 0.01504685 |
| Biological Process | GO:1903233 regulation of calcium ion-dependent exocytosis of neurotransmitter | 0.029868307 |
| Biological Process | GO:1903305 regulation of regulated secretory pathway | 0.01051978 |
| Biological Process | GO:1903364 positive regulation of cellular protein catabolic process | 0.046824157 |
| Biological Process | GO:1903420 protein localization to endoplasmic reticulum tubular network | 0.01504685 |
| Biological Process | GO:1903515 calcium ion transport from cytosol to endoplasmic reticulum | 0.029868307 |
| Biological Process | GO:1903523 negative regulation of blood circulation | 0.02943371 |
| Biological Process | GO:1903530 regulation of secretion by cell | 0.046752604 |
| Biological Process | GO:1903593 regulation of histamine secretion by mast cell | 0.04446773 |
| Biological Process | GO:1903595 positive regulation of histamine secretion by mast cell | 0.04446773 |
| Biological Process | GO:1903690 negative regulation of wound healing, spreading of epidermal cells | 0.029868307 |
| Biological Process | GO:1903827 regulation of cellular protein localization | 0.023731052 |
| Biological Process | GO:1903898 negative regulation of PERK-mediated unfolded protein response | 0.04446773 |
| Biological Process | GO:1903998 regulation of eating behavior | 0.01504685 |
| Biological Process | GO:1903999 negative regulation of eating behavior | 0.01504685 |
| Biological Process | GO:1904064 positive regulation of cation transmembrane transport | 0.025316771 |
| Biological Process | GO:1904356 regulation of telomere maintenance via telomere lengthening | 0.004585964 |
| Biological Process | GO:1904358 positive regulation of telomere maintenance via telomere lengthening | 0.001005458 |
| Biological Process | GO:1904377 positive regulation of protein localization to cell periphery | 0.03731412 |
| Biological Process | GO:1904378 maintenance of unfolded protein involved in ERAD pathway | 0.029868307 |
| Biological Process | GO:1904412 regulation of cardiac ventricle development | 0.01504685 |
| Biological Process | GO:1904414 positive regulation of cardiac ventricle development | 0.01504685 |
| Biological Process | GO:1904427 positive regulation of calcium ion transmembrane transport | 0.023547622 |
| Biological Process | GO:1904594 regulation of termination of RNA polymerase II transcription | 0.04446773 |
| Biological Process | GO:1904691 negative regulation of type B pancreatic cell proliferation | 0.029868307 |
| Biological Process | GO:1904779 regulation of protein localization to centrosome | 0.009366768 |
| Biological Process | GO:1904780 negative regulation of protein localization to centrosome | 0.029868307 |
| Biological Process | GO:1904887 Wnt signalosome assembly | 0.01504685 |
| Biological Process | GO:1905037 autophagosome organization | 0.019198955 |
| Biological Process | GO:1905285 fibrous ring of heart morphogenesis | 0.01504685 |
| Biological Process | GO:1905289 regulation of CAMKK-AMPK signaling cascade | 0.029868307 |
| Biological Process | GO:1905463 negative regulation of DNA duplex unwinding | 0.04446773 |
| Biological Process | GO:1905616 regulation of miRNA mediated inhibition of translation | 0.004503174 |
| Biological Process | GO:1905618 positive regulation of miRNA mediated inhibition of translation | 0.004503174 |
| Biological Process | GO:1905775 negative regulation of DNA helicase activity | 0.029868307 |
| Biological Process | GO:1905799 regulation of intraciliary retrograde transport | 0.01504685 |
| Biological Process | GO:1990258 histone glutamine methylation | 0.04446773 |
| Biological Process | GO:1990441 negative regulation of transcription from RNA polymerase II promoter in response to endoplasmic reticulum stress | 0.04446773 |
| Biological Process | GO:1990456 mitochondrion-endoplasmic reticulum membrane tethering | 0.003248694 |
| Biological Process | GO:1990962 drug transport across blood-brain barrier | 0.029868307 |
| Biological Process | GO:2000008 regulation of protein localization to cell surface | 0.006053953 |
| Biological Process | GO:2000058 regulation of ubiquitin-dependent protein catabolic process | 0.024416041 |
| Biological Process | GO:2000112 regulation of cellular macromolecule biosynthetic process | 0.026232981 |
| Biological Process | GO:2000113 negative regulation of cellular macromolecule biosynthetic process | 0.03027049 |
| Biological Process | GO:2000144 positive regulation of DNA-templated transcription, initiation | 0.039230896 |
| Biological Process | GO:2000209 regulation of anoikis | 0.026421141 |
| Biological Process | GO:2000252 negative regulation of feeding behavior | 0.029868307 |
| Biological Process | GO:2000278 regulation of DNA biosynthetic process | 0.031216809 |
| Biological Process | GO:2000300 regulation of synaptic vesicle exocytosis | 0.033558954 |
| Biological Process | GO:2000312 regulation of kainate selective glutamate receptor activity | 0.04446773 |
| Biological Process | GO:2000318 positive regulation of T-helper 17 type immune response | 0.04446773 |
| Biological Process | GO:2000380 regulation of mesoderm development | 0.013468928 |
| Biological Process | GO:2000468 regulation of peroxidase activity | 0.04446773 |
| Biological Process | GO:2000469 negative regulation of peroxidase activity | 0.01504685 |
| Biological Process | GO:2000569 regulation of T-helper 2 cell activation | 0.01504685 |
| Biological Process | GO:2000570 positive regulation of T-helper 2 cell activation | 0.01504685 |
| Biological Process | GO:2000573 positive regulation of DNA biosynthetic process | 0.043331851 |
| Biological Process | GO:2000626 negative regulation of miRNA catabolic process | 0.01504685 |
| Biological Process | GO:2000628 regulation of miRNA metabolic process | 0.013468928 |
| Biological Process | GO:2000629 negative regulation of miRNA metabolic process | 0.01504685 |
| Biological Process | GO:2000793 cell proliferation involved in heart valve development | 0.04446773 |
| Biological Process | GO:2000804 regulation of termination of RNA polymerase II transcription, poly(A)-coupled | 0.04446773 |
| Biological Process | GO:2000805 negative regulation of termination of RNA polymerase II transcription, poly(A)-coupled | 0.029868307 |
| Biological Process | GO:2000811 negative regulation of anoikis | 0.015761503 |
| Biological Process | GO:2001252 positive regulation of chromosome organization | 0.004878194 |
| Cellular Component | GO:0000151 ubiquitin ligase complex | 0.01255109 |
| Cellular Component | GO:0000152 nuclear ubiquitin ligase complex | 0.011499379 |
| Cellular Component | GO:0000421 autophagosome membrane | 0.001553218 |
| Cellular Component | GO:0000781 chromosome, telomeric region | 0.003434807 |
| Cellular Component | GO:0000803 sex chromosome | 0.043339395 |
| Cellular Component | GO:0005622 intracellular | 1.31E-14 |
| Cellular Component | GO:0005623 cell | 9.16E-17 |
| Cellular Component | GO:0005634 nucleus | 7.45E-07 |
| Cellular Component | GO:0005654 nucleoplasm | 5.23E-06 |
| Cellular Component | GO:0005658 alpha DNA polymerase:primase complex | 0.041079502 |
| Cellular Component | GO:0005680 anaphase-promoting complex | 0.028088909 |
| Cellular Component | GO:0005694 chromosome | 0.005552639 |
| Cellular Component | GO:0005730 nucleolus | 0.001923002 |
| Cellular Component | GO:0005737 cytoplasm | 2.18E-08 |
| Cellular Component | GO:0005768 endosome | 0.038366078 |
| Cellular Component | GO:0005776 autophagosome | 0.001338243 |
| Cellular Component | GO:0005777 peroxisome | 0.03056225 |
| Cellular Component | GO:0005783 endoplasmic reticulum | 0.024340403 |
| Cellular Component | GO:0005789 endoplasmic reticulum membrane | 0.022864089 |
| Cellular Component | GO:0005794 Golgi apparatus | 0.031060476 |
| Cellular Component | GO:0005802 trans-Golgi network | 6.12E-05 |
| Cellular Component | GO:0005813 centrosome | 0.015425033 |
| Cellular Component | GO:0005815 microtubule organizing center | 0.01205781 |
| Cellular Component | GO:0005829 cytosol | 7.92E-07 |
| Cellular Component | GO:0005856 cytoskeleton | 0.001732811 |
| Cellular Component | GO:0005901 caveola | 0.037202786 |
| Cellular Component | GO:0005902 microvillus | 0.027341288 |
| Cellular Component | GO:0005912 adherens junction | 0.01327666 |
| Cellular Component | GO:0005943 phosphatidylinositol 3-kinase complex, class IA | 0.013884116 |
| Cellular Component | GO:0008282 inward rectifying potassium channel | 0.041079502 |
| Cellular Component | GO:0010008 endosome membrane | 0.010236174 |
| Cellular Component | GO:0012505 endomembrane system | 0.002870565 |
| Cellular Component | GO:0015630 microtubule cytoskeleton | 0.004250343 |
| Cellular Component | GO:0016035 zeta DNA polymerase complex | 0.027576404 |
| Cellular Component | GO:0016234 inclusion body | 0.008531265 |
| Cellular Component | GO:0016442 RISC complex | 0.000680569 |
| Cellular Component | GO:0016514 SWI/SNF complex | 0.020254156 |
| Cellular Component | GO:0016528 sarcoplasm | 0.000744906 |
| Cellular Component | GO:0016529 sarcoplasmic reticulum | 0.000363004 |
| Cellular Component | GO:0016604 nuclear body | 0.001131399 |
| Cellular Component | GO:0016605 PML body | 0.040842232 |
| Cellular Component | GO:0016607 nuclear speck | 0.002148376 |
| Cellular Component | GO:0019008 molybdopterin synthase complex | 0.027576404 |
| Cellular Component | GO:0019907 cyclin-dependent protein kinase activating kinase holoenzyme complex | 0.041079502 |
| Cellular Component | GO:0030015 CCR4-NOT core complex | 0.041079502 |
| Cellular Component | GO:0030054 cell junction | 0.044785276 |
| Cellular Component | GO:0030496 midbody | 0.008268964 |
| Cellular Component | GO:0030981 cortical microtubule cytoskeleton | 0.041079502 |
| Cellular Component | GO:0031090 organelle membrane | 0.016341689 |
| Cellular Component | GO:0031265 CD95 death-inducing signaling complex | 0.041079502 |
| Cellular Component | GO:0031332 RNAi effector complex | 0.000680569 |
| Cellular Component | GO:0031410 cytoplasmic vesicle | 0.019811997 |
| Cellular Component | GO:0031461 cullin-RING ubiquitin ligase complex | 0.022073044 |
| Cellular Component | GO:0031515 tRNA (m1A) methyltransferase complex | 0.041079502 |
| Cellular Component | GO:0031592 centrosomal corona | 0.013884116 |
| Cellular Component | GO:0031974 membrane-enclosed lumen | 1.93E-08 |
| Cellular Component | GO:0031981 nuclear lumen | 1.16E-07 |
| Cellular Component | GO:0031982 vesicle | 0.004307176 |
| Cellular Component | GO:0031984 organelle subcompartment | 0.00089206 |
| Cellular Component | GO:0032116 SMC loading complex | 0.027576404 |
| Cellular Component | GO:0032302 MutSbeta complex | 0.027576404 |
| Cellular Component | GO:0032473 cytoplasmic side of mitochondrial outer membrane | 0.013884116 |
| Cellular Component | GO:0032839 dendrite cytoplasm | 0.003847512 |
| Cellular Component | GO:0032991 protein-containing complex | 0.000449461 |
| Cellular Component | GO:0033017 sarcoplasmic reticulum membrane | 0.008021519 |
| Cellular Component | GO:0033018 sarcoplasmic reticulum lumen | 0.013884116 |
| Cellular Component | GO:0034274 Atg12-Atg5-Atg16 complex | 0.013884116 |
| Cellular Component | GO:0034451 centriolar satellite | 0.032896705 |
| Cellular Component | GO:0034673 inhibin-betaglycan-ActRII complex | 0.041079502 |
| Cellular Component | GO:0035770 ribonucleoprotein granule | 0.021750426 |
| Cellular Component | GO:0042175 nuclear outer membrane-endoplasmic reticulum membrane network | 0.029473205 |
| Cellular Component | GO:0042579 microbody | 0.03056225 |
| Cellular Component | GO:0042587 glycogen granule | 0.013884116 |
| Cellular Component | GO:0043226 organelle | 4.87E-11 |
| Cellular Component | GO:0043227 membrane-bounded organelle | 3.68E-08 |
| Cellular Component | GO:0043228 non-membrane-bounded organelle | 2.55E-07 |
| Cellular Component | GO:0043229 intracellular organelle | 7.07E-11 |
| Cellular Component | GO:0043231 intracellular membrane-bounded organelle | 1.65E-08 |
| Cellular Component | GO:0043232 intracellular non-membrane-bounded organelle | 2.52E-07 |
| Cellular Component | GO:0043233 organelle lumen | 1.93E-08 |
| Cellular Component | GO:0043540 6-phosphofructo-2-kinase/fructose-2,6-biphosphatase complex | 0.013884116 |
| Cellular Component | GO:0044422 organelle part | 2.34E-13 |
| Cellular Component | GO:0044424 intracellular part | 1.31E-14 |
| Cellular Component | GO:0044427 chromosomal part | 0.007315683 |
| Cellular Component | GO:0044428 nuclear part | 3.14E-09 |
| Cellular Component | GO:0044430 cytoskeletal part | 0.019527149 |
| Cellular Component | GO:0044431 Golgi apparatus part | 0.037890106 |
| Cellular Component | GO:0044432 endoplasmic reticulum part | 0.013495726 |
| Cellular Component | GO:0044433 cytoplasmic vesicle part | 0.038516716 |
| Cellular Component | GO:0044440 endosomal part | 0.004926627 |
| Cellular Component | GO:0044444 cytoplasmic part | 9.21E-08 |
| Cellular Component | GO:0044446 intracellular organelle part | 1.42E-11 |
| Cellular Component | GO:0044449 contractile fiber part | 0.043253511 |
| Cellular Component | GO:0044451 nucleoplasm part | 0.001381248 |
| Cellular Component | GO:0044464 cell part | 9.16E-17 |
| Cellular Component | GO:0044753 amphisome | 0.027576404 |
| Cellular Component | GO:0061695 transferase complex, transferring phosphorus-containing groups | 0.009062524 |
| Cellular Component | GO:0070013 intracellular organelle lumen | 1.93E-08 |
| Cellular Component | GO:0070161 anchoring junction | 0.017362039 |
| Cellular Component | GO:0070381 endosome to plasma membrane transport vesicle | 0.027576404 |
| Cellular Component | GO:0070418 DNA-dependent protein kinase complex | 0.027576404 |
| Cellular Component | GO:0070578 RISC-loading complex | 0.005083256 |
| Cellular Component | GO:0070985 transcription factor TFIIK complex | 0.041079502 |
| Cellular Component | GO:0090694 Scc2-Scc4 cohesin loading complex | 0.027576404 |
| Cellular Component | GO:0097487 multivesicular body, internal vesicle | 0.041079502 |
| Cellular Component | GO:0097543 ciliary inversin compartment | 0.041079502 |
| Cellular Component | GO:0097635 extrinsic component of autophagosome membrane | 0.027576404 |
| Cellular Component | GO:0097708 intracellular vesicle | 0.021099351 |
| Cellular Component | GO:0098588 bounding membrane of organelle | 0.005526112 |
| Cellular Component | GO:0098687 chromosomal region | 0.006871528 |
| Cellular Component | GO:0098791 Golgi subcompartment | 0.017127055 |
| Cellular Component | GO:0098805 whole membrane | 0.007611624 |
| Cellular Component | GO:0098827 endoplasmic reticulum subcompartment | 0.026684452 |
| Cellular Component | GO:0099023 tethering complex | 0.033738386 |
| Cellular Component | GO:0099400 caveola neck | 0.013884116 |
| Cellular Component | GO:0120111 neuron projection cytoplasm | 0.009715018 |
| Cellular Component | GO:0150005 enzyme activator complex | 0.041079502 |
| Cellular Component | GO:1902494 catalytic complex | 6.34E-06 |
| Cellular Component | GO:1902554 serine/threonine protein kinase complex | 0.042726966 |
| Cellular Component | GO:1902555 endoribonuclease complex | 0.043339395 |
| Cellular Component | GO:1904115 axon cytoplasm | 0.041079502 |
| Cellular Component | GO:1905348 endonuclease complex | 0.043339395 |
| Cellular Component | GO:1990234 transferase complex | 4.96E-05 |
| Cellular Component | GO:1990391 DNA repair complex | 0.043339395 |
| Cellular Component | GO:1990752 microtubule end | 0.033878036 |
| Molecular Function | GO:0000166 nucleotide binding | 1.48E-07 |
| Molecular Function | GO:0000287 magnesium ion binding | 0.018666744 |
| Molecular Function | GO:0001098 basal transcription machinery binding | 0.020591105 |
| Molecular Function | GO:0001099 basal RNA polymerase II transcription machinery binding | 0.020591105 |
| Molecular Function | GO:0002039 p53 binding | 0.008743255 |
| Molecular Function | GO:0003678 DNA helicase activity | 0.028261003 |
| Molecular Function | GO:0003682 chromatin binding | 0.023619562 |
| Molecular Function | GO:0003723 RNA binding | 0.009509306 |
| Molecular Function | GO:0003725 double-stranded RNA binding | 0.001900709 |
| Molecular Function | GO:0003779 actin binding | 0.009265282 |
| Molecular Function | GO:0003824 catalytic activity | 1.36E-05 |
| Molecular Function | GO:0003887 DNA-directed DNA polymerase activity | 0.020773257 |
| Molecular Function | GO:0003938 IMP dehydrogenase activity | 0.029845028 |
| Molecular Function | GO:0004077 biotin-[acetyl-CoA-carboxylase] ligase activity | 0.015035036 |
| Molecular Function | GO:0004105 choline-phosphate cytidylyltransferase activity | 0.029845028 |
| Molecular Function | GO:0004133 glycogen debranching enzyme activity | 0.015035036 |
| Molecular Function | GO:0004134 4-alpha-glucanotransferase activity | 0.015035036 |
| Molecular Function | GO:0004135 amylo-alpha-1,6-glucosidase activity | 0.015035036 |
| Molecular Function | GO:0004331 fructose-2,6-bisphosphate 2-phosphatase activity | 0.029845028 |
| Molecular Function | GO:0004386 helicase activity | 0.021885373 |
| Molecular Function | GO:0004450 isocitrate dehydrogenase (NADP+) activity | 0.029845028 |
| Molecular Function | GO:0004525 ribonuclease III activity | 0.044433328 |
| Molecular Function | GO:0004587 ornithine-oxo-acid transaminase activity | 0.029845028 |
| Molecular Function | GO:0004672 protein kinase activity | 0.014713021 |
| Molecular Function | GO:0004674 protein serine/threonine kinase activity | 0.00198415 |
| Molecular Function | GO:0004675 transmembrane receptor protein serine/threonine kinase activity | 0.00162471 |
| Molecular Function | GO:0004676 3-phosphoinositide-dependent protein kinase activity | 0.015035036 |
| Molecular Function | GO:0004719 protein-L-isoaspartate (D-aspartate) O-methyltransferase activity | 0.000668428 |
| Molecular Function | GO:0004823 leucine-tRNA ligase activity | 0.029845028 |
| Molecular Function | GO:0004843 thiol-dependent ubiquitin-specific protease activity | 0.049936951 |
| Molecular Function | GO:0005096 GTPase activator activity | 0.018582115 |
| Molecular Function | GO:0005388 calcium-transporting ATPase activity | 0.000263428 |
| Molecular Function | GO:0005488 binding | 2.69E-13 |
| Molecular Function | GO:0005515 protein binding | 9.03E-12 |
| Molecular Function | GO:0005524 ATP binding | 3.98E-06 |
| Molecular Function | GO:0005543 phospholipid binding | 0.035883928 |
| Molecular Function | GO:0008017 microtubule binding | 0.001229522 |
| Molecular Function | GO:0008047 enzyme activator activity | 0.029780016 |
| Molecular Function | GO:0008092 cytoskeletal protein binding | 6.04E-06 |
| Molecular Function | GO:0008144 drug binding | 1.26E-05 |
| Molecular Function | GO:0008171 O-methyltransferase activity | 0.023508763 |
| Molecular Function | GO:0008234 cysteine-type peptidase activity | 0.033105645 |
| Molecular Function | GO:0008276 protein methyltransferase activity | 0.028698586 |
| Molecular Function | GO:0008281 sulfonylurea receptor activity | 0.029845028 |
| Molecular Function | GO:0008432 JUN kinase binding | 0.002184123 |
| Molecular Function | GO:0008510 sodium:bicarbonate symporter activity | 0.044433328 |
| Molecular Function | GO:0008609 alkylglycerone-phosphate synthase activity | 0.015035036 |
| Molecular Function | GO:0008940 nitrate reductase activity | 0.029845028 |
| Molecular Function | GO:0009378 four-way junction helicase activity | 0.029845028 |
| Molecular Function | GO:0010340 carboxyl-O-methyltransferase activity | 0.003243757 |
| Molecular Function | GO:0015085 calcium ion transmembrane transporter activity | 0.048311432 |
| Molecular Function | GO:0015399 primary active transmembrane transporter activity | 0.004386149 |
| Molecular Function | GO:0015405 P-P-bond-hydrolysis-driven transmembrane transporter activity | 0.004386149 |
| Molecular Function | GO:0015631 tubulin binding | 0.004244812 |
| Molecular Function | GO:0015662 ATPase activity, coupled to transmembrane movement of ions, phosphorylative mechanism | 0.001334801 |
| Molecular Function | GO:0016301 kinase activity | 0.005841922 |
| Molecular Function | GO:0016462 pyrophosphatase activity | 0.000125353 |
| Molecular Function | GO:0016740 transferase activity | 0.00040579 |
| Molecular Function | GO:0016772 transferase activity, transferring phosphorus-containing groups | 0.000810603 |
| Molecular Function | GO:0016773 phosphotransferase activity, alcohol group as acceptor | 0.005987232 |
| Molecular Function | GO:0016783 sulfurtransferase activity | 0.00935281 |
| Molecular Function | GO:0016787 hydrolase activity | 0.002559727 |
| Molecular Function | GO:0016817 hydrolase activity, acting on acid anhydrides | 0.000133835 |
| Molecular Function | GO:0016818 hydrolase activity, acting on acid anhydrides, in phosphorus-containing anhydrides | 0.000133835 |
| Molecular Function | GO:0016887 ATPase activity | 0.005895533 |
| Molecular Function | GO:0017022 myosin binding | 0.004216016 |
| Molecular Function | GO:0017076 purine nucleotide binding | 1.45E-05 |
| Molecular Function | GO:0017111 nucleoside-triphosphatase activity | 5.00E-05 |
| Molecular Function | GO:0018271 biotin-protein ligase activity | 0.015035036 |
| Molecular Function | GO:0019150 D-ribulokinase activity | 0.015035036 |
| Molecular Function | GO:0019200 carbohydrate kinase activity | 0.032529458 |
| Molecular Function | GO:0019829 cation-transporting ATPase activity | 0.002225954 |
| Molecular Function | GO:0019870 potassium channel inhibitor activity | 0.044433328 |
| Molecular Function | GO:0019899 enzyme binding | 8.16E-05 |
| Molecular Function | GO:0019902 phosphatase binding | 0.011946061 |
| Molecular Function | GO:0019903 protein phosphatase binding | 0.048311432 |
| Molecular Function | GO:0019904 protein domain specific binding | 0.000596133 |
| Molecular Function | GO:0022853 active ion transmembrane transporter activity | 0.002225954 |
| Molecular Function | GO:0030331 estrogen receptor binding | 0.006754341 |
| Molecular Function | GO:0030366 molybdopterin synthase activity | 0.015035036 |
| Molecular Function | GO:0030374 nuclear receptor transcription coactivator activity | 0.014238983 |
| Molecular Function | GO:0030554 adenyl nucleotide binding | 3.46E-06 |
| Molecular Function | GO:0030695 GTPase regulator activity | 0.033105645 |
| Molecular Function | GO:0031071 cysteine desulfurase activity | 0.015035036 |
| Molecular Function | GO:0031267 small GTPase binding | 0.007071313 |
| Molecular Function | GO:0031624 ubiquitin conjugating enzyme binding | 0.011318757 |
| Molecular Function | GO:0032139 dinucleotide insertion or deletion binding | 0.044433328 |
| Molecular Function | GO:0032142 single guanine insertion binding | 0.044433328 |
| Molecular Function | GO:0032181 dinucleotide repeat insertion binding | 0.029845028 |
| Molecular Function | GO:0032296 double-stranded RNA-specific ribonuclease activity | 0.044433328 |
| Molecular Function | GO:0032451 demethylase activity | 0.008325322 |
| Molecular Function | GO:0032452 histone demethylase activity | 0.003650949 |
| Molecular Function | GO:0032453 histone demethylase activity (H3-K4 specific) | 0.004496364 |
| Molecular Function | GO:0032454 histone demethylase activity (H3-K9 specific) | 0.007556679 |
| Molecular Function | GO:0032553 ribonucleotide binding | 3.06E-05 |
| Molecular Function | GO:0032555 purine ribonucleotide binding | 2.52E-05 |
| Molecular Function | GO:0032559 adenyl ribonucleotide binding | 2.93E-06 |
| Molecular Function | GO:0033149 FFAT motif binding | 0.029845028 |
| Molecular Function | GO:0033743 peptide-methionine (R)-S-oxide reductase activity | 0.044433328 |
| Molecular Function | GO:0033745 L-methionine-(R)-S-oxide reductase activity | 0.029845028 |
| Molecular Function | GO:0034061 DNA polymerase activity | 0.046286419 |
| Molecular Function | GO:0034211 GTP-dependent protein kinase activity | 0.015035036 |
| Molecular Function | GO:0034647 histone demethylase activity (H3-trimethyl-K4 specific) | 0.044433328 |
| Molecular Function | GO:0034648 histone demethylase activity (H3-dimethyl-K4 specific) | 0.001323591 |
| Molecular Function | GO:0035257 nuclear hormone receptor binding | 0.015492864 |
| Molecular Function | GO:0035258 steroid hormone receptor binding | 0.00150391 |
| Molecular Function | GO:0035400 histone tyrosine kinase activity | 0.015035036 |
| Molecular Function | GO:0035401 histone kinase activity (H3-Y41 specific) | 0.015035036 |
| Molecular Function | GO:0035639 purine ribonucleoside triphosphate binding | 3.19E-05 |
| Molecular Function | GO:0036094 small molecule binding | 2.73E-06 |
| Molecular Function | GO:0036122 BMP binding | 0.011318757 |
| Molecular Function | GO:0036459 thiol-dependent ubiquitinyl hydrolase activity | 0.049936951 |
| Molecular Function | GO:0036479 peroxidase inhibitor activity | 0.015035036 |
| Molecular Function | GO:0042169 SH2 domain binding | 0.011053134 |
| Molecular Function | GO:0042623 ATPase activity, coupled | 0.009953753 |
| Molecular Function | GO:0042625 ATPase coupled ion transmembrane transporter activity | 0.002225954 |
| Molecular Function | GO:0042626 ATPase activity, coupled to transmembrane movement of substances | 0.003979872 |
| Molecular Function | GO:0042805 actinin binding | 0.029391562 |
| Molecular Function | GO:0042974 retinoic acid receptor binding | 0.00085786 |
| Molecular Function | GO:0043167 ion binding | 2.13E-06 |
| Molecular Function | GO:0043168 anion binding | 1.33E-06 |
| Molecular Function | GO:0043169 cation binding | 0.008935353 |
| Molecular Function | GO:0043425 bHLH transcription factor binding | 0.042675024 |
| Molecular Function | GO:0043426 MRF binding | 0.002184123 |
| Molecular Function | GO:0043492 ATPase activity, coupled to movement of substances | 0.004386149 |
| Molecular Function | GO:0044325 ion channel binding | 0.005712575 |
| Molecular Function | GO:0044390 ubiquitin-like protein conjugating enzyme binding | 0.018181463 |
| Molecular Function | GO:0044877 protein-containing complex binding | 0.004086373 |
| Molecular Function | GO:0046872 metal ion binding | 0.00775561 |
| Molecular Function | GO:0047369 succinate-hydroxymethylglutarate CoA-transferase activity | 0.015035036 |
| Molecular Function | GO:0047485 protein N-terminus binding | 0.005102361 |
| Molecular Function | GO:0048037 cofactor binding | 0.01902722 |
| Molecular Function | GO:0050115 myosin-light-chain-phosphatase activity | 0.015035036 |
| Molecular Function | GO:0050660 flavin adenine dinucleotide binding | 0.003328469 |
| Molecular Function | GO:0050662 coenzyme binding | 0.002805455 |
| Molecular Function | GO:0050839 cell adhesion molecule binding | 0.044038083 |
| Molecular Function | GO:0051020 GTPase binding | 0.003535407 |
| Molecular Function | GO:0051427 hormone receptor binding | 0.026978987 |
| Molecular Function | GO:0051880 G-quadruplex DNA binding | 0.003243757 |
| Molecular Function | GO:0051998 protein carboxyl O-methyltransferase activity | 0.003243757 |
| Molecular Function | GO:0061749 forked DNA-dependent helicase activity | 0.029845028 |
| Molecular Function | GO:0061752 telomeric repeat-containing RNA binding | 0.015035036 |
| Molecular Function | GO:0061821 telomeric D-loop binding | 0.044433328 |
| Molecular Function | GO:0070026 nitric oxide binding | 0.015035036 |
| Molecular Function | GO:0070123 transforming growth factor beta receptor activity, type III | 0.015035036 |
| Molecular Function | GO:0070336 flap-structured DNA binding | 0.029845028 |
| Molecular Function | GO:0070337 3'-flap-structured DNA binding | 0.015035036 |
| Molecular Function | GO:0071949 FAD binding | 0.009184487 |
| Molecular Function | GO:0071987 WD40-repeat domain binding | 0.044433328 |
| Molecular Function | GO:0086039 calcium-transporting ATPase activity involved in regulation of cardiac muscle cell membrane potential | 0.015035036 |
| Molecular Function | GO:0090599 alpha-glucosidase activity | 0.044433328 |
| Molecular Function | GO:0097159 organic cyclic compound binding | 4.52E-05 |
| Molecular Function | GO:0097367 carbohydrate derivative binding | 0.000397309 |
| Molecular Function | GO:0098821 BMP receptor activity | 0.004496364 |
| Molecular Function | GO:0102500 beta-maltose 4-alpha-glucanotransferase activity | 0.015035036 |
| Molecular Function | GO:0140096 catalytic activity, acting on a protein | 0.000107928 |
| Molecular Function | GO:1901265 nucleoside phosphate binding | 1.48E-07 |
| Molecular Function | GO:1901363 heterocyclic compound binding | 2.78E-05 |
| Molecular Function | GO:1904713 beta-catenin destruction complex binding | 0.029845028 |
| Molecular Function | GO:1905773 8-hydroxy-2'-deoxyguanosine DNA binding | 0.044433328 |
| Molecular Function | GO:1990174 phosphodiesterase decapping endonuclease activity | 0.029845028 |
| Molecular Function | GO:1990269 RNA polymerase II C-terminal domain phosphoserine binding | 0.044433328 |
| Molecular Function | GO:1990460 leptin receptor binding | 0.044433328 |
| Molecular Function | GO:1990841 promoter-specific chromatin binding | 0.004216016 |

Supplementary Table 6 KEGG analysis of source genes of DE circRNAs

| B_ Pathway | C_ Pathway | L-vs-H |
| --- | --- | --- |
| Cancer: overview | ko05235 PD-L1 expression and PD-1 checkpoint pathway in cancer | 0.006246017 |
| Cancers | ko05206 MicroRNAs in cancer | 0.000690696 |
| Cancers | ko05210 Colorectal cancer | 0.004603762 |
| Cancers | ko05212 Pancreatic cancer | 0.01459443 |
| Cancers | ko05213 Endometrial cancer | 0.000651114 |
| Cancers | ko05215 Prostate cancer | 0.000175027 |
| Cancers | ko05221 Acute myeloid leukemia | 0.04906559 |
| Cancers | ko05223 Non-small cell lung cancer | 0.008673175 |
| Cancers | ko05231 Choline metabolism in cancer | 0.001106889 |
| Carbohydrate metabolism | ko00020 Citrate cycle (TCA cycle) | 0.0476813 |
| Cardiovascular diseases | ko05418 Fluid shear stress and atherosclerosis | 0.03352238 |
| Cell growth and death | ko04110 Cell cycle | 0.00432771 |
| Cell growth and death | ko04210 Apoptosis | 0.006926393 |
| Cell growth and death | ko04218 Cellular senescence | 0.000131104 |
| Cellular community - eukaryotes | ko04510 Focal adhesion | 0.002227384 |
| Cellular community - eukaryotes | ko04520 Adherens junction | 0.01059874 |
| Cellular community - eukaryotes | ko04550 Signaling pathways regulating pluripotency of stem cells | 0.001986714 |
| Development | ko04360 Axon guidance | 0.005913929 |
| Development and regeneration | ko04361 Axon regeneration | 0.02696969 |
| Drug resistance | ko01521 EGFR tyrosine kinase inhibitor resistance | 0.000453933 |
| Drug resistance | ko01522 Endocrine resistance | 0.005291282 |
| Drug resistance | ko01524 Platinum drug resistance | 0.001849574 |
| Endocrine and metabolic diseases | ko04931 Insulin resistance | 0.000468642 |
| Endocrine system | ko04910 Insulin signaling pathway | 0.008721131 |
| Endocrine system | ko04914 Progesterone-mediated oocyte maturation | 0.02429009 |
| Endocrine system | ko04917 Prolactin signaling pathway | 0.001634109 |
| Endocrine system | ko04919 Thyroid hormone signaling pathway | 1.21E-07 |
| Endocrine system | ko04935 Growth hormone synthesis, secretion and action | 0.003009981 |
| Excretory system | ko04960 Aldosterone-regulated sodium reabsorption | 0.009214413 |
| Folding, sorting and degradation | ko03018 RNA degradation | 0.01459443 |
| Folding, sorting and degradation | ko04120 Ubiquitin mediated proteolysis | 0.002145532 |
| Folding, sorting and degradation | ko04122 Sulfur relay system | 0.004944572 |
| Immune system | ko04062 Chemokine signaling pathway | 0.004919839 |
| Immune system | ko04660 T cell receptor signaling pathway | 0.002401998 |
| Immune system | ko04662 B cell receptor signaling pathway | 0.003982418 |
| Immune system | ko04664 Fc epsilon RI signaling pathway | 0.01059874 |
| Immune system | ko04666 Fc gamma R-mediated phagocytosis | 0.02790075 |
| Infectious diseases | ko05130 Pathogenic Escherichia coli infection | 0.04262946 |
| Infectious diseases | ko05131 Shigellosis | 0.02778578 |
| Infectious diseases | ko05160 Hepatitis C | 0.03968795 |
| Infectious diseases | ko05161 Hepatitis B | 0.003431018 |
| Infectious diseases | ko05165 Human papillomavirus infection | 0.006113367 |
| Infectious diseases | ko05167 Kaposi sarcoma-associated herpesvirus infection | 0.02344279 |
| Metabolism of cofactors and vitamins | ko00780 Biotin metabolism | 0.03588411 |
| Metabolism of cofactors and vitamins | ko00790 Folate biosynthesis | 0.03113193 |
| Nervous system | ko04720 Long-term potentiation | 0.04730378 |
| Nervous system | ko04722 Neurotrophin signaling pathway | 0.01553312 |
| Signal transduction | ko04012 ErbB signaling pathway | 0.000484557 |
| Signal transduction | ko04022 cGMP - PKG signaling pathway | 0.01608418 |
| Signal transduction | ko04024 cAMP signaling pathway | 0.006990475 |
| Signal transduction | ko04064 NF-kappa B signaling pathway | 0.03968795 |
| Signal transduction | ko04068 FoxO signaling pathway | 0.001164121 |
| Signal transduction | ko04071 Sphingolipid signaling pathway | 0.002884769 |
| Signal transduction | ko04072 Phospholipase D signaling pathway | 0.03968795 |
| Signal transduction | ko04151 PI3K-Akt signaling pathway | 0.02568459 |
| Signal transduction | ko04350 TGF-beta signaling pathway | 0.03080837 |
| Signal transduction | ko04370 VEGF signaling pathway | 0.03148168 |
| Signal transduction | ko04630 Jak-STAT signaling pathway | 0.04753155 |
| Transport and catabolism | ko04140 Autophagy - animal | 0.008177677 |
| Transport and catabolism | ko04144 Endocytosis | 0.0300072 |

Supplementary Table 7 GO analysis of source genes of DE circRNAs related to skeletal muscle development

| Type | Term | L-vs-H |
| --- | --- | --- |
| Biological Process | GO:0000038 very long-chain fatty acid metabolic process | 0.037107 |
| Biological Process | GO:0000186 activation of MAPKK activity | 0.035522 |
| Biological Process | GO:0000187 activation of MAPK activity | 0.005989 |
| Biological Process | GO:0001101 response to acid chemical | 0.022185 |
| Biological Process | GO:0001544 initiation of primordial ovarian follicle growth | 0.00328 |
| Biological Process | GO:0001547 antral ovarian follicle growth | 0.011436 |
| Biological Process | GO:0001568 blood vessel development | 0.041579 |
| Biological Process | GO:0001934 positive regulation of protein phosphorylation | 0.017498 |
| Biological Process | GO:0001935 endothelial cell proliferation | 0.012952 |
| Biological Process | GO:0001936 regulation of endothelial cell proliferation | 0.010086 |
| Biological Process | GO:0001938 positive regulation of endothelial cell proliferation | 0.003767 |
| Biological Process | GO:0001944 vasculature development | 0.047471 |
| Biological Process | GO:0002026 regulation of the force of heart contraction | 0.025956 |
| Biological Process | GO:0002068 glandular epithelial cell development | 0.027556 |
| Biological Process | GO:0002092 positive regulation of receptor internalization | 0.029155 |
| Biological Process | GO:0002115 store-operated calcium entry | 0.021139 |
| Biological Process | GO:0002279 mast cell activation involved in immune response | 0.040271 |
| Biological Process | GO:0002448 mast cell mediated immunity | 0.03869 |
| Biological Process | GO:0003009 skeletal muscle contraction | 0.037107 |
| Biological Process | GO:0003012 muscle system process | 2.48E-05 |
| Biological Process | GO:0003015 heart process | 0.021023 |
| Biological Process | GO:0003299 muscle hypertrophy in response to stress | 0.022747 |
| Biological Process | GO:0003309 type B pancreatic cell differentiation | 0.029155 |
| Biological Process | GO:0003323 type B pancreatic cell development | 0.021139 |
| Biological Process | GO:0006024 glycosaminoglycan biosynthetic process | 0.043425 |
| Biological Process | GO:0006082 organic acid metabolic process | 0.002931 |
| Biological Process | GO:0006091 generation of precursor metabolites and energy | 0.008899 |
| Biological Process | GO:0006099 tricarboxylic acid cycle | 0.025956 |
| Biological Process | GO:0006101 citrate metabolic process | 0.03075 |
| Biological Process | GO:0006468 protein phosphorylation | 0.018983 |
| Biological Process | GO:0006629 lipid metabolic process | 0.048319 |
| Biological Process | GO:0006631 fatty acid metabolic process | 0.004187 |
| Biological Process | GO:0006635 fatty acid beta-oxidation | 0.002238 |
| Biological Process | GO:0006692 prostanoid metabolic process | 0.046568 |
| Biological Process | GO:0006693 prostaglandin metabolic process | 0.046568 |
| Biological Process | GO:0006811 ion transport | 0.030743 |
| Biological Process | GO:0006812 cation transport | 0.006062 |
| Biological Process | GO:0006816 calcium ion transport | 4.32E-05 |
| Biological Process | GO:0006851 mitochondrial calcium ion transmembrane transport | 0.022747 |
| Biological Process | GO:0006873 cellular ion homeostasis | 0.018966 |
| Biological Process | GO:0006874 cellular calcium ion homeostasis | 0.005593 |
| Biological Process | GO:0006875 cellular metal ion homeostasis | 0.013978 |
| Biological Process | GO:0006887 exocytosis | 0.004988 |
| Biological Process | GO:0006936 muscle contraction | 0.000164 |
| Biological Process | GO:0006941 striated muscle contraction | 0.010934 |
| Biological Process | GO:0006972 hyperosmotic response | 0.017915 |
| Biological Process | GO:0007166 cell surface receptor signaling pathway | 0.014504 |
| Biological Process | GO:0007167 enzyme linked receptor protein signaling pathway | 0.003618 |
| Biological Process | GO:0007169 transmembrane receptor protein tyrosine kinase signaling pathway | 0.003833 |
| Biological Process | GO:0007173 epidermal growth factor receptor signaling pathway | 0.005506 |
| Biological Process | GO:0007176 regulation of epidermal growth factor-activated receptor activity | 0.025956 |
| Biological Process | GO:0007178 transmembrane receptor protein serine/threonine kinase signaling pathway | 0.009461 |
| Biological Process | GO:0007179 transforming growth factor beta receptor signaling pathway | 0.022776 |
| Biological Process | GO:0007204 positive regulation of cytosolic calcium ion concentration | 0.01878 |
| Biological Process | GO:0007257 activation of JUN kinase activity | 0.029155 |
| Biological Process | GO:0007276 gamete generation | 0.022883 |
| Biological Process | GO:0009062 fatty acid catabolic process | 0.004173 |
| Biological Process | GO:0009415 response to water | 0.00981 |
| Biological Process | GO:0009653 anatomical structure morphogenesis | 0.019945 |
| Biological Process | GO:0009725 response to hormone | 0.022254 |
| Biological Process | GO:0010243 response to organonitrogen compound | 0.040062 |
| Biological Process | GO:0010256 endomembrane system organization | 0.009847 |
| Biological Process | GO:0010517 regulation of phospholipase activity | 0.049702 |
| Biological Process | GO:0010518 positive regulation of phospholipase activity | 0.041849 |
| Biological Process | GO:0010522 regulation of calcium ion transport into cytosol | 0.004036 |
| Biological Process | GO:0010524 positive regulation of calcium ion transport into cytosol | 0.001322 |
| Biological Process | GO:0010562 positive regulation of phosphorus metabolic process | 0.025434 |
| Biological Process | GO:0010594 regulation of endothelial cell migration | 0.017169 |
| Biological Process | GO:0010595 positive regulation of endothelial cell migration | 0.007009 |
| Biological Process | GO:0010631 epithelial cell migration | 0.047257 |
| Biological Process | GO:0010632 regulation of epithelial cell migration | 0.029365 |
| Biological Process | GO:0010634 positive regulation of epithelial cell migration | 0.01272 |
| Biological Process | GO:0010656 negative regulation of muscle cell apoptotic process | 0.025956 |
| Biological Process | GO:0010657 muscle cell apoptotic process | 0.044998 |
| Biological Process | GO:0010658 striated muscle cell apoptotic process | 0.021139 |
| Biological Process | GO:0010659 cardiac muscle cell apoptotic process | 0.019528 |
| Biological Process | GO:0010660 regulation of muscle cell apoptotic process | 0.044998 |
| Biological Process | GO:0010662 regulation of striated muscle cell apoptotic process | 0.021139 |
| Biological Process | GO:0010664 negative regulation of striated muscle cell apoptotic process | 0.017915 |
| Biological Process | GO:0010665 regulation of cardiac muscle cell apoptotic process | 0.019528 |
| Biological Process | GO:0010667 negative regulation of cardiac muscle cell apoptotic process | 0.016299 |
| Biological Process | GO:0010737 protein kinase A signaling | 0.029155 |
| Biological Process | GO:0010800 positive regulation of peptidyl-threonine phosphorylation | 0.032343 |
| Biological Process | GO:0010874 regulation of cholesterol efflux | 0.041849 |
| Biological Process | GO:0010880 regulation of release of sequestered calcium ion into cytosol by sarcoplasmic reticulum | 0.022747 |
| Biological Process | GO:0010927 cellular component assembly involved in morphogenesis | 0.003636 |
| Biological Process | GO:0010959 regulation of metal ion transport | 0.004246 |
| Biological Process | GO:0014065 phosphatidylinositol 3-kinase signaling | 0.008293 |
| Biological Process | GO:0014722 regulation of skeletal muscle contraction by calcium ion signaling | 0.004917 |
| Biological Process | GO:0014733 regulation of skeletal muscle adaptation | 0.016299 |
| Biological Process | GO:0014808 release of sequestered calcium ion into cytosol by sarcoplasmic reticulum | 0.027556 |
| Biological Process | GO:0014809 regulation of skeletal muscle contraction by regulation of release of sequestered calcium ion | 0.00328 |
| Biological Process | GO:0014819 regulation of skeletal muscle contraction | 0.017915 |
| Biological Process | GO:0014883 transition between fast and slow fiber | 0.011436 |
| Biological Process | GO:0014887 cardiac muscle adaptation | 0.022747 |
| Biological Process | GO:0014888 striated muscle adaptation | 0.043425 |
| Biological Process | GO:0014898 cardiac muscle hypertrophy in response to stress | 0.022747 |
| Biological Process | GO:0015980 energy derivation by oxidation of organic compounds | 0.027092 |
| Biological Process | GO:0016042 lipid catabolic process | 0.037712 |
| Biological Process | GO:0016054 organic acid catabolic process | 0.022776 |
| Biological Process | GO:0016240 autophagosome membrane docking | 0.00981 |
| Biological Process | GO:0016310 phosphorylation | 0.037596 |
| Biological Process | GO:0016572 histone phosphorylation | 0.03869 |
| Biological Process | GO:0016999 antibiotic metabolic process | 0.004036 |
| Biological Process | GO:0017000 antibiotic biosynthetic process | 0.016299 |
| Biological Process | GO:0017157 regulation of exocytosis | 0.016907 |
| Biological Process | GO:0018107 peptidyl-threonine phosphorylation | 0.007009 |
| Biological Process | GO:0018108 peptidyl-tyrosine phosphorylation | 0.046069 |
| Biological Process | GO:0018193 peptidyl-amino acid modification | 0.039463 |
| Biological Process | GO:0018210 peptidyl-threonine modification | 0.008293 |
| Biological Process | GO:0018212 peptidyl-tyrosine modification | 0.047257 |
| Biological Process | GO:0019395 fatty acid oxidation | 0.004313 |
| Biological Process | GO:0019722 calcium-mediated signaling | 0.015369 |
| Biological Process | GO:0019725 cellular homeostasis | 0.007661 |
| Biological Process | GO:0019752 carboxylic acid metabolic process | 0.00211 |
| Biological Process | GO:0019932 second-messenger-mediated signaling | 0.047257 |
| Biological Process | GO:0019953 sexual reproduction | 0.042018 |
| Biological Process | GO:0021534 cell proliferation in hindbrain | 0.022747 |
| Biological Process | GO:0021924 cell proliferation in external granule layer | 0.022747 |
| Biological Process | GO:0021930 cerebellar granule cell precursor proliferation | 0.022747 |
| Biological Process | GO:0021936 regulation of cerebellar granule cell precursor proliferation | 0.017915 |
| Biological Process | GO:0021940 positive regulation of cerebellar granule cell precursor proliferation | 0.014681 |
| Biological Process | GO:0022602 ovulation cycle process | 0.044998 |
| Biological Process | GO:0023051 regulation of signaling | 0.020389 |
| Biological Process | GO:0030001 metal ion transport | 0.00164 |
| Biological Process | GO:0030003 cellular cation homeostasis | 0.017706 |
| Biological Process | GO:0030029 actin filament-based process | 0.04493 |
| Biological Process | GO:0030036 actin cytoskeleton organization | 0.035687 |
| Biological Process | GO:0030212 hyaluronan metabolic process | 0.029155 |
| Biological Process | GO:0030213 hyaluronan biosynthetic process | 0.01306 |
| Biological Process | GO:0030239 myofibril assembly | 0.001166 |
| Biological Process | GO:0030242 autophagy of peroxisome | 0.00328 |
| Biological Process | GO:0030258 lipid modification | 0.001726 |
| Biological Process | GO:0030728 ovulation | 0.019528 |
| Biological Process | GO:0030834 regulation of actin filament depolymerization | 0.048137 |
| Biological Process | GO:0030835 negative regulation of actin filament depolymerization | 0.037107 |
| Biological Process | GO:0030859 polarized epithelial cell differentiation | 0.03075 |
| Biological Process | GO:0031018 endocrine pancreas development | 0.049702 |
| Biological Process | GO:0031032 actomyosin structure organization | 0.016387 |
| Biological Process | GO:0031401 positive regulation of protein modification process | 0.038543 |
| Biological Process | GO:0032147 activation of protein kinase activity | 0.00176 |
| Biological Process | GO:0032148 activation of protein kinase B activity | 0.025956 |
| Biological Process | GO:0032354 response to follicle-stimulating hormone | 0.00655 |
| Biological Process | GO:0032369 negative regulation of lipid transport | 0.027556 |
| Biological Process | GO:0032372 negative regulation of sterol transport | 0.011436 |
| Biological Process | GO:0032375 negative regulation of cholesterol transport | 0.011436 |
| Biological Process | GO:0032469 endoplasmic reticulum calcium ion homeostasis | 0.03075 |
| Biological Process | GO:0032470 positive regulation of endoplasmic reticulum calcium ion concentration | 0.004917 |
| Biological Process | GO:0032504 multicellular organism reproduction | 0.0365 |
| Biological Process | GO:0032787 monocarboxylic acid metabolic process | 0.015332 |
| Biological Process | GO:0032881 regulation of polysaccharide metabolic process | 0.033934 |
| Biological Process | GO:0032885 regulation of polysaccharide biosynthetic process | 0.03075 |
| Biological Process | GO:0032940 secretion by cell | 0.006091 |
| Biological Process | GO:0032984 protein-containing complex disassembly | 0.022776 |
| Biological Process | GO:0033003 regulation of mast cell activation | 0.032343 |
| Biological Process | GO:0033006 regulation of mast cell activation involved in immune response | 0.029155 |
| Biological Process | GO:0033292 T-tubule organization | 0.00655 |
| Biological Process | GO:0033539 fatty acid beta-oxidation using acyl-CoA dehydrogenase | 0.01306 |
| Biological Process | GO:0033540 fatty acid beta-oxidation using acyl-CoA oxidase | 0.004917 |
| Biological Process | GO:0033674 positive regulation of kinase activity | 0.015586 |
| Biological Process | GO:0034122 negative regulation of toll-like receptor signaling pathway | 0.044998 |
| Biological Process | GO:0034220 ion transmembrane transport | 0.016578 |
| Biological Process | GO:0034440 lipid oxidation | 0.004598 |
| Biological Process | GO:0034694 response to prostaglandin | 0.021139 |
| Biological Process | GO:0034695 response to prostaglandin E | 0.019528 |
| Biological Process | GO:0034698 response to gonadotropin | 0.011436 |
| Biological Process | GO:0034699 response to luteinizing hormone | 0.001641 |
| Biological Process | GO:0034764 positive regulation of transmembrane transport | 0.014625 |
| Biological Process | GO:0034765 regulation of ion transmembrane transport | 0.043726 |
| Biological Process | GO:0034767 positive regulation of ion transmembrane transport | 0.007916 |
| Biological Process | GO:0035088 establishment or maintenance of apical/basal cell polarity | 0.046568 |
| Biological Process | GO:0035089 establishment of apical/basal cell polarity | 0.029155 |
| Biological Process | GO:0035406 histone-tyrosine phosphorylation | 0.001641 |
| Biological Process | GO:0035409 histone H3-Y41 phosphorylation | 0.001641 |
| Biological Process | GO:0035556 intracellular signal transduction | 0.001132 |
| Biological Process | GO:0035722 interleukin-12-mediated signaling pathway | 0.00328 |
| Biological Process | GO:0035883 enteroendocrine cell differentiation | 0.033934 |
| Biological Process | GO:0035994 response to muscle stretch | 0.024353 |
| Biological Process | GO:0036010 protein localization to endosome | 0.032343 |
| Biological Process | GO:0038029 epidermal growth factor receptor signaling pathway via MAPK cascade | 0.001641 |
| Biological Process | GO:0038127 ERBB signaling pathway | 0.006834 |
| Biological Process | GO:0042327 positive regulation of phosphorylation | 0.020657 |
| Biological Process | GO:0042692 muscle cell differentiation | 0.000418 |
| Biological Process | GO:0042743 hydrogen peroxide metabolic process | 0.035522 |
| Biological Process | GO:0042759 long-chain fatty acid biosynthetic process | 0.014681 |
| Biological Process | GO:0043085 positive regulation of catalytic activity | 0.027619 |
| Biological Process | GO:0043269 regulation of ion transport | 0.014098 |
| Biological Process | GO:0043270 positive regulation of ion transport | 0.02248 |
| Biological Process | GO:0043276 anoikis | 0.03869 |
| Biological Process | GO:0043300 regulation of leukocyte degranulation | 0.040271 |
| Biological Process | GO:0043303 mast cell degranulation | 0.037107 |
| Biological Process | GO:0043304 regulation of mast cell degranulation | 0.025956 |
| Biological Process | GO:0043405 regulation of MAP kinase activity | 0.038078 |
| Biological Process | GO:0043406 positive regulation of MAP kinase activity | 0.021311 |
| Biological Process | GO:0043434 response to peptide hormone | 0.002278 |
| Biological Process | GO:0043436 oxoacid metabolic process | 0.002598 |
| Biological Process | GO:0043457 regulation of cellular respiration | 0.041849 |
| Biological Process | GO:0043491 protein kinase B signaling | 0.019888 |
| Biological Process | GO:0043501 skeletal muscle adaptation | 0.022747 |
| Biological Process | GO:0043542 endothelial cell migration | 0.027092 |
| Biological Process | GO:0043549 regulation of kinase activity | 0.048413 |
| Biological Process | GO:0044028 DNA hypomethylation | 0.001641 |
| Biological Process | GO:0044029 hypomethylation of CpG island | 0.001641 |
| Biological Process | GO:0044030 regulation of DNA methylation | 0.021139 |
| Biological Process | GO:0044093 positive regulation of molecular function | 0.018627 |
| Biological Process | GO:0044242 cellular lipid catabolic process | 0.020452 |
| Biological Process | GO:0044255 cellular lipid metabolic process | 0.020657 |
| Biological Process | GO:0044281 small molecule metabolic process | 0.01189 |
| Biological Process | GO:0044342 type B pancreatic cell proliferation | 0.024353 |
| Biological Process | GO:0044346 fibroblast apoptotic process | 0.024353 |
| Biological Process | GO:0045054 constitutive secretory pathway | 0.00655 |
| Biological Process | GO:0045055 regulated exocytosis | 0.020169 |
| Biological Process | GO:0045197 establishment or maintenance of epithelial cell apical/basal polarity | 0.040271 |
| Biological Process | GO:0045198 establishment of epithelial cell apical/basal polarity | 0.024353 |
| Biological Process | GO:0045214 sarcomere organization | 0.000757 |
| Biological Process | GO:0045333 cellular respiration | 0.01249 |
| Biological Process | GO:0045576 mast cell activation | 0.046568 |
| Biological Process | GO:0045740 positive regulation of DNA replication | 0.037107 |
| Biological Process | GO:0045741 positive regulation of epidermal growth factor-activated receptor activity | 0.011436 |
| Biological Process | GO:0045742 positive regulation of epidermal growth factor receptor signaling pathway | 0.032343 |
| Biological Process | GO:0045822 negative regulation of heart contraction | 0.017915 |
| Biological Process | GO:0045859 regulation of protein kinase activity | 0.037737 |
| Biological Process | GO:0045860 positive regulation of protein kinase activity | 0.012246 |
| Biological Process | GO:0045937 positive regulation of phosphate metabolic process | 0.025434 |
| Biological Process | GO:0046395 carboxylic acid catabolic process | 0.022776 |
| Biological Process | GO:0046834 lipid phosphorylation | 0.049702 |
| Biological Process | GO:0046854 phosphatidylinositol phosphorylation | 0.040271 |
| Biological Process | GO:0046903 secretion | 0.001667 |
| Biological Process | GO:0048015 phosphatidylinositol-mediated signaling | 0.01272 |
| Biological Process | GO:0048017 inositol lipid-mediated signaling | 0.01342 |
| Biological Process | GO:0048514 blood vessel morphogenesis | 0.02774 |
| Biological Process | GO:0048609 multicellular organismal reproductive process | 0.03409 |
| Biological Process | GO:0048646 anatomical structure formation involved in morphogenesis | 0.033713 |
| Biological Process | GO:0048791 calcium ion-regulated exocytosis of neurotransmitter | 0.008182 |
| Biological Process | GO:0048878 chemical homeostasis | 0.023584 |
| Biological Process | GO:0050434 positive regulation of viral transcription | 0.027556 |
| Biological Process | GO:0050665 hydrogen peroxide biosynthetic process | 0.014681 |
| Biological Process | GO:0050673 epithelial cell proliferation | 0.011591 |
| Biological Process | GO:0050678 regulation of epithelial cell proliferation | 0.007011 |
| Biological Process | GO:0050679 positive regulation of epithelial cell proliferation | 0.017699 |
| Biological Process | GO:0050801 ion homeostasis | 0.037116 |
| Biological Process | GO:0051046 regulation of secretion | 0.002181 |
| Biological Process | GO:0051049 regulation of transport | 0.019631 |
| Biological Process | GO:0051050 positive regulation of transport | 0.008335 |
| Biological Process | GO:0051146 striated muscle cell differentiation | 0.029036 |
| Biological Process | GO:0051208 sequestering of calcium ion | 0.00649 |
| Biological Process | GO:0051209 release of sequestered calcium ion into cytosol | 0.006154 |
| Biological Process | GO:0051235 maintenance of location | 0.044113 |
| Biological Process | GO:0051279 regulation of release of sequestered calcium ion into cytosol | 0.002897 |
| Biological Process | GO:0051281 positive regulation of release of sequestered calcium ion into cytosol | 0.000951 |
| Biological Process | GO:0051282 regulation of sequestering of calcium ion | 0.006321 |
| Biological Process | GO:0051283 negative regulation of sequestering of calcium ion | 0.006154 |
| Biological Process | GO:0051347 positive regulation of transferase activity | 0.024008 |
| Biological Process | GO:0051480 regulation of cytosolic calcium ion concentration | 0.023977 |
| Biological Process | GO:0051599 response to hydrostatic pressure | 0.004917 |
| Biological Process | GO:0051896 regulation of protein kinase B signaling | 0.01438 |
| Biological Process | GO:0051897 positive regulation of protein kinase B signaling | 0.006661 |
| Biological Process | GO:0051924 regulation of calcium ion transport | 0.001213 |
| Biological Process | GO:0051928 positive regulation of calcium ion transport | 0.005043 |
| Biological Process | GO:0055001 muscle cell development | 0.000472 |
| Biological Process | GO:0055002 striated muscle cell development | 0.008484 |
| Biological Process | GO:0055065 metal ion homeostasis | 0.019109 |
| Biological Process | GO:0055074 calcium ion homeostasis | 0.006241 |
| Biological Process | GO:0055080 cation homeostasis | 0.025837 |
| Biological Process | GO:0055082 cellular chemical homeostasis | 0.004754 |
| Biological Process | GO:0055114 oxidation-reduction process | 0.021075 |
| Biological Process | GO:0055119 relaxation of cardiac muscle | 0.01306 |
| Biological Process | GO:0060047 heart contraction | 0.01878 |
| Biological Process | GO:0060317 cardiac epithelial to mesenchymal transition | 0.03869 |
| Biological Process | GO:0060341 regulation of cellular localization | 0.014748 |
| Biological Process | GO:0060390 regulation of SMAD protein signal transduction | 0.035522 |
| Biological Process | GO:0060391 positive regulation of SMAD protein signal transduction | 0.017915 |
| Biological Process | GO:0060396 growth hormone receptor signaling pathway | 0.014681 |
| Biological Process | GO:0060401 cytosolic calcium ion transport | 0.000566 |
| Biological Process | GO:0060402 calcium ion transport into cytosol | 0.009471 |
| Biological Process | GO:0060416 response to growth hormone | 0.016299 |
| Biological Process | GO:0060612 adipose tissue development | 0.044998 |
| Biological Process | GO:0060627 regulation of vesicle-mediated transport | 0.014831 |
| Biological Process | GO:0060749 mammary gland alveolus development | 0.025956 |
| Biological Process | GO:0061061 muscle structure development | 0.003313 |
| Biological Process | GO:0061162 establishment of monopolar cell polarity | 0.032343 |
| Biological Process | GO:0061245 establishment or maintenance of bipolar cell polarity | 0.046568 |
| Biological Process | GO:0061339 establishment or maintenance of monopolar cell polarity | 0.033934 |
| Biological Process | GO:0061377 mammary gland lobule development | 0.025956 |
| Biological Process | GO:0061469 regulation of type B pancreatic cell proliferation | 0.014681 |
| Biological Process | GO:0061635 regulation of protein complex stability | 0.00981 |
| Biological Process | GO:0065009 regulation of molecular function | 0.049965 |
| Biological Process | GO:0070296 sarcoplasmic reticulum calcium ion transport | 0.000434 |
| Biological Process | GO:0070542 response to fatty acid | 0.043425 |
| Biological Process | GO:0070588 calcium ion transmembrane transport | 0.000187 |
| Biological Process | GO:0070671 response to interleukin-12 | 0.008182 |
| Biological Process | GO:0070838 divalent metal ion transport | 7.58E-05 |
| Biological Process | GO:0070848 response to growth factor | 0.03409 |
| Biological Process | GO:0070849 response to epidermal growth factor | 0.043425 |
| Biological Process | GO:0071349 cellular response to interleukin-12 | 0.004917 |
| Biological Process | GO:0071363 cellular response to growth factor stimulus | 0.032341 |
| Biological Process | GO:0071364 cellular response to epidermal growth factor stimulus | 0.040271 |
| Biological Process | GO:0071375 cellular response to peptide hormone stimulus | 0.021601 |
| Biological Process | GO:0071378 cellular response to growth hormone stimulus | 0.014681 |
| Biological Process | GO:0071462 cellular response to water stimulus | 0.004917 |
| Biological Process | GO:0071464 cellular response to hydrostatic pressure | 0.00328 |
| Biological Process | GO:0071514 genetic imprinting | 0.03075 |
| Biological Process | GO:0071559 response to transforming growth factor beta | 0.032741 |
| Biological Process | GO:0071560 cellular response to transforming growth factor beta stimulus | 0.031713 |
| Biological Process | GO:0071711 basement membrane organization | 0.029155 |
| Biological Process | GO:0071902 positive regulation of protein serine/threonine kinase activity | 0.037712 |
| Biological Process | GO:0072329 monocarboxylic acid catabolic process | 0.006154 |
| Biological Process | GO:0072350 tricarboxylic acid metabolic process | 0.037107 |
| Biological Process | GO:0072358 cardiovascular system development | 0.047471 |
| Biological Process | GO:0072359 circulatory system development | 0.027371 |
| Biological Process | GO:0072503 cellular divalent inorganic cation homeostasis | 0.007011 |
| Biological Process | GO:0072507 divalent inorganic cation homeostasis | 0.008181 |
| Biological Process | GO:0072511 divalent inorganic cation transport | 7.58E-05 |
| Biological Process | GO:0086036 regulation of cardiac muscle cell membrane potential | 0.00328 |
| Biological Process | GO:0090075 relaxation of muscle | 0.029155 |
| Biological Process | GO:0090092 regulation of transmembrane receptor protein serine/threonine kinase signaling pathway | 0.037348 |
| Biological Process | GO:0090130 tissue migration | 0.04886 |
| Biological Process | GO:0090132 epithelium migration | 0.048056 |
| Biological Process | GO:0090162 establishment of epithelial cell polarity | 0.037107 |
| Biological Process | GO:0090257 regulation of muscle system process | 0.01878 |
| Biological Process | GO:0090279 regulation of calcium ion import | 0.03869 |
| Biological Process | GO:0090370 negative regulation of cholesterol efflux | 0.004917 |
| Biological Process | GO:0097009 energy homeostasis | 0.035522 |
| Biological Process | GO:0097352 autophagosome maturation | 0.048137 |
| Biological Process | GO:0097435 supramolecular fiber organization | 0.041579 |
| Biological Process | GO:0097553 calcium ion transmembrane import into cytosol | 0.007731 |
| Biological Process | GO:0098655 cation transmembrane transport | 0.00672 |
| Biological Process | GO:0098660 inorganic ion transmembrane transport | 0.005203 |
| Biological Process | GO:0098662 inorganic cation transmembrane transport | 0.004077 |
| Biological Process | GO:0098771 inorganic ion homeostasis | 0.027389 |
| Biological Process | GO:0101023 vascular endothelial cell proliferation | 0.024353 |
| Biological Process | GO:1900120 regulation of receptor binding | 0.033934 |
| Biological Process | GO:1900121 negative regulation of receptor binding | 0.022747 |
| Biological Process | GO:1900125 regulation of hyaluronan biosynthetic process | 0.008182 |
| Biological Process | GO:1900127 positive regulation of hyaluronan biosynthetic process | 0.004917 |
| Biological Process | GO:1901021 positive regulation of calcium ion transmembrane transporter activity | 0.033934 |
| Biological Process | GO:1901186 positive regulation of ERBB signaling pathway | 0.035522 |
| Biological Process | GO:1901339 regulation of store-operated calcium channel activity | 0.011436 |
| Biological Process | GO:1901341 positive regulation of store-operated calcium channel activity | 0.00981 |
| Biological Process | GO:1901652 response to peptide | 0.004546 |
| Biological Process | GO:1901653 cellular response to peptide | 0.031035 |
| Biological Process | GO:1901700 response to oxygen-containing compound | 0.038696 |
| Biological Process | GO:1902946 protein localization to early endosome | 0.01306 |
| Biological Process | GO:1902965 regulation of protein localization to early endosome | 0.011436 |
| Biological Process | GO:1902966 positive regulation of protein localization to early endosome | 0.011436 |
| Biological Process | GO:1903169 regulation of calcium ion transmembrane transport | 0.007916 |
| Biological Process | GO:1903233 regulation of calcium ion-dependent exocytosis of neurotransmitter | 0.00328 |
| Biological Process | GO:1903305 regulation of regulated secretory pathway | 0.00907 |
| Biological Process | GO:1903514 release of sequestered calcium ion into cytosol by endoplasmic reticulum | 0.029155 |
| Biological Process | GO:1903515 calcium ion transport from cytosol to endoplasmic reticulum | 0.00328 |
| Biological Process | GO:1903523 negative regulation of blood circulation | 0.029155 |
| Biological Process | GO:1903530 regulation of secretion by cell | 0.013978 |
| Biological Process | GO:1903672 positive regulation of sprouting angiogenesis | 0.049702 |
| Biological Process | GO:1903829 positive regulation of cellular protein localization | 0.047656 |
| Biological Process | GO:1904062 regulation of cation transmembrane transport | 0.033434 |
| Biological Process | GO:1904064 positive regulation of cation transmembrane transport | 0.00649 |
| Biological Process | GO:1904427 positive regulation of calcium ion transmembrane transport | 0.002036 |
| Biological Process | GO:1904691 negative regulation of type B pancreatic cell proliferation | 0.00328 |
| Biological Process | GO:1905276 regulation of epithelial tube formation | 0.025956 |
| Biological Process | GO:1905278 positive regulation of epithelial tube formation | 0.00981 |
| Biological Process | GO:1905332 positive regulation of morphogenesis of an epithelium | 0.046568 |
| Biological Process | GO:1905562 regulation of vascular endothelial cell proliferation | 0.024353 |
| Biological Process | GO:1905564 positive regulation of vascular endothelial cell proliferation | 0.017915 |
| Biological Process | GO:1905666 regulation of protein localization to endosome | 0.017915 |
| Biological Process | GO:1905668 positive regulation of protein localization to endosome | 0.016299 |
| Biological Process | GO:1905953 negative regulation of lipid localization | 0.041849 |
| Biological Process | GO:1990456 mitochondrion-endoplasmic reticulum membrane tethering | 0.00981 |
| Biological Process | GO:2000008 regulation of protein localization to cell surface | 0.040271 |
| Biological Process | GO:2000209 regulation of anoikis | 0.027556 |
| Biological Process | GO:2000269 regulation of fibroblast apoptotic process | 0.022747 |
| Biological Process | GO:2000270 negative regulation of fibroblast apoptotic process | 0.011436 |
| Biological Process | GO:2000273 positive regulation of signaling receptor activity | 0.03869 |
| Biological Process | GO:2000352 negative regulation of endothelial cell apoptotic process | 0.03869 |
| Biological Process | GO:2000653 regulation of genetic imprinting | 0.00655 |
| Biological Process | GO:2000811 negative regulation of anoikis | 0.021139 |
| Biological Process | GO:2001256 regulation of store-operated calcium entry | 0.01306 |
| Cellular Component | GO:0005622 intracellular | 0.033608 |
| Cellular Component | GO:0005623 cell | 0.002538 |
| Cellular Component | GO:0005777 peroxisome | 0.006591 |
| Cellular Component | GO:0005778 peroxisomal membrane | 0.039673 |
| Cellular Component | GO:0005856 cytoskeleton | 0.045212 |
| Cellular Component | GO:0005942 phosphatidylinositol 3-kinase complex | 0.01719 |
| Cellular Component | GO:0005943 phosphatidylinositol 3-kinase complex, class IA | 0.001443 |
| Cellular Component | GO:0014704 intercalated disc | 0.03689 |
| Cellular Component | GO:0016528 sarcoplasm | 0.000842 |
| Cellular Component | GO:0016529 sarcoplasmic reticulum | 0.000583 |
| Cellular Component | GO:0030008 TRAPP complex | 0.012919 |
| Cellular Component | GO:0030016 myofibril | 0.009727 |
| Cellular Component | GO:0030017 sarcomere | 0.007049 |
| Cellular Component | GO:0031430 M band | 0.010062 |
| Cellular Component | GO:0031672 A band | 0.01861 |
| Cellular Component | GO:0031903 microbody membrane | 0.039673 |
| Cellular Component | GO:0033017 sarcoplasmic reticulum membrane | 0.014345 |
| Cellular Component | GO:0033018 sarcoplasmic reticulum lumen | 0.001443 |
| Cellular Component | GO:0034673 inhibin-betaglycan-ActRII complex | 0.004324 |
| Cellular Component | GO:0042579 microbody | 0.006591 |
| Cellular Component | GO:0043226 organelle | 0.00138 |
| Cellular Component | GO:0043227 membrane-bounded organelle | 0.038884 |
| Cellular Component | GO:0043229 intracellular organelle | 0.004698 |
| Cellular Component | GO:0043292 contractile fiber | 0.010643 |
| Cellular Component | GO:0044424 intracellular part | 0.033608 |
| Cellular Component | GO:0044438 microbody part | 0.045217 |
| Cellular Component | GO:0044439 peroxisomal part | 0.045217 |
| Cellular Component | GO:0044449 contractile fiber part | 0.008172 |
| Cellular Component | GO:0044464 cell part | 0.002538 |
| Cellular Component | GO:0045202 synapse | 0.048116 |
| Cellular Component | GO:0097470 ribbon synapse | 0.011492 |
| Cellular Component | GO:0097651 phosphatidylinositol 3-kinase complex, class I | 0.005762 |
| Molecular Function | GO:0000062 fatty-acyl-CoA binding | 0.019292 |
| Molecular Function | GO:0000166 nucleotide binding | 0.006113 |
| Molecular Function | GO:0003997 acyl-CoA oxidase activity | 0.010568 |
| Molecular Function | GO:0004077 biotin-[acetyl-CoA-carboxylase] ligase activity | 0.001769 |
| Molecular Function | GO:0004675 transmembrane receptor protein serine/threonine kinase activity | 0.027943 |
| Molecular Function | GO:0004676 3-phosphoinositide-dependent protein kinase activity | 0.001769 |
| Molecular Function | GO:0005024 transforming growth factor beta-activated receptor activity | 0.010568 |
| Molecular Function | GO:0005114 type II transforming growth factor beta receptor binding | 0.008814 |
| Molecular Function | GO:0005154 epidermal growth factor receptor binding | 0.041631 |
| Molecular Function | GO:0005160 transforming growth factor beta receptor binding | 0.031383 |
| Molecular Function | GO:0005388 calcium-transporting ATPase activity | 0.015812 |
| Molecular Function | GO:0005488 binding | 0.001559 |
| Molecular Function | GO:0005509 calcium ion binding | 0.016484 |
| Molecular Function | GO:0005515 protein binding | 0.004526 |
| Molecular Function | GO:0005524 ATP binding | 0.043041 |
| Molecular Function | GO:0008047 enzyme activator activity | 0.01374 |
| Molecular Function | GO:0015026 coreceptor activity | 0.03481 |
| Molecular Function | GO:0015662 ATPase activity, coupled to transmembrane movement of ions, phosphorylative mechanism | 0.026219 |
| Molecular Function | GO:0016004 phospholipase activator activity | 0.014067 |
| Molecular Function | GO:0016301 kinase activity | 0.028516 |
| Molecular Function | GO:0016303 1-phosphatidylinositol-3-kinase activity | 0.007057 |
| Molecular Function | GO:0016634 oxidoreductase activity, acting on the CH-CH group of donors, oxygen as acceptor | 0.015812 |
| Molecular Function | GO:0016772 transferase activity, transferring phosphorus-containing groups | 0.048083 |
| Molecular Function | GO:0016773 phosphotransferase activity, alcohol group as acceptor | 0.022516 |
| Molecular Function | GO:0017076 purine nucleotide binding | 0.039728 |
| Molecular Function | GO:0018271 biotin-protein ligase activity | 0.001769 |
| Molecular Function | GO:0019904 protein domain specific binding | 0.036576 |
| Molecular Function | GO:0030332 cyclin binding | 0.043329 |
| Molecular Function | GO:0030554 adenyl nucleotide binding | 0.013602 |
| Molecular Function | GO:0030957 Tat protein binding | 0.014067 |
| Molecular Function | GO:0032553 ribonucleotide binding | 0.039273 |
| Molecular Function | GO:0032555 purine ribonucleotide binding | 0.03793 |
| Molecular Function | GO:0032559 adenyl ribonucleotide binding | 0.013172 |
| Molecular Function | GO:0035004 phosphatidylinositol 3-kinase activity | 0.014067 |
| Molecular Function | GO:0035173 histone kinase activity | 0.021028 |
| Molecular Function | GO:0035400 histone tyrosine kinase activity | 0.001769 |
| Molecular Function | GO:0035401 histone kinase activity (H3-Y41 specific) | 0.001769 |
| Molecular Function | GO:0036094 small molecule binding | 0.014725 |
| Molecular Function | GO:0042805 actinin binding | 0.031383 |
| Molecular Function | GO:0043167 ion binding | 0.037873 |
| Molecular Function | GO:0043168 anion binding | 0.02307 |
| Molecular Function | GO:0043274 phospholipase binding | 0.022761 |
| Molecular Function | GO:0043560 insulin receptor substrate binding | 0.012319 |
| Molecular Function | GO:0044548 S100 protein binding | 0.019292 |
| Molecular Function | GO:0048037 cofactor binding | 0.005159 |
| Molecular Function | GO:0050431 transforming growth factor beta binding | 0.031383 |
| Molecular Function | GO:0050660 flavin adenine dinucleotide binding | 0.006199 |
| Molecular Function | GO:0050662 coenzyme binding | 0.007811 |
| Molecular Function | GO:0051371 muscle alpha-actinin binding | 0.007057 |
| Molecular Function | GO:0051393 alpha-actinin binding | 0.019292 |
| Molecular Function | GO:0052742 phosphatidylinositol kinase activity | 0.014067 |
| Molecular Function | GO:0060229 lipase activator activity | 0.015812 |
| Molecular Function | GO:0070123 transforming growth factor beta receptor activity, type III | 0.001769 |
| Molecular Function | GO:0086039 calcium-transporting ATPase activity involved in regulation of cardiac muscle cell membrane potential | 0.001769 |
| Molecular Function | GO:1901265 nucleoside phosphate binding | 0.006113 |
| Molecular Function | GO:1901567 fatty acid derivative binding | 0.024492 |

Supplementary Table 8 KEGG analysis of source genes of DE circRNAs related to skeletal muscle development

| B_Pathway | C_Pathway | L-vs-H |
| --- | --- | --- |
| Cancer: overview | ko05235 PD-L1 expression and PD-1 checkpoint pathway in cancer | 0.000415 |
| Cancers | ko05200 Pathways in cancer | 0.018447 |
| Cancers | ko05205 Proteoglycans in cancer | 0.022492 |
| Cancers | ko05210 Colorectal cancer | 0.004456 |
| Cancers | ko05212 Pancreatic cancer | 0.003366 |
| Cancers | ko05213 Endometrial cancer | 3.20E-05 |
| Cancers | ko05214 Glioma | 0.003282 |
| Cancers | ko05215 Prostate cancer | 0.000154 |
| Cancers | ko05218 Melanoma | 0.002875 |
| Cancers | ko05219 Bladder cancer | 0.043681 |
| Cancers | ko05223 Non-small cell lung cancer | 4.95E-05 |
| Cancers | ko05224 Breast cancer | 0.011233 |
| Cancers | ko05226 Gastric cancer | 0.011683 |
| Cancers | ko05231 Choline metabolism in cancer | 0.000149 |
| Carbohydrate metabolism | ko00020 Citrate cycle (TCA cycle) | 0.032647 |
| Carbohydrate metabolism | ko00640 Propanoate metabolism | 0.000646 |
| Cell growth and death | ko04210 Apoptosis | 0.010501 |
| Cell motility | ko04810 Regulation of actin cytoskeleton | 0.040752 |
| Cellular community - eukaryotes | ko04510 Focal adhesion | 0.001112 |
| Cellular community - eukaryotes | ko04550 Signaling pathways regulating pluripotency of stem cells | 0.011532 |
| Drug resistance | ko01521 EGFR tyrosine kinase inhibitor resistance | 9.06E-05 |
| Drug resistance | ko01524 Platinum drug resistance | 0.002955 |
| Endocrine and metabolic diseases | ko04931 Insulin resistance | 0.007183 |
| Endocrine and metabolic diseases | ko04933 AGE-RAGE signaling pathway in diabetic complications | 0.006474 |
| Endocrine system | ko03320 PPAR signaling pathway | 0.003713 |
| Endocrine system | ko04910 Insulin signaling pathway | 0.011532 |
| Endocrine system | ko04917 Prolactin signaling pathway | 0.002797 |
| Endocrine system | ko04919 Thyroid hormone signaling pathway | 0.000248 |
| Endocrine system | ko04935 Growth hormone synthesis, secretion and action | 0.007551 |
| Excretory system | ko04960 Aldosterone-regulated sodium reabsorption | 0.000724 |
| Global and overview maps | ko01200 Carbon metabolism | 0.007183 |
| Immune system | ko04062 Chemokine signaling pathway | 0.015737 |
| Immune system | ko04660 T cell receptor signaling pathway | 0.012295 |
| Immune system | ko04664 Fc epsilon RI signaling pathway | 0.002797 |
| Infectious diseases | ko05145 Toxoplasmosis | 0.007305 |
| Infectious diseases | ko05160 Hepatitis C | 0.012764 |
| Infectious diseases | ko05161 Hepatitis B | 0.013887 |
| Infectious diseases | ko05164 Influenza A | 0.015223 |
| Infectious diseases | ko05167 Kaposi sarcoma-associated herpesvirus infection | 0.017508 |
| Lipid metabolism | ko00071 Fatty acid degradation | 0.048062 |
| Lipid metabolism | ko00592 alpha-Linolenic acid metabolism | 0.025973 |
| Lipid metabolism | ko01040 Biosynthesis of unsaturated fatty acids | 0.029315 |
| Metabolism of cofactors and vitamins | ko00780 Biotin metabolism | 0.003423 |
| Metabolism of other amino acids | ko00410 beta-Alanine metabolism | 0.038178 |
| Nervous system | ko04722 Neurotrophin signaling pathway | 0.007927 |
| Nervous system | ko04725 Cholinergic synapse | 0.007305 |
| Neurodegenerative disease | ko05017 Spinocerebellar ataxia | 0.005159 |
| Signal transduction | ko04012 ErbB signaling pathway | 0.003803 |
| Signal transduction | ko04014 Ras signaling pathway | 0.037446 |
| Signal transduction | ko04020 Calcium signaling pathway | 0.031179 |
| Signal transduction | ko04024 cAMP signaling pathway | 0.00192 |
| Signal transduction | ko04066 HIF-1 signaling pathway | 0.007551 |
| Signal transduction | ko04068 FoxO signaling pathway | 0.000389 |
| Signal transduction | ko04071 Sphingolipid signaling pathway | 0.007427 |
| Signal transduction | ko04072 Phospholipase D signaling pathway | 0.012764 |
| Signal transduction | ko04150 mTOR signaling pathway | 0.014051 |
| Signal transduction | ko04151 PI3K-Akt signaling pathway | 0.000435 |
| Signal transduction | ko04152 AMPK signaling pathway | 0.008704 |
| Signal transduction | ko04630 Jak-STAT signaling pathway | 0.00069 |
| Transport and catabolism | ko04140 Autophagy - animal | 0.011233 |

Supplementary Table 9 The specific information of QTL region

| DE gene | Chr | Start | End | QTL | Chr | Start | End |
| --- | --- | --- | --- | --- | --- | --- | --- |
| ENSSSCG00000049158 | 1 | 13972615 | 14024472 | Drip loss QTL (8365) | 1 | 13770316 | 14003408 |
| ENSSSCG00000049158 | 1 | 13972615 | 14024472 | Drip loss QTL (8487) | 1 | 13770316 | 14003408 |
| ENSSSCG00000049158 | 1 | 13972615 | 14024472 | Drip loss QTL (8571) | 1 | 13770316 | 14003408 |
| ENSSSCG00000049158 | 1 | 13972615 | 14024472 | Drip loss QTL (7859) | 1 | 13770316 | 14003408 |
| ENSSSCG00000049158 | 1 | 13972615 | 14024472 | Drip loss QTL (8294) | 1 | 13770316 | 14003408 |
| ENSSSCG00000049158 | 1 | 13972615 | 14024472 | Drip loss QTL (8302) | 1 | 13770316 | 14003408 |
| ENSSSCG00000049158 | 1 | 13972615 | 14024472 | Water holding capacity QTL (3848) | 1 | 5379030 | 52355892 |
| ENSSSCG00000049158 | 1 | 13972615 | 14024472 | Palmitoleic acid content QTL (3109) | 1 | 5379030 | 29405363 |
| ENSSSCG00000049158 | 1 | 13972615 | 14024472 | Stearic acid content QTL (3111) | 1 | 5379030 | 29405363 |
| ENSSSCG00000049158 | 1 | 13972615 | 14024472 | Palmitoleic acid content QTL (3118) | 1 | 5379030 | 29405363 |
| ENSSSCG00000049158 | 1 | 13972615 | 14024472 | Stearic acid content QTL (3119) | 1 | 5379030 | 29405363 |
| ENSSSCG00000049158 | 1 | 13972615 | 14024472 | Average daily gain QTL (5680) | 1 | 5379030 | 2.52E+08 |
| ENSSSCG00000049158 | 1 | 13972615 | 14024472 | Shoulder subcutaneous fat thickness QTL (5671) | 1 | 5379030 | 1.98E+08 |
| ENSSSCG00000049158 | 1 | 13972615 | 14024472 | Average backfat thickness QTL (5670) | 1 | 5379030 | 1.68E+08 |
| ENSSSCG00000049158 | 1 | 13972615 | 14024472 | Shoulder subcutaneous fat thickness QTL (21209) | 1 | 7172040 | 2.51E+08 |
| ENSSSCG00000049158 | 1 | 13972615 | 14024472 | Drip loss QTL (8349) | 1 | 10256017 | 1E+08 |
| ENSSSCG00000049158 | 1 | 13972615 | 14024472 | Drip loss QTL (8411) | 1 | 10256017 | 1E+08 |
| ENSSSCG00000049158 | 1 | 13972615 | 14024472 | Drip loss QTL (8562) | 1 | 10256017 | 1E+08 |
| ENSSSCG00000049158 | 1 | 13972615 | 14024472 | Drip loss QTL (7776) | 1 | 10256017 | 1E+08 |
| ENSSSCG00000049158 | 1 | 13972615 | 14024472 | Drip loss QTL (7815) | 1 | 10256017 | 1E+08 |
| ENSSSCG00000049158 | 1 | 13972615 | 14024472 | Drip loss QTL (8012) | 1 | 10256017 | 1E+08 |
| ENSSSCG00000049158 | 1 | 13972615 | 14024472 | Drip loss QTL (8043) | 1 | 10256017 | 1E+08 |
| ENSSSCG00000049158 | 1 | 13972615 | 14024472 | Drip loss QTL (8110) | 1 | 10256017 | 1E+08 |
| ENSSSCG00000049158 | 1 | 13972615 | 14024472 | Drip loss QTL (8154) | 1 | 10256017 | 1E+08 |
| ENSSSCG00000049158 | 1 | 13972615 | 14024472 | Drip loss QTL (7751) | 1 | 10256017 | 1E+08 |
| ENSSSCG00000049158 | 1 | 13972615 | 14024472 | Drip loss QTL (7763) | 1 | 10256017 | 1E+08 |
| ENSSSCG00000004203 | 1 | 33198301 | 33319367 | Water holding capacity QTL (3848) | 1 | 5379030 | 52355892 |
| ENSSSCG00000004203 | 1 | 33198301 | 33319367 | Loin muscle area QTL (21704) | 1 | 14344080 | 46618260 |
| ENSSSCG00000004203 | 1 | 33198301 | 33319367 | Drip loss QTL (8324) | 1 | 31252164 | 39840682 |
| ENSSSCG00000004203 | 1 | 33198301 | 33319367 | Drip loss QTL (8597) | 1 | 31252164 | 39840682 |
| ENSSSCG00000004203 | 1 | 33198301 | 33319367 | Drip loss QTL (7791) | 1 | 31252164 | 39840682 |
| ENSSSCG00000004203 | 1 | 33198301 | 33319367 | Drip loss QTL (7793) | 1 | 31252164 | 39840682 |
| ENSSSCG00000004203 | 1 | 33198301 | 33319367 | Drip loss QTL (7821) | 1 | 31252164 | 39840682 |
| ENSSSCG00000004203 | 1 | 33198301 | 33319367 | Drip loss QTL (7828) | 1 | 31252164 | 39840682 |
| ENSSSCG00000004203 | 1 | 33198301 | 33319367 | Drip loss QTL (7963) | 1 | 31252164 | 39840682 |

| ENSSSCG00000004203 | 1 | 33198301 | 33319367 | Drip loss QTL (8052) | 1 | 31252164 | 39840682 |
| --- | --- | --- | --- | --- | --- | --- | --- |
| ENSSSCG00000004203 | 1 | 33198301 | 33319367 | Average daily gain QTL (5680) | 1 | 5379030 | 2.52E+08 |
| ENSSSCG00000004203 | 1 | 33198301 | 33319367 | Shoulder subcutaneous fat thickness QTL (5671) | 1 | 5379030 | 1.98E+08 |
| ENSSSCG00000004203 | 1 | 33198301 | 33319367 | Average backfat thickness QTL (5670) | 1 | 5379030 | 1.68E+08 |
| ENSSSCG00000004203 | 1 | 33198301 | 33319367 | Shoulder subcutaneous fat thickness QTL (21209) | 1 | 7172040 | 2.51E+08 |
| ENSSSCG00000004203 | 1 | 33198301 | 33319367 | Drip loss QTL (8349) | 1 | 10256017 | 1E+08 |
| ENSSSCG00000004203 | 1 | 33198301 | 33319367 | Drip loss QTL (8411) | 1 | 10256017 | 1E+08 |
| ENSSSCG00000004203 | 1 | 33198301 | 33319367 | Drip loss QTL (8562) | 1 | 10256017 | 1E+08 |
| ENSSSCG00000004203 | 1 | 33198301 | 33319367 | Drip loss QTL (7776) | 1 | 10256017 | 1E+08 |
| ENSSSCG00000004203 | 1 | 33198301 | 33319367 | Drip loss QTL (7815) | 1 | 10256017 | 1E+08 |
| ENSSSCG00000004203 | 1 | 33198301 | 33319367 | Drip loss QTL (8012) | 1 | 10256017 | 1E+08 |
| ENSSSCG00000004203 | 1 | 33198301 | 33319367 | Drip loss QTL (8043) | 1 | 10256017 | 1E+08 |
| ENSSSCG00000004203 | 1 | 33198301 | 33319367 | Drip loss QTL (8110) | 1 | 10256017 | 1E+08 |
| ENSSSCG00000004203 | 1 | 33198301 | 33319367 | Drip loss QTL (8154) | 1 | 10256017 | 1E+08 |
| ENSSSCG00000004203 | 1 | 33198301 | 33319367 | Drip loss QTL (7751) | 1 | 10256017 | 1E+08 |
| ENSSSCG00000004203 | 1 | 33198301 | 33319367 | Drip loss QTL (7763) | 1 | 10256017 | 1E+08 |
| ENSSSCG00000004203 | 1 | 33198301 | 33319367 | Backfat at last rib QTL (21208) | 1 | 15061284 | 2.48E+08 |
| ENSSSCG00000004203 | 1 | 33198301 | 33319367 | Shoulder subcutaneous fat thickness QTL (5217) | 1 | 29405363 | 2.48E+08 |
| ENSSSCG00000004203 | 1 | 33198301 | 33319367 | Backfat at last rib QTL (5674) | 1 | 29405363 | 1.98E+08 |
| ENSSSCG00000004203 | 1 | 33198301 | 33319367 | Percentage type IIa fibers QTL (7015) | 1 | 29405363 | 93774423 |
| ENSSSCG00000004203 | 1 | 33198301 | 33319367 | PH for Longissmus dorsi QTL (2787) | 1 | 29405363 | 79968246 |
| ENSSSCG00000004203 | 1 | 33198301 | 33319367 | Leaf fat weight QTL (3863) | 1 | 30301868 | 93595122 |
| ENSSSCG00000004276 | 1 | 50721676 | 50942991 | Water holding capacity QTL (3848) | 1 | 5379030 | 52355892 |
| ENSSSCG00000004276 | 1 | 50721676 | 50942991 | Average daily gain QTL (5680) | 1 | 5379030 | 2.52E+08 |
| ENSSSCG00000004276 | 1 | 50721676 | 50942991 | Shoulder subcutaneous fat thickness QTL (5671) | 1 | 5379030 | 1.98E+08 |
| ENSSSCG00000004276 | 1 | 50721676 | 50942991 | Average backfat thickness QTL (5670) | 1 | 5379030 | 1.68E+08 |
| ENSSSCG00000004276 | 1 | 50721676 | 50942991 | Shoulder subcutaneous fat thickness QTL (21209) | 1 | 7172040 | 2.51E+08 |
| ENSSSCG00000004276 | 1 | 50721676 | 50942991 | Drip loss QTL (8349) | 1 | 10256017 | 1E+08 |
| ENSSSCG00000004276 | 1 | 50721676 | 50942991 | Drip loss QTL (8411) | 1 | 10256017 | 1E+08 |
| ENSSSCG00000004276 | 1 | 50721676 | 50942991 | Drip loss QTL (8562) | 1 | 10256017 | 1E+08 |
| ENSSSCG00000004276 | 1 | 50721676 | 50942991 | Drip loss QTL (7776) | 1 | 10256017 | 1E+08 |
| ENSSSCG00000004276 | 1 | 50721676 | 50942991 | Drip loss QTL (7815) | 1 | 10256017 | 1E+08 |
| ENSSSCG00000004276 | 1 | 50721676 | 50942991 | Drip loss QTL (8012) | 1 | 10256017 | 1E+08 |
| ENSSSCG00000004276 | 1 | 50721676 | 50942991 | Drip loss QTL (8043) | 1 | 10256017 | 1E+08 |
| ENSSSCG00000004276 | 1 | 50721676 | 50942991 | Drip loss QTL (8110) | 1 | 10256017 | 1E+08 |
| ENSSSCG00000004276 | 1 | 50721676 | 50942991 | Drip loss QTL (8154) | 1 | 10256017 | 1E+08 |
| ENSSSCG00000004276 | 1 | 50721676 | 50942991 | Drip loss QTL (7751) | 1 | 10256017 | 1E+08 |
| ENSSSCG00000004276 | 1 | 50721676 | 50942991 | Drip loss QTL (7763) | 1 | 10256017 | 1E+08 |
| ENSSSCG00000004276 | 1 | 50721676 | 50942991 | Backfat at last rib QTL (21208) | 1 | 15061284 | 2.48E+08 |
| ENSSSCG00000004276 | 1 | 50721676 | 50942991 | Shoulder subcutaneous fat thickness QTL (5217) | 1 | 29405363 | 2.48E+08 |
| ENSSSCG00000004276 | 1 | 50721676 | 50942991 | Backfat at last rib QTL (5674) | 1 | 29405363 | 1.98E+08 |
| ENSSSCG00000004276 | 1 | 50721676 | 50942991 | Percentage type IIa fibers QTL (7015) | 1 | 29405363 | 93774423 |
| ENSSSCG00000004276 | 1 | 50721676 | 50942991 | PH for Longissmus dorsi QTL (2787) | 1 | 29405363 | 79968246 |
| ENSSSCG00000004276 | 1 | 50721676 | 50942991 | Leaf fat weight QTL (3863) | 1 | 30301868 | 93595122 |
| ENSSSCG00000004276 | 1 | 50721676 | 50942991 | Body weight (20 weeks) QTL (29689) | 1 | 42135735 | 1.44E+08 |
| ENSSSCG00000004276 | 1 | 50721676 | 50942991 | Meat color a* QTL (3980) | 1 | 42135735 | 88574694 |
| ENSSSCG00000004276 | 1 | 50721676 | 50942991 | Average daily gain QTL (338) | 1 | 42135735 | 79968246 |
| ENSSSCG00000004328 | 1 | 57660224 | 57826368 | Fat androstenone level QTL (15821) | 1 | 57670915 | 57670919 |
| ENSSSCG00000004328 | 1 | 57660224 | 57826368 | Drip loss QTL (7922) | 1 | 56730836 | 58595566 |
| ENSSSCG00000004328 | 1 | 57660224 | 57826368 | Drip loss QTL (7929) | 1 | 56730836 | 58595566 |
| ENSSSCG00000004328 | 1 | 57660224 | 57826368 | Drip loss QTL (7956) | 1 | 56730836 | 58595566 |
| ENSSSCG00000004328 | 1 | 57660224 | 57826368 | Drip loss QTL (7987) | 1 | 56730836 | 58595566 |
| ENSSSCG00000004328 | 1 | 57660224 | 57826368 | Drip loss QTL (8096) | 1 | 56730836 | 58595566 |
| ENSSSCG00000004328 | 1 | 57660224 | 57826368 | Drip loss QTL (8201) | 1 | 56730836 | 58595566 |
| ENSSSCG00000004328 | 1 | 57660224 | 57826368 | Drip loss QTL (8243) | 1 | 56730836 | 58595566 |
| ENSSSCG00000004328 | 1 | 57660224 | 57826368 | Drip loss QTL (8335) | 1 | 56730836 | 58595566 |
| ENSSSCG00000004328 | 1 | 57660224 | 57826368 | Drip loss QTL (8346) | 1 | 56730836 | 58595566 |
| ENSSSCG00000004328 | 1 | 57660224 | 57826368 | Drip loss QTL (8496) | 1 | 56730836 | 58595566 |
| ENSSSCG00000004328 | 1 | 57660224 | 57826368 | Drip loss QTL (8505) | 1 | 56730836 | 58595566 |
| ENSSSCG00000004328 | 1 | 57660224 | 57826368 | Drip loss QTL (7737) | 1 | 56730836 | 58595566 |
| ENSSSCG00000004328 | 1 | 57660224 | 57826368 | pH 24 hr post mortem (ham) QTL (31524) | 1 | 54471643 | 63920806 |
| ENSSSCG00000004328 | 1 | 57660224 | 57826368 | Average daily gain QTL (5680) | 1 | 5379030 | 2.52E+08 |
| ENSSSCG00000004328 | 1 | 57660224 | 57826368 | Shoulder subcutaneous fat thickness QTL (5671) | 1 | 5379030 | 1.98E+08 |
| ENSSSCG00000004328 | 1 | 57660224 | 57826368 | Average backfat thickness QTL (5670) | 1 | 5379030 | 1.68E+08 |
| ENSSSCG00000004328 | 1 | 57660224 | 57826368 | Shoulder subcutaneous fat thickness QTL (21209) | 1 | 7172040 | 2.51E+08 |
| ENSSSCG00000004328 | 1 | 57660224 | 57826368 | Drip loss QTL (8349) | 1 | 10256017 | 1E+08 |
| ENSSSCG00000004328 | 1 | 57660224 | 57826368 | Drip loss QTL (8411) | 1 | 10256017 | 1E+08 |
| ENSSSCG00000004328 | 1 | 57660224 | 57826368 | Drip loss QTL (8562) | 1 | 10256017 | 1E+08 |
| ENSSSCG00000004328 | 1 | 57660224 | 57826368 | Drip loss QTL (7776) | 1 | 10256017 | 1E+08 |
| ENSSSCG00000004328 | 1 | 57660224 | 57826368 | Drip loss QTL (7815) | 1 | 10256017 | 1E+08 |
| ENSSSCG00000004328 | 1 | 57660224 | 57826368 | Drip loss QTL (8012) | 1 | 10256017 | 1E+08 |
| ENSSSCG00000004328 | 1 | 57660224 | 57826368 | Drip loss QTL (8043) | 1 | 10256017 | 1E+08 |
| ENSSSCG00000004328 | 1 | 57660224 | 57826368 | Drip loss QTL (8110) | 1 | 10256017 | 1E+08 |
| ENSSSCG00000004328 | 1 | 57660224 | 57826368 | Drip loss QTL (8154) | 1 | 10256017 | 1E+08 |
| ENSSSCG00000004328 | 1 | 57660224 | 57826368 | Drip loss QTL (7751) | 1 | 10256017 | 1E+08 |
| ENSSSCG00000004328 | 1 | 57660224 | 57826368 | Drip loss QTL (7763) | 1 | 10256017 | 1E+08 |
| ENSSSCG00000004328 | 1 | 57660224 | 57826368 | Backfat at last rib QTL (21208) | 1 | 15061284 | 2.48E+08 |
| ENSSSCG00000004328 | 1 | 57660224 | 57826368 | Shoulder subcutaneous fat thickness QTL (5217) | 1 | 29405363 | 2.48E+08 |
| ENSSSCG00000004328 | 1 | 57660224 | 57826368 | Backfat at last rib QTL (5674) | 1 | 29405363 | 1.98E+08 |
| ENSSSCG00000004328 | 1 | 57660224 | 57826368 | Percentage type IIa fibers QTL (7015) | 1 | 29405363 | 93774423 |
| ENSSSCG00000004328 | 1 | 57660224 | 57826368 | PH for Longissmus dorsi QTL (2787) | 1 | 29405363 | 79968246 |
| ENSSSCG00000004328 | 1 | 57660224 | 57826368 | Leaf fat weight QTL (3863) | 1 | 30301868 | 93595122 |
| ENSSSCG00000004328 | 1 | 57660224 | 57826368 | Body weight (20 weeks) QTL (29689) | 1 | 42135735 | 1.44E+08 |
| ENSSSCG00000004328 | 1 | 57660224 | 57826368 | Meat color a* QTL (3980) | 1 | 42135735 | 88574694 |
| ENSSSCG00000004328 | 1 | 57660224 | 57826368 | Average daily gain QTL (338) | 1 | 42135735 | 79968246 |
| ENSSSCG00000004328 | 1 | 57660224 | 57826368 | muscle protein percentage QTL (3838) | 1 | 52355892 | 88574694 |
| ENSSSCG00000004328 | 1 | 57660224 | 57826368 | Backfat at tenth rib QTL (43) | 1 | 52355892 | 88574694 |
| ENSSSCG00000004328 | 1 | 57660224 | 57826368 | Loin muscle area QTL (3866) | 1 | 56820486 | 83016363 |
| ENSSSCG00000025703 | 1 | 77197409 | 77400137 | Carcass length QTL (13374) | 1 | 75898113 | 77529752 |
| ENSSSCG00000025703 | 1 | 77197409 | 77400137 | Average daily gain QTL (5680) | 1 | 5379030 | 2.52E+08 |
| ENSSSCG00000025703 | 1 | 77197409 | 77400137 | Shoulder subcutaneous fat thickness QTL (5671) | 1 | 5379030 | 1.98E+08 |
| ENSSSCG00000025703 | 1 | 77197409 | 77400137 | Average backfat thickness QTL (5670) | 1 | 5379030 | 1.68E+08 |
| ENSSSCG00000025703 | 1 | 77197409 | 77400137 | Shoulder subcutaneous fat thickness QTL (21209) | 1 | 7172040 | 2.51E+08 |
| ENSSSCG00000025703 | 1 | 77197409 | 77400137 | Drip loss QTL (8349) | 1 | 10256017 | 1E+08 |
| ENSSSCG00000025703 | 1 | 77197409 | 77400137 | Drip loss QTL (8411) | 1 | 10256017 | 1E+08 |
| ENSSSCG00000025703 | 1 | 77197409 | 77400137 | Drip loss QTL (8562) | 1 | 10256017 | 1E+08 |
| ENSSSCG00000025703 | 1 | 77197409 | 77400137 | Drip loss QTL (7776) | 1 | 10256017 | 1E+08 |
| ENSSSCG00000025703 | 1 | 77197409 | 77400137 | Drip loss QTL (7815) | 1 | 10256017 | 1E+08 |
| ENSSSCG00000025703 | 1 | 77197409 | 77400137 | Drip loss QTL (8012) | 1 | 10256017 | 1E+08 |
| ENSSSCG00000025703 | 1 | 77197409 | 77400137 | Drip loss QTL (8043) | 1 | 10256017 | 1E+08 |
| ENSSSCG00000025703 | 1 | 77197409 | 77400137 | Drip loss QTL (8110) | 1 | 10256017 | 1E+08 |
| ENSSSCG00000025703 | 1 | 77197409 | 77400137 | Drip loss QTL (8154) | 1 | 10256017 | 1E+08 |
| ENSSSCG00000025703 | 1 | 77197409 | 77400137 | Drip loss QTL (7751) | 1 | 10256017 | 1E+08 |
| ENSSSCG00000025703 | 1 | 77197409 | 77400137 | Drip loss QTL (7763) | 1 | 10256017 | 1E+08 |
| ENSSSCG00000025703 | 1 | 77197409 | 77400137 | Backfat at last rib QTL (21208) | 1 | 15061284 | 2.48E+08 |
| ENSSSCG00000025703 | 1 | 77197409 | 77400137 | Shoulder subcutaneous fat thickness QTL (5217) | 1 | 29405363 | 2.48E+08 |
| ENSSSCG00000025703 | 1 | 77197409 | 77400137 | Backfat at last rib QTL (5674) | 1 | 29405363 | 1.98E+08 |
| ENSSSCG00000025703 | 1 | 77197409 | 77400137 | Percentage type IIa fibers QTL (7015) | 1 | 29405363 | 93774423 |
| ENSSSCG00000025703 | 1 | 77197409 | 77400137 | PH for Longissmus dorsi QTL (2787) | 1 | 29405363 | 79968246 |
| ENSSSCG00000025703 | 1 | 77197409 | 77400137 | Leaf fat weight QTL (3863) | 1 | 30301868 | 93595122 |
| ENSSSCG00000025703 | 1 | 77197409 | 77400137 | Body weight (20 weeks) QTL (29689) | 1 | 42135735 | 1.44E+08 |
| ENSSSCG00000025703 | 1 | 77197409 | 77400137 | Meat color a* QTL (3980) | 1 | 42135735 | 88574694 |
| ENSSSCG00000025703 | 1 | 77197409 | 77400137 | Average daily gain QTL (338) | 1 | 42135735 | 79968246 |
| ENSSSCG00000025703 | 1 | 77197409 | 77400137 | muscle protein percentage QTL (3838) | 1 | 52355892 | 88574694 |
| ENSSSCG00000025703 | 1 | 77197409 | 77400137 | Backfat at tenth rib QTL (43) | 1 | 52355892 | 88574694 |
| ENSSSCG00000025703 | 1 | 77197409 | 77400137 | Loin muscle area QTL (3866) | 1 | 56820486 | 83016363 |
| ENSSSCG00000025703 | 1 | 77197409 | 77400137 | Subcutanous fat area QTL (5676) | 1 | 59886534 | 1.68E+08 |
| ENSSSCG00000025703 | 1 | 77197409 | 77400137 | Average daily gain QTL (16885) | 1 | 59886534 | 88574694 |
| ENSSSCG00000025703 | 1 | 77197409 | 77400137 | Average backfat thickness QTL (42) | 1 | 59886534 | 88574694 |
| ENSSSCG00000025703 | 1 | 77197409 | 77400137 | Loin muscle area QTL (46) | 1 | 59886534 | 88574694 |
| ENSSSCG00000004636 | 1 | 1.21E+08 | 1.21E+08 | Backfat at tenth rib QTL (5672) | 1 | 79968246 | 1.26E+08 |
| ENSSSCG00000004636 | 1 | 1.21E+08 | 1.21E+08 | Carcass weight (cold) QTL (12711) | 1 | 88574694 | 1.31E+08 |
| ENSSSCG00000004636 | 1 | 1.21E+08 | 1.21E+08 | Average daily gain QTL (3673) | 1 | 93774423 | 1.21E+08 |
| ENSSSCG00000004636 | 1 | 1.21E+08 | 1.21E+08 | PH for Longissmus dorsi QTL (3693) | 1 | 93774423 | 1.21E+08 |
| ENSSSCG00000004636 | 1 | 1.21E+08 | 1.21E+08 | PH for Longissmus dorsi QTL (3694) | 1 | 93774423 | 1.21E+08 |
| ENSSSCG00000004636 | 1 | 1.21E+08 | 1.21E+08 | PH for Longissmus dorsi QTL (4110) | 1 | 93774423 | 1.21E+08 |
| ENSSSCG00000004636 | 1 | 1.21E+08 | 1.21E+08 | Meat color L* QTL (4326) | 1 | 1.05E+08 | 1.24E+08 |
| ENSSSCG00000004636 | 1 | 1.21E+08 | 1.21E+08 | Cholesterol level QTL (17711) | 1 | 1.06E+08 | 1.26E+08 |
| ENSSSCG00000004636 | 1 | 1.21E+08 | 1.21E+08 | HDL/LDL ratio QTL (17712) | 1 | 1.06E+08 | 1.26E+08 |
| ENSSSCG00000004636 | 1 | 1.21E+08 | 1.21E+08 | LDL cholesterol QTL (17713) | 1 | 1.06E+08 | 1.26E+08 |
| ENSSSCG00000004636 | 1 | 1.21E+08 | 1.21E+08 | Average daily gain QTL (5928) | 1 | 1.06E+08 | 1.26E+08 |
| ENSSSCG00000004636 | 1 | 1.21E+08 | 1.21E+08 | Backfat at last lumbar QTL (17787) | 1 | 1.08E+08 | 1.21E+08 |
| ENSSSCG00000004636 | 1 | 1.21E+08 | 1.21E+08 | Shear force QTL (17788) | 1 | 1.08E+08 | 1.21E+08 |
| ENSSSCG00000004636 | 1 | 1.21E+08 | 1.21E+08 | Cross-sectional area of type IIa muscle fibers QTL (10236) | 1 | 1.16E+08 | 1.23E+08 |
| ENSSSCG00000004636 | 1 | 1.21E+08 | 1.21E+08 | Average daily gain QTL (5680) | 1 | 5379030 | 2.52E+08 |
| ENSSSCG00000004636 | 1 | 1.21E+08 | 1.21E+08 | Shoulder subcutaneous fat thickness QTL (5671) | 1 | 5379030 | 1.98E+08 |
| ENSSSCG00000004636 | 1 | 1.21E+08 | 1.21E+08 | Average backfat thickness QTL (5670) | 1 | 5379030 | 1.68E+08 |
| ENSSSCG00000004636 | 1 | 1.21E+08 | 1.21E+08 | Shoulder subcutaneous fat thickness QTL (21209) | 1 | 7172040 | 2.51E+08 |
| ENSSSCG00000004636 | 1 | 1.21E+08 | 1.21E+08 | Backfat at last rib QTL (21208) | 1 | 15061284 | 2.48E+08 |
| ENSSSCG00000004636 | 1 | 1.21E+08 | 1.21E+08 | Shoulder subcutaneous fat thickness QTL (5217) | 1 | 29405363 | 2.48E+08 |
| ENSSSCG00000004636 | 1 | 1.21E+08 | 1.21E+08 | Backfat at last rib QTL (5674) | 1 | 29405363 | 1.98E+08 |
| ENSSSCG00000004636 | 1 | 1.21E+08 | 1.21E+08 | Body weight (20 weeks) QTL (29689) | 1 | 42135735 | 1.44E+08 |
| ENSSSCG00000004636 | 1 | 1.21E+08 | 1.21E+08 | Subcutanous fat area QTL (5676) | 1 | 59886534 | 1.68E+08 |
| ENSSSCG00000004636 | 1 | 1.21E+08 | 1.21E+08 | Skin thickness QTL (31809) | 1 | 77637333 | 2.53E+08 |
| ENSSSCG00000004636 | 1 | 1.21E+08 | 1.21E+08 | Muscle fat content QTL (79) | 1 | 77995935 | 1.49E+08 |
| ENSSSCG00000004636 | 1 | 1.21E+08 | 1.21E+08 | Linoleic acid content QTL (156913) | 1 | 79071741 | 2.52E+08 |
| ENSSSCG00000004636 | 1 | 1.21E+08 | 1.21E+08 | Leaf fat weight QTL (21207) | 1 | 79609644 | 2.42E+08 |
| ENSSSCG00000004636 | 1 | 1.21E+08 | 1.21E+08 | Average daily gain QTL (16886) | 1 | 88574694 | 1.42E+08 |
| ENSSSCG00000004636 | 1 | 1.21E+08 | 1.21E+08 | Drip loss QTL (8382) | 1 | 91802112 | 2.68E+08 |
| ENSSSCG00000004636 | 1 | 1.21E+08 | 1.21E+08 | Drip loss QTL (8480) | 1 | 91802112 | 2.68E+08 |
| ENSSSCG00000004636 | 1 | 1.21E+08 | 1.21E+08 | Drip loss QTL (8564) | 1 | 91802112 | 2.68E+08 |
| ENSSSCG00000004636 | 1 | 1.21E+08 | 1.21E+08 | Drip loss QTL (8602) | 1 | 91802112 | 2.68E+08 |
| ENSSSCG00000004636 | 1 | 1.21E+08 | 1.21E+08 | Drip loss QTL (7936) | 1 | 91802112 | 2.68E+08 |
| ENSSSCG00000004636 | 1 | 1.21E+08 | 1.21E+08 | Drip loss QTL (8063) | 1 | 91802112 | 2.68E+08 |
| ENSSSCG00000004636 | 1 | 1.21E+08 | 1.21E+08 | Drip loss QTL (8131) | 1 | 91802112 | 2.68E+08 |
| ENSSSCG00000004636 | 1 | 1.21E+08 | 1.21E+08 | Drip loss QTL (8300) | 1 | 91802112 | 2.68E+08 |
| ENSSSCG00000004636 | 1 | 1.21E+08 | 1.21E+08 | Drip loss QTL (8323) | 1 | 91802112 | 2.68E+08 |
| ENSSSCG00000004636 | 1 | 1.21E+08 | 1.21E+08 | Average daily gain QTL (329) | 1 | 93774423 | 1.41E+08 |
| ENSSSCG00000004636 | 1 | 1.21E+08 | 1.21E+08 | Backfat at mid-back QTL (5206) | 1 | 93953724 | 2.51E+08 |
| ENSSSCG00000004636 | 1 | 1.21E+08 | 1.21E+08 | Creatinine level QTL (6366) | 1 | 94850229 | 1.45E+08 |
| ENSSSCG00000004636 | 1 | 1.21E+08 | 1.21E+08 | Potassium level QTL (6380) | 1 | 94850229 | 1.45E+08 |
| ENSSSCG00000004636 | 1 | 1.21E+08 | 1.21E+08 | Backfat at rump QTL (651) | 1 | 1E+08 | 1.68E+08 |
| ENSSSCG00000004636 | 1 | 1.21E+08 | 1.21E+08 | Average backfat thickness QTL (3793) | 1 | 1.06E+08 | 2.14E+08 |
| ENSSSCG00000004636 | 1 | 1.21E+08 | 1.21E+08 | Backfat at last rib QTL (3794) | 1 | 1.06E+08 | 2.14E+08 |
| ENSSSCG00000004636 | 1 | 1.21E+08 | 1.21E+08 | Meat color OPTO QTL (5667) | 1 | 1.06E+08 | 1.98E+08 |
| ENSSSCG00000004636 | 1 | 1.21E+08 | 1.21E+08 | Backfat at tenth rib QTL (3795) | 1 | 1.06E+08 | 1.85E+08 |
| ENSSSCG00000004636 | 1 | 1.21E+08 | 1.21E+08 | Loin muscle area QTL (3796) | 1 | 1.06E+08 | 1.85E+08 |
| ENSSSCG00000004636 | 1 | 1.21E+08 | 1.21E+08 | PH for Longissmus dorsi QTL (5664) | 1 | 1.06E+08 | 1.44E+08 |
| ENSSSCG00000004636 | 1 | 1.21E+08 | 1.21E+08 | PH for Longissmus dorsi QTL (5666) | 1 | 1.06E+08 | 1.44E+08 |
| ENSSSCG00000004636 | 1 | 1.21E+08 | 1.21E+08 | Polyunsaturated fatty acid content QTL (156916) | 1 | 1.1E+08 | 2.52E+08 |
| ENSSSCG00000004725 | 1 | 1.29E+08 | 1.29E+08 | Carcass weight (cold) QTL (12711) | 1 | 88574694 | 1.31E+08 |
| ENSSSCG00000004725 | 1 | 1.29E+08 | 1.29E+08 | Average daily gain QTL (5680) | 1 | 5379030 | 2.52E+08 |
| ENSSSCG00000004725 | 1 | 1.29E+08 | 1.29E+08 | Shoulder subcutaneous fat thickness QTL (5671) | 1 | 5379030 | 1.98E+08 |
| ENSSSCG00000004725 | 1 | 1.29E+08 | 1.29E+08 | Average backfat thickness QTL (5670) | 1 | 5379030 | 1.68E+08 |
| ENSSSCG00000004725 | 1 | 1.29E+08 | 1.29E+08 | Shoulder subcutaneous fat thickness QTL (21209) | 1 | 7172040 | 2.51E+08 |
| ENSSSCG00000004725 | 1 | 1.29E+08 | 1.29E+08 | Backfat at last rib QTL (21208) | 1 | 15061284 | 2.48E+08 |
| ENSSSCG00000004725 | 1 | 1.29E+08 | 1.29E+08 | Shoulder subcutaneous fat thickness QTL (5217) | 1 | 29405363 | 2.48E+08 |
| ENSSSCG00000004725 | 1 | 1.29E+08 | 1.29E+08 | Backfat at last rib QTL (5674) | 1 | 29405363 | 1.98E+08 |
| ENSSSCG00000004725 | 1 | 1.29E+08 | 1.29E+08 | Body weight (20 weeks) QTL (29689) | 1 | 42135735 | 1.44E+08 |
| ENSSSCG00000004725 | 1 | 1.29E+08 | 1.29E+08 | Subcutanous fat area QTL (5676) | 1 | 59886534 | 1.68E+08 |
| ENSSSCG00000004725 | 1 | 1.29E+08 | 1.29E+08 | Skin thickness QTL (31809) | 1 | 77637333 | 2.53E+08 |
| ENSSSCG00000004725 | 1 | 1.29E+08 | 1.29E+08 | Muscle fat content QTL (79) | 1 | 77995935 | 1.49E+08 |
| ENSSSCG00000004725 | 1 | 1.29E+08 | 1.29E+08 | Linoleic acid content QTL (156913) | 1 | 79071741 | 2.52E+08 |
| ENSSSCG00000004725 | 1 | 1.29E+08 | 1.29E+08 | Leaf fat weight QTL (21207) | 1 | 79609644 | 2.42E+08 |
| ENSSSCG00000004725 | 1 | 1.29E+08 | 1.29E+08 | Average daily gain QTL (16886) | 1 | 88574694 | 1.42E+08 |
| ENSSSCG00000004725 | 1 | 1.29E+08 | 1.29E+08 | Drip loss QTL (8382) | 1 | 91802112 | 2.68E+08 |
| ENSSSCG00000004725 | 1 | 1.29E+08 | 1.29E+08 | Drip loss QTL (8480) | 1 | 91802112 | 2.68E+08 |
| ENSSSCG00000004725 | 1 | 1.29E+08 | 1.29E+08 | Drip loss QTL (8564) | 1 | 91802112 | 2.68E+08 |
| ENSSSCG00000004725 | 1 | 1.29E+08 | 1.29E+08 | Drip loss QTL (8602) | 1 | 91802112 | 2.68E+08 |
| ENSSSCG00000004725 | 1 | 1.29E+08 | 1.29E+08 | Drip loss QTL (7936) | 1 | 91802112 | 2.68E+08 |
| ENSSSCG00000004725 | 1 | 1.29E+08 | 1.29E+08 | Drip loss QTL (8063) | 1 | 91802112 | 2.68E+08 |
| ENSSSCG00000004725 | 1 | 1.29E+08 | 1.29E+08 | Drip loss QTL (8131) | 1 | 91802112 | 2.68E+08 |
| ENSSSCG00000004725 | 1 | 1.29E+08 | 1.29E+08 | Drip loss QTL (8300) | 1 | 91802112 | 2.68E+08 |
| ENSSSCG00000004725 | 1 | 1.29E+08 | 1.29E+08 | Drip loss QTL (8323) | 1 | 91802112 | 2.68E+08 |
| ENSSSCG00000004725 | 1 | 1.29E+08 | 1.29E+08 | Average daily gain QTL (329) | 1 | 93774423 | 1.41E+08 |
| ENSSSCG00000004725 | 1 | 1.29E+08 | 1.29E+08 | Backfat at mid-back QTL (5206) | 1 | 93953724 | 2.51E+08 |
| ENSSSCG00000004725 | 1 | 1.29E+08 | 1.29E+08 | Creatinine level QTL (6366) | 1 | 94850229 | 1.45E+08 |
| ENSSSCG00000004725 | 1 | 1.29E+08 | 1.29E+08 | Potassium level QTL (6380) | 1 | 94850229 | 1.45E+08 |
| ENSSSCG00000004725 | 1 | 1.29E+08 | 1.29E+08 | Backfat at rump QTL (651) | 1 | 1E+08 | 1.68E+08 |
| ENSSSCG00000004725 | 1 | 1.29E+08 | 1.29E+08 | Average backfat thickness QTL (3793) | 1 | 1.06E+08 | 2.14E+08 |
| ENSSSCG00000004725 | 1 | 1.29E+08 | 1.29E+08 | Backfat at last rib QTL (3794) | 1 | 1.06E+08 | 2.14E+08 |
| ENSSSCG00000004725 | 1 | 1.29E+08 | 1.29E+08 | Meat color OPTO QTL (5667) | 1 | 1.06E+08 | 1.98E+08 |
| ENSSSCG00000004725 | 1 | 1.29E+08 | 1.29E+08 | Backfat at tenth rib QTL (3795) | 1 | 1.06E+08 | 1.85E+08 |
| ENSSSCG00000004725 | 1 | 1.29E+08 | 1.29E+08 | Loin muscle area QTL (3796) | 1 | 1.06E+08 | 1.85E+08 |
| ENSSSCG00000004725 | 1 | 1.29E+08 | 1.29E+08 | PH for Longissmus dorsi QTL (5664) | 1 | 1.06E+08 | 1.44E+08 |
| ENSSSCG00000004725 | 1 | 1.29E+08 | 1.29E+08 | PH for Longissmus dorsi QTL (5666) | 1 | 1.06E+08 | 1.44E+08 |
| ENSSSCG00000004725 | 1 | 1.29E+08 | 1.29E+08 | Polyunsaturated fatty acid content QTL (156916) | 1 | 1.1E+08 | 2.52E+08 |
| ENSSSCG00000004725 | 1 | 1.29E+08 | 1.29E+08 | Backfat at last lumbar QTL (45) | 1 | 1.21E+08 | 1.81E+08 |
| ENSSSCG00000004725 | 1 | 1.29E+08 | 1.29E+08 | Marbling QTL (4020) | 1 | 1.27E+08 | 1.85E+08 |
| ENSSSCG00000004725 | 1 | 1.29E+08 | 1.29E+08 | Average glycolytic potential QTL (17789) | 1 | 1.29E+08 | 1.48E+08 |
| ENSSSCG00000023533 | 1 | 1.48E+08 | 1.48E+08 | Average backfat thickness QTL (797) | 1 | 1.34E+08 | 1.52E+08 |
| ENSSSCG00000023533 | 1 | 1.48E+08 | 1.48E+08 | Average daily gain QTL (16887) | 1 | 1.42E+08 | 1.69E+08 |
| ENSSSCG00000023533 | 1 | 1.48E+08 | 1.48E+08 | backfat above muscle dorsi QTL (5673) | 1 | 1.44E+08 | 1.98E+08 |
| ENSSSCG00000023533 | 1 | 1.48E+08 | 1.48E+08 | Palmitoleic acid content QTL (156911) | 1 | 1.44E+08 | 1.98E+08 |
| ENSSSCG00000023533 | 1 | 1.48E+08 | 1.48E+08 | Linolenic acid content QTL (686) | 1 | 1.44E+08 | 1.85E+08 |
| ENSSSCG00000023533 | 1 | 1.48E+08 | 1.48E+08 | Backfat at last rib QTL (16832) | 1 | 1.45E+08 | 1.78E+08 |
| ENSSSCG00000023533 | 1 | 1.48E+08 | 1.48E+08 | Backfat at last rib QTL (16880) | 1 | 1.45E+08 | 1.78E+08 |
| ENSSSCG00000023533 | 1 | 1.48E+08 | 1.48E+08 | Backfat at last rib QTL (16882) | 1 | 1.45E+08 | 1.78E+08 |
| ENSSSCG00000023533 | 1 | 1.48E+08 | 1.48E+08 | Subcutanous fat area QTL (12737) | 1 | 1.45E+08 | 1.69E+08 |
| ENSSSCG00000023533 | 1 | 1.48E+08 | 1.48E+08 | Aspartate aminotransferase activity QTL (7480) | 1 | 1.45E+08 | 1.69E+08 |
| ENSSSCG00000023533 | 1 | 1.48E+08 | 1.48E+08 | Average daily gain QTL (16845) | 1 | 1.45E+08 | 1.69E+08 |
| ENSSSCG00000023533 | 1 | 1.48E+08 | 1.48E+08 | Backfat at last rib QTL (850) | 1 | 1.45E+08 | 1.69E+08 |
| ENSSSCG00000023533 | 1 | 1.48E+08 | 1.48E+08 | Average backfat thickness QTL (864) | 1 | 1.45E+08 | 1.69E+08 |
| ENSSSCG00000023533 | 1 | 1.48E+08 | 1.48E+08 | Backfat thickness between 3rd and 4th rib QTL (867) | 1 | 1.45E+08 | 1.69E+08 |
| ENSSSCG00000023533 | 1 | 1.48E+08 | 1.48E+08 | Average daily gain QTL (5680) | 1 | 5379030 | 2.52E+08 |
| ENSSSCG00000023533 | 1 | 1.48E+08 | 1.48E+08 | Shoulder subcutaneous fat thickness QTL (5671) | 1 | 5379030 | 1.98E+08 |
| ENSSSCG00000023533 | 1 | 1.48E+08 | 1.48E+08 | Average backfat thickness QTL (5670) | 1 | 5379030 | 1.68E+08 |
| ENSSSCG00000023533 | 1 | 1.48E+08 | 1.48E+08 | Shoulder subcutaneous fat thickness QTL (21209) | 1 | 7172040 | 2.51E+08 |
| ENSSSCG00000023533 | 1 | 1.48E+08 | 1.48E+08 | Backfat at last rib QTL (21208) | 1 | 15061284 | 2.48E+08 |
| ENSSSCG00000023533 | 1 | 1.48E+08 | 1.48E+08 | Shoulder subcutaneous fat thickness QTL (5217) | 1 | 29405363 | 2.48E+08 |
| ENSSSCG00000023533 | 1 | 1.48E+08 | 1.48E+08 | Backfat at last rib QTL (5674) | 1 | 29405363 | 1.98E+08 |
| ENSSSCG00000023533 | 1 | 1.48E+08 | 1.48E+08 | Subcutanous fat area QTL (5676) | 1 | 59886534 | 1.68E+08 |
| ENSSSCG00000023533 | 1 | 1.48E+08 | 1.48E+08 | Skin thickness QTL (31809) | 1 | 77637333 | 2.53E+08 |
| ENSSSCG00000023533 | 1 | 1.48E+08 | 1.48E+08 | Muscle fat content QTL (79) | 1 | 77995935 | 1.49E+08 |
| ENSSSCG00000023533 | 1 | 1.48E+08 | 1.48E+08 | Linoleic acid content QTL (156913) | 1 | 79071741 | 2.52E+08 |
| ENSSSCG00000023533 | 1 | 1.48E+08 | 1.48E+08 | Leaf fat weight QTL (21207) | 1 | 79609644 | 2.42E+08 |
| ENSSSCG00000023533 | 1 | 1.48E+08 | 1.48E+08 | Drip loss QTL (8382) | 1 | 91802112 | 2.68E+08 |
| ENSSSCG00000023533 | 1 | 1.48E+08 | 1.48E+08 | Drip loss QTL (8480) | 1 | 91802112 | 2.68E+08 |
| ENSSSCG00000023533 | 1 | 1.48E+08 | 1.48E+08 | Drip loss QTL (8564) | 1 | 91802112 | 2.68E+08 |
| ENSSSCG00000023533 | 1 | 1.48E+08 | 1.48E+08 | Drip loss QTL (8602) | 1 | 91802112 | 2.68E+08 |
| ENSSSCG00000023533 | 1 | 1.48E+08 | 1.48E+08 | Drip loss QTL (7936) | 1 | 91802112 | 2.68E+08 |
| ENSSSCG00000023533 | 1 | 1.48E+08 | 1.48E+08 | Drip loss QTL (8063) | 1 | 91802112 | 2.68E+08 |
| ENSSSCG00000023533 | 1 | 1.48E+08 | 1.48E+08 | Drip loss QTL (8131) | 1 | 91802112 | 2.68E+08 |
| ENSSSCG00000023533 | 1 | 1.48E+08 | 1.48E+08 | Drip loss QTL (8300) | 1 | 91802112 | 2.68E+08 |
| ENSSSCG00000023533 | 1 | 1.48E+08 | 1.48E+08 | Drip loss QTL (8323) | 1 | 91802112 | 2.68E+08 |
| ENSSSCG00000023533 | 1 | 1.48E+08 | 1.48E+08 | Backfat at mid-back QTL (5206) | 1 | 93953724 | 2.51E+08 |
| ENSSSCG00000023533 | 1 | 1.48E+08 | 1.48E+08 | Backfat at rump QTL (651) | 1 | 1E+08 | 1.68E+08 |
| ENSSSCG00000023533 | 1 | 1.48E+08 | 1.48E+08 | Average backfat thickness QTL (3793) | 1 | 1.06E+08 | 2.14E+08 |
| ENSSSCG00000023533 | 1 | 1.48E+08 | 1.48E+08 | Backfat at last rib QTL (3794) | 1 | 1.06E+08 | 2.14E+08 |
| ENSSSCG00000023533 | 1 | 1.48E+08 | 1.48E+08 | Meat color OPTO QTL (5667) | 1 | 1.06E+08 | 1.98E+08 |
| ENSSSCG00000023533 | 1 | 1.48E+08 | 1.48E+08 | Backfat at tenth rib QTL (3795) | 1 | 1.06E+08 | 1.85E+08 |
| ENSSSCG00000023533 | 1 | 1.48E+08 | 1.48E+08 | Loin muscle area QTL (3796) | 1 | 1.06E+08 | 1.85E+08 |
| ENSSSCG00000023533 | 1 | 1.48E+08 | 1.48E+08 | Polyunsaturated fatty acid content QTL (156916) | 1 | 1.1E+08 | 2.52E+08 |
| ENSSSCG00000023533 | 1 | 1.48E+08 | 1.48E+08 | Backfat at last lumbar QTL (45) | 1 | 1.21E+08 | 1.81E+08 |
| ENSSSCG00000023533 | 1 | 1.48E+08 | 1.48E+08 | Marbling QTL (4020) | 1 | 1.27E+08 | 1.85E+08 |
| ENSSSCG00000023533 | 1 | 1.48E+08 | 1.48E+08 | Average glycolytic potential QTL (17789) | 1 | 1.29E+08 | 1.48E+08 |
| ENSSSCG00000023533 | 1 | 1.48E+08 | 1.48E+08 | Average daily gain QTL (3914) | 1 | 1.31E+08 | 2.52E+08 |
| ENSSSCG00000023533 | 1 | 1.48E+08 | 1.48E+08 | Average daily gain QTL (3917) | 1 | 1.31E+08 | 2.52E+08 |
| ENSSSCG00000023533 | 1 | 1.48E+08 | 1.48E+08 | Backfat at last rib QTL (44) | 1 | 1.31E+08 | 2.02E+08 |
| ENSSSCG00000023533 | 1 | 1.48E+08 | 1.48E+08 | Loin muscle area QTL (3640) | 1 | 1.31E+08 | 1.85E+08 |
| ENSSSCG00000023533 | 1 | 1.48E+08 | 1.48E+08 | Loin muscle area QTL (2742) | 1 | 1.31E+08 | 1.85E+08 |
| ENSSSCG00000023533 | 1 | 1.48E+08 | 1.48E+08 | Average backfat thickness QTL (17996) | 1 | 1.31E+08 | 1.69E+08 |
| ENSSSCG00000023533 | 1 | 1.48E+08 | 1.48E+08 | Muscle fat content QTL (4017) | 1 | 1.33E+08 | 1.56E+08 |
| ENSSSCG00000023533 | 1 | 1.48E+08 | 1.48E+08 | Drip loss QTL (7784) | 1 | 1.41E+08 | 2.02E+08 |
| ENSSSCG00000023533 | 1 | 1.48E+08 | 1.48E+08 | Drip loss QTL (7861) | 1 | 1.41E+08 | 2.02E+08 |
| ENSSSCG00000023533 | 1 | 1.48E+08 | 1.48E+08 | Drip loss QTL (8102) | 1 | 1.41E+08 | 2.02E+08 |
| ENSSSCG00000023533 | 1 | 1.48E+08 | 1.48E+08 | Drip loss QTL (8156) | 1 | 1.41E+08 | 2.02E+08 |
| ENSSSCG00000023533 | 1 | 1.48E+08 | 1.48E+08 | Drip loss QTL (8348) | 1 | 1.41E+08 | 2.02E+08 |
| ENSSSCG00000023533 | 1 | 1.48E+08 | 1.48E+08 | Average daily gain QTL (5681) | 1 | 1.44E+08 | 2.52E+08 |
| ENSSSCG00000023533 | 1 | 1.48E+08 | 1.48E+08 | Stearic acid content QTL (156864) | 1 | 1.44E+08 | 2.52E+08 |
| ENSSSCG00000004919 | 1 | 1.62E+08 | 1.63E+08 | Intramuscular fat content QTL (169088) | 1 | 1.62E+08 | 1.62E+08 |
| ENSSSCG00000004919 | 1 | 1.62E+08 | 1.63E+08 | Average daily gain QTL (171467) | 1 | 1.62E+08 | 1.62E+08 |
| ENSSSCG00000004919 | 1 | 1.62E+08 | 1.63E+08 | Average daily gain QTL (171468) | 1 | 1.63E+08 | 1.63E+08 |
| ENSSSCG00000004919 | 1 | 1.62E+08 | 1.63E+08 | Drip loss QTL (7738) | 1 | 1.63E+08 | 1.63E+08 |
| ENSSSCG00000004919 | 1 | 1.62E+08 | 1.63E+08 | Drip loss QTL (8375) | 1 | 1.63E+08 | 1.63E+08 |
| ENSSSCG00000004919 | 1 | 1.62E+08 | 1.63E+08 | Drip loss QTL (8386) | 1 | 1.63E+08 | 1.63E+08 |
| ENSSSCG00000004919 | 1 | 1.62E+08 | 1.63E+08 | Drip loss QTL (8395) | 1 | 1.63E+08 | 1.63E+08 |
| ENSSSCG00000004919 | 1 | 1.62E+08 | 1.63E+08 | Drip loss QTL (8471) | 1 | 1.63E+08 | 1.63E+08 |
| ENSSSCG00000004919 | 1 | 1.62E+08 | 1.63E+08 | Drip loss QTL (8485) | 1 | 1.63E+08 | 1.63E+08 |
| ENSSSCG00000004919 | 1 | 1.62E+08 | 1.63E+08 | Drip loss QTL (8489) | 1 | 1.63E+08 | 1.63E+08 |
| ENSSSCG00000004919 | 1 | 1.62E+08 | 1.63E+08 | Drip loss QTL (8525) | 1 | 1.63E+08 | 1.63E+08 |
| ENSSSCG00000004919 | 1 | 1.62E+08 | 1.63E+08 | Drip loss QTL (8532) | 1 | 1.63E+08 | 1.63E+08 |
| ENSSSCG00000004919 | 1 | 1.62E+08 | 1.63E+08 | Drip loss QTL (8550) | 1 | 1.63E+08 | 1.63E+08 |
| ENSSSCG00000004919 | 1 | 1.62E+08 | 1.63E+08 | Drip loss QTL (8552) | 1 | 1.63E+08 | 1.63E+08 |
| ENSSSCG00000004919 | 1 | 1.62E+08 | 1.63E+08 | Drip loss QTL (8574) | 1 | 1.63E+08 | 1.63E+08 |
| ENSSSCG00000004919 | 1 | 1.62E+08 | 1.63E+08 | Drip loss QTL (8589) | 1 | 1.63E+08 | 1.63E+08 |
| ENSSSCG00000004919 | 1 | 1.62E+08 | 1.63E+08 | Drip loss QTL (8594) | 1 | 1.63E+08 | 1.63E+08 |
| ENSSSCG00000004919 | 1 | 1.62E+08 | 1.63E+08 | Drip loss QTL (8603) | 1 | 1.63E+08 | 1.63E+08 |
| ENSSSCG00000004919 | 1 | 1.62E+08 | 1.63E+08 | Drip loss QTL (8193) | 1 | 1.63E+08 | 1.63E+08 |
| ENSSSCG00000004919 | 1 | 1.62E+08 | 1.63E+08 | Drip loss QTL (8237) | 1 | 1.63E+08 | 1.63E+08 |
| ENSSSCG00000004919 | 1 | 1.62E+08 | 1.63E+08 | Drip loss QTL (8255) | 1 | 1.63E+08 | 1.63E+08 |
| ENSSSCG00000004919 | 1 | 1.62E+08 | 1.63E+08 | Drip loss QTL (8261) | 1 | 1.63E+08 | 1.63E+08 |
| ENSSSCG00000004919 | 1 | 1.62E+08 | 1.63E+08 | Drip loss QTL (8274) | 1 | 1.63E+08 | 1.63E+08 |
| ENSSSCG00000004919 | 1 | 1.62E+08 | 1.63E+08 | Drip loss QTL (8326) | 1 | 1.63E+08 | 1.63E+08 |
| ENSSSCG00000004919 | 1 | 1.62E+08 | 1.63E+08 | Drip loss QTL (8341) | 1 | 1.63E+08 | 1.63E+08 |
| ENSSSCG00000004919 | 1 | 1.62E+08 | 1.63E+08 | Drip loss QTL (7775) | 1 | 1.63E+08 | 1.63E+08 |
| ENSSSCG00000004919 | 1 | 1.62E+08 | 1.63E+08 | Drip loss QTL (7778) | 1 | 1.63E+08 | 1.63E+08 |
| ENSSSCG00000004919 | 1 | 1.62E+08 | 1.63E+08 | Drip loss QTL (7856) | 1 | 1.63E+08 | 1.63E+08 |
| ENSSSCG00000004919 | 1 | 1.62E+08 | 1.63E+08 | Drip loss QTL (7865) | 1 | 1.63E+08 | 1.63E+08 |
| ENSSSCG00000004919 | 1 | 1.62E+08 | 1.63E+08 | Drip loss QTL (7878) | 1 | 1.63E+08 | 1.63E+08 |
| ENSSSCG00000004919 | 1 | 1.62E+08 | 1.63E+08 | Drip loss QTL (7879) | 1 | 1.63E+08 | 1.63E+08 |
| ENSSSCG00000004919 | 1 | 1.62E+08 | 1.63E+08 | Drip loss QTL (7911) | 1 | 1.63E+08 | 1.63E+08 |
| ENSSSCG00000004919 | 1 | 1.62E+08 | 1.63E+08 | Drip loss QTL (7952) | 1 | 1.63E+08 | 1.63E+08 |
| ENSSSCG00000004919 | 1 | 1.62E+08 | 1.63E+08 | Drip loss QTL (7996) | 1 | 1.63E+08 | 1.63E+08 |
| ENSSSCG00000004919 | 1 | 1.62E+08 | 1.63E+08 | Drip loss QTL (8026) | 1 | 1.63E+08 | 1.63E+08 |
| ENSSSCG00000004919 | 1 | 1.62E+08 | 1.63E+08 | Average daily gain QTL (16887) | 1 | 1.42E+08 | 1.69E+08 |
| ENSSSCG00000004919 | 1 | 1.62E+08 | 1.63E+08 | backfat above muscle dorsi QTL (5673) | 1 | 1.44E+08 | 1.98E+08 |
| ENSSSCG00000004919 | 1 | 1.62E+08 | 1.63E+08 | Palmitoleic acid content QTL (156911) | 1 | 1.44E+08 | 1.98E+08 |
| ENSSSCG00000004919 | 1 | 1.62E+08 | 1.63E+08 | Linolenic acid content QTL (686) | 1 | 1.44E+08 | 1.85E+08 |
| ENSSSCG00000004919 | 1 | 1.62E+08 | 1.63E+08 | Backfat at last rib QTL (16832) | 1 | 1.45E+08 | 1.78E+08 |
| ENSSSCG00000004919 | 1 | 1.62E+08 | 1.63E+08 | Backfat at last rib QTL (16880) | 1 | 1.45E+08 | 1.78E+08 |
| ENSSSCG00000004919 | 1 | 1.62E+08 | 1.63E+08 | Backfat at last rib QTL (16882) | 1 | 1.45E+08 | 1.78E+08 |
| ENSSSCG00000004919 | 1 | 1.62E+08 | 1.63E+08 | Subcutanous fat area QTL (12737) | 1 | 1.45E+08 | 1.69E+08 |
| ENSSSCG00000004919 | 1 | 1.62E+08 | 1.63E+08 | Aspartate aminotransferase activity QTL (7480) | 1 | 1.45E+08 | 1.69E+08 |
| ENSSSCG00000004919 | 1 | 1.62E+08 | 1.63E+08 | Average daily gain QTL (16845) | 1 | 1.45E+08 | 1.69E+08 |
| ENSSSCG00000004919 | 1 | 1.62E+08 | 1.63E+08 | Backfat at last rib QTL (850) | 1 | 1.45E+08 | 1.69E+08 |
| ENSSSCG00000004919 | 1 | 1.62E+08 | 1.63E+08 | Average backfat thickness QTL (864) | 1 | 1.45E+08 | 1.69E+08 |
| ENSSSCG00000004919 | 1 | 1.62E+08 | 1.63E+08 | Backfat thickness between 3rd and 4th rib QTL (867) | 1 | 1.45E+08 | 1.69E+08 |
| ENSSSCG00000004919 | 1 | 1.62E+08 | 1.63E+08 | Average backfat thickness QTL (4016) | 1 | 1.48E+08 | 1.64E+08 |
| ENSSSCG00000004919 | 1 | 1.62E+08 | 1.63E+08 | Meat color a* QTL (12663) | 1 | 1.52E+08 | 1.99E+08 |
| ENSSSCG00000004919 | 1 | 1.62E+08 | 1.63E+08 | Percentage type I fibers QTL (4021) | 1 | 1.53E+08 | 1.74E+08 |
| ENSSSCG00000004919 | 1 | 1.62E+08 | 1.63E+08 | Average backfat thickness QTL (2844) | 1 | 1.55E+08 | 1.81E+08 |
| ENSSSCG00000004919 | 1 | 1.62E+08 | 1.63E+08 | Average daily gain QTL (5680) | 1 | 5379030 | 2.52E+08 |
| ENSSSCG00000004919 | 1 | 1.62E+08 | 1.63E+08 | Shoulder subcutaneous fat thickness QTL (5671) | 1 | 5379030 | 1.98E+08 |
| ENSSSCG00000004919 | 1 | 1.62E+08 | 1.63E+08 | Average backfat thickness QTL (5670) | 1 | 5379030 | 1.68E+08 |
| ENSSSCG00000004919 | 1 | 1.62E+08 | 1.63E+08 | Shoulder subcutaneous fat thickness QTL (21209) | 1 | 7172040 | 2.51E+08 |
| ENSSSCG00000004919 | 1 | 1.62E+08 | 1.63E+08 | Backfat at last rib QTL (21208) | 1 | 15061284 | 2.48E+08 |
| ENSSSCG00000004919 | 1 | 1.62E+08 | 1.63E+08 | Shoulder subcutaneous fat thickness QTL (5217) | 1 | 29405363 | 2.48E+08 |
| ENSSSCG00000004919 | 1 | 1.62E+08 | 1.63E+08 | Backfat at last rib QTL (5674) | 1 | 29405363 | 1.98E+08 |
| ENSSSCG00000004919 | 1 | 1.62E+08 | 1.63E+08 | Subcutanous fat area QTL (5676) | 1 | 59886534 | 1.68E+08 |
| ENSSSCG00000004919 | 1 | 1.62E+08 | 1.63E+08 | Skin thickness QTL (31809) | 1 | 77637333 | 2.53E+08 |
| ENSSSCG00000004919 | 1 | 1.62E+08 | 1.63E+08 | Linoleic acid content QTL (156913) | 1 | 79071741 | 2.52E+08 |
| ENSSSCG00000004919 | 1 | 1.62E+08 | 1.63E+08 | Leaf fat weight QTL (21207) | 1 | 79609644 | 2.42E+08 |
| ENSSSCG00000004919 | 1 | 1.62E+08 | 1.63E+08 | Drip loss QTL (8382) | 1 | 91802112 | 2.68E+08 |
| ENSSSCG00000004919 | 1 | 1.62E+08 | 1.63E+08 | Drip loss QTL (8480) | 1 | 91802112 | 2.68E+08 |
| ENSSSCG00000004919 | 1 | 1.62E+08 | 1.63E+08 | Drip loss QTL (8564) | 1 | 91802112 | 2.68E+08 |
| ENSSSCG00000004919 | 1 | 1.62E+08 | 1.63E+08 | Drip loss QTL (8602) | 1 | 91802112 | 2.68E+08 |
| ENSSSCG00000004919 | 1 | 1.62E+08 | 1.63E+08 | Drip loss QTL (7936) | 1 | 91802112 | 2.68E+08 |
| ENSSSCG00000004919 | 1 | 1.62E+08 | 1.63E+08 | Drip loss QTL (8063) | 1 | 91802112 | 2.68E+08 |
| ENSSSCG00000004919 | 1 | 1.62E+08 | 1.63E+08 | Drip loss QTL (8131) | 1 | 91802112 | 2.68E+08 |
| ENSSSCG00000004919 | 1 | 1.62E+08 | 1.63E+08 | Drip loss QTL (8300) | 1 | 91802112 | 2.68E+08 |
| ENSSSCG00000004919 | 1 | 1.62E+08 | 1.63E+08 | Drip loss QTL (8323) | 1 | 91802112 | 2.68E+08 |
| ENSSSCG00000004919 | 1 | 1.62E+08 | 1.63E+08 | Backfat at mid-back QTL (5206) | 1 | 93953724 | 2.51E+08 |
| ENSSSCG00000004919 | 1 | 1.62E+08 | 1.63E+08 | Backfat at rump QTL (651) | 1 | 1E+08 | 1.68E+08 |
| ENSSSCG00000004919 | 1 | 1.62E+08 | 1.63E+08 | Average backfat thickness QTL (3793) | 1 | 1.06E+08 | 2.14E+08 |
| ENSSSCG00000004919 | 1 | 1.62E+08 | 1.63E+08 | Backfat at last rib QTL (3794) | 1 | 1.06E+08 | 2.14E+08 |
| ENSSSCG00000004919 | 1 | 1.62E+08 | 1.63E+08 | Meat color OPTO QTL (5667) | 1 | 1.06E+08 | 1.98E+08 |
| ENSSSCG00000004919 | 1 | 1.62E+08 | 1.63E+08 | Backfat at tenth rib QTL (3795) | 1 | 1.06E+08 | 1.85E+08 |
| ENSSSCG00000004919 | 1 | 1.62E+08 | 1.63E+08 | Loin muscle area QTL (3796) | 1 | 1.06E+08 | 1.85E+08 |
| ENSSSCG00000004919 | 1 | 1.62E+08 | 1.63E+08 | Polyunsaturated fatty acid content QTL (156916) | 1 | 1.1E+08 | 2.52E+08 |
| ENSSSCG00000004919 | 1 | 1.62E+08 | 1.63E+08 | Backfat at last lumbar QTL (45) | 1 | 1.21E+08 | 1.81E+08 |
| ENSSSCG00000004919 | 1 | 1.62E+08 | 1.63E+08 | Marbling QTL (4020) | 1 | 1.27E+08 | 1.85E+08 |
| ENSSSCG00000004919 | 1 | 1.62E+08 | 1.63E+08 | Average daily gain QTL (3914) | 1 | 1.31E+08 | 2.52E+08 |
| ENSSSCG00000004919 | 1 | 1.62E+08 | 1.63E+08 | Average daily gain QTL (3917) | 1 | 1.31E+08 | 2.52E+08 |
| ENSSSCG00000004919 | 1 | 1.62E+08 | 1.63E+08 | Backfat at last rib QTL (44) | 1 | 1.31E+08 | 2.02E+08 |
| ENSSSCG00000004919 | 1 | 1.62E+08 | 1.63E+08 | Loin muscle area QTL (3640) | 1 | 1.31E+08 | 1.85E+08 |
| ENSSSCG00000004919 | 1 | 1.62E+08 | 1.63E+08 | Loin muscle area QTL (2742) | 1 | 1.31E+08 | 1.85E+08 |
| ENSSSCG00000004919 | 1 | 1.62E+08 | 1.63E+08 | Average backfat thickness QTL (17996) | 1 | 1.31E+08 | 1.69E+08 |
| ENSSSCG00000004919 | 1 | 1.62E+08 | 1.63E+08 | Drip loss QTL (7784) | 1 | 1.41E+08 | 2.02E+08 |
| ENSSSCG00000004919 | 1 | 1.62E+08 | 1.63E+08 | Drip loss QTL (7861) | 1 | 1.41E+08 | 2.02E+08 |
| ENSSSCG00000004919 | 1 | 1.62E+08 | 1.63E+08 | Drip loss QTL (8102) | 1 | 1.41E+08 | 2.02E+08 |
| ENSSSCG00000004919 | 1 | 1.62E+08 | 1.63E+08 | Drip loss QTL (8156) | 1 | 1.41E+08 | 2.02E+08 |
| ENSSSCG00000004919 | 1 | 1.62E+08 | 1.63E+08 | Drip loss QTL (8348) | 1 | 1.41E+08 | 2.02E+08 |
| ENSSSCG00000004919 | 1 | 1.62E+08 | 1.63E+08 | Average daily gain QTL (5681) | 1 | 1.44E+08 | 2.52E+08 |
| ENSSSCG00000004919 | 1 | 1.62E+08 | 1.63E+08 | Stearic acid content QTL (156864) | 1 | 1.44E+08 | 2.52E+08 |
| ENSSSCG00000004919 | 1 | 1.62E+08 | 1.63E+08 | Percentage type I fibers QTL (2794) | 1 | 1.5E+08 | 2.52E+08 |
| ENSSSCG00000004919 | 1 | 1.62E+08 | 1.63E+08 | Diameter of type IIb muscle fibers QTL (2795) | 1 | 1.5E+08 | 2.52E+08 |
| ENSSSCG00000004938 | 1 | 1.64E+08 | 1.64E+08 | Average daily gain QTL (16887) | 1 | 1.42E+08 | 1.69E+08 |
| ENSSSCG00000004938 | 1 | 1.64E+08 | 1.64E+08 | backfat above muscle dorsi QTL (5673) | 1 | 1.44E+08 | 1.98E+08 |
| ENSSSCG00000004938 | 1 | 1.64E+08 | 1.64E+08 | Palmitoleic acid content QTL (156911) | 1 | 1.44E+08 | 1.98E+08 |
| ENSSSCG00000004938 | 1 | 1.64E+08 | 1.64E+08 | Linolenic acid content QTL (686) | 1 | 1.44E+08 | 1.85E+08 |
| ENSSSCG00000004938 | 1 | 1.64E+08 | 1.64E+08 | Backfat at last rib QTL (16832) | 1 | 1.45E+08 | 1.78E+08 |
| ENSSSCG00000004938 | 1 | 1.64E+08 | 1.64E+08 | Backfat at last rib QTL (16880) | 1 | 1.45E+08 | 1.78E+08 |
| ENSSSCG00000004938 | 1 | 1.64E+08 | 1.64E+08 | Backfat at last rib QTL (16882) | 1 | 1.45E+08 | 1.78E+08 |
| ENSSSCG00000004938 | 1 | 1.64E+08 | 1.64E+08 | Subcutanous fat area QTL (12737) | 1 | 1.45E+08 | 1.69E+08 |
| ENSSSCG00000004938 | 1 | 1.64E+08 | 1.64E+08 | Aspartate aminotransferase activity QTL (7480) | 1 | 1.45E+08 | 1.69E+08 |
| ENSSSCG00000004938 | 1 | 1.64E+08 | 1.64E+08 | Average daily gain QTL (16845) | 1 | 1.45E+08 | 1.69E+08 |
| ENSSSCG00000004938 | 1 | 1.64E+08 | 1.64E+08 | Backfat at last rib QTL (850) | 1 | 1.45E+08 | 1.69E+08 |
| ENSSSCG00000004938 | 1 | 1.64E+08 | 1.64E+08 | Average backfat thickness QTL (864) | 1 | 1.45E+08 | 1.69E+08 |
| ENSSSCG00000004938 | 1 | 1.64E+08 | 1.64E+08 | Backfat thickness between 3rd and 4th rib QTL (867) | 1 | 1.45E+08 | 1.69E+08 |
| ENSSSCG00000004938 | 1 | 1.64E+08 | 1.64E+08 | Meat color a* QTL (12663) | 1 | 1.52E+08 | 1.99E+08 |
| ENSSSCG00000004938 | 1 | 1.64E+08 | 1.64E+08 | Percentage type I fibers QTL (4021) | 1 | 1.53E+08 | 1.74E+08 |
| ENSSSCG00000004938 | 1 | 1.64E+08 | 1.64E+08 | Average backfat thickness QTL (2844) | 1 | 1.55E+08 | 1.81E+08 |
| ENSSSCG00000004938 | 1 | 1.64E+08 | 1.64E+08 | Average daily gain QTL (5680) | 1 | 5379030 | 2.52E+08 |
| ENSSSCG00000004938 | 1 | 1.64E+08 | 1.64E+08 | Shoulder subcutaneous fat thickness QTL (5671) | 1 | 5379030 | 1.98E+08 |
| ENSSSCG00000004938 | 1 | 1.64E+08 | 1.64E+08 | Average backfat thickness QTL (5670) | 1 | 5379030 | 1.68E+08 |
| ENSSSCG00000004938 | 1 | 1.64E+08 | 1.64E+08 | Shoulder subcutaneous fat thickness QTL (21209) | 1 | 7172040 | 2.51E+08 |
| ENSSSCG00000004938 | 1 | 1.64E+08 | 1.64E+08 | Backfat at last rib QTL (21208) | 1 | 15061284 | 2.48E+08 |
| ENSSSCG00000004938 | 1 | 1.64E+08 | 1.64E+08 | Shoulder subcutaneous fat thickness QTL (5217) | 1 | 29405363 | 2.48E+08 |
| ENSSSCG00000004938 | 1 | 1.64E+08 | 1.64E+08 | Backfat at last rib QTL (5674) | 1 | 29405363 | 1.98E+08 |
| ENSSSCG00000004938 | 1 | 1.64E+08 | 1.64E+08 | Subcutanous fat area QTL (5676) | 1 | 59886534 | 1.68E+08 |
| ENSSSCG00000004938 | 1 | 1.64E+08 | 1.64E+08 | Skin thickness QTL (31809) | 1 | 77637333 | 2.53E+08 |
| ENSSSCG00000004938 | 1 | 1.64E+08 | 1.64E+08 | Linoleic acid content QTL (156913) | 1 | 79071741 | 2.52E+08 |
| ENSSSCG00000004938 | 1 | 1.64E+08 | 1.64E+08 | Leaf fat weight QTL (21207) | 1 | 79609644 | 2.42E+08 |
| ENSSSCG00000004938 | 1 | 1.64E+08 | 1.64E+08 | Drip loss QTL (8382) | 1 | 91802112 | 2.68E+08 |
| ENSSSCG00000004938 | 1 | 1.64E+08 | 1.64E+08 | Drip loss QTL (8480) | 1 | 91802112 | 2.68E+08 |
| ENSSSCG00000004938 | 1 | 1.64E+08 | 1.64E+08 | Drip loss QTL (8564) | 1 | 91802112 | 2.68E+08 |
| ENSSSCG00000004938 | 1 | 1.64E+08 | 1.64E+08 | Drip loss QTL (8602) | 1 | 91802112 | 2.68E+08 |
| ENSSSCG00000004938 | 1 | 1.64E+08 | 1.64E+08 | Drip loss QTL (7936) | 1 | 91802112 | 2.68E+08 |
| ENSSSCG00000004938 | 1 | 1.64E+08 | 1.64E+08 | Drip loss QTL (8063) | 1 | 91802112 | 2.68E+08 |
| ENSSSCG00000004938 | 1 | 1.64E+08 | 1.64E+08 | Drip loss QTL (8131) | 1 | 91802112 | 2.68E+08 |
| ENSSSCG00000004938 | 1 | 1.64E+08 | 1.64E+08 | Drip loss QTL (8300) | 1 | 91802112 | 2.68E+08 |
| ENSSSCG00000004938 | 1 | 1.64E+08 | 1.64E+08 | Drip loss QTL (8323) | 1 | 91802112 | 2.68E+08 |
| ENSSSCG00000004938 | 1 | 1.64E+08 | 1.64E+08 | Backfat at mid-back QTL (5206) | 1 | 93953724 | 2.51E+08 |
| ENSSSCG00000004938 | 1 | 1.64E+08 | 1.64E+08 | Backfat at rump QTL (651) | 1 | 1E+08 | 1.68E+08 |
| ENSSSCG00000004938 | 1 | 1.64E+08 | 1.64E+08 | Average backfat thickness QTL (3793) | 1 | 1.06E+08 | 2.14E+08 |
| ENSSSCG00000004938 | 1 | 1.64E+08 | 1.64E+08 | Backfat at last rib QTL (3794) | 1 | 1.06E+08 | 2.14E+08 |
| ENSSSCG00000004938 | 1 | 1.64E+08 | 1.64E+08 | Meat color OPTO QTL (5667) | 1 | 1.06E+08 | 1.98E+08 |
| ENSSSCG00000004938 | 1 | 1.64E+08 | 1.64E+08 | Backfat at tenth rib QTL (3795) | 1 | 1.06E+08 | 1.85E+08 |
| ENSSSCG00000004938 | 1 | 1.64E+08 | 1.64E+08 | Loin muscle area QTL (3796) | 1 | 1.06E+08 | 1.85E+08 |
| ENSSSCG00000004938 | 1 | 1.64E+08 | 1.64E+08 | Polyunsaturated fatty acid content QTL (156916) | 1 | 1.1E+08 | 2.52E+08 |
| ENSSSCG00000004938 | 1 | 1.64E+08 | 1.64E+08 | Backfat at last lumbar QTL (45) | 1 | 1.21E+08 | 1.81E+08 |
| ENSSSCG00000004938 | 1 | 1.64E+08 | 1.64E+08 | Marbling QTL (4020) | 1 | 1.27E+08 | 1.85E+08 |
| ENSSSCG00000004938 | 1 | 1.64E+08 | 1.64E+08 | Average daily gain QTL (3914) | 1 | 1.31E+08 | 2.52E+08 |
| ENSSSCG00000004938 | 1 | 1.64E+08 | 1.64E+08 | Average daily gain QTL (3917) | 1 | 1.31E+08 | 2.52E+08 |
| ENSSSCG00000004938 | 1 | 1.64E+08 | 1.64E+08 | Backfat at last rib QTL (44) | 1 | 1.31E+08 | 2.02E+08 |
| ENSSSCG00000004938 | 1 | 1.64E+08 | 1.64E+08 | Loin muscle area QTL (3640) | 1 | 1.31E+08 | 1.85E+08 |
| ENSSSCG00000004938 | 1 | 1.64E+08 | 1.64E+08 | Loin muscle area QTL (2742) | 1 | 1.31E+08 | 1.85E+08 |
| ENSSSCG00000004938 | 1 | 1.64E+08 | 1.64E+08 | Average backfat thickness QTL (17996) | 1 | 1.31E+08 | 1.69E+08 |
| ENSSSCG00000004938 | 1 | 1.64E+08 | 1.64E+08 | Drip loss QTL (7784) | 1 | 1.41E+08 | 2.02E+08 |
| ENSSSCG00000004938 | 1 | 1.64E+08 | 1.64E+08 | Drip loss QTL (7861) | 1 | 1.41E+08 | 2.02E+08 |
| ENSSSCG00000004938 | 1 | 1.64E+08 | 1.64E+08 | Drip loss QTL (8102) | 1 | 1.41E+08 | 2.02E+08 |
| ENSSSCG00000004938 | 1 | 1.64E+08 | 1.64E+08 | Drip loss QTL (8156) | 1 | 1.41E+08 | 2.02E+08 |
| ENSSSCG00000004938 | 1 | 1.64E+08 | 1.64E+08 | Drip loss QTL (8348) | 1 | 1.41E+08 | 2.02E+08 |
| ENSSSCG00000004938 | 1 | 1.64E+08 | 1.64E+08 | Average daily gain QTL (5681) | 1 | 1.44E+08 | 2.52E+08 |
| ENSSSCG00000004938 | 1 | 1.64E+08 | 1.64E+08 | Stearic acid content QTL (156864) | 1 | 1.44E+08 | 2.52E+08 |
| ENSSSCG00000004938 | 1 | 1.64E+08 | 1.64E+08 | Percentage type I fibers QTL (2794) | 1 | 1.5E+08 | 2.52E+08 |
| ENSSSCG00000004938 | 1 | 1.64E+08 | 1.64E+08 | Diameter of type IIb muscle fibers QTL (2795) | 1 | 1.5E+08 | 2.52E+08 |
| ENSSSCG00000004979 | 1 | 1.69E+08 | 1.7E+08 | backfat above muscle dorsi QTL (5673) | 1 | 1.44E+08 | 1.98E+08 |
| ENSSSCG00000004979 | 1 | 1.69E+08 | 1.7E+08 | Palmitoleic acid content QTL (156911) | 1 | 1.44E+08 | 1.98E+08 |
| ENSSSCG00000004979 | 1 | 1.69E+08 | 1.7E+08 | Linolenic acid content QTL (686) | 1 | 1.44E+08 | 1.85E+08 |
| ENSSSCG00000004979 | 1 | 1.69E+08 | 1.7E+08 | Backfat at last rib QTL (16832) | 1 | 1.45E+08 | 1.78E+08 |
| ENSSSCG00000004979 | 1 | 1.69E+08 | 1.7E+08 | Backfat at last rib QTL (16880) | 1 | 1.45E+08 | 1.78E+08 |
| ENSSSCG00000004979 | 1 | 1.69E+08 | 1.7E+08 | Backfat at last rib QTL (16882) | 1 | 1.45E+08 | 1.78E+08 |
| ENSSSCG00000004979 | 1 | 1.69E+08 | 1.7E+08 | Meat color a* QTL (12663) | 1 | 1.52E+08 | 1.99E+08 |
| ENSSSCG00000004979 | 1 | 1.69E+08 | 1.7E+08 | Percentage type I fibers QTL (4021) | 1 | 1.53E+08 | 1.74E+08 |
| ENSSSCG00000004979 | 1 | 1.69E+08 | 1.7E+08 | Average backfat thickness QTL (2844) | 1 | 1.55E+08 | 1.81E+08 |
| ENSSSCG00000004979 | 1 | 1.69E+08 | 1.7E+08 | Average daily gain QTL (5929) | 1 | 1.68E+08 | 1.98E+08 |
| ENSSSCG00000004979 | 1 | 1.69E+08 | 1.7E+08 | Backfat at last rib QTL (5957) | 1 | 1.68E+08 | 1.98E+08 |
| ENSSSCG00000004979 | 1 | 1.69E+08 | 1.7E+08 | Saturated fatty acid content QTL (156866) | 1 | 1.69E+08 | 1.98E+08 |
| ENSSSCG00000004979 | 1 | 1.69E+08 | 1.7E+08 | Unsaturated fatty acid content QTL (156867) | 1 | 1.69E+08 | 1.98E+08 |
| ENSSSCG00000004979 | 1 | 1.69E+08 | 1.7E+08 | Subcutanous fat area QTL (18002) | 1 | 1.69E+08 | 1.8E+08 |
| ENSSSCG00000004979 | 1 | 1.69E+08 | 1.7E+08 | Average daily gain QTL (5680) | 1 | 5379030 | 2.52E+08 |
| ENSSSCG00000004979 | 1 | 1.69E+08 | 1.7E+08 | Shoulder subcutaneous fat thickness QTL (5671) | 1 | 5379030 | 1.98E+08 |
| ENSSSCG00000004979 | 1 | 1.69E+08 | 1.7E+08 | Shoulder subcutaneous fat thickness QTL (21209) | 1 | 7172040 | 2.51E+08 |
| ENSSSCG00000004979 | 1 | 1.69E+08 | 1.7E+08 | Backfat at last rib QTL (21208) | 1 | 15061284 | 2.48E+08 |
| ENSSSCG00000004979 | 1 | 1.69E+08 | 1.7E+08 | Shoulder subcutaneous fat thickness QTL (5217) | 1 | 29405363 | 2.48E+08 |
| ENSSSCG00000004979 | 1 | 1.69E+08 | 1.7E+08 | Backfat at last rib QTL (5674) | 1 | 29405363 | 1.98E+08 |
| ENSSSCG00000004979 | 1 | 1.69E+08 | 1.7E+08 | Skin thickness QTL (31809) | 1 | 77637333 | 2.53E+08 |
| ENSSSCG00000004979 | 1 | 1.69E+08 | 1.7E+08 | Linoleic acid content QTL (156913) | 1 | 79071741 | 2.52E+08 |
| ENSSSCG00000004979 | 1 | 1.69E+08 | 1.7E+08 | Leaf fat weight QTL (21207) | 1 | 79609644 | 2.42E+08 |
| ENSSSCG00000004979 | 1 | 1.69E+08 | 1.7E+08 | Drip loss QTL (8382) | 1 | 91802112 | 2.68E+08 |
| ENSSSCG00000004979 | 1 | 1.69E+08 | 1.7E+08 | Drip loss QTL (8480) | 1 | 91802112 | 2.68E+08 |
| ENSSSCG00000004979 | 1 | 1.69E+08 | 1.7E+08 | Drip loss QTL (8564) | 1 | 91802112 | 2.68E+08 |
| ENSSSCG00000004979 | 1 | 1.69E+08 | 1.7E+08 | Drip loss QTL (8602) | 1 | 91802112 | 2.68E+08 |
| ENSSSCG00000004979 | 1 | 1.69E+08 | 1.7E+08 | Drip loss QTL (7936) | 1 | 91802112 | 2.68E+08 |
| ENSSSCG00000004979 | 1 | 1.69E+08 | 1.7E+08 | Drip loss QTL (8063) | 1 | 91802112 | 2.68E+08 |
| ENSSSCG00000004979 | 1 | 1.69E+08 | 1.7E+08 | Drip loss QTL (8131) | 1 | 91802112 | 2.68E+08 |
| ENSSSCG00000004979 | 1 | 1.69E+08 | 1.7E+08 | Drip loss QTL (8300) | 1 | 91802112 | 2.68E+08 |
| ENSSSCG00000004979 | 1 | 1.69E+08 | 1.7E+08 | Drip loss QTL (8323) | 1 | 91802112 | 2.68E+08 |
| ENSSSCG00000004979 | 1 | 1.69E+08 | 1.7E+08 | Backfat at mid-back QTL (5206) | 1 | 93953724 | 2.51E+08 |
| ENSSSCG00000004979 | 1 | 1.69E+08 | 1.7E+08 | Average backfat thickness QTL (3793) | 1 | 1.06E+08 | 2.14E+08 |
| ENSSSCG00000004979 | 1 | 1.69E+08 | 1.7E+08 | Backfat at last rib QTL (3794) | 1 | 1.06E+08 | 2.14E+08 |
| ENSSSCG00000004979 | 1 | 1.69E+08 | 1.7E+08 | Meat color OPTO QTL (5667) | 1 | 1.06E+08 | 1.98E+08 |
| ENSSSCG00000004979 | 1 | 1.69E+08 | 1.7E+08 | Backfat at tenth rib QTL (3795) | 1 | 1.06E+08 | 1.85E+08 |
| ENSSSCG00000004979 | 1 | 1.69E+08 | 1.7E+08 | Loin muscle area QTL (3796) | 1 | 1.06E+08 | 1.85E+08 |
| ENSSSCG00000004979 | 1 | 1.69E+08 | 1.7E+08 | Polyunsaturated fatty acid content QTL (156916) | 1 | 1.1E+08 | 2.52E+08 |
| ENSSSCG00000004979 | 1 | 1.69E+08 | 1.7E+08 | Backfat at last lumbar QTL (45) | 1 | 1.21E+08 | 1.81E+08 |
| ENSSSCG00000004979 | 1 | 1.69E+08 | 1.7E+08 | Marbling QTL (4020) | 1 | 1.27E+08 | 1.85E+08 |
| ENSSSCG00000004979 | 1 | 1.69E+08 | 1.7E+08 | Average daily gain QTL (3914) | 1 | 1.31E+08 | 2.52E+08 |
| ENSSSCG00000004979 | 1 | 1.69E+08 | 1.7E+08 | Average daily gain QTL (3917) | 1 | 1.31E+08 | 2.52E+08 |
| ENSSSCG00000004979 | 1 | 1.69E+08 | 1.7E+08 | Backfat at last rib QTL (44) | 1 | 1.31E+08 | 2.02E+08 |
| ENSSSCG00000004979 | 1 | 1.69E+08 | 1.7E+08 | Loin muscle area QTL (3640) | 1 | 1.31E+08 | 1.85E+08 |
| ENSSSCG00000004979 | 1 | 1.69E+08 | 1.7E+08 | Loin muscle area QTL (2742) | 1 | 1.31E+08 | 1.85E+08 |
| ENSSSCG00000004979 | 1 | 1.69E+08 | 1.7E+08 | Drip loss QTL (7784) | 1 | 1.41E+08 | 2.02E+08 |
| ENSSSCG00000004979 | 1 | 1.69E+08 | 1.7E+08 | Drip loss QTL (7861) | 1 | 1.41E+08 | 2.02E+08 |
| ENSSSCG00000004979 | 1 | 1.69E+08 | 1.7E+08 | Drip loss QTL (8102) | 1 | 1.41E+08 | 2.02E+08 |
| ENSSSCG00000004979 | 1 | 1.69E+08 | 1.7E+08 | Drip loss QTL (8156) | 1 | 1.41E+08 | 2.02E+08 |
| ENSSSCG00000004979 | 1 | 1.69E+08 | 1.7E+08 | Drip loss QTL (8348) | 1 | 1.41E+08 | 2.02E+08 |
| ENSSSCG00000004979 | 1 | 1.69E+08 | 1.7E+08 | Average daily gain QTL (5681) | 1 | 1.44E+08 | 2.52E+08 |
| ENSSSCG00000004979 | 1 | 1.69E+08 | 1.7E+08 | Stearic acid content QTL (156864) | 1 | 1.44E+08 | 2.52E+08 |
| ENSSSCG00000004979 | 1 | 1.69E+08 | 1.7E+08 | Percentage type I fibers QTL (2794) | 1 | 1.5E+08 | 2.52E+08 |
| ENSSSCG00000004979 | 1 | 1.69E+08 | 1.7E+08 | Diameter of type IIb muscle fibers QTL (2795) | 1 | 1.5E+08 | 2.52E+08 |
| ENSSSCG00000004979 | 1 | 1.69E+08 | 1.7E+08 | Drip loss QTL (80) | 1 | 1.68E+08 | 2.74E+08 |
| ENSSSCG00000004979 | 1 | 1.69E+08 | 1.7E+08 | Carcass length QTL (828) | 1 | 1.69E+08 | 2.2E+08 |
| ENSSSCG00000004979 | 1 | 1.69E+08 | 1.7E+08 | Carcass length QTL (851) | 1 | 1.69E+08 | 2.2E+08 |
| ENSSSCG00000004979 | 1 | 1.69E+08 | 1.7E+08 | Carcass length QTL (857) | 1 | 1.69E+08 | 2.2E+08 |
| ENSSSCG00000004979 | 1 | 1.69E+08 | 1.7E+08 | Body weight (slaughter) QTL (862) | 1 | 1.69E+08 | 2.2E+08 |
| ENSSSCG00000004979 | 1 | 1.69E+08 | 1.7E+08 | Loin muscle area QTL (865) | 1 | 1.69E+08 | 2.2E+08 |
| ENSSSCG00000004979 | 1 | 1.69E+08 | 1.7E+08 | Alkaline phosphatase activity QTL (6352) | 1 | 1.69E+08 | 2.2E+08 |
| ENSSSCG00000004979 | 1 | 1.69E+08 | 1.7E+08 | Alkaline phosphatase activity QTL (6355) | 1 | 1.69E+08 | 2.2E+08 |
| ENSSSCG00000004979 | 1 | 1.69E+08 | 1.7E+08 | Adipocyte diameter QTL (12803) | 1 | 1.69E+08 | 2.2E+08 |
| ENSSSCG00000005090 | 1 | 1.9E+08 | 1.9E+08 | Musculus biceps femoris and ham ratio QTL (1273) | 1 | 1.87E+08 | 1.91E+08 |
| ENSSSCG00000005090 | 1 | 1.9E+08 | 1.9E+08 | Drip loss QTL (8082) | 1 | 1.89E+08 | 1.9E+08 |
| ENSSSCG00000005090 | 1 | 1.9E+08 | 1.9E+08 | Drip loss QTL (8090) | 1 | 1.89E+08 | 1.9E+08 |
| ENSSSCG00000005090 | 1 | 1.9E+08 | 1.9E+08 | Drip loss QTL (8333) | 1 | 1.89E+08 | 1.9E+08 |
| ENSSSCG00000005090 | 1 | 1.9E+08 | 1.9E+08 | Drip loss QTL (8512) | 1 | 1.89E+08 | 1.9E+08 |
| ENSSSCG00000005090 | 1 | 1.9E+08 | 1.9E+08 | Drip loss QTL (7783) | 1 | 1.89E+08 | 1.9E+08 |
| ENSSSCG00000005090 | 1 | 1.9E+08 | 1.9E+08 | Drip loss QTL (7920) | 1 | 1.89E+08 | 1.9E+08 |
| ENSSSCG00000005090 | 1 | 1.9E+08 | 1.9E+08 | Drip loss QTL (8038) | 1 | 1.89E+08 | 1.9E+08 |
| ENSSSCG00000005090 | 1 | 1.9E+08 | 1.9E+08 | Drip loss QTL (8049) | 1 | 1.89E+08 | 1.9E+08 |
| ENSSSCG00000005090 | 1 | 1.9E+08 | 1.9E+08 | backfat above muscle dorsi QTL (5673) | 1 | 1.44E+08 | 1.98E+08 |
| ENSSSCG00000005090 | 1 | 1.9E+08 | 1.9E+08 | Palmitoleic acid content QTL (156911) | 1 | 1.44E+08 | 1.98E+08 |
| ENSSSCG00000005090 | 1 | 1.9E+08 | 1.9E+08 | Meat color a* QTL (12663) | 1 | 1.52E+08 | 1.99E+08 |
| ENSSSCG00000005090 | 1 | 1.9E+08 | 1.9E+08 | Average daily gain QTL (5929) | 1 | 1.68E+08 | 1.98E+08 |
| ENSSSCG00000005090 | 1 | 1.9E+08 | 1.9E+08 | Backfat at last rib QTL (5957) | 1 | 1.68E+08 | 1.98E+08 |
| ENSSSCG00000005090 | 1 | 1.9E+08 | 1.9E+08 | Saturated fatty acid content QTL (156866) | 1 | 1.69E+08 | 1.98E+08 |
| ENSSSCG00000005090 | 1 | 1.9E+08 | 1.9E+08 | Unsaturated fatty acid content QTL (156867) | 1 | 1.69E+08 | 1.98E+08 |
| ENSSSCG00000005090 | 1 | 1.9E+08 | 1.9E+08 | Carcass length QTL (9) | 1 | 1.72E+08 | 1.91E+08 |
| ENSSSCG00000005090 | 1 | 1.9E+08 | 1.9E+08 | Average daily gain QTL (5680) | 1 | 5379030 | 2.52E+08 |
| ENSSSCG00000005090 | 1 | 1.9E+08 | 1.9E+08 | Shoulder subcutaneous fat thickness QTL (5671) | 1 | 5379030 | 1.98E+08 |
| ENSSSCG00000005090 | 1 | 1.9E+08 | 1.9E+08 | Shoulder subcutaneous fat thickness QTL (21209) | 1 | 7172040 | 2.51E+08 |
| ENSSSCG00000005090 | 1 | 1.9E+08 | 1.9E+08 | Backfat at last rib QTL (21208) | 1 | 15061284 | 2.48E+08 |
| ENSSSCG00000005090 | 1 | 1.9E+08 | 1.9E+08 | Shoulder subcutaneous fat thickness QTL (5217) | 1 | 29405363 | 2.48E+08 |
| ENSSSCG00000005090 | 1 | 1.9E+08 | 1.9E+08 | Backfat at last rib QTL (5674) | 1 | 29405363 | 1.98E+08 |
| ENSSSCG00000005090 | 1 | 1.9E+08 | 1.9E+08 | Skin thickness QTL (31809) | 1 | 77637333 | 2.53E+08 |
| ENSSSCG00000005090 | 1 | 1.9E+08 | 1.9E+08 | Linoleic acid content QTL (156913) | 1 | 79071741 | 2.52E+08 |
| ENSSSCG00000005090 | 1 | 1.9E+08 | 1.9E+08 | Leaf fat weight QTL (21207) | 1 | 79609644 | 2.42E+08 |
| ENSSSCG00000005090 | 1 | 1.9E+08 | 1.9E+08 | Drip loss QTL (8382) | 1 | 91802112 | 2.68E+08 |
| ENSSSCG00000005090 | 1 | 1.9E+08 | 1.9E+08 | Drip loss QTL (8480) | 1 | 91802112 | 2.68E+08 |
| ENSSSCG00000005090 | 1 | 1.9E+08 | 1.9E+08 | Drip loss QTL (8564) | 1 | 91802112 | 2.68E+08 |
| ENSSSCG00000005090 | 1 | 1.9E+08 | 1.9E+08 | Drip loss QTL (8602) | 1 | 91802112 | 2.68E+08 |
| ENSSSCG00000005090 | 1 | 1.9E+08 | 1.9E+08 | Drip loss QTL (7936) | 1 | 91802112 | 2.68E+08 |
| ENSSSCG00000005090 | 1 | 1.9E+08 | 1.9E+08 | Drip loss QTL (8063) | 1 | 91802112 | 2.68E+08 |
| ENSSSCG00000005090 | 1 | 1.9E+08 | 1.9E+08 | Drip loss QTL (8131) | 1 | 91802112 | 2.68E+08 |
| ENSSSCG00000005090 | 1 | 1.9E+08 | 1.9E+08 | Drip loss QTL (8300) | 1 | 91802112 | 2.68E+08 |
| ENSSSCG00000005090 | 1 | 1.9E+08 | 1.9E+08 | Drip loss QTL (8323) | 1 | 91802112 | 2.68E+08 |
| ENSSSCG00000005090 | 1 | 1.9E+08 | 1.9E+08 | Backfat at mid-back QTL (5206) | 1 | 93953724 | 2.51E+08 |
| ENSSSCG00000005090 | 1 | 1.9E+08 | 1.9E+08 | Average backfat thickness QTL (3793) | 1 | 1.06E+08 | 2.14E+08 |
| ENSSSCG00000005090 | 1 | 1.9E+08 | 1.9E+08 | Backfat at last rib QTL (3794) | 1 | 1.06E+08 | 2.14E+08 |
| ENSSSCG00000005090 | 1 | 1.9E+08 | 1.9E+08 | Meat color OPTO QTL (5667) | 1 | 1.06E+08 | 1.98E+08 |
| ENSSSCG00000005090 | 1 | 1.9E+08 | 1.9E+08 | Polyunsaturated fatty acid content QTL (156916) | 1 | 1.1E+08 | 2.52E+08 |
| ENSSSCG00000005090 | 1 | 1.9E+08 | 1.9E+08 | Average daily gain QTL (3914) | 1 | 1.31E+08 | 2.52E+08 |
| ENSSSCG00000005090 | 1 | 1.9E+08 | 1.9E+08 | Average daily gain QTL (3917) | 1 | 1.31E+08 | 2.52E+08 |
| ENSSSCG00000005090 | 1 | 1.9E+08 | 1.9E+08 | Backfat at last rib QTL (44) | 1 | 1.31E+08 | 2.02E+08 |
| ENSSSCG00000005090 | 1 | 1.9E+08 | 1.9E+08 | Drip loss QTL (7784) | 1 | 1.41E+08 | 2.02E+08 |
| ENSSSCG00000005090 | 1 | 1.9E+08 | 1.9E+08 | Drip loss QTL (7861) | 1 | 1.41E+08 | 2.02E+08 |
| ENSSSCG00000005090 | 1 | 1.9E+08 | 1.9E+08 | Drip loss QTL (8102) | 1 | 1.41E+08 | 2.02E+08 |
| ENSSSCG00000005090 | 1 | 1.9E+08 | 1.9E+08 | Drip loss QTL (8156) | 1 | 1.41E+08 | 2.02E+08 |
| ENSSSCG00000005090 | 1 | 1.9E+08 | 1.9E+08 | Drip loss QTL (8348) | 1 | 1.41E+08 | 2.02E+08 |
| ENSSSCG00000005090 | 1 | 1.9E+08 | 1.9E+08 | Average daily gain QTL (5681) | 1 | 1.44E+08 | 2.52E+08 |
| ENSSSCG00000005090 | 1 | 1.9E+08 | 1.9E+08 | Stearic acid content QTL (156864) | 1 | 1.44E+08 | 2.52E+08 |
| ENSSSCG00000005090 | 1 | 1.9E+08 | 1.9E+08 | Percentage type I fibers QTL (2794) | 1 | 1.5E+08 | 2.52E+08 |
| ENSSSCG00000005090 | 1 | 1.9E+08 | 1.9E+08 | Diameter of type IIb muscle fibers QTL (2795) | 1 | 1.5E+08 | 2.52E+08 |
| ENSSSCG00000005090 | 1 | 1.9E+08 | 1.9E+08 | Drip loss QTL (80) | 1 | 1.68E+08 | 2.74E+08 |
| ENSSSCG00000005090 | 1 | 1.9E+08 | 1.9E+08 | Carcass length QTL (828) | 1 | 1.69E+08 | 2.2E+08 |
| ENSSSCG00000005090 | 1 | 1.9E+08 | 1.9E+08 | Carcass length QTL (851) | 1 | 1.69E+08 | 2.2E+08 |
| ENSSSCG00000005090 | 1 | 1.9E+08 | 1.9E+08 | Carcass length QTL (857) | 1 | 1.69E+08 | 2.2E+08 |
| ENSSSCG00000005090 | 1 | 1.9E+08 | 1.9E+08 | Body weight (slaughter) QTL (862) | 1 | 1.69E+08 | 2.2E+08 |
| ENSSSCG00000005090 | 1 | 1.9E+08 | 1.9E+08 | Loin muscle area QTL (865) | 1 | 1.69E+08 | 2.2E+08 |
| ENSSSCG00000005090 | 1 | 1.9E+08 | 1.9E+08 | Alkaline phosphatase activity QTL (6352) | 1 | 1.69E+08 | 2.2E+08 |
| ENSSSCG00000005090 | 1 | 1.9E+08 | 1.9E+08 | Alkaline phosphatase activity QTL (6355) | 1 | 1.69E+08 | 2.2E+08 |
| ENSSSCG00000005090 | 1 | 1.9E+08 | 1.9E+08 | Adipocyte diameter QTL (12803) | 1 | 1.69E+08 | 2.2E+08 |
| ENSSSCG00000005090 | 1 | 1.9E+08 | 1.9E+08 | Average backfat thickness QTL (310) | 1 | 1.72E+08 | 2.17E+08 |
| ENSSSCG00000005090 | 1 | 1.9E+08 | 1.9E+08 | Locomotion QTL (478) | 1 | 1.77E+08 | 2.23E+08 |
| ENSSSCG00000005090 | 1 | 1.9E+08 | 1.9E+08 | Adipocyte diameter QTL (18019) | 1 | 1.79E+08 | 2.56E+08 |
| ENSSSCG00000005090 | 1 | 1.9E+08 | 1.9E+08 | Backfat at last rib QTL (16833) | 1 | 1.79E+08 | 2.56E+08 |
| ENSSSCG00000005090 | 1 | 1.9E+08 | 1.9E+08 | Average daily gain QTL (2846) | 1 | 1.81E+08 | 2.12E+08 |
| ENSSSCG00000005090 | 1 | 1.9E+08 | 1.9E+08 | Average daily gain QTL (2847) | 1 | 1.81E+08 | 2.12E+08 |
| ENSSSCG00000005090 | 1 | 1.9E+08 | 1.9E+08 | Protein accretion rate QTL (2849) | 1 | 1.81E+08 | 2.12E+08 |
| ENSSSCG00000005090 | 1 | 1.9E+08 | 1.9E+08 | Fat area percentage in carcass QTL (18634) | 1 | 1.85E+08 | 2.58E+08 |
| ENSSSCG00000005090 | 1 | 1.9E+08 | 1.9E+08 | Arachidonic acid content QTL (156914) | 1 | 1.85E+08 | 2.52E+08 |
| ENSSSCG00000005090 | 1 | 1.9E+08 | 1.9E+08 | Meat color score QTL (3773) | 1 | 1.85E+08 | 2.52E+08 |
| ENSSSCG00000005090 | 1 | 1.9E+08 | 1.9E+08 | pH 48 hr post-mortem (loin) QTL (3772) | 1 | 1.85E+08 | 2.14E+08 |
| ENSSSCG00000005215 | 1 | 2.17E+08 | 2.17E+08 | Drip loss QTL (8006) | 1 | 2.17E+08 | 2.17E+08 |
| ENSSSCG00000005215 | 1 | 2.17E+08 | 2.17E+08 | Average backfat thickness QTL (21204) | 1 | 2.09E+08 | 2.41E+08 |
| ENSSSCG00000005215 | 1 | 2.17E+08 | 2.17E+08 | Shoulder subcutaneous fat thickness QTL (5216) | 1 | 2.12E+08 | 2.52E+08 |
| ENSSSCG00000005215 | 1 | 2.17E+08 | 2.17E+08 | Average daily gain QTL (3133) | 1 | 2.12E+08 | 2.58E+08 |
| ENSSSCG00000005215 | 1 | 2.17E+08 | 2.17E+08 | Average backfat thickness QTL (3145) | 1 | 2.12E+08 | 2.58E+08 |
| ENSSSCG00000005215 | 1 | 2.17E+08 | 2.17E+08 | pH for Semispinalis Dorsi QTL (3168) | 1 | 2.12E+08 | 2.58E+08 |
| ENSSSCG00000005215 | 1 | 2.17E+08 | 2.17E+08 | PH for Longissmus dorsi QTL (3169) | 1 | 2.12E+08 | 2.58E+08 |
| ENSSSCG00000005215 | 1 | 2.17E+08 | 2.17E+08 | PH for Biceps femoris QTL (3170) | 1 | 2.12E+08 | 2.58E+08 |
| ENSSSCG00000005215 | 1 | 2.17E+08 | 2.17E+08 | Average backfat thickness QTL (35) | 1 | 2.12E+08 | 2.58E+08 |
| ENSSSCG00000005215 | 1 | 2.17E+08 | 2.17E+08 | Intestinal fat weight QTL (21210) | 1 | 2.12E+08 | 2.45E+08 |
| ENSSSCG00000005215 | 1 | 2.17E+08 | 2.17E+08 | Average daily gain QTL (5930) | 1 | 2.14E+08 | 2.52E+08 |
| ENSSSCG00000005215 | 1 | 2.17E+08 | 2.17E+08 | Marbling QTL (2931) | 1 | 2.14E+08 | 2.52E+08 |
| ENSSSCG00000005215 | 1 | 2.17E+08 | 2.17E+08 | Average daily gain QTL (446) | 1 | 2.14E+08 | 2.52E+08 |
| ENSSSCG00000005215 | 1 | 2.17E+08 | 2.17E+08 | Average daily gain QTL (447) | 1 | 2.14E+08 | 2.52E+08 |
| ENSSSCG00000005215 | 1 | 2.17E+08 | 2.17E+08 | Average daily gain QTL (448) | 1 | 2.14E+08 | 2.52E+08 |
| ENSSSCG00000005215 | 1 | 2.17E+08 | 2.17E+08 | cis-9-Heptadecenoic acid content QTL (156912) | 1 | 2.14E+08 | 2.52E+08 |
| ENSSSCG00000005215 | 1 | 2.17E+08 | 2.17E+08 | Average daily gain QTL (5680) | 1 | 5379030 | 2.52E+08 |
| ENSSSCG00000005215 | 1 | 2.17E+08 | 2.17E+08 | Shoulder subcutaneous fat thickness QTL (21209) | 1 | 7172040 | 2.51E+08 |
| ENSSSCG00000005215 | 1 | 2.17E+08 | 2.17E+08 | Backfat at last rib QTL (21208) | 1 | 15061284 | 2.48E+08 |
| ENSSSCG00000005215 | 1 | 2.17E+08 | 2.17E+08 | Shoulder subcutaneous fat thickness QTL (5217) | 1 | 29405363 | 2.48E+08 |
| ENSSSCG00000005215 | 1 | 2.17E+08 | 2.17E+08 | Skin thickness QTL (31809) | 1 | 77637333 | 2.53E+08 |
| ENSSSCG00000005215 | 1 | 2.17E+08 | 2.17E+08 | Linoleic acid content QTL (156913) | 1 | 79071741 | 2.52E+08 |
| ENSSSCG00000005215 | 1 | 2.17E+08 | 2.17E+08 | Leaf fat weight QTL (21207) | 1 | 79609644 | 2.42E+08 |
| ENSSSCG00000005215 | 1 | 2.17E+08 | 2.17E+08 | Drip loss QTL (8382) | 1 | 91802112 | 2.68E+08 |
| ENSSSCG00000005215 | 1 | 2.17E+08 | 2.17E+08 | Drip loss QTL (8480) | 1 | 91802112 | 2.68E+08 |
| ENSSSCG00000005215 | 1 | 2.17E+08 | 2.17E+08 | Drip loss QTL (8564) | 1 | 91802112 | 2.68E+08 |
| ENSSSCG00000005215 | 1 | 2.17E+08 | 2.17E+08 | Drip loss QTL (8602) | 1 | 91802112 | 2.68E+08 |
| ENSSSCG00000005215 | 1 | 2.17E+08 | 2.17E+08 | Drip loss QTL (7936) | 1 | 91802112 | 2.68E+08 |
| ENSSSCG00000005215 | 1 | 2.17E+08 | 2.17E+08 | Drip loss QTL (8063) | 1 | 91802112 | 2.68E+08 |
| ENSSSCG00000005215 | 1 | 2.17E+08 | 2.17E+08 | Drip loss QTL (8131) | 1 | 91802112 | 2.68E+08 |
| ENSSSCG00000005215 | 1 | 2.17E+08 | 2.17E+08 | Drip loss QTL (8300) | 1 | 91802112 | 2.68E+08 |
| ENSSSCG00000005215 | 1 | 2.17E+08 | 2.17E+08 | Drip loss QTL (8323) | 1 | 91802112 | 2.68E+08 |
| ENSSSCG00000005215 | 1 | 2.17E+08 | 2.17E+08 | Backfat at mid-back QTL (5206) | 1 | 93953724 | 2.51E+08 |
| ENSSSCG00000005215 | 1 | 2.17E+08 | 2.17E+08 | Polyunsaturated fatty acid content QTL (156916) | 1 | 1.1E+08 | 2.52E+08 |
| ENSSSCG00000005215 | 1 | 2.17E+08 | 2.17E+08 | Average daily gain QTL (3914) | 1 | 1.31E+08 | 2.52E+08 |
| ENSSSCG00000005215 | 1 | 2.17E+08 | 2.17E+08 | Average daily gain QTL (3917) | 1 | 1.31E+08 | 2.52E+08 |
| ENSSSCG00000005215 | 1 | 2.17E+08 | 2.17E+08 | Average daily gain QTL (5681) | 1 | 1.44E+08 | 2.52E+08 |
| ENSSSCG00000005215 | 1 | 2.17E+08 | 2.17E+08 | Stearic acid content QTL (156864) | 1 | 1.44E+08 | 2.52E+08 |
| ENSSSCG00000005215 | 1 | 2.17E+08 | 2.17E+08 | Percentage type I fibers QTL (2794) | 1 | 1.5E+08 | 2.52E+08 |
| ENSSSCG00000005215 | 1 | 2.17E+08 | 2.17E+08 | Diameter of type IIb muscle fibers QTL (2795) | 1 | 1.5E+08 | 2.52E+08 |
| ENSSSCG00000005215 | 1 | 2.17E+08 | 2.17E+08 | Drip loss QTL (80) | 1 | 1.68E+08 | 2.74E+08 |
| ENSSSCG00000005215 | 1 | 2.17E+08 | 2.17E+08 | Carcass length QTL (828) | 1 | 1.69E+08 | 2.2E+08 |
| ENSSSCG00000005215 | 1 | 2.17E+08 | 2.17E+08 | Carcass length QTL (851) | 1 | 1.69E+08 | 2.2E+08 |
| ENSSSCG00000005215 | 1 | 2.17E+08 | 2.17E+08 | Carcass length QTL (857) | 1 | 1.69E+08 | 2.2E+08 |
| ENSSSCG00000005215 | 1 | 2.17E+08 | 2.17E+08 | Body weight (slaughter) QTL (862) | 1 | 1.69E+08 | 2.2E+08 |
| ENSSSCG00000005215 | 1 | 2.17E+08 | 2.17E+08 | Loin muscle area QTL (865) | 1 | 1.69E+08 | 2.2E+08 |
| ENSSSCG00000005215 | 1 | 2.17E+08 | 2.17E+08 | Alkaline phosphatase activity QTL (6352) | 1 | 1.69E+08 | 2.2E+08 |
| ENSSSCG00000005215 | 1 | 2.17E+08 | 2.17E+08 | Alkaline phosphatase activity QTL (6355) | 1 | 1.69E+08 | 2.2E+08 |
| ENSSSCG00000005215 | 1 | 2.17E+08 | 2.17E+08 | Adipocyte diameter QTL (12803) | 1 | 1.69E+08 | 2.2E+08 |
| ENSSSCG00000005215 | 1 | 2.17E+08 | 2.17E+08 | Average backfat thickness QTL (310) | 1 | 1.72E+08 | 2.17E+08 |
| ENSSSCG00000005215 | 1 | 2.17E+08 | 2.17E+08 | Locomotion QTL (478) | 1 | 1.77E+08 | 2.23E+08 |
| ENSSSCG00000005215 | 1 | 2.17E+08 | 2.17E+08 | Adipocyte diameter QTL (18019) | 1 | 1.79E+08 | 2.56E+08 |
| ENSSSCG00000005215 | 1 | 2.17E+08 | 2.17E+08 | Backfat at last rib QTL (16833) | 1 | 1.79E+08 | 2.56E+08 |
| ENSSSCG00000005215 | 1 | 2.17E+08 | 2.17E+08 | Fat area percentage in carcass QTL (18634) | 1 | 1.85E+08 | 2.58E+08 |
| ENSSSCG00000005215 | 1 | 2.17E+08 | 2.17E+08 | Arachidonic acid content QTL (156914) | 1 | 1.85E+08 | 2.52E+08 |
| ENSSSCG00000005215 | 1 | 2.17E+08 | 2.17E+08 | Meat color score QTL (3773) | 1 | 1.85E+08 | 2.52E+08 |
| ENSSSCG00000005215 | 1 | 2.17E+08 | 2.17E+08 | Capric acid content QTL (156910) | 1 | 1.98E+08 | 2.52E+08 |
| ENSSSCG00000005215 | 1 | 2.17E+08 | 2.17E+08 | Average daily gain QTL (659) | 1 | 2.12E+08 | 2.74E+08 |
| ENSSSCG00000005215 | 1 | 2.17E+08 | 2.17E+08 | body weight (10 weeks) QTL (660) | 1 | 2.12E+08 | 2.74E+08 |
| ENSSSCG00000005215 | 1 | 2.17E+08 | 2.17E+08 | body weight (13 weeks) QTL (661) | 1 | 2.12E+08 | 2.74E+08 |
| ENSSSCG00000005215 | 1 | 2.17E+08 | 2.17E+08 | body weight (17 weeks) QTL (662) | 1 | 2.12E+08 | 2.74E+08 |
| ENSSSCG00000023520 | 1 | 2.29E+08 | 2.29E+08 | LDL cholesterol QTL (171085) | 1 | 2.29E+08 | 2.29E+08 |
| ENSSSCG00000023520 | 1 | 2.29E+08 | 2.29E+08 | Adipocyte diameter QTL (12818) | 1 | 2.29E+08 | 2.29E+08 |
| ENSSSCG00000023520 | 1 | 2.29E+08 | 2.29E+08 | Average backfat thickness QTL (21204) | 1 | 2.09E+08 | 2.41E+08 |
| ENSSSCG00000023520 | 1 | 2.29E+08 | 2.29E+08 | Shoulder subcutaneous fat thickness QTL (5216) | 1 | 2.12E+08 | 2.52E+08 |
| ENSSSCG00000023520 | 1 | 2.29E+08 | 2.29E+08 | Average daily gain QTL (3133) | 1 | 2.12E+08 | 2.58E+08 |
| ENSSSCG00000023520 | 1 | 2.29E+08 | 2.29E+08 | Average backfat thickness QTL (3145) | 1 | 2.12E+08 | 2.58E+08 |
| ENSSSCG00000023520 | 1 | 2.29E+08 | 2.29E+08 | pH for Semispinalis Dorsi QTL (3168) | 1 | 2.12E+08 | 2.58E+08 |
| ENSSSCG00000023520 | 1 | 2.29E+08 | 2.29E+08 | PH for Longissmus dorsi QTL (3169) | 1 | 2.12E+08 | 2.58E+08 |
| ENSSSCG00000023520 | 1 | 2.29E+08 | 2.29E+08 | PH for Biceps femoris QTL (3170) | 1 | 2.12E+08 | 2.58E+08 |
| ENSSSCG00000023520 | 1 | 2.29E+08 | 2.29E+08 | Average backfat thickness QTL (35) | 1 | 2.12E+08 | 2.58E+08 |
| ENSSSCG00000023520 | 1 | 2.29E+08 | 2.29E+08 | Intestinal fat weight QTL (21210) | 1 | 2.12E+08 | 2.45E+08 |
| ENSSSCG00000023520 | 1 | 2.29E+08 | 2.29E+08 | Average daily gain QTL (5930) | 1 | 2.14E+08 | 2.52E+08 |
| ENSSSCG00000023520 | 1 | 2.29E+08 | 2.29E+08 | Marbling QTL (2931) | 1 | 2.14E+08 | 2.52E+08 |
| ENSSSCG00000023520 | 1 | 2.29E+08 | 2.29E+08 | Average daily gain QTL (446) | 1 | 2.14E+08 | 2.52E+08 |
| ENSSSCG00000023520 | 1 | 2.29E+08 | 2.29E+08 | Average daily gain QTL (447) | 1 | 2.14E+08 | 2.52E+08 |
| ENSSSCG00000023520 | 1 | 2.29E+08 | 2.29E+08 | Average daily gain QTL (448) | 1 | 2.14E+08 | 2.52E+08 |
| ENSSSCG00000023520 | 1 | 2.29E+08 | 2.29E+08 | cis-9-Heptadecenoic acid content QTL (156912) | 1 | 2.14E+08 | 2.52E+08 |
| ENSSSCG00000023520 | 1 | 2.29E+08 | 2.29E+08 | Loin muscle area QTL (4) | 1 | 2.17E+08 | 2.58E+08 |
| ENSSSCG00000023520 | 1 | 2.29E+08 | 2.29E+08 | Backfat at first rib QTL (21205) | 1 | 2.18E+08 | 2.47E+08 |
| ENSSSCG00000023520 | 1 | 2.29E+08 | 2.29E+08 | Backfat at last rib QTL (27) | 1 | 2.2E+08 | 2.58E+08 |
| ENSSSCG00000023520 | 1 | 2.29E+08 | 2.29E+08 | Backfat at last lumbar QTL (30) | 1 | 2.2E+08 | 2.58E+08 |
| ENSSSCG00000023520 | 1 | 2.29E+08 | 2.29E+08 | Conductivity 24 hours post-mortem QTL (847) | 1 | 2.2E+08 | 2.56E+08 |
| ENSSSCG00000023520 | 1 | 2.29E+08 | 2.29E+08 | Shoulder subcutaneous fat thickness QTL (5250) | 1 | 2.21E+08 | 2.52E+08 |
| ENSSSCG00000023520 | 1 | 2.29E+08 | 2.29E+08 | Average daily gain QTL (3665) | 1 | 2.28E+08 | 2.52E+08 |
| ENSSSCG00000023520 | 1 | 2.29E+08 | 2.29E+08 | Backfat at last rib QTL (3666) | 1 | 2.28E+08 | 2.52E+08 |
| ENSSSCG00000023520 | 1 | 2.29E+08 | 2.29E+08 | Average daily gain QTL (5680) | 1 | 5379030 | 2.52E+08 |
| ENSSSCG00000023520 | 1 | 2.29E+08 | 2.29E+08 | Shoulder subcutaneous fat thickness QTL (21209) | 1 | 7172040 | 2.51E+08 |
| ENSSSCG00000023520 | 1 | 2.29E+08 | 2.29E+08 | Backfat at last rib QTL (21208) | 1 | 15061284 | 2.48E+08 |
| ENSSSCG00000023520 | 1 | 2.29E+08 | 2.29E+08 | Shoulder subcutaneous fat thickness QTL (5217) | 1 | 29405363 | 2.48E+08 |
| ENSSSCG00000023520 | 1 | 2.29E+08 | 2.29E+08 | Skin thickness QTL (31809) | 1 | 77637333 | 2.53E+08 |
| ENSSSCG00000023520 | 1 | 2.29E+08 | 2.29E+08 | Linoleic acid content QTL (156913) | 1 | 79071741 | 2.52E+08 |
| ENSSSCG00000023520 | 1 | 2.29E+08 | 2.29E+08 | Leaf fat weight QTL (21207) | 1 | 79609644 | 2.42E+08 |
| ENSSSCG00000023520 | 1 | 2.29E+08 | 2.29E+08 | Drip loss QTL (8382) | 1 | 91802112 | 2.68E+08 |
| ENSSSCG00000023520 | 1 | 2.29E+08 | 2.29E+08 | Drip loss QTL (8480) | 1 | 91802112 | 2.68E+08 |
| ENSSSCG00000023520 | 1 | 2.29E+08 | 2.29E+08 | Drip loss QTL (8564) | 1 | 91802112 | 2.68E+08 |
| ENSSSCG00000023520 | 1 | 2.29E+08 | 2.29E+08 | Drip loss QTL (8602) | 1 | 91802112 | 2.68E+08 |
| ENSSSCG00000023520 | 1 | 2.29E+08 | 2.29E+08 | Drip loss QTL (7936) | 1 | 91802112 | 2.68E+08 |
| ENSSSCG00000023520 | 1 | 2.29E+08 | 2.29E+08 | Drip loss QTL (8063) | 1 | 91802112 | 2.68E+08 |
| ENSSSCG00000023520 | 1 | 2.29E+08 | 2.29E+08 | Drip loss QTL (8131) | 1 | 91802112 | 2.68E+08 |
| ENSSSCG00000023520 | 1 | 2.29E+08 | 2.29E+08 | Drip loss QTL (8300) | 1 | 91802112 | 2.68E+08 |
| ENSSSCG00000023520 | 1 | 2.29E+08 | 2.29E+08 | Drip loss QTL (8323) | 1 | 91802112 | 2.68E+08 |
| ENSSSCG00000023520 | 1 | 2.29E+08 | 2.29E+08 | Backfat at mid-back QTL (5206) | 1 | 93953724 | 2.51E+08 |
| ENSSSCG00000023520 | 1 | 2.29E+08 | 2.29E+08 | Polyunsaturated fatty acid content QTL (156916) | 1 | 1.1E+08 | 2.52E+08 |
| ENSSSCG00000023520 | 1 | 2.29E+08 | 2.29E+08 | Average daily gain QTL (3914) | 1 | 1.31E+08 | 2.52E+08 |
| ENSSSCG00000023520 | 1 | 2.29E+08 | 2.29E+08 | Average daily gain QTL (3917) | 1 | 1.31E+08 | 2.52E+08 |
| ENSSSCG00000023520 | 1 | 2.29E+08 | 2.29E+08 | Average daily gain QTL (5681) | 1 | 1.44E+08 | 2.52E+08 |
| ENSSSCG00000023520 | 1 | 2.29E+08 | 2.29E+08 | Stearic acid content QTL (156864) | 1 | 1.44E+08 | 2.52E+08 |
| ENSSSCG00000023520 | 1 | 2.29E+08 | 2.29E+08 | Percentage type I fibers QTL (2794) | 1 | 1.5E+08 | 2.52E+08 |
| ENSSSCG00000023520 | 1 | 2.29E+08 | 2.29E+08 | Diameter of type IIb muscle fibers QTL (2795) | 1 | 1.5E+08 | 2.52E+08 |
| ENSSSCG00000023520 | 1 | 2.29E+08 | 2.29E+08 | Drip loss QTL (80) | 1 | 1.68E+08 | 2.74E+08 |
| ENSSSCG00000023520 | 1 | 2.29E+08 | 2.29E+08 | Adipocyte diameter QTL (18019) | 1 | 1.79E+08 | 2.56E+08 |
| ENSSSCG00000023520 | 1 | 2.29E+08 | 2.29E+08 | Backfat at last rib QTL (16833) | 1 | 1.79E+08 | 2.56E+08 |
| ENSSSCG00000023520 | 1 | 2.29E+08 | 2.29E+08 | Fat area percentage in carcass QTL (18634) | 1 | 1.85E+08 | 2.58E+08 |
| ENSSSCG00000023520 | 1 | 2.29E+08 | 2.29E+08 | Arachidonic acid content QTL (156914) | 1 | 1.85E+08 | 2.52E+08 |
| ENSSSCG00000023520 | 1 | 2.29E+08 | 2.29E+08 | Meat color score QTL (3773) | 1 | 1.85E+08 | 2.52E+08 |
| ENSSSCG00000023520 | 1 | 2.29E+08 | 2.29E+08 | Capric acid content QTL (156910) | 1 | 1.98E+08 | 2.52E+08 |
| ENSSSCG00000023520 | 1 | 2.29E+08 | 2.29E+08 | Average daily gain QTL (659) | 1 | 2.12E+08 | 2.74E+08 |
| ENSSSCG00000023520 | 1 | 2.29E+08 | 2.29E+08 | body weight (10 weeks) QTL (660) | 1 | 2.12E+08 | 2.74E+08 |
| ENSSSCG00000023520 | 1 | 2.29E+08 | 2.29E+08 | body weight (13 weeks) QTL (661) | 1 | 2.12E+08 | 2.74E+08 |
| ENSSSCG00000023520 | 1 | 2.29E+08 | 2.29E+08 | body weight (17 weeks) QTL (662) | 1 | 2.12E+08 | 2.74E+08 |
| ENSSSCG00000023520 | 1 | 2.29E+08 | 2.29E+08 | Backfat at tenth rib QTL (23) | 1 | 2.22E+08 | 2.69E+08 |
| ENSSSCG00000005444 | 1 | 2.5E+08 | 2.5E+08 | Shoulder subcutaneous fat thickness QTL (5216) | 1 | 2.12E+08 | 2.52E+08 |
| ENSSSCG00000005444 | 1 | 2.5E+08 | 2.5E+08 | Average daily gain QTL (3133) | 1 | 2.12E+08 | 2.58E+08 |
| ENSSSCG00000005444 | 1 | 2.5E+08 | 2.5E+08 | Average backfat thickness QTL (3145) | 1 | 2.12E+08 | 2.58E+08 |
| ENSSSCG00000005444 | 1 | 2.5E+08 | 2.5E+08 | pH for Semispinalis Dorsi QTL (3168) | 1 | 2.12E+08 | 2.58E+08 |
| ENSSSCG00000005444 | 1 | 2.5E+08 | 2.5E+08 | PH for Longissmus dorsi QTL (3169) | 1 | 2.12E+08 | 2.58E+08 |
| ENSSSCG00000005444 | 1 | 2.5E+08 | 2.5E+08 | PH for Biceps femoris QTL (3170) | 1 | 2.12E+08 | 2.58E+08 |
| ENSSSCG00000005444 | 1 | 2.5E+08 | 2.5E+08 | Average backfat thickness QTL (35) | 1 | 2.12E+08 | 2.58E+08 |
| ENSSSCG00000005444 | 1 | 2.5E+08 | 2.5E+08 | Average daily gain QTL (5930) | 1 | 2.14E+08 | 2.52E+08 |
| ENSSSCG00000005444 | 1 | 2.5E+08 | 2.5E+08 | Marbling QTL (2931) | 1 | 2.14E+08 | 2.52E+08 |
| ENSSSCG00000005444 | 1 | 2.5E+08 | 2.5E+08 | Average daily gain QTL (446) | 1 | 2.14E+08 | 2.52E+08 |
| ENSSSCG00000005444 | 1 | 2.5E+08 | 2.5E+08 | Average daily gain QTL (447) | 1 | 2.14E+08 | 2.52E+08 |
| ENSSSCG00000005444 | 1 | 2.5E+08 | 2.5E+08 | Average daily gain QTL (448) | 1 | 2.14E+08 | 2.52E+08 |
| ENSSSCG00000005444 | 1 | 2.5E+08 | 2.5E+08 | cis-9-Heptadecenoic acid content QTL (156912) | 1 | 2.14E+08 | 2.52E+08 |
| ENSSSCG00000005444 | 1 | 2.5E+08 | 2.5E+08 | Loin muscle area QTL (4) | 1 | 2.17E+08 | 2.58E+08 |
| ENSSSCG00000005444 | 1 | 2.5E+08 | 2.5E+08 | Backfat at last rib QTL (27) | 1 | 2.2E+08 | 2.58E+08 |
| ENSSSCG00000005444 | 1 | 2.5E+08 | 2.5E+08 | Backfat at last lumbar QTL (30) | 1 | 2.2E+08 | 2.58E+08 |
| ENSSSCG00000005444 | 1 | 2.5E+08 | 2.5E+08 | Conductivity 24 hours post-mortem QTL (847) | 1 | 2.2E+08 | 2.56E+08 |
| ENSSSCG00000005444 | 1 | 2.5E+08 | 2.5E+08 | Shoulder subcutaneous fat thickness QTL (5250) | 1 | 2.21E+08 | 2.52E+08 |
| ENSSSCG00000005444 | 1 | 2.5E+08 | 2.5E+08 | Average daily gain QTL (3665) | 1 | 2.28E+08 | 2.52E+08 |
| ENSSSCG00000005444 | 1 | 2.5E+08 | 2.5E+08 | Backfat at last rib QTL (3666) | 1 | 2.28E+08 | 2.52E+08 |
| ENSSSCG00000005444 | 1 | 2.5E+08 | 2.5E+08 | backfat above muscle dorsi QTL (5207) | 1 | 2.32E+08 | 2.52E+08 |
| ENSSSCG00000005444 | 1 | 2.5E+08 | 2.5E+08 | body weight (10 weeks) QTL (168) | 1 | 2.33E+08 | 2.52E+08 |
| ENSSSCG00000005444 | 1 | 2.5E+08 | 2.5E+08 | Average backfat thickness QTL (192) | 1 | 2.35E+08 | 2.54E+08 |
| ENSSSCG00000005444 | 1 | 2.5E+08 | 2.5E+08 | Average backfat thickness QTL (193) | 1 | 2.35E+08 | 2.54E+08 |
| ENSSSCG00000005444 | 1 | 2.5E+08 | 2.5E+08 | Average backfat thickness QTL (194) | 1 | 2.35E+08 | 2.54E+08 |
| ENSSSCG00000005444 | 1 | 2.5E+08 | 2.5E+08 | Average backfat thickness QTL (195) | 1 | 2.35E+08 | 2.54E+08 |
| ENSSSCG00000005444 | 1 | 2.5E+08 | 2.5E+08 | Average backfat thickness QTL (196) | 1 | 2.35E+08 | 2.54E+08 |
| ENSSSCG00000005444 | 1 | 2.5E+08 | 2.5E+08 | Backfat at mid-back QTL (5241) | 1 | 2.35E+08 | 2.52E+08 |
| ENSSSCG00000005444 | 1 | 2.5E+08 | 2.5E+08 | Average daily gain QTL (170) | 1 | 2.37E+08 | 2.54E+08 |
| ENSSSCG00000005444 | 1 | 2.5E+08 | 2.5E+08 | body weight (13 weeks) QTL (169) | 1 | 2.4E+08 | 2.62E+08 |
| ENSSSCG00000005444 | 1 | 2.5E+08 | 2.5E+08 | Body weight (3 weeks) QTL (167) | 1 | 2.47E+08 | 2.52E+08 |
| ENSSSCG00000005444 | 1 | 2.5E+08 | 2.5E+08 | Average daily gain QTL (5680) | 1 | 5379030 | 2.52E+08 |
| ENSSSCG00000005444 | 1 | 2.5E+08 | 2.5E+08 | Shoulder subcutaneous fat thickness QTL (21209) | 1 | 7172040 | 2.51E+08 |
| ENSSSCG00000005444 | 1 | 2.5E+08 | 2.5E+08 | Skin thickness QTL (31809) | 1 | 77637333 | 2.53E+08 |
| ENSSSCG00000005444 | 1 | 2.5E+08 | 2.5E+08 | Linoleic acid content QTL (156913) | 1 | 79071741 | 2.52E+08 |
| ENSSSCG00000005444 | 1 | 2.5E+08 | 2.5E+08 | Drip loss QTL (8382) | 1 | 91802112 | 2.68E+08 |
| ENSSSCG00000005444 | 1 | 2.5E+08 | 2.5E+08 | Drip loss QTL (8480) | 1 | 91802112 | 2.68E+08 |
| ENSSSCG00000005444 | 1 | 2.5E+08 | 2.5E+08 | Drip loss QTL (8564) | 1 | 91802112 | 2.68E+08 |
| ENSSSCG00000005444 | 1 | 2.5E+08 | 2.5E+08 | Drip loss QTL (8602) | 1 | 91802112 | 2.68E+08 |
| ENSSSCG00000005444 | 1 | 2.5E+08 | 2.5E+08 | Drip loss QTL (7936) | 1 | 91802112 | 2.68E+08 |
| ENSSSCG00000005444 | 1 | 2.5E+08 | 2.5E+08 | Drip loss QTL (8063) | 1 | 91802112 | 2.68E+08 |
| ENSSSCG00000005444 | 1 | 2.5E+08 | 2.5E+08 | Drip loss QTL (8131) | 1 | 91802112 | 2.68E+08 |
| ENSSSCG00000005444 | 1 | 2.5E+08 | 2.5E+08 | Drip loss QTL (8300) | 1 | 91802112 | 2.68E+08 |
| ENSSSCG00000005444 | 1 | 2.5E+08 | 2.5E+08 | Drip loss QTL (8323) | 1 | 91802112 | 2.68E+08 |
| ENSSSCG00000005444 | 1 | 2.5E+08 | 2.5E+08 | Backfat at mid-back QTL (5206) | 1 | 93953724 | 2.51E+08 |
| ENSSSCG00000005444 | 1 | 2.5E+08 | 2.5E+08 | Polyunsaturated fatty acid content QTL (156916) | 1 | 1.1E+08 | 2.52E+08 |
| ENSSSCG00000005444 | 1 | 2.5E+08 | 2.5E+08 | Average daily gain QTL (3914) | 1 | 1.31E+08 | 2.52E+08 |
| ENSSSCG00000005444 | 1 | 2.5E+08 | 2.5E+08 | Average daily gain QTL (3917) | 1 | 1.31E+08 | 2.52E+08 |
| ENSSSCG00000005444 | 1 | 2.5E+08 | 2.5E+08 | Average daily gain QTL (5681) | 1 | 1.44E+08 | 2.52E+08 |
| ENSSSCG00000005444 | 1 | 2.5E+08 | 2.5E+08 | Stearic acid content QTL (156864) | 1 | 1.44E+08 | 2.52E+08 |
| ENSSSCG00000005444 | 1 | 2.5E+08 | 2.5E+08 | Percentage type I fibers QTL (2794) | 1 | 1.5E+08 | 2.52E+08 |
| ENSSSCG00000005444 | 1 | 2.5E+08 | 2.5E+08 | Diameter of type IIb muscle fibers QTL (2795) | 1 | 1.5E+08 | 2.52E+08 |
| ENSSSCG00000005444 | 1 | 2.5E+08 | 2.5E+08 | Drip loss QTL (80) | 1 | 1.68E+08 | 2.74E+08 |
| ENSSSCG00000005444 | 1 | 2.5E+08 | 2.5E+08 | Adipocyte diameter QTL (18019) | 1 | 1.79E+08 | 2.56E+08 |
| ENSSSCG00000005444 | 1 | 2.5E+08 | 2.5E+08 | Backfat at last rib QTL (16833) | 1 | 1.79E+08 | 2.56E+08 |
| ENSSSCG00000005444 | 1 | 2.5E+08 | 2.5E+08 | Fat area percentage in carcass QTL (18634) | 1 | 1.85E+08 | 2.58E+08 |
| ENSSSCG00000005444 | 1 | 2.5E+08 | 2.5E+08 | Arachidonic acid content QTL (156914) | 1 | 1.85E+08 | 2.52E+08 |
| ENSSSCG00000005444 | 1 | 2.5E+08 | 2.5E+08 | Meat color score QTL (3773) | 1 | 1.85E+08 | 2.52E+08 |
| ENSSSCG00000005444 | 1 | 2.5E+08 | 2.5E+08 | Capric acid content QTL (156910) | 1 | 1.98E+08 | 2.52E+08 |
| ENSSSCG00000005444 | 1 | 2.5E+08 | 2.5E+08 | Average daily gain QTL (659) | 1 | 2.12E+08 | 2.74E+08 |
| ENSSSCG00000005444 | 1 | 2.5E+08 | 2.5E+08 | body weight (10 weeks) QTL (660) | 1 | 2.12E+08 | 2.74E+08 |
| ENSSSCG00000005444 | 1 | 2.5E+08 | 2.5E+08 | body weight (13 weeks) QTL (661) | 1 | 2.12E+08 | 2.74E+08 |
| ENSSSCG00000005444 | 1 | 2.5E+08 | 2.5E+08 | body weight (17 weeks) QTL (662) | 1 | 2.12E+08 | 2.74E+08 |
| ENSSSCG00000005444 | 1 | 2.5E+08 | 2.5E+08 | Backfat at tenth rib QTL (23) | 1 | 2.22E+08 | 2.69E+08 |
| ENSSSCG00000020743 | 1 | 2.64E+08 | 2.64E+08 | Drip loss QTL (8382) | 1 | 91802112 | 2.68E+08 |
| ENSSSCG00000020743 | 1 | 2.64E+08 | 2.64E+08 | Drip loss QTL (8480) | 1 | 91802112 | 2.68E+08 |
| ENSSSCG00000020743 | 1 | 2.64E+08 | 2.64E+08 | Drip loss QTL (8564) | 1 | 91802112 | 2.68E+08 |
| ENSSSCG00000020743 | 1 | 2.64E+08 | 2.64E+08 | Drip loss QTL (8602) | 1 | 91802112 | 2.68E+08 |
| ENSSSCG00000020743 | 1 | 2.64E+08 | 2.64E+08 | Drip loss QTL (7936) | 1 | 91802112 | 2.68E+08 |
| ENSSSCG00000020743 | 1 | 2.64E+08 | 2.64E+08 | Drip loss QTL (8063) | 1 | 91802112 | 2.68E+08 |
| ENSSSCG00000020743 | 1 | 2.64E+08 | 2.64E+08 | Drip loss QTL (8131) | 1 | 91802112 | 2.68E+08 |
| ENSSSCG00000020743 | 1 | 2.64E+08 | 2.64E+08 | Drip loss QTL (8300) | 1 | 91802112 | 2.68E+08 |
| ENSSSCG00000020743 | 1 | 2.64E+08 | 2.64E+08 | Drip loss QTL (8323) | 1 | 91802112 | 2.68E+08 |
| ENSSSCG00000020743 | 1 | 2.64E+08 | 2.64E+08 | Drip loss QTL (80) | 1 | 1.68E+08 | 2.74E+08 |
| ENSSSCG00000020743 | 1 | 2.64E+08 | 2.64E+08 | Average daily gain QTL (659) | 1 | 2.12E+08 | 2.74E+08 |
| ENSSSCG00000020743 | 1 | 2.64E+08 | 2.64E+08 | body weight (10 weeks) QTL (660) | 1 | 2.12E+08 | 2.74E+08 |
| ENSSSCG00000020743 | 1 | 2.64E+08 | 2.64E+08 | body weight (13 weeks) QTL (661) | 1 | 2.12E+08 | 2.74E+08 |
| ENSSSCG00000020743 | 1 | 2.64E+08 | 2.64E+08 | body weight (17 weeks) QTL (662) | 1 | 2.12E+08 | 2.74E+08 |
| ENSSSCG00000020743 | 1 | 2.64E+08 | 2.64E+08 | Backfat at tenth rib QTL (23) | 1 | 2.22E+08 | 2.69E+08 |
| ENSSSCG00000020743 | 1 | 2.64E+08 | 2.64E+08 | Leaf fat weight QTL (402) | 1 | 2.56E+08 | 2.78E+08 |
| ENSSSCG00000010882 | 10 | 19046380 | 19139658 | Shoulder subcutaneous fat thickness QTL (5253) | 10 | 2167480 | 66758384 |
| ENSSSCG00000010882 | 10 | 19046380 | 19139658 | Percentage type I fibers QTL (7012) | 10 | 2330041 | 33758501 |
| ENSSSCG00000010882 | 10 | 19046380 | 19139658 | Percentage type I fibers QTL (7026) | 10 | 2330041 | 33758501 |
| ENSSSCG00000010882 | 10 | 19046380 | 19139658 | Percentage type IIa fibers QTL (7034) | 10 | 2330041 | 33758501 |
| ENSSSCG00000010882 | 10 | 19046380 | 19139658 | Backfat at mid-back QTL (5247) | 10 | 3847277 | 62965294 |
| ENSSSCG00000010882 | 10 | 19046380 | 19139658 | Palmitic acid content QTL (156946) | 10 | 4118212 | 46763381 |
| ENSSSCG00000010882 | 10 | 19046380 | 19139658 | Average daily gain QTL (16856) | 10 | 11054148 | 31265899 |
| ENSSSCG00000010882 | 10 | 19046380 | 19139658 | Carcass weight (hot) QTL (16871) | 10 | 11054148 | 31265899 |
| ENSSSCG00000010882 | 10 | 19046380 | 19139658 | Creatinine level QTL (6363) | 10 | 11054148 | 21295491 |
| ENSSSCG00000010882 | 10 | 19046380 | 19139658 | Creatinine level QTL (6365) | 10 | 11054148 | 21295491 |
| ENSSSCG00000010882 | 10 | 19046380 | 19139658 | Average daily lean meat gain QTL (3939) | 10 | 12571384 | 23842280 |
| ENSSSCG00000010882 | 10 | 19046380 | 19139658 | Meat color a* QTL (3067) | 10 | 12571384 | 21295491 |
| ENSSSCG00000010882 | 10 | 19046380 | 19139658 | Linoleic acid content QTL (15863) | 10 | 13709311 | 28123053 |
| ENSSSCG00000010882 | 10 | 19046380 | 19139658 | Average daily gain QTL (250) | 10 | 15172360 | 40640250 |
| ENSSSCG00000010882 | 10 | 19046380 | 19139658 | Marbling QTL (38100) | 10 | 4118212 | 69359360 |
| ENSSSCG00000011022 | 10 | 41177977 | 41462766 | Myristic acid content QTL (7190) | 10 | 41252563 | 41691477 |
| ENSSSCG00000011022 | 10 | 41177977 | 41462766 | Shoulder subcutaneous fat thickness QTL (5253) | 10 | 2167480 | 66758384 |
| ENSSSCG00000011022 | 10 | 41177977 | 41462766 | Backfat at mid-back QTL (5247) | 10 | 3847277 | 62965294 |
| ENSSSCG00000011022 | 10 | 41177977 | 41462766 | Palmitic acid content QTL (156946) | 10 | 4118212 | 46763381 |
| ENSSSCG00000011022 | 10 | 41177977 | 41462766 | Creatinine level QTL (15074) | 10 | 30398907 | 52019520 |
| ENSSSCG00000011022 | 10 | 41177977 | 41462766 | External fat on loin QTL (5193) | 10 | 31043732 | 54127394 |
| ENSSSCG00000011022 | 10 | 41177977 | 41462766 | Percentage type IIa fibers QTL (7031) | 10 | 33758501 | 58521960 |
| ENSSSCG00000011022 | 10 | 41177977 | 41462766 | Body weight (slaughter) QTL (5948) | 10 | 33758501 | 54728870 |
| ENSSSCG00000011022 | 10 | 41177977 | 41462766 | Shear force QTL (6011) | 10 | 33758501 | 54728870 |
| ENSSSCG00000011022 | 10 | 41177977 | 41462766 | Carcass weight (cold) QTL (3786) | 10 | 35275737 | 46763381 |
| ENSSSCG00000011022 | 10 | 41177977 | 41462766 | Backfat at last rib QTL (3787) | 10 | 35275737 | 46763381 |
| ENSSSCG00000011022 | 10 | 41177977 | 41462766 | Average instron (star probe) force QTL (3813) | 10 | 35275737 | 46763381 |
| ENSSSCG00000011022 | 10 | 41177977 | 41462766 | Body weight (46 days) QTL (21263) | 10 | 36305290 | 53211634 |
| ENSSSCG00000011022 | 10 | 41177977 | 41462766 | Lactate dehydrogenase level QTL (15111) | 10 | 36305290 | 48768300 |
| ENSSSCG00000011022 | 10 | 41177977 | 41462766 | subjective abnormal odor QTL (4211) | 10 | 36576225 | 54728870 |
| ENSSSCG00000011022 | 10 | 41177977 | 41462766 | Connective tissue amount QTL (21440) | 10 | 36576225 | 46763381 |
| ENSSSCG00000011022 | 10 | 41177977 | 41462766 | Body weight (slaughter) QTL (3278) | 10 | 36576225 | 46763381 |
| ENSSSCG00000011022 | 10 | 41177977 | 41462766 | Carcass weight (hot) QTL (3279) | 10 | 36576225 | 46763381 |
| ENSSSCG00000011022 | 10 | 41177977 | 41462766 | Marbling QTL (3280) | 10 | 36576225 | 46763381 |
| ENSSSCG00000011022 | 10 | 41177977 | 41462766 | Backfat linear at last rib QTL (3656) | 10 | 36576225 | 46763381 |
| ENSSSCG00000011022 | 10 | 41177977 | 41462766 | Marbling QTL (38100) | 10 | 4118212 | 69359360 |
| ENSSSCG00000011068 | 10 | 48694806 | 48855155 | Backfat at first rib QTL (21) | 10 | 46221511 | 49310170 |
| ENSSSCG00000011068 | 10 | 48694806 | 48855155 | Shoulder subcutaneous fat thickness QTL (5253) | 10 | 2167480 | 66758384 |
| ENSSSCG00000011068 | 10 | 48694806 | 48855155 | Backfat at mid-back QTL (5247) | 10 | 3847277 | 62965294 |
| ENSSSCG00000011068 | 10 | 48694806 | 48855155 | Creatinine level QTL (15074) | 10 | 30398907 | 52019520 |
| ENSSSCG00000011068 | 10 | 48694806 | 48855155 | External fat on loin QTL (5193) | 10 | 31043732 | 54127394 |
| ENSSSCG00000011068 | 10 | 48694806 | 48855155 | Percentage type IIa fibers QTL (7031) | 10 | 33758501 | 58521960 |
| ENSSSCG00000011068 | 10 | 48694806 | 48855155 | Body weight (slaughter) QTL (5948) | 10 | 33758501 | 54728870 |
| ENSSSCG00000011068 | 10 | 48694806 | 48855155 | Shear force QTL (6011) | 10 | 33758501 | 54728870 |
| ENSSSCG00000011068 | 10 | 48694806 | 48855155 | Body weight (46 days) QTL (21263) | 10 | 36305290 | 53211634 |
| ENSSSCG00000011068 | 10 | 48694806 | 48855155 | subjective abnormal odor QTL (4211) | 10 | 36576225 | 54728870 |
| ENSSSCG00000011068 | 10 | 48694806 | 48855155 | Linoleic acid content QTL (15861) | 10 | 43024478 | 54728870 |
| ENSSSCG00000011068 | 10 | 48694806 | 48855155 | Average daily gain QTL (327) | 10 | 43338762 | 53049073 |
| ENSSSCG00000011068 | 10 | 48694806 | 48855155 | Loin muscle area QTL (3821) | 10 | 46763381 | 58521960 |
| ENSSSCG00000011068 | 10 | 48694806 | 48855155 | Backfat at last rib QTL (2952) | 10 | 46763381 | 58521960 |
| ENSSSCG00000011068 | 10 | 48694806 | 48855155 | Cortisol level QTL (21328) | 10 | 47521999 | 66108140 |
| ENSSSCG00000011068 | 10 | 48694806 | 48855155 | Marbling QTL (38100) | 10 | 4118212 | 69359360 |
| ENSSSCG00000011064 | 10 | 48883201 | 48922289 | Backfat at first rib QTL (21) | 10 | 46221511 | 49310170 |
| ENSSSCG00000011064 | 10 | 48883201 | 48922289 | Shoulder subcutaneous fat thickness QTL (5253) | 10 | 2167480 | 66758384 |
| ENSSSCG00000011064 | 10 | 48883201 | 48922289 | Backfat at mid-back QTL (5247) | 10 | 3847277 | 62965294 |
| ENSSSCG00000011064 | 10 | 48883201 | 48922289 | Creatinine level QTL (15074) | 10 | 30398907 | 52019520 |
| ENSSSCG00000011064 | 10 | 48883201 | 48922289 | External fat on loin QTL (5193) | 10 | 31043732 | 54127394 |
| ENSSSCG00000011064 | 10 | 48883201 | 48922289 | Percentage type IIa fibers QTL (7031) | 10 | 33758501 | 58521960 |
| ENSSSCG00000011064 | 10 | 48883201 | 48922289 | Body weight (slaughter) QTL (5948) | 10 | 33758501 | 54728870 |
| ENSSSCG00000011064 | 10 | 48883201 | 48922289 | Shear force QTL (6011) | 10 | 33758501 | 54728870 |
| ENSSSCG00000011064 | 10 | 48883201 | 48922289 | Body weight (46 days) QTL (21263) | 10 | 36305290 | 53211634 |
| ENSSSCG00000011064 | 10 | 48883201 | 48922289 | subjective abnormal odor QTL (4211) | 10 | 36576225 | 54728870 |
| ENSSSCG00000011064 | 10 | 48883201 | 48922289 | Linoleic acid content QTL (15861) | 10 | 43024478 | 54728870 |
| ENSSSCG00000011064 | 10 | 48883201 | 48922289 | Average daily gain QTL (327) | 10 | 43338762 | 53049073 |
| ENSSSCG00000011064 | 10 | 48883201 | 48922289 | Loin muscle area QTL (3821) | 10 | 46763381 | 58521960 |
| ENSSSCG00000011064 | 10 | 48883201 | 48922289 | Backfat at last rib QTL (2952) | 10 | 46763381 | 58521960 |
| ENSSSCG00000011064 | 10 | 48883201 | 48922289 | Cortisol level QTL (21328) | 10 | 47521999 | 66108140 |
| ENSSSCG00000011064 | 10 | 48883201 | 48922289 | Marbling QTL (38100) | 10 | 4118212 | 69359360 |
| ENSSSCG00000011103 | 10 | 56967424 | 57600466 | Musculus semitendinosus (ST) weight QTL (1283) | 10 | 52019520 | 58521960 |
| ENSSSCG00000011103 | 10 | 56967424 | 57600466 | Musculus semimembranosus et adductor (SMA) weight QTL (1282) | 10 | 54728870 | 58521960 |
| ENSSSCG00000011103 | 10 | 56967424 | 57600466 | Plasma FSH concentration QTL (646) | 10 | 54728870 | 58521960 |
| ENSSSCG00000011103 | 10 | 56967424 | 57600466 | Shoulder subcutaneous fat thickness QTL (5253) | 10 | 2167480 | 66758384 |
| ENSSSCG00000011103 | 10 | 56967424 | 57600466 | Backfat at mid-back QTL (5247) | 10 | 3847277 | 62965294 |
| ENSSSCG00000011103 | 10 | 56967424 | 57600466 | Percentage type IIa fibers QTL (7031) | 10 | 33758501 | 58521960 |
| ENSSSCG00000011103 | 10 | 56967424 | 57600466 | Loin muscle area QTL (3821) | 10 | 46763381 | 58521960 |
| ENSSSCG00000011103 | 10 | 56967424 | 57600466 | Backfat at last rib QTL (2952) | 10 | 46763381 | 58521960 |
| ENSSSCG00000011103 | 10 | 56967424 | 57600466 | Cortisol level QTL (21328) | 10 | 47521999 | 66108140 |
| ENSSSCG00000011103 | 10 | 56967424 | 57600466 | Marbling QTL (38100) | 10 | 4118212 | 69359360 |
| ENSSSCG00000011103 | 10 | 56967424 | 57600466 | Creatinine level QTL (6369) | 10 | 54728870 | 69359360 |
| ENSSSCG00000011103 | 10 | 56967424 | 57600466 | Aspartate aminotransferase activity QTL (7507) | 10 | 54728870 | 69359360 |
| ENSSSCG00000034086 | 10 | 63583693 | 63599453 | Shoulder subcutaneous fat thickness QTL (5253) | 10 | 2167480 | 66758384 |
| ENSSSCG00000034086 | 10 | 63583693 | 63599453 | Cortisol level QTL (21328) | 10 | 47521999 | 66108140 |
| ENSSSCG00000034086 | 10 | 63583693 | 63599453 | Marbling QTL (38100) | 10 | 4118212 | 69359360 |
| ENSSSCG00000034086 | 10 | 63583693 | 63599453 | Creatinine level QTL (6369) | 10 | 54728870 | 69359360 |
| ENSSSCG00000034086 | 10 | 63583693 | 63599453 | Aspartate aminotransferase activity QTL (7507) | 10 | 54728870 | 69359360 |
| ENSSSCG00000034086 | 10 | 63583693 | 63599453 | Cholesterol level in meat QTL (3822) | 10 | 58521960 | 69359360 |
| ENSSSCG00000011131 | 10 | 64443251 | 64600339 | Meat color a* QTL (164857) | 10 | 64577071 | 64577075 |
| ENSSSCG00000011131 | 10 | 64443251 | 64600339 | Shoulder subcutaneous fat thickness QTL (5253) | 10 | 2167480 | 66758384 |
| ENSSSCG00000011131 | 10 | 64443251 | 64600339 | Cortisol level QTL (21328) | 10 | 47521999 | 66108140 |
| ENSSSCG00000011131 | 10 | 64443251 | 64600339 | Marbling QTL (38100) | 10 | 4118212 | 69359360 |
| ENSSSCG00000011131 | 10 | 64443251 | 64600339 | Creatinine level QTL (6369) | 10 | 54728870 | 69359360 |
| ENSSSCG00000011131 | 10 | 64443251 | 64600339 | Aspartate aminotransferase activity QTL (7507) | 10 | 54728870 | 69359360 |
| ENSSSCG00000011131 | 10 | 64443251 | 64600339 | Cholesterol level in meat QTL (3822) | 10 | 58521960 | 69359360 |
| ENSSSCG00000009300 | 11 | 4261177 | 4366254 | Loin muscle area QTL (6) | 11 | 932508 | 27975240 |
| ENSSSCG00000009405 | 11 | 19616499 | 19675791 | Loin muscle area QTL (6) | 11 | 932508 | 27975240 |
| ENSSSCG00000009405 | 11 | 19616499 | 19675791 | Backfat thickness between 3rd and 4th rib QTL (425) | 11 | 4662540 | 43827876 |
| ENSSSCG00000009405 | 11 | 19616499 | 19675791 | Backfat at tenth rib QTL (3657) | 11 | 4662540 | 40750599 |
| ENSSSCG00000009405 | 11 | 19616499 | 19675791 | Backfat at last rib QTL (3220) | 11 | 4662540 | 40750599 |
| ENSSSCG00000009405 | 11 | 19616499 | 19675791 | Loin muscle depth QTL (17807) | 11 | 13148362 | 41310104 |
| ENSSSCG00000009405 | 11 | 19616499 | 19675791 | Loin muscle area QTL (17808) | 11 | 13148362 | 41310104 |
| ENSSSCG00000009405 | 11 | 19616499 | 19675791 | Loin muscle depth QTL (17809) | 11 | 13148362 | 41310104 |
| ENSSSCG00000009405 | 11 | 19616499 | 19675791 | Interleukin-2 level QTL (17601) | 11 | 13148362 | 40750599 |
| ENSSSCG00000009405 | 11 | 19616499 | 19675791 | Interleukin-10 level QTL (17606) | 11 | 13148362 | 40750599 |
| ENSSSCG00000009405 | 11 | 19616499 | 19675791 | Interferon-gamma level QTL (17612) | 11 | 13148362 | 40750599 |
| ENSSSCG00000009405 | 11 | 19616499 | 19675791 | Toll-like receptor 2 level QTL (17615) | 11 | 13148362 | 40750599 |
| ENSSSCG00000009405 | 11 | 19616499 | 19675791 | Toll-like receptor 9 level QTL (17621) | 11 | 13148362 | 40750599 |
| ENSSSCG00000009405 | 11 | 19616499 | 19675791 | Toll-like receptor 9 level QTL (17622) | 11 | 13148362 | 40750599 |
| ENSSSCG00000009405 | 11 | 19616499 | 19675791 | Triglyceride level QTL (17722) | 11 | 13148362 | 40750599 |
| ENSSSCG00000009405 | 11 | 19616499 | 19675791 | Percentage type IIa fibers QTL (2816) | 11 | 13148362 | 40750599 |
| ENSSSCG00000009405 | 11 | 19616499 | 19675791 | Change in interferon-gamma level QTL (12342) | 11 | 13148362 | 40750599 |
| ENSSSCG00000009405 | 11 | 19616499 | 19675791 | Loin muscle depth QTL (3070) | 11 | 15479632 | 40750599 |
| ENSSSCG00000009405 | 11 | 19616499 | 19675791 | Loin meat weight QTL (3071) | 11 | 15479632 | 40750599 |
| ENSSSCG00000009405 | 11 | 19616499 | 19675791 | Lauric acid content QTL (156947) | 11 | 8392572 | 78050919 |
| ENSSSCG00000009405 | 11 | 19616499 | 19675791 | Oleic acid content QTL (156948) | 11 | 8392572 | 78050919 |
| ENSSSCG00000009405 | 11 | 19616499 | 19675791 | Monounsaturated fatty acid content QTL (156949) | 11 | 8392572 | 78050919 |
| ENSSSCG00000009405 | 11 | 19616499 | 19675791 | Loin muscle area QTL (38104) | 11 | 15479632 | 78050919 |
| ENSSSCG00000009405 | 11 | 19616499 | 19675791 | Meat color b* QTL (38105) | 11 | 15479632 | 78050919 |
| ENSSSCG00000009405 | 11 | 19616499 | 19675791 | Backfat at mid-back QTL (5214) | 11 | 15479632 | 72828874 |
| ENSSSCG00000009435 | 11 | 25528895 | 25595134 | Loin muscle area QTL (6) | 11 | 932508 | 27975240 |
| ENSSSCG00000009435 | 11 | 25528895 | 25595134 | Backfat thickness between 3rd and 4th rib QTL (425) | 11 | 4662540 | 43827876 |
| ENSSSCG00000009435 | 11 | 25528895 | 25595134 | Backfat at tenth rib QTL (3657) | 11 | 4662540 | 40750599 |
| ENSSSCG00000009435 | 11 | 25528895 | 25595134 | Backfat at last rib QTL (3220) | 11 | 4662540 | 40750599 |
| ENSSSCG00000009435 | 11 | 25528895 | 25595134 | Loin muscle depth QTL (17807) | 11 | 13148362 | 41310104 |
| ENSSSCG00000009435 | 11 | 25528895 | 25595134 | Loin muscle area QTL (17808) | 11 | 13148362 | 41310104 |
| ENSSSCG00000009435 | 11 | 25528895 | 25595134 | Loin muscle depth QTL (17809) | 11 | 13148362 | 41310104 |
| ENSSSCG00000009435 | 11 | 25528895 | 25595134 | Interleukin-2 level QTL (17601) | 11 | 13148362 | 40750599 |
| ENSSSCG00000009435 | 11 | 25528895 | 25595134 | Interleukin-10 level QTL (17606) | 11 | 13148362 | 40750599 |
| ENSSSCG00000009435 | 11 | 25528895 | 25595134 | Interferon-gamma level QTL (17612) | 11 | 13148362 | 40750599 |
| ENSSSCG00000009435 | 11 | 25528895 | 25595134 | Toll-like receptor 2 level QTL (17615) | 11 | 13148362 | 40750599 |
| ENSSSCG00000009435 | 11 | 25528895 | 25595134 | Toll-like receptor 9 level QTL (17621) | 11 | 13148362 | 40750599 |
| ENSSSCG00000009435 | 11 | 25528895 | 25595134 | Toll-like receptor 9 level QTL (17622) | 11 | 13148362 | 40750599 |
| ENSSSCG00000009435 | 11 | 25528895 | 25595134 | Triglyceride level QTL (17722) | 11 | 13148362 | 40750599 |
| ENSSSCG00000009435 | 11 | 25528895 | 25595134 | Percentage type IIa fibers QTL (2816) | 11 | 13148362 | 40750599 |
| ENSSSCG00000009435 | 11 | 25528895 | 25595134 | Change in interferon-gamma level QTL (12342) | 11 | 13148362 | 40750599 |
| ENSSSCG00000009435 | 11 | 25528895 | 25595134 | Loin muscle depth QTL (3070) | 11 | 15479632 | 40750599 |
| ENSSSCG00000009435 | 11 | 25528895 | 25595134 | Loin meat weight QTL (3071) | 11 | 15479632 | 40750599 |
| ENSSSCG00000009435 | 11 | 25528895 | 25595134 | Lauric acid content QTL (156947) | 11 | 8392572 | 78050919 |
| ENSSSCG00000009435 | 11 | 25528895 | 25595134 | Oleic acid content QTL (156948) | 11 | 8392572 | 78050919 |
| ENSSSCG00000009435 | 11 | 25528895 | 25595134 | Monounsaturated fatty acid content QTL (156949) | 11 | 8392572 | 78050919 |
| ENSSSCG00000009435 | 11 | 25528895 | 25595134 | Loin muscle area QTL (38104) | 11 | 15479632 | 78050919 |
| ENSSSCG00000009435 | 11 | 25528895 | 25595134 | Meat color b* QTL (38105) | 11 | 15479632 | 78050919 |
| ENSSSCG00000009435 | 11 | 25528895 | 25595134 | Backfat at mid-back QTL (5214) | 11 | 15479632 | 72828874 |
| ENSSSCG00000009464 | 11 | 47543814 | 47818561 | Interferon-gamma level QTL (12340) | 11 | 47669808 | 47753734 |
| ENSSSCG00000009464 | 11 | 47543814 | 47818561 | Interferon-gamma level QTL (17613) | 11 | 40750599 | 56230232 |
| ENSSSCG00000009464 | 11 | 47543814 | 47818561 | Percentage angular fibers QTL (2817) | 11 | 40750599 | 56230232 |
| ENSSSCG00000009464 | 11 | 47543814 | 47818561 | Haptoglobin concentration QTL (9649) | 11 | 40750599 | 56230232 |
| ENSSSCG00000009464 | 11 | 47543814 | 47818561 | body weight (19 weeks) QTL (3209) | 11 | 40750599 | 52593451 |
| ENSSSCG00000009464 | 11 | 47543814 | 47818561 | Backfat at last rib QTL (3210) | 11 | 40750599 | 52593451 |
| ENSSSCG00000009464 | 11 | 47543814 | 47818561 | Interferon-gamma level QTL (12267) | 11 | 41310104 | 52593451 |
| ENSSSCG00000009464 | 11 | 47543814 | 47818561 | Interferon-gamma to interleukin-10 ratio QTL (12271) | 11 | 41310104 | 52593451 |
| ENSSSCG00000009464 | 11 | 47543814 | 47818561 | Lauric acid content QTL (156947) | 11 | 8392572 | 78050919 |
| ENSSSCG00000009464 | 11 | 47543814 | 47818561 | Oleic acid content QTL (156948) | 11 | 8392572 | 78050919 |
| ENSSSCG00000009464 | 11 | 47543814 | 47818561 | Monounsaturated fatty acid content QTL (156949) | 11 | 8392572 | 78050919 |
| ENSSSCG00000009464 | 11 | 47543814 | 47818561 | Loin muscle area QTL (38104) | 11 | 15479632 | 78050919 |
| ENSSSCG00000009464 | 11 | 47543814 | 47818561 | Meat color b* QTL (38105) | 11 | 15479632 | 78050919 |
| ENSSSCG00000009464 | 11 | 47543814 | 47818561 | Backfat at mid-back QTL (5214) | 11 | 15479632 | 72828874 |
| ENSSSCG00000009464 | 11 | 47543814 | 47818561 | Meat color score QTL (2954) | 11 | 40750599 | 75066894 |
| ENSSSCG00000009464 | 11 | 47543814 | 47818561 | Drip loss QTL (2955) | 11 | 40750599 | 75066894 |
| ENSSSCG00000009464 | 11 | 47543814 | 47818561 | Percentage type I fibers QTL (7027) | 11 | 40750599 | 71057109 |
| ENSSSCG00000040184 | 11 | 47911096 | 48133446 | Interferon-gamma level QTL (17613) | 11 | 40750599 | 56230232 |
| ENSSSCG00000040184 | 11 | 47911096 | 48133446 | Percentage angular fibers QTL (2817) | 11 | 40750599 | 56230232 |
| ENSSSCG00000040184 | 11 | 47911096 | 48133446 | Haptoglobin concentration QTL (9649) | 11 | 40750599 | 56230232 |
| ENSSSCG00000040184 | 11 | 47911096 | 48133446 | body weight (19 weeks) QTL (3209) | 11 | 40750599 | 52593451 |
| ENSSSCG00000040184 | 11 | 47911096 | 48133446 | Backfat at last rib QTL (3210) | 11 | 40750599 | 52593451 |
| ENSSSCG00000040184 | 11 | 47911096 | 48133446 | Interferon-gamma level QTL (12267) | 11 | 41310104 | 52593451 |
| ENSSSCG00000040184 | 11 | 47911096 | 48133446 | Interferon-gamma to interleukin-10 ratio QTL (12271) | 11 | 41310104 | 52593451 |
| ENSSSCG00000040184 | 11 | 47911096 | 48133446 | Lauric acid content QTL (156947) | 11 | 8392572 | 78050919 |
| ENSSSCG00000040184 | 11 | 47911096 | 48133446 | Oleic acid content QTL (156948) | 11 | 8392572 | 78050919 |
| ENSSSCG00000040184 | 11 | 47911096 | 48133446 | Monounsaturated fatty acid content QTL (156949) | 11 | 8392572 | 78050919 |
| ENSSSCG00000040184 | 11 | 47911096 | 48133446 | Loin muscle area QTL (38104) | 11 | 15479632 | 78050919 |
| ENSSSCG00000040184 | 11 | 47911096 | 48133446 | Meat color b* QTL (38105) | 11 | 15479632 | 78050919 |
| ENSSSCG00000040184 | 11 | 47911096 | 48133446 | Backfat at mid-back QTL (5214) | 11 | 15479632 | 72828874 |
| ENSSSCG00000040184 | 11 | 47911096 | 48133446 | Meat color score QTL (2954) | 11 | 40750599 | 75066894 |
| ENSSSCG00000040184 | 11 | 47911096 | 48133446 | Drip loss QTL (2955) | 11 | 40750599 | 75066894 |
| ENSSSCG00000040184 | 11 | 47911096 | 48133446 | Percentage type I fibers QTL (7027) | 11 | 40750599 | 71057109 |
| ENSSSCG00000017198 | 12 | 5428360 | 5479544 | Triglyceride level QTL (15076) | 12 | 3594855 | 61602742 |
| ENSSSCG00000017198 | 12 | 5428360 | 5479544 | Muscle moisture percentage QTL (38106) | 12 | 3594855 | 56101525 |
| ENSSSCG00000017198 | 12 | 5428360 | 5479544 | Backfat at last rib QTL (16839) | 12 | 3594855 | 43682935 |
| ENSSSCG00000017198 | 12 | 5428360 | 5479544 | HDL cholesterol QTL (15114) | 12 | 3594855 | 38399587 |
| ENSSSCG00000017198 | 12 | 5428360 | 5479544 | Cholesterol level QTL (17723) | 12 | 3594855 | 35240472 |
| ENSSSCG00000017198 | 12 | 5428360 | 5479544 | LDL cholesterol QTL (17724) | 12 | 3594855 | 35240472 |
| ENSSSCG00000017198 | 12 | 5428360 | 5479544 | Carcass length QTL (5716) | 12 | 3594855 | 35240472 |
| ENSSSCG00000017198 | 12 | 5428360 | 5479544 | Linolenic acid content QTL (473) | 12 | 3594855 | 34205590 |
| ENSSSCG00000017198 | 12 | 5428360 | 5479544 | pH 24 hr post-mortem (loin) QTL (21860) | 12 | 3594855 | 18192145 |
| ENSSSCG00000017198 | 12 | 5428360 | 5479544 | Myristic acid content QTL (9570) | 12 | 3594855 | 18192145 |
| ENSSSCG00000017198 | 12 | 5428360 | 5479544 | Myristic acid content QTL (9571) | 12 | 3594855 | 18192145 |
| ENSSSCG00000017505 | 12 | 22839005 | 22872407 | Calcium level QTL (103462) | 12 | 22868278 | 22868282 |
| ENSSSCG00000017505 | 12 | 22839005 | 22872407 | Triglyceride level QTL (15076) | 12 | 3594855 | 61602742 |
| ENSSSCG00000017505 | 12 | 22839005 | 22872407 | Muscle moisture percentage QTL (38106) | 12 | 3594855 | 56101525 |
| ENSSSCG00000017505 | 12 | 22839005 | 22872407 | Backfat at last rib QTL (16839) | 12 | 3594855 | 43682935 |
| ENSSSCG00000017505 | 12 | 22839005 | 22872407 | HDL cholesterol QTL (15114) | 12 | 3594855 | 38399587 |
| ENSSSCG00000017505 | 12 | 22839005 | 22872407 | Cholesterol level QTL (17723) | 12 | 3594855 | 35240472 |
| ENSSSCG00000017505 | 12 | 22839005 | 22872407 | LDL cholesterol QTL (17724) | 12 | 3594855 | 35240472 |
| ENSSSCG00000017505 | 12 | 22839005 | 22872407 | Carcass length QTL (5716) | 12 | 3594855 | 35240472 |
| ENSSSCG00000017505 | 12 | 22839005 | 22872407 | Linolenic acid content QTL (473) | 12 | 3594855 | 34205590 |
| ENSSSCG00000017505 | 12 | 22839005 | 22872407 | Backfat at last rib QTL (1121) | 12 | 9096072 | 35240472 |
| ENSSSCG00000017505 | 12 | 22839005 | 22872407 | Backfat at last rib QTL (1124) | 12 | 9096072 | 35240472 |
| ENSSSCG00000017505 | 12 | 22839005 | 22872407 | body weight (10 weeks) QTL (29707) | 12 | 10512227 | 61602742 |
| ENSSSCG00000017505 | 12 | 22839005 | 22872407 | Leaf fat weight QTL (21264) | 12 | 11492642 | 51853060 |
| ENSSSCG00000017505 | 12 | 22839005 | 22872407 | Average backfat thickness QTL (4197) | 12 | 15414302 | 24891647 |
| ENSSSCG00000017505 | 12 | 22839005 | 22872407 | Gadoleic acid content QTL (156898) | 12 | 18192145 | 48203737 |
| ENSSSCG00000017505 | 12 | 22839005 | 22872407 | Marbling QTL (21399) | 12 | 18192145 | 35240472 |
| ENSSSCG00000017505 | 12 | 22839005 | 22872407 | Loin fat percentage QTL (21435) | 12 | 18192145 | 35240472 |
| ENSSSCG00000017505 | 12 | 22839005 | 22872407 | Backfat at tenth rib QTL (1122) | 12 | 21024455 | 35240472 |
| ENSSSCG00000017505 | 12 | 22839005 | 22872407 | backfat between the last 3rd and 4th lumbar QTL (3967) | 12 | 21895935 | 35240472 |
| ENSSSCG00000017505 | 12 | 22839005 | 22872407 | Percentage type IIa fibers QTL (7033) | 12 | 21895935 | 35240472 |
| ENSSSCG00000017505 | 12 | 22839005 | 22872407 | Fat area percentage in carcass QTL (18639) | 12 | 21895935 | 35240472 |
| ENSSSCG00000017513 | 12 | 23799430 | 23892051 | Triglyceride level QTL (15076) | 12 | 3594855 | 61602742 |
| ENSSSCG00000017513 | 12 | 23799430 | 23892051 | Muscle moisture percentage QTL (38106) | 12 | 3594855 | 56101525 |
| ENSSSCG00000017513 | 12 | 23799430 | 23892051 | Backfat at last rib QTL (16839) | 12 | 3594855 | 43682935 |
| ENSSSCG00000017513 | 12 | 23799430 | 23892051 | HDL cholesterol QTL (15114) | 12 | 3594855 | 38399587 |
| ENSSSCG00000017513 | 12 | 23799430 | 23892051 | Cholesterol level QTL (17723) | 12 | 3594855 | 35240472 |
| ENSSSCG00000017513 | 12 | 23799430 | 23892051 | LDL cholesterol QTL (17724) | 12 | 3594855 | 35240472 |
| ENSSSCG00000017513 | 12 | 23799430 | 23892051 | Carcass length QTL (5716) | 12 | 3594855 | 35240472 |
| ENSSSCG00000017513 | 12 | 23799430 | 23892051 | Linolenic acid content QTL (473) | 12 | 3594855 | 34205590 |
| ENSSSCG00000017513 | 12 | 23799430 | 23892051 | Backfat at last rib QTL (1121) | 12 | 9096072 | 35240472 |
| ENSSSCG00000017513 | 12 | 23799430 | 23892051 | Backfat at last rib QTL (1124) | 12 | 9096072 | 35240472 |
| ENSSSCG00000017513 | 12 | 23799430 | 23892051 | body weight (10 weeks) QTL (29707) | 12 | 10512227 | 61602742 |
| ENSSSCG00000017513 | 12 | 23799430 | 23892051 | Leaf fat weight QTL (21264) | 12 | 11492642 | 51853060 |
| ENSSSCG00000017513 | 12 | 23799430 | 23892051 | Average backfat thickness QTL (4197) | 12 | 15414302 | 24891647 |
| ENSSSCG00000017513 | 12 | 23799430 | 23892051 | Gadoleic acid content QTL (156898) | 12 | 18192145 | 48203737 |
| ENSSSCG00000017513 | 12 | 23799430 | 23892051 | Marbling QTL (21399) | 12 | 18192145 | 35240472 |
| ENSSSCG00000017513 | 12 | 23799430 | 23892051 | Loin fat percentage QTL (21435) | 12 | 18192145 | 35240472 |
| ENSSSCG00000017513 | 12 | 23799430 | 23892051 | Backfat at tenth rib QTL (1122) | 12 | 21024455 | 35240472 |
| ENSSSCG00000017513 | 12 | 23799430 | 23892051 | backfat between the last 3rd and 4th lumbar QTL (3967) | 12 | 21895935 | 35240472 |
| ENSSSCG00000017513 | 12 | 23799430 | 23892051 | Percentage type IIa fibers QTL (7033) | 12 | 21895935 | 35240472 |
| ENSSSCG00000017513 | 12 | 23799430 | 23892051 | Fat area percentage in carcass QTL (18639) | 12 | 21895935 | 35240472 |
| ENSSSCG00000017668 | 12 | 35933192 | 36065956 | Cooling loss QTL (3072) | 12 | 35240472 | 38399587 |
| ENSSSCG00000017668 | 12 | 35933192 | 36065956 | Triglyceride level QTL (15076) | 12 | 3594855 | 61602742 |
| ENSSSCG00000017668 | 12 | 35933192 | 36065956 | Muscle moisture percentage QTL (38106) | 12 | 3594855 | 56101525 |
| ENSSSCG00000017668 | 12 | 35933192 | 36065956 | Backfat at last rib QTL (16839) | 12 | 3594855 | 43682935 |
| ENSSSCG00000017668 | 12 | 35933192 | 36065956 | HDL cholesterol QTL (15114) | 12 | 3594855 | 38399587 |
| ENSSSCG00000017668 | 12 | 35933192 | 36065956 | body weight (10 weeks) QTL (29707) | 12 | 10512227 | 61602742 |
| ENSSSCG00000017668 | 12 | 35933192 | 36065956 | Leaf fat weight QTL (21264) | 12 | 11492642 | 51853060 |
| ENSSSCG00000017668 | 12 | 35933192 | 36065956 | Gadoleic acid content QTL (156898) | 12 | 18192145 | 48203737 |
| ENSSSCG00000017668 | 12 | 35933192 | 36065956 | Muscle moisture percentage QTL (21718) | 12 | 25055050 | 40305950 |
| ENSSSCG00000017668 | 12 | 35933192 | 36065956 | pH 45 minutes post mortem QTL (9844) | 12 | 35240472 | 58988302 |
| ENSSSCG00000017668 | 12 | 35933192 | 36065956 | pH 24 hr post-mortem (loin) QTL (9845) | 12 | 35240472 | 58988302 |
| ENSSSCG00000017668 | 12 | 35933192 | 36065956 | Drip loss QTL (9846) | 12 | 35240472 | 58988302 |
| ENSSSCG00000017668 | 12 | 35933192 | 36065956 | Conductivity 24 hours postmortem (loin) QTL (12115) | 12 | 35240472 | 58988302 |
| ENSSSCG00000017668 | 12 | 35933192 | 36065956 | Loin muscle depth QTL (12116) | 12 | 35240472 | 58988302 |
| ENSSSCG00000017668 | 12 | 35933192 | 36065956 | Interleukin-10 level QTL (17608) | 12 | 35240472 | 58988302 |
| ENSSSCG00000017668 | 12 | 35933192 | 36065956 | Toll-like receptor 2 level QTL (17616) | 12 | 35240472 | 58988302 |
| ENSSSCG00000017668 | 12 | 35933192 | 36065956 | Toll-like receptor 9 level QTL (17625) | 12 | 35240472 | 58988302 |
| ENSSSCG00000017668 | 12 | 35933192 | 36065956 | Average backfat thickness QTL (5990) | 12 | 35240472 | 58988302 |
| ENSSSCG00000017668 | 12 | 35933192 | 36065956 | Number of muscle fibers per unit area QTL (2818) | 12 | 35240472 | 58988302 |
| ENSSSCG00000017668 | 12 | 35933192 | 36065956 | Diameter of type I muscle fibers QTL (2819) | 12 | 35240472 | 58988302 |
| ENSSSCG00000017668 | 12 | 35933192 | 36065956 | Total muscle fiber number QTL (2820) | 12 | 35240472 | 58988302 |
| ENSSSCG00000017668 | 12 | 35933192 | 36065956 | Diameter of type I muscle fibers QTL (2821) | 12 | 35240472 | 58988302 |
| ENSSSCG00000017668 | 12 | 35933192 | 36065956 | Loin muscle area QTL (3085) | 12 | 35240472 | 58988302 |
| ENSSSCG00000017668 | 12 | 35933192 | 36065956 | Carcass weight (hot) QTL (16872) | 12 | 35240472 | 52179865 |
| ENSSSCG00000017668 | 12 | 35933192 | 36065956 | Creatine kinase level QTL (1123) | 12 | 35240472 | 43682935 |
| ENSSSCG00000017668 | 12 | 35933192 | 36065956 | Muscle moisture percentage QTL (3285) | 12 | 35240472 | 43682935 |
| ENSSSCG00000017668 | 12 | 35933192 | 36065956 | Muscle fat content QTL (3286) | 12 | 35240472 | 43682935 |
| ENSSSCG00000017668 | 12 | 35933192 | 36065956 | pH 24 hr post-mortem (loin) QTL (3287) | 12 | 35240472 | 43682935 |
| ENSSSCG00000017668 | 12 | 35933192 | 36065956 | Meat color-L QTL (2956) | 12 | 35240472 | 43682935 |
| ENSSSCG00000017748 | 12 | 43489171 | 43754176 | Triglyceride level QTL (15076) | 12 | 3594855 | 61602742 |
| ENSSSCG00000017748 | 12 | 43489171 | 43754176 | Muscle moisture percentage QTL (38106) | 12 | 3594855 | 56101525 |
| ENSSSCG00000017748 | 12 | 43489171 | 43754176 | Backfat at last rib QTL (16839) | 12 | 3594855 | 43682935 |
| ENSSSCG00000017748 | 12 | 43489171 | 43754176 | body weight (10 weeks) QTL (29707) | 12 | 10512227 | 61602742 |
| ENSSSCG00000017748 | 12 | 43489171 | 43754176 | Leaf fat weight QTL (21264) | 12 | 11492642 | 51853060 |
| ENSSSCG00000017748 | 12 | 43489171 | 43754176 | Gadoleic acid content QTL (156898) | 12 | 18192145 | 48203737 |
| ENSSSCG00000017748 | 12 | 43489171 | 43754176 | pH 45 minutes post mortem QTL (9844) | 12 | 35240472 | 58988302 |
| ENSSSCG00000017748 | 12 | 43489171 | 43754176 | pH 24 hr post-mortem (loin) QTL (9845) | 12 | 35240472 | 58988302 |
| ENSSSCG00000017748 | 12 | 43489171 | 43754176 | Drip loss QTL (9846) | 12 | 35240472 | 58988302 |
| ENSSSCG00000017748 | 12 | 43489171 | 43754176 | Conductivity 24 hours postmortem (loin) QTL (12115) | 12 | 35240472 | 58988302 |
| ENSSSCG00000017748 | 12 | 43489171 | 43754176 | Loin muscle depth QTL (12116) | 12 | 35240472 | 58988302 |
| ENSSSCG00000017748 | 12 | 43489171 | 43754176 | Interleukin-10 level QTL (17608) | 12 | 35240472 | 58988302 |
| ENSSSCG00000017748 | 12 | 43489171 | 43754176 | Toll-like receptor 2 level QTL (17616) | 12 | 35240472 | 58988302 |
| ENSSSCG00000017748 | 12 | 43489171 | 43754176 | Toll-like receptor 9 level QTL (17625) | 12 | 35240472 | 58988302 |
| ENSSSCG00000017748 | 12 | 43489171 | 43754176 | Average backfat thickness QTL (5990) | 12 | 35240472 | 58988302 |
| ENSSSCG00000017748 | 12 | 43489171 | 43754176 | Number of muscle fibers per unit area QTL (2818) | 12 | 35240472 | 58988302 |
| ENSSSCG00000017748 | 12 | 43489171 | 43754176 | Diameter of type I muscle fibers QTL (2819) | 12 | 35240472 | 58988302 |
| ENSSSCG00000017748 | 12 | 43489171 | 43754176 | Total muscle fiber number QTL (2820) | 12 | 35240472 | 58988302 |
| ENSSSCG00000017748 | 12 | 43489171 | 43754176 | Diameter of type I muscle fibers QTL (2821) | 12 | 35240472 | 58988302 |
| ENSSSCG00000017748 | 12 | 43489171 | 43754176 | Loin muscle area QTL (3085) | 12 | 35240472 | 58988302 |
| ENSSSCG00000017748 | 12 | 43489171 | 43754176 | Carcass weight (hot) QTL (16872) | 12 | 35240472 | 52179865 |
| ENSSSCG00000017748 | 12 | 43489171 | 43754176 | Creatine kinase level QTL (1123) | 12 | 35240472 | 43682935 |
| ENSSSCG00000017748 | 12 | 43489171 | 43754176 | Muscle moisture percentage QTL (3285) | 12 | 35240472 | 43682935 |
| ENSSSCG00000017748 | 12 | 43489171 | 43754176 | Muscle fat content QTL (3286) | 12 | 35240472 | 43682935 |
| ENSSSCG00000017748 | 12 | 43489171 | 43754176 | pH 24 hr post-mortem (loin) QTL (3287) | 12 | 35240472 | 43682935 |
| ENSSSCG00000017748 | 12 | 43489171 | 43754176 | Meat color-L QTL (2956) | 12 | 35240472 | 43682935 |
| ENSSSCG00000017748 | 12 | 43489171 | 43754176 | Body weight (20 weeks) QTL (29708) | 12 | 38399587 | 56101525 |
| ENSSSCG00000017748 | 12 | 43489171 | 43754176 | Muscle moisture percentage QTL (21401) | 12 | 38399587 | 43682935 |
| ENSSSCG00000017748 | 12 | 43489171 | 43754176 | Meat color a* QTL (4003) | 12 | 38399587 | 43682935 |
| ENSSSCG00000017812 | 12 | 47125248 | 47291169 | Meat color-L QTL (2958) | 12 | 43682935 | 48966282 |
| ENSSSCG00000017812 | 12 | 47125248 | 47291169 | Triglyceride level QTL (15076) | 12 | 3594855 | 61602742 |
| ENSSSCG00000017812 | 12 | 47125248 | 47291169 | Muscle moisture percentage QTL (38106) | 12 | 3594855 | 56101525 |
| ENSSSCG00000017812 | 12 | 47125248 | 47291169 | body weight (10 weeks) QTL (29707) | 12 | 10512227 | 61602742 |
| ENSSSCG00000017812 | 12 | 47125248 | 47291169 | Leaf fat weight QTL (21264) | 12 | 11492642 | 51853060 |
| ENSSSCG00000017812 | 12 | 47125248 | 47291169 | Gadoleic acid content QTL (156898) | 12 | 18192145 | 48203737 |
| ENSSSCG00000017812 | 12 | 47125248 | 47291169 | pH 45 minutes post mortem QTL (9844) | 12 | 35240472 | 58988302 |
| ENSSSCG00000017812 | 12 | 47125248 | 47291169 | pH 24 hr post-mortem (loin) QTL (9845) | 12 | 35240472 | 58988302 |
| ENSSSCG00000017812 | 12 | 47125248 | 47291169 | Drip loss QTL (9846) | 12 | 35240472 | 58988302 |
| ENSSSCG00000017812 | 12 | 47125248 | 47291169 | Conductivity 24 hours postmortem (loin) QTL (12115) | 12 | 35240472 | 58988302 |
| ENSSSCG00000017812 | 12 | 47125248 | 47291169 | Loin muscle depth QTL (12116) | 12 | 35240472 | 58988302 |
| ENSSSCG00000017812 | 12 | 47125248 | 47291169 | Interleukin-10 level QTL (17608) | 12 | 35240472 | 58988302 |
| ENSSSCG00000017812 | 12 | 47125248 | 47291169 | Toll-like receptor 2 level QTL (17616) | 12 | 35240472 | 58988302 |
| ENSSSCG00000017812 | 12 | 47125248 | 47291169 | Toll-like receptor 9 level QTL (17625) | 12 | 35240472 | 58988302 |
| ENSSSCG00000017812 | 12 | 47125248 | 47291169 | Average backfat thickness QTL (5990) | 12 | 35240472 | 58988302 |
| ENSSSCG00000017812 | 12 | 47125248 | 47291169 | Number of muscle fibers per unit area QTL (2818) | 12 | 35240472 | 58988302 |
| ENSSSCG00000017812 | 12 | 47125248 | 47291169 | Diameter of type I muscle fibers QTL (2819) | 12 | 35240472 | 58988302 |
| ENSSSCG00000017812 | 12 | 47125248 | 47291169 | Total muscle fiber number QTL (2820) | 12 | 35240472 | 58988302 |
| ENSSSCG00000017812 | 12 | 47125248 | 47291169 | Diameter of type I muscle fibers QTL (2821) | 12 | 35240472 | 58988302 |
| ENSSSCG00000017812 | 12 | 47125248 | 47291169 | Loin muscle area QTL (3085) | 12 | 35240472 | 58988302 |
| ENSSSCG00000017812 | 12 | 47125248 | 47291169 | Carcass weight (hot) QTL (16872) | 12 | 35240472 | 52179865 |
| ENSSSCG00000017812 | 12 | 47125248 | 47291169 | Body weight (20 weeks) QTL (29708) | 12 | 38399587 | 56101525 |
| ENSSSCG00000017812 | 12 | 47125248 | 47291169 | Marbling QTL (3288) | 12 | 43682935 | 57408745 |
| ENSSSCG00000017812 | 12 | 47125248 | 47291169 | Average daily gain QTL (335) | 12 | 43682935 | 52179865 |
| ENSSSCG00000017812 | 12 | 47125248 | 47291169 | Meat color score QTL (112) | 12 | 45861635 | 52179865 |
| ENSSSCG00000017877 | 12 | 50167957 | 50348689 | Fat androstenone level QTL (194759) | 12 | 50190725 | 50190729 |
| ENSSSCG00000017877 | 12 | 50167957 | 50348689 | Marbling QTL (37703) | 12 | 50305228 | 50305232 |
| ENSSSCG00000017877 | 12 | 50167957 | 50348689 | Triglyceride level QTL (15076) | 12 | 3594855 | 61602742 |
| ENSSSCG00000017877 | 12 | 50167957 | 50348689 | Muscle moisture percentage QTL (38106) | 12 | 3594855 | 56101525 |
| ENSSSCG00000017877 | 12 | 50167957 | 50348689 | body weight (10 weeks) QTL (29707) | 12 | 10512227 | 61602742 |
| ENSSSCG00000017877 | 12 | 50167957 | 50348689 | Leaf fat weight QTL (21264) | 12 | 11492642 | 51853060 |
| ENSSSCG00000017877 | 12 | 50167957 | 50348689 | pH 45 minutes post mortem QTL (9844) | 12 | 35240472 | 58988302 |
| ENSSSCG00000017877 | 12 | 50167957 | 50348689 | pH 24 hr post-mortem (loin) QTL (9845) | 12 | 35240472 | 58988302 |
| ENSSSCG00000017877 | 12 | 50167957 | 50348689 | Drip loss QTL (9846) | 12 | 35240472 | 58988302 |
| ENSSSCG00000017877 | 12 | 50167957 | 50348689 | Conductivity 24 hours postmortem (loin) QTL (12115) | 12 | 35240472 | 58988302 |
| ENSSSCG00000017877 | 12 | 50167957 | 50348689 | Loin muscle depth QTL (12116) | 12 | 35240472 | 58988302 |
| ENSSSCG00000017877 | 12 | 50167957 | 50348689 | Interleukin-10 level QTL (17608) | 12 | 35240472 | 58988302 |
| ENSSSCG00000017877 | 12 | 50167957 | 50348689 | Toll-like receptor 2 level QTL (17616) | 12 | 35240472 | 58988302 |
| ENSSSCG00000017877 | 12 | 50167957 | 50348689 | Toll-like receptor 9 level QTL (17625) | 12 | 35240472 | 58988302 |
| ENSSSCG00000017877 | 12 | 50167957 | 50348689 | Average backfat thickness QTL (5990) | 12 | 35240472 | 58988302 |
| ENSSSCG00000017877 | 12 | 50167957 | 50348689 | Number of muscle fibers per unit area QTL (2818) | 12 | 35240472 | 58988302 |
| ENSSSCG00000017877 | 12 | 50167957 | 50348689 | Diameter of type I muscle fibers QTL (2819) | 12 | 35240472 | 58988302 |
| ENSSSCG00000017877 | 12 | 50167957 | 50348689 | Total muscle fiber number QTL (2820) | 12 | 35240472 | 58988302 |
| ENSSSCG00000017877 | 12 | 50167957 | 50348689 | Diameter of type I muscle fibers QTL (2821) | 12 | 35240472 | 58988302 |
| ENSSSCG00000017877 | 12 | 50167957 | 50348689 | Loin muscle area QTL (3085) | 12 | 35240472 | 58988302 |
| ENSSSCG00000017877 | 12 | 50167957 | 50348689 | Carcass weight (hot) QTL (16872) | 12 | 35240472 | 52179865 |
| ENSSSCG00000017877 | 12 | 50167957 | 50348689 | Body weight (20 weeks) QTL (29708) | 12 | 38399587 | 56101525 |
| ENSSSCG00000017877 | 12 | 50167957 | 50348689 | Marbling QTL (3288) | 12 | 43682935 | 57408745 |
| ENSSSCG00000017877 | 12 | 50167957 | 50348689 | Average daily gain QTL (335) | 12 | 43682935 | 52179865 |
| ENSSSCG00000017877 | 12 | 50167957 | 50348689 | Meat color score QTL (112) | 12 | 45861635 | 52179865 |
| ENSSSCG00000017877 | 12 | 50167957 | 50348689 | Creatinine level QTL (15075) | 12 | 48203737 | 61602742 |
| ENSSSCG00000017877 | 12 | 50167957 | 50348689 | Meat color chroma QTL (38066) | 12 | 48203737 | 61602742 |
| ENSSSCG00000017877 | 12 | 50167957 | 50348689 | Carcass weight (hot) QTL (29709) | 12 | 48203737 | 61602742 |
| ENSSSCG00000017877 | 12 | 50167957 | 50348689 | Capric acid content QTL (156889) | 12 | 48203737 | 61602742 |
| ENSSSCG00000017877 | 12 | 50167957 | 50348689 | Stearic acid content QTL (156893) | 12 | 48203737 | 61602742 |
| ENSSSCG00000017877 | 12 | 50167957 | 50348689 | Linolenic acid content QTL (156896) | 12 | 48203737 | 61602742 |
| ENSSSCG00000017877 | 12 | 50167957 | 50348689 | Unsaturated fatty acid content QTL (156900) | 12 | 48203737 | 61602742 |
| ENSSSCG00000017877 | 12 | 50167957 | 50348689 | Meat color-L QTL (2957) | 12 | 48966282 | 52179865 |
| ENSSSCG00000017877 | 12 | 50167957 | 50348689 | Meat color-L QTL (2959) | 12 | 48966282 | 52179865 |
| ENSSSCG00000017877 | 12 | 50167957 | 50348689 | Oleic acid content QTL (7680) | 12 | 50235375 | 50376990 |
| ENSSSCG00000050244 | 12 | 59273169 | 59353895 | Triglyceride level QTL (15076) | 12 | 3594855 | 61602742 |
| ENSSSCG00000050244 | 12 | 59273169 | 59353895 | body weight (10 weeks) QTL (29707) | 12 | 10512227 | 61602742 |
| ENSSSCG00000050244 | 12 | 59273169 | 59353895 | Creatinine level QTL (15075) | 12 | 48203737 | 61602742 |
| ENSSSCG00000050244 | 12 | 59273169 | 59353895 | Meat color chroma QTL (38066) | 12 | 48203737 | 61602742 |
| ENSSSCG00000050244 | 12 | 59273169 | 59353895 | Carcass weight (hot) QTL (29709) | 12 | 48203737 | 61602742 |
| ENSSSCG00000050244 | 12 | 59273169 | 59353895 | Capric acid content QTL (156889) | 12 | 48203737 | 61602742 |
| ENSSSCG00000050244 | 12 | 59273169 | 59353895 | Stearic acid content QTL (156893) | 12 | 48203737 | 61602742 |
| ENSSSCG00000050244 | 12 | 59273169 | 59353895 | Linolenic acid content QTL (156896) | 12 | 48203737 | 61602742 |
| ENSSSCG00000050244 | 12 | 59273169 | 59353895 | Unsaturated fatty acid content QTL (156900) | 12 | 48203737 | 61602742 |
| ENSSSCG00000050244 | 12 | 59273169 | 59353895 | Palmitoleic acid content QTL (156950) | 12 | 52179865 | 61602742 |
| ENSSSCG00000050244 | 12 | 59273169 | 59353895 | Capric acid content QTL (156888) | 12 | 52179865 | 61602742 |
| ENSSSCG00000050244 | 12 | 59273169 | 59353895 | Palmitic acid content QTL (156890) | 12 | 52179865 | 61602742 |
| ENSSSCG00000050244 | 12 | 59273169 | 59353895 | Margaric acid content QTL (156891) | 12 | 52179865 | 61602742 |
| ENSSSCG00000050244 | 12 | 59273169 | 59353895 | cis-9-Heptadecenoic acid content QTL (156892) | 12 | 52179865 | 61602742 |
| ENSSSCG00000050244 | 12 | 59273169 | 59353895 | Oleic acid content QTL (156894) | 12 | 52179865 | 61602742 |
| ENSSSCG00000050244 | 12 | 59273169 | 59353895 | Linoleic acid content QTL (156895) | 12 | 52179865 | 61602742 |
| ENSSSCG00000050244 | 12 | 59273169 | 59353895 | Arachidic acid content QTL (156897) | 12 | 52179865 | 61602742 |
| ENSSSCG00000050244 | 12 | 59273169 | 59353895 | Arachidonic acid content QTL (156899) | 12 | 52179865 | 61602742 |
| ENSSSCG00000050244 | 12 | 59273169 | 59353895 | Monounsaturated fatty acid content QTL (156901) | 12 | 52179865 | 61602742 |
| ENSSSCG00000050244 | 12 | 59273169 | 59353895 | Polyunsaturated fatty acid content QTL (156902) | 12 | 52179865 | 61602742 |
| ENSSSCG00000050244 | 12 | 59273169 | 59353895 | Loin muscle area QTL (38057) | 12 | 52179865 | 61602742 |
| ENSSSCG00000050244 | 12 | 59273169 | 59353895 | Loin muscle area QTL (38058) | 12 | 52179865 | 61602742 |
| ENSSSCG00000050244 | 12 | 59273169 | 59353895 | Marbling QTL (38059) | 12 | 52179865 | 61602742 |
| ENSSSCG00000050244 | 12 | 59273169 | 59353895 | Loin fat percentage QTL (38060) | 12 | 52179865 | 61602742 |
| ENSSSCG00000050244 | 12 | 59273169 | 59353895 | Shear force QTL (38061) | 12 | 52179865 | 61602742 |
| ENSSSCG00000050244 | 12 | 59273169 | 59353895 | Drip loss QTL (38062) | 12 | 52179865 | 61602742 |
| ENSSSCG00000050244 | 12 | 59273169 | 59353895 | Meat color a* QTL (38064) | 12 | 52179865 | 61602742 |
| ENSSSCG00000050244 | 12 | 59273169 | 59353895 | Percentage type IIb fibers QTL (7021) | 12 | 52179865 | 61602742 |
| ENSSSCG00000050244 | 12 | 59273169 | 59353895 | Percentage type IIb fibers QTL (7036) | 12 | 52179865 | 61602742 |
| ENSSSCG00000050244 | 12 | 59273169 | 59353895 | Adipocyte diameter QTL (12844) | 12 | 52179865 | 61602742 |
| ENSSSCG00000034364 | 12 | 59466791 | 59654130 | Triglyceride level QTL (15076) | 12 | 3594855 | 61602742 |
| ENSSSCG00000034364 | 12 | 59466791 | 59654130 | body weight (10 weeks) QTL (29707) | 12 | 10512227 | 61602742 |
| ENSSSCG00000034364 | 12 | 59466791 | 59654130 | Creatinine level QTL (15075) | 12 | 48203737 | 61602742 |
| ENSSSCG00000034364 | 12 | 59466791 | 59654130 | Meat color chroma QTL (38066) | 12 | 48203737 | 61602742 |
| ENSSSCG00000034364 | 12 | 59466791 | 59654130 | Carcass weight (hot) QTL (29709) | 12 | 48203737 | 61602742 |
| ENSSSCG00000034364 | 12 | 59466791 | 59654130 | Capric acid content QTL (156889) | 12 | 48203737 | 61602742 |
| ENSSSCG00000034364 | 12 | 59466791 | 59654130 | Stearic acid content QTL (156893) | 12 | 48203737 | 61602742 |
| ENSSSCG00000034364 | 12 | 59466791 | 59654130 | Linolenic acid content QTL (156896) | 12 | 48203737 | 61602742 |
| ENSSSCG00000034364 | 12 | 59466791 | 59654130 | Unsaturated fatty acid content QTL (156900) | 12 | 48203737 | 61602742 |
| ENSSSCG00000034364 | 12 | 59466791 | 59654130 | Palmitoleic acid content QTL (156950) | 12 | 52179865 | 61602742 |
| ENSSSCG00000034364 | 12 | 59466791 | 59654130 | Capric acid content QTL (156888) | 12 | 52179865 | 61602742 |
| ENSSSCG00000034364 | 12 | 59466791 | 59654130 | Palmitic acid content QTL (156890) | 12 | 52179865 | 61602742 |
| ENSSSCG00000034364 | 12 | 59466791 | 59654130 | Margaric acid content QTL (156891) | 12 | 52179865 | 61602742 |
| ENSSSCG00000034364 | 12 | 59466791 | 59654130 | cis-9-Heptadecenoic acid content QTL (156892) | 12 | 52179865 | 61602742 |
| ENSSSCG00000034364 | 12 | 59466791 | 59654130 | Oleic acid content QTL (156894) | 12 | 52179865 | 61602742 |
| ENSSSCG00000034364 | 12 | 59466791 | 59654130 | Linoleic acid content QTL (156895) | 12 | 52179865 | 61602742 |
| ENSSSCG00000034364 | 12 | 59466791 | 59654130 | Arachidic acid content QTL (156897) | 12 | 52179865 | 61602742 |
| ENSSSCG00000034364 | 12 | 59466791 | 59654130 | Arachidonic acid content QTL (156899) | 12 | 52179865 | 61602742 |
| ENSSSCG00000034364 | 12 | 59466791 | 59654130 | Monounsaturated fatty acid content QTL (156901) | 12 | 52179865 | 61602742 |
| ENSSSCG00000034364 | 12 | 59466791 | 59654130 | Polyunsaturated fatty acid content QTL (156902) | 12 | 52179865 | 61602742 |
| ENSSSCG00000034364 | 12 | 59466791 | 59654130 | Loin muscle area QTL (38057) | 12 | 52179865 | 61602742 |
| ENSSSCG00000034364 | 12 | 59466791 | 59654130 | Loin muscle area QTL (38058) | 12 | 52179865 | 61602742 |
| ENSSSCG00000034364 | 12 | 59466791 | 59654130 | Marbling QTL (38059) | 12 | 52179865 | 61602742 |
| ENSSSCG00000034364 | 12 | 59466791 | 59654130 | Loin fat percentage QTL (38060) | 12 | 52179865 | 61602742 |
| ENSSSCG00000034364 | 12 | 59466791 | 59654130 | Shear force QTL (38061) | 12 | 52179865 | 61602742 |
| ENSSSCG00000034364 | 12 | 59466791 | 59654130 | Drip loss QTL (38062) | 12 | 52179865 | 61602742 |
| ENSSSCG00000034364 | 12 | 59466791 | 59654130 | Meat color a* QTL (38064) | 12 | 52179865 | 61602742 |
| ENSSSCG00000034364 | 12 | 59466791 | 59654130 | Percentage type IIb fibers QTL (7021) | 12 | 52179865 | 61602742 |
| ENSSSCG00000034364 | 12 | 59466791 | 59654130 | Percentage type IIb fibers QTL (7036) | 12 | 52179865 | 61602742 |
| ENSSSCG00000034364 | 12 | 59466791 | 59654130 | Adipocyte diameter QTL (12844) | 12 | 52179865 | 61602742 |
| ENSSSCG00000018049 | 12 | 60281878 | 60396630 | Triglyceride level QTL (15076) | 12 | 3594855 | 61602742 |
| ENSSSCG00000018049 | 12 | 60281878 | 60396630 | body weight (10 weeks) QTL (29707) | 12 | 10512227 | 61602742 |
| ENSSSCG00000018049 | 12 | 60281878 | 60396630 | Creatinine level QTL (15075) | 12 | 48203737 | 61602742 |
| ENSSSCG00000018049 | 12 | 60281878 | 60396630 | Meat color chroma QTL (38066) | 12 | 48203737 | 61602742 |
| ENSSSCG00000018049 | 12 | 60281878 | 60396630 | Carcass weight (hot) QTL (29709) | 12 | 48203737 | 61602742 |
| ENSSSCG00000018049 | 12 | 60281878 | 60396630 | Capric acid content QTL (156889) | 12 | 48203737 | 61602742 |
| ENSSSCG00000018049 | 12 | 60281878 | 60396630 | Stearic acid content QTL (156893) | 12 | 48203737 | 61602742 |
| ENSSSCG00000018049 | 12 | 60281878 | 60396630 | Linolenic acid content QTL (156896) | 12 | 48203737 | 61602742 |
| ENSSSCG00000018049 | 12 | 60281878 | 60396630 | Unsaturated fatty acid content QTL (156900) | 12 | 48203737 | 61602742 |
| ENSSSCG00000018049 | 12 | 60281878 | 60396630 | Palmitoleic acid content QTL (156950) | 12 | 52179865 | 61602742 |
| ENSSSCG00000018049 | 12 | 60281878 | 60396630 | Capric acid content QTL (156888) | 12 | 52179865 | 61602742 |
| ENSSSCG00000018049 | 12 | 60281878 | 60396630 | Palmitic acid content QTL (156890) | 12 | 52179865 | 61602742 |
| ENSSSCG00000018049 | 12 | 60281878 | 60396630 | Margaric acid content QTL (156891) | 12 | 52179865 | 61602742 |
| ENSSSCG00000018049 | 12 | 60281878 | 60396630 | cis-9-Heptadecenoic acid content QTL (156892) | 12 | 52179865 | 61602742 |
| ENSSSCG00000018049 | 12 | 60281878 | 60396630 | Oleic acid content QTL (156894) | 12 | 52179865 | 61602742 |
| ENSSSCG00000018049 | 12 | 60281878 | 60396630 | Linoleic acid content QTL (156895) | 12 | 52179865 | 61602742 |
| ENSSSCG00000018049 | 12 | 60281878 | 60396630 | Arachidic acid content QTL (156897) | 12 | 52179865 | 61602742 |
| ENSSSCG00000018049 | 12 | 60281878 | 60396630 | Arachidonic acid content QTL (156899) | 12 | 52179865 | 61602742 |
| ENSSSCG00000018049 | 12 | 60281878 | 60396630 | Monounsaturated fatty acid content QTL (156901) | 12 | 52179865 | 61602742 |
| ENSSSCG00000018049 | 12 | 60281878 | 60396630 | Polyunsaturated fatty acid content QTL (156902) | 12 | 52179865 | 61602742 |
| ENSSSCG00000018049 | 12 | 60281878 | 60396630 | Loin muscle area QTL (38057) | 12 | 52179865 | 61602742 |
| ENSSSCG00000018049 | 12 | 60281878 | 60396630 | Loin muscle area QTL (38058) | 12 | 52179865 | 61602742 |
| ENSSSCG00000018049 | 12 | 60281878 | 60396630 | Marbling QTL (38059) | 12 | 52179865 | 61602742 |
| ENSSSCG00000018049 | 12 | 60281878 | 60396630 | Loin fat percentage QTL (38060) | 12 | 52179865 | 61602742 |
| ENSSSCG00000018049 | 12 | 60281878 | 60396630 | Shear force QTL (38061) | 12 | 52179865 | 61602742 |
| ENSSSCG00000018049 | 12 | 60281878 | 60396630 | Drip loss QTL (38062) | 12 | 52179865 | 61602742 |
| ENSSSCG00000018049 | 12 | 60281878 | 60396630 | Meat color a* QTL (38064) | 12 | 52179865 | 61602742 |
| ENSSSCG00000018049 | 12 | 60281878 | 60396630 | Percentage type IIb fibers QTL (7021) | 12 | 52179865 | 61602742 |
| ENSSSCG00000018049 | 12 | 60281878 | 60396630 | Percentage type IIb fibers QTL (7036) | 12 | 52179865 | 61602742 |
| ENSSSCG00000018049 | 12 | 60281878 | 60396630 | Adipocyte diameter QTL (12844) | 12 | 52179865 | 61602742 |
| ENSSSCG00000011183 | 13 | 1438615 | 1612676 | Salmonella count in liver and spleen QTL (11575) | 13 | 75702 | 16806054 |
| ENSSSCG00000034200 | 13 | 26082587 | 26124920 | Average backfat thickness QTL (5718) | 13 | 2422494 | 53597688 |
| ENSSSCG00000034200 | 13 | 26082587 | 26124920 | Backfat at tenth rib QTL (5719) | 13 | 2422494 | 53597688 |
| ENSSSCG00000034200 | 13 | 26082587 | 26124920 | Average daily gain QTL (5721) | 13 | 2422494 | 53597688 |
| ENSSSCG00000034200 | 13 | 26082587 | 26124920 | Sarcocystis miescheriana IgM levels QTL (7515) | 13 | 2422494 | 53597688 |
| ENSSSCG00000034200 | 13 | 26082587 | 26124920 | Meat color-b QTL (238) | 13 | 2422494 | 42242246 |
| ENSSSCG00000034200 | 13 | 26082587 | 26124920 | Loin muscle area QTL (5499) | 13 | 9387165 | 38457098 |
| ENSSSCG00000034200 | 13 | 26082587 | 26124920 | Loin muscle area QTL (38067) | 13 | 21348231 | 65255942 |
| ENSSSCG00000034200 | 13 | 26082587 | 26124920 | LDL cholesterol QTL (17725) | 13 | 2422494 | 1.2E+08 |
| ENSSSCG00000034200 | 13 | 26082587 | 26124920 | Lactate dehydrogenase level QTL (15118) | 13 | 18168708 | 1.12E+08 |
| ENSSSCG00000034200 | 13 | 26082587 | 26124920 | Triglyceride level QTL (15078) | 13 | 21348231 | 1.84E+08 |
| ENSSSCG00000034200 | 13 | 26082587 | 26124920 | Arachidic acid content QTL (156954) | 13 | 21348231 | 1.84E+08 |
| ENSSSCG00000034200 | 13 | 26082587 | 26124920 | Linoleic acid content QTL (156953) | 13 | 21348231 | 1.54E+08 |
| ENSSSCG00000034200 | 13 | 26082587 | 26124920 | Polyunsaturated fatty acid content QTL (156956) | 13 | 21348231 | 1.54E+08 |
| ENSSSCG00000034200 | 13 | 26082587 | 26124920 | Loin muscle area QTL (38068) | 13 | 21348231 | 98111023 |
| ENSSSCG00000011312 | 13 | 28524813 | 28692327 | Water holding capacity QTL (113) | 13 | 27661857 | 29281901 |
| ENSSSCG00000011312 | 13 | 28524813 | 28692327 | pH 45 minutes post mortem QTL (18682) | 13 | 27661857 | 29281901 |
| ENSSSCG00000011312 | 13 | 28524813 | 28692327 | Average backfat thickness QTL (5718) | 13 | 2422494 | 53597688 |
| ENSSSCG00000011312 | 13 | 28524813 | 28692327 | Backfat at tenth rib QTL (5719) | 13 | 2422494 | 53597688 |
| ENSSSCG00000011312 | 13 | 28524813 | 28692327 | Average daily gain QTL (5721) | 13 | 2422494 | 53597688 |
| ENSSSCG00000011312 | 13 | 28524813 | 28692327 | Sarcocystis miescheriana IgM levels QTL (7515) | 13 | 2422494 | 53597688 |
| ENSSSCG00000011312 | 13 | 28524813 | 28692327 | Meat color-b QTL (238) | 13 | 2422494 | 42242246 |
| ENSSSCG00000011312 | 13 | 28524813 | 28692327 | Loin muscle area QTL (5499) | 13 | 9387165 | 38457098 |
| ENSSSCG00000011312 | 13 | 28524813 | 28692327 | Loin muscle area QTL (38067) | 13 | 21348231 | 65255942 |
| ENSSSCG00000011312 | 13 | 28524813 | 28692327 | LDL cholesterol QTL (17725) | 13 | 2422494 | 1.2E+08 |
| ENSSSCG00000011312 | 13 | 28524813 | 28692327 | Lactate dehydrogenase level QTL (15118) | 13 | 18168708 | 1.12E+08 |
| ENSSSCG00000011312 | 13 | 28524813 | 28692327 | Triglyceride level QTL (15078) | 13 | 21348231 | 1.84E+08 |
| ENSSSCG00000011312 | 13 | 28524813 | 28692327 | Arachidic acid content QTL (156954) | 13 | 21348231 | 1.84E+08 |
| ENSSSCG00000011312 | 13 | 28524813 | 28692327 | Linoleic acid content QTL (156953) | 13 | 21348231 | 1.54E+08 |
| ENSSSCG00000011312 | 13 | 28524813 | 28692327 | Polyunsaturated fatty acid content QTL (156956) | 13 | 21348231 | 1.54E+08 |
| ENSSSCG00000011312 | 13 | 28524813 | 28692327 | Loin muscle area QTL (38068) | 13 | 21348231 | 98111023 |
| ENSSSCG00000011317 | 13 | 29066986 | 29146755 | Water holding capacity QTL (113) | 13 | 27661857 | 29281901 |
| ENSSSCG00000011317 | 13 | 29066986 | 29146755 | pH 45 minutes post mortem QTL (18682) | 13 | 27661857 | 29281901 |
| ENSSSCG00000011317 | 13 | 29066986 | 29146755 | Average backfat thickness QTL (5718) | 13 | 2422494 | 53597688 |
| ENSSSCG00000011317 | 13 | 29066986 | 29146755 | Backfat at tenth rib QTL (5719) | 13 | 2422494 | 53597688 |
| ENSSSCG00000011317 | 13 | 29066986 | 29146755 | Average daily gain QTL (5721) | 13 | 2422494 | 53597688 |
| ENSSSCG00000011317 | 13 | 29066986 | 29146755 | Sarcocystis miescheriana IgM levels QTL (7515) | 13 | 2422494 | 53597688 |
| ENSSSCG00000011317 | 13 | 29066986 | 29146755 | Meat color-b QTL (238) | 13 | 2422494 | 42242246 |
| ENSSSCG00000011317 | 13 | 29066986 | 29146755 | Loin muscle area QTL (5499) | 13 | 9387165 | 38457098 |
| ENSSSCG00000011317 | 13 | 29066986 | 29146755 | Loin muscle area QTL (38067) | 13 | 21348231 | 65255942 |
| ENSSSCG00000011317 | 13 | 29066986 | 29146755 | LDL cholesterol QTL (17725) | 13 | 2422494 | 1.2E+08 |
| ENSSSCG00000011317 | 13 | 29066986 | 29146755 | Lactate dehydrogenase level QTL (15118) | 13 | 18168708 | 1.12E+08 |
| ENSSSCG00000011317 | 13 | 29066986 | 29146755 | Triglyceride level QTL (15078) | 13 | 21348231 | 1.84E+08 |
| ENSSSCG00000011317 | 13 | 29066986 | 29146755 | Arachidic acid content QTL (156954) | 13 | 21348231 | 1.84E+08 |
| ENSSSCG00000011317 | 13 | 29066986 | 29146755 | Linoleic acid content QTL (156953) | 13 | 21348231 | 1.54E+08 |
| ENSSSCG00000011317 | 13 | 29066986 | 29146755 | Polyunsaturated fatty acid content QTL (156956) | 13 | 21348231 | 1.54E+08 |
| ENSSSCG00000011317 | 13 | 29066986 | 29146755 | Loin muscle area QTL (38068) | 13 | 21348231 | 98111023 |
| ENSSSCG00000011317 | 13 | 29066986 | 29146755 | Meat color a* QTL (38109) | 13 | 28918526 | 1.73E+08 |
| ENSSSCG00000011317 | 13 | 29066986 | 29146755 | Shear force QTL (38107) | 13 | 28918526 | 1.17E+08 |
| ENSSSCG00000011317 | 13 | 29066986 | 29146755 | Gadoleic acid content QTL (156955) | 13 | 28918526 | 98111023 |
| ENSSSCG00000011340 | 13 | 30409982 | 30601441 | Average backfat thickness QTL (5718) | 13 | 2422494 | 53597688 |
| ENSSSCG00000011340 | 13 | 30409982 | 30601441 | Backfat at tenth rib QTL (5719) | 13 | 2422494 | 53597688 |
| ENSSSCG00000011340 | 13 | 30409982 | 30601441 | Average daily gain QTL (5721) | 13 | 2422494 | 53597688 |
| ENSSSCG00000011340 | 13 | 30409982 | 30601441 | Sarcocystis miescheriana IgM levels QTL (7515) | 13 | 2422494 | 53597688 |
| ENSSSCG00000011340 | 13 | 30409982 | 30601441 | Meat color-b QTL (238) | 13 | 2422494 | 42242246 |
| ENSSSCG00000011340 | 13 | 30409982 | 30601441 | Loin muscle area QTL (5499) | 13 | 9387165 | 38457098 |
| ENSSSCG00000011340 | 13 | 30409982 | 30601441 | Loin muscle area QTL (38067) | 13 | 21348231 | 65255942 |
| ENSSSCG00000011340 | 13 | 30409982 | 30601441 | LDL cholesterol QTL (17725) | 13 | 2422494 | 1.2E+08 |
| ENSSSCG00000011340 | 13 | 30409982 | 30601441 | Lactate dehydrogenase level QTL (15118) | 13 | 18168708 | 1.12E+08 |
| ENSSSCG00000011340 | 13 | 30409982 | 30601441 | Triglyceride level QTL (15078) | 13 | 21348231 | 1.84E+08 |
| ENSSSCG00000011340 | 13 | 30409982 | 30601441 | Arachidic acid content QTL (156954) | 13 | 21348231 | 1.84E+08 |
| ENSSSCG00000011340 | 13 | 30409982 | 30601441 | Linoleic acid content QTL (156953) | 13 | 21348231 | 1.54E+08 |
| ENSSSCG00000011340 | 13 | 30409982 | 30601441 | Polyunsaturated fatty acid content QTL (156956) | 13 | 21348231 | 1.54E+08 |
| ENSSSCG00000011340 | 13 | 30409982 | 30601441 | Loin muscle area QTL (38068) | 13 | 21348231 | 98111023 |
| ENSSSCG00000011340 | 13 | 30409982 | 30601441 | Meat color a* QTL (38109) | 13 | 28918526 | 1.73E+08 |
| ENSSSCG00000011340 | 13 | 30409982 | 30601441 | Shear force QTL (38107) | 13 | 28918526 | 1.17E+08 |
| ENSSSCG00000011340 | 13 | 30409982 | 30601441 | Gadoleic acid content QTL (156955) | 13 | 28918526 | 98111023 |
| ENSSSCG00000011342 | 13 | 30602034 | 30632504 | Average backfat thickness QTL (5718) | 13 | 2422494 | 53597688 |
| ENSSSCG00000011342 | 13 | 30602034 | 30632504 | Backfat at tenth rib QTL (5719) | 13 | 2422494 | 53597688 |
| ENSSSCG00000011342 | 13 | 30602034 | 30632504 | Average daily gain QTL (5721) | 13 | 2422494 | 53597688 |
| ENSSSCG00000011342 | 13 | 30602034 | 30632504 | Sarcocystis miescheriana IgM levels QTL (7515) | 13 | 2422494 | 53597688 |
| ENSSSCG00000011342 | 13 | 30602034 | 30632504 | Meat color-b QTL (238) | 13 | 2422494 | 42242246 |
| ENSSSCG00000011342 | 13 | 30602034 | 30632504 | Loin muscle area QTL (5499) | 13 | 9387165 | 38457098 |
| ENSSSCG00000011342 | 13 | 30602034 | 30632504 | Loin muscle area QTL (38067) | 13 | 21348231 | 65255942 |
| ENSSSCG00000011342 | 13 | 30602034 | 30632504 | LDL cholesterol QTL (17725) | 13 | 2422494 | 1.2E+08 |
| ENSSSCG00000011342 | 13 | 30602034 | 30632504 | Lactate dehydrogenase level QTL (15118) | 13 | 18168708 | 1.12E+08 |
| ENSSSCG00000011342 | 13 | 30602034 | 30632504 | Triglyceride level QTL (15078) | 13 | 21348231 | 1.84E+08 |
| ENSSSCG00000011342 | 13 | 30602034 | 30632504 | Arachidic acid content QTL (156954) | 13 | 21348231 | 1.84E+08 |
| ENSSSCG00000011342 | 13 | 30602034 | 30632504 | Linoleic acid content QTL (156953) | 13 | 21348231 | 1.54E+08 |
| ENSSSCG00000011342 | 13 | 30602034 | 30632504 | Polyunsaturated fatty acid content QTL (156956) | 13 | 21348231 | 1.54E+08 |
| ENSSSCG00000011342 | 13 | 30602034 | 30632504 | Loin muscle area QTL (38068) | 13 | 21348231 | 98111023 |
| ENSSSCG00000011342 | 13 | 30602034 | 30632504 | Meat color a* QTL (38109) | 13 | 28918526 | 1.73E+08 |
| ENSSSCG00000011342 | 13 | 30602034 | 30632504 | Shear force QTL (38107) | 13 | 28918526 | 1.17E+08 |
| ENSSSCG00000011342 | 13 | 30602034 | 30632504 | Gadoleic acid content QTL (156955) | 13 | 28918526 | 98111023 |
| ENSSSCG00000029251 | 13 | 30631924 | 30804774 | Average backfat thickness QTL (5718) | 13 | 2422494 | 53597688 |
| ENSSSCG00000029251 | 13 | 30631924 | 30804774 | Backfat at tenth rib QTL (5719) | 13 | 2422494 | 53597688 |
| ENSSSCG00000029251 | 13 | 30631924 | 30804774 | Average daily gain QTL (5721) | 13 | 2422494 | 53597688 |
| ENSSSCG00000029251 | 13 | 30631924 | 30804774 | Sarcocystis miescheriana IgM levels QTL (7515) | 13 | 2422494 | 53597688 |
| ENSSSCG00000029251 | 13 | 30631924 | 30804774 | Meat color-b QTL (238) | 13 | 2422494 | 42242246 |
| ENSSSCG00000029251 | 13 | 30631924 | 30804774 | Loin muscle area QTL (5499) | 13 | 9387165 | 38457098 |
| ENSSSCG00000029251 | 13 | 30631924 | 30804774 | Loin muscle area QTL (38067) | 13 | 21348231 | 65255942 |
| ENSSSCG00000029251 | 13 | 30631924 | 30804774 | LDL cholesterol QTL (17725) | 13 | 2422494 | 1.2E+08 |
| ENSSSCG00000029251 | 13 | 30631924 | 30804774 | Lactate dehydrogenase level QTL (15118) | 13 | 18168708 | 1.12E+08 |
| ENSSSCG00000029251 | 13 | 30631924 | 30804774 | Triglyceride level QTL (15078) | 13 | 21348231 | 1.84E+08 |
| ENSSSCG00000029251 | 13 | 30631924 | 30804774 | Arachidic acid content QTL (156954) | 13 | 21348231 | 1.84E+08 |
| ENSSSCG00000029251 | 13 | 30631924 | 30804774 | Linoleic acid content QTL (156953) | 13 | 21348231 | 1.54E+08 |
| ENSSSCG00000029251 | 13 | 30631924 | 30804774 | Polyunsaturated fatty acid content QTL (156956) | 13 | 21348231 | 1.54E+08 |
| ENSSSCG00000029251 | 13 | 30631924 | 30804774 | Loin muscle area QTL (38068) | 13 | 21348231 | 98111023 |
| ENSSSCG00000029251 | 13 | 30631924 | 30804774 | Meat color a* QTL (38109) | 13 | 28918526 | 1.73E+08 |
| ENSSSCG00000029251 | 13 | 30631924 | 30804774 | Shear force QTL (38107) | 13 | 28918526 | 1.17E+08 |
| ENSSSCG00000029251 | 13 | 30631924 | 30804774 | Gadoleic acid content QTL (156955) | 13 | 28918526 | 98111023 |
| ENSSSCG00000011373 | 13 | 31658642 | 31714455 | Average backfat thickness QTL (5718) | 13 | 2422494 | 53597688 |
| ENSSSCG00000011373 | 13 | 31658642 | 31714455 | Backfat at tenth rib QTL (5719) | 13 | 2422494 | 53597688 |
| ENSSSCG00000011373 | 13 | 31658642 | 31714455 | Average daily gain QTL (5721) | 13 | 2422494 | 53597688 |
| ENSSSCG00000011373 | 13 | 31658642 | 31714455 | Sarcocystis miescheriana IgM levels QTL (7515) | 13 | 2422494 | 53597688 |
| ENSSSCG00000011373 | 13 | 31658642 | 31714455 | Meat color-b QTL (238) | 13 | 2422494 | 42242246 |
| ENSSSCG00000011373 | 13 | 31658642 | 31714455 | Loin muscle area QTL (5499) | 13 | 9387165 | 38457098 |
| ENSSSCG00000011373 | 13 | 31658642 | 31714455 | Loin muscle area QTL (38067) | 13 | 21348231 | 65255942 |
| ENSSSCG00000011373 | 13 | 31658642 | 31714455 | LDL cholesterol QTL (17725) | 13 | 2422494 | 1.2E+08 |
| ENSSSCG00000011373 | 13 | 31658642 | 31714455 | Lactate dehydrogenase level QTL (15118) | 13 | 18168708 | 1.12E+08 |
| ENSSSCG00000011373 | 13 | 31658642 | 31714455 | Triglyceride level QTL (15078) | 13 | 21348231 | 1.84E+08 |
| ENSSSCG00000011373 | 13 | 31658642 | 31714455 | Arachidic acid content QTL (156954) | 13 | 21348231 | 1.84E+08 |
| ENSSSCG00000011373 | 13 | 31658642 | 31714455 | Linoleic acid content QTL (156953) | 13 | 21348231 | 1.54E+08 |
| ENSSSCG00000011373 | 13 | 31658642 | 31714455 | Polyunsaturated fatty acid content QTL (156956) | 13 | 21348231 | 1.54E+08 |
| ENSSSCG00000011373 | 13 | 31658642 | 31714455 | Loin muscle area QTL (38068) | 13 | 21348231 | 98111023 |
| ENSSSCG00000011373 | 13 | 31658642 | 31714455 | Meat color a* QTL (38109) | 13 | 28918526 | 1.73E+08 |
| ENSSSCG00000011373 | 13 | 31658642 | 31714455 | Shear force QTL (38107) | 13 | 28918526 | 1.17E+08 |
| ENSSSCG00000011373 | 13 | 31658642 | 31714455 | Gadoleic acid content QTL (156955) | 13 | 28918526 | 98111023 |
| ENSSSCG00000011394 | 13 | 32444950 | 32569816 | Marbling QTL (12659) | 13 | 32355440 | 32552268 |
| ENSSSCG00000011394 | 13 | 32444950 | 32569816 | Backfat at last rib QTL (12660) | 13 | 32355440 | 32552268 |
| ENSSSCG00000011394 | 13 | 32444950 | 32569816 | Meat color L* QTL (12684) | 13 | 32355440 | 32552268 |
| ENSSSCG00000011394 | 13 | 32444950 | 32569816 | Average backfat thickness QTL (5718) | 13 | 2422494 | 53597688 |
| ENSSSCG00000011394 | 13 | 32444950 | 32569816 | Backfat at tenth rib QTL (5719) | 13 | 2422494 | 53597688 |
| ENSSSCG00000011394 | 13 | 32444950 | 32569816 | Average daily gain QTL (5721) | 13 | 2422494 | 53597688 |
| ENSSSCG00000011394 | 13 | 32444950 | 32569816 | Sarcocystis miescheriana IgM levels QTL (7515) | 13 | 2422494 | 53597688 |
| ENSSSCG00000011394 | 13 | 32444950 | 32569816 | Meat color-b QTL (238) | 13 | 2422494 | 42242246 |
| ENSSSCG00000011394 | 13 | 32444950 | 32569816 | Loin muscle area QTL (5499) | 13 | 9387165 | 38457098 |
| ENSSSCG00000011394 | 13 | 32444950 | 32569816 | Loin muscle area QTL (38067) | 13 | 21348231 | 65255942 |
| ENSSSCG00000011394 | 13 | 32444950 | 32569816 | LDL cholesterol QTL (17725) | 13 | 2422494 | 1.2E+08 |
| ENSSSCG00000011394 | 13 | 32444950 | 32569816 | Lactate dehydrogenase level QTL (15118) | 13 | 18168708 | 1.12E+08 |
| ENSSSCG00000011394 | 13 | 32444950 | 32569816 | Triglyceride level QTL (15078) | 13 | 21348231 | 1.84E+08 |
| ENSSSCG00000011394 | 13 | 32444950 | 32569816 | Arachidic acid content QTL (156954) | 13 | 21348231 | 1.84E+08 |
| ENSSSCG00000011394 | 13 | 32444950 | 32569816 | Linoleic acid content QTL (156953) | 13 | 21348231 | 1.54E+08 |
| ENSSSCG00000011394 | 13 | 32444950 | 32569816 | Polyunsaturated fatty acid content QTL (156956) | 13 | 21348231 | 1.54E+08 |
| ENSSSCG00000011394 | 13 | 32444950 | 32569816 | Loin muscle area QTL (38068) | 13 | 21348231 | 98111023 |
| ENSSSCG00000011394 | 13 | 32444950 | 32569816 | Meat color a* QTL (38109) | 13 | 28918526 | 1.73E+08 |
| ENSSSCG00000011394 | 13 | 32444950 | 32569816 | Shear force QTL (38107) | 13 | 28918526 | 1.17E+08 |
| ENSSSCG00000011394 | 13 | 32444950 | 32569816 | Gadoleic acid content QTL (156955) | 13 | 28918526 | 98111023 |
| ENSSSCG00000011424 | 13 | 33771816 | 33926156 | Average backfat thickness QTL (5718) | 13 | 2422494 | 53597688 |
| ENSSSCG00000011424 | 13 | 33771816 | 33926156 | Backfat at tenth rib QTL (5719) | 13 | 2422494 | 53597688 |
| ENSSSCG00000011424 | 13 | 33771816 | 33926156 | Average daily gain QTL (5721) | 13 | 2422494 | 53597688 |
| ENSSSCG00000011424 | 13 | 33771816 | 33926156 | Sarcocystis miescheriana IgM levels QTL (7515) | 13 | 2422494 | 53597688 |
| ENSSSCG00000011424 | 13 | 33771816 | 33926156 | Meat color-b QTL (238) | 13 | 2422494 | 42242246 |
| ENSSSCG00000011424 | 13 | 33771816 | 33926156 | Loin muscle area QTL (5499) | 13 | 9387165 | 38457098 |
| ENSSSCG00000011424 | 13 | 33771816 | 33926156 | Loin muscle area QTL (38067) | 13 | 21348231 | 65255942 |
| ENSSSCG00000011424 | 13 | 33771816 | 33926156 | LDL cholesterol QTL (17725) | 13 | 2422494 | 1.2E+08 |
| ENSSSCG00000011424 | 13 | 33771816 | 33926156 | Lactate dehydrogenase level QTL (15118) | 13 | 18168708 | 1.12E+08 |
| ENSSSCG00000011424 | 13 | 33771816 | 33926156 | Triglyceride level QTL (15078) | 13 | 21348231 | 1.84E+08 |
| ENSSSCG00000011424 | 13 | 33771816 | 33926156 | Arachidic acid content QTL (156954) | 13 | 21348231 | 1.84E+08 |
| ENSSSCG00000011424 | 13 | 33771816 | 33926156 | Linoleic acid content QTL (156953) | 13 | 21348231 | 1.54E+08 |
| ENSSSCG00000011424 | 13 | 33771816 | 33926156 | Polyunsaturated fatty acid content QTL (156956) | 13 | 21348231 | 1.54E+08 |
| ENSSSCG00000011424 | 13 | 33771816 | 33926156 | Loin muscle area QTL (38068) | 13 | 21348231 | 98111023 |
| ENSSSCG00000011424 | 13 | 33771816 | 33926156 | Meat color a* QTL (38109) | 13 | 28918526 | 1.73E+08 |
| ENSSSCG00000011424 | 13 | 33771816 | 33926156 | Shear force QTL (38107) | 13 | 28918526 | 1.17E+08 |
| ENSSSCG00000011424 | 13 | 33771816 | 33926156 | Gadoleic acid content QTL (156955) | 13 | 28918526 | 98111023 |
| ENSSSCG00000024453 | 13 | 68580812 | 68657829 | Testosterone level QTL (7213) | 13 | 67633015 | 69434745 |
| ENSSSCG00000024453 | 13 | 68580812 | 68657829 | Carcass length QTL (16978) | 13 | 67829843 | 75309294 |
| ENSSSCG00000024453 | 13 | 68580812 | 68657829 | LDL cholesterol QTL (17725) | 13 | 2422494 | 1.2E+08 |
| ENSSSCG00000024453 | 13 | 68580812 | 68657829 | Lactate dehydrogenase level QTL (15118) | 13 | 18168708 | 1.12E+08 |
| ENSSSCG00000024453 | 13 | 68580812 | 68657829 | Triglyceride level QTL (15078) | 13 | 21348231 | 1.84E+08 |
| ENSSSCG00000024453 | 13 | 68580812 | 68657829 | Arachidic acid content QTL (156954) | 13 | 21348231 | 1.84E+08 |
| ENSSSCG00000024453 | 13 | 68580812 | 68657829 | Linoleic acid content QTL (156953) | 13 | 21348231 | 1.54E+08 |
| ENSSSCG00000024453 | 13 | 68580812 | 68657829 | Polyunsaturated fatty acid content QTL (156956) | 13 | 21348231 | 1.54E+08 |
| ENSSSCG00000024453 | 13 | 68580812 | 68657829 | Loin muscle area QTL (38068) | 13 | 21348231 | 98111023 |
| ENSSSCG00000024453 | 13 | 68580812 | 68657829 | Meat color a* QTL (38109) | 13 | 28918526 | 1.73E+08 |
| ENSSSCG00000024453 | 13 | 68580812 | 68657829 | Shear force QTL (38107) | 13 | 28918526 | 1.17E+08 |
| ENSSSCG00000024453 | 13 | 68580812 | 68657829 | Gadoleic acid content QTL (156955) | 13 | 28918526 | 98111023 |
| ENSSSCG00000024453 | 13 | 68580812 | 68657829 | Palmitoleic acid content QTL (156952) | 13 | 41182404 | 1.54E+08 |
| ENSSSCG00000024453 | 13 | 68580812 | 68657829 | Muscle moisture percentage QTL (38108) | 13 | 41182404 | 1.17E+08 |
| ENSSSCG00000024453 | 13 | 68580812 | 68657829 | Loin fat percentage QTL (38069) | 13 | 41182404 | 98111023 |
| ENSSSCG00000024453 | 13 | 68580812 | 68657829 | androstenone, sensory panel QTL (4227) | 13 | 42242246 | 94174469 |
| ENSSSCG00000024453 | 13 | 68580812 | 68657829 | Average daily gain QTL (328) | 13 | 42242246 | 94174469 |
| ENSSSCG00000024453 | 13 | 68580812 | 68657829 | Average daily gain QTL (336) | 13 | 42242246 | 94174469 |
| ENSSSCG00000024453 | 13 | 68580812 | 68657829 | Average daily gain QTL (347) | 13 | 42242246 | 94174469 |
| ENSSSCG00000024453 | 13 | 68580812 | 68657829 | Backfat at last rib QTL (16840) | 13 | 42242246 | 85998551 |
| ENSSSCG00000024453 | 13 | 68580812 | 68657829 | Capric acid content QTL (156951) | 13 | 53597688 | 1.54E+08 |
| ENSSSCG00000024453 | 13 | 68580812 | 68657829 | Subcutanous fat area QTL (5720) | 13 | 53597688 | 1.2E+08 |
| ENSSSCG00000024453 | 13 | 68580812 | 68657829 | Conductivity 24 hours post-mortem QTL (1138) | 13 | 65255942 | 80245127 |
| ENSSSCG00000011632 | 13 | 73843805 | 73900967 | Carcass length QTL (16978) | 13 | 67829843 | 75309294 |
| ENSSSCG00000011632 | 13 | 73843805 | 73900967 | Muscle moisture percentage QTL (3004) | 13 | 71160773 | 94174469 |
| ENSSSCG00000011632 | 13 | 73843805 | 73900967 | LDL cholesterol QTL (17725) | 13 | 2422494 | 1.2E+08 |
| ENSSSCG00000011632 | 13 | 73843805 | 73900967 | Lactate dehydrogenase level QTL (15118) | 13 | 18168708 | 1.12E+08 |
| ENSSSCG00000011632 | 13 | 73843805 | 73900967 | Triglyceride level QTL (15078) | 13 | 21348231 | 1.84E+08 |
| ENSSSCG00000011632 | 13 | 73843805 | 73900967 | Arachidic acid content QTL (156954) | 13 | 21348231 | 1.84E+08 |
| ENSSSCG00000011632 | 13 | 73843805 | 73900967 | Linoleic acid content QTL (156953) | 13 | 21348231 | 1.54E+08 |
| ENSSSCG00000011632 | 13 | 73843805 | 73900967 | Polyunsaturated fatty acid content QTL (156956) | 13 | 21348231 | 1.54E+08 |
| ENSSSCG00000011632 | 13 | 73843805 | 73900967 | Loin muscle area QTL (38068) | 13 | 21348231 | 98111023 |
| ENSSSCG00000011632 | 13 | 73843805 | 73900967 | Meat color a* QTL (38109) | 13 | 28918526 | 1.73E+08 |
| ENSSSCG00000011632 | 13 | 73843805 | 73900967 | Shear force QTL (38107) | 13 | 28918526 | 1.17E+08 |
| ENSSSCG00000011632 | 13 | 73843805 | 73900967 | Gadoleic acid content QTL (156955) | 13 | 28918526 | 98111023 |
| ENSSSCG00000011632 | 13 | 73843805 | 73900967 | Palmitoleic acid content QTL (156952) | 13 | 41182404 | 1.54E+08 |
| ENSSSCG00000011632 | 13 | 73843805 | 73900967 | Muscle moisture percentage QTL (38108) | 13 | 41182404 | 1.17E+08 |
| ENSSSCG00000011632 | 13 | 73843805 | 73900967 | Loin fat percentage QTL (38069) | 13 | 41182404 | 98111023 |
| ENSSSCG00000011632 | 13 | 73843805 | 73900967 | androstenone, sensory panel QTL (4227) | 13 | 42242246 | 94174469 |
| ENSSSCG00000011632 | 13 | 73843805 | 73900967 | Average daily gain QTL (328) | 13 | 42242246 | 94174469 |
| ENSSSCG00000011632 | 13 | 73843805 | 73900967 | Average daily gain QTL (336) | 13 | 42242246 | 94174469 |
| ENSSSCG00000011632 | 13 | 73843805 | 73900967 | Average daily gain QTL (347) | 13 | 42242246 | 94174469 |
| ENSSSCG00000011632 | 13 | 73843805 | 73900967 | Backfat at last rib QTL (16840) | 13 | 42242246 | 85998551 |
| ENSSSCG00000011632 | 13 | 73843805 | 73900967 | Capric acid content QTL (156951) | 13 | 53597688 | 1.54E+08 |
| ENSSSCG00000011632 | 13 | 73843805 | 73900967 | Subcutanous fat area QTL (5720) | 13 | 53597688 | 1.2E+08 |
| ENSSSCG00000011632 | 13 | 73843805 | 73900967 | Conductivity 24 hours post-mortem QTL (1138) | 13 | 65255942 | 80245127 |
| ENSSSCG00000027459 | 13 | 90034344 | 90178129 | Muscle moisture percentage QTL (3004) | 13 | 71160773 | 94174469 |
| ENSSSCG00000027459 | 13 | 90034344 | 90178129 | Loin muscle area QTL (5780) | 13 | 77716648 | 1.17E+08 |
| ENSSSCG00000027459 | 13 | 90034344 | 90178129 | Backfat at last rib QTL (17784) | 13 | 80245127 | 1.2E+08 |
| ENSSSCG00000027459 | 13 | 90034344 | 90178129 | Fat area percentage in carcass QTL (18641) | 13 | 80245127 | 94174469 |
| ENSSSCG00000027459 | 13 | 90034344 | 90178129 | pH 40 minutes post mortem (loin) QTL (4004) | 13 | 87815422 | 1.2E+08 |
| ENSSSCG00000027459 | 13 | 90034344 | 90178129 | Skin percentage QTL (5507) | 13 | 87815422 | 1.13E+08 |
| ENSSSCG00000027459 | 13 | 90034344 | 90178129 | LDL cholesterol QTL (17725) | 13 | 2422494 | 1.2E+08 |
| ENSSSCG00000027459 | 13 | 90034344 | 90178129 | Lactate dehydrogenase level QTL (15118) | 13 | 18168708 | 1.12E+08 |
| ENSSSCG00000027459 | 13 | 90034344 | 90178129 | Triglyceride level QTL (15078) | 13 | 21348231 | 1.84E+08 |
| ENSSSCG00000027459 | 13 | 90034344 | 90178129 | Arachidic acid content QTL (156954) | 13 | 21348231 | 1.84E+08 |
| ENSSSCG00000027459 | 13 | 90034344 | 90178129 | Linoleic acid content QTL (156953) | 13 | 21348231 | 1.54E+08 |
| ENSSSCG00000027459 | 13 | 90034344 | 90178129 | Polyunsaturated fatty acid content QTL (156956) | 13 | 21348231 | 1.54E+08 |
| ENSSSCG00000027459 | 13 | 90034344 | 90178129 | Loin muscle area QTL (38068) | 13 | 21348231 | 98111023 |
| ENSSSCG00000027459 | 13 | 90034344 | 90178129 | Meat color a* QTL (38109) | 13 | 28918526 | 1.73E+08 |
| ENSSSCG00000027459 | 13 | 90034344 | 90178129 | Shear force QTL (38107) | 13 | 28918526 | 1.17E+08 |
| ENSSSCG00000027459 | 13 | 90034344 | 90178129 | Gadoleic acid content QTL (156955) | 13 | 28918526 | 98111023 |
| ENSSSCG00000027459 | 13 | 90034344 | 90178129 | Palmitoleic acid content QTL (156952) | 13 | 41182404 | 1.54E+08 |
| ENSSSCG00000027459 | 13 | 90034344 | 90178129 | Muscle moisture percentage QTL (38108) | 13 | 41182404 | 1.17E+08 |
| ENSSSCG00000027459 | 13 | 90034344 | 90178129 | Loin fat percentage QTL (38069) | 13 | 41182404 | 98111023 |
| ENSSSCG00000027459 | 13 | 90034344 | 90178129 | androstenone, sensory panel QTL (4227) | 13 | 42242246 | 94174469 |
| ENSSSCG00000027459 | 13 | 90034344 | 90178129 | Average daily gain QTL (328) | 13 | 42242246 | 94174469 |
| ENSSSCG00000027459 | 13 | 90034344 | 90178129 | Average daily gain QTL (336) | 13 | 42242246 | 94174469 |
| ENSSSCG00000027459 | 13 | 90034344 | 90178129 | Average daily gain QTL (347) | 13 | 42242246 | 94174469 |
| ENSSSCG00000027459 | 13 | 90034344 | 90178129 | Capric acid content QTL (156951) | 13 | 53597688 | 1.54E+08 |
| ENSSSCG00000027459 | 13 | 90034344 | 90178129 | Subcutanous fat area QTL (5720) | 13 | 53597688 | 1.2E+08 |
| ENSSSCG00000027459 | 13 | 90034344 | 90178129 | Temperature 40 minutes post mortem (loin) QTL (4005) | 13 | 80472235 | 1.75E+08 |
| ENSSSCG00000024916 | 13 | 94298790 | 94350312 | Loin muscle area QTL (5780) | 13 | 77716648 | 1.17E+08 |
| ENSSSCG00000024916 | 13 | 94298790 | 94350312 | Backfat at last rib QTL (17784) | 13 | 80245127 | 1.2E+08 |
| ENSSSCG00000024916 | 13 | 94298790 | 94350312 | pH 40 minutes post mortem (loin) QTL (4004) | 13 | 87815422 | 1.2E+08 |
| ENSSSCG00000024916 | 13 | 94298790 | 94350312 | Skin percentage QTL (5507) | 13 | 87815422 | 1.13E+08 |
| ENSSSCG00000024916 | 13 | 94298790 | 94350312 | backfat at P2 position QTL (4163) | 13 | 94174469 | 1.2E+08 |
| ENSSSCG00000024916 | 13 | 94298790 | 94350312 | Carcass length QTL (4164) | 13 | 94174469 | 1.2E+08 |
| ENSSSCG00000024916 | 13 | 94298790 | 94350312 | Meat color a* QTL (3074) | 13 | 94174469 | 1.2E+08 |
| ENSSSCG00000024916 | 13 | 94298790 | 94350312 | Loin meat weight QTL (3077) | 13 | 94174469 | 1.2E+08 |
| ENSSSCG00000024916 | 13 | 94298790 | 94350312 | Backfat at last rib QTL (3672) | 13 | 94174469 | 1.2E+08 |
| ENSSSCG00000024916 | 13 | 94298790 | 94350312 | PH for Longissmus dorsi QTL (3689) | 13 | 94174469 | 1.2E+08 |
| ENSSSCG00000024916 | 13 | 94298790 | 94350312 | Carcass length QTL (3690) | 13 | 94174469 | 1.2E+08 |
| ENSSSCG00000024916 | 13 | 94298790 | 94350312 | PH for Longissmus dorsi QTL (3692) | 13 | 94174469 | 1.2E+08 |
| ENSSSCG00000024916 | 13 | 94298790 | 94350312 | LDL cholesterol QTL (17725) | 13 | 2422494 | 1.2E+08 |
| ENSSSCG00000024916 | 13 | 94298790 | 94350312 | Lactate dehydrogenase level QTL (15118) | 13 | 18168708 | 1.12E+08 |
| ENSSSCG00000024916 | 13 | 94298790 | 94350312 | Triglyceride level QTL (15078) | 13 | 21348231 | 1.84E+08 |
| ENSSSCG00000024916 | 13 | 94298790 | 94350312 | Arachidic acid content QTL (156954) | 13 | 21348231 | 1.84E+08 |
| ENSSSCG00000024916 | 13 | 94298790 | 94350312 | Linoleic acid content QTL (156953) | 13 | 21348231 | 1.54E+08 |
| ENSSSCG00000024916 | 13 | 94298790 | 94350312 | Polyunsaturated fatty acid content QTL (156956) | 13 | 21348231 | 1.54E+08 |
| ENSSSCG00000024916 | 13 | 94298790 | 94350312 | Loin muscle area QTL (38068) | 13 | 21348231 | 98111023 |
| ENSSSCG00000024916 | 13 | 94298790 | 94350312 | Meat color a* QTL (38109) | 13 | 28918526 | 1.73E+08 |
| ENSSSCG00000024916 | 13 | 94298790 | 94350312 | Shear force QTL (38107) | 13 | 28918526 | 1.17E+08 |
| ENSSSCG00000024916 | 13 | 94298790 | 94350312 | Gadoleic acid content QTL (156955) | 13 | 28918526 | 98111023 |
| ENSSSCG00000024916 | 13 | 94298790 | 94350312 | Palmitoleic acid content QTL (156952) | 13 | 41182404 | 1.54E+08 |
| ENSSSCG00000024916 | 13 | 94298790 | 94350312 | Muscle moisture percentage QTL (38108) | 13 | 41182404 | 1.17E+08 |
| ENSSSCG00000024916 | 13 | 94298790 | 94350312 | Loin fat percentage QTL (38069) | 13 | 41182404 | 98111023 |
| ENSSSCG00000024916 | 13 | 94298790 | 94350312 | Capric acid content QTL (156951) | 13 | 53597688 | 1.54E+08 |
| ENSSSCG00000024916 | 13 | 94298790 | 94350312 | Subcutanous fat area QTL (5720) | 13 | 53597688 | 1.2E+08 |
| ENSSSCG00000024916 | 13 | 94298790 | 94350312 | Temperature 40 minutes post mortem (loin) QTL (4005) | 13 | 80472235 | 1.75E+08 |
| ENSSSCG00000011752 | 13 | 1.11E+08 | 1.11E+08 | Loin muscle area QTL (5780) | 13 | 77716648 | 1.17E+08 |
| ENSSSCG00000011752 | 13 | 1.11E+08 | 1.11E+08 | Backfat at last rib QTL (17784) | 13 | 80245127 | 1.2E+08 |
| ENSSSCG00000011752 | 13 | 1.11E+08 | 1.11E+08 | pH 40 minutes post mortem (loin) QTL (4004) | 13 | 87815422 | 1.2E+08 |
| ENSSSCG00000011752 | 13 | 1.11E+08 | 1.11E+08 | Skin percentage QTL (5507) | 13 | 87815422 | 1.13E+08 |
| ENSSSCG00000011752 | 13 | 1.11E+08 | 1.11E+08 | backfat at P2 position QTL (4163) | 13 | 94174469 | 1.2E+08 |
| ENSSSCG00000011752 | 13 | 1.11E+08 | 1.11E+08 | Carcass length QTL (4164) | 13 | 94174469 | 1.2E+08 |
| ENSSSCG00000011752 | 13 | 1.11E+08 | 1.11E+08 | Meat color a* QTL (3074) | 13 | 94174469 | 1.2E+08 |
| ENSSSCG00000011752 | 13 | 1.11E+08 | 1.11E+08 | Loin meat weight QTL (3077) | 13 | 94174469 | 1.2E+08 |
| ENSSSCG00000011752 | 13 | 1.11E+08 | 1.11E+08 | Backfat at last rib QTL (3672) | 13 | 94174469 | 1.2E+08 |
| ENSSSCG00000011752 | 13 | 1.11E+08 | 1.11E+08 | PH for Longissmus dorsi QTL (3689) | 13 | 94174469 | 1.2E+08 |
| ENSSSCG00000011752 | 13 | 1.11E+08 | 1.11E+08 | Carcass length QTL (3690) | 13 | 94174469 | 1.2E+08 |
| ENSSSCG00000011752 | 13 | 1.11E+08 | 1.11E+08 | PH for Longissmus dorsi QTL (3692) | 13 | 94174469 | 1.2E+08 |
| ENSSSCG00000011752 | 13 | 1.11E+08 | 1.11E+08 | Meat color-a QTL (235) | 13 | 1.06E+08 | 1.2E+08 |
| ENSSSCG00000011752 | 13 | 1.11E+08 | 1.11E+08 | Meat color a* QTL (4006) | 13 | 1.07E+08 | 1.21E+08 |
| ENSSSCG00000011752 | 13 | 1.11E+08 | 1.11E+08 | LDL cholesterol QTL (17725) | 13 | 2422494 | 1.2E+08 |
| ENSSSCG00000011752 | 13 | 1.11E+08 | 1.11E+08 | Lactate dehydrogenase level QTL (15118) | 13 | 18168708 | 1.12E+08 |
| ENSSSCG00000011752 | 13 | 1.11E+08 | 1.11E+08 | Triglyceride level QTL (15078) | 13 | 21348231 | 1.84E+08 |
| ENSSSCG00000011752 | 13 | 1.11E+08 | 1.11E+08 | Arachidic acid content QTL (156954) | 13 | 21348231 | 1.84E+08 |
| ENSSSCG00000011752 | 13 | 1.11E+08 | 1.11E+08 | Linoleic acid content QTL (156953) | 13 | 21348231 | 1.54E+08 |
| ENSSSCG00000011752 | 13 | 1.11E+08 | 1.11E+08 | Polyunsaturated fatty acid content QTL (156956) | 13 | 21348231 | 1.54E+08 |
| ENSSSCG00000011752 | 13 | 1.11E+08 | 1.11E+08 | Meat color a* QTL (38109) | 13 | 28918526 | 1.73E+08 |
| ENSSSCG00000011752 | 13 | 1.11E+08 | 1.11E+08 | Shear force QTL (38107) | 13 | 28918526 | 1.17E+08 |
| ENSSSCG00000011752 | 13 | 1.11E+08 | 1.11E+08 | Palmitoleic acid content QTL (156952) | 13 | 41182404 | 1.54E+08 |
| ENSSSCG00000011752 | 13 | 1.11E+08 | 1.11E+08 | Muscle moisture percentage QTL (38108) | 13 | 41182404 | 1.17E+08 |
| ENSSSCG00000011752 | 13 | 1.11E+08 | 1.11E+08 | Capric acid content QTL (156951) | 13 | 53597688 | 1.54E+08 |
| ENSSSCG00000011752 | 13 | 1.11E+08 | 1.11E+08 | Subcutanous fat area QTL (5720) | 13 | 53597688 | 1.2E+08 |
| ENSSSCG00000011752 | 13 | 1.11E+08 | 1.11E+08 | Temperature 40 minutes post mortem (loin) QTL (4005) | 13 | 80472235 | 1.75E+08 |
| ENSSSCG00000011752 | 13 | 1.11E+08 | 1.11E+08 | Sodium level QTL (6385) | 13 | 1.06E+08 | 1.55E+08 |
| ENSSSCG00000011752 | 13 | 1.11E+08 | 1.11E+08 | Phagocytic activity QTL (21320) | 13 | 1.09E+08 | 1.46E+08 |
| ENSSSCG00000030413 | 13 | 1.17E+08 | 1.17E+08 | Loin muscle area QTL (5780) | 13 | 77716648 | 1.17E+08 |
| ENSSSCG00000030413 | 13 | 1.17E+08 | 1.17E+08 | Backfat at last rib QTL (17784) | 13 | 80245127 | 1.2E+08 |
| ENSSSCG00000030413 | 13 | 1.17E+08 | 1.17E+08 | pH 40 minutes post mortem (loin) QTL (4004) | 13 | 87815422 | 1.2E+08 |
| ENSSSCG00000030413 | 13 | 1.17E+08 | 1.17E+08 | backfat at P2 position QTL (4163) | 13 | 94174469 | 1.2E+08 |
| ENSSSCG00000030413 | 13 | 1.17E+08 | 1.17E+08 | Carcass length QTL (4164) | 13 | 94174469 | 1.2E+08 |
| ENSSSCG00000030413 | 13 | 1.17E+08 | 1.17E+08 | Meat color a* QTL (3074) | 13 | 94174469 | 1.2E+08 |
| ENSSSCG00000030413 | 13 | 1.17E+08 | 1.17E+08 | Loin meat weight QTL (3077) | 13 | 94174469 | 1.2E+08 |
| ENSSSCG00000030413 | 13 | 1.17E+08 | 1.17E+08 | Backfat at last rib QTL (3672) | 13 | 94174469 | 1.2E+08 |
| ENSSSCG00000030413 | 13 | 1.17E+08 | 1.17E+08 | PH for Longissmus dorsi QTL (3689) | 13 | 94174469 | 1.2E+08 |
| ENSSSCG00000030413 | 13 | 1.17E+08 | 1.17E+08 | Carcass length QTL (3690) | 13 | 94174469 | 1.2E+08 |
| ENSSSCG00000030413 | 13 | 1.17E+08 | 1.17E+08 | PH for Longissmus dorsi QTL (3692) | 13 | 94174469 | 1.2E+08 |
| ENSSSCG00000030413 | 13 | 1.17E+08 | 1.17E+08 | Meat color-a QTL (235) | 13 | 1.06E+08 | 1.2E+08 |
| ENSSSCG00000030413 | 13 | 1.17E+08 | 1.17E+08 | Meat color a* QTL (4006) | 13 | 1.07E+08 | 1.21E+08 |
| ENSSSCG00000030413 | 13 | 1.17E+08 | 1.17E+08 | 3 hr-9 hr pH decline QTL (5841) | 13 | 1.16E+08 | 1.19E+08 |
| ENSSSCG00000030413 | 13 | 1.17E+08 | 1.17E+08 | LDL cholesterol QTL (17725) | 13 | 2422494 | 1.2E+08 |
| ENSSSCG00000030413 | 13 | 1.17E+08 | 1.17E+08 | Triglyceride level QTL (15078) | 13 | 21348231 | 1.84E+08 |
| ENSSSCG00000030413 | 13 | 1.17E+08 | 1.17E+08 | Arachidic acid content QTL (156954) | 13 | 21348231 | 1.84E+08 |
| ENSSSCG00000030413 | 13 | 1.17E+08 | 1.17E+08 | Linoleic acid content QTL (156953) | 13 | 21348231 | 1.54E+08 |
| ENSSSCG00000030413 | 13 | 1.17E+08 | 1.17E+08 | Polyunsaturated fatty acid content QTL (156956) | 13 | 21348231 | 1.54E+08 |
| ENSSSCG00000030413 | 13 | 1.17E+08 | 1.17E+08 | Meat color a* QTL (38109) | 13 | 28918526 | 1.73E+08 |
| ENSSSCG00000030413 | 13 | 1.17E+08 | 1.17E+08 | Palmitoleic acid content QTL (156952) | 13 | 41182404 | 1.54E+08 |
| ENSSSCG00000030413 | 13 | 1.17E+08 | 1.17E+08 | Capric acid content QTL (156951) | 13 | 53597688 | 1.54E+08 |
| ENSSSCG00000030413 | 13 | 1.17E+08 | 1.17E+08 | Subcutanous fat area QTL (5720) | 13 | 53597688 | 1.2E+08 |
| ENSSSCG00000030413 | 13 | 1.17E+08 | 1.17E+08 | Temperature 40 minutes post mortem (loin) QTL (4005) | 13 | 80472235 | 1.75E+08 |
| ENSSSCG00000030413 | 13 | 1.17E+08 | 1.17E+08 | Sodium level QTL (6385) | 13 | 1.06E+08 | 1.55E+08 |
| ENSSSCG00000030413 | 13 | 1.17E+08 | 1.17E+08 | Phagocytic activity QTL (21320) | 13 | 1.09E+08 | 1.46E+08 |
| ENSSSCG00000030413 | 13 | 1.17E+08 | 1.17E+08 | Body weight (5 weeks) QTL (1136) | 13 | 1.13E+08 | 1.84E+08 |
| ENSSSCG00000030413 | 13 | 1.17E+08 | 1.17E+08 | Body weight (slaughter) QTL (1130) | 13 | 1.13E+08 | 1.54E+08 |
| ENSSSCG00000030413 | 13 | 1.17E+08 | 1.17E+08 | Backfat at last rib QTL (1135) | 13 | 1.13E+08 | 1.54E+08 |
| ENSSSCG00000030413 | 13 | 1.17E+08 | 1.17E+08 | Adipocyte diameter QTL (12814) | 13 | 1.13E+08 | 1.54E+08 |
| ENSSSCG00000030413 | 13 | 1.17E+08 | 1.17E+08 | Adipocyte diameter QTL (12845) | 13 | 1.13E+08 | 1.54E+08 |
| ENSSSCG00000030413 | 13 | 1.17E+08 | 1.17E+08 | Cholesterol level QTL (15077) | 13 | 1.17E+08 | 1.84E+08 |
| ENSSSCG00000011765 | 13 | 1.18E+08 | 1.18E+08 | Backfat at last rib QTL (17784) | 13 | 80245127 | 1.2E+08 |
| ENSSSCG00000011765 | 13 | 1.18E+08 | 1.18E+08 | pH 40 minutes post mortem (loin) QTL (4004) | 13 | 87815422 | 1.2E+08 |
| ENSSSCG00000011765 | 13 | 1.18E+08 | 1.18E+08 | backfat at P2 position QTL (4163) | 13 | 94174469 | 1.2E+08 |
| ENSSSCG00000011765 | 13 | 1.18E+08 | 1.18E+08 | Carcass length QTL (4164) | 13 | 94174469 | 1.2E+08 |
| ENSSSCG00000011765 | 13 | 1.18E+08 | 1.18E+08 | Meat color a* QTL (3074) | 13 | 94174469 | 1.2E+08 |
| ENSSSCG00000011765 | 13 | 1.18E+08 | 1.18E+08 | Loin meat weight QTL (3077) | 13 | 94174469 | 1.2E+08 |
| ENSSSCG00000011765 | 13 | 1.18E+08 | 1.18E+08 | Backfat at last rib QTL (3672) | 13 | 94174469 | 1.2E+08 |
| ENSSSCG00000011765 | 13 | 1.18E+08 | 1.18E+08 | PH for Longissmus dorsi QTL (3689) | 13 | 94174469 | 1.2E+08 |
| ENSSSCG00000011765 | 13 | 1.18E+08 | 1.18E+08 | Carcass length QTL (3690) | 13 | 94174469 | 1.2E+08 |
| ENSSSCG00000011765 | 13 | 1.18E+08 | 1.18E+08 | PH for Longissmus dorsi QTL (3692) | 13 | 94174469 | 1.2E+08 |
| ENSSSCG00000011765 | 13 | 1.18E+08 | 1.18E+08 | Meat color-a QTL (235) | 13 | 1.06E+08 | 1.2E+08 |
| ENSSSCG00000011765 | 13 | 1.18E+08 | 1.18E+08 | Meat color a* QTL (4006) | 13 | 1.07E+08 | 1.21E+08 |
| ENSSSCG00000011765 | 13 | 1.18E+08 | 1.18E+08 | 3 hr-9 hr pH decline QTL (5841) | 13 | 1.16E+08 | 1.19E+08 |
| ENSSSCG00000011765 | 13 | 1.18E+08 | 1.18E+08 | LDL cholesterol QTL (17725) | 13 | 2422494 | 1.2E+08 |
| ENSSSCG00000011765 | 13 | 1.18E+08 | 1.18E+08 | Triglyceride level QTL (15078) | 13 | 21348231 | 1.84E+08 |
| ENSSSCG00000011765 | 13 | 1.18E+08 | 1.18E+08 | Arachidic acid content QTL (156954) | 13 | 21348231 | 1.84E+08 |
| ENSSSCG00000011765 | 13 | 1.18E+08 | 1.18E+08 | Linoleic acid content QTL (156953) | 13 | 21348231 | 1.54E+08 |
| ENSSSCG00000011765 | 13 | 1.18E+08 | 1.18E+08 | Polyunsaturated fatty acid content QTL (156956) | 13 | 21348231 | 1.54E+08 |
| ENSSSCG00000011765 | 13 | 1.18E+08 | 1.18E+08 | Meat color a* QTL (38109) | 13 | 28918526 | 1.73E+08 |
| ENSSSCG00000011765 | 13 | 1.18E+08 | 1.18E+08 | Palmitoleic acid content QTL (156952) | 13 | 41182404 | 1.54E+08 |
| ENSSSCG00000011765 | 13 | 1.18E+08 | 1.18E+08 | Capric acid content QTL (156951) | 13 | 53597688 | 1.54E+08 |
| ENSSSCG00000011765 | 13 | 1.18E+08 | 1.18E+08 | Subcutanous fat area QTL (5720) | 13 | 53597688 | 1.2E+08 |
| ENSSSCG00000011765 | 13 | 1.18E+08 | 1.18E+08 | Temperature 40 minutes post mortem (loin) QTL (4005) | 13 | 80472235 | 1.75E+08 |
| ENSSSCG00000011765 | 13 | 1.18E+08 | 1.18E+08 | Sodium level QTL (6385) | 13 | 1.06E+08 | 1.55E+08 |
| ENSSSCG00000011765 | 13 | 1.18E+08 | 1.18E+08 | Phagocytic activity QTL (21320) | 13 | 1.09E+08 | 1.46E+08 |
| ENSSSCG00000011765 | 13 | 1.18E+08 | 1.18E+08 | Body weight (5 weeks) QTL (1136) | 13 | 1.13E+08 | 1.84E+08 |
| ENSSSCG00000011765 | 13 | 1.18E+08 | 1.18E+08 | Body weight (slaughter) QTL (1130) | 13 | 1.13E+08 | 1.54E+08 |
| ENSSSCG00000011765 | 13 | 1.18E+08 | 1.18E+08 | Backfat at last rib QTL (1135) | 13 | 1.13E+08 | 1.54E+08 |
| ENSSSCG00000011765 | 13 | 1.18E+08 | 1.18E+08 | Adipocyte diameter QTL (12814) | 13 | 1.13E+08 | 1.54E+08 |
| ENSSSCG00000011765 | 13 | 1.18E+08 | 1.18E+08 | Adipocyte diameter QTL (12845) | 13 | 1.13E+08 | 1.54E+08 |
| ENSSSCG00000011765 | 13 | 1.18E+08 | 1.18E+08 | Cholesterol level QTL (15077) | 13 | 1.17E+08 | 1.84E+08 |
| ENSSSCG00000011765 | 13 | 1.18E+08 | 1.18E+08 | Protein accretion rate QTL (2883) | 13 | 1.17E+08 | 1.75E+08 |
| ENSSSCG00000011775 | 13 | 1.22E+08 | 1.22E+08 | Triglyceride level QTL (15078) | 13 | 21348231 | 1.84E+08 |
| ENSSSCG00000011775 | 13 | 1.22E+08 | 1.22E+08 | Arachidic acid content QTL (156954) | 13 | 21348231 | 1.84E+08 |
| ENSSSCG00000011775 | 13 | 1.22E+08 | 1.22E+08 | Linoleic acid content QTL (156953) | 13 | 21348231 | 1.54E+08 |
| ENSSSCG00000011775 | 13 | 1.22E+08 | 1.22E+08 | Polyunsaturated fatty acid content QTL (156956) | 13 | 21348231 | 1.54E+08 |
| ENSSSCG00000011775 | 13 | 1.22E+08 | 1.22E+08 | Meat color a* QTL (38109) | 13 | 28918526 | 1.73E+08 |
| ENSSSCG00000011775 | 13 | 1.22E+08 | 1.22E+08 | Palmitoleic acid content QTL (156952) | 13 | 41182404 | 1.54E+08 |
| ENSSSCG00000011775 | 13 | 1.22E+08 | 1.22E+08 | Capric acid content QTL (156951) | 13 | 53597688 | 1.54E+08 |
| ENSSSCG00000011775 | 13 | 1.22E+08 | 1.22E+08 | Temperature 40 minutes post mortem (loin) QTL (4005) | 13 | 80472235 | 1.75E+08 |
| ENSSSCG00000011775 | 13 | 1.22E+08 | 1.22E+08 | Sodium level QTL (6385) | 13 | 1.06E+08 | 1.55E+08 |
| ENSSSCG00000011775 | 13 | 1.22E+08 | 1.22E+08 | Phagocytic activity QTL (21320) | 13 | 1.09E+08 | 1.46E+08 |
| ENSSSCG00000011775 | 13 | 1.22E+08 | 1.22E+08 | Body weight (5 weeks) QTL (1136) | 13 | 1.13E+08 | 1.84E+08 |
| ENSSSCG00000011775 | 13 | 1.22E+08 | 1.22E+08 | Body weight (slaughter) QTL (1130) | 13 | 1.13E+08 | 1.54E+08 |
| ENSSSCG00000011775 | 13 | 1.22E+08 | 1.22E+08 | Backfat at last rib QTL (1135) | 13 | 1.13E+08 | 1.54E+08 |
| ENSSSCG00000011775 | 13 | 1.22E+08 | 1.22E+08 | Adipocyte diameter QTL (12814) | 13 | 1.13E+08 | 1.54E+08 |
| ENSSSCG00000011775 | 13 | 1.22E+08 | 1.22E+08 | Adipocyte diameter QTL (12845) | 13 | 1.13E+08 | 1.54E+08 |
| ENSSSCG00000011775 | 13 | 1.22E+08 | 1.22E+08 | Cholesterol level QTL (15077) | 13 | 1.17E+08 | 1.84E+08 |
| ENSSSCG00000011775 | 13 | 1.22E+08 | 1.22E+08 | Protein accretion rate QTL (2883) | 13 | 1.17E+08 | 1.75E+08 |
| ENSSSCG00000011775 | 13 | 1.22E+08 | 1.22E+08 | Average daily gain QTL (5950) | 13 | 1.2E+08 | 1.7E+08 |
| ENSSSCG00000011775 | 13 | 1.22E+08 | 1.22E+08 | Meat color a* QTL (21847) | 13 | 1.2E+08 | 1.55E+08 |
| ENSSSCG00000011775 | 13 | 1.22E+08 | 1.22E+08 | Meat color a* QTL (21844) | 13 | 1.2E+08 | 1.55E+08 |
| ENSSSCG00000011775 | 13 | 1.22E+08 | 1.22E+08 | Backfat at last rib QTL (21404) | 13 | 1.2E+08 | 1.55E+08 |
| ENSSSCG00000011775 | 13 | 1.22E+08 | 1.22E+08 | Shear force QTL (224) | 13 | 1.2E+08 | 1.54E+08 |
| ENSSSCG00000011775 | 13 | 1.22E+08 | 1.22E+08 | Interleukin-10 level QTL (12270) | 13 | 1.2E+08 | 1.46E+08 |
| ENSSSCG00000011775 | 13 | 1.22E+08 | 1.22E+08 | subjective abnormal flavor in lean QTL (4229) | 13 | 1.2E+08 | 1.46E+08 |
| ENSSSCG00000011811 | 13 | 1.26E+08 | 1.26E+08 | Triglyceride level QTL (32732) | 13 | 1.26E+08 | 1.27E+08 |
| ENSSSCG00000011811 | 13 | 1.26E+08 | 1.26E+08 | Cholesterol level QTL (32733) | 13 | 1.26E+08 | 1.27E+08 |
| ENSSSCG00000011811 | 13 | 1.26E+08 | 1.26E+08 | LDL cholesterol QTL (32734) | 13 | 1.26E+08 | 1.27E+08 |
| ENSSSCG00000011811 | 13 | 1.26E+08 | 1.26E+08 | Loin muscle width QTL (30913) | 13 | 1.26E+08 | 1.27E+08 |
| ENSSSCG00000011811 | 13 | 1.26E+08 | 1.26E+08 | Loin muscle depth QTL (30914) | 13 | 1.26E+08 | 1.27E+08 |
| ENSSSCG00000011811 | 13 | 1.26E+08 | 1.26E+08 | Loin muscle width QTL (30915) | 13 | 1.26E+08 | 1.27E+08 |
| ENSSSCG00000011811 | 13 | 1.26E+08 | 1.26E+08 | Loin muscle area QTL (30916) | 13 | 1.26E+08 | 1.27E+08 |
| ENSSSCG00000011811 | 13 | 1.26E+08 | 1.26E+08 | Flavor score QTL (28188) | 13 | 1.26E+08 | 1.27E+08 |
| ENSSSCG00000011811 | 13 | 1.26E+08 | 1.26E+08 | Overall impression, sensory panel QTL (28190) | 13 | 1.26E+08 | 1.27E+08 |
| ENSSSCG00000011811 | 13 | 1.26E+08 | 1.26E+08 | Palmitoleic acid content QTL (7192) | 13 | 1.26E+08 | 1.27E+08 |
| ENSSSCG00000011811 | 13 | 1.26E+08 | 1.26E+08 | Triglyceride level QTL (15078) | 13 | 21348231 | 1.84E+08 |
| ENSSSCG00000011811 | 13 | 1.26E+08 | 1.26E+08 | Arachidic acid content QTL (156954) | 13 | 21348231 | 1.84E+08 |
| ENSSSCG00000011811 | 13 | 1.26E+08 | 1.26E+08 | Linoleic acid content QTL (156953) | 13 | 21348231 | 1.54E+08 |
| ENSSSCG00000011811 | 13 | 1.26E+08 | 1.26E+08 | Polyunsaturated fatty acid content QTL (156956) | 13 | 21348231 | 1.54E+08 |
| ENSSSCG00000011811 | 13 | 1.26E+08 | 1.26E+08 | Meat color a* QTL (38109) | 13 | 28918526 | 1.73E+08 |
| ENSSSCG00000011811 | 13 | 1.26E+08 | 1.26E+08 | Palmitoleic acid content QTL (156952) | 13 | 41182404 | 1.54E+08 |
| ENSSSCG00000011811 | 13 | 1.26E+08 | 1.26E+08 | Capric acid content QTL (156951) | 13 | 53597688 | 1.54E+08 |
| ENSSSCG00000011811 | 13 | 1.26E+08 | 1.26E+08 | Temperature 40 minutes post mortem (loin) QTL (4005) | 13 | 80472235 | 1.75E+08 |
| ENSSSCG00000011811 | 13 | 1.26E+08 | 1.26E+08 | Sodium level QTL (6385) | 13 | 1.06E+08 | 1.55E+08 |
| ENSSSCG00000011811 | 13 | 1.26E+08 | 1.26E+08 | Phagocytic activity QTL (21320) | 13 | 1.09E+08 | 1.46E+08 |
| ENSSSCG00000011811 | 13 | 1.26E+08 | 1.26E+08 | Body weight (5 weeks) QTL (1136) | 13 | 1.13E+08 | 1.84E+08 |
| ENSSSCG00000011811 | 13 | 1.26E+08 | 1.26E+08 | Body weight (slaughter) QTL (1130) | 13 | 1.13E+08 | 1.54E+08 |
| ENSSSCG00000011811 | 13 | 1.26E+08 | 1.26E+08 | Backfat at last rib QTL (1135) | 13 | 1.13E+08 | 1.54E+08 |
| ENSSSCG00000011811 | 13 | 1.26E+08 | 1.26E+08 | Adipocyte diameter QTL (12814) | 13 | 1.13E+08 | 1.54E+08 |
| ENSSSCG00000011811 | 13 | 1.26E+08 | 1.26E+08 | Adipocyte diameter QTL (12845) | 13 | 1.13E+08 | 1.54E+08 |
| ENSSSCG00000011811 | 13 | 1.26E+08 | 1.26E+08 | Cholesterol level QTL (15077) | 13 | 1.17E+08 | 1.84E+08 |
| ENSSSCG00000011811 | 13 | 1.26E+08 | 1.26E+08 | Protein accretion rate QTL (2883) | 13 | 1.17E+08 | 1.75E+08 |
| ENSSSCG00000011811 | 13 | 1.26E+08 | 1.26E+08 | Average daily gain QTL (5950) | 13 | 1.2E+08 | 1.7E+08 |
| ENSSSCG00000011811 | 13 | 1.26E+08 | 1.26E+08 | Meat color a* QTL (21847) | 13 | 1.2E+08 | 1.55E+08 |
| ENSSSCG00000011811 | 13 | 1.26E+08 | 1.26E+08 | Meat color a* QTL (21844) | 13 | 1.2E+08 | 1.55E+08 |
| ENSSSCG00000011811 | 13 | 1.26E+08 | 1.26E+08 | Backfat at last rib QTL (21404) | 13 | 1.2E+08 | 1.55E+08 |
| ENSSSCG00000011811 | 13 | 1.26E+08 | 1.26E+08 | Shear force QTL (224) | 13 | 1.2E+08 | 1.54E+08 |
| ENSSSCG00000011811 | 13 | 1.26E+08 | 1.26E+08 | Interleukin-10 level QTL (12270) | 13 | 1.2E+08 | 1.46E+08 |
| ENSSSCG00000011811 | 13 | 1.26E+08 | 1.26E+08 | subjective abnormal flavor in lean QTL (4229) | 13 | 1.2E+08 | 1.46E+08 |
| ENSSSCG00000011845 | 13 | 1.34E+08 | 1.34E+08 | Triglyceride level QTL (15078) | 13 | 21348231 | 1.84E+08 |
| ENSSSCG00000011845 | 13 | 1.34E+08 | 1.34E+08 | Arachidic acid content QTL (156954) | 13 | 21348231 | 1.84E+08 |
| ENSSSCG00000011845 | 13 | 1.34E+08 | 1.34E+08 | Linoleic acid content QTL (156953) | 13 | 21348231 | 1.54E+08 |
| ENSSSCG00000011845 | 13 | 1.34E+08 | 1.34E+08 | Polyunsaturated fatty acid content QTL (156956) | 13 | 21348231 | 1.54E+08 |
| ENSSSCG00000011845 | 13 | 1.34E+08 | 1.34E+08 | Meat color a* QTL (38109) | 13 | 28918526 | 1.73E+08 |
| ENSSSCG00000011845 | 13 | 1.34E+08 | 1.34E+08 | Palmitoleic acid content QTL (156952) | 13 | 41182404 | 1.54E+08 |
| ENSSSCG00000011845 | 13 | 1.34E+08 | 1.34E+08 | Capric acid content QTL (156951) | 13 | 53597688 | 1.54E+08 |
| ENSSSCG00000011845 | 13 | 1.34E+08 | 1.34E+08 | Temperature 40 minutes post mortem (loin) QTL (4005) | 13 | 80472235 | 1.75E+08 |
| ENSSSCG00000011845 | 13 | 1.34E+08 | 1.34E+08 | Sodium level QTL (6385) | 13 | 1.06E+08 | 1.55E+08 |
| ENSSSCG00000011845 | 13 | 1.34E+08 | 1.34E+08 | Phagocytic activity QTL (21320) | 13 | 1.09E+08 | 1.46E+08 |
| ENSSSCG00000011845 | 13 | 1.34E+08 | 1.34E+08 | Body weight (5 weeks) QTL (1136) | 13 | 1.13E+08 | 1.84E+08 |
| ENSSSCG00000011845 | 13 | 1.34E+08 | 1.34E+08 | Body weight (slaughter) QTL (1130) | 13 | 1.13E+08 | 1.54E+08 |
| ENSSSCG00000011845 | 13 | 1.34E+08 | 1.34E+08 | Backfat at last rib QTL (1135) | 13 | 1.13E+08 | 1.54E+08 |
| ENSSSCG00000011845 | 13 | 1.34E+08 | 1.34E+08 | Adipocyte diameter QTL (12814) | 13 | 1.13E+08 | 1.54E+08 |
| ENSSSCG00000011845 | 13 | 1.34E+08 | 1.34E+08 | Adipocyte diameter QTL (12845) | 13 | 1.13E+08 | 1.54E+08 |
| ENSSSCG00000011845 | 13 | 1.34E+08 | 1.34E+08 | Cholesterol level QTL (15077) | 13 | 1.17E+08 | 1.84E+08 |
| ENSSSCG00000011845 | 13 | 1.34E+08 | 1.34E+08 | Protein accretion rate QTL (2883) | 13 | 1.17E+08 | 1.75E+08 |
| ENSSSCG00000011845 | 13 | 1.34E+08 | 1.34E+08 | Average daily gain QTL (5950) | 13 | 1.2E+08 | 1.7E+08 |
| ENSSSCG00000011845 | 13 | 1.34E+08 | 1.34E+08 | Meat color a* QTL (21847) | 13 | 1.2E+08 | 1.55E+08 |
| ENSSSCG00000011845 | 13 | 1.34E+08 | 1.34E+08 | Meat color a* QTL (21844) | 13 | 1.2E+08 | 1.55E+08 |
| ENSSSCG00000011845 | 13 | 1.34E+08 | 1.34E+08 | Backfat at last rib QTL (21404) | 13 | 1.2E+08 | 1.55E+08 |
| ENSSSCG00000011845 | 13 | 1.34E+08 | 1.34E+08 | Shear force QTL (224) | 13 | 1.2E+08 | 1.54E+08 |
| ENSSSCG00000011845 | 13 | 1.34E+08 | 1.34E+08 | Interleukin-10 level QTL (12270) | 13 | 1.2E+08 | 1.46E+08 |
| ENSSSCG00000011845 | 13 | 1.34E+08 | 1.34E+08 | subjective abnormal flavor in lean QTL (4229) | 13 | 1.2E+08 | 1.46E+08 |
| ENSSSCG00000036828 | 13 | 1.35E+08 | 1.35E+08 | Average daily gain QTL (24021) | 13 | 1.35E+08 | 1.35E+08 |
| ENSSSCG00000036828 | 13 | 1.35E+08 | 1.35E+08 | Backfat at rump QTL (24022) | 13 | 1.35E+08 | 1.35E+08 |
| ENSSSCG00000036828 | 13 | 1.35E+08 | 1.35E+08 | Average daily gain QTL (24023) | 13 | 1.35E+08 | 1.35E+08 |
| ENSSSCG00000036828 | 13 | 1.35E+08 | 1.35E+08 | Triglyceride level QTL (15078) | 13 | 21348231 | 1.84E+08 |
| ENSSSCG00000036828 | 13 | 1.35E+08 | 1.35E+08 | Arachidic acid content QTL (156954) | 13 | 21348231 | 1.84E+08 |
| ENSSSCG00000036828 | 13 | 1.35E+08 | 1.35E+08 | Linoleic acid content QTL (156953) | 13 | 21348231 | 1.54E+08 |
| ENSSSCG00000036828 | 13 | 1.35E+08 | 1.35E+08 | Polyunsaturated fatty acid content QTL (156956) | 13 | 21348231 | 1.54E+08 |
| ENSSSCG00000036828 | 13 | 1.35E+08 | 1.35E+08 | Meat color a* QTL (38109) | 13 | 28918526 | 1.73E+08 |
| ENSSSCG00000036828 | 13 | 1.35E+08 | 1.35E+08 | Palmitoleic acid content QTL (156952) | 13 | 41182404 | 1.54E+08 |
| ENSSSCG00000036828 | 13 | 1.35E+08 | 1.35E+08 | Capric acid content QTL (156951) | 13 | 53597688 | 1.54E+08 |
| ENSSSCG00000036828 | 13 | 1.35E+08 | 1.35E+08 | Temperature 40 minutes post mortem (loin) QTL (4005) | 13 | 80472235 | 1.75E+08 |
| ENSSSCG00000036828 | 13 | 1.35E+08 | 1.35E+08 | Sodium level QTL (6385) | 13 | 1.06E+08 | 1.55E+08 |
| ENSSSCG00000036828 | 13 | 1.35E+08 | 1.35E+08 | Phagocytic activity QTL (21320) | 13 | 1.09E+08 | 1.46E+08 |
| ENSSSCG00000036828 | 13 | 1.35E+08 | 1.35E+08 | Body weight (5 weeks) QTL (1136) | 13 | 1.13E+08 | 1.84E+08 |
| ENSSSCG00000036828 | 13 | 1.35E+08 | 1.35E+08 | Body weight (slaughter) QTL (1130) | 13 | 1.13E+08 | 1.54E+08 |
| ENSSSCG00000036828 | 13 | 1.35E+08 | 1.35E+08 | Backfat at last rib QTL (1135) | 13 | 1.13E+08 | 1.54E+08 |
| ENSSSCG00000036828 | 13 | 1.35E+08 | 1.35E+08 | Adipocyte diameter QTL (12814) | 13 | 1.13E+08 | 1.54E+08 |
| ENSSSCG00000036828 | 13 | 1.35E+08 | 1.35E+08 | Adipocyte diameter QTL (12845) | 13 | 1.13E+08 | 1.54E+08 |
| ENSSSCG00000036828 | 13 | 1.35E+08 | 1.35E+08 | Cholesterol level QTL (15077) | 13 | 1.17E+08 | 1.84E+08 |
| ENSSSCG00000036828 | 13 | 1.35E+08 | 1.35E+08 | Protein accretion rate QTL (2883) | 13 | 1.17E+08 | 1.75E+08 |
| ENSSSCG00000036828 | 13 | 1.35E+08 | 1.35E+08 | Average daily gain QTL (5950) | 13 | 1.2E+08 | 1.7E+08 |
| ENSSSCG00000036828 | 13 | 1.35E+08 | 1.35E+08 | Meat color a* QTL (21847) | 13 | 1.2E+08 | 1.55E+08 |
| ENSSSCG00000036828 | 13 | 1.35E+08 | 1.35E+08 | Meat color a* QTL (21844) | 13 | 1.2E+08 | 1.55E+08 |
| ENSSSCG00000036828 | 13 | 1.35E+08 | 1.35E+08 | Backfat at last rib QTL (21404) | 13 | 1.2E+08 | 1.55E+08 |
| ENSSSCG00000036828 | 13 | 1.35E+08 | 1.35E+08 | Shear force QTL (224) | 13 | 1.2E+08 | 1.54E+08 |
| ENSSSCG00000036828 | 13 | 1.35E+08 | 1.35E+08 | Interleukin-10 level QTL (12270) | 13 | 1.2E+08 | 1.46E+08 |
| ENSSSCG00000036828 | 13 | 1.35E+08 | 1.35E+08 | subjective abnormal flavor in lean QTL (4229) | 13 | 1.2E+08 | 1.46E+08 |
| ENSSSCG00000011858 | 13 | 1.35E+08 | 1.35E+08 | Average daily gain QTL (24021) | 13 | 1.35E+08 | 1.35E+08 |
| ENSSSCG00000011858 | 13 | 1.35E+08 | 1.35E+08 | Backfat at rump QTL (24022) | 13 | 1.35E+08 | 1.35E+08 |
| ENSSSCG00000011858 | 13 | 1.35E+08 | 1.35E+08 | Average daily gain QTL (24023) | 13 | 1.35E+08 | 1.35E+08 |
| ENSSSCG00000011858 | 13 | 1.35E+08 | 1.35E+08 | Triglyceride level QTL (15078) | 13 | 21348231 | 1.84E+08 |
| ENSSSCG00000011858 | 13 | 1.35E+08 | 1.35E+08 | Arachidic acid content QTL (156954) | 13 | 21348231 | 1.84E+08 |
| ENSSSCG00000011858 | 13 | 1.35E+08 | 1.35E+08 | Linoleic acid content QTL (156953) | 13 | 21348231 | 1.54E+08 |
| ENSSSCG00000011858 | 13 | 1.35E+08 | 1.35E+08 | Polyunsaturated fatty acid content QTL (156956) | 13 | 21348231 | 1.54E+08 |
| ENSSSCG00000011858 | 13 | 1.35E+08 | 1.35E+08 | Meat color a* QTL (38109) | 13 | 28918526 | 1.73E+08 |
| ENSSSCG00000011858 | 13 | 1.35E+08 | 1.35E+08 | Palmitoleic acid content QTL (156952) | 13 | 41182404 | 1.54E+08 |
| ENSSSCG00000011858 | 13 | 1.35E+08 | 1.35E+08 | Capric acid content QTL (156951) | 13 | 53597688 | 1.54E+08 |
| ENSSSCG00000011858 | 13 | 1.35E+08 | 1.35E+08 | Temperature 40 minutes post mortem (loin) QTL (4005) | 13 | 80472235 | 1.75E+08 |
| ENSSSCG00000011858 | 13 | 1.35E+08 | 1.35E+08 | Sodium level QTL (6385) | 13 | 1.06E+08 | 1.55E+08 |
| ENSSSCG00000011858 | 13 | 1.35E+08 | 1.35E+08 | Phagocytic activity QTL (21320) | 13 | 1.09E+08 | 1.46E+08 |
| ENSSSCG00000011858 | 13 | 1.35E+08 | 1.35E+08 | Body weight (5 weeks) QTL (1136) | 13 | 1.13E+08 | 1.84E+08 |
| ENSSSCG00000011858 | 13 | 1.35E+08 | 1.35E+08 | Body weight (slaughter) QTL (1130) | 13 | 1.13E+08 | 1.54E+08 |
| ENSSSCG00000011858 | 13 | 1.35E+08 | 1.35E+08 | Backfat at last rib QTL (1135) | 13 | 1.13E+08 | 1.54E+08 |
| ENSSSCG00000011858 | 13 | 1.35E+08 | 1.35E+08 | Adipocyte diameter QTL (12814) | 13 | 1.13E+08 | 1.54E+08 |
| ENSSSCG00000011858 | 13 | 1.35E+08 | 1.35E+08 | Adipocyte diameter QTL (12845) | 13 | 1.13E+08 | 1.54E+08 |
| ENSSSCG00000011858 | 13 | 1.35E+08 | 1.35E+08 | Cholesterol level QTL (15077) | 13 | 1.17E+08 | 1.84E+08 |
| ENSSSCG00000011858 | 13 | 1.35E+08 | 1.35E+08 | Protein accretion rate QTL (2883) | 13 | 1.17E+08 | 1.75E+08 |
| ENSSSCG00000011858 | 13 | 1.35E+08 | 1.35E+08 | Average daily gain QTL (5950) | 13 | 1.2E+08 | 1.7E+08 |
| ENSSSCG00000011858 | 13 | 1.35E+08 | 1.35E+08 | Meat color a* QTL (21847) | 13 | 1.2E+08 | 1.55E+08 |
| ENSSSCG00000011858 | 13 | 1.35E+08 | 1.35E+08 | Meat color a* QTL (21844) | 13 | 1.2E+08 | 1.55E+08 |
| ENSSSCG00000011858 | 13 | 1.35E+08 | 1.35E+08 | Backfat at last rib QTL (21404) | 13 | 1.2E+08 | 1.55E+08 |
| ENSSSCG00000011858 | 13 | 1.35E+08 | 1.35E+08 | Shear force QTL (224) | 13 | 1.2E+08 | 1.54E+08 |
| ENSSSCG00000011858 | 13 | 1.35E+08 | 1.35E+08 | Interleukin-10 level QTL (12270) | 13 | 1.2E+08 | 1.46E+08 |
| ENSSSCG00000011858 | 13 | 1.35E+08 | 1.35E+08 | subjective abnormal flavor in lean QTL (4229) | 13 | 1.2E+08 | 1.46E+08 |
| ENSSSCG00000011889 | 13 | 1.4E+08 | 1.4E+08 | Triglyceride level QTL (15078) | 13 | 21348231 | 1.84E+08 |
| ENSSSCG00000011889 | 13 | 1.4E+08 | 1.4E+08 | Arachidic acid content QTL (156954) | 13 | 21348231 | 1.84E+08 |
| ENSSSCG00000011889 | 13 | 1.4E+08 | 1.4E+08 | Linoleic acid content QTL (156953) | 13 | 21348231 | 1.54E+08 |
| ENSSSCG00000011889 | 13 | 1.4E+08 | 1.4E+08 | Polyunsaturated fatty acid content QTL (156956) | 13 | 21348231 | 1.54E+08 |
| ENSSSCG00000011889 | 13 | 1.4E+08 | 1.4E+08 | Meat color a* QTL (38109) | 13 | 28918526 | 1.73E+08 |
| ENSSSCG00000011889 | 13 | 1.4E+08 | 1.4E+08 | Palmitoleic acid content QTL (156952) | 13 | 41182404 | 1.54E+08 |
| ENSSSCG00000011889 | 13 | 1.4E+08 | 1.4E+08 | Capric acid content QTL (156951) | 13 | 53597688 | 1.54E+08 |
| ENSSSCG00000011889 | 13 | 1.4E+08 | 1.4E+08 | Temperature 40 minutes post mortem (loin) QTL (4005) | 13 | 80472235 | 1.75E+08 |
| ENSSSCG00000011889 | 13 | 1.4E+08 | 1.4E+08 | Sodium level QTL (6385) | 13 | 1.06E+08 | 1.55E+08 |
| ENSSSCG00000011889 | 13 | 1.4E+08 | 1.4E+08 | Phagocytic activity QTL (21320) | 13 | 1.09E+08 | 1.46E+08 |
| ENSSSCG00000011889 | 13 | 1.4E+08 | 1.4E+08 | Body weight (5 weeks) QTL (1136) | 13 | 1.13E+08 | 1.84E+08 |
| ENSSSCG00000011889 | 13 | 1.4E+08 | 1.4E+08 | Body weight (slaughter) QTL (1130) | 13 | 1.13E+08 | 1.54E+08 |
| ENSSSCG00000011889 | 13 | 1.4E+08 | 1.4E+08 | Backfat at last rib QTL (1135) | 13 | 1.13E+08 | 1.54E+08 |
| ENSSSCG00000011889 | 13 | 1.4E+08 | 1.4E+08 | Adipocyte diameter QTL (12814) | 13 | 1.13E+08 | 1.54E+08 |
| ENSSSCG00000011889 | 13 | 1.4E+08 | 1.4E+08 | Adipocyte diameter QTL (12845) | 13 | 1.13E+08 | 1.54E+08 |
| ENSSSCG00000011889 | 13 | 1.4E+08 | 1.4E+08 | Cholesterol level QTL (15077) | 13 | 1.17E+08 | 1.84E+08 |
| ENSSSCG00000011889 | 13 | 1.4E+08 | 1.4E+08 | Protein accretion rate QTL (2883) | 13 | 1.17E+08 | 1.75E+08 |
| ENSSSCG00000011889 | 13 | 1.4E+08 | 1.4E+08 | Average daily gain QTL (5950) | 13 | 1.2E+08 | 1.7E+08 |
| ENSSSCG00000011889 | 13 | 1.4E+08 | 1.4E+08 | Meat color a* QTL (21847) | 13 | 1.2E+08 | 1.55E+08 |
| ENSSSCG00000011889 | 13 | 1.4E+08 | 1.4E+08 | Meat color a* QTL (21844) | 13 | 1.2E+08 | 1.55E+08 |
| ENSSSCG00000011889 | 13 | 1.4E+08 | 1.4E+08 | Backfat at last rib QTL (21404) | 13 | 1.2E+08 | 1.55E+08 |
| ENSSSCG00000011889 | 13 | 1.4E+08 | 1.4E+08 | Shear force QTL (224) | 13 | 1.2E+08 | 1.54E+08 |
| ENSSSCG00000011889 | 13 | 1.4E+08 | 1.4E+08 | Interleukin-10 level QTL (12270) | 13 | 1.2E+08 | 1.46E+08 |
| ENSSSCG00000011889 | 13 | 1.4E+08 | 1.4E+08 | subjective abnormal flavor in lean QTL (4229) | 13 | 1.2E+08 | 1.46E+08 |
| ENSSSCG00000011949 | 13 | 1.57E+08 | 1.57E+08 | Cortisol level QTL (21329) | 13 | 1.46E+08 | 1.91E+08 |
| ENSSSCG00000011949 | 13 | 1.57E+08 | 1.57E+08 | subjective abnormal odor QTL (4230) | 13 | 1.46E+08 | 1.84E+08 |
| ENSSSCG00000011949 | 13 | 1.57E+08 | 1.57E+08 | subjective pork odor QTL (4231) | 13 | 1.46E+08 | 1.84E+08 |
| ENSSSCG00000011949 | 13 | 1.57E+08 | 1.57E+08 | Percentage type IIa fibers QTL (7032) | 13 | 1.46E+08 | 1.78E+08 |
| ENSSSCG00000011949 | 13 | 1.57E+08 | 1.57E+08 | Calcium level QTL (6377) | 13 | 1.55E+08 | 1.84E+08 |
| ENSSSCG00000011949 | 13 | 1.57E+08 | 1.57E+08 | Triglyceride level QTL (15078) | 13 | 21348231 | 1.84E+08 |
| ENSSSCG00000011949 | 13 | 1.57E+08 | 1.57E+08 | Arachidic acid content QTL (156954) | 13 | 21348231 | 1.84E+08 |
| ENSSSCG00000011949 | 13 | 1.57E+08 | 1.57E+08 | Meat color a* QTL (38109) | 13 | 28918526 | 1.73E+08 |
| ENSSSCG00000011949 | 13 | 1.57E+08 | 1.57E+08 | Temperature 40 minutes post mortem (loin) QTL (4005) | 13 | 80472235 | 1.75E+08 |
| ENSSSCG00000011949 | 13 | 1.57E+08 | 1.57E+08 | Body weight (5 weeks) QTL (1136) | 13 | 1.13E+08 | 1.84E+08 |
| ENSSSCG00000011949 | 13 | 1.57E+08 | 1.57E+08 | Cholesterol level QTL (15077) | 13 | 1.17E+08 | 1.84E+08 |
| ENSSSCG00000011949 | 13 | 1.57E+08 | 1.57E+08 | Protein accretion rate QTL (2883) | 13 | 1.17E+08 | 1.75E+08 |
| ENSSSCG00000011949 | 13 | 1.57E+08 | 1.57E+08 | Average daily gain QTL (5950) | 13 | 1.2E+08 | 1.7E+08 |
| ENSSSCG00000011949 | 13 | 1.57E+08 | 1.57E+08 | Body weight (5 weeks) QTL (1139) | 13 | 1.54E+08 | 2.08E+08 |
| ENSSSCG00000021791 | 13 | 1.58E+08 | 1.58E+08 | Cortisol level QTL (21329) | 13 | 1.46E+08 | 1.91E+08 |
| ENSSSCG00000021791 | 13 | 1.58E+08 | 1.58E+08 | subjective abnormal odor QTL (4230) | 13 | 1.46E+08 | 1.84E+08 |
| ENSSSCG00000021791 | 13 | 1.58E+08 | 1.58E+08 | subjective pork odor QTL (4231) | 13 | 1.46E+08 | 1.84E+08 |
| ENSSSCG00000021791 | 13 | 1.58E+08 | 1.58E+08 | Percentage type IIa fibers QTL (7032) | 13 | 1.46E+08 | 1.78E+08 |
| ENSSSCG00000021791 | 13 | 1.58E+08 | 1.58E+08 | Calcium level QTL (6377) | 13 | 1.55E+08 | 1.84E+08 |
| ENSSSCG00000021791 | 13 | 1.58E+08 | 1.58E+08 | Triglyceride level QTL (15078) | 13 | 21348231 | 1.84E+08 |
| ENSSSCG00000021791 | 13 | 1.58E+08 | 1.58E+08 | Arachidic acid content QTL (156954) | 13 | 21348231 | 1.84E+08 |
| ENSSSCG00000021791 | 13 | 1.58E+08 | 1.58E+08 | Meat color a* QTL (38109) | 13 | 28918526 | 1.73E+08 |
| ENSSSCG00000021791 | 13 | 1.58E+08 | 1.58E+08 | Temperature 40 minutes post mortem (loin) QTL (4005) | 13 | 80472235 | 1.75E+08 |
| ENSSSCG00000021791 | 13 | 1.58E+08 | 1.58E+08 | Body weight (5 weeks) QTL (1136) | 13 | 1.13E+08 | 1.84E+08 |
| ENSSSCG00000021791 | 13 | 1.58E+08 | 1.58E+08 | Cholesterol level QTL (15077) | 13 | 1.17E+08 | 1.84E+08 |
| ENSSSCG00000021791 | 13 | 1.58E+08 | 1.58E+08 | Protein accretion rate QTL (2883) | 13 | 1.17E+08 | 1.75E+08 |
| ENSSSCG00000021791 | 13 | 1.58E+08 | 1.58E+08 | Average daily gain QTL (5950) | 13 | 1.2E+08 | 1.7E+08 |
| ENSSSCG00000021791 | 13 | 1.58E+08 | 1.58E+08 | Body weight (5 weeks) QTL (1139) | 13 | 1.54E+08 | 2.08E+08 |
| ENSSSCG00000030486 | 13 | 1.59E+08 | 1.59E+08 | Cortisol level QTL (21329) | 13 | 1.46E+08 | 1.91E+08 |
| ENSSSCG00000030486 | 13 | 1.59E+08 | 1.59E+08 | subjective abnormal odor QTL (4230) | 13 | 1.46E+08 | 1.84E+08 |
| ENSSSCG00000030486 | 13 | 1.59E+08 | 1.59E+08 | subjective pork odor QTL (4231) | 13 | 1.46E+08 | 1.84E+08 |
| ENSSSCG00000030486 | 13 | 1.59E+08 | 1.59E+08 | Percentage type IIa fibers QTL (7032) | 13 | 1.46E+08 | 1.78E+08 |
| ENSSSCG00000030486 | 13 | 1.59E+08 | 1.59E+08 | Calcium level QTL (6377) | 13 | 1.55E+08 | 1.84E+08 |
| ENSSSCG00000030486 | 13 | 1.59E+08 | 1.59E+08 | Triglyceride level QTL (15078) | 13 | 21348231 | 1.84E+08 |
| ENSSSCG00000030486 | 13 | 1.59E+08 | 1.59E+08 | Arachidic acid content QTL (156954) | 13 | 21348231 | 1.84E+08 |
| ENSSSCG00000030486 | 13 | 1.59E+08 | 1.59E+08 | Meat color a* QTL (38109) | 13 | 28918526 | 1.73E+08 |
| ENSSSCG00000030486 | 13 | 1.59E+08 | 1.59E+08 | Temperature 40 minutes post mortem (loin) QTL (4005) | 13 | 80472235 | 1.75E+08 |
| ENSSSCG00000030486 | 13 | 1.59E+08 | 1.59E+08 | Body weight (5 weeks) QTL (1136) | 13 | 1.13E+08 | 1.84E+08 |
| ENSSSCG00000030486 | 13 | 1.59E+08 | 1.59E+08 | Cholesterol level QTL (15077) | 13 | 1.17E+08 | 1.84E+08 |
| ENSSSCG00000030486 | 13 | 1.59E+08 | 1.59E+08 | Protein accretion rate QTL (2883) | 13 | 1.17E+08 | 1.75E+08 |
| ENSSSCG00000030486 | 13 | 1.59E+08 | 1.59E+08 | Average daily gain QTL (5950) | 13 | 1.2E+08 | 1.7E+08 |
| ENSSSCG00000030486 | 13 | 1.59E+08 | 1.59E+08 | Body weight (5 weeks) QTL (1139) | 13 | 1.54E+08 | 2.08E+08 |
| ENSSSCG00000021038 | 13 | 1.8E+08 | 1.8E+08 | Cortisol level QTL (21329) | 13 | 1.46E+08 | 1.91E+08 |
| ENSSSCG00000021038 | 13 | 1.8E+08 | 1.8E+08 | subjective abnormal odor QTL (4230) | 13 | 1.46E+08 | 1.84E+08 |
| ENSSSCG00000021038 | 13 | 1.8E+08 | 1.8E+08 | subjective pork odor QTL (4231) | 13 | 1.46E+08 | 1.84E+08 |
| ENSSSCG00000021038 | 13 | 1.8E+08 | 1.8E+08 | Calcium level QTL (6377) | 13 | 1.55E+08 | 1.84E+08 |
| ENSSSCG00000021038 | 13 | 1.8E+08 | 1.8E+08 | Triglyceride level QTL (15078) | 13 | 21348231 | 1.84E+08 |
| ENSSSCG00000021038 | 13 | 1.8E+08 | 1.8E+08 | Arachidic acid content QTL (156954) | 13 | 21348231 | 1.84E+08 |
| ENSSSCG00000021038 | 13 | 1.8E+08 | 1.8E+08 | Body weight (5 weeks) QTL (1136) | 13 | 1.13E+08 | 1.84E+08 |
| ENSSSCG00000021038 | 13 | 1.8E+08 | 1.8E+08 | Cholesterol level QTL (15077) | 13 | 1.17E+08 | 1.84E+08 |
| ENSSSCG00000021038 | 13 | 1.8E+08 | 1.8E+08 | Body weight (5 weeks) QTL (1139) | 13 | 1.54E+08 | 2.08E+08 |
| ENSSSCG00000021023 | 13 | 1.95E+08 | 1.95E+08 | Body weight (5 weeks) QTL (1139) | 13 | 1.54E+08 | 2.08E+08 |
| ENSSSCG00000012055 | 13 | 2E+08 | 2E+08 | Body weight (5 weeks) QTL (1139) | 13 | 1.54E+08 | 2.08E+08 |
| ENSSSCG00000012059 | 13 | 2E+08 | 2.01E+08 | Body weight (5 weeks) QTL (1139) | 13 | 1.54E+08 | 2.08E+08 |
| ENSSSCG00000021042 | 13 | 2.03E+08 | 2.03E+08 | Body weight (5 weeks) QTL (1139) | 13 | 1.54E+08 | 2.08E+08 |
| ENSSSCG00000039085 | 13 | 2.07E+08 | 2.07E+08 | Umami flavor intensity QTL (164982) | 13 | 2.07E+08 | 2.07E+08 |
| ENSSSCG00000039085 | 13 | 2.07E+08 | 2.07E+08 | Body weight (5 weeks) QTL (1139) | 13 | 1.54E+08 | 2.08E+08 |
| ENSSSCG00000009652 | 14 | 9455053 | 9517675 | Meat color OPTO QTL (18718) | 14 | 9331701 | 9509690 |
| ENSSSCG00000009652 | 14 | 9455053 | 9517675 | Stearic acid content QTL (15870) | 14 | 9407982 | 57464974 |
| ENSSSCG00000009652 | 14 | 9455053 | 9517675 | Linoleic acid content QTL (15871) | 14 | 9407982 | 57464974 |
| ENSSSCG00000009652 | 14 | 9455053 | 9517675 | Meat color-L QTL (3825) | 14 | 9407982 | 50091150 |
| ENSSSCG00000009652 | 14 | 9455053 | 9517675 | Meat color-a QTL (236) | 14 | 9407982 | 45387159 |
| ENSSSCG00000009652 | 14 | 9455053 | 9517675 | Average backfat thickness QTL (354) | 14 | 9407982 | 45387159 |
| ENSSSCG00000009652 | 14 | 9455053 | 9517675 | Backfat at last rib QTL (16841) | 14 | 9407982 | 40047493 |
| ENSSSCG00000009652 | 14 | 9455053 | 9517675 | androstenone, sensory panel QTL (4234) | 14 | 9407982 | 27334003 |
| ENSSSCG00000009652 | 14 | 9455053 | 9517675 | Marbling QTL (3079) | 14 | 9407982 | 27334003 |
| ENSSSCG00000009652 | 14 | 9455053 | 9517675 | Meat color L* QTL (38110) | 14 | 9407982 | 1.38E+08 |
| ENSSSCG00000009652 | 14 | 9455053 | 9517675 | Arachidonic acid content QTL (156959) | 14 | 9407982 | 1.38E+08 |
| ENSSSCG00000009652 | 14 | 9455053 | 9517675 | Haptoglobin concentration QTL (9653) | 14 | 9407982 | 76280940 |
| ENSSSCG00000009652 | 14 | 9455053 | 9517675 | backfat above muscle dorsi QTL (5991) | 14 | 9407982 | 76280940 |
| ENSSSCG00000009652 | 14 | 9455053 | 9517675 | Percentage type I fibers QTL (2822) | 14 | 9407982 | 76280940 |
| ENSSSCG00000009652 | 14 | 9455053 | 9517675 | Percentage type IIb fibers QTL (2823) | 14 | 9407982 | 76280940 |
| ENSSSCG00000009652 | 14 | 9455053 | 9517675 | Percentage type I fibers QTL (2824) | 14 | 9407982 | 76280940 |
| ENSSSCG00000009776 | 14 | 29570471 | 29620091 | Average daily gain QTL (22240) | 14 | 29608051 | 29608055 |
| ENSSSCG00000009776 | 14 | 29570471 | 29620091 | Stearic acid content QTL (15870) | 14 | 9407982 | 57464974 |
| ENSSSCG00000009776 | 14 | 29570471 | 29620091 | Linoleic acid content QTL (15871) | 14 | 9407982 | 57464974 |
| ENSSSCG00000009776 | 14 | 29570471 | 29620091 | Meat color-L QTL (3825) | 14 | 9407982 | 50091150 |
| ENSSSCG00000009776 | 14 | 29570471 | 29620091 | Meat color-a QTL (236) | 14 | 9407982 | 45387159 |
| ENSSSCG00000009776 | 14 | 29570471 | 29620091 | Average backfat thickness QTL (354) | 14 | 9407982 | 45387159 |
| ENSSSCG00000009776 | 14 | 29570471 | 29620091 | Backfat at last rib QTL (16841) | 14 | 9407982 | 40047493 |
| ENSSSCG00000009776 | 14 | 29570471 | 29620091 | Calcium level QTL (15079) | 14 | 24537035 | 64838799 |
| ENSSSCG00000009776 | 14 | 29570471 | 29620091 | Creatinine level QTL (6362) | 14 | 24537035 | 64838799 |
| ENSSSCG00000009776 | 14 | 29570471 | 29620091 | Creatinine level QTL (6364) | 14 | 24537035 | 64838799 |
| ENSSSCG00000009776 | 14 | 29570471 | 29620091 | Potassium level QTL (6384) | 14 | 24537035 | 64838799 |
| ENSSSCG00000009776 | 14 | 29570471 | 29620091 | Subcutanous fat area QTL (18007) | 14 | 24537035 | 40047493 |
| ENSSSCG00000009776 | 14 | 29570471 | 29620091 | Adipocyte diameter QTL (18032) | 14 | 24537035 | 40047493 |
| ENSSSCG00000009776 | 14 | 29570471 | 29620091 | skatole, sensory panel QTL (4237) | 14 | 27334003 | 45387159 |
| ENSSSCG00000009776 | 14 | 29570471 | 29620091 | subjective boar flavor in lean QTL (4238) | 14 | 27334003 | 45387159 |
| ENSSSCG00000009776 | 14 | 29570471 | 29620091 | subjective boar flavor in fat QTL (4241) | 14 | 27334003 | 45387159 |
| ENSSSCG00000009776 | 14 | 29570471 | 29620091 | subjective abnormal odor QTL (4242) | 14 | 27334003 | 45387159 |
| ENSSSCG00000009776 | 14 | 29570471 | 29620091 | Relative area of type IIb fibers QTL (4034) | 14 | 27334003 | 37123390 |
| ENSSSCG00000009776 | 14 | 29570471 | 29620091 | Percentage type IIb fibers QTL (4032) | 14 | 27334003 | 35216367 |
| ENSSSCG00000009776 | 14 | 29570471 | 29620091 | Diameter of type IIa muscle fibers QTL (7018) | 14 | 28223947 | 58863458 |
| ENSSSCG00000009776 | 14 | 29570471 | 29620091 | Percentage type IIb fibers QTL (7024) | 14 | 28223947 | 58863458 |
| ENSSSCG00000009776 | 14 | 29570471 | 29620091 | Diameter of type IIb muscle fibers QTL (7038) | 14 | 28223947 | 58863458 |
| ENSSSCG00000009776 | 14 | 29570471 | 29620091 | Loin muscle area QTL (3861) | 14 | 28223947 | 56702165 |
| ENSSSCG00000009776 | 14 | 29570471 | 29620091 | Meat color L* QTL (38110) | 14 | 9407982 | 1.38E+08 |
| ENSSSCG00000009776 | 14 | 29570471 | 29620091 | Arachidonic acid content QTL (156959) | 14 | 9407982 | 1.38E+08 |
| ENSSSCG00000009776 | 14 | 29570471 | 29620091 | Haptoglobin concentration QTL (9653) | 14 | 9407982 | 76280940 |
| ENSSSCG00000009776 | 14 | 29570471 | 29620091 | backfat above muscle dorsi QTL (5991) | 14 | 9407982 | 76280940 |
| ENSSSCG00000009776 | 14 | 29570471 | 29620091 | Percentage type I fibers QTL (2822) | 14 | 9407982 | 76280940 |
| ENSSSCG00000009776 | 14 | 29570471 | 29620091 | Percentage type IIb fibers QTL (2823) | 14 | 9407982 | 76280940 |
| ENSSSCG00000009776 | 14 | 29570471 | 29620091 | Percentage type I fibers QTL (2824) | 14 | 9407982 | 76280940 |
| ENSSSCG00000009776 | 14 | 29570471 | 29620091 | Shear force at first peak QTL (3929) | 14 | 10933601 | 1.38E+08 |
| ENSSSCG00000009776 | 14 | 29570471 | 29620091 | Shear force at first peak QTL (3930) | 14 | 10933601 | 1.38E+08 |
| ENSSSCG00000009776 | 14 | 29570471 | 29620091 | Shear force at first peak QTL (3931) | 14 | 10933601 | 1.38E+08 |
| ENSSSCG00000009776 | 14 | 29570471 | 29620091 | Shear force QTL (3925) | 14 | 10933601 | 1.12E+08 |
| ENSSSCG00000009776 | 14 | 29570471 | 29620091 | Loin muscle area QTL (7) | 14 | 15256188 | 97893873 |
| ENSSSCG00000009776 | 14 | 29570471 | 29620091 | Abdominal fat weight QTL (21266) | 14 | 16273267 | 83654764 |
| ENSSSCG00000009776 | 14 | 29570471 | 29620091 | Leaf fat weight QTL (21267) | 14 | 16273267 | 71322678 |
| ENSSSCG00000009776 | 14 | 29570471 | 29620091 | Capric acid content QTL (156957) | 14 | 24537035 | 1.38E+08 |
| ENSSSCG00000009776 | 14 | 29570471 | 29620091 | Margaric acid content QTL (156958) | 14 | 24537035 | 1.38E+08 |
| ENSSSCG00000009776 | 14 | 29570471 | 29620091 | Average instron (star probe) force QTL (3814) | 14 | 27334003 | 76280940 |
| ENSSSCG00000009776 | 14 | 29570471 | 29620091 | Fat androstenone level QTL (606) | 14 | 28351082 | 76280940 |
| ENSSSCG00000009776 | 14 | 29570471 | 29620091 | Fat androstenone level QTL (607) | 14 | 28351082 | 76280940 |
| ENSSSCG00000009776 | 14 | 29570471 | 29620091 | Fat androstenone level QTL (608) | 14 | 28351082 | 76280940 |
| ENSSSCG00000009776 | 14 | 29570471 | 29620091 | Fat androstenone level QTL (609) | 14 | 28351082 | 76280940 |
| ENSSSCG00000009814 | 14 | 31259727 | 31306442 | Stearic acid content QTL (15870) | 14 | 9407982 | 57464974 |
| ENSSSCG00000009814 | 14 | 31259727 | 31306442 | Linoleic acid content QTL (15871) | 14 | 9407982 | 57464974 |
| ENSSSCG00000009814 | 14 | 31259727 | 31306442 | Meat color-L QTL (3825) | 14 | 9407982 | 50091150 |
| ENSSSCG00000009814 | 14 | 31259727 | 31306442 | Meat color-a QTL (236) | 14 | 9407982 | 45387159 |
| ENSSSCG00000009814 | 14 | 31259727 | 31306442 | Average backfat thickness QTL (354) | 14 | 9407982 | 45387159 |
| ENSSSCG00000009814 | 14 | 31259727 | 31306442 | Backfat at last rib QTL (16841) | 14 | 9407982 | 40047493 |
| ENSSSCG00000009814 | 14 | 31259727 | 31306442 | Calcium level QTL (15079) | 14 | 24537035 | 64838799 |
| ENSSSCG00000009814 | 14 | 31259727 | 31306442 | Creatinine level QTL (6362) | 14 | 24537035 | 64838799 |
| ENSSSCG00000009814 | 14 | 31259727 | 31306442 | Creatinine level QTL (6364) | 14 | 24537035 | 64838799 |
| ENSSSCG00000009814 | 14 | 31259727 | 31306442 | Potassium level QTL (6384) | 14 | 24537035 | 64838799 |
| ENSSSCG00000009814 | 14 | 31259727 | 31306442 | Subcutanous fat area QTL (18007) | 14 | 24537035 | 40047493 |
| ENSSSCG00000009814 | 14 | 31259727 | 31306442 | Adipocyte diameter QTL (18032) | 14 | 24537035 | 40047493 |
| ENSSSCG00000009814 | 14 | 31259727 | 31306442 | skatole, sensory panel QTL (4237) | 14 | 27334003 | 45387159 |
| ENSSSCG00000009814 | 14 | 31259727 | 31306442 | subjective boar flavor in lean QTL (4238) | 14 | 27334003 | 45387159 |
| ENSSSCG00000009814 | 14 | 31259727 | 31306442 | subjective boar flavor in fat QTL (4241) | 14 | 27334003 | 45387159 |
| ENSSSCG00000009814 | 14 | 31259727 | 31306442 | subjective abnormal odor QTL (4242) | 14 | 27334003 | 45387159 |
| ENSSSCG00000009814 | 14 | 31259727 | 31306442 | Relative area of type IIb fibers QTL (4034) | 14 | 27334003 | 37123390 |
| ENSSSCG00000009814 | 14 | 31259727 | 31306442 | Percentage type IIb fibers QTL (4032) | 14 | 27334003 | 35216367 |
| ENSSSCG00000009814 | 14 | 31259727 | 31306442 | Diameter of type IIa muscle fibers QTL (7018) | 14 | 28223947 | 58863458 |
| ENSSSCG00000009814 | 14 | 31259727 | 31306442 | Percentage type IIb fibers QTL (7024) | 14 | 28223947 | 58863458 |
| ENSSSCG00000009814 | 14 | 31259727 | 31306442 | Diameter of type IIb muscle fibers QTL (7038) | 14 | 28223947 | 58863458 |
| ENSSSCG00000009814 | 14 | 31259727 | 31306442 | Loin muscle area QTL (3861) | 14 | 28223947 | 56702165 |
| ENSSSCG00000009814 | 14 | 31259727 | 31306442 | Percentage type IIa fibers QTL (4027) | 14 | 30258106 | 34707827 |
| ENSSSCG00000009814 | 14 | 31259727 | 31306442 | Meat color L* QTL (38110) | 14 | 9407982 | 1.38E+08 |
| ENSSSCG00000009814 | 14 | 31259727 | 31306442 | Arachidonic acid content QTL (156959) | 14 | 9407982 | 1.38E+08 |
| ENSSSCG00000009814 | 14 | 31259727 | 31306442 | Haptoglobin concentration QTL (9653) | 14 | 9407982 | 76280940 |
| ENSSSCG00000009814 | 14 | 31259727 | 31306442 | backfat above muscle dorsi QTL (5991) | 14 | 9407982 | 76280940 |
| ENSSSCG00000009814 | 14 | 31259727 | 31306442 | Percentage type I fibers QTL (2822) | 14 | 9407982 | 76280940 |
| ENSSSCG00000009814 | 14 | 31259727 | 31306442 | Percentage type IIb fibers QTL (2823) | 14 | 9407982 | 76280940 |
| ENSSSCG00000009814 | 14 | 31259727 | 31306442 | Percentage type I fibers QTL (2824) | 14 | 9407982 | 76280940 |
| ENSSSCG00000009814 | 14 | 31259727 | 31306442 | Shear force at first peak QTL (3929) | 14 | 10933601 | 1.38E+08 |
| ENSSSCG00000009814 | 14 | 31259727 | 31306442 | Shear force at first peak QTL (3930) | 14 | 10933601 | 1.38E+08 |
| ENSSSCG00000009814 | 14 | 31259727 | 31306442 | Shear force at first peak QTL (3931) | 14 | 10933601 | 1.38E+08 |
| ENSSSCG00000009814 | 14 | 31259727 | 31306442 | Shear force QTL (3925) | 14 | 10933601 | 1.12E+08 |
| ENSSSCG00000009814 | 14 | 31259727 | 31306442 | Loin muscle area QTL (7) | 14 | 15256188 | 97893873 |
| ENSSSCG00000009814 | 14 | 31259727 | 31306442 | Abdominal fat weight QTL (21266) | 14 | 16273267 | 83654764 |
| ENSSSCG00000009814 | 14 | 31259727 | 31306442 | Leaf fat weight QTL (21267) | 14 | 16273267 | 71322678 |
| ENSSSCG00000009814 | 14 | 31259727 | 31306442 | Capric acid content QTL (156957) | 14 | 24537035 | 1.38E+08 |
| ENSSSCG00000009814 | 14 | 31259727 | 31306442 | Margaric acid content QTL (156958) | 14 | 24537035 | 1.38E+08 |
| ENSSSCG00000009814 | 14 | 31259727 | 31306442 | Average instron (star probe) force QTL (3814) | 14 | 27334003 | 76280940 |
| ENSSSCG00000009814 | 14 | 31259727 | 31306442 | Fat androstenone level QTL (606) | 14 | 28351082 | 76280940 |
| ENSSSCG00000009814 | 14 | 31259727 | 31306442 | Fat androstenone level QTL (607) | 14 | 28351082 | 76280940 |
| ENSSSCG00000009814 | 14 | 31259727 | 31306442 | Fat androstenone level QTL (608) | 14 | 28351082 | 76280940 |
| ENSSSCG00000009814 | 14 | 31259727 | 31306442 | Fat androstenone level QTL (609) | 14 | 28351082 | 76280940 |
| ENSSSCG00000034386 | 14 | 31674757 | 31744973 | Stearic acid content QTL (15870) | 14 | 9407982 | 57464974 |
| ENSSSCG00000034386 | 14 | 31674757 | 31744973 | Linoleic acid content QTL (15871) | 14 | 9407982 | 57464974 |
| ENSSSCG00000034386 | 14 | 31674757 | 31744973 | Meat color-L QTL (3825) | 14 | 9407982 | 50091150 |
| ENSSSCG00000034386 | 14 | 31674757 | 31744973 | Meat color-a QTL (236) | 14 | 9407982 | 45387159 |
| ENSSSCG00000034386 | 14 | 31674757 | 31744973 | Average backfat thickness QTL (354) | 14 | 9407982 | 45387159 |
| ENSSSCG00000034386 | 14 | 31674757 | 31744973 | Backfat at last rib QTL (16841) | 14 | 9407982 | 40047493 |
| ENSSSCG00000034386 | 14 | 31674757 | 31744973 | Calcium level QTL (15079) | 14 | 24537035 | 64838799 |
| ENSSSCG00000034386 | 14 | 31674757 | 31744973 | Creatinine level QTL (6362) | 14 | 24537035 | 64838799 |
| ENSSSCG00000034386 | 14 | 31674757 | 31744973 | Creatinine level QTL (6364) | 14 | 24537035 | 64838799 |
| ENSSSCG00000034386 | 14 | 31674757 | 31744973 | Potassium level QTL (6384) | 14 | 24537035 | 64838799 |
| ENSSSCG00000034386 | 14 | 31674757 | 31744973 | Subcutanous fat area QTL (18007) | 14 | 24537035 | 40047493 |
| ENSSSCG00000034386 | 14 | 31674757 | 31744973 | Adipocyte diameter QTL (18032) | 14 | 24537035 | 40047493 |
| ENSSSCG00000034386 | 14 | 31674757 | 31744973 | skatole, sensory panel QTL (4237) | 14 | 27334003 | 45387159 |
| ENSSSCG00000034386 | 14 | 31674757 | 31744973 | subjective boar flavor in lean QTL (4238) | 14 | 27334003 | 45387159 |
| ENSSSCG00000034386 | 14 | 31674757 | 31744973 | subjective boar flavor in fat QTL (4241) | 14 | 27334003 | 45387159 |
| ENSSSCG00000034386 | 14 | 31674757 | 31744973 | subjective abnormal odor QTL (4242) | 14 | 27334003 | 45387159 |
| ENSSSCG00000034386 | 14 | 31674757 | 31744973 | Relative area of type IIb fibers QTL (4034) | 14 | 27334003 | 37123390 |
| ENSSSCG00000034386 | 14 | 31674757 | 31744973 | Percentage type IIb fibers QTL (4032) | 14 | 27334003 | 35216367 |
| ENSSSCG00000034386 | 14 | 31674757 | 31744973 | Diameter of type IIa muscle fibers QTL (7018) | 14 | 28223947 | 58863458 |
| ENSSSCG00000034386 | 14 | 31674757 | 31744973 | Percentage type IIb fibers QTL (7024) | 14 | 28223947 | 58863458 |
| ENSSSCG00000034386 | 14 | 31674757 | 31744973 | Diameter of type IIb muscle fibers QTL (7038) | 14 | 28223947 | 58863458 |
| ENSSSCG00000034386 | 14 | 31674757 | 31744973 | Loin muscle area QTL (3861) | 14 | 28223947 | 56702165 |
| ENSSSCG00000034386 | 14 | 31674757 | 31744973 | Percentage type IIa fibers QTL (4027) | 14 | 30258106 | 34707827 |
| ENSSSCG00000034386 | 14 | 31674757 | 31744973 | Meat color L* QTL (38110) | 14 | 9407982 | 1.38E+08 |
| ENSSSCG00000034386 | 14 | 31674757 | 31744973 | Arachidonic acid content QTL (156959) | 14 | 9407982 | 1.38E+08 |
| ENSSSCG00000034386 | 14 | 31674757 | 31744973 | Haptoglobin concentration QTL (9653) | 14 | 9407982 | 76280940 |
| ENSSSCG00000034386 | 14 | 31674757 | 31744973 | backfat above muscle dorsi QTL (5991) | 14 | 9407982 | 76280940 |
| ENSSSCG00000034386 | 14 | 31674757 | 31744973 | Percentage type I fibers QTL (2822) | 14 | 9407982 | 76280940 |
| ENSSSCG00000034386 | 14 | 31674757 | 31744973 | Percentage type IIb fibers QTL (2823) | 14 | 9407982 | 76280940 |
| ENSSSCG00000034386 | 14 | 31674757 | 31744973 | Percentage type I fibers QTL (2824) | 14 | 9407982 | 76280940 |
| ENSSSCG00000034386 | 14 | 31674757 | 31744973 | Shear force at first peak QTL (3929) | 14 | 10933601 | 1.38E+08 |
| ENSSSCG00000034386 | 14 | 31674757 | 31744973 | Shear force at first peak QTL (3930) | 14 | 10933601 | 1.38E+08 |
| ENSSSCG00000034386 | 14 | 31674757 | 31744973 | Shear force at first peak QTL (3931) | 14 | 10933601 | 1.38E+08 |
| ENSSSCG00000034386 | 14 | 31674757 | 31744973 | Shear force QTL (3925) | 14 | 10933601 | 1.12E+08 |
| ENSSSCG00000034386 | 14 | 31674757 | 31744973 | Loin muscle area QTL (7) | 14 | 15256188 | 97893873 |
| ENSSSCG00000034386 | 14 | 31674757 | 31744973 | Abdominal fat weight QTL (21266) | 14 | 16273267 | 83654764 |
| ENSSSCG00000034386 | 14 | 31674757 | 31744973 | Leaf fat weight QTL (21267) | 14 | 16273267 | 71322678 |
| ENSSSCG00000034386 | 14 | 31674757 | 31744973 | Capric acid content QTL (156957) | 14 | 24537035 | 1.38E+08 |
| ENSSSCG00000034386 | 14 | 31674757 | 31744973 | Margaric acid content QTL (156958) | 14 | 24537035 | 1.38E+08 |
| ENSSSCG00000034386 | 14 | 31674757 | 31744973 | Average instron (star probe) force QTL (3814) | 14 | 27334003 | 76280940 |
| ENSSSCG00000034386 | 14 | 31674757 | 31744973 | Fat androstenone level QTL (606) | 14 | 28351082 | 76280940 |
| ENSSSCG00000034386 | 14 | 31674757 | 31744973 | Fat androstenone level QTL (607) | 14 | 28351082 | 76280940 |
| ENSSSCG00000034386 | 14 | 31674757 | 31744973 | Fat androstenone level QTL (608) | 14 | 28351082 | 76280940 |
| ENSSSCG00000034386 | 14 | 31674757 | 31744973 | Fat androstenone level QTL (609) | 14 | 28351082 | 76280940 |
| ENSSSCG00000009968 | 14 | 45320588 | 45925027 | Stearic acid content QTL (15870) | 14 | 9407982 | 57464974 |
| ENSSSCG00000009968 | 14 | 45320588 | 45925027 | Linoleic acid content QTL (15871) | 14 | 9407982 | 57464974 |
| ENSSSCG00000009968 | 14 | 45320588 | 45925027 | Meat color-L QTL (3825) | 14 | 9407982 | 50091150 |
| ENSSSCG00000009968 | 14 | 45320588 | 45925027 | Calcium level QTL (15079) | 14 | 24537035 | 64838799 |
| ENSSSCG00000009968 | 14 | 45320588 | 45925027 | Creatinine level QTL (6362) | 14 | 24537035 | 64838799 |
| ENSSSCG00000009968 | 14 | 45320588 | 45925027 | Creatinine level QTL (6364) | 14 | 24537035 | 64838799 |
| ENSSSCG00000009968 | 14 | 45320588 | 45925027 | Potassium level QTL (6384) | 14 | 24537035 | 64838799 |
| ENSSSCG00000009968 | 14 | 45320588 | 45925027 | Diameter of type IIa muscle fibers QTL (7018) | 14 | 28223947 | 58863458 |
| ENSSSCG00000009968 | 14 | 45320588 | 45925027 | Percentage type IIb fibers QTL (7024) | 14 | 28223947 | 58863458 |
| ENSSSCG00000009968 | 14 | 45320588 | 45925027 | Diameter of type IIb muscle fibers QTL (7038) | 14 | 28223947 | 58863458 |
| ENSSSCG00000009968 | 14 | 45320588 | 45925027 | Loin muscle area QTL (3861) | 14 | 28223947 | 56702165 |
| ENSSSCG00000009968 | 14 | 45320588 | 45925027 | Backfat at last lumbar QTL (33) | 14 | 36869121 | 57464974 |
| ENSSSCG00000009968 | 14 | 45320588 | 45925027 | Average backfat thickness QTL (37) | 14 | 36869121 | 52760983 |
| ENSSSCG00000009968 | 14 | 45320588 | 45925027 | Interferon-gamma to interleukin-10 ratio QTL (12272) | 14 | 40047493 | 52760983 |
| ENSSSCG00000009968 | 14 | 45320588 | 45925027 | Loin muscle area QTL (1148) | 14 | 40047493 | 51235364 |
| ENSSSCG00000009968 | 14 | 45320588 | 45925027 | Meat color-L QTL (234) | 14 | 45387159 | 58863458 |
| ENSSSCG00000009968 | 14 | 45320588 | 45925027 | subjective boar flavor in lean QTL (4239) | 14 | 45387159 | 58863458 |
| ENSSSCG00000009968 | 14 | 45320588 | 45925027 | Linoleic acid content QTL (30907) | 14 | 45387159 | 51743904 |
| ENSSSCG00000009968 | 14 | 45320588 | 45925027 | Meat color L* QTL (38110) | 14 | 9407982 | 1.38E+08 |
| ENSSSCG00000009968 | 14 | 45320588 | 45925027 | Arachidonic acid content QTL (156959) | 14 | 9407982 | 1.38E+08 |
| ENSSSCG00000009968 | 14 | 45320588 | 45925027 | Haptoglobin concentration QTL (9653) | 14 | 9407982 | 76280940 |
| ENSSSCG00000009968 | 14 | 45320588 | 45925027 | backfat above muscle dorsi QTL (5991) | 14 | 9407982 | 76280940 |
| ENSSSCG00000009968 | 14 | 45320588 | 45925027 | Percentage type I fibers QTL (2822) | 14 | 9407982 | 76280940 |
| ENSSSCG00000009968 | 14 | 45320588 | 45925027 | Percentage type IIb fibers QTL (2823) | 14 | 9407982 | 76280940 |
| ENSSSCG00000009968 | 14 | 45320588 | 45925027 | Percentage type I fibers QTL (2824) | 14 | 9407982 | 76280940 |
| ENSSSCG00000009968 | 14 | 45320588 | 45925027 | Shear force at first peak QTL (3929) | 14 | 10933601 | 1.38E+08 |
| ENSSSCG00000009968 | 14 | 45320588 | 45925027 | Shear force at first peak QTL (3930) | 14 | 10933601 | 1.38E+08 |
| ENSSSCG00000009968 | 14 | 45320588 | 45925027 | Shear force at first peak QTL (3931) | 14 | 10933601 | 1.38E+08 |
| ENSSSCG00000009968 | 14 | 45320588 | 45925027 | Shear force QTL (3925) | 14 | 10933601 | 1.12E+08 |
| ENSSSCG00000009968 | 14 | 45320588 | 45925027 | Loin muscle area QTL (7) | 14 | 15256188 | 97893873 |
| ENSSSCG00000009968 | 14 | 45320588 | 45925027 | Abdominal fat weight QTL (21266) | 14 | 16273267 | 83654764 |
| ENSSSCG00000009968 | 14 | 45320588 | 45925027 | Leaf fat weight QTL (21267) | 14 | 16273267 | 71322678 |
| ENSSSCG00000009968 | 14 | 45320588 | 45925027 | Capric acid content QTL (156957) | 14 | 24537035 | 1.38E+08 |
| ENSSSCG00000009968 | 14 | 45320588 | 45925027 | Margaric acid content QTL (156958) | 14 | 24537035 | 1.38E+08 |
| ENSSSCG00000009968 | 14 | 45320588 | 45925027 | Average instron (star probe) force QTL (3814) | 14 | 27334003 | 76280940 |
| ENSSSCG00000009968 | 14 | 45320588 | 45925027 | Fat androstenone level QTL (606) | 14 | 28351082 | 76280940 |
| ENSSSCG00000009968 | 14 | 45320588 | 45925027 | Fat androstenone level QTL (607) | 14 | 28351082 | 76280940 |
| ENSSSCG00000009968 | 14 | 45320588 | 45925027 | Fat androstenone level QTL (608) | 14 | 28351082 | 76280940 |
| ENSSSCG00000009968 | 14 | 45320588 | 45925027 | Fat androstenone level QTL (609) | 14 | 28351082 | 76280940 |
| ENSSSCG00000009968 | 14 | 45320588 | 45925027 | Carcass weight (hot) QTL (16873) | 14 | 40047493 | 85180383 |
| ENSSSCG00000009968 | 14 | 45320588 | 45925027 | Gadoleic acid content QTL (156905) | 14 | 42081651 | 80476391 |
| ENSSSCG00000009968 | 14 | 45320588 | 45925027 | Body weight QTL (8810) | 14 | 42081651 | 76280940 |
| ENSSSCG00000010164 | 14 | 57424904 | 57481988 | Half carcass weight QTL (13263) | 14 | 56422468 | 57528542 |
| ENSSSCG00000010164 | 14 | 57424904 | 57481988 | Carcass length QTL (13371) | 14 | 56422468 | 57528542 |
| ENSSSCG00000010164 | 14 | 57424904 | 57481988 | Stearic acid content QTL (15870) | 14 | 9407982 | 57464974 |
| ENSSSCG00000010164 | 14 | 57424904 | 57481988 | Linoleic acid content QTL (15871) | 14 | 9407982 | 57464974 |
| ENSSSCG00000010164 | 14 | 57424904 | 57481988 | Calcium level QTL (15079) | 14 | 24537035 | 64838799 |
| ENSSSCG00000010164 | 14 | 57424904 | 57481988 | Creatinine level QTL (6362) | 14 | 24537035 | 64838799 |
| ENSSSCG00000010164 | 14 | 57424904 | 57481988 | Creatinine level QTL (6364) | 14 | 24537035 | 64838799 |
| ENSSSCG00000010164 | 14 | 57424904 | 57481988 | Potassium level QTL (6384) | 14 | 24537035 | 64838799 |
| ENSSSCG00000010164 | 14 | 57424904 | 57481988 | Diameter of type IIa muscle fibers QTL (7018) | 14 | 28223947 | 58863458 |
| ENSSSCG00000010164 | 14 | 57424904 | 57481988 | Percentage type IIb fibers QTL (7024) | 14 | 28223947 | 58863458 |
| ENSSSCG00000010164 | 14 | 57424904 | 57481988 | Diameter of type IIb muscle fibers QTL (7038) | 14 | 28223947 | 58863458 |
| ENSSSCG00000010164 | 14 | 57424904 | 57481988 | Backfat at last lumbar QTL (33) | 14 | 36869121 | 57464974 |
| ENSSSCG00000010164 | 14 | 57424904 | 57481988 | Meat color-L QTL (234) | 14 | 45387159 | 58863458 |
| ENSSSCG00000010164 | 14 | 57424904 | 57481988 | subjective boar flavor in lean QTL (4239) | 14 | 45387159 | 58863458 |
| ENSSSCG00000010164 | 14 | 57424904 | 57481988 | Salmonella count in liver and spleen QTL (11579) | 14 | 51743904 | 58863458 |
| ENSSSCG00000010164 | 14 | 57424904 | 57481988 | Meat color L* QTL (38110) | 14 | 9407982 | 1.38E+08 |
| ENSSSCG00000010164 | 14 | 57424904 | 57481988 | Arachidonic acid content QTL (156959) | 14 | 9407982 | 1.38E+08 |
| ENSSSCG00000010164 | 14 | 57424904 | 57481988 | Haptoglobin concentration QTL (9653) | 14 | 9407982 | 76280940 |
| ENSSSCG00000010164 | 14 | 57424904 | 57481988 | backfat above muscle dorsi QTL (5991) | 14 | 9407982 | 76280940 |
| ENSSSCG00000010164 | 14 | 57424904 | 57481988 | Percentage type I fibers QTL (2822) | 14 | 9407982 | 76280940 |
| ENSSSCG00000010164 | 14 | 57424904 | 57481988 | Percentage type IIb fibers QTL (2823) | 14 | 9407982 | 76280940 |
| ENSSSCG00000010164 | 14 | 57424904 | 57481988 | Percentage type I fibers QTL (2824) | 14 | 9407982 | 76280940 |
| ENSSSCG00000010164 | 14 | 57424904 | 57481988 | Shear force at first peak QTL (3929) | 14 | 10933601 | 1.38E+08 |
| ENSSSCG00000010164 | 14 | 57424904 | 57481988 | Shear force at first peak QTL (3930) | 14 | 10933601 | 1.38E+08 |
| ENSSSCG00000010164 | 14 | 57424904 | 57481988 | Shear force at first peak QTL (3931) | 14 | 10933601 | 1.38E+08 |
| ENSSSCG00000010164 | 14 | 57424904 | 57481988 | Shear force QTL (3925) | 14 | 10933601 | 1.12E+08 |
| ENSSSCG00000010164 | 14 | 57424904 | 57481988 | Loin muscle area QTL (7) | 14 | 15256188 | 97893873 |
| ENSSSCG00000010164 | 14 | 57424904 | 57481988 | Abdominal fat weight QTL (21266) | 14 | 16273267 | 83654764 |
| ENSSSCG00000010164 | 14 | 57424904 | 57481988 | Leaf fat weight QTL (21267) | 14 | 16273267 | 71322678 |
| ENSSSCG00000010164 | 14 | 57424904 | 57481988 | Capric acid content QTL (156957) | 14 | 24537035 | 1.38E+08 |
| ENSSSCG00000010164 | 14 | 57424904 | 57481988 | Margaric acid content QTL (156958) | 14 | 24537035 | 1.38E+08 |
| ENSSSCG00000010164 | 14 | 57424904 | 57481988 | Average instron (star probe) force QTL (3814) | 14 | 27334003 | 76280940 |
| ENSSSCG00000010164 | 14 | 57424904 | 57481988 | Fat androstenone level QTL (606) | 14 | 28351082 | 76280940 |
| ENSSSCG00000010164 | 14 | 57424904 | 57481988 | Fat androstenone level QTL (607) | 14 | 28351082 | 76280940 |
| ENSSSCG00000010164 | 14 | 57424904 | 57481988 | Fat androstenone level QTL (608) | 14 | 28351082 | 76280940 |
| ENSSSCG00000010164 | 14 | 57424904 | 57481988 | Fat androstenone level QTL (609) | 14 | 28351082 | 76280940 |
| ENSSSCG00000010164 | 14 | 57424904 | 57481988 | Carcass weight (hot) QTL (16873) | 14 | 40047493 | 85180383 |
| ENSSSCG00000010164 | 14 | 57424904 | 57481988 | Gadoleic acid content QTL (156905) | 14 | 42081651 | 80476391 |
| ENSSSCG00000010164 | 14 | 57424904 | 57481988 | Body weight QTL (8810) | 14 | 42081651 | 76280940 |
| ENSSSCG00000038913 | 14 | 71122787 | 71269415 | Percentage type IIb fibers QTL (4045) | 14 | 67762901 | 72339758 |
| ENSSSCG00000038913 | 14 | 71122787 | 71269415 | Meat color L* QTL (38110) | 14 | 9407982 | 1.38E+08 |
| ENSSSCG00000038913 | 14 | 71122787 | 71269415 | Arachidonic acid content QTL (156959) | 14 | 9407982 | 1.38E+08 |
| ENSSSCG00000038913 | 14 | 71122787 | 71269415 | Haptoglobin concentration QTL (9653) | 14 | 9407982 | 76280940 |
| ENSSSCG00000038913 | 14 | 71122787 | 71269415 | backfat above muscle dorsi QTL (5991) | 14 | 9407982 | 76280940 |
| ENSSSCG00000038913 | 14 | 71122787 | 71269415 | Percentage type I fibers QTL (2822) | 14 | 9407982 | 76280940 |
| ENSSSCG00000038913 | 14 | 71122787 | 71269415 | Percentage type IIb fibers QTL (2823) | 14 | 9407982 | 76280940 |
| ENSSSCG00000038913 | 14 | 71122787 | 71269415 | Percentage type I fibers QTL (2824) | 14 | 9407982 | 76280940 |
| ENSSSCG00000038913 | 14 | 71122787 | 71269415 | Shear force at first peak QTL (3929) | 14 | 10933601 | 1.38E+08 |
| ENSSSCG00000038913 | 14 | 71122787 | 71269415 | Shear force at first peak QTL (3930) | 14 | 10933601 | 1.38E+08 |
| ENSSSCG00000038913 | 14 | 71122787 | 71269415 | Shear force at first peak QTL (3931) | 14 | 10933601 | 1.38E+08 |
| ENSSSCG00000038913 | 14 | 71122787 | 71269415 | Shear force QTL (3925) | 14 | 10933601 | 1.12E+08 |
| ENSSSCG00000038913 | 14 | 71122787 | 71269415 | Loin muscle area QTL (7) | 14 | 15256188 | 97893873 |
| ENSSSCG00000038913 | 14 | 71122787 | 71269415 | Abdominal fat weight QTL (21266) | 14 | 16273267 | 83654764 |
| ENSSSCG00000038913 | 14 | 71122787 | 71269415 | Leaf fat weight QTL (21267) | 14 | 16273267 | 71322678 |
| ENSSSCG00000038913 | 14 | 71122787 | 71269415 | Capric acid content QTL (156957) | 14 | 24537035 | 1.38E+08 |
| ENSSSCG00000038913 | 14 | 71122787 | 71269415 | Margaric acid content QTL (156958) | 14 | 24537035 | 1.38E+08 |
| ENSSSCG00000038913 | 14 | 71122787 | 71269415 | Average instron (star probe) force QTL (3814) | 14 | 27334003 | 76280940 |
| ENSSSCG00000038913 | 14 | 71122787 | 71269415 | Fat androstenone level QTL (606) | 14 | 28351082 | 76280940 |
| ENSSSCG00000038913 | 14 | 71122787 | 71269415 | Fat androstenone level QTL (607) | 14 | 28351082 | 76280940 |
| ENSSSCG00000038913 | 14 | 71122787 | 71269415 | Fat androstenone level QTL (608) | 14 | 28351082 | 76280940 |
| ENSSSCG00000038913 | 14 | 71122787 | 71269415 | Fat androstenone level QTL (609) | 14 | 28351082 | 76280940 |
| ENSSSCG00000038913 | 14 | 71122787 | 71269415 | Carcass weight (hot) QTL (16873) | 14 | 40047493 | 85180383 |
| ENSSSCG00000038913 | 14 | 71122787 | 71269415 | Gadoleic acid content QTL (156905) | 14 | 42081651 | 80476391 |
| ENSSSCG00000038913 | 14 | 71122787 | 71269415 | Body weight QTL (8810) | 14 | 42081651 | 76280940 |
| ENSSSCG00000038913 | 14 | 71122787 | 71269415 | pH 24 hr post-mortem (loin) QTL (21861) | 14 | 58863458 | 1.38E+08 |
| ENSSSCG00000038913 | 14 | 71122787 | 71269415 | Fat protein content QTL (18013) | 14 | 58863458 | 1E+08 |
| ENSSSCG00000038913 | 14 | 71122787 | 71269415 | Salmonella count in liver and spleen QTL (11581) | 14 | 58863458 | 99546626 |
| ENSSSCG00000038913 | 14 | 71122787 | 71269415 | Adipocyte diameter QTL (12850) | 14 | 58863458 | 85180383 |
| ENSSSCG00000038913 | 14 | 71122787 | 71269415 | Adipocyte diameter QTL (12851) | 14 | 58863458 | 85180383 |
| ENSSSCG00000038913 | 14 | 71122787 | 71269415 | Adipocyte diameter QTL (12852) | 14 | 58863458 | 85180383 |
| ENSSSCG00000038913 | 14 | 71122787 | 71269415 | Backfat at last rib QTL (1169) | 14 | 58863458 | 85180383 |
| ENSSSCG00000038913 | 14 | 71122787 | 71269415 | Carcass length QTL (1261) | 14 | 58863458 | 85180383 |
| ENSSSCG00000038913 | 14 | 71122787 | 71269415 | Carcass length QTL (1153) | 14 | 58863458 | 85180383 |
| ENSSSCG00000038913 | 14 | 71122787 | 71269415 | Loin muscle area QTL (1154) | 14 | 58863458 | 85180383 |
| ENSSSCG00000038913 | 14 | 71122787 | 71269415 | Carcass weight (cold) QTL (1156) | 14 | 58863458 | 85180383 |
| ENSSSCG00000038913 | 14 | 71122787 | 71269415 | Body weight (slaughter) QTL (1159) | 14 | 58863458 | 85180383 |
| ENSSSCG00000038913 | 14 | 71122787 | 71269415 | Shoulder subcutaneous fat thickness QTL (1160) | 14 | 58863458 | 85180383 |
| ENSSSCG00000038913 | 14 | 71122787 | 71269415 | Average backfat thickness QTL (1161) | 14 | 58863458 | 85180383 |
| ENSSSCG00000038913 | 14 | 71122787 | 71269415 | Backfat at last rib QTL (1163) | 14 | 58863458 | 85180383 |
| ENSSSCG00000038913 | 14 | 71122787 | 71269415 | Meat color-a QTL (3289) | 14 | 58863458 | 80476391 |
| ENSSSCG00000038913 | 14 | 71122787 | 71269415 | Meat color a* QTL (21405) | 14 | 58863458 | 80476391 |
| ENSSSCG00000038913 | 14 | 71122787 | 71269415 | Shoulder weight, Boston QTL (21441) | 14 | 58863458 | 80476391 |
| ENSSSCG00000038913 | 14 | 71122787 | 71269415 | Average daily gain QTL (337) | 14 | 58863458 | 76280940 |
| ENSSSCG00000038913 | 14 | 71122787 | 71269415 | Meat color b* QTL (4007) | 14 | 58863458 | 76280940 |
| ENSSSCG00000038913 | 14 | 71122787 | 71269415 | Total shear work QTL (4008) | 14 | 58863458 | 76280940 |
| ENSSSCG00000038913 | 14 | 71122787 | 71269415 | Saturated fatty acid content QTL (156906) | 14 | 64838799 | 1.38E+08 |
| ENSSSCG00000038913 | 14 | 71122787 | 71269415 | Unsaturated fatty acid content QTL (156907) | 14 | 64838799 | 1.38E+08 |
| ENSSSCG00000038913 | 14 | 71122787 | 71269415 | Palmitoleic acid content QTL (156903) | 14 | 64838799 | 99546626 |
| ENSSSCG00000038913 | 14 | 71122787 | 71269415 | Stearic acid content QTL (156904) | 14 | 64838799 | 99546626 |
| ENSSSCG00000038913 | 14 | 71122787 | 71269415 | Aspartate aminotransferase activity QTL (10223) | 14 | 64838799 | 96876793 |
| ENSSSCG00000038913 | 14 | 71122787 | 71269415 | Aspartate aminotransferase activity QTL (10224) | 14 | 64838799 | 96876793 |
| ENSSSCG00000038913 | 14 | 71122787 | 71269415 | Aspartate aminotransferase activity QTL (10225) | 14 | 64838799 | 96876793 |
| ENSSSCG00000038913 | 14 | 71122787 | 71269415 | Aspartate aminotransferase activity QTL (10226) | 14 | 64838799 | 96876793 |
| ENSSSCG00000038913 | 14 | 71122787 | 71269415 | Aspartate aminotransferase activity QTL (10227) | 14 | 64838799 | 96876793 |
| ENSSSCG00000038913 | 14 | 71122787 | 71269415 | Aspartate aminotransferase activity QTL (10228) | 14 | 64838799 | 96876793 |
| ENSSSCG00000038913 | 14 | 71122787 | 71269415 | Aspartate aminotransferase activity QTL (10229) | 14 | 64838799 | 96876793 |
| ENSSSCG00000038913 | 14 | 71122787 | 71269415 | Aspartate aminotransferase activity QTL (10230) | 14 | 64838799 | 96876793 |
| ENSSSCG00000038913 | 14 | 71122787 | 71269415 | Aspartate aminotransferase activity QTL (10231) | 14 | 64838799 | 96876793 |
| ENSSSCG00000038913 | 14 | 71122787 | 71269415 | Aspartate aminotransferase activity QTL (10232) | 14 | 64838799 | 96876793 |
| ENSSSCG00000038913 | 14 | 71122787 | 71269415 | Aspartate aminotransferase activity QTL (10233) | 14 | 64838799 | 96876793 |
| ENSSSCG00000038913 | 14 | 71122787 | 71269415 | Aspartate aminotransferase activity QTL (10234) | 14 | 64838799 | 96876793 |
| ENSSSCG00000038913 | 14 | 71122787 | 71269415 | Aspartate aminotransferase activity QTL (10235) | 14 | 64838799 | 96876793 |
| ENSSSCG00000038913 | 14 | 71122787 | 71269415 | Aspartate aminotransferase activity QTL (7516) | 14 | 64838799 | 96876793 |
| ENSSSCG00000038913 | 14 | 71122787 | 71269415 | Granular leukocyte to lymphocyte ratio QTL (21335) | 14 | 64838799 | 88231620 |
| ENSSSCG00000038913 | 14 | 71122787 | 71269415 | Percentage type I fibers QTL (4022) | 14 | 66491552 | 83400494 |
| ENSSSCG00000029311 | 14 | 71298907 | 71394600 | Percentage type IIb fibers QTL (4045) | 14 | 67762901 | 72339758 |
| ENSSSCG00000029311 | 14 | 71298907 | 71394600 | Meat color L* QTL (38110) | 14 | 9407982 | 1.38E+08 |
| ENSSSCG00000029311 | 14 | 71298907 | 71394600 | Arachidonic acid content QTL (156959) | 14 | 9407982 | 1.38E+08 |
| ENSSSCG00000029311 | 14 | 71298907 | 71394600 | Haptoglobin concentration QTL (9653) | 14 | 9407982 | 76280940 |
| ENSSSCG00000029311 | 14 | 71298907 | 71394600 | backfat above muscle dorsi QTL (5991) | 14 | 9407982 | 76280940 |
| ENSSSCG00000029311 | 14 | 71298907 | 71394600 | Percentage type I fibers QTL (2822) | 14 | 9407982 | 76280940 |
| ENSSSCG00000029311 | 14 | 71298907 | 71394600 | Percentage type IIb fibers QTL (2823) | 14 | 9407982 | 76280940 |
| ENSSSCG00000029311 | 14 | 71298907 | 71394600 | Percentage type I fibers QTL (2824) | 14 | 9407982 | 76280940 |
| ENSSSCG00000029311 | 14 | 71298907 | 71394600 | Shear force at first peak QTL (3929) | 14 | 10933601 | 1.38E+08 |
| ENSSSCG00000029311 | 14 | 71298907 | 71394600 | Shear force at first peak QTL (3930) | 14 | 10933601 | 1.38E+08 |
| ENSSSCG00000029311 | 14 | 71298907 | 71394600 | Shear force at first peak QTL (3931) | 14 | 10933601 | 1.38E+08 |
| ENSSSCG00000029311 | 14 | 71298907 | 71394600 | Shear force QTL (3925) | 14 | 10933601 | 1.12E+08 |
| ENSSSCG00000029311 | 14 | 71298907 | 71394600 | Loin muscle area QTL (7) | 14 | 15256188 | 97893873 |
| ENSSSCG00000029311 | 14 | 71298907 | 71394600 | Abdominal fat weight QTL (21266) | 14 | 16273267 | 83654764 |
| ENSSSCG00000029311 | 14 | 71298907 | 71394600 | Capric acid content QTL (156957) | 14 | 24537035 | 1.38E+08 |
| ENSSSCG00000029311 | 14 | 71298907 | 71394600 | Margaric acid content QTL (156958) | 14 | 24537035 | 1.38E+08 |
| ENSSSCG00000029311 | 14 | 71298907 | 71394600 | Average instron (star probe) force QTL (3814) | 14 | 27334003 | 76280940 |
| ENSSSCG00000029311 | 14 | 71298907 | 71394600 | Fat androstenone level QTL (606) | 14 | 28351082 | 76280940 |
| ENSSSCG00000029311 | 14 | 71298907 | 71394600 | Fat androstenone level QTL (607) | 14 | 28351082 | 76280940 |
| ENSSSCG00000029311 | 14 | 71298907 | 71394600 | Fat androstenone level QTL (608) | 14 | 28351082 | 76280940 |
| ENSSSCG00000029311 | 14 | 71298907 | 71394600 | Fat androstenone level QTL (609) | 14 | 28351082 | 76280940 |
| ENSSSCG00000029311 | 14 | 71298907 | 71394600 | Carcass weight (hot) QTL (16873) | 14 | 40047493 | 85180383 |
| ENSSSCG00000029311 | 14 | 71298907 | 71394600 | Gadoleic acid content QTL (156905) | 14 | 42081651 | 80476391 |
| ENSSSCG00000029311 | 14 | 71298907 | 71394600 | Body weight QTL (8810) | 14 | 42081651 | 76280940 |
| ENSSSCG00000029311 | 14 | 71298907 | 71394600 | pH 24 hr post-mortem (loin) QTL (21861) | 14 | 58863458 | 1.38E+08 |
| ENSSSCG00000029311 | 14 | 71298907 | 71394600 | Fat protein content QTL (18013) | 14 | 58863458 | 1E+08 |
| ENSSSCG00000029311 | 14 | 71298907 | 71394600 | Salmonella count in liver and spleen QTL (11581) | 14 | 58863458 | 99546626 |
| ENSSSCG00000029311 | 14 | 71298907 | 71394600 | Adipocyte diameter QTL (12850) | 14 | 58863458 | 85180383 |
| ENSSSCG00000029311 | 14 | 71298907 | 71394600 | Adipocyte diameter QTL (12851) | 14 | 58863458 | 85180383 |
| ENSSSCG00000029311 | 14 | 71298907 | 71394600 | Adipocyte diameter QTL (12852) | 14 | 58863458 | 85180383 |
| ENSSSCG00000029311 | 14 | 71298907 | 71394600 | Backfat at last rib QTL (1169) | 14 | 58863458 | 85180383 |
| ENSSSCG00000029311 | 14 | 71298907 | 71394600 | Carcass length QTL (1261) | 14 | 58863458 | 85180383 |
| ENSSSCG00000029311 | 14 | 71298907 | 71394600 | Carcass length QTL (1153) | 14 | 58863458 | 85180383 |
| ENSSSCG00000029311 | 14 | 71298907 | 71394600 | Loin muscle area QTL (1154) | 14 | 58863458 | 85180383 |
| ENSSSCG00000029311 | 14 | 71298907 | 71394600 | Carcass weight (cold) QTL (1156) | 14 | 58863458 | 85180383 |
| ENSSSCG00000029311 | 14 | 71298907 | 71394600 | Body weight (slaughter) QTL (1159) | 14 | 58863458 | 85180383 |
| ENSSSCG00000029311 | 14 | 71298907 | 71394600 | Shoulder subcutaneous fat thickness QTL (1160) | 14 | 58863458 | 85180383 |
| ENSSSCG00000029311 | 14 | 71298907 | 71394600 | Average backfat thickness QTL (1161) | 14 | 58863458 | 85180383 |
| ENSSSCG00000029311 | 14 | 71298907 | 71394600 | Backfat at last rib QTL (1163) | 14 | 58863458 | 85180383 |
| ENSSSCG00000029311 | 14 | 71298907 | 71394600 | Meat color-a QTL (3289) | 14 | 58863458 | 80476391 |
| ENSSSCG00000029311 | 14 | 71298907 | 71394600 | Meat color a* QTL (21405) | 14 | 58863458 | 80476391 |
| ENSSSCG00000029311 | 14 | 71298907 | 71394600 | Shoulder weight, Boston QTL (21441) | 14 | 58863458 | 80476391 |
| ENSSSCG00000029311 | 14 | 71298907 | 71394600 | Average daily gain QTL (337) | 14 | 58863458 | 76280940 |
| ENSSSCG00000029311 | 14 | 71298907 | 71394600 | Meat color b* QTL (4007) | 14 | 58863458 | 76280940 |
| ENSSSCG00000029311 | 14 | 71298907 | 71394600 | Total shear work QTL (4008) | 14 | 58863458 | 76280940 |
| ENSSSCG00000029311 | 14 | 71298907 | 71394600 | Saturated fatty acid content QTL (156906) | 14 | 64838799 | 1.38E+08 |
| ENSSSCG00000029311 | 14 | 71298907 | 71394600 | Unsaturated fatty acid content QTL (156907) | 14 | 64838799 | 1.38E+08 |
| ENSSSCG00000029311 | 14 | 71298907 | 71394600 | Palmitoleic acid content QTL (156903) | 14 | 64838799 | 99546626 |
| ENSSSCG00000029311 | 14 | 71298907 | 71394600 | Stearic acid content QTL (156904) | 14 | 64838799 | 99546626 |
| ENSSSCG00000029311 | 14 | 71298907 | 71394600 | Aspartate aminotransferase activity QTL (10223) | 14 | 64838799 | 96876793 |
| ENSSSCG00000029311 | 14 | 71298907 | 71394600 | Aspartate aminotransferase activity QTL (10224) | 14 | 64838799 | 96876793 |
| ENSSSCG00000029311 | 14 | 71298907 | 71394600 | Aspartate aminotransferase activity QTL (10225) | 14 | 64838799 | 96876793 |
| ENSSSCG00000029311 | 14 | 71298907 | 71394600 | Aspartate aminotransferase activity QTL (10226) | 14 | 64838799 | 96876793 |
| ENSSSCG00000029311 | 14 | 71298907 | 71394600 | Aspartate aminotransferase activity QTL (10227) | 14 | 64838799 | 96876793 |
| ENSSSCG00000029311 | 14 | 71298907 | 71394600 | Aspartate aminotransferase activity QTL (10228) | 14 | 64838799 | 96876793 |
| ENSSSCG00000029311 | 14 | 71298907 | 71394600 | Aspartate aminotransferase activity QTL (10229) | 14 | 64838799 | 96876793 |
| ENSSSCG00000029311 | 14 | 71298907 | 71394600 | Aspartate aminotransferase activity QTL (10230) | 14 | 64838799 | 96876793 |
| ENSSSCG00000029311 | 14 | 71298907 | 71394600 | Aspartate aminotransferase activity QTL (10231) | 14 | 64838799 | 96876793 |
| ENSSSCG00000029311 | 14 | 71298907 | 71394600 | Aspartate aminotransferase activity QTL (10232) | 14 | 64838799 | 96876793 |
| ENSSSCG00000029311 | 14 | 71298907 | 71394600 | Aspartate aminotransferase activity QTL (10233) | 14 | 64838799 | 96876793 |
| ENSSSCG00000029311 | 14 | 71298907 | 71394600 | Aspartate aminotransferase activity QTL (10234) | 14 | 64838799 | 96876793 |
| ENSSSCG00000029311 | 14 | 71298907 | 71394600 | Aspartate aminotransferase activity QTL (10235) | 14 | 64838799 | 96876793 |
| ENSSSCG00000029311 | 14 | 71298907 | 71394600 | Aspartate aminotransferase activity QTL (7516) | 14 | 64838799 | 96876793 |
| ENSSSCG00000029311 | 14 | 71298907 | 71394600 | Granular leukocyte to lymphocyte ratio QTL (21335) | 14 | 64838799 | 88231620 |
| ENSSSCG00000029311 | 14 | 71298907 | 71394600 | Percentage type I fibers QTL (4022) | 14 | 66491552 | 83400494 |
| ENSSSCG00000010243 | 14 | 71827530 | 71894754 | Percentage type IIb fibers QTL (4045) | 14 | 67762901 | 72339758 |
| ENSSSCG00000010243 | 14 | 71827530 | 71894754 | Meat color L* QTL (38110) | 14 | 9407982 | 1.38E+08 |
| ENSSSCG00000010243 | 14 | 71827530 | 71894754 | Arachidonic acid content QTL (156959) | 14 | 9407982 | 1.38E+08 |
| ENSSSCG00000010243 | 14 | 71827530 | 71894754 | Haptoglobin concentration QTL (9653) | 14 | 9407982 | 76280940 |
| ENSSSCG00000010243 | 14 | 71827530 | 71894754 | backfat above muscle dorsi QTL (5991) | 14 | 9407982 | 76280940 |
| ENSSSCG00000010243 | 14 | 71827530 | 71894754 | Percentage type I fibers QTL (2822) | 14 | 9407982 | 76280940 |
| ENSSSCG00000010243 | 14 | 71827530 | 71894754 | Percentage type IIb fibers QTL (2823) | 14 | 9407982 | 76280940 |
| ENSSSCG00000010243 | 14 | 71827530 | 71894754 | Percentage type I fibers QTL (2824) | 14 | 9407982 | 76280940 |
| ENSSSCG00000010243 | 14 | 71827530 | 71894754 | Shear force at first peak QTL (3929) | 14 | 10933601 | 1.38E+08 |
| ENSSSCG00000010243 | 14 | 71827530 | 71894754 | Shear force at first peak QTL (3930) | 14 | 10933601 | 1.38E+08 |
| ENSSSCG00000010243 | 14 | 71827530 | 71894754 | Shear force at first peak QTL (3931) | 14 | 10933601 | 1.38E+08 |
| ENSSSCG00000010243 | 14 | 71827530 | 71894754 | Shear force QTL (3925) | 14 | 10933601 | 1.12E+08 |
| ENSSSCG00000010243 | 14 | 71827530 | 71894754 | Loin muscle area QTL (7) | 14 | 15256188 | 97893873 |
| ENSSSCG00000010243 | 14 | 71827530 | 71894754 | Abdominal fat weight QTL (21266) | 14 | 16273267 | 83654764 |
| ENSSSCG00000010243 | 14 | 71827530 | 71894754 | Capric acid content QTL (156957) | 14 | 24537035 | 1.38E+08 |
| ENSSSCG00000010243 | 14 | 71827530 | 71894754 | Margaric acid content QTL (156958) | 14 | 24537035 | 1.38E+08 |
| ENSSSCG00000010243 | 14 | 71827530 | 71894754 | Average instron (star probe) force QTL (3814) | 14 | 27334003 | 76280940 |
| ENSSSCG00000010243 | 14 | 71827530 | 71894754 | Fat androstenone level QTL (606) | 14 | 28351082 | 76280940 |
| ENSSSCG00000010243 | 14 | 71827530 | 71894754 | Fat androstenone level QTL (607) | 14 | 28351082 | 76280940 |
| ENSSSCG00000010243 | 14 | 71827530 | 71894754 | Fat androstenone level QTL (608) | 14 | 28351082 | 76280940 |
| ENSSSCG00000010243 | 14 | 71827530 | 71894754 | Fat androstenone level QTL (609) | 14 | 28351082 | 76280940 |
| ENSSSCG00000010243 | 14 | 71827530 | 71894754 | Carcass weight (hot) QTL (16873) | 14 | 40047493 | 85180383 |
| ENSSSCG00000010243 | 14 | 71827530 | 71894754 | Gadoleic acid content QTL (156905) | 14 | 42081651 | 80476391 |
| ENSSSCG00000010243 | 14 | 71827530 | 71894754 | Body weight QTL (8810) | 14 | 42081651 | 76280940 |
| ENSSSCG00000010243 | 14 | 71827530 | 71894754 | pH 24 hr post-mortem (loin) QTL (21861) | 14 | 58863458 | 1.38E+08 |
| ENSSSCG00000010243 | 14 | 71827530 | 71894754 | Fat protein content QTL (18013) | 14 | 58863458 | 1E+08 |
| ENSSSCG00000010243 | 14 | 71827530 | 71894754 | Salmonella count in liver and spleen QTL (11581) | 14 | 58863458 | 99546626 |
| ENSSSCG00000010243 | 14 | 71827530 | 71894754 | Adipocyte diameter QTL (12850) | 14 | 58863458 | 85180383 |
| ENSSSCG00000010243 | 14 | 71827530 | 71894754 | Adipocyte diameter QTL (12851) | 14 | 58863458 | 85180383 |
| ENSSSCG00000010243 | 14 | 71827530 | 71894754 | Adipocyte diameter QTL (12852) | 14 | 58863458 | 85180383 |
| ENSSSCG00000010243 | 14 | 71827530 | 71894754 | Backfat at last rib QTL (1169) | 14 | 58863458 | 85180383 |
| ENSSSCG00000010243 | 14 | 71827530 | 71894754 | Carcass length QTL (1261) | 14 | 58863458 | 85180383 |
| ENSSSCG00000010243 | 14 | 71827530 | 71894754 | Carcass length QTL (1153) | 14 | 58863458 | 85180383 |
| ENSSSCG00000010243 | 14 | 71827530 | 71894754 | Loin muscle area QTL (1154) | 14 | 58863458 | 85180383 |
| ENSSSCG00000010243 | 14 | 71827530 | 71894754 | Carcass weight (cold) QTL (1156) | 14 | 58863458 | 85180383 |
| ENSSSCG00000010243 | 14 | 71827530 | 71894754 | Body weight (slaughter) QTL (1159) | 14 | 58863458 | 85180383 |
| ENSSSCG00000010243 | 14 | 71827530 | 71894754 | Shoulder subcutaneous fat thickness QTL (1160) | 14 | 58863458 | 85180383 |
| ENSSSCG00000010243 | 14 | 71827530 | 71894754 | Average backfat thickness QTL (1161) | 14 | 58863458 | 85180383 |
| ENSSSCG00000010243 | 14 | 71827530 | 71894754 | Backfat at last rib QTL (1163) | 14 | 58863458 | 85180383 |
| ENSSSCG00000010243 | 14 | 71827530 | 71894754 | Meat color-a QTL (3289) | 14 | 58863458 | 80476391 |
| ENSSSCG00000010243 | 14 | 71827530 | 71894754 | Meat color a* QTL (21405) | 14 | 58863458 | 80476391 |
| ENSSSCG00000010243 | 14 | 71827530 | 71894754 | Shoulder weight, Boston QTL (21441) | 14 | 58863458 | 80476391 |
| ENSSSCG00000010243 | 14 | 71827530 | 71894754 | Average daily gain QTL (337) | 14 | 58863458 | 76280940 |
| ENSSSCG00000010243 | 14 | 71827530 | 71894754 | Meat color b* QTL (4007) | 14 | 58863458 | 76280940 |
| ENSSSCG00000010243 | 14 | 71827530 | 71894754 | Total shear work QTL (4008) | 14 | 58863458 | 76280940 |
| ENSSSCG00000010243 | 14 | 71827530 | 71894754 | Saturated fatty acid content QTL (156906) | 14 | 64838799 | 1.38E+08 |
| ENSSSCG00000010243 | 14 | 71827530 | 71894754 | Unsaturated fatty acid content QTL (156907) | 14 | 64838799 | 1.38E+08 |
| ENSSSCG00000010243 | 14 | 71827530 | 71894754 | Palmitoleic acid content QTL (156903) | 14 | 64838799 | 99546626 |
| ENSSSCG00000010243 | 14 | 71827530 | 71894754 | Stearic acid content QTL (156904) | 14 | 64838799 | 99546626 |
| ENSSSCG00000010243 | 14 | 71827530 | 71894754 | Aspartate aminotransferase activity QTL (10223) | 14 | 64838799 | 96876793 |
| ENSSSCG00000010243 | 14 | 71827530 | 71894754 | Aspartate aminotransferase activity QTL (10224) | 14 | 64838799 | 96876793 |
| ENSSSCG00000010243 | 14 | 71827530 | 71894754 | Aspartate aminotransferase activity QTL (10225) | 14 | 64838799 | 96876793 |
| ENSSSCG00000010243 | 14 | 71827530 | 71894754 | Aspartate aminotransferase activity QTL (10226) | 14 | 64838799 | 96876793 |
| ENSSSCG00000010243 | 14 | 71827530 | 71894754 | Aspartate aminotransferase activity QTL (10227) | 14 | 64838799 | 96876793 |
| ENSSSCG00000010243 | 14 | 71827530 | 71894754 | Aspartate aminotransferase activity QTL (10228) | 14 | 64838799 | 96876793 |
| ENSSSCG00000010243 | 14 | 71827530 | 71894754 | Aspartate aminotransferase activity QTL (10229) | 14 | 64838799 | 96876793 |
| ENSSSCG00000010243 | 14 | 71827530 | 71894754 | Aspartate aminotransferase activity QTL (10230) | 14 | 64838799 | 96876793 |
| ENSSSCG00000010243 | 14 | 71827530 | 71894754 | Aspartate aminotransferase activity QTL (10231) | 14 | 64838799 | 96876793 |
| ENSSSCG00000010243 | 14 | 71827530 | 71894754 | Aspartate aminotransferase activity QTL (10232) | 14 | 64838799 | 96876793 |
| ENSSSCG00000010243 | 14 | 71827530 | 71894754 | Aspartate aminotransferase activity QTL (10233) | 14 | 64838799 | 96876793 |
| ENSSSCG00000010243 | 14 | 71827530 | 71894754 | Aspartate aminotransferase activity QTL (10234) | 14 | 64838799 | 96876793 |
| ENSSSCG00000010243 | 14 | 71827530 | 71894754 | Aspartate aminotransferase activity QTL (10235) | 14 | 64838799 | 96876793 |
| ENSSSCG00000010243 | 14 | 71827530 | 71894754 | Aspartate aminotransferase activity QTL (7516) | 14 | 64838799 | 96876793 |
| ENSSSCG00000010243 | 14 | 71827530 | 71894754 | Granular leukocyte to lymphocyte ratio QTL (21335) | 14 | 64838799 | 88231620 |
| ENSSSCG00000010243 | 14 | 71827530 | 71894754 | Percentage type I fibers QTL (4022) | 14 | 66491552 | 83400494 |
| ENSSSCG00000010299 | 14 | 76205224 | 76234631 | Stearic acid content QTL (15872) | 14 | 74246781 | 82891954 |
| ENSSSCG00000010299 | 14 | 76205224 | 76234631 | Meat color L* QTL (38110) | 14 | 9407982 | 1.38E+08 |
| ENSSSCG00000010299 | 14 | 76205224 | 76234631 | Arachidonic acid content QTL (156959) | 14 | 9407982 | 1.38E+08 |
| ENSSSCG00000010299 | 14 | 76205224 | 76234631 | Haptoglobin concentration QTL (9653) | 14 | 9407982 | 76280940 |
| ENSSSCG00000010299 | 14 | 76205224 | 76234631 | backfat above muscle dorsi QTL (5991) | 14 | 9407982 | 76280940 |
| ENSSSCG00000010299 | 14 | 76205224 | 76234631 | Percentage type I fibers QTL (2822) | 14 | 9407982 | 76280940 |
| ENSSSCG00000010299 | 14 | 76205224 | 76234631 | Percentage type IIb fibers QTL (2823) | 14 | 9407982 | 76280940 |
| ENSSSCG00000010299 | 14 | 76205224 | 76234631 | Percentage type I fibers QTL (2824) | 14 | 9407982 | 76280940 |
| ENSSSCG00000010299 | 14 | 76205224 | 76234631 | Shear force at first peak QTL (3929) | 14 | 10933601 | 1.38E+08 |
| ENSSSCG00000010299 | 14 | 76205224 | 76234631 | Shear force at first peak QTL (3930) | 14 | 10933601 | 1.38E+08 |
| ENSSSCG00000010299 | 14 | 76205224 | 76234631 | Shear force at first peak QTL (3931) | 14 | 10933601 | 1.38E+08 |
| ENSSSCG00000010299 | 14 | 76205224 | 76234631 | Shear force QTL (3925) | 14 | 10933601 | 1.12E+08 |
| ENSSSCG00000010299 | 14 | 76205224 | 76234631 | Loin muscle area QTL (7) | 14 | 15256188 | 97893873 |
| ENSSSCG00000010299 | 14 | 76205224 | 76234631 | Abdominal fat weight QTL (21266) | 14 | 16273267 | 83654764 |
| ENSSSCG00000010299 | 14 | 76205224 | 76234631 | Capric acid content QTL (156957) | 14 | 24537035 | 1.38E+08 |
| ENSSSCG00000010299 | 14 | 76205224 | 76234631 | Margaric acid content QTL (156958) | 14 | 24537035 | 1.38E+08 |
| ENSSSCG00000010299 | 14 | 76205224 | 76234631 | Average instron (star probe) force QTL (3814) | 14 | 27334003 | 76280940 |
| ENSSSCG00000010299 | 14 | 76205224 | 76234631 | Fat androstenone level QTL (606) | 14 | 28351082 | 76280940 |
| ENSSSCG00000010299 | 14 | 76205224 | 76234631 | Fat androstenone level QTL (607) | 14 | 28351082 | 76280940 |
| ENSSSCG00000010299 | 14 | 76205224 | 76234631 | Fat androstenone level QTL (608) | 14 | 28351082 | 76280940 |
| ENSSSCG00000010299 | 14 | 76205224 | 76234631 | Fat androstenone level QTL (609) | 14 | 28351082 | 76280940 |
| ENSSSCG00000010299 | 14 | 76205224 | 76234631 | Carcass weight (hot) QTL (16873) | 14 | 40047493 | 85180383 |
| ENSSSCG00000010299 | 14 | 76205224 | 76234631 | Gadoleic acid content QTL (156905) | 14 | 42081651 | 80476391 |
| ENSSSCG00000010299 | 14 | 76205224 | 76234631 | Body weight QTL (8810) | 14 | 42081651 | 76280940 |
| ENSSSCG00000010299 | 14 | 76205224 | 76234631 | pH 24 hr post-mortem (loin) QTL (21861) | 14 | 58863458 | 1.38E+08 |
| ENSSSCG00000010299 | 14 | 76205224 | 76234631 | Fat protein content QTL (18013) | 14 | 58863458 | 1E+08 |
| ENSSSCG00000010299 | 14 | 76205224 | 76234631 | Salmonella count in liver and spleen QTL (11581) | 14 | 58863458 | 99546626 |
| ENSSSCG00000010299 | 14 | 76205224 | 76234631 | Adipocyte diameter QTL (12850) | 14 | 58863458 | 85180383 |
| ENSSSCG00000010299 | 14 | 76205224 | 76234631 | Adipocyte diameter QTL (12851) | 14 | 58863458 | 85180383 |
| ENSSSCG00000010299 | 14 | 76205224 | 76234631 | Adipocyte diameter QTL (12852) | 14 | 58863458 | 85180383 |
| ENSSSCG00000010299 | 14 | 76205224 | 76234631 | Backfat at last rib QTL (1169) | 14 | 58863458 | 85180383 |
| ENSSSCG00000010299 | 14 | 76205224 | 76234631 | Carcass length QTL (1261) | 14 | 58863458 | 85180383 |
| ENSSSCG00000010299 | 14 | 76205224 | 76234631 | Carcass length QTL (1153) | 14 | 58863458 | 85180383 |
| ENSSSCG00000010299 | 14 | 76205224 | 76234631 | Loin muscle area QTL (1154) | 14 | 58863458 | 85180383 |
| ENSSSCG00000010299 | 14 | 76205224 | 76234631 | Carcass weight (cold) QTL (1156) | 14 | 58863458 | 85180383 |
| ENSSSCG00000010299 | 14 | 76205224 | 76234631 | Body weight (slaughter) QTL (1159) | 14 | 58863458 | 85180383 |
| ENSSSCG00000010299 | 14 | 76205224 | 76234631 | Shoulder subcutaneous fat thickness QTL (1160) | 14 | 58863458 | 85180383 |
| ENSSSCG00000010299 | 14 | 76205224 | 76234631 | Average backfat thickness QTL (1161) | 14 | 58863458 | 85180383 |
| ENSSSCG00000010299 | 14 | 76205224 | 76234631 | Backfat at last rib QTL (1163) | 14 | 58863458 | 85180383 |
| ENSSSCG00000010299 | 14 | 76205224 | 76234631 | Meat color-a QTL (3289) | 14 | 58863458 | 80476391 |
| ENSSSCG00000010299 | 14 | 76205224 | 76234631 | Meat color a* QTL (21405) | 14 | 58863458 | 80476391 |
| ENSSSCG00000010299 | 14 | 76205224 | 76234631 | Shoulder weight, Boston QTL (21441) | 14 | 58863458 | 80476391 |
| ENSSSCG00000010299 | 14 | 76205224 | 76234631 | Average daily gain QTL (337) | 14 | 58863458 | 76280940 |
| ENSSSCG00000010299 | 14 | 76205224 | 76234631 | Meat color b* QTL (4007) | 14 | 58863458 | 76280940 |
| ENSSSCG00000010299 | 14 | 76205224 | 76234631 | Total shear work QTL (4008) | 14 | 58863458 | 76280940 |
| ENSSSCG00000010299 | 14 | 76205224 | 76234631 | Saturated fatty acid content QTL (156906) | 14 | 64838799 | 1.38E+08 |
| ENSSSCG00000010299 | 14 | 76205224 | 76234631 | Unsaturated fatty acid content QTL (156907) | 14 | 64838799 | 1.38E+08 |
| ENSSSCG00000010299 | 14 | 76205224 | 76234631 | Palmitoleic acid content QTL (156903) | 14 | 64838799 | 99546626 |
| ENSSSCG00000010299 | 14 | 76205224 | 76234631 | Stearic acid content QTL (156904) | 14 | 64838799 | 99546626 |
| ENSSSCG00000010299 | 14 | 76205224 | 76234631 | Aspartate aminotransferase activity QTL (10223) | 14 | 64838799 | 96876793 |
| ENSSSCG00000010299 | 14 | 76205224 | 76234631 | Aspartate aminotransferase activity QTL (10224) | 14 | 64838799 | 96876793 |
| ENSSSCG00000010299 | 14 | 76205224 | 76234631 | Aspartate aminotransferase activity QTL (10225) | 14 | 64838799 | 96876793 |
| ENSSSCG00000010299 | 14 | 76205224 | 76234631 | Aspartate aminotransferase activity QTL (10226) | 14 | 64838799 | 96876793 |
| ENSSSCG00000010299 | 14 | 76205224 | 76234631 | Aspartate aminotransferase activity QTL (10227) | 14 | 64838799 | 96876793 |
| ENSSSCG00000010299 | 14 | 76205224 | 76234631 | Aspartate aminotransferase activity QTL (10228) | 14 | 64838799 | 96876793 |
| ENSSSCG00000010299 | 14 | 76205224 | 76234631 | Aspartate aminotransferase activity QTL (10229) | 14 | 64838799 | 96876793 |
| ENSSSCG00000010299 | 14 | 76205224 | 76234631 | Aspartate aminotransferase activity QTL (10230) | 14 | 64838799 | 96876793 |
| ENSSSCG00000010299 | 14 | 76205224 | 76234631 | Aspartate aminotransferase activity QTL (10231) | 14 | 64838799 | 96876793 |
| ENSSSCG00000010299 | 14 | 76205224 | 76234631 | Aspartate aminotransferase activity QTL (10232) | 14 | 64838799 | 96876793 |
| ENSSSCG00000010299 | 14 | 76205224 | 76234631 | Aspartate aminotransferase activity QTL (10233) | 14 | 64838799 | 96876793 |
| ENSSSCG00000010299 | 14 | 76205224 | 76234631 | Aspartate aminotransferase activity QTL (10234) | 14 | 64838799 | 96876793 |
| ENSSSCG00000010299 | 14 | 76205224 | 76234631 | Aspartate aminotransferase activity QTL (10235) | 14 | 64838799 | 96876793 |
| ENSSSCG00000010299 | 14 | 76205224 | 76234631 | Aspartate aminotransferase activity QTL (7516) | 14 | 64838799 | 96876793 |
| ENSSSCG00000010299 | 14 | 76205224 | 76234631 | Granular leukocyte to lymphocyte ratio QTL (21335) | 14 | 64838799 | 88231620 |
| ENSSSCG00000010299 | 14 | 76205224 | 76234631 | Percentage type I fibers QTL (4022) | 14 | 66491552 | 83400494 |
| ENSSSCG00000026268 | 14 | 80249693 | 80377505 | Stearic acid content QTL (15872) | 14 | 74246781 | 82891954 |
| ENSSSCG00000026268 | 14 | 80249693 | 80377505 | pH 24 hr post-mortem (loin) QTL (228) | 14 | 76280940 | 1.12E+08 |
| ENSSSCG00000026268 | 14 | 80249693 | 80377505 | Meat color-b QTL (239) | 14 | 76280940 | 1.12E+08 |
| ENSSSCG00000026268 | 14 | 80249693 | 80377505 | Percentage type IIb fibers QTL (7044) | 14 | 76280940 | 1.12E+08 |
| ENSSSCG00000026268 | 14 | 80249693 | 80377505 | Oleic acid content QTL (15865) | 14 | 76280940 | 96876793 |
| ENSSSCG00000026268 | 14 | 80249693 | 80377505 | Melting point QTL (15866) | 14 | 76280940 | 96876793 |
| ENSSSCG00000026268 | 14 | 80249693 | 80377505 | Stearic acid content QTL (15867) | 14 | 76280940 | 96876793 |
| ENSSSCG00000026268 | 14 | 80249693 | 80377505 | Oleic acid content QTL (15868) | 14 | 76280940 | 96876793 |
| ENSSSCG00000026268 | 14 | 80249693 | 80377505 | Melting point QTL (15869) | 14 | 76280940 | 96876793 |
| ENSSSCG00000026268 | 14 | 80249693 | 80377505 | Oleic acid content QTL (15873) | 14 | 76280940 | 96876793 |
| ENSSSCG00000026268 | 14 | 80249693 | 80377505 | androstenone, sensory panel QTL (4235) | 14 | 76280940 | 96876793 |
| ENSSSCG00000026268 | 14 | 80249693 | 80377505 | Backfat at last rib QTL (74) | 14 | 77323446 | 1.03E+08 |
| ENSSSCG00000026268 | 14 | 80249693 | 80377505 | Meat color L* QTL (38110) | 14 | 9407982 | 1.38E+08 |
| ENSSSCG00000026268 | 14 | 80249693 | 80377505 | Arachidonic acid content QTL (156959) | 14 | 9407982 | 1.38E+08 |
| ENSSSCG00000026268 | 14 | 80249693 | 80377505 | Shear force at first peak QTL (3929) | 14 | 10933601 | 1.38E+08 |
| ENSSSCG00000026268 | 14 | 80249693 | 80377505 | Shear force at first peak QTL (3930) | 14 | 10933601 | 1.38E+08 |
| ENSSSCG00000026268 | 14 | 80249693 | 80377505 | Shear force at first peak QTL (3931) | 14 | 10933601 | 1.38E+08 |
| ENSSSCG00000026268 | 14 | 80249693 | 80377505 | Shear force QTL (3925) | 14 | 10933601 | 1.12E+08 |
| ENSSSCG00000026268 | 14 | 80249693 | 80377505 | Loin muscle area QTL (7) | 14 | 15256188 | 97893873 |
| ENSSSCG00000026268 | 14 | 80249693 | 80377505 | Abdominal fat weight QTL (21266) | 14 | 16273267 | 83654764 |
| ENSSSCG00000026268 | 14 | 80249693 | 80377505 | Capric acid content QTL (156957) | 14 | 24537035 | 1.38E+08 |
| ENSSSCG00000026268 | 14 | 80249693 | 80377505 | Margaric acid content QTL (156958) | 14 | 24537035 | 1.38E+08 |
| ENSSSCG00000026268 | 14 | 80249693 | 80377505 | Carcass weight (hot) QTL (16873) | 14 | 40047493 | 85180383 |
| ENSSSCG00000026268 | 14 | 80249693 | 80377505 | Gadoleic acid content QTL (156905) | 14 | 42081651 | 80476391 |
| ENSSSCG00000026268 | 14 | 80249693 | 80377505 | pH 24 hr post-mortem (loin) QTL (21861) | 14 | 58863458 | 1.38E+08 |
| ENSSSCG00000026268 | 14 | 80249693 | 80377505 | Fat protein content QTL (18013) | 14 | 58863458 | 1E+08 |
| ENSSSCG00000026268 | 14 | 80249693 | 80377505 | Salmonella count in liver and spleen QTL (11581) | 14 | 58863458 | 99546626 |
| ENSSSCG00000026268 | 14 | 80249693 | 80377505 | Adipocyte diameter QTL (12850) | 14 | 58863458 | 85180383 |
| ENSSSCG00000026268 | 14 | 80249693 | 80377505 | Adipocyte diameter QTL (12851) | 14 | 58863458 | 85180383 |
| ENSSSCG00000026268 | 14 | 80249693 | 80377505 | Adipocyte diameter QTL (12852) | 14 | 58863458 | 85180383 |
| ENSSSCG00000026268 | 14 | 80249693 | 80377505 | Backfat at last rib QTL (1169) | 14 | 58863458 | 85180383 |
| ENSSSCG00000026268 | 14 | 80249693 | 80377505 | Carcass length QTL (1261) | 14 | 58863458 | 85180383 |
| ENSSSCG00000026268 | 14 | 80249693 | 80377505 | Carcass length QTL (1153) | 14 | 58863458 | 85180383 |
| ENSSSCG00000026268 | 14 | 80249693 | 80377505 | Loin muscle area QTL (1154) | 14 | 58863458 | 85180383 |
| ENSSSCG00000026268 | 14 | 80249693 | 80377505 | Carcass weight (cold) QTL (1156) | 14 | 58863458 | 85180383 |
| ENSSSCG00000026268 | 14 | 80249693 | 80377505 | Body weight (slaughter) QTL (1159) | 14 | 58863458 | 85180383 |
| ENSSSCG00000026268 | 14 | 80249693 | 80377505 | Shoulder subcutaneous fat thickness QTL (1160) | 14 | 58863458 | 85180383 |
| ENSSSCG00000026268 | 14 | 80249693 | 80377505 | Average backfat thickness QTL (1161) | 14 | 58863458 | 85180383 |
| ENSSSCG00000026268 | 14 | 80249693 | 80377505 | Backfat at last rib QTL (1163) | 14 | 58863458 | 85180383 |
| ENSSSCG00000026268 | 14 | 80249693 | 80377505 | Meat color-a QTL (3289) | 14 | 58863458 | 80476391 |
| ENSSSCG00000026268 | 14 | 80249693 | 80377505 | Meat color a* QTL (21405) | 14 | 58863458 | 80476391 |
| ENSSSCG00000026268 | 14 | 80249693 | 80377505 | Shoulder weight, Boston QTL (21441) | 14 | 58863458 | 80476391 |
| ENSSSCG00000026268 | 14 | 80249693 | 80377505 | Saturated fatty acid content QTL (156906) | 14 | 64838799 | 1.38E+08 |
| ENSSSCG00000026268 | 14 | 80249693 | 80377505 | Unsaturated fatty acid content QTL (156907) | 14 | 64838799 | 1.38E+08 |
| ENSSSCG00000026268 | 14 | 80249693 | 80377505 | Palmitoleic acid content QTL (156903) | 14 | 64838799 | 99546626 |
| ENSSSCG00000026268 | 14 | 80249693 | 80377505 | Stearic acid content QTL (156904) | 14 | 64838799 | 99546626 |
| ENSSSCG00000026268 | 14 | 80249693 | 80377505 | Aspartate aminotransferase activity QTL (10223) | 14 | 64838799 | 96876793 |
| ENSSSCG00000026268 | 14 | 80249693 | 80377505 | Aspartate aminotransferase activity QTL (10224) | 14 | 64838799 | 96876793 |
| ENSSSCG00000026268 | 14 | 80249693 | 80377505 | Aspartate aminotransferase activity QTL (10225) | 14 | 64838799 | 96876793 |
| ENSSSCG00000026268 | 14 | 80249693 | 80377505 | Aspartate aminotransferase activity QTL (10226) | 14 | 64838799 | 96876793 |
| ENSSSCG00000026268 | 14 | 80249693 | 80377505 | Aspartate aminotransferase activity QTL (10227) | 14 | 64838799 | 96876793 |
| ENSSSCG00000026268 | 14 | 80249693 | 80377505 | Aspartate aminotransferase activity QTL (10228) | 14 | 64838799 | 96876793 |
| ENSSSCG00000026268 | 14 | 80249693 | 80377505 | Aspartate aminotransferase activity QTL (10229) | 14 | 64838799 | 96876793 |
| ENSSSCG00000026268 | 14 | 80249693 | 80377505 | Aspartate aminotransferase activity QTL (10230) | 14 | 64838799 | 96876793 |
| ENSSSCG00000026268 | 14 | 80249693 | 80377505 | Aspartate aminotransferase activity QTL (10231) | 14 | 64838799 | 96876793 |
| ENSSSCG00000026268 | 14 | 80249693 | 80377505 | Aspartate aminotransferase activity QTL (10232) | 14 | 64838799 | 96876793 |
| ENSSSCG00000026268 | 14 | 80249693 | 80377505 | Aspartate aminotransferase activity QTL (10233) | 14 | 64838799 | 96876793 |
| ENSSSCG00000026268 | 14 | 80249693 | 80377505 | Aspartate aminotransferase activity QTL (10234) | 14 | 64838799 | 96876793 |
| ENSSSCG00000026268 | 14 | 80249693 | 80377505 | Aspartate aminotransferase activity QTL (10235) | 14 | 64838799 | 96876793 |
| ENSSSCG00000026268 | 14 | 80249693 | 80377505 | Aspartate aminotransferase activity QTL (7516) | 14 | 64838799 | 96876793 |
| ENSSSCG00000026268 | 14 | 80249693 | 80377505 | Granular leukocyte to lymphocyte ratio QTL (21335) | 14 | 64838799 | 88231620 |
| ENSSSCG00000026268 | 14 | 80249693 | 80377505 | Percentage type I fibers QTL (4022) | 14 | 66491552 | 83400494 |
| ENSSSCG00000026268 | 14 | 80249693 | 80377505 | Toll-like receptor 9 level QTL (17626) | 14 | 76280940 | 1.42E+08 |
| ENSSSCG00000026268 | 14 | 80249693 | 80377505 | Meat color OPTO QTL (12076) | 14 | 76280940 | 1.42E+08 |
| ENSSSCG00000026268 | 14 | 80249693 | 80377505 | Thawing loss QTL (12077) | 14 | 76280940 | 1.42E+08 |
| ENSSSCG00000026268 | 14 | 80249693 | 80377505 | Shear force QTL (12079) | 14 | 76280940 | 1.42E+08 |
| ENSSSCG00000026268 | 14 | 80249693 | 80377505 | Shear force QTL (12080) | 14 | 76280940 | 1.42E+08 |
| ENSSSCG00000026268 | 14 | 80249693 | 80377505 | Estimated carcass lean content QTL (12081) | 14 | 76280940 | 1.42E+08 |
| ENSSSCG00000026268 | 14 | 80249693 | 80377505 | Loin muscle area QTL (12082) | 14 | 76280940 | 1.42E+08 |
| ENSSSCG00000026268 | 14 | 80249693 | 80377505 | pH 24 hr post mortem (ham) QTL (12110) | 14 | 76280940 | 1.42E+08 |
| ENSSSCG00000026268 | 14 | 80249693 | 80377505 | Estimated carcass lean content QTL (12111) | 14 | 76280940 | 1.42E+08 |
| ENSSSCG00000026268 | 14 | 80249693 | 80377505 | Loin muscle depth QTL (12112) | 14 | 76280940 | 1.42E+08 |
| ENSSSCG00000026268 | 14 | 80249693 | 80377505 | Loin muscle area QTL (12113) | 14 | 76280940 | 1.42E+08 |
| ENSSSCG00000026268 | 14 | 80249693 | 80377505 | Loin muscle area QTL (12114) | 14 | 76280940 | 1.42E+08 |
| ENSSSCG00000010360 | 14 | 87742459 | 87891793 | pH 24 hr post-mortem (loin) QTL (228) | 14 | 76280940 | 1.12E+08 |
| ENSSSCG00000010360 | 14 | 87742459 | 87891793 | Meat color-b QTL (239) | 14 | 76280940 | 1.12E+08 |
| ENSSSCG00000010360 | 14 | 87742459 | 87891793 | Percentage type IIb fibers QTL (7044) | 14 | 76280940 | 1.12E+08 |
| ENSSSCG00000010360 | 14 | 87742459 | 87891793 | Oleic acid content QTL (15865) | 14 | 76280940 | 96876793 |
| ENSSSCG00000010360 | 14 | 87742459 | 87891793 | Melting point QTL (15866) | 14 | 76280940 | 96876793 |
| ENSSSCG00000010360 | 14 | 87742459 | 87891793 | Stearic acid content QTL (15867) | 14 | 76280940 | 96876793 |
| ENSSSCG00000010360 | 14 | 87742459 | 87891793 | Oleic acid content QTL (15868) | 14 | 76280940 | 96876793 |
| ENSSSCG00000010360 | 14 | 87742459 | 87891793 | Melting point QTL (15869) | 14 | 76280940 | 96876793 |
| ENSSSCG00000010360 | 14 | 87742459 | 87891793 | Oleic acid content QTL (15873) | 14 | 76280940 | 96876793 |
| ENSSSCG00000010360 | 14 | 87742459 | 87891793 | androstenone, sensory panel QTL (4235) | 14 | 76280940 | 96876793 |
| ENSSSCG00000010360 | 14 | 87742459 | 87891793 | Backfat at last rib QTL (74) | 14 | 77323446 | 1.03E+08 |
| ENSSSCG00000010360 | 14 | 87742459 | 87891793 | Carcass weight (cold) QTL (75) | 14 | 81099352 | 1.04E+08 |
| ENSSSCG00000010360 | 14 | 87742459 | 87891793 | Meat color L* QTL (38110) | 14 | 9407982 | 1.38E+08 |
| ENSSSCG00000010360 | 14 | 87742459 | 87891793 | Arachidonic acid content QTL (156959) | 14 | 9407982 | 1.38E+08 |
| ENSSSCG00000010360 | 14 | 87742459 | 87891793 | Shear force at first peak QTL (3929) | 14 | 10933601 | 1.38E+08 |
| ENSSSCG00000010360 | 14 | 87742459 | 87891793 | Shear force at first peak QTL (3930) | 14 | 10933601 | 1.38E+08 |
| ENSSSCG00000010360 | 14 | 87742459 | 87891793 | Shear force at first peak QTL (3931) | 14 | 10933601 | 1.38E+08 |
| ENSSSCG00000010360 | 14 | 87742459 | 87891793 | Shear force QTL (3925) | 14 | 10933601 | 1.12E+08 |
| ENSSSCG00000010360 | 14 | 87742459 | 87891793 | Loin muscle area QTL (7) | 14 | 15256188 | 97893873 |
| ENSSSCG00000010360 | 14 | 87742459 | 87891793 | Capric acid content QTL (156957) | 14 | 24537035 | 1.38E+08 |
| ENSSSCG00000010360 | 14 | 87742459 | 87891793 | Margaric acid content QTL (156958) | 14 | 24537035 | 1.38E+08 |
| ENSSSCG00000010360 | 14 | 87742459 | 87891793 | pH 24 hr post-mortem (loin) QTL (21861) | 14 | 58863458 | 1.38E+08 |
| ENSSSCG00000010360 | 14 | 87742459 | 87891793 | Fat protein content QTL (18013) | 14 | 58863458 | 1E+08 |
| ENSSSCG00000010360 | 14 | 87742459 | 87891793 | Salmonella count in liver and spleen QTL (11581) | 14 | 58863458 | 99546626 |
| ENSSSCG00000010360 | 14 | 87742459 | 87891793 | Saturated fatty acid content QTL (156906) | 14 | 64838799 | 1.38E+08 |
| ENSSSCG00000010360 | 14 | 87742459 | 87891793 | Unsaturated fatty acid content QTL (156907) | 14 | 64838799 | 1.38E+08 |
| ENSSSCG00000010360 | 14 | 87742459 | 87891793 | Palmitoleic acid content QTL (156903) | 14 | 64838799 | 99546626 |
| ENSSSCG00000010360 | 14 | 87742459 | 87891793 | Stearic acid content QTL (156904) | 14 | 64838799 | 99546626 |
| ENSSSCG00000010360 | 14 | 87742459 | 87891793 | Aspartate aminotransferase activity QTL (10223) | 14 | 64838799 | 96876793 |
| ENSSSCG00000010360 | 14 | 87742459 | 87891793 | Aspartate aminotransferase activity QTL (10224) | 14 | 64838799 | 96876793 |
| ENSSSCG00000010360 | 14 | 87742459 | 87891793 | Aspartate aminotransferase activity QTL (10225) | 14 | 64838799 | 96876793 |
| ENSSSCG00000010360 | 14 | 87742459 | 87891793 | Aspartate aminotransferase activity QTL (10226) | 14 | 64838799 | 96876793 |
| ENSSSCG00000010360 | 14 | 87742459 | 87891793 | Aspartate aminotransferase activity QTL (10227) | 14 | 64838799 | 96876793 |
| ENSSSCG00000010360 | 14 | 87742459 | 87891793 | Aspartate aminotransferase activity QTL (10228) | 14 | 64838799 | 96876793 |
| ENSSSCG00000010360 | 14 | 87742459 | 87891793 | Aspartate aminotransferase activity QTL (10229) | 14 | 64838799 | 96876793 |
| ENSSSCG00000010360 | 14 | 87742459 | 87891793 | Aspartate aminotransferase activity QTL (10230) | 14 | 64838799 | 96876793 |
| ENSSSCG00000010360 | 14 | 87742459 | 87891793 | Aspartate aminotransferase activity QTL (10231) | 14 | 64838799 | 96876793 |
| ENSSSCG00000010360 | 14 | 87742459 | 87891793 | Aspartate aminotransferase activity QTL (10232) | 14 | 64838799 | 96876793 |
| ENSSSCG00000010360 | 14 | 87742459 | 87891793 | Aspartate aminotransferase activity QTL (10233) | 14 | 64838799 | 96876793 |
| ENSSSCG00000010360 | 14 | 87742459 | 87891793 | Aspartate aminotransferase activity QTL (10234) | 14 | 64838799 | 96876793 |
| ENSSSCG00000010360 | 14 | 87742459 | 87891793 | Aspartate aminotransferase activity QTL (10235) | 14 | 64838799 | 96876793 |
| ENSSSCG00000010360 | 14 | 87742459 | 87891793 | Aspartate aminotransferase activity QTL (7516) | 14 | 64838799 | 96876793 |
| ENSSSCG00000010360 | 14 | 87742459 | 87891793 | Granular leukocyte to lymphocyte ratio QTL (21335) | 14 | 64838799 | 88231620 |
| ENSSSCG00000010360 | 14 | 87742459 | 87891793 | Toll-like receptor 9 level QTL (17626) | 14 | 76280940 | 1.42E+08 |
| ENSSSCG00000010360 | 14 | 87742459 | 87891793 | Meat color OPTO QTL (12076) | 14 | 76280940 | 1.42E+08 |
| ENSSSCG00000010360 | 14 | 87742459 | 87891793 | Thawing loss QTL (12077) | 14 | 76280940 | 1.42E+08 |
| ENSSSCG00000010360 | 14 | 87742459 | 87891793 | Shear force QTL (12079) | 14 | 76280940 | 1.42E+08 |
| ENSSSCG00000010360 | 14 | 87742459 | 87891793 | Shear force QTL (12080) | 14 | 76280940 | 1.42E+08 |
| ENSSSCG00000010360 | 14 | 87742459 | 87891793 | Estimated carcass lean content QTL (12081) | 14 | 76280940 | 1.42E+08 |
| ENSSSCG00000010360 | 14 | 87742459 | 87891793 | Loin muscle area QTL (12082) | 14 | 76280940 | 1.42E+08 |
| ENSSSCG00000010360 | 14 | 87742459 | 87891793 | pH 24 hr post mortem (ham) QTL (12110) | 14 | 76280940 | 1.42E+08 |
| ENSSSCG00000010360 | 14 | 87742459 | 87891793 | Estimated carcass lean content QTL (12111) | 14 | 76280940 | 1.42E+08 |
| ENSSSCG00000010360 | 14 | 87742459 | 87891793 | Loin muscle depth QTL (12112) | 14 | 76280940 | 1.42E+08 |
| ENSSSCG00000010360 | 14 | 87742459 | 87891793 | Loin muscle area QTL (12113) | 14 | 76280940 | 1.42E+08 |
| ENSSSCG00000010360 | 14 | 87742459 | 87891793 | Loin muscle area QTL (12114) | 14 | 76280940 | 1.42E+08 |
| ENSSSCG00000033323 | 14 | 92294642 | 92382213 | pH 24 hr post-mortem (loin) QTL (228) | 14 | 76280940 | 1.12E+08 |
| ENSSSCG00000033323 | 14 | 92294642 | 92382213 | Meat color-b QTL (239) | 14 | 76280940 | 1.12E+08 |
| ENSSSCG00000033323 | 14 | 92294642 | 92382213 | Percentage type IIb fibers QTL (7044) | 14 | 76280940 | 1.12E+08 |
| ENSSSCG00000033323 | 14 | 92294642 | 92382213 | Oleic acid content QTL (15865) | 14 | 76280940 | 96876793 |
| ENSSSCG00000033323 | 14 | 92294642 | 92382213 | Melting point QTL (15866) | 14 | 76280940 | 96876793 |
| ENSSSCG00000033323 | 14 | 92294642 | 92382213 | Stearic acid content QTL (15867) | 14 | 76280940 | 96876793 |
| ENSSSCG00000033323 | 14 | 92294642 | 92382213 | Oleic acid content QTL (15868) | 14 | 76280940 | 96876793 |
| ENSSSCG00000033323 | 14 | 92294642 | 92382213 | Melting point QTL (15869) | 14 | 76280940 | 96876793 |
| ENSSSCG00000033323 | 14 | 92294642 | 92382213 | Oleic acid content QTL (15873) | 14 | 76280940 | 96876793 |
| ENSSSCG00000033323 | 14 | 92294642 | 92382213 | androstenone, sensory panel QTL (4235) | 14 | 76280940 | 96876793 |
| ENSSSCG00000033323 | 14 | 92294642 | 92382213 | Backfat at last rib QTL (74) | 14 | 77323446 | 1.03E+08 |
| ENSSSCG00000033323 | 14 | 92294642 | 92382213 | Carcass weight (cold) QTL (75) | 14 | 81099352 | 1.04E+08 |
| ENSSSCG00000033323 | 14 | 92294642 | 92382213 | Light butt ham weight QTL (3080) | 14 | 88231620 | 1.12E+08 |
| ENSSSCG00000033323 | 14 | 92294642 | 92382213 | Meat color density QTL (3005) | 14 | 91664262 | 1.12E+08 |
| ENSSSCG00000033323 | 14 | 92294642 | 92382213 | Meat color L* QTL (3006) | 14 | 91664262 | 1.12E+08 |
| ENSSSCG00000033323 | 14 | 92294642 | 92382213 | Meat color score QTL (3007) | 14 | 91664262 | 1.12E+08 |
| ENSSSCG00000033323 | 14 | 92294642 | 92382213 | Drip loss QTL (3008) | 14 | 91664262 | 1.12E+08 |
| ENSSSCG00000033323 | 14 | 92294642 | 92382213 | Myristic acid content QTL (30904) | 14 | 91664262 | 96876793 |
| ENSSSCG00000033323 | 14 | 92294642 | 92382213 | Meat color L* QTL (38110) | 14 | 9407982 | 1.38E+08 |
| ENSSSCG00000033323 | 14 | 92294642 | 92382213 | Arachidonic acid content QTL (156959) | 14 | 9407982 | 1.38E+08 |
| ENSSSCG00000033323 | 14 | 92294642 | 92382213 | Shear force at first peak QTL (3929) | 14 | 10933601 | 1.38E+08 |
| ENSSSCG00000033323 | 14 | 92294642 | 92382213 | Shear force at first peak QTL (3930) | 14 | 10933601 | 1.38E+08 |
| ENSSSCG00000033323 | 14 | 92294642 | 92382213 | Shear force at first peak QTL (3931) | 14 | 10933601 | 1.38E+08 |
| ENSSSCG00000033323 | 14 | 92294642 | 92382213 | Shear force QTL (3925) | 14 | 10933601 | 1.12E+08 |
| ENSSSCG00000033323 | 14 | 92294642 | 92382213 | Loin muscle area QTL (7) | 14 | 15256188 | 97893873 |
| ENSSSCG00000033323 | 14 | 92294642 | 92382213 | Capric acid content QTL (156957) | 14 | 24537035 | 1.38E+08 |
| ENSSSCG00000033323 | 14 | 92294642 | 92382213 | Margaric acid content QTL (156958) | 14 | 24537035 | 1.38E+08 |
| ENSSSCG00000033323 | 14 | 92294642 | 92382213 | pH 24 hr post-mortem (loin) QTL (21861) | 14 | 58863458 | 1.38E+08 |
| ENSSSCG00000033323 | 14 | 92294642 | 92382213 | Fat protein content QTL (18013) | 14 | 58863458 | 1E+08 |
| ENSSSCG00000033323 | 14 | 92294642 | 92382213 | Salmonella count in liver and spleen QTL (11581) | 14 | 58863458 | 99546626 |
| ENSSSCG00000033323 | 14 | 92294642 | 92382213 | Saturated fatty acid content QTL (156906) | 14 | 64838799 | 1.38E+08 |
| ENSSSCG00000033323 | 14 | 92294642 | 92382213 | Unsaturated fatty acid content QTL (156907) | 14 | 64838799 | 1.38E+08 |
| ENSSSCG00000033323 | 14 | 92294642 | 92382213 | Palmitoleic acid content QTL (156903) | 14 | 64838799 | 99546626 |
| ENSSSCG00000033323 | 14 | 92294642 | 92382213 | Stearic acid content QTL (156904) | 14 | 64838799 | 99546626 |
| ENSSSCG00000033323 | 14 | 92294642 | 92382213 | Aspartate aminotransferase activity QTL (10223) | 14 | 64838799 | 96876793 |
| ENSSSCG00000033323 | 14 | 92294642 | 92382213 | Aspartate aminotransferase activity QTL (10224) | 14 | 64838799 | 96876793 |
| ENSSSCG00000033323 | 14 | 92294642 | 92382213 | Aspartate aminotransferase activity QTL (10225) | 14 | 64838799 | 96876793 |
| ENSSSCG00000033323 | 14 | 92294642 | 92382213 | Aspartate aminotransferase activity QTL (10226) | 14 | 64838799 | 96876793 |
| ENSSSCG00000033323 | 14 | 92294642 | 92382213 | Aspartate aminotransferase activity QTL (10227) | 14 | 64838799 | 96876793 |
| ENSSSCG00000033323 | 14 | 92294642 | 92382213 | Aspartate aminotransferase activity QTL (10228) | 14 | 64838799 | 96876793 |
| ENSSSCG00000033323 | 14 | 92294642 | 92382213 | Aspartate aminotransferase activity QTL (10229) | 14 | 64838799 | 96876793 |
| ENSSSCG00000033323 | 14 | 92294642 | 92382213 | Aspartate aminotransferase activity QTL (10230) | 14 | 64838799 | 96876793 |
| ENSSSCG00000033323 | 14 | 92294642 | 92382213 | Aspartate aminotransferase activity QTL (10231) | 14 | 64838799 | 96876793 |
| ENSSSCG00000033323 | 14 | 92294642 | 92382213 | Aspartate aminotransferase activity QTL (10232) | 14 | 64838799 | 96876793 |
| ENSSSCG00000033323 | 14 | 92294642 | 92382213 | Aspartate aminotransferase activity QTL (10233) | 14 | 64838799 | 96876793 |
| ENSSSCG00000033323 | 14 | 92294642 | 92382213 | Aspartate aminotransferase activity QTL (10234) | 14 | 64838799 | 96876793 |
| ENSSSCG00000033323 | 14 | 92294642 | 92382213 | Aspartate aminotransferase activity QTL (10235) | 14 | 64838799 | 96876793 |
| ENSSSCG00000033323 | 14 | 92294642 | 92382213 | Aspartate aminotransferase activity QTL (7516) | 14 | 64838799 | 96876793 |
| ENSSSCG00000033323 | 14 | 92294642 | 92382213 | Toll-like receptor 9 level QTL (17626) | 14 | 76280940 | 1.42E+08 |
| ENSSSCG00000033323 | 14 | 92294642 | 92382213 | Meat color OPTO QTL (12076) | 14 | 76280940 | 1.42E+08 |
| ENSSSCG00000033323 | 14 | 92294642 | 92382213 | Thawing loss QTL (12077) | 14 | 76280940 | 1.42E+08 |
| ENSSSCG00000033323 | 14 | 92294642 | 92382213 | Shear force QTL (12079) | 14 | 76280940 | 1.42E+08 |
| ENSSSCG00000033323 | 14 | 92294642 | 92382213 | Shear force QTL (12080) | 14 | 76280940 | 1.42E+08 |
| ENSSSCG00000033323 | 14 | 92294642 | 92382213 | Estimated carcass lean content QTL (12081) | 14 | 76280940 | 1.42E+08 |
| ENSSSCG00000033323 | 14 | 92294642 | 92382213 | Loin muscle area QTL (12082) | 14 | 76280940 | 1.42E+08 |
| ENSSSCG00000033323 | 14 | 92294642 | 92382213 | pH 24 hr post mortem (ham) QTL (12110) | 14 | 76280940 | 1.42E+08 |
| ENSSSCG00000033323 | 14 | 92294642 | 92382213 | Estimated carcass lean content QTL (12111) | 14 | 76280940 | 1.42E+08 |
| ENSSSCG00000033323 | 14 | 92294642 | 92382213 | Loin muscle depth QTL (12112) | 14 | 76280940 | 1.42E+08 |
| ENSSSCG00000033323 | 14 | 92294642 | 92382213 | Loin muscle area QTL (12113) | 14 | 76280940 | 1.42E+08 |
| ENSSSCG00000033323 | 14 | 92294642 | 92382213 | Loin muscle area QTL (12114) | 14 | 76280940 | 1.42E+08 |
| ENSSSCG00000036083 | 14 | 1.03E+08 | 1.03E+08 | pH 24 hr post-mortem (loin) QTL (228) | 14 | 76280940 | 1.12E+08 |
| ENSSSCG00000036083 | 14 | 1.03E+08 | 1.03E+08 | Meat color-b QTL (239) | 14 | 76280940 | 1.12E+08 |
| ENSSSCG00000036083 | 14 | 1.03E+08 | 1.03E+08 | Percentage type IIb fibers QTL (7044) | 14 | 76280940 | 1.12E+08 |
| ENSSSCG00000036083 | 14 | 1.03E+08 | 1.03E+08 | Backfat at last rib QTL (74) | 14 | 77323446 | 1.03E+08 |
| ENSSSCG00000036083 | 14 | 1.03E+08 | 1.03E+08 | Carcass weight (cold) QTL (75) | 14 | 81099352 | 1.04E+08 |
| ENSSSCG00000036083 | 14 | 1.03E+08 | 1.03E+08 | Light butt ham weight QTL (3080) | 14 | 88231620 | 1.12E+08 |
| ENSSSCG00000036083 | 14 | 1.03E+08 | 1.03E+08 | Meat color density QTL (3005) | 14 | 91664262 | 1.12E+08 |
| ENSSSCG00000036083 | 14 | 1.03E+08 | 1.03E+08 | Meat color L* QTL (3006) | 14 | 91664262 | 1.12E+08 |
| ENSSSCG00000036083 | 14 | 1.03E+08 | 1.03E+08 | Meat color score QTL (3007) | 14 | 91664262 | 1.12E+08 |
| ENSSSCG00000036083 | 14 | 1.03E+08 | 1.03E+08 | Drip loss QTL (3008) | 14 | 91664262 | 1.12E+08 |
| ENSSSCG00000036083 | 14 | 1.03E+08 | 1.03E+08 | subjective pork flavor in lean QTL (4243) | 14 | 96876793 | 1.12E+08 |
| ENSSSCG00000036083 | 14 | 1.03E+08 | 1.03E+08 | Meat color L* QTL (38110) | 14 | 9407982 | 1.38E+08 |
| ENSSSCG00000036083 | 14 | 1.03E+08 | 1.03E+08 | Arachidonic acid content QTL (156959) | 14 | 9407982 | 1.38E+08 |
| ENSSSCG00000036083 | 14 | 1.03E+08 | 1.03E+08 | Shear force at first peak QTL (3929) | 14 | 10933601 | 1.38E+08 |
| ENSSSCG00000036083 | 14 | 1.03E+08 | 1.03E+08 | Shear force at first peak QTL (3930) | 14 | 10933601 | 1.38E+08 |
| ENSSSCG00000036083 | 14 | 1.03E+08 | 1.03E+08 | Shear force at first peak QTL (3931) | 14 | 10933601 | 1.38E+08 |
| ENSSSCG00000036083 | 14 | 1.03E+08 | 1.03E+08 | Shear force QTL (3925) | 14 | 10933601 | 1.12E+08 |
| ENSSSCG00000036083 | 14 | 1.03E+08 | 1.03E+08 | Capric acid content QTL (156957) | 14 | 24537035 | 1.38E+08 |
| ENSSSCG00000036083 | 14 | 1.03E+08 | 1.03E+08 | Margaric acid content QTL (156958) | 14 | 24537035 | 1.38E+08 |
| ENSSSCG00000036083 | 14 | 1.03E+08 | 1.03E+08 | pH 24 hr post-mortem (loin) QTL (21861) | 14 | 58863458 | 1.38E+08 |
| ENSSSCG00000036083 | 14 | 1.03E+08 | 1.03E+08 | Saturated fatty acid content QTL (156906) | 14 | 64838799 | 1.38E+08 |
| ENSSSCG00000036083 | 14 | 1.03E+08 | 1.03E+08 | Unsaturated fatty acid content QTL (156907) | 14 | 64838799 | 1.38E+08 |
| ENSSSCG00000036083 | 14 | 1.03E+08 | 1.03E+08 | Toll-like receptor 9 level QTL (17626) | 14 | 76280940 | 1.42E+08 |
| ENSSSCG00000036083 | 14 | 1.03E+08 | 1.03E+08 | Meat color OPTO QTL (12076) | 14 | 76280940 | 1.42E+08 |
| ENSSSCG00000036083 | 14 | 1.03E+08 | 1.03E+08 | Thawing loss QTL (12077) | 14 | 76280940 | 1.42E+08 |
| ENSSSCG00000036083 | 14 | 1.03E+08 | 1.03E+08 | Shear force QTL (12079) | 14 | 76280940 | 1.42E+08 |
| ENSSSCG00000036083 | 14 | 1.03E+08 | 1.03E+08 | Shear force QTL (12080) | 14 | 76280940 | 1.42E+08 |
| ENSSSCG00000036083 | 14 | 1.03E+08 | 1.03E+08 | Estimated carcass lean content QTL (12081) | 14 | 76280940 | 1.42E+08 |
| ENSSSCG00000036083 | 14 | 1.03E+08 | 1.03E+08 | Loin muscle area QTL (12082) | 14 | 76280940 | 1.42E+08 |
| ENSSSCG00000036083 | 14 | 1.03E+08 | 1.03E+08 | pH 24 hr post mortem (ham) QTL (12110) | 14 | 76280940 | 1.42E+08 |
| ENSSSCG00000036083 | 14 | 1.03E+08 | 1.03E+08 | Estimated carcass lean content QTL (12111) | 14 | 76280940 | 1.42E+08 |
| ENSSSCG00000036083 | 14 | 1.03E+08 | 1.03E+08 | Loin muscle depth QTL (12112) | 14 | 76280940 | 1.42E+08 |
| ENSSSCG00000036083 | 14 | 1.03E+08 | 1.03E+08 | Loin muscle area QTL (12113) | 14 | 76280940 | 1.42E+08 |
| ENSSSCG00000036083 | 14 | 1.03E+08 | 1.03E+08 | Loin muscle area QTL (12114) | 14 | 76280940 | 1.42E+08 |
| ENSSSCG00000036083 | 14 | 1.03E+08 | 1.03E+08 | Lactate level QTL (6358) | 14 | 96876793 | 1.38E+08 |
| ENSSSCG00000010485 | 14 | 1.06E+08 | 1.06E+08 | pH 24 hr post-mortem (loin) QTL (36111) | 14 | 1.06E+08 | 1.06E+08 |
| ENSSSCG00000010485 | 14 | 1.06E+08 | 1.06E+08 | pH 24 hr post-mortem (loin) QTL (228) | 14 | 76280940 | 1.12E+08 |
| ENSSSCG00000010485 | 14 | 1.06E+08 | 1.06E+08 | Meat color-b QTL (239) | 14 | 76280940 | 1.12E+08 |
| ENSSSCG00000010485 | 14 | 1.06E+08 | 1.06E+08 | Percentage type IIb fibers QTL (7044) | 14 | 76280940 | 1.12E+08 |
| ENSSSCG00000010485 | 14 | 1.06E+08 | 1.06E+08 | Light butt ham weight QTL (3080) | 14 | 88231620 | 1.12E+08 |
| ENSSSCG00000010485 | 14 | 1.06E+08 | 1.06E+08 | Meat color density QTL (3005) | 14 | 91664262 | 1.12E+08 |
| ENSSSCG00000010485 | 14 | 1.06E+08 | 1.06E+08 | Meat color L* QTL (3006) | 14 | 91664262 | 1.12E+08 |
| ENSSSCG00000010485 | 14 | 1.06E+08 | 1.06E+08 | Meat color score QTL (3007) | 14 | 91664262 | 1.12E+08 |
| ENSSSCG00000010485 | 14 | 1.06E+08 | 1.06E+08 | Drip loss QTL (3008) | 14 | 91664262 | 1.12E+08 |
| ENSSSCG00000010485 | 14 | 1.06E+08 | 1.06E+08 | subjective pork flavor in lean QTL (4243) | 14 | 96876793 | 1.12E+08 |
| ENSSSCG00000010485 | 14 | 1.06E+08 | 1.06E+08 | Meat color L* QTL (38110) | 14 | 9407982 | 1.38E+08 |
| ENSSSCG00000010485 | 14 | 1.06E+08 | 1.06E+08 | Arachidonic acid content QTL (156959) | 14 | 9407982 | 1.38E+08 |
| ENSSSCG00000010485 | 14 | 1.06E+08 | 1.06E+08 | Shear force at first peak QTL (3929) | 14 | 10933601 | 1.38E+08 |
| ENSSSCG00000010485 | 14 | 1.06E+08 | 1.06E+08 | Shear force at first peak QTL (3930) | 14 | 10933601 | 1.38E+08 |
| ENSSSCG00000010485 | 14 | 1.06E+08 | 1.06E+08 | Shear force at first peak QTL (3931) | 14 | 10933601 | 1.38E+08 |
| ENSSSCG00000010485 | 14 | 1.06E+08 | 1.06E+08 | Shear force QTL (3925) | 14 | 10933601 | 1.12E+08 |
| ENSSSCG00000010485 | 14 | 1.06E+08 | 1.06E+08 | Capric acid content QTL (156957) | 14 | 24537035 | 1.38E+08 |
| ENSSSCG00000010485 | 14 | 1.06E+08 | 1.06E+08 | Margaric acid content QTL (156958) | 14 | 24537035 | 1.38E+08 |
| ENSSSCG00000010485 | 14 | 1.06E+08 | 1.06E+08 | pH 24 hr post-mortem (loin) QTL (21861) | 14 | 58863458 | 1.38E+08 |
| ENSSSCG00000010485 | 14 | 1.06E+08 | 1.06E+08 | Saturated fatty acid content QTL (156906) | 14 | 64838799 | 1.38E+08 |
| ENSSSCG00000010485 | 14 | 1.06E+08 | 1.06E+08 | Unsaturated fatty acid content QTL (156907) | 14 | 64838799 | 1.38E+08 |
| ENSSSCG00000010485 | 14 | 1.06E+08 | 1.06E+08 | Toll-like receptor 9 level QTL (17626) | 14 | 76280940 | 1.42E+08 |
| ENSSSCG00000010485 | 14 | 1.06E+08 | 1.06E+08 | Meat color OPTO QTL (12076) | 14 | 76280940 | 1.42E+08 |
| ENSSSCG00000010485 | 14 | 1.06E+08 | 1.06E+08 | Thawing loss QTL (12077) | 14 | 76280940 | 1.42E+08 |
| ENSSSCG00000010485 | 14 | 1.06E+08 | 1.06E+08 | Shear force QTL (12079) | 14 | 76280940 | 1.42E+08 |
| ENSSSCG00000010485 | 14 | 1.06E+08 | 1.06E+08 | Shear force QTL (12080) | 14 | 76280940 | 1.42E+08 |
| ENSSSCG00000010485 | 14 | 1.06E+08 | 1.06E+08 | Estimated carcass lean content QTL (12081) | 14 | 76280940 | 1.42E+08 |
| ENSSSCG00000010485 | 14 | 1.06E+08 | 1.06E+08 | Loin muscle area QTL (12082) | 14 | 76280940 | 1.42E+08 |
| ENSSSCG00000010485 | 14 | 1.06E+08 | 1.06E+08 | pH 24 hr post mortem (ham) QTL (12110) | 14 | 76280940 | 1.42E+08 |
| ENSSSCG00000010485 | 14 | 1.06E+08 | 1.06E+08 | Estimated carcass lean content QTL (12111) | 14 | 76280940 | 1.42E+08 |
| ENSSSCG00000010485 | 14 | 1.06E+08 | 1.06E+08 | Loin muscle depth QTL (12112) | 14 | 76280940 | 1.42E+08 |
| ENSSSCG00000010485 | 14 | 1.06E+08 | 1.06E+08 | Loin muscle area QTL (12113) | 14 | 76280940 | 1.42E+08 |
| ENSSSCG00000010485 | 14 | 1.06E+08 | 1.06E+08 | Loin muscle area QTL (12114) | 14 | 76280940 | 1.42E+08 |
| ENSSSCG00000010485 | 14 | 1.06E+08 | 1.06E+08 | Lactate level QTL (6358) | 14 | 96876793 | 1.38E+08 |
| ENSSSCG00000010508 | 14 | 1.08E+08 | 1.08E+08 | Stearic acid content QTL (133468) | 14 | 1.08E+08 | 1.08E+08 |
| ENSSSCG00000010508 | 14 | 1.08E+08 | 1.08E+08 | Palmitic acid content QTL (132098) | 14 | 1.08E+08 | 1.08E+08 |
| ENSSSCG00000010508 | 14 | 1.08E+08 | 1.08E+08 | Saturated fatty acid content QTL (135261) | 14 | 1.08E+08 | 1.08E+08 |
| ENSSSCG00000010508 | 14 | 1.08E+08 | 1.08E+08 | Palmitoleic acid content QTL (135937) | 14 | 1.08E+08 | 1.08E+08 |
| ENSSSCG00000010508 | 14 | 1.08E+08 | 1.08E+08 | Oleic acid content QTL (136009) | 14 | 1.08E+08 | 1.08E+08 |
| ENSSSCG00000010508 | 14 | 1.08E+08 | 1.08E+08 | Monounsaturated fatty acid content QTL (136103) | 14 | 1.08E+08 | 1.08E+08 |
| ENSSSCG00000010508 | 14 | 1.08E+08 | 1.08E+08 | Oleic acid content QTL (135986) | 14 | 1.08E+08 | 1.08E+08 |
| ENSSSCG00000010508 | 14 | 1.08E+08 | 1.08E+08 | Monounsaturated fatty acid content QTL (136086) | 14 | 1.08E+08 | 1.08E+08 |
| ENSSSCG00000010508 | 14 | 1.08E+08 | 1.08E+08 | Saturated fatty acid content QTL (135231) | 14 | 1.08E+08 | 1.08E+08 |
| ENSSSCG00000010508 | 14 | 1.08E+08 | 1.08E+08 | Palmitic acid content QTL (132068) | 14 | 1.08E+08 | 1.08E+08 |
| ENSSSCG00000010508 | 14 | 1.08E+08 | 1.08E+08 | Stearic acid content QTL (133417) | 14 | 1.08E+08 | 1.08E+08 |
| ENSSSCG00000010508 | 14 | 1.08E+08 | 1.08E+08 | Palmitoleic acid content QTL (132781) | 14 | 1.08E+08 | 1.08E+08 |
| ENSSSCG00000010508 | 14 | 1.08E+08 | 1.08E+08 | Palmitoleic acid content QTL (132782) | 14 | 1.08E+08 | 1.08E+08 |
| ENSSSCG00000010508 | 14 | 1.08E+08 | 1.08E+08 | Stearic acid content QTL (133418) | 14 | 1.08E+08 | 1.08E+08 |
| ENSSSCG00000010508 | 14 | 1.08E+08 | 1.08E+08 | Palmitic acid content QTL (132069) | 14 | 1.08E+08 | 1.08E+08 |
| ENSSSCG00000010508 | 14 | 1.08E+08 | 1.08E+08 | Saturated fatty acid content QTL (135232) | 14 | 1.08E+08 | 1.08E+08 |
| ENSSSCG00000010508 | 14 | 1.08E+08 | 1.08E+08 | Oleic acid content QTL (136013) | 14 | 1.08E+08 | 1.08E+08 |
| ENSSSCG00000010508 | 14 | 1.08E+08 | 1.08E+08 | Monounsaturated fatty acid content QTL (136107) | 14 | 1.08E+08 | 1.08E+08 |
| ENSSSCG00000010508 | 14 | 1.08E+08 | 1.08E+08 | pH 24 hr post-mortem (loin) QTL (228) | 14 | 76280940 | 1.12E+08 |
| ENSSSCG00000010508 | 14 | 1.08E+08 | 1.08E+08 | Meat color-b QTL (239) | 14 | 76280940 | 1.12E+08 |
| ENSSSCG00000010508 | 14 | 1.08E+08 | 1.08E+08 | Percentage type IIb fibers QTL (7044) | 14 | 76280940 | 1.12E+08 |
| ENSSSCG00000010508 | 14 | 1.08E+08 | 1.08E+08 | Light butt ham weight QTL (3080) | 14 | 88231620 | 1.12E+08 |
| ENSSSCG00000010508 | 14 | 1.08E+08 | 1.08E+08 | Meat color density QTL (3005) | 14 | 91664262 | 1.12E+08 |
| ENSSSCG00000010508 | 14 | 1.08E+08 | 1.08E+08 | Meat color L* QTL (3006) | 14 | 91664262 | 1.12E+08 |
| ENSSSCG00000010508 | 14 | 1.08E+08 | 1.08E+08 | Meat color score QTL (3007) | 14 | 91664262 | 1.12E+08 |
| ENSSSCG00000010508 | 14 | 1.08E+08 | 1.08E+08 | Drip loss QTL (3008) | 14 | 91664262 | 1.12E+08 |
| ENSSSCG00000010508 | 14 | 1.08E+08 | 1.08E+08 | subjective pork flavor in lean QTL (4243) | 14 | 96876793 | 1.12E+08 |
| ENSSSCG00000010508 | 14 | 1.08E+08 | 1.08E+08 | Meat color L* QTL (38110) | 14 | 9407982 | 1.38E+08 |
| ENSSSCG00000010508 | 14 | 1.08E+08 | 1.08E+08 | Arachidonic acid content QTL (156959) | 14 | 9407982 | 1.38E+08 |
| ENSSSCG00000010508 | 14 | 1.08E+08 | 1.08E+08 | Shear force at first peak QTL (3929) | 14 | 10933601 | 1.38E+08 |
| ENSSSCG00000010508 | 14 | 1.08E+08 | 1.08E+08 | Shear force at first peak QTL (3930) | 14 | 10933601 | 1.38E+08 |
| ENSSSCG00000010508 | 14 | 1.08E+08 | 1.08E+08 | Shear force at first peak QTL (3931) | 14 | 10933601 | 1.38E+08 |
| ENSSSCG00000010508 | 14 | 1.08E+08 | 1.08E+08 | Shear force QTL (3925) | 14 | 10933601 | 1.12E+08 |
| ENSSSCG00000010508 | 14 | 1.08E+08 | 1.08E+08 | Capric acid content QTL (156957) | 14 | 24537035 | 1.38E+08 |
| ENSSSCG00000010508 | 14 | 1.08E+08 | 1.08E+08 | Margaric acid content QTL (156958) | 14 | 24537035 | 1.38E+08 |
| ENSSSCG00000010508 | 14 | 1.08E+08 | 1.08E+08 | pH 24 hr post-mortem (loin) QTL (21861) | 14 | 58863458 | 1.38E+08 |
| ENSSSCG00000010508 | 14 | 1.08E+08 | 1.08E+08 | Saturated fatty acid content QTL (156906) | 14 | 64838799 | 1.38E+08 |
| ENSSSCG00000010508 | 14 | 1.08E+08 | 1.08E+08 | Unsaturated fatty acid content QTL (156907) | 14 | 64838799 | 1.38E+08 |
| ENSSSCG00000010508 | 14 | 1.08E+08 | 1.08E+08 | Toll-like receptor 9 level QTL (17626) | 14 | 76280940 | 1.42E+08 |
| ENSSSCG00000010508 | 14 | 1.08E+08 | 1.08E+08 | Meat color OPTO QTL (12076) | 14 | 76280940 | 1.42E+08 |
| ENSSSCG00000010508 | 14 | 1.08E+08 | 1.08E+08 | Thawing loss QTL (12077) | 14 | 76280940 | 1.42E+08 |
| ENSSSCG00000010508 | 14 | 1.08E+08 | 1.08E+08 | Shear force QTL (12079) | 14 | 76280940 | 1.42E+08 |
| ENSSSCG00000010508 | 14 | 1.08E+08 | 1.08E+08 | Shear force QTL (12080) | 14 | 76280940 | 1.42E+08 |
| ENSSSCG00000010508 | 14 | 1.08E+08 | 1.08E+08 | Estimated carcass lean content QTL (12081) | 14 | 76280940 | 1.42E+08 |
| ENSSSCG00000010508 | 14 | 1.08E+08 | 1.08E+08 | Loin muscle area QTL (12082) | 14 | 76280940 | 1.42E+08 |
| ENSSSCG00000010508 | 14 | 1.08E+08 | 1.08E+08 | pH 24 hr post mortem (ham) QTL (12110) | 14 | 76280940 | 1.42E+08 |
| ENSSSCG00000010508 | 14 | 1.08E+08 | 1.08E+08 | Estimated carcass lean content QTL (12111) | 14 | 76280940 | 1.42E+08 |
| ENSSSCG00000010508 | 14 | 1.08E+08 | 1.08E+08 | Loin muscle depth QTL (12112) | 14 | 76280940 | 1.42E+08 |
| ENSSSCG00000010508 | 14 | 1.08E+08 | 1.08E+08 | Loin muscle area QTL (12113) | 14 | 76280940 | 1.42E+08 |
| ENSSSCG00000010508 | 14 | 1.08E+08 | 1.08E+08 | Loin muscle area QTL (12114) | 14 | 76280940 | 1.42E+08 |
| ENSSSCG00000010508 | 14 | 1.08E+08 | 1.08E+08 | Lactate level QTL (6358) | 14 | 96876793 | 1.38E+08 |
| ENSSSCG00000010579 | 14 | 1.13E+08 | 1.13E+08 | Palmitic acid content QTL (131962) | 14 | 1.13E+08 | 1.13E+08 |
| ENSSSCG00000010579 | 14 | 1.13E+08 | 1.13E+08 | Saturated fatty acid content QTL (135145) | 14 | 1.13E+08 | 1.13E+08 |
| ENSSSCG00000010579 | 14 | 1.13E+08 | 1.13E+08 | Monounsaturated fatty acid content QTL (134560) | 14 | 1.13E+08 | 1.13E+08 |
| ENSSSCG00000010579 | 14 | 1.13E+08 | 1.13E+08 | Stearic acid content QTL (133294) | 14 | 1.13E+08 | 1.13E+08 |
| ENSSSCG00000010579 | 14 | 1.13E+08 | 1.13E+08 | Palmitoleic acid content QTL (132622) | 14 | 1.13E+08 | 1.13E+08 |
| ENSSSCG00000010579 | 14 | 1.13E+08 | 1.13E+08 | Oleic acid content QTL (133996) | 14 | 1.13E+08 | 1.13E+08 |
| ENSSSCG00000010579 | 14 | 1.13E+08 | 1.13E+08 | Oleic acid content QTL (134044) | 14 | 1.13E+08 | 1.13E+08 |
| ENSSSCG00000010579 | 14 | 1.13E+08 | 1.13E+08 | Stearic acid content QTL (133322) | 14 | 1.13E+08 | 1.13E+08 |
| ENSSSCG00000010579 | 14 | 1.13E+08 | 1.13E+08 | Palmitoleic acid content QTL (132648) | 14 | 1.13E+08 | 1.13E+08 |
| ENSSSCG00000010579 | 14 | 1.13E+08 | 1.13E+08 | Palmitic acid content QTL (132010) | 14 | 1.13E+08 | 1.13E+08 |
| ENSSSCG00000010579 | 14 | 1.13E+08 | 1.13E+08 | Monounsaturated fatty acid content QTL (134607) | 14 | 1.13E+08 | 1.13E+08 |
| ENSSSCG00000010579 | 14 | 1.13E+08 | 1.13E+08 | Saturated fatty acid content QTL (135170) | 14 | 1.13E+08 | 1.13E+08 |
| ENSSSCG00000010579 | 14 | 1.13E+08 | 1.13E+08 | Saturated fatty acid content QTL (135171) | 14 | 1.13E+08 | 1.13E+08 |
| ENSSSCG00000010579 | 14 | 1.13E+08 | 1.13E+08 | Monounsaturated fatty acid content QTL (134608) | 14 | 1.13E+08 | 1.13E+08 |
| ENSSSCG00000010579 | 14 | 1.13E+08 | 1.13E+08 | Palmitoleic acid content QTL (132649) | 14 | 1.13E+08 | 1.13E+08 |
| ENSSSCG00000010579 | 14 | 1.13E+08 | 1.13E+08 | Oleic acid content QTL (134045) | 14 | 1.13E+08 | 1.13E+08 |
| ENSSSCG00000010579 | 14 | 1.13E+08 | 1.13E+08 | Stearic acid content QTL (133323) | 14 | 1.13E+08 | 1.13E+08 |
| ENSSSCG00000010579 | 14 | 1.13E+08 | 1.13E+08 | Palmitic acid content QTL (132011) | 14 | 1.13E+08 | 1.13E+08 |
| ENSSSCG00000010579 | 14 | 1.13E+08 | 1.13E+08 | Palmitic acid content QTL (131963) | 14 | 1.13E+08 | 1.13E+08 |
| ENSSSCG00000010579 | 14 | 1.13E+08 | 1.13E+08 | Monounsaturated fatty acid content QTL (134561) | 14 | 1.13E+08 | 1.13E+08 |
| ENSSSCG00000010579 | 14 | 1.13E+08 | 1.13E+08 | Saturated fatty acid content QTL (135146) | 14 | 1.13E+08 | 1.13E+08 |
| ENSSSCG00000010579 | 14 | 1.13E+08 | 1.13E+08 | Stearic acid content QTL (133295) | 14 | 1.13E+08 | 1.13E+08 |
| ENSSSCG00000010579 | 14 | 1.13E+08 | 1.13E+08 | Palmitoleic acid content QTL (132623) | 14 | 1.13E+08 | 1.13E+08 |
| ENSSSCG00000010579 | 14 | 1.13E+08 | 1.13E+08 | Oleic acid content QTL (133997) | 14 | 1.13E+08 | 1.13E+08 |
| ENSSSCG00000010579 | 14 | 1.13E+08 | 1.13E+08 | Oleic acid content QTL (134046) | 14 | 1.13E+08 | 1.13E+08 |
| ENSSSCG00000010579 | 14 | 1.13E+08 | 1.13E+08 | Stearic acid content QTL (133324) | 14 | 1.13E+08 | 1.13E+08 |
| ENSSSCG00000010579 | 14 | 1.13E+08 | 1.13E+08 | Palmitoleic acid content QTL (132650) | 14 | 1.13E+08 | 1.13E+08 |
| ENSSSCG00000010579 | 14 | 1.13E+08 | 1.13E+08 | Palmitic acid content QTL (132012) | 14 | 1.13E+08 | 1.13E+08 |
| ENSSSCG00000010579 | 14 | 1.13E+08 | 1.13E+08 | Saturated fatty acid content QTL (135172) | 14 | 1.13E+08 | 1.13E+08 |
| ENSSSCG00000010579 | 14 | 1.13E+08 | 1.13E+08 | Monounsaturated fatty acid content QTL (134609) | 14 | 1.13E+08 | 1.13E+08 |
| ENSSSCG00000010579 | 14 | 1.13E+08 | 1.13E+08 | Monounsaturated fatty acid content QTL (134413) | 14 | 1.13E+08 | 1.13E+08 |
| ENSSSCG00000010579 | 14 | 1.13E+08 | 1.13E+08 | Saturated fatty acid content QTL (135000) | 14 | 1.13E+08 | 1.13E+08 |
| ENSSSCG00000010579 | 14 | 1.13E+08 | 1.13E+08 | Palmitoleic acid content QTL (132426) | 14 | 1.13E+08 | 1.13E+08 |
| ENSSSCG00000010579 | 14 | 1.13E+08 | 1.13E+08 | Oleic acid content QTL (133852) | 14 | 1.13E+08 | 1.13E+08 |
| ENSSSCG00000010579 | 14 | 1.13E+08 | 1.13E+08 | Stearic acid content QTL (133132) | 14 | 1.13E+08 | 1.13E+08 |
| ENSSSCG00000010579 | 14 | 1.13E+08 | 1.13E+08 | Palmitic acid content QTL (131853) | 14 | 1.13E+08 | 1.13E+08 |
| ENSSSCG00000010579 | 14 | 1.13E+08 | 1.13E+08 | Palmitic acid content QTL (131860) | 14 | 1.13E+08 | 1.13E+08 |
| ENSSSCG00000010579 | 14 | 1.13E+08 | 1.13E+08 | Saturated fatty acid content QTL (135140) | 14 | 1.13E+08 | 1.13E+08 |
| ENSSSCG00000010579 | 14 | 1.13E+08 | 1.13E+08 | Monounsaturated fatty acid content QTL (134557) | 14 | 1.13E+08 | 1.13E+08 |
| ENSSSCG00000010579 | 14 | 1.13E+08 | 1.13E+08 | Oleic acid content QTL (133994) | 14 | 1.13E+08 | 1.13E+08 |
| ENSSSCG00000010579 | 14 | 1.13E+08 | 1.13E+08 | Palmitoleic acid content QTL (132620) | 14 | 1.13E+08 | 1.13E+08 |
| ENSSSCG00000010579 | 14 | 1.13E+08 | 1.13E+08 | Stearic acid content QTL (133290) | 14 | 1.13E+08 | 1.13E+08 |
| ENSSSCG00000010579 | 14 | 1.13E+08 | 1.13E+08 | Stearic acid content QTL (133296) | 14 | 1.13E+08 | 1.13E+08 |
| ENSSSCG00000010579 | 14 | 1.13E+08 | 1.13E+08 | Oleic acid content QTL (133998) | 14 | 1.13E+08 | 1.13E+08 |
| ENSSSCG00000010579 | 14 | 1.13E+08 | 1.13E+08 | Palmitoleic acid content QTL (132624) | 14 | 1.13E+08 | 1.13E+08 |
| ENSSSCG00000010579 | 14 | 1.13E+08 | 1.13E+08 | Palmitic acid content QTL (131964) | 14 | 1.13E+08 | 1.13E+08 |
| ENSSSCG00000010579 | 14 | 1.13E+08 | 1.13E+08 | Monounsaturated fatty acid content QTL (134562) | 14 | 1.13E+08 | 1.13E+08 |
| ENSSSCG00000010579 | 14 | 1.13E+08 | 1.13E+08 | Saturated fatty acid content QTL (135147) | 14 | 1.13E+08 | 1.13E+08 |
| ENSSSCG00000010579 | 14 | 1.13E+08 | 1.13E+08 | Saturated fatty acid content QTL (135173) | 14 | 1.13E+08 | 1.13E+08 |
| ENSSSCG00000010579 | 14 | 1.13E+08 | 1.13E+08 | Monounsaturated fatty acid content QTL (134610) | 14 | 1.13E+08 | 1.13E+08 |
| ENSSSCG00000010579 | 14 | 1.13E+08 | 1.13E+08 | Palmitoleic acid content QTL (132651) | 14 | 1.13E+08 | 1.13E+08 |
| ENSSSCG00000010579 | 14 | 1.13E+08 | 1.13E+08 | Stearic acid content QTL (133325) | 14 | 1.13E+08 | 1.13E+08 |
| ENSSSCG00000010579 | 14 | 1.13E+08 | 1.13E+08 | Oleic acid content QTL (134047) | 14 | 1.13E+08 | 1.13E+08 |
| ENSSSCG00000010579 | 14 | 1.13E+08 | 1.13E+08 | Palmitic acid content QTL (132013) | 14 | 1.13E+08 | 1.13E+08 |
| ENSSSCG00000010579 | 14 | 1.13E+08 | 1.13E+08 | Palmitic acid content QTL (131965) | 14 | 1.13E+08 | 1.13E+08 |
| ENSSSCG00000010579 | 14 | 1.13E+08 | 1.13E+08 | Monounsaturated fatty acid content QTL (134563) | 14 | 1.13E+08 | 1.13E+08 |
| ENSSSCG00000010579 | 14 | 1.13E+08 | 1.13E+08 | Saturated fatty acid content QTL (135148) | 14 | 1.13E+08 | 1.13E+08 |
| ENSSSCG00000010579 | 14 | 1.13E+08 | 1.13E+08 | Oleic acid content QTL (133999) | 14 | 1.13E+08 | 1.13E+08 |
| ENSSSCG00000010579 | 14 | 1.13E+08 | 1.13E+08 | Palmitoleic acid content QTL (132625) | 14 | 1.13E+08 | 1.13E+08 |
| ENSSSCG00000010579 | 14 | 1.13E+08 | 1.13E+08 | Stearic acid content QTL (133297) | 14 | 1.13E+08 | 1.13E+08 |
| ENSSSCG00000010579 | 14 | 1.13E+08 | 1.13E+08 | Oleic acid content QTL (133853) | 14 | 1.13E+08 | 1.13E+08 |
| ENSSSCG00000010579 | 14 | 1.13E+08 | 1.13E+08 | Stearic acid content QTL (133133) | 14 | 1.13E+08 | 1.13E+08 |
| ENSSSCG00000010579 | 14 | 1.13E+08 | 1.13E+08 | Palmitoleic acid content QTL (132427) | 14 | 1.13E+08 | 1.13E+08 |
| ENSSSCG00000010579 | 14 | 1.13E+08 | 1.13E+08 | Palmitic acid content QTL (131854) | 14 | 1.13E+08 | 1.13E+08 |
| ENSSSCG00000010579 | 14 | 1.13E+08 | 1.13E+08 | Saturated fatty acid content QTL (135001) | 14 | 1.13E+08 | 1.13E+08 |
| ENSSSCG00000010579 | 14 | 1.13E+08 | 1.13E+08 | Monounsaturated fatty acid content QTL (134414) | 14 | 1.13E+08 | 1.13E+08 |
| ENSSSCG00000010579 | 14 | 1.13E+08 | 1.13E+08 | Monounsaturated fatty acid content QTL (134415) | 14 | 1.13E+08 | 1.13E+08 |
| ENSSSCG00000010579 | 14 | 1.13E+08 | 1.13E+08 | Saturated fatty acid content QTL (135002) | 14 | 1.13E+08 | 1.13E+08 |
| ENSSSCG00000010579 | 14 | 1.13E+08 | 1.13E+08 | Palmitoleic acid content QTL (132428) | 14 | 1.13E+08 | 1.13E+08 |
| ENSSSCG00000010579 | 14 | 1.13E+08 | 1.13E+08 | Oleic acid content QTL (133854) | 14 | 1.13E+08 | 1.13E+08 |
| ENSSSCG00000010579 | 14 | 1.13E+08 | 1.13E+08 | Stearic acid content QTL (133134) | 14 | 1.13E+08 | 1.13E+08 |
| ENSSSCG00000010579 | 14 | 1.13E+08 | 1.13E+08 | Palmitic acid content QTL (131855) | 14 | 1.13E+08 | 1.13E+08 |
| ENSSSCG00000010579 | 14 | 1.13E+08 | 1.13E+08 | Palmitic acid content QTL (131765) | 14 | 1.13E+08 | 1.13E+08 |
| ENSSSCG00000010579 | 14 | 1.13E+08 | 1.13E+08 | Palmitoleic acid content QTL (132269) | 14 | 1.13E+08 | 1.13E+08 |
| ENSSSCG00000010579 | 14 | 1.13E+08 | 1.13E+08 | Saturated fatty acid content QTL (134962) | 14 | 1.13E+08 | 1.13E+08 |
| ENSSSCG00000010579 | 14 | 1.13E+08 | 1.13E+08 | Stearic acid content QTL (133089) | 14 | 1.13E+08 | 1.13E+08 |
| ENSSSCG00000010579 | 14 | 1.13E+08 | 1.13E+08 | Monounsaturated fatty acid content QTL (134326) | 14 | 1.13E+08 | 1.13E+08 |
| ENSSSCG00000010579 | 14 | 1.13E+08 | 1.13E+08 | Oleic acid content QTL (133768) | 14 | 1.13E+08 | 1.13E+08 |
| ENSSSCG00000010579 | 14 | 1.13E+08 | 1.13E+08 | Oleic acid content QTL (133769) | 14 | 1.13E+08 | 1.13E+08 |
| ENSSSCG00000010579 | 14 | 1.13E+08 | 1.13E+08 | Stearic acid content QTL (133090) | 14 | 1.13E+08 | 1.13E+08 |
| ENSSSCG00000010579 | 14 | 1.13E+08 | 1.13E+08 | Monounsaturated fatty acid content QTL (134327) | 14 | 1.13E+08 | 1.13E+08 |
| ENSSSCG00000010579 | 14 | 1.13E+08 | 1.13E+08 | Palmitoleic acid content QTL (132270) | 14 | 1.13E+08 | 1.13E+08 |
| ENSSSCG00000010579 | 14 | 1.13E+08 | 1.13E+08 | Palmitic acid content QTL (131766) | 14 | 1.13E+08 | 1.13E+08 |
| ENSSSCG00000010579 | 14 | 1.13E+08 | 1.13E+08 | Saturated fatty acid content QTL (134963) | 14 | 1.13E+08 | 1.13E+08 |
| ENSSSCG00000010579 | 14 | 1.13E+08 | 1.13E+08 | Saturated fatty acid content QTL (134965) | 14 | 1.13E+08 | 1.13E+08 |
| ENSSSCG00000010579 | 14 | 1.13E+08 | 1.13E+08 | Monounsaturated fatty acid content QTL (134329) | 14 | 1.13E+08 | 1.13E+08 |
| ENSSSCG00000010579 | 14 | 1.13E+08 | 1.13E+08 | Oleic acid content QTL (133771) | 14 | 1.13E+08 | 1.13E+08 |
| ENSSSCG00000010579 | 14 | 1.13E+08 | 1.13E+08 | Stearic acid content QTL (133092) | 14 | 1.13E+08 | 1.13E+08 |
| ENSSSCG00000010579 | 14 | 1.13E+08 | 1.13E+08 | Palmitic acid content QTL (131768) | 14 | 1.13E+08 | 1.13E+08 |
| ENSSSCG00000010579 | 14 | 1.13E+08 | 1.13E+08 | Palmitoleic acid content QTL (132272) | 14 | 1.13E+08 | 1.13E+08 |
| ENSSSCG00000010579 | 14 | 1.13E+08 | 1.13E+08 | Palmitoleic acid content QTL (132273) | 14 | 1.13E+08 | 1.13E+08 |
| ENSSSCG00000010579 | 14 | 1.13E+08 | 1.13E+08 | Palmitic acid content QTL (131769) | 14 | 1.13E+08 | 1.13E+08 |
| ENSSSCG00000010579 | 14 | 1.13E+08 | 1.13E+08 | Saturated fatty acid content QTL (134966) | 14 | 1.13E+08 | 1.13E+08 |
| ENSSSCG00000010579 | 14 | 1.13E+08 | 1.13E+08 | Stearic acid content QTL (133093) | 14 | 1.13E+08 | 1.13E+08 |
| ENSSSCG00000010579 | 14 | 1.13E+08 | 1.13E+08 | Monounsaturated fatty acid content QTL (134330) | 14 | 1.13E+08 | 1.13E+08 |
| ENSSSCG00000010579 | 14 | 1.13E+08 | 1.13E+08 | Oleic acid content QTL (133772) | 14 | 1.13E+08 | 1.13E+08 |
| ENSSSCG00000010579 | 14 | 1.13E+08 | 1.13E+08 | Stearic acid content QTL (133136) | 14 | 1.13E+08 | 1.13E+08 |
| ENSSSCG00000010579 | 14 | 1.13E+08 | 1.13E+08 | Palmitoleic acid content QTL (132430) | 14 | 1.13E+08 | 1.13E+08 |
| ENSSSCG00000010579 | 14 | 1.13E+08 | 1.13E+08 | Oleic acid content QTL (133856) | 14 | 1.13E+08 | 1.13E+08 |
| ENSSSCG00000010579 | 14 | 1.13E+08 | 1.13E+08 | Palmitic acid content QTL (131857) | 14 | 1.13E+08 | 1.13E+08 |
| ENSSSCG00000010579 | 14 | 1.13E+08 | 1.13E+08 | Saturated fatty acid content QTL (135004) | 14 | 1.13E+08 | 1.13E+08 |
| ENSSSCG00000010579 | 14 | 1.13E+08 | 1.13E+08 | Monounsaturated fatty acid content QTL (134417) | 14 | 1.13E+08 | 1.13E+08 |
| ENSSSCG00000010579 | 14 | 1.13E+08 | 1.13E+08 | Saturated fatty acid content QTL (134968) | 14 | 1.13E+08 | 1.13E+08 |
| ENSSSCG00000010579 | 14 | 1.13E+08 | 1.13E+08 | Stearic acid content QTL (133095) | 14 | 1.13E+08 | 1.13E+08 |
| ENSSSCG00000010579 | 14 | 1.13E+08 | 1.13E+08 | Oleic acid content QTL (133774) | 14 | 1.13E+08 | 1.13E+08 |
| ENSSSCG00000010579 | 14 | 1.13E+08 | 1.13E+08 | Monounsaturated fatty acid content QTL (134332) | 14 | 1.13E+08 | 1.13E+08 |
| ENSSSCG00000010579 | 14 | 1.13E+08 | 1.13E+08 | Palmitic acid content QTL (131771) | 14 | 1.13E+08 | 1.13E+08 |
| ENSSSCG00000010579 | 14 | 1.13E+08 | 1.13E+08 | Palmitoleic acid content QTL (132275) | 14 | 1.13E+08 | 1.13E+08 |
| ENSSSCG00000010579 | 14 | 1.13E+08 | 1.13E+08 | Palmitic acid content QTL (131858) | 14 | 1.13E+08 | 1.13E+08 |
| ENSSSCG00000010579 | 14 | 1.13E+08 | 1.13E+08 | Monounsaturated fatty acid content QTL (134418) | 14 | 1.13E+08 | 1.13E+08 |
| ENSSSCG00000010579 | 14 | 1.13E+08 | 1.13E+08 | Saturated fatty acid content QTL (135005) | 14 | 1.13E+08 | 1.13E+08 |
| ENSSSCG00000010579 | 14 | 1.13E+08 | 1.13E+08 | Stearic acid content QTL (133137) | 14 | 1.13E+08 | 1.13E+08 |
| ENSSSCG00000010579 | 14 | 1.13E+08 | 1.13E+08 | Oleic acid content QTL (133857) | 14 | 1.13E+08 | 1.13E+08 |
| ENSSSCG00000010579 | 14 | 1.13E+08 | 1.13E+08 | Palmitoleic acid content QTL (132431) | 14 | 1.13E+08 | 1.13E+08 |
| ENSSSCG00000010579 | 14 | 1.13E+08 | 1.13E+08 | Stearic acid content QTL (133445) | 14 | 1.13E+08 | 1.13E+08 |
| ENSSSCG00000010579 | 14 | 1.13E+08 | 1.13E+08 | Palmitic acid content QTL (132119) | 14 | 1.13E+08 | 1.13E+08 |
| ENSSSCG00000010579 | 14 | 1.13E+08 | 1.13E+08 | Saturated fatty acid content QTL (135281) | 14 | 1.13E+08 | 1.13E+08 |
| ENSSSCG00000010579 | 14 | 1.13E+08 | 1.13E+08 | Palmitoleic acid content QTL (135931) | 14 | 1.13E+08 | 1.13E+08 |
| ENSSSCG00000010579 | 14 | 1.13E+08 | 1.13E+08 | Oleic acid content QTL (135989) | 14 | 1.13E+08 | 1.13E+08 |
| ENSSSCG00000010579 | 14 | 1.13E+08 | 1.13E+08 | Monounsaturated fatty acid content QTL (136090) | 14 | 1.13E+08 | 1.13E+08 |
| ENSSSCG00000010579 | 14 | 1.13E+08 | 1.13E+08 | Saturated fatty acid content QTL (135006) | 14 | 1.13E+08 | 1.13E+08 |
| ENSSSCG00000010579 | 14 | 1.13E+08 | 1.13E+08 | Monounsaturated fatty acid content QTL (134419) | 14 | 1.13E+08 | 1.13E+08 |
| ENSSSCG00000010579 | 14 | 1.13E+08 | 1.13E+08 | Palmitoleic acid content QTL (132432) | 14 | 1.13E+08 | 1.13E+08 |
| ENSSSCG00000010579 | 14 | 1.13E+08 | 1.13E+08 | Stearic acid content QTL (133138) | 14 | 1.13E+08 | 1.13E+08 |
| ENSSSCG00000010579 | 14 | 1.13E+08 | 1.13E+08 | Oleic acid content QTL (133858) | 14 | 1.13E+08 | 1.13E+08 |
| ENSSSCG00000010579 | 14 | 1.13E+08 | 1.13E+08 | Palmitic acid content QTL (131859) | 14 | 1.13E+08 | 1.13E+08 |
| ENSSSCG00000010579 | 14 | 1.13E+08 | 1.13E+08 | Palmitoleic acid content QTL (132276) | 14 | 1.13E+08 | 1.13E+08 |
| ENSSSCG00000010579 | 14 | 1.13E+08 | 1.13E+08 | Palmitic acid content QTL (131772) | 14 | 1.13E+08 | 1.13E+08 |
| ENSSSCG00000010579 | 14 | 1.13E+08 | 1.13E+08 | Saturated fatty acid content QTL (134969) | 14 | 1.13E+08 | 1.13E+08 |
| ENSSSCG00000010579 | 14 | 1.13E+08 | 1.13E+08 | Monounsaturated fatty acid content QTL (134333) | 14 | 1.13E+08 | 1.13E+08 |
| ENSSSCG00000010579 | 14 | 1.13E+08 | 1.13E+08 | Stearic acid content QTL (133096) | 14 | 1.13E+08 | 1.13E+08 |
| ENSSSCG00000010579 | 14 | 1.13E+08 | 1.13E+08 | Oleic acid content QTL (133775) | 14 | 1.13E+08 | 1.13E+08 |
| ENSSSCG00000010579 | 14 | 1.13E+08 | 1.13E+08 | Oleic acid content QTL (133776) | 14 | 1.13E+08 | 1.13E+08 |
| ENSSSCG00000010579 | 14 | 1.13E+08 | 1.13E+08 | Stearic acid content QTL (133097) | 14 | 1.13E+08 | 1.13E+08 |
| ENSSSCG00000010579 | 14 | 1.13E+08 | 1.13E+08 | Monounsaturated fatty acid content QTL (134334) | 14 | 1.13E+08 | 1.13E+08 |
| ENSSSCG00000010579 | 14 | 1.13E+08 | 1.13E+08 | Palmitic acid content QTL (131773) | 14 | 1.13E+08 | 1.13E+08 |
| ENSSSCG00000010579 | 14 | 1.13E+08 | 1.13E+08 | Palmitoleic acid content QTL (132277) | 14 | 1.13E+08 | 1.13E+08 |
| ENSSSCG00000010579 | 14 | 1.13E+08 | 1.13E+08 | Saturated fatty acid content QTL (134970) | 14 | 1.13E+08 | 1.13E+08 |
| ENSSSCG00000010579 | 14 | 1.13E+08 | 1.13E+08 | Saturated fatty acid content QTL (134971) | 14 | 1.13E+08 | 1.13E+08 |
| ENSSSCG00000010579 | 14 | 1.13E+08 | 1.13E+08 | Monounsaturated fatty acid content QTL (134335) | 14 | 1.13E+08 | 1.13E+08 |
| ENSSSCG00000010579 | 14 | 1.13E+08 | 1.13E+08 | Stearic acid content QTL (133098) | 14 | 1.13E+08 | 1.13E+08 |
| ENSSSCG00000010579 | 14 | 1.13E+08 | 1.13E+08 | Oleic acid content QTL (133777) | 14 | 1.13E+08 | 1.13E+08 |
| ENSSSCG00000010579 | 14 | 1.13E+08 | 1.13E+08 | Oleic acid to stearic acid ratio QTL (218077) | 14 | 1.13E+08 | 1.13E+08 |
| ENSSSCG00000010579 | 14 | 1.13E+08 | 1.13E+08 | Palmitoleic acid to palmitic acid ratio QTL (101450) | 14 | 1.13E+08 | 1.13E+08 |
| ENSSSCG00000010579 | 14 | 1.13E+08 | 1.13E+08 | Stearic acid to palmitic acid ratio QTL (101484) | 14 | 1.13E+08 | 1.13E+08 |
| ENSSSCG00000010579 | 14 | 1.13E+08 | 1.13E+08 | Oleic acid to stearic acid ratio QTL (101539) | 14 | 1.13E+08 | 1.13E+08 |
| ENSSSCG00000010579 | 14 | 1.13E+08 | 1.13E+08 | Monounsaturated fatty acid to saturated fatty acid ratio QTL (101815) | 14 | 1.13E+08 | 1.13E+08 |
| ENSSSCG00000010579 | 14 | 1.13E+08 | 1.13E+08 | Saturated fatty acid content QTL (101856) | 14 | 1.13E+08 | 1.13E+08 |
| ENSSSCG00000010579 | 14 | 1.13E+08 | 1.13E+08 | Unsaturated to saturated fatty acid ratio QTL (101888) | 14 | 1.13E+08 | 1.13E+08 |
| ENSSSCG00000010579 | 14 | 1.13E+08 | 1.13E+08 | Fatty acid thrombogenic index QTL (101940) | 14 | 1.13E+08 | 1.13E+08 |
| ENSSSCG00000010579 | 14 | 1.13E+08 | 1.13E+08 | Palmitoleic acid content QTL (132278) | 14 | 1.13E+08 | 1.13E+08 |
| ENSSSCG00000010579 | 14 | 1.13E+08 | 1.13E+08 | Palmitic acid content QTL (131774) | 14 | 1.13E+08 | 1.13E+08 |
| ENSSSCG00000010579 | 14 | 1.13E+08 | 1.13E+08 | Oleic acid content QTL (160400) | 14 | 1.13E+08 | 1.13E+08 |
| ENSSSCG00000010579 | 14 | 1.13E+08 | 1.13E+08 | Stearic acid content QTL (160401) | 14 | 1.13E+08 | 1.13E+08 |
| ENSSSCG00000010579 | 14 | 1.13E+08 | 1.13E+08 | Saturated fatty acid content QTL (134973) | 14 | 1.13E+08 | 1.13E+08 |
| ENSSSCG00000010579 | 14 | 1.13E+08 | 1.13E+08 | Oleic acid content QTL (133779) | 14 | 1.13E+08 | 1.13E+08 |
| ENSSSCG00000010579 | 14 | 1.13E+08 | 1.13E+08 | Stearic acid content QTL (133100) | 14 | 1.13E+08 | 1.13E+08 |
| ENSSSCG00000010579 | 14 | 1.13E+08 | 1.13E+08 | Monounsaturated fatty acid content QTL (134337) | 14 | 1.13E+08 | 1.13E+08 |
| ENSSSCG00000010579 | 14 | 1.13E+08 | 1.13E+08 | Palmitic acid content QTL (131776) | 14 | 1.13E+08 | 1.13E+08 |
| ENSSSCG00000010579 | 14 | 1.13E+08 | 1.13E+08 | Palmitoleic acid content QTL (132280) | 14 | 1.13E+08 | 1.13E+08 |
| ENSSSCG00000010579 | 14 | 1.13E+08 | 1.13E+08 | Meat color L* QTL (38110) | 14 | 9407982 | 1.38E+08 |
| ENSSSCG00000010579 | 14 | 1.13E+08 | 1.13E+08 | Arachidonic acid content QTL (156959) | 14 | 9407982 | 1.38E+08 |
| ENSSSCG00000010579 | 14 | 1.13E+08 | 1.13E+08 | Shear force at first peak QTL (3929) | 14 | 10933601 | 1.38E+08 |
| ENSSSCG00000010579 | 14 | 1.13E+08 | 1.13E+08 | Shear force at first peak QTL (3930) | 14 | 10933601 | 1.38E+08 |
| ENSSSCG00000010579 | 14 | 1.13E+08 | 1.13E+08 | Shear force at first peak QTL (3931) | 14 | 10933601 | 1.38E+08 |
| ENSSSCG00000010579 | 14 | 1.13E+08 | 1.13E+08 | Capric acid content QTL (156957) | 14 | 24537035 | 1.38E+08 |
| ENSSSCG00000010579 | 14 | 1.13E+08 | 1.13E+08 | Margaric acid content QTL (156958) | 14 | 24537035 | 1.38E+08 |
| ENSSSCG00000010579 | 14 | 1.13E+08 | 1.13E+08 | pH 24 hr post-mortem (loin) QTL (21861) | 14 | 58863458 | 1.38E+08 |
| ENSSSCG00000010579 | 14 | 1.13E+08 | 1.13E+08 | Saturated fatty acid content QTL (156906) | 14 | 64838799 | 1.38E+08 |
| ENSSSCG00000010579 | 14 | 1.13E+08 | 1.13E+08 | Unsaturated fatty acid content QTL (156907) | 14 | 64838799 | 1.38E+08 |
| ENSSSCG00000010579 | 14 | 1.13E+08 | 1.13E+08 | Toll-like receptor 9 level QTL (17626) | 14 | 76280940 | 1.42E+08 |
| ENSSSCG00000010579 | 14 | 1.13E+08 | 1.13E+08 | Meat color OPTO QTL (12076) | 14 | 76280940 | 1.42E+08 |
| ENSSSCG00000010579 | 14 | 1.13E+08 | 1.13E+08 | Thawing loss QTL (12077) | 14 | 76280940 | 1.42E+08 |
| ENSSSCG00000010579 | 14 | 1.13E+08 | 1.13E+08 | Shear force QTL (12079) | 14 | 76280940 | 1.42E+08 |
| ENSSSCG00000010579 | 14 | 1.13E+08 | 1.13E+08 | Shear force QTL (12080) | 14 | 76280940 | 1.42E+08 |
| ENSSSCG00000010579 | 14 | 1.13E+08 | 1.13E+08 | Estimated carcass lean content QTL (12081) | 14 | 76280940 | 1.42E+08 |
| ENSSSCG00000010579 | 14 | 1.13E+08 | 1.13E+08 | Loin muscle area QTL (12082) | 14 | 76280940 | 1.42E+08 |
| ENSSSCG00000010579 | 14 | 1.13E+08 | 1.13E+08 | pH 24 hr post mortem (ham) QTL (12110) | 14 | 76280940 | 1.42E+08 |
| ENSSSCG00000010579 | 14 | 1.13E+08 | 1.13E+08 | Estimated carcass lean content QTL (12111) | 14 | 76280940 | 1.42E+08 |
| ENSSSCG00000010579 | 14 | 1.13E+08 | 1.13E+08 | Loin muscle depth QTL (12112) | 14 | 76280940 | 1.42E+08 |
| ENSSSCG00000010579 | 14 | 1.13E+08 | 1.13E+08 | Loin muscle area QTL (12113) | 14 | 76280940 | 1.42E+08 |
| ENSSSCG00000010579 | 14 | 1.13E+08 | 1.13E+08 | Loin muscle area QTL (12114) | 14 | 76280940 | 1.42E+08 |
| ENSSSCG00000010579 | 14 | 1.13E+08 | 1.13E+08 | Lactate level QTL (6358) | 14 | 96876793 | 1.38E+08 |
| ENSSSCG00000010579 | 14 | 1.13E+08 | 1.13E+08 | androstenone, sensory panel QTL (4236) | 14 | 1.12E+08 | 1.38E+08 |
| ENSSSCG00000010579 | 14 | 1.13E+08 | 1.13E+08 | subjective boar flavor in lean QTL (4240) | 14 | 1.12E+08 | 1.38E+08 |
| ENSSSCG00000010652 | 14 | 1.25E+08 | 1.25E+08 | Meat color L* QTL (38110) | 14 | 9407982 | 1.38E+08 |
| ENSSSCG00000010652 | 14 | 1.25E+08 | 1.25E+08 | Arachidonic acid content QTL (156959) | 14 | 9407982 | 1.38E+08 |
| ENSSSCG00000010652 | 14 | 1.25E+08 | 1.25E+08 | Shear force at first peak QTL (3929) | 14 | 10933601 | 1.38E+08 |
| ENSSSCG00000010652 | 14 | 1.25E+08 | 1.25E+08 | Shear force at first peak QTL (3930) | 14 | 10933601 | 1.38E+08 |
| ENSSSCG00000010652 | 14 | 1.25E+08 | 1.25E+08 | Shear force at first peak QTL (3931) | 14 | 10933601 | 1.38E+08 |
| ENSSSCG00000010652 | 14 | 1.25E+08 | 1.25E+08 | Capric acid content QTL (156957) | 14 | 24537035 | 1.38E+08 |
| ENSSSCG00000010652 | 14 | 1.25E+08 | 1.25E+08 | Margaric acid content QTL (156958) | 14 | 24537035 | 1.38E+08 |
| ENSSSCG00000010652 | 14 | 1.25E+08 | 1.25E+08 | pH 24 hr post-mortem (loin) QTL (21861) | 14 | 58863458 | 1.38E+08 |
| ENSSSCG00000010652 | 14 | 1.25E+08 | 1.25E+08 | Saturated fatty acid content QTL (156906) | 14 | 64838799 | 1.38E+08 |
| ENSSSCG00000010652 | 14 | 1.25E+08 | 1.25E+08 | Unsaturated fatty acid content QTL (156907) | 14 | 64838799 | 1.38E+08 |
| ENSSSCG00000010652 | 14 | 1.25E+08 | 1.25E+08 | Toll-like receptor 9 level QTL (17626) | 14 | 76280940 | 1.42E+08 |
| ENSSSCG00000010652 | 14 | 1.25E+08 | 1.25E+08 | Meat color OPTO QTL (12076) | 14 | 76280940 | 1.42E+08 |
| ENSSSCG00000010652 | 14 | 1.25E+08 | 1.25E+08 | Thawing loss QTL (12077) | 14 | 76280940 | 1.42E+08 |
| ENSSSCG00000010652 | 14 | 1.25E+08 | 1.25E+08 | Shear force QTL (12079) | 14 | 76280940 | 1.42E+08 |
| ENSSSCG00000010652 | 14 | 1.25E+08 | 1.25E+08 | Shear force QTL (12080) | 14 | 76280940 | 1.42E+08 |
| ENSSSCG00000010652 | 14 | 1.25E+08 | 1.25E+08 | Estimated carcass lean content QTL (12081) | 14 | 76280940 | 1.42E+08 |
| ENSSSCG00000010652 | 14 | 1.25E+08 | 1.25E+08 | Loin muscle area QTL (12082) | 14 | 76280940 | 1.42E+08 |
| ENSSSCG00000010652 | 14 | 1.25E+08 | 1.25E+08 | pH 24 hr post mortem (ham) QTL (12110) | 14 | 76280940 | 1.42E+08 |
| ENSSSCG00000010652 | 14 | 1.25E+08 | 1.25E+08 | Estimated carcass lean content QTL (12111) | 14 | 76280940 | 1.42E+08 |
| ENSSSCG00000010652 | 14 | 1.25E+08 | 1.25E+08 | Loin muscle depth QTL (12112) | 14 | 76280940 | 1.42E+08 |
| ENSSSCG00000010652 | 14 | 1.25E+08 | 1.25E+08 | Loin muscle area QTL (12113) | 14 | 76280940 | 1.42E+08 |
| ENSSSCG00000010652 | 14 | 1.25E+08 | 1.25E+08 | Loin muscle area QTL (12114) | 14 | 76280940 | 1.42E+08 |
| ENSSSCG00000010652 | 14 | 1.25E+08 | 1.25E+08 | Lactate level QTL (6358) | 14 | 96876793 | 1.38E+08 |
| ENSSSCG00000010652 | 14 | 1.25E+08 | 1.25E+08 | androstenone, sensory panel QTL (4236) | 14 | 1.12E+08 | 1.38E+08 |
| ENSSSCG00000010652 | 14 | 1.25E+08 | 1.25E+08 | subjective boar flavor in lean QTL (4240) | 14 | 1.12E+08 | 1.38E+08 |
| ENSSSCG00000010652 | 14 | 1.25E+08 | 1.25E+08 | Number of muscle fibers per unit area QTL (2825) | 14 | 1.16E+08 | 1.64E+08 |
| ENSSSCG00000010652 | 14 | 1.25E+08 | 1.25E+08 | Total muscle fiber number QTL (2826) | 14 | 1.16E+08 | 1.64E+08 |
| ENSSSCG00000010652 | 14 | 1.25E+08 | 1.25E+08 | Diameter of type IIa muscle fibers QTL (2827) | 14 | 1.16E+08 | 1.64E+08 |
| ENSSSCG00000010652 | 14 | 1.25E+08 | 1.25E+08 | Diameter of type IIb muscle fibers QTL (2828) | 14 | 1.16E+08 | 1.64E+08 |
| ENSSSCG00000010703 | 14 | 1.32E+08 | 1.32E+08 | Meat color L* QTL (38110) | 14 | 9407982 | 1.38E+08 |
| ENSSSCG00000010703 | 14 | 1.32E+08 | 1.32E+08 | Arachidonic acid content QTL (156959) | 14 | 9407982 | 1.38E+08 |
| ENSSSCG00000010703 | 14 | 1.32E+08 | 1.32E+08 | Shear force at first peak QTL (3929) | 14 | 10933601 | 1.38E+08 |
| ENSSSCG00000010703 | 14 | 1.32E+08 | 1.32E+08 | Shear force at first peak QTL (3930) | 14 | 10933601 | 1.38E+08 |
| ENSSSCG00000010703 | 14 | 1.32E+08 | 1.32E+08 | Shear force at first peak QTL (3931) | 14 | 10933601 | 1.38E+08 |
| ENSSSCG00000010703 | 14 | 1.32E+08 | 1.32E+08 | Capric acid content QTL (156957) | 14 | 24537035 | 1.38E+08 |
| ENSSSCG00000010703 | 14 | 1.32E+08 | 1.32E+08 | Margaric acid content QTL (156958) | 14 | 24537035 | 1.38E+08 |
| ENSSSCG00000010703 | 14 | 1.32E+08 | 1.32E+08 | pH 24 hr post-mortem (loin) QTL (21861) | 14 | 58863458 | 1.38E+08 |
| ENSSSCG00000010703 | 14 | 1.32E+08 | 1.32E+08 | Saturated fatty acid content QTL (156906) | 14 | 64838799 | 1.38E+08 |
| ENSSSCG00000010703 | 14 | 1.32E+08 | 1.32E+08 | Unsaturated fatty acid content QTL (156907) | 14 | 64838799 | 1.38E+08 |
| ENSSSCG00000010703 | 14 | 1.32E+08 | 1.32E+08 | Toll-like receptor 9 level QTL (17626) | 14 | 76280940 | 1.42E+08 |
| ENSSSCG00000010703 | 14 | 1.32E+08 | 1.32E+08 | Meat color OPTO QTL (12076) | 14 | 76280940 | 1.42E+08 |
| ENSSSCG00000010703 | 14 | 1.32E+08 | 1.32E+08 | Thawing loss QTL (12077) | 14 | 76280940 | 1.42E+08 |
| ENSSSCG00000010703 | 14 | 1.32E+08 | 1.32E+08 | Shear force QTL (12079) | 14 | 76280940 | 1.42E+08 |
| ENSSSCG00000010703 | 14 | 1.32E+08 | 1.32E+08 | Shear force QTL (12080) | 14 | 76280940 | 1.42E+08 |
| ENSSSCG00000010703 | 14 | 1.32E+08 | 1.32E+08 | Estimated carcass lean content QTL (12081) | 14 | 76280940 | 1.42E+08 |
| ENSSSCG00000010703 | 14 | 1.32E+08 | 1.32E+08 | Loin muscle area QTL (12082) | 14 | 76280940 | 1.42E+08 |
| ENSSSCG00000010703 | 14 | 1.32E+08 | 1.32E+08 | pH 24 hr post mortem (ham) QTL (12110) | 14 | 76280940 | 1.42E+08 |
| ENSSSCG00000010703 | 14 | 1.32E+08 | 1.32E+08 | Estimated carcass lean content QTL (12111) | 14 | 76280940 | 1.42E+08 |
| ENSSSCG00000010703 | 14 | 1.32E+08 | 1.32E+08 | Loin muscle depth QTL (12112) | 14 | 76280940 | 1.42E+08 |
| ENSSSCG00000010703 | 14 | 1.32E+08 | 1.32E+08 | Loin muscle area QTL (12113) | 14 | 76280940 | 1.42E+08 |
| ENSSSCG00000010703 | 14 | 1.32E+08 | 1.32E+08 | Loin muscle area QTL (12114) | 14 | 76280940 | 1.42E+08 |
| ENSSSCG00000010703 | 14 | 1.32E+08 | 1.32E+08 | Lactate level QTL (6358) | 14 | 96876793 | 1.38E+08 |
| ENSSSCG00000010703 | 14 | 1.32E+08 | 1.32E+08 | androstenone, sensory panel QTL (4236) | 14 | 1.12E+08 | 1.38E+08 |
| ENSSSCG00000010703 | 14 | 1.32E+08 | 1.32E+08 | subjective boar flavor in lean QTL (4240) | 14 | 1.12E+08 | 1.38E+08 |
| ENSSSCG00000010703 | 14 | 1.32E+08 | 1.32E+08 | Number of muscle fibers per unit area QTL (2825) | 14 | 1.16E+08 | 1.64E+08 |
| ENSSSCG00000010703 | 14 | 1.32E+08 | 1.32E+08 | Total muscle fiber number QTL (2826) | 14 | 1.16E+08 | 1.64E+08 |
| ENSSSCG00000010703 | 14 | 1.32E+08 | 1.32E+08 | Diameter of type IIa muscle fibers QTL (2827) | 14 | 1.16E+08 | 1.64E+08 |
| ENSSSCG00000010703 | 14 | 1.32E+08 | 1.32E+08 | Diameter of type IIb muscle fibers QTL (2828) | 14 | 1.16E+08 | 1.64E+08 |
| ENSSSCG00000010719 | 14 | 1.32E+08 | 1.32E+08 | Meat color L* QTL (38110) | 14 | 9407982 | 1.38E+08 |
| ENSSSCG00000010719 | 14 | 1.32E+08 | 1.32E+08 | Arachidonic acid content QTL (156959) | 14 | 9407982 | 1.38E+08 |
| ENSSSCG00000010719 | 14 | 1.32E+08 | 1.32E+08 | Shear force at first peak QTL (3929) | 14 | 10933601 | 1.38E+08 |
| ENSSSCG00000010719 | 14 | 1.32E+08 | 1.32E+08 | Shear force at first peak QTL (3930) | 14 | 10933601 | 1.38E+08 |
| ENSSSCG00000010719 | 14 | 1.32E+08 | 1.32E+08 | Shear force at first peak QTL (3931) | 14 | 10933601 | 1.38E+08 |
| ENSSSCG00000010719 | 14 | 1.32E+08 | 1.32E+08 | Capric acid content QTL (156957) | 14 | 24537035 | 1.38E+08 |
| ENSSSCG00000010719 | 14 | 1.32E+08 | 1.32E+08 | Margaric acid content QTL (156958) | 14 | 24537035 | 1.38E+08 |
| ENSSSCG00000010719 | 14 | 1.32E+08 | 1.32E+08 | pH 24 hr post-mortem (loin) QTL (21861) | 14 | 58863458 | 1.38E+08 |
| ENSSSCG00000010719 | 14 | 1.32E+08 | 1.32E+08 | Saturated fatty acid content QTL (156906) | 14 | 64838799 | 1.38E+08 |
| ENSSSCG00000010719 | 14 | 1.32E+08 | 1.32E+08 | Unsaturated fatty acid content QTL (156907) | 14 | 64838799 | 1.38E+08 |
| ENSSSCG00000010719 | 14 | 1.32E+08 | 1.32E+08 | Toll-like receptor 9 level QTL (17626) | 14 | 76280940 | 1.42E+08 |
| ENSSSCG00000010719 | 14 | 1.32E+08 | 1.32E+08 | Meat color OPTO QTL (12076) | 14 | 76280940 | 1.42E+08 |
| ENSSSCG00000010719 | 14 | 1.32E+08 | 1.32E+08 | Thawing loss QTL (12077) | 14 | 76280940 | 1.42E+08 |
| ENSSSCG00000010719 | 14 | 1.32E+08 | 1.32E+08 | Shear force QTL (12079) | 14 | 76280940 | 1.42E+08 |
| ENSSSCG00000010719 | 14 | 1.32E+08 | 1.32E+08 | Shear force QTL (12080) | 14 | 76280940 | 1.42E+08 |
| ENSSSCG00000010719 | 14 | 1.32E+08 | 1.32E+08 | Estimated carcass lean content QTL (12081) | 14 | 76280940 | 1.42E+08 |
| ENSSSCG00000010719 | 14 | 1.32E+08 | 1.32E+08 | Loin muscle area QTL (12082) | 14 | 76280940 | 1.42E+08 |
| ENSSSCG00000010719 | 14 | 1.32E+08 | 1.32E+08 | pH 24 hr post mortem (ham) QTL (12110) | 14 | 76280940 | 1.42E+08 |
| ENSSSCG00000010719 | 14 | 1.32E+08 | 1.32E+08 | Estimated carcass lean content QTL (12111) | 14 | 76280940 | 1.42E+08 |
| ENSSSCG00000010719 | 14 | 1.32E+08 | 1.32E+08 | Loin muscle depth QTL (12112) | 14 | 76280940 | 1.42E+08 |
| ENSSSCG00000010719 | 14 | 1.32E+08 | 1.32E+08 | Loin muscle area QTL (12113) | 14 | 76280940 | 1.42E+08 |
| ENSSSCG00000010719 | 14 | 1.32E+08 | 1.32E+08 | Loin muscle area QTL (12114) | 14 | 76280940 | 1.42E+08 |
| ENSSSCG00000010719 | 14 | 1.32E+08 | 1.32E+08 | Lactate level QTL (6358) | 14 | 96876793 | 1.38E+08 |
| ENSSSCG00000010719 | 14 | 1.32E+08 | 1.32E+08 | androstenone, sensory panel QTL (4236) | 14 | 1.12E+08 | 1.38E+08 |
| ENSSSCG00000010719 | 14 | 1.32E+08 | 1.32E+08 | subjective boar flavor in lean QTL (4240) | 14 | 1.12E+08 | 1.38E+08 |
| ENSSSCG00000010719 | 14 | 1.32E+08 | 1.32E+08 | Number of muscle fibers per unit area QTL (2825) | 14 | 1.16E+08 | 1.64E+08 |
| ENSSSCG00000010719 | 14 | 1.32E+08 | 1.32E+08 | Total muscle fiber number QTL (2826) | 14 | 1.16E+08 | 1.64E+08 |
| ENSSSCG00000010719 | 14 | 1.32E+08 | 1.32E+08 | Diameter of type IIa muscle fibers QTL (2827) | 14 | 1.16E+08 | 1.64E+08 |
| ENSSSCG00000010719 | 14 | 1.32E+08 | 1.32E+08 | Diameter of type IIb muscle fibers QTL (2828) | 14 | 1.16E+08 | 1.64E+08 |
| ENSSSCG00000023865 | 14 | 1.33E+08 | 1.33E+08 | Meat color L* QTL (38110) | 14 | 9407982 | 1.38E+08 |
| ENSSSCG00000023865 | 14 | 1.33E+08 | 1.33E+08 | Arachidonic acid content QTL (156959) | 14 | 9407982 | 1.38E+08 |
| ENSSSCG00000023865 | 14 | 1.33E+08 | 1.33E+08 | Shear force at first peak QTL (3929) | 14 | 10933601 | 1.38E+08 |
| ENSSSCG00000023865 | 14 | 1.33E+08 | 1.33E+08 | Shear force at first peak QTL (3930) | 14 | 10933601 | 1.38E+08 |
| ENSSSCG00000023865 | 14 | 1.33E+08 | 1.33E+08 | Shear force at first peak QTL (3931) | 14 | 10933601 | 1.38E+08 |
| ENSSSCG00000023865 | 14 | 1.33E+08 | 1.33E+08 | Capric acid content QTL (156957) | 14 | 24537035 | 1.38E+08 |
| ENSSSCG00000023865 | 14 | 1.33E+08 | 1.33E+08 | Margaric acid content QTL (156958) | 14 | 24537035 | 1.38E+08 |
| ENSSSCG00000023865 | 14 | 1.33E+08 | 1.33E+08 | pH 24 hr post-mortem (loin) QTL (21861) | 14 | 58863458 | 1.38E+08 |
| ENSSSCG00000023865 | 14 | 1.33E+08 | 1.33E+08 | Saturated fatty acid content QTL (156906) | 14 | 64838799 | 1.38E+08 |
| ENSSSCG00000023865 | 14 | 1.33E+08 | 1.33E+08 | Unsaturated fatty acid content QTL (156907) | 14 | 64838799 | 1.38E+08 |
| ENSSSCG00000023865 | 14 | 1.33E+08 | 1.33E+08 | Toll-like receptor 9 level QTL (17626) | 14 | 76280940 | 1.42E+08 |
| ENSSSCG00000023865 | 14 | 1.33E+08 | 1.33E+08 | Meat color OPTO QTL (12076) | 14 | 76280940 | 1.42E+08 |
| ENSSSCG00000023865 | 14 | 1.33E+08 | 1.33E+08 | Thawing loss QTL (12077) | 14 | 76280940 | 1.42E+08 |
| ENSSSCG00000023865 | 14 | 1.33E+08 | 1.33E+08 | Shear force QTL (12079) | 14 | 76280940 | 1.42E+08 |
| ENSSSCG00000023865 | 14 | 1.33E+08 | 1.33E+08 | Shear force QTL (12080) | 14 | 76280940 | 1.42E+08 |
| ENSSSCG00000023865 | 14 | 1.33E+08 | 1.33E+08 | Estimated carcass lean content QTL (12081) | 14 | 76280940 | 1.42E+08 |
| ENSSSCG00000023865 | 14 | 1.33E+08 | 1.33E+08 | Loin muscle area QTL (12082) | 14 | 76280940 | 1.42E+08 |
| ENSSSCG00000023865 | 14 | 1.33E+08 | 1.33E+08 | pH 24 hr post mortem (ham) QTL (12110) | 14 | 76280940 | 1.42E+08 |
| ENSSSCG00000023865 | 14 | 1.33E+08 | 1.33E+08 | Estimated carcass lean content QTL (12111) | 14 | 76280940 | 1.42E+08 |
| ENSSSCG00000023865 | 14 | 1.33E+08 | 1.33E+08 | Loin muscle depth QTL (12112) | 14 | 76280940 | 1.42E+08 |
| ENSSSCG00000023865 | 14 | 1.33E+08 | 1.33E+08 | Loin muscle area QTL (12113) | 14 | 76280940 | 1.42E+08 |
| ENSSSCG00000023865 | 14 | 1.33E+08 | 1.33E+08 | Loin muscle area QTL (12114) | 14 | 76280940 | 1.42E+08 |
| ENSSSCG00000023865 | 14 | 1.33E+08 | 1.33E+08 | Lactate level QTL (6358) | 14 | 96876793 | 1.38E+08 |
| ENSSSCG00000023865 | 14 | 1.33E+08 | 1.33E+08 | androstenone, sensory panel QTL (4236) | 14 | 1.12E+08 | 1.38E+08 |
| ENSSSCG00000023865 | 14 | 1.33E+08 | 1.33E+08 | subjective boar flavor in lean QTL (4240) | 14 | 1.12E+08 | 1.38E+08 |
| ENSSSCG00000023865 | 14 | 1.33E+08 | 1.33E+08 | Number of muscle fibers per unit area QTL (2825) | 14 | 1.16E+08 | 1.64E+08 |
| ENSSSCG00000023865 | 14 | 1.33E+08 | 1.33E+08 | Total muscle fiber number QTL (2826) | 14 | 1.16E+08 | 1.64E+08 |
| ENSSSCG00000023865 | 14 | 1.33E+08 | 1.33E+08 | Diameter of type IIa muscle fibers QTL (2827) | 14 | 1.16E+08 | 1.64E+08 |
| ENSSSCG00000023865 | 14 | 1.33E+08 | 1.33E+08 | Diameter of type IIb muscle fibers QTL (2828) | 14 | 1.16E+08 | 1.64E+08 |
| ENSSSCG00000010734 | 14 | 1.34E+08 | 1.34E+08 | Meat color L* QTL (38110) | 14 | 9407982 | 1.38E+08 |
| ENSSSCG00000010734 | 14 | 1.34E+08 | 1.34E+08 | Arachidonic acid content QTL (156959) | 14 | 9407982 | 1.38E+08 |
| ENSSSCG00000010734 | 14 | 1.34E+08 | 1.34E+08 | Shear force at first peak QTL (3929) | 14 | 10933601 | 1.38E+08 |
| ENSSSCG00000010734 | 14 | 1.34E+08 | 1.34E+08 | Shear force at first peak QTL (3930) | 14 | 10933601 | 1.38E+08 |
| ENSSSCG00000010734 | 14 | 1.34E+08 | 1.34E+08 | Shear force at first peak QTL (3931) | 14 | 10933601 | 1.38E+08 |
| ENSSSCG00000010734 | 14 | 1.34E+08 | 1.34E+08 | Capric acid content QTL (156957) | 14 | 24537035 | 1.38E+08 |
| ENSSSCG00000010734 | 14 | 1.34E+08 | 1.34E+08 | Margaric acid content QTL (156958) | 14 | 24537035 | 1.38E+08 |
| ENSSSCG00000010734 | 14 | 1.34E+08 | 1.34E+08 | pH 24 hr post-mortem (loin) QTL (21861) | 14 | 58863458 | 1.38E+08 |
| ENSSSCG00000010734 | 14 | 1.34E+08 | 1.34E+08 | Saturated fatty acid content QTL (156906) | 14 | 64838799 | 1.38E+08 |
| ENSSSCG00000010734 | 14 | 1.34E+08 | 1.34E+08 | Unsaturated fatty acid content QTL (156907) | 14 | 64838799 | 1.38E+08 |
| ENSSSCG00000010734 | 14 | 1.34E+08 | 1.34E+08 | Toll-like receptor 9 level QTL (17626) | 14 | 76280940 | 1.42E+08 |
| ENSSSCG00000010734 | 14 | 1.34E+08 | 1.34E+08 | Meat color OPTO QTL (12076) | 14 | 76280940 | 1.42E+08 |
| ENSSSCG00000010734 | 14 | 1.34E+08 | 1.34E+08 | Thawing loss QTL (12077) | 14 | 76280940 | 1.42E+08 |
| ENSSSCG00000010734 | 14 | 1.34E+08 | 1.34E+08 | Shear force QTL (12079) | 14 | 76280940 | 1.42E+08 |
| ENSSSCG00000010734 | 14 | 1.34E+08 | 1.34E+08 | Shear force QTL (12080) | 14 | 76280940 | 1.42E+08 |
| ENSSSCG00000010734 | 14 | 1.34E+08 | 1.34E+08 | Estimated carcass lean content QTL (12081) | 14 | 76280940 | 1.42E+08 |
| ENSSSCG00000010734 | 14 | 1.34E+08 | 1.34E+08 | Loin muscle area QTL (12082) | 14 | 76280940 | 1.42E+08 |
| ENSSSCG00000010734 | 14 | 1.34E+08 | 1.34E+08 | pH 24 hr post mortem (ham) QTL (12110) | 14 | 76280940 | 1.42E+08 |
| ENSSSCG00000010734 | 14 | 1.34E+08 | 1.34E+08 | Estimated carcass lean content QTL (12111) | 14 | 76280940 | 1.42E+08 |
| ENSSSCG00000010734 | 14 | 1.34E+08 | 1.34E+08 | Loin muscle depth QTL (12112) | 14 | 76280940 | 1.42E+08 |
| ENSSSCG00000010734 | 14 | 1.34E+08 | 1.34E+08 | Loin muscle area QTL (12113) | 14 | 76280940 | 1.42E+08 |
| ENSSSCG00000010734 | 14 | 1.34E+08 | 1.34E+08 | Loin muscle area QTL (12114) | 14 | 76280940 | 1.42E+08 |
| ENSSSCG00000010734 | 14 | 1.34E+08 | 1.34E+08 | Lactate level QTL (6358) | 14 | 96876793 | 1.38E+08 |
| ENSSSCG00000010734 | 14 | 1.34E+08 | 1.34E+08 | androstenone, sensory panel QTL (4236) | 14 | 1.12E+08 | 1.38E+08 |
| ENSSSCG00000010734 | 14 | 1.34E+08 | 1.34E+08 | subjective boar flavor in lean QTL (4240) | 14 | 1.12E+08 | 1.38E+08 |
| ENSSSCG00000010734 | 14 | 1.34E+08 | 1.34E+08 | Number of muscle fibers per unit area QTL (2825) | 14 | 1.16E+08 | 1.64E+08 |
| ENSSSCG00000010734 | 14 | 1.34E+08 | 1.34E+08 | Total muscle fiber number QTL (2826) | 14 | 1.16E+08 | 1.64E+08 |
| ENSSSCG00000010734 | 14 | 1.34E+08 | 1.34E+08 | Diameter of type IIa muscle fibers QTL (2827) | 14 | 1.16E+08 | 1.64E+08 |
| ENSSSCG00000010734 | 14 | 1.34E+08 | 1.34E+08 | Diameter of type IIb muscle fibers QTL (2828) | 14 | 1.16E+08 | 1.64E+08 |
| ENSSSCG00000039157 | 15 | 29782604 | 30042734 | Meat color score QTL (3009) | 15 | 3388881 | 33323996 |
| ENSSSCG00000039157 | 15 | 29782604 | 30042734 | Cooking loss QTL (3010) | 15 | 3388881 | 33323996 |
| ENSSSCG00000039157 | 15 | 29782604 | 30042734 | Shear force QTL (3011) | 15 | 3388881 | 33323996 |
| ENSSSCG00000039157 | 15 | 29782604 | 30042734 | Shear force QTL (4009) | 15 | 14120337 | 32646220 |
| ENSSSCG00000039157 | 15 | 29782604 | 30042734 | Percentage type I fibers QTL (7013) | 15 | 29031413 | 44959154 |
| ENSSSCG00000039157 | 15 | 29782604 | 30042734 | Shear force QTL (38113) | 15 | 15588852 | 1.4E+08 |
| ENSSSCG00000039157 | 15 | 29782604 | 30042734 | Backfat at tenth rib QTL (5725) | 15 | 15588852 | 1.35E+08 |
| ENSSSCG00000015743 | 15 | 31307444 | 31502924 | Meat color score QTL (3009) | 15 | 3388881 | 33323996 |
| ENSSSCG00000015743 | 15 | 31307444 | 31502924 | Cooking loss QTL (3010) | 15 | 3388881 | 33323996 |
| ENSSSCG00000015743 | 15 | 31307444 | 31502924 | Shear force QTL (3011) | 15 | 3388881 | 33323996 |
| ENSSSCG00000015743 | 15 | 31307444 | 31502924 | Shear force QTL (4009) | 15 | 14120337 | 32646220 |
| ENSSSCG00000015743 | 15 | 31307444 | 31502924 | Percentage type I fibers QTL (7013) | 15 | 29031413 | 44959154 |
| ENSSSCG00000015743 | 15 | 31307444 | 31502924 | Average backfat thickness QTL (17811) | 15 | 30048078 | 57498014 |
| ENSSSCG00000015743 | 15 | 31307444 | 31502924 | Shear force QTL (38113) | 15 | 15588852 | 1.4E+08 |
| ENSSSCG00000015743 | 15 | 31307444 | 31502924 | Backfat at tenth rib QTL (5725) | 15 | 15588852 | 1.35E+08 |
| ENSSSCG00000015777 | 15 | 44989636 | 45040761 | Average backfat thickness QTL (2925) | 15 | 44767118 | 45230265 |
| ENSSSCG00000015777 | 15 | 44989636 | 45040761 | Average backfat thickness QTL (17811) | 15 | 30048078 | 57498014 |
| ENSSSCG00000015777 | 15 | 44989636 | 45040761 | Palmitic acid content QTL (3117) | 15 | 32646220 | 57272088 |
| ENSSSCG00000015777 | 15 | 44989636 | 45040761 | Saturated fatty acid content QTL (3122) | 15 | 32646220 | 57272088 |
| ENSSSCG00000015777 | 15 | 44989636 | 45040761 | Loin muscle area QTL (16937) | 15 | 39085094 | 65405403 |
| ENSSSCG00000015777 | 15 | 44989636 | 45040761 | Dressing - Ham over carcass QTL (1243) | 15 | 39085094 | 64490405 |
| ENSSSCG00000015777 | 15 | 44989636 | 45040761 | Average backfat thickness QTL (1174) | 15 | 39085094 | 53544319 |
| ENSSSCG00000015777 | 15 | 44989636 | 45040761 | Backfat at tenth rib QTL (1175) | 15 | 39085094 | 53544319 |
| ENSSSCG00000015777 | 15 | 44989636 | 45040761 | Backfat at last rib QTL (1176) | 15 | 39085094 | 53544319 |
| ENSSSCG00000015777 | 15 | 44989636 | 45040761 | Shear force QTL (38113) | 15 | 15588852 | 1.4E+08 |
| ENSSSCG00000015777 | 15 | 44989636 | 45040761 | Backfat at tenth rib QTL (5725) | 15 | 15588852 | 1.35E+08 |
| ENSSSCG00000015777 | 15 | 44989636 | 45040761 | Loin muscle area QTL (3658) | 15 | 39085094 | 73538717 |
| ENSSSCG00000015777 | 15 | 44989636 | 45040761 | Time spent drinking QTL (5915) | 15 | 39085094 | 67777620 |
| ENSSSCG00000015777 | 15 | 44989636 | 45040761 | Meat color OPTO QTL (12094) | 15 | 44959154 | 99971989 |
| ENSSSCG00000015777 | 15 | 44989636 | 45040761 | Conductivity 24 hours postmortem (loin) QTL (12095) | 15 | 44959154 | 99971989 |
| ENSSSCG00000015777 | 15 | 44989636 | 45040761 | Loin muscle area QTL (12097) | 15 | 44959154 | 99971989 |
| ENSSSCG00000015777 | 15 | 44989636 | 45040761 | Total muscle fiber number QTL (2829) | 15 | 44959154 | 99971989 |
| ENSSSCG00000015777 | 15 | 44989636 | 45040761 | Percentage angular fibers QTL (2830) | 15 | 44959154 | 99971989 |
| ENSSSCG00000015777 | 15 | 44989636 | 45040761 | Diameter of giant fibers QTL (2831) | 15 | 44959154 | 99971989 |
| ENSSSCG00000015777 | 15 | 44989636 | 45040761 | Diameter of type I muscle fibers QTL (2832) | 15 | 44959154 | 99971989 |
| ENSSSCG00000015777 | 15 | 44989636 | 45040761 | Diameter of type IIa muscle fibers QTL (2833) | 15 | 44959154 | 99971989 |
| ENSSSCG00000015777 | 15 | 44989636 | 45040761 | Average backfat thickness QTL (5724) | 15 | 44959154 | 99971989 |
| ENSSSCG00000015777 | 15 | 44989636 | 45040761 | Backfat at tenth rib QTL (5992) | 15 | 44959154 | 99971989 |
| ENSSSCG00000015777 | 15 | 44989636 | 45040761 | pH 24 hr post-mortem (loin) QTL (123) | 15 | 44959154 | 82575733 |
| ENSSSCG00000015840 | 15 | 53966972 | 54094587 | Average backfat thickness QTL (17811) | 15 | 30048078 | 57498014 |
| ENSSSCG00000015840 | 15 | 53966972 | 54094587 | Palmitic acid content QTL (3117) | 15 | 32646220 | 57272088 |
| ENSSSCG00000015840 | 15 | 53966972 | 54094587 | Saturated fatty acid content QTL (3122) | 15 | 32646220 | 57272088 |
| ENSSSCG00000015840 | 15 | 53966972 | 54094587 | Loin muscle area QTL (16937) | 15 | 39085094 | 65405403 |
| ENSSSCG00000015840 | 15 | 53966972 | 54094587 | Dressing - Ham over carcass QTL (1243) | 15 | 39085094 | 64490405 |
| ENSSSCG00000015840 | 15 | 53966972 | 54094587 | Shear force QTL (38113) | 15 | 15588852 | 1.4E+08 |
| ENSSSCG00000015840 | 15 | 53966972 | 54094587 | Backfat at tenth rib QTL (5725) | 15 | 15588852 | 1.35E+08 |
| ENSSSCG00000015840 | 15 | 53966972 | 54094587 | Loin muscle area QTL (3658) | 15 | 39085094 | 73538717 |
| ENSSSCG00000015840 | 15 | 53966972 | 54094587 | Time spent drinking QTL (5915) | 15 | 39085094 | 67777620 |
| ENSSSCG00000015840 | 15 | 53966972 | 54094587 | Meat color OPTO QTL (12094) | 15 | 44959154 | 99971989 |
| ENSSSCG00000015840 | 15 | 53966972 | 54094587 | Conductivity 24 hours postmortem (loin) QTL (12095) | 15 | 44959154 | 99971989 |
| ENSSSCG00000015840 | 15 | 53966972 | 54094587 | Loin muscle area QTL (12097) | 15 | 44959154 | 99971989 |
| ENSSSCG00000015840 | 15 | 53966972 | 54094587 | Total muscle fiber number QTL (2829) | 15 | 44959154 | 99971989 |
| ENSSSCG00000015840 | 15 | 53966972 | 54094587 | Percentage angular fibers QTL (2830) | 15 | 44959154 | 99971989 |
| ENSSSCG00000015840 | 15 | 53966972 | 54094587 | Diameter of giant fibers QTL (2831) | 15 | 44959154 | 99971989 |
| ENSSSCG00000015840 | 15 | 53966972 | 54094587 | Diameter of type I muscle fibers QTL (2832) | 15 | 44959154 | 99971989 |
| ENSSSCG00000015840 | 15 | 53966972 | 54094587 | Diameter of type IIa muscle fibers QTL (2833) | 15 | 44959154 | 99971989 |
| ENSSSCG00000015840 | 15 | 53966972 | 54094587 | Average backfat thickness QTL (5724) | 15 | 44959154 | 99971989 |
| ENSSSCG00000015840 | 15 | 53966972 | 54094587 | Backfat at tenth rib QTL (5992) | 15 | 44959154 | 99971989 |
| ENSSSCG00000015840 | 15 | 53966972 | 54094587 | pH 24 hr post-mortem (loin) QTL (123) | 15 | 44959154 | 82575733 |
| ENSSSCG00000015840 | 15 | 53966972 | 54094587 | Creatinine level QTL (15081) | 15 | 46314707 | 1.4E+08 |
| ENSSSCG00000015840 | 15 | 53966972 | 54094587 | Loin muscle area QTL (21719) | 15 | 50833215 | 72296128 |
| ENSSSCG00000015867 | 15 | 60386596 | 60440523 | Loin muscle area QTL (16937) | 15 | 39085094 | 65405403 |
| ENSSSCG00000015867 | 15 | 60386596 | 60440523 | Dressing - Ham over carcass QTL (1243) | 15 | 39085094 | 64490405 |
| ENSSSCG00000015867 | 15 | 60386596 | 60440523 | Shear force QTL (38113) | 15 | 15588852 | 1.4E+08 |
| ENSSSCG00000015867 | 15 | 60386596 | 60440523 | Backfat at tenth rib QTL (5725) | 15 | 15588852 | 1.35E+08 |
| ENSSSCG00000015867 | 15 | 60386596 | 60440523 | Loin muscle area QTL (3658) | 15 | 39085094 | 73538717 |
| ENSSSCG00000015867 | 15 | 60386596 | 60440523 | Time spent drinking QTL (5915) | 15 | 39085094 | 67777620 |
| ENSSSCG00000015867 | 15 | 60386596 | 60440523 | Meat color OPTO QTL (12094) | 15 | 44959154 | 99971989 |
| ENSSSCG00000015867 | 15 | 60386596 | 60440523 | Conductivity 24 hours postmortem (loin) QTL (12095) | 15 | 44959154 | 99971989 |
| ENSSSCG00000015867 | 15 | 60386596 | 60440523 | Loin muscle area QTL (12097) | 15 | 44959154 | 99971989 |
| ENSSSCG00000015867 | 15 | 60386596 | 60440523 | Total muscle fiber number QTL (2829) | 15 | 44959154 | 99971989 |
| ENSSSCG00000015867 | 15 | 60386596 | 60440523 | Percentage angular fibers QTL (2830) | 15 | 44959154 | 99971989 |
| ENSSSCG00000015867 | 15 | 60386596 | 60440523 | Diameter of giant fibers QTL (2831) | 15 | 44959154 | 99971989 |
| ENSSSCG00000015867 | 15 | 60386596 | 60440523 | Diameter of type I muscle fibers QTL (2832) | 15 | 44959154 | 99971989 |
| ENSSSCG00000015867 | 15 | 60386596 | 60440523 | Diameter of type IIa muscle fibers QTL (2833) | 15 | 44959154 | 99971989 |
| ENSSSCG00000015867 | 15 | 60386596 | 60440523 | Average backfat thickness QTL (5724) | 15 | 44959154 | 99971989 |
| ENSSSCG00000015867 | 15 | 60386596 | 60440523 | Backfat at tenth rib QTL (5992) | 15 | 44959154 | 99971989 |
| ENSSSCG00000015867 | 15 | 60386596 | 60440523 | pH 24 hr post-mortem (loin) QTL (123) | 15 | 44959154 | 82575733 |
| ENSSSCG00000015867 | 15 | 60386596 | 60440523 | Creatinine level QTL (15081) | 15 | 46314707 | 1.4E+08 |
| ENSSSCG00000015867 | 15 | 60386596 | 60440523 | Loin muscle area QTL (21719) | 15 | 50833215 | 72296128 |
| ENSSSCG00000015867 | 15 | 60386596 | 60440523 | Backfat at first rib QTL (3975) | 15 | 56470053 | 1.35E+08 |
| ENSSSCG00000015867 | 15 | 60386596 | 60440523 | Drip loss QTL (3014) | 15 | 57272088 | 79638703 |
| ENSSSCG00000015867 | 15 | 60386596 | 60440523 | Shear force QTL (3015) | 15 | 57272088 | 79638703 |
| ENSSSCG00000015867 | 15 | 60386596 | 60440523 | pH 48 hr post-mortem (loin) QTL (2964) | 15 | 57272088 | 76249822 |
| ENSSSCG00000015908 | 15 | 72430725 | 72512491 | Shear force QTL (38113) | 15 | 15588852 | 1.4E+08 |
| ENSSSCG00000015908 | 15 | 72430725 | 72512491 | Backfat at tenth rib QTL (5725) | 15 | 15588852 | 1.35E+08 |
| ENSSSCG00000015908 | 15 | 72430725 | 72512491 | Loin muscle area QTL (3658) | 15 | 39085094 | 73538717 |
| ENSSSCG00000015908 | 15 | 72430725 | 72512491 | Meat color OPTO QTL (12094) | 15 | 44959154 | 99971989 |
| ENSSSCG00000015908 | 15 | 72430725 | 72512491 | Conductivity 24 hours postmortem (loin) QTL (12095) | 15 | 44959154 | 99971989 |
| ENSSSCG00000015908 | 15 | 72430725 | 72512491 | Loin muscle area QTL (12097) | 15 | 44959154 | 99971989 |
| ENSSSCG00000015908 | 15 | 72430725 | 72512491 | Total muscle fiber number QTL (2829) | 15 | 44959154 | 99971989 |
| ENSSSCG00000015908 | 15 | 72430725 | 72512491 | Percentage angular fibers QTL (2830) | 15 | 44959154 | 99971989 |
| ENSSSCG00000015908 | 15 | 72430725 | 72512491 | Diameter of giant fibers QTL (2831) | 15 | 44959154 | 99971989 |
| ENSSSCG00000015908 | 15 | 72430725 | 72512491 | Diameter of type I muscle fibers QTL (2832) | 15 | 44959154 | 99971989 |
| ENSSSCG00000015908 | 15 | 72430725 | 72512491 | Diameter of type IIa muscle fibers QTL (2833) | 15 | 44959154 | 99971989 |
| ENSSSCG00000015908 | 15 | 72430725 | 72512491 | Average backfat thickness QTL (5724) | 15 | 44959154 | 99971989 |
| ENSSSCG00000015908 | 15 | 72430725 | 72512491 | Backfat at tenth rib QTL (5992) | 15 | 44959154 | 99971989 |
| ENSSSCG00000015908 | 15 | 72430725 | 72512491 | pH 24 hr post-mortem (loin) QTL (123) | 15 | 44959154 | 82575733 |
| ENSSSCG00000015908 | 15 | 72430725 | 72512491 | Creatinine level QTL (15081) | 15 | 46314707 | 1.4E+08 |
| ENSSSCG00000015908 | 15 | 72430725 | 72512491 | Backfat at first rib QTL (3975) | 15 | 56470053 | 1.35E+08 |
| ENSSSCG00000015908 | 15 | 72430725 | 72512491 | Drip loss QTL (3014) | 15 | 57272088 | 79638703 |
| ENSSSCG00000015908 | 15 | 72430725 | 72512491 | Shear force QTL (3015) | 15 | 57272088 | 79638703 |
| ENSSSCG00000015908 | 15 | 72430725 | 72512491 | pH 48 hr post-mortem (loin) QTL (2964) | 15 | 57272088 | 76249822 |
| ENSSSCG00000015908 | 15 | 72430725 | 72512491 | Shoulder subcutaneous fat thickness QTL (21270) | 15 | 60999858 | 86190540 |
| ENSSSCG00000015908 | 15 | 72430725 | 72512491 | Meat color a* QTL (21721) | 15 | 62129485 | 83592398 |
| ENSSSCG00000015908 | 15 | 72430725 | 72512491 | Meat color L* QTL (21723) | 15 | 62129485 | 83592398 |
| ENSSSCG00000015908 | 15 | 72430725 | 72512491 | pH 24 hr post-mortem (loin) QTL (293) | 15 | 63259112 | 1.35E+08 |
| ENSSSCG00000015908 | 15 | 72430725 | 72512491 | pH 24 hr post-mortem (loin) QTL (122) | 15 | 63259112 | 1.28E+08 |
| ENSSSCG00000015908 | 15 | 72430725 | 72512491 | Skin thickness QTL (31812) | 15 | 65179477 | 98051623 |
| ENSSSCG00000015908 | 15 | 72430725 | 72512491 | Arachidic acid content QTL (156960) | 15 | 65405403 | 1.34E+08 |
| ENSSSCG00000015908 | 15 | 72430725 | 72512491 | Intramuscular fat content QTL (4043) | 15 | 65857254 | 83027584 |
| ENSSSCG00000023983 | 15 | 83145573 | 83391970 | Meat color b* QTL (55968) | 15 | 83209842 | 83209846 |
| ENSSSCG00000023983 | 15 | 83145573 | 83391970 | Backfat between 3rd and 4th last ribs QTL (31286) | 15 | 83361854 | 83361858 |
| ENSSSCG00000023983 | 15 | 83145573 | 83391970 | Abdominal fat weight QTL (21269) | 15 | 72860941 | 1.27E+08 |
| ENSSSCG00000023983 | 15 | 83145573 | 83391970 | pH 24 hr post-mortem (loin) QTL (4042) | 15 | 73425755 | 93533115 |
| ENSSSCG00000023983 | 15 | 83145573 | 83391970 | Average daily gain QTL (16857) | 15 | 73538717 | 1.15E+08 |
| ENSSSCG00000023983 | 15 | 83145573 | 83391970 | Average daily gain QTL (16892) | 15 | 73538717 | 1.15E+08 |
| ENSSSCG00000023983 | 15 | 83145573 | 83391970 | Meat color-a QTL (237) | 15 | 73538717 | 1.01E+08 |
| ENSSSCG00000023983 | 15 | 83145573 | 83391970 | Loin muscle area QTL (16938) | 15 | 73538717 | 89579421 |
| ENSSSCG00000023983 | 15 | 83145573 | 83391970 | muscle protein percentage QTL (3291) | 15 | 73538717 | 89579421 |
| ENSSSCG00000023983 | 15 | 83145573 | 83391970 | Meat color-a QTL (3292) | 15 | 73538717 | 89579421 |
| ENSSSCG00000023983 | 15 | 83145573 | 83391970 | Meat color score QTL (3293) | 15 | 73538717 | 89579421 |
| ENSSSCG00000023983 | 15 | 83145573 | 83391970 | Meat color-L QTL (3311) | 15 | 73538717 | 89579421 |
| ENSSSCG00000023983 | 15 | 83145573 | 83391970 | Backfat at first rib QTL (21408) | 15 | 73538717 | 89579421 |
| ENSSSCG00000023983 | 15 | 83145573 | 83391970 | Backfat at tenth rib QTL (21409) | 15 | 73538717 | 89579421 |
| ENSSSCG00000023983 | 15 | 83145573 | 83391970 | Intramuscular fat content QTL (4044) | 15 | 75323528 | 98424400 |
| ENSSSCG00000023983 | 15 | 83145573 | 83391970 | Meat color b* QTL (4040) | 15 | 75346120 | 93533115 |
| ENSSSCG00000023983 | 15 | 83145573 | 83391970 | Average glycogen QTL (290) | 15 | 75797971 | 1.15E+08 |
| ENSSSCG00000023983 | 15 | 83145573 | 83391970 | Drip loss QTL (3790) | 15 | 76249822 | 89579421 |
| ENSSSCG00000023983 | 15 | 83145573 | 83391970 | Off-flavor score QTL (3811) | 15 | 76249822 | 89579421 |
| ENSSSCG00000023983 | 15 | 83145573 | 83391970 | Meat color-L QTL (120) | 15 | 79525740 | 1.09E+08 |
| ENSSSCG00000023983 | 15 | 83145573 | 83391970 | muscle protein percentage QTL (21722) | 15 | 81333144 | 96018295 |
| ENSSSCG00000023983 | 15 | 83145573 | 83391970 | Shear force QTL (38113) | 15 | 15588852 | 1.4E+08 |
| ENSSSCG00000023983 | 15 | 83145573 | 83391970 | Backfat at tenth rib QTL (5725) | 15 | 15588852 | 1.35E+08 |
| ENSSSCG00000023983 | 15 | 83145573 | 83391970 | Meat color OPTO QTL (12094) | 15 | 44959154 | 99971989 |
| ENSSSCG00000023983 | 15 | 83145573 | 83391970 | Conductivity 24 hours postmortem (loin) QTL (12095) | 15 | 44959154 | 99971989 |
| ENSSSCG00000023983 | 15 | 83145573 | 83391970 | Loin muscle area QTL (12097) | 15 | 44959154 | 99971989 |
| ENSSSCG00000023983 | 15 | 83145573 | 83391970 | Total muscle fiber number QTL (2829) | 15 | 44959154 | 99971989 |
| ENSSSCG00000023983 | 15 | 83145573 | 83391970 | Percentage angular fibers QTL (2830) | 15 | 44959154 | 99971989 |
| ENSSSCG00000023983 | 15 | 83145573 | 83391970 | Diameter of giant fibers QTL (2831) | 15 | 44959154 | 99971989 |
| ENSSSCG00000023983 | 15 | 83145573 | 83391970 | Diameter of type I muscle fibers QTL (2832) | 15 | 44959154 | 99971989 |
| ENSSSCG00000023983 | 15 | 83145573 | 83391970 | Diameter of type IIa muscle fibers QTL (2833) | 15 | 44959154 | 99971989 |
| ENSSSCG00000023983 | 15 | 83145573 | 83391970 | Average backfat thickness QTL (5724) | 15 | 44959154 | 99971989 |
| ENSSSCG00000023983 | 15 | 83145573 | 83391970 | Backfat at tenth rib QTL (5992) | 15 | 44959154 | 99971989 |
| ENSSSCG00000023983 | 15 | 83145573 | 83391970 | Creatinine level QTL (15081) | 15 | 46314707 | 1.4E+08 |
| ENSSSCG00000023983 | 15 | 83145573 | 83391970 | Backfat at first rib QTL (3975) | 15 | 56470053 | 1.35E+08 |
| ENSSSCG00000023983 | 15 | 83145573 | 83391970 | Shoulder subcutaneous fat thickness QTL (21270) | 15 | 60999858 | 86190540 |
| ENSSSCG00000023983 | 15 | 83145573 | 83391970 | Meat color a* QTL (21721) | 15 | 62129485 | 83592398 |
| ENSSSCG00000023983 | 15 | 83145573 | 83391970 | Meat color L* QTL (21723) | 15 | 62129485 | 83592398 |
| ENSSSCG00000023983 | 15 | 83145573 | 83391970 | pH 24 hr post-mortem (loin) QTL (293) | 15 | 63259112 | 1.35E+08 |
| ENSSSCG00000023983 | 15 | 83145573 | 83391970 | pH 24 hr post-mortem (loin) QTL (122) | 15 | 63259112 | 1.28E+08 |
| ENSSSCG00000023983 | 15 | 83145573 | 83391970 | Skin thickness QTL (31812) | 15 | 65179477 | 98051623 |
| ENSSSCG00000023983 | 15 | 83145573 | 83391970 | Arachidic acid content QTL (156960) | 15 | 65405403 | 1.34E+08 |
| ENSSSCG00000023133 | 15 | 83872742 | 84101299 | Abdominal fat weight QTL (21269) | 15 | 72860941 | 1.27E+08 |
| ENSSSCG00000023133 | 15 | 83872742 | 84101299 | pH 24 hr post-mortem (loin) QTL (4042) | 15 | 73425755 | 93533115 |
| ENSSSCG00000023133 | 15 | 83872742 | 84101299 | Average daily gain QTL (16857) | 15 | 73538717 | 1.15E+08 |
| ENSSSCG00000023133 | 15 | 83872742 | 84101299 | Average daily gain QTL (16892) | 15 | 73538717 | 1.15E+08 |
| ENSSSCG00000023133 | 15 | 83872742 | 84101299 | Meat color-a QTL (237) | 15 | 73538717 | 1.01E+08 |
| ENSSSCG00000023133 | 15 | 83872742 | 84101299 | Loin muscle area QTL (16938) | 15 | 73538717 | 89579421 |
| ENSSSCG00000023133 | 15 | 83872742 | 84101299 | muscle protein percentage QTL (3291) | 15 | 73538717 | 89579421 |
| ENSSSCG00000023133 | 15 | 83872742 | 84101299 | Meat color-a QTL (3292) | 15 | 73538717 | 89579421 |
| ENSSSCG00000023133 | 15 | 83872742 | 84101299 | Meat color score QTL (3293) | 15 | 73538717 | 89579421 |
| ENSSSCG00000023133 | 15 | 83872742 | 84101299 | Meat color-L QTL (3311) | 15 | 73538717 | 89579421 |
| ENSSSCG00000023133 | 15 | 83872742 | 84101299 | Backfat at first rib QTL (21408) | 15 | 73538717 | 89579421 |
| ENSSSCG00000023133 | 15 | 83872742 | 84101299 | Backfat at tenth rib QTL (21409) | 15 | 73538717 | 89579421 |
| ENSSSCG00000023133 | 15 | 83872742 | 84101299 | Intramuscular fat content QTL (4044) | 15 | 75323528 | 98424400 |
| ENSSSCG00000023133 | 15 | 83872742 | 84101299 | Meat color b* QTL (4040) | 15 | 75346120 | 93533115 |
| ENSSSCG00000023133 | 15 | 83872742 | 84101299 | Average glycogen QTL (290) | 15 | 75797971 | 1.15E+08 |
| ENSSSCG00000023133 | 15 | 83872742 | 84101299 | Drip loss QTL (3790) | 15 | 76249822 | 89579421 |
| ENSSSCG00000023133 | 15 | 83872742 | 84101299 | Off-flavor score QTL (3811) | 15 | 76249822 | 89579421 |
| ENSSSCG00000023133 | 15 | 83872742 | 84101299 | Meat color-L QTL (120) | 15 | 79525740 | 1.09E+08 |
| ENSSSCG00000023133 | 15 | 83872742 | 84101299 | muscle protein percentage QTL (21722) | 15 | 81333144 | 96018295 |
| ENSSSCG00000023133 | 15 | 83872742 | 84101299 | Shear force QTL (38113) | 15 | 15588852 | 1.4E+08 |
| ENSSSCG00000023133 | 15 | 83872742 | 84101299 | Backfat at tenth rib QTL (5725) | 15 | 15588852 | 1.35E+08 |
| ENSSSCG00000023133 | 15 | 83872742 | 84101299 | Meat color OPTO QTL (12094) | 15 | 44959154 | 99971989 |
| ENSSSCG00000023133 | 15 | 83872742 | 84101299 | Conductivity 24 hours postmortem (loin) QTL (12095) | 15 | 44959154 | 99971989 |
| ENSSSCG00000023133 | 15 | 83872742 | 84101299 | Loin muscle area QTL (12097) | 15 | 44959154 | 99971989 |
| ENSSSCG00000023133 | 15 | 83872742 | 84101299 | Total muscle fiber number QTL (2829) | 15 | 44959154 | 99971989 |
| ENSSSCG00000023133 | 15 | 83872742 | 84101299 | Percentage angular fibers QTL (2830) | 15 | 44959154 | 99971989 |
| ENSSSCG00000023133 | 15 | 83872742 | 84101299 | Diameter of giant fibers QTL (2831) | 15 | 44959154 | 99971989 |
| ENSSSCG00000023133 | 15 | 83872742 | 84101299 | Diameter of type I muscle fibers QTL (2832) | 15 | 44959154 | 99971989 |
| ENSSSCG00000023133 | 15 | 83872742 | 84101299 | Diameter of type IIa muscle fibers QTL (2833) | 15 | 44959154 | 99971989 |
| ENSSSCG00000023133 | 15 | 83872742 | 84101299 | Average backfat thickness QTL (5724) | 15 | 44959154 | 99971989 |
| ENSSSCG00000023133 | 15 | 83872742 | 84101299 | Backfat at tenth rib QTL (5992) | 15 | 44959154 | 99971989 |
| ENSSSCG00000023133 | 15 | 83872742 | 84101299 | Creatinine level QTL (15081) | 15 | 46314707 | 1.4E+08 |
| ENSSSCG00000023133 | 15 | 83872742 | 84101299 | Backfat at first rib QTL (3975) | 15 | 56470053 | 1.35E+08 |
| ENSSSCG00000023133 | 15 | 83872742 | 84101299 | Shoulder subcutaneous fat thickness QTL (21270) | 15 | 60999858 | 86190540 |
| ENSSSCG00000023133 | 15 | 83872742 | 84101299 | pH 24 hr post-mortem (loin) QTL (293) | 15 | 63259112 | 1.35E+08 |
| ENSSSCG00000023133 | 15 | 83872742 | 84101299 | pH 24 hr post-mortem (loin) QTL (122) | 15 | 63259112 | 1.28E+08 |
| ENSSSCG00000023133 | 15 | 83872742 | 84101299 | Skin thickness QTL (31812) | 15 | 65179477 | 98051623 |
| ENSSSCG00000023133 | 15 | 83872742 | 84101299 | Arachidic acid content QTL (156960) | 15 | 65405403 | 1.34E+08 |
| ENSSSCG00000016061 | 15 | 95874411 | 96066991 | Abdominal fat weight QTL (21269) | 15 | 72860941 | 1.27E+08 |
| ENSSSCG00000016061 | 15 | 95874411 | 96066991 | Average daily gain QTL (16857) | 15 | 73538717 | 1.15E+08 |
| ENSSSCG00000016061 | 15 | 95874411 | 96066991 | Average daily gain QTL (16892) | 15 | 73538717 | 1.15E+08 |
| ENSSSCG00000016061 | 15 | 95874411 | 96066991 | Meat color-a QTL (237) | 15 | 73538717 | 1.01E+08 |
| ENSSSCG00000016061 | 15 | 95874411 | 96066991 | Intramuscular fat content QTL (4044) | 15 | 75323528 | 98424400 |
| ENSSSCG00000016061 | 15 | 95874411 | 96066991 | Average glycogen QTL (290) | 15 | 75797971 | 1.15E+08 |
| ENSSSCG00000016061 | 15 | 95874411 | 96066991 | Meat color-L QTL (120) | 15 | 79525740 | 1.09E+08 |
| ENSSSCG00000016061 | 15 | 95874411 | 96066991 | muscle protein percentage QTL (21722) | 15 | 81333144 | 96018295 |
| ENSSSCG00000016061 | 15 | 95874411 | 96066991 | Shear force QTL (3296) | 15 | 89579421 | 1.15E+08 |
| ENSSSCG00000016061 | 15 | 95874411 | 96066991 | Cooking loss QTL (3314) | 15 | 89579421 | 1.15E+08 |
| ENSSSCG00000016061 | 15 | 95874411 | 96066991 | Backfat at tenth rib QTL (3789) | 15 | 89579421 | 1.15E+08 |
| ENSSSCG00000016061 | 15 | 95874411 | 96066991 | Average glycolytic potential QTL (291) | 15 | 89579421 | 1.09E+08 |
| ENSSSCG00000016061 | 15 | 95874411 | 96066991 | Drip loss QTL (3081) | 15 | 89579421 | 1.01E+08 |
| ENSSSCG00000016061 | 15 | 95874411 | 96066991 | Backfat at tenth rib QTL (16939) | 15 | 89579421 | 1.01E+08 |
| ENSSSCG00000016061 | 15 | 95874411 | 96066991 | Meat color score QTL (21410) | 15 | 89579421 | 1.01E+08 |
| ENSSSCG00000016061 | 15 | 95874411 | 96066991 | Meat color L* QTL (21411) | 15 | 89579421 | 1.01E+08 |
| ENSSSCG00000016061 | 15 | 95874411 | 96066991 | Muscle moisture percentage QTL (21413) | 15 | 89579421 | 1.01E+08 |
| ENSSSCG00000016061 | 15 | 95874411 | 96066991 | Shear force QTL (21414) | 15 | 89579421 | 1.01E+08 |
| ENSSSCG00000016061 | 15 | 95874411 | 96066991 | muscle protein percentage QTL (21416) | 15 | 89579421 | 1.01E+08 |
| ENSSSCG00000016061 | 15 | 95874411 | 96066991 | Meat color a* QTL (21418) | 15 | 89579421 | 1.01E+08 |
| ENSSSCG00000016061 | 15 | 95874411 | 96066991 | pH 24 hr post-mortem (loin) QTL (21419) | 15 | 89579421 | 1.01E+08 |
| ENSSSCG00000016061 | 15 | 95874411 | 96066991 | Loin muscle depth QTL (4328) | 15 | 89579421 | 1.01E+08 |
| ENSSSCG00000016061 | 15 | 95874411 | 96066991 | Cooking loss QTL (4329) | 15 | 89579421 | 1.01E+08 |
| ENSSSCG00000016061 | 15 | 95874411 | 96066991 | 1h-48h pH decline QTL (4330) | 15 | 89579421 | 1.01E+08 |
| ENSSSCG00000016061 | 15 | 95874411 | 96066991 | Average lactate QTL (3826) | 15 | 89579421 | 99971989 |
| ENSSSCG00000016061 | 15 | 95874411 | 96066991 | Meat color-L QTL (2960) | 15 | 89579421 | 97486810 |
| ENSSSCG00000016061 | 15 | 95874411 | 96066991 | Meat color-L QTL (2961) | 15 | 89579421 | 97486810 |
| ENSSSCG00000016061 | 15 | 95874411 | 96066991 | pH 48 hr post-mortem (loin) QTL (2965) | 15 | 89579421 | 97486810 |
| ENSSSCG00000016061 | 15 | 95874411 | 96066991 | Average glycogen QTL (2966) | 15 | 89579421 | 97486810 |
| ENSSSCG00000016061 | 15 | 95874411 | 96066991 | Average glycolytic potential QTL (125) | 15 | 90596085 | 1.07E+08 |
| ENSSSCG00000016061 | 15 | 95874411 | 96066991 | Meat color score QTL (21855) | 15 | 91612749 | 1.15E+08 |
| ENSSSCG00000016061 | 15 | 95874411 | 96066991 | pH 24 hr post mortem (ham) QTL (292) | 15 | 91612749 | 1.07E+08 |
| ENSSSCG00000016061 | 15 | 95874411 | 96066991 | Shear force QTL (38113) | 15 | 15588852 | 1.4E+08 |
| ENSSSCG00000016061 | 15 | 95874411 | 96066991 | Backfat at tenth rib QTL (5725) | 15 | 15588852 | 1.35E+08 |
| ENSSSCG00000016061 | 15 | 95874411 | 96066991 | Meat color OPTO QTL (12094) | 15 | 44959154 | 99971989 |
| ENSSSCG00000016061 | 15 | 95874411 | 96066991 | Conductivity 24 hours postmortem (loin) QTL (12095) | 15 | 44959154 | 99971989 |
| ENSSSCG00000016061 | 15 | 95874411 | 96066991 | Loin muscle area QTL (12097) | 15 | 44959154 | 99971989 |
| ENSSSCG00000016061 | 15 | 95874411 | 96066991 | Total muscle fiber number QTL (2829) | 15 | 44959154 | 99971989 |
| ENSSSCG00000016061 | 15 | 95874411 | 96066991 | Percentage angular fibers QTL (2830) | 15 | 44959154 | 99971989 |
| ENSSSCG00000016061 | 15 | 95874411 | 96066991 | Diameter of giant fibers QTL (2831) | 15 | 44959154 | 99971989 |
| ENSSSCG00000016061 | 15 | 95874411 | 96066991 | Diameter of type I muscle fibers QTL (2832) | 15 | 44959154 | 99971989 |
| ENSSSCG00000016061 | 15 | 95874411 | 96066991 | Diameter of type IIa muscle fibers QTL (2833) | 15 | 44959154 | 99971989 |
| ENSSSCG00000016061 | 15 | 95874411 | 96066991 | Average backfat thickness QTL (5724) | 15 | 44959154 | 99971989 |
| ENSSSCG00000016061 | 15 | 95874411 | 96066991 | Backfat at tenth rib QTL (5992) | 15 | 44959154 | 99971989 |
| ENSSSCG00000016061 | 15 | 95874411 | 96066991 | Creatinine level QTL (15081) | 15 | 46314707 | 1.4E+08 |
| ENSSSCG00000016061 | 15 | 95874411 | 96066991 | Backfat at first rib QTL (3975) | 15 | 56470053 | 1.35E+08 |
| ENSSSCG00000016061 | 15 | 95874411 | 96066991 | pH 24 hr post-mortem (loin) QTL (293) | 15 | 63259112 | 1.35E+08 |
| ENSSSCG00000016061 | 15 | 95874411 | 96066991 | pH 24 hr post-mortem (loin) QTL (122) | 15 | 63259112 | 1.28E+08 |
| ENSSSCG00000016061 | 15 | 95874411 | 96066991 | Skin thickness QTL (31812) | 15 | 65179477 | 98051623 |
| ENSSSCG00000016061 | 15 | 95874411 | 96066991 | Arachidic acid content QTL (156960) | 15 | 65405403 | 1.34E+08 |
| ENSSSCG00000026940 | 15 | 1.05E+08 | 1.05E+08 | Abdominal fat weight QTL (21269) | 15 | 72860941 | 1.27E+08 |
| ENSSSCG00000026940 | 15 | 1.05E+08 | 1.05E+08 | Average daily gain QTL (16857) | 15 | 73538717 | 1.15E+08 |
| ENSSSCG00000026940 | 15 | 1.05E+08 | 1.05E+08 | Average daily gain QTL (16892) | 15 | 73538717 | 1.15E+08 |
| ENSSSCG00000026940 | 15 | 1.05E+08 | 1.05E+08 | Average glycogen QTL (290) | 15 | 75797971 | 1.15E+08 |
| ENSSSCG00000026940 | 15 | 1.05E+08 | 1.05E+08 | Meat color-L QTL (120) | 15 | 79525740 | 1.09E+08 |
| ENSSSCG00000026940 | 15 | 1.05E+08 | 1.05E+08 | Shear force QTL (3296) | 15 | 89579421 | 1.15E+08 |
| ENSSSCG00000026940 | 15 | 1.05E+08 | 1.05E+08 | Cooking loss QTL (3314) | 15 | 89579421 | 1.15E+08 |
| ENSSSCG00000026940 | 15 | 1.05E+08 | 1.05E+08 | Backfat at tenth rib QTL (3789) | 15 | 89579421 | 1.15E+08 |
| ENSSSCG00000026940 | 15 | 1.05E+08 | 1.05E+08 | Average glycolytic potential QTL (291) | 15 | 89579421 | 1.09E+08 |
| ENSSSCG00000026940 | 15 | 1.05E+08 | 1.05E+08 | Average glycolytic potential QTL (125) | 15 | 90596085 | 1.07E+08 |
| ENSSSCG00000026940 | 15 | 1.05E+08 | 1.05E+08 | Meat color score QTL (21855) | 15 | 91612749 | 1.15E+08 |
| ENSSSCG00000026940 | 15 | 1.05E+08 | 1.05E+08 | pH 24 hr post mortem (ham) QTL (292) | 15 | 91612749 | 1.07E+08 |
| ENSSSCG00000026940 | 15 | 1.05E+08 | 1.05E+08 | pH 24 hr post mortem (ham) QTL (2962) | 15 | 99971989 | 1.15E+08 |
| ENSSSCG00000026940 | 15 | 1.05E+08 | 1.05E+08 | pH 24 hr post-mortem (loin) QTL (2963) | 15 | 99971989 | 1.15E+08 |
| ENSSSCG00000026940 | 15 | 1.05E+08 | 1.05E+08 | Drip loss QTL (21421) | 15 | 1.01E+08 | 1.15E+08 |
| ENSSSCG00000026940 | 15 | 1.05E+08 | 1.05E+08 | Cooking yield QTL (21438) | 15 | 1.01E+08 | 1.15E+08 |
| ENSSSCG00000026940 | 15 | 1.05E+08 | 1.05E+08 | Connective tissue amount QTL (21442) | 15 | 1.01E+08 | 1.15E+08 |
| ENSSSCG00000026940 | 15 | 1.05E+08 | 1.05E+08 | Shear force QTL (38113) | 15 | 15588852 | 1.4E+08 |
| ENSSSCG00000026940 | 15 | 1.05E+08 | 1.05E+08 | Backfat at tenth rib QTL (5725) | 15 | 15588852 | 1.35E+08 |
| ENSSSCG00000026940 | 15 | 1.05E+08 | 1.05E+08 | Creatinine level QTL (15081) | 15 | 46314707 | 1.4E+08 |
| ENSSSCG00000026940 | 15 | 1.05E+08 | 1.05E+08 | Backfat at first rib QTL (3975) | 15 | 56470053 | 1.35E+08 |
| ENSSSCG00000026940 | 15 | 1.05E+08 | 1.05E+08 | pH 24 hr post-mortem (loin) QTL (293) | 15 | 63259112 | 1.35E+08 |
| ENSSSCG00000026940 | 15 | 1.05E+08 | 1.05E+08 | pH 24 hr post-mortem (loin) QTL (122) | 15 | 63259112 | 1.28E+08 |
| ENSSSCG00000026940 | 15 | 1.05E+08 | 1.05E+08 | Arachidic acid content QTL (156960) | 15 | 65405403 | 1.34E+08 |
| ENSSSCG00000026940 | 15 | 1.05E+08 | 1.05E+08 | Gadoleic acid content QTL (156961) | 15 | 99971989 | 1.4E+08 |
| ENSSSCG00000026940 | 15 | 1.05E+08 | 1.05E+08 | Meat color b* QTL (21845) | 15 | 99971989 | 1.38E+08 |
| ENSSSCG00000026940 | 15 | 1.05E+08 | 1.05E+08 | Average daily gain QTL (5951) | 15 | 99971989 | 1.35E+08 |
| ENSSSCG00000026940 | 15 | 1.05E+08 | 1.05E+08 | PH for Longissmus dorsi QTL (6012) | 15 | 99971989 | 1.35E+08 |
| ENSSSCG00000026940 | 15 | 1.05E+08 | 1.05E+08 | Loin muscle area QTL (5726) | 15 | 99971989 | 1.35E+08 |
| ENSSSCG00000026940 | 15 | 1.05E+08 | 1.05E+08 | Toll-like receptor 2 level QTL (17617) | 15 | 99971989 | 1.35E+08 |
| ENSSSCG00000026940 | 15 | 1.05E+08 | 1.05E+08 | Backfat at last rib QTL (17786) | 15 | 99971989 | 1.35E+08 |
| ENSSSCG00000022225 | 15 | 1.06E+08 | 1.06E+08 | Abdominal fat weight QTL (21269) | 15 | 72860941 | 1.27E+08 |
| ENSSSCG00000022225 | 15 | 1.06E+08 | 1.06E+08 | Average daily gain QTL (16857) | 15 | 73538717 | 1.15E+08 |
| ENSSSCG00000022225 | 15 | 1.06E+08 | 1.06E+08 | Average daily gain QTL (16892) | 15 | 73538717 | 1.15E+08 |
| ENSSSCG00000022225 | 15 | 1.06E+08 | 1.06E+08 | Average glycogen QTL (290) | 15 | 75797971 | 1.15E+08 |
| ENSSSCG00000022225 | 15 | 1.06E+08 | 1.06E+08 | Meat color-L QTL (120) | 15 | 79525740 | 1.09E+08 |
| ENSSSCG00000022225 | 15 | 1.06E+08 | 1.06E+08 | Shear force QTL (3296) | 15 | 89579421 | 1.15E+08 |
| ENSSSCG00000022225 | 15 | 1.06E+08 | 1.06E+08 | Cooking loss QTL (3314) | 15 | 89579421 | 1.15E+08 |
| ENSSSCG00000022225 | 15 | 1.06E+08 | 1.06E+08 | Backfat at tenth rib QTL (3789) | 15 | 89579421 | 1.15E+08 |
| ENSSSCG00000022225 | 15 | 1.06E+08 | 1.06E+08 | Average glycolytic potential QTL (291) | 15 | 89579421 | 1.09E+08 |
| ENSSSCG00000022225 | 15 | 1.06E+08 | 1.06E+08 | Average glycolytic potential QTL (125) | 15 | 90596085 | 1.07E+08 |
| ENSSSCG00000022225 | 15 | 1.06E+08 | 1.06E+08 | Meat color score QTL (21855) | 15 | 91612749 | 1.15E+08 |
| ENSSSCG00000022225 | 15 | 1.06E+08 | 1.06E+08 | pH 24 hr post mortem (ham) QTL (292) | 15 | 91612749 | 1.07E+08 |
| ENSSSCG00000022225 | 15 | 1.06E+08 | 1.06E+08 | pH 24 hr post mortem (ham) QTL (2962) | 15 | 99971989 | 1.15E+08 |
| ENSSSCG00000022225 | 15 | 1.06E+08 | 1.06E+08 | pH 24 hr post-mortem (loin) QTL (2963) | 15 | 99971989 | 1.15E+08 |
| ENSSSCG00000022225 | 15 | 1.06E+08 | 1.06E+08 | Drip loss QTL (21421) | 15 | 1.01E+08 | 1.15E+08 |
| ENSSSCG00000022225 | 15 | 1.06E+08 | 1.06E+08 | Cooking yield QTL (21438) | 15 | 1.01E+08 | 1.15E+08 |
| ENSSSCG00000022225 | 15 | 1.06E+08 | 1.06E+08 | Connective tissue amount QTL (21442) | 15 | 1.01E+08 | 1.15E+08 |
| ENSSSCG00000022225 | 15 | 1.06E+08 | 1.06E+08 | Shear force QTL (38113) | 15 | 15588852 | 1.4E+08 |
| ENSSSCG00000022225 | 15 | 1.06E+08 | 1.06E+08 | Backfat at tenth rib QTL (5725) | 15 | 15588852 | 1.35E+08 |
| ENSSSCG00000022225 | 15 | 1.06E+08 | 1.06E+08 | Creatinine level QTL (15081) | 15 | 46314707 | 1.4E+08 |
| ENSSSCG00000022225 | 15 | 1.06E+08 | 1.06E+08 | Backfat at first rib QTL (3975) | 15 | 56470053 | 1.35E+08 |
| ENSSSCG00000022225 | 15 | 1.06E+08 | 1.06E+08 | pH 24 hr post-mortem (loin) QTL (293) | 15 | 63259112 | 1.35E+08 |
| ENSSSCG00000022225 | 15 | 1.06E+08 | 1.06E+08 | pH 24 hr post-mortem (loin) QTL (122) | 15 | 63259112 | 1.28E+08 |
| ENSSSCG00000022225 | 15 | 1.06E+08 | 1.06E+08 | Arachidic acid content QTL (156960) | 15 | 65405403 | 1.34E+08 |
| ENSSSCG00000022225 | 15 | 1.06E+08 | 1.06E+08 | Gadoleic acid content QTL (156961) | 15 | 99971989 | 1.4E+08 |
| ENSSSCG00000022225 | 15 | 1.06E+08 | 1.06E+08 | Meat color b* QTL (21845) | 15 | 99971989 | 1.38E+08 |
| ENSSSCG00000022225 | 15 | 1.06E+08 | 1.06E+08 | Average daily gain QTL (5951) | 15 | 99971989 | 1.35E+08 |
| ENSSSCG00000022225 | 15 | 1.06E+08 | 1.06E+08 | PH for Longissmus dorsi QTL (6012) | 15 | 99971989 | 1.35E+08 |
| ENSSSCG00000022225 | 15 | 1.06E+08 | 1.06E+08 | Loin muscle area QTL (5726) | 15 | 99971989 | 1.35E+08 |
| ENSSSCG00000022225 | 15 | 1.06E+08 | 1.06E+08 | Toll-like receptor 2 level QTL (17617) | 15 | 99971989 | 1.35E+08 |
| ENSSSCG00000022225 | 15 | 1.06E+08 | 1.06E+08 | Backfat at last rib QTL (17786) | 15 | 99971989 | 1.35E+08 |
| ENSSSCG00000016113 | 15 | 1.06E+08 | 1.06E+08 | Abdominal fat weight QTL (21269) | 15 | 72860941 | 1.27E+08 |
| ENSSSCG00000016113 | 15 | 1.06E+08 | 1.06E+08 | Average daily gain QTL (16857) | 15 | 73538717 | 1.15E+08 |
| ENSSSCG00000016113 | 15 | 1.06E+08 | 1.06E+08 | Average daily gain QTL (16892) | 15 | 73538717 | 1.15E+08 |
| ENSSSCG00000016113 | 15 | 1.06E+08 | 1.06E+08 | Average glycogen QTL (290) | 15 | 75797971 | 1.15E+08 |
| ENSSSCG00000016113 | 15 | 1.06E+08 | 1.06E+08 | Meat color-L QTL (120) | 15 | 79525740 | 1.09E+08 |
| ENSSSCG00000016113 | 15 | 1.06E+08 | 1.06E+08 | Shear force QTL (3296) | 15 | 89579421 | 1.15E+08 |
| ENSSSCG00000016113 | 15 | 1.06E+08 | 1.06E+08 | Cooking loss QTL (3314) | 15 | 89579421 | 1.15E+08 |
| ENSSSCG00000016113 | 15 | 1.06E+08 | 1.06E+08 | Backfat at tenth rib QTL (3789) | 15 | 89579421 | 1.15E+08 |
| ENSSSCG00000016113 | 15 | 1.06E+08 | 1.06E+08 | Average glycolytic potential QTL (291) | 15 | 89579421 | 1.09E+08 |
| ENSSSCG00000016113 | 15 | 1.06E+08 | 1.06E+08 | Average glycolytic potential QTL (125) | 15 | 90596085 | 1.07E+08 |
| ENSSSCG00000016113 | 15 | 1.06E+08 | 1.06E+08 | Meat color score QTL (21855) | 15 | 91612749 | 1.15E+08 |
| ENSSSCG00000016113 | 15 | 1.06E+08 | 1.06E+08 | pH 24 hr post mortem (ham) QTL (292) | 15 | 91612749 | 1.07E+08 |
| ENSSSCG00000016113 | 15 | 1.06E+08 | 1.06E+08 | pH 24 hr post mortem (ham) QTL (2962) | 15 | 99971989 | 1.15E+08 |
| ENSSSCG00000016113 | 15 | 1.06E+08 | 1.06E+08 | pH 24 hr post-mortem (loin) QTL (2963) | 15 | 99971989 | 1.15E+08 |
| ENSSSCG00000016113 | 15 | 1.06E+08 | 1.06E+08 | Drip loss QTL (21421) | 15 | 1.01E+08 | 1.15E+08 |
| ENSSSCG00000016113 | 15 | 1.06E+08 | 1.06E+08 | Cooking yield QTL (21438) | 15 | 1.01E+08 | 1.15E+08 |
| ENSSSCG00000016113 | 15 | 1.06E+08 | 1.06E+08 | Connective tissue amount QTL (21442) | 15 | 1.01E+08 | 1.15E+08 |
| ENSSSCG00000016113 | 15 | 1.06E+08 | 1.06E+08 | Shear force QTL (38113) | 15 | 15588852 | 1.4E+08 |
| ENSSSCG00000016113 | 15 | 1.06E+08 | 1.06E+08 | Backfat at tenth rib QTL (5725) | 15 | 15588852 | 1.35E+08 |
| ENSSSCG00000016113 | 15 | 1.06E+08 | 1.06E+08 | Creatinine level QTL (15081) | 15 | 46314707 | 1.4E+08 |
| ENSSSCG00000016113 | 15 | 1.06E+08 | 1.06E+08 | Backfat at first rib QTL (3975) | 15 | 56470053 | 1.35E+08 |
| ENSSSCG00000016113 | 15 | 1.06E+08 | 1.06E+08 | pH 24 hr post-mortem (loin) QTL (293) | 15 | 63259112 | 1.35E+08 |
| ENSSSCG00000016113 | 15 | 1.06E+08 | 1.06E+08 | pH 24 hr post-mortem (loin) QTL (122) | 15 | 63259112 | 1.28E+08 |
| ENSSSCG00000016113 | 15 | 1.06E+08 | 1.06E+08 | Arachidic acid content QTL (156960) | 15 | 65405403 | 1.34E+08 |
| ENSSSCG00000016113 | 15 | 1.06E+08 | 1.06E+08 | Gadoleic acid content QTL (156961) | 15 | 99971989 | 1.4E+08 |
| ENSSSCG00000016113 | 15 | 1.06E+08 | 1.06E+08 | Meat color b* QTL (21845) | 15 | 99971989 | 1.38E+08 |
| ENSSSCG00000016113 | 15 | 1.06E+08 | 1.06E+08 | Average daily gain QTL (5951) | 15 | 99971989 | 1.35E+08 |
| ENSSSCG00000016113 | 15 | 1.06E+08 | 1.06E+08 | PH for Longissmus dorsi QTL (6012) | 15 | 99971989 | 1.35E+08 |
| ENSSSCG00000016113 | 15 | 1.06E+08 | 1.06E+08 | Loin muscle area QTL (5726) | 15 | 99971989 | 1.35E+08 |
| ENSSSCG00000016113 | 15 | 1.06E+08 | 1.06E+08 | Toll-like receptor 2 level QTL (17617) | 15 | 99971989 | 1.35E+08 |
| ENSSSCG00000016113 | 15 | 1.06E+08 | 1.06E+08 | Backfat at last rib QTL (17786) | 15 | 99971989 | 1.35E+08 |
| ENSSSCG00000022830 | 15 | 1.13E+08 | 1.13E+08 | Abdominal fat weight QTL (21269) | 15 | 72860941 | 1.27E+08 |
| ENSSSCG00000022830 | 15 | 1.13E+08 | 1.13E+08 | Average daily gain QTL (16857) | 15 | 73538717 | 1.15E+08 |
| ENSSSCG00000022830 | 15 | 1.13E+08 | 1.13E+08 | Average daily gain QTL (16892) | 15 | 73538717 | 1.15E+08 |
| ENSSSCG00000022830 | 15 | 1.13E+08 | 1.13E+08 | Average glycogen QTL (290) | 15 | 75797971 | 1.15E+08 |
| ENSSSCG00000022830 | 15 | 1.13E+08 | 1.13E+08 | Shear force QTL (3296) | 15 | 89579421 | 1.15E+08 |
| ENSSSCG00000022830 | 15 | 1.13E+08 | 1.13E+08 | Cooking loss QTL (3314) | 15 | 89579421 | 1.15E+08 |
| ENSSSCG00000022830 | 15 | 1.13E+08 | 1.13E+08 | Backfat at tenth rib QTL (3789) | 15 | 89579421 | 1.15E+08 |
| ENSSSCG00000022830 | 15 | 1.13E+08 | 1.13E+08 | Meat color score QTL (21855) | 15 | 91612749 | 1.15E+08 |
| ENSSSCG00000022830 | 15 | 1.13E+08 | 1.13E+08 | pH 24 hr post mortem (ham) QTL (2962) | 15 | 99971989 | 1.15E+08 |
| ENSSSCG00000022830 | 15 | 1.13E+08 | 1.13E+08 | pH 24 hr post-mortem (loin) QTL (2963) | 15 | 99971989 | 1.15E+08 |
| ENSSSCG00000022830 | 15 | 1.13E+08 | 1.13E+08 | Drip loss QTL (21421) | 15 | 1.01E+08 | 1.15E+08 |
| ENSSSCG00000022830 | 15 | 1.13E+08 | 1.13E+08 | Cooking yield QTL (21438) | 15 | 1.01E+08 | 1.15E+08 |
| ENSSSCG00000022830 | 15 | 1.13E+08 | 1.13E+08 | Connective tissue amount QTL (21442) | 15 | 1.01E+08 | 1.15E+08 |
| ENSSSCG00000022830 | 15 | 1.13E+08 | 1.13E+08 | Shear force QTL (38113) | 15 | 15588852 | 1.4E+08 |
| ENSSSCG00000022830 | 15 | 1.13E+08 | 1.13E+08 | Backfat at tenth rib QTL (5725) | 15 | 15588852 | 1.35E+08 |
| ENSSSCG00000022830 | 15 | 1.13E+08 | 1.13E+08 | Creatinine level QTL (15081) | 15 | 46314707 | 1.4E+08 |
| ENSSSCG00000022830 | 15 | 1.13E+08 | 1.13E+08 | Backfat at first rib QTL (3975) | 15 | 56470053 | 1.35E+08 |
| ENSSSCG00000022830 | 15 | 1.13E+08 | 1.13E+08 | pH 24 hr post-mortem (loin) QTL (293) | 15 | 63259112 | 1.35E+08 |
| ENSSSCG00000022830 | 15 | 1.13E+08 | 1.13E+08 | pH 24 hr post-mortem (loin) QTL (122) | 15 | 63259112 | 1.28E+08 |
| ENSSSCG00000022830 | 15 | 1.13E+08 | 1.13E+08 | Arachidic acid content QTL (156960) | 15 | 65405403 | 1.34E+08 |
| ENSSSCG00000022830 | 15 | 1.13E+08 | 1.13E+08 | Gadoleic acid content QTL (156961) | 15 | 99971989 | 1.4E+08 |
| ENSSSCG00000022830 | 15 | 1.13E+08 | 1.13E+08 | Meat color b* QTL (21845) | 15 | 99971989 | 1.38E+08 |
| ENSSSCG00000022830 | 15 | 1.13E+08 | 1.13E+08 | Average daily gain QTL (5951) | 15 | 99971989 | 1.35E+08 |
| ENSSSCG00000022830 | 15 | 1.13E+08 | 1.13E+08 | PH for Longissmus dorsi QTL (6012) | 15 | 99971989 | 1.35E+08 |
| ENSSSCG00000022830 | 15 | 1.13E+08 | 1.13E+08 | Loin muscle area QTL (5726) | 15 | 99971989 | 1.35E+08 |
| ENSSSCG00000022830 | 15 | 1.13E+08 | 1.13E+08 | Toll-like receptor 2 level QTL (17617) | 15 | 99971989 | 1.35E+08 |
| ENSSSCG00000022830 | 15 | 1.13E+08 | 1.13E+08 | Backfat at last rib QTL (17786) | 15 | 99971989 | 1.35E+08 |
| ENSSSCG00000024373 | 15 | 1.31E+08 | 1.31E+08 | Shear force QTL (38113) | 15 | 15588852 | 1.4E+08 |
| ENSSSCG00000024373 | 15 | 1.31E+08 | 1.31E+08 | Backfat at tenth rib QTL (5725) | 15 | 15588852 | 1.35E+08 |
| ENSSSCG00000024373 | 15 | 1.31E+08 | 1.31E+08 | Creatinine level QTL (15081) | 15 | 46314707 | 1.4E+08 |
| ENSSSCG00000024373 | 15 | 1.31E+08 | 1.31E+08 | Backfat at first rib QTL (3975) | 15 | 56470053 | 1.35E+08 |
| ENSSSCG00000024373 | 15 | 1.31E+08 | 1.31E+08 | pH 24 hr post-mortem (loin) QTL (293) | 15 | 63259112 | 1.35E+08 |
| ENSSSCG00000024373 | 15 | 1.31E+08 | 1.31E+08 | Arachidic acid content QTL (156960) | 15 | 65405403 | 1.34E+08 |
| ENSSSCG00000024373 | 15 | 1.31E+08 | 1.31E+08 | Gadoleic acid content QTL (156961) | 15 | 99971989 | 1.4E+08 |
| ENSSSCG00000024373 | 15 | 1.31E+08 | 1.31E+08 | Meat color b* QTL (21845) | 15 | 99971989 | 1.38E+08 |
| ENSSSCG00000024373 | 15 | 1.31E+08 | 1.31E+08 | Average daily gain QTL (5951) | 15 | 99971989 | 1.35E+08 |
| ENSSSCG00000024373 | 15 | 1.31E+08 | 1.31E+08 | PH for Longissmus dorsi QTL (6012) | 15 | 99971989 | 1.35E+08 |
| ENSSSCG00000024373 | 15 | 1.31E+08 | 1.31E+08 | Loin muscle area QTL (5726) | 15 | 99971989 | 1.35E+08 |
| ENSSSCG00000024373 | 15 | 1.31E+08 | 1.31E+08 | Toll-like receptor 2 level QTL (17617) | 15 | 99971989 | 1.35E+08 |
| ENSSSCG00000024373 | 15 | 1.31E+08 | 1.31E+08 | Backfat at last rib QTL (17786) | 15 | 99971989 | 1.35E+08 |
| ENSSSCG00000024373 | 15 | 1.31E+08 | 1.31E+08 | Palmitic acid content QTL (15886) | 15 | 1.15E+08 | 1.35E+08 |
| ENSSSCG00000024373 | 15 | 1.31E+08 | 1.31E+08 | Backfat at tenth rib QTL (16940) | 15 | 1.15E+08 | 1.35E+08 |
| ENSSSCG00000024373 | 15 | 1.31E+08 | 1.31E+08 | Monounsaturated fatty acid content QTL (156962) | 15 | 1.16E+08 | 1.4E+08 |
| ENSSSCG00000024373 | 15 | 1.31E+08 | 1.31E+08 | Calcium level QTL (15080) | 15 | 1.16E+08 | 1.4E+08 |
| ENSSSCG00000024373 | 15 | 1.31E+08 | 1.31E+08 | Meat color-L QTL (118) | 15 | 1.2E+08 | 1.35E+08 |
| ENSSSCG00000016306 | 15 | 1.34E+08 | 1.34E+08 | Shear force QTL (38113) | 15 | 15588852 | 1.4E+08 |
| ENSSSCG00000016306 | 15 | 1.34E+08 | 1.34E+08 | Backfat at tenth rib QTL (5725) | 15 | 15588852 | 1.35E+08 |
| ENSSSCG00000016306 | 15 | 1.34E+08 | 1.34E+08 | Creatinine level QTL (15081) | 15 | 46314707 | 1.4E+08 |
| ENSSSCG00000016306 | 15 | 1.34E+08 | 1.34E+08 | Backfat at first rib QTL (3975) | 15 | 56470053 | 1.35E+08 |
| ENSSSCG00000016306 | 15 | 1.34E+08 | 1.34E+08 | pH 24 hr post-mortem (loin) QTL (293) | 15 | 63259112 | 1.35E+08 |
| ENSSSCG00000016306 | 15 | 1.34E+08 | 1.34E+08 | Arachidic acid content QTL (156960) | 15 | 65405403 | 1.34E+08 |
| ENSSSCG00000016306 | 15 | 1.34E+08 | 1.34E+08 | Gadoleic acid content QTL (156961) | 15 | 99971989 | 1.4E+08 |
| ENSSSCG00000016306 | 15 | 1.34E+08 | 1.34E+08 | Meat color b* QTL (21845) | 15 | 99971989 | 1.38E+08 |
| ENSSSCG00000016306 | 15 | 1.34E+08 | 1.34E+08 | Average daily gain QTL (5951) | 15 | 99971989 | 1.35E+08 |
| ENSSSCG00000016306 | 15 | 1.34E+08 | 1.34E+08 | PH for Longissmus dorsi QTL (6012) | 15 | 99971989 | 1.35E+08 |
| ENSSSCG00000016306 | 15 | 1.34E+08 | 1.34E+08 | Loin muscle area QTL (5726) | 15 | 99971989 | 1.35E+08 |
| ENSSSCG00000016306 | 15 | 1.34E+08 | 1.34E+08 | Toll-like receptor 2 level QTL (17617) | 15 | 99971989 | 1.35E+08 |
| ENSSSCG00000016306 | 15 | 1.34E+08 | 1.34E+08 | Backfat at last rib QTL (17786) | 15 | 99971989 | 1.35E+08 |
| ENSSSCG00000016306 | 15 | 1.34E+08 | 1.34E+08 | Palmitic acid content QTL (15886) | 15 | 1.15E+08 | 1.35E+08 |
| ENSSSCG00000016306 | 15 | 1.34E+08 | 1.34E+08 | Backfat at tenth rib QTL (16940) | 15 | 1.15E+08 | 1.35E+08 |
| ENSSSCG00000016306 | 15 | 1.34E+08 | 1.34E+08 | Monounsaturated fatty acid content QTL (156962) | 15 | 1.16E+08 | 1.4E+08 |
| ENSSSCG00000016306 | 15 | 1.34E+08 | 1.34E+08 | Calcium level QTL (15080) | 15 | 1.16E+08 | 1.4E+08 |
| ENSSSCG00000016306 | 15 | 1.34E+08 | 1.34E+08 | Meat color-L QTL (118) | 15 | 1.2E+08 | 1.35E+08 |
| ENSSSCG00000016374 | 15 | 1.4E+08 | 1.4E+08 | Shear force QTL (38113) | 15 | 15588852 | 1.4E+08 |
| ENSSSCG00000016374 | 15 | 1.4E+08 | 1.4E+08 | Creatinine level QTL (15081) | 15 | 46314707 | 1.4E+08 |
| ENSSSCG00000016374 | 15 | 1.4E+08 | 1.4E+08 | Gadoleic acid content QTL (156961) | 15 | 99971989 | 1.4E+08 |
| ENSSSCG00000016374 | 15 | 1.4E+08 | 1.4E+08 | Monounsaturated fatty acid content QTL (156962) | 15 | 1.16E+08 | 1.4E+08 |
| ENSSSCG00000016374 | 15 | 1.4E+08 | 1.4E+08 | Calcium level QTL (15080) | 15 | 1.16E+08 | 1.4E+08 |
| ENSSSCG00000031705 | 16 | 18367282 | 18416501 | Shear force QTL (17812) | 16 | 12952342 | 31994858 |
| ENSSSCG00000031705 | 16 | 18367282 | 18416501 | Average daily gain QTL (1179) | 16 | 12952342 | 20843835 |
| ENSSSCG00000031705 | 16 | 18367282 | 18416501 | Meat color b* QTL (38071) | 16 | 12952342 | 79429594 |
| ENSSSCG00000031705 | 16 | 18367282 | 18416501 | Cooking loss QTL (38115) | 16 | 12952342 | 79429594 |
| ENSSSCG00000031705 | 16 | 18367282 | 18416501 | Meat color a* QTL (38116) | 16 | 12952342 | 79429594 |
| ENSSSCG00000031705 | 16 | 18367282 | 18416501 | Oleic acid content QTL (156966) | 16 | 12952342 | 73939860 |
| ENSSSCG00000031705 | 16 | 18367282 | 18416501 | Linoleic acid content QTL (156967) | 16 | 12952342 | 73939860 |
| ENSSSCG00000031705 | 16 | 18367282 | 18416501 | Monounsaturated fatty acid content QTL (156969) | 16 | 12952342 | 73939860 |
| ENSSSCG00000031705 | 16 | 18367282 | 18416501 | Polyunsaturated fatty acid content QTL (156970) | 16 | 12952342 | 73939860 |
| ENSSSCG00000016834 | 16 | 21402113 | 21462909 | Shear force QTL (17812) | 16 | 12952342 | 31994858 |
| ENSSSCG00000016834 | 16 | 21402113 | 21462909 | Total body fat tissue linear QTL (3659) | 16 | 20843835 | 47777844 |
| ENSSSCG00000016834 | 16 | 21402113 | 21462909 | Backfat at last rib QTL (3212) | 16 | 20843835 | 47777844 |
| ENSSSCG00000016834 | 16 | 21402113 | 21462909 | Loin fat percentage QTL (38070) | 16 | 20843835 | 47777844 |
| ENSSSCG00000016834 | 16 | 21402113 | 21462909 | Myristic acid content QTL (156908) | 16 | 20843835 | 47777844 |
| ENSSSCG00000016834 | 16 | 21402113 | 21462909 | Arachidic acid content QTL (156909) | 16 | 20843835 | 47777844 |
| ENSSSCG00000016834 | 16 | 21402113 | 21462909 | Lauric acid content QTL (156963) | 16 | 20843835 | 47777844 |
| ENSSSCG00000016834 | 16 | 21402113 | 21462909 | cis-9-Heptadecenoic acid content QTL (156965) | 16 | 20843835 | 47777844 |
| ENSSSCG00000016834 | 16 | 21402113 | 21462909 | Arachidonic acid content QTL (156968) | 16 | 20843835 | 47777844 |
| ENSSSCG00000016834 | 16 | 21402113 | 21462909 | Loin muscle area QTL (21423) | 16 | 20843835 | 38428140 |
| ENSSSCG00000016834 | 16 | 21402113 | 21462909 | pH 45 minutes post mortem QTL (1185) | 16 | 20843835 | 28477997 |
| ENSSSCG00000016834 | 16 | 21402113 | 21462909 | Meat color b* QTL (38071) | 16 | 12952342 | 79429594 |
| ENSSSCG00000016834 | 16 | 21402113 | 21462909 | Cooking loss QTL (38115) | 16 | 12952342 | 79429594 |
| ENSSSCG00000016834 | 16 | 21402113 | 21462909 | Meat color a* QTL (38116) | 16 | 12952342 | 79429594 |
| ENSSSCG00000016834 | 16 | 21402113 | 21462909 | Oleic acid content QTL (156966) | 16 | 12952342 | 73939860 |
| ENSSSCG00000016834 | 16 | 21402113 | 21462909 | Linoleic acid content QTL (156967) | 16 | 12952342 | 73939860 |
| ENSSSCG00000016834 | 16 | 21402113 | 21462909 | Monounsaturated fatty acid content QTL (156969) | 16 | 12952342 | 73939860 |
| ENSSSCG00000016834 | 16 | 21402113 | 21462909 | Polyunsaturated fatty acid content QTL (156970) | 16 | 12952342 | 73939860 |
| ENSSSCG00000016834 | 16 | 21402113 | 21462909 | Loin muscle area QTL (38114) | 16 | 20843835 | 79429594 |
| ENSSSCG00000016842 | 16 | 22152501 | 22363847 | Average daily gain QTL (170903) | 16 | 22170405 | 22170409 |
| ENSSSCG00000016842 | 16 | 22152501 | 22363847 | Shear force QTL (17812) | 16 | 12952342 | 31994858 |
| ENSSSCG00000016842 | 16 | 22152501 | 22363847 | Total body fat tissue linear QTL (3659) | 16 | 20843835 | 47777844 |
| ENSSSCG00000016842 | 16 | 22152501 | 22363847 | Backfat at last rib QTL (3212) | 16 | 20843835 | 47777844 |
| ENSSSCG00000016842 | 16 | 22152501 | 22363847 | Loin fat percentage QTL (38070) | 16 | 20843835 | 47777844 |
| ENSSSCG00000016842 | 16 | 22152501 | 22363847 | Myristic acid content QTL (156908) | 16 | 20843835 | 47777844 |
| ENSSSCG00000016842 | 16 | 22152501 | 22363847 | Arachidic acid content QTL (156909) | 16 | 20843835 | 47777844 |
| ENSSSCG00000016842 | 16 | 22152501 | 22363847 | Lauric acid content QTL (156963) | 16 | 20843835 | 47777844 |
| ENSSSCG00000016842 | 16 | 22152501 | 22363847 | cis-9-Heptadecenoic acid content QTL (156965) | 16 | 20843835 | 47777844 |
| ENSSSCG00000016842 | 16 | 22152501 | 22363847 | Arachidonic acid content QTL (156968) | 16 | 20843835 | 47777844 |
| ENSSSCG00000016842 | 16 | 22152501 | 22363847 | Loin muscle area QTL (21423) | 16 | 20843835 | 38428140 |
| ENSSSCG00000016842 | 16 | 22152501 | 22363847 | pH 45 minutes post mortem QTL (1185) | 16 | 20843835 | 28477997 |
| ENSSSCG00000016842 | 16 | 22152501 | 22363847 | Meat color b* QTL (38071) | 16 | 12952342 | 79429594 |
| ENSSSCG00000016842 | 16 | 22152501 | 22363847 | Cooking loss QTL (38115) | 16 | 12952342 | 79429594 |
| ENSSSCG00000016842 | 16 | 22152501 | 22363847 | Meat color a* QTL (38116) | 16 | 12952342 | 79429594 |
| ENSSSCG00000016842 | 16 | 22152501 | 22363847 | Oleic acid content QTL (156966) | 16 | 12952342 | 73939860 |
| ENSSSCG00000016842 | 16 | 22152501 | 22363847 | Linoleic acid content QTL (156967) | 16 | 12952342 | 73939860 |
| ENSSSCG00000016842 | 16 | 22152501 | 22363847 | Monounsaturated fatty acid content QTL (156969) | 16 | 12952342 | 73939860 |
| ENSSSCG00000016842 | 16 | 22152501 | 22363847 | Polyunsaturated fatty acid content QTL (156970) | 16 | 12952342 | 73939860 |
| ENSSSCG00000016842 | 16 | 22152501 | 22363847 | Loin muscle area QTL (38114) | 16 | 20843835 | 79429594 |
| ENSSSCG00000016888 | 16 | 32424645 | 32462521 | Body depth QTL (16972) | 16 | 32423743 | 32826896 |
| ENSSSCG00000016888 | 16 | 32424645 | 32462521 | Total body fat tissue linear QTL (3659) | 16 | 20843835 | 47777844 |
| ENSSSCG00000016888 | 16 | 32424645 | 32462521 | Backfat at last rib QTL (3212) | 16 | 20843835 | 47777844 |
| ENSSSCG00000016888 | 16 | 32424645 | 32462521 | Loin fat percentage QTL (38070) | 16 | 20843835 | 47777844 |
| ENSSSCG00000016888 | 16 | 32424645 | 32462521 | Myristic acid content QTL (156908) | 16 | 20843835 | 47777844 |
| ENSSSCG00000016888 | 16 | 32424645 | 32462521 | Arachidic acid content QTL (156909) | 16 | 20843835 | 47777844 |
| ENSSSCG00000016888 | 16 | 32424645 | 32462521 | Lauric acid content QTL (156963) | 16 | 20843835 | 47777844 |
| ENSSSCG00000016888 | 16 | 32424645 | 32462521 | cis-9-Heptadecenoic acid content QTL (156965) | 16 | 20843835 | 47777844 |
| ENSSSCG00000016888 | 16 | 32424645 | 32462521 | Arachidonic acid content QTL (156968) | 16 | 20843835 | 47777844 |
| ENSSSCG00000016888 | 16 | 32424645 | 32462521 | Loin muscle area QTL (21423) | 16 | 20843835 | 38428140 |
| ENSSSCG00000016888 | 16 | 32424645 | 32462521 | Adipocyte diameter QTL (12815) | 16 | 27620226 | 40229459 |
| ENSSSCG00000016888 | 16 | 32424645 | 32462521 | Creatine kinase level QTL (1182) | 16 | 28477997 | 40229459 |
| ENSSSCG00000016888 | 16 | 32424645 | 32462521 | Creatinine level QTL (6370) | 16 | 31994858 | 47777844 |
| ENSSSCG00000016888 | 16 | 32424645 | 32462521 | Meat color b* QTL (38071) | 16 | 12952342 | 79429594 |
| ENSSSCG00000016888 | 16 | 32424645 | 32462521 | Cooking loss QTL (38115) | 16 | 12952342 | 79429594 |
| ENSSSCG00000016888 | 16 | 32424645 | 32462521 | Meat color a* QTL (38116) | 16 | 12952342 | 79429594 |
| ENSSSCG00000016888 | 16 | 32424645 | 32462521 | Oleic acid content QTL (156966) | 16 | 12952342 | 73939860 |
| ENSSSCG00000016888 | 16 | 32424645 | 32462521 | Linoleic acid content QTL (156967) | 16 | 12952342 | 73939860 |
| ENSSSCG00000016888 | 16 | 32424645 | 32462521 | Monounsaturated fatty acid content QTL (156969) | 16 | 12952342 | 73939860 |
| ENSSSCG00000016888 | 16 | 32424645 | 32462521 | Polyunsaturated fatty acid content QTL (156970) | 16 | 12952342 | 73939860 |
| ENSSSCG00000016888 | 16 | 32424645 | 32462521 | Loin muscle area QTL (38114) | 16 | 20843835 | 79429594 |
| ENSSSCG00000016888 | 16 | 32424645 | 32462521 | Conductivity 24 hours post-mortem QTL (1178) | 16 | 28477997 | 79429594 |
| ENSSSCG00000016888 | 16 | 32424645 | 32462521 | Margaric acid content QTL (156964) | 16 | 31994858 | 79429594 |
| ENSSSCG00000025194 | 16 | 40105426 | 40305376 | Arachidic acid to stearic acid ratio QTL (101585) | 16 | 40127480 | 40127484 |
| ENSSSCG00000025194 | 16 | 40105426 | 40305376 | Cis-11-Eicosenoic acid to arachidic acid ratio QTL (218148) | 16 | 40203539 | 40203543 |
| ENSSSCG00000025194 | 16 | 40105426 | 40305376 | Meat color L* QTL (21424) | 16 | 39997861 | 40306659 |
| ENSSSCG00000025194 | 16 | 40105426 | 40305376 | Semimembranosus width (maximum) QTL (3977) | 16 | 37913478 | 40229459 |
| ENSSSCG00000025194 | 16 | 40105426 | 40305376 | Total body fat tissue linear QTL (3659) | 16 | 20843835 | 47777844 |
| ENSSSCG00000025194 | 16 | 40105426 | 40305376 | Backfat at last rib QTL (3212) | 16 | 20843835 | 47777844 |
| ENSSSCG00000025194 | 16 | 40105426 | 40305376 | Loin fat percentage QTL (38070) | 16 | 20843835 | 47777844 |
| ENSSSCG00000025194 | 16 | 40105426 | 40305376 | Myristic acid content QTL (156908) | 16 | 20843835 | 47777844 |
| ENSSSCG00000025194 | 16 | 40105426 | 40305376 | Arachidic acid content QTL (156909) | 16 | 20843835 | 47777844 |
| ENSSSCG00000025194 | 16 | 40105426 | 40305376 | Lauric acid content QTL (156963) | 16 | 20843835 | 47777844 |
| ENSSSCG00000025194 | 16 | 40105426 | 40305376 | cis-9-Heptadecenoic acid content QTL (156965) | 16 | 20843835 | 47777844 |
| ENSSSCG00000025194 | 16 | 40105426 | 40305376 | Arachidonic acid content QTL (156968) | 16 | 20843835 | 47777844 |
| ENSSSCG00000025194 | 16 | 40105426 | 40305376 | Adipocyte diameter QTL (12815) | 16 | 27620226 | 40229459 |
| ENSSSCG00000025194 | 16 | 40105426 | 40305376 | Creatine kinase level QTL (1182) | 16 | 28477997 | 40229459 |
| ENSSSCG00000025194 | 16 | 40105426 | 40305376 | Creatinine level QTL (6370) | 16 | 31994858 | 47777844 |
| ENSSSCG00000025194 | 16 | 40105426 | 40305376 | Meat color a* QTL (21425) | 16 | 38428140 | 47777844 |
| ENSSSCG00000025194 | 16 | 40105426 | 40305376 | Meat color b* QTL (38071) | 16 | 12952342 | 79429594 |
| ENSSSCG00000025194 | 16 | 40105426 | 40305376 | Cooking loss QTL (38115) | 16 | 12952342 | 79429594 |
| ENSSSCG00000025194 | 16 | 40105426 | 40305376 | Meat color a* QTL (38116) | 16 | 12952342 | 79429594 |
| ENSSSCG00000025194 | 16 | 40105426 | 40305376 | Oleic acid content QTL (156966) | 16 | 12952342 | 73939860 |
| ENSSSCG00000025194 | 16 | 40105426 | 40305376 | Linoleic acid content QTL (156967) | 16 | 12952342 | 73939860 |
| ENSSSCG00000025194 | 16 | 40105426 | 40305376 | Monounsaturated fatty acid content QTL (156969) | 16 | 12952342 | 73939860 |
| ENSSSCG00000025194 | 16 | 40105426 | 40305376 | Polyunsaturated fatty acid content QTL (156970) | 16 | 12952342 | 73939860 |
| ENSSSCG00000025194 | 16 | 40105426 | 40305376 | Loin muscle area QTL (38114) | 16 | 20843835 | 79429594 |
| ENSSSCG00000025194 | 16 | 40105426 | 40305376 | Conductivity 24 hours post-mortem QTL (1178) | 16 | 28477997 | 79429594 |
| ENSSSCG00000025194 | 16 | 40105426 | 40305376 | Margaric acid content QTL (156964) | 16 | 31994858 | 79429594 |
| ENSSSCG00000017094 | 16 | 72044869 | 72127864 | Meat color b* QTL (38071) | 16 | 12952342 | 79429594 |
| ENSSSCG00000017094 | 16 | 72044869 | 72127864 | Cooking loss QTL (38115) | 16 | 12952342 | 79429594 |
| ENSSSCG00000017094 | 16 | 72044869 | 72127864 | Meat color a* QTL (38116) | 16 | 12952342 | 79429594 |
| ENSSSCG00000017094 | 16 | 72044869 | 72127864 | Oleic acid content QTL (156966) | 16 | 12952342 | 73939860 |
| ENSSSCG00000017094 | 16 | 72044869 | 72127864 | Linoleic acid content QTL (156967) | 16 | 12952342 | 73939860 |
| ENSSSCG00000017094 | 16 | 72044869 | 72127864 | Monounsaturated fatty acid content QTL (156969) | 16 | 12952342 | 73939860 |
| ENSSSCG00000017094 | 16 | 72044869 | 72127864 | Polyunsaturated fatty acid content QTL (156970) | 16 | 12952342 | 73939860 |
| ENSSSCG00000017094 | 16 | 72044869 | 72127864 | Loin muscle area QTL (38114) | 16 | 20843835 | 79429594 |
| ENSSSCG00000017094 | 16 | 72044869 | 72127864 | Conductivity 24 hours post-mortem QTL (1178) | 16 | 28477997 | 79429594 |
| ENSSSCG00000017094 | 16 | 72044869 | 72127864 | Margaric acid content QTL (156964) | 16 | 31994858 | 79429594 |
| ENSSSCG00000017094 | 16 | 72044869 | 72127864 | Average backfat thickness QTL (5993) | 16 | 40229459 | 79429594 |
| ENSSSCG00000017094 | 16 | 72044869 | 72127864 | Average backfat thickness QTL (5728) | 16 | 40229459 | 79429594 |
| ENSSSCG00000017094 | 16 | 72044869 | 72127864 | Shoulder subcutaneous fat thickness QTL (5729) | 16 | 40229459 | 79429594 |
| ENSSSCG00000017094 | 16 | 72044869 | 72127864 | backfat above muscle dorsi QTL (5730) | 16 | 40229459 | 79429594 |
| ENSSSCG00000017094 | 16 | 72044869 | 72127864 | Interferon-gamma level QTL (12343) | 16 | 40229459 | 79429594 |
| ENSSSCG00000017094 | 16 | 72044869 | 72127864 | pH 24 hr post-mortem (loin) QTL (1184) | 16 | 40229459 | 79429594 |
| ENSSSCG00000017094 | 16 | 72044869 | 72127864 | Percentage giant fibers QTL (2834) | 16 | 40229459 | 79429594 |
| ENSSSCG00000017094 | 16 | 72044869 | 72127864 | HDL cholesterol QTL (17726) | 16 | 40229459 | 79429594 |
| ENSSSCG00000017094 | 16 | 72044869 | 72127864 | Backfat at last rib QTL (17813) | 16 | 47777844 | 79429594 |
| ENSSSCG00000017094 | 16 | 72044869 | 72127864 | Average daily gain QTL (16858) | 16 | 47777844 | 79429594 |
| ENSSSCG00000017094 | 16 | 72044869 | 72127864 | Backfat at tenth rib QTL (3213) | 16 | 47777844 | 79429594 |
| ENSSSCG00000017094 | 16 | 72044869 | 72127864 | body weight (22 weeks) QTL (3214) | 16 | 47777844 | 79429594 |
| ENSSSCG00000017094 | 16 | 72044869 | 72127864 | body weight (19 weeks) QTL (3215) | 16 | 47777844 | 79429594 |
| ENSSSCG00000017094 | 16 | 72044869 | 72127864 | Average daily gain QTL (3216) | 16 | 47777844 | 79429594 |
| ENSSSCG00000017094 | 16 | 72044869 | 72127864 | pH 24 hr post-mortem (loin) QTL (3297) | 16 | 47777844 | 79429594 |
| ENSSSCG00000017094 | 16 | 72044869 | 72127864 | Meat color-L QTL (3828) | 16 | 47777844 | 79429594 |
| ENSSSCG00000017094 | 16 | 72044869 | 72127864 | Muscle moisture percentage QTL (21426) | 16 | 47777844 | 73939860 |
| ENSSSCG00000017094 | 16 | 72044869 | 72127864 | Leptin level QTL (18646) | 16 | 47777844 | 73939860 |
| ENSSSCG00000017094 | 16 | 72044869 | 72127864 | Time spent drinking QTL (5918) | 16 | 47777844 | 73939860 |
| ENSSSCG00000017094 | 16 | 72044869 | 72127864 | Ratio of ham fat weight to half carcass weight QTL (1286) | 16 | 47777844 | 73939860 |
| ENSSSCG00000040013 | 17 | 5329744 | 5507189 | Average daily gain QTL (28911) | 17 | 5471762 | 5471766 |
| ENSSSCG00000007026 | 17 | 11410392 | 11425388 | Average daily gain QTL (1193) | 17 | 11324199 | 20946496 |
| ENSSSCG00000007046 | 17 | 14777683 | 14970100 | Average daily gain QTL (1193) | 17 | 11324199 | 20946496 |
| ENSSSCG00000007046 | 17 | 14777683 | 14970100 | Average backfat thickness QTL (7531) | 17 | 11651488 | 36918199 |
| ENSSSCG00000007046 | 17 | 14777683 | 14970100 | Capric acid content QTL (156971) | 17 | 11651488 | 25135795 |
| ENSSSCG00000007187 | 17 | 33869526 | 33892065 | Average backfat thickness QTL (7531) | 17 | 11651488 | 36918199 |
| ENSSSCG00000007187 | 17 | 33869526 | 33892065 | Potassium level QTL (6381) | 17 | 20946496 | 41500245 |
| ENSSSCG00000007187 | 17 | 33869526 | 33892065 | Body weight (slaughter) QTL (1187) | 17 | 20946496 | 36918199 |
| ENSSSCG00000007187 | 17 | 33869526 | 33892065 | Backfat at tenth rib QTL (1189) | 17 | 20946496 | 36918199 |
| ENSSSCG00000007187 | 17 | 33869526 | 33892065 | muscle protein percentage QTL (3298) | 17 | 26575866 | 41500245 |
| ENSSSCG00000007187 | 17 | 33869526 | 33892065 | Haptoglobin concentration QTL (9657) | 17 | 31812490 | 61530332 |
| ENSSSCG00000007187 | 17 | 33869526 | 33892065 | Triglyceride level QTL (17728) | 17 | 31812490 | 61530332 |
| ENSSSCG00000007187 | 17 | 33869526 | 33892065 | Interferon-gamma level QTL (12344) | 17 | 32316515 | 62034357 |
| ENSSSCG00000007307 | 17 | 39000642 | 39029186 | Potassium level QTL (6381) | 17 | 20946496 | 41500245 |
| ENSSSCG00000007307 | 17 | 39000642 | 39029186 | muscle protein percentage QTL (3298) | 17 | 26575866 | 41500245 |
| ENSSSCG00000007307 | 17 | 39000642 | 39029186 | Haptoglobin concentration QTL (9657) | 17 | 31812490 | 61530332 |
| ENSSSCG00000007307 | 17 | 39000642 | 39029186 | Triglyceride level QTL (17728) | 17 | 31812490 | 61530332 |
| ENSSSCG00000007307 | 17 | 39000642 | 39029186 | Interferon-gamma level QTL (12344) | 17 | 32316515 | 62034357 |
| ENSSSCG00000007307 | 17 | 39000642 | 39029186 | Backfat at last rib QTL (16842) | 17 | 38030981 | 63494066 |
| ENSSSCG00000007307 | 17 | 39000642 | 39029186 | Adipocyte diameter QTL (18026) | 17 | 38030981 | 50337048 |
| ENSSSCG00000007307 | 17 | 39000642 | 39029186 | Carcass length QTL (1186) | 17 | 38685559 | 63494066 |
| ENSSSCG00000007307 | 17 | 39000642 | 39029186 | Backfat at last rib QTL (1191) | 17 | 38685559 | 63494066 |
| ENSSSCG00000007331 | 17 | 40194949 | 40279350 | Potassium level QTL (6381) | 17 | 20946496 | 41500245 |
| ENSSSCG00000007331 | 17 | 40194949 | 40279350 | muscle protein percentage QTL (3298) | 17 | 26575866 | 41500245 |
| ENSSSCG00000007331 | 17 | 40194949 | 40279350 | Haptoglobin concentration QTL (9657) | 17 | 31812490 | 61530332 |
| ENSSSCG00000007331 | 17 | 40194949 | 40279350 | Triglyceride level QTL (17728) | 17 | 31812490 | 61530332 |
| ENSSSCG00000007331 | 17 | 40194949 | 40279350 | Interferon-gamma level QTL (12344) | 17 | 32316515 | 62034357 |
| ENSSSCG00000007331 | 17 | 40194949 | 40279350 | Backfat at last rib QTL (16842) | 17 | 38030981 | 63494066 |
| ENSSSCG00000007331 | 17 | 40194949 | 40279350 | Adipocyte diameter QTL (18026) | 17 | 38030981 | 50337048 |
| ENSSSCG00000007331 | 17 | 40194949 | 40279350 | Carcass length QTL (1186) | 17 | 38685559 | 63494066 |
| ENSSSCG00000007331 | 17 | 40194949 | 40279350 | Backfat at last rib QTL (1191) | 17 | 38685559 | 63494066 |
| ENSSSCG00000007478 | 17 | 52936173 | 53103190 | Blood total protein level QTL (171126) | 17 | 52958669 | 52958673 |
| ENSSSCG00000007478 | 17 | 52936173 | 53103190 | pH 24 hr post-mortem (loin) QTL (21865) | 17 | 50337048 | 58061068 |
| ENSSSCG00000007478 | 17 | 52936173 | 53103190 | Haptoglobin concentration QTL (9657) | 17 | 31812490 | 61530332 |
| ENSSSCG00000007478 | 17 | 52936173 | 53103190 | Triglyceride level QTL (17728) | 17 | 31812490 | 61530332 |
| ENSSSCG00000007478 | 17 | 52936173 | 53103190 | Interferon-gamma level QTL (12344) | 17 | 32316515 | 62034357 |
| ENSSSCG00000007478 | 17 | 52936173 | 53103190 | Backfat at last rib QTL (16842) | 17 | 38030981 | 63494066 |
| ENSSSCG00000007478 | 17 | 52936173 | 53103190 | Carcass length QTL (1186) | 17 | 38685559 | 63494066 |
| ENSSSCG00000007478 | 17 | 52936173 | 53103190 | Backfat at last rib QTL (1191) | 17 | 38685559 | 63494066 |
| ENSSSCG00000007478 | 17 | 52936173 | 53103190 | 45 min-24 h pH decline QTL (3299) | 17 | 41500245 | 63494066 |
| ENSSSCG00000007478 | 17 | 52936173 | 53103190 | Interferon-gamma to interleukin-10 ratio QTL (12274) | 17 | 44511304 | 63494066 |
| ENSSSCG00000007478 | 17 | 52936173 | 53103190 | Meat color score QTL (129) | 17 | 44511304 | 63494066 |
| ENSSSCG00000007478 | 17 | 52936173 | 53103190 | Meat color-L QTL (130) | 17 | 44511304 | 63494066 |
| ENSSSCG00000007478 | 17 | 52936173 | 53103190 | Meat color-L QTL (131) | 17 | 44511304 | 63494066 |
| ENSSSCG00000007478 | 17 | 52936173 | 53103190 | Average lactate QTL (132) | 17 | 44511304 | 63494066 |
| ENSSSCG00000007478 | 17 | 52936173 | 53103190 | Average glycolytic potential QTL (133) | 17 | 44511304 | 63494066 |
| ENSSSCG00000007478 | 17 | 52936173 | 53103190 | Meat color b* QTL (4010) | 17 | 44511304 | 58061068 |
| ENSSSCG00000007478 | 17 | 52936173 | 53103190 | Meat color score QTL (2967) | 17 | 44511304 | 58061068 |
| ENSSSCG00000007478 | 17 | 52936173 | 53103190 | Meat color-L QTL (2968) | 17 | 44511304 | 58061068 |
| ENSSSCG00000007478 | 17 | 52936173 | 53103190 | Meat color-L QTL (2969) | 17 | 44511304 | 58061068 |
| ENSSSCG00000007478 | 17 | 52936173 | 53103190 | Fat protein content QTL (18014) | 17 | 50337048 | 63494066 |
| ENSSSCG00000007478 | 17 | 52936173 | 53103190 | Subcutanous fat area QTL (18008) | 17 | 50402506 | 63494066 |
| ENSSSCG00000029119 | 17 | 62815284 | 62861865 | Backfat at last rib QTL (16842) | 17 | 38030981 | 63494066 |
| ENSSSCG00000029119 | 17 | 62815284 | 62861865 | Carcass length QTL (1186) | 17 | 38685559 | 63494066 |
| ENSSSCG00000029119 | 17 | 62815284 | 62861865 | Backfat at last rib QTL (1191) | 17 | 38685559 | 63494066 |
| ENSSSCG00000029119 | 17 | 62815284 | 62861865 | 45 min-24 h pH decline QTL (3299) | 17 | 41500245 | 63494066 |
| ENSSSCG00000029119 | 17 | 62815284 | 62861865 | Interferon-gamma to interleukin-10 ratio QTL (12274) | 17 | 44511304 | 63494066 |
| ENSSSCG00000029119 | 17 | 62815284 | 62861865 | Meat color score QTL (129) | 17 | 44511304 | 63494066 |
| ENSSSCG00000029119 | 17 | 62815284 | 62861865 | Meat color-L QTL (130) | 17 | 44511304 | 63494066 |
| ENSSSCG00000029119 | 17 | 62815284 | 62861865 | Meat color-L QTL (131) | 17 | 44511304 | 63494066 |
| ENSSSCG00000029119 | 17 | 62815284 | 62861865 | Average lactate QTL (132) | 17 | 44511304 | 63494066 |
| ENSSSCG00000029119 | 17 | 62815284 | 62861865 | Average glycolytic potential QTL (133) | 17 | 44511304 | 63494066 |
| ENSSSCG00000029119 | 17 | 62815284 | 62861865 | Fat protein content QTL (18014) | 17 | 50337048 | 63494066 |
| ENSSSCG00000029119 | 17 | 62815284 | 62861865 | Subcutanous fat area QTL (18008) | 17 | 50402506 | 63494066 |
| ENSSSCG00000038617 | 17 | 63029289 | 63049669 | Backfat at last rib QTL (16842) | 17 | 38030981 | 63494066 |
| ENSSSCG00000038617 | 17 | 63029289 | 63049669 | Carcass length QTL (1186) | 17 | 38685559 | 63494066 |
| ENSSSCG00000038617 | 17 | 63029289 | 63049669 | Backfat at last rib QTL (1191) | 17 | 38685559 | 63494066 |
| ENSSSCG00000038617 | 17 | 63029289 | 63049669 | 45 min-24 h pH decline QTL (3299) | 17 | 41500245 | 63494066 |
| ENSSSCG00000038617 | 17 | 63029289 | 63049669 | Interferon-gamma to interleukin-10 ratio QTL (12274) | 17 | 44511304 | 63494066 |
| ENSSSCG00000038617 | 17 | 63029289 | 63049669 | Meat color score QTL (129) | 17 | 44511304 | 63494066 |
| ENSSSCG00000038617 | 17 | 63029289 | 63049669 | Meat color-L QTL (130) | 17 | 44511304 | 63494066 |
| ENSSSCG00000038617 | 17 | 63029289 | 63049669 | Meat color-L QTL (131) | 17 | 44511304 | 63494066 |
| ENSSSCG00000038617 | 17 | 63029289 | 63049669 | Average lactate QTL (132) | 17 | 44511304 | 63494066 |
| ENSSSCG00000038617 | 17 | 63029289 | 63049669 | Average glycolytic potential QTL (133) | 17 | 44511304 | 63494066 |
| ENSSSCG00000038617 | 17 | 63029289 | 63049669 | Fat protein content QTL (18014) | 17 | 50337048 | 63494066 |
| ENSSSCG00000038617 | 17 | 63029289 | 63049669 | Subcutanous fat area QTL (18008) | 17 | 50402506 | 63494066 |
| ENSSSCG00000020663 | 18 | 4993196 | 5279042 | Body weight (end of test) QTL (5202) | 18 | 1332927 | 39721224 |
| ENSSSCG00000020663 | 18 | 4993196 | 5279042 | Abdominal fat weight QTL (21272) | 18 | 2932439 | 36433338 |
| ENSSSCG00000020663 | 18 | 4993196 | 5279042 | Triglyceride level QTL (15084) | 18 | 3909919 | 39810086 |
| ENSSSCG00000020663 | 18 | 4993196 | 5279042 | Meat color a* QTL (38118) | 18 | 3909919 | 39810086 |
| ENSSSCG00000020663 | 18 | 4993196 | 5279042 | Calcium level QTL (15082) | 18 | 3909919 | 38654883 |
| ENSSSCG00000020663 | 18 | 4993196 | 5279042 | Capric acid content QTL (156972) | 18 | 3909919 | 38654883 |
| ENSSSCG00000020663 | 18 | 4993196 | 5279042 | Palmitoleic acid content QTL (156973) | 18 | 3909919 | 26658540 |
| ENSSSCG00000020663 | 18 | 4993196 | 5279042 | Drip loss QTL (220) | 18 | 4443090 | 28080328 |
| ENSSSCG00000020663 | 18 | 4993196 | 5279042 | pH 24 hr post-mortem (loin) QTL (3300) | 18 | 4443090 | 26125369 |
| ENSSSCG00000020663 | 18 | 4993196 | 5279042 | Backfat at last rib QTL (3301) | 18 | 4443090 | 26125369 |
| ENSSSCG00000020663 | 18 | 4993196 | 5279042 | Backfat at tenth rib QTL (3302) | 18 | 4443090 | 26125369 |
| ENSSSCG00000020663 | 18 | 4993196 | 5279042 | Backfat linear at tenth rib QTL (3661) | 18 | 4443090 | 26125369 |
| ENSSSCG00000020663 | 18 | 4993196 | 5279042 | Linoleic acid content QTL (15880) | 18 | 4443090 | 23370653 |
| ENSSSCG00000020663 | 18 | 4993196 | 5279042 | Linoleic acid content QTL (15882) | 18 | 4443090 | 23370653 |
| ENSSSCG00000020663 | 18 | 4993196 | 5279042 | Palmitic acid content QTL (15881) | 18 | 4443090 | 21149108 |
| ENSSSCG00000016430 | 18 | 5289917 | 5348058 | Body weight (end of test) QTL (5202) | 18 | 1332927 | 39721224 |
| ENSSSCG00000016430 | 18 | 5289917 | 5348058 | Abdominal fat weight QTL (21272) | 18 | 2932439 | 36433338 |
| ENSSSCG00000016430 | 18 | 5289917 | 5348058 | Triglyceride level QTL (15084) | 18 | 3909919 | 39810086 |
| ENSSSCG00000016430 | 18 | 5289917 | 5348058 | Meat color a* QTL (38118) | 18 | 3909919 | 39810086 |
| ENSSSCG00000016430 | 18 | 5289917 | 5348058 | Calcium level QTL (15082) | 18 | 3909919 | 38654883 |
| ENSSSCG00000016430 | 18 | 5289917 | 5348058 | Capric acid content QTL (156972) | 18 | 3909919 | 38654883 |
| ENSSSCG00000016430 | 18 | 5289917 | 5348058 | Palmitoleic acid content QTL (156973) | 18 | 3909919 | 26658540 |
| ENSSSCG00000016430 | 18 | 5289917 | 5348058 | Drip loss QTL (220) | 18 | 4443090 | 28080328 |
| ENSSSCG00000016430 | 18 | 5289917 | 5348058 | pH 24 hr post-mortem (loin) QTL (3300) | 18 | 4443090 | 26125369 |
| ENSSSCG00000016430 | 18 | 5289917 | 5348058 | Backfat at last rib QTL (3301) | 18 | 4443090 | 26125369 |
| ENSSSCG00000016430 | 18 | 5289917 | 5348058 | Backfat at tenth rib QTL (3302) | 18 | 4443090 | 26125369 |
| ENSSSCG00000016430 | 18 | 5289917 | 5348058 | Backfat linear at tenth rib QTL (3661) | 18 | 4443090 | 26125369 |
| ENSSSCG00000016430 | 18 | 5289917 | 5348058 | Linoleic acid content QTL (15880) | 18 | 4443090 | 23370653 |
| ENSSSCG00000016430 | 18 | 5289917 | 5348058 | Linoleic acid content QTL (15882) | 18 | 4443090 | 23370653 |
| ENSSSCG00000016430 | 18 | 5289917 | 5348058 | Palmitic acid content QTL (15881) | 18 | 4443090 | 21149108 |
| ENSSSCG00000016510 | 18 | 10381209 | 10451255 | Body weight (end of test) QTL (5202) | 18 | 1332927 | 39721224 |
| ENSSSCG00000016510 | 18 | 10381209 | 10451255 | Abdominal fat weight QTL (21272) | 18 | 2932439 | 36433338 |
| ENSSSCG00000016510 | 18 | 10381209 | 10451255 | Triglyceride level QTL (15084) | 18 | 3909919 | 39810086 |
| ENSSSCG00000016510 | 18 | 10381209 | 10451255 | Meat color a* QTL (38118) | 18 | 3909919 | 39810086 |
| ENSSSCG00000016510 | 18 | 10381209 | 10451255 | Calcium level QTL (15082) | 18 | 3909919 | 38654883 |
| ENSSSCG00000016510 | 18 | 10381209 | 10451255 | Capric acid content QTL (156972) | 18 | 3909919 | 38654883 |
| ENSSSCG00000016510 | 18 | 10381209 | 10451255 | Palmitoleic acid content QTL (156973) | 18 | 3909919 | 26658540 |
| ENSSSCG00000016510 | 18 | 10381209 | 10451255 | Drip loss QTL (220) | 18 | 4443090 | 28080328 |
| ENSSSCG00000016510 | 18 | 10381209 | 10451255 | pH 24 hr post-mortem (loin) QTL (3300) | 18 | 4443090 | 26125369 |
| ENSSSCG00000016510 | 18 | 10381209 | 10451255 | Backfat at last rib QTL (3301) | 18 | 4443090 | 26125369 |
| ENSSSCG00000016510 | 18 | 10381209 | 10451255 | Backfat at tenth rib QTL (3302) | 18 | 4443090 | 26125369 |
| ENSSSCG00000016510 | 18 | 10381209 | 10451255 | Backfat linear at tenth rib QTL (3661) | 18 | 4443090 | 26125369 |
| ENSSSCG00000016510 | 18 | 10381209 | 10451255 | Linoleic acid content QTL (15880) | 18 | 4443090 | 23370653 |
| ENSSSCG00000016510 | 18 | 10381209 | 10451255 | Linoleic acid content QTL (15882) | 18 | 4443090 | 23370653 |
| ENSSSCG00000016510 | 18 | 10381209 | 10451255 | Palmitic acid content QTL (15881) | 18 | 4443090 | 21149108 |
| ENSSSCG00000016510 | 18 | 10381209 | 10451255 | Backfat at last rib QTL (16843) | 18 | 7020082 | 28080328 |
| ENSSSCG00000016510 | 18 | 10381209 | 10451255 | Subcutanous fat area QTL (18009) | 18 | 7020082 | 28080328 |
| ENSSSCG00000016510 | 18 | 10381209 | 10451255 | Fat protein content QTL (18015) | 18 | 7197805 | 38654883 |
| ENSSSCG00000016543 | 18 | 15105138 | 15906707 | Loin muscle area QTL (106194) | 18 | 15130519 | 15130523 |
| ENSSSCG00000016543 | 18 | 15105138 | 15906707 | Body weight (end of test) QTL (5202) | 18 | 1332927 | 39721224 |
| ENSSSCG00000016543 | 18 | 15105138 | 15906707 | Abdominal fat weight QTL (21272) | 18 | 2932439 | 36433338 |
| ENSSSCG00000016543 | 18 | 15105138 | 15906707 | Triglyceride level QTL (15084) | 18 | 3909919 | 39810086 |
| ENSSSCG00000016543 | 18 | 15105138 | 15906707 | Meat color a* QTL (38118) | 18 | 3909919 | 39810086 |
| ENSSSCG00000016543 | 18 | 15105138 | 15906707 | Calcium level QTL (15082) | 18 | 3909919 | 38654883 |
| ENSSSCG00000016543 | 18 | 15105138 | 15906707 | Capric acid content QTL (156972) | 18 | 3909919 | 38654883 |
| ENSSSCG00000016543 | 18 | 15105138 | 15906707 | Palmitoleic acid content QTL (156973) | 18 | 3909919 | 26658540 |
| ENSSSCG00000016543 | 18 | 15105138 | 15906707 | Drip loss QTL (220) | 18 | 4443090 | 28080328 |
| ENSSSCG00000016543 | 18 | 15105138 | 15906707 | pH 24 hr post-mortem (loin) QTL (3300) | 18 | 4443090 | 26125369 |
| ENSSSCG00000016543 | 18 | 15105138 | 15906707 | Backfat at last rib QTL (3301) | 18 | 4443090 | 26125369 |
| ENSSSCG00000016543 | 18 | 15105138 | 15906707 | Backfat at tenth rib QTL (3302) | 18 | 4443090 | 26125369 |
| ENSSSCG00000016543 | 18 | 15105138 | 15906707 | Backfat linear at tenth rib QTL (3661) | 18 | 4443090 | 26125369 |
| ENSSSCG00000016543 | 18 | 15105138 | 15906707 | Linoleic acid content QTL (15880) | 18 | 4443090 | 23370653 |
| ENSSSCG00000016543 | 18 | 15105138 | 15906707 | Linoleic acid content QTL (15882) | 18 | 4443090 | 23370653 |
| ENSSSCG00000016543 | 18 | 15105138 | 15906707 | Palmitic acid content QTL (15881) | 18 | 4443090 | 21149108 |
| ENSSSCG00000016543 | 18 | 15105138 | 15906707 | Backfat at last rib QTL (16843) | 18 | 7020082 | 28080328 |
| ENSSSCG00000016543 | 18 | 15105138 | 15906707 | Subcutanous fat area QTL (18009) | 18 | 7020082 | 28080328 |
| ENSSSCG00000016543 | 18 | 15105138 | 15906707 | Fat protein content QTL (18015) | 18 | 7197805 | 38654883 |
| ENSSSCG00000021885 | 18 | 31027031 | 31125465 | Body weight (end of test) QTL (5202) | 18 | 1332927 | 39721224 |
| ENSSSCG00000021885 | 18 | 31027031 | 31125465 | Abdominal fat weight QTL (21272) | 18 | 2932439 | 36433338 |
| ENSSSCG00000021885 | 18 | 31027031 | 31125465 | Triglyceride level QTL (15084) | 18 | 3909919 | 39810086 |
| ENSSSCG00000021885 | 18 | 31027031 | 31125465 | Meat color a* QTL (38118) | 18 | 3909919 | 39810086 |
| ENSSSCG00000021885 | 18 | 31027031 | 31125465 | Calcium level QTL (15082) | 18 | 3909919 | 38654883 |
| ENSSSCG00000021885 | 18 | 31027031 | 31125465 | Capric acid content QTL (156972) | 18 | 3909919 | 38654883 |
| ENSSSCG00000021885 | 18 | 31027031 | 31125465 | Fat protein content QTL (18015) | 18 | 7197805 | 38654883 |
| ENSSSCG00000021885 | 18 | 31027031 | 31125465 | Average glycogen QTL (3831) | 18 | 26125369 | 40165533 |
| ENSSSCG00000021885 | 18 | 31027031 | 31125465 | Backfat at last rib QTL (3217) | 18 | 26125369 | 38654883 |
| ENSSSCG00000021885 | 18 | 31027031 | 31125465 | Backfat at tenth rib QTL (3219) | 18 | 26125369 | 38654883 |
| ENSSSCG00000021885 | 18 | 31027031 | 31125465 | Potassium level QTL (15083) | 18 | 26658540 | 39810086 |
| ENSSSCG00000021885 | 18 | 31027031 | 31125465 | Interleukin-10 level QTL (17609) | 18 | 28080328 | 51184396 |
| ENSSSCG00000021885 | 18 | 31027031 | 31125465 | Toll-like receptor 9 level QTL (17630) | 18 | 28080328 | 51184396 |
| ENSSSCG00000021885 | 18 | 31027031 | 31125465 | Percentage giant fibers QTL (2839) | 18 | 28080328 | 51184396 |
| ENSSSCG00000021885 | 18 | 31027031 | 31125465 | Bicarbonate level QTL (6332) | 18 | 28080328 | 40165533 |
| ENSSSCG00000021885 | 18 | 31027031 | 31125465 | Carcass weight (hot) QTL (5955) | 18 | 28080328 | 38654883 |
| ENSSSCG00000021885 | 18 | 31027031 | 31125465 | pH 24 hr post-mortem (loin) QTL (1194) | 18 | 28080328 | 38654883 |
| ENSSSCG00000021885 | 18 | 31027031 | 31125465 | pH 45 minutes post mortem QTL (3082) | 18 | 28080328 | 38654883 |
| ENSSSCG00000021885 | 18 | 31027031 | 31125465 | Change in tetanus antibody titer QTL (12333) | 18 | 28080328 | 38654883 |
| ENSSSCG00000021885 | 18 | 31027031 | 31125465 | Interferon-gamma level QTL (12345) | 18 | 28080328 | 38654883 |
| ENSSSCG00000036350 | 18 | 46205418 | 46215858 | Interleukin-10 level QTL (17609) | 18 | 28080328 | 51184396 |
| ENSSSCG00000036350 | 18 | 46205418 | 46215858 | Toll-like receptor 9 level QTL (17630) | 18 | 28080328 | 51184396 |
| ENSSSCG00000036350 | 18 | 46205418 | 46215858 | Percentage giant fibers QTL (2839) | 18 | 28080328 | 51184396 |
| ENSSSCG00000036350 | 18 | 46205418 | 46215858 | Drip loss QTL (5733) | 18 | 38654883 | 51184396 |
| ENSSSCG00000036350 | 18 | 46205418 | 46215858 | Carcass length QTL (5734) | 18 | 38654883 | 51184396 |
| ENSSSCG00000016717 | 18 | 47593049 | 47710570 | Palmitic acid content QTL (18616) | 18 | 47705311 | 47705315 |
| ENSSSCG00000016717 | 18 | 47593049 | 47710570 | Interleukin-10 level QTL (17609) | 18 | 28080328 | 51184396 |
| ENSSSCG00000016717 | 18 | 47593049 | 47710570 | Toll-like receptor 9 level QTL (17630) | 18 | 28080328 | 51184396 |
| ENSSSCG00000016717 | 18 | 47593049 | 47710570 | Percentage giant fibers QTL (2839) | 18 | 28080328 | 51184396 |
| ENSSSCG00000016717 | 18 | 47593049 | 47710570 | Drip loss QTL (5733) | 18 | 38654883 | 51184396 |
| ENSSSCG00000016717 | 18 | 47593049 | 47710570 | Carcass length QTL (5734) | 18 | 38654883 | 51184396 |
| ENSSSCG00000035581 | 18 | 53639593 | 54283251 | Meat color L* QTL (17814) | 18 | 50295778 | 59448544 |
| ENSSSCG00000012884 | 2 | 4394675 | 4523412 | Linoleic acid content QTL (218164) | 2 | 4412668 | 4412672 |
| ENSSSCG00000012884 | 2 | 4394675 | 4523412 | Polyunsaturated fatty acid content QTL (218166) | 2 | 4412668 | 4412672 |
| ENSSSCG00000012884 | 2 | 4394675 | 4523412 | Monounsaturated fatty acid to polyunsaturated fatty acid ratio QTL (218171) | 2 | 4412668 | 4412672 |
| ENSSSCG00000012884 | 2 | 4394675 | 4523412 | Polyunsaturated fatty acid to saturated fatty acid ratio QTL (218256) | 2 | 4412668 | 4412672 |
| ENSSSCG00000012884 | 2 | 4394675 | 4523412 | Monounsaturated fatty acid to polyunsaturated fatty acid ratio QTL (218205) | 2 | 4444551 | 4444555 |
| ENSSSCG00000012884 | 2 | 4394675 | 4523412 | Linoleic acid content QTL (218224) | 2 | 4444551 | 4444555 |
| ENSSSCG00000012884 | 2 | 4394675 | 4523412 | Polyunsaturated fatty acid content QTL (218229) | 2 | 4444551 | 4444555 |
| ENSSSCG00000012884 | 2 | 4394675 | 4523412 | Average daily gain QTL (17580) | 2 | 4428108 | 4520120 |
| ENSSSCG00000012884 | 2 | 4394675 | 4523412 | Average daily gain QTL (17581) | 2 | 4428108 | 4520120 |
| ENSSSCG00000012884 | 2 | 4394675 | 4523412 | Average backfat thickness QTL (17582) | 2 | 4428108 | 4520120 |
| ENSSSCG00000012884 | 2 | 4394675 | 4523412 | Lean meat + bone in ham QTL (17584) | 2 | 4428108 | 4520120 |
| ENSSSCG00000012884 | 2 | 4394675 | 4523412 | Drip loss QTL (17585) | 2 | 4428108 | 4520120 |
| ENSSSCG00000012884 | 2 | 4394675 | 4523412 | Fiber type II myosin isoform ratio QTL (17586) | 2 | 4428108 | 4520120 |
| ENSSSCG00000012884 | 2 | 4394675 | 4523412 | Shear force QTL (17587) | 2 | 4428108 | 4520120 |
| ENSSSCG00000012884 | 2 | 4394675 | 4523412 | Average lactate QTL (17588) | 2 | 4428108 | 4520120 |
| ENSSSCG00000012884 | 2 | 4394675 | 4523412 | Backfat between 6th and 7th ribs QTL (3942) | 2 | 552075 | 44051051 |
| ENSSSCG00000012884 | 2 | 4394675 | 4523412 | Backfat at first rib QTL (21216) | 2 | 690094 | 62108532 |
| ENSSSCG00000012884 | 2 | 4394675 | 4523412 | Subcutanous fat area QTL (18003) | 2 | 690094 | 35884929 |
| ENSSSCG00000012884 | 2 | 4394675 | 4523412 | Fat protein content QTL (18010) | 2 | 690094 | 35884929 |
| ENSSSCG00000012884 | 2 | 4394675 | 4523412 | NADP-malate dehydrogenase activity QTL (18016) | 2 | 690094 | 35884929 |
| ENSSSCG00000012884 | 2 | 4394675 | 4523412 | Average backfat thickness QTL (311) | 2 | 690094 | 35884929 |
| ENSSSCG00000012884 | 2 | 4394675 | 4523412 | Backfat at rump QTL (21217) | 2 | 690094 | 27718807 |
| ENSSSCG00000012884 | 2 | 4394675 | 4523412 | PH for Longissmus dorsi QTL (2972) | 2 | 690094 | 15757164 |
| ENSSSCG00000012884 | 2 | 4394675 | 4523412 | Backfat at last lumbar QTL (2933) | 2 | 690094 | 11271548 |
| ENSSSCG00000012884 | 2 | 4394675 | 4523412 | Cooking loss QTL (2970) | 2 | 690094 | 11156532 |
| ENSSSCG00000012884 | 2 | 4394675 | 4523412 | Meat color L* QTL (2971) | 2 | 690094 | 8856216 |
| ENSSSCG00000012884 | 2 | 4394675 | 4523412 | Abdominal fat weight QTL (21214) | 2 | 690094 | 1.13E+08 |
| ENSSSCG00000012884 | 2 | 4394675 | 4523412 | Average backfat thickness QTL (21212) | 2 | 690094 | 1.12E+08 |
| ENSSSCG00000012884 | 2 | 4394675 | 4523412 | Abdominal fat weight QTL (21213) | 2 | 2875395 | 1.02E+08 |
| ENSSSCG00000012884 | 2 | 4394675 | 4523412 | Backfat at mid-back QTL (5242) | 2 | 4140568 | 1.36E+08 |
| ENSSSCG00000013054 | 2 | 8361774 | 8408917 | Backfat between 6th and 7th ribs QTL (3942) | 2 | 552075 | 44051051 |
| ENSSSCG00000013054 | 2 | 8361774 | 8408917 | Backfat at first rib QTL (21216) | 2 | 690094 | 62108532 |
| ENSSSCG00000013054 | 2 | 8361774 | 8408917 | Subcutanous fat area QTL (18003) | 2 | 690094 | 35884929 |
| ENSSSCG00000013054 | 2 | 8361774 | 8408917 | Fat protein content QTL (18010) | 2 | 690094 | 35884929 |
| ENSSSCG00000013054 | 2 | 8361774 | 8408917 | NADP-malate dehydrogenase activity QTL (18016) | 2 | 690094 | 35884929 |
| ENSSSCG00000013054 | 2 | 8361774 | 8408917 | Average backfat thickness QTL (311) | 2 | 690094 | 35884929 |
| ENSSSCG00000013054 | 2 | 8361774 | 8408917 | Backfat at rump QTL (21217) | 2 | 690094 | 27718807 |
| ENSSSCG00000013054 | 2 | 8361774 | 8408917 | PH for Longissmus dorsi QTL (2972) | 2 | 690094 | 15757164 |
| ENSSSCG00000013054 | 2 | 8361774 | 8408917 | Backfat at last lumbar QTL (2933) | 2 | 690094 | 11271548 |
| ENSSSCG00000013054 | 2 | 8361774 | 8408917 | Cooking loss QTL (2970) | 2 | 690094 | 11156532 |
| ENSSSCG00000013054 | 2 | 8361774 | 8408917 | Meat color L* QTL (2971) | 2 | 690094 | 8856216 |
| ENSSSCG00000013054 | 2 | 8361774 | 8408917 | Abdominal fat weight QTL (21214) | 2 | 690094 | 1.13E+08 |
| ENSSSCG00000013054 | 2 | 8361774 | 8408917 | Average backfat thickness QTL (21212) | 2 | 690094 | 1.12E+08 |
| ENSSSCG00000013054 | 2 | 8361774 | 8408917 | Abdominal fat weight QTL (21213) | 2 | 2875395 | 1.02E+08 |
| ENSSSCG00000013054 | 2 | 8361774 | 8408917 | Backfat at mid-back QTL (5242) | 2 | 4140568 | 1.36E+08 |
| ENSSSCG00000013054 | 2 | 8361774 | 8408917 | Shoulder subcutaneous fat thickness QTL (5218) | 2 | 7936090 | 1.19E+08 |
| ENSSSCG00000013223 | 2 | 14840391 | 14893036 | Backfat between 6th and 7th ribs QTL (3942) | 2 | 552075 | 44051051 |
| ENSSSCG00000013223 | 2 | 14840391 | 14893036 | Backfat at first rib QTL (21216) | 2 | 690094 | 62108532 |
| ENSSSCG00000013223 | 2 | 14840391 | 14893036 | Subcutanous fat area QTL (18003) | 2 | 690094 | 35884929 |
| ENSSSCG00000013223 | 2 | 14840391 | 14893036 | Fat protein content QTL (18010) | 2 | 690094 | 35884929 |
| ENSSSCG00000013223 | 2 | 14840391 | 14893036 | NADP-malate dehydrogenase activity QTL (18016) | 2 | 690094 | 35884929 |
| ENSSSCG00000013223 | 2 | 14840391 | 14893036 | Average backfat thickness QTL (311) | 2 | 690094 | 35884929 |
| ENSSSCG00000013223 | 2 | 14840391 | 14893036 | Backfat at rump QTL (21217) | 2 | 690094 | 27718807 |
| ENSSSCG00000013223 | 2 | 14840391 | 14893036 | PH for Longissmus dorsi QTL (2972) | 2 | 690094 | 15757164 |
| ENSSSCG00000013223 | 2 | 14840391 | 14893036 | Average backfat thickness QTL (5965) | 2 | 11271548 | 63258690 |
| ENSSSCG00000013223 | 2 | 14840391 | 14893036 | Loin muscle area QTL (38051) | 2 | 11271548 | 48306636 |
| ENSSSCG00000013223 | 2 | 14840391 | 14893036 | Average daily gain QTL (929) | 2 | 11271548 | 48306636 |
| ENSSSCG00000013223 | 2 | 14840391 | 14893036 | Carcass weight (hot) QTL (5937) | 2 | 11271548 | 35884929 |
| ENSSSCG00000013223 | 2 | 14840391 | 14893036 | Shoulder subcutaneous fat thickness QTL (5961) | 2 | 11271548 | 35884929 |
| ENSSSCG00000013223 | 2 | 14840391 | 14893036 | Backfat at last rib QTL (5966) | 2 | 11271548 | 35884929 |
| ENSSSCG00000013223 | 2 | 14840391 | 14893036 | Loin muscle area QTL (5968) | 2 | 11271548 | 35884929 |
| ENSSSCG00000013223 | 2 | 14840391 | 14893036 | Subcutanous fat area QTL (5969) | 2 | 11271548 | 35884929 |
| ENSSSCG00000013223 | 2 | 14840391 | 14893036 | Estimated carcass lean content QTL (5972) | 2 | 11271548 | 35884929 |
| ENSSSCG00000013223 | 2 | 14840391 | 14893036 | Drip loss QTL (5683) | 2 | 11271548 | 35884929 |
| ENSSSCG00000013223 | 2 | 14840391 | 14893036 | Interferon-gamma level QTL (12334) | 2 | 11271548 | 35884929 |
| ENSSSCG00000013223 | 2 | 14840391 | 14893036 | Average backfat thickness QTL (923) | 2 | 11271548 | 35884929 |
| ENSSSCG00000013223 | 2 | 14840391 | 14893036 | Carcass length QTL (924) | 2 | 11271548 | 35884929 |
| ENSSSCG00000013223 | 2 | 14840391 | 14893036 | Body weight (3 weeks) QTL (928) | 2 | 11271548 | 35884929 |
| ENSSSCG00000013223 | 2 | 14840391 | 14893036 | Loin muscle area QTL (38052) | 2 | 11271548 | 22083033 |
| ENSSSCG00000013223 | 2 | 14840391 | 14893036 | Abdominal fat weight QTL (21214) | 2 | 690094 | 1.13E+08 |
| ENSSSCG00000013223 | 2 | 14840391 | 14893036 | Average backfat thickness QTL (21212) | 2 | 690094 | 1.12E+08 |
| ENSSSCG00000013223 | 2 | 14840391 | 14893036 | Abdominal fat weight QTL (21213) | 2 | 2875395 | 1.02E+08 |
| ENSSSCG00000013223 | 2 | 14840391 | 14893036 | Backfat at mid-back QTL (5242) | 2 | 4140568 | 1.36E+08 |
| ENSSSCG00000013223 | 2 | 14840391 | 14893036 | Shoulder subcutaneous fat thickness QTL (5218) | 2 | 7936090 | 1.19E+08 |
| ENSSSCG00000013223 | 2 | 14840391 | 14893036 | Backfat at mid-back QTL (5208) | 2 | 9431295 | 1.35E+08 |
| ENSSSCG00000013223 | 2 | 14840391 | 14893036 | Estimated carcass lean content QTL (5691) | 2 | 11271548 | 1.47E+08 |
| ENSSSCG00000013223 | 2 | 14840391 | 14893036 | Shear force QTL (38074) | 2 | 11271548 | 1.17E+08 |
| ENSSSCG00000013223 | 2 | 14840391 | 14893036 | Meat color L* QTL (38076) | 2 | 11271548 | 1.17E+08 |
| ENSSSCG00000013223 | 2 | 14840391 | 14893036 | Arachidic acid content QTL (156919) | 2 | 11271548 | 1.17E+08 |
| ENSSSCG00000013223 | 2 | 14840391 | 14893036 | Arachidonic acid content QTL (156920) | 2 | 11271548 | 1.17E+08 |
| ENSSSCG00000013223 | 2 | 14840391 | 14893036 | Unsaturated fatty acid content QTL (156922) | 2 | 11271548 | 1.17E+08 |
| ENSSSCG00000013223 | 2 | 14840391 | 14893036 | Linolenic acid content QTL (156918) | 2 | 11271548 | 97878445 |
| ENSSSCG00000013223 | 2 | 14840391 | 14893036 | Saturated fatty acid content QTL (156921) | 2 | 11271548 | 97878445 |
| ENSSSCG00000013223 | 2 | 14840391 | 14893036 | cis-9-Heptadecenoic acid content QTL (156917) | 2 | 11271548 | 72689985 |
| ENSSSCG00000013223 | 2 | 14840391 | 14893036 | Palmitic acid content QTL (156868) | 2 | 11271548 | 72689985 |
| ENSSSCG00000013223 | 2 | 14840391 | 14893036 | Body weight (end of test) QTL (5199) | 2 | 14031927 | 1.34E+08 |
| ENSSSCG00000030610 | 2 | 14896175 | 14932185 | Backfat between 6th and 7th ribs QTL (3942) | 2 | 552075 | 44051051 |
| ENSSSCG00000030610 | 2 | 14896175 | 14932185 | Backfat at first rib QTL (21216) | 2 | 690094 | 62108532 |
| ENSSSCG00000030610 | 2 | 14896175 | 14932185 | Subcutanous fat area QTL (18003) | 2 | 690094 | 35884929 |
| ENSSSCG00000030610 | 2 | 14896175 | 14932185 | Fat protein content QTL (18010) | 2 | 690094 | 35884929 |
| ENSSSCG00000030610 | 2 | 14896175 | 14932185 | NADP-malate dehydrogenase activity QTL (18016) | 2 | 690094 | 35884929 |
| ENSSSCG00000030610 | 2 | 14896175 | 14932185 | Average backfat thickness QTL (311) | 2 | 690094 | 35884929 |
| ENSSSCG00000030610 | 2 | 14896175 | 14932185 | Backfat at rump QTL (21217) | 2 | 690094 | 27718807 |
| ENSSSCG00000030610 | 2 | 14896175 | 14932185 | PH for Longissmus dorsi QTL (2972) | 2 | 690094 | 15757164 |
| ENSSSCG00000030610 | 2 | 14896175 | 14932185 | Average backfat thickness QTL (5965) | 2 | 11271548 | 63258690 |
| ENSSSCG00000030610 | 2 | 14896175 | 14932185 | Loin muscle area QTL (38051) | 2 | 11271548 | 48306636 |
| ENSSSCG00000030610 | 2 | 14896175 | 14932185 | Average daily gain QTL (929) | 2 | 11271548 | 48306636 |
| ENSSSCG00000030610 | 2 | 14896175 | 14932185 | Carcass weight (hot) QTL (5937) | 2 | 11271548 | 35884929 |
| ENSSSCG00000030610 | 2 | 14896175 | 14932185 | Shoulder subcutaneous fat thickness QTL (5961) | 2 | 11271548 | 35884929 |
| ENSSSCG00000030610 | 2 | 14896175 | 14932185 | Backfat at last rib QTL (5966) | 2 | 11271548 | 35884929 |
| ENSSSCG00000030610 | 2 | 14896175 | 14932185 | Loin muscle area QTL (5968) | 2 | 11271548 | 35884929 |
| ENSSSCG00000030610 | 2 | 14896175 | 14932185 | Subcutanous fat area QTL (5969) | 2 | 11271548 | 35884929 |
| ENSSSCG00000030610 | 2 | 14896175 | 14932185 | Estimated carcass lean content QTL (5972) | 2 | 11271548 | 35884929 |
| ENSSSCG00000030610 | 2 | 14896175 | 14932185 | Drip loss QTL (5683) | 2 | 11271548 | 35884929 |
| ENSSSCG00000030610 | 2 | 14896175 | 14932185 | Interferon-gamma level QTL (12334) | 2 | 11271548 | 35884929 |
| ENSSSCG00000030610 | 2 | 14896175 | 14932185 | Average backfat thickness QTL (923) | 2 | 11271548 | 35884929 |
| ENSSSCG00000030610 | 2 | 14896175 | 14932185 | Carcass length QTL (924) | 2 | 11271548 | 35884929 |
| ENSSSCG00000030610 | 2 | 14896175 | 14932185 | Body weight (3 weeks) QTL (928) | 2 | 11271548 | 35884929 |
| ENSSSCG00000030610 | 2 | 14896175 | 14932185 | Loin muscle area QTL (38052) | 2 | 11271548 | 22083033 |
| ENSSSCG00000030610 | 2 | 14896175 | 14932185 | Abdominal fat weight QTL (21214) | 2 | 690094 | 1.13E+08 |
| ENSSSCG00000030610 | 2 | 14896175 | 14932185 | Average backfat thickness QTL (21212) | 2 | 690094 | 1.12E+08 |
| ENSSSCG00000030610 | 2 | 14896175 | 14932185 | Abdominal fat weight QTL (21213) | 2 | 2875395 | 1.02E+08 |
| ENSSSCG00000030610 | 2 | 14896175 | 14932185 | Backfat at mid-back QTL (5242) | 2 | 4140568 | 1.36E+08 |
| ENSSSCG00000030610 | 2 | 14896175 | 14932185 | Shoulder subcutaneous fat thickness QTL (5218) | 2 | 7936090 | 1.19E+08 |
| ENSSSCG00000030610 | 2 | 14896175 | 14932185 | Backfat at mid-back QTL (5208) | 2 | 9431295 | 1.35E+08 |
| ENSSSCG00000030610 | 2 | 14896175 | 14932185 | Estimated carcass lean content QTL (5691) | 2 | 11271548 | 1.47E+08 |
| ENSSSCG00000030610 | 2 | 14896175 | 14932185 | Shear force QTL (38074) | 2 | 11271548 | 1.17E+08 |
| ENSSSCG00000030610 | 2 | 14896175 | 14932185 | Meat color L* QTL (38076) | 2 | 11271548 | 1.17E+08 |
| ENSSSCG00000030610 | 2 | 14896175 | 14932185 | Arachidic acid content QTL (156919) | 2 | 11271548 | 1.17E+08 |
| ENSSSCG00000030610 | 2 | 14896175 | 14932185 | Arachidonic acid content QTL (156920) | 2 | 11271548 | 1.17E+08 |
| ENSSSCG00000030610 | 2 | 14896175 | 14932185 | Unsaturated fatty acid content QTL (156922) | 2 | 11271548 | 1.17E+08 |
| ENSSSCG00000030610 | 2 | 14896175 | 14932185 | Linolenic acid content QTL (156918) | 2 | 11271548 | 97878445 |
| ENSSSCG00000030610 | 2 | 14896175 | 14932185 | Saturated fatty acid content QTL (156921) | 2 | 11271548 | 97878445 |
| ENSSSCG00000030610 | 2 | 14896175 | 14932185 | cis-9-Heptadecenoic acid content QTL (156917) | 2 | 11271548 | 72689985 |
| ENSSSCG00000030610 | 2 | 14896175 | 14932185 | Palmitic acid content QTL (156868) | 2 | 11271548 | 72689985 |
| ENSSSCG00000030610 | 2 | 14896175 | 14932185 | Body weight (end of test) QTL (5199) | 2 | 14031927 | 1.34E+08 |
| ENSSSCG00000013284 | 2 | 18807523 | 18954650 | Backfat between 6th and 7th ribs QTL (3942) | 2 | 552075 | 44051051 |
| ENSSSCG00000013284 | 2 | 18807523 | 18954650 | Backfat at first rib QTL (21216) | 2 | 690094 | 62108532 |
| ENSSSCG00000013284 | 2 | 18807523 | 18954650 | Subcutanous fat area QTL (18003) | 2 | 690094 | 35884929 |
| ENSSSCG00000013284 | 2 | 18807523 | 18954650 | Fat protein content QTL (18010) | 2 | 690094 | 35884929 |
| ENSSSCG00000013284 | 2 | 18807523 | 18954650 | NADP-malate dehydrogenase activity QTL (18016) | 2 | 690094 | 35884929 |
| ENSSSCG00000013284 | 2 | 18807523 | 18954650 | Average backfat thickness QTL (311) | 2 | 690094 | 35884929 |
| ENSSSCG00000013284 | 2 | 18807523 | 18954650 | Backfat at rump QTL (21217) | 2 | 690094 | 27718807 |
| ENSSSCG00000013284 | 2 | 18807523 | 18954650 | Average backfat thickness QTL (5965) | 2 | 11271548 | 63258690 |
| ENSSSCG00000013284 | 2 | 18807523 | 18954650 | Loin muscle area QTL (38051) | 2 | 11271548 | 48306636 |
| ENSSSCG00000013284 | 2 | 18807523 | 18954650 | Average daily gain QTL (929) | 2 | 11271548 | 48306636 |
| ENSSSCG00000013284 | 2 | 18807523 | 18954650 | Carcass weight (hot) QTL (5937) | 2 | 11271548 | 35884929 |
| ENSSSCG00000013284 | 2 | 18807523 | 18954650 | Shoulder subcutaneous fat thickness QTL (5961) | 2 | 11271548 | 35884929 |
| ENSSSCG00000013284 | 2 | 18807523 | 18954650 | Backfat at last rib QTL (5966) | 2 | 11271548 | 35884929 |
| ENSSSCG00000013284 | 2 | 18807523 | 18954650 | Loin muscle area QTL (5968) | 2 | 11271548 | 35884929 |
| ENSSSCG00000013284 | 2 | 18807523 | 18954650 | Subcutanous fat area QTL (5969) | 2 | 11271548 | 35884929 |
| ENSSSCG00000013284 | 2 | 18807523 | 18954650 | Estimated carcass lean content QTL (5972) | 2 | 11271548 | 35884929 |
| ENSSSCG00000013284 | 2 | 18807523 | 18954650 | Drip loss QTL (5683) | 2 | 11271548 | 35884929 |
| ENSSSCG00000013284 | 2 | 18807523 | 18954650 | Interferon-gamma level QTL (12334) | 2 | 11271548 | 35884929 |
| ENSSSCG00000013284 | 2 | 18807523 | 18954650 | Average backfat thickness QTL (923) | 2 | 11271548 | 35884929 |
| ENSSSCG00000013284 | 2 | 18807523 | 18954650 | Carcass length QTL (924) | 2 | 11271548 | 35884929 |
| ENSSSCG00000013284 | 2 | 18807523 | 18954650 | Body weight (3 weeks) QTL (928) | 2 | 11271548 | 35884929 |
| ENSSSCG00000013284 | 2 | 18807523 | 18954650 | Loin muscle area QTL (38052) | 2 | 11271548 | 22083033 |
| ENSSSCG00000013284 | 2 | 18807523 | 18954650 | Average backfat thickness QTL (17550) | 2 | 17712433 | 43706004 |
| ENSSSCG00000013284 | 2 | 18807523 | 18954650 | Meat color a* QTL (17552) | 2 | 18862591 | 34964803 |
| ENSSSCG00000013284 | 2 | 18807523 | 18954650 | Abdominal fat weight QTL (21214) | 2 | 690094 | 1.13E+08 |
| ENSSSCG00000013284 | 2 | 18807523 | 18954650 | Average backfat thickness QTL (21212) | 2 | 690094 | 1.12E+08 |
| ENSSSCG00000013284 | 2 | 18807523 | 18954650 | Abdominal fat weight QTL (21213) | 2 | 2875395 | 1.02E+08 |
| ENSSSCG00000013284 | 2 | 18807523 | 18954650 | Backfat at mid-back QTL (5242) | 2 | 4140568 | 1.36E+08 |
| ENSSSCG00000013284 | 2 | 18807523 | 18954650 | Shoulder subcutaneous fat thickness QTL (5218) | 2 | 7936090 | 1.19E+08 |
| ENSSSCG00000013284 | 2 | 18807523 | 18954650 | Backfat at mid-back QTL (5208) | 2 | 9431295 | 1.35E+08 |
| ENSSSCG00000013284 | 2 | 18807523 | 18954650 | Estimated carcass lean content QTL (5691) | 2 | 11271548 | 1.47E+08 |
| ENSSSCG00000013284 | 2 | 18807523 | 18954650 | Shear force QTL (38074) | 2 | 11271548 | 1.17E+08 |
| ENSSSCG00000013284 | 2 | 18807523 | 18954650 | Meat color L* QTL (38076) | 2 | 11271548 | 1.17E+08 |
| ENSSSCG00000013284 | 2 | 18807523 | 18954650 | Arachidic acid content QTL (156919) | 2 | 11271548 | 1.17E+08 |
| ENSSSCG00000013284 | 2 | 18807523 | 18954650 | Arachidonic acid content QTL (156920) | 2 | 11271548 | 1.17E+08 |
| ENSSSCG00000013284 | 2 | 18807523 | 18954650 | Unsaturated fatty acid content QTL (156922) | 2 | 11271548 | 1.17E+08 |
| ENSSSCG00000013284 | 2 | 18807523 | 18954650 | Linolenic acid content QTL (156918) | 2 | 11271548 | 97878445 |
| ENSSSCG00000013284 | 2 | 18807523 | 18954650 | Saturated fatty acid content QTL (156921) | 2 | 11271548 | 97878445 |
| ENSSSCG00000013284 | 2 | 18807523 | 18954650 | cis-9-Heptadecenoic acid content QTL (156917) | 2 | 11271548 | 72689985 |
| ENSSSCG00000013284 | 2 | 18807523 | 18954650 | Palmitic acid content QTL (156868) | 2 | 11271548 | 72689985 |
| ENSSSCG00000013284 | 2 | 18807523 | 18954650 | Body weight (end of test) QTL (5199) | 2 | 14031927 | 1.34E+08 |
| ENSSSCG00000031940 | 2 | 36711609 | 36885620 | Backfat between 6th and 7th ribs QTL (3942) | 2 | 552075 | 44051051 |
| ENSSSCG00000031940 | 2 | 36711609 | 36885620 | Backfat at first rib QTL (21216) | 2 | 690094 | 62108532 |
| ENSSSCG00000031940 | 2 | 36711609 | 36885620 | Average backfat thickness QTL (5965) | 2 | 11271548 | 63258690 |
| ENSSSCG00000031940 | 2 | 36711609 | 36885620 | Loin muscle area QTL (38051) | 2 | 11271548 | 48306636 |
| ENSSSCG00000031940 | 2 | 36711609 | 36885620 | Average daily gain QTL (929) | 2 | 11271548 | 48306636 |
| ENSSSCG00000031940 | 2 | 36711609 | 36885620 | Average backfat thickness QTL (17550) | 2 | 17712433 | 43706004 |
| ENSSSCG00000031940 | 2 | 36711609 | 36885620 | Drip loss QTL (86) | 2 | 22083033 | 63258690 |
| ENSSSCG00000031940 | 2 | 36711609 | 36885620 | Drip loss QTL (3058) | 2 | 27258744 | 48306636 |
| ENSSSCG00000031940 | 2 | 36711609 | 36885620 | Alkaline phosphatase activity QTL (6354) | 2 | 31399313 | 48306636 |
| ENSSSCG00000031940 | 2 | 36711609 | 36885620 | Drip loss QTL (12665) | 2 | 32227427 | 59440165 |
| ENSSSCG00000031940 | 2 | 36711609 | 36885620 | backfat above muscle dorsi QTL (5963) | 2 | 34642758 | 53861899 |
| ENSSSCG00000031940 | 2 | 36711609 | 36885620 | pH 24 hr post-mortem (loin) QTL (902) | 2 | 35884929 | 66019069 |
| ENSSSCG00000031940 | 2 | 36711609 | 36885620 | Drip loss QTL (21848) | 2 | 35884929 | 63488721 |
| ENSSSCG00000031940 | 2 | 36711609 | 36885620 | Drip loss QTL (2937) | 2 | 35884929 | 52907268 |
| ENSSSCG00000031940 | 2 | 36711609 | 36885620 | Off-flavor score QTL (2938) | 2 | 35884929 | 52907268 |
| ENSSSCG00000031940 | 2 | 36711609 | 36885620 | Backfat at tenth rib QTL (925) | 2 | 35884929 | 48306636 |
| ENSSSCG00000031940 | 2 | 36711609 | 36885620 | Shoulder subcutaneous fat thickness QTL (926) | 2 | 35884929 | 48306636 |
| ENSSSCG00000031940 | 2 | 36711609 | 36885620 | Abdominal fat weight QTL (21214) | 2 | 690094 | 1.13E+08 |
| ENSSSCG00000031940 | 2 | 36711609 | 36885620 | Average backfat thickness QTL (21212) | 2 | 690094 | 1.12E+08 |
| ENSSSCG00000031940 | 2 | 36711609 | 36885620 | Abdominal fat weight QTL (21213) | 2 | 2875395 | 1.02E+08 |
| ENSSSCG00000031940 | 2 | 36711609 | 36885620 | Backfat at mid-back QTL (5242) | 2 | 4140568 | 1.36E+08 |
| ENSSSCG00000031940 | 2 | 36711609 | 36885620 | Shoulder subcutaneous fat thickness QTL (5218) | 2 | 7936090 | 1.19E+08 |
| ENSSSCG00000031940 | 2 | 36711609 | 36885620 | Backfat at mid-back QTL (5208) | 2 | 9431295 | 1.35E+08 |
| ENSSSCG00000031940 | 2 | 36711609 | 36885620 | Estimated carcass lean content QTL (5691) | 2 | 11271548 | 1.47E+08 |
| ENSSSCG00000031940 | 2 | 36711609 | 36885620 | Shear force QTL (38074) | 2 | 11271548 | 1.17E+08 |
| ENSSSCG00000031940 | 2 | 36711609 | 36885620 | Meat color L* QTL (38076) | 2 | 11271548 | 1.17E+08 |
| ENSSSCG00000031940 | 2 | 36711609 | 36885620 | Arachidic acid content QTL (156919) | 2 | 11271548 | 1.17E+08 |
| ENSSSCG00000031940 | 2 | 36711609 | 36885620 | Arachidonic acid content QTL (156920) | 2 | 11271548 | 1.17E+08 |
| ENSSSCG00000031940 | 2 | 36711609 | 36885620 | Unsaturated fatty acid content QTL (156922) | 2 | 11271548 | 1.17E+08 |
| ENSSSCG00000031940 | 2 | 36711609 | 36885620 | Linolenic acid content QTL (156918) | 2 | 11271548 | 97878445 |
| ENSSSCG00000031940 | 2 | 36711609 | 36885620 | Saturated fatty acid content QTL (156921) | 2 | 11271548 | 97878445 |
| ENSSSCG00000031940 | 2 | 36711609 | 36885620 | cis-9-Heptadecenoic acid content QTL (156917) | 2 | 11271548 | 72689985 |
| ENSSSCG00000031940 | 2 | 36711609 | 36885620 | Palmitic acid content QTL (156868) | 2 | 11271548 | 72689985 |
| ENSSSCG00000031940 | 2 | 36711609 | 36885620 | Body weight (end of test) QTL (5199) | 2 | 14031927 | 1.34E+08 |
| ENSSSCG00000031940 | 2 | 36711609 | 36885620 | Drip loss QTL (38075) | 2 | 22083033 | 97878445 |
| ENSSSCG00000031940 | 2 | 36711609 | 36885620 | Shoulder subcutaneous fat thickness QTL (21219) | 2 | 32894518 | 91897624 |
| ENSSSCG00000031940 | 2 | 36711609 | 36885620 | Backfat at last rib QTL (5685) | 2 | 35884929 | 1.47E+08 |
| ENSSSCG00000031940 | 2 | 36711609 | 36885620 | Cholesterol level QTL (15066) | 2 | 35884929 | 97878445 |
| ENSSSCG00000031940 | 2 | 36711609 | 36885620 | Drip loss QTL (3780) | 2 | 35884929 | 86031818 |
| ENSSSCG00000031940 | 2 | 36711609 | 36885620 | Off-flavor score QTL (3810) | 2 | 35884929 | 86031818 |
| ENSSSCG00000031940 | 2 | 36711609 | 36885620 | Average backfat thickness QTL (17998) | 2 | 35884929 | 76025443 |
| ENSSSCG00000034191 | 2 | 42448216 | 43068325 | pH 45 minutes post mortem QTL (56220) | 2 | 42812064 | 42812068 |
| ENSSSCG00000034191 | 2 | 42448216 | 43068325 | Backfat at mid-back QTL (56221) | 2 | 42812064 | 42812068 |
| ENSSSCG00000034191 | 2 | 42448216 | 43068325 | Average backfat thickness QTL (56222) | 2 | 42812064 | 42812068 |
| ENSSSCG00000034191 | 2 | 42448216 | 43068325 | Carcass length QTL (56224) | 2 | 42812064 | 42812068 |
| ENSSSCG00000034191 | 2 | 42448216 | 43068325 | Meat color score QTL (56225) | 2 | 43023572 | 43023576 |
| ENSSSCG00000034191 | 2 | 42448216 | 43068325 | Backfat between 6th and 7th ribs QTL (3942) | 2 | 552075 | 44051051 |
| ENSSSCG00000034191 | 2 | 42448216 | 43068325 | Backfat at first rib QTL (21216) | 2 | 690094 | 62108532 |
| ENSSSCG00000034191 | 2 | 42448216 | 43068325 | Average backfat thickness QTL (5965) | 2 | 11271548 | 63258690 |
| ENSSSCG00000034191 | 2 | 42448216 | 43068325 | Loin muscle area QTL (38051) | 2 | 11271548 | 48306636 |
| ENSSSCG00000034191 | 2 | 42448216 | 43068325 | Average daily gain QTL (929) | 2 | 11271548 | 48306636 |
| ENSSSCG00000034191 | 2 | 42448216 | 43068325 | Average backfat thickness QTL (17550) | 2 | 17712433 | 43706004 |
| ENSSSCG00000034191 | 2 | 42448216 | 43068325 | Drip loss QTL (86) | 2 | 22083033 | 63258690 |
| ENSSSCG00000034191 | 2 | 42448216 | 43068325 | Drip loss QTL (3058) | 2 | 27258744 | 48306636 |
| ENSSSCG00000034191 | 2 | 42448216 | 43068325 | Alkaline phosphatase activity QTL (6354) | 2 | 31399313 | 48306636 |
| ENSSSCG00000034191 | 2 | 42448216 | 43068325 | Drip loss QTL (12665) | 2 | 32227427 | 59440165 |
| ENSSSCG00000034191 | 2 | 42448216 | 43068325 | backfat above muscle dorsi QTL (5963) | 2 | 34642758 | 53861899 |
| ENSSSCG00000034191 | 2 | 42448216 | 43068325 | pH 24 hr post-mortem (loin) QTL (902) | 2 | 35884929 | 66019069 |
| ENSSSCG00000034191 | 2 | 42448216 | 43068325 | Drip loss QTL (21848) | 2 | 35884929 | 63488721 |
| ENSSSCG00000034191 | 2 | 42448216 | 43068325 | Drip loss QTL (2937) | 2 | 35884929 | 52907268 |
| ENSSSCG00000034191 | 2 | 42448216 | 43068325 | Off-flavor score QTL (2938) | 2 | 35884929 | 52907268 |
| ENSSSCG00000034191 | 2 | 42448216 | 43068325 | Backfat at tenth rib QTL (925) | 2 | 35884929 | 48306636 |
| ENSSSCG00000034191 | 2 | 42448216 | 43068325 | Shoulder subcutaneous fat thickness QTL (926) | 2 | 35884929 | 48306636 |
| ENSSSCG00000034191 | 2 | 42448216 | 43068325 | Stearic acid content QTL (15887) | 2 | 37035087 | 52907268 |
| ENSSSCG00000034191 | 2 | 42448216 | 43068325 | pH 24 hr post-mortem (loin) QTL (6447) | 2 | 39496425 | 46052326 |
| ENSSSCG00000034191 | 2 | 42448216 | 43068325 | Average backfat thickness QTL (9816) | 2 | 39496425 | 46052326 |
| ENSSSCG00000034191 | 2 | 42448216 | 43068325 | Loin muscle area QTL (9817) | 2 | 39496425 | 46052326 |
| ENSSSCG00000034191 | 2 | 42448216 | 43068325 | Backfat at last lumbar QTL (9818) | 2 | 39496425 | 46052326 |
| ENSSSCG00000034191 | 2 | 42448216 | 43068325 | Marbling QTL (9819) | 2 | 39496425 | 46052326 |
| ENSSSCG00000034191 | 2 | 42448216 | 43068325 | Backfat at tenth rib QTL (9820) | 2 | 39496425 | 46052326 |
| ENSSSCG00000034191 | 2 | 42448216 | 43068325 | Average daily gain QTL (9821) | 2 | 39496425 | 46052326 |
| ENSSSCG00000034191 | 2 | 42448216 | 43068325 | Drip loss QTL (9822) | 2 | 39496425 | 46052326 |
| ENSSSCG00000034191 | 2 | 42448216 | 43068325 | Fiber type II myosin isoform ratio QTL (9823) | 2 | 39496425 | 46052326 |
| ENSSSCG00000034191 | 2 | 42448216 | 43068325 | Leaf fat weight QTL (3867) | 2 | 42210798 | 58428026 |
| ENSSSCG00000034191 | 2 | 42448216 | 43068325 | Abdominal fat weight QTL (21214) | 2 | 690094 | 1.13E+08 |
| ENSSSCG00000034191 | 2 | 42448216 | 43068325 | Average backfat thickness QTL (21212) | 2 | 690094 | 1.12E+08 |
| ENSSSCG00000034191 | 2 | 42448216 | 43068325 | Abdominal fat weight QTL (21213) | 2 | 2875395 | 1.02E+08 |
| ENSSSCG00000034191 | 2 | 42448216 | 43068325 | Backfat at mid-back QTL (5242) | 2 | 4140568 | 1.36E+08 |
| ENSSSCG00000034191 | 2 | 42448216 | 43068325 | Shoulder subcutaneous fat thickness QTL (5218) | 2 | 7936090 | 1.19E+08 |
| ENSSSCG00000034191 | 2 | 42448216 | 43068325 | Backfat at mid-back QTL (5208) | 2 | 9431295 | 1.35E+08 |
| ENSSSCG00000034191 | 2 | 42448216 | 43068325 | Estimated carcass lean content QTL (5691) | 2 | 11271548 | 1.47E+08 |
| ENSSSCG00000034191 | 2 | 42448216 | 43068325 | Shear force QTL (38074) | 2 | 11271548 | 1.17E+08 |
| ENSSSCG00000034191 | 2 | 42448216 | 43068325 | Meat color L* QTL (38076) | 2 | 11271548 | 1.17E+08 |
| ENSSSCG00000034191 | 2 | 42448216 | 43068325 | Arachidic acid content QTL (156919) | 2 | 11271548 | 1.17E+08 |
| ENSSSCG00000034191 | 2 | 42448216 | 43068325 | Arachidonic acid content QTL (156920) | 2 | 11271548 | 1.17E+08 |
| ENSSSCG00000034191 | 2 | 42448216 | 43068325 | Unsaturated fatty acid content QTL (156922) | 2 | 11271548 | 1.17E+08 |
| ENSSSCG00000034191 | 2 | 42448216 | 43068325 | Linolenic acid content QTL (156918) | 2 | 11271548 | 97878445 |
| ENSSSCG00000034191 | 2 | 42448216 | 43068325 | Saturated fatty acid content QTL (156921) | 2 | 11271548 | 97878445 |
| ENSSSCG00000034191 | 2 | 42448216 | 43068325 | cis-9-Heptadecenoic acid content QTL (156917) | 2 | 11271548 | 72689985 |
| ENSSSCG00000034191 | 2 | 42448216 | 43068325 | Palmitic acid content QTL (156868) | 2 | 11271548 | 72689985 |
| ENSSSCG00000034191 | 2 | 42448216 | 43068325 | Body weight (end of test) QTL (5199) | 2 | 14031927 | 1.34E+08 |
| ENSSSCG00000034191 | 2 | 42448216 | 43068325 | Drip loss QTL (38075) | 2 | 22083033 | 97878445 |
| ENSSSCG00000034191 | 2 | 42448216 | 43068325 | Shoulder subcutaneous fat thickness QTL (21219) | 2 | 32894518 | 91897624 |
| ENSSSCG00000034191 | 2 | 42448216 | 43068325 | Backfat at last rib QTL (5685) | 2 | 35884929 | 1.47E+08 |
| ENSSSCG00000034191 | 2 | 42448216 | 43068325 | Cholesterol level QTL (15066) | 2 | 35884929 | 97878445 |
| ENSSSCG00000034191 | 2 | 42448216 | 43068325 | Drip loss QTL (3780) | 2 | 35884929 | 86031818 |
| ENSSSCG00000034191 | 2 | 42448216 | 43068325 | Off-flavor score QTL (3810) | 2 | 35884929 | 86031818 |
| ENSSSCG00000034191 | 2 | 42448216 | 43068325 | Average backfat thickness QTL (17998) | 2 | 35884929 | 76025443 |
| ENSSSCG00000013402 | 2 | 47448122 | 47582814 | Backfat at first rib QTL (21216) | 2 | 690094 | 62108532 |
| ENSSSCG00000013402 | 2 | 47448122 | 47582814 | Average backfat thickness QTL (5965) | 2 | 11271548 | 63258690 |
| ENSSSCG00000013402 | 2 | 47448122 | 47582814 | Loin muscle area QTL (38051) | 2 | 11271548 | 48306636 |
| ENSSSCG00000013402 | 2 | 47448122 | 47582814 | Average daily gain QTL (929) | 2 | 11271548 | 48306636 |
| ENSSSCG00000013402 | 2 | 47448122 | 47582814 | Drip loss QTL (86) | 2 | 22083033 | 63258690 |
| ENSSSCG00000013402 | 2 | 47448122 | 47582814 | Drip loss QTL (3058) | 2 | 27258744 | 48306636 |
| ENSSSCG00000013402 | 2 | 47448122 | 47582814 | Alkaline phosphatase activity QTL (6354) | 2 | 31399313 | 48306636 |
| ENSSSCG00000013402 | 2 | 47448122 | 47582814 | Drip loss QTL (12665) | 2 | 32227427 | 59440165 |
| ENSSSCG00000013402 | 2 | 47448122 | 47582814 | backfat above muscle dorsi QTL (5963) | 2 | 34642758 | 53861899 |
| ENSSSCG00000013402 | 2 | 47448122 | 47582814 | pH 24 hr post-mortem (loin) QTL (902) | 2 | 35884929 | 66019069 |
| ENSSSCG00000013402 | 2 | 47448122 | 47582814 | Drip loss QTL (21848) | 2 | 35884929 | 63488721 |
| ENSSSCG00000013402 | 2 | 47448122 | 47582814 | Drip loss QTL (2937) | 2 | 35884929 | 52907268 |
| ENSSSCG00000013402 | 2 | 47448122 | 47582814 | Off-flavor score QTL (2938) | 2 | 35884929 | 52907268 |
| ENSSSCG00000013402 | 2 | 47448122 | 47582814 | Backfat at tenth rib QTL (925) | 2 | 35884929 | 48306636 |
| ENSSSCG00000013402 | 2 | 47448122 | 47582814 | Shoulder subcutaneous fat thickness QTL (926) | 2 | 35884929 | 48306636 |
| ENSSSCG00000013402 | 2 | 47448122 | 47582814 | Stearic acid content QTL (15887) | 2 | 37035087 | 52907268 |
| ENSSSCG00000013402 | 2 | 47448122 | 47582814 | Leaf fat weight QTL (3867) | 2 | 42210798 | 58428026 |
| ENSSSCG00000013402 | 2 | 47448122 | 47582814 | Abdominal fat weight QTL (21214) | 2 | 690094 | 1.13E+08 |
| ENSSSCG00000013402 | 2 | 47448122 | 47582814 | Average backfat thickness QTL (21212) | 2 | 690094 | 1.12E+08 |
| ENSSSCG00000013402 | 2 | 47448122 | 47582814 | Abdominal fat weight QTL (21213) | 2 | 2875395 | 1.02E+08 |
| ENSSSCG00000013402 | 2 | 47448122 | 47582814 | Backfat at mid-back QTL (5242) | 2 | 4140568 | 1.36E+08 |
| ENSSSCG00000013402 | 2 | 47448122 | 47582814 | Shoulder subcutaneous fat thickness QTL (5218) | 2 | 7936090 | 1.19E+08 |
| ENSSSCG00000013402 | 2 | 47448122 | 47582814 | Backfat at mid-back QTL (5208) | 2 | 9431295 | 1.35E+08 |
| ENSSSCG00000013402 | 2 | 47448122 | 47582814 | Estimated carcass lean content QTL (5691) | 2 | 11271548 | 1.47E+08 |
| ENSSSCG00000013402 | 2 | 47448122 | 47582814 | Shear force QTL (38074) | 2 | 11271548 | 1.17E+08 |
| ENSSSCG00000013402 | 2 | 47448122 | 47582814 | Meat color L* QTL (38076) | 2 | 11271548 | 1.17E+08 |
| ENSSSCG00000013402 | 2 | 47448122 | 47582814 | Arachidic acid content QTL (156919) | 2 | 11271548 | 1.17E+08 |
| ENSSSCG00000013402 | 2 | 47448122 | 47582814 | Arachidonic acid content QTL (156920) | 2 | 11271548 | 1.17E+08 |
| ENSSSCG00000013402 | 2 | 47448122 | 47582814 | Unsaturated fatty acid content QTL (156922) | 2 | 11271548 | 1.17E+08 |
| ENSSSCG00000013402 | 2 | 47448122 | 47582814 | Linolenic acid content QTL (156918) | 2 | 11271548 | 97878445 |
| ENSSSCG00000013402 | 2 | 47448122 | 47582814 | Saturated fatty acid content QTL (156921) | 2 | 11271548 | 97878445 |
| ENSSSCG00000013402 | 2 | 47448122 | 47582814 | cis-9-Heptadecenoic acid content QTL (156917) | 2 | 11271548 | 72689985 |
| ENSSSCG00000013402 | 2 | 47448122 | 47582814 | Palmitic acid content QTL (156868) | 2 | 11271548 | 72689985 |
| ENSSSCG00000013402 | 2 | 47448122 | 47582814 | Body weight (end of test) QTL (5199) | 2 | 14031927 | 1.34E+08 |
| ENSSSCG00000013402 | 2 | 47448122 | 47582814 | Drip loss QTL (38075) | 2 | 22083033 | 97878445 |
| ENSSSCG00000013402 | 2 | 47448122 | 47582814 | Shoulder subcutaneous fat thickness QTL (21219) | 2 | 32894518 | 91897624 |
| ENSSSCG00000013402 | 2 | 47448122 | 47582814 | Backfat at last rib QTL (5685) | 2 | 35884929 | 1.47E+08 |
| ENSSSCG00000013402 | 2 | 47448122 | 47582814 | Cholesterol level QTL (15066) | 2 | 35884929 | 97878445 |
| ENSSSCG00000013402 | 2 | 47448122 | 47582814 | Drip loss QTL (3780) | 2 | 35884929 | 86031818 |
| ENSSSCG00000013402 | 2 | 47448122 | 47582814 | Off-flavor score QTL (3810) | 2 | 35884929 | 86031818 |
| ENSSSCG00000013402 | 2 | 47448122 | 47582814 | Average backfat thickness QTL (17998) | 2 | 35884929 | 76025443 |
| ENSSSCG00000013984 | 2 | 50831116 | 51006694 | Backfat at first rib QTL (21216) | 2 | 690094 | 62108532 |
| ENSSSCG00000013984 | 2 | 50831116 | 51006694 | Average backfat thickness QTL (5965) | 2 | 11271548 | 63258690 |
| ENSSSCG00000013984 | 2 | 50831116 | 51006694 | Drip loss QTL (86) | 2 | 22083033 | 63258690 |
| ENSSSCG00000013984 | 2 | 50831116 | 51006694 | Drip loss QTL (12665) | 2 | 32227427 | 59440165 |
| ENSSSCG00000013984 | 2 | 50831116 | 51006694 | backfat above muscle dorsi QTL (5963) | 2 | 34642758 | 53861899 |
| ENSSSCG00000013984 | 2 | 50831116 | 51006694 | pH 24 hr post-mortem (loin) QTL (902) | 2 | 35884929 | 66019069 |
| ENSSSCG00000013984 | 2 | 50831116 | 51006694 | Drip loss QTL (21848) | 2 | 35884929 | 63488721 |
| ENSSSCG00000013984 | 2 | 50831116 | 51006694 | Drip loss QTL (2937) | 2 | 35884929 | 52907268 |
| ENSSSCG00000013984 | 2 | 50831116 | 51006694 | Off-flavor score QTL (2938) | 2 | 35884929 | 52907268 |
| ENSSSCG00000013984 | 2 | 50831116 | 51006694 | Stearic acid content QTL (15887) | 2 | 37035087 | 52907268 |
| ENSSSCG00000013984 | 2 | 50831116 | 51006694 | Leaf fat weight QTL (3867) | 2 | 42210798 | 58428026 |
| ENSSSCG00000013984 | 2 | 50831116 | 51006694 | Loin muscle area QTL (3868) | 2 | 47616541 | 58428026 |
| ENSSSCG00000013984 | 2 | 50831116 | 51006694 | Loin muscle area QTL (903) | 2 | 48306636 | 66019069 |
| ENSSSCG00000013984 | 2 | 50831116 | 51006694 | HDL cholesterol QTL (17715) | 2 | 48306636 | 63258690 |
| ENSSSCG00000013984 | 2 | 50831116 | 51006694 | Abdominal fat weight QTL (21214) | 2 | 690094 | 1.13E+08 |
| ENSSSCG00000013984 | 2 | 50831116 | 51006694 | Average backfat thickness QTL (21212) | 2 | 690094 | 1.12E+08 |
| ENSSSCG00000013984 | 2 | 50831116 | 51006694 | Abdominal fat weight QTL (21213) | 2 | 2875395 | 1.02E+08 |
| ENSSSCG00000013984 | 2 | 50831116 | 51006694 | Backfat at mid-back QTL (5242) | 2 | 4140568 | 1.36E+08 |
| ENSSSCG00000013984 | 2 | 50831116 | 51006694 | Shoulder subcutaneous fat thickness QTL (5218) | 2 | 7936090 | 1.19E+08 |
| ENSSSCG00000013984 | 2 | 50831116 | 51006694 | Backfat at mid-back QTL (5208) | 2 | 9431295 | 1.35E+08 |
| ENSSSCG00000013984 | 2 | 50831116 | 51006694 | Estimated carcass lean content QTL (5691) | 2 | 11271548 | 1.47E+08 |
| ENSSSCG00000013984 | 2 | 50831116 | 51006694 | Shear force QTL (38074) | 2 | 11271548 | 1.17E+08 |
| ENSSSCG00000013984 | 2 | 50831116 | 51006694 | Meat color L* QTL (38076) | 2 | 11271548 | 1.17E+08 |
| ENSSSCG00000013984 | 2 | 50831116 | 51006694 | Arachidic acid content QTL (156919) | 2 | 11271548 | 1.17E+08 |
| ENSSSCG00000013984 | 2 | 50831116 | 51006694 | Arachidonic acid content QTL (156920) | 2 | 11271548 | 1.17E+08 |
| ENSSSCG00000013984 | 2 | 50831116 | 51006694 | Unsaturated fatty acid content QTL (156922) | 2 | 11271548 | 1.17E+08 |
| ENSSSCG00000013984 | 2 | 50831116 | 51006694 | Linolenic acid content QTL (156918) | 2 | 11271548 | 97878445 |
| ENSSSCG00000013984 | 2 | 50831116 | 51006694 | Saturated fatty acid content QTL (156921) | 2 | 11271548 | 97878445 |
| ENSSSCG00000013984 | 2 | 50831116 | 51006694 | cis-9-Heptadecenoic acid content QTL (156917) | 2 | 11271548 | 72689985 |
| ENSSSCG00000013984 | 2 | 50831116 | 51006694 | Palmitic acid content QTL (156868) | 2 | 11271548 | 72689985 |
| ENSSSCG00000013984 | 2 | 50831116 | 51006694 | Body weight (end of test) QTL (5199) | 2 | 14031927 | 1.34E+08 |
| ENSSSCG00000013984 | 2 | 50831116 | 51006694 | Drip loss QTL (38075) | 2 | 22083033 | 97878445 |
| ENSSSCG00000013984 | 2 | 50831116 | 51006694 | Shoulder subcutaneous fat thickness QTL (21219) | 2 | 32894518 | 91897624 |
| ENSSSCG00000013984 | 2 | 50831116 | 51006694 | Backfat at last rib QTL (5685) | 2 | 35884929 | 1.47E+08 |
| ENSSSCG00000013984 | 2 | 50831116 | 51006694 | Cholesterol level QTL (15066) | 2 | 35884929 | 97878445 |
| ENSSSCG00000013984 | 2 | 50831116 | 51006694 | Drip loss QTL (3780) | 2 | 35884929 | 86031818 |
| ENSSSCG00000013984 | 2 | 50831116 | 51006694 | Off-flavor score QTL (3810) | 2 | 35884929 | 86031818 |
| ENSSSCG00000013984 | 2 | 50831116 | 51006694 | Average backfat thickness QTL (17998) | 2 | 35884929 | 76025443 |
| ENSSSCG00000013984 | 2 | 50831116 | 51006694 | Carcass length QTL (29690) | 2 | 48306636 | 1.17E+08 |
| ENSSSCG00000013984 | 2 | 50831116 | 51006694 | androstenone, laboratory QTL (4205) | 2 | 48306636 | 92587719 |
| ENSSSCG00000013984 | 2 | 50831116 | 51006694 | Average daily gain QTL (16888) | 2 | 48306636 | 89597308 |
| ENSSSCG00000013984 | 2 | 50831116 | 51006694 | Backfat at last rib QTL (16883) | 2 | 48306636 | 76025443 |
| ENSSSCG00000013984 | 2 | 50831116 | 51006694 | Carcass weight (hot) QTL (16898) | 2 | 48306636 | 76025443 |
| ENSSSCG00000013984 | 2 | 50831116 | 51006694 | Percentage type IIa fibers QTL (4025) | 2 | 48306636 | 76025443 |
| ENSSSCG00000013984 | 2 | 50831116 | 51006694 | Average backfat thickness QTL (307) | 2 | 48306636 | 73955159 |
| ENSSSCG00000013984 | 2 | 50831116 | 51006694 | Interferon-gamma level QTL (17610) | 2 | 48306636 | 73955159 |
| ENSSSCG00000013984 | 2 | 50831116 | 51006694 | pH 24 hr post mortem (ham) QTL (7313) | 2 | 48410150 | 77336623 |
| ENSSSCG00000034569 | 2 | 51307201 | 51519405 | Backfat at first rib QTL (21216) | 2 | 690094 | 62108532 |
| ENSSSCG00000034569 | 2 | 51307201 | 51519405 | Average backfat thickness QTL (5965) | 2 | 11271548 | 63258690 |
| ENSSSCG00000034569 | 2 | 51307201 | 51519405 | Drip loss QTL (86) | 2 | 22083033 | 63258690 |
| ENSSSCG00000034569 | 2 | 51307201 | 51519405 | Drip loss QTL (12665) | 2 | 32227427 | 59440165 |
| ENSSSCG00000034569 | 2 | 51307201 | 51519405 | backfat above muscle dorsi QTL (5963) | 2 | 34642758 | 53861899 |
| ENSSSCG00000034569 | 2 | 51307201 | 51519405 | pH 24 hr post-mortem (loin) QTL (902) | 2 | 35884929 | 66019069 |
| ENSSSCG00000034569 | 2 | 51307201 | 51519405 | Drip loss QTL (21848) | 2 | 35884929 | 63488721 |
| ENSSSCG00000034569 | 2 | 51307201 | 51519405 | Drip loss QTL (2937) | 2 | 35884929 | 52907268 |
| ENSSSCG00000034569 | 2 | 51307201 | 51519405 | Off-flavor score QTL (2938) | 2 | 35884929 | 52907268 |
| ENSSSCG00000034569 | 2 | 51307201 | 51519405 | Stearic acid content QTL (15887) | 2 | 37035087 | 52907268 |
| ENSSSCG00000034569 | 2 | 51307201 | 51519405 | Leaf fat weight QTL (3867) | 2 | 42210798 | 58428026 |
| ENSSSCG00000034569 | 2 | 51307201 | 51519405 | Loin muscle area QTL (3868) | 2 | 47616541 | 58428026 |
| ENSSSCG00000034569 | 2 | 51307201 | 51519405 | Loin muscle area QTL (903) | 2 | 48306636 | 66019069 |
| ENSSSCG00000034569 | 2 | 51307201 | 51519405 | HDL cholesterol QTL (17715) | 2 | 48306636 | 63258690 |
| ENSSSCG00000034569 | 2 | 51307201 | 51519405 | Abdominal fat weight QTL (21214) | 2 | 690094 | 1.13E+08 |
| ENSSSCG00000034569 | 2 | 51307201 | 51519405 | Average backfat thickness QTL (21212) | 2 | 690094 | 1.12E+08 |
| ENSSSCG00000034569 | 2 | 51307201 | 51519405 | Abdominal fat weight QTL (21213) | 2 | 2875395 | 1.02E+08 |
| ENSSSCG00000034569 | 2 | 51307201 | 51519405 | Backfat at mid-back QTL (5242) | 2 | 4140568 | 1.36E+08 |
| ENSSSCG00000034569 | 2 | 51307201 | 51519405 | Shoulder subcutaneous fat thickness QTL (5218) | 2 | 7936090 | 1.19E+08 |
| ENSSSCG00000034569 | 2 | 51307201 | 51519405 | Backfat at mid-back QTL (5208) | 2 | 9431295 | 1.35E+08 |
| ENSSSCG00000034569 | 2 | 51307201 | 51519405 | Estimated carcass lean content QTL (5691) | 2 | 11271548 | 1.47E+08 |
| ENSSSCG00000034569 | 2 | 51307201 | 51519405 | Shear force QTL (38074) | 2 | 11271548 | 1.17E+08 |
| ENSSSCG00000034569 | 2 | 51307201 | 51519405 | Meat color L* QTL (38076) | 2 | 11271548 | 1.17E+08 |
| ENSSSCG00000034569 | 2 | 51307201 | 51519405 | Arachidic acid content QTL (156919) | 2 | 11271548 | 1.17E+08 |
| ENSSSCG00000034569 | 2 | 51307201 | 51519405 | Arachidonic acid content QTL (156920) | 2 | 11271548 | 1.17E+08 |
| ENSSSCG00000034569 | 2 | 51307201 | 51519405 | Unsaturated fatty acid content QTL (156922) | 2 | 11271548 | 1.17E+08 |
| ENSSSCG00000034569 | 2 | 51307201 | 51519405 | Linolenic acid content QTL (156918) | 2 | 11271548 | 97878445 |
| ENSSSCG00000034569 | 2 | 51307201 | 51519405 | Saturated fatty acid content QTL (156921) | 2 | 11271548 | 97878445 |
| ENSSSCG00000034569 | 2 | 51307201 | 51519405 | cis-9-Heptadecenoic acid content QTL (156917) | 2 | 11271548 | 72689985 |
| ENSSSCG00000034569 | 2 | 51307201 | 51519405 | Palmitic acid content QTL (156868) | 2 | 11271548 | 72689985 |
| ENSSSCG00000034569 | 2 | 51307201 | 51519405 | Body weight (end of test) QTL (5199) | 2 | 14031927 | 1.34E+08 |
| ENSSSCG00000034569 | 2 | 51307201 | 51519405 | Drip loss QTL (38075) | 2 | 22083033 | 97878445 |
| ENSSSCG00000034569 | 2 | 51307201 | 51519405 | Shoulder subcutaneous fat thickness QTL (21219) | 2 | 32894518 | 91897624 |
| ENSSSCG00000034569 | 2 | 51307201 | 51519405 | Backfat at last rib QTL (5685) | 2 | 35884929 | 1.47E+08 |
| ENSSSCG00000034569 | 2 | 51307201 | 51519405 | Cholesterol level QTL (15066) | 2 | 35884929 | 97878445 |
| ENSSSCG00000034569 | 2 | 51307201 | 51519405 | Drip loss QTL (3780) | 2 | 35884929 | 86031818 |
| ENSSSCG00000034569 | 2 | 51307201 | 51519405 | Off-flavor score QTL (3810) | 2 | 35884929 | 86031818 |
| ENSSSCG00000034569 | 2 | 51307201 | 51519405 | Average backfat thickness QTL (17998) | 2 | 35884929 | 76025443 |
| ENSSSCG00000034569 | 2 | 51307201 | 51519405 | Carcass length QTL (29690) | 2 | 48306636 | 1.17E+08 |
| ENSSSCG00000034569 | 2 | 51307201 | 51519405 | androstenone, laboratory QTL (4205) | 2 | 48306636 | 92587719 |
| ENSSSCG00000034569 | 2 | 51307201 | 51519405 | Average daily gain QTL (16888) | 2 | 48306636 | 89597308 |
| ENSSSCG00000034569 | 2 | 51307201 | 51519405 | Backfat at last rib QTL (16883) | 2 | 48306636 | 76025443 |
| ENSSSCG00000034569 | 2 | 51307201 | 51519405 | Carcass weight (hot) QTL (16898) | 2 | 48306636 | 76025443 |
| ENSSSCG00000034569 | 2 | 51307201 | 51519405 | Percentage type IIa fibers QTL (4025) | 2 | 48306636 | 76025443 |
| ENSSSCG00000034569 | 2 | 51307201 | 51519405 | Average backfat thickness QTL (307) | 2 | 48306636 | 73955159 |
| ENSSSCG00000034569 | 2 | 51307201 | 51519405 | Interferon-gamma level QTL (17610) | 2 | 48306636 | 73955159 |
| ENSSSCG00000034569 | 2 | 51307201 | 51519405 | pH 24 hr post mortem (ham) QTL (7313) | 2 | 48410150 | 77336623 |
| ENSSSCG00000024570 | 2 | 73747641 | 73889865 | Cholesterol level QTL (23524) | 2 | 73837974 | 73837978 |
| ENSSSCG00000024570 | 2 | 73747641 | 73889865 | LDL cholesterol QTL (23569) | 2 | 73837974 | 73837978 |
| ENSSSCG00000024570 | 2 | 73747641 | 73889865 | HDL/LDL ratio QTL (23606) | 2 | 73837974 | 73837978 |
| ENSSSCG00000024570 | 2 | 73747641 | 73889865 | Dihomo-gamma-linolenic acid content QTL (213659) | 2 | 73882466 | 73882470 |
| ENSSSCG00000024570 | 2 | 73747641 | 73889865 | LDL cholesterol QTL (23570) | 2 | 73887385 | 73887389 |
| ENSSSCG00000024570 | 2 | 73747641 | 73889865 | PH for Longissmus dorsi QTL (5825) | 2 | 67767309 | 79429911 |
| ENSSSCG00000024570 | 2 | 73747641 | 73889865 | Backfat at first rib QTL (21215) | 2 | 69584559 | 95923177 |
| ENSSSCG00000024570 | 2 | 73747641 | 73889865 | Carcass temperature (45 minutes post-mortem) QTL (21355) | 2 | 69699574 | 97878445 |
| ENSSSCG00000024570 | 2 | 73747641 | 73889865 | Off-flavor score QTL (3225) | 2 | 69699574 | 97878445 |
| ENSSSCG00000024570 | 2 | 73747641 | 73889865 | Loin muscle area QTL (670) | 2 | 70159638 | 92012640 |
| ENSSSCG00000024570 | 2 | 73747641 | 73889865 | Diameter of type IIb muscle fibers QTL (2796) | 2 | 72344938 | 98798572 |
| ENSSSCG00000024570 | 2 | 73747641 | 73889865 | Diameter of type IIa muscle fibers QTL (2797) | 2 | 72344938 | 98798572 |
| ENSSSCG00000024570 | 2 | 73747641 | 73889865 | Diameter of muscle fibers QTL (2798) | 2 | 72344938 | 98798572 |
| ENSSSCG00000024570 | 2 | 73747641 | 73889865 | Muscle moisture percentage QTL (2975) | 2 | 72689985 | 86031818 |
| ENSSSCG00000024570 | 2 | 73747641 | 73889865 | PH for Longissmus dorsi QTL (2976) | 2 | 72689985 | 86031818 |
| ENSSSCG00000024570 | 2 | 73747641 | 73889865 | Leaf fat weight QTL (21218) | 2 | 73725127 | 1.33E+08 |
| ENSSSCG00000024570 | 2 | 73747641 | 73889865 | Abdominal fat weight QTL (21214) | 2 | 690094 | 1.13E+08 |
| ENSSSCG00000024570 | 2 | 73747641 | 73889865 | Average backfat thickness QTL (21212) | 2 | 690094 | 1.12E+08 |
| ENSSSCG00000024570 | 2 | 73747641 | 73889865 | Abdominal fat weight QTL (21213) | 2 | 2875395 | 1.02E+08 |
| ENSSSCG00000024570 | 2 | 73747641 | 73889865 | Backfat at mid-back QTL (5242) | 2 | 4140568 | 1.36E+08 |
| ENSSSCG00000024570 | 2 | 73747641 | 73889865 | Shoulder subcutaneous fat thickness QTL (5218) | 2 | 7936090 | 1.19E+08 |
| ENSSSCG00000024570 | 2 | 73747641 | 73889865 | Backfat at mid-back QTL (5208) | 2 | 9431295 | 1.35E+08 |
| ENSSSCG00000024570 | 2 | 73747641 | 73889865 | Estimated carcass lean content QTL (5691) | 2 | 11271548 | 1.47E+08 |
| ENSSSCG00000024570 | 2 | 73747641 | 73889865 | Shear force QTL (38074) | 2 | 11271548 | 1.17E+08 |
| ENSSSCG00000024570 | 2 | 73747641 | 73889865 | Meat color L* QTL (38076) | 2 | 11271548 | 1.17E+08 |
| ENSSSCG00000024570 | 2 | 73747641 | 73889865 | Arachidic acid content QTL (156919) | 2 | 11271548 | 1.17E+08 |
| ENSSSCG00000024570 | 2 | 73747641 | 73889865 | Arachidonic acid content QTL (156920) | 2 | 11271548 | 1.17E+08 |
| ENSSSCG00000024570 | 2 | 73747641 | 73889865 | Unsaturated fatty acid content QTL (156922) | 2 | 11271548 | 1.17E+08 |
| ENSSSCG00000024570 | 2 | 73747641 | 73889865 | Linolenic acid content QTL (156918) | 2 | 11271548 | 97878445 |
| ENSSSCG00000024570 | 2 | 73747641 | 73889865 | Saturated fatty acid content QTL (156921) | 2 | 11271548 | 97878445 |
| ENSSSCG00000024570 | 2 | 73747641 | 73889865 | Body weight (end of test) QTL (5199) | 2 | 14031927 | 1.34E+08 |
| ENSSSCG00000024570 | 2 | 73747641 | 73889865 | Drip loss QTL (38075) | 2 | 22083033 | 97878445 |
| ENSSSCG00000024570 | 2 | 73747641 | 73889865 | Shoulder subcutaneous fat thickness QTL (21219) | 2 | 32894518 | 91897624 |
| ENSSSCG00000024570 | 2 | 73747641 | 73889865 | Backfat at last rib QTL (5685) | 2 | 35884929 | 1.47E+08 |
| ENSSSCG00000024570 | 2 | 73747641 | 73889865 | Cholesterol level QTL (15066) | 2 | 35884929 | 97878445 |
| ENSSSCG00000024570 | 2 | 73747641 | 73889865 | Drip loss QTL (3780) | 2 | 35884929 | 86031818 |
| ENSSSCG00000024570 | 2 | 73747641 | 73889865 | Off-flavor score QTL (3810) | 2 | 35884929 | 86031818 |
| ENSSSCG00000024570 | 2 | 73747641 | 73889865 | Average backfat thickness QTL (17998) | 2 | 35884929 | 76025443 |
| ENSSSCG00000024570 | 2 | 73747641 | 73889865 | Carcass length QTL (29690) | 2 | 48306636 | 1.17E+08 |
| ENSSSCG00000024570 | 2 | 73747641 | 73889865 | androstenone, laboratory QTL (4205) | 2 | 48306636 | 92587719 |
| ENSSSCG00000024570 | 2 | 73747641 | 73889865 | Average daily gain QTL (16888) | 2 | 48306636 | 89597308 |
| ENSSSCG00000024570 | 2 | 73747641 | 73889865 | Backfat at last rib QTL (16883) | 2 | 48306636 | 76025443 |
| ENSSSCG00000024570 | 2 | 73747641 | 73889865 | Carcass weight (hot) QTL (16898) | 2 | 48306636 | 76025443 |
| ENSSSCG00000024570 | 2 | 73747641 | 73889865 | Percentage type IIa fibers QTL (4025) | 2 | 48306636 | 76025443 |
| ENSSSCG00000024570 | 2 | 73747641 | 73889865 | Average backfat thickness QTL (307) | 2 | 48306636 | 73955159 |
| ENSSSCG00000024570 | 2 | 73747641 | 73889865 | Interferon-gamma level QTL (17610) | 2 | 48306636 | 73955159 |
| ENSSSCG00000024570 | 2 | 73747641 | 73889865 | pH 24 hr post mortem (ham) QTL (7313) | 2 | 48410150 | 77336623 |
| ENSSSCG00000024570 | 2 | 73747641 | 73889865 | Loin muscle depth QTL (669) | 2 | 51757110 | 93162798 |
| ENSSSCG00000024570 | 2 | 73747641 | 73889865 | Average daily gain QTL (3779) | 2 | 52907268 | 1.02E+08 |
| ENSSSCG00000024570 | 2 | 73747641 | 73889865 | Body weight (16 days) QTL (3778) | 2 | 52907268 | 86031818 |
| ENSSSCG00000024570 | 2 | 73747641 | 73889865 | Meat color b* QTL (3982) | 2 | 57496398 | 1.52E+08 |
| ENSSSCG00000024570 | 2 | 73747641 | 73889865 | Water holding capacity QTL (3984) | 2 | 57496398 | 1.52E+08 |
| ENSSSCG00000024570 | 2 | 73747641 | 73889865 | Cooking loss QTL (3985) | 2 | 57496398 | 1.03E+08 |
| ENSSSCG00000024570 | 2 | 73747641 | 73889865 | Shoulder subcutaneous fat thickness QTL (5251) | 2 | 61303421 | 1.17E+08 |
| ENSSSCG00000024570 | 2 | 73747641 | 73889865 | Backfat at last rib QTL (16835) | 2 | 63143674 | 76025443 |
| ENSSSCG00000024570 | 2 | 73747641 | 73889865 | pH for Semimembranosus QTL (5682) | 2 | 63258690 | 1.52E+08 |
| ENSSSCG00000024570 | 2 | 73747641 | 73889865 | Meat color-L QTL (82) | 2 | 63258690 | 89597308 |
| ENSSSCG00000024570 | 2 | 73747641 | 73889865 | Water holding capacity QTL (88) | 2 | 63258690 | 86031818 |
| ENSSSCG00000024570 | 2 | 73747641 | 73889865 | Body weight (slaughter) QTL (5936) | 2 | 63258690 | 73955159 |
| ENSSSCG00000024570 | 2 | 73747641 | 73889865 | Shoulder subcutaneous fat thickness QTL (5962) | 2 | 63258690 | 73955159 |
| ENSSSCG00000024570 | 2 | 73747641 | 73889865 | Carcass weight (hot) QTL (16860) | 2 | 66019069 | 76025443 |
| ENSSSCG00000024570 | 2 | 73747641 | 73889865 | Subcutanous fat area QTL (18004) | 2 | 66019069 | 76025443 |
| ENSSSCG00000024570 | 2 | 73747641 | 73889865 | pH 24 hr post-mortem (loin) QTL (906) | 2 | 66019069 | 76025443 |
| ENSSSCG00000024570 | 2 | 73747641 | 73889865 | Abdominal fat weight QTL (907) | 2 | 66019069 | 76025443 |
| ENSSSCG00000024570 | 2 | 73747641 | 73889865 | Loin muscle area QTL (913) | 2 | 66019069 | 76025443 |
| ENSSSCG00000014114 | 2 | 88254320 | 88502342 | Intramuscular fat content QTL (147267) | 2 | 88396521 | 88396525 |
| ENSSSCG00000014114 | 2 | 88254320 | 88502342 | Backfat at first rib QTL (21215) | 2 | 69584559 | 95923177 |
| ENSSSCG00000014114 | 2 | 88254320 | 88502342 | Carcass temperature (45 minutes post-mortem) QTL (21355) | 2 | 69699574 | 97878445 |
| ENSSSCG00000014114 | 2 | 88254320 | 88502342 | Off-flavor score QTL (3225) | 2 | 69699574 | 97878445 |
| ENSSSCG00000014114 | 2 | 88254320 | 88502342 | Loin muscle area QTL (670) | 2 | 70159638 | 92012640 |
| ENSSSCG00000014114 | 2 | 88254320 | 88502342 | Diameter of type IIb muscle fibers QTL (2796) | 2 | 72344938 | 98798572 |
| ENSSSCG00000014114 | 2 | 88254320 | 88502342 | Diameter of type IIa muscle fibers QTL (2797) | 2 | 72344938 | 98798572 |
| ENSSSCG00000014114 | 2 | 88254320 | 88502342 | Diameter of muscle fibers QTL (2798) | 2 | 72344938 | 98798572 |
| ENSSSCG00000014114 | 2 | 88254320 | 88502342 | Leaf fat weight QTL (21218) | 2 | 73725127 | 1.33E+08 |
| ENSSSCG00000014114 | 2 | 88254320 | 88502342 | Carcass weight (hot) QTL (16899) | 2 | 76025443 | 1.12E+08 |
| ENSSSCG00000014114 | 2 | 88254320 | 88502342 | Carcass weight (hot) QTL (7309) | 2 | 76025443 | 92702734 |
| ENSSSCG00000014114 | 2 | 88254320 | 88502342 | Loin muscle area QTL (7310) | 2 | 76025443 | 92702734 |
| ENSSSCG00000014114 | 2 | 88254320 | 88502342 | Salmonella count in liver and spleen QTL (11558) | 2 | 76025443 | 91322545 |
| ENSSSCG00000014114 | 2 | 88254320 | 88502342 | Carcass weight (cold) QTL (12743) | 2 | 76025443 | 89597308 |
| ENSSSCG00000014114 | 2 | 88254320 | 88502342 | Average backfat thickness QTL (21211) | 2 | 76600522 | 1.02E+08 |
| ENSSSCG00000014114 | 2 | 88254320 | 88502342 | Body weight (15 weeks) QTL (6240) | 2 | 79268889 | 95256085 |
| ENSSSCG00000014114 | 2 | 88254320 | 88502342 | Average instron (star probe) force QTL (4273) | 2 | 82581344 | 97659915 |
| ENSSSCG00000014114 | 2 | 88254320 | 88502342 | Abdominal fat weight QTL (21214) | 2 | 690094 | 1.13E+08 |
| ENSSSCG00000014114 | 2 | 88254320 | 88502342 | Average backfat thickness QTL (21212) | 2 | 690094 | 1.12E+08 |
| ENSSSCG00000014114 | 2 | 88254320 | 88502342 | Abdominal fat weight QTL (21213) | 2 | 2875395 | 1.02E+08 |
| ENSSSCG00000014114 | 2 | 88254320 | 88502342 | Backfat at mid-back QTL (5242) | 2 | 4140568 | 1.36E+08 |
| ENSSSCG00000014114 | 2 | 88254320 | 88502342 | Shoulder subcutaneous fat thickness QTL (5218) | 2 | 7936090 | 1.19E+08 |
| ENSSSCG00000014114 | 2 | 88254320 | 88502342 | Backfat at mid-back QTL (5208) | 2 | 9431295 | 1.35E+08 |
| ENSSSCG00000014114 | 2 | 88254320 | 88502342 | Estimated carcass lean content QTL (5691) | 2 | 11271548 | 1.47E+08 |
| ENSSSCG00000014114 | 2 | 88254320 | 88502342 | Shear force QTL (38074) | 2 | 11271548 | 1.17E+08 |
| ENSSSCG00000014114 | 2 | 88254320 | 88502342 | Meat color L* QTL (38076) | 2 | 11271548 | 1.17E+08 |
| ENSSSCG00000014114 | 2 | 88254320 | 88502342 | Arachidic acid content QTL (156919) | 2 | 11271548 | 1.17E+08 |
| ENSSSCG00000014114 | 2 | 88254320 | 88502342 | Arachidonic acid content QTL (156920) | 2 | 11271548 | 1.17E+08 |
| ENSSSCG00000014114 | 2 | 88254320 | 88502342 | Unsaturated fatty acid content QTL (156922) | 2 | 11271548 | 1.17E+08 |
| ENSSSCG00000014114 | 2 | 88254320 | 88502342 | Linolenic acid content QTL (156918) | 2 | 11271548 | 97878445 |
| ENSSSCG00000014114 | 2 | 88254320 | 88502342 | Saturated fatty acid content QTL (156921) | 2 | 11271548 | 97878445 |
| ENSSSCG00000014114 | 2 | 88254320 | 88502342 | Body weight (end of test) QTL (5199) | 2 | 14031927 | 1.34E+08 |
| ENSSSCG00000014114 | 2 | 88254320 | 88502342 | Drip loss QTL (38075) | 2 | 22083033 | 97878445 |
| ENSSSCG00000014114 | 2 | 88254320 | 88502342 | Shoulder subcutaneous fat thickness QTL (21219) | 2 | 32894518 | 91897624 |
| ENSSSCG00000014114 | 2 | 88254320 | 88502342 | Backfat at last rib QTL (5685) | 2 | 35884929 | 1.47E+08 |
| ENSSSCG00000014114 | 2 | 88254320 | 88502342 | Cholesterol level QTL (15066) | 2 | 35884929 | 97878445 |
| ENSSSCG00000014114 | 2 | 88254320 | 88502342 | Carcass length QTL (29690) | 2 | 48306636 | 1.17E+08 |
| ENSSSCG00000014114 | 2 | 88254320 | 88502342 | androstenone, laboratory QTL (4205) | 2 | 48306636 | 92587719 |
| ENSSSCG00000014114 | 2 | 88254320 | 88502342 | Average daily gain QTL (16888) | 2 | 48306636 | 89597308 |
| ENSSSCG00000014114 | 2 | 88254320 | 88502342 | Loin muscle depth QTL (669) | 2 | 51757110 | 93162798 |
| ENSSSCG00000014114 | 2 | 88254320 | 88502342 | Average daily gain QTL (3779) | 2 | 52907268 | 1.02E+08 |
| ENSSSCG00000014114 | 2 | 88254320 | 88502342 | Meat color b* QTL (3982) | 2 | 57496398 | 1.52E+08 |
| ENSSSCG00000014114 | 2 | 88254320 | 88502342 | Water holding capacity QTL (3984) | 2 | 57496398 | 1.52E+08 |
| ENSSSCG00000014114 | 2 | 88254320 | 88502342 | Cooking loss QTL (3985) | 2 | 57496398 | 1.03E+08 |
| ENSSSCG00000014114 | 2 | 88254320 | 88502342 | Shoulder subcutaneous fat thickness QTL (5251) | 2 | 61303421 | 1.17E+08 |
| ENSSSCG00000014114 | 2 | 88254320 | 88502342 | pH for Semimembranosus QTL (5682) | 2 | 63258690 | 1.52E+08 |
| ENSSSCG00000014114 | 2 | 88254320 | 88502342 | Meat color-L QTL (82) | 2 | 63258690 | 89597308 |
| ENSSSCG00000014114 | 2 | 88254320 | 88502342 | Shear force QTL (5684) | 2 | 73955159 | 1.52E+08 |
| ENSSSCG00000014114 | 2 | 88254320 | 88502342 | pH 24 hr post-mortem (loin) QTL (12102) | 2 | 73955159 | 1.52E+08 |
| ENSSSCG00000014114 | 2 | 88254320 | 88502342 | Cooking loss QTL (12103) | 2 | 73955159 | 1.52E+08 |
| ENSSSCG00000014114 | 2 | 88254320 | 88502342 | Cooking loss QTL (12104) | 2 | 73955159 | 1.52E+08 |
| ENSSSCG00000014114 | 2 | 88254320 | 88502342 | Intramuscular fat content QTL (12105) | 2 | 73955159 | 1.52E+08 |
| ENSSSCG00000014114 | 2 | 88254320 | 88502342 | Shear force QTL (12106) | 2 | 73955159 | 1.52E+08 |
| ENSSSCG00000014114 | 2 | 88254320 | 88502342 | Estimated carcass lean content QTL (12108) | 2 | 73955159 | 1.52E+08 |
| ENSSSCG00000014126 | 2 | 89250189 | 89453936 | Backfat at first rib QTL (21215) | 2 | 69584559 | 95923177 |
| ENSSSCG00000014126 | 2 | 89250189 | 89453936 | Carcass temperature (45 minutes post-mortem) QTL (21355) | 2 | 69699574 | 97878445 |
| ENSSSCG00000014126 | 2 | 89250189 | 89453936 | Off-flavor score QTL (3225) | 2 | 69699574 | 97878445 |
| ENSSSCG00000014126 | 2 | 89250189 | 89453936 | Loin muscle area QTL (670) | 2 | 70159638 | 92012640 |
| ENSSSCG00000014126 | 2 | 89250189 | 89453936 | Diameter of type IIb muscle fibers QTL (2796) | 2 | 72344938 | 98798572 |
| ENSSSCG00000014126 | 2 | 89250189 | 89453936 | Diameter of type IIa muscle fibers QTL (2797) | 2 | 72344938 | 98798572 |
| ENSSSCG00000014126 | 2 | 89250189 | 89453936 | Diameter of muscle fibers QTL (2798) | 2 | 72344938 | 98798572 |
| ENSSSCG00000014126 | 2 | 89250189 | 89453936 | Leaf fat weight QTL (21218) | 2 | 73725127 | 1.33E+08 |
| ENSSSCG00000014126 | 2 | 89250189 | 89453936 | Carcass weight (hot) QTL (16899) | 2 | 76025443 | 1.12E+08 |
| ENSSSCG00000014126 | 2 | 89250189 | 89453936 | Carcass weight (hot) QTL (7309) | 2 | 76025443 | 92702734 |
| ENSSSCG00000014126 | 2 | 89250189 | 89453936 | Loin muscle area QTL (7310) | 2 | 76025443 | 92702734 |
| ENSSSCG00000014126 | 2 | 89250189 | 89453936 | Salmonella count in liver and spleen QTL (11558) | 2 | 76025443 | 91322545 |
| ENSSSCG00000014126 | 2 | 89250189 | 89453936 | Carcass weight (cold) QTL (12743) | 2 | 76025443 | 89597308 |
| ENSSSCG00000014126 | 2 | 89250189 | 89453936 | Average backfat thickness QTL (21211) | 2 | 76600522 | 1.02E+08 |
| ENSSSCG00000014126 | 2 | 89250189 | 89453936 | Body weight (15 weeks) QTL (6240) | 2 | 79268889 | 95256085 |
| ENSSSCG00000014126 | 2 | 89250189 | 89453936 | Average instron (star probe) force QTL (4273) | 2 | 82581344 | 97659915 |
| ENSSSCG00000014126 | 2 | 89250189 | 89453936 | Abdominal fat weight QTL (21214) | 2 | 690094 | 1.13E+08 |
| ENSSSCG00000014126 | 2 | 89250189 | 89453936 | Average backfat thickness QTL (21212) | 2 | 690094 | 1.12E+08 |
| ENSSSCG00000014126 | 2 | 89250189 | 89453936 | Abdominal fat weight QTL (21213) | 2 | 2875395 | 1.02E+08 |
| ENSSSCG00000014126 | 2 | 89250189 | 89453936 | Backfat at mid-back QTL (5242) | 2 | 4140568 | 1.36E+08 |
| ENSSSCG00000014126 | 2 | 89250189 | 89453936 | Shoulder subcutaneous fat thickness QTL (5218) | 2 | 7936090 | 1.19E+08 |
| ENSSSCG00000014126 | 2 | 89250189 | 89453936 | Backfat at mid-back QTL (5208) | 2 | 9431295 | 1.35E+08 |
| ENSSSCG00000014126 | 2 | 89250189 | 89453936 | Estimated carcass lean content QTL (5691) | 2 | 11271548 | 1.47E+08 |
| ENSSSCG00000014126 | 2 | 89250189 | 89453936 | Shear force QTL (38074) | 2 | 11271548 | 1.17E+08 |
| ENSSSCG00000014126 | 2 | 89250189 | 89453936 | Meat color L* QTL (38076) | 2 | 11271548 | 1.17E+08 |
| ENSSSCG00000014126 | 2 | 89250189 | 89453936 | Arachidic acid content QTL (156919) | 2 | 11271548 | 1.17E+08 |
| ENSSSCG00000014126 | 2 | 89250189 | 89453936 | Arachidonic acid content QTL (156920) | 2 | 11271548 | 1.17E+08 |
| ENSSSCG00000014126 | 2 | 89250189 | 89453936 | Unsaturated fatty acid content QTL (156922) | 2 | 11271548 | 1.17E+08 |
| ENSSSCG00000014126 | 2 | 89250189 | 89453936 | Linolenic acid content QTL (156918) | 2 | 11271548 | 97878445 |
| ENSSSCG00000014126 | 2 | 89250189 | 89453936 | Saturated fatty acid content QTL (156921) | 2 | 11271548 | 97878445 |
| ENSSSCG00000014126 | 2 | 89250189 | 89453936 | Body weight (end of test) QTL (5199) | 2 | 14031927 | 1.34E+08 |
| ENSSSCG00000014126 | 2 | 89250189 | 89453936 | Drip loss QTL (38075) | 2 | 22083033 | 97878445 |
| ENSSSCG00000014126 | 2 | 89250189 | 89453936 | Shoulder subcutaneous fat thickness QTL (21219) | 2 | 32894518 | 91897624 |
| ENSSSCG00000014126 | 2 | 89250189 | 89453936 | Backfat at last rib QTL (5685) | 2 | 35884929 | 1.47E+08 |
| ENSSSCG00000014126 | 2 | 89250189 | 89453936 | Cholesterol level QTL (15066) | 2 | 35884929 | 97878445 |
| ENSSSCG00000014126 | 2 | 89250189 | 89453936 | Carcass length QTL (29690) | 2 | 48306636 | 1.17E+08 |
| ENSSSCG00000014126 | 2 | 89250189 | 89453936 | androstenone, laboratory QTL (4205) | 2 | 48306636 | 92587719 |
| ENSSSCG00000014126 | 2 | 89250189 | 89453936 | Average daily gain QTL (16888) | 2 | 48306636 | 89597308 |
| ENSSSCG00000014126 | 2 | 89250189 | 89453936 | Loin muscle depth QTL (669) | 2 | 51757110 | 93162798 |
| ENSSSCG00000014126 | 2 | 89250189 | 89453936 | Average daily gain QTL (3779) | 2 | 52907268 | 1.02E+08 |
| ENSSSCG00000014126 | 2 | 89250189 | 89453936 | Meat color b* QTL (3982) | 2 | 57496398 | 1.52E+08 |
| ENSSSCG00000014126 | 2 | 89250189 | 89453936 | Water holding capacity QTL (3984) | 2 | 57496398 | 1.52E+08 |
| ENSSSCG00000014126 | 2 | 89250189 | 89453936 | Cooking loss QTL (3985) | 2 | 57496398 | 1.03E+08 |
| ENSSSCG00000014126 | 2 | 89250189 | 89453936 | Shoulder subcutaneous fat thickness QTL (5251) | 2 | 61303421 | 1.17E+08 |
| ENSSSCG00000014126 | 2 | 89250189 | 89453936 | pH for Semimembranosus QTL (5682) | 2 | 63258690 | 1.52E+08 |
| ENSSSCG00000014126 | 2 | 89250189 | 89453936 | Meat color-L QTL (82) | 2 | 63258690 | 89597308 |
| ENSSSCG00000014126 | 2 | 89250189 | 89453936 | Shear force QTL (5684) | 2 | 73955159 | 1.52E+08 |
| ENSSSCG00000014126 | 2 | 89250189 | 89453936 | pH 24 hr post-mortem (loin) QTL (12102) | 2 | 73955159 | 1.52E+08 |
| ENSSSCG00000014126 | 2 | 89250189 | 89453936 | Cooking loss QTL (12103) | 2 | 73955159 | 1.52E+08 |
| ENSSSCG00000014126 | 2 | 89250189 | 89453936 | Cooking loss QTL (12104) | 2 | 73955159 | 1.52E+08 |
| ENSSSCG00000014126 | 2 | 89250189 | 89453936 | Intramuscular fat content QTL (12105) | 2 | 73955159 | 1.52E+08 |
| ENSSSCG00000014126 | 2 | 89250189 | 89453936 | Shear force QTL (12106) | 2 | 73955159 | 1.52E+08 |
| ENSSSCG00000014126 | 2 | 89250189 | 89453936 | Estimated carcass lean content QTL (12108) | 2 | 73955159 | 1.52E+08 |
| ENSSSCG00000026606 | 2 | 1.41E+08 | 1.41E+08 | Meat color score QTL (2978) | 2 | 1.37E+08 | 1.47E+08 |
| ENSSSCG00000026606 | 2 | 1.41E+08 | 1.41E+08 | Average daily gain QTL (171) | 2 | 1.39E+08 | 1.53E+08 |
| ENSSSCG00000026606 | 2 | 1.41E+08 | 1.41E+08 | Estimated carcass lean content QTL (5691) | 2 | 11271548 | 1.47E+08 |
| ENSSSCG00000026606 | 2 | 1.41E+08 | 1.41E+08 | Backfat at last rib QTL (5685) | 2 | 35884929 | 1.47E+08 |
| ENSSSCG00000026606 | 2 | 1.41E+08 | 1.41E+08 | Meat color b* QTL (3982) | 2 | 57496398 | 1.52E+08 |
| ENSSSCG00000026606 | 2 | 1.41E+08 | 1.41E+08 | Water holding capacity QTL (3984) | 2 | 57496398 | 1.52E+08 |
| ENSSSCG00000026606 | 2 | 1.41E+08 | 1.41E+08 | pH for Semimembranosus QTL (5682) | 2 | 63258690 | 1.52E+08 |
| ENSSSCG00000026606 | 2 | 1.41E+08 | 1.41E+08 | Shear force QTL (5684) | 2 | 73955159 | 1.52E+08 |
| ENSSSCG00000026606 | 2 | 1.41E+08 | 1.41E+08 | pH 24 hr post-mortem (loin) QTL (12102) | 2 | 73955159 | 1.52E+08 |
| ENSSSCG00000026606 | 2 | 1.41E+08 | 1.41E+08 | Cooking loss QTL (12103) | 2 | 73955159 | 1.52E+08 |
| ENSSSCG00000026606 | 2 | 1.41E+08 | 1.41E+08 | Cooking loss QTL (12104) | 2 | 73955159 | 1.52E+08 |
| ENSSSCG00000026606 | 2 | 1.41E+08 | 1.41E+08 | Intramuscular fat content QTL (12105) | 2 | 73955159 | 1.52E+08 |
| ENSSSCG00000026606 | 2 | 1.41E+08 | 1.41E+08 | Shear force QTL (12106) | 2 | 73955159 | 1.52E+08 |
| ENSSSCG00000026606 | 2 | 1.41E+08 | 1.41E+08 | Estimated carcass lean content QTL (12108) | 2 | 73955159 | 1.52E+08 |
| ENSSSCG00000026606 | 2 | 1.41E+08 | 1.41E+08 | Drip loss QTL (85) | 2 | 1.03E+08 | 1.47E+08 |
| ENSSSCG00000026606 | 2 | 1.41E+08 | 1.41E+08 | Percentage type IIb fibers QTL (7040) | 2 | 1.12E+08 | 1.47E+08 |
| ENSSSCG00000026606 | 2 | 1.41E+08 | 1.41E+08 | Meat color-L QTL (83) | 2 | 1.24E+08 | 1.52E+08 |
| ENSSSCG00000026606 | 2 | 1.41E+08 | 1.41E+08 | Meat color-L QTL (84) | 2 | 1.24E+08 | 1.52E+08 |
| ENSSSCG00000026606 | 2 | 1.41E+08 | 1.41E+08 | Average daily gain QTL (5933) | 2 | 1.24E+08 | 1.47E+08 |
| ENSSSCG00000026606 | 2 | 1.41E+08 | 1.41E+08 | Triglyceride level QTL (17714) | 2 | 1.24E+08 | 1.47E+08 |
| ENSSSCG00000026606 | 2 | 1.41E+08 | 1.41E+08 | Meat color-L QTL (3802) | 2 | 1.24E+08 | 1.47E+08 |
| ENSSSCG00000026606 | 2 | 1.41E+08 | 1.41E+08 | pH 48 hr post-mortem (loin) QTL (3803) | 2 | 1.24E+08 | 1.47E+08 |
| ENSSSCG00000026606 | 2 | 1.41E+08 | 1.41E+08 | Water holding capacity QTL (87) | 2 | 1.24E+08 | 1.47E+08 |
| ENSSSCG00000026606 | 2 | 1.41E+08 | 1.41E+08 | Lactate level QTL (6359) | 2 | 1.32E+08 | 1.47E+08 |
| ENSSSCG00000014364 | 2 | 1.42E+08 | 1.42E+08 | Shear force QTL (5555) | 2 | 1.42E+08 | 1.42E+08 |
| ENSSSCG00000014364 | 2 | 1.42E+08 | 1.42E+08 | Meat color score QTL (2978) | 2 | 1.37E+08 | 1.47E+08 |
| ENSSSCG00000014364 | 2 | 1.42E+08 | 1.42E+08 | Average daily gain QTL (171) | 2 | 1.39E+08 | 1.53E+08 |
| ENSSSCG00000014364 | 2 | 1.42E+08 | 1.42E+08 | Estimated carcass lean content QTL (5691) | 2 | 11271548 | 1.47E+08 |
| ENSSSCG00000014364 | 2 | 1.42E+08 | 1.42E+08 | Backfat at last rib QTL (5685) | 2 | 35884929 | 1.47E+08 |
| ENSSSCG00000014364 | 2 | 1.42E+08 | 1.42E+08 | Meat color b* QTL (3982) | 2 | 57496398 | 1.52E+08 |
| ENSSSCG00000014364 | 2 | 1.42E+08 | 1.42E+08 | Water holding capacity QTL (3984) | 2 | 57496398 | 1.52E+08 |
| ENSSSCG00000014364 | 2 | 1.42E+08 | 1.42E+08 | pH for Semimembranosus QTL (5682) | 2 | 63258690 | 1.52E+08 |
| ENSSSCG00000014364 | 2 | 1.42E+08 | 1.42E+08 | Shear force QTL (5684) | 2 | 73955159 | 1.52E+08 |
| ENSSSCG00000014364 | 2 | 1.42E+08 | 1.42E+08 | pH 24 hr post-mortem (loin) QTL (12102) | 2 | 73955159 | 1.52E+08 |
| ENSSSCG00000014364 | 2 | 1.42E+08 | 1.42E+08 | Cooking loss QTL (12103) | 2 | 73955159 | 1.52E+08 |
| ENSSSCG00000014364 | 2 | 1.42E+08 | 1.42E+08 | Cooking loss QTL (12104) | 2 | 73955159 | 1.52E+08 |
| ENSSSCG00000014364 | 2 | 1.42E+08 | 1.42E+08 | Intramuscular fat content QTL (12105) | 2 | 73955159 | 1.52E+08 |
| ENSSSCG00000014364 | 2 | 1.42E+08 | 1.42E+08 | Shear force QTL (12106) | 2 | 73955159 | 1.52E+08 |
| ENSSSCG00000014364 | 2 | 1.42E+08 | 1.42E+08 | Estimated carcass lean content QTL (12108) | 2 | 73955159 | 1.52E+08 |
| ENSSSCG00000014364 | 2 | 1.42E+08 | 1.42E+08 | Drip loss QTL (85) | 2 | 1.03E+08 | 1.47E+08 |
| ENSSSCG00000014364 | 2 | 1.42E+08 | 1.42E+08 | Percentage type IIb fibers QTL (7040) | 2 | 1.12E+08 | 1.47E+08 |
| ENSSSCG00000014364 | 2 | 1.42E+08 | 1.42E+08 | Meat color-L QTL (83) | 2 | 1.24E+08 | 1.52E+08 |
| ENSSSCG00000014364 | 2 | 1.42E+08 | 1.42E+08 | Meat color-L QTL (84) | 2 | 1.24E+08 | 1.52E+08 |
| ENSSSCG00000014364 | 2 | 1.42E+08 | 1.42E+08 | Average daily gain QTL (5933) | 2 | 1.24E+08 | 1.47E+08 |
| ENSSSCG00000014364 | 2 | 1.42E+08 | 1.42E+08 | Triglyceride level QTL (17714) | 2 | 1.24E+08 | 1.47E+08 |
| ENSSSCG00000014364 | 2 | 1.42E+08 | 1.42E+08 | Meat color-L QTL (3802) | 2 | 1.24E+08 | 1.47E+08 |
| ENSSSCG00000014364 | 2 | 1.42E+08 | 1.42E+08 | pH 48 hr post-mortem (loin) QTL (3803) | 2 | 1.24E+08 | 1.47E+08 |
| ENSSSCG00000014364 | 2 | 1.42E+08 | 1.42E+08 | Water holding capacity QTL (87) | 2 | 1.24E+08 | 1.47E+08 |
| ENSSSCG00000014364 | 2 | 1.42E+08 | 1.42E+08 | Lactate level QTL (6359) | 2 | 1.32E+08 | 1.47E+08 |
| ENSSSCG00000014406 | 2 | 1.47E+08 | 1.47E+08 | Meat color score QTL (2978) | 2 | 1.37E+08 | 1.47E+08 |
| ENSSSCG00000014406 | 2 | 1.47E+08 | 1.47E+08 | Average daily gain QTL (171) | 2 | 1.39E+08 | 1.53E+08 |
| ENSSSCG00000014406 | 2 | 1.47E+08 | 1.47E+08 | Estimated carcass lean content QTL (5691) | 2 | 11271548 | 1.47E+08 |
| ENSSSCG00000014406 | 2 | 1.47E+08 | 1.47E+08 | Backfat at last rib QTL (5685) | 2 | 35884929 | 1.47E+08 |
| ENSSSCG00000014406 | 2 | 1.47E+08 | 1.47E+08 | Meat color b* QTL (3982) | 2 | 57496398 | 1.52E+08 |
| ENSSSCG00000014406 | 2 | 1.47E+08 | 1.47E+08 | Water holding capacity QTL (3984) | 2 | 57496398 | 1.52E+08 |
| ENSSSCG00000014406 | 2 | 1.47E+08 | 1.47E+08 | pH for Semimembranosus QTL (5682) | 2 | 63258690 | 1.52E+08 |
| ENSSSCG00000014406 | 2 | 1.47E+08 | 1.47E+08 | Shear force QTL (5684) | 2 | 73955159 | 1.52E+08 |
| ENSSSCG00000014406 | 2 | 1.47E+08 | 1.47E+08 | pH 24 hr post-mortem (loin) QTL (12102) | 2 | 73955159 | 1.52E+08 |
| ENSSSCG00000014406 | 2 | 1.47E+08 | 1.47E+08 | Cooking loss QTL (12103) | 2 | 73955159 | 1.52E+08 |
| ENSSSCG00000014406 | 2 | 1.47E+08 | 1.47E+08 | Cooking loss QTL (12104) | 2 | 73955159 | 1.52E+08 |
| ENSSSCG00000014406 | 2 | 1.47E+08 | 1.47E+08 | Intramuscular fat content QTL (12105) | 2 | 73955159 | 1.52E+08 |
| ENSSSCG00000014406 | 2 | 1.47E+08 | 1.47E+08 | Shear force QTL (12106) | 2 | 73955159 | 1.52E+08 |
| ENSSSCG00000014406 | 2 | 1.47E+08 | 1.47E+08 | Estimated carcass lean content QTL (12108) | 2 | 73955159 | 1.52E+08 |
| ENSSSCG00000014406 | 2 | 1.47E+08 | 1.47E+08 | Drip loss QTL (85) | 2 | 1.03E+08 | 1.47E+08 |
| ENSSSCG00000014406 | 2 | 1.47E+08 | 1.47E+08 | Percentage type IIb fibers QTL (7040) | 2 | 1.12E+08 | 1.47E+08 |
| ENSSSCG00000014406 | 2 | 1.47E+08 | 1.47E+08 | Meat color-L QTL (83) | 2 | 1.24E+08 | 1.52E+08 |
| ENSSSCG00000014406 | 2 | 1.47E+08 | 1.47E+08 | Meat color-L QTL (84) | 2 | 1.24E+08 | 1.52E+08 |
| ENSSSCG00000014406 | 2 | 1.47E+08 | 1.47E+08 | Average daily gain QTL (5933) | 2 | 1.24E+08 | 1.47E+08 |
| ENSSSCG00000014406 | 2 | 1.47E+08 | 1.47E+08 | Triglyceride level QTL (17714) | 2 | 1.24E+08 | 1.47E+08 |
| ENSSSCG00000014406 | 2 | 1.47E+08 | 1.47E+08 | Meat color-L QTL (3802) | 2 | 1.24E+08 | 1.47E+08 |
| ENSSSCG00000014406 | 2 | 1.47E+08 | 1.47E+08 | pH 48 hr post-mortem (loin) QTL (3803) | 2 | 1.24E+08 | 1.47E+08 |
| ENSSSCG00000014406 | 2 | 1.47E+08 | 1.47E+08 | Water holding capacity QTL (87) | 2 | 1.24E+08 | 1.47E+08 |
| ENSSSCG00000014406 | 2 | 1.47E+08 | 1.47E+08 | Lactate level QTL (6359) | 2 | 1.32E+08 | 1.47E+08 |
| ENSSSCG00000014416 | 2 | 1.48E+08 | 1.48E+08 | Average daily gain QTL (171) | 2 | 1.39E+08 | 1.53E+08 |
| ENSSSCG00000014416 | 2 | 1.48E+08 | 1.48E+08 | Meat color b* QTL (3982) | 2 | 57496398 | 1.52E+08 |
| ENSSSCG00000014416 | 2 | 1.48E+08 | 1.48E+08 | Water holding capacity QTL (3984) | 2 | 57496398 | 1.52E+08 |
| ENSSSCG00000014416 | 2 | 1.48E+08 | 1.48E+08 | pH for Semimembranosus QTL (5682) | 2 | 63258690 | 1.52E+08 |
| ENSSSCG00000014416 | 2 | 1.48E+08 | 1.48E+08 | Shear force QTL (5684) | 2 | 73955159 | 1.52E+08 |
| ENSSSCG00000014416 | 2 | 1.48E+08 | 1.48E+08 | pH 24 hr post-mortem (loin) QTL (12102) | 2 | 73955159 | 1.52E+08 |
| ENSSSCG00000014416 | 2 | 1.48E+08 | 1.48E+08 | Cooking loss QTL (12103) | 2 | 73955159 | 1.52E+08 |
| ENSSSCG00000014416 | 2 | 1.48E+08 | 1.48E+08 | Cooking loss QTL (12104) | 2 | 73955159 | 1.52E+08 |
| ENSSSCG00000014416 | 2 | 1.48E+08 | 1.48E+08 | Intramuscular fat content QTL (12105) | 2 | 73955159 | 1.52E+08 |
| ENSSSCG00000014416 | 2 | 1.48E+08 | 1.48E+08 | Shear force QTL (12106) | 2 | 73955159 | 1.52E+08 |
| ENSSSCG00000014416 | 2 | 1.48E+08 | 1.48E+08 | Estimated carcass lean content QTL (12108) | 2 | 73955159 | 1.52E+08 |
| ENSSSCG00000014416 | 2 | 1.48E+08 | 1.48E+08 | Meat color-L QTL (83) | 2 | 1.24E+08 | 1.52E+08 |
| ENSSSCG00000014416 | 2 | 1.48E+08 | 1.48E+08 | Meat color-L QTL (84) | 2 | 1.24E+08 | 1.52E+08 |
| ENSSSCG00000007805 | 3 | 18547976 | 18568313 | Carcass weight (hot) QTL (16861) | 3 | 6267426 | 43461008 |
| ENSSSCG00000007805 | 3 | 18547976 | 18568313 | Carcass temperature (45 minutes post-mortem) QTL (21356) | 3 | 12740342 | 43461008 |
| ENSSSCG00000007805 | 3 | 18547976 | 18568313 | Backfat at tenth rib QTL (2743) | 3 | 12740342 | 43461008 |
| ENSSSCG00000007805 | 3 | 18547976 | 18568313 | pH 24 hr post-mortem (loin) QTL (300) | 3 | 17774833 | 53838222 |
| ENSSSCG00000007805 | 3 | 18547976 | 18568313 | Average daily gain QTL (497) | 3 | 17774833 | 43461008 |
| ENSSSCG00000007805 | 3 | 18547976 | 18568313 | Number of muscle fibers per unit area QTL (2802) | 3 | 18288556 | 62160543 |
| ENSSSCG00000007805 | 3 | 18547976 | 18568313 | Number of capillaries per type I fiber QTL (2803) | 3 | 18288556 | 62160543 |
| ENSSSCG00000007805 | 3 | 18547976 | 18568313 | Drip loss QTL (5692) | 3 | 18288556 | 62160543 |
| ENSSSCG00000007805 | 3 | 18547976 | 18568313 | HDL cholesterol QTL (17716) | 3 | 18288556 | 62160543 |
| ENSSSCG00000007805 | 3 | 18547976 | 18568313 | Haptoglobin concentration QTL (9642) | 3 | 18288556 | 62160543 |
| ENSSSCG00000007805 | 3 | 18547976 | 18568313 | Carcass length QTL (875) | 3 | 18288556 | 43461008 |
| ENSSSCG00000007805 | 3 | 18547976 | 18568313 | Body weight (slaughter) QTL (890) | 3 | 18288556 | 43461008 |
| ENSSSCG00000007805 | 3 | 18547976 | 18568313 | Average daily gain QTL (891) | 3 | 18288556 | 43461008 |
| ENSSSCG00000007805 | 3 | 18547976 | 18568313 | Average daily gain QTL (3667) | 3 | 18288556 | 43461008 |
| ENSSSCG00000007805 | 3 | 18547976 | 18568313 | Backfat at last rib QTL (3668) | 3 | 18288556 | 43461008 |
| ENSSSCG00000007805 | 3 | 18547976 | 18568313 | PH for Longissmus dorsi QTL (3678) | 3 | 18288556 | 43461008 |
| ENSSSCG00000007805 | 3 | 18547976 | 18568313 | Carcass length QTL (3679) | 3 | 18288556 | 43461008 |
| ENSSSCG00000007805 | 3 | 18547976 | 18568313 | PH for Longissmus dorsi QTL (3680) | 3 | 18288556 | 43461008 |
| ENSSSCG00000007805 | 3 | 18547976 | 18568313 | Average daily gain QTL (4119) | 3 | 18288556 | 43461008 |
| ENSSSCG00000007805 | 3 | 18547976 | 18568313 | Average daily gain QTL (4120) | 3 | 18288556 | 43461008 |
| ENSSSCG00000007805 | 3 | 18547976 | 18568313 | backfat at P2 position QTL (4121) | 3 | 18288556 | 43461008 |
| ENSSSCG00000007805 | 3 | 18547976 | 18568313 | Carcass length QTL (4122) | 3 | 18288556 | 43461008 |
| ENSSSCG00000007805 | 3 | 18547976 | 18568313 | PH for Longissmus dorsi QTL (4123) | 3 | 18288556 | 43461008 |
| ENSSSCG00000007805 | 3 | 18547976 | 18568313 | Blood pH QTL (6322) | 3 | 18288556 | 43461008 |
| ENSSSCG00000007805 | 3 | 18547976 | 18568313 | Blood pH QTL (6324) | 3 | 18288556 | 43461008 |
| ENSSSCG00000007805 | 3 | 18547976 | 18568313 | Lactate level QTL (6360) | 3 | 18288556 | 43461008 |
| ENSSSCG00000007805 | 3 | 18547976 | 18568313 | Body weight (end of test) QTL (5234) | 3 | 2979596 | 1.33E+08 |
| ENSSSCG00000007805 | 3 | 18547976 | 18568313 | Cooking loss QTL (38079) | 3 | 12740342 | 1.33E+08 |
| ENSSSCG00000032531 | 3 | 26653792 | 26754882 | Carcass weight (hot) QTL (16861) | 3 | 6267426 | 43461008 |
| ENSSSCG00000032531 | 3 | 26653792 | 26754882 | Carcass temperature (45 minutes post-mortem) QTL (21356) | 3 | 12740342 | 43461008 |
| ENSSSCG00000032531 | 3 | 26653792 | 26754882 | Backfat at tenth rib QTL (2743) | 3 | 12740342 | 43461008 |
| ENSSSCG00000032531 | 3 | 26653792 | 26754882 | pH 24 hr post-mortem (loin) QTL (300) | 3 | 17774833 | 53838222 |
| ENSSSCG00000032531 | 3 | 26653792 | 26754882 | Average daily gain QTL (497) | 3 | 17774833 | 43461008 |
| ENSSSCG00000032531 | 3 | 26653792 | 26754882 | Number of muscle fibers per unit area QTL (2802) | 3 | 18288556 | 62160543 |
| ENSSSCG00000032531 | 3 | 26653792 | 26754882 | Number of capillaries per type I fiber QTL (2803) | 3 | 18288556 | 62160543 |
| ENSSSCG00000032531 | 3 | 26653792 | 26754882 | Drip loss QTL (5692) | 3 | 18288556 | 62160543 |
| ENSSSCG00000032531 | 3 | 26653792 | 26754882 | HDL cholesterol QTL (17716) | 3 | 18288556 | 62160543 |
| ENSSSCG00000032531 | 3 | 26653792 | 26754882 | Haptoglobin concentration QTL (9642) | 3 | 18288556 | 62160543 |
| ENSSSCG00000032531 | 3 | 26653792 | 26754882 | Carcass length QTL (875) | 3 | 18288556 | 43461008 |
| ENSSSCG00000032531 | 3 | 26653792 | 26754882 | Body weight (slaughter) QTL (890) | 3 | 18288556 | 43461008 |
| ENSSSCG00000032531 | 3 | 26653792 | 26754882 | Average daily gain QTL (891) | 3 | 18288556 | 43461008 |
| ENSSSCG00000032531 | 3 | 26653792 | 26754882 | Average daily gain QTL (3667) | 3 | 18288556 | 43461008 |
| ENSSSCG00000032531 | 3 | 26653792 | 26754882 | Backfat at last rib QTL (3668) | 3 | 18288556 | 43461008 |
| ENSSSCG00000032531 | 3 | 26653792 | 26754882 | PH for Longissmus dorsi QTL (3678) | 3 | 18288556 | 43461008 |
| ENSSSCG00000032531 | 3 | 26653792 | 26754882 | Carcass length QTL (3679) | 3 | 18288556 | 43461008 |
| ENSSSCG00000032531 | 3 | 26653792 | 26754882 | PH for Longissmus dorsi QTL (3680) | 3 | 18288556 | 43461008 |
| ENSSSCG00000032531 | 3 | 26653792 | 26754882 | Average daily gain QTL (4119) | 3 | 18288556 | 43461008 |
| ENSSSCG00000032531 | 3 | 26653792 | 26754882 | Average daily gain QTL (4120) | 3 | 18288556 | 43461008 |
| ENSSSCG00000032531 | 3 | 26653792 | 26754882 | backfat at P2 position QTL (4121) | 3 | 18288556 | 43461008 |
| ENSSSCG00000032531 | 3 | 26653792 | 26754882 | Carcass length QTL (4122) | 3 | 18288556 | 43461008 |
| ENSSSCG00000032531 | 3 | 26653792 | 26754882 | PH for Longissmus dorsi QTL (4123) | 3 | 18288556 | 43461008 |
| ENSSSCG00000032531 | 3 | 26653792 | 26754882 | Blood pH QTL (6322) | 3 | 18288556 | 43461008 |
| ENSSSCG00000032531 | 3 | 26653792 | 26754882 | Blood pH QTL (6324) | 3 | 18288556 | 43461008 |
| ENSSSCG00000032531 | 3 | 26653792 | 26754882 | Lactate level QTL (6360) | 3 | 18288556 | 43461008 |
| ENSSSCG00000032531 | 3 | 26653792 | 26754882 | Body weight (end of test) QTL (5234) | 3 | 2979596 | 1.33E+08 |
| ENSSSCG00000032531 | 3 | 26653792 | 26754882 | Cooking loss QTL (38079) | 3 | 12740342 | 1.33E+08 |
| ENSSSCG00000032531 | 3 | 26653792 | 26754882 | pH 45 minutes post mortem QTL (3227) | 3 | 26302643 | 1.22E+08 |
| ENSSSCG00000007874 | 3 | 28360868 | 28485477 | Carcass weight (hot) QTL (16861) | 3 | 6267426 | 43461008 |
| ENSSSCG00000007874 | 3 | 28360868 | 28485477 | Carcass temperature (45 minutes post-mortem) QTL (21356) | 3 | 12740342 | 43461008 |
| ENSSSCG00000007874 | 3 | 28360868 | 28485477 | Backfat at tenth rib QTL (2743) | 3 | 12740342 | 43461008 |
| ENSSSCG00000007874 | 3 | 28360868 | 28485477 | pH 24 hr post-mortem (loin) QTL (300) | 3 | 17774833 | 53838222 |
| ENSSSCG00000007874 | 3 | 28360868 | 28485477 | Average daily gain QTL (497) | 3 | 17774833 | 43461008 |
| ENSSSCG00000007874 | 3 | 28360868 | 28485477 | Number of muscle fibers per unit area QTL (2802) | 3 | 18288556 | 62160543 |
| ENSSSCG00000007874 | 3 | 28360868 | 28485477 | Number of capillaries per type I fiber QTL (2803) | 3 | 18288556 | 62160543 |
| ENSSSCG00000007874 | 3 | 28360868 | 28485477 | Drip loss QTL (5692) | 3 | 18288556 | 62160543 |
| ENSSSCG00000007874 | 3 | 28360868 | 28485477 | HDL cholesterol QTL (17716) | 3 | 18288556 | 62160543 |
| ENSSSCG00000007874 | 3 | 28360868 | 28485477 | Haptoglobin concentration QTL (9642) | 3 | 18288556 | 62160543 |
| ENSSSCG00000007874 | 3 | 28360868 | 28485477 | Carcass length QTL (875) | 3 | 18288556 | 43461008 |
| ENSSSCG00000007874 | 3 | 28360868 | 28485477 | Body weight (slaughter) QTL (890) | 3 | 18288556 | 43461008 |
| ENSSSCG00000007874 | 3 | 28360868 | 28485477 | Average daily gain QTL (891) | 3 | 18288556 | 43461008 |
| ENSSSCG00000007874 | 3 | 28360868 | 28485477 | Average daily gain QTL (3667) | 3 | 18288556 | 43461008 |
| ENSSSCG00000007874 | 3 | 28360868 | 28485477 | Backfat at last rib QTL (3668) | 3 | 18288556 | 43461008 |
| ENSSSCG00000007874 | 3 | 28360868 | 28485477 | PH for Longissmus dorsi QTL (3678) | 3 | 18288556 | 43461008 |
| ENSSSCG00000007874 | 3 | 28360868 | 28485477 | Carcass length QTL (3679) | 3 | 18288556 | 43461008 |
| ENSSSCG00000007874 | 3 | 28360868 | 28485477 | PH for Longissmus dorsi QTL (3680) | 3 | 18288556 | 43461008 |
| ENSSSCG00000007874 | 3 | 28360868 | 28485477 | Average daily gain QTL (4119) | 3 | 18288556 | 43461008 |
| ENSSSCG00000007874 | 3 | 28360868 | 28485477 | Average daily gain QTL (4120) | 3 | 18288556 | 43461008 |
| ENSSSCG00000007874 | 3 | 28360868 | 28485477 | backfat at P2 position QTL (4121) | 3 | 18288556 | 43461008 |
| ENSSSCG00000007874 | 3 | 28360868 | 28485477 | Carcass length QTL (4122) | 3 | 18288556 | 43461008 |
| ENSSSCG00000007874 | 3 | 28360868 | 28485477 | PH for Longissmus dorsi QTL (4123) | 3 | 18288556 | 43461008 |
| ENSSSCG00000007874 | 3 | 28360868 | 28485477 | Blood pH QTL (6322) | 3 | 18288556 | 43461008 |
| ENSSSCG00000007874 | 3 | 28360868 | 28485477 | Blood pH QTL (6324) | 3 | 18288556 | 43461008 |
| ENSSSCG00000007874 | 3 | 28360868 | 28485477 | Lactate level QTL (6360) | 3 | 18288556 | 43461008 |
| ENSSSCG00000007874 | 3 | 28360868 | 28485477 | Skin thickness QTL (283) | 3 | 28357537 | 43461008 |
| ENSSSCG00000007874 | 3 | 28360868 | 28485477 | Body weight (end of test) QTL (5234) | 3 | 2979596 | 1.33E+08 |
| ENSSSCG00000007874 | 3 | 28360868 | 28485477 | Cooking loss QTL (38079) | 3 | 12740342 | 1.33E+08 |
| ENSSSCG00000007874 | 3 | 28360868 | 28485477 | pH 45 minutes post mortem QTL (3227) | 3 | 26302643 | 1.22E+08 |
| ENSSSCG00000037065 | 3 | 32450604 | 32492722 | 45 min-3 hr pH decline QTL (5885) | 3 | 32364580 | 32816657 |
| ENSSSCG00000037065 | 3 | 32450604 | 32492722 | Carcass weight (hot) QTL (16861) | 3 | 6267426 | 43461008 |
| ENSSSCG00000037065 | 3 | 32450604 | 32492722 | Carcass temperature (45 minutes post-mortem) QTL (21356) | 3 | 12740342 | 43461008 |
| ENSSSCG00000037065 | 3 | 32450604 | 32492722 | Backfat at tenth rib QTL (2743) | 3 | 12740342 | 43461008 |
| ENSSSCG00000037065 | 3 | 32450604 | 32492722 | pH 24 hr post-mortem (loin) QTL (300) | 3 | 17774833 | 53838222 |
| ENSSSCG00000037065 | 3 | 32450604 | 32492722 | Average daily gain QTL (497) | 3 | 17774833 | 43461008 |
| ENSSSCG00000037065 | 3 | 32450604 | 32492722 | Number of muscle fibers per unit area QTL (2802) | 3 | 18288556 | 62160543 |
| ENSSSCG00000037065 | 3 | 32450604 | 32492722 | Number of capillaries per type I fiber QTL (2803) | 3 | 18288556 | 62160543 |
| ENSSSCG00000037065 | 3 | 32450604 | 32492722 | Drip loss QTL (5692) | 3 | 18288556 | 62160543 |
| ENSSSCG00000037065 | 3 | 32450604 | 32492722 | HDL cholesterol QTL (17716) | 3 | 18288556 | 62160543 |
| ENSSSCG00000037065 | 3 | 32450604 | 32492722 | Haptoglobin concentration QTL (9642) | 3 | 18288556 | 62160543 |
| ENSSSCG00000037065 | 3 | 32450604 | 32492722 | Carcass length QTL (875) | 3 | 18288556 | 43461008 |
| ENSSSCG00000037065 | 3 | 32450604 | 32492722 | Body weight (slaughter) QTL (890) | 3 | 18288556 | 43461008 |
| ENSSSCG00000037065 | 3 | 32450604 | 32492722 | Average daily gain QTL (891) | 3 | 18288556 | 43461008 |
| ENSSSCG00000037065 | 3 | 32450604 | 32492722 | Average daily gain QTL (3667) | 3 | 18288556 | 43461008 |
| ENSSSCG00000037065 | 3 | 32450604 | 32492722 | Backfat at last rib QTL (3668) | 3 | 18288556 | 43461008 |
| ENSSSCG00000037065 | 3 | 32450604 | 32492722 | PH for Longissmus dorsi QTL (3678) | 3 | 18288556 | 43461008 |
| ENSSSCG00000037065 | 3 | 32450604 | 32492722 | Carcass length QTL (3679) | 3 | 18288556 | 43461008 |
| ENSSSCG00000037065 | 3 | 32450604 | 32492722 | PH for Longissmus dorsi QTL (3680) | 3 | 18288556 | 43461008 |
| ENSSSCG00000037065 | 3 | 32450604 | 32492722 | Average daily gain QTL (4119) | 3 | 18288556 | 43461008 |
| ENSSSCG00000037065 | 3 | 32450604 | 32492722 | Average daily gain QTL (4120) | 3 | 18288556 | 43461008 |
| ENSSSCG00000037065 | 3 | 32450604 | 32492722 | backfat at P2 position QTL (4121) | 3 | 18288556 | 43461008 |
| ENSSSCG00000037065 | 3 | 32450604 | 32492722 | Carcass length QTL (4122) | 3 | 18288556 | 43461008 |
| ENSSSCG00000037065 | 3 | 32450604 | 32492722 | PH for Longissmus dorsi QTL (4123) | 3 | 18288556 | 43461008 |
| ENSSSCG00000037065 | 3 | 32450604 | 32492722 | Blood pH QTL (6322) | 3 | 18288556 | 43461008 |
| ENSSSCG00000037065 | 3 | 32450604 | 32492722 | Blood pH QTL (6324) | 3 | 18288556 | 43461008 |
| ENSSSCG00000037065 | 3 | 32450604 | 32492722 | Lactate level QTL (6360) | 3 | 18288556 | 43461008 |
| ENSSSCG00000037065 | 3 | 32450604 | 32492722 | Skin thickness QTL (283) | 3 | 28357537 | 43461008 |
| ENSSSCG00000037065 | 3 | 32450604 | 32492722 | Body weight (end of test) QTL (5234) | 3 | 2979596 | 1.33E+08 |
| ENSSSCG00000037065 | 3 | 32450604 | 32492722 | Cooking loss QTL (38079) | 3 | 12740342 | 1.33E+08 |
| ENSSSCG00000037065 | 3 | 32450604 | 32492722 | pH 45 minutes post mortem QTL (3227) | 3 | 26302643 | 1.22E+08 |
| ENSSSCG00000037065 | 3 | 32450604 | 32492722 | body weight (17 weeks) QTL (172) | 3 | 31850857 | 67811502 |
| ENSSSCG00000007951 | 3 | 38388547 | 38530230 | Carcass weight (hot) QTL (16861) | 3 | 6267426 | 43461008 |
| ENSSSCG00000007951 | 3 | 38388547 | 38530230 | Carcass temperature (45 minutes post-mortem) QTL (21356) | 3 | 12740342 | 43461008 |
| ENSSSCG00000007951 | 3 | 38388547 | 38530230 | Backfat at tenth rib QTL (2743) | 3 | 12740342 | 43461008 |
| ENSSSCG00000007951 | 3 | 38388547 | 38530230 | pH 24 hr post-mortem (loin) QTL (300) | 3 | 17774833 | 53838222 |
| ENSSSCG00000007951 | 3 | 38388547 | 38530230 | Average daily gain QTL (497) | 3 | 17774833 | 43461008 |
| ENSSSCG00000007951 | 3 | 38388547 | 38530230 | Number of muscle fibers per unit area QTL (2802) | 3 | 18288556 | 62160543 |
| ENSSSCG00000007951 | 3 | 38388547 | 38530230 | Number of capillaries per type I fiber QTL (2803) | 3 | 18288556 | 62160543 |
| ENSSSCG00000007951 | 3 | 38388547 | 38530230 | Drip loss QTL (5692) | 3 | 18288556 | 62160543 |
| ENSSSCG00000007951 | 3 | 38388547 | 38530230 | HDL cholesterol QTL (17716) | 3 | 18288556 | 62160543 |
| ENSSSCG00000007951 | 3 | 38388547 | 38530230 | Haptoglobin concentration QTL (9642) | 3 | 18288556 | 62160543 |
| ENSSSCG00000007951 | 3 | 38388547 | 38530230 | Carcass length QTL (875) | 3 | 18288556 | 43461008 |
| ENSSSCG00000007951 | 3 | 38388547 | 38530230 | Body weight (slaughter) QTL (890) | 3 | 18288556 | 43461008 |
| ENSSSCG00000007951 | 3 | 38388547 | 38530230 | Average daily gain QTL (891) | 3 | 18288556 | 43461008 |
| ENSSSCG00000007951 | 3 | 38388547 | 38530230 | Average daily gain QTL (3667) | 3 | 18288556 | 43461008 |
| ENSSSCG00000007951 | 3 | 38388547 | 38530230 | Backfat at last rib QTL (3668) | 3 | 18288556 | 43461008 |
| ENSSSCG00000007951 | 3 | 38388547 | 38530230 | PH for Longissmus dorsi QTL (3678) | 3 | 18288556 | 43461008 |
| ENSSSCG00000007951 | 3 | 38388547 | 38530230 | Carcass length QTL (3679) | 3 | 18288556 | 43461008 |
| ENSSSCG00000007951 | 3 | 38388547 | 38530230 | PH for Longissmus dorsi QTL (3680) | 3 | 18288556 | 43461008 |
| ENSSSCG00000007951 | 3 | 38388547 | 38530230 | Average daily gain QTL (4119) | 3 | 18288556 | 43461008 |
| ENSSSCG00000007951 | 3 | 38388547 | 38530230 | Average daily gain QTL (4120) | 3 | 18288556 | 43461008 |
| ENSSSCG00000007951 | 3 | 38388547 | 38530230 | backfat at P2 position QTL (4121) | 3 | 18288556 | 43461008 |
| ENSSSCG00000007951 | 3 | 38388547 | 38530230 | Carcass length QTL (4122) | 3 | 18288556 | 43461008 |
| ENSSSCG00000007951 | 3 | 38388547 | 38530230 | PH for Longissmus dorsi QTL (4123) | 3 | 18288556 | 43461008 |
| ENSSSCG00000007951 | 3 | 38388547 | 38530230 | Blood pH QTL (6322) | 3 | 18288556 | 43461008 |
| ENSSSCG00000007951 | 3 | 38388547 | 38530230 | Blood pH QTL (6324) | 3 | 18288556 | 43461008 |
| ENSSSCG00000007951 | 3 | 38388547 | 38530230 | Lactate level QTL (6360) | 3 | 18288556 | 43461008 |
| ENSSSCG00000007951 | 3 | 38388547 | 38530230 | Skin thickness QTL (283) | 3 | 28357537 | 43461008 |
| ENSSSCG00000007951 | 3 | 38388547 | 38530230 | Body weight (end of test) QTL (5234) | 3 | 2979596 | 1.33E+08 |
| ENSSSCG00000007951 | 3 | 38388547 | 38530230 | Cooking loss QTL (38079) | 3 | 12740342 | 1.33E+08 |
| ENSSSCG00000007951 | 3 | 38388547 | 38530230 | pH 45 minutes post mortem QTL (3227) | 3 | 26302643 | 1.22E+08 |
| ENSSSCG00000007951 | 3 | 38388547 | 38530230 | body weight (17 weeks) QTL (172) | 3 | 31850857 | 67811502 |
| ENSSSCG00000037719 | 3 | 39429931 | 39532583 | Carcass weight (hot) QTL (16861) | 3 | 6267426 | 43461008 |
| ENSSSCG00000037719 | 3 | 39429931 | 39532583 | Carcass temperature (45 minutes post-mortem) QTL (21356) | 3 | 12740342 | 43461008 |
| ENSSSCG00000037719 | 3 | 39429931 | 39532583 | Backfat at tenth rib QTL (2743) | 3 | 12740342 | 43461008 |
| ENSSSCG00000037719 | 3 | 39429931 | 39532583 | pH 24 hr post-mortem (loin) QTL (300) | 3 | 17774833 | 53838222 |
| ENSSSCG00000037719 | 3 | 39429931 | 39532583 | Average daily gain QTL (497) | 3 | 17774833 | 43461008 |
| ENSSSCG00000037719 | 3 | 39429931 | 39532583 | Number of muscle fibers per unit area QTL (2802) | 3 | 18288556 | 62160543 |
| ENSSSCG00000037719 | 3 | 39429931 | 39532583 | Number of capillaries per type I fiber QTL (2803) | 3 | 18288556 | 62160543 |
| ENSSSCG00000037719 | 3 | 39429931 | 39532583 | Drip loss QTL (5692) | 3 | 18288556 | 62160543 |
| ENSSSCG00000037719 | 3 | 39429931 | 39532583 | HDL cholesterol QTL (17716) | 3 | 18288556 | 62160543 |
| ENSSSCG00000037719 | 3 | 39429931 | 39532583 | Haptoglobin concentration QTL (9642) | 3 | 18288556 | 62160543 |
| ENSSSCG00000037719 | 3 | 39429931 | 39532583 | Carcass length QTL (875) | 3 | 18288556 | 43461008 |
| ENSSSCG00000037719 | 3 | 39429931 | 39532583 | Body weight (slaughter) QTL (890) | 3 | 18288556 | 43461008 |
| ENSSSCG00000037719 | 3 | 39429931 | 39532583 | Average daily gain QTL (891) | 3 | 18288556 | 43461008 |
| ENSSSCG00000037719 | 3 | 39429931 | 39532583 | Average daily gain QTL (3667) | 3 | 18288556 | 43461008 |
| ENSSSCG00000037719 | 3 | 39429931 | 39532583 | Backfat at last rib QTL (3668) | 3 | 18288556 | 43461008 |
| ENSSSCG00000037719 | 3 | 39429931 | 39532583 | PH for Longissmus dorsi QTL (3678) | 3 | 18288556 | 43461008 |
| ENSSSCG00000037719 | 3 | 39429931 | 39532583 | Carcass length QTL (3679) | 3 | 18288556 | 43461008 |
| ENSSSCG00000037719 | 3 | 39429931 | 39532583 | PH for Longissmus dorsi QTL (3680) | 3 | 18288556 | 43461008 |
| ENSSSCG00000037719 | 3 | 39429931 | 39532583 | Average daily gain QTL (4119) | 3 | 18288556 | 43461008 |
| ENSSSCG00000037719 | 3 | 39429931 | 39532583 | Average daily gain QTL (4120) | 3 | 18288556 | 43461008 |
| ENSSSCG00000037719 | 3 | 39429931 | 39532583 | backfat at P2 position QTL (4121) | 3 | 18288556 | 43461008 |
| ENSSSCG00000037719 | 3 | 39429931 | 39532583 | Carcass length QTL (4122) | 3 | 18288556 | 43461008 |
| ENSSSCG00000037719 | 3 | 39429931 | 39532583 | PH for Longissmus dorsi QTL (4123) | 3 | 18288556 | 43461008 |
| ENSSSCG00000037719 | 3 | 39429931 | 39532583 | Blood pH QTL (6322) | 3 | 18288556 | 43461008 |
| ENSSSCG00000037719 | 3 | 39429931 | 39532583 | Blood pH QTL (6324) | 3 | 18288556 | 43461008 |
| ENSSSCG00000037719 | 3 | 39429931 | 39532583 | Lactate level QTL (6360) | 3 | 18288556 | 43461008 |
| ENSSSCG00000037719 | 3 | 39429931 | 39532583 | Skin thickness QTL (283) | 3 | 28357537 | 43461008 |
| ENSSSCG00000037719 | 3 | 39429931 | 39532583 | Body weight (end of test) QTL (5234) | 3 | 2979596 | 1.33E+08 |
| ENSSSCG00000037719 | 3 | 39429931 | 39532583 | Cooking loss QTL (38079) | 3 | 12740342 | 1.33E+08 |
| ENSSSCG00000037719 | 3 | 39429931 | 39532583 | pH 45 minutes post mortem QTL (3227) | 3 | 26302643 | 1.22E+08 |
| ENSSSCG00000037719 | 3 | 39429931 | 39532583 | body weight (17 weeks) QTL (172) | 3 | 31850857 | 67811502 |
| ENSSSCG00000035854 | 3 | 41211684 | 41285196 | Carcass weight (hot) QTL (16861) | 3 | 6267426 | 43461008 |
| ENSSSCG00000035854 | 3 | 41211684 | 41285196 | Carcass temperature (45 minutes post-mortem) QTL (21356) | 3 | 12740342 | 43461008 |
| ENSSSCG00000035854 | 3 | 41211684 | 41285196 | Backfat at tenth rib QTL (2743) | 3 | 12740342 | 43461008 |
| ENSSSCG00000035854 | 3 | 41211684 | 41285196 | pH 24 hr post-mortem (loin) QTL (300) | 3 | 17774833 | 53838222 |
| ENSSSCG00000035854 | 3 | 41211684 | 41285196 | Average daily gain QTL (497) | 3 | 17774833 | 43461008 |
| ENSSSCG00000035854 | 3 | 41211684 | 41285196 | Number of muscle fibers per unit area QTL (2802) | 3 | 18288556 | 62160543 |
| ENSSSCG00000035854 | 3 | 41211684 | 41285196 | Number of capillaries per type I fiber QTL (2803) | 3 | 18288556 | 62160543 |
| ENSSSCG00000035854 | 3 | 41211684 | 41285196 | Drip loss QTL (5692) | 3 | 18288556 | 62160543 |
| ENSSSCG00000035854 | 3 | 41211684 | 41285196 | HDL cholesterol QTL (17716) | 3 | 18288556 | 62160543 |
| ENSSSCG00000035854 | 3 | 41211684 | 41285196 | Haptoglobin concentration QTL (9642) | 3 | 18288556 | 62160543 |
| ENSSSCG00000035854 | 3 | 41211684 | 41285196 | Carcass length QTL (875) | 3 | 18288556 | 43461008 |
| ENSSSCG00000035854 | 3 | 41211684 | 41285196 | Body weight (slaughter) QTL (890) | 3 | 18288556 | 43461008 |
| ENSSSCG00000035854 | 3 | 41211684 | 41285196 | Average daily gain QTL (891) | 3 | 18288556 | 43461008 |
| ENSSSCG00000035854 | 3 | 41211684 | 41285196 | Average daily gain QTL (3667) | 3 | 18288556 | 43461008 |
| ENSSSCG00000035854 | 3 | 41211684 | 41285196 | Backfat at last rib QTL (3668) | 3 | 18288556 | 43461008 |
| ENSSSCG00000035854 | 3 | 41211684 | 41285196 | PH for Longissmus dorsi QTL (3678) | 3 | 18288556 | 43461008 |
| ENSSSCG00000035854 | 3 | 41211684 | 41285196 | Carcass length QTL (3679) | 3 | 18288556 | 43461008 |
| ENSSSCG00000035854 | 3 | 41211684 | 41285196 | PH for Longissmus dorsi QTL (3680) | 3 | 18288556 | 43461008 |
| ENSSSCG00000035854 | 3 | 41211684 | 41285196 | Average daily gain QTL (4119) | 3 | 18288556 | 43461008 |
| ENSSSCG00000035854 | 3 | 41211684 | 41285196 | Average daily gain QTL (4120) | 3 | 18288556 | 43461008 |
| ENSSSCG00000035854 | 3 | 41211684 | 41285196 | backfat at P2 position QTL (4121) | 3 | 18288556 | 43461008 |
| ENSSSCG00000035854 | 3 | 41211684 | 41285196 | Carcass length QTL (4122) | 3 | 18288556 | 43461008 |
| ENSSSCG00000035854 | 3 | 41211684 | 41285196 | PH for Longissmus dorsi QTL (4123) | 3 | 18288556 | 43461008 |
| ENSSSCG00000035854 | 3 | 41211684 | 41285196 | Blood pH QTL (6322) | 3 | 18288556 | 43461008 |
| ENSSSCG00000035854 | 3 | 41211684 | 41285196 | Blood pH QTL (6324) | 3 | 18288556 | 43461008 |
| ENSSSCG00000035854 | 3 | 41211684 | 41285196 | Lactate level QTL (6360) | 3 | 18288556 | 43461008 |
| ENSSSCG00000035854 | 3 | 41211684 | 41285196 | Skin thickness QTL (283) | 3 | 28357537 | 43461008 |
| ENSSSCG00000035854 | 3 | 41211684 | 41285196 | Meat color b* QTL (4038) | 3 | 40789645 | 56612329 |
| ENSSSCG00000035854 | 3 | 41211684 | 41285196 | Total shear work QTL (3989) | 3 | 41200624 | 60002904 |
| ENSSSCG00000035854 | 3 | 41211684 | 41285196 | Cortisol level QTL (21327) | 3 | 41200624 | 60002904 |
| ENSSSCG00000035854 | 3 | 41211684 | 41285196 | Body weight (end of test) QTL (5234) | 3 | 2979596 | 1.33E+08 |
| ENSSSCG00000035854 | 3 | 41211684 | 41285196 | Cooking loss QTL (38079) | 3 | 12740342 | 1.33E+08 |
| ENSSSCG00000035854 | 3 | 41211684 | 41285196 | pH 45 minutes post mortem QTL (3227) | 3 | 26302643 | 1.22E+08 |
| ENSSSCG00000035854 | 3 | 41211684 | 41285196 | body weight (17 weeks) QTL (172) | 3 | 31850857 | 67811502 |
| ENSSSCG00000035854 | 3 | 41211684 | 41285196 | Off-flavor score QTL (3815) | 3 | 41200624 | 96785507 |
| ENSSSCG00000035854 | 3 | 41211684 | 41285196 | Carcass length QTL (3946) | 3 | 41200624 | 87024760 |
| ENSSSCG00000008105 | 3 | 44509692 | 44605014 | Intramuscular fat content QTL (17748) | 3 | 44488455 | 44868610 |
| ENSSSCG00000008105 | 3 | 44509692 | 44605014 | pH 24 hr post-mortem (loin) QTL (300) | 3 | 17774833 | 53838222 |
| ENSSSCG00000008105 | 3 | 44509692 | 44605014 | Number of muscle fibers per unit area QTL (2802) | 3 | 18288556 | 62160543 |
| ENSSSCG00000008105 | 3 | 44509692 | 44605014 | Number of capillaries per type I fiber QTL (2803) | 3 | 18288556 | 62160543 |
| ENSSSCG00000008105 | 3 | 44509692 | 44605014 | Drip loss QTL (5692) | 3 | 18288556 | 62160543 |
| ENSSSCG00000008105 | 3 | 44509692 | 44605014 | HDL cholesterol QTL (17716) | 3 | 18288556 | 62160543 |
| ENSSSCG00000008105 | 3 | 44509692 | 44605014 | Haptoglobin concentration QTL (9642) | 3 | 18288556 | 62160543 |
| ENSSSCG00000008105 | 3 | 44509692 | 44605014 | Meat color b* QTL (4038) | 3 | 40789645 | 56612329 |
| ENSSSCG00000008105 | 3 | 44509692 | 44605014 | Total shear work QTL (3989) | 3 | 41200624 | 60002904 |
| ENSSSCG00000008105 | 3 | 44509692 | 44605014 | Cortisol level QTL (21327) | 3 | 41200624 | 60002904 |
| ENSSSCG00000008105 | 3 | 44509692 | 44605014 | Average daily gain QTL (3099) | 3 | 43461008 | 66578565 |
| ENSSSCG00000008105 | 3 | 44509692 | 44605014 | Carcass length QTL (3100) | 3 | 43461008 | 66578565 |
| ENSSSCG00000008105 | 3 | 44509692 | 44605014 | Loin muscle area QTL (3101) | 3 | 43461008 | 66578565 |
| ENSSSCG00000008105 | 3 | 44509692 | 44605014 | Carcass weight (cold) QTL (3103) | 3 | 43461008 | 66578565 |
| ENSSSCG00000008105 | 3 | 44509692 | 44605014 | Average daily gain QTL (879) | 3 | 43461008 | 66578565 |
| ENSSSCG00000008105 | 3 | 44509692 | 44605014 | Average daily gain QTL (340) | 3 | 43461008 | 60002904 |
| ENSSSCG00000008105 | 3 | 44509692 | 44605014 | Loin muscle area QTL (876) | 3 | 43461008 | 60002904 |
| ENSSSCG00000008105 | 3 | 44509692 | 44605014 | pH 24 hr post-mortem (loin) QTL (896) | 3 | 43461008 | 60002904 |
| ENSSSCG00000008105 | 3 | 44509692 | 44605014 | Creatine kinase level QTL (7490) | 3 | 43461008 | 54351946 |
| ENSSSCG00000008105 | 3 | 44509692 | 44605014 | Bicarbonate level QTL (6333) | 3 | 43461008 | 54351946 |
| ENSSSCG00000008105 | 3 | 44509692 | 44605014 | Plasma FSH concentration QTL (644) | 3 | 43461008 | 52194307 |
| ENSSSCG00000008105 | 3 | 44509692 | 44605014 | Body weight (end of test) QTL (5234) | 3 | 2979596 | 1.33E+08 |
| ENSSSCG00000008105 | 3 | 44509692 | 44605014 | Cooking loss QTL (38079) | 3 | 12740342 | 1.33E+08 |
| ENSSSCG00000008105 | 3 | 44509692 | 44605014 | pH 45 minutes post mortem QTL (3227) | 3 | 26302643 | 1.22E+08 |
| ENSSSCG00000008105 | 3 | 44509692 | 44605014 | body weight (17 weeks) QTL (172) | 3 | 31850857 | 67811502 |
| ENSSSCG00000008105 | 3 | 44509692 | 44605014 | Off-flavor score QTL (3815) | 3 | 41200624 | 96785507 |
| ENSSSCG00000008105 | 3 | 44509692 | 44605014 | Carcass length QTL (3946) | 3 | 41200624 | 87024760 |
| ENSSSCG00000008105 | 3 | 44509692 | 44605014 | Diameter of type IIb muscle fibers QTL (7037) | 3 | 43461008 | 88463186 |
| ENSSSCG00000008144 | 3 | 49063988 | 49198664 | pH 24 hr post-mortem (loin) QTL (300) | 3 | 17774833 | 53838222 |
| ENSSSCG00000008144 | 3 | 49063988 | 49198664 | Number of muscle fibers per unit area QTL (2802) | 3 | 18288556 | 62160543 |
| ENSSSCG00000008144 | 3 | 49063988 | 49198664 | Number of capillaries per type I fiber QTL (2803) | 3 | 18288556 | 62160543 |
| ENSSSCG00000008144 | 3 | 49063988 | 49198664 | Drip loss QTL (5692) | 3 | 18288556 | 62160543 |
| ENSSSCG00000008144 | 3 | 49063988 | 49198664 | HDL cholesterol QTL (17716) | 3 | 18288556 | 62160543 |
| ENSSSCG00000008144 | 3 | 49063988 | 49198664 | Haptoglobin concentration QTL (9642) | 3 | 18288556 | 62160543 |
| ENSSSCG00000008144 | 3 | 49063988 | 49198664 | Meat color b* QTL (4038) | 3 | 40789645 | 56612329 |
| ENSSSCG00000008144 | 3 | 49063988 | 49198664 | Total shear work QTL (3989) | 3 | 41200624 | 60002904 |
| ENSSSCG00000008144 | 3 | 49063988 | 49198664 | Cortisol level QTL (21327) | 3 | 41200624 | 60002904 |
| ENSSSCG00000008144 | 3 | 49063988 | 49198664 | Average daily gain QTL (3099) | 3 | 43461008 | 66578565 |
| ENSSSCG00000008144 | 3 | 49063988 | 49198664 | Carcass length QTL (3100) | 3 | 43461008 | 66578565 |
| ENSSSCG00000008144 | 3 | 49063988 | 49198664 | Loin muscle area QTL (3101) | 3 | 43461008 | 66578565 |
| ENSSSCG00000008144 | 3 | 49063988 | 49198664 | Carcass weight (cold) QTL (3103) | 3 | 43461008 | 66578565 |
| ENSSSCG00000008144 | 3 | 49063988 | 49198664 | Average daily gain QTL (879) | 3 | 43461008 | 66578565 |
| ENSSSCG00000008144 | 3 | 49063988 | 49198664 | Average daily gain QTL (340) | 3 | 43461008 | 60002904 |
| ENSSSCG00000008144 | 3 | 49063988 | 49198664 | Loin muscle area QTL (876) | 3 | 43461008 | 60002904 |
| ENSSSCG00000008144 | 3 | 49063988 | 49198664 | pH 24 hr post-mortem (loin) QTL (896) | 3 | 43461008 | 60002904 |
| ENSSSCG00000008144 | 3 | 49063988 | 49198664 | Creatine kinase level QTL (7490) | 3 | 43461008 | 54351946 |
| ENSSSCG00000008144 | 3 | 49063988 | 49198664 | Bicarbonate level QTL (6333) | 3 | 43461008 | 54351946 |
| ENSSSCG00000008144 | 3 | 49063988 | 49198664 | Plasma FSH concentration QTL (644) | 3 | 43461008 | 52194307 |
| ENSSSCG00000008144 | 3 | 49063988 | 49198664 | Body weight (end of test) QTL (5234) | 3 | 2979596 | 1.33E+08 |
| ENSSSCG00000008144 | 3 | 49063988 | 49198664 | Cooking loss QTL (38079) | 3 | 12740342 | 1.33E+08 |
| ENSSSCG00000008144 | 3 | 49063988 | 49198664 | pH 45 minutes post mortem QTL (3227) | 3 | 26302643 | 1.22E+08 |
| ENSSSCG00000008144 | 3 | 49063988 | 49198664 | body weight (17 weeks) QTL (172) | 3 | 31850857 | 67811502 |
| ENSSSCG00000008144 | 3 | 49063988 | 49198664 | Off-flavor score QTL (3815) | 3 | 41200624 | 96785507 |
| ENSSSCG00000008144 | 3 | 49063988 | 49198664 | Carcass length QTL (3946) | 3 | 41200624 | 87024760 |
| ENSSSCG00000008144 | 3 | 49063988 | 49198664 | Diameter of type IIb muscle fibers QTL (7037) | 3 | 43461008 | 88463186 |
| ENSSSCG00000008144 | 3 | 49063988 | 49198664 | Average daily gain QTL (173) | 3 | 46235115 | 79113419 |
| ENSSSCG00000008144 | 3 | 49063988 | 49198664 | Carcass weight (hot) QTL (29691) | 3 | 47262562 | 77366759 |
| ENSSSCG00000008144 | 3 | 49063988 | 49198664 | Fat androstenone level QTL (596) | 3 | 47262562 | 75003631 |
| ENSSSCG00000008144 | 3 | 49063988 | 49198664 | Fat androstenone level QTL (597) | 3 | 47262562 | 75003631 |
| ENSSSCG00000008150 | 3 | 51400165 | 51496293 | pH 24 hr post-mortem (loin) QTL (300) | 3 | 17774833 | 53838222 |
| ENSSSCG00000008150 | 3 | 51400165 | 51496293 | Number of muscle fibers per unit area QTL (2802) | 3 | 18288556 | 62160543 |
| ENSSSCG00000008150 | 3 | 51400165 | 51496293 | Number of capillaries per type I fiber QTL (2803) | 3 | 18288556 | 62160543 |
| ENSSSCG00000008150 | 3 | 51400165 | 51496293 | Drip loss QTL (5692) | 3 | 18288556 | 62160543 |
| ENSSSCG00000008150 | 3 | 51400165 | 51496293 | HDL cholesterol QTL (17716) | 3 | 18288556 | 62160543 |
| ENSSSCG00000008150 | 3 | 51400165 | 51496293 | Haptoglobin concentration QTL (9642) | 3 | 18288556 | 62160543 |
| ENSSSCG00000008150 | 3 | 51400165 | 51496293 | Meat color b* QTL (4038) | 3 | 40789645 | 56612329 |
| ENSSSCG00000008150 | 3 | 51400165 | 51496293 | Total shear work QTL (3989) | 3 | 41200624 | 60002904 |
| ENSSSCG00000008150 | 3 | 51400165 | 51496293 | Cortisol level QTL (21327) | 3 | 41200624 | 60002904 |
| ENSSSCG00000008150 | 3 | 51400165 | 51496293 | Average daily gain QTL (3099) | 3 | 43461008 | 66578565 |
| ENSSSCG00000008150 | 3 | 51400165 | 51496293 | Carcass length QTL (3100) | 3 | 43461008 | 66578565 |
| ENSSSCG00000008150 | 3 | 51400165 | 51496293 | Loin muscle area QTL (3101) | 3 | 43461008 | 66578565 |
| ENSSSCG00000008150 | 3 | 51400165 | 51496293 | Carcass weight (cold) QTL (3103) | 3 | 43461008 | 66578565 |
| ENSSSCG00000008150 | 3 | 51400165 | 51496293 | Average daily gain QTL (879) | 3 | 43461008 | 66578565 |
| ENSSSCG00000008150 | 3 | 51400165 | 51496293 | Average daily gain QTL (340) | 3 | 43461008 | 60002904 |
| ENSSSCG00000008150 | 3 | 51400165 | 51496293 | Loin muscle area QTL (876) | 3 | 43461008 | 60002904 |
| ENSSSCG00000008150 | 3 | 51400165 | 51496293 | pH 24 hr post-mortem (loin) QTL (896) | 3 | 43461008 | 60002904 |
| ENSSSCG00000008150 | 3 | 51400165 | 51496293 | Creatine kinase level QTL (7490) | 3 | 43461008 | 54351946 |
| ENSSSCG00000008150 | 3 | 51400165 | 51496293 | Bicarbonate level QTL (6333) | 3 | 43461008 | 54351946 |
| ENSSSCG00000008150 | 3 | 51400165 | 51496293 | Plasma FSH concentration QTL (644) | 3 | 43461008 | 52194307 |
| ENSSSCG00000008150 | 3 | 51400165 | 51496293 | Body weight (end of test) QTL (5234) | 3 | 2979596 | 1.33E+08 |
| ENSSSCG00000008150 | 3 | 51400165 | 51496293 | Cooking loss QTL (38079) | 3 | 12740342 | 1.33E+08 |
| ENSSSCG00000008150 | 3 | 51400165 | 51496293 | pH 45 minutes post mortem QTL (3227) | 3 | 26302643 | 1.22E+08 |
| ENSSSCG00000008150 | 3 | 51400165 | 51496293 | body weight (17 weeks) QTL (172) | 3 | 31850857 | 67811502 |
| ENSSSCG00000008150 | 3 | 51400165 | 51496293 | Off-flavor score QTL (3815) | 3 | 41200624 | 96785507 |
| ENSSSCG00000008150 | 3 | 51400165 | 51496293 | Carcass length QTL (3946) | 3 | 41200624 | 87024760 |
| ENSSSCG00000008150 | 3 | 51400165 | 51496293 | Diameter of type IIb muscle fibers QTL (7037) | 3 | 43461008 | 88463186 |
| ENSSSCG00000008150 | 3 | 51400165 | 51496293 | Average daily gain QTL (173) | 3 | 46235115 | 79113419 |
| ENSSSCG00000008150 | 3 | 51400165 | 51496293 | Carcass weight (hot) QTL (29691) | 3 | 47262562 | 77366759 |
| ENSSSCG00000008150 | 3 | 51400165 | 51496293 | Fat androstenone level QTL (596) | 3 | 47262562 | 75003631 |
| ENSSSCG00000008150 | 3 | 51400165 | 51496293 | Fat androstenone level QTL (597) | 3 | 47262562 | 75003631 |
| ENSSSCG00000008540 | 3 | 1.1E+08 | 1.1E+08 | Creatinine level QTL (6367) | 3 | 74284418 | 1.16E+08 |
| ENSSSCG00000008540 | 3 | 1.1E+08 | 1.1E+08 | Sodium level QTL (6387) | 3 | 74284418 | 1.16E+08 |
| ENSSSCG00000008540 | 3 | 1.1E+08 | 1.1E+08 | Average backfat thickness QTL (7530) | 3 | 96785507 | 1.13E+08 |
| ENSSSCG00000008540 | 3 | 1.1E+08 | 1.1E+08 | Backfat at mid-back QTL (17791) | 3 | 99045890 | 1.14E+08 |
| ENSSSCG00000008540 | 3 | 1.1E+08 | 1.1E+08 | Thawing loss QTL (21608) | 3 | 1.05E+08 | 1.12E+08 |
| ENSSSCG00000008540 | 3 | 1.1E+08 | 1.1E+08 | Shear force QTL (21607) | 3 | 1.05E+08 | 1.12E+08 |
| ENSSSCG00000008540 | 3 | 1.1E+08 | 1.1E+08 | pH 24 hr post mortem (ham) QTL (21604) | 3 | 1.05E+08 | 1.12E+08 |
| ENSSSCG00000008540 | 3 | 1.1E+08 | 1.1E+08 | pH 24 hr post-mortem (loin) QTL (21605) | 3 | 1.05E+08 | 1.12E+08 |
| ENSSSCG00000008540 | 3 | 1.1E+08 | 1.1E+08 | pH 24 hr post-mortem (loin) QTL (21606) | 3 | 1.05E+08 | 1.12E+08 |
| ENSSSCG00000008540 | 3 | 1.1E+08 | 1.1E+08 | pH 24 hr post mortem (ham) QTL (21603) | 3 | 1.05E+08 | 1.12E+08 |
| ENSSSCG00000008540 | 3 | 1.1E+08 | 1.1E+08 | 45 min-24 h pH decline QTL (21600) | 3 | 1.05E+08 | 1.12E+08 |
| ENSSSCG00000008540 | 3 | 1.1E+08 | 1.1E+08 | pH 24 hr post mortem (ham) QTL (21601) | 3 | 1.05E+08 | 1.12E+08 |
| ENSSSCG00000008540 | 3 | 1.1E+08 | 1.1E+08 | pH 24 hr post mortem (ham) QTL (21602) | 3 | 1.05E+08 | 1.12E+08 |
| ENSSSCG00000008540 | 3 | 1.1E+08 | 1.1E+08 | 45 min-24 h pH decline QTL (21599) | 3 | 1.05E+08 | 1.12E+08 |
| ENSSSCG00000008540 | 3 | 1.1E+08 | 1.1E+08 | Drip loss QTL (21596) | 3 | 1.05E+08 | 1.12E+08 |
| ENSSSCG00000008540 | 3 | 1.1E+08 | 1.1E+08 | Conductivity 24 hours post-mortem QTL (21597) | 3 | 1.05E+08 | 1.12E+08 |
| ENSSSCG00000008540 | 3 | 1.1E+08 | 1.1E+08 | Drip loss QTL (21595) | 3 | 1.05E+08 | 1.12E+08 |
| ENSSSCG00000008540 | 3 | 1.1E+08 | 1.1E+08 | muscle protein percentage QTL (5545) | 3 | 1.06E+08 | 1.23E+08 |
| ENSSSCG00000008540 | 3 | 1.1E+08 | 1.1E+08 | Body weight (end of test) QTL (5234) | 3 | 2979596 | 1.33E+08 |
| ENSSSCG00000008540 | 3 | 1.1E+08 | 1.1E+08 | Cooking loss QTL (38079) | 3 | 12740342 | 1.33E+08 |
| ENSSSCG00000008540 | 3 | 1.1E+08 | 1.1E+08 | pH 45 minutes post mortem QTL (3227) | 3 | 26302643 | 1.22E+08 |
| ENSSSCG00000008540 | 3 | 1.1E+08 | 1.1E+08 | Fat protein content QTL (18011) | 3 | 66578565 | 1.16E+08 |
| ENSSSCG00000026465 | 3 | 1.11E+08 | 1.11E+08 | Creatinine level QTL (6367) | 3 | 74284418 | 1.16E+08 |
| ENSSSCG00000026465 | 3 | 1.11E+08 | 1.11E+08 | Sodium level QTL (6387) | 3 | 74284418 | 1.16E+08 |
| ENSSSCG00000026465 | 3 | 1.11E+08 | 1.11E+08 | Average backfat thickness QTL (7530) | 3 | 96785507 | 1.13E+08 |
| ENSSSCG00000026465 | 3 | 1.11E+08 | 1.11E+08 | Backfat at mid-back QTL (17791) | 3 | 99045890 | 1.14E+08 |
| ENSSSCG00000026465 | 3 | 1.11E+08 | 1.11E+08 | Thawing loss QTL (21608) | 3 | 1.05E+08 | 1.12E+08 |
| ENSSSCG00000026465 | 3 | 1.11E+08 | 1.11E+08 | Shear force QTL (21607) | 3 | 1.05E+08 | 1.12E+08 |
| ENSSSCG00000026465 | 3 | 1.11E+08 | 1.11E+08 | pH 24 hr post mortem (ham) QTL (21604) | 3 | 1.05E+08 | 1.12E+08 |
| ENSSSCG00000026465 | 3 | 1.11E+08 | 1.11E+08 | pH 24 hr post-mortem (loin) QTL (21605) | 3 | 1.05E+08 | 1.12E+08 |
| ENSSSCG00000026465 | 3 | 1.11E+08 | 1.11E+08 | pH 24 hr post-mortem (loin) QTL (21606) | 3 | 1.05E+08 | 1.12E+08 |
| ENSSSCG00000026465 | 3 | 1.11E+08 | 1.11E+08 | pH 24 hr post mortem (ham) QTL (21603) | 3 | 1.05E+08 | 1.12E+08 |
| ENSSSCG00000026465 | 3 | 1.11E+08 | 1.11E+08 | 45 min-24 h pH decline QTL (21600) | 3 | 1.05E+08 | 1.12E+08 |
| ENSSSCG00000026465 | 3 | 1.11E+08 | 1.11E+08 | pH 24 hr post mortem (ham) QTL (21601) | 3 | 1.05E+08 | 1.12E+08 |
| ENSSSCG00000026465 | 3 | 1.11E+08 | 1.11E+08 | pH 24 hr post mortem (ham) QTL (21602) | 3 | 1.05E+08 | 1.12E+08 |
| ENSSSCG00000026465 | 3 | 1.11E+08 | 1.11E+08 | 45 min-24 h pH decline QTL (21599) | 3 | 1.05E+08 | 1.12E+08 |
| ENSSSCG00000026465 | 3 | 1.11E+08 | 1.11E+08 | Drip loss QTL (21596) | 3 | 1.05E+08 | 1.12E+08 |
| ENSSSCG00000026465 | 3 | 1.11E+08 | 1.11E+08 | Conductivity 24 hours post-mortem QTL (21597) | 3 | 1.05E+08 | 1.12E+08 |
| ENSSSCG00000026465 | 3 | 1.11E+08 | 1.11E+08 | Drip loss QTL (21595) | 3 | 1.05E+08 | 1.12E+08 |
| ENSSSCG00000026465 | 3 | 1.11E+08 | 1.11E+08 | muscle protein percentage QTL (5545) | 3 | 1.06E+08 | 1.23E+08 |
| ENSSSCG00000026465 | 3 | 1.11E+08 | 1.11E+08 | Body weight (end of test) QTL (5234) | 3 | 2979596 | 1.33E+08 |
| ENSSSCG00000026465 | 3 | 1.11E+08 | 1.11E+08 | Cooking loss QTL (38079) | 3 | 12740342 | 1.33E+08 |
| ENSSSCG00000026465 | 3 | 1.11E+08 | 1.11E+08 | pH 45 minutes post mortem QTL (3227) | 3 | 26302643 | 1.22E+08 |
| ENSSSCG00000026465 | 3 | 1.11E+08 | 1.11E+08 | Fat protein content QTL (18011) | 3 | 66578565 | 1.16E+08 |
| ENSSSCG00000008635 | 3 | 1.26E+08 | 1.26E+08 | Loin muscle area QTL (3229) | 3 | 1.13E+08 | 1.33E+08 |
| ENSSSCG00000008635 | 3 | 1.26E+08 | 1.26E+08 | Body weight (end of test) QTL (5234) | 3 | 2979596 | 1.33E+08 |
| ENSSSCG00000008635 | 3 | 1.26E+08 | 1.26E+08 | Cooking loss QTL (38079) | 3 | 12740342 | 1.33E+08 |
| ENSSSCG00000005916 | 4 | 588636 | 597695 | Average daily gain QTL (2890) | 4 | 19986 | 679536 |
| ENSSSCG00000005916 | 4 | 588636 | 597695 | Average backfat thickness QTL (2909) | 4 | 19986 | 679536 |
| ENSSSCG00000006137 | 4 | 50370088 | 50509113 | Average daily gain QTL (442) | 4 | 27081544 | 66054985 |
| ENSSSCG00000006137 | 4 | 50370088 | 50509113 | Carcass length QTL (5076) | 4 | 33197377 | 56101768 |
| ENSSSCG00000006137 | 4 | 50370088 | 50509113 | Body weight (slaughter) QTL (5095) | 4 | 34996151 | 56821278 |
| ENSSSCG00000006137 | 4 | 50370088 | 50509113 | Shoulder subcutaneous fat thickness QTL (7266) | 4 | 34996151 | 56821278 |
| ENSSSCG00000006137 | 4 | 50370088 | 50509113 | Backfat at last rib QTL (7273) | 4 | 34996151 | 56821278 |
| ENSSSCG00000006137 | 4 | 50370088 | 50509113 | Carcass length QTL (7286) | 4 | 34996151 | 56821278 |
| ENSSSCG00000006137 | 4 | 50370088 | 50509113 | Body weight (slaughter) QTL (7290) | 4 | 34996151 | 56821278 |
| ENSSSCG00000006137 | 4 | 50370088 | 50509113 | Loin muscle area QTL (3869) | 4 | 34996151 | 56821278 |
| ENSSSCG00000006137 | 4 | 50370088 | 50509113 | Shoulder subcutaneous fat thickness QTL (7263) | 4 | 35685681 | 56821278 |
| ENSSSCG00000006137 | 4 | 50370088 | 50509113 | Shoulder subcutaneous fat thickness QTL (7264) | 4 | 35685681 | 56821278 |
| ENSSSCG00000006137 | 4 | 50370088 | 50509113 | Shoulder subcutaneous fat thickness QTL (7265) | 4 | 35685681 | 56821278 |
| ENSSSCG00000006137 | 4 | 50370088 | 50509113 | Backfat at last rib QTL (7270) | 4 | 35685681 | 56821278 |
| ENSSSCG00000006137 | 4 | 50370088 | 50509113 | Backfat at last rib QTL (7271) | 4 | 35685681 | 56821278 |
| ENSSSCG00000006137 | 4 | 50370088 | 50509113 | Backfat at last rib QTL (7272) | 4 | 35685681 | 56821278 |
| ENSSSCG00000006137 | 4 | 50370088 | 50509113 | Carcass length QTL (7283) | 4 | 35685681 | 56821278 |
| ENSSSCG00000006137 | 4 | 50370088 | 50509113 | Carcass length QTL (7284) | 4 | 35685681 | 56821278 |
| ENSSSCG00000006137 | 4 | 50370088 | 50509113 | Carcass length QTL (7285) | 4 | 35685681 | 56821278 |
| ENSSSCG00000006137 | 4 | 50370088 | 50509113 | Stearic acid content QTL (9576) | 4 | 41771534 | 51165132 |
| ENSSSCG00000006137 | 4 | 50370088 | 50509113 | Oleic acid content QTL (9578) | 4 | 41771534 | 51165132 |
| ENSSSCG00000006137 | 4 | 50370088 | 50509113 | Carcass length QTL (4891) | 4 | 47367720 | 61558050 |
| ENSSSCG00000006137 | 4 | 50370088 | 50509113 | Body weight (30 weeks) QTL (21225) | 4 | 47467652 | 66154917 |
| ENSSSCG00000006137 | 4 | 50370088 | 50509113 | Adipocyte diameter QTL (12827) | 4 | 47967312 | 62257573 |
| ENSSSCG00000006137 | 4 | 50370088 | 50509113 | Adipocyte diameter QTL (12828) | 4 | 47967312 | 62257573 |
| ENSSSCG00000006137 | 4 | 50370088 | 50509113 | Intramuscular fat content QTL (313) | 4 | 4097207 | 69552602 |
| ENSSSCG00000006137 | 4 | 50370088 | 50509113 | Palmitoleic acid content QTL (156925) | 4 | 7994552 | 1.2E+08 |
| ENSSSCG00000006137 | 4 | 50370088 | 50509113 | Oleic acid content QTL (156927) | 4 | 7994552 | 1.2E+08 |
| ENSSSCG00000006137 | 4 | 50370088 | 50509113 | Linoleic acid content QTL (156928) | 4 | 7994552 | 1.2E+08 |
| ENSSSCG00000006137 | 4 | 50370088 | 50509113 | Monounsaturated fatty acid content QTL (156929) | 4 | 7994552 | 1.2E+08 |
| ENSSSCG00000006137 | 4 | 50370088 | 50509113 | Drip loss QTL (38084) | 4 | 7994552 | 1.2E+08 |
| ENSSSCG00000006137 | 4 | 50370088 | 50509113 | Intestinal fat weight QTL (21233) | 4 | 12391555 | 80744975 |
| ENSSSCG00000006137 | 4 | 50370088 | 50509113 | Average daily gain QTL (245) | 4 | 26981613 | 1.25E+08 |
| ENSSSCG00000006137 | 4 | 50370088 | 50509113 | Backfat at mid-back QTL (5243) | 4 | 26981613 | 1.19E+08 |
| ENSSSCG00000006137 | 4 | 50370088 | 50509113 | Marbling QTL (38082) | 4 | 27081544 | 1.2E+08 |
| ENSSSCG00000006137 | 4 | 50370088 | 50509113 | Average daily gain QTL (370) | 4 | 27081544 | 1.06E+08 |
| ENSSSCG00000006137 | 4 | 50370088 | 50509113 | Carcass weight (hot) QTL (16894) | 4 | 27081544 | 86940753 |
| ENSSSCG00000006137 | 4 | 50370088 | 50509113 | Average daily gain QTL (443) | 4 | 27081544 | 79245996 |
| ENSSSCG00000006137 | 4 | 50370088 | 50509113 | Body weight (end of test) QTL (5235) | 4 | 33177390 | 1.11E+08 |
| ENSSSCG00000006137 | 4 | 50370088 | 50509113 | Body weight (46 days) QTL (21227) | 4 | 33277322 | 1.21E+08 |
| ENSSSCG00000006137 | 4 | 50370088 | 50509113 | Average daily gain QTL (3091) | 4 | 39972760 | 91937348 |
| ENSSSCG00000006137 | 4 | 50370088 | 50509113 | Loin muscle area QTL (3032) | 4 | 41771534 | 79245996 |
| ENSSSCG00000006137 | 4 | 50370088 | 50509113 | Carcass length QTL (3033) | 4 | 41771534 | 79245996 |
| ENSSSCG00000006137 | 4 | 50370088 | 50509113 | Average backfat thickness QTL (3034) | 4 | 41771534 | 79245996 |
| ENSSSCG00000006137 | 4 | 50370088 | 50509113 | Average daily gain QTL (3035) | 4 | 41771534 | 79245996 |
| ENSSSCG00000006137 | 4 | 50370088 | 50509113 | Fat androstenone level QTL (598) | 4 | 41771534 | 79245996 |
| ENSSSCG00000006137 | 4 | 50370088 | 50509113 | Fat androstenone level QTL (599) | 4 | 41771534 | 79245996 |
| ENSSSCG00000006137 | 4 | 50370088 | 50509113 | Fat androstenone level QTL (600) | 4 | 41771534 | 79245996 |
| ENSSSCG00000006137 | 4 | 50370088 | 50509113 | Loin muscle area QTL (461) | 4 | 41771534 | 79245996 |
| ENSSSCG00000006137 | 4 | 50370088 | 50509113 | Carcass length QTL (462) | 4 | 41771534 | 79245996 |
| ENSSSCG00000006137 | 4 | 50370088 | 50509113 | Backfat at mid-back QTL (463) | 4 | 41771534 | 79245996 |
| ENSSSCG00000006137 | 4 | 50370088 | 50509113 | Average backfat thickness QTL (200) | 4 | 41771534 | 79245996 |
| ENSSSCG00000006137 | 4 | 50370088 | 50509113 | Average backfat thickness QTL (201) | 4 | 41771534 | 79245996 |
| ENSSSCG00000006137 | 4 | 50370088 | 50509113 | HDL/LDL ratio QTL (17717) | 4 | 41771534 | 79245996 |
| ENSSSCG00000006137 | 4 | 50370088 | 50509113 | Average daily gain QTL (444) | 4 | 41771534 | 74349333 |
| ENSSSCG00000006137 | 4 | 50370088 | 50509113 | Body weight QTL (8801) | 4 | 41771534 | 69552602 |
| ENSSSCG00000006137 | 4 | 50370088 | 50509113 | Body weight QTL (8802) | 4 | 41771534 | 69552602 |
| ENSSSCG00000006137 | 4 | 50370088 | 50509113 | Average backfat thickness QTL (270) | 4 | 42970717 | 78946201 |
| ENSSSCG00000006137 | 4 | 50370088 | 50509113 | body weight (17 weeks) QTL (21224) | 4 | 43970036 | 1.01E+08 |
| ENSSSCG00000006137 | 4 | 50370088 | 50509113 | Abdominal fat weight QTL (261) | 4 | 45968674 | 84942115 |
| ENSSSCG00000006137 | 4 | 50370088 | 50509113 | Carcass length QTL (4890) | 4 | 46668197 | 75548516 |
| ENSSSCG00000006137 | 4 | 50370088 | 50509113 | Average daily gain QTL (458) | 4 | 47767448 | 1.06E+08 |
| ENSSSCG00000006137 | 4 | 50370088 | 50509113 | Body weight (slaughter) QTL (634) | 4 | 48287094 | 93646183 |
| ENSSSCG00000006137 | 4 | 50370088 | 50509113 | Carcass fat-free weight QTL (635) | 4 | 48287094 | 93646183 |
| ENSSSCG00000006137 | 4 | 50370088 | 50509113 | Age at slaughter QTL (638) | 4 | 48287094 | 93646183 |
| ENSSSCG00000006137 | 4 | 50370088 | 50509113 | Average backfat thickness QTL (371) | 4 | 48966631 | 1.03E+08 |
| ENSSSCG00000006179 | 4 | 62139505 | 62455501 | Loin muscle area QTL (16956) | 4 | 62257573 | 66054985 |
| ENSSSCG00000006179 | 4 | 62139505 | 62455501 | Carcass length QTL (16957) | 4 | 62257573 | 66054985 |
| ENSSSCG00000006179 | 4 | 62139505 | 62455501 | Average backfat thickness QTL (16958) | 4 | 62257573 | 66054985 |
| ENSSSCG00000006179 | 4 | 62139505 | 62455501 | Average daily gain QTL (16959) | 4 | 62257573 | 66054985 |
| ENSSSCG00000006179 | 4 | 62139505 | 62455501 | Average daily gain QTL (442) | 4 | 27081544 | 66054985 |
| ENSSSCG00000006179 | 4 | 62139505 | 62455501 | Body weight (30 weeks) QTL (21225) | 4 | 47467652 | 66154917 |
| ENSSSCG00000006179 | 4 | 62139505 | 62455501 | Intramuscular fat content QTL (313) | 4 | 4097207 | 69552602 |
| ENSSSCG00000006179 | 4 | 62139505 | 62455501 | Palmitoleic acid content QTL (156925) | 4 | 7994552 | 1.2E+08 |
| ENSSSCG00000006179 | 4 | 62139505 | 62455501 | Oleic acid content QTL (156927) | 4 | 7994552 | 1.2E+08 |
| ENSSSCG00000006179 | 4 | 62139505 | 62455501 | Linoleic acid content QTL (156928) | 4 | 7994552 | 1.2E+08 |
| ENSSSCG00000006179 | 4 | 62139505 | 62455501 | Monounsaturated fatty acid content QTL (156929) | 4 | 7994552 | 1.2E+08 |
| ENSSSCG00000006179 | 4 | 62139505 | 62455501 | Drip loss QTL (38084) | 4 | 7994552 | 1.2E+08 |
| ENSSSCG00000006179 | 4 | 62139505 | 62455501 | Intestinal fat weight QTL (21233) | 4 | 12391555 | 80744975 |
| ENSSSCG00000006179 | 4 | 62139505 | 62455501 | Average daily gain QTL (245) | 4 | 26981613 | 1.25E+08 |
| ENSSSCG00000006179 | 4 | 62139505 | 62455501 | Backfat at mid-back QTL (5243) | 4 | 26981613 | 1.19E+08 |
| ENSSSCG00000006179 | 4 | 62139505 | 62455501 | Marbling QTL (38082) | 4 | 27081544 | 1.2E+08 |
| ENSSSCG00000006179 | 4 | 62139505 | 62455501 | Average daily gain QTL (370) | 4 | 27081544 | 1.06E+08 |
| ENSSSCG00000006179 | 4 | 62139505 | 62455501 | Carcass weight (hot) QTL (16894) | 4 | 27081544 | 86940753 |
| ENSSSCG00000006179 | 4 | 62139505 | 62455501 | Average daily gain QTL (443) | 4 | 27081544 | 79245996 |
| ENSSSCG00000006179 | 4 | 62139505 | 62455501 | Body weight (end of test) QTL (5235) | 4 | 33177390 | 1.11E+08 |
| ENSSSCG00000006179 | 4 | 62139505 | 62455501 | Body weight (46 days) QTL (21227) | 4 | 33277322 | 1.21E+08 |
| ENSSSCG00000006179 | 4 | 62139505 | 62455501 | Average daily gain QTL (3091) | 4 | 39972760 | 91937348 |
| ENSSSCG00000006179 | 4 | 62139505 | 62455501 | Loin muscle area QTL (3032) | 4 | 41771534 | 79245996 |
| ENSSSCG00000006179 | 4 | 62139505 | 62455501 | Carcass length QTL (3033) | 4 | 41771534 | 79245996 |
| ENSSSCG00000006179 | 4 | 62139505 | 62455501 | Average backfat thickness QTL (3034) | 4 | 41771534 | 79245996 |
| ENSSSCG00000006179 | 4 | 62139505 | 62455501 | Average daily gain QTL (3035) | 4 | 41771534 | 79245996 |
| ENSSSCG00000006179 | 4 | 62139505 | 62455501 | Fat androstenone level QTL (598) | 4 | 41771534 | 79245996 |
| ENSSSCG00000006179 | 4 | 62139505 | 62455501 | Fat androstenone level QTL (599) | 4 | 41771534 | 79245996 |
| ENSSSCG00000006179 | 4 | 62139505 | 62455501 | Fat androstenone level QTL (600) | 4 | 41771534 | 79245996 |
| ENSSSCG00000006179 | 4 | 62139505 | 62455501 | Loin muscle area QTL (461) | 4 | 41771534 | 79245996 |
| ENSSSCG00000006179 | 4 | 62139505 | 62455501 | Carcass length QTL (462) | 4 | 41771534 | 79245996 |
| ENSSSCG00000006179 | 4 | 62139505 | 62455501 | Backfat at mid-back QTL (463) | 4 | 41771534 | 79245996 |
| ENSSSCG00000006179 | 4 | 62139505 | 62455501 | Average backfat thickness QTL (200) | 4 | 41771534 | 79245996 |
| ENSSSCG00000006179 | 4 | 62139505 | 62455501 | Average backfat thickness QTL (201) | 4 | 41771534 | 79245996 |
| ENSSSCG00000006179 | 4 | 62139505 | 62455501 | HDL/LDL ratio QTL (17717) | 4 | 41771534 | 79245996 |
| ENSSSCG00000006179 | 4 | 62139505 | 62455501 | Average daily gain QTL (444) | 4 | 41771534 | 74349333 |
| ENSSSCG00000006179 | 4 | 62139505 | 62455501 | Body weight QTL (8801) | 4 | 41771534 | 69552602 |
| ENSSSCG00000006179 | 4 | 62139505 | 62455501 | Body weight QTL (8802) | 4 | 41771534 | 69552602 |
| ENSSSCG00000006179 | 4 | 62139505 | 62455501 | Average backfat thickness QTL (270) | 4 | 42970717 | 78946201 |
| ENSSSCG00000006179 | 4 | 62139505 | 62455501 | body weight (17 weeks) QTL (21224) | 4 | 43970036 | 1.01E+08 |
| ENSSSCG00000006179 | 4 | 62139505 | 62455501 | Abdominal fat weight QTL (261) | 4 | 45968674 | 84942115 |
| ENSSSCG00000006179 | 4 | 62139505 | 62455501 | Carcass length QTL (4890) | 4 | 46668197 | 75548516 |
| ENSSSCG00000006179 | 4 | 62139505 | 62455501 | Average daily gain QTL (458) | 4 | 47767448 | 1.06E+08 |
| ENSSSCG00000006179 | 4 | 62139505 | 62455501 | Body weight (slaughter) QTL (634) | 4 | 48287094 | 93646183 |
| ENSSSCG00000006179 | 4 | 62139505 | 62455501 | Carcass fat-free weight QTL (635) | 4 | 48287094 | 93646183 |
| ENSSSCG00000006179 | 4 | 62139505 | 62455501 | Age at slaughter QTL (638) | 4 | 48287094 | 93646183 |
| ENSSSCG00000006179 | 4 | 62139505 | 62455501 | Average backfat thickness QTL (371) | 4 | 48966631 | 1.03E+08 |
| ENSSSCG00000006179 | 4 | 62139505 | 62455501 | Body weight (34 weeks) QTL (21226) | 4 | 50965269 | 69752466 |
| ENSSSCG00000006179 | 4 | 62139505 | 62455501 | Carcass length QTL (10) | 4 | 51165132 | 71151512 |
| ENSSSCG00000006179 | 4 | 62139505 | 62455501 | Body weight (end of test) QTL (5200) | 4 | 51664792 | 1.01E+08 |
| ENSSSCG00000006179 | 4 | 62139505 | 62455501 | Shoulder subcutaneous fat thickness QTL (21232) | 4 | 52164451 | 74349333 |
| ENSSSCG00000006179 | 4 | 62139505 | 62455501 | Body weight (3 weeks) QTL (175) | 4 | 53963226 | 98932581 |
| ENSSSCG00000006179 | 4 | 62139505 | 62455501 | Intramuscular fat content QTL (3871) | 4 | 55861932 | 1.3E+08 |
| ENSSSCG00000006179 | 4 | 62139505 | 62455501 | Average daily gain QTL (511) | 4 | 55861932 | 79245996 |
| ENSSSCG00000006179 | 4 | 62139505 | 62455501 | Abdominal fat weight QTL (631) | 4 | 55861932 | 79245996 |
| ENSSSCG00000006179 | 4 | 62139505 | 62455501 | Subcutaneous fat thickness QTL (632) | 4 | 55861932 | 79245996 |
| ENSSSCG00000006179 | 4 | 62139505 | 62455501 | Average daily gain QTL (636) | 4 | 55861932 | 79245996 |
| ENSSSCG00000006179 | 4 | 62139505 | 62455501 | Carcass length QTL (637) | 4 | 55861932 | 79245996 |
| ENSSSCG00000006179 | 4 | 62139505 | 62455501 | Average backfat thickness QTL (350) | 4 | 55961864 | 69552602 |
| ENSSSCG00000006179 | 4 | 62139505 | 62455501 | Average daily gain QTL (179) | 4 | 56961183 | 97933262 |
| ENSSSCG00000006179 | 4 | 62139505 | 62455501 | body weight (10 weeks) QTL (176) | 4 | 56961183 | 78946201 |
| ENSSSCG00000006179 | 4 | 62139505 | 62455501 | Abdominal fat weight QTL (21223) | 4 | 56961183 | 69752466 |
| ENSSSCG00000006179 | 4 | 62139505 | 62455501 | Average daily gain QTL (180) | 4 | 57960502 | 85941434 |
| ENSSSCG00000006179 | 4 | 62139505 | 62455501 | Average backfat thickness QTL (17541) | 4 | 58360229 | 71851036 |
| ENSSSCG00000006179 | 4 | 62139505 | 62455501 | body weight (17 weeks) QTL (178) | 4 | 58959821 | 83942796 |
| ENSSSCG00000006179 | 4 | 62139505 | 62455501 | Average daily gain QTL (17542) | 4 | 59559412 | 78046813 |
| ENSSSCG00000006179 | 4 | 62139505 | 62455501 | Backfat thickness between 3rd and 4th rib QTL (410) | 4 | 59959140 | 80944839 |
| ENSSSCG00000006179 | 4 | 62139505 | 62455501 | body weight (13 weeks) QTL (177) | 4 | 60958459 | 82943477 |
| ENSSSCG00000006179 | 4 | 62139505 | 62455501 | Carcass length QTL (671) | 4 | 60958459 | 76947563 |
| ENSSSCG00000006179 | 4 | 62139505 | 62455501 | Backfat at last rib QTL (411) | 4 | 61957778 | 80944839 |
| ENSSSCG00000006179 | 4 | 62139505 | 62455501 | Shear force at first peak QTL (3933) | 4 | 62257573 | 1.3E+08 |
| ENSSSCG00000006179 | 4 | 62139505 | 62455501 | Total shear work QTL (3934) | 4 | 62257573 | 1.3E+08 |
| ENSSSCG00000006179 | 4 | 62139505 | 62455501 | Average backfat thickness QTL (162) | 4 | 62257573 | 1.2E+08 |
| ENSSSCG00000006179 | 4 | 62139505 | 62455501 | Average daily gain QTL (3137) | 4 | 62257573 | 76947563 |
| ENSSSCG00000006179 | 4 | 62139505 | 62455501 | Average daily gain QTL (3138) | 4 | 62257573 | 76947563 |
| ENSSSCG00000006179 | 4 | 62139505 | 62455501 | Backfat at last lumbar QTL (3157) | 4 | 62257573 | 76947563 |
| ENSSSCG00000006179 | 4 | 62139505 | 62455501 | Backfat thickness between 3rd and 4th rib QTL (3158) | 4 | 62257573 | 76947563 |
| ENSSSCG00000006179 | 4 | 62139505 | 62455501 | Carcass weight (hot) QTL (16863) | 4 | 62257573 | 75648448 |
| ENSSSCG00000006179 | 4 | 62139505 | 62455501 | Intramuscular fat content QTL (309) | 4 | 62257573 | 71950968 |
| ENSSSCG00000006179 | 4 | 62139505 | 62455501 | Loin muscle area QTL (976) | 4 | 62257573 | 71950968 |
| ENSSSCG00000006179 | 4 | 62139505 | 62455501 | Oleic acid content QTL (3127) | 4 | 62257573 | 69552602 |
| ENSSSCG00000006179 | 4 | 62139505 | 62455501 | Linoleic acid content QTL (3130) | 4 | 62257573 | 69552602 |
| ENSSSCG00000006179 | 4 | 62139505 | 62455501 | Linoleic acid content QTL (3131) | 4 | 62257573 | 69552602 |
| ENSSSCG00000006179 | 4 | 62139505 | 62455501 | Average daily gain QTL (942) | 4 | 62257573 | 69552602 |
| ENSSSCG00000006267 | 4 | 78098999 | 78162969 | Loin muscle area QTL (672) | 4 | 67953692 | 99931900 |
| ENSSSCG00000006267 | 4 | 78098999 | 78162969 | Average daily gain QTL (456) | 4 | 69552602 | 1.03E+08 |
| ENSSSCG00000006267 | 4 | 78098999 | 78162969 | Linoleic acid content QTL (4203) | 4 | 69552602 | 80445179 |
| ENSSSCG00000006267 | 4 | 78098999 | 78162969 | Linoleic acid content QTL (535) | 4 | 70951649 | 85941434 |
| ENSSSCG00000006267 | 4 | 78098999 | 78162969 | Water holding capacity QTL (3847) | 4 | 71151512 | 1.06E+08 |
| ENSSSCG00000006267 | 4 | 78098999 | 78162969 | Backfat at mid-back QTL (445) | 4 | 71151512 | 80445179 |
| ENSSSCG00000006267 | 4 | 78098999 | 78162969 | Stearic acid content QTL (156926) | 4 | 71950968 | 1.2E+08 |
| ENSSSCG00000006267 | 4 | 78098999 | 78162969 | Capric acid content QTL (156870) | 4 | 71950968 | 1.2E+08 |
| ENSSSCG00000006267 | 4 | 78098999 | 78162969 | Loin muscle area QTL (528) | 4 | 71950968 | 92936667 |
| ENSSSCG00000006267 | 4 | 78098999 | 78162969 | Diameter of type IIb muscle fibers QTL (2807) | 4 | 72950287 | 1.01E+08 |
| ENSSSCG00000006267 | 4 | 78098999 | 78162969 | Number of muscle fibers per unit area QTL (2808) | 4 | 72950287 | 1.01E+08 |
| ENSSSCG00000006267 | 4 | 78098999 | 78162969 | Diameter of muscle fibers QTL (2809) | 4 | 72950287 | 1.01E+08 |
| ENSSSCG00000006267 | 4 | 78098999 | 78162969 | Diameter of type IIb muscle fibers QTL (2810) | 4 | 72950287 | 1.01E+08 |
| ENSSSCG00000006267 | 4 | 78098999 | 78162969 | Average backfat thickness QTL (527) | 4 | 72950287 | 90938029 |
| ENSSSCG00000006267 | 4 | 78098999 | 78162969 | subjective pork odor QTL (4207) | 4 | 74349333 | 1.06E+08 |
| ENSSSCG00000006267 | 4 | 78098999 | 78162969 | Margaric acid content QTL (158) | 4 | 74349333 | 1.06E+08 |
| ENSSSCG00000006267 | 4 | 78098999 | 78162969 | Conductivity 24 hours post-mortem QTL (961) | 4 | 74349333 | 80944839 |
| ENSSSCG00000006267 | 4 | 78098999 | 78162969 | Backfat at last rib QTL (965) | 4 | 74349333 | 80944839 |
| ENSSSCG00000006267 | 4 | 78098999 | 78162969 | Average daily gain QTL (972) | 4 | 74349333 | 80944839 |
| ENSSSCG00000006267 | 4 | 78098999 | 78162969 | Average daily gain QTL (331) | 4 | 74349333 | 79245996 |
| ENSSSCG00000006267 | 4 | 78098999 | 78162969 | Average daily gain QTL (341) | 4 | 74349333 | 79245996 |
| ENSSSCG00000006267 | 4 | 78098999 | 78162969 | Meat color-L QTL (233) | 4 | 74349333 | 79245996 |
| ENSSSCG00000006267 | 4 | 78098999 | 78162969 | Conductivity 24 hours postmortem (loin) QTL (21856) | 4 | 76148107 | 85641638 |
| ENSSSCG00000006267 | 4 | 78098999 | 78162969 | pH 45 minutes post mortem QTL (21850) | 4 | 76647767 | 85641638 |
| ENSSSCG00000006267 | 4 | 78098999 | 78162969 | Meat color-a QTL (294) | 4 | 76947563 | 1.12E+08 |
| ENSSSCG00000006267 | 4 | 78098999 | 78162969 | Palmitoleic acid content QTL (156925) | 4 | 7994552 | 1.2E+08 |
| ENSSSCG00000006267 | 4 | 78098999 | 78162969 | Oleic acid content QTL (156927) | 4 | 7994552 | 1.2E+08 |
| ENSSSCG00000006267 | 4 | 78098999 | 78162969 | Linoleic acid content QTL (156928) | 4 | 7994552 | 1.2E+08 |
| ENSSSCG00000006267 | 4 | 78098999 | 78162969 | Monounsaturated fatty acid content QTL (156929) | 4 | 7994552 | 1.2E+08 |
| ENSSSCG00000006267 | 4 | 78098999 | 78162969 | Drip loss QTL (38084) | 4 | 7994552 | 1.2E+08 |
| ENSSSCG00000006267 | 4 | 78098999 | 78162969 | Intestinal fat weight QTL (21233) | 4 | 12391555 | 80744975 |
| ENSSSCG00000006267 | 4 | 78098999 | 78162969 | Average daily gain QTL (245) | 4 | 26981613 | 1.25E+08 |
| ENSSSCG00000006267 | 4 | 78098999 | 78162969 | Backfat at mid-back QTL (5243) | 4 | 26981613 | 1.19E+08 |
| ENSSSCG00000006267 | 4 | 78098999 | 78162969 | Marbling QTL (38082) | 4 | 27081544 | 1.2E+08 |
| ENSSSCG00000006267 | 4 | 78098999 | 78162969 | Average daily gain QTL (370) | 4 | 27081544 | 1.06E+08 |
| ENSSSCG00000006267 | 4 | 78098999 | 78162969 | Carcass weight (hot) QTL (16894) | 4 | 27081544 | 86940753 |
| ENSSSCG00000006267 | 4 | 78098999 | 78162969 | Average daily gain QTL (443) | 4 | 27081544 | 79245996 |
| ENSSSCG00000006267 | 4 | 78098999 | 78162969 | Body weight (end of test) QTL (5235) | 4 | 33177390 | 1.11E+08 |
| ENSSSCG00000006267 | 4 | 78098999 | 78162969 | Body weight (46 days) QTL (21227) | 4 | 33277322 | 1.21E+08 |
| ENSSSCG00000006267 | 4 | 78098999 | 78162969 | Average daily gain QTL (3091) | 4 | 39972760 | 91937348 |
| ENSSSCG00000006267 | 4 | 78098999 | 78162969 | Loin muscle area QTL (3032) | 4 | 41771534 | 79245996 |
| ENSSSCG00000006267 | 4 | 78098999 | 78162969 | Carcass length QTL (3033) | 4 | 41771534 | 79245996 |
| ENSSSCG00000006267 | 4 | 78098999 | 78162969 | Average backfat thickness QTL (3034) | 4 | 41771534 | 79245996 |
| ENSSSCG00000006267 | 4 | 78098999 | 78162969 | Average daily gain QTL (3035) | 4 | 41771534 | 79245996 |
| ENSSSCG00000006267 | 4 | 78098999 | 78162969 | Fat androstenone level QTL (598) | 4 | 41771534 | 79245996 |
| ENSSSCG00000006267 | 4 | 78098999 | 78162969 | Fat androstenone level QTL (599) | 4 | 41771534 | 79245996 |
| ENSSSCG00000006267 | 4 | 78098999 | 78162969 | Fat androstenone level QTL (600) | 4 | 41771534 | 79245996 |
| ENSSSCG00000006267 | 4 | 78098999 | 78162969 | Loin muscle area QTL (461) | 4 | 41771534 | 79245996 |
| ENSSSCG00000006267 | 4 | 78098999 | 78162969 | Carcass length QTL (462) | 4 | 41771534 | 79245996 |
| ENSSSCG00000006267 | 4 | 78098999 | 78162969 | Backfat at mid-back QTL (463) | 4 | 41771534 | 79245996 |
| ENSSSCG00000006267 | 4 | 78098999 | 78162969 | Average backfat thickness QTL (200) | 4 | 41771534 | 79245996 |
| ENSSSCG00000006267 | 4 | 78098999 | 78162969 | Average backfat thickness QTL (201) | 4 | 41771534 | 79245996 |
| ENSSSCG00000006267 | 4 | 78098999 | 78162969 | HDL/LDL ratio QTL (17717) | 4 | 41771534 | 79245996 |
| ENSSSCG00000006267 | 4 | 78098999 | 78162969 | Average backfat thickness QTL (270) | 4 | 42970717 | 78946201 |
| ENSSSCG00000006267 | 4 | 78098999 | 78162969 | body weight (17 weeks) QTL (21224) | 4 | 43970036 | 1.01E+08 |
| ENSSSCG00000006267 | 4 | 78098999 | 78162969 | Abdominal fat weight QTL (261) | 4 | 45968674 | 84942115 |
| ENSSSCG00000006267 | 4 | 78098999 | 78162969 | Average daily gain QTL (458) | 4 | 47767448 | 1.06E+08 |
| ENSSSCG00000006267 | 4 | 78098999 | 78162969 | Body weight (slaughter) QTL (634) | 4 | 48287094 | 93646183 |
| ENSSSCG00000006267 | 4 | 78098999 | 78162969 | Carcass fat-free weight QTL (635) | 4 | 48287094 | 93646183 |
| ENSSSCG00000006267 | 4 | 78098999 | 78162969 | Age at slaughter QTL (638) | 4 | 48287094 | 93646183 |
| ENSSSCG00000006267 | 4 | 78098999 | 78162969 | Average backfat thickness QTL (371) | 4 | 48966631 | 1.03E+08 |
| ENSSSCG00000006267 | 4 | 78098999 | 78162969 | Body weight (end of test) QTL (5200) | 4 | 51664792 | 1.01E+08 |
| ENSSSCG00000006267 | 4 | 78098999 | 78162969 | Body weight (3 weeks) QTL (175) | 4 | 53963226 | 98932581 |
| ENSSSCG00000006267 | 4 | 78098999 | 78162969 | Intramuscular fat content QTL (3871) | 4 | 55861932 | 1.3E+08 |
| ENSSSCG00000006267 | 4 | 78098999 | 78162969 | Average daily gain QTL (511) | 4 | 55861932 | 79245996 |
| ENSSSCG00000006267 | 4 | 78098999 | 78162969 | Abdominal fat weight QTL (631) | 4 | 55861932 | 79245996 |
| ENSSSCG00000006267 | 4 | 78098999 | 78162969 | Subcutaneous fat thickness QTL (632) | 4 | 55861932 | 79245996 |
| ENSSSCG00000006267 | 4 | 78098999 | 78162969 | Average daily gain QTL (636) | 4 | 55861932 | 79245996 |
| ENSSSCG00000006267 | 4 | 78098999 | 78162969 | Carcass length QTL (637) | 4 | 55861932 | 79245996 |
| ENSSSCG00000006267 | 4 | 78098999 | 78162969 | Average daily gain QTL (179) | 4 | 56961183 | 97933262 |
| ENSSSCG00000006267 | 4 | 78098999 | 78162969 | body weight (10 weeks) QTL (176) | 4 | 56961183 | 78946201 |
| ENSSSCG00000006267 | 4 | 78098999 | 78162969 | Average daily gain QTL (180) | 4 | 57960502 | 85941434 |
| ENSSSCG00000006267 | 4 | 78098999 | 78162969 | body weight (17 weeks) QTL (178) | 4 | 58959821 | 83942796 |
| ENSSSCG00000006267 | 4 | 78098999 | 78162969 | Backfat thickness between 3rd and 4th rib QTL (410) | 4 | 59959140 | 80944839 |
| ENSSSCG00000006267 | 4 | 78098999 | 78162969 | body weight (13 weeks) QTL (177) | 4 | 60958459 | 82943477 |
| ENSSSCG00000006267 | 4 | 78098999 | 78162969 | Backfat at last rib QTL (411) | 4 | 61957778 | 80944839 |
| ENSSSCG00000006267 | 4 | 78098999 | 78162969 | Shear force at first peak QTL (3933) | 4 | 62257573 | 1.3E+08 |
| ENSSSCG00000006267 | 4 | 78098999 | 78162969 | Total shear work QTL (3934) | 4 | 62257573 | 1.3E+08 |
| ENSSSCG00000006267 | 4 | 78098999 | 78162969 | Average backfat thickness QTL (162) | 4 | 62257573 | 1.2E+08 |
| ENSSSCG00000006267 | 4 | 78098999 | 78162969 | Average daily gain QTL (17562) | 4 | 62657301 | 97833330 |
| ENSSSCG00000006267 | 4 | 78098999 | 78162969 | Body weight (slaughter) QTL (4892) | 4 | 65855122 | 90938029 |
| ENSSSCG00000006267 | 4 | 78098999 | 78162969 | Backfat at last rib QTL (675) | 4 | 65955054 | 87940072 |
| ENSSSCG00000006267 | 4 | 78098999 | 78162969 | Intramuscular fat content QTL (2982) | 4 | 66054985 | 79245996 |
| ENSSSCG00000006388 | 4 | 90197088 | 90261551 | Loin muscle area QTL (672) | 4 | 67953692 | 99931900 |
| ENSSSCG00000006388 | 4 | 90197088 | 90261551 | Average daily gain QTL (456) | 4 | 69552602 | 1.03E+08 |
| ENSSSCG00000006388 | 4 | 90197088 | 90261551 | Water holding capacity QTL (3847) | 4 | 71151512 | 1.06E+08 |
| ENSSSCG00000006388 | 4 | 90197088 | 90261551 | Stearic acid content QTL (156926) | 4 | 71950968 | 1.2E+08 |
| ENSSSCG00000006388 | 4 | 90197088 | 90261551 | Capric acid content QTL (156870) | 4 | 71950968 | 1.2E+08 |
| ENSSSCG00000006388 | 4 | 90197088 | 90261551 | Loin muscle area QTL (528) | 4 | 71950968 | 92936667 |
| ENSSSCG00000006388 | 4 | 90197088 | 90261551 | Diameter of type IIb muscle fibers QTL (2807) | 4 | 72950287 | 1.01E+08 |
| ENSSSCG00000006388 | 4 | 90197088 | 90261551 | Number of muscle fibers per unit area QTL (2808) | 4 | 72950287 | 1.01E+08 |
| ENSSSCG00000006388 | 4 | 90197088 | 90261551 | Diameter of muscle fibers QTL (2809) | 4 | 72950287 | 1.01E+08 |
| ENSSSCG00000006388 | 4 | 90197088 | 90261551 | Diameter of type IIb muscle fibers QTL (2810) | 4 | 72950287 | 1.01E+08 |
| ENSSSCG00000006388 | 4 | 90197088 | 90261551 | Average backfat thickness QTL (527) | 4 | 72950287 | 90938029 |
| ENSSSCG00000006388 | 4 | 90197088 | 90261551 | subjective pork odor QTL (4207) | 4 | 74349333 | 1.06E+08 |
| ENSSSCG00000006388 | 4 | 90197088 | 90261551 | Margaric acid content QTL (158) | 4 | 74349333 | 1.06E+08 |
| ENSSSCG00000006388 | 4 | 90197088 | 90261551 | Meat color-a QTL (294) | 4 | 76947563 | 1.12E+08 |
| ENSSSCG00000006388 | 4 | 90197088 | 90261551 | Carcass length QTL (460) | 4 | 79245996 | 1.21E+08 |
| ENSSSCG00000006388 | 4 | 90197088 | 90261551 | Drip loss QTL (6003) | 4 | 79245996 | 1.2E+08 |
| ENSSSCG00000006388 | 4 | 90197088 | 90261551 | Oleic acid content QTL (15875) | 4 | 79245996 | 1.03E+08 |
| ENSSSCG00000006388 | 4 | 90197088 | 90261551 | Palmitic acid content QTL (15876) | 4 | 80045451 | 1.14E+08 |
| ENSSSCG00000006388 | 4 | 90197088 | 90261551 | Carcass weight (cold) QTL (3804) | 4 | 80445179 | 1.3E+08 |
| ENSSSCG00000006388 | 4 | 90197088 | 90261551 | Loin muscle area QTL (51) | 4 | 80445179 | 1.2E+08 |
| ENSSSCG00000006388 | 4 | 90197088 | 90261551 | Average daily gain QTL (49) | 4 | 80445179 | 1.08E+08 |
| ENSSSCG00000006388 | 4 | 90197088 | 90261551 | Japanese color scale QTL (4288) | 4 | 80844907 | 1.14E+08 |
| ENSSSCG00000006388 | 4 | 90197088 | 90261551 | Average backfat thickness QTL (676) | 4 | 80944839 | 99931900 |
| ENSSSCG00000006388 | 4 | 90197088 | 90261551 | Skin thickness QTL (31810) | 4 | 81744294 | 1.19E+08 |
| ENSSSCG00000006388 | 4 | 90197088 | 90261551 | Meat color L* QTL (4898) | 4 | 82643681 | 1.04E+08 |
| ENSSSCG00000006388 | 4 | 90197088 | 90261551 | Carcass weight (hot) QTL (16864) | 4 | 82943477 | 1.03E+08 |
| ENSSSCG00000006388 | 4 | 90197088 | 90261551 | Adipocyte diameter QTL (18020) | 4 | 85641638 | 1.08E+08 |
| ENSSSCG00000006388 | 4 | 90197088 | 90261551 | Meat color a* QTL (4899) | 4 | 85941434 | 99232376 |
| ENSSSCG00000006388 | 4 | 90197088 | 90261551 | pH 24 hr post mortem (ham) QTL (4897) | 4 | 89039322 | 1.15E+08 |
| ENSSSCG00000006388 | 4 | 90197088 | 90261551 | Palmitoleic acid content QTL (156925) | 4 | 7994552 | 1.2E+08 |
| ENSSSCG00000006388 | 4 | 90197088 | 90261551 | Oleic acid content QTL (156927) | 4 | 7994552 | 1.2E+08 |
| ENSSSCG00000006388 | 4 | 90197088 | 90261551 | Linoleic acid content QTL (156928) | 4 | 7994552 | 1.2E+08 |
| ENSSSCG00000006388 | 4 | 90197088 | 90261551 | Monounsaturated fatty acid content QTL (156929) | 4 | 7994552 | 1.2E+08 |
| ENSSSCG00000006388 | 4 | 90197088 | 90261551 | Drip loss QTL (38084) | 4 | 7994552 | 1.2E+08 |
| ENSSSCG00000006388 | 4 | 90197088 | 90261551 | Average daily gain QTL (245) | 4 | 26981613 | 1.25E+08 |
| ENSSSCG00000006388 | 4 | 90197088 | 90261551 | Backfat at mid-back QTL (5243) | 4 | 26981613 | 1.19E+08 |
| ENSSSCG00000006388 | 4 | 90197088 | 90261551 | Marbling QTL (38082) | 4 | 27081544 | 1.2E+08 |
| ENSSSCG00000006388 | 4 | 90197088 | 90261551 | Average daily gain QTL (370) | 4 | 27081544 | 1.06E+08 |
| ENSSSCG00000006388 | 4 | 90197088 | 90261551 | Body weight (end of test) QTL (5235) | 4 | 33177390 | 1.11E+08 |
| ENSSSCG00000006388 | 4 | 90197088 | 90261551 | Body weight (46 days) QTL (21227) | 4 | 33277322 | 1.21E+08 |
| ENSSSCG00000006388 | 4 | 90197088 | 90261551 | Average daily gain QTL (3091) | 4 | 39972760 | 91937348 |
| ENSSSCG00000006388 | 4 | 90197088 | 90261551 | body weight (17 weeks) QTL (21224) | 4 | 43970036 | 1.01E+08 |
| ENSSSCG00000006388 | 4 | 90197088 | 90261551 | Average daily gain QTL (458) | 4 | 47767448 | 1.06E+08 |
| ENSSSCG00000006388 | 4 | 90197088 | 90261551 | Body weight (slaughter) QTL (634) | 4 | 48287094 | 93646183 |
| ENSSSCG00000006388 | 4 | 90197088 | 90261551 | Carcass fat-free weight QTL (635) | 4 | 48287094 | 93646183 |
| ENSSSCG00000006388 | 4 | 90197088 | 90261551 | Age at slaughter QTL (638) | 4 | 48287094 | 93646183 |
| ENSSSCG00000006388 | 4 | 90197088 | 90261551 | Average backfat thickness QTL (371) | 4 | 48966631 | 1.03E+08 |
| ENSSSCG00000006388 | 4 | 90197088 | 90261551 | Body weight (end of test) QTL (5200) | 4 | 51664792 | 1.01E+08 |
| ENSSSCG00000006388 | 4 | 90197088 | 90261551 | Body weight (3 weeks) QTL (175) | 4 | 53963226 | 98932581 |
| ENSSSCG00000006388 | 4 | 90197088 | 90261551 | Intramuscular fat content QTL (3871) | 4 | 55861932 | 1.3E+08 |
| ENSSSCG00000006388 | 4 | 90197088 | 90261551 | Average daily gain QTL (179) | 4 | 56961183 | 97933262 |
| ENSSSCG00000006388 | 4 | 90197088 | 90261551 | Shear force at first peak QTL (3933) | 4 | 62257573 | 1.3E+08 |
| ENSSSCG00000006388 | 4 | 90197088 | 90261551 | Total shear work QTL (3934) | 4 | 62257573 | 1.3E+08 |
| ENSSSCG00000006388 | 4 | 90197088 | 90261551 | Average backfat thickness QTL (162) | 4 | 62257573 | 1.2E+08 |
| ENSSSCG00000006388 | 4 | 90197088 | 90261551 | Average daily gain QTL (17562) | 4 | 62657301 | 97833330 |
| ENSSSCG00000006388 | 4 | 90197088 | 90261551 | Body weight (slaughter) QTL (4892) | 4 | 65855122 | 90938029 |
| ENSSSCG00000006390 | 4 | 90276754 | 90288011 | pH 24 hr post-mortem (loin) QTL (7613) | 4 | 90248498 | 90778137 |
| ENSSSCG00000006390 | 4 | 90276754 | 90288011 | Loin muscle area QTL (672) | 4 | 67953692 | 99931900 |
| ENSSSCG00000006390 | 4 | 90276754 | 90288011 | Average daily gain QTL (456) | 4 | 69552602 | 1.03E+08 |
| ENSSSCG00000006390 | 4 | 90276754 | 90288011 | Water holding capacity QTL (3847) | 4 | 71151512 | 1.06E+08 |
| ENSSSCG00000006390 | 4 | 90276754 | 90288011 | Stearic acid content QTL (156926) | 4 | 71950968 | 1.2E+08 |
| ENSSSCG00000006390 | 4 | 90276754 | 90288011 | Capric acid content QTL (156870) | 4 | 71950968 | 1.2E+08 |
| ENSSSCG00000006390 | 4 | 90276754 | 90288011 | Loin muscle area QTL (528) | 4 | 71950968 | 92936667 |
| ENSSSCG00000006390 | 4 | 90276754 | 90288011 | Diameter of type IIb muscle fibers QTL (2807) | 4 | 72950287 | 1.01E+08 |
| ENSSSCG00000006390 | 4 | 90276754 | 90288011 | Number of muscle fibers per unit area QTL (2808) | 4 | 72950287 | 1.01E+08 |
| ENSSSCG00000006390 | 4 | 90276754 | 90288011 | Diameter of muscle fibers QTL (2809) | 4 | 72950287 | 1.01E+08 |
| ENSSSCG00000006390 | 4 | 90276754 | 90288011 | Diameter of type IIb muscle fibers QTL (2810) | 4 | 72950287 | 1.01E+08 |
| ENSSSCG00000006390 | 4 | 90276754 | 90288011 | Average backfat thickness QTL (527) | 4 | 72950287 | 90938029 |
| ENSSSCG00000006390 | 4 | 90276754 | 90288011 | subjective pork odor QTL (4207) | 4 | 74349333 | 1.06E+08 |
| ENSSSCG00000006390 | 4 | 90276754 | 90288011 | Margaric acid content QTL (158) | 4 | 74349333 | 1.06E+08 |
| ENSSSCG00000006390 | 4 | 90276754 | 90288011 | Meat color-a QTL (294) | 4 | 76947563 | 1.12E+08 |
| ENSSSCG00000006390 | 4 | 90276754 | 90288011 | Carcass length QTL (460) | 4 | 79245996 | 1.21E+08 |
| ENSSSCG00000006390 | 4 | 90276754 | 90288011 | Drip loss QTL (6003) | 4 | 79245996 | 1.2E+08 |
| ENSSSCG00000006390 | 4 | 90276754 | 90288011 | Oleic acid content QTL (15875) | 4 | 79245996 | 1.03E+08 |
| ENSSSCG00000006390 | 4 | 90276754 | 90288011 | Palmitic acid content QTL (15876) | 4 | 80045451 | 1.14E+08 |
| ENSSSCG00000006390 | 4 | 90276754 | 90288011 | Carcass weight (cold) QTL (3804) | 4 | 80445179 | 1.3E+08 |
| ENSSSCG00000006390 | 4 | 90276754 | 90288011 | Loin muscle area QTL (51) | 4 | 80445179 | 1.2E+08 |
| ENSSSCG00000006390 | 4 | 90276754 | 90288011 | Average daily gain QTL (49) | 4 | 80445179 | 1.08E+08 |
| ENSSSCG00000006390 | 4 | 90276754 | 90288011 | Japanese color scale QTL (4288) | 4 | 80844907 | 1.14E+08 |
| ENSSSCG00000006390 | 4 | 90276754 | 90288011 | Average backfat thickness QTL (676) | 4 | 80944839 | 99931900 |
| ENSSSCG00000006390 | 4 | 90276754 | 90288011 | Skin thickness QTL (31810) | 4 | 81744294 | 1.19E+08 |
| ENSSSCG00000006390 | 4 | 90276754 | 90288011 | Meat color L* QTL (4898) | 4 | 82643681 | 1.04E+08 |
| ENSSSCG00000006390 | 4 | 90276754 | 90288011 | Carcass weight (hot) QTL (16864) | 4 | 82943477 | 1.03E+08 |
| ENSSSCG00000006390 | 4 | 90276754 | 90288011 | Adipocyte diameter QTL (18020) | 4 | 85641638 | 1.08E+08 |
| ENSSSCG00000006390 | 4 | 90276754 | 90288011 | Meat color a* QTL (4899) | 4 | 85941434 | 99232376 |
| ENSSSCG00000006390 | 4 | 90276754 | 90288011 | pH 24 hr post mortem (ham) QTL (4897) | 4 | 89039322 | 1.15E+08 |
| ENSSSCG00000006390 | 4 | 90276754 | 90288011 | Palmitoleic acid content QTL (156925) | 4 | 7994552 | 1.2E+08 |
| ENSSSCG00000006390 | 4 | 90276754 | 90288011 | Oleic acid content QTL (156927) | 4 | 7994552 | 1.2E+08 |
| ENSSSCG00000006390 | 4 | 90276754 | 90288011 | Linoleic acid content QTL (156928) | 4 | 7994552 | 1.2E+08 |
| ENSSSCG00000006390 | 4 | 90276754 | 90288011 | Monounsaturated fatty acid content QTL (156929) | 4 | 7994552 | 1.2E+08 |
| ENSSSCG00000006390 | 4 | 90276754 | 90288011 | Drip loss QTL (38084) | 4 | 7994552 | 1.2E+08 |
| ENSSSCG00000006390 | 4 | 90276754 | 90288011 | Average daily gain QTL (245) | 4 | 26981613 | 1.25E+08 |
| ENSSSCG00000006390 | 4 | 90276754 | 90288011 | Backfat at mid-back QTL (5243) | 4 | 26981613 | 1.19E+08 |
| ENSSSCG00000006390 | 4 | 90276754 | 90288011 | Marbling QTL (38082) | 4 | 27081544 | 1.2E+08 |
| ENSSSCG00000006390 | 4 | 90276754 | 90288011 | Average daily gain QTL (370) | 4 | 27081544 | 1.06E+08 |
| ENSSSCG00000006390 | 4 | 90276754 | 90288011 | Body weight (end of test) QTL (5235) | 4 | 33177390 | 1.11E+08 |
| ENSSSCG00000006390 | 4 | 90276754 | 90288011 | Body weight (46 days) QTL (21227) | 4 | 33277322 | 1.21E+08 |
| ENSSSCG00000006390 | 4 | 90276754 | 90288011 | Average daily gain QTL (3091) | 4 | 39972760 | 91937348 |
| ENSSSCG00000006390 | 4 | 90276754 | 90288011 | body weight (17 weeks) QTL (21224) | 4 | 43970036 | 1.01E+08 |
| ENSSSCG00000006390 | 4 | 90276754 | 90288011 | Average daily gain QTL (458) | 4 | 47767448 | 1.06E+08 |
| ENSSSCG00000006390 | 4 | 90276754 | 90288011 | Body weight (slaughter) QTL (634) | 4 | 48287094 | 93646183 |
| ENSSSCG00000006390 | 4 | 90276754 | 90288011 | Carcass fat-free weight QTL (635) | 4 | 48287094 | 93646183 |
| ENSSSCG00000006390 | 4 | 90276754 | 90288011 | Age at slaughter QTL (638) | 4 | 48287094 | 93646183 |
| ENSSSCG00000006390 | 4 | 90276754 | 90288011 | Average backfat thickness QTL (371) | 4 | 48966631 | 1.03E+08 |
| ENSSSCG00000006390 | 4 | 90276754 | 90288011 | Body weight (end of test) QTL (5200) | 4 | 51664792 | 1.01E+08 |
| ENSSSCG00000006390 | 4 | 90276754 | 90288011 | Body weight (3 weeks) QTL (175) | 4 | 53963226 | 98932581 |
| ENSSSCG00000006390 | 4 | 90276754 | 90288011 | Intramuscular fat content QTL (3871) | 4 | 55861932 | 1.3E+08 |
| ENSSSCG00000006390 | 4 | 90276754 | 90288011 | Average daily gain QTL (179) | 4 | 56961183 | 97933262 |
| ENSSSCG00000006390 | 4 | 90276754 | 90288011 | Shear force at first peak QTL (3933) | 4 | 62257573 | 1.3E+08 |
| ENSSSCG00000006390 | 4 | 90276754 | 90288011 | Total shear work QTL (3934) | 4 | 62257573 | 1.3E+08 |
| ENSSSCG00000006390 | 4 | 90276754 | 90288011 | Average backfat thickness QTL (162) | 4 | 62257573 | 1.2E+08 |
| ENSSSCG00000006390 | 4 | 90276754 | 90288011 | Average daily gain QTL (17562) | 4 | 62657301 | 97833330 |
| ENSSSCG00000006390 | 4 | 90276754 | 90288011 | Body weight (slaughter) QTL (4892) | 4 | 65855122 | 90938029 |
| ENSSSCG00000006487 | 4 | 93733917 | 93753340 | Palmitic acid content QTL (530) | 4 | 93506278 | 94045911 |
| ENSSSCG00000006487 | 4 | 93733917 | 93753340 | Loin muscle area QTL (672) | 4 | 67953692 | 99931900 |
| ENSSSCG00000006487 | 4 | 93733917 | 93753340 | Average daily gain QTL (456) | 4 | 69552602 | 1.03E+08 |
| ENSSSCG00000006487 | 4 | 93733917 | 93753340 | Water holding capacity QTL (3847) | 4 | 71151512 | 1.06E+08 |
| ENSSSCG00000006487 | 4 | 93733917 | 93753340 | Stearic acid content QTL (156926) | 4 | 71950968 | 1.2E+08 |
| ENSSSCG00000006487 | 4 | 93733917 | 93753340 | Capric acid content QTL (156870) | 4 | 71950968 | 1.2E+08 |
| ENSSSCG00000006487 | 4 | 93733917 | 93753340 | Diameter of type IIb muscle fibers QTL (2807) | 4 | 72950287 | 1.01E+08 |
| ENSSSCG00000006487 | 4 | 93733917 | 93753340 | Number of muscle fibers per unit area QTL (2808) | 4 | 72950287 | 1.01E+08 |
| ENSSSCG00000006487 | 4 | 93733917 | 93753340 | Diameter of muscle fibers QTL (2809) | 4 | 72950287 | 1.01E+08 |
| ENSSSCG00000006487 | 4 | 93733917 | 93753340 | Diameter of type IIb muscle fibers QTL (2810) | 4 | 72950287 | 1.01E+08 |
| ENSSSCG00000006487 | 4 | 93733917 | 93753340 | subjective pork odor QTL (4207) | 4 | 74349333 | 1.06E+08 |
| ENSSSCG00000006487 | 4 | 93733917 | 93753340 | Margaric acid content QTL (158) | 4 | 74349333 | 1.06E+08 |
| ENSSSCG00000006487 | 4 | 93733917 | 93753340 | Meat color-a QTL (294) | 4 | 76947563 | 1.12E+08 |
| ENSSSCG00000006487 | 4 | 93733917 | 93753340 | Carcass length QTL (460) | 4 | 79245996 | 1.21E+08 |
| ENSSSCG00000006487 | 4 | 93733917 | 93753340 | Drip loss QTL (6003) | 4 | 79245996 | 1.2E+08 |
| ENSSSCG00000006487 | 4 | 93733917 | 93753340 | Oleic acid content QTL (15875) | 4 | 79245996 | 1.03E+08 |
| ENSSSCG00000006487 | 4 | 93733917 | 93753340 | Palmitic acid content QTL (15876) | 4 | 80045451 | 1.14E+08 |
| ENSSSCG00000006487 | 4 | 93733917 | 93753340 | Carcass weight (cold) QTL (3804) | 4 | 80445179 | 1.3E+08 |
| ENSSSCG00000006487 | 4 | 93733917 | 93753340 | Loin muscle area QTL (51) | 4 | 80445179 | 1.2E+08 |
| ENSSSCG00000006487 | 4 | 93733917 | 93753340 | Average daily gain QTL (49) | 4 | 80445179 | 1.08E+08 |
| ENSSSCG00000006487 | 4 | 93733917 | 93753340 | Japanese color scale QTL (4288) | 4 | 80844907 | 1.14E+08 |
| ENSSSCG00000006487 | 4 | 93733917 | 93753340 | Average backfat thickness QTL (676) | 4 | 80944839 | 99931900 |
| ENSSSCG00000006487 | 4 | 93733917 | 93753340 | Skin thickness QTL (31810) | 4 | 81744294 | 1.19E+08 |
| ENSSSCG00000006487 | 4 | 93733917 | 93753340 | Meat color L* QTL (4898) | 4 | 82643681 | 1.04E+08 |
| ENSSSCG00000006487 | 4 | 93733917 | 93753340 | Carcass weight (hot) QTL (16864) | 4 | 82943477 | 1.03E+08 |
| ENSSSCG00000006487 | 4 | 93733917 | 93753340 | Adipocyte diameter QTL (18020) | 4 | 85641638 | 1.08E+08 |
| ENSSSCG00000006487 | 4 | 93733917 | 93753340 | Meat color a* QTL (4899) | 4 | 85941434 | 99232376 |
| ENSSSCG00000006487 | 4 | 93733917 | 93753340 | pH 24 hr post mortem (ham) QTL (4897) | 4 | 89039322 | 1.15E+08 |
| ENSSSCG00000006487 | 4 | 93733917 | 93753340 | Palmitoleic acid content QTL (156925) | 4 | 7994552 | 1.2E+08 |
| ENSSSCG00000006487 | 4 | 93733917 | 93753340 | Oleic acid content QTL (156927) | 4 | 7994552 | 1.2E+08 |
| ENSSSCG00000006487 | 4 | 93733917 | 93753340 | Linoleic acid content QTL (156928) | 4 | 7994552 | 1.2E+08 |
| ENSSSCG00000006487 | 4 | 93733917 | 93753340 | Monounsaturated fatty acid content QTL (156929) | 4 | 7994552 | 1.2E+08 |
| ENSSSCG00000006487 | 4 | 93733917 | 93753340 | Drip loss QTL (38084) | 4 | 7994552 | 1.2E+08 |
| ENSSSCG00000006487 | 4 | 93733917 | 93753340 | Average daily gain QTL (245) | 4 | 26981613 | 1.25E+08 |
| ENSSSCG00000006487 | 4 | 93733917 | 93753340 | Backfat at mid-back QTL (5243) | 4 | 26981613 | 1.19E+08 |
| ENSSSCG00000006487 | 4 | 93733917 | 93753340 | Marbling QTL (38082) | 4 | 27081544 | 1.2E+08 |
| ENSSSCG00000006487 | 4 | 93733917 | 93753340 | Average daily gain QTL (370) | 4 | 27081544 | 1.06E+08 |
| ENSSSCG00000006487 | 4 | 93733917 | 93753340 | Body weight (end of test) QTL (5235) | 4 | 33177390 | 1.11E+08 |
| ENSSSCG00000006487 | 4 | 93733917 | 93753340 | Body weight (46 days) QTL (21227) | 4 | 33277322 | 1.21E+08 |
| ENSSSCG00000006487 | 4 | 93733917 | 93753340 | body weight (17 weeks) QTL (21224) | 4 | 43970036 | 1.01E+08 |
| ENSSSCG00000006487 | 4 | 93733917 | 93753340 | Average daily gain QTL (458) | 4 | 47767448 | 1.06E+08 |
| ENSSSCG00000006487 | 4 | 93733917 | 93753340 | Average backfat thickness QTL (371) | 4 | 48966631 | 1.03E+08 |
| ENSSSCG00000006487 | 4 | 93733917 | 93753340 | Body weight (end of test) QTL (5200) | 4 | 51664792 | 1.01E+08 |
| ENSSSCG00000006487 | 4 | 93733917 | 93753340 | Body weight (3 weeks) QTL (175) | 4 | 53963226 | 98932581 |
| ENSSSCG00000006487 | 4 | 93733917 | 93753340 | Intramuscular fat content QTL (3871) | 4 | 55861932 | 1.3E+08 |
| ENSSSCG00000006487 | 4 | 93733917 | 93753340 | Average daily gain QTL (179) | 4 | 56961183 | 97933262 |
| ENSSSCG00000006487 | 4 | 93733917 | 93753340 | Shear force at first peak QTL (3933) | 4 | 62257573 | 1.3E+08 |
| ENSSSCG00000006487 | 4 | 93733917 | 93753340 | Total shear work QTL (3934) | 4 | 62257573 | 1.3E+08 |
| ENSSSCG00000006487 | 4 | 93733917 | 93753340 | Average backfat thickness QTL (162) | 4 | 62257573 | 1.2E+08 |
| ENSSSCG00000006487 | 4 | 93733917 | 93753340 | Average daily gain QTL (17562) | 4 | 62657301 | 97833330 |
| ENSSSCG00000006701 | 4 | 1E+08 | 1E+08 | Average daily gain QTL (456) | 4 | 69552602 | 1.03E+08 |
| ENSSSCG00000006701 | 4 | 1E+08 | 1E+08 | Water holding capacity QTL (3847) | 4 | 71151512 | 1.06E+08 |
| ENSSSCG00000006701 | 4 | 1E+08 | 1E+08 | Stearic acid content QTL (156926) | 4 | 71950968 | 1.2E+08 |
| ENSSSCG00000006701 | 4 | 1E+08 | 1E+08 | Capric acid content QTL (156870) | 4 | 71950968 | 1.2E+08 |
| ENSSSCG00000006701 | 4 | 1E+08 | 1E+08 | Diameter of type IIb muscle fibers QTL (2807) | 4 | 72950287 | 1.01E+08 |
| ENSSSCG00000006701 | 4 | 1E+08 | 1E+08 | Number of muscle fibers per unit area QTL (2808) | 4 | 72950287 | 1.01E+08 |
| ENSSSCG00000006701 | 4 | 1E+08 | 1E+08 | Diameter of muscle fibers QTL (2809) | 4 | 72950287 | 1.01E+08 |
| ENSSSCG00000006701 | 4 | 1E+08 | 1E+08 | Diameter of type IIb muscle fibers QTL (2810) | 4 | 72950287 | 1.01E+08 |
| ENSSSCG00000006701 | 4 | 1E+08 | 1E+08 | subjective pork odor QTL (4207) | 4 | 74349333 | 1.06E+08 |
| ENSSSCG00000006701 | 4 | 1E+08 | 1E+08 | Margaric acid content QTL (158) | 4 | 74349333 | 1.06E+08 |
| ENSSSCG00000006701 | 4 | 1E+08 | 1E+08 | Meat color-a QTL (294) | 4 | 76947563 | 1.12E+08 |
| ENSSSCG00000006701 | 4 | 1E+08 | 1E+08 | Carcass length QTL (460) | 4 | 79245996 | 1.21E+08 |
| ENSSSCG00000006701 | 4 | 1E+08 | 1E+08 | Drip loss QTL (6003) | 4 | 79245996 | 1.2E+08 |
| ENSSSCG00000006701 | 4 | 1E+08 | 1E+08 | Oleic acid content QTL (15875) | 4 | 79245996 | 1.03E+08 |
| ENSSSCG00000006701 | 4 | 1E+08 | 1E+08 | Palmitic acid content QTL (15876) | 4 | 80045451 | 1.14E+08 |
| ENSSSCG00000006701 | 4 | 1E+08 | 1E+08 | Carcass weight (cold) QTL (3804) | 4 | 80445179 | 1.3E+08 |
| ENSSSCG00000006701 | 4 | 1E+08 | 1E+08 | Loin muscle area QTL (51) | 4 | 80445179 | 1.2E+08 |
| ENSSSCG00000006701 | 4 | 1E+08 | 1E+08 | Average daily gain QTL (49) | 4 | 80445179 | 1.08E+08 |
| ENSSSCG00000006701 | 4 | 1E+08 | 1E+08 | Japanese color scale QTL (4288) | 4 | 80844907 | 1.14E+08 |
| ENSSSCG00000006701 | 4 | 1E+08 | 1E+08 | Skin thickness QTL (31810) | 4 | 81744294 | 1.19E+08 |
| ENSSSCG00000006701 | 4 | 1E+08 | 1E+08 | Meat color L* QTL (4898) | 4 | 82643681 | 1.04E+08 |
| ENSSSCG00000006701 | 4 | 1E+08 | 1E+08 | Carcass weight (hot) QTL (16864) | 4 | 82943477 | 1.03E+08 |
| ENSSSCG00000006701 | 4 | 1E+08 | 1E+08 | Adipocyte diameter QTL (18020) | 4 | 85641638 | 1.08E+08 |
| ENSSSCG00000006701 | 4 | 1E+08 | 1E+08 | pH 24 hr post mortem (ham) QTL (4897) | 4 | 89039322 | 1.15E+08 |
| ENSSSCG00000006701 | 4 | 1E+08 | 1E+08 | Triglyceride level QTL (15068) | 4 | 99232376 | 1.2E+08 |
| ENSSSCG00000006701 | 4 | 1E+08 | 1E+08 | Backfat at last rib QTL (52) | 4 | 99232376 | 1.06E+08 |
| ENSSSCG00000006701 | 4 | 1E+08 | 1E+08 | Percentage type IIb fibers QTL (7041) | 4 | 99232376 | 1.06E+08 |
| ENSSSCG00000006701 | 4 | 1E+08 | 1E+08 | Palmitoleic acid content QTL (156925) | 4 | 7994552 | 1.2E+08 |
| ENSSSCG00000006701 | 4 | 1E+08 | 1E+08 | Oleic acid content QTL (156927) | 4 | 7994552 | 1.2E+08 |
| ENSSSCG00000006701 | 4 | 1E+08 | 1E+08 | Linoleic acid content QTL (156928) | 4 | 7994552 | 1.2E+08 |
| ENSSSCG00000006701 | 4 | 1E+08 | 1E+08 | Monounsaturated fatty acid content QTL (156929) | 4 | 7994552 | 1.2E+08 |
| ENSSSCG00000006701 | 4 | 1E+08 | 1E+08 | Drip loss QTL (38084) | 4 | 7994552 | 1.2E+08 |
| ENSSSCG00000006701 | 4 | 1E+08 | 1E+08 | Average daily gain QTL (245) | 4 | 26981613 | 1.25E+08 |
| ENSSSCG00000006701 | 4 | 1E+08 | 1E+08 | Backfat at mid-back QTL (5243) | 4 | 26981613 | 1.19E+08 |
| ENSSSCG00000006701 | 4 | 1E+08 | 1E+08 | Marbling QTL (38082) | 4 | 27081544 | 1.2E+08 |
| ENSSSCG00000006701 | 4 | 1E+08 | 1E+08 | Average daily gain QTL (370) | 4 | 27081544 | 1.06E+08 |
| ENSSSCG00000006701 | 4 | 1E+08 | 1E+08 | Body weight (end of test) QTL (5235) | 4 | 33177390 | 1.11E+08 |
| ENSSSCG00000006701 | 4 | 1E+08 | 1E+08 | Body weight (46 days) QTL (21227) | 4 | 33277322 | 1.21E+08 |
| ENSSSCG00000006701 | 4 | 1E+08 | 1E+08 | body weight (17 weeks) QTL (21224) | 4 | 43970036 | 1.01E+08 |
| ENSSSCG00000006701 | 4 | 1E+08 | 1E+08 | Average daily gain QTL (458) | 4 | 47767448 | 1.06E+08 |
| ENSSSCG00000006701 | 4 | 1E+08 | 1E+08 | Average backfat thickness QTL (371) | 4 | 48966631 | 1.03E+08 |
| ENSSSCG00000006701 | 4 | 1E+08 | 1E+08 | Body weight (end of test) QTL (5200) | 4 | 51664792 | 1.01E+08 |
| ENSSSCG00000006701 | 4 | 1E+08 | 1E+08 | Intramuscular fat content QTL (3871) | 4 | 55861932 | 1.3E+08 |
| ENSSSCG00000006701 | 4 | 1E+08 | 1E+08 | Shear force at first peak QTL (3933) | 4 | 62257573 | 1.3E+08 |
| ENSSSCG00000006701 | 4 | 1E+08 | 1E+08 | Total shear work QTL (3934) | 4 | 62257573 | 1.3E+08 |
| ENSSSCG00000006701 | 4 | 1E+08 | 1E+08 | Average backfat thickness QTL (162) | 4 | 62257573 | 1.2E+08 |
| ENSSSCG00000006867 | 4 | 1.18E+08 | 1.18E+08 | pH 24 hr post-mortem (loin) QTL (4287) | 4 | 1.18E+08 | 1.18E+08 |
| ENSSSCG00000006867 | 4 | 1.18E+08 | 1.18E+08 | Average daily ham weight gain QTL (4299) | 4 | 1.18E+08 | 1.18E+08 |
| ENSSSCG00000006867 | 4 | 1.18E+08 | 1.18E+08 | Stearic acid content QTL (156926) | 4 | 71950968 | 1.2E+08 |
| ENSSSCG00000006867 | 4 | 1.18E+08 | 1.18E+08 | Capric acid content QTL (156870) | 4 | 71950968 | 1.2E+08 |
| ENSSSCG00000006867 | 4 | 1.18E+08 | 1.18E+08 | Carcass length QTL (460) | 4 | 79245996 | 1.21E+08 |
| ENSSSCG00000006867 | 4 | 1.18E+08 | 1.18E+08 | Drip loss QTL (6003) | 4 | 79245996 | 1.2E+08 |
| ENSSSCG00000006867 | 4 | 1.18E+08 | 1.18E+08 | Carcass weight (cold) QTL (3804) | 4 | 80445179 | 1.3E+08 |
| ENSSSCG00000006867 | 4 | 1.18E+08 | 1.18E+08 | Loin muscle area QTL (51) | 4 | 80445179 | 1.2E+08 |
| ENSSSCG00000006867 | 4 | 1.18E+08 | 1.18E+08 | Skin thickness QTL (31810) | 4 | 81744294 | 1.19E+08 |
| ENSSSCG00000006867 | 4 | 1.18E+08 | 1.18E+08 | Triglyceride level QTL (15068) | 4 | 99232376 | 1.2E+08 |
| ENSSSCG00000006867 | 4 | 1.18E+08 | 1.18E+08 | Calcium level QTL (6378) | 4 | 1.03E+08 | 1.3E+08 |
| ENSSSCG00000006867 | 4 | 1.18E+08 | 1.18E+08 | Meat color-L QTL (297) | 4 | 1.03E+08 | 1.2E+08 |
| ENSSSCG00000006867 | 4 | 1.18E+08 | 1.18E+08 | Body weight (3 weeks) QTL (977) | 4 | 1.03E+08 | 1.2E+08 |
| ENSSSCG00000006867 | 4 | 1.18E+08 | 1.18E+08 | Intramuscular fat content QTL (3841) | 4 | 1.06E+08 | 1.21E+08 |
| ENSSSCG00000006867 | 4 | 1.18E+08 | 1.18E+08 | Intramuscular fat content QTL (3842) | 4 | 1.06E+08 | 1.21E+08 |
| ENSSSCG00000006867 | 4 | 1.18E+08 | 1.18E+08 | androstenone, laboratory QTL (4206) | 4 | 1.06E+08 | 1.2E+08 |
| ENSSSCG00000006867 | 4 | 1.18E+08 | 1.18E+08 | Japanese color scale QTL (4321) | 4 | 1.06E+08 | 1.2E+08 |
| ENSSSCG00000006867 | 4 | 1.18E+08 | 1.18E+08 | Meat color L* QTL (4322) | 4 | 1.06E+08 | 1.2E+08 |
| ENSSSCG00000006867 | 4 | 1.18E+08 | 1.18E+08 | pH 24 hr post-mortem (loin) QTL (4323) | 4 | 1.06E+08 | 1.2E+08 |
| ENSSSCG00000006867 | 4 | 1.18E+08 | 1.18E+08 | Carcass weight (cold) QTL (50) | 4 | 1.08E+08 | 1.3E+08 |
| ENSSSCG00000006867 | 4 | 1.18E+08 | 1.18E+08 | Carcass weight (cold) QTL (2939) | 4 | 1.08E+08 | 1.21E+08 |
| ENSSSCG00000006867 | 4 | 1.18E+08 | 1.18E+08 | Meat color a* QTL (4036) | 4 | 1.14E+08 | 1.2E+08 |
| ENSSSCG00000006867 | 4 | 1.18E+08 | 1.18E+08 | Meat color a* QTL (4286) | 4 | 1.17E+08 | 1.2E+08 |
| ENSSSCG00000006867 | 4 | 1.18E+08 | 1.18E+08 | Palmitoleic acid content QTL (156925) | 4 | 7994552 | 1.2E+08 |
| ENSSSCG00000006867 | 4 | 1.18E+08 | 1.18E+08 | Oleic acid content QTL (156927) | 4 | 7994552 | 1.2E+08 |
| ENSSSCG00000006867 | 4 | 1.18E+08 | 1.18E+08 | Linoleic acid content QTL (156928) | 4 | 7994552 | 1.2E+08 |
| ENSSSCG00000006867 | 4 | 1.18E+08 | 1.18E+08 | Monounsaturated fatty acid content QTL (156929) | 4 | 7994552 | 1.2E+08 |
| ENSSSCG00000006867 | 4 | 1.18E+08 | 1.18E+08 | Drip loss QTL (38084) | 4 | 7994552 | 1.2E+08 |
| ENSSSCG00000006867 | 4 | 1.18E+08 | 1.18E+08 | Average daily gain QTL (245) | 4 | 26981613 | 1.25E+08 |
| ENSSSCG00000006867 | 4 | 1.18E+08 | 1.18E+08 | Backfat at mid-back QTL (5243) | 4 | 26981613 | 1.19E+08 |
| ENSSSCG00000006867 | 4 | 1.18E+08 | 1.18E+08 | Marbling QTL (38082) | 4 | 27081544 | 1.2E+08 |
| ENSSSCG00000006867 | 4 | 1.18E+08 | 1.18E+08 | Body weight (46 days) QTL (21227) | 4 | 33277322 | 1.21E+08 |
| ENSSSCG00000006867 | 4 | 1.18E+08 | 1.18E+08 | Intramuscular fat content QTL (3871) | 4 | 55861932 | 1.3E+08 |
| ENSSSCG00000006867 | 4 | 1.18E+08 | 1.18E+08 | Shear force at first peak QTL (3933) | 4 | 62257573 | 1.3E+08 |
| ENSSSCG00000006867 | 4 | 1.18E+08 | 1.18E+08 | Total shear work QTL (3934) | 4 | 62257573 | 1.3E+08 |
| ENSSSCG00000006867 | 4 | 1.18E+08 | 1.18E+08 | Average backfat thickness QTL (162) | 4 | 62257573 | 1.2E+08 |
| ENSSSCG00000006872 | 4 | 1.18E+08 | 1.18E+08 | Backfat thickness between 3rd and 4th rib QTL (23175) | 4 | 1.18E+08 | 1.18E+08 |
| ENSSSCG00000006872 | 4 | 1.18E+08 | 1.18E+08 | Backfat thickness between 3rd and 4th rib QTL (23176) | 4 | 1.18E+08 | 1.18E+08 |
| ENSSSCG00000006872 | 4 | 1.18E+08 | 1.18E+08 | Stearic acid content QTL (156926) | 4 | 71950968 | 1.2E+08 |
| ENSSSCG00000006872 | 4 | 1.18E+08 | 1.18E+08 | Capric acid content QTL (156870) | 4 | 71950968 | 1.2E+08 |
| ENSSSCG00000006872 | 4 | 1.18E+08 | 1.18E+08 | Carcass length QTL (460) | 4 | 79245996 | 1.21E+08 |
| ENSSSCG00000006872 | 4 | 1.18E+08 | 1.18E+08 | Drip loss QTL (6003) | 4 | 79245996 | 1.2E+08 |
| ENSSSCG00000006872 | 4 | 1.18E+08 | 1.18E+08 | Carcass weight (cold) QTL (3804) | 4 | 80445179 | 1.3E+08 |
| ENSSSCG00000006872 | 4 | 1.18E+08 | 1.18E+08 | Loin muscle area QTL (51) | 4 | 80445179 | 1.2E+08 |
| ENSSSCG00000006872 | 4 | 1.18E+08 | 1.18E+08 | Skin thickness QTL (31810) | 4 | 81744294 | 1.19E+08 |
| ENSSSCG00000006872 | 4 | 1.18E+08 | 1.18E+08 | Triglyceride level QTL (15068) | 4 | 99232376 | 1.2E+08 |
| ENSSSCG00000006872 | 4 | 1.18E+08 | 1.18E+08 | Calcium level QTL (6378) | 4 | 1.03E+08 | 1.3E+08 |
| ENSSSCG00000006872 | 4 | 1.18E+08 | 1.18E+08 | Meat color-L QTL (297) | 4 | 1.03E+08 | 1.2E+08 |
| ENSSSCG00000006872 | 4 | 1.18E+08 | 1.18E+08 | Body weight (3 weeks) QTL (977) | 4 | 1.03E+08 | 1.2E+08 |
| ENSSSCG00000006872 | 4 | 1.18E+08 | 1.18E+08 | Intramuscular fat content QTL (3841) | 4 | 1.06E+08 | 1.21E+08 |
| ENSSSCG00000006872 | 4 | 1.18E+08 | 1.18E+08 | Intramuscular fat content QTL (3842) | 4 | 1.06E+08 | 1.21E+08 |
| ENSSSCG00000006872 | 4 | 1.18E+08 | 1.18E+08 | androstenone, laboratory QTL (4206) | 4 | 1.06E+08 | 1.2E+08 |
| ENSSSCG00000006872 | 4 | 1.18E+08 | 1.18E+08 | Japanese color scale QTL (4321) | 4 | 1.06E+08 | 1.2E+08 |
| ENSSSCG00000006872 | 4 | 1.18E+08 | 1.18E+08 | Meat color L* QTL (4322) | 4 | 1.06E+08 | 1.2E+08 |
| ENSSSCG00000006872 | 4 | 1.18E+08 | 1.18E+08 | pH 24 hr post-mortem (loin) QTL (4323) | 4 | 1.06E+08 | 1.2E+08 |
| ENSSSCG00000006872 | 4 | 1.18E+08 | 1.18E+08 | Carcass weight (cold) QTL (50) | 4 | 1.08E+08 | 1.3E+08 |
| ENSSSCG00000006872 | 4 | 1.18E+08 | 1.18E+08 | Carcass weight (cold) QTL (2939) | 4 | 1.08E+08 | 1.21E+08 |
| ENSSSCG00000006872 | 4 | 1.18E+08 | 1.18E+08 | Meat color a* QTL (4036) | 4 | 1.14E+08 | 1.2E+08 |
| ENSSSCG00000006872 | 4 | 1.18E+08 | 1.18E+08 | Meat color a* QTL (4286) | 4 | 1.17E+08 | 1.2E+08 |
| ENSSSCG00000006872 | 4 | 1.18E+08 | 1.18E+08 | Palmitoleic acid content QTL (156925) | 4 | 7994552 | 1.2E+08 |
| ENSSSCG00000006872 | 4 | 1.18E+08 | 1.18E+08 | Oleic acid content QTL (156927) | 4 | 7994552 | 1.2E+08 |
| ENSSSCG00000006872 | 4 | 1.18E+08 | 1.18E+08 | Linoleic acid content QTL (156928) | 4 | 7994552 | 1.2E+08 |
| ENSSSCG00000006872 | 4 | 1.18E+08 | 1.18E+08 | Monounsaturated fatty acid content QTL (156929) | 4 | 7994552 | 1.2E+08 |
| ENSSSCG00000006872 | 4 | 1.18E+08 | 1.18E+08 | Drip loss QTL (38084) | 4 | 7994552 | 1.2E+08 |
| ENSSSCG00000006872 | 4 | 1.18E+08 | 1.18E+08 | Average daily gain QTL (245) | 4 | 26981613 | 1.25E+08 |
| ENSSSCG00000006872 | 4 | 1.18E+08 | 1.18E+08 | Backfat at mid-back QTL (5243) | 4 | 26981613 | 1.19E+08 |
| ENSSSCG00000006872 | 4 | 1.18E+08 | 1.18E+08 | Marbling QTL (38082) | 4 | 27081544 | 1.2E+08 |
| ENSSSCG00000006872 | 4 | 1.18E+08 | 1.18E+08 | Body weight (46 days) QTL (21227) | 4 | 33277322 | 1.21E+08 |
| ENSSSCG00000006872 | 4 | 1.18E+08 | 1.18E+08 | Intramuscular fat content QTL (3871) | 4 | 55861932 | 1.3E+08 |
| ENSSSCG00000006872 | 4 | 1.18E+08 | 1.18E+08 | Shear force at first peak QTL (3933) | 4 | 62257573 | 1.3E+08 |
| ENSSSCG00000006872 | 4 | 1.18E+08 | 1.18E+08 | Total shear work QTL (3934) | 4 | 62257573 | 1.3E+08 |
| ENSSSCG00000006872 | 4 | 1.18E+08 | 1.18E+08 | Average backfat thickness QTL (162) | 4 | 62257573 | 1.2E+08 |
| ENSSSCG00000006911 | 4 | 1.25E+08 | 1.25E+08 | Carcass weight (cold) QTL (3804) | 4 | 80445179 | 1.3E+08 |
| ENSSSCG00000006911 | 4 | 1.25E+08 | 1.25E+08 | Calcium level QTL (6378) | 4 | 1.03E+08 | 1.3E+08 |
| ENSSSCG00000006911 | 4 | 1.25E+08 | 1.25E+08 | Carcass weight (cold) QTL (50) | 4 | 1.08E+08 | 1.3E+08 |
| ENSSSCG00000006911 | 4 | 1.25E+08 | 1.25E+08 | Intramuscular fat content QTL (3871) | 4 | 55861932 | 1.3E+08 |
| ENSSSCG00000006911 | 4 | 1.25E+08 | 1.25E+08 | Shear force at first peak QTL (3933) | 4 | 62257573 | 1.3E+08 |
| ENSSSCG00000006911 | 4 | 1.25E+08 | 1.25E+08 | Total shear work QTL (3934) | 4 | 62257573 | 1.3E+08 |
| ENSSSCG00000000078 | 5 | 8060979 | 8311418 | Average daily gain QTL (28828) | 5 | 8186692 | 8186696 |
| ENSSSCG00000000078 | 5 | 8060979 | 8311418 | Drip loss QTL (3805) | 5 | 6017944 | 56382411 |
| ENSSSCG00000000078 | 5 | 8060979 | 8311418 | Backfat at tenth rib QTL (981) | 5 | 6017944 | 41480833 |
| ENSSSCG00000000078 | 5 | 8060979 | 8311418 | Activity during lying QTL (5903) | 5 | 6017944 | 28585237 |
| ENSSSCG00000000078 | 5 | 8060979 | 8311418 | Creatinine level QTL (6372) | 5 | 6017944 | 28585237 |
| ENSSSCG00000000078 | 5 | 8060979 | 8311418 | Shoulder subcutaneous fat thickness QTL (5219) | 5 | 6017944 | 1.04E+08 |
| ENSSSCG00000000078 | 5 | 8060979 | 8311418 | backfat above muscle dorsi QTL (5203) | 5 | 6017944 | 96502043 |
| ENSSSCG00000000078 | 5 | 8060979 | 8311418 | backfat above muscle dorsi QTL (5238) | 5 | 6017944 | 90699025 |
| ENSSSCG00000000078 | 5 | 8060979 | 8311418 | Meat color L* QTL (38088) | 5 | 6017944 | 89552750 |
| ENSSSCG00000000078 | 5 | 8060979 | 8311418 | Loin muscle area QTL (38085) | 5 | 6017944 | 84036300 |
| ENSSSCG00000026004 | 5 | 29695839 | 29863599 | Drip loss QTL (3805) | 5 | 6017944 | 56382411 |
| ENSSSCG00000026004 | 5 | 29695839 | 29863599 | Backfat at tenth rib QTL (981) | 5 | 6017944 | 41480833 |
| ENSSSCG00000026004 | 5 | 29695839 | 29863599 | Backfat at last lumbar QTL (31) | 5 | 22567293 | 56382411 |
| ENSSSCG00000026004 | 5 | 29695839 | 29863599 | Backfat thickness between 3rd and 4th rib QTL (412) | 5 | 22925504 | 57313760 |
| ENSSSCG00000026004 | 5 | 29695839 | 29863599 | Meat color-b QTL (12083) | 5 | 28585237 | 63188420 |
| ENSSSCG00000026004 | 5 | 29695839 | 29863599 | Meat color-L QTL (12084) | 5 | 28585237 | 63188420 |
| ENSSSCG00000026004 | 5 | 29695839 | 29863599 | pH 24 hr post-mortem (loin) QTL (12085) | 5 | 28585237 | 63188420 |
| ENSSSCG00000026004 | 5 | 29695839 | 29863599 | Shear force QTL (12086) | 5 | 28585237 | 63188420 |
| ENSSSCG00000026004 | 5 | 29695839 | 29863599 | Myofibril fragmentation index QTL (2983) | 5 | 28585237 | 51797310 |
| ENSSSCG00000026004 | 5 | 29695839 | 29863599 | Shoulder subcutaneous fat thickness QTL (5219) | 5 | 6017944 | 1.04E+08 |
| ENSSSCG00000026004 | 5 | 29695839 | 29863599 | backfat above muscle dorsi QTL (5203) | 5 | 6017944 | 96502043 |
| ENSSSCG00000026004 | 5 | 29695839 | 29863599 | backfat above muscle dorsi QTL (5238) | 5 | 6017944 | 90699025 |
| ENSSSCG00000026004 | 5 | 29695839 | 29863599 | Meat color L* QTL (38088) | 5 | 6017944 | 89552750 |
| ENSSSCG00000026004 | 5 | 29695839 | 29863599 | Loin muscle area QTL (38085) | 5 | 6017944 | 84036300 |
| ENSSSCG00000026004 | 5 | 29695839 | 29863599 | Backfat at mid-back QTL (5210) | 5 | 11104541 | 1.04E+08 |
| ENSSSCG00000026004 | 5 | 29695839 | 29863599 | Backfat at mid-back QTL (5209) | 5 | 20059816 | 78663135 |
| ENSSSCG00000026004 | 5 | 29695839 | 29863599 | Backfat at first rib QTL (21236) | 5 | 23355357 | 86901988 |
| ENSSSCG00000026004 | 5 | 29695839 | 29863599 | Marbling QTL (38086) | 5 | 28585237 | 1.05E+08 |
| ENSSSCG00000026004 | 5 | 29695839 | 29863599 | Meat color b* QTL (38087) | 5 | 28585237 | 89552750 |
| ENSSSCG00000026004 | 5 | 29695839 | 29863599 | body weight (10 weeks) QTL (29692) | 5 | 28585237 | 68418301 |
| ENSSSCG00000026004 | 5 | 29695839 | 29863599 | Body weight (20 weeks) QTL (29693) | 5 | 28585237 | 68418301 |
| ENSSSCG00000000492 | 5 | 33525744 | 33618242 | Drip loss QTL (3805) | 5 | 6017944 | 56382411 |
| ENSSSCG00000000492 | 5 | 33525744 | 33618242 | Backfat at tenth rib QTL (981) | 5 | 6017944 | 41480833 |
| ENSSSCG00000000492 | 5 | 33525744 | 33618242 | Backfat at last lumbar QTL (31) | 5 | 22567293 | 56382411 |
| ENSSSCG00000000492 | 5 | 33525744 | 33618242 | Backfat thickness between 3rd and 4th rib QTL (412) | 5 | 22925504 | 57313760 |
| ENSSSCG00000000492 | 5 | 33525744 | 33618242 | Meat color-b QTL (12083) | 5 | 28585237 | 63188420 |
| ENSSSCG00000000492 | 5 | 33525744 | 33618242 | Meat color-L QTL (12084) | 5 | 28585237 | 63188420 |
| ENSSSCG00000000492 | 5 | 33525744 | 33618242 | pH 24 hr post-mortem (loin) QTL (12085) | 5 | 28585237 | 63188420 |
| ENSSSCG00000000492 | 5 | 33525744 | 33618242 | Shear force QTL (12086) | 5 | 28585237 | 63188420 |
| ENSSSCG00000000492 | 5 | 33525744 | 33618242 | Myofibril fragmentation index QTL (2983) | 5 | 28585237 | 51797310 |
| ENSSSCG00000000492 | 5 | 33525744 | 33618242 | Shoulder subcutaneous fat thickness QTL (5219) | 5 | 6017944 | 1.04E+08 |
| ENSSSCG00000000492 | 5 | 33525744 | 33618242 | backfat above muscle dorsi QTL (5203) | 5 | 6017944 | 96502043 |
| ENSSSCG00000000492 | 5 | 33525744 | 33618242 | backfat above muscle dorsi QTL (5238) | 5 | 6017944 | 90699025 |
| ENSSSCG00000000492 | 5 | 33525744 | 33618242 | Meat color L* QTL (38088) | 5 | 6017944 | 89552750 |
| ENSSSCG00000000492 | 5 | 33525744 | 33618242 | Loin muscle area QTL (38085) | 5 | 6017944 | 84036300 |
| ENSSSCG00000000492 | 5 | 33525744 | 33618242 | Backfat at mid-back QTL (5210) | 5 | 11104541 | 1.04E+08 |
| ENSSSCG00000000492 | 5 | 33525744 | 33618242 | Backfat at mid-back QTL (5209) | 5 | 20059816 | 78663135 |
| ENSSSCG00000000492 | 5 | 33525744 | 33618242 | Backfat at first rib QTL (21236) | 5 | 23355357 | 86901988 |
| ENSSSCG00000000492 | 5 | 33525744 | 33618242 | Marbling QTL (38086) | 5 | 28585237 | 1.05E+08 |
| ENSSSCG00000000492 | 5 | 33525744 | 33618242 | Meat color b* QTL (38087) | 5 | 28585237 | 89552750 |
| ENSSSCG00000000492 | 5 | 33525744 | 33618242 | body weight (10 weeks) QTL (29692) | 5 | 28585237 | 68418301 |
| ENSSSCG00000000492 | 5 | 33525744 | 33618242 | Body weight (20 weeks) QTL (29693) | 5 | 28585237 | 68418301 |
| ENSSSCG00000000492 | 5 | 33525744 | 33618242 | Backfat at rump QTL (21237) | 5 | 30877788 | 86543777 |
| ENSSSCG00000000492 | 5 | 33525744 | 33618242 | Shoulder subcutaneous fat thickness QTL (21239) | 5 | 32238990 | 97863245 |
| ENSSSCG00000000530 | 5 | 41570282 | 41879062 | Drip loss QTL (3805) | 5 | 6017944 | 56382411 |
| ENSSSCG00000000530 | 5 | 41570282 | 41879062 | Backfat at last lumbar QTL (31) | 5 | 22567293 | 56382411 |
| ENSSSCG00000000530 | 5 | 41570282 | 41879062 | Backfat thickness between 3rd and 4th rib QTL (412) | 5 | 22925504 | 57313760 |
| ENSSSCG00000000530 | 5 | 41570282 | 41879062 | Meat color-b QTL (12083) | 5 | 28585237 | 63188420 |
| ENSSSCG00000000530 | 5 | 41570282 | 41879062 | Meat color-L QTL (12084) | 5 | 28585237 | 63188420 |
| ENSSSCG00000000530 | 5 | 41570282 | 41879062 | pH 24 hr post-mortem (loin) QTL (12085) | 5 | 28585237 | 63188420 |
| ENSSSCG00000000530 | 5 | 41570282 | 41879062 | Shear force QTL (12086) | 5 | 28585237 | 63188420 |
| ENSSSCG00000000530 | 5 | 41570282 | 41879062 | Myofibril fragmentation index QTL (2983) | 5 | 28585237 | 51797310 |
| ENSSSCG00000000530 | 5 | 41570282 | 41879062 | Ear size QTL (5641) | 5 | 41480833 | 56883906 |
| ENSSSCG00000000530 | 5 | 41570282 | 41879062 | Conductivity 24 hours postmortem (loin) QTL (21857) | 5 | 41480833 | 56382411 |
| ENSSSCG00000000530 | 5 | 41570282 | 41879062 | Backfat at first rib QTL (21363) | 5 | 41480833 | 56382411 |
| ENSSSCG00000000530 | 5 | 41570282 | 41879062 | Average daily gain QTL (323) | 5 | 41480833 | 52728659 |
| ENSSSCG00000000530 | 5 | 41570282 | 41879062 | Toll-like receptor 9 level QTL (17619) | 5 | 41480833 | 51797310 |
| ENSSSCG00000000530 | 5 | 41570282 | 41879062 | Interferon-gamma level QTL (12337) | 5 | 41480833 | 51797310 |
| ENSSSCG00000000530 | 5 | 41570282 | 41879062 | Change in interferon-gamma level QTL (12338) | 5 | 41480833 | 51797310 |
| ENSSSCG00000000530 | 5 | 41570282 | 41879062 | Shoulder subcutaneous fat thickness QTL (5219) | 5 | 6017944 | 1.04E+08 |
| ENSSSCG00000000530 | 5 | 41570282 | 41879062 | backfat above muscle dorsi QTL (5203) | 5 | 6017944 | 96502043 |
| ENSSSCG00000000530 | 5 | 41570282 | 41879062 | backfat above muscle dorsi QTL (5238) | 5 | 6017944 | 90699025 |
| ENSSSCG00000000530 | 5 | 41570282 | 41879062 | Meat color L* QTL (38088) | 5 | 6017944 | 89552750 |
| ENSSSCG00000000530 | 5 | 41570282 | 41879062 | Loin muscle area QTL (38085) | 5 | 6017944 | 84036300 |
| ENSSSCG00000000530 | 5 | 41570282 | 41879062 | Backfat at mid-back QTL (5210) | 5 | 11104541 | 1.04E+08 |
| ENSSSCG00000000530 | 5 | 41570282 | 41879062 | Backfat at mid-back QTL (5209) | 5 | 20059816 | 78663135 |
| ENSSSCG00000000530 | 5 | 41570282 | 41879062 | Backfat at first rib QTL (21236) | 5 | 23355357 | 86901988 |
| ENSSSCG00000000530 | 5 | 41570282 | 41879062 | Marbling QTL (38086) | 5 | 28585237 | 1.05E+08 |
| ENSSSCG00000000530 | 5 | 41570282 | 41879062 | Meat color b* QTL (38087) | 5 | 28585237 | 89552750 |
| ENSSSCG00000000530 | 5 | 41570282 | 41879062 | body weight (10 weeks) QTL (29692) | 5 | 28585237 | 68418301 |
| ENSSSCG00000000530 | 5 | 41570282 | 41879062 | Body weight (20 weeks) QTL (29693) | 5 | 28585237 | 68418301 |
| ENSSSCG00000000530 | 5 | 41570282 | 41879062 | Backfat at rump QTL (21237) | 5 | 30877788 | 86543777 |
| ENSSSCG00000000530 | 5 | 41570282 | 41879062 | Shoulder subcutaneous fat thickness QTL (21239) | 5 | 32238990 | 97863245 |
| ENSSSCG00000000530 | 5 | 41570282 | 41879062 | Meat color b* QTL (3918) | 5 | 35813935 | 79594484 |
| ENSSSCG00000000530 | 5 | 41570282 | 41879062 | Meat color b* QTL (3919) | 5 | 35813935 | 79594484 |
| ENSSSCG00000000530 | 5 | 41570282 | 41879062 | Meat color b* QTL (3920) | 5 | 35813935 | 79594484 |
| ENSSSCG00000000571 | 5 | 51536526 | 51673583 | Drip loss QTL (3805) | 5 | 6017944 | 56382411 |
| ENSSSCG00000000571 | 5 | 51536526 | 51673583 | Backfat at last lumbar QTL (31) | 5 | 22567293 | 56382411 |
| ENSSSCG00000000571 | 5 | 51536526 | 51673583 | Backfat thickness between 3rd and 4th rib QTL (412) | 5 | 22925504 | 57313760 |
| ENSSSCG00000000571 | 5 | 51536526 | 51673583 | Meat color-b QTL (12083) | 5 | 28585237 | 63188420 |
| ENSSSCG00000000571 | 5 | 51536526 | 51673583 | Meat color-L QTL (12084) | 5 | 28585237 | 63188420 |
| ENSSSCG00000000571 | 5 | 51536526 | 51673583 | pH 24 hr post-mortem (loin) QTL (12085) | 5 | 28585237 | 63188420 |
| ENSSSCG00000000571 | 5 | 51536526 | 51673583 | Shear force QTL (12086) | 5 | 28585237 | 63188420 |
| ENSSSCG00000000571 | 5 | 51536526 | 51673583 | Myofibril fragmentation index QTL (2983) | 5 | 28585237 | 51797310 |
| ENSSSCG00000000571 | 5 | 51536526 | 51673583 | Ear size QTL (5641) | 5 | 41480833 | 56883906 |
| ENSSSCG00000000571 | 5 | 51536526 | 51673583 | Conductivity 24 hours postmortem (loin) QTL (21857) | 5 | 41480833 | 56382411 |
| ENSSSCG00000000571 | 5 | 51536526 | 51673583 | Backfat at first rib QTL (21363) | 5 | 41480833 | 56382411 |
| ENSSSCG00000000571 | 5 | 51536526 | 51673583 | Average daily gain QTL (323) | 5 | 41480833 | 52728659 |
| ENSSSCG00000000571 | 5 | 51536526 | 51673583 | Toll-like receptor 9 level QTL (17619) | 5 | 41480833 | 51797310 |
| ENSSSCG00000000571 | 5 | 51536526 | 51673583 | Interferon-gamma level QTL (12337) | 5 | 41480833 | 51797310 |
| ENSSSCG00000000571 | 5 | 51536526 | 51673583 | Change in interferon-gamma level QTL (12338) | 5 | 41480833 | 51797310 |
| ENSSSCG00000000571 | 5 | 51536526 | 51673583 | Average backfat thickness QTL (21234) | 5 | 47212209 | 63833200 |
| ENSSSCG00000000571 | 5 | 51536526 | 51673583 | Backfat at last rib QTL (21238) | 5 | 48859980 | 62185429 |
| ENSSSCG00000000571 | 5 | 51536526 | 51673583 | Shoulder subcutaneous fat thickness QTL (5219) | 5 | 6017944 | 1.04E+08 |
| ENSSSCG00000000571 | 5 | 51536526 | 51673583 | backfat above muscle dorsi QTL (5203) | 5 | 6017944 | 96502043 |
| ENSSSCG00000000571 | 5 | 51536526 | 51673583 | backfat above muscle dorsi QTL (5238) | 5 | 6017944 | 90699025 |
| ENSSSCG00000000571 | 5 | 51536526 | 51673583 | Meat color L* QTL (38088) | 5 | 6017944 | 89552750 |
| ENSSSCG00000000571 | 5 | 51536526 | 51673583 | Loin muscle area QTL (38085) | 5 | 6017944 | 84036300 |
| ENSSSCG00000000571 | 5 | 51536526 | 51673583 | Backfat at mid-back QTL (5210) | 5 | 11104541 | 1.04E+08 |
| ENSSSCG00000000571 | 5 | 51536526 | 51673583 | Backfat at mid-back QTL (5209) | 5 | 20059816 | 78663135 |
| ENSSSCG00000000571 | 5 | 51536526 | 51673583 | Backfat at first rib QTL (21236) | 5 | 23355357 | 86901988 |
| ENSSSCG00000000571 | 5 | 51536526 | 51673583 | Marbling QTL (38086) | 5 | 28585237 | 1.05E+08 |
| ENSSSCG00000000571 | 5 | 51536526 | 51673583 | Meat color b* QTL (38087) | 5 | 28585237 | 89552750 |
| ENSSSCG00000000571 | 5 | 51536526 | 51673583 | body weight (10 weeks) QTL (29692) | 5 | 28585237 | 68418301 |
| ENSSSCG00000000571 | 5 | 51536526 | 51673583 | Body weight (20 weeks) QTL (29693) | 5 | 28585237 | 68418301 |
| ENSSSCG00000000571 | 5 | 51536526 | 51673583 | Backfat at rump QTL (21237) | 5 | 30877788 | 86543777 |
| ENSSSCG00000000571 | 5 | 51536526 | 51673583 | Shoulder subcutaneous fat thickness QTL (21239) | 5 | 32238990 | 97863245 |
| ENSSSCG00000000571 | 5 | 51536526 | 51673583 | Meat color b* QTL (3918) | 5 | 35813935 | 79594484 |
| ENSSSCG00000000571 | 5 | 51536526 | 51673583 | Meat color b* QTL (3919) | 5 | 35813935 | 79594484 |
| ENSSSCG00000000571 | 5 | 51536526 | 51673583 | Meat color b* QTL (3920) | 5 | 35813935 | 79594484 |
| ENSSSCG00000000571 | 5 | 51536526 | 51673583 | Backfat at mid-back QTL (5244) | 5 | 44633090 | 87546768 |
| ENSSSCG00000029464 | 5 | 67629380 | 67716651 | Shoulder subcutaneous fat thickness QTL (5219) | 5 | 6017944 | 1.04E+08 |
| ENSSSCG00000029464 | 5 | 67629380 | 67716651 | backfat above muscle dorsi QTL (5203) | 5 | 6017944 | 96502043 |
| ENSSSCG00000029464 | 5 | 67629380 | 67716651 | backfat above muscle dorsi QTL (5238) | 5 | 6017944 | 90699025 |
| ENSSSCG00000029464 | 5 | 67629380 | 67716651 | Meat color L* QTL (38088) | 5 | 6017944 | 89552750 |
| ENSSSCG00000029464 | 5 | 67629380 | 67716651 | Loin muscle area QTL (38085) | 5 | 6017944 | 84036300 |
| ENSSSCG00000029464 | 5 | 67629380 | 67716651 | Backfat at mid-back QTL (5210) | 5 | 11104541 | 1.04E+08 |
| ENSSSCG00000029464 | 5 | 67629380 | 67716651 | Backfat at mid-back QTL (5209) | 5 | 20059816 | 78663135 |
| ENSSSCG00000029464 | 5 | 67629380 | 67716651 | Backfat at first rib QTL (21236) | 5 | 23355357 | 86901988 |
| ENSSSCG00000029464 | 5 | 67629380 | 67716651 | Marbling QTL (38086) | 5 | 28585237 | 1.05E+08 |
| ENSSSCG00000029464 | 5 | 67629380 | 67716651 | Meat color b* QTL (38087) | 5 | 28585237 | 89552750 |
| ENSSSCG00000029464 | 5 | 67629380 | 67716651 | body weight (10 weeks) QTL (29692) | 5 | 28585237 | 68418301 |
| ENSSSCG00000029464 | 5 | 67629380 | 67716651 | Body weight (20 weeks) QTL (29693) | 5 | 28585237 | 68418301 |
| ENSSSCG00000029464 | 5 | 67629380 | 67716651 | Backfat at rump QTL (21237) | 5 | 30877788 | 86543777 |
| ENSSSCG00000029464 | 5 | 67629380 | 67716651 | Shoulder subcutaneous fat thickness QTL (21239) | 5 | 32238990 | 97863245 |
| ENSSSCG00000029464 | 5 | 67629380 | 67716651 | Meat color b* QTL (3918) | 5 | 35813935 | 79594484 |
| ENSSSCG00000029464 | 5 | 67629380 | 67716651 | Meat color b* QTL (3919) | 5 | 35813935 | 79594484 |
| ENSSSCG00000029464 | 5 | 67629380 | 67716651 | Meat color b* QTL (3920) | 5 | 35813935 | 79594484 |
| ENSSSCG00000029464 | 5 | 67629380 | 67716651 | Backfat at mid-back QTL (5244) | 5 | 44633090 | 87546768 |
| ENSSSCG00000029464 | 5 | 67629380 | 67716651 | Carcass weight (hot) QTL (29694) | 5 | 51797310 | 78878062 |
| ENSSSCG00000029464 | 5 | 67629380 | 67716651 | pH 24 hr post-mortem (loin) QTL (101) | 5 | 56883906 | 93349786 |
| ENSSSCG00000029464 | 5 | 67629380 | 67716651 | Haptoglobin concentration QTL (9643) | 5 | 57886897 | 85039291 |
| ENSSSCG00000029464 | 5 | 67629380 | 67716651 | Lauric acid content QTL (156930) | 5 | 59033172 | 1.05E+08 |
| ENSSSCG00000029464 | 5 | 67629380 | 67716651 | Linoleic acid content QTL (156931) | 5 | 59033172 | 1.05E+08 |
| ENSSSCG00000029464 | 5 | 67629380 | 67716651 | Cholesterol level QTL (15069) | 5 | 59033172 | 1.05E+08 |
| ENSSSCG00000029464 | 5 | 67629380 | 67716651 | Average daily gain QTL (342) | 5 | 63188420 | 85039291 |
| ENSSSCG00000029464 | 5 | 67629380 | 67716651 | Average backfat thickness QTL (351) | 5 | 63188420 | 85039291 |
| ENSSSCG00000029464 | 5 | 67629380 | 67716651 | Meat color-L QTL (12087) | 5 | 63188420 | 85039291 |
| ENSSSCG00000029464 | 5 | 67629380 | 67716651 | Meat color OPTO QTL (12088) | 5 | 63188420 | 85039291 |
| ENSSSCG00000029464 | 5 | 67629380 | 67716651 | Meat color OPTO QTL (12089) | 5 | 63188420 | 85039291 |
| ENSSSCG00000029464 | 5 | 67629380 | 67716651 | Japanese color scale QTL (12090) | 5 | 63188420 | 85039291 |
| ENSSSCG00000029464 | 5 | 67629380 | 67716651 | Loin muscle area QTL (12091) | 5 | 63188420 | 85039291 |
| ENSSSCG00000029464 | 5 | 67629380 | 67716651 | subjective abnormal odor QTL (4208) | 5 | 63188420 | 85039291 |
| ENSSSCG00000029464 | 5 | 67629380 | 67716651 | Inside ham weight QTL (3060) | 5 | 63188420 | 77373576 |
| ENSSSCG00000029464 | 5 | 67629380 | 67716651 | Carcass temperature (24 hr post-mortem) QTL (3307) | 5 | 63188420 | 77373576 |
| ENSSSCG00000029464 | 5 | 67629380 | 67716651 | Empty body protein linear QTL (3644) | 5 | 63188420 | 77373576 |
| ENSSSCG00000029464 | 5 | 67629380 | 67716651 | Carcass temperature (24 hr post-mortem) QTL (21364) | 5 | 63188420 | 77373576 |
| ENSSSCG00000029464 | 5 | 67629380 | 67716651 | Alkaline phosphatase activity QTL (6353) | 5 | 63188420 | 77373576 |
| ENSSSCG00000029464 | 5 | 67629380 | 67716651 | Carcass weight (cold) QTL (12752) | 5 | 63188420 | 76657154 |
| ENSSSCG00000029464 | 5 | 67629380 | 67716651 | Conductivity 24 hours post-mortem QTL (987) | 5 | 63188420 | 76657154 |
| ENSSSCG00000029464 | 5 | 67629380 | 67716651 | Backfat between 6th and 7th ribs QTL (3949) | 5 | 63188420 | 76657154 |
| ENSSSCG00000029464 | 5 | 67629380 | 67716651 | Estimated carcass lean content QTL (5979) | 5 | 63188420 | 72788475 |
| ENSSSCG00000029464 | 5 | 67629380 | 67716651 | Intramuscular fat content QTL (17793) | 5 | 63188420 | 69851145 |
| ENSSSCG00000029464 | 5 | 67629380 | 67716651 | Body weight (34 weeks) QTL (21235) | 5 | 65409328 | 86113924 |
| ENSSSCG00000000755 | 5 | 68223389 | 68608703 | Shoulder subcutaneous fat thickness QTL (5219) | 5 | 6017944 | 1.04E+08 |
| ENSSSCG00000000755 | 5 | 68223389 | 68608703 | backfat above muscle dorsi QTL (5203) | 5 | 6017944 | 96502043 |
| ENSSSCG00000000755 | 5 | 68223389 | 68608703 | backfat above muscle dorsi QTL (5238) | 5 | 6017944 | 90699025 |
| ENSSSCG00000000755 | 5 | 68223389 | 68608703 | Meat color L* QTL (38088) | 5 | 6017944 | 89552750 |
| ENSSSCG00000000755 | 5 | 68223389 | 68608703 | Loin muscle area QTL (38085) | 5 | 6017944 | 84036300 |
| ENSSSCG00000000755 | 5 | 68223389 | 68608703 | Backfat at mid-back QTL (5210) | 5 | 11104541 | 1.04E+08 |
| ENSSSCG00000000755 | 5 | 68223389 | 68608703 | Backfat at mid-back QTL (5209) | 5 | 20059816 | 78663135 |
| ENSSSCG00000000755 | 5 | 68223389 | 68608703 | Backfat at first rib QTL (21236) | 5 | 23355357 | 86901988 |
| ENSSSCG00000000755 | 5 | 68223389 | 68608703 | Marbling QTL (38086) | 5 | 28585237 | 1.05E+08 |
| ENSSSCG00000000755 | 5 | 68223389 | 68608703 | Meat color b* QTL (38087) | 5 | 28585237 | 89552750 |
| ENSSSCG00000000755 | 5 | 68223389 | 68608703 | body weight (10 weeks) QTL (29692) | 5 | 28585237 | 68418301 |
| ENSSSCG00000000755 | 5 | 68223389 | 68608703 | Body weight (20 weeks) QTL (29693) | 5 | 28585237 | 68418301 |
| ENSSSCG00000000755 | 5 | 68223389 | 68608703 | Backfat at rump QTL (21237) | 5 | 30877788 | 86543777 |
| ENSSSCG00000000755 | 5 | 68223389 | 68608703 | Shoulder subcutaneous fat thickness QTL (21239) | 5 | 32238990 | 97863245 |
| ENSSSCG00000000755 | 5 | 68223389 | 68608703 | Meat color b* QTL (3918) | 5 | 35813935 | 79594484 |
| ENSSSCG00000000755 | 5 | 68223389 | 68608703 | Meat color b* QTL (3919) | 5 | 35813935 | 79594484 |
| ENSSSCG00000000755 | 5 | 68223389 | 68608703 | Meat color b* QTL (3920) | 5 | 35813935 | 79594484 |
| ENSSSCG00000000755 | 5 | 68223389 | 68608703 | Backfat at mid-back QTL (5244) | 5 | 44633090 | 87546768 |
| ENSSSCG00000000755 | 5 | 68223389 | 68608703 | Carcass weight (hot) QTL (29694) | 5 | 51797310 | 78878062 |
| ENSSSCG00000000755 | 5 | 68223389 | 68608703 | pH 24 hr post-mortem (loin) QTL (101) | 5 | 56883906 | 93349786 |
| ENSSSCG00000000755 | 5 | 68223389 | 68608703 | Haptoglobin concentration QTL (9643) | 5 | 57886897 | 85039291 |
| ENSSSCG00000000755 | 5 | 68223389 | 68608703 | Lauric acid content QTL (156930) | 5 | 59033172 | 1.05E+08 |
| ENSSSCG00000000755 | 5 | 68223389 | 68608703 | Linoleic acid content QTL (156931) | 5 | 59033172 | 1.05E+08 |
| ENSSSCG00000000755 | 5 | 68223389 | 68608703 | Cholesterol level QTL (15069) | 5 | 59033172 | 1.05E+08 |
| ENSSSCG00000000755 | 5 | 68223389 | 68608703 | Average daily gain QTL (342) | 5 | 63188420 | 85039291 |
| ENSSSCG00000000755 | 5 | 68223389 | 68608703 | Average backfat thickness QTL (351) | 5 | 63188420 | 85039291 |
| ENSSSCG00000000755 | 5 | 68223389 | 68608703 | Meat color-L QTL (12087) | 5 | 63188420 | 85039291 |
| ENSSSCG00000000755 | 5 | 68223389 | 68608703 | Meat color OPTO QTL (12088) | 5 | 63188420 | 85039291 |
| ENSSSCG00000000755 | 5 | 68223389 | 68608703 | Meat color OPTO QTL (12089) | 5 | 63188420 | 85039291 |
| ENSSSCG00000000755 | 5 | 68223389 | 68608703 | Japanese color scale QTL (12090) | 5 | 63188420 | 85039291 |
| ENSSSCG00000000755 | 5 | 68223389 | 68608703 | Loin muscle area QTL (12091) | 5 | 63188420 | 85039291 |
| ENSSSCG00000000755 | 5 | 68223389 | 68608703 | subjective abnormal odor QTL (4208) | 5 | 63188420 | 85039291 |
| ENSSSCG00000000755 | 5 | 68223389 | 68608703 | Inside ham weight QTL (3060) | 5 | 63188420 | 77373576 |
| ENSSSCG00000000755 | 5 | 68223389 | 68608703 | Carcass temperature (24 hr post-mortem) QTL (3307) | 5 | 63188420 | 77373576 |
| ENSSSCG00000000755 | 5 | 68223389 | 68608703 | Empty body protein linear QTL (3644) | 5 | 63188420 | 77373576 |
| ENSSSCG00000000755 | 5 | 68223389 | 68608703 | Carcass temperature (24 hr post-mortem) QTL (21364) | 5 | 63188420 | 77373576 |
| ENSSSCG00000000755 | 5 | 68223389 | 68608703 | Alkaline phosphatase activity QTL (6353) | 5 | 63188420 | 77373576 |
| ENSSSCG00000000755 | 5 | 68223389 | 68608703 | Carcass weight (cold) QTL (12752) | 5 | 63188420 | 76657154 |
| ENSSSCG00000000755 | 5 | 68223389 | 68608703 | Conductivity 24 hours post-mortem QTL (987) | 5 | 63188420 | 76657154 |
| ENSSSCG00000000755 | 5 | 68223389 | 68608703 | Backfat between 6th and 7th ribs QTL (3949) | 5 | 63188420 | 76657154 |
| ENSSSCG00000000755 | 5 | 68223389 | 68608703 | Estimated carcass lean content QTL (5979) | 5 | 63188420 | 72788475 |
| ENSSSCG00000000755 | 5 | 68223389 | 68608703 | Intramuscular fat content QTL (17793) | 5 | 63188420 | 69851145 |
| ENSSSCG00000000755 | 5 | 68223389 | 68608703 | Body weight (34 weeks) QTL (21235) | 5 | 65409328 | 86113924 |
| ENSSSCG00000000770 | 5 | 69887884 | 70133277 | Average backfat thickness QTL (193584) | 5 | 70055268 | 70055272 |
| ENSSSCG00000000770 | 5 | 69887884 | 70133277 | Loin muscle area QTL (8960) | 5 | 69836816 | 70137713 |
| ENSSSCG00000000770 | 5 | 69887884 | 70133277 | Marbling QTL (9029) | 5 | 69836816 | 70137713 |
| ENSSSCG00000000770 | 5 | 69887884 | 70133277 | Carcass length QTL (29695) | 5 | 68418301 | 1.05E+08 |
| ENSSSCG00000000770 | 5 | 69887884 | 70133277 | Arachidic acid content QTL (156932) | 5 | 68418301 | 1.05E+08 |
| ENSSSCG00000000770 | 5 | 69887884 | 70133277 | Unsaturated fatty acid content QTL (3113) | 5 | 68418301 | 78376566 |
| ENSSSCG00000000770 | 5 | 69887884 | 70133277 | Saturated fatty acid content QTL (3114) | 5 | 68418301 | 78376566 |
| ENSSSCG00000000770 | 5 | 69887884 | 70133277 | Unsaturated fatty acid content QTL (3128) | 5 | 68418301 | 78376566 |
| ENSSSCG00000000770 | 5 | 69887884 | 70133277 | Shoulder subcutaneous fat thickness QTL (5219) | 5 | 6017944 | 1.04E+08 |
| ENSSSCG00000000770 | 5 | 69887884 | 70133277 | backfat above muscle dorsi QTL (5203) | 5 | 6017944 | 96502043 |
| ENSSSCG00000000770 | 5 | 69887884 | 70133277 | backfat above muscle dorsi QTL (5238) | 5 | 6017944 | 90699025 |
| ENSSSCG00000000770 | 5 | 69887884 | 70133277 | Meat color L* QTL (38088) | 5 | 6017944 | 89552750 |
| ENSSSCG00000000770 | 5 | 69887884 | 70133277 | Loin muscle area QTL (38085) | 5 | 6017944 | 84036300 |
| ENSSSCG00000000770 | 5 | 69887884 | 70133277 | Backfat at mid-back QTL (5210) | 5 | 11104541 | 1.04E+08 |
| ENSSSCG00000000770 | 5 | 69887884 | 70133277 | Backfat at mid-back QTL (5209) | 5 | 20059816 | 78663135 |
| ENSSSCG00000000770 | 5 | 69887884 | 70133277 | Backfat at first rib QTL (21236) | 5 | 23355357 | 86901988 |
| ENSSSCG00000000770 | 5 | 69887884 | 70133277 | Marbling QTL (38086) | 5 | 28585237 | 1.05E+08 |
| ENSSSCG00000000770 | 5 | 69887884 | 70133277 | Meat color b* QTL (38087) | 5 | 28585237 | 89552750 |
| ENSSSCG00000000770 | 5 | 69887884 | 70133277 | Backfat at rump QTL (21237) | 5 | 30877788 | 86543777 |
| ENSSSCG00000000770 | 5 | 69887884 | 70133277 | Shoulder subcutaneous fat thickness QTL (21239) | 5 | 32238990 | 97863245 |
| ENSSSCG00000000770 | 5 | 69887884 | 70133277 | Meat color b* QTL (3918) | 5 | 35813935 | 79594484 |
| ENSSSCG00000000770 | 5 | 69887884 | 70133277 | Meat color b* QTL (3919) | 5 | 35813935 | 79594484 |
| ENSSSCG00000000770 | 5 | 69887884 | 70133277 | Meat color b* QTL (3920) | 5 | 35813935 | 79594484 |
| ENSSSCG00000000770 | 5 | 69887884 | 70133277 | Backfat at mid-back QTL (5244) | 5 | 44633090 | 87546768 |
| ENSSSCG00000000770 | 5 | 69887884 | 70133277 | Carcass weight (hot) QTL (29694) | 5 | 51797310 | 78878062 |
| ENSSSCG00000000770 | 5 | 69887884 | 70133277 | pH 24 hr post-mortem (loin) QTL (101) | 5 | 56883906 | 93349786 |
| ENSSSCG00000000770 | 5 | 69887884 | 70133277 | Haptoglobin concentration QTL (9643) | 5 | 57886897 | 85039291 |
| ENSSSCG00000000770 | 5 | 69887884 | 70133277 | Lauric acid content QTL (156930) | 5 | 59033172 | 1.05E+08 |
| ENSSSCG00000000770 | 5 | 69887884 | 70133277 | Linoleic acid content QTL (156931) | 5 | 59033172 | 1.05E+08 |
| ENSSSCG00000000770 | 5 | 69887884 | 70133277 | Cholesterol level QTL (15069) | 5 | 59033172 | 1.05E+08 |
| ENSSSCG00000000770 | 5 | 69887884 | 70133277 | Average daily gain QTL (342) | 5 | 63188420 | 85039291 |
| ENSSSCG00000000770 | 5 | 69887884 | 70133277 | Average backfat thickness QTL (351) | 5 | 63188420 | 85039291 |
| ENSSSCG00000000770 | 5 | 69887884 | 70133277 | Meat color-L QTL (12087) | 5 | 63188420 | 85039291 |
| ENSSSCG00000000770 | 5 | 69887884 | 70133277 | Meat color OPTO QTL (12088) | 5 | 63188420 | 85039291 |
| ENSSSCG00000000770 | 5 | 69887884 | 70133277 | Meat color OPTO QTL (12089) | 5 | 63188420 | 85039291 |
| ENSSSCG00000000770 | 5 | 69887884 | 70133277 | Japanese color scale QTL (12090) | 5 | 63188420 | 85039291 |
| ENSSSCG00000000770 | 5 | 69887884 | 70133277 | Loin muscle area QTL (12091) | 5 | 63188420 | 85039291 |
| ENSSSCG00000000770 | 5 | 69887884 | 70133277 | subjective abnormal odor QTL (4208) | 5 | 63188420 | 85039291 |
| ENSSSCG00000000770 | 5 | 69887884 | 70133277 | Inside ham weight QTL (3060) | 5 | 63188420 | 77373576 |
| ENSSSCG00000000770 | 5 | 69887884 | 70133277 | Carcass temperature (24 hr post-mortem) QTL (3307) | 5 | 63188420 | 77373576 |
| ENSSSCG00000000770 | 5 | 69887884 | 70133277 | Empty body protein linear QTL (3644) | 5 | 63188420 | 77373576 |
| ENSSSCG00000000770 | 5 | 69887884 | 70133277 | Carcass temperature (24 hr post-mortem) QTL (21364) | 5 | 63188420 | 77373576 |
| ENSSSCG00000000770 | 5 | 69887884 | 70133277 | Alkaline phosphatase activity QTL (6353) | 5 | 63188420 | 77373576 |
| ENSSSCG00000000770 | 5 | 69887884 | 70133277 | Carcass weight (cold) QTL (12752) | 5 | 63188420 | 76657154 |
| ENSSSCG00000000770 | 5 | 69887884 | 70133277 | Conductivity 24 hours post-mortem QTL (987) | 5 | 63188420 | 76657154 |
| ENSSSCG00000000770 | 5 | 69887884 | 70133277 | Backfat between 6th and 7th ribs QTL (3949) | 5 | 63188420 | 76657154 |
| ENSSSCG00000000770 | 5 | 69887884 | 70133277 | Estimated carcass lean content QTL (5979) | 5 | 63188420 | 72788475 |
| ENSSSCG00000000770 | 5 | 69887884 | 70133277 | Body weight (34 weeks) QTL (21235) | 5 | 65409328 | 86113924 |
| ENSSSCG00000000784 | 5 | 71800240 | 71944938 | Carcass length QTL (29695) | 5 | 68418301 | 1.05E+08 |
| ENSSSCG00000000784 | 5 | 71800240 | 71944938 | Arachidic acid content QTL (156932) | 5 | 68418301 | 1.05E+08 |
| ENSSSCG00000000784 | 5 | 71800240 | 71944938 | Unsaturated fatty acid content QTL (3113) | 5 | 68418301 | 78376566 |
| ENSSSCG00000000784 | 5 | 71800240 | 71944938 | Saturated fatty acid content QTL (3114) | 5 | 68418301 | 78376566 |
| ENSSSCG00000000784 | 5 | 71800240 | 71944938 | Unsaturated fatty acid content QTL (3128) | 5 | 68418301 | 78376566 |
| ENSSSCG00000000784 | 5 | 71800240 | 71944938 | Shoulder subcutaneous fat thickness QTL (5219) | 5 | 6017944 | 1.04E+08 |
| ENSSSCG00000000784 | 5 | 71800240 | 71944938 | backfat above muscle dorsi QTL (5203) | 5 | 6017944 | 96502043 |
| ENSSSCG00000000784 | 5 | 71800240 | 71944938 | backfat above muscle dorsi QTL (5238) | 5 | 6017944 | 90699025 |
| ENSSSCG00000000784 | 5 | 71800240 | 71944938 | Meat color L* QTL (38088) | 5 | 6017944 | 89552750 |
| ENSSSCG00000000784 | 5 | 71800240 | 71944938 | Loin muscle area QTL (38085) | 5 | 6017944 | 84036300 |
| ENSSSCG00000000784 | 5 | 71800240 | 71944938 | Backfat at mid-back QTL (5210) | 5 | 11104541 | 1.04E+08 |
| ENSSSCG00000000784 | 5 | 71800240 | 71944938 | Backfat at mid-back QTL (5209) | 5 | 20059816 | 78663135 |
| ENSSSCG00000000784 | 5 | 71800240 | 71944938 | Backfat at first rib QTL (21236) | 5 | 23355357 | 86901988 |
| ENSSSCG00000000784 | 5 | 71800240 | 71944938 | Marbling QTL (38086) | 5 | 28585237 | 1.05E+08 |
| ENSSSCG00000000784 | 5 | 71800240 | 71944938 | Meat color b* QTL (38087) | 5 | 28585237 | 89552750 |
| ENSSSCG00000000784 | 5 | 71800240 | 71944938 | Backfat at rump QTL (21237) | 5 | 30877788 | 86543777 |
| ENSSSCG00000000784 | 5 | 71800240 | 71944938 | Shoulder subcutaneous fat thickness QTL (21239) | 5 | 32238990 | 97863245 |
| ENSSSCG00000000784 | 5 | 71800240 | 71944938 | Meat color b* QTL (3918) | 5 | 35813935 | 79594484 |
| ENSSSCG00000000784 | 5 | 71800240 | 71944938 | Meat color b* QTL (3919) | 5 | 35813935 | 79594484 |
| ENSSSCG00000000784 | 5 | 71800240 | 71944938 | Meat color b* QTL (3920) | 5 | 35813935 | 79594484 |
| ENSSSCG00000000784 | 5 | 71800240 | 71944938 | Backfat at mid-back QTL (5244) | 5 | 44633090 | 87546768 |
| ENSSSCG00000000784 | 5 | 71800240 | 71944938 | Carcass weight (hot) QTL (29694) | 5 | 51797310 | 78878062 |
| ENSSSCG00000000784 | 5 | 71800240 | 71944938 | pH 24 hr post-mortem (loin) QTL (101) | 5 | 56883906 | 93349786 |
| ENSSSCG00000000784 | 5 | 71800240 | 71944938 | Haptoglobin concentration QTL (9643) | 5 | 57886897 | 85039291 |
| ENSSSCG00000000784 | 5 | 71800240 | 71944938 | Lauric acid content QTL (156930) | 5 | 59033172 | 1.05E+08 |
| ENSSSCG00000000784 | 5 | 71800240 | 71944938 | Linoleic acid content QTL (156931) | 5 | 59033172 | 1.05E+08 |
| ENSSSCG00000000784 | 5 | 71800240 | 71944938 | Cholesterol level QTL (15069) | 5 | 59033172 | 1.05E+08 |
| ENSSSCG00000000784 | 5 | 71800240 | 71944938 | Average daily gain QTL (342) | 5 | 63188420 | 85039291 |
| ENSSSCG00000000784 | 5 | 71800240 | 71944938 | Average backfat thickness QTL (351) | 5 | 63188420 | 85039291 |
| ENSSSCG00000000784 | 5 | 71800240 | 71944938 | Meat color-L QTL (12087) | 5 | 63188420 | 85039291 |
| ENSSSCG00000000784 | 5 | 71800240 | 71944938 | Meat color OPTO QTL (12088) | 5 | 63188420 | 85039291 |
| ENSSSCG00000000784 | 5 | 71800240 | 71944938 | Meat color OPTO QTL (12089) | 5 | 63188420 | 85039291 |
| ENSSSCG00000000784 | 5 | 71800240 | 71944938 | Japanese color scale QTL (12090) | 5 | 63188420 | 85039291 |
| ENSSSCG00000000784 | 5 | 71800240 | 71944938 | Loin muscle area QTL (12091) | 5 | 63188420 | 85039291 |
| ENSSSCG00000000784 | 5 | 71800240 | 71944938 | subjective abnormal odor QTL (4208) | 5 | 63188420 | 85039291 |
| ENSSSCG00000000784 | 5 | 71800240 | 71944938 | Inside ham weight QTL (3060) | 5 | 63188420 | 77373576 |
| ENSSSCG00000000784 | 5 | 71800240 | 71944938 | Carcass temperature (24 hr post-mortem) QTL (3307) | 5 | 63188420 | 77373576 |
| ENSSSCG00000000784 | 5 | 71800240 | 71944938 | Empty body protein linear QTL (3644) | 5 | 63188420 | 77373576 |
| ENSSSCG00000000784 | 5 | 71800240 | 71944938 | Carcass temperature (24 hr post-mortem) QTL (21364) | 5 | 63188420 | 77373576 |
| ENSSSCG00000000784 | 5 | 71800240 | 71944938 | Alkaline phosphatase activity QTL (6353) | 5 | 63188420 | 77373576 |
| ENSSSCG00000000784 | 5 | 71800240 | 71944938 | Carcass weight (cold) QTL (12752) | 5 | 63188420 | 76657154 |
| ENSSSCG00000000784 | 5 | 71800240 | 71944938 | Conductivity 24 hours post-mortem QTL (987) | 5 | 63188420 | 76657154 |
| ENSSSCG00000000784 | 5 | 71800240 | 71944938 | Backfat between 6th and 7th ribs QTL (3949) | 5 | 63188420 | 76657154 |
| ENSSSCG00000000784 | 5 | 71800240 | 71944938 | Estimated carcass lean content QTL (5979) | 5 | 63188420 | 72788475 |
| ENSSSCG00000000784 | 5 | 71800240 | 71944938 | Body weight (34 weeks) QTL (21235) | 5 | 65409328 | 86113924 |
| ENSSSCG00000000801 | 5 | 74917463 | 75406367 | Palmitoleic acid content QTL (18584) | 5 | 74981464 | 74981468 |
| ENSSSCG00000000801 | 5 | 74917463 | 75406367 | Half carcass weight QTL (13260) | 5 | 75131175 | 75209981 |
| ENSSSCG00000000801 | 5 | 74917463 | 75406367 | Meat color b* QTL (12672) | 5 | 75159832 | 75181324 |
| ENSSSCG00000000801 | 5 | 74917463 | 75406367 | Carcass length QTL (13376) | 5 | 74808785 | 75346101 |
| ENSSSCG00000000801 | 5 | 74917463 | 75406367 | Carcass length QTL (29695) | 5 | 68418301 | 1.05E+08 |
| ENSSSCG00000000801 | 5 | 74917463 | 75406367 | Arachidic acid content QTL (156932) | 5 | 68418301 | 1.05E+08 |
| ENSSSCG00000000801 | 5 | 74917463 | 75406367 | Unsaturated fatty acid content QTL (3113) | 5 | 68418301 | 78376566 |
| ENSSSCG00000000801 | 5 | 74917463 | 75406367 | Saturated fatty acid content QTL (3114) | 5 | 68418301 | 78376566 |
| ENSSSCG00000000801 | 5 | 74917463 | 75406367 | Unsaturated fatty acid content QTL (3128) | 5 | 68418301 | 78376566 |
| ENSSSCG00000000801 | 5 | 74917463 | 75406367 | Interleukin-2 level QTL (17598) | 5 | 72788475 | 85039291 |
| ENSSSCG00000000801 | 5 | 74917463 | 75406367 | Loin muscle area QTL (5976) | 5 | 72788475 | 85039291 |
| ENSSSCG00000000801 | 5 | 74917463 | 75406367 | Backfat at tenth rib QTL (2988) | 5 | 72788475 | 81170612 |
| ENSSSCG00000000801 | 5 | 74917463 | 75406367 | Shear force QTL (3872) | 5 | 74006392 | 84394511 |
| ENSSSCG00000000801 | 5 | 74917463 | 75406367 | Shoulder subcutaneous fat thickness QTL (5219) | 5 | 6017944 | 1.04E+08 |
| ENSSSCG00000000801 | 5 | 74917463 | 75406367 | backfat above muscle dorsi QTL (5203) | 5 | 6017944 | 96502043 |
| ENSSSCG00000000801 | 5 | 74917463 | 75406367 | backfat above muscle dorsi QTL (5238) | 5 | 6017944 | 90699025 |
| ENSSSCG00000000801 | 5 | 74917463 | 75406367 | Meat color L* QTL (38088) | 5 | 6017944 | 89552750 |
| ENSSSCG00000000801 | 5 | 74917463 | 75406367 | Loin muscle area QTL (38085) | 5 | 6017944 | 84036300 |
| ENSSSCG00000000801 | 5 | 74917463 | 75406367 | Backfat at mid-back QTL (5210) | 5 | 11104541 | 1.04E+08 |
| ENSSSCG00000000801 | 5 | 74917463 | 75406367 | Backfat at mid-back QTL (5209) | 5 | 20059816 | 78663135 |
| ENSSSCG00000000801 | 5 | 74917463 | 75406367 | Backfat at first rib QTL (21236) | 5 | 23355357 | 86901988 |
| ENSSSCG00000000801 | 5 | 74917463 | 75406367 | Marbling QTL (38086) | 5 | 28585237 | 1.05E+08 |
| ENSSSCG00000000801 | 5 | 74917463 | 75406367 | Meat color b* QTL (38087) | 5 | 28585237 | 89552750 |
| ENSSSCG00000000801 | 5 | 74917463 | 75406367 | Backfat at rump QTL (21237) | 5 | 30877788 | 86543777 |
| ENSSSCG00000000801 | 5 | 74917463 | 75406367 | Shoulder subcutaneous fat thickness QTL (21239) | 5 | 32238990 | 97863245 |
| ENSSSCG00000000801 | 5 | 74917463 | 75406367 | Meat color b* QTL (3918) | 5 | 35813935 | 79594484 |
| ENSSSCG00000000801 | 5 | 74917463 | 75406367 | Meat color b* QTL (3919) | 5 | 35813935 | 79594484 |
| ENSSSCG00000000801 | 5 | 74917463 | 75406367 | Meat color b* QTL (3920) | 5 | 35813935 | 79594484 |
| ENSSSCG00000000801 | 5 | 74917463 | 75406367 | Backfat at mid-back QTL (5244) | 5 | 44633090 | 87546768 |
| ENSSSCG00000000801 | 5 | 74917463 | 75406367 | Carcass weight (hot) QTL (29694) | 5 | 51797310 | 78878062 |
| ENSSSCG00000000801 | 5 | 74917463 | 75406367 | pH 24 hr post-mortem (loin) QTL (101) | 5 | 56883906 | 93349786 |
| ENSSSCG00000000801 | 5 | 74917463 | 75406367 | Haptoglobin concentration QTL (9643) | 5 | 57886897 | 85039291 |
| ENSSSCG00000000801 | 5 | 74917463 | 75406367 | Lauric acid content QTL (156930) | 5 | 59033172 | 1.05E+08 |
| ENSSSCG00000000801 | 5 | 74917463 | 75406367 | Linoleic acid content QTL (156931) | 5 | 59033172 | 1.05E+08 |
| ENSSSCG00000000801 | 5 | 74917463 | 75406367 | Cholesterol level QTL (15069) | 5 | 59033172 | 1.05E+08 |
| ENSSSCG00000000801 | 5 | 74917463 | 75406367 | Average daily gain QTL (342) | 5 | 63188420 | 85039291 |
| ENSSSCG00000000801 | 5 | 74917463 | 75406367 | Average backfat thickness QTL (351) | 5 | 63188420 | 85039291 |
| ENSSSCG00000000801 | 5 | 74917463 | 75406367 | Meat color-L QTL (12087) | 5 | 63188420 | 85039291 |
| ENSSSCG00000000801 | 5 | 74917463 | 75406367 | Meat color OPTO QTL (12088) | 5 | 63188420 | 85039291 |
| ENSSSCG00000000801 | 5 | 74917463 | 75406367 | Meat color OPTO QTL (12089) | 5 | 63188420 | 85039291 |
| ENSSSCG00000000801 | 5 | 74917463 | 75406367 | Japanese color scale QTL (12090) | 5 | 63188420 | 85039291 |
| ENSSSCG00000000801 | 5 | 74917463 | 75406367 | Loin muscle area QTL (12091) | 5 | 63188420 | 85039291 |
| ENSSSCG00000000801 | 5 | 74917463 | 75406367 | subjective abnormal odor QTL (4208) | 5 | 63188420 | 85039291 |
| ENSSSCG00000000801 | 5 | 74917463 | 75406367 | Inside ham weight QTL (3060) | 5 | 63188420 | 77373576 |
| ENSSSCG00000000801 | 5 | 74917463 | 75406367 | Carcass temperature (24 hr post-mortem) QTL (3307) | 5 | 63188420 | 77373576 |
| ENSSSCG00000000801 | 5 | 74917463 | 75406367 | Empty body protein linear QTL (3644) | 5 | 63188420 | 77373576 |
| ENSSSCG00000000801 | 5 | 74917463 | 75406367 | Carcass temperature (24 hr post-mortem) QTL (21364) | 5 | 63188420 | 77373576 |
| ENSSSCG00000000801 | 5 | 74917463 | 75406367 | Alkaline phosphatase activity QTL (6353) | 5 | 63188420 | 77373576 |
| ENSSSCG00000000801 | 5 | 74917463 | 75406367 | Carcass weight (cold) QTL (12752) | 5 | 63188420 | 76657154 |
| ENSSSCG00000000801 | 5 | 74917463 | 75406367 | Conductivity 24 hours post-mortem QTL (987) | 5 | 63188420 | 76657154 |
| ENSSSCG00000000801 | 5 | 74917463 | 75406367 | Backfat between 6th and 7th ribs QTL (3949) | 5 | 63188420 | 76657154 |
| ENSSSCG00000000801 | 5 | 74917463 | 75406367 | Body weight (34 weeks) QTL (21235) | 5 | 65409328 | 86113924 |
| ENSSSCG00000000866 | 5 | 82557607 | 82696126 | Carcass length QTL (29695) | 5 | 68418301 | 1.05E+08 |
| ENSSSCG00000000866 | 5 | 82557607 | 82696126 | Arachidic acid content QTL (156932) | 5 | 68418301 | 1.05E+08 |
| ENSSSCG00000000866 | 5 | 82557607 | 82696126 | Interleukin-2 level QTL (17598) | 5 | 72788475 | 85039291 |
| ENSSSCG00000000866 | 5 | 82557607 | 82696126 | Loin muscle area QTL (5976) | 5 | 72788475 | 85039291 |
| ENSSSCG00000000866 | 5 | 82557607 | 82696126 | Shear force QTL (3872) | 5 | 74006392 | 84394511 |
| ENSSSCG00000000866 | 5 | 82557607 | 82696126 | Average daily gain QTL (16849) | 5 | 76657154 | 1.01E+08 |
| ENSSSCG00000000866 | 5 | 82557607 | 82696126 | Adipocyte diameter QTL (18021) | 5 | 76657154 | 88478117 |
| ENSSSCG00000000866 | 5 | 82557607 | 82696126 | Interferon-gamma level QTL (12264) | 5 | 76657154 | 85039291 |
| ENSSSCG00000000866 | 5 | 82557607 | 82696126 | Carcass length QTL (982) | 5 | 76657154 | 85039291 |
| ENSSSCG00000000866 | 5 | 82557607 | 82696126 | Activity during lying QTL (5901) | 5 | 77373576 | 96287116 |
| ENSSSCG00000000866 | 5 | 82557607 | 82696126 | Time spent drinking QTL (5913) | 5 | 77373576 | 96287116 |
| ENSSSCG00000000866 | 5 | 82557607 | 82696126 | Empty body protein content QTL (3645) | 5 | 77373576 | 89552750 |
| ENSSSCG00000000866 | 5 | 82557607 | 82696126 | Phagocytic activity QTL (21330) | 5 | 77373576 | 89552750 |
| ENSSSCG00000000866 | 5 | 82557607 | 82696126 | Backfat at last rib QTL (55) | 5 | 79594484 | 1.05E+08 |
| ENSSSCG00000000866 | 5 | 82557607 | 82696126 | Meat color-L QTL (97) | 5 | 81528823 | 1.05E+08 |
| ENSSSCG00000000866 | 5 | 82557607 | 82696126 | Shoulder subcutaneous fat thickness QTL (5219) | 5 | 6017944 | 1.04E+08 |
| ENSSSCG00000000866 | 5 | 82557607 | 82696126 | backfat above muscle dorsi QTL (5203) | 5 | 6017944 | 96502043 |
| ENSSSCG00000000866 | 5 | 82557607 | 82696126 | backfat above muscle dorsi QTL (5238) | 5 | 6017944 | 90699025 |
| ENSSSCG00000000866 | 5 | 82557607 | 82696126 | Meat color L* QTL (38088) | 5 | 6017944 | 89552750 |
| ENSSSCG00000000866 | 5 | 82557607 | 82696126 | Loin muscle area QTL (38085) | 5 | 6017944 | 84036300 |
| ENSSSCG00000000866 | 5 | 82557607 | 82696126 | Backfat at mid-back QTL (5210) | 5 | 11104541 | 1.04E+08 |
| ENSSSCG00000000866 | 5 | 82557607 | 82696126 | Backfat at first rib QTL (21236) | 5 | 23355357 | 86901988 |
| ENSSSCG00000000866 | 5 | 82557607 | 82696126 | Marbling QTL (38086) | 5 | 28585237 | 1.05E+08 |
| ENSSSCG00000000866 | 5 | 82557607 | 82696126 | Meat color b* QTL (38087) | 5 | 28585237 | 89552750 |
| ENSSSCG00000000866 | 5 | 82557607 | 82696126 | Backfat at rump QTL (21237) | 5 | 30877788 | 86543777 |
| ENSSSCG00000000866 | 5 | 82557607 | 82696126 | Shoulder subcutaneous fat thickness QTL (21239) | 5 | 32238990 | 97863245 |
| ENSSSCG00000000866 | 5 | 82557607 | 82696126 | Backfat at mid-back QTL (5244) | 5 | 44633090 | 87546768 |
| ENSSSCG00000000866 | 5 | 82557607 | 82696126 | pH 24 hr post-mortem (loin) QTL (101) | 5 | 56883906 | 93349786 |
| ENSSSCG00000000866 | 5 | 82557607 | 82696126 | Haptoglobin concentration QTL (9643) | 5 | 57886897 | 85039291 |
| ENSSSCG00000000866 | 5 | 82557607 | 82696126 | Lauric acid content QTL (156930) | 5 | 59033172 | 1.05E+08 |
| ENSSSCG00000000866 | 5 | 82557607 | 82696126 | Linoleic acid content QTL (156931) | 5 | 59033172 | 1.05E+08 |
| ENSSSCG00000000866 | 5 | 82557607 | 82696126 | Cholesterol level QTL (15069) | 5 | 59033172 | 1.05E+08 |
| ENSSSCG00000000866 | 5 | 82557607 | 82696126 | Average daily gain QTL (342) | 5 | 63188420 | 85039291 |
| ENSSSCG00000000866 | 5 | 82557607 | 82696126 | Average backfat thickness QTL (351) | 5 | 63188420 | 85039291 |
| ENSSSCG00000000866 | 5 | 82557607 | 82696126 | Meat color-L QTL (12087) | 5 | 63188420 | 85039291 |
| ENSSSCG00000000866 | 5 | 82557607 | 82696126 | Meat color OPTO QTL (12088) | 5 | 63188420 | 85039291 |
| ENSSSCG00000000866 | 5 | 82557607 | 82696126 | Meat color OPTO QTL (12089) | 5 | 63188420 | 85039291 |
| ENSSSCG00000000866 | 5 | 82557607 | 82696126 | Japanese color scale QTL (12090) | 5 | 63188420 | 85039291 |
| ENSSSCG00000000866 | 5 | 82557607 | 82696126 | Loin muscle area QTL (12091) | 5 | 63188420 | 85039291 |
| ENSSSCG00000000866 | 5 | 82557607 | 82696126 | subjective abnormal odor QTL (4208) | 5 | 63188420 | 85039291 |
| ENSSSCG00000000866 | 5 | 82557607 | 82696126 | Body weight (34 weeks) QTL (21235) | 5 | 65409328 | 86113924 |
| ENSSSCG00000000900 | 5 | 88031979 | 88104742 | Conductivity 24 hours post-mortem QTL (983) | 5 | 85039291 | 89552750 |
| ENSSSCG00000000900 | 5 | 88031979 | 88104742 | Carcass length QTL (29695) | 5 | 68418301 | 1.05E+08 |
| ENSSSCG00000000900 | 5 | 88031979 | 88104742 | Arachidic acid content QTL (156932) | 5 | 68418301 | 1.05E+08 |
| ENSSSCG00000000900 | 5 | 88031979 | 88104742 | Average daily gain QTL (16849) | 5 | 76657154 | 1.01E+08 |
| ENSSSCG00000000900 | 5 | 88031979 | 88104742 | Adipocyte diameter QTL (18021) | 5 | 76657154 | 88478117 |
| ENSSSCG00000000900 | 5 | 88031979 | 88104742 | Activity during lying QTL (5901) | 5 | 77373576 | 96287116 |
| ENSSSCG00000000900 | 5 | 88031979 | 88104742 | Time spent drinking QTL (5913) | 5 | 77373576 | 96287116 |
| ENSSSCG00000000900 | 5 | 88031979 | 88104742 | Empty body protein content QTL (3645) | 5 | 77373576 | 89552750 |
| ENSSSCG00000000900 | 5 | 88031979 | 88104742 | Phagocytic activity QTL (21330) | 5 | 77373576 | 89552750 |
| ENSSSCG00000000900 | 5 | 88031979 | 88104742 | Backfat at last rib QTL (55) | 5 | 79594484 | 1.05E+08 |
| ENSSSCG00000000900 | 5 | 88031979 | 88104742 | Meat color-L QTL (97) | 5 | 81528823 | 1.05E+08 |
| ENSSSCG00000000900 | 5 | 88031979 | 88104742 | Average backfat thickness QTL (54) | 5 | 82675098 | 1.05E+08 |
| ENSSSCG00000000900 | 5 | 88031979 | 88104742 | pH 24 hr post-mortem (loin) QTL (98) | 5 | 82675098 | 1.05E+08 |
| ENSSSCG00000000900 | 5 | 88031979 | 88104742 | Backfat at last lumbar QTL (56) | 5 | 82675098 | 96287116 |
| ENSSSCG00000000900 | 5 | 88031979 | 88104742 | Creatinine level QTL (15070) | 5 | 84036300 | 1.05E+08 |
| ENSSSCG00000000900 | 5 | 88031979 | 88104742 | Carcass weight (hot) QTL (16865) | 5 | 85039291 | 1.01E+08 |
| ENSSSCG00000000900 | 5 | 88031979 | 88104742 | Average backfat thickness QTL (17795) | 5 | 85039291 | 96287116 |
| ENSSSCG00000000900 | 5 | 88031979 | 88104742 | Backfat at last rib QTL (17796) | 5 | 85039291 | 96287116 |
| ENSSSCG00000000900 | 5 | 88031979 | 88104742 | Backfat at last lumbar QTL (17797) | 5 | 85039291 | 96287116 |
| ENSSSCG00000000900 | 5 | 88031979 | 88104742 | Linolenic acid content QTL (9581) | 5 | 85039291 | 93349786 |
| ENSSSCG00000000900 | 5 | 88031979 | 88104742 | Shoulder subcutaneous fat thickness QTL (5219) | 5 | 6017944 | 1.04E+08 |
| ENSSSCG00000000900 | 5 | 88031979 | 88104742 | backfat above muscle dorsi QTL (5203) | 5 | 6017944 | 96502043 |
| ENSSSCG00000000900 | 5 | 88031979 | 88104742 | backfat above muscle dorsi QTL (5238) | 5 | 6017944 | 90699025 |
| ENSSSCG00000000900 | 5 | 88031979 | 88104742 | Meat color L* QTL (38088) | 5 | 6017944 | 89552750 |
| ENSSSCG00000000900 | 5 | 88031979 | 88104742 | Backfat at mid-back QTL (5210) | 5 | 11104541 | 1.04E+08 |
| ENSSSCG00000000900 | 5 | 88031979 | 88104742 | Marbling QTL (38086) | 5 | 28585237 | 1.05E+08 |
| ENSSSCG00000000900 | 5 | 88031979 | 88104742 | Meat color b* QTL (38087) | 5 | 28585237 | 89552750 |
| ENSSSCG00000000900 | 5 | 88031979 | 88104742 | Shoulder subcutaneous fat thickness QTL (21239) | 5 | 32238990 | 97863245 |
| ENSSSCG00000000900 | 5 | 88031979 | 88104742 | pH 24 hr post-mortem (loin) QTL (101) | 5 | 56883906 | 93349786 |
| ENSSSCG00000000900 | 5 | 88031979 | 88104742 | Lauric acid content QTL (156930) | 5 | 59033172 | 1.05E+08 |
| ENSSSCG00000000900 | 5 | 88031979 | 88104742 | Linoleic acid content QTL (156931) | 5 | 59033172 | 1.05E+08 |
| ENSSSCG00000000900 | 5 | 88031979 | 88104742 | Cholesterol level QTL (15069) | 5 | 59033172 | 1.05E+08 |
| ENSSSCG00000029249 | 5 | 1.03E+08 | 1.03E+08 | Carcass length QTL (29695) | 5 | 68418301 | 1.05E+08 |
| ENSSSCG00000029249 | 5 | 1.03E+08 | 1.03E+08 | Arachidic acid content QTL (156932) | 5 | 68418301 | 1.05E+08 |
| ENSSSCG00000029249 | 5 | 1.03E+08 | 1.03E+08 | Backfat at last rib QTL (55) | 5 | 79594484 | 1.05E+08 |
| ENSSSCG00000029249 | 5 | 1.03E+08 | 1.03E+08 | Meat color-L QTL (97) | 5 | 81528823 | 1.05E+08 |
| ENSSSCG00000029249 | 5 | 1.03E+08 | 1.03E+08 | Average backfat thickness QTL (54) | 5 | 82675098 | 1.05E+08 |
| ENSSSCG00000029249 | 5 | 1.03E+08 | 1.03E+08 | pH 24 hr post-mortem (loin) QTL (98) | 5 | 82675098 | 1.05E+08 |
| ENSSSCG00000029249 | 5 | 1.03E+08 | 1.03E+08 | Creatinine level QTL (15070) | 5 | 84036300 | 1.05E+08 |
| ENSSSCG00000029249 | 5 | 1.03E+08 | 1.03E+08 | Meat color-L QTL (99) | 5 | 93349786 | 1.05E+08 |
| ENSSSCG00000029249 | 5 | 1.03E+08 | 1.03E+08 | Meat color-L QTL (100) | 5 | 93349786 | 1.05E+08 |
| ENSSSCG00000029249 | 5 | 1.03E+08 | 1.03E+08 | pH 45 minutes post mortem QTL (9851) | 5 | 93349786 | 1.05E+08 |
| ENSSSCG00000029249 | 5 | 1.03E+08 | 1.03E+08 | pH 24 hr post-mortem (loin) QTL (9852) | 5 | 93349786 | 1.05E+08 |
| ENSSSCG00000029249 | 5 | 1.03E+08 | 1.03E+08 | Shoulder subcutaneous fat thickness QTL (5219) | 5 | 6017944 | 1.04E+08 |
| ENSSSCG00000029249 | 5 | 1.03E+08 | 1.03E+08 | Backfat at mid-back QTL (5210) | 5 | 11104541 | 1.04E+08 |
| ENSSSCG00000029249 | 5 | 1.03E+08 | 1.03E+08 | Marbling QTL (38086) | 5 | 28585237 | 1.05E+08 |
| ENSSSCG00000029249 | 5 | 1.03E+08 | 1.03E+08 | Lauric acid content QTL (156930) | 5 | 59033172 | 1.05E+08 |
| ENSSSCG00000029249 | 5 | 1.03E+08 | 1.03E+08 | Linoleic acid content QTL (156931) | 5 | 59033172 | 1.05E+08 |
| ENSSSCG00000029249 | 5 | 1.03E+08 | 1.03E+08 | Cholesterol level QTL (15069) | 5 | 59033172 | 1.05E+08 |
| ENSSSCG00000002703 | 6 | 12014243 | 12057445 | Backfat at last rib QTL (5982) | 6 | 6235784 | 53644827 |
| ENSSSCG00000002703 | 6 | 12014243 | 12057445 | Subcutanous fat area QTL (5983) | 6 | 6235784 | 53644827 |
| ENSSSCG00000002703 | 6 | 12014243 | 12057445 | Estimated carcass lean content QTL (5986) | 6 | 6235784 | 53644827 |
| ENSSSCG00000002703 | 6 | 12014243 | 12057445 | Conductivity 24 hours postmortem (loin) QTL (6004) | 6 | 6235784 | 53644827 |
| ENSSSCG00000002703 | 6 | 12014243 | 12057445 | Drip loss QTL (6005) | 6 | 6235784 | 53644827 |
| ENSSSCG00000002703 | 6 | 12014243 | 12057445 | Loin muscle area QTL (5698) | 6 | 6235784 | 53644827 |
| ENSSSCG00000002703 | 6 | 12014243 | 12057445 | Average daily gain QTL (5699) | 6 | 6235784 | 53644827 |
| ENSSSCG00000002703 | 6 | 12014243 | 12057445 | Lauric acid content QTL (156933) | 6 | 6235784 | 40233620 |
| ENSSSCG00000002703 | 6 | 12014243 | 12057445 | CO2 partial pressure QTL (6329) | 6 | 6235784 | 40233620 |
| ENSSSCG00000002703 | 6 | 12014243 | 12057445 | Adipocyte diameter QTL (18022) | 6 | 6235784 | 40233620 |
| ENSSSCG00000002703 | 6 | 12014243 | 12057445 | Average daily gain QTL (344) | 6 | 6235784 | 18280243 |
| ENSSSCG00000002703 | 6 | 12014243 | 12057445 | Intramuscular fat content QTL (367) | 6 | 8115061 | 35450005 |
| ENSSSCG00000002703 | 6 | 12014243 | 12057445 | Backfat at tenth rib QTL (3859) | 6 | 8115061 | 35450005 |
| ENSSSCG00000002703 | 6 | 12014243 | 12057445 | Inside ham weight QTL (3063) | 6 | 8115061 | 24943136 |
| ENSSSCG00000002703 | 6 | 12014243 | 12057445 | Relative area of type IIa fibers QTL (4029) | 6 | 10677712 | 25113979 |
| ENSSSCG00000002703 | 6 | 12014243 | 12057445 | Backfat at tenth rib QTL (3874) | 6 | 11959038 | 58684707 |
| ENSSSCG00000002703 | 6 | 12014243 | 12057445 | Marbling QTL (38089) | 6 | 6235784 | 1.28E+08 |
| ENSSSCG00000002703 | 6 | 12014243 | 12057445 | Meat color a* QTL (3921) | 6 | 6235784 | 94305556 |
| ENSSSCG00000002703 | 6 | 12014243 | 12057445 | Meat color a* QTL (3922) | 6 | 6235784 | 94305556 |
| ENSSSCG00000002703 | 6 | 12014243 | 12057445 | Meat color a* QTL (3923) | 6 | 6235784 | 94305556 |
| ENSSSCG00000002703 | 6 | 12014243 | 12057445 | Meat color a* QTL (3924) | 6 | 6235784 | 94305556 |
| ENSSSCG00000002703 | 6 | 12014243 | 12057445 | Body weight (46 days) QTL (21240) | 6 | 8029639 | 91486640 |
| ENSSSCG00000025323 | 6 | 13524710 | 13578679 | Backfat at last rib QTL (5982) | 6 | 6235784 | 53644827 |
| ENSSSCG00000025323 | 6 | 13524710 | 13578679 | Subcutanous fat area QTL (5983) | 6 | 6235784 | 53644827 |
| ENSSSCG00000025323 | 6 | 13524710 | 13578679 | Estimated carcass lean content QTL (5986) | 6 | 6235784 | 53644827 |
| ENSSSCG00000025323 | 6 | 13524710 | 13578679 | Conductivity 24 hours postmortem (loin) QTL (6004) | 6 | 6235784 | 53644827 |
| ENSSSCG00000025323 | 6 | 13524710 | 13578679 | Drip loss QTL (6005) | 6 | 6235784 | 53644827 |
| ENSSSCG00000025323 | 6 | 13524710 | 13578679 | Loin muscle area QTL (5698) | 6 | 6235784 | 53644827 |
| ENSSSCG00000025323 | 6 | 13524710 | 13578679 | Average daily gain QTL (5699) | 6 | 6235784 | 53644827 |
| ENSSSCG00000025323 | 6 | 13524710 | 13578679 | Lauric acid content QTL (156933) | 6 | 6235784 | 40233620 |
| ENSSSCG00000025323 | 6 | 13524710 | 13578679 | CO2 partial pressure QTL (6329) | 6 | 6235784 | 40233620 |
| ENSSSCG00000025323 | 6 | 13524710 | 13578679 | Adipocyte diameter QTL (18022) | 6 | 6235784 | 40233620 |
| ENSSSCG00000025323 | 6 | 13524710 | 13578679 | Average daily gain QTL (344) | 6 | 6235784 | 18280243 |
| ENSSSCG00000025323 | 6 | 13524710 | 13578679 | Intramuscular fat content QTL (367) | 6 | 8115061 | 35450005 |
| ENSSSCG00000025323 | 6 | 13524710 | 13578679 | Backfat at tenth rib QTL (3859) | 6 | 8115061 | 35450005 |
| ENSSSCG00000025323 | 6 | 13524710 | 13578679 | Inside ham weight QTL (3063) | 6 | 8115061 | 24943136 |
| ENSSSCG00000025323 | 6 | 13524710 | 13578679 | Relative area of type IIa fibers QTL (4029) | 6 | 10677712 | 25113979 |
| ENSSSCG00000025323 | 6 | 13524710 | 13578679 | Backfat at tenth rib QTL (3874) | 6 | 11959038 | 58684707 |
| ENSSSCG00000025323 | 6 | 13524710 | 13578679 | Meat color a* QTL (4037) | 6 | 12129881 | 33826993 |
| ENSSSCG00000025323 | 6 | 13524710 | 13578679 | Marbling QTL (38089) | 6 | 6235784 | 1.28E+08 |
| ENSSSCG00000025323 | 6 | 13524710 | 13578679 | Meat color a* QTL (3921) | 6 | 6235784 | 94305556 |
| ENSSSCG00000025323 | 6 | 13524710 | 13578679 | Meat color a* QTL (3922) | 6 | 6235784 | 94305556 |
| ENSSSCG00000025323 | 6 | 13524710 | 13578679 | Meat color a* QTL (3923) | 6 | 6235784 | 94305556 |
| ENSSSCG00000025323 | 6 | 13524710 | 13578679 | Meat color a* QTL (3924) | 6 | 6235784 | 94305556 |
| ENSSSCG00000025323 | 6 | 13524710 | 13578679 | Body weight (46 days) QTL (21240) | 6 | 8029639 | 91486640 |
| ENSSSCG00000025323 | 6 | 13524710 | 13578679 | Body weight (end of test) QTL (5201) | 6 | 12044459 | 1.35E+08 |
| ENSSSCG00000002755 | 6 | 17313388 | 17434926 | Backfat at last rib QTL (5982) | 6 | 6235784 | 53644827 |
| ENSSSCG00000002755 | 6 | 17313388 | 17434926 | Subcutanous fat area QTL (5983) | 6 | 6235784 | 53644827 |
| ENSSSCG00000002755 | 6 | 17313388 | 17434926 | Estimated carcass lean content QTL (5986) | 6 | 6235784 | 53644827 |
| ENSSSCG00000002755 | 6 | 17313388 | 17434926 | Conductivity 24 hours postmortem (loin) QTL (6004) | 6 | 6235784 | 53644827 |
| ENSSSCG00000002755 | 6 | 17313388 | 17434926 | Drip loss QTL (6005) | 6 | 6235784 | 53644827 |
| ENSSSCG00000002755 | 6 | 17313388 | 17434926 | Loin muscle area QTL (5698) | 6 | 6235784 | 53644827 |
| ENSSSCG00000002755 | 6 | 17313388 | 17434926 | Average daily gain QTL (5699) | 6 | 6235784 | 53644827 |
| ENSSSCG00000002755 | 6 | 17313388 | 17434926 | Lauric acid content QTL (156933) | 6 | 6235784 | 40233620 |
| ENSSSCG00000002755 | 6 | 17313388 | 17434926 | CO2 partial pressure QTL (6329) | 6 | 6235784 | 40233620 |
| ENSSSCG00000002755 | 6 | 17313388 | 17434926 | Adipocyte diameter QTL (18022) | 6 | 6235784 | 40233620 |
| ENSSSCG00000002755 | 6 | 17313388 | 17434926 | Average daily gain QTL (344) | 6 | 6235784 | 18280243 |
| ENSSSCG00000002755 | 6 | 17313388 | 17434926 | Intramuscular fat content QTL (367) | 6 | 8115061 | 35450005 |
| ENSSSCG00000002755 | 6 | 17313388 | 17434926 | Backfat at tenth rib QTL (3859) | 6 | 8115061 | 35450005 |
| ENSSSCG00000002755 | 6 | 17313388 | 17434926 | Inside ham weight QTL (3063) | 6 | 8115061 | 24943136 |
| ENSSSCG00000002755 | 6 | 17313388 | 17434926 | Relative area of type IIa fibers QTL (4029) | 6 | 10677712 | 25113979 |
| ENSSSCG00000002755 | 6 | 17313388 | 17434926 | Backfat at tenth rib QTL (3874) | 6 | 11959038 | 58684707 |
| ENSSSCG00000002755 | 6 | 17313388 | 17434926 | Meat color a* QTL (4037) | 6 | 12129881 | 33826993 |
| ENSSSCG00000002755 | 6 | 17313388 | 17434926 | Relative area of type IIb fibers QTL (4033) | 6 | 14436267 | 24943136 |
| ENSSSCG00000002755 | 6 | 17313388 | 17434926 | Marbling QTL (38089) | 6 | 6235784 | 1.28E+08 |
| ENSSSCG00000002755 | 6 | 17313388 | 17434926 | Meat color a* QTL (3921) | 6 | 6235784 | 94305556 |
| ENSSSCG00000002755 | 6 | 17313388 | 17434926 | Meat color a* QTL (3922) | 6 | 6235784 | 94305556 |
| ENSSSCG00000002755 | 6 | 17313388 | 17434926 | Meat color a* QTL (3923) | 6 | 6235784 | 94305556 |
| ENSSSCG00000002755 | 6 | 17313388 | 17434926 | Meat color a* QTL (3924) | 6 | 6235784 | 94305556 |
| ENSSSCG00000002755 | 6 | 17313388 | 17434926 | Body weight (46 days) QTL (21240) | 6 | 8029639 | 91486640 |
| ENSSSCG00000002755 | 6 | 17313388 | 17434926 | Body weight (end of test) QTL (5201) | 6 | 12044459 | 1.35E+08 |
| ENSSSCG00000002799 | 6 | 20271596 | 20372280 | Backfat at last rib QTL (5982) | 6 | 6235784 | 53644827 |
| ENSSSCG00000002799 | 6 | 20271596 | 20372280 | Subcutanous fat area QTL (5983) | 6 | 6235784 | 53644827 |
| ENSSSCG00000002799 | 6 | 20271596 | 20372280 | Estimated carcass lean content QTL (5986) | 6 | 6235784 | 53644827 |
| ENSSSCG00000002799 | 6 | 20271596 | 20372280 | Conductivity 24 hours postmortem (loin) QTL (6004) | 6 | 6235784 | 53644827 |
| ENSSSCG00000002799 | 6 | 20271596 | 20372280 | Drip loss QTL (6005) | 6 | 6235784 | 53644827 |
| ENSSSCG00000002799 | 6 | 20271596 | 20372280 | Loin muscle area QTL (5698) | 6 | 6235784 | 53644827 |
| ENSSSCG00000002799 | 6 | 20271596 | 20372280 | Average daily gain QTL (5699) | 6 | 6235784 | 53644827 |
| ENSSSCG00000002799 | 6 | 20271596 | 20372280 | Lauric acid content QTL (156933) | 6 | 6235784 | 40233620 |
| ENSSSCG00000002799 | 6 | 20271596 | 20372280 | CO2 partial pressure QTL (6329) | 6 | 6235784 | 40233620 |
| ENSSSCG00000002799 | 6 | 20271596 | 20372280 | Adipocyte diameter QTL (18022) | 6 | 6235784 | 40233620 |
| ENSSSCG00000002799 | 6 | 20271596 | 20372280 | Intramuscular fat content QTL (367) | 6 | 8115061 | 35450005 |
| ENSSSCG00000002799 | 6 | 20271596 | 20372280 | Backfat at tenth rib QTL (3859) | 6 | 8115061 | 35450005 |
| ENSSSCG00000002799 | 6 | 20271596 | 20372280 | Inside ham weight QTL (3063) | 6 | 8115061 | 24943136 |
| ENSSSCG00000002799 | 6 | 20271596 | 20372280 | Relative area of type IIa fibers QTL (4029) | 6 | 10677712 | 25113979 |
| ENSSSCG00000002799 | 6 | 20271596 | 20372280 | Backfat at tenth rib QTL (3874) | 6 | 11959038 | 58684707 |
| ENSSSCG00000002799 | 6 | 20271596 | 20372280 | Meat color a* QTL (4037) | 6 | 12129881 | 33826993 |
| ENSSSCG00000002799 | 6 | 20271596 | 20372280 | Relative area of type IIb fibers QTL (4033) | 6 | 14436267 | 24943136 |
| ENSSSCG00000002799 | 6 | 20271596 | 20372280 | Average daily gain QTL (332) | 6 | 18280243 | 40233620 |
| ENSSSCG00000002799 | 6 | 20271596 | 20372280 | Average daily gain QTL (343) | 6 | 18280243 | 40233620 |
| ENSSSCG00000002799 | 6 | 20271596 | 20372280 | Meat color-a QTL (3236) | 6 | 18280243 | 39208560 |
| ENSSSCG00000002799 | 6 | 20271596 | 20372280 | Marbling QTL (38089) | 6 | 6235784 | 1.28E+08 |
| ENSSSCG00000002799 | 6 | 20271596 | 20372280 | Meat color a* QTL (3921) | 6 | 6235784 | 94305556 |
| ENSSSCG00000002799 | 6 | 20271596 | 20372280 | Meat color a* QTL (3922) | 6 | 6235784 | 94305556 |
| ENSSSCG00000002799 | 6 | 20271596 | 20372280 | Meat color a* QTL (3923) | 6 | 6235784 | 94305556 |
| ENSSSCG00000002799 | 6 | 20271596 | 20372280 | Meat color a* QTL (3924) | 6 | 6235784 | 94305556 |
| ENSSSCG00000002799 | 6 | 20271596 | 20372280 | Body weight (46 days) QTL (21240) | 6 | 8029639 | 91486640 |
| ENSSSCG00000002799 | 6 | 20271596 | 20372280 | Body weight (end of test) QTL (5201) | 6 | 12044459 | 1.35E+08 |
| ENSSSCG00000002799 | 6 | 20271596 | 20372280 | cis-9-Heptadecenoic acid content QTL (156934) | 6 | 18280243 | 79271337 |
| ENSSSCG00000002799 | 6 | 20271596 | 20372280 | Loin muscle area QTL (38054) | 6 | 18280243 | 79271337 |
| ENSSSCG00000033591 | 6 | 31830505 | 32013734 | Backfat at last rib QTL (5982) | 6 | 6235784 | 53644827 |
| ENSSSCG00000033591 | 6 | 31830505 | 32013734 | Subcutanous fat area QTL (5983) | 6 | 6235784 | 53644827 |
| ENSSSCG00000033591 | 6 | 31830505 | 32013734 | Estimated carcass lean content QTL (5986) | 6 | 6235784 | 53644827 |
| ENSSSCG00000033591 | 6 | 31830505 | 32013734 | Conductivity 24 hours postmortem (loin) QTL (6004) | 6 | 6235784 | 53644827 |
| ENSSSCG00000033591 | 6 | 31830505 | 32013734 | Drip loss QTL (6005) | 6 | 6235784 | 53644827 |
| ENSSSCG00000033591 | 6 | 31830505 | 32013734 | Loin muscle area QTL (5698) | 6 | 6235784 | 53644827 |
| ENSSSCG00000033591 | 6 | 31830505 | 32013734 | Average daily gain QTL (5699) | 6 | 6235784 | 53644827 |
| ENSSSCG00000033591 | 6 | 31830505 | 32013734 | Lauric acid content QTL (156933) | 6 | 6235784 | 40233620 |
| ENSSSCG00000033591 | 6 | 31830505 | 32013734 | CO2 partial pressure QTL (6329) | 6 | 6235784 | 40233620 |
| ENSSSCG00000033591 | 6 | 31830505 | 32013734 | Adipocyte diameter QTL (18022) | 6 | 6235784 | 40233620 |
| ENSSSCG00000033591 | 6 | 31830505 | 32013734 | Intramuscular fat content QTL (367) | 6 | 8115061 | 35450005 |
| ENSSSCG00000033591 | 6 | 31830505 | 32013734 | Backfat at tenth rib QTL (3859) | 6 | 8115061 | 35450005 |
| ENSSSCG00000033591 | 6 | 31830505 | 32013734 | Backfat at tenth rib QTL (3874) | 6 | 11959038 | 58684707 |
| ENSSSCG00000033591 | 6 | 31830505 | 32013734 | Meat color a* QTL (4037) | 6 | 12129881 | 33826993 |
| ENSSSCG00000033591 | 6 | 31830505 | 32013734 | Average daily gain QTL (332) | 6 | 18280243 | 40233620 |
| ENSSSCG00000033591 | 6 | 31830505 | 32013734 | Average daily gain QTL (343) | 6 | 18280243 | 40233620 |
| ENSSSCG00000033591 | 6 | 31830505 | 32013734 | Meat color-a QTL (3236) | 6 | 18280243 | 39208560 |
| ENSSSCG00000033591 | 6 | 31830505 | 32013734 | Outside ham weight QTL (3062) | 6 | 24943136 | 60991093 |
| ENSSSCG00000033591 | 6 | 31830505 | 32013734 | Marbling QTL (3065) | 6 | 24943136 | 60991093 |
| ENSSSCG00000033591 | 6 | 31830505 | 32013734 | Pseudorabies susceptibility QTL (304) | 6 | 24943136 | 40233620 |
| ENSSSCG00000033591 | 6 | 31830505 | 32013734 | subjective pork odor QTL (4224) | 6 | 24943136 | 40233620 |
| ENSSSCG00000033591 | 6 | 31830505 | 32013734 | Muscle moisture percentage QTL (5546) | 6 | 30922655 | 60393141 |
| ENSSSCG00000033591 | 6 | 31830505 | 32013734 | Marbling QTL (38089) | 6 | 6235784 | 1.28E+08 |
| ENSSSCG00000033591 | 6 | 31830505 | 32013734 | Meat color a* QTL (3921) | 6 | 6235784 | 94305556 |
| ENSSSCG00000033591 | 6 | 31830505 | 32013734 | Meat color a* QTL (3922) | 6 | 6235784 | 94305556 |
| ENSSSCG00000033591 | 6 | 31830505 | 32013734 | Meat color a* QTL (3923) | 6 | 6235784 | 94305556 |
| ENSSSCG00000033591 | 6 | 31830505 | 32013734 | Meat color a* QTL (3924) | 6 | 6235784 | 94305556 |
| ENSSSCG00000033591 | 6 | 31830505 | 32013734 | Body weight (46 days) QTL (21240) | 6 | 8029639 | 91486640 |
| ENSSSCG00000033591 | 6 | 31830505 | 32013734 | Body weight (end of test) QTL (5201) | 6 | 12044459 | 1.35E+08 |
| ENSSSCG00000033591 | 6 | 31830505 | 32013734 | cis-9-Heptadecenoic acid content QTL (156934) | 6 | 18280243 | 79271337 |
| ENSSSCG00000033591 | 6 | 31830505 | 32013734 | Loin muscle area QTL (38054) | 6 | 18280243 | 79271337 |
| ENSSSCG00000002844 | 6 | 36991241 | 37193587 | Backfat at last rib QTL (5982) | 6 | 6235784 | 53644827 |
| ENSSSCG00000002844 | 6 | 36991241 | 37193587 | Subcutanous fat area QTL (5983) | 6 | 6235784 | 53644827 |
| ENSSSCG00000002844 | 6 | 36991241 | 37193587 | Estimated carcass lean content QTL (5986) | 6 | 6235784 | 53644827 |
| ENSSSCG00000002844 | 6 | 36991241 | 37193587 | Conductivity 24 hours postmortem (loin) QTL (6004) | 6 | 6235784 | 53644827 |
| ENSSSCG00000002844 | 6 | 36991241 | 37193587 | Drip loss QTL (6005) | 6 | 6235784 | 53644827 |
| ENSSSCG00000002844 | 6 | 36991241 | 37193587 | Loin muscle area QTL (5698) | 6 | 6235784 | 53644827 |
| ENSSSCG00000002844 | 6 | 36991241 | 37193587 | Average daily gain QTL (5699) | 6 | 6235784 | 53644827 |
| ENSSSCG00000002844 | 6 | 36991241 | 37193587 | Lauric acid content QTL (156933) | 6 | 6235784 | 40233620 |
| ENSSSCG00000002844 | 6 | 36991241 | 37193587 | CO2 partial pressure QTL (6329) | 6 | 6235784 | 40233620 |
| ENSSSCG00000002844 | 6 | 36991241 | 37193587 | Adipocyte diameter QTL (18022) | 6 | 6235784 | 40233620 |
| ENSSSCG00000002844 | 6 | 36991241 | 37193587 | Backfat at tenth rib QTL (3874) | 6 | 11959038 | 58684707 |
| ENSSSCG00000002844 | 6 | 36991241 | 37193587 | Average daily gain QTL (332) | 6 | 18280243 | 40233620 |
| ENSSSCG00000002844 | 6 | 36991241 | 37193587 | Average daily gain QTL (343) | 6 | 18280243 | 40233620 |
| ENSSSCG00000002844 | 6 | 36991241 | 37193587 | Meat color-a QTL (3236) | 6 | 18280243 | 39208560 |
| ENSSSCG00000002844 | 6 | 36991241 | 37193587 | Outside ham weight QTL (3062) | 6 | 24943136 | 60991093 |
| ENSSSCG00000002844 | 6 | 36991241 | 37193587 | Marbling QTL (3065) | 6 | 24943136 | 60991093 |
| ENSSSCG00000002844 | 6 | 36991241 | 37193587 | Pseudorabies susceptibility QTL (304) | 6 | 24943136 | 40233620 |
| ENSSSCG00000002844 | 6 | 36991241 | 37193587 | subjective pork odor QTL (4224) | 6 | 24943136 | 40233620 |
| ENSSSCG00000002844 | 6 | 36991241 | 37193587 | Muscle moisture percentage QTL (5546) | 6 | 30922655 | 60393141 |
| ENSSSCG00000002844 | 6 | 36991241 | 37193587 | Palmitic acid content QTL (15878) | 6 | 32033137 | 40319042 |
| ENSSSCG00000002844 | 6 | 36991241 | 37193587 | Leaf fat weight QTL (3858) | 6 | 35450005 | 60991093 |
| ENSSSCG00000002844 | 6 | 36991241 | 37193587 | Empty body protein content QTL (2873) | 6 | 35450005 | 53644827 |
| ENSSSCG00000002844 | 6 | 36991241 | 37193587 | Empty body protein content QTL (2875) | 6 | 35450005 | 53644827 |
| ENSSSCG00000002844 | 6 | 36991241 | 37193587 | Empty body lipid content QTL (2877) | 6 | 35450005 | 53644827 |
| ENSSSCG00000002844 | 6 | 36991241 | 37193587 | Shoulder subcutaneous fat thickness QTL (5498) | 6 | 35450005 | 43992175 |
| ENSSSCG00000002844 | 6 | 36991241 | 37193587 | Marbling QTL (38089) | 6 | 6235784 | 1.28E+08 |
| ENSSSCG00000002844 | 6 | 36991241 | 37193587 | Meat color a* QTL (3921) | 6 | 6235784 | 94305556 |
| ENSSSCG00000002844 | 6 | 36991241 | 37193587 | Meat color a* QTL (3922) | 6 | 6235784 | 94305556 |
| ENSSSCG00000002844 | 6 | 36991241 | 37193587 | Meat color a* QTL (3923) | 6 | 6235784 | 94305556 |
| ENSSSCG00000002844 | 6 | 36991241 | 37193587 | Meat color a* QTL (3924) | 6 | 6235784 | 94305556 |
| ENSSSCG00000002844 | 6 | 36991241 | 37193587 | Body weight (46 days) QTL (21240) | 6 | 8029639 | 91486640 |
| ENSSSCG00000002844 | 6 | 36991241 | 37193587 | Body weight (end of test) QTL (5201) | 6 | 12044459 | 1.35E+08 |
| ENSSSCG00000002844 | 6 | 36991241 | 37193587 | cis-9-Heptadecenoic acid content QTL (156934) | 6 | 18280243 | 79271337 |
| ENSSSCG00000002844 | 6 | 36991241 | 37193587 | Loin muscle area QTL (38054) | 6 | 18280243 | 79271337 |
| ENSSSCG00000002844 | 6 | 36991241 | 37193587 | Alkaline phosphatase activity QTL (15097) | 6 | 35450005 | 1.38E+08 |
| ENSSSCG00000002844 | 6 | 36991241 | 37193587 | Loin fat percentage QTL (38090) | 6 | 35450005 | 1.28E+08 |
| ENSSSCG00000002875 | 6 | 44074125 | 44097065 | Backfat at last rib QTL (5982) | 6 | 6235784 | 53644827 |
| ENSSSCG00000002875 | 6 | 44074125 | 44097065 | Subcutanous fat area QTL (5983) | 6 | 6235784 | 53644827 |
| ENSSSCG00000002875 | 6 | 44074125 | 44097065 | Estimated carcass lean content QTL (5986) | 6 | 6235784 | 53644827 |
| ENSSSCG00000002875 | 6 | 44074125 | 44097065 | Conductivity 24 hours postmortem (loin) QTL (6004) | 6 | 6235784 | 53644827 |
| ENSSSCG00000002875 | 6 | 44074125 | 44097065 | Drip loss QTL (6005) | 6 | 6235784 | 53644827 |
| ENSSSCG00000002875 | 6 | 44074125 | 44097065 | Loin muscle area QTL (5698) | 6 | 6235784 | 53644827 |
| ENSSSCG00000002875 | 6 | 44074125 | 44097065 | Average daily gain QTL (5699) | 6 | 6235784 | 53644827 |
| ENSSSCG00000002875 | 6 | 44074125 | 44097065 | Backfat at tenth rib QTL (3874) | 6 | 11959038 | 58684707 |
| ENSSSCG00000002875 | 6 | 44074125 | 44097065 | Outside ham weight QTL (3062) | 6 | 24943136 | 60991093 |
| ENSSSCG00000002875 | 6 | 44074125 | 44097065 | Marbling QTL (3065) | 6 | 24943136 | 60991093 |
| ENSSSCG00000002875 | 6 | 44074125 | 44097065 | Muscle moisture percentage QTL (5546) | 6 | 30922655 | 60393141 |
| ENSSSCG00000002875 | 6 | 44074125 | 44097065 | Leaf fat weight QTL (3858) | 6 | 35450005 | 60991093 |
| ENSSSCG00000002875 | 6 | 44074125 | 44097065 | Empty body protein content QTL (2873) | 6 | 35450005 | 53644827 |
| ENSSSCG00000002875 | 6 | 44074125 | 44097065 | Empty body protein content QTL (2875) | 6 | 35450005 | 53644827 |
| ENSSSCG00000002875 | 6 | 44074125 | 44097065 | Empty body lipid content QTL (2877) | 6 | 35450005 | 53644827 |
| ENSSSCG00000002875 | 6 | 44074125 | 44097065 | Shoulder weight, picnic QTL (21439) | 6 | 39208560 | 53644827 |
| ENSSSCG00000002875 | 6 | 44074125 | 44097065 | Shoulder subcutaneous fat thickness QTL (1044) | 6 | 40233620 | 53644827 |
| ENSSSCG00000002875 | 6 | 44074125 | 44097065 | Backfat at first rib QTL (4965) | 6 | 40233620 | 53644827 |
| ENSSSCG00000002875 | 6 | 44074125 | 44097065 | Meat color a* QTL (17798) | 6 | 41942054 | 60991093 |
| ENSSSCG00000002875 | 6 | 44074125 | 44097065 | Meat color b* QTL (17799) | 6 | 41942054 | 60991093 |
| ENSSSCG00000002875 | 6 | 44074125 | 44097065 | Average backfat thickness QTL (3876) | 6 | 42454584 | 57745069 |
| ENSSSCG00000002875 | 6 | 44074125 | 44097065 | pH 24 hr post mortem (ham) QTL (2944) | 6 | 43992175 | 55524105 |
| ENSSSCG00000002875 | 6 | 44074125 | 44097065 | Skin percentage QTL (5504) | 6 | 43992175 | 55524105 |
| ENSSSCG00000002875 | 6 | 44074125 | 44097065 | Loin muscle area QTL (9855) | 6 | 43992175 | 55524105 |
| ENSSSCG00000002875 | 6 | 44074125 | 44097065 | Loin muscle area QTL (9856) | 6 | 43992175 | 55524105 |
| ENSSSCG00000002875 | 6 | 44074125 | 44097065 | Marbling QTL (38089) | 6 | 6235784 | 1.28E+08 |
| ENSSSCG00000002875 | 6 | 44074125 | 44097065 | Meat color a* QTL (3921) | 6 | 6235784 | 94305556 |
| ENSSSCG00000002875 | 6 | 44074125 | 44097065 | Meat color a* QTL (3922) | 6 | 6235784 | 94305556 |
| ENSSSCG00000002875 | 6 | 44074125 | 44097065 | Meat color a* QTL (3923) | 6 | 6235784 | 94305556 |
| ENSSSCG00000002875 | 6 | 44074125 | 44097065 | Meat color a* QTL (3924) | 6 | 6235784 | 94305556 |
| ENSSSCG00000002875 | 6 | 44074125 | 44097065 | Body weight (46 days) QTL (21240) | 6 | 8029639 | 91486640 |
| ENSSSCG00000002875 | 6 | 44074125 | 44097065 | Body weight (end of test) QTL (5201) | 6 | 12044459 | 1.35E+08 |
| ENSSSCG00000002875 | 6 | 44074125 | 44097065 | cis-9-Heptadecenoic acid content QTL (156934) | 6 | 18280243 | 79271337 |
| ENSSSCG00000002875 | 6 | 44074125 | 44097065 | Loin muscle area QTL (38054) | 6 | 18280243 | 79271337 |
| ENSSSCG00000002875 | 6 | 44074125 | 44097065 | Alkaline phosphatase activity QTL (15097) | 6 | 35450005 | 1.38E+08 |
| ENSSSCG00000002875 | 6 | 44074125 | 44097065 | Loin fat percentage QTL (38090) | 6 | 35450005 | 1.28E+08 |
| ENSSSCG00000002875 | 6 | 44074125 | 44097065 | Lactate dehydrogenase level QTL (15099) | 6 | 37585548 | 1.2E+08 |
| ENSSSCG00000002875 | 6 | 44074125 | 44097065 | Carcass length QTL (3873) | 6 | 38610608 | 1.2E+08 |
| ENSSSCG00000002875 | 6 | 44074125 | 44097065 | Drip loss QTL (218) | 6 | 40233620 | 75171096 |
| ENSSSCG00000002875 | 6 | 44074125 | 44097065 | Average daily gain QTL (3937) | 6 | 42702307 | 80210976 |
| ENSSSCG00000003401 | 6 | 70495144 | 70663463 | Overall impression, sensory panel QTL (5566) | 6 | 67363552 | 86865326 |
| ENSSSCG00000003401 | 6 | 70495144 | 70663463 | Average backfat thickness QTL (12859) | 6 | 68508203 | 89863628 |
| ENSSSCG00000003401 | 6 | 70495144 | 70663463 | Muscle moisture percentage QTL (12046) | 6 | 68508203 | 89863628 |
| ENSSSCG00000003401 | 6 | 70495144 | 70663463 | muscle protein percentage QTL (12047) | 6 | 68508203 | 89863628 |
| ENSSSCG00000003401 | 6 | 70495144 | 70663463 | Cholesterol level in meat QTL (12048) | 6 | 68508203 | 89863628 |
| ENSSSCG00000003401 | 6 | 70495144 | 70663463 | Muscle moisture percentage QTL (12050) | 6 | 68508203 | 89863628 |
| ENSSSCG00000003401 | 6 | 70495144 | 70663463 | Flavor score QTL (12052) | 6 | 68508203 | 89863628 |
| ENSSSCG00000003401 | 6 | 70495144 | 70663463 | Muscle moisture percentage QTL (12053) | 6 | 68508203 | 89863628 |
| ENSSSCG00000003401 | 6 | 70495144 | 70663463 | Marbling QTL (12054) | 6 | 68508203 | 89863628 |
| ENSSSCG00000003401 | 6 | 70495144 | 70663463 | Meat color score QTL (12055) | 6 | 68508203 | 89863628 |
| ENSSSCG00000003401 | 6 | 70495144 | 70663463 | Flavor score QTL (12056) | 6 | 68508203 | 89863628 |
| ENSSSCG00000003401 | 6 | 70495144 | 70663463 | Liver and gallbladder weight QTL (8660) | 6 | 68508203 | 80210976 |
| ENSSSCG00000003401 | 6 | 70495144 | 70663463 | Marbling QTL (38089) | 6 | 6235784 | 1.28E+08 |
| ENSSSCG00000003401 | 6 | 70495144 | 70663463 | Meat color a* QTL (3921) | 6 | 6235784 | 94305556 |
| ENSSSCG00000003401 | 6 | 70495144 | 70663463 | Meat color a* QTL (3922) | 6 | 6235784 | 94305556 |
| ENSSSCG00000003401 | 6 | 70495144 | 70663463 | Meat color a* QTL (3923) | 6 | 6235784 | 94305556 |
| ENSSSCG00000003401 | 6 | 70495144 | 70663463 | Meat color a* QTL (3924) | 6 | 6235784 | 94305556 |
| ENSSSCG00000003401 | 6 | 70495144 | 70663463 | Body weight (46 days) QTL (21240) | 6 | 8029639 | 91486640 |
| ENSSSCG00000003401 | 6 | 70495144 | 70663463 | Body weight (end of test) QTL (5201) | 6 | 12044459 | 1.35E+08 |
| ENSSSCG00000003401 | 6 | 70495144 | 70663463 | cis-9-Heptadecenoic acid content QTL (156934) | 6 | 18280243 | 79271337 |
| ENSSSCG00000003401 | 6 | 70495144 | 70663463 | Loin muscle area QTL (38054) | 6 | 18280243 | 79271337 |
| ENSSSCG00000003401 | 6 | 70495144 | 70663463 | Alkaline phosphatase activity QTL (15097) | 6 | 35450005 | 1.38E+08 |
| ENSSSCG00000003401 | 6 | 70495144 | 70663463 | Loin fat percentage QTL (38090) | 6 | 35450005 | 1.28E+08 |
| ENSSSCG00000003401 | 6 | 70495144 | 70663463 | Lactate dehydrogenase level QTL (15099) | 6 | 37585548 | 1.2E+08 |
| ENSSSCG00000003401 | 6 | 70495144 | 70663463 | Carcass length QTL (3873) | 6 | 38610608 | 1.2E+08 |
| ENSSSCG00000003401 | 6 | 70495144 | 70663463 | Drip loss QTL (218) | 6 | 40233620 | 75171096 |
| ENSSSCG00000003401 | 6 | 70495144 | 70663463 | Average daily gain QTL (3937) | 6 | 42702307 | 80210976 |
| ENSSSCG00000003401 | 6 | 70495144 | 70663463 | Average daily gain QTL (186) | 6 | 46981935 | 98234955 |
| ENSSSCG00000003401 | 6 | 70495144 | 70663463 | Backfat at last rib QTL (17800) | 6 | 49971694 | 73889770 |
| ENSSSCG00000003401 | 6 | 70495144 | 70663463 | Shear force QTL (38091) | 6 | 50398803 | 1.38E+08 |
| ENSSSCG00000003401 | 6 | 70495144 | 70663463 | Oleic acid content QTL (156935) | 6 | 50398803 | 88496881 |
| ENSSSCG00000003401 | 6 | 70495144 | 70663463 | Monounsaturated fatty acid content QTL (156937) | 6 | 50398803 | 88496881 |
| ENSSSCG00000003401 | 6 | 70495144 | 70663463 | Arachidonic acid content QTL (156871) | 6 | 50398803 | 79271337 |
| ENSSSCG00000003401 | 6 | 70495144 | 70663463 | Polyunsaturated fatty acid content QTL (156938) | 6 | 50398803 | 79271337 |
| ENSSSCG00000003401 | 6 | 70495144 | 70663463 | Linoleic acid content QTL (156936) | 6 | 50398803 | 71156276 |
| ENSSSCG00000003401 | 6 | 70495144 | 70663463 | Average backfat thickness QTL (1004) | 6 | 53644827 | 95672304 |
| ENSSSCG00000003401 | 6 | 70495144 | 70663463 | Abdominal fat weight QTL (1007) | 6 | 53644827 | 95672304 |
| ENSSSCG00000003401 | 6 | 70495144 | 70663463 | Backfat at tenth rib QTL (1011) | 6 | 53644827 | 95672304 |
| ENSSSCG00000003401 | 6 | 70495144 | 70663463 | Carcass weight (cold) QTL (1028) | 6 | 53644827 | 95672304 |
| ENSSSCG00000003401 | 6 | 70495144 | 70663463 | Body weight (slaughter) QTL (1031) | 6 | 53644827 | 95672304 |
| ENSSSCG00000003401 | 6 | 70495144 | 70663463 | Body weight (slaughter) QTL (1035) | 6 | 53644827 | 95672304 |
| ENSSSCG00000003401 | 6 | 70495144 | 70663463 | Creatine kinase level QTL (1045) | 6 | 53644827 | 95672304 |
| ENSSSCG00000003401 | 6 | 70495144 | 70663463 | Carcass weight (hot) QTL (16866) | 6 | 53644827 | 90632423 |
| ENSSSCG00000003401 | 6 | 70495144 | 70663463 | Conductivity 24 hours post-mortem QTL (1013) | 6 | 53644827 | 89692785 |
| ENSSSCG00000003401 | 6 | 70495144 | 70663463 | Smell intensity QTL (688) | 6 | 53644827 | 87130134 |
| ENSSSCG00000003401 | 6 | 70495144 | 70663463 | Muscle moisture percentage QTL (21366) | 6 | 53644827 | 75171096 |
| ENSSSCG00000003401 | 6 | 70495144 | 70663463 | Meat color score QTL (3237) | 6 | 53644827 | 71156276 |
| ENSSSCG00000003401 | 6 | 70495144 | 70663463 | Shear force QTL (3238) | 6 | 53644827 | 71156276 |
| ENSSSCG00000003401 | 6 | 70495144 | 70663463 | Average daily gain QTL (16850) | 6 | 53644827 | 70985432 |
| ENSSSCG00000003401 | 6 | 70495144 | 70663463 | Average backfat thickness QTL (148) | 6 | 55524105 | 1.33E+08 |
| ENSSSCG00000003401 | 6 | 70495144 | 70663463 | Average backfat thickness QTL (149) | 6 | 55524105 | 1.33E+08 |
| ENSSSCG00000003401 | 6 | 70495144 | 70663463 | Intramuscular fat content QTL (150) | 6 | 55524105 | 1.33E+08 |
| ENSSSCG00000003401 | 6 | 70495144 | 70663463 | Intramuscular fat content QTL (151) | 6 | 55524105 | 1.33E+08 |
| ENSSSCG00000003401 | 6 | 70495144 | 70663463 | Alkaline phosphatase activity QTL (6347) | 6 | 58684707 | 94305556 |
| ENSSSCG00000003401 | 6 | 70495144 | 70663463 | Calcium level QTL (6376) | 6 | 58684707 | 94305556 |
| ENSSSCG00000003401 | 6 | 70495144 | 70663463 | Loin muscle area QTL (38053) | 6 | 58684707 | 71156276 |
| ENSSSCG00000003401 | 6 | 70495144 | 70663463 | Backfat between 3rd and 4th last ribs QTL (4167) | 6 | 59368081 | 1.24E+08 |
| ENSSSCG00000003401 | 6 | 70495144 | 70663463 | Conductivity 24 hours post-mortem QTL (1245) | 6 | 59795190 | 82004832 |
| ENSSSCG00000003401 | 6 | 70495144 | 70663463 | Conductivity 24 hours post-mortem QTL (1247) | 6 | 59795190 | 82004832 |
| ENSSSCG00000003401 | 6 | 70495144 | 70663463 | Average backfat thickness QTL (680) | 6 | 60649407 | 92255436 |
| ENSSSCG00000003401 | 6 | 70495144 | 70663463 | Body weight QTL (7552) | 6 | 61050888 | 78007096 |
| ENSSSCG00000003401 | 6 | 70495144 | 70663463 | Adipocyte diameter QTL (18030) | 6 | 61418202 | 77990012 |
| ENSSSCG00000003401 | 6 | 70495144 | 70663463 | Backfat at last rib QTL (682) | 6 | 61503624 | 79442181 |
| ENSSSCG00000003401 | 6 | 70495144 | 70663463 | body weight (17 weeks) QTL (184) | 6 | 61759889 | 1.15E+08 |
| ENSSSCG00000003401 | 6 | 70495144 | 70663463 | body weight (13 weeks) QTL (183) | 6 | 61759889 | 1.07E+08 |
| ENSSSCG00000003401 | 6 | 70495144 | 70663463 | Average daily gain QTL (185) | 6 | 61759889 | 97209894 |
| ENSSSCG00000003401 | 6 | 70495144 | 70663463 | Intramuscular fat content QTL (4169) | 6 | 61930732 | 1.26E+08 |
| ENSSSCG00000003401 | 6 | 70495144 | 70663463 | Conductivity 24 hours post-mortem QTL (988) | 6 | 65005913 | 95672304 |
| ENSSSCG00000003401 | 6 | 70495144 | 70663463 | Loin muscle area QTL (1006) | 6 | 65005913 | 95672304 |
| ENSSSCG00000003401 | 6 | 70495144 | 70663463 | Meat color score QTL (21854) | 6 | 65005913 | 89692785 |
| ENSSSCG00000003401 | 6 | 70495144 | 70663463 | Meat color-a QTL (991) | 6 | 65005913 | 89692785 |
| ENSSSCG00000003401 | 6 | 70495144 | 70663463 | Conductivity 24 hours post-mortem QTL (992) | 6 | 65005913 | 89692785 |
| ENSSSCG00000003401 | 6 | 70495144 | 70663463 | pH 45 minutes post mortem QTL (994) | 6 | 65005913 | 89692785 |
| ENSSSCG00000003401 | 6 | 70495144 | 70663463 | pH 45 minutes post mortem QTL (1015) | 6 | 65005913 | 89692785 |
| ENSSSCG00000003401 | 6 | 70495144 | 70663463 | Creatine kinase level QTL (1041) | 6 | 65005913 | 89692785 |
| ENSSSCG00000003401 | 6 | 70495144 | 70663463 | Backfat at last rib QTL (16877) | 6 | 65005913 | 89692785 |
| ENSSSCG00000003401 | 6 | 70495144 | 70663463 | Conductivity 24 hours postmortem (loin) QTL (21858) | 6 | 65005913 | 75598204 |
| ENSSSCG00000003401 | 6 | 70495144 | 70663463 | pH 45 minutes post mortem QTL (21851) | 6 | 65005913 | 75598204 |
| ENSSSCG00000003401 | 6 | 70495144 | 70663463 | Body weight (20 weeks) QTL (29696) | 6 | 65347600 | 1.28E+08 |
| ENSSSCG00000003401 | 6 | 70495144 | 70663463 | Carcass weight (hot) QTL (29697) | 6 | 65347600 | 1.28E+08 |
| ENSSSCG00000003401 | 6 | 70495144 | 70663463 | subjective boar flavor in lean QTL (4219) | 6 | 65347600 | 1.28E+08 |
| ENSSSCG00000003401 | 6 | 70495144 | 70663463 | subjective pork flavor in fat QTL (4223) | 6 | 65347600 | 99089172 |
| ENSSSCG00000003401 | 6 | 70495144 | 70663463 | androstenone, laboratory QTL (4215) | 6 | 65347600 | 77477481 |
| ENSSSCG00000003401 | 6 | 70495144 | 70663463 | PH for Longissmus dorsi QTL (3845) | 6 | 65945552 | 80210976 |
| ENSSSCG00000003401 | 6 | 70495144 | 70663463 | Water holding capacity QTL (3850) | 6 | 65945552 | 80210976 |
| ENSSSCG00000003461 | 6 | 74809349 | 74855427 | Lean meat weight QTL (16910) | 6 | 71156276 | 75171096 |
| ENSSSCG00000003461 | 6 | 74809349 | 74855427 | Empty body protein content QTL (16911) | 6 | 71156276 | 75171096 |
| ENSSSCG00000003461 | 6 | 74809349 | 74855427 | Overall impression, sensory panel QTL (5566) | 6 | 67363552 | 86865326 |
| ENSSSCG00000003461 | 6 | 74809349 | 74855427 | Average backfat thickness QTL (12859) | 6 | 68508203 | 89863628 |
| ENSSSCG00000003461 | 6 | 74809349 | 74855427 | Muscle moisture percentage QTL (12046) | 6 | 68508203 | 89863628 |
| ENSSSCG00000003461 | 6 | 74809349 | 74855427 | muscle protein percentage QTL (12047) | 6 | 68508203 | 89863628 |
| ENSSSCG00000003461 | 6 | 74809349 | 74855427 | Cholesterol level in meat QTL (12048) | 6 | 68508203 | 89863628 |
| ENSSSCG00000003461 | 6 | 74809349 | 74855427 | Muscle moisture percentage QTL (12050) | 6 | 68508203 | 89863628 |
| ENSSSCG00000003461 | 6 | 74809349 | 74855427 | Flavor score QTL (12052) | 6 | 68508203 | 89863628 |
| ENSSSCG00000003461 | 6 | 74809349 | 74855427 | Muscle moisture percentage QTL (12053) | 6 | 68508203 | 89863628 |
| ENSSSCG00000003461 | 6 | 74809349 | 74855427 | Marbling QTL (12054) | 6 | 68508203 | 89863628 |
| ENSSSCG00000003461 | 6 | 74809349 | 74855427 | Meat color score QTL (12055) | 6 | 68508203 | 89863628 |
| ENSSSCG00000003461 | 6 | 74809349 | 74855427 | Flavor score QTL (12056) | 6 | 68508203 | 89863628 |
| ENSSSCG00000003461 | 6 | 74809349 | 74855427 | Liver and gallbladder weight QTL (8660) | 6 | 68508203 | 80210976 |
| ENSSSCG00000003461 | 6 | 74809349 | 74855427 | Loin muscle area QTL (3190) | 6 | 71156276 | 1.03E+08 |
| ENSSSCG00000003461 | 6 | 74809349 | 74855427 | Loin muscle area QTL (3191) | 6 | 71156276 | 1.03E+08 |
| ENSSSCG00000003461 | 6 | 74809349 | 74855427 | Estimated carcass lean content QTL (3192) | 6 | 71156276 | 1.03E+08 |
| ENSSSCG00000003461 | 6 | 74809349 | 74855427 | Empty body protein content QTL (3193) | 6 | 71156276 | 1.03E+08 |
| ENSSSCG00000003461 | 6 | 74809349 | 74855427 | Backfat at last rib QTL (3194) | 6 | 71156276 | 1.03E+08 |
| ENSSSCG00000003461 | 6 | 74809349 | 74855427 | Marbling QTL (3239) | 6 | 71156276 | 1.03E+08 |
| ENSSSCG00000003461 | 6 | 74809349 | 74855427 | Muscle fat content QTL (3240) | 6 | 71156276 | 1.03E+08 |
| ENSSSCG00000003461 | 6 | 74809349 | 74855427 | Backfat at tenth rib QTL (3242) | 6 | 71156276 | 1.03E+08 |
| ENSSSCG00000003461 | 6 | 74809349 | 74855427 | Backfat at last lumbar QTL (3243) | 6 | 71156276 | 1.03E+08 |
| ENSSSCG00000003461 | 6 | 74809349 | 74855427 | Empty body lipid content QTL (3646) | 6 | 71156276 | 1.03E+08 |
| ENSSSCG00000003461 | 6 | 74809349 | 74855427 | Loin muscle area QTL (3648) | 6 | 71156276 | 1.03E+08 |
| ENSSSCG00000003461 | 6 | 74809349 | 74855427 | Backfat at last rib QTL (2852) | 6 | 71156276 | 89863628 |
| ENSSSCG00000003461 | 6 | 74809349 | 74855427 | Loin muscle area QTL (2863) | 6 | 71156276 | 89863628 |
| ENSSSCG00000003461 | 6 | 74809349 | 74855427 | Empty body protein content QTL (2874) | 6 | 71156276 | 89863628 |
| ENSSSCG00000003461 | 6 | 74809349 | 74855427 | Empty body protein content QTL (2876) | 6 | 71156276 | 89863628 |
| ENSSSCG00000003461 | 6 | 74809349 | 74855427 | Empty body lipid content QTL (2878) | 6 | 71156276 | 89863628 |
| ENSSSCG00000003461 | 6 | 74809349 | 74855427 | Intramuscular fat content QTL (163) | 6 | 71156276 | 89863628 |
| ENSSSCG00000003461 | 6 | 74809349 | 74855427 | Intramuscular fat content QTL (7231) | 6 | 71156276 | 77477481 |
| ENSSSCG00000003461 | 6 | 74809349 | 74855427 | Average backfat thickness QTL (690) | 6 | 71156276 | 77477481 |
| ENSSSCG00000003461 | 6 | 74809349 | 74855427 | Intramuscular fat content QTL (692) | 6 | 71156276 | 77477481 |
| ENSSSCG00000003461 | 6 | 74809349 | 74855427 | Marbling QTL (38089) | 6 | 6235784 | 1.28E+08 |
| ENSSSCG00000003461 | 6 | 74809349 | 74855427 | Meat color a* QTL (3921) | 6 | 6235784 | 94305556 |
| ENSSSCG00000003461 | 6 | 74809349 | 74855427 | Meat color a* QTL (3922) | 6 | 6235784 | 94305556 |
| ENSSSCG00000003461 | 6 | 74809349 | 74855427 | Meat color a* QTL (3923) | 6 | 6235784 | 94305556 |
| ENSSSCG00000003461 | 6 | 74809349 | 74855427 | Meat color a* QTL (3924) | 6 | 6235784 | 94305556 |
| ENSSSCG00000003461 | 6 | 74809349 | 74855427 | Body weight (46 days) QTL (21240) | 6 | 8029639 | 91486640 |
| ENSSSCG00000003461 | 6 | 74809349 | 74855427 | Body weight (end of test) QTL (5201) | 6 | 12044459 | 1.35E+08 |
| ENSSSCG00000003461 | 6 | 74809349 | 74855427 | cis-9-Heptadecenoic acid content QTL (156934) | 6 | 18280243 | 79271337 |
| ENSSSCG00000003461 | 6 | 74809349 | 74855427 | Loin muscle area QTL (38054) | 6 | 18280243 | 79271337 |
| ENSSSCG00000003461 | 6 | 74809349 | 74855427 | Alkaline phosphatase activity QTL (15097) | 6 | 35450005 | 1.38E+08 |
| ENSSSCG00000003461 | 6 | 74809349 | 74855427 | Loin fat percentage QTL (38090) | 6 | 35450005 | 1.28E+08 |
| ENSSSCG00000003461 | 6 | 74809349 | 74855427 | Lactate dehydrogenase level QTL (15099) | 6 | 37585548 | 1.2E+08 |
| ENSSSCG00000003461 | 6 | 74809349 | 74855427 | Carcass length QTL (3873) | 6 | 38610608 | 1.2E+08 |
| ENSSSCG00000003461 | 6 | 74809349 | 74855427 | Drip loss QTL (218) | 6 | 40233620 | 75171096 |
| ENSSSCG00000003461 | 6 | 74809349 | 74855427 | Average daily gain QTL (3937) | 6 | 42702307 | 80210976 |
| ENSSSCG00000003461 | 6 | 74809349 | 74855427 | Average daily gain QTL (186) | 6 | 46981935 | 98234955 |
| ENSSSCG00000003461 | 6 | 74809349 | 74855427 | Shear force QTL (38091) | 6 | 50398803 | 1.38E+08 |
| ENSSSCG00000003461 | 6 | 74809349 | 74855427 | Oleic acid content QTL (156935) | 6 | 50398803 | 88496881 |
| ENSSSCG00000003461 | 6 | 74809349 | 74855427 | Monounsaturated fatty acid content QTL (156937) | 6 | 50398803 | 88496881 |
| ENSSSCG00000003461 | 6 | 74809349 | 74855427 | Arachidonic acid content QTL (156871) | 6 | 50398803 | 79271337 |
| ENSSSCG00000003461 | 6 | 74809349 | 74855427 | Polyunsaturated fatty acid content QTL (156938) | 6 | 50398803 | 79271337 |
| ENSSSCG00000003461 | 6 | 74809349 | 74855427 | Average backfat thickness QTL (1004) | 6 | 53644827 | 95672304 |
| ENSSSCG00000003461 | 6 | 74809349 | 74855427 | Abdominal fat weight QTL (1007) | 6 | 53644827 | 95672304 |
| ENSSSCG00000003461 | 6 | 74809349 | 74855427 | Backfat at tenth rib QTL (1011) | 6 | 53644827 | 95672304 |
| ENSSSCG00000003461 | 6 | 74809349 | 74855427 | Carcass weight (cold) QTL (1028) | 6 | 53644827 | 95672304 |
| ENSSSCG00000003461 | 6 | 74809349 | 74855427 | Body weight (slaughter) QTL (1031) | 6 | 53644827 | 95672304 |
| ENSSSCG00000003461 | 6 | 74809349 | 74855427 | Body weight (slaughter) QTL (1035) | 6 | 53644827 | 95672304 |
| ENSSSCG00000003461 | 6 | 74809349 | 74855427 | Creatine kinase level QTL (1045) | 6 | 53644827 | 95672304 |
| ENSSSCG00000003461 | 6 | 74809349 | 74855427 | Carcass weight (hot) QTL (16866) | 6 | 53644827 | 90632423 |
| ENSSSCG00000003461 | 6 | 74809349 | 74855427 | Conductivity 24 hours post-mortem QTL (1013) | 6 | 53644827 | 89692785 |
| ENSSSCG00000003461 | 6 | 74809349 | 74855427 | Smell intensity QTL (688) | 6 | 53644827 | 87130134 |
| ENSSSCG00000003461 | 6 | 74809349 | 74855427 | Muscle moisture percentage QTL (21366) | 6 | 53644827 | 75171096 |
| ENSSSCG00000003461 | 6 | 74809349 | 74855427 | Average backfat thickness QTL (148) | 6 | 55524105 | 1.33E+08 |
| ENSSSCG00000003461 | 6 | 74809349 | 74855427 | Average backfat thickness QTL (149) | 6 | 55524105 | 1.33E+08 |
| ENSSSCG00000003461 | 6 | 74809349 | 74855427 | Intramuscular fat content QTL (150) | 6 | 55524105 | 1.33E+08 |
| ENSSSCG00000003461 | 6 | 74809349 | 74855427 | Intramuscular fat content QTL (151) | 6 | 55524105 | 1.33E+08 |
| ENSSSCG00000003461 | 6 | 74809349 | 74855427 | Alkaline phosphatase activity QTL (6347) | 6 | 58684707 | 94305556 |
| ENSSSCG00000003461 | 6 | 74809349 | 74855427 | Calcium level QTL (6376) | 6 | 58684707 | 94305556 |
| ENSSSCG00000003461 | 6 | 74809349 | 74855427 | Backfat between 3rd and 4th last ribs QTL (4167) | 6 | 59368081 | 1.24E+08 |
| ENSSSCG00000003461 | 6 | 74809349 | 74855427 | Conductivity 24 hours post-mortem QTL (1245) | 6 | 59795190 | 82004832 |
| ENSSSCG00000003461 | 6 | 74809349 | 74855427 | Conductivity 24 hours post-mortem QTL (1247) | 6 | 59795190 | 82004832 |
| ENSSSCG00000003461 | 6 | 74809349 | 74855427 | Average backfat thickness QTL (680) | 6 | 60649407 | 92255436 |
| ENSSSCG00000003461 | 6 | 74809349 | 74855427 | Body weight QTL (7552) | 6 | 61050888 | 78007096 |
| ENSSSCG00000003461 | 6 | 74809349 | 74855427 | Adipocyte diameter QTL (18030) | 6 | 61418202 | 77990012 |
| ENSSSCG00000003461 | 6 | 74809349 | 74855427 | Backfat at last rib QTL (682) | 6 | 61503624 | 79442181 |
| ENSSSCG00000003461 | 6 | 74809349 | 74855427 | body weight (17 weeks) QTL (184) | 6 | 61759889 | 1.15E+08 |
| ENSSSCG00000003461 | 6 | 74809349 | 74855427 | body weight (13 weeks) QTL (183) | 6 | 61759889 | 1.07E+08 |
| ENSSSCG00000003461 | 6 | 74809349 | 74855427 | Average daily gain QTL (185) | 6 | 61759889 | 97209894 |
| ENSSSCG00000003461 | 6 | 74809349 | 74855427 | Intramuscular fat content QTL (4169) | 6 | 61930732 | 1.26E+08 |
| ENSSSCG00000003461 | 6 | 74809349 | 74855427 | Conductivity 24 hours post-mortem QTL (988) | 6 | 65005913 | 95672304 |
| ENSSSCG00000003461 | 6 | 74809349 | 74855427 | Loin muscle area QTL (1006) | 6 | 65005913 | 95672304 |
| ENSSSCG00000003461 | 6 | 74809349 | 74855427 | Meat color score QTL (21854) | 6 | 65005913 | 89692785 |
| ENSSSCG00000003461 | 6 | 74809349 | 74855427 | Meat color-a QTL (991) | 6 | 65005913 | 89692785 |
| ENSSSCG00000003461 | 6 | 74809349 | 74855427 | Conductivity 24 hours post-mortem QTL (992) | 6 | 65005913 | 89692785 |
| ENSSSCG00000003461 | 6 | 74809349 | 74855427 | pH 45 minutes post mortem QTL (994) | 6 | 65005913 | 89692785 |
| ENSSSCG00000003461 | 6 | 74809349 | 74855427 | pH 45 minutes post mortem QTL (1015) | 6 | 65005913 | 89692785 |
| ENSSSCG00000003461 | 6 | 74809349 | 74855427 | Creatine kinase level QTL (1041) | 6 | 65005913 | 89692785 |
| ENSSSCG00000003461 | 6 | 74809349 | 74855427 | Backfat at last rib QTL (16877) | 6 | 65005913 | 89692785 |
| ENSSSCG00000003461 | 6 | 74809349 | 74855427 | Conductivity 24 hours postmortem (loin) QTL (21858) | 6 | 65005913 | 75598204 |
| ENSSSCG00000003461 | 6 | 74809349 | 74855427 | pH 45 minutes post mortem QTL (21851) | 6 | 65005913 | 75598204 |
| ENSSSCG00000003461 | 6 | 74809349 | 74855427 | Body weight (20 weeks) QTL (29696) | 6 | 65347600 | 1.28E+08 |
| ENSSSCG00000003461 | 6 | 74809349 | 74855427 | Carcass weight (hot) QTL (29697) | 6 | 65347600 | 1.28E+08 |
| ENSSSCG00000003461 | 6 | 74809349 | 74855427 | subjective boar flavor in lean QTL (4219) | 6 | 65347600 | 1.28E+08 |
| ENSSSCG00000003461 | 6 | 74809349 | 74855427 | subjective pork flavor in fat QTL (4223) | 6 | 65347600 | 99089172 |
| ENSSSCG00000003461 | 6 | 74809349 | 74855427 | androstenone, laboratory QTL (4215) | 6 | 65347600 | 77477481 |
| ENSSSCG00000003461 | 6 | 74809349 | 74855427 | PH for Longissmus dorsi QTL (3845) | 6 | 65945552 | 80210976 |
| ENSSSCG00000003461 | 6 | 74809349 | 74855427 | Water holding capacity QTL (3850) | 6 | 65945552 | 80210976 |
| ENSSSCG00000003531 | 6 | 80903496 | 80971595 | Overall impression, sensory panel QTL (5566) | 6 | 67363552 | 86865326 |
| ENSSSCG00000003531 | 6 | 80903496 | 80971595 | Average backfat thickness QTL (12859) | 6 | 68508203 | 89863628 |
| ENSSSCG00000003531 | 6 | 80903496 | 80971595 | Muscle moisture percentage QTL (12046) | 6 | 68508203 | 89863628 |
| ENSSSCG00000003531 | 6 | 80903496 | 80971595 | muscle protein percentage QTL (12047) | 6 | 68508203 | 89863628 |
| ENSSSCG00000003531 | 6 | 80903496 | 80971595 | Cholesterol level in meat QTL (12048) | 6 | 68508203 | 89863628 |
| ENSSSCG00000003531 | 6 | 80903496 | 80971595 | Muscle moisture percentage QTL (12050) | 6 | 68508203 | 89863628 |
| ENSSSCG00000003531 | 6 | 80903496 | 80971595 | Flavor score QTL (12052) | 6 | 68508203 | 89863628 |
| ENSSSCG00000003531 | 6 | 80903496 | 80971595 | Muscle moisture percentage QTL (12053) | 6 | 68508203 | 89863628 |
| ENSSSCG00000003531 | 6 | 80903496 | 80971595 | Marbling QTL (12054) | 6 | 68508203 | 89863628 |
| ENSSSCG00000003531 | 6 | 80903496 | 80971595 | Meat color score QTL (12055) | 6 | 68508203 | 89863628 |
| ENSSSCG00000003531 | 6 | 80903496 | 80971595 | Flavor score QTL (12056) | 6 | 68508203 | 89863628 |
| ENSSSCG00000003531 | 6 | 80903496 | 80971595 | Loin muscle area QTL (3190) | 6 | 71156276 | 1.03E+08 |
| ENSSSCG00000003531 | 6 | 80903496 | 80971595 | Loin muscle area QTL (3191) | 6 | 71156276 | 1.03E+08 |
| ENSSSCG00000003531 | 6 | 80903496 | 80971595 | Estimated carcass lean content QTL (3192) | 6 | 71156276 | 1.03E+08 |
| ENSSSCG00000003531 | 6 | 80903496 | 80971595 | Empty body protein content QTL (3193) | 6 | 71156276 | 1.03E+08 |
| ENSSSCG00000003531 | 6 | 80903496 | 80971595 | Backfat at last rib QTL (3194) | 6 | 71156276 | 1.03E+08 |
| ENSSSCG00000003531 | 6 | 80903496 | 80971595 | Marbling QTL (3239) | 6 | 71156276 | 1.03E+08 |
| ENSSSCG00000003531 | 6 | 80903496 | 80971595 | Muscle fat content QTL (3240) | 6 | 71156276 | 1.03E+08 |
| ENSSSCG00000003531 | 6 | 80903496 | 80971595 | Backfat at tenth rib QTL (3242) | 6 | 71156276 | 1.03E+08 |
| ENSSSCG00000003531 | 6 | 80903496 | 80971595 | Backfat at last lumbar QTL (3243) | 6 | 71156276 | 1.03E+08 |
| ENSSSCG00000003531 | 6 | 80903496 | 80971595 | Empty body lipid content QTL (3646) | 6 | 71156276 | 1.03E+08 |
| ENSSSCG00000003531 | 6 | 80903496 | 80971595 | Loin muscle area QTL (3648) | 6 | 71156276 | 1.03E+08 |
| ENSSSCG00000003531 | 6 | 80903496 | 80971595 | Backfat at last rib QTL (2852) | 6 | 71156276 | 89863628 |
| ENSSSCG00000003531 | 6 | 80903496 | 80971595 | Loin muscle area QTL (2863) | 6 | 71156276 | 89863628 |
| ENSSSCG00000003531 | 6 | 80903496 | 80971595 | Empty body protein content QTL (2874) | 6 | 71156276 | 89863628 |
| ENSSSCG00000003531 | 6 | 80903496 | 80971595 | Empty body protein content QTL (2876) | 6 | 71156276 | 89863628 |
| ENSSSCG00000003531 | 6 | 80903496 | 80971595 | Empty body lipid content QTL (2878) | 6 | 71156276 | 89863628 |
| ENSSSCG00000003531 | 6 | 80903496 | 80971595 | Intramuscular fat content QTL (163) | 6 | 71156276 | 89863628 |
| ENSSSCG00000003531 | 6 | 80903496 | 80971595 | Intramuscular fat content QTL (368) | 6 | 76281578 | 91401219 |
| ENSSSCG00000003531 | 6 | 80903496 | 80971595 | Average daily gain QTL (3674) | 6 | 76281578 | 87130134 |
| ENSSSCG00000003531 | 6 | 80903496 | 80971595 | Backfat at last rib QTL (3675) | 6 | 76281578 | 87130134 |
| ENSSSCG00000003531 | 6 | 80903496 | 80971595 | Carcass weight (hot) QTL (3696) | 6 | 76281578 | 87130134 |
| ENSSSCG00000003531 | 6 | 80903496 | 80971595 | Carcass length QTL (3697) | 6 | 76281578 | 87130134 |
| ENSSSCG00000003531 | 6 | 80903496 | 80971595 | PH for Longissmus dorsi QTL (3698) | 6 | 76281578 | 87130134 |
| ENSSSCG00000003531 | 6 | 80903496 | 80971595 | Average daily gain QTL (4131) | 6 | 76281578 | 87130134 |
| ENSSSCG00000003531 | 6 | 80903496 | 80971595 | Average daily gain QTL (4132) | 6 | 76281578 | 87130134 |
| ENSSSCG00000003531 | 6 | 80903496 | 80971595 | Carcass length QTL (4133) | 6 | 76281578 | 87130134 |
| ENSSSCG00000003531 | 6 | 80903496 | 80971595 | PH for Longissmus dorsi QTL (4135) | 6 | 76281578 | 87130134 |
| ENSSSCG00000003531 | 6 | 80903496 | 80971595 | PH for Longissmus dorsi QTL (4136) | 6 | 76281578 | 87130134 |
| ENSSSCG00000003531 | 6 | 80903496 | 80971595 | PH for Longissmus dorsi QTL (4139) | 6 | 76281578 | 87130134 |
| ENSSSCG00000003531 | 6 | 80903496 | 80971595 | Conductivity 24 hours postmortem (loin) QTL (4141) | 6 | 76281578 | 87130134 |
| ENSSSCG00000003531 | 6 | 80903496 | 80971595 | Conductivity 24 hours postmortem (loin) QTL (4193) | 6 | 76281578 | 87130134 |
| ENSSSCG00000003531 | 6 | 80903496 | 80971595 | Backfat at first rib QTL (681) | 6 | 76879530 | 92255436 |
| ENSSSCG00000003531 | 6 | 80903496 | 80971595 | Intramuscular fat content QTL (668) | 6 | 77477481 | 89863628 |
| ENSSSCG00000003531 | 6 | 80903496 | 80971595 | Conductivity 24 hours post-mortem QTL (1016) | 6 | 77990012 | 98234955 |
| ENSSSCG00000003531 | 6 | 80903496 | 80971595 | Meat color-a QTL (1018) | 6 | 77990012 | 98234955 |
| ENSSSCG00000003531 | 6 | 80903496 | 80971595 | pH 45 minutes post mortem QTL (1019) | 6 | 77990012 | 98234955 |
| ENSSSCG00000003531 | 6 | 80903496 | 80971595 | pH 24 hr post-mortem (loin) QTL (1030) | 6 | 77990012 | 98234955 |
| ENSSSCG00000003531 | 6 | 80903496 | 80971595 | Loin muscle area QTL (1034) | 6 | 77990012 | 98234955 |
| ENSSSCG00000003531 | 6 | 80903496 | 80971595 | pH 24 hr post-mortem (loin) QTL (990) | 6 | 77990012 | 89692785 |
| ENSSSCG00000003531 | 6 | 80903496 | 80971595 | Backfat at last rib QTL (16878) | 6 | 77990012 | 89692785 |
| ENSSSCG00000003531 | 6 | 80903496 | 80971595 | Interleukin-2 level QTL (17599) | 6 | 79271337 | 87130134 |
| ENSSSCG00000003531 | 6 | 80903496 | 80971595 | Cholesterol level QTL (17718) | 6 | 79271337 | 87130134 |
| ENSSSCG00000003531 | 6 | 80903496 | 80971595 | Meat color L* QTL (17556) | 6 | 79527602 | 96611942 |
| ENSSSCG00000003531 | 6 | 80903496 | 80971595 | Loin muscle area QTL (7298) | 6 | 80210976 | 99089172 |
| ENSSSCG00000003531 | 6 | 80903496 | 80971595 | Marbling QTL (38089) | 6 | 6235784 | 1.28E+08 |
| ENSSSCG00000003531 | 6 | 80903496 | 80971595 | Meat color a* QTL (3921) | 6 | 6235784 | 94305556 |
| ENSSSCG00000003531 | 6 | 80903496 | 80971595 | Meat color a* QTL (3922) | 6 | 6235784 | 94305556 |
| ENSSSCG00000003531 | 6 | 80903496 | 80971595 | Meat color a* QTL (3923) | 6 | 6235784 | 94305556 |
| ENSSSCG00000003531 | 6 | 80903496 | 80971595 | Meat color a* QTL (3924) | 6 | 6235784 | 94305556 |
| ENSSSCG00000003531 | 6 | 80903496 | 80971595 | Body weight (46 days) QTL (21240) | 6 | 8029639 | 91486640 |
| ENSSSCG00000003531 | 6 | 80903496 | 80971595 | Body weight (end of test) QTL (5201) | 6 | 12044459 | 1.35E+08 |
| ENSSSCG00000003531 | 6 | 80903496 | 80971595 | Alkaline phosphatase activity QTL (15097) | 6 | 35450005 | 1.38E+08 |
| ENSSSCG00000003531 | 6 | 80903496 | 80971595 | Loin fat percentage QTL (38090) | 6 | 35450005 | 1.28E+08 |
| ENSSSCG00000003531 | 6 | 80903496 | 80971595 | Lactate dehydrogenase level QTL (15099) | 6 | 37585548 | 1.2E+08 |
| ENSSSCG00000003531 | 6 | 80903496 | 80971595 | Carcass length QTL (3873) | 6 | 38610608 | 1.2E+08 |
| ENSSSCG00000003531 | 6 | 80903496 | 80971595 | Average daily gain QTL (186) | 6 | 46981935 | 98234955 |
| ENSSSCG00000003531 | 6 | 80903496 | 80971595 | Shear force QTL (38091) | 6 | 50398803 | 1.38E+08 |
| ENSSSCG00000003531 | 6 | 80903496 | 80971595 | Oleic acid content QTL (156935) | 6 | 50398803 | 88496881 |
| ENSSSCG00000003531 | 6 | 80903496 | 80971595 | Monounsaturated fatty acid content QTL (156937) | 6 | 50398803 | 88496881 |
| ENSSSCG00000003531 | 6 | 80903496 | 80971595 | Average backfat thickness QTL (1004) | 6 | 53644827 | 95672304 |
| ENSSSCG00000003531 | 6 | 80903496 | 80971595 | Abdominal fat weight QTL (1007) | 6 | 53644827 | 95672304 |
| ENSSSCG00000003531 | 6 | 80903496 | 80971595 | Backfat at tenth rib QTL (1011) | 6 | 53644827 | 95672304 |
| ENSSSCG00000003531 | 6 | 80903496 | 80971595 | Carcass weight (cold) QTL (1028) | 6 | 53644827 | 95672304 |
| ENSSSCG00000003531 | 6 | 80903496 | 80971595 | Body weight (slaughter) QTL (1031) | 6 | 53644827 | 95672304 |
| ENSSSCG00000003531 | 6 | 80903496 | 80971595 | Body weight (slaughter) QTL (1035) | 6 | 53644827 | 95672304 |
| ENSSSCG00000003531 | 6 | 80903496 | 80971595 | Creatine kinase level QTL (1045) | 6 | 53644827 | 95672304 |
| ENSSSCG00000003531 | 6 | 80903496 | 80971595 | Carcass weight (hot) QTL (16866) | 6 | 53644827 | 90632423 |
| ENSSSCG00000003531 | 6 | 80903496 | 80971595 | Conductivity 24 hours post-mortem QTL (1013) | 6 | 53644827 | 89692785 |
| ENSSSCG00000003531 | 6 | 80903496 | 80971595 | Smell intensity QTL (688) | 6 | 53644827 | 87130134 |
| ENSSSCG00000003531 | 6 | 80903496 | 80971595 | Average backfat thickness QTL (148) | 6 | 55524105 | 1.33E+08 |
| ENSSSCG00000003531 | 6 | 80903496 | 80971595 | Average backfat thickness QTL (149) | 6 | 55524105 | 1.33E+08 |
| ENSSSCG00000003531 | 6 | 80903496 | 80971595 | Intramuscular fat content QTL (150) | 6 | 55524105 | 1.33E+08 |
| ENSSSCG00000003531 | 6 | 80903496 | 80971595 | Intramuscular fat content QTL (151) | 6 | 55524105 | 1.33E+08 |
| ENSSSCG00000003531 | 6 | 80903496 | 80971595 | Alkaline phosphatase activity QTL (6347) | 6 | 58684707 | 94305556 |
| ENSSSCG00000003531 | 6 | 80903496 | 80971595 | Calcium level QTL (6376) | 6 | 58684707 | 94305556 |
| ENSSSCG00000003531 | 6 | 80903496 | 80971595 | Backfat between 3rd and 4th last ribs QTL (4167) | 6 | 59368081 | 1.24E+08 |
| ENSSSCG00000003531 | 6 | 80903496 | 80971595 | Conductivity 24 hours post-mortem QTL (1245) | 6 | 59795190 | 82004832 |
| ENSSSCG00000003531 | 6 | 80903496 | 80971595 | Conductivity 24 hours post-mortem QTL (1247) | 6 | 59795190 | 82004832 |
| ENSSSCG00000003531 | 6 | 80903496 | 80971595 | Average backfat thickness QTL (680) | 6 | 60649407 | 92255436 |
| ENSSSCG00000003531 | 6 | 80903496 | 80971595 | body weight (17 weeks) QTL (184) | 6 | 61759889 | 1.15E+08 |
| ENSSSCG00000003531 | 6 | 80903496 | 80971595 | body weight (13 weeks) QTL (183) | 6 | 61759889 | 1.07E+08 |
| ENSSSCG00000003531 | 6 | 80903496 | 80971595 | Average daily gain QTL (185) | 6 | 61759889 | 97209894 |
| ENSSSCG00000003531 | 6 | 80903496 | 80971595 | Intramuscular fat content QTL (4169) | 6 | 61930732 | 1.26E+08 |
| ENSSSCG00000003531 | 6 | 80903496 | 80971595 | Conductivity 24 hours post-mortem QTL (988) | 6 | 65005913 | 95672304 |
| ENSSSCG00000003531 | 6 | 80903496 | 80971595 | Loin muscle area QTL (1006) | 6 | 65005913 | 95672304 |
| ENSSSCG00000003531 | 6 | 80903496 | 80971595 | Meat color score QTL (21854) | 6 | 65005913 | 89692785 |
| ENSSSCG00000003531 | 6 | 80903496 | 80971595 | Meat color-a QTL (991) | 6 | 65005913 | 89692785 |
| ENSSSCG00000003531 | 6 | 80903496 | 80971595 | Conductivity 24 hours post-mortem QTL (992) | 6 | 65005913 | 89692785 |
| ENSSSCG00000003531 | 6 | 80903496 | 80971595 | pH 45 minutes post mortem QTL (994) | 6 | 65005913 | 89692785 |
| ENSSSCG00000003531 | 6 | 80903496 | 80971595 | pH 45 minutes post mortem QTL (1015) | 6 | 65005913 | 89692785 |
| ENSSSCG00000003531 | 6 | 80903496 | 80971595 | Creatine kinase level QTL (1041) | 6 | 65005913 | 89692785 |
| ENSSSCG00000003531 | 6 | 80903496 | 80971595 | Backfat at last rib QTL (16877) | 6 | 65005913 | 89692785 |
| ENSSSCG00000003531 | 6 | 80903496 | 80971595 | Body weight (20 weeks) QTL (29696) | 6 | 65347600 | 1.28E+08 |
| ENSSSCG00000003531 | 6 | 80903496 | 80971595 | Carcass weight (hot) QTL (29697) | 6 | 65347600 | 1.28E+08 |
| ENSSSCG00000003531 | 6 | 80903496 | 80971595 | subjective boar flavor in lean QTL (4219) | 6 | 65347600 | 1.28E+08 |
| ENSSSCG00000003531 | 6 | 80903496 | 80971595 | subjective pork flavor in fat QTL (4223) | 6 | 65347600 | 99089172 |
| ENSSSCG00000038994 | 6 | 82401188 | 82483724 | Overall impression, sensory panel QTL (5566) | 6 | 67363552 | 86865326 |
| ENSSSCG00000038994 | 6 | 82401188 | 82483724 | Average backfat thickness QTL (12859) | 6 | 68508203 | 89863628 |
| ENSSSCG00000038994 | 6 | 82401188 | 82483724 | Muscle moisture percentage QTL (12046) | 6 | 68508203 | 89863628 |
| ENSSSCG00000038994 | 6 | 82401188 | 82483724 | muscle protein percentage QTL (12047) | 6 | 68508203 | 89863628 |
| ENSSSCG00000038994 | 6 | 82401188 | 82483724 | Cholesterol level in meat QTL (12048) | 6 | 68508203 | 89863628 |
| ENSSSCG00000038994 | 6 | 82401188 | 82483724 | Muscle moisture percentage QTL (12050) | 6 | 68508203 | 89863628 |
| ENSSSCG00000038994 | 6 | 82401188 | 82483724 | Flavor score QTL (12052) | 6 | 68508203 | 89863628 |
| ENSSSCG00000038994 | 6 | 82401188 | 82483724 | Muscle moisture percentage QTL (12053) | 6 | 68508203 | 89863628 |
| ENSSSCG00000038994 | 6 | 82401188 | 82483724 | Marbling QTL (12054) | 6 | 68508203 | 89863628 |
| ENSSSCG00000038994 | 6 | 82401188 | 82483724 | Meat color score QTL (12055) | 6 | 68508203 | 89863628 |
| ENSSSCG00000038994 | 6 | 82401188 | 82483724 | Flavor score QTL (12056) | 6 | 68508203 | 89863628 |
| ENSSSCG00000038994 | 6 | 82401188 | 82483724 | Loin muscle area QTL (3190) | 6 | 71156276 | 1.03E+08 |
| ENSSSCG00000038994 | 6 | 82401188 | 82483724 | Loin muscle area QTL (3191) | 6 | 71156276 | 1.03E+08 |
| ENSSSCG00000038994 | 6 | 82401188 | 82483724 | Estimated carcass lean content QTL (3192) | 6 | 71156276 | 1.03E+08 |
| ENSSSCG00000038994 | 6 | 82401188 | 82483724 | Empty body protein content QTL (3193) | 6 | 71156276 | 1.03E+08 |
| ENSSSCG00000038994 | 6 | 82401188 | 82483724 | Backfat at last rib QTL (3194) | 6 | 71156276 | 1.03E+08 |
| ENSSSCG00000038994 | 6 | 82401188 | 82483724 | Marbling QTL (3239) | 6 | 71156276 | 1.03E+08 |
| ENSSSCG00000038994 | 6 | 82401188 | 82483724 | Muscle fat content QTL (3240) | 6 | 71156276 | 1.03E+08 |
| ENSSSCG00000038994 | 6 | 82401188 | 82483724 | Backfat at tenth rib QTL (3242) | 6 | 71156276 | 1.03E+08 |
| ENSSSCG00000038994 | 6 | 82401188 | 82483724 | Backfat at last lumbar QTL (3243) | 6 | 71156276 | 1.03E+08 |
| ENSSSCG00000038994 | 6 | 82401188 | 82483724 | Empty body lipid content QTL (3646) | 6 | 71156276 | 1.03E+08 |
| ENSSSCG00000038994 | 6 | 82401188 | 82483724 | Loin muscle area QTL (3648) | 6 | 71156276 | 1.03E+08 |
| ENSSSCG00000038994 | 6 | 82401188 | 82483724 | Backfat at last rib QTL (2852) | 6 | 71156276 | 89863628 |
| ENSSSCG00000038994 | 6 | 82401188 | 82483724 | Loin muscle area QTL (2863) | 6 | 71156276 | 89863628 |
| ENSSSCG00000038994 | 6 | 82401188 | 82483724 | Empty body protein content QTL (2874) | 6 | 71156276 | 89863628 |
| ENSSSCG00000038994 | 6 | 82401188 | 82483724 | Empty body protein content QTL (2876) | 6 | 71156276 | 89863628 |
| ENSSSCG00000038994 | 6 | 82401188 | 82483724 | Empty body lipid content QTL (2878) | 6 | 71156276 | 89863628 |
| ENSSSCG00000038994 | 6 | 82401188 | 82483724 | Intramuscular fat content QTL (163) | 6 | 71156276 | 89863628 |
| ENSSSCG00000038994 | 6 | 82401188 | 82483724 | Intramuscular fat content QTL (368) | 6 | 76281578 | 91401219 |
| ENSSSCG00000038994 | 6 | 82401188 | 82483724 | Average daily gain QTL (3674) | 6 | 76281578 | 87130134 |
| ENSSSCG00000038994 | 6 | 82401188 | 82483724 | Backfat at last rib QTL (3675) | 6 | 76281578 | 87130134 |
| ENSSSCG00000038994 | 6 | 82401188 | 82483724 | Carcass weight (hot) QTL (3696) | 6 | 76281578 | 87130134 |
| ENSSSCG00000038994 | 6 | 82401188 | 82483724 | Carcass length QTL (3697) | 6 | 76281578 | 87130134 |
| ENSSSCG00000038994 | 6 | 82401188 | 82483724 | PH for Longissmus dorsi QTL (3698) | 6 | 76281578 | 87130134 |
| ENSSSCG00000038994 | 6 | 82401188 | 82483724 | Average daily gain QTL (4131) | 6 | 76281578 | 87130134 |
| ENSSSCG00000038994 | 6 | 82401188 | 82483724 | Average daily gain QTL (4132) | 6 | 76281578 | 87130134 |
| ENSSSCG00000038994 | 6 | 82401188 | 82483724 | Carcass length QTL (4133) | 6 | 76281578 | 87130134 |
| ENSSSCG00000038994 | 6 | 82401188 | 82483724 | PH for Longissmus dorsi QTL (4135) | 6 | 76281578 | 87130134 |
| ENSSSCG00000038994 | 6 | 82401188 | 82483724 | PH for Longissmus dorsi QTL (4136) | 6 | 76281578 | 87130134 |
| ENSSSCG00000038994 | 6 | 82401188 | 82483724 | PH for Longissmus dorsi QTL (4139) | 6 | 76281578 | 87130134 |
| ENSSSCG00000038994 | 6 | 82401188 | 82483724 | Conductivity 24 hours postmortem (loin) QTL (4141) | 6 | 76281578 | 87130134 |
| ENSSSCG00000038994 | 6 | 82401188 | 82483724 | Conductivity 24 hours postmortem (loin) QTL (4193) | 6 | 76281578 | 87130134 |
| ENSSSCG00000038994 | 6 | 82401188 | 82483724 | Backfat at first rib QTL (681) | 6 | 76879530 | 92255436 |
| ENSSSCG00000038994 | 6 | 82401188 | 82483724 | Intramuscular fat content QTL (668) | 6 | 77477481 | 89863628 |
| ENSSSCG00000038994 | 6 | 82401188 | 82483724 | Conductivity 24 hours post-mortem QTL (1016) | 6 | 77990012 | 98234955 |
| ENSSSCG00000038994 | 6 | 82401188 | 82483724 | Meat color-a QTL (1018) | 6 | 77990012 | 98234955 |
| ENSSSCG00000038994 | 6 | 82401188 | 82483724 | pH 45 minutes post mortem QTL (1019) | 6 | 77990012 | 98234955 |
| ENSSSCG00000038994 | 6 | 82401188 | 82483724 | pH 24 hr post-mortem (loin) QTL (1030) | 6 | 77990012 | 98234955 |
| ENSSSCG00000038994 | 6 | 82401188 | 82483724 | Loin muscle area QTL (1034) | 6 | 77990012 | 98234955 |
| ENSSSCG00000038994 | 6 | 82401188 | 82483724 | pH 24 hr post-mortem (loin) QTL (990) | 6 | 77990012 | 89692785 |
| ENSSSCG00000038994 | 6 | 82401188 | 82483724 | Backfat at last rib QTL (16878) | 6 | 77990012 | 89692785 |
| ENSSSCG00000038994 | 6 | 82401188 | 82483724 | Interleukin-2 level QTL (17599) | 6 | 79271337 | 87130134 |
| ENSSSCG00000038994 | 6 | 82401188 | 82483724 | Cholesterol level QTL (17718) | 6 | 79271337 | 87130134 |
| ENSSSCG00000038994 | 6 | 82401188 | 82483724 | Meat color L* QTL (17556) | 6 | 79527602 | 96611942 |
| ENSSSCG00000038994 | 6 | 82401188 | 82483724 | Loin muscle area QTL (7298) | 6 | 80210976 | 99089172 |
| ENSSSCG00000038994 | 6 | 82401188 | 82483724 | Marbling QTL (38089) | 6 | 6235784 | 1.28E+08 |
| ENSSSCG00000038994 | 6 | 82401188 | 82483724 | Meat color a* QTL (3921) | 6 | 6235784 | 94305556 |
| ENSSSCG00000038994 | 6 | 82401188 | 82483724 | Meat color a* QTL (3922) | 6 | 6235784 | 94305556 |
| ENSSSCG00000038994 | 6 | 82401188 | 82483724 | Meat color a* QTL (3923) | 6 | 6235784 | 94305556 |
| ENSSSCG00000038994 | 6 | 82401188 | 82483724 | Meat color a* QTL (3924) | 6 | 6235784 | 94305556 |
| ENSSSCG00000038994 | 6 | 82401188 | 82483724 | Body weight (46 days) QTL (21240) | 6 | 8029639 | 91486640 |
| ENSSSCG00000038994 | 6 | 82401188 | 82483724 | Body weight (end of test) QTL (5201) | 6 | 12044459 | 1.35E+08 |
| ENSSSCG00000038994 | 6 | 82401188 | 82483724 | Alkaline phosphatase activity QTL (15097) | 6 | 35450005 | 1.38E+08 |
| ENSSSCG00000038994 | 6 | 82401188 | 82483724 | Loin fat percentage QTL (38090) | 6 | 35450005 | 1.28E+08 |
| ENSSSCG00000038994 | 6 | 82401188 | 82483724 | Lactate dehydrogenase level QTL (15099) | 6 | 37585548 | 1.2E+08 |
| ENSSSCG00000038994 | 6 | 82401188 | 82483724 | Carcass length QTL (3873) | 6 | 38610608 | 1.2E+08 |
| ENSSSCG00000038994 | 6 | 82401188 | 82483724 | Average daily gain QTL (186) | 6 | 46981935 | 98234955 |
| ENSSSCG00000038994 | 6 | 82401188 | 82483724 | Shear force QTL (38091) | 6 | 50398803 | 1.38E+08 |
| ENSSSCG00000038994 | 6 | 82401188 | 82483724 | Oleic acid content QTL (156935) | 6 | 50398803 | 88496881 |
| ENSSSCG00000038994 | 6 | 82401188 | 82483724 | Monounsaturated fatty acid content QTL (156937) | 6 | 50398803 | 88496881 |
| ENSSSCG00000038994 | 6 | 82401188 | 82483724 | Average backfat thickness QTL (1004) | 6 | 53644827 | 95672304 |
| ENSSSCG00000038994 | 6 | 82401188 | 82483724 | Abdominal fat weight QTL (1007) | 6 | 53644827 | 95672304 |
| ENSSSCG00000038994 | 6 | 82401188 | 82483724 | Backfat at tenth rib QTL (1011) | 6 | 53644827 | 95672304 |
| ENSSSCG00000038994 | 6 | 82401188 | 82483724 | Carcass weight (cold) QTL (1028) | 6 | 53644827 | 95672304 |
| ENSSSCG00000038994 | 6 | 82401188 | 82483724 | Body weight (slaughter) QTL (1031) | 6 | 53644827 | 95672304 |
| ENSSSCG00000038994 | 6 | 82401188 | 82483724 | Body weight (slaughter) QTL (1035) | 6 | 53644827 | 95672304 |
| ENSSSCG00000038994 | 6 | 82401188 | 82483724 | Creatine kinase level QTL (1045) | 6 | 53644827 | 95672304 |
| ENSSSCG00000038994 | 6 | 82401188 | 82483724 | Carcass weight (hot) QTL (16866) | 6 | 53644827 | 90632423 |
| ENSSSCG00000038994 | 6 | 82401188 | 82483724 | Conductivity 24 hours post-mortem QTL (1013) | 6 | 53644827 | 89692785 |
| ENSSSCG00000038994 | 6 | 82401188 | 82483724 | Smell intensity QTL (688) | 6 | 53644827 | 87130134 |
| ENSSSCG00000038994 | 6 | 82401188 | 82483724 | Average backfat thickness QTL (148) | 6 | 55524105 | 1.33E+08 |
| ENSSSCG00000038994 | 6 | 82401188 | 82483724 | Average backfat thickness QTL (149) | 6 | 55524105 | 1.33E+08 |
| ENSSSCG00000038994 | 6 | 82401188 | 82483724 | Intramuscular fat content QTL (150) | 6 | 55524105 | 1.33E+08 |
| ENSSSCG00000038994 | 6 | 82401188 | 82483724 | Intramuscular fat content QTL (151) | 6 | 55524105 | 1.33E+08 |
| ENSSSCG00000038994 | 6 | 82401188 | 82483724 | Alkaline phosphatase activity QTL (6347) | 6 | 58684707 | 94305556 |
| ENSSSCG00000038994 | 6 | 82401188 | 82483724 | Calcium level QTL (6376) | 6 | 58684707 | 94305556 |
| ENSSSCG00000038994 | 6 | 82401188 | 82483724 | Backfat between 3rd and 4th last ribs QTL (4167) | 6 | 59368081 | 1.24E+08 |
| ENSSSCG00000038994 | 6 | 82401188 | 82483724 | Average backfat thickness QTL (680) | 6 | 60649407 | 92255436 |
| ENSSSCG00000038994 | 6 | 82401188 | 82483724 | body weight (17 weeks) QTL (184) | 6 | 61759889 | 1.15E+08 |
| ENSSSCG00000038994 | 6 | 82401188 | 82483724 | body weight (13 weeks) QTL (183) | 6 | 61759889 | 1.07E+08 |
| ENSSSCG00000038994 | 6 | 82401188 | 82483724 | Average daily gain QTL (185) | 6 | 61759889 | 97209894 |
| ENSSSCG00000038994 | 6 | 82401188 | 82483724 | Intramuscular fat content QTL (4169) | 6 | 61930732 | 1.26E+08 |
| ENSSSCG00000038994 | 6 | 82401188 | 82483724 | Conductivity 24 hours post-mortem QTL (988) | 6 | 65005913 | 95672304 |
| ENSSSCG00000038994 | 6 | 82401188 | 82483724 | Loin muscle area QTL (1006) | 6 | 65005913 | 95672304 |
| ENSSSCG00000038994 | 6 | 82401188 | 82483724 | Meat color score QTL (21854) | 6 | 65005913 | 89692785 |
| ENSSSCG00000038994 | 6 | 82401188 | 82483724 | Meat color-a QTL (991) | 6 | 65005913 | 89692785 |
| ENSSSCG00000038994 | 6 | 82401188 | 82483724 | Conductivity 24 hours post-mortem QTL (992) | 6 | 65005913 | 89692785 |
| ENSSSCG00000038994 | 6 | 82401188 | 82483724 | pH 45 minutes post mortem QTL (994) | 6 | 65005913 | 89692785 |
| ENSSSCG00000038994 | 6 | 82401188 | 82483724 | pH 45 minutes post mortem QTL (1015) | 6 | 65005913 | 89692785 |
| ENSSSCG00000038994 | 6 | 82401188 | 82483724 | Creatine kinase level QTL (1041) | 6 | 65005913 | 89692785 |
| ENSSSCG00000038994 | 6 | 82401188 | 82483724 | Backfat at last rib QTL (16877) | 6 | 65005913 | 89692785 |
| ENSSSCG00000038994 | 6 | 82401188 | 82483724 | Body weight (20 weeks) QTL (29696) | 6 | 65347600 | 1.28E+08 |
| ENSSSCG00000038994 | 6 | 82401188 | 82483724 | Carcass weight (hot) QTL (29697) | 6 | 65347600 | 1.28E+08 |
| ENSSSCG00000038994 | 6 | 82401188 | 82483724 | subjective boar flavor in lean QTL (4219) | 6 | 65347600 | 1.28E+08 |
| ENSSSCG00000038994 | 6 | 82401188 | 82483724 | subjective pork flavor in fat QTL (4223) | 6 | 65347600 | 99089172 |
| ENSSSCG00000039885 | 6 | 88419387 | 88452589 | Loin muscle area QTL (17801) | 6 | 84225796 | 89863628 |
| ENSSSCG00000039885 | 6 | 88419387 | 88452589 | Backfat at last rib QTL (679) | 6 | 85421700 | 91401219 |
| ENSSSCG00000039885 | 6 | 88419387 | 88452589 | Average backfat thickness QTL (12859) | 6 | 68508203 | 89863628 |
| ENSSSCG00000039885 | 6 | 88419387 | 88452589 | Muscle moisture percentage QTL (12046) | 6 | 68508203 | 89863628 |
| ENSSSCG00000039885 | 6 | 88419387 | 88452589 | muscle protein percentage QTL (12047) | 6 | 68508203 | 89863628 |
| ENSSSCG00000039885 | 6 | 88419387 | 88452589 | Cholesterol level in meat QTL (12048) | 6 | 68508203 | 89863628 |
| ENSSSCG00000039885 | 6 | 88419387 | 88452589 | Muscle moisture percentage QTL (12050) | 6 | 68508203 | 89863628 |
| ENSSSCG00000039885 | 6 | 88419387 | 88452589 | Flavor score QTL (12052) | 6 | 68508203 | 89863628 |
| ENSSSCG00000039885 | 6 | 88419387 | 88452589 | Muscle moisture percentage QTL (12053) | 6 | 68508203 | 89863628 |
| ENSSSCG00000039885 | 6 | 88419387 | 88452589 | Marbling QTL (12054) | 6 | 68508203 | 89863628 |
| ENSSSCG00000039885 | 6 | 88419387 | 88452589 | Meat color score QTL (12055) | 6 | 68508203 | 89863628 |
| ENSSSCG00000039885 | 6 | 88419387 | 88452589 | Flavor score QTL (12056) | 6 | 68508203 | 89863628 |
| ENSSSCG00000039885 | 6 | 88419387 | 88452589 | Loin muscle area QTL (3190) | 6 | 71156276 | 1.03E+08 |
| ENSSSCG00000039885 | 6 | 88419387 | 88452589 | Loin muscle area QTL (3191) | 6 | 71156276 | 1.03E+08 |
| ENSSSCG00000039885 | 6 | 88419387 | 88452589 | Estimated carcass lean content QTL (3192) | 6 | 71156276 | 1.03E+08 |
| ENSSSCG00000039885 | 6 | 88419387 | 88452589 | Empty body protein content QTL (3193) | 6 | 71156276 | 1.03E+08 |
| ENSSSCG00000039885 | 6 | 88419387 | 88452589 | Backfat at last rib QTL (3194) | 6 | 71156276 | 1.03E+08 |
| ENSSSCG00000039885 | 6 | 88419387 | 88452589 | Marbling QTL (3239) | 6 | 71156276 | 1.03E+08 |
| ENSSSCG00000039885 | 6 | 88419387 | 88452589 | Muscle fat content QTL (3240) | 6 | 71156276 | 1.03E+08 |
| ENSSSCG00000039885 | 6 | 88419387 | 88452589 | Backfat at tenth rib QTL (3242) | 6 | 71156276 | 1.03E+08 |
| ENSSSCG00000039885 | 6 | 88419387 | 88452589 | Backfat at last lumbar QTL (3243) | 6 | 71156276 | 1.03E+08 |
| ENSSSCG00000039885 | 6 | 88419387 | 88452589 | Empty body lipid content QTL (3646) | 6 | 71156276 | 1.03E+08 |
| ENSSSCG00000039885 | 6 | 88419387 | 88452589 | Loin muscle area QTL (3648) | 6 | 71156276 | 1.03E+08 |
| ENSSSCG00000039885 | 6 | 88419387 | 88452589 | Backfat at last rib QTL (2852) | 6 | 71156276 | 89863628 |
| ENSSSCG00000039885 | 6 | 88419387 | 88452589 | Loin muscle area QTL (2863) | 6 | 71156276 | 89863628 |
| ENSSSCG00000039885 | 6 | 88419387 | 88452589 | Empty body protein content QTL (2874) | 6 | 71156276 | 89863628 |
| ENSSSCG00000039885 | 6 | 88419387 | 88452589 | Empty body protein content QTL (2876) | 6 | 71156276 | 89863628 |
| ENSSSCG00000039885 | 6 | 88419387 | 88452589 | Empty body lipid content QTL (2878) | 6 | 71156276 | 89863628 |
| ENSSSCG00000039885 | 6 | 88419387 | 88452589 | Intramuscular fat content QTL (163) | 6 | 71156276 | 89863628 |
| ENSSSCG00000039885 | 6 | 88419387 | 88452589 | Intramuscular fat content QTL (368) | 6 | 76281578 | 91401219 |
| ENSSSCG00000039885 | 6 | 88419387 | 88452589 | Backfat at first rib QTL (681) | 6 | 76879530 | 92255436 |
| ENSSSCG00000039885 | 6 | 88419387 | 88452589 | Intramuscular fat content QTL (668) | 6 | 77477481 | 89863628 |
| ENSSSCG00000039885 | 6 | 88419387 | 88452589 | Conductivity 24 hours post-mortem QTL (1016) | 6 | 77990012 | 98234955 |
| ENSSSCG00000039885 | 6 | 88419387 | 88452589 | Meat color-a QTL (1018) | 6 | 77990012 | 98234955 |
| ENSSSCG00000039885 | 6 | 88419387 | 88452589 | pH 45 minutes post mortem QTL (1019) | 6 | 77990012 | 98234955 |
| ENSSSCG00000039885 | 6 | 88419387 | 88452589 | pH 24 hr post-mortem (loin) QTL (1030) | 6 | 77990012 | 98234955 |
| ENSSSCG00000039885 | 6 | 88419387 | 88452589 | Loin muscle area QTL (1034) | 6 | 77990012 | 98234955 |
| ENSSSCG00000039885 | 6 | 88419387 | 88452589 | pH 24 hr post-mortem (loin) QTL (990) | 6 | 77990012 | 89692785 |
| ENSSSCG00000039885 | 6 | 88419387 | 88452589 | Backfat at last rib QTL (16878) | 6 | 77990012 | 89692785 |
| ENSSSCG00000039885 | 6 | 88419387 | 88452589 | Meat color L* QTL (17556) | 6 | 79527602 | 96611942 |
| ENSSSCG00000039885 | 6 | 88419387 | 88452589 | Loin muscle area QTL (7298) | 6 | 80210976 | 99089172 |
| ENSSSCG00000039885 | 6 | 88419387 | 88452589 | Marbling QTL (21368) | 6 | 82859049 | 92853387 |
| ENSSSCG00000039885 | 6 | 88419387 | 88452589 | Meat color a* QTL (21369) | 6 | 82859049 | 92853387 |
| ENSSSCG00000039885 | 6 | 88419387 | 88452589 | Loin fat percentage QTL (21434) | 6 | 82859049 | 92853387 |
| ENSSSCG00000039885 | 6 | 88419387 | 88452589 | Intramuscular fat content QTL (299) | 6 | 82859049 | 90632423 |
| ENSSSCG00000039885 | 6 | 88419387 | 88452589 | Loin muscle area QTL (4168) | 6 | 84567483 | 1.29E+08 |
| ENSSSCG00000039885 | 6 | 88419387 | 88452589 | Loin muscle depth QTL (673) | 6 | 86275917 | 1.05E+08 |
| ENSSSCG00000039885 | 6 | 88419387 | 88452589 | Smell intensity QTL (164) | 6 | 87130134 | 1.28E+08 |
| ENSSSCG00000039885 | 6 | 88419387 | 88452589 | Marbling QTL (38089) | 6 | 6235784 | 1.28E+08 |
| ENSSSCG00000039885 | 6 | 88419387 | 88452589 | Meat color a* QTL (3921) | 6 | 6235784 | 94305556 |
| ENSSSCG00000039885 | 6 | 88419387 | 88452589 | Meat color a* QTL (3922) | 6 | 6235784 | 94305556 |
| ENSSSCG00000039885 | 6 | 88419387 | 88452589 | Meat color a* QTL (3923) | 6 | 6235784 | 94305556 |
| ENSSSCG00000039885 | 6 | 88419387 | 88452589 | Meat color a* QTL (3924) | 6 | 6235784 | 94305556 |
| ENSSSCG00000039885 | 6 | 88419387 | 88452589 | Body weight (46 days) QTL (21240) | 6 | 8029639 | 91486640 |
| ENSSSCG00000039885 | 6 | 88419387 | 88452589 | Body weight (end of test) QTL (5201) | 6 | 12044459 | 1.35E+08 |
| ENSSSCG00000039885 | 6 | 88419387 | 88452589 | Alkaline phosphatase activity QTL (15097) | 6 | 35450005 | 1.38E+08 |
| ENSSSCG00000039885 | 6 | 88419387 | 88452589 | Loin fat percentage QTL (38090) | 6 | 35450005 | 1.28E+08 |
| ENSSSCG00000039885 | 6 | 88419387 | 88452589 | Lactate dehydrogenase level QTL (15099) | 6 | 37585548 | 1.2E+08 |
| ENSSSCG00000039885 | 6 | 88419387 | 88452589 | Carcass length QTL (3873) | 6 | 38610608 | 1.2E+08 |
| ENSSSCG00000039885 | 6 | 88419387 | 88452589 | Average daily gain QTL (186) | 6 | 46981935 | 98234955 |
| ENSSSCG00000039885 | 6 | 88419387 | 88452589 | Shear force QTL (38091) | 6 | 50398803 | 1.38E+08 |
| ENSSSCG00000039885 | 6 | 88419387 | 88452589 | Oleic acid content QTL (156935) | 6 | 50398803 | 88496881 |
| ENSSSCG00000039885 | 6 | 88419387 | 88452589 | Monounsaturated fatty acid content QTL (156937) | 6 | 50398803 | 88496881 |
| ENSSSCG00000039885 | 6 | 88419387 | 88452589 | Average backfat thickness QTL (1004) | 6 | 53644827 | 95672304 |
| ENSSSCG00000039885 | 6 | 88419387 | 88452589 | Abdominal fat weight QTL (1007) | 6 | 53644827 | 95672304 |
| ENSSSCG00000039885 | 6 | 88419387 | 88452589 | Backfat at tenth rib QTL (1011) | 6 | 53644827 | 95672304 |
| ENSSSCG00000039885 | 6 | 88419387 | 88452589 | Carcass weight (cold) QTL (1028) | 6 | 53644827 | 95672304 |
| ENSSSCG00000039885 | 6 | 88419387 | 88452589 | Body weight (slaughter) QTL (1031) | 6 | 53644827 | 95672304 |
| ENSSSCG00000039885 | 6 | 88419387 | 88452589 | Body weight (slaughter) QTL (1035) | 6 | 53644827 | 95672304 |
| ENSSSCG00000039885 | 6 | 88419387 | 88452589 | Creatine kinase level QTL (1045) | 6 | 53644827 | 95672304 |
| ENSSSCG00000039885 | 6 | 88419387 | 88452589 | Carcass weight (hot) QTL (16866) | 6 | 53644827 | 90632423 |
| ENSSSCG00000039885 | 6 | 88419387 | 88452589 | Conductivity 24 hours post-mortem QTL (1013) | 6 | 53644827 | 89692785 |
| ENSSSCG00000039885 | 6 | 88419387 | 88452589 | Average backfat thickness QTL (148) | 6 | 55524105 | 1.33E+08 |
| ENSSSCG00000039885 | 6 | 88419387 | 88452589 | Average backfat thickness QTL (149) | 6 | 55524105 | 1.33E+08 |
| ENSSSCG00000039885 | 6 | 88419387 | 88452589 | Intramuscular fat content QTL (150) | 6 | 55524105 | 1.33E+08 |
| ENSSSCG00000039885 | 6 | 88419387 | 88452589 | Intramuscular fat content QTL (151) | 6 | 55524105 | 1.33E+08 |
| ENSSSCG00000039885 | 6 | 88419387 | 88452589 | Alkaline phosphatase activity QTL (6347) | 6 | 58684707 | 94305556 |
| ENSSSCG00000039885 | 6 | 88419387 | 88452589 | Calcium level QTL (6376) | 6 | 58684707 | 94305556 |
| ENSSSCG00000039885 | 6 | 88419387 | 88452589 | Backfat between 3rd and 4th last ribs QTL (4167) | 6 | 59368081 | 1.24E+08 |
| ENSSSCG00000039885 | 6 | 88419387 | 88452589 | Average backfat thickness QTL (680) | 6 | 60649407 | 92255436 |
| ENSSSCG00000039885 | 6 | 88419387 | 88452589 | body weight (17 weeks) QTL (184) | 6 | 61759889 | 1.15E+08 |
| ENSSSCG00000039885 | 6 | 88419387 | 88452589 | body weight (13 weeks) QTL (183) | 6 | 61759889 | 1.07E+08 |
| ENSSSCG00000039885 | 6 | 88419387 | 88452589 | Average daily gain QTL (185) | 6 | 61759889 | 97209894 |
| ENSSSCG00000039885 | 6 | 88419387 | 88452589 | Intramuscular fat content QTL (4169) | 6 | 61930732 | 1.26E+08 |
| ENSSSCG00000039885 | 6 | 88419387 | 88452589 | Conductivity 24 hours post-mortem QTL (988) | 6 | 65005913 | 95672304 |
| ENSSSCG00000039885 | 6 | 88419387 | 88452589 | Loin muscle area QTL (1006) | 6 | 65005913 | 95672304 |
| ENSSSCG00000039885 | 6 | 88419387 | 88452589 | Meat color score QTL (21854) | 6 | 65005913 | 89692785 |
| ENSSSCG00000039885 | 6 | 88419387 | 88452589 | Meat color-a QTL (991) | 6 | 65005913 | 89692785 |
| ENSSSCG00000039885 | 6 | 88419387 | 88452589 | Conductivity 24 hours post-mortem QTL (992) | 6 | 65005913 | 89692785 |
| ENSSSCG00000039885 | 6 | 88419387 | 88452589 | pH 45 minutes post mortem QTL (994) | 6 | 65005913 | 89692785 |
| ENSSSCG00000039885 | 6 | 88419387 | 88452589 | pH 45 minutes post mortem QTL (1015) | 6 | 65005913 | 89692785 |
| ENSSSCG00000039885 | 6 | 88419387 | 88452589 | Creatine kinase level QTL (1041) | 6 | 65005913 | 89692785 |
| ENSSSCG00000039885 | 6 | 88419387 | 88452589 | Backfat at last rib QTL (16877) | 6 | 65005913 | 89692785 |
| ENSSSCG00000039885 | 6 | 88419387 | 88452589 | Body weight (20 weeks) QTL (29696) | 6 | 65347600 | 1.28E+08 |
| ENSSSCG00000039885 | 6 | 88419387 | 88452589 | Carcass weight (hot) QTL (29697) | 6 | 65347600 | 1.28E+08 |
| ENSSSCG00000039885 | 6 | 88419387 | 88452589 | subjective boar flavor in lean QTL (4219) | 6 | 65347600 | 1.28E+08 |
| ENSSSCG00000039885 | 6 | 88419387 | 88452589 | subjective pork flavor in fat QTL (4223) | 6 | 65347600 | 99089172 |
| ENSSSCG00000039885 | 6 | 88419387 | 88452589 | Intramuscular fat content QTL (314) | 6 | 87130134 | 1.38E+08 |
| ENSSSCG00000003627 | 6 | 91455778 | 91623819 | Loin muscle area QTL (6260) | 6 | 91512267 | 91853954 |
| ENSSSCG00000003627 | 6 | 91455778 | 91623819 | Loin muscle area QTL (3190) | 6 | 71156276 | 1.03E+08 |
| ENSSSCG00000003627 | 6 | 91455778 | 91623819 | Loin muscle area QTL (3191) | 6 | 71156276 | 1.03E+08 |
| ENSSSCG00000003627 | 6 | 91455778 | 91623819 | Estimated carcass lean content QTL (3192) | 6 | 71156276 | 1.03E+08 |
| ENSSSCG00000003627 | 6 | 91455778 | 91623819 | Empty body protein content QTL (3193) | 6 | 71156276 | 1.03E+08 |
| ENSSSCG00000003627 | 6 | 91455778 | 91623819 | Backfat at last rib QTL (3194) | 6 | 71156276 | 1.03E+08 |
| ENSSSCG00000003627 | 6 | 91455778 | 91623819 | Marbling QTL (3239) | 6 | 71156276 | 1.03E+08 |
| ENSSSCG00000003627 | 6 | 91455778 | 91623819 | Muscle fat content QTL (3240) | 6 | 71156276 | 1.03E+08 |
| ENSSSCG00000003627 | 6 | 91455778 | 91623819 | Backfat at tenth rib QTL (3242) | 6 | 71156276 | 1.03E+08 |
| ENSSSCG00000003627 | 6 | 91455778 | 91623819 | Backfat at last lumbar QTL (3243) | 6 | 71156276 | 1.03E+08 |
| ENSSSCG00000003627 | 6 | 91455778 | 91623819 | Empty body lipid content QTL (3646) | 6 | 71156276 | 1.03E+08 |
| ENSSSCG00000003627 | 6 | 91455778 | 91623819 | Loin muscle area QTL (3648) | 6 | 71156276 | 1.03E+08 |
| ENSSSCG00000003627 | 6 | 91455778 | 91623819 | Backfat at first rib QTL (681) | 6 | 76879530 | 92255436 |
| ENSSSCG00000003627 | 6 | 91455778 | 91623819 | Conductivity 24 hours post-mortem QTL (1016) | 6 | 77990012 | 98234955 |
| ENSSSCG00000003627 | 6 | 91455778 | 91623819 | Meat color-a QTL (1018) | 6 | 77990012 | 98234955 |
| ENSSSCG00000003627 | 6 | 91455778 | 91623819 | pH 45 minutes post mortem QTL (1019) | 6 | 77990012 | 98234955 |
| ENSSSCG00000003627 | 6 | 91455778 | 91623819 | pH 24 hr post-mortem (loin) QTL (1030) | 6 | 77990012 | 98234955 |
| ENSSSCG00000003627 | 6 | 91455778 | 91623819 | Loin muscle area QTL (1034) | 6 | 77990012 | 98234955 |
| ENSSSCG00000003627 | 6 | 91455778 | 91623819 | Meat color L* QTL (17556) | 6 | 79527602 | 96611942 |
| ENSSSCG00000003627 | 6 | 91455778 | 91623819 | Loin muscle area QTL (7298) | 6 | 80210976 | 99089172 |
| ENSSSCG00000003627 | 6 | 91455778 | 91623819 | Marbling QTL (21368) | 6 | 82859049 | 92853387 |
| ENSSSCG00000003627 | 6 | 91455778 | 91623819 | Meat color a* QTL (21369) | 6 | 82859049 | 92853387 |
| ENSSSCG00000003627 | 6 | 91455778 | 91623819 | Loin fat percentage QTL (21434) | 6 | 82859049 | 92853387 |
| ENSSSCG00000003627 | 6 | 91455778 | 91623819 | Loin muscle area QTL (4168) | 6 | 84567483 | 1.29E+08 |
| ENSSSCG00000003627 | 6 | 91455778 | 91623819 | Loin muscle depth QTL (673) | 6 | 86275917 | 1.05E+08 |
| ENSSSCG00000003627 | 6 | 91455778 | 91623819 | Smell intensity QTL (164) | 6 | 87130134 | 1.28E+08 |
| ENSSSCG00000003627 | 6 | 91455778 | 91623819 | Loin muscle area QTL (674) | 6 | 88838568 | 1.06E+08 |
| ENSSSCG00000003627 | 6 | 91455778 | 91623819 | pH 24 hr post-mortem (loin) QTL (1246) | 6 | 89692785 | 98234955 |
| ENSSSCG00000003627 | 6 | 91455778 | 91623819 | Meat color-a QTL (1248) | 6 | 89692785 | 98234955 |
| ENSSSCG00000003627 | 6 | 91455778 | 91623819 | Conductivity 24 hours post-mortem QTL (315) | 6 | 89692785 | 98234955 |
| ENSSSCG00000003627 | 6 | 91455778 | 91623819 | pH 45 minutes post mortem QTL (316) | 6 | 89692785 | 98234955 |
| ENSSSCG00000003627 | 6 | 91455778 | 91623819 | Creatine kinase level QTL (317) | 6 | 89692785 | 98234955 |
| ENSSSCG00000003627 | 6 | 91455778 | 91623819 | Average backfat thickness QTL (17999) | 6 | 89692785 | 95672304 |
| ENSSSCG00000003627 | 6 | 91455778 | 91623819 | Subcutanous fat area QTL (18005) | 6 | 89692785 | 95672304 |
| ENSSSCG00000003627 | 6 | 91455778 | 91623819 | Backfat at last rib QTL (16836) | 6 | 89692785 | 95672304 |
| ENSSSCG00000003627 | 6 | 91455778 | 91623819 | Loin muscle area QTL (152) | 6 | 89863628 | 1.33E+08 |
| ENSSSCG00000003627 | 6 | 91455778 | 91623819 | Loin muscle area QTL (153) | 6 | 89863628 | 1.33E+08 |
| ENSSSCG00000003627 | 6 | 91455778 | 91623819 | Average backfat thickness QTL (2865) | 6 | 89863628 | 1.03E+08 |
| ENSSSCG00000003627 | 6 | 91455778 | 91623819 | Muscle moisture percentage QTL (21706) | 6 | 90547002 | 1.06E+08 |
| ENSSSCG00000003627 | 6 | 91455778 | 91623819 | Marbling QTL (38089) | 6 | 6235784 | 1.28E+08 |
| ENSSSCG00000003627 | 6 | 91455778 | 91623819 | Meat color a* QTL (3921) | 6 | 6235784 | 94305556 |
| ENSSSCG00000003627 | 6 | 91455778 | 91623819 | Meat color a* QTL (3922) | 6 | 6235784 | 94305556 |
| ENSSSCG00000003627 | 6 | 91455778 | 91623819 | Meat color a* QTL (3923) | 6 | 6235784 | 94305556 |
| ENSSSCG00000003627 | 6 | 91455778 | 91623819 | Meat color a* QTL (3924) | 6 | 6235784 | 94305556 |
| ENSSSCG00000003627 | 6 | 91455778 | 91623819 | Body weight (end of test) QTL (5201) | 6 | 12044459 | 1.35E+08 |
| ENSSSCG00000003627 | 6 | 91455778 | 91623819 | Alkaline phosphatase activity QTL (15097) | 6 | 35450005 | 1.38E+08 |
| ENSSSCG00000003627 | 6 | 91455778 | 91623819 | Loin fat percentage QTL (38090) | 6 | 35450005 | 1.28E+08 |
| ENSSSCG00000003627 | 6 | 91455778 | 91623819 | Lactate dehydrogenase level QTL (15099) | 6 | 37585548 | 1.2E+08 |
| ENSSSCG00000003627 | 6 | 91455778 | 91623819 | Carcass length QTL (3873) | 6 | 38610608 | 1.2E+08 |
| ENSSSCG00000003627 | 6 | 91455778 | 91623819 | Average daily gain QTL (186) | 6 | 46981935 | 98234955 |
| ENSSSCG00000003627 | 6 | 91455778 | 91623819 | Shear force QTL (38091) | 6 | 50398803 | 1.38E+08 |
| ENSSSCG00000003627 | 6 | 91455778 | 91623819 | Average backfat thickness QTL (1004) | 6 | 53644827 | 95672304 |
| ENSSSCG00000003627 | 6 | 91455778 | 91623819 | Abdominal fat weight QTL (1007) | 6 | 53644827 | 95672304 |
| ENSSSCG00000003627 | 6 | 91455778 | 91623819 | Backfat at tenth rib QTL (1011) | 6 | 53644827 | 95672304 |
| ENSSSCG00000003627 | 6 | 91455778 | 91623819 | Carcass weight (cold) QTL (1028) | 6 | 53644827 | 95672304 |
| ENSSSCG00000003627 | 6 | 91455778 | 91623819 | Body weight (slaughter) QTL (1031) | 6 | 53644827 | 95672304 |
| ENSSSCG00000003627 | 6 | 91455778 | 91623819 | Body weight (slaughter) QTL (1035) | 6 | 53644827 | 95672304 |
| ENSSSCG00000003627 | 6 | 91455778 | 91623819 | Creatine kinase level QTL (1045) | 6 | 53644827 | 95672304 |
| ENSSSCG00000003627 | 6 | 91455778 | 91623819 | Average backfat thickness QTL (148) | 6 | 55524105 | 1.33E+08 |
| ENSSSCG00000003627 | 6 | 91455778 | 91623819 | Average backfat thickness QTL (149) | 6 | 55524105 | 1.33E+08 |
| ENSSSCG00000003627 | 6 | 91455778 | 91623819 | Intramuscular fat content QTL (150) | 6 | 55524105 | 1.33E+08 |
| ENSSSCG00000003627 | 6 | 91455778 | 91623819 | Intramuscular fat content QTL (151) | 6 | 55524105 | 1.33E+08 |
| ENSSSCG00000003627 | 6 | 91455778 | 91623819 | Alkaline phosphatase activity QTL (6347) | 6 | 58684707 | 94305556 |
| ENSSSCG00000003627 | 6 | 91455778 | 91623819 | Calcium level QTL (6376) | 6 | 58684707 | 94305556 |
| ENSSSCG00000003627 | 6 | 91455778 | 91623819 | Backfat between 3rd and 4th last ribs QTL (4167) | 6 | 59368081 | 1.24E+08 |
| ENSSSCG00000003627 | 6 | 91455778 | 91623819 | Average backfat thickness QTL (680) | 6 | 60649407 | 92255436 |
| ENSSSCG00000003627 | 6 | 91455778 | 91623819 | body weight (17 weeks) QTL (184) | 6 | 61759889 | 1.15E+08 |
| ENSSSCG00000003627 | 6 | 91455778 | 91623819 | body weight (13 weeks) QTL (183) | 6 | 61759889 | 1.07E+08 |
| ENSSSCG00000003627 | 6 | 91455778 | 91623819 | Average daily gain QTL (185) | 6 | 61759889 | 97209894 |
| ENSSSCG00000003627 | 6 | 91455778 | 91623819 | Intramuscular fat content QTL (4169) | 6 | 61930732 | 1.26E+08 |
| ENSSSCG00000003627 | 6 | 91455778 | 91623819 | Conductivity 24 hours post-mortem QTL (988) | 6 | 65005913 | 95672304 |
| ENSSSCG00000003627 | 6 | 91455778 | 91623819 | Loin muscle area QTL (1006) | 6 | 65005913 | 95672304 |
| ENSSSCG00000003627 | 6 | 91455778 | 91623819 | Body weight (20 weeks) QTL (29696) | 6 | 65347600 | 1.28E+08 |
| ENSSSCG00000003627 | 6 | 91455778 | 91623819 | Carcass weight (hot) QTL (29697) | 6 | 65347600 | 1.28E+08 |
| ENSSSCG00000003627 | 6 | 91455778 | 91623819 | subjective boar flavor in lean QTL (4219) | 6 | 65347600 | 1.28E+08 |
| ENSSSCG00000003627 | 6 | 91455778 | 91623819 | subjective pork flavor in fat QTL (4223) | 6 | 65347600 | 99089172 |
| ENSSSCG00000003627 | 6 | 91455778 | 91623819 | Intramuscular fat content QTL (314) | 6 | 87130134 | 1.38E+08 |
| ENSSSCG00000003630 | 6 | 92036834 | 92215149 | Loin muscle area QTL (3190) | 6 | 71156276 | 1.03E+08 |
| ENSSSCG00000003630 | 6 | 92036834 | 92215149 | Loin muscle area QTL (3191) | 6 | 71156276 | 1.03E+08 |
| ENSSSCG00000003630 | 6 | 92036834 | 92215149 | Estimated carcass lean content QTL (3192) | 6 | 71156276 | 1.03E+08 |
| ENSSSCG00000003630 | 6 | 92036834 | 92215149 | Empty body protein content QTL (3193) | 6 | 71156276 | 1.03E+08 |
| ENSSSCG00000003630 | 6 | 92036834 | 92215149 | Backfat at last rib QTL (3194) | 6 | 71156276 | 1.03E+08 |
| ENSSSCG00000003630 | 6 | 92036834 | 92215149 | Marbling QTL (3239) | 6 | 71156276 | 1.03E+08 |
| ENSSSCG00000003630 | 6 | 92036834 | 92215149 | Muscle fat content QTL (3240) | 6 | 71156276 | 1.03E+08 |
| ENSSSCG00000003630 | 6 | 92036834 | 92215149 | Backfat at tenth rib QTL (3242) | 6 | 71156276 | 1.03E+08 |
| ENSSSCG00000003630 | 6 | 92036834 | 92215149 | Backfat at last lumbar QTL (3243) | 6 | 71156276 | 1.03E+08 |
| ENSSSCG00000003630 | 6 | 92036834 | 92215149 | Empty body lipid content QTL (3646) | 6 | 71156276 | 1.03E+08 |
| ENSSSCG00000003630 | 6 | 92036834 | 92215149 | Loin muscle area QTL (3648) | 6 | 71156276 | 1.03E+08 |
| ENSSSCG00000003630 | 6 | 92036834 | 92215149 | Backfat at first rib QTL (681) | 6 | 76879530 | 92255436 |
| ENSSSCG00000003630 | 6 | 92036834 | 92215149 | Conductivity 24 hours post-mortem QTL (1016) | 6 | 77990012 | 98234955 |
| ENSSSCG00000003630 | 6 | 92036834 | 92215149 | Meat color-a QTL (1018) | 6 | 77990012 | 98234955 |
| ENSSSCG00000003630 | 6 | 92036834 | 92215149 | pH 45 minutes post mortem QTL (1019) | 6 | 77990012 | 98234955 |
| ENSSSCG00000003630 | 6 | 92036834 | 92215149 | pH 24 hr post-mortem (loin) QTL (1030) | 6 | 77990012 | 98234955 |
| ENSSSCG00000003630 | 6 | 92036834 | 92215149 | Loin muscle area QTL (1034) | 6 | 77990012 | 98234955 |
| ENSSSCG00000003630 | 6 | 92036834 | 92215149 | Meat color L* QTL (17556) | 6 | 79527602 | 96611942 |
| ENSSSCG00000003630 | 6 | 92036834 | 92215149 | Loin muscle area QTL (7298) | 6 | 80210976 | 99089172 |
| ENSSSCG00000003630 | 6 | 92036834 | 92215149 | Marbling QTL (21368) | 6 | 82859049 | 92853387 |
| ENSSSCG00000003630 | 6 | 92036834 | 92215149 | Meat color a* QTL (21369) | 6 | 82859049 | 92853387 |
| ENSSSCG00000003630 | 6 | 92036834 | 92215149 | Loin fat percentage QTL (21434) | 6 | 82859049 | 92853387 |
| ENSSSCG00000003630 | 6 | 92036834 | 92215149 | Loin muscle area QTL (4168) | 6 | 84567483 | 1.29E+08 |
| ENSSSCG00000003630 | 6 | 92036834 | 92215149 | Loin muscle depth QTL (673) | 6 | 86275917 | 1.05E+08 |
| ENSSSCG00000003630 | 6 | 92036834 | 92215149 | Smell intensity QTL (164) | 6 | 87130134 | 1.28E+08 |
| ENSSSCG00000003630 | 6 | 92036834 | 92215149 | Loin muscle area QTL (674) | 6 | 88838568 | 1.06E+08 |
| ENSSSCG00000003630 | 6 | 92036834 | 92215149 | pH 24 hr post-mortem (loin) QTL (1246) | 6 | 89692785 | 98234955 |
| ENSSSCG00000003630 | 6 | 92036834 | 92215149 | Meat color-a QTL (1248) | 6 | 89692785 | 98234955 |
| ENSSSCG00000003630 | 6 | 92036834 | 92215149 | Conductivity 24 hours post-mortem QTL (315) | 6 | 89692785 | 98234955 |
| ENSSSCG00000003630 | 6 | 92036834 | 92215149 | pH 45 minutes post mortem QTL (316) | 6 | 89692785 | 98234955 |
| ENSSSCG00000003630 | 6 | 92036834 | 92215149 | Creatine kinase level QTL (317) | 6 | 89692785 | 98234955 |
| ENSSSCG00000003630 | 6 | 92036834 | 92215149 | Average backfat thickness QTL (17999) | 6 | 89692785 | 95672304 |
| ENSSSCG00000003630 | 6 | 92036834 | 92215149 | Subcutanous fat area QTL (18005) | 6 | 89692785 | 95672304 |
| ENSSSCG00000003630 | 6 | 92036834 | 92215149 | Backfat at last rib QTL (16836) | 6 | 89692785 | 95672304 |
| ENSSSCG00000003630 | 6 | 92036834 | 92215149 | Loin muscle area QTL (152) | 6 | 89863628 | 1.33E+08 |
| ENSSSCG00000003630 | 6 | 92036834 | 92215149 | Loin muscle area QTL (153) | 6 | 89863628 | 1.33E+08 |
| ENSSSCG00000003630 | 6 | 92036834 | 92215149 | Average backfat thickness QTL (2865) | 6 | 89863628 | 1.03E+08 |
| ENSSSCG00000003630 | 6 | 92036834 | 92215149 | Muscle moisture percentage QTL (21706) | 6 | 90547002 | 1.06E+08 |
| ENSSSCG00000003630 | 6 | 92036834 | 92215149 | Marbling QTL (38089) | 6 | 6235784 | 1.28E+08 |
| ENSSSCG00000003630 | 6 | 92036834 | 92215149 | Meat color a* QTL (3921) | 6 | 6235784 | 94305556 |
| ENSSSCG00000003630 | 6 | 92036834 | 92215149 | Meat color a* QTL (3922) | 6 | 6235784 | 94305556 |
| ENSSSCG00000003630 | 6 | 92036834 | 92215149 | Meat color a* QTL (3923) | 6 | 6235784 | 94305556 |
| ENSSSCG00000003630 | 6 | 92036834 | 92215149 | Meat color a* QTL (3924) | 6 | 6235784 | 94305556 |
| ENSSSCG00000003630 | 6 | 92036834 | 92215149 | Body weight (end of test) QTL (5201) | 6 | 12044459 | 1.35E+08 |
| ENSSSCG00000003630 | 6 | 92036834 | 92215149 | Alkaline phosphatase activity QTL (15097) | 6 | 35450005 | 1.38E+08 |
| ENSSSCG00000003630 | 6 | 92036834 | 92215149 | Loin fat percentage QTL (38090) | 6 | 35450005 | 1.28E+08 |
| ENSSSCG00000003630 | 6 | 92036834 | 92215149 | Lactate dehydrogenase level QTL (15099) | 6 | 37585548 | 1.2E+08 |
| ENSSSCG00000003630 | 6 | 92036834 | 92215149 | Carcass length QTL (3873) | 6 | 38610608 | 1.2E+08 |
| ENSSSCG00000003630 | 6 | 92036834 | 92215149 | Average daily gain QTL (186) | 6 | 46981935 | 98234955 |
| ENSSSCG00000003630 | 6 | 92036834 | 92215149 | Shear force QTL (38091) | 6 | 50398803 | 1.38E+08 |
| ENSSSCG00000003630 | 6 | 92036834 | 92215149 | Average backfat thickness QTL (1004) | 6 | 53644827 | 95672304 |
| ENSSSCG00000003630 | 6 | 92036834 | 92215149 | Abdominal fat weight QTL (1007) | 6 | 53644827 | 95672304 |
| ENSSSCG00000003630 | 6 | 92036834 | 92215149 | Backfat at tenth rib QTL (1011) | 6 | 53644827 | 95672304 |
| ENSSSCG00000003630 | 6 | 92036834 | 92215149 | Carcass weight (cold) QTL (1028) | 6 | 53644827 | 95672304 |
| ENSSSCG00000003630 | 6 | 92036834 | 92215149 | Body weight (slaughter) QTL (1031) | 6 | 53644827 | 95672304 |
| ENSSSCG00000003630 | 6 | 92036834 | 92215149 | Body weight (slaughter) QTL (1035) | 6 | 53644827 | 95672304 |
| ENSSSCG00000003630 | 6 | 92036834 | 92215149 | Creatine kinase level QTL (1045) | 6 | 53644827 | 95672304 |
| ENSSSCG00000003630 | 6 | 92036834 | 92215149 | Average backfat thickness QTL (148) | 6 | 55524105 | 1.33E+08 |
| ENSSSCG00000003630 | 6 | 92036834 | 92215149 | Average backfat thickness QTL (149) | 6 | 55524105 | 1.33E+08 |
| ENSSSCG00000003630 | 6 | 92036834 | 92215149 | Intramuscular fat content QTL (150) | 6 | 55524105 | 1.33E+08 |
| ENSSSCG00000003630 | 6 | 92036834 | 92215149 | Intramuscular fat content QTL (151) | 6 | 55524105 | 1.33E+08 |
| ENSSSCG00000003630 | 6 | 92036834 | 92215149 | Alkaline phosphatase activity QTL (6347) | 6 | 58684707 | 94305556 |
| ENSSSCG00000003630 | 6 | 92036834 | 92215149 | Calcium level QTL (6376) | 6 | 58684707 | 94305556 |
| ENSSSCG00000003630 | 6 | 92036834 | 92215149 | Backfat between 3rd and 4th last ribs QTL (4167) | 6 | 59368081 | 1.24E+08 |
| ENSSSCG00000003630 | 6 | 92036834 | 92215149 | Average backfat thickness QTL (680) | 6 | 60649407 | 92255436 |
| ENSSSCG00000003630 | 6 | 92036834 | 92215149 | body weight (17 weeks) QTL (184) | 6 | 61759889 | 1.15E+08 |
| ENSSSCG00000003630 | 6 | 92036834 | 92215149 | body weight (13 weeks) QTL (183) | 6 | 61759889 | 1.07E+08 |
| ENSSSCG00000003630 | 6 | 92036834 | 92215149 | Average daily gain QTL (185) | 6 | 61759889 | 97209894 |
| ENSSSCG00000003630 | 6 | 92036834 | 92215149 | Intramuscular fat content QTL (4169) | 6 | 61930732 | 1.26E+08 |
| ENSSSCG00000003630 | 6 | 92036834 | 92215149 | Conductivity 24 hours post-mortem QTL (988) | 6 | 65005913 | 95672304 |
| ENSSSCG00000003630 | 6 | 92036834 | 92215149 | Loin muscle area QTL (1006) | 6 | 65005913 | 95672304 |
| ENSSSCG00000003630 | 6 | 92036834 | 92215149 | Body weight (20 weeks) QTL (29696) | 6 | 65347600 | 1.28E+08 |
| ENSSSCG00000003630 | 6 | 92036834 | 92215149 | Carcass weight (hot) QTL (29697) | 6 | 65347600 | 1.28E+08 |
| ENSSSCG00000003630 | 6 | 92036834 | 92215149 | subjective boar flavor in lean QTL (4219) | 6 | 65347600 | 1.28E+08 |
| ENSSSCG00000003630 | 6 | 92036834 | 92215149 | subjective pork flavor in fat QTL (4223) | 6 | 65347600 | 99089172 |
| ENSSSCG00000003630 | 6 | 92036834 | 92215149 | Intramuscular fat content QTL (314) | 6 | 87130134 | 1.38E+08 |
| ENSSSCG00000003670 | 6 | 95900086 | 95994044 | Backfat at last rib QTL (4956) | 6 | 94647243 | 99345437 |
| ENSSSCG00000003670 | 6 | 95900086 | 95994044 | Backfat between 3rd and 4th last ribs QTL (4957) | 6 | 95416038 | 99345437 |
| ENSSSCG00000003670 | 6 | 95900086 | 95994044 | Loin muscle area QTL (3190) | 6 | 71156276 | 1.03E+08 |
| ENSSSCG00000003670 | 6 | 95900086 | 95994044 | Loin muscle area QTL (3191) | 6 | 71156276 | 1.03E+08 |
| ENSSSCG00000003670 | 6 | 95900086 | 95994044 | Estimated carcass lean content QTL (3192) | 6 | 71156276 | 1.03E+08 |
| ENSSSCG00000003670 | 6 | 95900086 | 95994044 | Empty body protein content QTL (3193) | 6 | 71156276 | 1.03E+08 |
| ENSSSCG00000003670 | 6 | 95900086 | 95994044 | Backfat at last rib QTL (3194) | 6 | 71156276 | 1.03E+08 |
| ENSSSCG00000003670 | 6 | 95900086 | 95994044 | Marbling QTL (3239) | 6 | 71156276 | 1.03E+08 |
| ENSSSCG00000003670 | 6 | 95900086 | 95994044 | Muscle fat content QTL (3240) | 6 | 71156276 | 1.03E+08 |
| ENSSSCG00000003670 | 6 | 95900086 | 95994044 | Backfat at tenth rib QTL (3242) | 6 | 71156276 | 1.03E+08 |
| ENSSSCG00000003670 | 6 | 95900086 | 95994044 | Backfat at last lumbar QTL (3243) | 6 | 71156276 | 1.03E+08 |
| ENSSSCG00000003670 | 6 | 95900086 | 95994044 | Empty body lipid content QTL (3646) | 6 | 71156276 | 1.03E+08 |
| ENSSSCG00000003670 | 6 | 95900086 | 95994044 | Loin muscle area QTL (3648) | 6 | 71156276 | 1.03E+08 |
| ENSSSCG00000003670 | 6 | 95900086 | 95994044 | Conductivity 24 hours post-mortem QTL (1016) | 6 | 77990012 | 98234955 |
| ENSSSCG00000003670 | 6 | 95900086 | 95994044 | Meat color-a QTL (1018) | 6 | 77990012 | 98234955 |
| ENSSSCG00000003670 | 6 | 95900086 | 95994044 | pH 45 minutes post mortem QTL (1019) | 6 | 77990012 | 98234955 |
| ENSSSCG00000003670 | 6 | 95900086 | 95994044 | pH 24 hr post-mortem (loin) QTL (1030) | 6 | 77990012 | 98234955 |
| ENSSSCG00000003670 | 6 | 95900086 | 95994044 | Loin muscle area QTL (1034) | 6 | 77990012 | 98234955 |
| ENSSSCG00000003670 | 6 | 95900086 | 95994044 | Meat color L* QTL (17556) | 6 | 79527602 | 96611942 |
| ENSSSCG00000003670 | 6 | 95900086 | 95994044 | Loin muscle area QTL (7298) | 6 | 80210976 | 99089172 |
| ENSSSCG00000003670 | 6 | 95900086 | 95994044 | Loin muscle area QTL (4168) | 6 | 84567483 | 1.29E+08 |
| ENSSSCG00000003670 | 6 | 95900086 | 95994044 | Loin muscle depth QTL (673) | 6 | 86275917 | 1.05E+08 |
| ENSSSCG00000003670 | 6 | 95900086 | 95994044 | Smell intensity QTL (164) | 6 | 87130134 | 1.28E+08 |
| ENSSSCG00000003670 | 6 | 95900086 | 95994044 | Loin muscle area QTL (674) | 6 | 88838568 | 1.06E+08 |
| ENSSSCG00000003670 | 6 | 95900086 | 95994044 | pH 24 hr post-mortem (loin) QTL (1246) | 6 | 89692785 | 98234955 |
| ENSSSCG00000003670 | 6 | 95900086 | 95994044 | Meat color-a QTL (1248) | 6 | 89692785 | 98234955 |
| ENSSSCG00000003670 | 6 | 95900086 | 95994044 | Conductivity 24 hours post-mortem QTL (315) | 6 | 89692785 | 98234955 |
| ENSSSCG00000003670 | 6 | 95900086 | 95994044 | pH 45 minutes post mortem QTL (316) | 6 | 89692785 | 98234955 |
| ENSSSCG00000003670 | 6 | 95900086 | 95994044 | Creatine kinase level QTL (317) | 6 | 89692785 | 98234955 |
| ENSSSCG00000003670 | 6 | 95900086 | 95994044 | Loin muscle area QTL (152) | 6 | 89863628 | 1.33E+08 |
| ENSSSCG00000003670 | 6 | 95900086 | 95994044 | Loin muscle area QTL (153) | 6 | 89863628 | 1.33E+08 |
| ENSSSCG00000003670 | 6 | 95900086 | 95994044 | Average backfat thickness QTL (2865) | 6 | 89863628 | 1.03E+08 |
| ENSSSCG00000003670 | 6 | 95900086 | 95994044 | Muscle moisture percentage QTL (21706) | 6 | 90547002 | 1.06E+08 |
| ENSSSCG00000003670 | 6 | 95900086 | 95994044 | Backfat at tenth rib QTL (16912) | 6 | 92853387 | 1.03E+08 |
| ENSSSCG00000003670 | 6 | 95900086 | 95994044 | Backfat at tenth rib QTL (16913) | 6 | 92853387 | 1.03E+08 |
| ENSSSCG00000003670 | 6 | 95900086 | 95994044 | Backfat at last rib QTL (16914) | 6 | 92853387 | 1.03E+08 |
| ENSSSCG00000003670 | 6 | 95900086 | 95994044 | Backfat at tenth rib QTL (16915) | 6 | 92853387 | 1.03E+08 |
| ENSSSCG00000003670 | 6 | 95900086 | 95994044 | Backfat at last rib QTL (16916) | 6 | 92853387 | 1.03E+08 |
| ENSSSCG00000003670 | 6 | 95900086 | 95994044 | Backfat at last rib QTL (16917) | 6 | 92853387 | 1.03E+08 |
| ENSSSCG00000003670 | 6 | 95900086 | 95994044 | Backfat at last rib QTL (16918) | 6 | 92853387 | 1.03E+08 |
| ENSSSCG00000003670 | 6 | 95900086 | 95994044 | Backfat at last rib QTL (16919) | 6 | 92853387 | 1.03E+08 |
| ENSSSCG00000003670 | 6 | 95900086 | 95994044 | Backfat at first rib QTL (21370) | 6 | 92853387 | 1.03E+08 |
| ENSSSCG00000003670 | 6 | 95900086 | 95994044 | Backfat at tenth rib QTL (21371) | 6 | 92853387 | 1.03E+08 |
| ENSSSCG00000003670 | 6 | 95900086 | 95994044 | Carcass length QTL (21372) | 6 | 92853387 | 1.03E+08 |
| ENSSSCG00000003670 | 6 | 95900086 | 95994044 | Backfat at last lumbar QTL (21374) | 6 | 92853387 | 1.03E+08 |
| ENSSSCG00000003670 | 6 | 95900086 | 95994044 | Fat area percentage in carcass QTL (18628) | 6 | 92853387 | 1.03E+08 |
| ENSSSCG00000003670 | 6 | 95900086 | 95994044 | Intramuscular fat content QTL (3862) | 6 | 94305556 | 1.28E+08 |
| ENSSSCG00000003670 | 6 | 95900086 | 95994044 | Oleic acid content QTL (15879) | 6 | 94561821 | 1.28E+08 |
| ENSSSCG00000003670 | 6 | 95900086 | 95994044 | Loin muscle depth QTL (4961) | 6 | 94647243 | 1.02E+08 |
| ENSSSCG00000003670 | 6 | 95900086 | 95994044 | Marbling QTL (38089) | 6 | 6235784 | 1.28E+08 |
| ENSSSCG00000003670 | 6 | 95900086 | 95994044 | Body weight (end of test) QTL (5201) | 6 | 12044459 | 1.35E+08 |
| ENSSSCG00000003670 | 6 | 95900086 | 95994044 | Alkaline phosphatase activity QTL (15097) | 6 | 35450005 | 1.38E+08 |
| ENSSSCG00000003670 | 6 | 95900086 | 95994044 | Loin fat percentage QTL (38090) | 6 | 35450005 | 1.28E+08 |
| ENSSSCG00000003670 | 6 | 95900086 | 95994044 | Lactate dehydrogenase level QTL (15099) | 6 | 37585548 | 1.2E+08 |
| ENSSSCG00000003670 | 6 | 95900086 | 95994044 | Carcass length QTL (3873) | 6 | 38610608 | 1.2E+08 |
| ENSSSCG00000003670 | 6 | 95900086 | 95994044 | Average daily gain QTL (186) | 6 | 46981935 | 98234955 |
| ENSSSCG00000003670 | 6 | 95900086 | 95994044 | Shear force QTL (38091) | 6 | 50398803 | 1.38E+08 |
| ENSSSCG00000003670 | 6 | 95900086 | 95994044 | Average backfat thickness QTL (148) | 6 | 55524105 | 1.33E+08 |
| ENSSSCG00000003670 | 6 | 95900086 | 95994044 | Average backfat thickness QTL (149) | 6 | 55524105 | 1.33E+08 |
| ENSSSCG00000003670 | 6 | 95900086 | 95994044 | Intramuscular fat content QTL (150) | 6 | 55524105 | 1.33E+08 |
| ENSSSCG00000003670 | 6 | 95900086 | 95994044 | Intramuscular fat content QTL (151) | 6 | 55524105 | 1.33E+08 |
| ENSSSCG00000003670 | 6 | 95900086 | 95994044 | Backfat between 3rd and 4th last ribs QTL (4167) | 6 | 59368081 | 1.24E+08 |
| ENSSSCG00000003670 | 6 | 95900086 | 95994044 | body weight (17 weeks) QTL (184) | 6 | 61759889 | 1.15E+08 |
| ENSSSCG00000003670 | 6 | 95900086 | 95994044 | body weight (13 weeks) QTL (183) | 6 | 61759889 | 1.07E+08 |
| ENSSSCG00000003670 | 6 | 95900086 | 95994044 | Average daily gain QTL (185) | 6 | 61759889 | 97209894 |
| ENSSSCG00000003670 | 6 | 95900086 | 95994044 | Intramuscular fat content QTL (4169) | 6 | 61930732 | 1.26E+08 |
| ENSSSCG00000003670 | 6 | 95900086 | 95994044 | Body weight (20 weeks) QTL (29696) | 6 | 65347600 | 1.28E+08 |
| ENSSSCG00000003670 | 6 | 95900086 | 95994044 | Carcass weight (hot) QTL (29697) | 6 | 65347600 | 1.28E+08 |
| ENSSSCG00000003670 | 6 | 95900086 | 95994044 | subjective boar flavor in lean QTL (4219) | 6 | 65347600 | 1.28E+08 |
| ENSSSCG00000003670 | 6 | 95900086 | 95994044 | subjective pork flavor in fat QTL (4223) | 6 | 65347600 | 99089172 |
| ENSSSCG00000003670 | 6 | 95900086 | 95994044 | Intramuscular fat content QTL (314) | 6 | 87130134 | 1.38E+08 |
| ENSSSCG00000038465 | 6 | 98207580 | 98254288 | Backfat at last rib QTL (4956) | 6 | 94647243 | 99345437 |
| ENSSSCG00000038465 | 6 | 98207580 | 98254288 | Backfat between 3rd and 4th last ribs QTL (4957) | 6 | 95416038 | 99345437 |
| ENSSSCG00000038465 | 6 | 98207580 | 98254288 | Loin muscle area QTL (3190) | 6 | 71156276 | 1.03E+08 |
| ENSSSCG00000038465 | 6 | 98207580 | 98254288 | Loin muscle area QTL (3191) | 6 | 71156276 | 1.03E+08 |
| ENSSSCG00000038465 | 6 | 98207580 | 98254288 | Estimated carcass lean content QTL (3192) | 6 | 71156276 | 1.03E+08 |
| ENSSSCG00000038465 | 6 | 98207580 | 98254288 | Empty body protein content QTL (3193) | 6 | 71156276 | 1.03E+08 |
| ENSSSCG00000038465 | 6 | 98207580 | 98254288 | Backfat at last rib QTL (3194) | 6 | 71156276 | 1.03E+08 |
| ENSSSCG00000038465 | 6 | 98207580 | 98254288 | Marbling QTL (3239) | 6 | 71156276 | 1.03E+08 |
| ENSSSCG00000038465 | 6 | 98207580 | 98254288 | Muscle fat content QTL (3240) | 6 | 71156276 | 1.03E+08 |
| ENSSSCG00000038465 | 6 | 98207580 | 98254288 | Backfat at tenth rib QTL (3242) | 6 | 71156276 | 1.03E+08 |
| ENSSSCG00000038465 | 6 | 98207580 | 98254288 | Backfat at last lumbar QTL (3243) | 6 | 71156276 | 1.03E+08 |
| ENSSSCG00000038465 | 6 | 98207580 | 98254288 | Empty body lipid content QTL (3646) | 6 | 71156276 | 1.03E+08 |
| ENSSSCG00000038465 | 6 | 98207580 | 98254288 | Loin muscle area QTL (3648) | 6 | 71156276 | 1.03E+08 |
| ENSSSCG00000038465 | 6 | 98207580 | 98254288 | Conductivity 24 hours post-mortem QTL (1016) | 6 | 77990012 | 98234955 |
| ENSSSCG00000038465 | 6 | 98207580 | 98254288 | Meat color-a QTL (1018) | 6 | 77990012 | 98234955 |
| ENSSSCG00000038465 | 6 | 98207580 | 98254288 | pH 45 minutes post mortem QTL (1019) | 6 | 77990012 | 98234955 |
| ENSSSCG00000038465 | 6 | 98207580 | 98254288 | pH 24 hr post-mortem (loin) QTL (1030) | 6 | 77990012 | 98234955 |
| ENSSSCG00000038465 | 6 | 98207580 | 98254288 | Loin muscle area QTL (1034) | 6 | 77990012 | 98234955 |
| ENSSSCG00000038465 | 6 | 98207580 | 98254288 | Loin muscle area QTL (7298) | 6 | 80210976 | 99089172 |
| ENSSSCG00000038465 | 6 | 98207580 | 98254288 | Loin muscle area QTL (4168) | 6 | 84567483 | 1.29E+08 |
| ENSSSCG00000038465 | 6 | 98207580 | 98254288 | Loin muscle depth QTL (673) | 6 | 86275917 | 1.05E+08 |
| ENSSSCG00000038465 | 6 | 98207580 | 98254288 | Smell intensity QTL (164) | 6 | 87130134 | 1.28E+08 |
| ENSSSCG00000038465 | 6 | 98207580 | 98254288 | Loin muscle area QTL (674) | 6 | 88838568 | 1.06E+08 |
| ENSSSCG00000038465 | 6 | 98207580 | 98254288 | pH 24 hr post-mortem (loin) QTL (1246) | 6 | 89692785 | 98234955 |
| ENSSSCG00000038465 | 6 | 98207580 | 98254288 | Meat color-a QTL (1248) | 6 | 89692785 | 98234955 |
| ENSSSCG00000038465 | 6 | 98207580 | 98254288 | Conductivity 24 hours post-mortem QTL (315) | 6 | 89692785 | 98234955 |
| ENSSSCG00000038465 | 6 | 98207580 | 98254288 | pH 45 minutes post mortem QTL (316) | 6 | 89692785 | 98234955 |
| ENSSSCG00000038465 | 6 | 98207580 | 98254288 | Creatine kinase level QTL (317) | 6 | 89692785 | 98234955 |
| ENSSSCG00000038465 | 6 | 98207580 | 98254288 | Loin muscle area QTL (152) | 6 | 89863628 | 1.33E+08 |
| ENSSSCG00000038465 | 6 | 98207580 | 98254288 | Loin muscle area QTL (153) | 6 | 89863628 | 1.33E+08 |
| ENSSSCG00000038465 | 6 | 98207580 | 98254288 | Average backfat thickness QTL (2865) | 6 | 89863628 | 1.03E+08 |
| ENSSSCG00000038465 | 6 | 98207580 | 98254288 | Muscle moisture percentage QTL (21706) | 6 | 90547002 | 1.06E+08 |
| ENSSSCG00000038465 | 6 | 98207580 | 98254288 | Backfat at tenth rib QTL (16912) | 6 | 92853387 | 1.03E+08 |
| ENSSSCG00000038465 | 6 | 98207580 | 98254288 | Backfat at tenth rib QTL (16913) | 6 | 92853387 | 1.03E+08 |
| ENSSSCG00000038465 | 6 | 98207580 | 98254288 | Backfat at last rib QTL (16914) | 6 | 92853387 | 1.03E+08 |
| ENSSSCG00000038465 | 6 | 98207580 | 98254288 | Backfat at tenth rib QTL (16915) | 6 | 92853387 | 1.03E+08 |
| ENSSSCG00000038465 | 6 | 98207580 | 98254288 | Backfat at last rib QTL (16916) | 6 | 92853387 | 1.03E+08 |
| ENSSSCG00000038465 | 6 | 98207580 | 98254288 | Backfat at last rib QTL (16917) | 6 | 92853387 | 1.03E+08 |
| ENSSSCG00000038465 | 6 | 98207580 | 98254288 | Backfat at last rib QTL (16918) | 6 | 92853387 | 1.03E+08 |
| ENSSSCG00000038465 | 6 | 98207580 | 98254288 | Backfat at last rib QTL (16919) | 6 | 92853387 | 1.03E+08 |
| ENSSSCG00000038465 | 6 | 98207580 | 98254288 | Backfat at first rib QTL (21370) | 6 | 92853387 | 1.03E+08 |
| ENSSSCG00000038465 | 6 | 98207580 | 98254288 | Backfat at tenth rib QTL (21371) | 6 | 92853387 | 1.03E+08 |
| ENSSSCG00000038465 | 6 | 98207580 | 98254288 | Carcass length QTL (21372) | 6 | 92853387 | 1.03E+08 |
| ENSSSCG00000038465 | 6 | 98207580 | 98254288 | Backfat at last lumbar QTL (21374) | 6 | 92853387 | 1.03E+08 |
| ENSSSCG00000038465 | 6 | 98207580 | 98254288 | Fat area percentage in carcass QTL (18628) | 6 | 92853387 | 1.03E+08 |
| ENSSSCG00000038465 | 6 | 98207580 | 98254288 | Intramuscular fat content QTL (3862) | 6 | 94305556 | 1.28E+08 |
| ENSSSCG00000038465 | 6 | 98207580 | 98254288 | Oleic acid content QTL (15879) | 6 | 94561821 | 1.28E+08 |
| ENSSSCG00000038465 | 6 | 98207580 | 98254288 | Loin muscle depth QTL (4961) | 6 | 94647243 | 1.02E+08 |
| ENSSSCG00000038465 | 6 | 98207580 | 98254288 | Loin muscle area QTL (4960) | 6 | 96184834 | 1.02E+08 |
| ENSSSCG00000038465 | 6 | 98207580 | 98254288 | Backfat at first rib QTL (4955) | 6 | 96184834 | 1.01E+08 |
| ENSSSCG00000038465 | 6 | 98207580 | 98254288 | Intramuscular fat content QTL (4958) | 6 | 97807846 | 1.03E+08 |
| ENSSSCG00000038465 | 6 | 98207580 | 98254288 | Marbling QTL (38089) | 6 | 6235784 | 1.28E+08 |
| ENSSSCG00000038465 | 6 | 98207580 | 98254288 | Body weight (end of test) QTL (5201) | 6 | 12044459 | 1.35E+08 |
| ENSSSCG00000038465 | 6 | 98207580 | 98254288 | Alkaline phosphatase activity QTL (15097) | 6 | 35450005 | 1.38E+08 |
| ENSSSCG00000038465 | 6 | 98207580 | 98254288 | Loin fat percentage QTL (38090) | 6 | 35450005 | 1.28E+08 |
| ENSSSCG00000038465 | 6 | 98207580 | 98254288 | Lactate dehydrogenase level QTL (15099) | 6 | 37585548 | 1.2E+08 |
| ENSSSCG00000038465 | 6 | 98207580 | 98254288 | Carcass length QTL (3873) | 6 | 38610608 | 1.2E+08 |
| ENSSSCG00000038465 | 6 | 98207580 | 98254288 | Average daily gain QTL (186) | 6 | 46981935 | 98234955 |
| ENSSSCG00000038465 | 6 | 98207580 | 98254288 | Shear force QTL (38091) | 6 | 50398803 | 1.38E+08 |
| ENSSSCG00000038465 | 6 | 98207580 | 98254288 | Average backfat thickness QTL (148) | 6 | 55524105 | 1.33E+08 |
| ENSSSCG00000038465 | 6 | 98207580 | 98254288 | Average backfat thickness QTL (149) | 6 | 55524105 | 1.33E+08 |
| ENSSSCG00000038465 | 6 | 98207580 | 98254288 | Intramuscular fat content QTL (150) | 6 | 55524105 | 1.33E+08 |
| ENSSSCG00000038465 | 6 | 98207580 | 98254288 | Intramuscular fat content QTL (151) | 6 | 55524105 | 1.33E+08 |
| ENSSSCG00000038465 | 6 | 98207580 | 98254288 | Backfat between 3rd and 4th last ribs QTL (4167) | 6 | 59368081 | 1.24E+08 |
| ENSSSCG00000038465 | 6 | 98207580 | 98254288 | body weight (17 weeks) QTL (184) | 6 | 61759889 | 1.15E+08 |
| ENSSSCG00000038465 | 6 | 98207580 | 98254288 | body weight (13 weeks) QTL (183) | 6 | 61759889 | 1.07E+08 |
| ENSSSCG00000038465 | 6 | 98207580 | 98254288 | Intramuscular fat content QTL (4169) | 6 | 61930732 | 1.26E+08 |
| ENSSSCG00000038465 | 6 | 98207580 | 98254288 | Body weight (20 weeks) QTL (29696) | 6 | 65347600 | 1.28E+08 |
| ENSSSCG00000038465 | 6 | 98207580 | 98254288 | Carcass weight (hot) QTL (29697) | 6 | 65347600 | 1.28E+08 |
| ENSSSCG00000038465 | 6 | 98207580 | 98254288 | subjective boar flavor in lean QTL (4219) | 6 | 65347600 | 1.28E+08 |
| ENSSSCG00000038465 | 6 | 98207580 | 98254288 | subjective pork flavor in fat QTL (4223) | 6 | 65347600 | 99089172 |
| ENSSSCG00000038465 | 6 | 98207580 | 98254288 | Intramuscular fat content QTL (314) | 6 | 87130134 | 1.38E+08 |
| ENSSSCG00000003693 | 6 | 1.03E+08 | 1.04E+08 | Loin muscle area QTL (4168) | 6 | 84567483 | 1.29E+08 |
| ENSSSCG00000003693 | 6 | 1.03E+08 | 1.04E+08 | Loin muscle depth QTL (673) | 6 | 86275917 | 1.05E+08 |
| ENSSSCG00000003693 | 6 | 1.03E+08 | 1.04E+08 | Smell intensity QTL (164) | 6 | 87130134 | 1.28E+08 |
| ENSSSCG00000003693 | 6 | 1.03E+08 | 1.04E+08 | Loin muscle area QTL (674) | 6 | 88838568 | 1.06E+08 |
| ENSSSCG00000003693 | 6 | 1.03E+08 | 1.04E+08 | Loin muscle area QTL (152) | 6 | 89863628 | 1.33E+08 |
| ENSSSCG00000003693 | 6 | 1.03E+08 | 1.04E+08 | Loin muscle area QTL (153) | 6 | 89863628 | 1.33E+08 |
| ENSSSCG00000003693 | 6 | 1.03E+08 | 1.04E+08 | Muscle moisture percentage QTL (21706) | 6 | 90547002 | 1.06E+08 |
| ENSSSCG00000003693 | 6 | 1.03E+08 | 1.04E+08 | Intramuscular fat content QTL (3862) | 6 | 94305556 | 1.28E+08 |
| ENSSSCG00000003693 | 6 | 1.03E+08 | 1.04E+08 | Oleic acid content QTL (15879) | 6 | 94561821 | 1.28E+08 |
| ENSSSCG00000003693 | 6 | 1.03E+08 | 1.04E+08 | skatole, sensory panel QTL (4218) | 6 | 99089172 | 1.28E+08 |
| ENSSSCG00000003693 | 6 | 1.03E+08 | 1.04E+08 | Muscle moisture percentage QTL (12057) | 6 | 99089172 | 1.28E+08 |
| ENSSSCG00000003693 | 6 | 1.03E+08 | 1.04E+08 | muscle protein percentage QTL (12058) | 6 | 99089172 | 1.28E+08 |
| ENSSSCG00000003693 | 6 | 1.03E+08 | 1.04E+08 | Intramuscular fat content QTL (12059) | 6 | 99089172 | 1.28E+08 |
| ENSSSCG00000003693 | 6 | 1.03E+08 | 1.04E+08 | Cholesterol level in meat QTL (12060) | 6 | 99089172 | 1.28E+08 |
| ENSSSCG00000003693 | 6 | 1.03E+08 | 1.04E+08 | Flavor score QTL (12061) | 6 | 99089172 | 1.28E+08 |
| ENSSSCG00000003693 | 6 | 1.03E+08 | 1.04E+08 | Average daily gain QTL (16851) | 6 | 1E+08 | 1.27E+08 |
| ENSSSCG00000003693 | 6 | 1.03E+08 | 1.04E+08 | Backfat at last rib QTL (17780) | 6 | 1.03E+08 | 1.33E+08 |
| ENSSSCG00000003693 | 6 | 1.03E+08 | 1.04E+08 | Backfat at last rib QTL (17781) | 6 | 1.03E+08 | 1.33E+08 |
| ENSSSCG00000003693 | 6 | 1.03E+08 | 1.04E+08 | Empty body lipid content QTL (3317) | 6 | 1.03E+08 | 1.28E+08 |
| ENSSSCG00000003693 | 6 | 1.03E+08 | 1.04E+08 | Backfat at last rib QTL (3649) | 6 | 1.03E+08 | 1.28E+08 |
| ENSSSCG00000003693 | 6 | 1.03E+08 | 1.04E+08 | Backfat linear at last rib QTL (3650) | 6 | 1.03E+08 | 1.28E+08 |
| ENSSSCG00000003693 | 6 | 1.03E+08 | 1.04E+08 | Backfat at tenth rib QTL (3651) | 6 | 1.03E+08 | 1.28E+08 |
| ENSSSCG00000003693 | 6 | 1.03E+08 | 1.04E+08 | Backfat linear at tenth rib QTL (3652) | 6 | 1.03E+08 | 1.28E+08 |
| ENSSSCG00000003693 | 6 | 1.03E+08 | 1.04E+08 | Backfat at tenth rib QTL (3195) | 6 | 1.03E+08 | 1.28E+08 |
| ENSSSCG00000003693 | 6 | 1.03E+08 | 1.04E+08 | Backfat at tenth rib QTL (3196) | 6 | 1.03E+08 | 1.28E+08 |
| ENSSSCG00000003693 | 6 | 1.03E+08 | 1.04E+08 | Backfat at last rib QTL (3197) | 6 | 1.03E+08 | 1.28E+08 |
| ENSSSCG00000003693 | 6 | 1.03E+08 | 1.04E+08 | Backfat at last rib QTL (3198) | 6 | 1.03E+08 | 1.28E+08 |
| ENSSSCG00000003693 | 6 | 1.03E+08 | 1.04E+08 | Backfat at last rib QTL (3199) | 6 | 1.03E+08 | 1.28E+08 |
| ENSSSCG00000003693 | 6 | 1.03E+08 | 1.04E+08 | Backfat at tenth rib QTL (3200) | 6 | 1.03E+08 | 1.28E+08 |
| ENSSSCG00000003693 | 6 | 1.03E+08 | 1.04E+08 | Backfat at last rib QTL (3201) | 6 | 1.03E+08 | 1.28E+08 |
| ENSSSCG00000003693 | 6 | 1.03E+08 | 1.04E+08 | Backfat at tenth rib QTL (3202) | 6 | 1.03E+08 | 1.28E+08 |
| ENSSSCG00000003693 | 6 | 1.03E+08 | 1.04E+08 | Backfat at tenth rib QTL (3203) | 6 | 1.03E+08 | 1.28E+08 |
| ENSSSCG00000003693 | 6 | 1.03E+08 | 1.04E+08 | Loin muscle area QTL (3204) | 6 | 1.03E+08 | 1.28E+08 |
| ENSSSCG00000003693 | 6 | 1.03E+08 | 1.04E+08 | Carcass length QTL (3244) | 6 | 1.03E+08 | 1.28E+08 |
| ENSSSCG00000003693 | 6 | 1.03E+08 | 1.04E+08 | Backfat at last rib QTL (3246) | 6 | 1.03E+08 | 1.28E+08 |
| ENSSSCG00000003693 | 6 | 1.03E+08 | 1.04E+08 | muscle protein percentage QTL (3247) | 6 | 1.03E+08 | 1.28E+08 |
| ENSSSCG00000003693 | 6 | 1.03E+08 | 1.04E+08 | Loin muscle area QTL (3249) | 6 | 1.03E+08 | 1.28E+08 |
| ENSSSCG00000003693 | 6 | 1.03E+08 | 1.04E+08 | Intramuscular fat content QTL (2854) | 6 | 1.03E+08 | 1.28E+08 |
| ENSSSCG00000003693 | 6 | 1.03E+08 | 1.04E+08 | External fat on loin QTL (2857) | 6 | 1.03E+08 | 1.28E+08 |
| ENSSSCG00000003693 | 6 | 1.03E+08 | 1.04E+08 | Backfat at tenth rib QTL (16921) | 6 | 1.03E+08 | 1.28E+08 |
| ENSSSCG00000003693 | 6 | 1.03E+08 | 1.04E+08 | Backfat at tenth rib QTL (16922) | 6 | 1.03E+08 | 1.28E+08 |
| ENSSSCG00000003693 | 6 | 1.03E+08 | 1.04E+08 | Empty body lipid content QTL (16923) | 6 | 1.03E+08 | 1.28E+08 |
| ENSSSCG00000003693 | 6 | 1.03E+08 | 1.04E+08 | Backfat at last rib QTL (21377) | 6 | 1.03E+08 | 1.28E+08 |
| ENSSSCG00000003693 | 6 | 1.03E+08 | 1.04E+08 | Loin muscle area QTL (21378) | 6 | 1.03E+08 | 1.28E+08 |
| ENSSSCG00000003693 | 6 | 1.03E+08 | 1.04E+08 | muscle protein percentage QTL (21379) | 6 | 1.03E+08 | 1.28E+08 |
| ENSSSCG00000003693 | 6 | 1.03E+08 | 1.04E+08 | Carcass weight (hot) QTL (21380) | 6 | 1.03E+08 | 1.28E+08 |
| ENSSSCG00000003693 | 6 | 1.03E+08 | 1.04E+08 | Carcass temperature (24 hr post-mortem) QTL (21381) | 6 | 1.03E+08 | 1.28E+08 |
| ENSSSCG00000003693 | 6 | 1.03E+08 | 1.04E+08 | Fat area percentage in carcass QTL (18630) | 6 | 1.03E+08 | 1.28E+08 |
| ENSSSCG00000003693 | 6 | 1.03E+08 | 1.04E+08 | Fat area percentage in carcass QTL (18635) | 6 | 1.03E+08 | 1.28E+08 |
| ENSSSCG00000003693 | 6 | 1.03E+08 | 1.04E+08 | Fat area percentage in carcass QTL (18637) | 6 | 1.03E+08 | 1.28E+08 |
| ENSSSCG00000003693 | 6 | 1.03E+08 | 1.04E+08 | Fat area percentage in carcass QTL (18642) | 6 | 1.03E+08 | 1.28E+08 |
| ENSSSCG00000003693 | 6 | 1.03E+08 | 1.04E+08 | Leptin level QTL (18643) | 6 | 1.03E+08 | 1.28E+08 |
| ENSSSCG00000003693 | 6 | 1.03E+08 | 1.04E+08 | Marbling QTL (38089) | 6 | 6235784 | 1.28E+08 |
| ENSSSCG00000003693 | 6 | 1.03E+08 | 1.04E+08 | Body weight (end of test) QTL (5201) | 6 | 12044459 | 1.35E+08 |
| ENSSSCG00000003693 | 6 | 1.03E+08 | 1.04E+08 | Alkaline phosphatase activity QTL (15097) | 6 | 35450005 | 1.38E+08 |
| ENSSSCG00000003693 | 6 | 1.03E+08 | 1.04E+08 | Loin fat percentage QTL (38090) | 6 | 35450005 | 1.28E+08 |
| ENSSSCG00000003693 | 6 | 1.03E+08 | 1.04E+08 | Lactate dehydrogenase level QTL (15099) | 6 | 37585548 | 1.2E+08 |
| ENSSSCG00000003693 | 6 | 1.03E+08 | 1.04E+08 | Carcass length QTL (3873) | 6 | 38610608 | 1.2E+08 |
| ENSSSCG00000003693 | 6 | 1.03E+08 | 1.04E+08 | Shear force QTL (38091) | 6 | 50398803 | 1.38E+08 |
| ENSSSCG00000003693 | 6 | 1.03E+08 | 1.04E+08 | Average backfat thickness QTL (148) | 6 | 55524105 | 1.33E+08 |
| ENSSSCG00000003693 | 6 | 1.03E+08 | 1.04E+08 | Average backfat thickness QTL (149) | 6 | 55524105 | 1.33E+08 |
| ENSSSCG00000003693 | 6 | 1.03E+08 | 1.04E+08 | Intramuscular fat content QTL (150) | 6 | 55524105 | 1.33E+08 |
| ENSSSCG00000003693 | 6 | 1.03E+08 | 1.04E+08 | Intramuscular fat content QTL (151) | 6 | 55524105 | 1.33E+08 |
| ENSSSCG00000003693 | 6 | 1.03E+08 | 1.04E+08 | Backfat between 3rd and 4th last ribs QTL (4167) | 6 | 59368081 | 1.24E+08 |
| ENSSSCG00000003693 | 6 | 1.03E+08 | 1.04E+08 | body weight (17 weeks) QTL (184) | 6 | 61759889 | 1.15E+08 |
| ENSSSCG00000003693 | 6 | 1.03E+08 | 1.04E+08 | body weight (13 weeks) QTL (183) | 6 | 61759889 | 1.07E+08 |
| ENSSSCG00000003693 | 6 | 1.03E+08 | 1.04E+08 | Intramuscular fat content QTL (4169) | 6 | 61930732 | 1.26E+08 |
| ENSSSCG00000003693 | 6 | 1.03E+08 | 1.04E+08 | Body weight (20 weeks) QTL (29696) | 6 | 65347600 | 1.28E+08 |
| ENSSSCG00000003693 | 6 | 1.03E+08 | 1.04E+08 | Carcass weight (hot) QTL (29697) | 6 | 65347600 | 1.28E+08 |
| ENSSSCG00000003693 | 6 | 1.03E+08 | 1.04E+08 | subjective boar flavor in lean QTL (4219) | 6 | 65347600 | 1.28E+08 |
| ENSSSCG00000003693 | 6 | 1.03E+08 | 1.04E+08 | Intramuscular fat content QTL (314) | 6 | 87130134 | 1.38E+08 |
| ENSSSCG00000003693 | 6 | 1.03E+08 | 1.04E+08 | body weight (10 weeks) QTL (182) | 6 | 1.02E+08 | 1.35E+08 |
| ENSSSCG00000003700 | 6 | 1.07E+08 | 1.07E+08 | Average backfat thickness QTL (66023) | 6 | 1.06E+08 | 1.07E+08 |
| ENSSSCG00000003700 | 6 | 1.07E+08 | 1.07E+08 | Loin muscle area QTL (4168) | 6 | 84567483 | 1.29E+08 |
| ENSSSCG00000003700 | 6 | 1.07E+08 | 1.07E+08 | Smell intensity QTL (164) | 6 | 87130134 | 1.28E+08 |
| ENSSSCG00000003700 | 6 | 1.07E+08 | 1.07E+08 | Loin muscle area QTL (152) | 6 | 89863628 | 1.33E+08 |
| ENSSSCG00000003700 | 6 | 1.07E+08 | 1.07E+08 | Loin muscle area QTL (153) | 6 | 89863628 | 1.33E+08 |
| ENSSSCG00000003700 | 6 | 1.07E+08 | 1.07E+08 | Intramuscular fat content QTL (3862) | 6 | 94305556 | 1.28E+08 |
| ENSSSCG00000003700 | 6 | 1.07E+08 | 1.07E+08 | Oleic acid content QTL (15879) | 6 | 94561821 | 1.28E+08 |
| ENSSSCG00000003700 | 6 | 1.07E+08 | 1.07E+08 | skatole, sensory panel QTL (4218) | 6 | 99089172 | 1.28E+08 |
| ENSSSCG00000003700 | 6 | 1.07E+08 | 1.07E+08 | Muscle moisture percentage QTL (12057) | 6 | 99089172 | 1.28E+08 |
| ENSSSCG00000003700 | 6 | 1.07E+08 | 1.07E+08 | muscle protein percentage QTL (12058) | 6 | 99089172 | 1.28E+08 |
| ENSSSCG00000003700 | 6 | 1.07E+08 | 1.07E+08 | Intramuscular fat content QTL (12059) | 6 | 99089172 | 1.28E+08 |
| ENSSSCG00000003700 | 6 | 1.07E+08 | 1.07E+08 | Cholesterol level in meat QTL (12060) | 6 | 99089172 | 1.28E+08 |
| ENSSSCG00000003700 | 6 | 1.07E+08 | 1.07E+08 | Flavor score QTL (12061) | 6 | 99089172 | 1.28E+08 |
| ENSSSCG00000003700 | 6 | 1.07E+08 | 1.07E+08 | Average daily gain QTL (16851) | 6 | 1E+08 | 1.27E+08 |
| ENSSSCG00000003700 | 6 | 1.07E+08 | 1.07E+08 | Backfat at last rib QTL (17780) | 6 | 1.03E+08 | 1.33E+08 |
| ENSSSCG00000003700 | 6 | 1.07E+08 | 1.07E+08 | Backfat at last rib QTL (17781) | 6 | 1.03E+08 | 1.33E+08 |
| ENSSSCG00000003700 | 6 | 1.07E+08 | 1.07E+08 | Empty body lipid content QTL (3317) | 6 | 1.03E+08 | 1.28E+08 |
| ENSSSCG00000003700 | 6 | 1.07E+08 | 1.07E+08 | Backfat at last rib QTL (3649) | 6 | 1.03E+08 | 1.28E+08 |
| ENSSSCG00000003700 | 6 | 1.07E+08 | 1.07E+08 | Backfat linear at last rib QTL (3650) | 6 | 1.03E+08 | 1.28E+08 |
| ENSSSCG00000003700 | 6 | 1.07E+08 | 1.07E+08 | Backfat at tenth rib QTL (3651) | 6 | 1.03E+08 | 1.28E+08 |
| ENSSSCG00000003700 | 6 | 1.07E+08 | 1.07E+08 | Backfat linear at tenth rib QTL (3652) | 6 | 1.03E+08 | 1.28E+08 |
| ENSSSCG00000003700 | 6 | 1.07E+08 | 1.07E+08 | Backfat at tenth rib QTL (3195) | 6 | 1.03E+08 | 1.28E+08 |
| ENSSSCG00000003700 | 6 | 1.07E+08 | 1.07E+08 | Backfat at tenth rib QTL (3196) | 6 | 1.03E+08 | 1.28E+08 |
| ENSSSCG00000003700 | 6 | 1.07E+08 | 1.07E+08 | Backfat at last rib QTL (3197) | 6 | 1.03E+08 | 1.28E+08 |
| ENSSSCG00000003700 | 6 | 1.07E+08 | 1.07E+08 | Backfat at last rib QTL (3198) | 6 | 1.03E+08 | 1.28E+08 |
| ENSSSCG00000003700 | 6 | 1.07E+08 | 1.07E+08 | Backfat at last rib QTL (3199) | 6 | 1.03E+08 | 1.28E+08 |
| ENSSSCG00000003700 | 6 | 1.07E+08 | 1.07E+08 | Backfat at tenth rib QTL (3200) | 6 | 1.03E+08 | 1.28E+08 |
| ENSSSCG00000003700 | 6 | 1.07E+08 | 1.07E+08 | Backfat at last rib QTL (3201) | 6 | 1.03E+08 | 1.28E+08 |
| ENSSSCG00000003700 | 6 | 1.07E+08 | 1.07E+08 | Backfat at tenth rib QTL (3202) | 6 | 1.03E+08 | 1.28E+08 |
| ENSSSCG00000003700 | 6 | 1.07E+08 | 1.07E+08 | Backfat at tenth rib QTL (3203) | 6 | 1.03E+08 | 1.28E+08 |
| ENSSSCG00000003700 | 6 | 1.07E+08 | 1.07E+08 | Loin muscle area QTL (3204) | 6 | 1.03E+08 | 1.28E+08 |
| ENSSSCG00000003700 | 6 | 1.07E+08 | 1.07E+08 | Carcass length QTL (3244) | 6 | 1.03E+08 | 1.28E+08 |
| ENSSSCG00000003700 | 6 | 1.07E+08 | 1.07E+08 | Backfat at last rib QTL (3246) | 6 | 1.03E+08 | 1.28E+08 |
| ENSSSCG00000003700 | 6 | 1.07E+08 | 1.07E+08 | muscle protein percentage QTL (3247) | 6 | 1.03E+08 | 1.28E+08 |
| ENSSSCG00000003700 | 6 | 1.07E+08 | 1.07E+08 | Loin muscle area QTL (3249) | 6 | 1.03E+08 | 1.28E+08 |
| ENSSSCG00000003700 | 6 | 1.07E+08 | 1.07E+08 | Intramuscular fat content QTL (2854) | 6 | 1.03E+08 | 1.28E+08 |
| ENSSSCG00000003700 | 6 | 1.07E+08 | 1.07E+08 | External fat on loin QTL (2857) | 6 | 1.03E+08 | 1.28E+08 |
| ENSSSCG00000003700 | 6 | 1.07E+08 | 1.07E+08 | Backfat at tenth rib QTL (16921) | 6 | 1.03E+08 | 1.28E+08 |
| ENSSSCG00000003700 | 6 | 1.07E+08 | 1.07E+08 | Backfat at tenth rib QTL (16922) | 6 | 1.03E+08 | 1.28E+08 |
| ENSSSCG00000003700 | 6 | 1.07E+08 | 1.07E+08 | Empty body lipid content QTL (16923) | 6 | 1.03E+08 | 1.28E+08 |
| ENSSSCG00000003700 | 6 | 1.07E+08 | 1.07E+08 | Backfat at last rib QTL (21377) | 6 | 1.03E+08 | 1.28E+08 |
| ENSSSCG00000003700 | 6 | 1.07E+08 | 1.07E+08 | Loin muscle area QTL (21378) | 6 | 1.03E+08 | 1.28E+08 |
| ENSSSCG00000003700 | 6 | 1.07E+08 | 1.07E+08 | muscle protein percentage QTL (21379) | 6 | 1.03E+08 | 1.28E+08 |
| ENSSSCG00000003700 | 6 | 1.07E+08 | 1.07E+08 | Carcass weight (hot) QTL (21380) | 6 | 1.03E+08 | 1.28E+08 |
| ENSSSCG00000003700 | 6 | 1.07E+08 | 1.07E+08 | Carcass temperature (24 hr post-mortem) QTL (21381) | 6 | 1.03E+08 | 1.28E+08 |
| ENSSSCG00000003700 | 6 | 1.07E+08 | 1.07E+08 | Fat area percentage in carcass QTL (18630) | 6 | 1.03E+08 | 1.28E+08 |
| ENSSSCG00000003700 | 6 | 1.07E+08 | 1.07E+08 | Fat area percentage in carcass QTL (18635) | 6 | 1.03E+08 | 1.28E+08 |
| ENSSSCG00000003700 | 6 | 1.07E+08 | 1.07E+08 | Fat area percentage in carcass QTL (18637) | 6 | 1.03E+08 | 1.28E+08 |
| ENSSSCG00000003700 | 6 | 1.07E+08 | 1.07E+08 | Fat area percentage in carcass QTL (18642) | 6 | 1.03E+08 | 1.28E+08 |
| ENSSSCG00000003700 | 6 | 1.07E+08 | 1.07E+08 | Leptin level QTL (18643) | 6 | 1.03E+08 | 1.28E+08 |
| ENSSSCG00000003700 | 6 | 1.07E+08 | 1.07E+08 | Fat area percentage in carcass QTL (66026) | 6 | 1.05E+08 | 1.15E+08 |
| ENSSSCG00000003700 | 6 | 1.07E+08 | 1.07E+08 | Fat area percentage in carcass QTL (66025) | 6 | 1.05E+08 | 1.15E+08 |
| ENSSSCG00000003700 | 6 | 1.07E+08 | 1.07E+08 | Leptin level QTL (66032) | 6 | 1.06E+08 | 1.21E+08 |
| ENSSSCG00000003700 | 6 | 1.07E+08 | 1.07E+08 | Fat area percentage in carcass QTL (66028) | 6 | 1.06E+08 | 1.15E+08 |
| ENSSSCG00000003700 | 6 | 1.07E+08 | 1.07E+08 | Fat area percentage in carcass QTL (66031) | 6 | 1.06E+08 | 1.14E+08 |
| ENSSSCG00000003700 | 6 | 1.07E+08 | 1.07E+08 | Average backfat thickness QTL (66024) | 6 | 1.06E+08 | 1.13E+08 |
| ENSSSCG00000003700 | 6 | 1.07E+08 | 1.07E+08 | Fat area percentage in carcass QTL (66027) | 6 | 1.06E+08 | 1.13E+08 |
| ENSSSCG00000003700 | 6 | 1.07E+08 | 1.07E+08 | Body weight (3 weeks) QTL (181) | 6 | 1.06E+08 | 1.32E+08 |
| ENSSSCG00000003700 | 6 | 1.07E+08 | 1.07E+08 | Fat area percentage in carcass QTL (66030) | 6 | 1.06E+08 | 1.17E+08 |
| ENSSSCG00000003700 | 6 | 1.07E+08 | 1.07E+08 | Fat area percentage in carcass QTL (66029) | 6 | 1.06E+08 | 1.12E+08 |
| ENSSSCG00000003700 | 6 | 1.07E+08 | 1.07E+08 | Marbling QTL (38089) | 6 | 6235784 | 1.28E+08 |
| ENSSSCG00000003700 | 6 | 1.07E+08 | 1.07E+08 | Body weight (end of test) QTL (5201) | 6 | 12044459 | 1.35E+08 |
| ENSSSCG00000003700 | 6 | 1.07E+08 | 1.07E+08 | Alkaline phosphatase activity QTL (15097) | 6 | 35450005 | 1.38E+08 |
| ENSSSCG00000003700 | 6 | 1.07E+08 | 1.07E+08 | Loin fat percentage QTL (38090) | 6 | 35450005 | 1.28E+08 |
| ENSSSCG00000003700 | 6 | 1.07E+08 | 1.07E+08 | Lactate dehydrogenase level QTL (15099) | 6 | 37585548 | 1.2E+08 |
| ENSSSCG00000003700 | 6 | 1.07E+08 | 1.07E+08 | Carcass length QTL (3873) | 6 | 38610608 | 1.2E+08 |
| ENSSSCG00000003700 | 6 | 1.07E+08 | 1.07E+08 | Shear force QTL (38091) | 6 | 50398803 | 1.38E+08 |
| ENSSSCG00000003700 | 6 | 1.07E+08 | 1.07E+08 | Average backfat thickness QTL (148) | 6 | 55524105 | 1.33E+08 |
| ENSSSCG00000003700 | 6 | 1.07E+08 | 1.07E+08 | Average backfat thickness QTL (149) | 6 | 55524105 | 1.33E+08 |
| ENSSSCG00000003700 | 6 | 1.07E+08 | 1.07E+08 | Intramuscular fat content QTL (150) | 6 | 55524105 | 1.33E+08 |
| ENSSSCG00000003700 | 6 | 1.07E+08 | 1.07E+08 | Intramuscular fat content QTL (151) | 6 | 55524105 | 1.33E+08 |
| ENSSSCG00000003700 | 6 | 1.07E+08 | 1.07E+08 | Backfat between 3rd and 4th last ribs QTL (4167) | 6 | 59368081 | 1.24E+08 |
| ENSSSCG00000003700 | 6 | 1.07E+08 | 1.07E+08 | body weight (17 weeks) QTL (184) | 6 | 61759889 | 1.15E+08 |
| ENSSSCG00000003700 | 6 | 1.07E+08 | 1.07E+08 | body weight (13 weeks) QTL (183) | 6 | 61759889 | 1.07E+08 |
| ENSSSCG00000003700 | 6 | 1.07E+08 | 1.07E+08 | Intramuscular fat content QTL (4169) | 6 | 61930732 | 1.26E+08 |
| ENSSSCG00000003700 | 6 | 1.07E+08 | 1.07E+08 | Body weight (20 weeks) QTL (29696) | 6 | 65347600 | 1.28E+08 |
| ENSSSCG00000003700 | 6 | 1.07E+08 | 1.07E+08 | Carcass weight (hot) QTL (29697) | 6 | 65347600 | 1.28E+08 |
| ENSSSCG00000003700 | 6 | 1.07E+08 | 1.07E+08 | subjective boar flavor in lean QTL (4219) | 6 | 65347600 | 1.28E+08 |
| ENSSSCG00000003700 | 6 | 1.07E+08 | 1.07E+08 | Intramuscular fat content QTL (314) | 6 | 87130134 | 1.38E+08 |
| ENSSSCG00000003700 | 6 | 1.07E+08 | 1.07E+08 | body weight (10 weeks) QTL (182) | 6 | 1.02E+08 | 1.35E+08 |
| ENSSSCG00000003700 | 6 | 1.07E+08 | 1.07E+08 | Cholesterol level QTL (15071) | 6 | 1.05E+08 | 1.38E+08 |
| ENSSSCG00000003771 | 6 | 1.35E+08 | 1.35E+08 | Alkaline phosphatase activity QTL (15097) | 6 | 35450005 | 1.38E+08 |
| ENSSSCG00000003771 | 6 | 1.35E+08 | 1.35E+08 | Shear force QTL (38091) | 6 | 50398803 | 1.38E+08 |
| ENSSSCG00000003771 | 6 | 1.35E+08 | 1.35E+08 | Intramuscular fat content QTL (314) | 6 | 87130134 | 1.38E+08 |
| ENSSSCG00000003771 | 6 | 1.35E+08 | 1.35E+08 | Cholesterol level QTL (15071) | 6 | 1.05E+08 | 1.38E+08 |
| ENSSSCG00000003771 | 6 | 1.35E+08 | 1.35E+08 | Intramuscular fat content QTL (4165) | 6 | 1.11E+08 | 1.53E+08 |
| ENSSSCG00000003771 | 6 | 1.35E+08 | 1.35E+08 | Backfat at tenth rib QTL (3875) | 6 | 1.27E+08 | 1.38E+08 |
| ENSSSCG00000003771 | 6 | 1.35E+08 | 1.35E+08 | muscle protein percentage QTL (3840) | 6 | 1.28E+08 | 1.42E+08 |
| ENSSSCG00000003771 | 6 | 1.35E+08 | 1.35E+08 | Muscle moisture percentage QTL (3852) | 6 | 1.28E+08 | 1.42E+08 |
| ENSSSCG00000003771 | 6 | 1.35E+08 | 1.35E+08 | Muscle fat content QTL (3816) | 6 | 1.28E+08 | 1.41E+08 |
| ENSSSCG00000003771 | 6 | 1.35E+08 | 1.35E+08 | Average backfat thickness QTL (2850) | 6 | 1.28E+08 | 1.41E+08 |
| ENSSSCG00000003771 | 6 | 1.35E+08 | 1.35E+08 | Subcutanous fat area QTL (2851) | 6 | 1.28E+08 | 1.41E+08 |
| ENSSSCG00000003771 | 6 | 1.35E+08 | 1.35E+08 | Backfat at last rib QTL (2853) | 6 | 1.28E+08 | 1.41E+08 |
| ENSSSCG00000003771 | 6 | 1.35E+08 | 1.35E+08 | Backfat at tenth rib QTL (2943) | 6 | 1.28E+08 | 1.41E+08 |
| ENSSSCG00000003771 | 6 | 1.35E+08 | 1.35E+08 | Carcass temperature (24 hr post-mortem) QTL (3308) | 6 | 1.28E+08 | 1.38E+08 |
| ENSSSCG00000003771 | 6 | 1.35E+08 | 1.35E+08 | Muscle moisture percentage QTL (3250) | 6 | 1.28E+08 | 1.38E+08 |
| ENSSSCG00000003828 | 6 | 1.53E+08 | 1.53E+08 | Carcass weight (hot) QTL (147184) | 6 | 1.53E+08 | 1.53E+08 |
| ENSSSCG00000003828 | 6 | 1.53E+08 | 1.53E+08 | Backfat at rump QTL (23288) | 6 | 1.53E+08 | 1.53E+08 |
| ENSSSCG00000020858 | 7 | 13498222 | 13716757 | Adipocyte diameter QTL (12809) | 7 | 13542543 | 13612390 |
| ENSSSCG00000020858 | 7 | 13498222 | 13716757 | Adipocyte diameter QTL (12831) | 7 | 13542543 | 13612390 |
| ENSSSCG00000020858 | 7 | 13498222 | 13716757 | Backfat at last rib QTL (16881) | 7 | 2871484 | 43537919 |
| ENSSSCG00000020858 | 7 | 13498222 | 13716757 | Carcass weight (hot) QTL (16867) | 7 | 2871484 | 25532933 |
| ENSSSCG00000020858 | 7 | 13498222 | 13716757 | CO2 partial pressure QTL (6325) | 7 | 2871484 | 23437525 |
| ENSSSCG00000020858 | 7 | 13498222 | 13716757 | Bilirubin level QTL (6375) | 7 | 2871484 | 23437525 |
| ENSSSCG00000020858 | 7 | 13498222 | 13716757 | Meat color score QTL (3252) | 7 | 2871484 | 23437525 |
| ENSSSCG00000020858 | 7 | 13498222 | 13716757 | muscle protein percentage QTL (3253) | 7 | 2871484 | 23437525 |
| ENSSSCG00000020858 | 7 | 13498222 | 13716757 | Backfat at tenth rib QTL (3860) | 7 | 2871484 | 23437525 |
| ENSSSCG00000020858 | 7 | 13498222 | 13716757 | Carcass length QTL (1064) | 7 | 2871484 | 23437525 |
| ENSSSCG00000020858 | 7 | 13498222 | 13716757 | Backfat at last rib QTL (1081) | 7 | 2871484 | 23437525 |
| ENSSSCG00000020858 | 7 | 13498222 | 13716757 | Meat color-L QTL (3251) | 7 | 2871484 | 23437525 |
| ENSSSCG00000020858 | 7 | 13498222 | 13716757 | muscle protein percentage QTL (21382) | 7 | 2871484 | 23437525 |
| ENSSSCG00000020858 | 7 | 13498222 | 13716757 | Subcutanous fat area QTL (2788) | 7 | 3104308 | 15521540 |
| ENSSSCG00000020858 | 7 | 13498222 | 13716757 | Carcass width (minimum) QTL (3954) | 7 | 8459239 | 37406911 |
| ENSSSCG00000020858 | 7 | 13498222 | 13716757 | Backfat at first rib QTL (3956) | 7 | 8459239 | 37406911 |
| ENSSSCG00000020858 | 7 | 13498222 | 13716757 | Backfat between 6th and 7th ribs QTL (3957) | 7 | 8459239 | 37406911 |
| ENSSSCG00000020858 | 7 | 13498222 | 13716757 | Linoleic acid content QTL (4204) | 7 | 12029193 | 24834464 |
| ENSSSCG00000020858 | 7 | 13498222 | 13716757 | Shoulder subcutaneous fat thickness QTL (5221) | 7 | 2871484 | 1.2E+08 |
| ENSSSCG00000020858 | 7 | 13498222 | 13716757 | Loin muscle area QTL (38092) | 7 | 8459239 | 1.1E+08 |
| ENSSSCG00000020858 | 7 | 13498222 | 13716757 | Meat color a* QTL (38094) | 7 | 8459239 | 69924537 |
| ENSSSCG00000039952 | 7 | 33378214 | 33718030 | Backfat at last rib QTL (16881) | 7 | 2871484 | 43537919 |
| ENSSSCG00000039952 | 7 | 33378214 | 33718030 | Carcass width (minimum) QTL (3954) | 7 | 8459239 | 37406911 |
| ENSSSCG00000039952 | 7 | 33378214 | 33718030 | Backfat at first rib QTL (3956) | 7 | 8459239 | 37406911 |
| ENSSSCG00000039952 | 7 | 33378214 | 33718030 | Backfat between 6th and 7th ribs QTL (3957) | 7 | 8459239 | 37406911 |
| ENSSSCG00000039952 | 7 | 33378214 | 33718030 | Average daily gain QTL (16852) | 7 | 17228909 | 57507305 |
| ENSSSCG00000039952 | 7 | 33378214 | 33718030 | Average backfat thickness QTL (308) | 7 | 17306517 | 64259175 |
| ENSSSCG00000039952 | 7 | 33378214 | 33718030 | Average backfat thickness QTL (3768) | 7 | 17306517 | 54402997 |
| ENSSSCG00000039952 | 7 | 33378214 | 33718030 | Backfat at last rib QTL (572) | 7 | 23398721 | 34504383 |
| ENSSSCG00000039952 | 7 | 33378214 | 33718030 | Backfat at last lumbar QTL (575) | 7 | 23398721 | 34504383 |
| ENSSSCG00000039952 | 7 | 33378214 | 33718030 | Shoulder subcutaneous fat thickness QTL (702) | 7 | 23398721 | 34504383 |
| ENSSSCG00000039952 | 7 | 33378214 | 33718030 | Loin muscle area QTL (705) | 7 | 23398721 | 34504383 |
| ENSSSCG00000039952 | 7 | 33378214 | 33718030 | Backfat at rump QTL (7315) | 7 | 23398721 | 34504383 |
| ENSSSCG00000039952 | 7 | 33378214 | 33718030 | Adipocyte diameter QTL (12810) | 7 | 23398721 | 34504383 |
| ENSSSCG00000039952 | 7 | 33378214 | 33718030 | Adipocyte diameter QTL (12835) | 7 | 23398721 | 34504383 |
| ENSSSCG00000039952 | 7 | 33378214 | 33718030 | Adipocyte diameter QTL (12840) | 7 | 23398721 | 34504383 |
| ENSSSCG00000039952 | 7 | 33378214 | 33718030 | Adipocyte diameter QTL (12847) | 7 | 23398721 | 34504383 |
| ENSSSCG00000039952 | 7 | 33378214 | 33718030 | Adipocyte diameter QTL (12848) | 7 | 23398721 | 34504383 |
| ENSSSCG00000039952 | 7 | 33378214 | 33718030 | Backfat at last rib QTL (28) | 7 | 23437525 | 64026352 |
| ENSSSCG00000039952 | 7 | 33378214 | 33718030 | Loin muscle depth QTL (366) | 7 | 23437525 | 63250275 |
| ENSSSCG00000039952 | 7 | 33378214 | 33718030 | Average backfat thickness QTL (365) | 7 | 23437525 | 61542906 |
| ENSSSCG00000039952 | 7 | 33378214 | 33718030 | Backfat at last lumbar QTL (2945) | 7 | 23437525 | 61542906 |
| ENSSSCG00000039952 | 7 | 33378214 | 33718030 | Carcass length QTL (11) | 7 | 23437525 | 58438598 |
| ENSSSCG00000039952 | 7 | 33378214 | 33718030 | Average backfat thickness QTL (36) | 7 | 23437525 | 56964051 |
| ENSSSCG00000039952 | 7 | 33378214 | 33718030 | NADP-malate dehydrogenase activity QTL (12775) | 7 | 23437525 | 54402997 |
| ENSSSCG00000039952 | 7 | 33378214 | 33718030 | NADP-malate dehydrogenase activity QTL (12781) | 7 | 23437525 | 54402997 |
| ENSSSCG00000039952 | 7 | 33378214 | 33718030 | Adipocyte diameter QTL (12807) | 7 | 23437525 | 54402997 |
| ENSSSCG00000039952 | 7 | 33378214 | 33718030 | Average daily gain QTL (4142) | 7 | 23437525 | 54402997 |
| ENSSSCG00000039952 | 7 | 33378214 | 33718030 | Average daily gain QTL (4143) | 7 | 23437525 | 54402997 |
| ENSSSCG00000039952 | 7 | 33378214 | 33718030 | Average daily gain QTL (4144) | 7 | 23437525 | 54402997 |
| ENSSSCG00000039952 | 7 | 33378214 | 33718030 | PH for Longissmus dorsi QTL (4145) | 7 | 23437525 | 54402997 |
| ENSSSCG00000039952 | 7 | 33378214 | 33718030 | Average daily gain QTL (4171) | 7 | 23437525 | 54402997 |
| ENSSSCG00000039952 | 7 | 33378214 | 33718030 | Average daily gain QTL (4172) | 7 | 23437525 | 54402997 |
| ENSSSCG00000039952 | 7 | 33378214 | 33718030 | PH for Longissmus dorsi QTL (4180) | 7 | 23437525 | 54402997 |
| ENSSSCG00000039952 | 7 | 33378214 | 33718030 | PH for Longissmus dorsi QTL (4181) | 7 | 23437525 | 54402997 |
| ENSSSCG00000039952 | 7 | 33378214 | 33718030 | Carcass weight (hot) QTL (4185) | 7 | 23437525 | 54402997 |
| ENSSSCG00000039952 | 7 | 33378214 | 33718030 | Average daily gain QTL (5943) | 7 | 23437525 | 54402997 |
| ENSSSCG00000039952 | 7 | 33378214 | 33718030 | Average daily gain QTL (5945) | 7 | 23437525 | 54402997 |
| ENSSSCG00000039952 | 7 | 33378214 | 33718030 | Body weight (slaughter) QTL (5946) | 7 | 23437525 | 54402997 |
| ENSSSCG00000039952 | 7 | 33378214 | 33718030 | Meat color-a QTL (295) | 7 | 23437525 | 54402997 |
| ENSSSCG00000039952 | 7 | 33378214 | 33718030 | Average backfat thickness QTL (312) | 7 | 23437525 | 54402997 |
| ENSSSCG00000039952 | 7 | 33378214 | 33718030 | Smell intensity - fat QTL (166) | 7 | 23437525 | 54402997 |
| ENSSSCG00000039952 | 7 | 33378214 | 33718030 | Fat area percentage in carcass QTL (18640) | 7 | 23437525 | 45012466 |
| ENSSSCG00000039952 | 7 | 33378214 | 33718030 | Alkaline phosphatase activity QTL (6349) | 7 | 23437525 | 45012466 |
| ENSSSCG00000039952 | 7 | 33378214 | 33718030 | Time spent drinking QTL (5916) | 7 | 23437525 | 45012466 |
| ENSSSCG00000039952 | 7 | 33378214 | 33718030 | Time spent rooting QTL (5920) | 7 | 23437525 | 45012466 |
| ENSSSCG00000039952 | 7 | 33378214 | 33718030 | Fat protein content QTL (12777) | 7 | 23437525 | 44779642 |
| ENSSSCG00000039952 | 7 | 33378214 | 33718030 | NADPH-generating enzyme activity QTL (12778) | 7 | 23437525 | 44779642 |
| ENSSSCG00000039952 | 7 | 33378214 | 33718030 | Average daily gain QTL (16891) | 7 | 23437525 | 43615527 |
| ENSSSCG00000039952 | 7 | 33378214 | 33718030 | Carcass length QTL (1048) | 7 | 23437525 | 41985765 |
| ENSSSCG00000039952 | 7 | 33378214 | 33718030 | Abdominal fat weight QTL (1062) | 7 | 23437525 | 41985765 |
| ENSSSCG00000039952 | 7 | 33378214 | 33718030 | Loin muscle area QTL (16925) | 7 | 23437525 | 37406911 |
| ENSSSCG00000039952 | 7 | 33378214 | 33718030 | Loin muscle area QTL (16926) | 7 | 23437525 | 37406911 |
| ENSSSCG00000039952 | 7 | 33378214 | 33718030 | Backfat at first rib QTL (20) | 7 | 25532933 | 58438598 |
| ENSSSCG00000039952 | 7 | 33378214 | 33718030 | Leaf fat weight QTL (39) | 7 | 25532933 | 56964051 |
| ENSSSCG00000039952 | 7 | 33378214 | 33718030 | Backfat at tenth rib QTL (24) | 7 | 25532933 | 54402997 |
| ENSSSCG00000039952 | 7 | 33378214 | 33718030 | Skin percentage QTL (5506) | 7 | 25532933 | 47728735 |
| ENSSSCG00000039952 | 7 | 33378214 | 33718030 | Muscle moisture percentage QTL (3853) | 7 | 25532933 | 47728735 |
| ENSSSCG00000039952 | 7 | 33378214 | 33718030 | Backfat at last lumbar QTL (32) | 7 | 25532933 | 45710935 |
| ENSSSCG00000039952 | 7 | 33378214 | 33718030 | Backfat at tenth rib QTL (1069) | 7 | 25532933 | 45012466 |
| ENSSSCG00000039952 | 7 | 33378214 | 33718030 | Backfat at last rib QTL (1070) | 7 | 25532933 | 45012466 |
| ENSSSCG00000039952 | 7 | 33378214 | 33718030 | Average backfat thickness QTL (3836) | 7 | 25532933 | 45012466 |
| ENSSSCG00000039952 | 7 | 33378214 | 33718030 | NADP-malate dehydrogenase activity QTL (12776) | 7 | 25532933 | 44779642 |
| ENSSSCG00000039952 | 7 | 33378214 | 33718030 | NADPH-generating enzyme activity QTL (12779) | 7 | 25532933 | 44779642 |
| ENSSSCG00000039952 | 7 | 33378214 | 33718030 | NADP-malate dehydrogenase activity QTL (12783) | 7 | 25532933 | 44779642 |
| ENSSSCG00000039952 | 7 | 33378214 | 33718030 | NADPH-generating enzyme activity QTL (12785) | 7 | 25532933 | 44779642 |
| ENSSSCG00000039952 | 7 | 33378214 | 33718030 | Carcass weight (hot) QTL (16903) | 7 | 25532933 | 44624427 |
| ENSSSCG00000039952 | 7 | 33378214 | 33718030 | Average backfat thickness QTL (1072) | 7 | 25532933 | 41985765 |
| ENSSSCG00000039952 | 7 | 33378214 | 33718030 | Backfat at mid-back QTL (281) | 7 | 25532933 | 37406911 |
| ENSSSCG00000039952 | 7 | 33378214 | 33718030 | backfat between the last 3rd and 4th lumbar QTL (6036) | 7 | 30437739 | 39510080 |
| ENSSSCG00000039952 | 7 | 33378214 | 33718030 | Backfat at last rib QTL (6037) | 7 | 30437739 | 39510080 |
| ENSSSCG00000039952 | 7 | 33378214 | 33718030 | Leaf fat weight QTL (18055) | 7 | 32595234 | 35777149 |
| ENSSSCG00000039952 | 7 | 33378214 | 33718030 | Backfat at rump QTL (18056) | 7 | 32595234 | 35777149 |
| ENSSSCG00000039952 | 7 | 33378214 | 33718030 | Average backfat thickness QTL (18058) | 7 | 32595234 | 35777149 |
| ENSSSCG00000039952 | 7 | 33378214 | 33718030 | Loin muscle area QTL (13358) | 7 | 33348028 | 34100823 |
| ENSSSCG00000039952 | 7 | 33378214 | 33718030 | Shoulder subcutaneous fat thickness QTL (5221) | 7 | 2871484 | 1.2E+08 |
| ENSSSCG00000039952 | 7 | 33378214 | 33718030 | Loin muscle area QTL (38092) | 7 | 8459239 | 1.1E+08 |
| ENSSSCG00000039952 | 7 | 33378214 | 33718030 | Meat color a* QTL (38094) | 7 | 8459239 | 69924537 |
| ENSSSCG00000039952 | 7 | 33378214 | 33718030 | Loin fat percentage QTL (38055) | 7 | 17306517 | 1.1E+08 |
| ENSSSCG00000039952 | 7 | 33378214 | 33718030 | Carcass weight (hot) QTL (3) | 7 | 17306517 | 69924537 |
| ENSSSCG00000039952 | 7 | 33378214 | 33718030 | Body weight (end of test) QTL (5236) | 7 | 22506233 | 1.09E+08 |
| ENSSSCG00000039952 | 7 | 33378214 | 33718030 | Carcass length QTL (5700) | 7 | 23437525 | 79315069 |
| ENSSSCG00000039952 | 7 | 33378214 | 33718030 | Backfat at last lumbar QTL (60) | 7 | 25532933 | 1.05E+08 |
| ENSSSCG00000001646 | 7 | 37824186 | 37933602 | Backfat at last rib QTL (16881) | 7 | 2871484 | 43537919 |
| ENSSSCG00000001646 | 7 | 37824186 | 37933602 | Average daily gain QTL (16852) | 7 | 17228909 | 57507305 |
| ENSSSCG00000001646 | 7 | 37824186 | 37933602 | Average backfat thickness QTL (308) | 7 | 17306517 | 64259175 |
| ENSSSCG00000001646 | 7 | 37824186 | 37933602 | Average backfat thickness QTL (3768) | 7 | 17306517 | 54402997 |
| ENSSSCG00000001646 | 7 | 37824186 | 37933602 | Backfat at last rib QTL (28) | 7 | 23437525 | 64026352 |
| ENSSSCG00000001646 | 7 | 37824186 | 37933602 | Loin muscle depth QTL (366) | 7 | 23437525 | 63250275 |
| ENSSSCG00000001646 | 7 | 37824186 | 37933602 | Average backfat thickness QTL (365) | 7 | 23437525 | 61542906 |
| ENSSSCG00000001646 | 7 | 37824186 | 37933602 | Backfat at last lumbar QTL (2945) | 7 | 23437525 | 61542906 |
| ENSSSCG00000001646 | 7 | 37824186 | 37933602 | Carcass length QTL (11) | 7 | 23437525 | 58438598 |
| ENSSSCG00000001646 | 7 | 37824186 | 37933602 | Average backfat thickness QTL (36) | 7 | 23437525 | 56964051 |
| ENSSSCG00000001646 | 7 | 37824186 | 37933602 | NADP-malate dehydrogenase activity QTL (12775) | 7 | 23437525 | 54402997 |
| ENSSSCG00000001646 | 7 | 37824186 | 37933602 | NADP-malate dehydrogenase activity QTL (12781) | 7 | 23437525 | 54402997 |
| ENSSSCG00000001646 | 7 | 37824186 | 37933602 | Adipocyte diameter QTL (12807) | 7 | 23437525 | 54402997 |
| ENSSSCG00000001646 | 7 | 37824186 | 37933602 | Average daily gain QTL (4142) | 7 | 23437525 | 54402997 |
| ENSSSCG00000001646 | 7 | 37824186 | 37933602 | Average daily gain QTL (4143) | 7 | 23437525 | 54402997 |
| ENSSSCG00000001646 | 7 | 37824186 | 37933602 | Average daily gain QTL (4144) | 7 | 23437525 | 54402997 |
| ENSSSCG00000001646 | 7 | 37824186 | 37933602 | PH for Longissmus dorsi QTL (4145) | 7 | 23437525 | 54402997 |
| ENSSSCG00000001646 | 7 | 37824186 | 37933602 | Average daily gain QTL (4171) | 7 | 23437525 | 54402997 |
| ENSSSCG00000001646 | 7 | 37824186 | 37933602 | Average daily gain QTL (4172) | 7 | 23437525 | 54402997 |
| ENSSSCG00000001646 | 7 | 37824186 | 37933602 | PH for Longissmus dorsi QTL (4180) | 7 | 23437525 | 54402997 |
| ENSSSCG00000001646 | 7 | 37824186 | 37933602 | PH for Longissmus dorsi QTL (4181) | 7 | 23437525 | 54402997 |
| ENSSSCG00000001646 | 7 | 37824186 | 37933602 | Carcass weight (hot) QTL (4185) | 7 | 23437525 | 54402997 |
| ENSSSCG00000001646 | 7 | 37824186 | 37933602 | Average daily gain QTL (5943) | 7 | 23437525 | 54402997 |
| ENSSSCG00000001646 | 7 | 37824186 | 37933602 | Average daily gain QTL (5945) | 7 | 23437525 | 54402997 |
| ENSSSCG00000001646 | 7 | 37824186 | 37933602 | Body weight (slaughter) QTL (5946) | 7 | 23437525 | 54402997 |
| ENSSSCG00000001646 | 7 | 37824186 | 37933602 | Meat color-a QTL (295) | 7 | 23437525 | 54402997 |
| ENSSSCG00000001646 | 7 | 37824186 | 37933602 | Average backfat thickness QTL (312) | 7 | 23437525 | 54402997 |
| ENSSSCG00000001646 | 7 | 37824186 | 37933602 | Smell intensity - fat QTL (166) | 7 | 23437525 | 54402997 |
| ENSSSCG00000001646 | 7 | 37824186 | 37933602 | Fat area percentage in carcass QTL (18640) | 7 | 23437525 | 45012466 |
| ENSSSCG00000001646 | 7 | 37824186 | 37933602 | Alkaline phosphatase activity QTL (6349) | 7 | 23437525 | 45012466 |
| ENSSSCG00000001646 | 7 | 37824186 | 37933602 | Time spent drinking QTL (5916) | 7 | 23437525 | 45012466 |
| ENSSSCG00000001646 | 7 | 37824186 | 37933602 | Time spent rooting QTL (5920) | 7 | 23437525 | 45012466 |
| ENSSSCG00000001646 | 7 | 37824186 | 37933602 | Fat protein content QTL (12777) | 7 | 23437525 | 44779642 |
| ENSSSCG00000001646 | 7 | 37824186 | 37933602 | NADPH-generating enzyme activity QTL (12778) | 7 | 23437525 | 44779642 |
| ENSSSCG00000001646 | 7 | 37824186 | 37933602 | Average daily gain QTL (16891) | 7 | 23437525 | 43615527 |
| ENSSSCG00000001646 | 7 | 37824186 | 37933602 | Carcass length QTL (1048) | 7 | 23437525 | 41985765 |
| ENSSSCG00000001646 | 7 | 37824186 | 37933602 | Abdominal fat weight QTL (1062) | 7 | 23437525 | 41985765 |
| ENSSSCG00000001646 | 7 | 37824186 | 37933602 | Backfat at first rib QTL (20) | 7 | 25532933 | 58438598 |
| ENSSSCG00000001646 | 7 | 37824186 | 37933602 | Leaf fat weight QTL (39) | 7 | 25532933 | 56964051 |
| ENSSSCG00000001646 | 7 | 37824186 | 37933602 | Backfat at tenth rib QTL (24) | 7 | 25532933 | 54402997 |
| ENSSSCG00000001646 | 7 | 37824186 | 37933602 | Skin percentage QTL (5506) | 7 | 25532933 | 47728735 |
| ENSSSCG00000001646 | 7 | 37824186 | 37933602 | Muscle moisture percentage QTL (3853) | 7 | 25532933 | 47728735 |
| ENSSSCG00000001646 | 7 | 37824186 | 37933602 | Backfat at last lumbar QTL (32) | 7 | 25532933 | 45710935 |
| ENSSSCG00000001646 | 7 | 37824186 | 37933602 | Backfat at tenth rib QTL (1069) | 7 | 25532933 | 45012466 |
| ENSSSCG00000001646 | 7 | 37824186 | 37933602 | Backfat at last rib QTL (1070) | 7 | 25532933 | 45012466 |
| ENSSSCG00000001646 | 7 | 37824186 | 37933602 | Average backfat thickness QTL (3836) | 7 | 25532933 | 45012466 |
| ENSSSCG00000001646 | 7 | 37824186 | 37933602 | NADP-malate dehydrogenase activity QTL (12776) | 7 | 25532933 | 44779642 |
| ENSSSCG00000001646 | 7 | 37824186 | 37933602 | NADPH-generating enzyme activity QTL (12779) | 7 | 25532933 | 44779642 |
| ENSSSCG00000001646 | 7 | 37824186 | 37933602 | NADP-malate dehydrogenase activity QTL (12783) | 7 | 25532933 | 44779642 |
| ENSSSCG00000001646 | 7 | 37824186 | 37933602 | NADPH-generating enzyme activity QTL (12785) | 7 | 25532933 | 44779642 |
| ENSSSCG00000001646 | 7 | 37824186 | 37933602 | Carcass weight (hot) QTL (16903) | 7 | 25532933 | 44624427 |
| ENSSSCG00000001646 | 7 | 37824186 | 37933602 | Average backfat thickness QTL (1072) | 7 | 25532933 | 41985765 |
| ENSSSCG00000001646 | 7 | 37824186 | 37933602 | backfat between the last 3rd and 4th lumbar QTL (6036) | 7 | 30437739 | 39510080 |
| ENSSSCG00000001646 | 7 | 37824186 | 37933602 | Backfat at last rib QTL (6037) | 7 | 30437739 | 39510080 |
| ENSSSCG00000001646 | 7 | 37824186 | 37933602 | Average backfat thickness QTL (18000) | 7 | 33914564 | 45012466 |
| ENSSSCG00000001646 | 7 | 37824186 | 37933602 | Leaf fat weight QTL (3880) | 7 | 34380211 | 58438598 |
| ENSSSCG00000001646 | 7 | 37824186 | 37933602 | Fat protein content QTL (18012) | 7 | 34457818 | 54402997 |
| ENSSSCG00000001646 | 7 | 37824186 | 37933602 | NADP-malate dehydrogenase activity QTL (18017) | 7 | 34457818 | 54402997 |
| ENSSSCG00000001646 | 7 | 37824186 | 37933602 | Adipocyte diameter QTL (18023) | 7 | 34457818 | 54402997 |
| ENSSSCG00000001646 | 7 | 37824186 | 37933602 | Adipocyte diameter QTL (18031) | 7 | 34457818 | 54402997 |
| ENSSSCG00000001646 | 7 | 37824186 | 37933602 | Backfat at mid-back QTL (5211) | 7 | 36630834 | 57740128 |
| ENSSSCG00000001646 | 7 | 37824186 | 37933602 | Carcass length QTL (3254) | 7 | 37406911 | 58438598 |
| ENSSSCG00000001646 | 7 | 37824186 | 37933602 | Fat androstenone level QTL (601) | 7 | 37406911 | 54402997 |
| ENSSSCG00000001646 | 7 | 37824186 | 37933602 | Fat androstenone level QTL (602) | 7 | 37406911 | 54402997 |
| ENSSSCG00000001646 | 7 | 37824186 | 37933602 | Fat androstenone level QTL (603) | 7 | 37406911 | 54402997 |
| ENSSSCG00000001646 | 7 | 37824186 | 37933602 | Fat androstenone level QTL (604) | 7 | 37406911 | 54402997 |
| ENSSSCG00000001646 | 7 | 37824186 | 37933602 | Fat androstenone level QTL (605) | 7 | 37406911 | 54402997 |
| ENSSSCG00000001646 | 7 | 37824186 | 37933602 | Average backfat thickness QTL (207) | 7 | 37406911 | 54402997 |
| ENSSSCG00000001646 | 7 | 37824186 | 37933602 | Average backfat thickness QTL (208) | 7 | 37406911 | 54402997 |
| ENSSSCG00000001646 | 7 | 37824186 | 37933602 | Average backfat thickness QTL (209) | 7 | 37406911 | 54402997 |
| ENSSSCG00000001646 | 7 | 37824186 | 37933602 | Average backfat thickness QTL (210) | 7 | 37406911 | 54402997 |
| ENSSSCG00000001646 | 7 | 37824186 | 37933602 | Average backfat thickness QTL (211) | 7 | 37406911 | 54402997 |
| ENSSSCG00000001646 | 7 | 37824186 | 37933602 | Average daily gain QTL (3143) | 7 | 37406911 | 54402997 |
| ENSSSCG00000001646 | 7 | 37824186 | 37933602 | Average daily gain QTL (3144) | 7 | 37406911 | 54402997 |
| ENSSSCG00000001646 | 7 | 37824186 | 37933602 | Average backfat thickness QTL (3146) | 7 | 37406911 | 54402997 |
| ENSSSCG00000001646 | 7 | 37824186 | 37933602 | Backfat at last lumbar QTL (3165) | 7 | 37406911 | 54402997 |
| ENSSSCG00000001646 | 7 | 37824186 | 37933602 | Backfat thickness between 3rd and 4th rib QTL (3166) | 7 | 37406911 | 54402997 |
| ENSSSCG00000001646 | 7 | 37824186 | 37933602 | Japanese color scale QTL (3993) | 7 | 37406911 | 47728735 |
| ENSSSCG00000001646 | 7 | 37824186 | 37933602 | Meat color L* QTL (3995) | 7 | 37406911 | 47728735 |
| ENSSSCG00000001646 | 7 | 37824186 | 37933602 | Shear force at first peak QTL (3997) | 7 | 37406911 | 47728735 |
| ENSSSCG00000001646 | 7 | 37824186 | 37933602 | Backfat at last rib QTL (5436) | 7 | 37406911 | 45012466 |
| ENSSSCG00000001646 | 7 | 37824186 | 37933602 | Shoulder subcutaneous fat thickness QTL (5221) | 7 | 2871484 | 1.2E+08 |
| ENSSSCG00000001646 | 7 | 37824186 | 37933602 | Loin muscle area QTL (38092) | 7 | 8459239 | 1.1E+08 |
| ENSSSCG00000001646 | 7 | 37824186 | 37933602 | Meat color a* QTL (38094) | 7 | 8459239 | 69924537 |
| ENSSSCG00000001646 | 7 | 37824186 | 37933602 | Loin fat percentage QTL (38055) | 7 | 17306517 | 1.1E+08 |
| ENSSSCG00000001646 | 7 | 37824186 | 37933602 | Carcass weight (hot) QTL (3) | 7 | 17306517 | 69924537 |
| ENSSSCG00000001646 | 7 | 37824186 | 37933602 | Body weight (end of test) QTL (5236) | 7 | 22506233 | 1.09E+08 |
| ENSSSCG00000001646 | 7 | 37824186 | 37933602 | Carcass length QTL (5700) | 7 | 23437525 | 79315069 |
| ENSSSCG00000001646 | 7 | 37824186 | 37933602 | Backfat at last lumbar QTL (60) | 7 | 25532933 | 1.05E+08 |
| ENSSSCG00000001646 | 7 | 37824186 | 37933602 | backfat above muscle dorsi QTL (5204) | 7 | 34768249 | 73339276 |
| ENSSSCG00000001646 | 7 | 37824186 | 37933602 | Shoulder subcutaneous fat thickness QTL (5220) | 7 | 36553226 | 1.2E+08 |
| ENSSSCG00000001646 | 7 | 37824186 | 37933602 | Backfat at mid-back QTL (5212) | 7 | 36863657 | 68449991 |
| ENSSSCG00000001646 | 7 | 37824186 | 37933602 | Linolenic acid content QTL (156940) | 7 | 37406911 | 1.21E+08 |
| ENSSSCG00000001646 | 7 | 37824186 | 37933602 | Polyunsaturated fatty acid content QTL (156941) | 7 | 37406911 | 1.1E+08 |
| ENSSSCG00000001646 | 7 | 37824186 | 37933602 | Linoleic acid content QTL (156874) | 7 | 37406911 | 1.1E+08 |
| ENSSSCG00000001646 | 7 | 37824186 | 37933602 | Margaric acid content QTL (156872) | 7 | 37406911 | 69924537 |
| ENSSSCG00000001852 | 7 | 55651606 | 55681426 | Average daily gain QTL (16852) | 7 | 17228909 | 57507305 |
| ENSSSCG00000001852 | 7 | 55651606 | 55681426 | Average backfat thickness QTL (308) | 7 | 17306517 | 64259175 |
| ENSSSCG00000001852 | 7 | 55651606 | 55681426 | Backfat at last rib QTL (28) | 7 | 23437525 | 64026352 |
| ENSSSCG00000001852 | 7 | 55651606 | 55681426 | Loin muscle depth QTL (366) | 7 | 23437525 | 63250275 |
| ENSSSCG00000001852 | 7 | 55651606 | 55681426 | Average backfat thickness QTL (365) | 7 | 23437525 | 61542906 |
| ENSSSCG00000001852 | 7 | 55651606 | 55681426 | Backfat at last lumbar QTL (2945) | 7 | 23437525 | 61542906 |
| ENSSSCG00000001852 | 7 | 55651606 | 55681426 | Carcass length QTL (11) | 7 | 23437525 | 58438598 |
| ENSSSCG00000001852 | 7 | 55651606 | 55681426 | Average backfat thickness QTL (36) | 7 | 23437525 | 56964051 |
| ENSSSCG00000001852 | 7 | 55651606 | 55681426 | Backfat at first rib QTL (20) | 7 | 25532933 | 58438598 |
| ENSSSCG00000001852 | 7 | 55651606 | 55681426 | Leaf fat weight QTL (39) | 7 | 25532933 | 56964051 |
| ENSSSCG00000001852 | 7 | 55651606 | 55681426 | Leaf fat weight QTL (3880) | 7 | 34380211 | 58438598 |
| ENSSSCG00000001852 | 7 | 55651606 | 55681426 | Backfat at mid-back QTL (5211) | 7 | 36630834 | 57740128 |
| ENSSSCG00000001852 | 7 | 55651606 | 55681426 | Carcass length QTL (3254) | 7 | 37406911 | 58438598 |
| ENSSSCG00000001852 | 7 | 55651606 | 55681426 | Subcutanous fat area QTL (18006) | 7 | 39890357 | 60456398 |
| ENSSSCG00000001852 | 7 | 55651606 | 55681426 | Backfat at mid-back QTL (282) | 7 | 44236389 | 58438598 |
| ENSSSCG00000001852 | 7 | 55651606 | 55681426 | Backfat thickness between 3rd and 4th rib QTL (419) | 7 | 44236389 | 56653621 |
| ENSSSCG00000001852 | 7 | 55651606 | 55681426 | Shoulder subcutaneous fat thickness QTL (5252) | 7 | 44702035 | 56187974 |
| ENSSSCG00000001852 | 7 | 55651606 | 55681426 | Backfat at last rib QTL (16837) | 7 | 44779642 | 61310083 |
| ENSSSCG00000001852 | 7 | 55651606 | 55681426 | Backfat at first rib QTL (512) | 7 | 44779642 | 56964051 |
| ENSSSCG00000001852 | 7 | 55651606 | 55681426 | Backfat at last rib QTL (513) | 7 | 44779642 | 56964051 |
| ENSSSCG00000001852 | 7 | 55651606 | 55681426 | Backfat at last lumbar QTL (514) | 7 | 44779642 | 56964051 |
| ENSSSCG00000001852 | 7 | 55651606 | 55681426 | androstenone, laboratory QTL (4209) | 7 | 45012466 | 64259175 |
| ENSSSCG00000001852 | 7 | 55651606 | 55681426 | Palmitoleic acid content QTL (9572) | 7 | 45012466 | 56964051 |
| ENSSSCG00000001852 | 7 | 55651606 | 55681426 | body weight (10 weeks) QTL (188) | 7 | 45710935 | 56964051 |
| ENSSSCG00000001852 | 7 | 55651606 | 55681426 | body weight (13 weeks) QTL (189) | 7 | 45710935 | 56964051 |
| ENSSSCG00000001852 | 7 | 55651606 | 55681426 | body weight (17 weeks) QTL (190) | 7 | 45710935 | 56964051 |
| ENSSSCG00000001852 | 7 | 55651606 | 55681426 | Average daily gain QTL (191) | 7 | 45710935 | 56964051 |
| ENSSSCG00000001852 | 7 | 55651606 | 55681426 | Loin muscle depth QTL (390) | 7 | 46564620 | 66742622 |
| ENSSSCG00000001852 | 7 | 55651606 | 55681426 | Backfat at last rib QTL (420) | 7 | 46564620 | 57429698 |
| ENSSSCG00000001852 | 7 | 55651606 | 55681426 | Carcass length QTL (21385) | 7 | 46952658 | 58438598 |
| ENSSSCG00000001852 | 7 | 55651606 | 55681426 | Loin muscle area QTL (21386) | 7 | 46952658 | 58438598 |
| ENSSSCG00000001852 | 7 | 55651606 | 55681426 | Backfat at last rib QTL (17802) | 7 | 47728735 | 66121760 |
| ENSSSCG00000001852 | 7 | 55651606 | 55681426 | Average daily gain QTL (5944) | 7 | 54402997 | 63250275 |
| ENSSSCG00000001852 | 7 | 55651606 | 55681426 | Carcass length QTL (5987) | 7 | 54402997 | 63250275 |
| ENSSSCG00000001852 | 7 | 55651606 | 55681426 | Conductivity 24 hours post-mortem QTL (1075) | 7 | 54402997 | 61310083 |
| ENSSSCG00000001852 | 7 | 55651606 | 55681426 | Abdominal fat weight QTL (12767) | 7 | 54402997 | 61310083 |
| ENSSSCG00000001852 | 7 | 55651606 | 55681426 | Adipocyte diameter QTL (12826) | 7 | 54402997 | 61310083 |
| ENSSSCG00000001852 | 7 | 55651606 | 55681426 | Adipocyte diameter QTL (12836) | 7 | 54402997 | 61310083 |
| ENSSSCG00000001852 | 7 | 55651606 | 55681426 | Adipocyte diameter QTL (12842) | 7 | 54402997 | 61310083 |
| ENSSSCG00000001852 | 7 | 55651606 | 55681426 | Shoulder subcutaneous fat thickness QTL (5221) | 7 | 2871484 | 1.2E+08 |
| ENSSSCG00000001852 | 7 | 55651606 | 55681426 | Loin muscle area QTL (38092) | 7 | 8459239 | 1.1E+08 |
| ENSSSCG00000001852 | 7 | 55651606 | 55681426 | Meat color a* QTL (38094) | 7 | 8459239 | 69924537 |
| ENSSSCG00000001852 | 7 | 55651606 | 55681426 | Loin fat percentage QTL (38055) | 7 | 17306517 | 1.1E+08 |
| ENSSSCG00000001852 | 7 | 55651606 | 55681426 | Carcass weight (hot) QTL (3) | 7 | 17306517 | 69924537 |
| ENSSSCG00000001852 | 7 | 55651606 | 55681426 | Body weight (end of test) QTL (5236) | 7 | 22506233 | 1.09E+08 |
| ENSSSCG00000001852 | 7 | 55651606 | 55681426 | Carcass length QTL (5700) | 7 | 23437525 | 79315069 |
| ENSSSCG00000001852 | 7 | 55651606 | 55681426 | Backfat at last lumbar QTL (60) | 7 | 25532933 | 1.05E+08 |
| ENSSSCG00000001852 | 7 | 55651606 | 55681426 | backfat above muscle dorsi QTL (5204) | 7 | 34768249 | 73339276 |
| ENSSSCG00000001852 | 7 | 55651606 | 55681426 | Shoulder subcutaneous fat thickness QTL (5220) | 7 | 36553226 | 1.2E+08 |
| ENSSSCG00000001852 | 7 | 55651606 | 55681426 | Backfat at mid-back QTL (5212) | 7 | 36863657 | 68449991 |
| ENSSSCG00000001852 | 7 | 55651606 | 55681426 | Linolenic acid content QTL (156940) | 7 | 37406911 | 1.21E+08 |
| ENSSSCG00000001852 | 7 | 55651606 | 55681426 | Polyunsaturated fatty acid content QTL (156941) | 7 | 37406911 | 1.1E+08 |
| ENSSSCG00000001852 | 7 | 55651606 | 55681426 | Linoleic acid content QTL (156874) | 7 | 37406911 | 1.1E+08 |
| ENSSSCG00000001852 | 7 | 55651606 | 55681426 | Margaric acid content QTL (156872) | 7 | 37406911 | 69924537 |
| ENSSSCG00000001852 | 7 | 55651606 | 55681426 | backfat above muscle dorsi QTL (5239) | 7 | 43770742 | 79237461 |
| ENSSSCG00000001852 | 7 | 55651606 | 55681426 | Average backfat thickness QTL (59) | 7 | 44236389 | 1.05E+08 |
| ENSSSCG00000001852 | 7 | 55651606 | 55681426 | Meat color-L QTL (298) | 7 | 44236389 | 68061952 |
| ENSSSCG00000001852 | 7 | 55651606 | 55681426 | Bicarbonate level QTL (6331) | 7 | 45012466 | 69924537 |
| ENSSSCG00000001852 | 7 | 55651606 | 55681426 | Alkaline phosphatase activity QTL (6346) | 7 | 45012466 | 69924537 |
| ENSSSCG00000001852 | 7 | 55651606 | 55681426 | Alkaline phosphatase activity QTL (6351) | 7 | 45012466 | 69924537 |
| ENSSSCG00000001852 | 7 | 55651606 | 55681426 | Creatinine level QTL (6371) | 7 | 45012466 | 69924537 |
| ENSSSCG00000001852 | 7 | 55651606 | 55681426 | Potassium level QTL (6379) | 7 | 45012466 | 69924537 |
| ENSSSCG00000001852 | 7 | 55651606 | 55681426 | Muscle moisture percentage QTL (38093) | 7 | 45090073 | 1.1E+08 |
| ENSSSCG00000001852 | 7 | 55651606 | 55681426 | Carcass length QTL (29698) | 7 | 45090073 | 81022438 |
| ENSSSCG00000001852 | 7 | 55651606 | 55681426 | Monounsaturated fatty acid content QTL (156875) | 7 | 45090073 | 81022438 |
| ENSSSCG00000001852 | 7 | 55651606 | 55681426 | Oleic acid content QTL (156873) | 7 | 45090073 | 77142053 |
| ENSSSCG00000001852 | 7 | 55651606 | 55681426 | Backfat at last rib QTL (62) | 7 | 46952658 | 1.08E+08 |
| ENSSSCG00000001852 | 7 | 55651606 | 55681426 | lean thickness between 3rd and 4th last ribs QTL (7360) | 7 | 49979358 | 1.27E+08 |
| ENSSSCG00000033954 | 7 | 55840604 | 55900458 | Average daily gain QTL (16852) | 7 | 17228909 | 57507305 |
| ENSSSCG00000033954 | 7 | 55840604 | 55900458 | Average backfat thickness QTL (308) | 7 | 17306517 | 64259175 |
| ENSSSCG00000033954 | 7 | 55840604 | 55900458 | Backfat at last rib QTL (28) | 7 | 23437525 | 64026352 |
| ENSSSCG00000033954 | 7 | 55840604 | 55900458 | Loin muscle depth QTL (366) | 7 | 23437525 | 63250275 |
| ENSSSCG00000033954 | 7 | 55840604 | 55900458 | Average backfat thickness QTL (365) | 7 | 23437525 | 61542906 |
| ENSSSCG00000033954 | 7 | 55840604 | 55900458 | Backfat at last lumbar QTL (2945) | 7 | 23437525 | 61542906 |
| ENSSSCG00000033954 | 7 | 55840604 | 55900458 | Carcass length QTL (11) | 7 | 23437525 | 58438598 |
| ENSSSCG00000033954 | 7 | 55840604 | 55900458 | Average backfat thickness QTL (36) | 7 | 23437525 | 56964051 |
| ENSSSCG00000033954 | 7 | 55840604 | 55900458 | Backfat at first rib QTL (20) | 7 | 25532933 | 58438598 |
| ENSSSCG00000033954 | 7 | 55840604 | 55900458 | Leaf fat weight QTL (39) | 7 | 25532933 | 56964051 |
| ENSSSCG00000033954 | 7 | 55840604 | 55900458 | Leaf fat weight QTL (3880) | 7 | 34380211 | 58438598 |
| ENSSSCG00000033954 | 7 | 55840604 | 55900458 | Backfat at mid-back QTL (5211) | 7 | 36630834 | 57740128 |
| ENSSSCG00000033954 | 7 | 55840604 | 55900458 | Carcass length QTL (3254) | 7 | 37406911 | 58438598 |
| ENSSSCG00000033954 | 7 | 55840604 | 55900458 | Subcutanous fat area QTL (18006) | 7 | 39890357 | 60456398 |
| ENSSSCG00000033954 | 7 | 55840604 | 55900458 | Backfat at mid-back QTL (282) | 7 | 44236389 | 58438598 |
| ENSSSCG00000033954 | 7 | 55840604 | 55900458 | Backfat thickness between 3rd and 4th rib QTL (419) | 7 | 44236389 | 56653621 |
| ENSSSCG00000033954 | 7 | 55840604 | 55900458 | Shoulder subcutaneous fat thickness QTL (5252) | 7 | 44702035 | 56187974 |
| ENSSSCG00000033954 | 7 | 55840604 | 55900458 | Backfat at last rib QTL (16837) | 7 | 44779642 | 61310083 |
| ENSSSCG00000033954 | 7 | 55840604 | 55900458 | Backfat at first rib QTL (512) | 7 | 44779642 | 56964051 |
| ENSSSCG00000033954 | 7 | 55840604 | 55900458 | Backfat at last rib QTL (513) | 7 | 44779642 | 56964051 |
| ENSSSCG00000033954 | 7 | 55840604 | 55900458 | Backfat at last lumbar QTL (514) | 7 | 44779642 | 56964051 |
| ENSSSCG00000033954 | 7 | 55840604 | 55900458 | androstenone, laboratory QTL (4209) | 7 | 45012466 | 64259175 |
| ENSSSCG00000033954 | 7 | 55840604 | 55900458 | Palmitoleic acid content QTL (9572) | 7 | 45012466 | 56964051 |
| ENSSSCG00000033954 | 7 | 55840604 | 55900458 | body weight (10 weeks) QTL (188) | 7 | 45710935 | 56964051 |
| ENSSSCG00000033954 | 7 | 55840604 | 55900458 | body weight (13 weeks) QTL (189) | 7 | 45710935 | 56964051 |
| ENSSSCG00000033954 | 7 | 55840604 | 55900458 | body weight (17 weeks) QTL (190) | 7 | 45710935 | 56964051 |
| ENSSSCG00000033954 | 7 | 55840604 | 55900458 | Average daily gain QTL (191) | 7 | 45710935 | 56964051 |
| ENSSSCG00000033954 | 7 | 55840604 | 55900458 | Loin muscle depth QTL (390) | 7 | 46564620 | 66742622 |
| ENSSSCG00000033954 | 7 | 55840604 | 55900458 | Backfat at last rib QTL (420) | 7 | 46564620 | 57429698 |
| ENSSSCG00000033954 | 7 | 55840604 | 55900458 | Carcass length QTL (21385) | 7 | 46952658 | 58438598 |
| ENSSSCG00000033954 | 7 | 55840604 | 55900458 | Loin muscle area QTL (21386) | 7 | 46952658 | 58438598 |
| ENSSSCG00000033954 | 7 | 55840604 | 55900458 | Backfat at last rib QTL (17802) | 7 | 47728735 | 66121760 |
| ENSSSCG00000033954 | 7 | 55840604 | 55900458 | Average daily gain QTL (5944) | 7 | 54402997 | 63250275 |
| ENSSSCG00000033954 | 7 | 55840604 | 55900458 | Carcass length QTL (5987) | 7 | 54402997 | 63250275 |
| ENSSSCG00000033954 | 7 | 55840604 | 55900458 | Conductivity 24 hours post-mortem QTL (1075) | 7 | 54402997 | 61310083 |
| ENSSSCG00000033954 | 7 | 55840604 | 55900458 | Abdominal fat weight QTL (12767) | 7 | 54402997 | 61310083 |
| ENSSSCG00000033954 | 7 | 55840604 | 55900458 | Adipocyte diameter QTL (12826) | 7 | 54402997 | 61310083 |
| ENSSSCG00000033954 | 7 | 55840604 | 55900458 | Adipocyte diameter QTL (12836) | 7 | 54402997 | 61310083 |
| ENSSSCG00000033954 | 7 | 55840604 | 55900458 | Adipocyte diameter QTL (12842) | 7 | 54402997 | 61310083 |
| ENSSSCG00000033954 | 7 | 55840604 | 55900458 | Shoulder subcutaneous fat thickness QTL (5221) | 7 | 2871484 | 1.2E+08 |
| ENSSSCG00000033954 | 7 | 55840604 | 55900458 | Loin muscle area QTL (38092) | 7 | 8459239 | 1.1E+08 |
| ENSSSCG00000033954 | 7 | 55840604 | 55900458 | Meat color a* QTL (38094) | 7 | 8459239 | 69924537 |
| ENSSSCG00000033954 | 7 | 55840604 | 55900458 | Loin fat percentage QTL (38055) | 7 | 17306517 | 1.1E+08 |
| ENSSSCG00000033954 | 7 | 55840604 | 55900458 | Carcass weight (hot) QTL (3) | 7 | 17306517 | 69924537 |
| ENSSSCG00000033954 | 7 | 55840604 | 55900458 | Body weight (end of test) QTL (5236) | 7 | 22506233 | 1.09E+08 |
| ENSSSCG00000033954 | 7 | 55840604 | 55900458 | Carcass length QTL (5700) | 7 | 23437525 | 79315069 |
| ENSSSCG00000033954 | 7 | 55840604 | 55900458 | Backfat at last lumbar QTL (60) | 7 | 25532933 | 1.05E+08 |
| ENSSSCG00000033954 | 7 | 55840604 | 55900458 | backfat above muscle dorsi QTL (5204) | 7 | 34768249 | 73339276 |
| ENSSSCG00000033954 | 7 | 55840604 | 55900458 | Shoulder subcutaneous fat thickness QTL (5220) | 7 | 36553226 | 1.2E+08 |
| ENSSSCG00000033954 | 7 | 55840604 | 55900458 | Backfat at mid-back QTL (5212) | 7 | 36863657 | 68449991 |
| ENSSSCG00000033954 | 7 | 55840604 | 55900458 | Linolenic acid content QTL (156940) | 7 | 37406911 | 1.21E+08 |
| ENSSSCG00000033954 | 7 | 55840604 | 55900458 | Polyunsaturated fatty acid content QTL (156941) | 7 | 37406911 | 1.1E+08 |
| ENSSSCG00000033954 | 7 | 55840604 | 55900458 | Linoleic acid content QTL (156874) | 7 | 37406911 | 1.1E+08 |
| ENSSSCG00000033954 | 7 | 55840604 | 55900458 | Margaric acid content QTL (156872) | 7 | 37406911 | 69924537 |
| ENSSSCG00000033954 | 7 | 55840604 | 55900458 | backfat above muscle dorsi QTL (5239) | 7 | 43770742 | 79237461 |
| ENSSSCG00000033954 | 7 | 55840604 | 55900458 | Average backfat thickness QTL (59) | 7 | 44236389 | 1.05E+08 |
| ENSSSCG00000033954 | 7 | 55840604 | 55900458 | Meat color-L QTL (298) | 7 | 44236389 | 68061952 |
| ENSSSCG00000033954 | 7 | 55840604 | 55900458 | Bicarbonate level QTL (6331) | 7 | 45012466 | 69924537 |
| ENSSSCG00000033954 | 7 | 55840604 | 55900458 | Alkaline phosphatase activity QTL (6346) | 7 | 45012466 | 69924537 |
| ENSSSCG00000033954 | 7 | 55840604 | 55900458 | Alkaline phosphatase activity QTL (6351) | 7 | 45012466 | 69924537 |
| ENSSSCG00000033954 | 7 | 55840604 | 55900458 | Creatinine level QTL (6371) | 7 | 45012466 | 69924537 |
| ENSSSCG00000033954 | 7 | 55840604 | 55900458 | Potassium level QTL (6379) | 7 | 45012466 | 69924537 |
| ENSSSCG00000033954 | 7 | 55840604 | 55900458 | Muscle moisture percentage QTL (38093) | 7 | 45090073 | 1.1E+08 |
| ENSSSCG00000033954 | 7 | 55840604 | 55900458 | Carcass length QTL (29698) | 7 | 45090073 | 81022438 |
| ENSSSCG00000033954 | 7 | 55840604 | 55900458 | Monounsaturated fatty acid content QTL (156875) | 7 | 45090073 | 81022438 |
| ENSSSCG00000033954 | 7 | 55840604 | 55900458 | Oleic acid content QTL (156873) | 7 | 45090073 | 77142053 |
| ENSSSCG00000033954 | 7 | 55840604 | 55900458 | Backfat at last rib QTL (62) | 7 | 46952658 | 1.08E+08 |
| ENSSSCG00000033954 | 7 | 55840604 | 55900458 | lean thickness between 3rd and 4th last ribs QTL (7360) | 7 | 49979358 | 1.27E+08 |
| ENSSSCG00000001862 | 7 | 56440620 | 56540253 | Average daily gain QTL (16852) | 7 | 17228909 | 57507305 |
| ENSSSCG00000001862 | 7 | 56440620 | 56540253 | Average backfat thickness QTL (308) | 7 | 17306517 | 64259175 |
| ENSSSCG00000001862 | 7 | 56440620 | 56540253 | Backfat at last rib QTL (28) | 7 | 23437525 | 64026352 |
| ENSSSCG00000001862 | 7 | 56440620 | 56540253 | Loin muscle depth QTL (366) | 7 | 23437525 | 63250275 |
| ENSSSCG00000001862 | 7 | 56440620 | 56540253 | Average backfat thickness QTL (365) | 7 | 23437525 | 61542906 |
| ENSSSCG00000001862 | 7 | 56440620 | 56540253 | Backfat at last lumbar QTL (2945) | 7 | 23437525 | 61542906 |
| ENSSSCG00000001862 | 7 | 56440620 | 56540253 | Carcass length QTL (11) | 7 | 23437525 | 58438598 |
| ENSSSCG00000001862 | 7 | 56440620 | 56540253 | Average backfat thickness QTL (36) | 7 | 23437525 | 56964051 |
| ENSSSCG00000001862 | 7 | 56440620 | 56540253 | Backfat at first rib QTL (20) | 7 | 25532933 | 58438598 |
| ENSSSCG00000001862 | 7 | 56440620 | 56540253 | Leaf fat weight QTL (39) | 7 | 25532933 | 56964051 |
| ENSSSCG00000001862 | 7 | 56440620 | 56540253 | Leaf fat weight QTL (3880) | 7 | 34380211 | 58438598 |
| ENSSSCG00000001862 | 7 | 56440620 | 56540253 | Backfat at mid-back QTL (5211) | 7 | 36630834 | 57740128 |
| ENSSSCG00000001862 | 7 | 56440620 | 56540253 | Carcass length QTL (3254) | 7 | 37406911 | 58438598 |
| ENSSSCG00000001862 | 7 | 56440620 | 56540253 | Subcutanous fat area QTL (18006) | 7 | 39890357 | 60456398 |
| ENSSSCG00000001862 | 7 | 56440620 | 56540253 | Backfat at mid-back QTL (282) | 7 | 44236389 | 58438598 |
| ENSSSCG00000001862 | 7 | 56440620 | 56540253 | Backfat thickness between 3rd and 4th rib QTL (419) | 7 | 44236389 | 56653621 |
| ENSSSCG00000001862 | 7 | 56440620 | 56540253 | Backfat at last rib QTL (16837) | 7 | 44779642 | 61310083 |
| ENSSSCG00000001862 | 7 | 56440620 | 56540253 | Backfat at first rib QTL (512) | 7 | 44779642 | 56964051 |
| ENSSSCG00000001862 | 7 | 56440620 | 56540253 | Backfat at last rib QTL (513) | 7 | 44779642 | 56964051 |
| ENSSSCG00000001862 | 7 | 56440620 | 56540253 | Backfat at last lumbar QTL (514) | 7 | 44779642 | 56964051 |
| ENSSSCG00000001862 | 7 | 56440620 | 56540253 | androstenone, laboratory QTL (4209) | 7 | 45012466 | 64259175 |
| ENSSSCG00000001862 | 7 | 56440620 | 56540253 | Palmitoleic acid content QTL (9572) | 7 | 45012466 | 56964051 |
| ENSSSCG00000001862 | 7 | 56440620 | 56540253 | body weight (10 weeks) QTL (188) | 7 | 45710935 | 56964051 |
| ENSSSCG00000001862 | 7 | 56440620 | 56540253 | body weight (13 weeks) QTL (189) | 7 | 45710935 | 56964051 |
| ENSSSCG00000001862 | 7 | 56440620 | 56540253 | body weight (17 weeks) QTL (190) | 7 | 45710935 | 56964051 |
| ENSSSCG00000001862 | 7 | 56440620 | 56540253 | Average daily gain QTL (191) | 7 | 45710935 | 56964051 |
| ENSSSCG00000001862 | 7 | 56440620 | 56540253 | Loin muscle depth QTL (390) | 7 | 46564620 | 66742622 |
| ENSSSCG00000001862 | 7 | 56440620 | 56540253 | Backfat at last rib QTL (420) | 7 | 46564620 | 57429698 |
| ENSSSCG00000001862 | 7 | 56440620 | 56540253 | Carcass length QTL (21385) | 7 | 46952658 | 58438598 |
| ENSSSCG00000001862 | 7 | 56440620 | 56540253 | Loin muscle area QTL (21386) | 7 | 46952658 | 58438598 |
| ENSSSCG00000001862 | 7 | 56440620 | 56540253 | Backfat at last rib QTL (17802) | 7 | 47728735 | 66121760 |
| ENSSSCG00000001862 | 7 | 56440620 | 56540253 | Average daily gain QTL (5944) | 7 | 54402997 | 63250275 |
| ENSSSCG00000001862 | 7 | 56440620 | 56540253 | Carcass length QTL (5987) | 7 | 54402997 | 63250275 |
| ENSSSCG00000001862 | 7 | 56440620 | 56540253 | Conductivity 24 hours post-mortem QTL (1075) | 7 | 54402997 | 61310083 |
| ENSSSCG00000001862 | 7 | 56440620 | 56540253 | Abdominal fat weight QTL (12767) | 7 | 54402997 | 61310083 |
| ENSSSCG00000001862 | 7 | 56440620 | 56540253 | Adipocyte diameter QTL (12826) | 7 | 54402997 | 61310083 |
| ENSSSCG00000001862 | 7 | 56440620 | 56540253 | Adipocyte diameter QTL (12836) | 7 | 54402997 | 61310083 |
| ENSSSCG00000001862 | 7 | 56440620 | 56540253 | Adipocyte diameter QTL (12842) | 7 | 54402997 | 61310083 |
| ENSSSCG00000001862 | 7 | 56440620 | 56540253 | Shoulder subcutaneous fat thickness QTL (5221) | 7 | 2871484 | 1.2E+08 |
| ENSSSCG00000001862 | 7 | 56440620 | 56540253 | Loin muscle area QTL (38092) | 7 | 8459239 | 1.1E+08 |
| ENSSSCG00000001862 | 7 | 56440620 | 56540253 | Meat color a* QTL (38094) | 7 | 8459239 | 69924537 |
| ENSSSCG00000001862 | 7 | 56440620 | 56540253 | Loin fat percentage QTL (38055) | 7 | 17306517 | 1.1E+08 |
| ENSSSCG00000001862 | 7 | 56440620 | 56540253 | Carcass weight (hot) QTL (3) | 7 | 17306517 | 69924537 |
| ENSSSCG00000001862 | 7 | 56440620 | 56540253 | Body weight (end of test) QTL (5236) | 7 | 22506233 | 1.09E+08 |
| ENSSSCG00000001862 | 7 | 56440620 | 56540253 | Carcass length QTL (5700) | 7 | 23437525 | 79315069 |
| ENSSSCG00000001862 | 7 | 56440620 | 56540253 | Backfat at last lumbar QTL (60) | 7 | 25532933 | 1.05E+08 |
| ENSSSCG00000001862 | 7 | 56440620 | 56540253 | backfat above muscle dorsi QTL (5204) | 7 | 34768249 | 73339276 |
| ENSSSCG00000001862 | 7 | 56440620 | 56540253 | Shoulder subcutaneous fat thickness QTL (5220) | 7 | 36553226 | 1.2E+08 |
| ENSSSCG00000001862 | 7 | 56440620 | 56540253 | Backfat at mid-back QTL (5212) | 7 | 36863657 | 68449991 |
| ENSSSCG00000001862 | 7 | 56440620 | 56540253 | Linolenic acid content QTL (156940) | 7 | 37406911 | 1.21E+08 |
| ENSSSCG00000001862 | 7 | 56440620 | 56540253 | Polyunsaturated fatty acid content QTL (156941) | 7 | 37406911 | 1.1E+08 |
| ENSSSCG00000001862 | 7 | 56440620 | 56540253 | Linoleic acid content QTL (156874) | 7 | 37406911 | 1.1E+08 |
| ENSSSCG00000001862 | 7 | 56440620 | 56540253 | Margaric acid content QTL (156872) | 7 | 37406911 | 69924537 |
| ENSSSCG00000001862 | 7 | 56440620 | 56540253 | backfat above muscle dorsi QTL (5239) | 7 | 43770742 | 79237461 |
| ENSSSCG00000001862 | 7 | 56440620 | 56540253 | Average backfat thickness QTL (59) | 7 | 44236389 | 1.05E+08 |
| ENSSSCG00000001862 | 7 | 56440620 | 56540253 | Meat color-L QTL (298) | 7 | 44236389 | 68061952 |
| ENSSSCG00000001862 | 7 | 56440620 | 56540253 | Bicarbonate level QTL (6331) | 7 | 45012466 | 69924537 |
| ENSSSCG00000001862 | 7 | 56440620 | 56540253 | Alkaline phosphatase activity QTL (6346) | 7 | 45012466 | 69924537 |
| ENSSSCG00000001862 | 7 | 56440620 | 56540253 | Alkaline phosphatase activity QTL (6351) | 7 | 45012466 | 69924537 |
| ENSSSCG00000001862 | 7 | 56440620 | 56540253 | Creatinine level QTL (6371) | 7 | 45012466 | 69924537 |
| ENSSSCG00000001862 | 7 | 56440620 | 56540253 | Potassium level QTL (6379) | 7 | 45012466 | 69924537 |
| ENSSSCG00000001862 | 7 | 56440620 | 56540253 | Muscle moisture percentage QTL (38093) | 7 | 45090073 | 1.1E+08 |
| ENSSSCG00000001862 | 7 | 56440620 | 56540253 | Carcass length QTL (29698) | 7 | 45090073 | 81022438 |
| ENSSSCG00000001862 | 7 | 56440620 | 56540253 | Monounsaturated fatty acid content QTL (156875) | 7 | 45090073 | 81022438 |
| ENSSSCG00000001862 | 7 | 56440620 | 56540253 | Oleic acid content QTL (156873) | 7 | 45090073 | 77142053 |
| ENSSSCG00000001862 | 7 | 56440620 | 56540253 | Backfat at last rib QTL (62) | 7 | 46952658 | 1.08E+08 |
| ENSSSCG00000001862 | 7 | 56440620 | 56540253 | lean thickness between 3rd and 4th last ribs QTL (7360) | 7 | 49979358 | 1.27E+08 |
| ENSSSCG00000001903 | 7 | 58828726 | 58897923 | Average glycolytic potential QTL (13459) | 7 | 58857679 | 59191392 |
| ENSSSCG00000001903 | 7 | 58828726 | 58897923 | Average lactate QTL (13460) | 7 | 58857679 | 59191392 |
| ENSSSCG00000001903 | 7 | 58828726 | 58897923 | ATP breakdown rate QTL (13461) | 7 | 58857679 | 59191392 |
| ENSSSCG00000001903 | 7 | 58828726 | 58897923 | pH 24 hr post-mortem (loin) QTL (13462) | 7 | 58857679 | 59191392 |
| ENSSSCG00000001903 | 7 | 58828726 | 58897923 | PH for Longissmus dorsi QTL (13463) | 7 | 58857679 | 59191392 |
| ENSSSCG00000001903 | 7 | 58828726 | 58897923 | Drip loss QTL (13464) | 7 | 58857679 | 59191392 |
| ENSSSCG00000001903 | 7 | 58828726 | 58897923 | Drip loss QTL (13465) | 7 | 58857679 | 59191392 |
| ENSSSCG00000001903 | 7 | 58828726 | 58897923 | Average glycolytic potential QTL (13466) | 7 | 58857679 | 59191392 |
| ENSSSCG00000001903 | 7 | 58828726 | 58897923 | Average glycogen QTL (13467) | 7 | 58857679 | 59191392 |
| ENSSSCG00000001903 | 7 | 58828726 | 58897923 | ATP breakdown rate QTL (13468) | 7 | 58857679 | 59191392 |
| ENSSSCG00000001903 | 7 | 58828726 | 58897923 | PH for Longissmus dorsi QTL (13469) | 7 | 58857679 | 59191392 |
| ENSSSCG00000001903 | 7 | 58828726 | 58897923 | PH for Longissmus dorsi QTL (13470) | 7 | 58857679 | 59191392 |
| ENSSSCG00000001903 | 7 | 58828726 | 58897923 | Meat color L* QTL (13471) | 7 | 58857679 | 59191392 |
| ENSSSCG00000001903 | 7 | 58828726 | 58897923 | Drip loss QTL (13472) | 7 | 58857679 | 59191392 |
| ENSSSCG00000001903 | 7 | 58828726 | 58897923 | Average lactate QTL (13473) | 7 | 58857679 | 59191392 |
| ENSSSCG00000001903 | 7 | 58828726 | 58897923 | cis-Vaccenic acid content QTL (17755) | 7 | 58857679 | 59191392 |
| ENSSSCG00000001903 | 7 | 58828726 | 58897923 | Average backfat thickness QTL (308) | 7 | 17306517 | 64259175 |
| ENSSSCG00000001903 | 7 | 58828726 | 58897923 | Backfat at last rib QTL (28) | 7 | 23437525 | 64026352 |
| ENSSSCG00000001903 | 7 | 58828726 | 58897923 | Loin muscle depth QTL (366) | 7 | 23437525 | 63250275 |
| ENSSSCG00000001903 | 7 | 58828726 | 58897923 | Average backfat thickness QTL (365) | 7 | 23437525 | 61542906 |
| ENSSSCG00000001903 | 7 | 58828726 | 58897923 | Backfat at last lumbar QTL (2945) | 7 | 23437525 | 61542906 |
| ENSSSCG00000001903 | 7 | 58828726 | 58897923 | Subcutanous fat area QTL (18006) | 7 | 39890357 | 60456398 |
| ENSSSCG00000001903 | 7 | 58828726 | 58897923 | Backfat at last rib QTL (16837) | 7 | 44779642 | 61310083 |
| ENSSSCG00000001903 | 7 | 58828726 | 58897923 | androstenone, laboratory QTL (4209) | 7 | 45012466 | 64259175 |
| ENSSSCG00000001903 | 7 | 58828726 | 58897923 | Loin muscle depth QTL (390) | 7 | 46564620 | 66742622 |
| ENSSSCG00000001903 | 7 | 58828726 | 58897923 | Backfat at last rib QTL (17802) | 7 | 47728735 | 66121760 |
| ENSSSCG00000001903 | 7 | 58828726 | 58897923 | Average daily gain QTL (5944) | 7 | 54402997 | 63250275 |
| ENSSSCG00000001903 | 7 | 58828726 | 58897923 | Carcass length QTL (5987) | 7 | 54402997 | 63250275 |
| ENSSSCG00000001903 | 7 | 58828726 | 58897923 | Conductivity 24 hours post-mortem QTL (1075) | 7 | 54402997 | 61310083 |
| ENSSSCG00000001903 | 7 | 58828726 | 58897923 | Abdominal fat weight QTL (12767) | 7 | 54402997 | 61310083 |
| ENSSSCG00000001903 | 7 | 58828726 | 58897923 | Adipocyte diameter QTL (12826) | 7 | 54402997 | 61310083 |
| ENSSSCG00000001903 | 7 | 58828726 | 58897923 | Adipocyte diameter QTL (12836) | 7 | 54402997 | 61310083 |
| ENSSSCG00000001903 | 7 | 58828726 | 58897923 | Adipocyte diameter QTL (12842) | 7 | 54402997 | 61310083 |
| ENSSSCG00000001903 | 7 | 58828726 | 58897923 | Shoulder subcutaneous fat thickness QTL (5221) | 7 | 2871484 | 1.2E+08 |
| ENSSSCG00000001903 | 7 | 58828726 | 58897923 | Loin muscle area QTL (38092) | 7 | 8459239 | 1.1E+08 |
| ENSSSCG00000001903 | 7 | 58828726 | 58897923 | Meat color a* QTL (38094) | 7 | 8459239 | 69924537 |
| ENSSSCG00000001903 | 7 | 58828726 | 58897923 | Loin fat percentage QTL (38055) | 7 | 17306517 | 1.1E+08 |
| ENSSSCG00000001903 | 7 | 58828726 | 58897923 | Carcass weight (hot) QTL (3) | 7 | 17306517 | 69924537 |
| ENSSSCG00000001903 | 7 | 58828726 | 58897923 | Body weight (end of test) QTL (5236) | 7 | 22506233 | 1.09E+08 |
| ENSSSCG00000001903 | 7 | 58828726 | 58897923 | Carcass length QTL (5700) | 7 | 23437525 | 79315069 |
| ENSSSCG00000001903 | 7 | 58828726 | 58897923 | Backfat at last lumbar QTL (60) | 7 | 25532933 | 1.05E+08 |
| ENSSSCG00000001903 | 7 | 58828726 | 58897923 | backfat above muscle dorsi QTL (5204) | 7 | 34768249 | 73339276 |
| ENSSSCG00000001903 | 7 | 58828726 | 58897923 | Shoulder subcutaneous fat thickness QTL (5220) | 7 | 36553226 | 1.2E+08 |
| ENSSSCG00000001903 | 7 | 58828726 | 58897923 | Backfat at mid-back QTL (5212) | 7 | 36863657 | 68449991 |
| ENSSSCG00000001903 | 7 | 58828726 | 58897923 | Linolenic acid content QTL (156940) | 7 | 37406911 | 1.21E+08 |
| ENSSSCG00000001903 | 7 | 58828726 | 58897923 | Polyunsaturated fatty acid content QTL (156941) | 7 | 37406911 | 1.1E+08 |
| ENSSSCG00000001903 | 7 | 58828726 | 58897923 | Linoleic acid content QTL (156874) | 7 | 37406911 | 1.1E+08 |
| ENSSSCG00000001903 | 7 | 58828726 | 58897923 | Margaric acid content QTL (156872) | 7 | 37406911 | 69924537 |
| ENSSSCG00000001903 | 7 | 58828726 | 58897923 | backfat above muscle dorsi QTL (5239) | 7 | 43770742 | 79237461 |
| ENSSSCG00000001903 | 7 | 58828726 | 58897923 | Average backfat thickness QTL (59) | 7 | 44236389 | 1.05E+08 |
| ENSSSCG00000001903 | 7 | 58828726 | 58897923 | Meat color-L QTL (298) | 7 | 44236389 | 68061952 |
| ENSSSCG00000001903 | 7 | 58828726 | 58897923 | Bicarbonate level QTL (6331) | 7 | 45012466 | 69924537 |
| ENSSSCG00000001903 | 7 | 58828726 | 58897923 | Alkaline phosphatase activity QTL (6346) | 7 | 45012466 | 69924537 |
| ENSSSCG00000001903 | 7 | 58828726 | 58897923 | Alkaline phosphatase activity QTL (6351) | 7 | 45012466 | 69924537 |
| ENSSSCG00000001903 | 7 | 58828726 | 58897923 | Creatinine level QTL (6371) | 7 | 45012466 | 69924537 |
| ENSSSCG00000001903 | 7 | 58828726 | 58897923 | Potassium level QTL (6379) | 7 | 45012466 | 69924537 |
| ENSSSCG00000001903 | 7 | 58828726 | 58897923 | Muscle moisture percentage QTL (38093) | 7 | 45090073 | 1.1E+08 |
| ENSSSCG00000001903 | 7 | 58828726 | 58897923 | Carcass length QTL (29698) | 7 | 45090073 | 81022438 |
| ENSSSCG00000001903 | 7 | 58828726 | 58897923 | Monounsaturated fatty acid content QTL (156875) | 7 | 45090073 | 81022438 |
| ENSSSCG00000001903 | 7 | 58828726 | 58897923 | Oleic acid content QTL (156873) | 7 | 45090073 | 77142053 |
| ENSSSCG00000001903 | 7 | 58828726 | 58897923 | Backfat at last rib QTL (62) | 7 | 46952658 | 1.08E+08 |
| ENSSSCG00000001903 | 7 | 58828726 | 58897923 | lean thickness between 3rd and 4th last ribs QTL (7360) | 7 | 49979358 | 1.27E+08 |
| ENSSSCG00000001903 | 7 | 58828726 | 58897923 | Muscle moisture percentage QTL (2989) | 7 | 56964051 | 69924537 |
| ENSSSCG00000001903 | 7 | 58828726 | 58897923 | Backfat at tenth rib QTL (61) | 7 | 58438598 | 1.05E+08 |
| ENSSSCG00000001903 | 7 | 58828726 | 58897923 | Loin muscle area QTL (3256) | 7 | 58438598 | 79315069 |
| ENSSSCG00000001903 | 7 | 58828726 | 58897923 | Average daily gain QTL (648) | 7 | 58438598 | 77142053 |
| ENSSSCG00000001903 | 7 | 58828726 | 58897923 | Loin muscle area QTL (16927) | 7 | 58438598 | 69846930 |
| ENSSSCG00000001903 | 7 | 58828726 | 58897923 | Marbling QTL (21388) | 7 | 58438598 | 69846930 |
| ENSSSCG00000001950 | 7 | 64578241 | 64763281 | Muscle moisture percentage QTL (3883) | 7 | 64662735 | 65011970 |
| ENSSSCG00000001950 | 7 | 64578241 | 64763281 | Loin muscle depth QTL (390) | 7 | 46564620 | 66742622 |
| ENSSSCG00000001950 | 7 | 64578241 | 64763281 | Backfat at last rib QTL (17802) | 7 | 47728735 | 66121760 |
| ENSSSCG00000001950 | 7 | 64578241 | 64763281 | Shoulder subcutaneous fat thickness QTL (5221) | 7 | 2871484 | 1.2E+08 |
| ENSSSCG00000001950 | 7 | 64578241 | 64763281 | Loin muscle area QTL (38092) | 7 | 8459239 | 1.1E+08 |
| ENSSSCG00000001950 | 7 | 64578241 | 64763281 | Meat color a* QTL (38094) | 7 | 8459239 | 69924537 |
| ENSSSCG00000001950 | 7 | 64578241 | 64763281 | Loin fat percentage QTL (38055) | 7 | 17306517 | 1.1E+08 |
| ENSSSCG00000001950 | 7 | 64578241 | 64763281 | Carcass weight (hot) QTL (3) | 7 | 17306517 | 69924537 |
| ENSSSCG00000001950 | 7 | 64578241 | 64763281 | Body weight (end of test) QTL (5236) | 7 | 22506233 | 1.09E+08 |
| ENSSSCG00000001950 | 7 | 64578241 | 64763281 | Carcass length QTL (5700) | 7 | 23437525 | 79315069 |
| ENSSSCG00000001950 | 7 | 64578241 | 64763281 | Backfat at last lumbar QTL (60) | 7 | 25532933 | 1.05E+08 |
| ENSSSCG00000001950 | 7 | 64578241 | 64763281 | backfat above muscle dorsi QTL (5204) | 7 | 34768249 | 73339276 |
| ENSSSCG00000001950 | 7 | 64578241 | 64763281 | Shoulder subcutaneous fat thickness QTL (5220) | 7 | 36553226 | 1.2E+08 |
| ENSSSCG00000001950 | 7 | 64578241 | 64763281 | Backfat at mid-back QTL (5212) | 7 | 36863657 | 68449991 |
| ENSSSCG00000001950 | 7 | 64578241 | 64763281 | Linolenic acid content QTL (156940) | 7 | 37406911 | 1.21E+08 |
| ENSSSCG00000001950 | 7 | 64578241 | 64763281 | Polyunsaturated fatty acid content QTL (156941) | 7 | 37406911 | 1.1E+08 |
| ENSSSCG00000001950 | 7 | 64578241 | 64763281 | Linoleic acid content QTL (156874) | 7 | 37406911 | 1.1E+08 |
| ENSSSCG00000001950 | 7 | 64578241 | 64763281 | Margaric acid content QTL (156872) | 7 | 37406911 | 69924537 |
| ENSSSCG00000001950 | 7 | 64578241 | 64763281 | backfat above muscle dorsi QTL (5239) | 7 | 43770742 | 79237461 |
| ENSSSCG00000001950 | 7 | 64578241 | 64763281 | Average backfat thickness QTL (59) | 7 | 44236389 | 1.05E+08 |
| ENSSSCG00000001950 | 7 | 64578241 | 64763281 | Meat color-L QTL (298) | 7 | 44236389 | 68061952 |
| ENSSSCG00000001950 | 7 | 64578241 | 64763281 | Bicarbonate level QTL (6331) | 7 | 45012466 | 69924537 |
| ENSSSCG00000001950 | 7 | 64578241 | 64763281 | Alkaline phosphatase activity QTL (6346) | 7 | 45012466 | 69924537 |
| ENSSSCG00000001950 | 7 | 64578241 | 64763281 | Alkaline phosphatase activity QTL (6351) | 7 | 45012466 | 69924537 |
| ENSSSCG00000001950 | 7 | 64578241 | 64763281 | Creatinine level QTL (6371) | 7 | 45012466 | 69924537 |
| ENSSSCG00000001950 | 7 | 64578241 | 64763281 | Potassium level QTL (6379) | 7 | 45012466 | 69924537 |
| ENSSSCG00000001950 | 7 | 64578241 | 64763281 | Muscle moisture percentage QTL (38093) | 7 | 45090073 | 1.1E+08 |
| ENSSSCG00000001950 | 7 | 64578241 | 64763281 | Carcass length QTL (29698) | 7 | 45090073 | 81022438 |
| ENSSSCG00000001950 | 7 | 64578241 | 64763281 | Monounsaturated fatty acid content QTL (156875) | 7 | 45090073 | 81022438 |
| ENSSSCG00000001950 | 7 | 64578241 | 64763281 | Oleic acid content QTL (156873) | 7 | 45090073 | 77142053 |
| ENSSSCG00000001950 | 7 | 64578241 | 64763281 | Backfat at last rib QTL (62) | 7 | 46952658 | 1.08E+08 |
| ENSSSCG00000001950 | 7 | 64578241 | 64763281 | lean thickness between 3rd and 4th last ribs QTL (7360) | 7 | 49979358 | 1.27E+08 |
| ENSSSCG00000001950 | 7 | 64578241 | 64763281 | Muscle moisture percentage QTL (2989) | 7 | 56964051 | 69924537 |
| ENSSSCG00000001950 | 7 | 64578241 | 64763281 | Backfat at tenth rib QTL (61) | 7 | 58438598 | 1.05E+08 |
| ENSSSCG00000001950 | 7 | 64578241 | 64763281 | Loin muscle area QTL (3256) | 7 | 58438598 | 79315069 |
| ENSSSCG00000001950 | 7 | 64578241 | 64763281 | Average daily gain QTL (648) | 7 | 58438598 | 77142053 |
| ENSSSCG00000001950 | 7 | 64578241 | 64763281 | Loin muscle area QTL (16927) | 7 | 58438598 | 69846930 |
| ENSSSCG00000001950 | 7 | 64578241 | 64763281 | Marbling QTL (21388) | 7 | 58438598 | 69846930 |
| ENSSSCG00000001950 | 7 | 64578241 | 64763281 | Backfat at last rib QTL (2947) | 7 | 61542906 | 69846930 |
| ENSSSCG00000001950 | 7 | 64578241 | 64763281 | Average backfat thickness QTL (2948) | 7 | 61542906 | 69846930 |
| ENSSSCG00000001950 | 7 | 64578241 | 64763281 | Interferon-gamma level QTL (12265) | 7 | 62707021 | 69924537 |
| ENSSSCG00000001950 | 7 | 64578241 | 64763281 | Meat color b* QTL (3994) | 7 | 63250275 | 81022438 |
| ENSSSCG00000001950 | 7 | 64578241 | 64763281 | Shear force QTL (3996) | 7 | 63250275 | 81022438 |
| ENSSSCG00000001950 | 7 | 64578241 | 64763281 | Total shear work QTL (3998) | 7 | 63250275 | 81022438 |
| ENSSSCG00000001950 | 7 | 64578241 | 64763281 | Drip loss QTL (9853) | 7 | 63250275 | 79315069 |
| ENSSSCG00000001950 | 7 | 64578241 | 64763281 | pH 24 hr post-mortem (loin) QTL (9854) | 7 | 63250275 | 79315069 |
| ENSSSCG00000001950 | 7 | 64578241 | 64763281 | Backfat at tenth rib QTL (5701) | 7 | 63250275 | 79315069 |
| ENSSSCG00000001950 | 7 | 64578241 | 64763281 | Interleukin-2 level QTL (17600) | 7 | 63250275 | 79315069 |
| ENSSSCG00000001950 | 7 | 64578241 | 64763281 | Cholesterol level QTL (17720) | 7 | 63250275 | 79315069 |
| ENSSSCG00000001950 | 7 | 64578241 | 64763281 | LDL cholesterol QTL (17721) | 7 | 63250275 | 79315069 |
| ENSSSCG00000001950 | 7 | 64578241 | 64763281 | Carcass length QTL (649) | 7 | 64259175 | 1.05E+08 |
| ENSSSCG00000001950 | 7 | 64578241 | 64763281 | Belly percentage QTL (650) | 7 | 64259175 | 1.05E+08 |
| ENSSSCG00000001950 | 7 | 64578241 | 64763281 | pH 24 hr post-mortem (loin) QTL (1065) | 7 | 64259175 | 79315069 |
| ENSSSCG00000001950 | 7 | 64578241 | 64763281 | Adipocyte diameter QTL (12822) | 7 | 64259175 | 79315069 |
| ENSSSCG00000001950 | 7 | 64578241 | 64763281 | Adipocyte diameter QTL (12823) | 7 | 64259175 | 79315069 |
| ENSSSCG00000001950 | 7 | 64578241 | 64763281 | Adipocyte diameter QTL (12825) | 7 | 64259175 | 79315069 |
| ENSSSCG00000001950 | 7 | 64578241 | 64763281 | Adipocyte diameter QTL (12837) | 7 | 64259175 | 79315069 |
| ENSSSCG00000001950 | 7 | 64578241 | 64763281 | Adipocyte diameter QTL (12841) | 7 | 64259175 | 79315069 |
| ENSSSCG00000001950 | 7 | 64578241 | 64763281 | Adipocyte diameter QTL (18024) | 7 | 64259175 | 79315069 |
| ENSSSCG00000024517 | 7 | 66915876 | 67462089 | Shoulder subcutaneous fat thickness QTL (5221) | 7 | 2871484 | 1.2E+08 |
| ENSSSCG00000024517 | 7 | 66915876 | 67462089 | Loin muscle area QTL (38092) | 7 | 8459239 | 1.1E+08 |
| ENSSSCG00000024517 | 7 | 66915876 | 67462089 | Meat color a* QTL (38094) | 7 | 8459239 | 69924537 |
| ENSSSCG00000024517 | 7 | 66915876 | 67462089 | Loin fat percentage QTL (38055) | 7 | 17306517 | 1.1E+08 |
| ENSSSCG00000024517 | 7 | 66915876 | 67462089 | Carcass weight (hot) QTL (3) | 7 | 17306517 | 69924537 |
| ENSSSCG00000024517 | 7 | 66915876 | 67462089 | Body weight (end of test) QTL (5236) | 7 | 22506233 | 1.09E+08 |
| ENSSSCG00000024517 | 7 | 66915876 | 67462089 | Carcass length QTL (5700) | 7 | 23437525 | 79315069 |
| ENSSSCG00000024517 | 7 | 66915876 | 67462089 | Backfat at last lumbar QTL (60) | 7 | 25532933 | 1.05E+08 |
| ENSSSCG00000024517 | 7 | 66915876 | 67462089 | backfat above muscle dorsi QTL (5204) | 7 | 34768249 | 73339276 |
| ENSSSCG00000024517 | 7 | 66915876 | 67462089 | Shoulder subcutaneous fat thickness QTL (5220) | 7 | 36553226 | 1.2E+08 |
| ENSSSCG00000024517 | 7 | 66915876 | 67462089 | Backfat at mid-back QTL (5212) | 7 | 36863657 | 68449991 |
| ENSSSCG00000024517 | 7 | 66915876 | 67462089 | Linolenic acid content QTL (156940) | 7 | 37406911 | 1.21E+08 |
| ENSSSCG00000024517 | 7 | 66915876 | 67462089 | Polyunsaturated fatty acid content QTL (156941) | 7 | 37406911 | 1.1E+08 |
| ENSSSCG00000024517 | 7 | 66915876 | 67462089 | Linoleic acid content QTL (156874) | 7 | 37406911 | 1.1E+08 |
| ENSSSCG00000024517 | 7 | 66915876 | 67462089 | Margaric acid content QTL (156872) | 7 | 37406911 | 69924537 |
| ENSSSCG00000024517 | 7 | 66915876 | 67462089 | backfat above muscle dorsi QTL (5239) | 7 | 43770742 | 79237461 |
| ENSSSCG00000024517 | 7 | 66915876 | 67462089 | Average backfat thickness QTL (59) | 7 | 44236389 | 1.05E+08 |
| ENSSSCG00000024517 | 7 | 66915876 | 67462089 | Meat color-L QTL (298) | 7 | 44236389 | 68061952 |
| ENSSSCG00000024517 | 7 | 66915876 | 67462089 | Bicarbonate level QTL (6331) | 7 | 45012466 | 69924537 |
| ENSSSCG00000024517 | 7 | 66915876 | 67462089 | Alkaline phosphatase activity QTL (6346) | 7 | 45012466 | 69924537 |
| ENSSSCG00000024517 | 7 | 66915876 | 67462089 | Alkaline phosphatase activity QTL (6351) | 7 | 45012466 | 69924537 |
| ENSSSCG00000024517 | 7 | 66915876 | 67462089 | Creatinine level QTL (6371) | 7 | 45012466 | 69924537 |
| ENSSSCG00000024517 | 7 | 66915876 | 67462089 | Potassium level QTL (6379) | 7 | 45012466 | 69924537 |
| ENSSSCG00000024517 | 7 | 66915876 | 67462089 | Muscle moisture percentage QTL (38093) | 7 | 45090073 | 1.1E+08 |
| ENSSSCG00000024517 | 7 | 66915876 | 67462089 | Carcass length QTL (29698) | 7 | 45090073 | 81022438 |
| ENSSSCG00000024517 | 7 | 66915876 | 67462089 | Monounsaturated fatty acid content QTL (156875) | 7 | 45090073 | 81022438 |
| ENSSSCG00000024517 | 7 | 66915876 | 67462089 | Oleic acid content QTL (156873) | 7 | 45090073 | 77142053 |
| ENSSSCG00000024517 | 7 | 66915876 | 67462089 | Backfat at last rib QTL (62) | 7 | 46952658 | 1.08E+08 |
| ENSSSCG00000024517 | 7 | 66915876 | 67462089 | lean thickness between 3rd and 4th last ribs QTL (7360) | 7 | 49979358 | 1.27E+08 |
| ENSSSCG00000024517 | 7 | 66915876 | 67462089 | Muscle moisture percentage QTL (2989) | 7 | 56964051 | 69924537 |
| ENSSSCG00000024517 | 7 | 66915876 | 67462089 | Backfat at tenth rib QTL (61) | 7 | 58438598 | 1.05E+08 |
| ENSSSCG00000024517 | 7 | 66915876 | 67462089 | Loin muscle area QTL (3256) | 7 | 58438598 | 79315069 |
| ENSSSCG00000024517 | 7 | 66915876 | 67462089 | Average daily gain QTL (648) | 7 | 58438598 | 77142053 |
| ENSSSCG00000024517 | 7 | 66915876 | 67462089 | Loin muscle area QTL (16927) | 7 | 58438598 | 69846930 |
| ENSSSCG00000024517 | 7 | 66915876 | 67462089 | Marbling QTL (21388) | 7 | 58438598 | 69846930 |
| ENSSSCG00000024517 | 7 | 66915876 | 67462089 | Backfat at last rib QTL (2947) | 7 | 61542906 | 69846930 |
| ENSSSCG00000024517 | 7 | 66915876 | 67462089 | Average backfat thickness QTL (2948) | 7 | 61542906 | 69846930 |
| ENSSSCG00000024517 | 7 | 66915876 | 67462089 | Interferon-gamma level QTL (12265) | 7 | 62707021 | 69924537 |
| ENSSSCG00000024517 | 7 | 66915876 | 67462089 | Meat color b* QTL (3994) | 7 | 63250275 | 81022438 |
| ENSSSCG00000024517 | 7 | 66915876 | 67462089 | Shear force QTL (3996) | 7 | 63250275 | 81022438 |
| ENSSSCG00000024517 | 7 | 66915876 | 67462089 | Total shear work QTL (3998) | 7 | 63250275 | 81022438 |
| ENSSSCG00000024517 | 7 | 66915876 | 67462089 | Drip loss QTL (9853) | 7 | 63250275 | 79315069 |
| ENSSSCG00000024517 | 7 | 66915876 | 67462089 | pH 24 hr post-mortem (loin) QTL (9854) | 7 | 63250275 | 79315069 |
| ENSSSCG00000024517 | 7 | 66915876 | 67462089 | Backfat at tenth rib QTL (5701) | 7 | 63250275 | 79315069 |
| ENSSSCG00000024517 | 7 | 66915876 | 67462089 | Interleukin-2 level QTL (17600) | 7 | 63250275 | 79315069 |
| ENSSSCG00000024517 | 7 | 66915876 | 67462089 | Cholesterol level QTL (17720) | 7 | 63250275 | 79315069 |
| ENSSSCG00000024517 | 7 | 66915876 | 67462089 | LDL cholesterol QTL (17721) | 7 | 63250275 | 79315069 |
| ENSSSCG00000024517 | 7 | 66915876 | 67462089 | Carcass length QTL (649) | 7 | 64259175 | 1.05E+08 |
| ENSSSCG00000024517 | 7 | 66915876 | 67462089 | Belly percentage QTL (650) | 7 | 64259175 | 1.05E+08 |
| ENSSSCG00000024517 | 7 | 66915876 | 67462089 | pH 24 hr post-mortem (loin) QTL (1065) | 7 | 64259175 | 79315069 |
| ENSSSCG00000024517 | 7 | 66915876 | 67462089 | Adipocyte diameter QTL (12822) | 7 | 64259175 | 79315069 |
| ENSSSCG00000024517 | 7 | 66915876 | 67462089 | Adipocyte diameter QTL (12823) | 7 | 64259175 | 79315069 |
| ENSSSCG00000024517 | 7 | 66915876 | 67462089 | Adipocyte diameter QTL (12825) | 7 | 64259175 | 79315069 |
| ENSSSCG00000024517 | 7 | 66915876 | 67462089 | Adipocyte diameter QTL (12837) | 7 | 64259175 | 79315069 |
| ENSSSCG00000024517 | 7 | 66915876 | 67462089 | Adipocyte diameter QTL (12841) | 7 | 64259175 | 79315069 |
| ENSSSCG00000024517 | 7 | 66915876 | 67462089 | Adipocyte diameter QTL (18024) | 7 | 64259175 | 79315069 |
| ENSSSCG00000024517 | 7 | 66915876 | 67462089 | Carcass length QTL (21713) | 7 | 65966545 | 78383777 |
| ENSSSCG00000024517 | 7 | 66915876 | 67462089 | Loin muscle area QTL (21714) | 7 | 65966545 | 78383777 |
| ENSSSCG00000001966 | 7 | 67494941 | 67631431 | Carcass length QTL (5822) | 7 | 67363483 | 67565263 |
| ENSSSCG00000001966 | 7 | 67494941 | 67631431 | Shoulder subcutaneous fat thickness QTL (5221) | 7 | 2871484 | 1.2E+08 |
| ENSSSCG00000001966 | 7 | 67494941 | 67631431 | Loin muscle area QTL (38092) | 7 | 8459239 | 1.1E+08 |
| ENSSSCG00000001966 | 7 | 67494941 | 67631431 | Meat color a* QTL (38094) | 7 | 8459239 | 69924537 |
| ENSSSCG00000001966 | 7 | 67494941 | 67631431 | Loin fat percentage QTL (38055) | 7 | 17306517 | 1.1E+08 |
| ENSSSCG00000001966 | 7 | 67494941 | 67631431 | Carcass weight (hot) QTL (3) | 7 | 17306517 | 69924537 |
| ENSSSCG00000001966 | 7 | 67494941 | 67631431 | Body weight (end of test) QTL (5236) | 7 | 22506233 | 1.09E+08 |
| ENSSSCG00000001966 | 7 | 67494941 | 67631431 | Carcass length QTL (5700) | 7 | 23437525 | 79315069 |
| ENSSSCG00000001966 | 7 | 67494941 | 67631431 | Backfat at last lumbar QTL (60) | 7 | 25532933 | 1.05E+08 |
| ENSSSCG00000001966 | 7 | 67494941 | 67631431 | backfat above muscle dorsi QTL (5204) | 7 | 34768249 | 73339276 |
| ENSSSCG00000001966 | 7 | 67494941 | 67631431 | Shoulder subcutaneous fat thickness QTL (5220) | 7 | 36553226 | 1.2E+08 |
| ENSSSCG00000001966 | 7 | 67494941 | 67631431 | Backfat at mid-back QTL (5212) | 7 | 36863657 | 68449991 |
| ENSSSCG00000001966 | 7 | 67494941 | 67631431 | Linolenic acid content QTL (156940) | 7 | 37406911 | 1.21E+08 |
| ENSSSCG00000001966 | 7 | 67494941 | 67631431 | Polyunsaturated fatty acid content QTL (156941) | 7 | 37406911 | 1.1E+08 |
| ENSSSCG00000001966 | 7 | 67494941 | 67631431 | Linoleic acid content QTL (156874) | 7 | 37406911 | 1.1E+08 |
| ENSSSCG00000001966 | 7 | 67494941 | 67631431 | Margaric acid content QTL (156872) | 7 | 37406911 | 69924537 |
| ENSSSCG00000001966 | 7 | 67494941 | 67631431 | backfat above muscle dorsi QTL (5239) | 7 | 43770742 | 79237461 |
| ENSSSCG00000001966 | 7 | 67494941 | 67631431 | Average backfat thickness QTL (59) | 7 | 44236389 | 1.05E+08 |
| ENSSSCG00000001966 | 7 | 67494941 | 67631431 | Meat color-L QTL (298) | 7 | 44236389 | 68061952 |
| ENSSSCG00000001966 | 7 | 67494941 | 67631431 | Bicarbonate level QTL (6331) | 7 | 45012466 | 69924537 |
| ENSSSCG00000001966 | 7 | 67494941 | 67631431 | Alkaline phosphatase activity QTL (6346) | 7 | 45012466 | 69924537 |
| ENSSSCG00000001966 | 7 | 67494941 | 67631431 | Alkaline phosphatase activity QTL (6351) | 7 | 45012466 | 69924537 |
| ENSSSCG00000001966 | 7 | 67494941 | 67631431 | Creatinine level QTL (6371) | 7 | 45012466 | 69924537 |
| ENSSSCG00000001966 | 7 | 67494941 | 67631431 | Potassium level QTL (6379) | 7 | 45012466 | 69924537 |
| ENSSSCG00000001966 | 7 | 67494941 | 67631431 | Muscle moisture percentage QTL (38093) | 7 | 45090073 | 1.1E+08 |
| ENSSSCG00000001966 | 7 | 67494941 | 67631431 | Carcass length QTL (29698) | 7 | 45090073 | 81022438 |
| ENSSSCG00000001966 | 7 | 67494941 | 67631431 | Monounsaturated fatty acid content QTL (156875) | 7 | 45090073 | 81022438 |
| ENSSSCG00000001966 | 7 | 67494941 | 67631431 | Oleic acid content QTL (156873) | 7 | 45090073 | 77142053 |
| ENSSSCG00000001966 | 7 | 67494941 | 67631431 | Backfat at last rib QTL (62) | 7 | 46952658 | 1.08E+08 |
| ENSSSCG00000001966 | 7 | 67494941 | 67631431 | lean thickness between 3rd and 4th last ribs QTL (7360) | 7 | 49979358 | 1.27E+08 |
| ENSSSCG00000001966 | 7 | 67494941 | 67631431 | Muscle moisture percentage QTL (2989) | 7 | 56964051 | 69924537 |
| ENSSSCG00000001966 | 7 | 67494941 | 67631431 | Backfat at tenth rib QTL (61) | 7 | 58438598 | 1.05E+08 |
| ENSSSCG00000001966 | 7 | 67494941 | 67631431 | Loin muscle area QTL (3256) | 7 | 58438598 | 79315069 |
| ENSSSCG00000001966 | 7 | 67494941 | 67631431 | Average daily gain QTL (648) | 7 | 58438598 | 77142053 |
| ENSSSCG00000001966 | 7 | 67494941 | 67631431 | Loin muscle area QTL (16927) | 7 | 58438598 | 69846930 |
| ENSSSCG00000001966 | 7 | 67494941 | 67631431 | Marbling QTL (21388) | 7 | 58438598 | 69846930 |
| ENSSSCG00000001966 | 7 | 67494941 | 67631431 | Backfat at last rib QTL (2947) | 7 | 61542906 | 69846930 |
| ENSSSCG00000001966 | 7 | 67494941 | 67631431 | Average backfat thickness QTL (2948) | 7 | 61542906 | 69846930 |
| ENSSSCG00000001966 | 7 | 67494941 | 67631431 | Interferon-gamma level QTL (12265) | 7 | 62707021 | 69924537 |
| ENSSSCG00000001966 | 7 | 67494941 | 67631431 | Meat color b* QTL (3994) | 7 | 63250275 | 81022438 |
| ENSSSCG00000001966 | 7 | 67494941 | 67631431 | Shear force QTL (3996) | 7 | 63250275 | 81022438 |
| ENSSSCG00000001966 | 7 | 67494941 | 67631431 | Total shear work QTL (3998) | 7 | 63250275 | 81022438 |
| ENSSSCG00000001966 | 7 | 67494941 | 67631431 | Drip loss QTL (9853) | 7 | 63250275 | 79315069 |
| ENSSSCG00000001966 | 7 | 67494941 | 67631431 | pH 24 hr post-mortem (loin) QTL (9854) | 7 | 63250275 | 79315069 |
| ENSSSCG00000001966 | 7 | 67494941 | 67631431 | Backfat at tenth rib QTL (5701) | 7 | 63250275 | 79315069 |
| ENSSSCG00000001966 | 7 | 67494941 | 67631431 | Interleukin-2 level QTL (17600) | 7 | 63250275 | 79315069 |
| ENSSSCG00000001966 | 7 | 67494941 | 67631431 | Cholesterol level QTL (17720) | 7 | 63250275 | 79315069 |
| ENSSSCG00000001966 | 7 | 67494941 | 67631431 | LDL cholesterol QTL (17721) | 7 | 63250275 | 79315069 |
| ENSSSCG00000001966 | 7 | 67494941 | 67631431 | Carcass length QTL (649) | 7 | 64259175 | 1.05E+08 |
| ENSSSCG00000001966 | 7 | 67494941 | 67631431 | Belly percentage QTL (650) | 7 | 64259175 | 1.05E+08 |
| ENSSSCG00000001966 | 7 | 67494941 | 67631431 | pH 24 hr post-mortem (loin) QTL (1065) | 7 | 64259175 | 79315069 |
| ENSSSCG00000001966 | 7 | 67494941 | 67631431 | Adipocyte diameter QTL (12822) | 7 | 64259175 | 79315069 |
| ENSSSCG00000001966 | 7 | 67494941 | 67631431 | Adipocyte diameter QTL (12823) | 7 | 64259175 | 79315069 |
| ENSSSCG00000001966 | 7 | 67494941 | 67631431 | Adipocyte diameter QTL (12825) | 7 | 64259175 | 79315069 |
| ENSSSCG00000001966 | 7 | 67494941 | 67631431 | Adipocyte diameter QTL (12837) | 7 | 64259175 | 79315069 |
| ENSSSCG00000001966 | 7 | 67494941 | 67631431 | Adipocyte diameter QTL (12841) | 7 | 64259175 | 79315069 |
| ENSSSCG00000001966 | 7 | 67494941 | 67631431 | Adipocyte diameter QTL (18024) | 7 | 64259175 | 79315069 |
| ENSSSCG00000001966 | 7 | 67494941 | 67631431 | Carcass length QTL (21713) | 7 | 65966545 | 78383777 |
| ENSSSCG00000001966 | 7 | 67494941 | 67631431 | Loin muscle area QTL (21714) | 7 | 65966545 | 78383777 |
| ENSSSCG00000002277 | 7 | 88812715 | 88954306 | Longissimus muscle length QTL (703) | 7 | 84980431 | 91033832 |
| ENSSSCG00000002277 | 7 | 88812715 | 88954306 | Intramuscular fat content QTL (3096) | 7 | 84980431 | 91033832 |
| ENSSSCG00000002277 | 7 | 88812715 | 88954306 | Myristic acid content QTL (156939) | 7 | 69924537 | 1.21E+08 |
| ENSSSCG00000002277 | 7 | 88812715 | 88954306 | Lean meat weight QTL (5500) | 7 | 77142053 | 96078332 |
| ENSSSCG00000002277 | 7 | 88812715 | 88954306 | Leaf fat weight QTL (3881) | 7 | 79315069 | 1.19E+08 |
| ENSSSCG00000002277 | 7 | 88812715 | 88954306 | Subcutanous fat area QTL (5988) | 7 | 79315069 | 1.05E+08 |
| ENSSSCG00000002277 | 7 | 88812715 | 88954306 | Diameter of type I muscle fibers QTL (2812) | 7 | 79315069 | 1.05E+08 |
| ENSSSCG00000002277 | 7 | 88812715 | 88954306 | Carcass weight (cold) QTL (63) | 7 | 79315069 | 96078332 |
| ENSSSCG00000002277 | 7 | 88812715 | 88954306 | Number of white fibers QTL (356) | 7 | 79315069 | 96078332 |
| ENSSSCG00000002277 | 7 | 88812715 | 88954306 | Percentage type IIb fibers QTL (357) | 7 | 79315069 | 96078332 |
| ENSSSCG00000002277 | 7 | 88812715 | 88954306 | Diameter of type IIb muscle fibers QTL (358) | 7 | 79315069 | 96078332 |
| ENSSSCG00000002277 | 7 | 88812715 | 88954306 | Average lactate QTL (360) | 7 | 79315069 | 96078332 |
| ENSSSCG00000002277 | 7 | 88812715 | 88954306 | Average glycolytic potential QTL (361) | 7 | 79315069 | 96078332 |
| ENSSSCG00000002277 | 7 | 88812715 | 88954306 | R-value / diameter of white fibers QTL (362) | 7 | 79315069 | 96078332 |
| ENSSSCG00000002277 | 7 | 88812715 | 88954306 | Lactate / diameter of white fibers QTL (363) | 7 | 79315069 | 96078332 |
| ENSSSCG00000002277 | 7 | 88812715 | 88954306 | Average daily gain QTL (3205) | 7 | 79315069 | 91033832 |
| ENSSSCG00000002277 | 7 | 88812715 | 88954306 | Cortisol level QTL (482) | 7 | 81022438 | 1.21E+08 |
| ENSSSCG00000002277 | 7 | 88812715 | 88954306 | Cortisol level QTL (483) | 7 | 81022438 | 1.21E+08 |
| ENSSSCG00000002277 | 7 | 88812715 | 88954306 | Potassium level QTL (15072) | 7 | 81022438 | 1.21E+08 |
| ENSSSCG00000002277 | 7 | 88812715 | 88954306 | Average backfat thickness QTL (3036) | 7 | 82675482 | 93649211 |
| ENSSSCG00000002277 | 7 | 88812715 | 88954306 | Average backfat thickness QTL (2915) | 7 | 82675482 | 93649211 |
| ENSSSCG00000002277 | 7 | 88812715 | 88954306 | Leaf fat weight QTL (727) | 7 | 82675482 | 93649211 |
| ENSSSCG00000002277 | 7 | 88812715 | 88954306 | Loin muscle area QTL (763) | 7 | 82675482 | 93649211 |
| ENSSSCG00000002277 | 7 | 88812715 | 88954306 | Backfat at last lumbar QTL (2946) | 7 | 84980431 | 1.05E+08 |
| ENSSSCG00000002277 | 7 | 88812715 | 88954306 | Average backfat thickness QTL (2949) | 7 | 84980431 | 1.05E+08 |
| ENSSSCG00000002277 | 7 | 88812715 | 88954306 | Shoulder subcutaneous fat thickness QTL (5221) | 7 | 2871484 | 1.2E+08 |
| ENSSSCG00000002277 | 7 | 88812715 | 88954306 | Loin muscle area QTL (38092) | 7 | 8459239 | 1.1E+08 |
| ENSSSCG00000002277 | 7 | 88812715 | 88954306 | Loin fat percentage QTL (38055) | 7 | 17306517 | 1.1E+08 |
| ENSSSCG00000002277 | 7 | 88812715 | 88954306 | Body weight (end of test) QTL (5236) | 7 | 22506233 | 1.09E+08 |
| ENSSSCG00000002277 | 7 | 88812715 | 88954306 | Backfat at last lumbar QTL (60) | 7 | 25532933 | 1.05E+08 |
| ENSSSCG00000002277 | 7 | 88812715 | 88954306 | Shoulder subcutaneous fat thickness QTL (5220) | 7 | 36553226 | 1.2E+08 |
| ENSSSCG00000002277 | 7 | 88812715 | 88954306 | Linolenic acid content QTL (156940) | 7 | 37406911 | 1.21E+08 |
| ENSSSCG00000002277 | 7 | 88812715 | 88954306 | Polyunsaturated fatty acid content QTL (156941) | 7 | 37406911 | 1.1E+08 |
| ENSSSCG00000002277 | 7 | 88812715 | 88954306 | Linoleic acid content QTL (156874) | 7 | 37406911 | 1.1E+08 |
| ENSSSCG00000002277 | 7 | 88812715 | 88954306 | Average backfat thickness QTL (59) | 7 | 44236389 | 1.05E+08 |
| ENSSSCG00000002277 | 7 | 88812715 | 88954306 | Muscle moisture percentage QTL (38093) | 7 | 45090073 | 1.1E+08 |
| ENSSSCG00000002277 | 7 | 88812715 | 88954306 | Backfat at last rib QTL (62) | 7 | 46952658 | 1.08E+08 |
| ENSSSCG00000002277 | 7 | 88812715 | 88954306 | lean thickness between 3rd and 4th last ribs QTL (7360) | 7 | 49979358 | 1.27E+08 |
| ENSSSCG00000002277 | 7 | 88812715 | 88954306 | Backfat at tenth rib QTL (61) | 7 | 58438598 | 1.05E+08 |
| ENSSSCG00000002277 | 7 | 88812715 | 88954306 | Carcass length QTL (649) | 7 | 64259175 | 1.05E+08 |
| ENSSSCG00000002277 | 7 | 88812715 | 88954306 | Belly percentage QTL (650) | 7 | 64259175 | 1.05E+08 |
| ENSSSCG00000002285 | 7 | 90346590 | 91041189 | Backfat between 3rd and 4th last ribs QTL (216295) | 7 | 90664158 | 90664162 |
| ENSSSCG00000002285 | 7 | 90346590 | 91041189 | Backfat at rump QTL (7325) | 7 | 90901899 | 91111439 |
| ENSSSCG00000002285 | 7 | 90346590 | 91041189 | Longissimus muscle length QTL (703) | 7 | 84980431 | 91033832 |
| ENSSSCG00000002285 | 7 | 90346590 | 91041189 | Intramuscular fat content QTL (3096) | 7 | 84980431 | 91033832 |
| ENSSSCG00000002285 | 7 | 90346590 | 91041189 | Myristic acid content QTL (156939) | 7 | 69924537 | 1.21E+08 |
| ENSSSCG00000002285 | 7 | 90346590 | 91041189 | Lean meat weight QTL (5500) | 7 | 77142053 | 96078332 |
| ENSSSCG00000002285 | 7 | 90346590 | 91041189 | Leaf fat weight QTL (3881) | 7 | 79315069 | 1.19E+08 |
| ENSSSCG00000002285 | 7 | 90346590 | 91041189 | Subcutanous fat area QTL (5988) | 7 | 79315069 | 1.05E+08 |
| ENSSSCG00000002285 | 7 | 90346590 | 91041189 | Diameter of type I muscle fibers QTL (2812) | 7 | 79315069 | 1.05E+08 |
| ENSSSCG00000002285 | 7 | 90346590 | 91041189 | Carcass weight (cold) QTL (63) | 7 | 79315069 | 96078332 |
| ENSSSCG00000002285 | 7 | 90346590 | 91041189 | Number of white fibers QTL (356) | 7 | 79315069 | 96078332 |
| ENSSSCG00000002285 | 7 | 90346590 | 91041189 | Percentage type IIb fibers QTL (357) | 7 | 79315069 | 96078332 |
| ENSSSCG00000002285 | 7 | 90346590 | 91041189 | Diameter of type IIb muscle fibers QTL (358) | 7 | 79315069 | 96078332 |
| ENSSSCG00000002285 | 7 | 90346590 | 91041189 | Average lactate QTL (360) | 7 | 79315069 | 96078332 |
| ENSSSCG00000002285 | 7 | 90346590 | 91041189 | Average glycolytic potential QTL (361) | 7 | 79315069 | 96078332 |
| ENSSSCG00000002285 | 7 | 90346590 | 91041189 | R-value / diameter of white fibers QTL (362) | 7 | 79315069 | 96078332 |
| ENSSSCG00000002285 | 7 | 90346590 | 91041189 | Lactate / diameter of white fibers QTL (363) | 7 | 79315069 | 96078332 |
| ENSSSCG00000002285 | 7 | 90346590 | 91041189 | Average daily gain QTL (3205) | 7 | 79315069 | 91033832 |
| ENSSSCG00000002285 | 7 | 90346590 | 91041189 | Cortisol level QTL (482) | 7 | 81022438 | 1.21E+08 |
| ENSSSCG00000002285 | 7 | 90346590 | 91041189 | Cortisol level QTL (483) | 7 | 81022438 | 1.21E+08 |
| ENSSSCG00000002285 | 7 | 90346590 | 91041189 | Potassium level QTL (15072) | 7 | 81022438 | 1.21E+08 |
| ENSSSCG00000002285 | 7 | 90346590 | 91041189 | Average backfat thickness QTL (3036) | 7 | 82675482 | 93649211 |
| ENSSSCG00000002285 | 7 | 90346590 | 91041189 | Average backfat thickness QTL (2915) | 7 | 82675482 | 93649211 |
| ENSSSCG00000002285 | 7 | 90346590 | 91041189 | Leaf fat weight QTL (727) | 7 | 82675482 | 93649211 |
| ENSSSCG00000002285 | 7 | 90346590 | 91041189 | Loin muscle area QTL (763) | 7 | 82675482 | 93649211 |
| ENSSSCG00000002285 | 7 | 90346590 | 91041189 | Backfat at last lumbar QTL (2946) | 7 | 84980431 | 1.05E+08 |
| ENSSSCG00000002285 | 7 | 90346590 | 91041189 | Average backfat thickness QTL (2949) | 7 | 84980431 | 1.05E+08 |
| ENSSSCG00000002285 | 7 | 90346590 | 91041189 | Shoulder subcutaneous fat thickness QTL (5221) | 7 | 2871484 | 1.2E+08 |
| ENSSSCG00000002285 | 7 | 90346590 | 91041189 | Loin muscle area QTL (38092) | 7 | 8459239 | 1.1E+08 |
| ENSSSCG00000002285 | 7 | 90346590 | 91041189 | Loin fat percentage QTL (38055) | 7 | 17306517 | 1.1E+08 |
| ENSSSCG00000002285 | 7 | 90346590 | 91041189 | Body weight (end of test) QTL (5236) | 7 | 22506233 | 1.09E+08 |
| ENSSSCG00000002285 | 7 | 90346590 | 91041189 | Backfat at last lumbar QTL (60) | 7 | 25532933 | 1.05E+08 |
| ENSSSCG00000002285 | 7 | 90346590 | 91041189 | Shoulder subcutaneous fat thickness QTL (5220) | 7 | 36553226 | 1.2E+08 |
| ENSSSCG00000002285 | 7 | 90346590 | 91041189 | Linolenic acid content QTL (156940) | 7 | 37406911 | 1.21E+08 |
| ENSSSCG00000002285 | 7 | 90346590 | 91041189 | Polyunsaturated fatty acid content QTL (156941) | 7 | 37406911 | 1.1E+08 |
| ENSSSCG00000002285 | 7 | 90346590 | 91041189 | Linoleic acid content QTL (156874) | 7 | 37406911 | 1.1E+08 |
| ENSSSCG00000002285 | 7 | 90346590 | 91041189 | Average backfat thickness QTL (59) | 7 | 44236389 | 1.05E+08 |
| ENSSSCG00000002285 | 7 | 90346590 | 91041189 | Muscle moisture percentage QTL (38093) | 7 | 45090073 | 1.1E+08 |
| ENSSSCG00000002285 | 7 | 90346590 | 91041189 | Backfat at last rib QTL (62) | 7 | 46952658 | 1.08E+08 |
| ENSSSCG00000002285 | 7 | 90346590 | 91041189 | lean thickness between 3rd and 4th last ribs QTL (7360) | 7 | 49979358 | 1.27E+08 |
| ENSSSCG00000002285 | 7 | 90346590 | 91041189 | Backfat at tenth rib QTL (61) | 7 | 58438598 | 1.05E+08 |
| ENSSSCG00000002285 | 7 | 90346590 | 91041189 | Carcass length QTL (649) | 7 | 64259175 | 1.05E+08 |
| ENSSSCG00000002285 | 7 | 90346590 | 91041189 | Belly percentage QTL (650) | 7 | 64259175 | 1.05E+08 |
| ENSSSCG00000002426 | 7 | 1.1E+08 | 1.11E+08 | Myristic acid content QTL (156939) | 7 | 69924537 | 1.21E+08 |
| ENSSSCG00000002426 | 7 | 1.1E+08 | 1.11E+08 | Leaf fat weight QTL (3881) | 7 | 79315069 | 1.19E+08 |
| ENSSSCG00000002426 | 7 | 1.1E+08 | 1.11E+08 | Cortisol level QTL (482) | 7 | 81022438 | 1.21E+08 |
| ENSSSCG00000002426 | 7 | 1.1E+08 | 1.11E+08 | Cortisol level QTL (483) | 7 | 81022438 | 1.21E+08 |
| ENSSSCG00000002426 | 7 | 1.1E+08 | 1.11E+08 | Potassium level QTL (15072) | 7 | 81022438 | 1.21E+08 |
| ENSSSCG00000002426 | 7 | 1.1E+08 | 1.11E+08 | Body weight linear QTL (3653) | 7 | 1.05E+08 | 1.21E+08 |
| ENSSSCG00000002426 | 7 | 1.1E+08 | 1.11E+08 | Cortisol level variations QTL (484) | 7 | 1.05E+08 | 1.21E+08 |
| ENSSSCG00000002426 | 7 | 1.1E+08 | 1.11E+08 | Drip loss QTL (7659) | 7 | 1.05E+08 | 1.21E+08 |
| ENSSSCG00000002426 | 7 | 1.1E+08 | 1.11E+08 | Percentage type IIa fibers QTL (7029) | 7 | 1.05E+08 | 1.21E+08 |
| ENSSSCG00000002426 | 7 | 1.1E+08 | 1.11E+08 | Shoulder subcutaneous fat thickness QTL (5221) | 7 | 2871484 | 1.2E+08 |
| ENSSSCG00000002426 | 7 | 1.1E+08 | 1.11E+08 | Shoulder subcutaneous fat thickness QTL (5220) | 7 | 36553226 | 1.2E+08 |
| ENSSSCG00000002426 | 7 | 1.1E+08 | 1.11E+08 | Linolenic acid content QTL (156940) | 7 | 37406911 | 1.21E+08 |
| ENSSSCG00000002426 | 7 | 1.1E+08 | 1.11E+08 | lean thickness between 3rd and 4th last ribs QTL (7360) | 7 | 49979358 | 1.27E+08 |
| ENSSSCG00000028225 | 7 | 1.16E+08 | 1.16E+08 | Myristic acid content QTL (156939) | 7 | 69924537 | 1.21E+08 |
| ENSSSCG00000028225 | 7 | 1.16E+08 | 1.16E+08 | Leaf fat weight QTL (3881) | 7 | 79315069 | 1.19E+08 |
| ENSSSCG00000028225 | 7 | 1.16E+08 | 1.16E+08 | Cortisol level QTL (482) | 7 | 81022438 | 1.21E+08 |
| ENSSSCG00000028225 | 7 | 1.16E+08 | 1.16E+08 | Cortisol level QTL (483) | 7 | 81022438 | 1.21E+08 |
| ENSSSCG00000028225 | 7 | 1.16E+08 | 1.16E+08 | Potassium level QTL (15072) | 7 | 81022438 | 1.21E+08 |
| ENSSSCG00000028225 | 7 | 1.16E+08 | 1.16E+08 | Body weight linear QTL (3653) | 7 | 1.05E+08 | 1.21E+08 |
| ENSSSCG00000028225 | 7 | 1.16E+08 | 1.16E+08 | Cortisol level variations QTL (484) | 7 | 1.05E+08 | 1.21E+08 |
| ENSSSCG00000028225 | 7 | 1.16E+08 | 1.16E+08 | Drip loss QTL (7659) | 7 | 1.05E+08 | 1.21E+08 |
| ENSSSCG00000028225 | 7 | 1.16E+08 | 1.16E+08 | Percentage type IIa fibers QTL (7029) | 7 | 1.05E+08 | 1.21E+08 |
| ENSSSCG00000028225 | 7 | 1.16E+08 | 1.16E+08 | Shoulder subcutaneous fat thickness QTL (5221) | 7 | 2871484 | 1.2E+08 |
| ENSSSCG00000028225 | 7 | 1.16E+08 | 1.16E+08 | Shoulder subcutaneous fat thickness QTL (5220) | 7 | 36553226 | 1.2E+08 |
| ENSSSCG00000028225 | 7 | 1.16E+08 | 1.16E+08 | Linolenic acid content QTL (156940) | 7 | 37406911 | 1.21E+08 |
| ENSSSCG00000028225 | 7 | 1.16E+08 | 1.16E+08 | lean thickness between 3rd and 4th last ribs QTL (7360) | 7 | 49979358 | 1.27E+08 |
| ENSSSCG00000027667 | 7 | 1.21E+08 | 1.21E+08 | Myristic acid content QTL (156939) | 7 | 69924537 | 1.21E+08 |
| ENSSSCG00000027667 | 7 | 1.21E+08 | 1.21E+08 | Cortisol level QTL (482) | 7 | 81022438 | 1.21E+08 |
| ENSSSCG00000027667 | 7 | 1.21E+08 | 1.21E+08 | Cortisol level QTL (483) | 7 | 81022438 | 1.21E+08 |
| ENSSSCG00000027667 | 7 | 1.21E+08 | 1.21E+08 | Potassium level QTL (15072) | 7 | 81022438 | 1.21E+08 |
| ENSSSCG00000027667 | 7 | 1.21E+08 | 1.21E+08 | Body weight linear QTL (3653) | 7 | 1.05E+08 | 1.21E+08 |
| ENSSSCG00000027667 | 7 | 1.21E+08 | 1.21E+08 | Cortisol level variations QTL (484) | 7 | 1.05E+08 | 1.21E+08 |
| ENSSSCG00000027667 | 7 | 1.21E+08 | 1.21E+08 | Drip loss QTL (7659) | 7 | 1.05E+08 | 1.21E+08 |
| ENSSSCG00000027667 | 7 | 1.21E+08 | 1.21E+08 | Percentage type IIa fibers QTL (7029) | 7 | 1.05E+08 | 1.21E+08 |
| ENSSSCG00000027667 | 7 | 1.21E+08 | 1.21E+08 | NADP-malate dehydrogenase activity QTL (18018) | 7 | 1.17E+08 | 1.29E+08 |
| ENSSSCG00000027667 | 7 | 1.21E+08 | 1.21E+08 | Linolenic acid content QTL (156940) | 7 | 37406911 | 1.21E+08 |
| ENSSSCG00000027667 | 7 | 1.21E+08 | 1.21E+08 | lean thickness between 3rd and 4th last ribs QTL (7360) | 7 | 49979358 | 1.27E+08 |
| ENSSSCG00000008761 | 8 | 20254765 | 20370215 | Meat color a* QTL (9082) | 8 | 20077732 | 20469493 |
| ENSSSCG00000008761 | 8 | 20254765 | 20370215 | Loin muscle area QTL (2951) | 8 | 13493977 | 41678979 |
| ENSSSCG00000008761 | 8 | 20254765 | 20370215 | Leaf fat weight QTL (3884) | 8 | 17846873 | 29490870 |
| ENSSSCG00000008761 | 8 | 20254765 | 20370215 | Loin muscle area QTL (5704) | 8 | 2720560 | 1.05E+08 |
| ENSSSCG00000008761 | 8 | 20254765 | 20370215 | Body weight (30 weeks) QTL (21253) | 8 | 3699961 | 90540236 |
| ENSSSCG00000008761 | 8 | 20254765 | 20370215 | Body weight (34 weeks) QTL (21254) | 8 | 10882240 | 87057920 |
| ENSSSCG00000008761 | 8 | 20254765 | 20370215 | Loin muscle area QTL (38095) | 8 | 12079286 | 1.32E+08 |
| ENSSSCG00000008761 | 8 | 20254765 | 20370215 | Backfat at rump QTL (21255) | 8 | 14908668 | 95110777 |
| ENSSSCG00000023112 | 8 | 30988368 | 31134320 | Unsaturated fatty acid content QTL (6433) | 8 | 29044698 | 32657602 |
| ENSSSCG00000023112 | 8 | 30988368 | 31134320 | Loin muscle area QTL (2951) | 8 | 13493977 | 41678979 |
| ENSSSCG00000023112 | 8 | 30988368 | 31134320 | Carcass weight (hot) QTL (16895) | 8 | 22635059 | 61049366 |
| ENSSSCG00000023112 | 8 | 30988368 | 31134320 | Average daily gain QTL (3671) | 8 | 22635059 | 51037705 |
| ENSSSCG00000023112 | 8 | 30988368 | 31134320 | Carcass length QTL (3684) | 8 | 22635059 | 51037705 |
| ENSSSCG00000023112 | 8 | 30988368 | 31134320 | PH for Longissmus dorsi QTL (2992) | 8 | 22635059 | 51037705 |
| ENSSSCG00000023112 | 8 | 30988368 | 31134320 | Average daily gain QTL (4149) | 8 | 22635059 | 51037705 |
| ENSSSCG00000023112 | 8 | 30988368 | 31134320 | Average daily gain QTL (4150) | 8 | 22635059 | 51037705 |
| ENSSSCG00000023112 | 8 | 30988368 | 31134320 | backfat at P2 position QTL (4151) | 8 | 22635059 | 51037705 |
| ENSSSCG00000023112 | 8 | 30988368 | 31134320 | backfat at P2 position QTL (4152) | 8 | 22635059 | 51037705 |
| ENSSSCG00000023112 | 8 | 30988368 | 31134320 | Average backfat thickness QTL (4153) | 8 | 22635059 | 51037705 |
| ENSSSCG00000023112 | 8 | 30988368 | 31134320 | Average daily gain QTL (16853) | 8 | 22635059 | 50384771 |
| ENSSSCG00000023112 | 8 | 30988368 | 31134320 | Carcass weight (hot) QTL (16869) | 8 | 22635059 | 50384771 |
| ENSSSCG00000023112 | 8 | 30988368 | 31134320 | Body weight (slaughter) QTL (1089) | 8 | 22635059 | 50384771 |
| ENSSSCG00000023112 | 8 | 30988368 | 31134320 | Average daily gain QTL (1092) | 8 | 22635059 | 50384771 |
| ENSSSCG00000023112 | 8 | 30988368 | 31134320 | Carcass length QTL (1093) | 8 | 22635059 | 50384771 |
| ENSSSCG00000023112 | 8 | 30988368 | 31134320 | Carcass weight (cold) QTL (1095) | 8 | 22635059 | 50384771 |
| ENSSSCG00000023112 | 8 | 30988368 | 31134320 | Creatine kinase level QTL (1097) | 8 | 22635059 | 50384771 |
| ENSSSCG00000023112 | 8 | 30988368 | 31134320 | Average backfat thickness QTL (5433) | 8 | 22635059 | 41678979 |
| ENSSSCG00000023112 | 8 | 30988368 | 31134320 | Meat color-b QTL (5441) | 8 | 22635059 | 41678979 |
| ENSSSCG00000023112 | 8 | 30988368 | 31134320 | Average daily gain QTL (16889) | 8 | 22635059 | 37326083 |
| ENSSSCG00000023112 | 8 | 30988368 | 31134320 | Leaf fat weight QTL (40) | 8 | 27423244 | 50384771 |
| ENSSSCG00000023112 | 8 | 30988368 | 31134320 | Average daily gain QTL (64) | 8 | 29349401 | 33593474 |
| ENSSSCG00000023112 | 8 | 30988368 | 31134320 | Carcass weight (cold) QTL (65) | 8 | 29349401 | 33593474 |
| ENSSSCG00000023112 | 8 | 30988368 | 31134320 | Average daily gain QTL (334) | 8 | 29382048 | 65728729 |
| ENSSSCG00000023112 | 8 | 30988368 | 31134320 | Loin muscle area QTL (5704) | 8 | 2720560 | 1.05E+08 |
| ENSSSCG00000023112 | 8 | 30988368 | 31134320 | Body weight (30 weeks) QTL (21253) | 8 | 3699961 | 90540236 |
| ENSSSCG00000023112 | 8 | 30988368 | 31134320 | Body weight (34 weeks) QTL (21254) | 8 | 10882240 | 87057920 |
| ENSSSCG00000023112 | 8 | 30988368 | 31134320 | Loin muscle area QTL (38095) | 8 | 12079286 | 1.32E+08 |
| ENSSSCG00000023112 | 8 | 30988368 | 31134320 | Backfat at rump QTL (21255) | 8 | 14908668 | 95110777 |
| ENSSSCG00000023112 | 8 | 30988368 | 31134320 | Abdominal fat weight QTL (21252) | 8 | 24158572 | 1.19E+08 |
| ENSSSCG00000023112 | 8 | 30988368 | 31134320 | Lauric acid content QTL (156942) | 8 | 27423244 | 1.32E+08 |
| ENSSSCG00000023112 | 8 | 30988368 | 31134320 | Alkaline phosphatase activity QTL (15105) | 8 | 27423244 | 1.05E+08 |
| ENSSSCG00000023112 | 8 | 30988368 | 31134320 | Body weight (3 weeks) QTL (29700) | 8 | 27423244 | 90104947 |
| ENSSSCG00000023112 | 8 | 30988368 | 31134320 | Body weight (20 weeks) QTL (29701) | 8 | 27423244 | 67687532 |
| ENSSSCG00000023112 | 8 | 30988368 | 31134320 | Carcass weight (hot) QTL (29702) | 8 | 27423244 | 67687532 |
| ENSSSCG00000023112 | 8 | 30988368 | 31134320 | Leaf fat weight QTL (21256) | 8 | 29055580 | 69102224 |
| ENSSSCG00000008803 | 8 | 33387590 | 33638841 | Loin muscle area QTL (2951) | 8 | 13493977 | 41678979 |
| ENSSSCG00000008803 | 8 | 33387590 | 33638841 | Carcass weight (hot) QTL (16895) | 8 | 22635059 | 61049366 |
| ENSSSCG00000008803 | 8 | 33387590 | 33638841 | Average daily gain QTL (3671) | 8 | 22635059 | 51037705 |
| ENSSSCG00000008803 | 8 | 33387590 | 33638841 | Carcass length QTL (3684) | 8 | 22635059 | 51037705 |
| ENSSSCG00000008803 | 8 | 33387590 | 33638841 | PH for Longissmus dorsi QTL (2992) | 8 | 22635059 | 51037705 |
| ENSSSCG00000008803 | 8 | 33387590 | 33638841 | Average daily gain QTL (4149) | 8 | 22635059 | 51037705 |
| ENSSSCG00000008803 | 8 | 33387590 | 33638841 | Average daily gain QTL (4150) | 8 | 22635059 | 51037705 |
| ENSSSCG00000008803 | 8 | 33387590 | 33638841 | backfat at P2 position QTL (4151) | 8 | 22635059 | 51037705 |
| ENSSSCG00000008803 | 8 | 33387590 | 33638841 | backfat at P2 position QTL (4152) | 8 | 22635059 | 51037705 |
| ENSSSCG00000008803 | 8 | 33387590 | 33638841 | Average backfat thickness QTL (4153) | 8 | 22635059 | 51037705 |
| ENSSSCG00000008803 | 8 | 33387590 | 33638841 | Average daily gain QTL (16853) | 8 | 22635059 | 50384771 |
| ENSSSCG00000008803 | 8 | 33387590 | 33638841 | Carcass weight (hot) QTL (16869) | 8 | 22635059 | 50384771 |
| ENSSSCG00000008803 | 8 | 33387590 | 33638841 | Body weight (slaughter) QTL (1089) | 8 | 22635059 | 50384771 |
| ENSSSCG00000008803 | 8 | 33387590 | 33638841 | Average daily gain QTL (1092) | 8 | 22635059 | 50384771 |
| ENSSSCG00000008803 | 8 | 33387590 | 33638841 | Carcass length QTL (1093) | 8 | 22635059 | 50384771 |
| ENSSSCG00000008803 | 8 | 33387590 | 33638841 | Carcass weight (cold) QTL (1095) | 8 | 22635059 | 50384771 |
| ENSSSCG00000008803 | 8 | 33387590 | 33638841 | Creatine kinase level QTL (1097) | 8 | 22635059 | 50384771 |
| ENSSSCG00000008803 | 8 | 33387590 | 33638841 | Average backfat thickness QTL (5433) | 8 | 22635059 | 41678979 |
| ENSSSCG00000008803 | 8 | 33387590 | 33638841 | Meat color-b QTL (5441) | 8 | 22635059 | 41678979 |
| ENSSSCG00000008803 | 8 | 33387590 | 33638841 | Average daily gain QTL (16889) | 8 | 22635059 | 37326083 |
| ENSSSCG00000008803 | 8 | 33387590 | 33638841 | Leaf fat weight QTL (40) | 8 | 27423244 | 50384771 |
| ENSSSCG00000008803 | 8 | 33387590 | 33638841 | Average daily gain QTL (64) | 8 | 29349401 | 33593474 |
| ENSSSCG00000008803 | 8 | 33387590 | 33638841 | Carcass weight (cold) QTL (65) | 8 | 29349401 | 33593474 |
| ENSSSCG00000008803 | 8 | 33387590 | 33638841 | Average daily gain QTL (334) | 8 | 29382048 | 65728729 |
| ENSSSCG00000008803 | 8 | 33387590 | 33638841 | Loin muscle area QTL (5704) | 8 | 2720560 | 1.05E+08 |
| ENSSSCG00000008803 | 8 | 33387590 | 33638841 | Body weight (30 weeks) QTL (21253) | 8 | 3699961 | 90540236 |
| ENSSSCG00000008803 | 8 | 33387590 | 33638841 | Body weight (34 weeks) QTL (21254) | 8 | 10882240 | 87057920 |
| ENSSSCG00000008803 | 8 | 33387590 | 33638841 | Loin muscle area QTL (38095) | 8 | 12079286 | 1.32E+08 |
| ENSSSCG00000008803 | 8 | 33387590 | 33638841 | Backfat at rump QTL (21255) | 8 | 14908668 | 95110777 |
| ENSSSCG00000008803 | 8 | 33387590 | 33638841 | Abdominal fat weight QTL (21252) | 8 | 24158572 | 1.19E+08 |
| ENSSSCG00000008803 | 8 | 33387590 | 33638841 | Lauric acid content QTL (156942) | 8 | 27423244 | 1.32E+08 |
| ENSSSCG00000008803 | 8 | 33387590 | 33638841 | Alkaline phosphatase activity QTL (15105) | 8 | 27423244 | 1.05E+08 |
| ENSSSCG00000008803 | 8 | 33387590 | 33638841 | Body weight (3 weeks) QTL (29700) | 8 | 27423244 | 90104947 |
| ENSSSCG00000008803 | 8 | 33387590 | 33638841 | Body weight (20 weeks) QTL (29701) | 8 | 27423244 | 67687532 |
| ENSSSCG00000008803 | 8 | 33387590 | 33638841 | Carcass weight (hot) QTL (29702) | 8 | 27423244 | 67687532 |
| ENSSSCG00000008803 | 8 | 33387590 | 33638841 | Leaf fat weight QTL (21256) | 8 | 29055580 | 69102224 |
| ENSSSCG00000008821 | 8 | 38241598 | 38322031 | Loin muscle area QTL (2951) | 8 | 13493977 | 41678979 |
| ENSSSCG00000008821 | 8 | 38241598 | 38322031 | Carcass weight (hot) QTL (16895) | 8 | 22635059 | 61049366 |
| ENSSSCG00000008821 | 8 | 38241598 | 38322031 | Average daily gain QTL (3671) | 8 | 22635059 | 51037705 |
| ENSSSCG00000008821 | 8 | 38241598 | 38322031 | Carcass length QTL (3684) | 8 | 22635059 | 51037705 |
| ENSSSCG00000008821 | 8 | 38241598 | 38322031 | PH for Longissmus dorsi QTL (2992) | 8 | 22635059 | 51037705 |
| ENSSSCG00000008821 | 8 | 38241598 | 38322031 | Average daily gain QTL (4149) | 8 | 22635059 | 51037705 |
| ENSSSCG00000008821 | 8 | 38241598 | 38322031 | Average daily gain QTL (4150) | 8 | 22635059 | 51037705 |
| ENSSSCG00000008821 | 8 | 38241598 | 38322031 | backfat at P2 position QTL (4151) | 8 | 22635059 | 51037705 |
| ENSSSCG00000008821 | 8 | 38241598 | 38322031 | backfat at P2 position QTL (4152) | 8 | 22635059 | 51037705 |
| ENSSSCG00000008821 | 8 | 38241598 | 38322031 | Average backfat thickness QTL (4153) | 8 | 22635059 | 51037705 |
| ENSSSCG00000008821 | 8 | 38241598 | 38322031 | Average daily gain QTL (16853) | 8 | 22635059 | 50384771 |
| ENSSSCG00000008821 | 8 | 38241598 | 38322031 | Carcass weight (hot) QTL (16869) | 8 | 22635059 | 50384771 |
| ENSSSCG00000008821 | 8 | 38241598 | 38322031 | Body weight (slaughter) QTL (1089) | 8 | 22635059 | 50384771 |
| ENSSSCG00000008821 | 8 | 38241598 | 38322031 | Average daily gain QTL (1092) | 8 | 22635059 | 50384771 |
| ENSSSCG00000008821 | 8 | 38241598 | 38322031 | Carcass length QTL (1093) | 8 | 22635059 | 50384771 |
| ENSSSCG00000008821 | 8 | 38241598 | 38322031 | Carcass weight (cold) QTL (1095) | 8 | 22635059 | 50384771 |
| ENSSSCG00000008821 | 8 | 38241598 | 38322031 | Creatine kinase level QTL (1097) | 8 | 22635059 | 50384771 |
| ENSSSCG00000008821 | 8 | 38241598 | 38322031 | Average backfat thickness QTL (5433) | 8 | 22635059 | 41678979 |
| ENSSSCG00000008821 | 8 | 38241598 | 38322031 | Meat color-b QTL (5441) | 8 | 22635059 | 41678979 |
| ENSSSCG00000008821 | 8 | 38241598 | 38322031 | Leaf fat weight QTL (40) | 8 | 27423244 | 50384771 |
| ENSSSCG00000008821 | 8 | 38241598 | 38322031 | Average daily gain QTL (334) | 8 | 29382048 | 65728729 |
| ENSSSCG00000008821 | 8 | 38241598 | 38322031 | Loin muscle area QTL (5704) | 8 | 2720560 | 1.05E+08 |
| ENSSSCG00000008821 | 8 | 38241598 | 38322031 | Body weight (30 weeks) QTL (21253) | 8 | 3699961 | 90540236 |
| ENSSSCG00000008821 | 8 | 38241598 | 38322031 | Body weight (34 weeks) QTL (21254) | 8 | 10882240 | 87057920 |
| ENSSSCG00000008821 | 8 | 38241598 | 38322031 | Loin muscle area QTL (38095) | 8 | 12079286 | 1.32E+08 |
| ENSSSCG00000008821 | 8 | 38241598 | 38322031 | Backfat at rump QTL (21255) | 8 | 14908668 | 95110777 |
| ENSSSCG00000008821 | 8 | 38241598 | 38322031 | Abdominal fat weight QTL (21252) | 8 | 24158572 | 1.19E+08 |
| ENSSSCG00000008821 | 8 | 38241598 | 38322031 | Lauric acid content QTL (156942) | 8 | 27423244 | 1.32E+08 |
| ENSSSCG00000008821 | 8 | 38241598 | 38322031 | Alkaline phosphatase activity QTL (15105) | 8 | 27423244 | 1.05E+08 |
| ENSSSCG00000008821 | 8 | 38241598 | 38322031 | Body weight (3 weeks) QTL (29700) | 8 | 27423244 | 90104947 |
| ENSSSCG00000008821 | 8 | 38241598 | 38322031 | Body weight (20 weeks) QTL (29701) | 8 | 27423244 | 67687532 |
| ENSSSCG00000008821 | 8 | 38241598 | 38322031 | Carcass weight (hot) QTL (29702) | 8 | 27423244 | 67687532 |
| ENSSSCG00000008821 | 8 | 38241598 | 38322031 | Leaf fat weight QTL (21256) | 8 | 29055580 | 69102224 |
| ENSSSCG00000008826 | 8 | 38397375 | 38697206 | Granulocyte percentage QTL (22149) | 8 | 38677771 | 38677775 |
| ENSSSCG00000008826 | 8 | 38397375 | 38697206 | Loin muscle area QTL (2951) | 8 | 13493977 | 41678979 |
| ENSSSCG00000008826 | 8 | 38397375 | 38697206 | Carcass weight (hot) QTL (16895) | 8 | 22635059 | 61049366 |
| ENSSSCG00000008826 | 8 | 38397375 | 38697206 | Average daily gain QTL (3671) | 8 | 22635059 | 51037705 |
| ENSSSCG00000008826 | 8 | 38397375 | 38697206 | Carcass length QTL (3684) | 8 | 22635059 | 51037705 |
| ENSSSCG00000008826 | 8 | 38397375 | 38697206 | PH for Longissmus dorsi QTL (2992) | 8 | 22635059 | 51037705 |
| ENSSSCG00000008826 | 8 | 38397375 | 38697206 | Average daily gain QTL (4149) | 8 | 22635059 | 51037705 |
| ENSSSCG00000008826 | 8 | 38397375 | 38697206 | Average daily gain QTL (4150) | 8 | 22635059 | 51037705 |
| ENSSSCG00000008826 | 8 | 38397375 | 38697206 | backfat at P2 position QTL (4151) | 8 | 22635059 | 51037705 |
| ENSSSCG00000008826 | 8 | 38397375 | 38697206 | backfat at P2 position QTL (4152) | 8 | 22635059 | 51037705 |
| ENSSSCG00000008826 | 8 | 38397375 | 38697206 | Average backfat thickness QTL (4153) | 8 | 22635059 | 51037705 |
| ENSSSCG00000008826 | 8 | 38397375 | 38697206 | Average daily gain QTL (16853) | 8 | 22635059 | 50384771 |
| ENSSSCG00000008826 | 8 | 38397375 | 38697206 | Carcass weight (hot) QTL (16869) | 8 | 22635059 | 50384771 |
| ENSSSCG00000008826 | 8 | 38397375 | 38697206 | Body weight (slaughter) QTL (1089) | 8 | 22635059 | 50384771 |
| ENSSSCG00000008826 | 8 | 38397375 | 38697206 | Average daily gain QTL (1092) | 8 | 22635059 | 50384771 |
| ENSSSCG00000008826 | 8 | 38397375 | 38697206 | Carcass length QTL (1093) | 8 | 22635059 | 50384771 |
| ENSSSCG00000008826 | 8 | 38397375 | 38697206 | Carcass weight (cold) QTL (1095) | 8 | 22635059 | 50384771 |
| ENSSSCG00000008826 | 8 | 38397375 | 38697206 | Creatine kinase level QTL (1097) | 8 | 22635059 | 50384771 |
| ENSSSCG00000008826 | 8 | 38397375 | 38697206 | Average backfat thickness QTL (5433) | 8 | 22635059 | 41678979 |
| ENSSSCG00000008826 | 8 | 38397375 | 38697206 | Meat color-b QTL (5441) | 8 | 22635059 | 41678979 |
| ENSSSCG00000008826 | 8 | 38397375 | 38697206 | Leaf fat weight QTL (40) | 8 | 27423244 | 50384771 |
| ENSSSCG00000008826 | 8 | 38397375 | 38697206 | Average daily gain QTL (334) | 8 | 29382048 | 65728729 |
| ENSSSCG00000008826 | 8 | 38397375 | 38697206 | Loin muscle area QTL (5704) | 8 | 2720560 | 1.05E+08 |
| ENSSSCG00000008826 | 8 | 38397375 | 38697206 | Body weight (30 weeks) QTL (21253) | 8 | 3699961 | 90540236 |
| ENSSSCG00000008826 | 8 | 38397375 | 38697206 | Body weight (34 weeks) QTL (21254) | 8 | 10882240 | 87057920 |
| ENSSSCG00000008826 | 8 | 38397375 | 38697206 | Loin muscle area QTL (38095) | 8 | 12079286 | 1.32E+08 |
| ENSSSCG00000008826 | 8 | 38397375 | 38697206 | Backfat at rump QTL (21255) | 8 | 14908668 | 95110777 |
| ENSSSCG00000008826 | 8 | 38397375 | 38697206 | Abdominal fat weight QTL (21252) | 8 | 24158572 | 1.19E+08 |
| ENSSSCG00000008826 | 8 | 38397375 | 38697206 | Lauric acid content QTL (156942) | 8 | 27423244 | 1.32E+08 |
| ENSSSCG00000008826 | 8 | 38397375 | 38697206 | Alkaline phosphatase activity QTL (15105) | 8 | 27423244 | 1.05E+08 |
| ENSSSCG00000008826 | 8 | 38397375 | 38697206 | Body weight (3 weeks) QTL (29700) | 8 | 27423244 | 90104947 |
| ENSSSCG00000008826 | 8 | 38397375 | 38697206 | Body weight (20 weeks) QTL (29701) | 8 | 27423244 | 67687532 |
| ENSSSCG00000008826 | 8 | 38397375 | 38697206 | Carcass weight (hot) QTL (29702) | 8 | 27423244 | 67687532 |
| ENSSSCG00000008826 | 8 | 38397375 | 38697206 | Leaf fat weight QTL (21256) | 8 | 29055580 | 69102224 |
| ENSSSCG00000008943 | 8 | 67706880 | 68290496 | Gadoleic acid content QTL (156882) | 8 | 67687532 | 1.17E+08 |
| ENSSSCG00000008943 | 8 | 67706880 | 68290496 | Estimated carcass lean content QTL (5707) | 8 | 67687532 | 1.05E+08 |
| ENSSSCG00000008943 | 8 | 67706880 | 68290496 | Toll-like receptor 9 level QTL (17620) | 8 | 67687532 | 1.05E+08 |
| ENSSSCG00000008943 | 8 | 67706880 | 68290496 | Loin muscle area QTL (5704) | 8 | 2720560 | 1.05E+08 |
| ENSSSCG00000008943 | 8 | 67706880 | 68290496 | Body weight (30 weeks) QTL (21253) | 8 | 3699961 | 90540236 |
| ENSSSCG00000008943 | 8 | 67706880 | 68290496 | Body weight (34 weeks) QTL (21254) | 8 | 10882240 | 87057920 |
| ENSSSCG00000008943 | 8 | 67706880 | 68290496 | Loin muscle area QTL (38095) | 8 | 12079286 | 1.32E+08 |
| ENSSSCG00000008943 | 8 | 67706880 | 68290496 | Backfat at rump QTL (21255) | 8 | 14908668 | 95110777 |
| ENSSSCG00000008943 | 8 | 67706880 | 68290496 | Abdominal fat weight QTL (21252) | 8 | 24158572 | 1.19E+08 |
| ENSSSCG00000008943 | 8 | 67706880 | 68290496 | Lauric acid content QTL (156942) | 8 | 27423244 | 1.32E+08 |
| ENSSSCG00000008943 | 8 | 67706880 | 68290496 | Alkaline phosphatase activity QTL (15105) | 8 | 27423244 | 1.05E+08 |
| ENSSSCG00000008943 | 8 | 67706880 | 68290496 | Body weight (3 weeks) QTL (29700) | 8 | 27423244 | 90104947 |
| ENSSSCG00000008943 | 8 | 67706880 | 68290496 | Leaf fat weight QTL (21256) | 8 | 29055580 | 69102224 |
| ENSSSCG00000008943 | 8 | 67706880 | 68290496 | Glucose level QTL (15106) | 8 | 57131760 | 1.05E+08 |
| ENSSSCG00000008943 | 8 | 67706880 | 68290496 | Stearic acid content QTL (156879) | 8 | 57131760 | 1.05E+08 |
| ENSSSCG00000008943 | 8 | 67706880 | 68290496 | Uterine capacity QTL (523) | 8 | 57675872 | 1.16E+08 |
| ENSSSCG00000008943 | 8 | 67706880 | 68290496 | Bilirubin level QTL (6374) | 8 | 63334636 | 87166742 |
| ENSSSCG00000008943 | 8 | 67706880 | 68290496 | Average daily gain QTL (498) | 8 | 65293440 | 70734560 |
| ENSSSCG00000008943 | 8 | 67706880 | 68290496 | Palmitic acid content QTL (469) | 8 | 65728729 | 1.22E+08 |
| ENSSSCG00000008943 | 8 | 67706880 | 68290496 | Palmitoleic acid content QTL (470) | 8 | 65728729 | 1.22E+08 |
| ENSSSCG00000008943 | 8 | 67706880 | 68290496 | Average chain length QTL (471) | 8 | 65728729 | 1.22E+08 |
| ENSSSCG00000008943 | 8 | 67706880 | 68290496 | Temperature 40 minutes post mortem (loin) QTL (3999) | 8 | 65728729 | 1.14E+08 |
| ENSSSCG00000008943 | 8 | 67706880 | 68290496 | Loin muscle area QTL (3264) | 8 | 65728729 | 87166742 |
| ENSSSCG00000008943 | 8 | 67706880 | 68290496 | Empty body lipid content QTL (16930) | 8 | 65728729 | 87166742 |
| ENSSSCG00000024592 | 8 | 69360649 | 69531286 | Cooking loss QTL (168344) | 8 | 69424527 | 69424531 |
| ENSSSCG00000024592 | 8 | 69360649 | 69531286 | Gadoleic acid content QTL (156882) | 8 | 67687532 | 1.17E+08 |
| ENSSSCG00000024592 | 8 | 69360649 | 69531286 | Estimated carcass lean content QTL (5707) | 8 | 67687532 | 1.05E+08 |
| ENSSSCG00000024592 | 8 | 69360649 | 69531286 | Toll-like receptor 9 level QTL (17620) | 8 | 67687532 | 1.05E+08 |
| ENSSSCG00000024592 | 8 | 69360649 | 69531286 | Loin muscle area QTL (5704) | 8 | 2720560 | 1.05E+08 |
| ENSSSCG00000024592 | 8 | 69360649 | 69531286 | Body weight (30 weeks) QTL (21253) | 8 | 3699961 | 90540236 |
| ENSSSCG00000024592 | 8 | 69360649 | 69531286 | Body weight (34 weeks) QTL (21254) | 8 | 10882240 | 87057920 |
| ENSSSCG00000024592 | 8 | 69360649 | 69531286 | Loin muscle area QTL (38095) | 8 | 12079286 | 1.32E+08 |
| ENSSSCG00000024592 | 8 | 69360649 | 69531286 | Backfat at rump QTL (21255) | 8 | 14908668 | 95110777 |
| ENSSSCG00000024592 | 8 | 69360649 | 69531286 | Abdominal fat weight QTL (21252) | 8 | 24158572 | 1.19E+08 |
| ENSSSCG00000024592 | 8 | 69360649 | 69531286 | Lauric acid content QTL (156942) | 8 | 27423244 | 1.32E+08 |
| ENSSSCG00000024592 | 8 | 69360649 | 69531286 | Alkaline phosphatase activity QTL (15105) | 8 | 27423244 | 1.05E+08 |
| ENSSSCG00000024592 | 8 | 69360649 | 69531286 | Body weight (3 weeks) QTL (29700) | 8 | 27423244 | 90104947 |
| ENSSSCG00000024592 | 8 | 69360649 | 69531286 | Glucose level QTL (15106) | 8 | 57131760 | 1.05E+08 |
| ENSSSCG00000024592 | 8 | 69360649 | 69531286 | Stearic acid content QTL (156879) | 8 | 57131760 | 1.05E+08 |
| ENSSSCG00000024592 | 8 | 69360649 | 69531286 | Uterine capacity QTL (523) | 8 | 57675872 | 1.16E+08 |
| ENSSSCG00000024592 | 8 | 69360649 | 69531286 | Bilirubin level QTL (6374) | 8 | 63334636 | 87166742 |
| ENSSSCG00000024592 | 8 | 69360649 | 69531286 | Average daily gain QTL (498) | 8 | 65293440 | 70734560 |
| ENSSSCG00000024592 | 8 | 69360649 | 69531286 | Palmitic acid content QTL (469) | 8 | 65728729 | 1.22E+08 |
| ENSSSCG00000024592 | 8 | 69360649 | 69531286 | Palmitoleic acid content QTL (470) | 8 | 65728729 | 1.22E+08 |
| ENSSSCG00000024592 | 8 | 69360649 | 69531286 | Average chain length QTL (471) | 8 | 65728729 | 1.22E+08 |
| ENSSSCG00000024592 | 8 | 69360649 | 69531286 | Temperature 40 minutes post mortem (loin) QTL (3999) | 8 | 65728729 | 1.14E+08 |
| ENSSSCG00000024592 | 8 | 69360649 | 69531286 | Loin muscle area QTL (3264) | 8 | 65728729 | 87166742 |
| ENSSSCG00000024592 | 8 | 69360649 | 69531286 | Empty body lipid content QTL (16930) | 8 | 65728729 | 87166742 |
| ENSSSCG00000008974 | 8 | 71613174 | 71646779 | Gadoleic acid content QTL (156882) | 8 | 67687532 | 1.17E+08 |
| ENSSSCG00000008974 | 8 | 71613174 | 71646779 | Estimated carcass lean content QTL (5707) | 8 | 67687532 | 1.05E+08 |
| ENSSSCG00000008974 | 8 | 71613174 | 71646779 | Toll-like receptor 9 level QTL (17620) | 8 | 67687532 | 1.05E+08 |
| ENSSSCG00000008974 | 8 | 71613174 | 71646779 | Loin muscle area QTL (5704) | 8 | 2720560 | 1.05E+08 |
| ENSSSCG00000008974 | 8 | 71613174 | 71646779 | Body weight (30 weeks) QTL (21253) | 8 | 3699961 | 90540236 |
| ENSSSCG00000008974 | 8 | 71613174 | 71646779 | Body weight (34 weeks) QTL (21254) | 8 | 10882240 | 87057920 |
| ENSSSCG00000008974 | 8 | 71613174 | 71646779 | Loin muscle area QTL (38095) | 8 | 12079286 | 1.32E+08 |
| ENSSSCG00000008974 | 8 | 71613174 | 71646779 | Backfat at rump QTL (21255) | 8 | 14908668 | 95110777 |
| ENSSSCG00000008974 | 8 | 71613174 | 71646779 | Abdominal fat weight QTL (21252) | 8 | 24158572 | 1.19E+08 |
| ENSSSCG00000008974 | 8 | 71613174 | 71646779 | Lauric acid content QTL (156942) | 8 | 27423244 | 1.32E+08 |
| ENSSSCG00000008974 | 8 | 71613174 | 71646779 | Alkaline phosphatase activity QTL (15105) | 8 | 27423244 | 1.05E+08 |
| ENSSSCG00000008974 | 8 | 71613174 | 71646779 | Body weight (3 weeks) QTL (29700) | 8 | 27423244 | 90104947 |
| ENSSSCG00000008974 | 8 | 71613174 | 71646779 | Glucose level QTL (15106) | 8 | 57131760 | 1.05E+08 |
| ENSSSCG00000008974 | 8 | 71613174 | 71646779 | Stearic acid content QTL (156879) | 8 | 57131760 | 1.05E+08 |
| ENSSSCG00000008974 | 8 | 71613174 | 71646779 | Uterine capacity QTL (523) | 8 | 57675872 | 1.16E+08 |
| ENSSSCG00000008974 | 8 | 71613174 | 71646779 | Bilirubin level QTL (6374) | 8 | 63334636 | 87166742 |
| ENSSSCG00000008974 | 8 | 71613174 | 71646779 | Palmitic acid content QTL (469) | 8 | 65728729 | 1.22E+08 |
| ENSSSCG00000008974 | 8 | 71613174 | 71646779 | Palmitoleic acid content QTL (470) | 8 | 65728729 | 1.22E+08 |
| ENSSSCG00000008974 | 8 | 71613174 | 71646779 | Average chain length QTL (471) | 8 | 65728729 | 1.22E+08 |
| ENSSSCG00000008974 | 8 | 71613174 | 71646779 | Temperature 40 minutes post mortem (loin) QTL (3999) | 8 | 65728729 | 1.14E+08 |
| ENSSSCG00000008974 | 8 | 71613174 | 71646779 | Loin muscle area QTL (3264) | 8 | 65728729 | 87166742 |
| ENSSSCG00000008974 | 8 | 71613174 | 71646779 | Empty body lipid content QTL (16930) | 8 | 65728729 | 87166742 |
| ENSSSCG00000009007 | 8 | 75466134 | 75645030 | Gadoleic acid content QTL (156882) | 8 | 67687532 | 1.17E+08 |
| ENSSSCG00000009007 | 8 | 75466134 | 75645030 | Estimated carcass lean content QTL (5707) | 8 | 67687532 | 1.05E+08 |
| ENSSSCG00000009007 | 8 | 75466134 | 75645030 | Toll-like receptor 9 level QTL (17620) | 8 | 67687532 | 1.05E+08 |
| ENSSSCG00000009007 | 8 | 75466134 | 75645030 | Coat color QTL (306) | 8 | 75457452 | 75783919 |
| ENSSSCG00000009007 | 8 | 75466134 | 75645030 | Loin muscle area QTL (5704) | 8 | 2720560 | 1.05E+08 |
| ENSSSCG00000009007 | 8 | 75466134 | 75645030 | Body weight (30 weeks) QTL (21253) | 8 | 3699961 | 90540236 |
| ENSSSCG00000009007 | 8 | 75466134 | 75645030 | Body weight (34 weeks) QTL (21254) | 8 | 10882240 | 87057920 |
| ENSSSCG00000009007 | 8 | 75466134 | 75645030 | Loin muscle area QTL (38095) | 8 | 12079286 | 1.32E+08 |
| ENSSSCG00000009007 | 8 | 75466134 | 75645030 | Backfat at rump QTL (21255) | 8 | 14908668 | 95110777 |
| ENSSSCG00000009007 | 8 | 75466134 | 75645030 | Abdominal fat weight QTL (21252) | 8 | 24158572 | 1.19E+08 |
| ENSSSCG00000009007 | 8 | 75466134 | 75645030 | Lauric acid content QTL (156942) | 8 | 27423244 | 1.32E+08 |
| ENSSSCG00000009007 | 8 | 75466134 | 75645030 | Alkaline phosphatase activity QTL (15105) | 8 | 27423244 | 1.05E+08 |
| ENSSSCG00000009007 | 8 | 75466134 | 75645030 | Body weight (3 weeks) QTL (29700) | 8 | 27423244 | 90104947 |
| ENSSSCG00000009007 | 8 | 75466134 | 75645030 | Glucose level QTL (15106) | 8 | 57131760 | 1.05E+08 |
| ENSSSCG00000009007 | 8 | 75466134 | 75645030 | Stearic acid content QTL (156879) | 8 | 57131760 | 1.05E+08 |
| ENSSSCG00000009007 | 8 | 75466134 | 75645030 | Uterine capacity QTL (523) | 8 | 57675872 | 1.16E+08 |
| ENSSSCG00000009007 | 8 | 75466134 | 75645030 | Bilirubin level QTL (6374) | 8 | 63334636 | 87166742 |
| ENSSSCG00000009007 | 8 | 75466134 | 75645030 | Palmitic acid content QTL (469) | 8 | 65728729 | 1.22E+08 |
| ENSSSCG00000009007 | 8 | 75466134 | 75645030 | Palmitoleic acid content QTL (470) | 8 | 65728729 | 1.22E+08 |
| ENSSSCG00000009007 | 8 | 75466134 | 75645030 | Average chain length QTL (471) | 8 | 65728729 | 1.22E+08 |
| ENSSSCG00000009007 | 8 | 75466134 | 75645030 | Temperature 40 minutes post mortem (loin) QTL (3999) | 8 | 65728729 | 1.14E+08 |
| ENSSSCG00000009007 | 8 | 75466134 | 75645030 | Loin muscle area QTL (3264) | 8 | 65728729 | 87166742 |
| ENSSSCG00000009007 | 8 | 75466134 | 75645030 | Empty body lipid content QTL (16930) | 8 | 65728729 | 87166742 |
| ENSSSCG00000009007 | 8 | 75466134 | 75645030 | pH 24 hr post mortem (ham) QTL (18040) | 8 | 72029546 | 1.46E+08 |
| ENSSSCG00000009029 | 8 | 80675001 | 81024492 | Gadoleic acid content QTL (156882) | 8 | 67687532 | 1.17E+08 |
| ENSSSCG00000009029 | 8 | 80675001 | 81024492 | Estimated carcass lean content QTL (5707) | 8 | 67687532 | 1.05E+08 |
| ENSSSCG00000009029 | 8 | 80675001 | 81024492 | Toll-like receptor 9 level QTL (17620) | 8 | 67687532 | 1.05E+08 |
| ENSSSCG00000009029 | 8 | 80675001 | 81024492 | Meat color-a QTL (296) | 8 | 76175680 | 1.08E+08 |
| ENSSSCG00000009029 | 8 | 80675001 | 81024492 | Palmitic acid content QTL (156877) | 8 | 80528576 | 1.17E+08 |
| ENSSSCG00000009029 | 8 | 80675001 | 81024492 | Palmitoleic acid content QTL (156878) | 8 | 80528576 | 1.17E+08 |
| ENSSSCG00000009029 | 8 | 80675001 | 81024492 | MHCII-positive leukocyte number QTL (4269) | 8 | 80528576 | 90104947 |
| ENSSSCG00000009029 | 8 | 80675001 | 81024492 | Loin muscle area QTL (5704) | 8 | 2720560 | 1.05E+08 |
| ENSSSCG00000009029 | 8 | 80675001 | 81024492 | Body weight (30 weeks) QTL (21253) | 8 | 3699961 | 90540236 |
| ENSSSCG00000009029 | 8 | 80675001 | 81024492 | Body weight (34 weeks) QTL (21254) | 8 | 10882240 | 87057920 |
| ENSSSCG00000009029 | 8 | 80675001 | 81024492 | Loin muscle area QTL (38095) | 8 | 12079286 | 1.32E+08 |
| ENSSSCG00000009029 | 8 | 80675001 | 81024492 | Backfat at rump QTL (21255) | 8 | 14908668 | 95110777 |
| ENSSSCG00000009029 | 8 | 80675001 | 81024492 | Abdominal fat weight QTL (21252) | 8 | 24158572 | 1.19E+08 |
| ENSSSCG00000009029 | 8 | 80675001 | 81024492 | Lauric acid content QTL (156942) | 8 | 27423244 | 1.32E+08 |
| ENSSSCG00000009029 | 8 | 80675001 | 81024492 | Alkaline phosphatase activity QTL (15105) | 8 | 27423244 | 1.05E+08 |
| ENSSSCG00000009029 | 8 | 80675001 | 81024492 | Body weight (3 weeks) QTL (29700) | 8 | 27423244 | 90104947 |
| ENSSSCG00000009029 | 8 | 80675001 | 81024492 | Glucose level QTL (15106) | 8 | 57131760 | 1.05E+08 |
| ENSSSCG00000009029 | 8 | 80675001 | 81024492 | Stearic acid content QTL (156879) | 8 | 57131760 | 1.05E+08 |
| ENSSSCG00000009029 | 8 | 80675001 | 81024492 | Uterine capacity QTL (523) | 8 | 57675872 | 1.16E+08 |
| ENSSSCG00000009029 | 8 | 80675001 | 81024492 | Bilirubin level QTL (6374) | 8 | 63334636 | 87166742 |
| ENSSSCG00000009029 | 8 | 80675001 | 81024492 | Palmitic acid content QTL (469) | 8 | 65728729 | 1.22E+08 |
| ENSSSCG00000009029 | 8 | 80675001 | 81024492 | Palmitoleic acid content QTL (470) | 8 | 65728729 | 1.22E+08 |
| ENSSSCG00000009029 | 8 | 80675001 | 81024492 | Average chain length QTL (471) | 8 | 65728729 | 1.22E+08 |
| ENSSSCG00000009029 | 8 | 80675001 | 81024492 | Temperature 40 minutes post mortem (loin) QTL (3999) | 8 | 65728729 | 1.14E+08 |
| ENSSSCG00000009029 | 8 | 80675001 | 81024492 | Loin muscle area QTL (3264) | 8 | 65728729 | 87166742 |
| ENSSSCG00000009029 | 8 | 80675001 | 81024492 | Empty body lipid content QTL (16930) | 8 | 65728729 | 87166742 |
| ENSSSCG00000009029 | 8 | 80675001 | 81024492 | pH 24 hr post mortem (ham) QTL (18040) | 8 | 72029546 | 1.46E+08 |
| ENSSSCG00000030396 | 8 | 87477808 | 87527190 | Gadoleic acid content QTL (156882) | 8 | 67687532 | 1.17E+08 |
| ENSSSCG00000030396 | 8 | 87477808 | 87527190 | Estimated carcass lean content QTL (5707) | 8 | 67687532 | 1.05E+08 |
| ENSSSCG00000030396 | 8 | 87477808 | 87527190 | Toll-like receptor 9 level QTL (17620) | 8 | 67687532 | 1.05E+08 |
| ENSSSCG00000030396 | 8 | 87477808 | 87527190 | Meat color-a QTL (296) | 8 | 76175680 | 1.08E+08 |
| ENSSSCG00000030396 | 8 | 87477808 | 87527190 | Palmitic acid content QTL (156877) | 8 | 80528576 | 1.17E+08 |
| ENSSSCG00000030396 | 8 | 87477808 | 87527190 | Palmitoleic acid content QTL (156878) | 8 | 80528576 | 1.17E+08 |
| ENSSSCG00000030396 | 8 | 87477808 | 87527190 | MHCII-positive leukocyte number QTL (4269) | 8 | 80528576 | 90104947 |
| ENSSSCG00000030396 | 8 | 87477808 | 87527190 | Backfat linear at last rib QTL (3654) | 8 | 87166742 | 1.08E+08 |
| ENSSSCG00000030396 | 8 | 87477808 | 87527190 | Total body fat tissue linear QTL (3655) | 8 | 87166742 | 1.08E+08 |
| ENSSSCG00000030396 | 8 | 87477808 | 87527190 | Backfat at tenth rib QTL (3206) | 8 | 87166742 | 1.08E+08 |
| ENSSSCG00000030396 | 8 | 87477808 | 87527190 | Myofibril fragmentation index QTL (2994) | 8 | 87166742 | 1.05E+08 |
| ENSSSCG00000030396 | 8 | 87477808 | 87527190 | Loin muscle area QTL (5704) | 8 | 2720560 | 1.05E+08 |
| ENSSSCG00000030396 | 8 | 87477808 | 87527190 | Body weight (30 weeks) QTL (21253) | 8 | 3699961 | 90540236 |
| ENSSSCG00000030396 | 8 | 87477808 | 87527190 | Loin muscle area QTL (38095) | 8 | 12079286 | 1.32E+08 |
| ENSSSCG00000030396 | 8 | 87477808 | 87527190 | Backfat at rump QTL (21255) | 8 | 14908668 | 95110777 |
| ENSSSCG00000030396 | 8 | 87477808 | 87527190 | Abdominal fat weight QTL (21252) | 8 | 24158572 | 1.19E+08 |
| ENSSSCG00000030396 | 8 | 87477808 | 87527190 | Lauric acid content QTL (156942) | 8 | 27423244 | 1.32E+08 |
| ENSSSCG00000030396 | 8 | 87477808 | 87527190 | Alkaline phosphatase activity QTL (15105) | 8 | 27423244 | 1.05E+08 |
| ENSSSCG00000030396 | 8 | 87477808 | 87527190 | Body weight (3 weeks) QTL (29700) | 8 | 27423244 | 90104947 |
| ENSSSCG00000030396 | 8 | 87477808 | 87527190 | Glucose level QTL (15106) | 8 | 57131760 | 1.05E+08 |
| ENSSSCG00000030396 | 8 | 87477808 | 87527190 | Stearic acid content QTL (156879) | 8 | 57131760 | 1.05E+08 |
| ENSSSCG00000030396 | 8 | 87477808 | 87527190 | Uterine capacity QTL (523) | 8 | 57675872 | 1.16E+08 |
| ENSSSCG00000030396 | 8 | 87477808 | 87527190 | Palmitic acid content QTL (469) | 8 | 65728729 | 1.22E+08 |
| ENSSSCG00000030396 | 8 | 87477808 | 87527190 | Palmitoleic acid content QTL (470) | 8 | 65728729 | 1.22E+08 |
| ENSSSCG00000030396 | 8 | 87477808 | 87527190 | Average chain length QTL (471) | 8 | 65728729 | 1.22E+08 |
| ENSSSCG00000030396 | 8 | 87477808 | 87527190 | Temperature 40 minutes post mortem (loin) QTL (3999) | 8 | 65728729 | 1.14E+08 |
| ENSSSCG00000030396 | 8 | 87477808 | 87527190 | pH 24 hr post mortem (ham) QTL (18040) | 8 | 72029546 | 1.46E+08 |
| ENSSSCG00000009090 | 8 | 1.02E+08 | 1.02E+08 | Cholesterol level QTL (9562) | 8 | 1.02E+08 | 1.02E+08 |
| ENSSSCG00000009090 | 8 | 1.02E+08 | 1.02E+08 | Gadoleic acid content QTL (156882) | 8 | 67687532 | 1.17E+08 |
| ENSSSCG00000009090 | 8 | 1.02E+08 | 1.02E+08 | Estimated carcass lean content QTL (5707) | 8 | 67687532 | 1.05E+08 |
| ENSSSCG00000009090 | 8 | 1.02E+08 | 1.02E+08 | Toll-like receptor 9 level QTL (17620) | 8 | 67687532 | 1.05E+08 |
| ENSSSCG00000009090 | 8 | 1.02E+08 | 1.02E+08 | Meat color-a QTL (296) | 8 | 76175680 | 1.08E+08 |
| ENSSSCG00000009090 | 8 | 1.02E+08 | 1.02E+08 | Palmitic acid content QTL (156877) | 8 | 80528576 | 1.17E+08 |
| ENSSSCG00000009090 | 8 | 1.02E+08 | 1.02E+08 | Palmitoleic acid content QTL (156878) | 8 | 80528576 | 1.17E+08 |
| ENSSSCG00000009090 | 8 | 1.02E+08 | 1.02E+08 | Backfat linear at last rib QTL (3654) | 8 | 87166742 | 1.08E+08 |
| ENSSSCG00000009090 | 8 | 1.02E+08 | 1.02E+08 | Total body fat tissue linear QTL (3655) | 8 | 87166742 | 1.08E+08 |
| ENSSSCG00000009090 | 8 | 1.02E+08 | 1.02E+08 | Backfat at tenth rib QTL (3206) | 8 | 87166742 | 1.08E+08 |
| ENSSSCG00000009090 | 8 | 1.02E+08 | 1.02E+08 | Myofibril fragmentation index QTL (2994) | 8 | 87166742 | 1.05E+08 |
| ENSSSCG00000009090 | 8 | 1.02E+08 | 1.02E+08 | Meat color a* QTL (21846) | 8 | 90104947 | 1.22E+08 |
| ENSSSCG00000009090 | 8 | 1.02E+08 | 1.02E+08 | Backfat at last rib QTL (17782) | 8 | 90104947 | 1.22E+08 |
| ENSSSCG00000009090 | 8 | 1.02E+08 | 1.02E+08 | Fat area percentage in carcass QTL (18632) | 8 | 90104947 | 1.15E+08 |
| ENSSSCG00000009090 | 8 | 1.02E+08 | 1.02E+08 | Meat color b* QTL (21715) | 8 | 90322592 | 1.21E+08 |
| ENSSSCG00000009090 | 8 | 1.02E+08 | 1.02E+08 | Backfat at last lumbar QTL (565) | 8 | 1E+08 | 1.02E+08 |
| ENSSSCG00000009090 | 8 | 1.02E+08 | 1.02E+08 | Muscle moisture percentage QTL (3886) | 8 | 1E+08 | 1.24E+08 |
| ENSSSCG00000009090 | 8 | 1.02E+08 | 1.02E+08 | Loin muscle area QTL (5704) | 8 | 2720560 | 1.05E+08 |
| ENSSSCG00000009090 | 8 | 1.02E+08 | 1.02E+08 | Loin muscle area QTL (38095) | 8 | 12079286 | 1.32E+08 |
| ENSSSCG00000009090 | 8 | 1.02E+08 | 1.02E+08 | Abdominal fat weight QTL (21252) | 8 | 24158572 | 1.19E+08 |
| ENSSSCG00000009090 | 8 | 1.02E+08 | 1.02E+08 | Lauric acid content QTL (156942) | 8 | 27423244 | 1.32E+08 |
| ENSSSCG00000009090 | 8 | 1.02E+08 | 1.02E+08 | Alkaline phosphatase activity QTL (15105) | 8 | 27423244 | 1.05E+08 |
| ENSSSCG00000009090 | 8 | 1.02E+08 | 1.02E+08 | Glucose level QTL (15106) | 8 | 57131760 | 1.05E+08 |
| ENSSSCG00000009090 | 8 | 1.02E+08 | 1.02E+08 | Stearic acid content QTL (156879) | 8 | 57131760 | 1.05E+08 |
| ENSSSCG00000009090 | 8 | 1.02E+08 | 1.02E+08 | Uterine capacity QTL (523) | 8 | 57675872 | 1.16E+08 |
| ENSSSCG00000009090 | 8 | 1.02E+08 | 1.02E+08 | Palmitic acid content QTL (469) | 8 | 65728729 | 1.22E+08 |
| ENSSSCG00000009090 | 8 | 1.02E+08 | 1.02E+08 | Palmitoleic acid content QTL (470) | 8 | 65728729 | 1.22E+08 |
| ENSSSCG00000009090 | 8 | 1.02E+08 | 1.02E+08 | Average chain length QTL (471) | 8 | 65728729 | 1.22E+08 |
| ENSSSCG00000009090 | 8 | 1.02E+08 | 1.02E+08 | Temperature 40 minutes post mortem (loin) QTL (3999) | 8 | 65728729 | 1.14E+08 |
| ENSSSCG00000009090 | 8 | 1.02E+08 | 1.02E+08 | pH 24 hr post mortem (ham) QTL (18040) | 8 | 72029546 | 1.46E+08 |
| ENSSSCG00000009134 | 8 | 1.12E+08 | 1.12E+08 | Days to 100 kg QTL (153445) | 8 | 1.12E+08 | 1.12E+08 |
| ENSSSCG00000009134 | 8 | 1.12E+08 | 1.12E+08 | 9 hr-24 hr pH decline QTL (5845) | 8 | 1.12E+08 | 1.13E+08 |
| ENSSSCG00000009134 | 8 | 1.12E+08 | 1.12E+08 | Average glycogen QTL (5866) | 8 | 1.12E+08 | 1.13E+08 |
| ENSSSCG00000009134 | 8 | 1.12E+08 | 1.12E+08 | Gadoleic acid content QTL (156882) | 8 | 67687532 | 1.17E+08 |
| ENSSSCG00000009134 | 8 | 1.12E+08 | 1.12E+08 | Palmitic acid content QTL (156877) | 8 | 80528576 | 1.17E+08 |
| ENSSSCG00000009134 | 8 | 1.12E+08 | 1.12E+08 | Palmitoleic acid content QTL (156878) | 8 | 80528576 | 1.17E+08 |
| ENSSSCG00000009134 | 8 | 1.12E+08 | 1.12E+08 | Meat color a* QTL (21846) | 8 | 90104947 | 1.22E+08 |
| ENSSSCG00000009134 | 8 | 1.12E+08 | 1.12E+08 | Backfat at last rib QTL (17782) | 8 | 90104947 | 1.22E+08 |
| ENSSSCG00000009134 | 8 | 1.12E+08 | 1.12E+08 | Fat area percentage in carcass QTL (18632) | 8 | 90104947 | 1.15E+08 |
| ENSSSCG00000009134 | 8 | 1.12E+08 | 1.12E+08 | Meat color b* QTL (21715) | 8 | 90322592 | 1.21E+08 |
| ENSSSCG00000009134 | 8 | 1.12E+08 | 1.12E+08 | Muscle moisture percentage QTL (3886) | 8 | 1E+08 | 1.24E+08 |
| ENSSSCG00000009134 | 8 | 1.12E+08 | 1.12E+08 | Arachidic acid content QTL (156881) | 8 | 1.05E+08 | 1.32E+08 |
| ENSSSCG00000009134 | 8 | 1.12E+08 | 1.12E+08 | Average backfat thickness QTL (5702) | 8 | 1.05E+08 | 1.22E+08 |
| ENSSSCG00000009134 | 8 | 1.12E+08 | 1.12E+08 | Shoulder subcutaneous fat thickness QTL (5703) | 8 | 1.05E+08 | 1.22E+08 |
| ENSSSCG00000009134 | 8 | 1.12E+08 | 1.12E+08 | Average daily gain QTL (5708) | 8 | 1.05E+08 | 1.22E+08 |
| ENSSSCG00000009134 | 8 | 1.12E+08 | 1.12E+08 | Marbling QTL (2995) | 8 | 1.05E+08 | 1.22E+08 |
| ENSSSCG00000009134 | 8 | 1.12E+08 | 1.12E+08 | Adipocyte diameter QTL (18025) | 8 | 1.05E+08 | 1.22E+08 |
| ENSSSCG00000009134 | 8 | 1.12E+08 | 1.12E+08 | Loin muscle area QTL (5) | 8 | 1.08E+08 | 1.15E+08 |
| ENSSSCG00000009134 | 8 | 1.12E+08 | 1.12E+08 | Loin muscle area QTL (38095) | 8 | 12079286 | 1.32E+08 |
| ENSSSCG00000009134 | 8 | 1.12E+08 | 1.12E+08 | Abdominal fat weight QTL (21252) | 8 | 24158572 | 1.19E+08 |
| ENSSSCG00000009134 | 8 | 1.12E+08 | 1.12E+08 | Lauric acid content QTL (156942) | 8 | 27423244 | 1.32E+08 |
| ENSSSCG00000009134 | 8 | 1.12E+08 | 1.12E+08 | Uterine capacity QTL (523) | 8 | 57675872 | 1.16E+08 |
| ENSSSCG00000009134 | 8 | 1.12E+08 | 1.12E+08 | Palmitic acid content QTL (469) | 8 | 65728729 | 1.22E+08 |
| ENSSSCG00000009134 | 8 | 1.12E+08 | 1.12E+08 | Palmitoleic acid content QTL (470) | 8 | 65728729 | 1.22E+08 |
| ENSSSCG00000009134 | 8 | 1.12E+08 | 1.12E+08 | Average chain length QTL (471) | 8 | 65728729 | 1.22E+08 |
| ENSSSCG00000009134 | 8 | 1.12E+08 | 1.12E+08 | Temperature 40 minutes post mortem (loin) QTL (3999) | 8 | 65728729 | 1.14E+08 |
| ENSSSCG00000009134 | 8 | 1.12E+08 | 1.12E+08 | pH 24 hr post mortem (ham) QTL (18040) | 8 | 72029546 | 1.46E+08 |
| ENSSSCG00000009134 | 8 | 1.12E+08 | 1.12E+08 | Off-flavor score QTL (21394) | 8 | 1.08E+08 | 1.39E+08 |
| ENSSSCG00000009134 | 8 | 1.12E+08 | 1.12E+08 | Cooking yield QTL (21437) | 8 | 1.08E+08 | 1.39E+08 |
| ENSSSCG00000009134 | 8 | 1.12E+08 | 1.12E+08 | Muscle moisture percentage QTL (3265) | 8 | 1.08E+08 | 1.39E+08 |
| ENSSSCG00000024168 | 8 | 1.15E+08 | 1.15E+08 | Fatty acid atherogenic index QTL (101918) | 8 | 1.15E+08 | 1.15E+08 |
| ENSSSCG00000024168 | 8 | 1.15E+08 | 1.15E+08 | Palmitoleic acid content QTL (218168) | 8 | 1.15E+08 | 1.15E+08 |
| ENSSSCG00000024168 | 8 | 1.15E+08 | 1.15E+08 | Palmitoleic acid content QTL (218169) | 8 | 1.15E+08 | 1.15E+08 |
| ENSSSCG00000024168 | 8 | 1.15E+08 | 1.15E+08 | Myristic acid content QTL (218201) | 8 | 1.15E+08 | 1.15E+08 |
| ENSSSCG00000024168 | 8 | 1.15E+08 | 1.15E+08 | Palmitic acid content QTL (218254) | 8 | 1.15E+08 | 1.15E+08 |
| ENSSSCG00000024168 | 8 | 1.15E+08 | 1.15E+08 | Gadoleic acid content QTL (156882) | 8 | 67687532 | 1.17E+08 |
| ENSSSCG00000024168 | 8 | 1.15E+08 | 1.15E+08 | Palmitic acid content QTL (156877) | 8 | 80528576 | 1.17E+08 |
| ENSSSCG00000024168 | 8 | 1.15E+08 | 1.15E+08 | Palmitoleic acid content QTL (156878) | 8 | 80528576 | 1.17E+08 |
| ENSSSCG00000024168 | 8 | 1.15E+08 | 1.15E+08 | Meat color a* QTL (21846) | 8 | 90104947 | 1.22E+08 |
| ENSSSCG00000024168 | 8 | 1.15E+08 | 1.15E+08 | Backfat at last rib QTL (17782) | 8 | 90104947 | 1.22E+08 |
| ENSSSCG00000024168 | 8 | 1.15E+08 | 1.15E+08 | Meat color b* QTL (21715) | 8 | 90322592 | 1.21E+08 |
| ENSSSCG00000024168 | 8 | 1.15E+08 | 1.15E+08 | Muscle moisture percentage QTL (3886) | 8 | 1E+08 | 1.24E+08 |
| ENSSSCG00000024168 | 8 | 1.15E+08 | 1.15E+08 | Arachidic acid content QTL (156881) | 8 | 1.05E+08 | 1.32E+08 |
| ENSSSCG00000024168 | 8 | 1.15E+08 | 1.15E+08 | Average backfat thickness QTL (5702) | 8 | 1.05E+08 | 1.22E+08 |
| ENSSSCG00000024168 | 8 | 1.15E+08 | 1.15E+08 | Shoulder subcutaneous fat thickness QTL (5703) | 8 | 1.05E+08 | 1.22E+08 |
| ENSSSCG00000024168 | 8 | 1.15E+08 | 1.15E+08 | Average daily gain QTL (5708) | 8 | 1.05E+08 | 1.22E+08 |
| ENSSSCG00000024168 | 8 | 1.15E+08 | 1.15E+08 | Marbling QTL (2995) | 8 | 1.05E+08 | 1.22E+08 |
| ENSSSCG00000024168 | 8 | 1.15E+08 | 1.15E+08 | Adipocyte diameter QTL (18025) | 8 | 1.05E+08 | 1.22E+08 |
| ENSSSCG00000024168 | 8 | 1.15E+08 | 1.15E+08 | Fat area percentage in carcass QTL (18627) | 8 | 1.15E+08 | 1.31E+08 |
| ENSSSCG00000024168 | 8 | 1.15E+08 | 1.15E+08 | Fat area percentage in carcass QTL (18629) | 8 | 1.15E+08 | 1.31E+08 |
| ENSSSCG00000024168 | 8 | 1.15E+08 | 1.15E+08 | Fat area percentage in carcass QTL (18638) | 8 | 1.15E+08 | 1.31E+08 |
| ENSSSCG00000024168 | 8 | 1.15E+08 | 1.15E+08 | Meat color-L QTL (3792) | 8 | 1.15E+08 | 1.31E+08 |
| ENSSSCG00000024168 | 8 | 1.15E+08 | 1.15E+08 | Loin muscle area QTL (38095) | 8 | 12079286 | 1.32E+08 |
| ENSSSCG00000024168 | 8 | 1.15E+08 | 1.15E+08 | Abdominal fat weight QTL (21252) | 8 | 24158572 | 1.19E+08 |
| ENSSSCG00000024168 | 8 | 1.15E+08 | 1.15E+08 | Lauric acid content QTL (156942) | 8 | 27423244 | 1.32E+08 |
| ENSSSCG00000024168 | 8 | 1.15E+08 | 1.15E+08 | Uterine capacity QTL (523) | 8 | 57675872 | 1.16E+08 |
| ENSSSCG00000024168 | 8 | 1.15E+08 | 1.15E+08 | Palmitic acid content QTL (469) | 8 | 65728729 | 1.22E+08 |
| ENSSSCG00000024168 | 8 | 1.15E+08 | 1.15E+08 | Palmitoleic acid content QTL (470) | 8 | 65728729 | 1.22E+08 |
| ENSSSCG00000024168 | 8 | 1.15E+08 | 1.15E+08 | Average chain length QTL (471) | 8 | 65728729 | 1.22E+08 |
| ENSSSCG00000024168 | 8 | 1.15E+08 | 1.15E+08 | pH 24 hr post mortem (ham) QTL (18040) | 8 | 72029546 | 1.46E+08 |
| ENSSSCG00000024168 | 8 | 1.15E+08 | 1.15E+08 | Off-flavor score QTL (21394) | 8 | 1.08E+08 | 1.39E+08 |
| ENSSSCG00000024168 | 8 | 1.15E+08 | 1.15E+08 | Cooking yield QTL (21437) | 8 | 1.08E+08 | 1.39E+08 |
| ENSSSCG00000024168 | 8 | 1.15E+08 | 1.15E+08 | Muscle moisture percentage QTL (3265) | 8 | 1.08E+08 | 1.39E+08 |
| ENSSSCG00000030957 | 8 | 1.18E+08 | 1.19E+08 | Meat color a* QTL (21846) | 8 | 90104947 | 1.22E+08 |
| ENSSSCG00000030957 | 8 | 1.18E+08 | 1.19E+08 | Backfat at last rib QTL (17782) | 8 | 90104947 | 1.22E+08 |
| ENSSSCG00000030957 | 8 | 1.18E+08 | 1.19E+08 | Meat color b* QTL (21715) | 8 | 90322592 | 1.21E+08 |
| ENSSSCG00000030957 | 8 | 1.18E+08 | 1.19E+08 | Muscle moisture percentage QTL (3886) | 8 | 1E+08 | 1.24E+08 |
| ENSSSCG00000030957 | 8 | 1.18E+08 | 1.19E+08 | Arachidic acid content QTL (156881) | 8 | 1.05E+08 | 1.32E+08 |
| ENSSSCG00000030957 | 8 | 1.18E+08 | 1.19E+08 | Average backfat thickness QTL (5702) | 8 | 1.05E+08 | 1.22E+08 |
| ENSSSCG00000030957 | 8 | 1.18E+08 | 1.19E+08 | Shoulder subcutaneous fat thickness QTL (5703) | 8 | 1.05E+08 | 1.22E+08 |
| ENSSSCG00000030957 | 8 | 1.18E+08 | 1.19E+08 | Average daily gain QTL (5708) | 8 | 1.05E+08 | 1.22E+08 |
| ENSSSCG00000030957 | 8 | 1.18E+08 | 1.19E+08 | Marbling QTL (2995) | 8 | 1.05E+08 | 1.22E+08 |
| ENSSSCG00000030957 | 8 | 1.18E+08 | 1.19E+08 | Adipocyte diameter QTL (18025) | 8 | 1.05E+08 | 1.22E+08 |
| ENSSSCG00000030957 | 8 | 1.18E+08 | 1.19E+08 | Fat area percentage in carcass QTL (18627) | 8 | 1.15E+08 | 1.31E+08 |
| ENSSSCG00000030957 | 8 | 1.18E+08 | 1.19E+08 | Fat area percentage in carcass QTL (18629) | 8 | 1.15E+08 | 1.31E+08 |
| ENSSSCG00000030957 | 8 | 1.18E+08 | 1.19E+08 | Fat area percentage in carcass QTL (18638) | 8 | 1.15E+08 | 1.31E+08 |
| ENSSSCG00000030957 | 8 | 1.18E+08 | 1.19E+08 | Meat color-L QTL (3792) | 8 | 1.15E+08 | 1.31E+08 |
| ENSSSCG00000030957 | 8 | 1.18E+08 | 1.19E+08 | Carcass weight (hot) QTL (16870) | 8 | 1.15E+08 | 1.31E+08 |
| ENSSSCG00000030957 | 8 | 1.18E+08 | 1.19E+08 | Adipocyte diameter QTL (12812) | 8 | 1.15E+08 | 1.22E+08 |
| ENSSSCG00000030957 | 8 | 1.18E+08 | 1.19E+08 | Adipocyte diameter QTL (12813) | 8 | 1.15E+08 | 1.22E+08 |
| ENSSSCG00000030957 | 8 | 1.18E+08 | 1.19E+08 | Adipocyte diameter QTL (12833) | 8 | 1.15E+08 | 1.22E+08 |
| ENSSSCG00000030957 | 8 | 1.18E+08 | 1.19E+08 | Loin muscle area QTL (38095) | 8 | 12079286 | 1.32E+08 |
| ENSSSCG00000030957 | 8 | 1.18E+08 | 1.19E+08 | Abdominal fat weight QTL (21252) | 8 | 24158572 | 1.19E+08 |
| ENSSSCG00000030957 | 8 | 1.18E+08 | 1.19E+08 | Lauric acid content QTL (156942) | 8 | 27423244 | 1.32E+08 |
| ENSSSCG00000030957 | 8 | 1.18E+08 | 1.19E+08 | Palmitic acid content QTL (469) | 8 | 65728729 | 1.22E+08 |
| ENSSSCG00000030957 | 8 | 1.18E+08 | 1.19E+08 | Palmitoleic acid content QTL (470) | 8 | 65728729 | 1.22E+08 |
| ENSSSCG00000030957 | 8 | 1.18E+08 | 1.19E+08 | Average chain length QTL (471) | 8 | 65728729 | 1.22E+08 |
| ENSSSCG00000030957 | 8 | 1.18E+08 | 1.19E+08 | pH 24 hr post mortem (ham) QTL (18040) | 8 | 72029546 | 1.46E+08 |
| ENSSSCG00000030957 | 8 | 1.18E+08 | 1.19E+08 | Off-flavor score QTL (21394) | 8 | 1.08E+08 | 1.39E+08 |
| ENSSSCG00000030957 | 8 | 1.18E+08 | 1.19E+08 | Cooking yield QTL (21437) | 8 | 1.08E+08 | 1.39E+08 |
| ENSSSCG00000030957 | 8 | 1.18E+08 | 1.19E+08 | Muscle moisture percentage QTL (3265) | 8 | 1.08E+08 | 1.39E+08 |
| ENSSSCG00000030957 | 8 | 1.18E+08 | 1.19E+08 | Sodium level QTL (6386) | 8 | 1.17E+08 | 1.37E+08 |
| ENSSSCG00000009230 | 8 | 1.34E+08 | 1.34E+08 | Drip loss QTL (55975) | 8 | 1.34E+08 | 1.34E+08 |
| ENSSSCG00000009230 | 8 | 1.34E+08 | 1.34E+08 | pH 24 hr post mortem (ham) QTL (18040) | 8 | 72029546 | 1.46E+08 |
| ENSSSCG00000009230 | 8 | 1.34E+08 | 1.34E+08 | Off-flavor score QTL (21394) | 8 | 1.08E+08 | 1.39E+08 |
| ENSSSCG00000009230 | 8 | 1.34E+08 | 1.34E+08 | Cooking yield QTL (21437) | 8 | 1.08E+08 | 1.39E+08 |
| ENSSSCG00000009230 | 8 | 1.34E+08 | 1.34E+08 | Muscle moisture percentage QTL (3265) | 8 | 1.08E+08 | 1.39E+08 |
| ENSSSCG00000009230 | 8 | 1.34E+08 | 1.34E+08 | Sodium level QTL (6386) | 8 | 1.17E+08 | 1.37E+08 |
| ENSSSCG00000009230 | 8 | 1.34E+08 | 1.34E+08 | pH 45 minutes post mortem QTL (18699) | 8 | 1.32E+08 | 1.34E+08 |
| ENSSSCG00000009230 | 8 | 1.34E+08 | 1.34E+08 | Conductivity 24 hours postmortem (loin) QTL (18700) | 8 | 1.32E+08 | 1.34E+08 |
| ENSSSCG00000009241 | 8 | 1.35E+08 | 1.35E+08 | pH 24 hr post mortem (ham) QTL (18040) | 8 | 72029546 | 1.46E+08 |
| ENSSSCG00000009241 | 8 | 1.35E+08 | 1.35E+08 | Off-flavor score QTL (21394) | 8 | 1.08E+08 | 1.39E+08 |
| ENSSSCG00000009241 | 8 | 1.35E+08 | 1.35E+08 | Cooking yield QTL (21437) | 8 | 1.08E+08 | 1.39E+08 |
| ENSSSCG00000009241 | 8 | 1.35E+08 | 1.35E+08 | Muscle moisture percentage QTL (3265) | 8 | 1.08E+08 | 1.39E+08 |
| ENSSSCG00000009241 | 8 | 1.35E+08 | 1.35E+08 | Sodium level QTL (6386) | 8 | 1.17E+08 | 1.37E+08 |
| ENSSSCG00000009242 | 8 | 1.35E+08 | 1.35E+08 | pH 24 hr post mortem (ham) QTL (18040) | 8 | 72029546 | 1.46E+08 |
| ENSSSCG00000009242 | 8 | 1.35E+08 | 1.35E+08 | Off-flavor score QTL (21394) | 8 | 1.08E+08 | 1.39E+08 |
| ENSSSCG00000009242 | 8 | 1.35E+08 | 1.35E+08 | Cooking yield QTL (21437) | 8 | 1.08E+08 | 1.39E+08 |
| ENSSSCG00000009242 | 8 | 1.35E+08 | 1.35E+08 | Muscle moisture percentage QTL (3265) | 8 | 1.08E+08 | 1.39E+08 |
| ENSSSCG00000009242 | 8 | 1.35E+08 | 1.35E+08 | Sodium level QTL (6386) | 8 | 1.17E+08 | 1.37E+08 |
| ENSSSCG00000027722 | 8 | 1.39E+08 | 1.39E+08 | pH 24 hr post mortem (ham) QTL (18040) | 8 | 72029546 | 1.46E+08 |
| ENSSSCG00000027722 | 8 | 1.39E+08 | 1.39E+08 | Off-flavor score QTL (21394) | 8 | 1.08E+08 | 1.39E+08 |
| ENSSSCG00000027722 | 8 | 1.39E+08 | 1.39E+08 | Cooking yield QTL (21437) | 8 | 1.08E+08 | 1.39E+08 |
| ENSSSCG00000027722 | 8 | 1.39E+08 | 1.39E+08 | Muscle moisture percentage QTL (3265) | 8 | 1.08E+08 | 1.39E+08 |
| ENSSSCG00000014828 | 9 | 8121954 | 8199099 | Shear force QTL (3271) | 9 | 3916128 | 36028377 |
| ENSSSCG00000014828 | 9 | 8121954 | 8199099 | Average daily gain QTL (4155) | 9 | 3916128 | 14783383 |
| ENSSSCG00000014828 | 9 | 8121954 | 8199099 | PH for Longissmus dorsi QTL (4157) | 9 | 3916128 | 14783383 |
| ENSSSCG00000014828 | 9 | 8121954 | 8199099 | Body weight (slaughter) QTL (4247) | 9 | 3916128 | 14783383 |
| ENSSSCG00000014828 | 9 | 8121954 | 8199099 | Carcass weight (hot) QTL (4248) | 9 | 3916128 | 14783383 |
| ENSSSCG00000014828 | 9 | 8121954 | 8199099 | body weight (22 weeks) QTL (16931) | 9 | 3916128 | 14783383 |
| ENSSSCG00000014828 | 9 | 8121954 | 8199099 | Empty body lipid content QTL (16932) | 9 | 3916128 | 14783383 |
| ENSSSCG00000014828 | 9 | 8121954 | 8199099 | body weight (19 weeks) QTL (16933) | 9 | 3916128 | 14783383 |
| ENSSSCG00000014828 | 9 | 8121954 | 8199099 | Drip loss QTL (3266) | 9 | 3916128 | 14783383 |
| ENSSSCG00000014828 | 9 | 8121954 | 8199099 | Carcass weight (hot) QTL (3267) | 9 | 3916128 | 14783383 |
| ENSSSCG00000014828 | 9 | 8121954 | 8199099 | Body weight (slaughter) QTL (3268) | 9 | 3916128 | 14783383 |
| ENSSSCG00000014828 | 9 | 8121954 | 8199099 | Carcass temperature (24 hr post-mortem) QTL (3309) | 9 | 3916128 | 14783383 |
| ENSSSCG00000014828 | 9 | 8121954 | 8199099 | Empty body lipid content QTL (3318) | 9 | 3916128 | 14783383 |
| ENSSSCG00000014828 | 9 | 8121954 | 8199099 | Backfat at last rib QTL (3676) | 9 | 3916128 | 14783383 |
| ENSSSCG00000014828 | 9 | 8121954 | 8199099 | Average daily gain QTL (3677) | 9 | 3916128 | 14783383 |
| ENSSSCG00000014828 | 9 | 8121954 | 8199099 | Carcass length QTL (3700) | 9 | 3916128 | 14783383 |
| ENSSSCG00000014828 | 9 | 8121954 | 8199099 | Carcass weight (hot) QTL (3701) | 9 | 3916128 | 14783383 |
| ENSSSCG00000014828 | 9 | 8121954 | 8199099 | PH for Longissmus dorsi QTL (3702) | 9 | 3916128 | 14783383 |
| ENSSSCG00000014828 | 9 | 8121954 | 8199099 | Empty body protein content QTL (3186) | 9 | 3916128 | 14783383 |
| ENSSSCG00000014828 | 9 | 8121954 | 8199099 | Days to 105 kg QTL (3187) | 9 | 3916128 | 14783383 |
| ENSSSCG00000014828 | 9 | 8121954 | 8199099 | Estimated carcass lean content QTL (3207) | 9 | 3916128 | 14783383 |
| ENSSSCG00000014828 | 9 | 8121954 | 8199099 | backfat above muscle dorsi QTL (5205) | 9 | 3916128 | 1.36E+08 |
| ENSSSCG00000014828 | 9 | 8121954 | 8199099 | Backfat at mid-back QTL (5246) | 9 | 3916128 | 1.22E+08 |
| ENSSSCG00000014828 | 9 | 8121954 | 8199099 | Backfat at mid-back QTL (5213) | 9 | 3916128 | 96532555 |
| ENSSSCG00000014828 | 9 | 8121954 | 8199099 | Muscle moisture percentage QTL (38097) | 9 | 6363708 | 1.36E+08 |
| ENSSSCG00000014828 | 9 | 8121954 | 8199099 | Meat color a* QTL (38098) | 9 | 6363708 | 1.36E+08 |
| ENSSSCG00000014828 | 9 | 8121954 | 8199099 | Meat color L* QTL (38099) | 9 | 6363708 | 1.36E+08 |
| ENSSSCG00000028979 | 9 | 10088798 | 10369037 | Arachidonic acid to dihomo-gamma-linolenic acid ratio QTL (213669) | 9 | 10212535 | 10212539 |
| ENSSSCG00000028979 | 9 | 10088798 | 10369037 | Arachidonic acid to dihomo-gamma-linolenic acid ratio QTL (213803) | 9 | 10212535 | 10212539 |
| ENSSSCG00000028979 | 9 | 10088798 | 10369037 | Stearic acid to palmitic acid ratio QTL (101470) | 9 | 10250584 | 10250588 |
| ENSSSCG00000028979 | 9 | 10088798 | 10369037 | Myristic acid content QTL (169958) | 9 | 10311853 | 10311857 |
| ENSSSCG00000028979 | 9 | 10088798 | 10369037 | Myristic acid content QTL (169959) | 9 | 10311853 | 10311857 |
| ENSSSCG00000028979 | 9 | 10088798 | 10369037 | Myristic acid content QTL (169960) | 9 | 10331865 | 10331869 |
| ENSSSCG00000028979 | 9 | 10088798 | 10369037 | Myristic acid content QTL (169961) | 9 | 10331865 | 10331869 |
| ENSSSCG00000028979 | 9 | 10088798 | 10369037 | Shear force QTL (3271) | 9 | 3916128 | 36028377 |
| ENSSSCG00000028979 | 9 | 10088798 | 10369037 | Average daily gain QTL (4155) | 9 | 3916128 | 14783383 |
| ENSSSCG00000028979 | 9 | 10088798 | 10369037 | PH for Longissmus dorsi QTL (4157) | 9 | 3916128 | 14783383 |
| ENSSSCG00000028979 | 9 | 10088798 | 10369037 | Body weight (slaughter) QTL (4247) | 9 | 3916128 | 14783383 |
| ENSSSCG00000028979 | 9 | 10088798 | 10369037 | Carcass weight (hot) QTL (4248) | 9 | 3916128 | 14783383 |
| ENSSSCG00000028979 | 9 | 10088798 | 10369037 | body weight (22 weeks) QTL (16931) | 9 | 3916128 | 14783383 |
| ENSSSCG00000028979 | 9 | 10088798 | 10369037 | Empty body lipid content QTL (16932) | 9 | 3916128 | 14783383 |
| ENSSSCG00000028979 | 9 | 10088798 | 10369037 | body weight (19 weeks) QTL (16933) | 9 | 3916128 | 14783383 |
| ENSSSCG00000028979 | 9 | 10088798 | 10369037 | Drip loss QTL (3266) | 9 | 3916128 | 14783383 |
| ENSSSCG00000028979 | 9 | 10088798 | 10369037 | Carcass weight (hot) QTL (3267) | 9 | 3916128 | 14783383 |
| ENSSSCG00000028979 | 9 | 10088798 | 10369037 | Body weight (slaughter) QTL (3268) | 9 | 3916128 | 14783383 |
| ENSSSCG00000028979 | 9 | 10088798 | 10369037 | Carcass temperature (24 hr post-mortem) QTL (3309) | 9 | 3916128 | 14783383 |
| ENSSSCG00000028979 | 9 | 10088798 | 10369037 | Empty body lipid content QTL (3318) | 9 | 3916128 | 14783383 |
| ENSSSCG00000028979 | 9 | 10088798 | 10369037 | Backfat at last rib QTL (3676) | 9 | 3916128 | 14783383 |
| ENSSSCG00000028979 | 9 | 10088798 | 10369037 | Average daily gain QTL (3677) | 9 | 3916128 | 14783383 |
| ENSSSCG00000028979 | 9 | 10088798 | 10369037 | Carcass length QTL (3700) | 9 | 3916128 | 14783383 |
| ENSSSCG00000028979 | 9 | 10088798 | 10369037 | Carcass weight (hot) QTL (3701) | 9 | 3916128 | 14783383 |
| ENSSSCG00000028979 | 9 | 10088798 | 10369037 | PH for Longissmus dorsi QTL (3702) | 9 | 3916128 | 14783383 |
| ENSSSCG00000028979 | 9 | 10088798 | 10369037 | Empty body protein content QTL (3186) | 9 | 3916128 | 14783383 |
| ENSSSCG00000028979 | 9 | 10088798 | 10369037 | Days to 105 kg QTL (3187) | 9 | 3916128 | 14783383 |
| ENSSSCG00000028979 | 9 | 10088798 | 10369037 | Estimated carcass lean content QTL (3207) | 9 | 3916128 | 14783383 |
| ENSSSCG00000028979 | 9 | 10088798 | 10369037 | backfat above muscle dorsi QTL (5205) | 9 | 3916128 | 1.36E+08 |
| ENSSSCG00000028979 | 9 | 10088798 | 10369037 | Backfat at mid-back QTL (5246) | 9 | 3916128 | 1.22E+08 |
| ENSSSCG00000028979 | 9 | 10088798 | 10369037 | Backfat at mid-back QTL (5213) | 9 | 3916128 | 96532555 |
| ENSSSCG00000028979 | 9 | 10088798 | 10369037 | Muscle moisture percentage QTL (38097) | 9 | 6363708 | 1.36E+08 |
| ENSSSCG00000028979 | 9 | 10088798 | 10369037 | Meat color a* QTL (38098) | 9 | 6363708 | 1.36E+08 |
| ENSSSCG00000028979 | 9 | 10088798 | 10369037 | Meat color L* QTL (38099) | 9 | 6363708 | 1.36E+08 |
| ENSSSCG00000014949 | 9 | 26167401 | 26228612 | Shear force QTL (3271) | 9 | 3916128 | 36028377 |
| ENSSSCG00000014949 | 9 | 26167401 | 26228612 | Average backfat thickness QTL (5434) | 9 | 14783383 | 48266277 |
| ENSSSCG00000014949 | 9 | 26167401 | 26228612 | Leptin level QTL (18644) | 9 | 14783383 | 36028377 |
| ENSSSCG00000014949 | 9 | 26167401 | 26228612 | Average daily gain QTL (5712) | 9 | 14783383 | 36028377 |
| ENSSSCG00000014949 | 9 | 26167401 | 26228612 | Average daily gain QTL (5713) | 9 | 14783383 | 36028377 |
| ENSSSCG00000014949 | 9 | 26167401 | 26228612 | Belly fat area QTL (5149) | 9 | 14783383 | 36028377 |
| ENSSSCG00000014949 | 9 | 26167401 | 26228612 | Time spent rooting QTL (5919) | 9 | 14783383 | 36028377 |
| ENSSSCG00000014949 | 9 | 26167401 | 26228612 | Myristic acid content QTL (15849) | 9 | 14783383 | 28294024 |
| ENSSSCG00000014949 | 9 | 26167401 | 26228612 | Linolenic acid content QTL (9582) | 9 | 14783383 | 26825476 |
| ENSSSCG00000014949 | 9 | 26167401 | 26228612 | backfat above muscle dorsi QTL (5205) | 9 | 3916128 | 1.36E+08 |
| ENSSSCG00000014949 | 9 | 26167401 | 26228612 | Backfat at mid-back QTL (5246) | 9 | 3916128 | 1.22E+08 |
| ENSSSCG00000014949 | 9 | 26167401 | 26228612 | Backfat at mid-back QTL (5213) | 9 | 3916128 | 96532555 |
| ENSSSCG00000014949 | 9 | 26167401 | 26228612 | Muscle moisture percentage QTL (38097) | 9 | 6363708 | 1.36E+08 |
| ENSSSCG00000014949 | 9 | 26167401 | 26228612 | Meat color a* QTL (38098) | 9 | 6363708 | 1.36E+08 |
| ENSSSCG00000014949 | 9 | 26167401 | 26228612 | Meat color L* QTL (38099) | 9 | 6363708 | 1.36E+08 |
| ENSSSCG00000014949 | 9 | 26167401 | 26228612 | Leaf fat weight QTL (21262) | 9 | 22321929 | 1.16E+08 |
| ENSSSCG00000025606 | 9 | 36620658 | 36759554 | HDL cholesterol QTL (7721) | 9 | 35519280 | 40688569 |
| ENSSSCG00000025606 | 9 | 36620658 | 36759554 | Average backfat thickness QTL (5434) | 9 | 14783383 | 48266277 |
| ENSSSCG00000025606 | 9 | 36620658 | 36759554 | Leaf fat weight QTL (41) | 9 | 26825476 | 48266277 |
| ENSSSCG00000025606 | 9 | 36620658 | 36759554 | Average daily gain QTL (279) | 9 | 36028377 | 64028692 |
| ENSSSCG00000025606 | 9 | 36620658 | 36759554 | Muscle fat content QTL (3273) | 9 | 36028377 | 55902727 |
| ENSSSCG00000025606 | 9 | 36620658 | 36759554 | backfat above muscle dorsi QTL (5205) | 9 | 3916128 | 1.36E+08 |
| ENSSSCG00000025606 | 9 | 36620658 | 36759554 | Backfat at mid-back QTL (5246) | 9 | 3916128 | 1.22E+08 |
| ENSSSCG00000025606 | 9 | 36620658 | 36759554 | Backfat at mid-back QTL (5213) | 9 | 3916128 | 96532555 |
| ENSSSCG00000025606 | 9 | 36620658 | 36759554 | Muscle moisture percentage QTL (38097) | 9 | 6363708 | 1.36E+08 |
| ENSSSCG00000025606 | 9 | 36620658 | 36759554 | Meat color a* QTL (38098) | 9 | 6363708 | 1.36E+08 |
| ENSSSCG00000025606 | 9 | 36620658 | 36759554 | Meat color L* QTL (38099) | 9 | 6363708 | 1.36E+08 |
| ENSSSCG00000025606 | 9 | 36620658 | 36759554 | Leaf fat weight QTL (21262) | 9 | 22321929 | 1.16E+08 |
| ENSSSCG00000025606 | 9 | 36620658 | 36759554 | pH 40 minutes post mortem (ham) QTL (4000) | 9 | 36028377 | 1.02E+08 |
| ENSSSCG00000025606 | 9 | 36620658 | 36759554 | androstenone, laboratory QTL (4210) | 9 | 36028377 | 75874980 |
| ENSSSCG00000025606 | 9 | 36620658 | 36759554 | Stearic acid content QTL (156884) | 9 | 36028377 | 68923852 |
| ENSSSCG00000015052 | 9 | 41417559 | 41480985 | Average backfat thickness QTL (5434) | 9 | 14783383 | 48266277 |
| ENSSSCG00000015052 | 9 | 41417559 | 41480985 | Leaf fat weight QTL (41) | 9 | 26825476 | 48266277 |
| ENSSSCG00000015052 | 9 | 41417559 | 41480985 | Average daily gain QTL (279) | 9 | 36028377 | 64028692 |
| ENSSSCG00000015052 | 9 | 41417559 | 41480985 | Muscle fat content QTL (3273) | 9 | 36028377 | 55902727 |
| ENSSSCG00000015052 | 9 | 41417559 | 41480985 | backfat above muscle dorsi QTL (5205) | 9 | 3916128 | 1.36E+08 |
| ENSSSCG00000015052 | 9 | 41417559 | 41480985 | Backfat at mid-back QTL (5246) | 9 | 3916128 | 1.22E+08 |
| ENSSSCG00000015052 | 9 | 41417559 | 41480985 | Backfat at mid-back QTL (5213) | 9 | 3916128 | 96532555 |
| ENSSSCG00000015052 | 9 | 41417559 | 41480985 | Muscle moisture percentage QTL (38097) | 9 | 6363708 | 1.36E+08 |
| ENSSSCG00000015052 | 9 | 41417559 | 41480985 | Meat color a* QTL (38098) | 9 | 6363708 | 1.36E+08 |
| ENSSSCG00000015052 | 9 | 41417559 | 41480985 | Meat color L* QTL (38099) | 9 | 6363708 | 1.36E+08 |
| ENSSSCG00000015052 | 9 | 41417559 | 41480985 | Leaf fat weight QTL (21262) | 9 | 22321929 | 1.16E+08 |
| ENSSSCG00000015052 | 9 | 41417559 | 41480985 | pH 40 minutes post mortem (ham) QTL (4000) | 9 | 36028377 | 1.02E+08 |
| ENSSSCG00000015052 | 9 | 41417559 | 41480985 | androstenone, laboratory QTL (4210) | 9 | 36028377 | 75874980 |
| ENSSSCG00000015052 | 9 | 41417559 | 41480985 | Stearic acid content QTL (156884) | 9 | 36028377 | 68923852 |
| ENSSSCG00000015052 | 9 | 41417559 | 41480985 | Palmitoleic acid content QTL (15852) | 9 | 40825634 | 72154658 |
| ENSSSCG00000015097 | 9 | 46025445 | 46063814 | Average backfat thickness QTL (5434) | 9 | 14783383 | 48266277 |
| ENSSSCG00000015097 | 9 | 46025445 | 46063814 | Leaf fat weight QTL (41) | 9 | 26825476 | 48266277 |
| ENSSSCG00000015097 | 9 | 46025445 | 46063814 | Average daily gain QTL (279) | 9 | 36028377 | 64028692 |
| ENSSSCG00000015097 | 9 | 46025445 | 46063814 | Muscle fat content QTL (3273) | 9 | 36028377 | 55902727 |
| ENSSSCG00000015097 | 9 | 46025445 | 46063814 | backfat above muscle dorsi QTL (5205) | 9 | 3916128 | 1.36E+08 |
| ENSSSCG00000015097 | 9 | 46025445 | 46063814 | Backfat at mid-back QTL (5246) | 9 | 3916128 | 1.22E+08 |
| ENSSSCG00000015097 | 9 | 46025445 | 46063814 | Backfat at mid-back QTL (5213) | 9 | 3916128 | 96532555 |
| ENSSSCG00000015097 | 9 | 46025445 | 46063814 | Muscle moisture percentage QTL (38097) | 9 | 6363708 | 1.36E+08 |
| ENSSSCG00000015097 | 9 | 46025445 | 46063814 | Meat color a* QTL (38098) | 9 | 6363708 | 1.36E+08 |
| ENSSSCG00000015097 | 9 | 46025445 | 46063814 | Meat color L* QTL (38099) | 9 | 6363708 | 1.36E+08 |
| ENSSSCG00000015097 | 9 | 46025445 | 46063814 | Leaf fat weight QTL (21262) | 9 | 22321929 | 1.16E+08 |
| ENSSSCG00000015097 | 9 | 46025445 | 46063814 | pH 40 minutes post mortem (ham) QTL (4000) | 9 | 36028377 | 1.02E+08 |
| ENSSSCG00000015097 | 9 | 46025445 | 46063814 | androstenone, laboratory QTL (4210) | 9 | 36028377 | 75874980 |
| ENSSSCG00000015097 | 9 | 46025445 | 46063814 | Stearic acid content QTL (156884) | 9 | 36028377 | 68923852 |
| ENSSSCG00000015097 | 9 | 46025445 | 46063814 | Palmitoleic acid content QTL (15852) | 9 | 40825634 | 72154658 |
| ENSSSCG00000015283 | 9 | 65073738 | 65154538 | Body weight (slaughter) QTL (1105) | 9 | 52965631 | 65301434 |
| ENSSSCG00000015283 | 9 | 65073738 | 65154538 | backfat above muscle dorsi QTL (5205) | 9 | 3916128 | 1.36E+08 |
| ENSSSCG00000015283 | 9 | 65073738 | 65154538 | Backfat at mid-back QTL (5246) | 9 | 3916128 | 1.22E+08 |
| ENSSSCG00000015283 | 9 | 65073738 | 65154538 | Backfat at mid-back QTL (5213) | 9 | 3916128 | 96532555 |
| ENSSSCG00000015283 | 9 | 65073738 | 65154538 | Muscle moisture percentage QTL (38097) | 9 | 6363708 | 1.36E+08 |
| ENSSSCG00000015283 | 9 | 65073738 | 65154538 | Meat color a* QTL (38098) | 9 | 6363708 | 1.36E+08 |
| ENSSSCG00000015283 | 9 | 65073738 | 65154538 | Meat color L* QTL (38099) | 9 | 6363708 | 1.36E+08 |
| ENSSSCG00000015283 | 9 | 65073738 | 65154538 | Leaf fat weight QTL (21262) | 9 | 22321929 | 1.16E+08 |
| ENSSSCG00000015283 | 9 | 65073738 | 65154538 | pH 40 minutes post mortem (ham) QTL (4000) | 9 | 36028377 | 1.02E+08 |
| ENSSSCG00000015283 | 9 | 65073738 | 65154538 | androstenone, laboratory QTL (4210) | 9 | 36028377 | 75874980 |
| ENSSSCG00000015283 | 9 | 65073738 | 65154538 | Stearic acid content QTL (156884) | 9 | 36028377 | 68923852 |
| ENSSSCG00000015283 | 9 | 65073738 | 65154538 | Palmitoleic acid content QTL (15852) | 9 | 40825634 | 72154658 |
| ENSSSCG00000015283 | 9 | 65073738 | 65154538 | Backfat at first rib QTL (21261) | 9 | 55804824 | 1.3E+08 |
| ENSSSCG00000015283 | 9 | 65073738 | 65154538 | Drip loss QTL (3806) | 9 | 55902727 | 1.2E+08 |
| ENSSSCG00000015283 | 9 | 65073738 | 65154538 | Meat color-L QTL (3807) | 9 | 55902727 | 1.2E+08 |
| ENSSSCG00000015283 | 9 | 65073738 | 65154538 | Meat color-L QTL (3817) | 9 | 55902727 | 1.2E+08 |
| ENSSSCG00000015283 | 9 | 65073738 | 65154538 | Off-flavor score QTL (3812) | 9 | 55902727 | 1.01E+08 |
| ENSSSCG00000015283 | 9 | 65073738 | 65154538 | Loin muscle area QTL (5178) | 9 | 55902727 | 84588364 |
| ENSSSCG00000015283 | 9 | 65073738 | 65154538 | External fat on loin QTL (5189) | 9 | 55902727 | 84588364 |
| ENSSSCG00000015283 | 9 | 65073738 | 65154538 | Average daily gain QTL (5196) | 9 | 55902727 | 84588364 |
| ENSSSCG00000015283 | 9 | 65073738 | 65154538 | Muscle moisture percentage QTL (3274) | 9 | 55902727 | 77343528 |
| ENSSSCG00000015283 | 9 | 65073738 | 65154538 | Off-flavor score QTL (3818) | 9 | 55902727 | 74895948 |
| ENSSSCG00000015283 | 9 | 65073738 | 65154538 | Average backfat thickness QTL (21258) | 9 | 56294340 | 1.31E+08 |
| ENSSSCG00000015283 | 9 | 65073738 | 65154538 | Palmitoleic acid content QTL (3110) | 9 | 60210468 | 81553365 |
| ENSSSCG00000015283 | 9 | 65073738 | 65154538 | Stearic acid content QTL (3112) | 9 | 60210468 | 81553365 |
| ENSSSCG00000015283 | 9 | 65073738 | 65154538 | Saturated fatty acid content QTL (3115) | 9 | 60210468 | 81553365 |
| ENSSSCG00000015283 | 9 | 65073738 | 65154538 | Stearic acid content QTL (3120) | 9 | 60210468 | 81553365 |
| ENSSSCG00000015283 | 9 | 65073738 | 65154538 | Average daily gain QTL (16854) | 9 | 64028692 | 79203688 |
| ENSSSCG00000026177 | 9 | 92481564 | 92528186 | Body weight (3 weeks) QTL (21260) | 9 | 70784013 | 1.25E+08 |
| ENSSSCG00000026177 | 9 | 92481564 | 92528186 | Diameter of type I muscle fibers QTL (2813) | 9 | 71763045 | 98392716 |
| ENSSSCG00000026177 | 9 | 92481564 | 92528186 | Haptoglobin concentration QTL (9646) | 9 | 71763045 | 98392716 |
| ENSSSCG00000026177 | 9 | 92481564 | 92528186 | Loin muscle area QTL (5710) | 9 | 71763045 | 98392716 |
| ENSSSCG00000026177 | 9 | 92481564 | 92528186 | Abdominal fat weight QTL (21259) | 9 | 72937884 | 1.24E+08 |
| ENSSSCG00000026177 | 9 | 92481564 | 92528186 | Backfat at tenth rib QTL (3208) | 9 | 77343528 | 93987072 |
| ENSSSCG00000026177 | 9 | 92481564 | 92528186 | Carcass length QTL (3887) | 9 | 78028850 | 1.03E+08 |
| ENSSSCG00000026177 | 9 | 92481564 | 92528186 | Average daily gain QTL (5163) | 9 | 78322560 | 96924168 |
| ENSSSCG00000026177 | 9 | 92481564 | 92528186 | Protein accretion rate QTL (5154) | 9 | 79791108 | 98882232 |
| ENSSSCG00000026177 | 9 | 92481564 | 92528186 | Immunoglobulin G level QTL (21337) | 9 | 81553365 | 98392716 |
| ENSSSCG00000026177 | 9 | 92481564 | 92528186 | Lipid accretion rate QTL (5155) | 9 | 87133848 | 94966104 |
| ENSSSCG00000026177 | 9 | 92481564 | 92528186 | backfat above muscle dorsi QTL (5205) | 9 | 3916128 | 1.36E+08 |
| ENSSSCG00000026177 | 9 | 92481564 | 92528186 | Backfat at mid-back QTL (5246) | 9 | 3916128 | 1.22E+08 |
| ENSSSCG00000026177 | 9 | 92481564 | 92528186 | Backfat at mid-back QTL (5213) | 9 | 3916128 | 96532555 |
| ENSSSCG00000026177 | 9 | 92481564 | 92528186 | Muscle moisture percentage QTL (38097) | 9 | 6363708 | 1.36E+08 |
| ENSSSCG00000026177 | 9 | 92481564 | 92528186 | Meat color a* QTL (38098) | 9 | 6363708 | 1.36E+08 |
| ENSSSCG00000026177 | 9 | 92481564 | 92528186 | Meat color L* QTL (38099) | 9 | 6363708 | 1.36E+08 |
| ENSSSCG00000026177 | 9 | 92481564 | 92528186 | Leaf fat weight QTL (21262) | 9 | 22321929 | 1.16E+08 |
| ENSSSCG00000026177 | 9 | 92481564 | 92528186 | pH 40 minutes post mortem (ham) QTL (4000) | 9 | 36028377 | 1.02E+08 |
| ENSSSCG00000026177 | 9 | 92481564 | 92528186 | Backfat at first rib QTL (21261) | 9 | 55804824 | 1.3E+08 |
| ENSSSCG00000026177 | 9 | 92481564 | 92528186 | Drip loss QTL (3806) | 9 | 55902727 | 1.2E+08 |
| ENSSSCG00000026177 | 9 | 92481564 | 92528186 | Meat color-L QTL (3807) | 9 | 55902727 | 1.2E+08 |
| ENSSSCG00000026177 | 9 | 92481564 | 92528186 | Meat color-L QTL (3817) | 9 | 55902727 | 1.2E+08 |
| ENSSSCG00000026177 | 9 | 92481564 | 92528186 | Off-flavor score QTL (3812) | 9 | 55902727 | 1.01E+08 |
| ENSSSCG00000026177 | 9 | 92481564 | 92528186 | Average backfat thickness QTL (21258) | 9 | 56294340 | 1.31E+08 |
| ENSSSCG00000015428 | 9 | 1.05E+08 | 1.05E+08 | Body weight (3 weeks) QTL (21260) | 9 | 70784013 | 1.25E+08 |
| ENSSSCG00000015428 | 9 | 1.05E+08 | 1.05E+08 | Abdominal fat weight QTL (21259) | 9 | 72937884 | 1.24E+08 |
| ENSSSCG00000015428 | 9 | 1.05E+08 | 1.05E+08 | Meat color-b QTL (3275) | 9 | 93987072 | 1.27E+08 |
| ENSSSCG00000015428 | 9 | 1.05E+08 | 1.05E+08 | Carcass temperature (45 minutes post-mortem) QTL (3310) | 9 | 93987072 | 1.27E+08 |
| ENSSSCG00000015428 | 9 | 1.05E+08 | 1.05E+08 | Shear force QTL (223) | 9 | 98392716 | 1.2E+08 |
| ENSSSCG00000015428 | 9 | 1.05E+08 | 1.05E+08 | Empty body protein content QTL (5156) | 9 | 1.01E+08 | 1.2E+08 |
| ENSSSCG00000015428 | 9 | 1.05E+08 | 1.05E+08 | Empty body protein content QTL (5157) | 9 | 1.01E+08 | 1.2E+08 |
| ENSSSCG00000015428 | 9 | 1.05E+08 | 1.05E+08 | Empty body lipid content QTL (5158) | 9 | 1.01E+08 | 1.2E+08 |
| ENSSSCG00000015428 | 9 | 1.05E+08 | 1.05E+08 | pH 24 hr post-mortem (loin) QTL (21859) | 9 | 1.01E+08 | 1.2E+08 |
| ENSSSCG00000015428 | 9 | 1.05E+08 | 1.05E+08 | backfat above muscle dorsi QTL (5205) | 9 | 3916128 | 1.36E+08 |
| ENSSSCG00000015428 | 9 | 1.05E+08 | 1.05E+08 | Backfat at mid-back QTL (5246) | 9 | 3916128 | 1.22E+08 |
| ENSSSCG00000015428 | 9 | 1.05E+08 | 1.05E+08 | Muscle moisture percentage QTL (38097) | 9 | 6363708 | 1.36E+08 |
| ENSSSCG00000015428 | 9 | 1.05E+08 | 1.05E+08 | Meat color a* QTL (38098) | 9 | 6363708 | 1.36E+08 |
| ENSSSCG00000015428 | 9 | 1.05E+08 | 1.05E+08 | Meat color L* QTL (38099) | 9 | 6363708 | 1.36E+08 |
| ENSSSCG00000015428 | 9 | 1.05E+08 | 1.05E+08 | Leaf fat weight QTL (21262) | 9 | 22321929 | 1.16E+08 |
| ENSSSCG00000015428 | 9 | 1.05E+08 | 1.05E+08 | Backfat at first rib QTL (21261) | 9 | 55804824 | 1.3E+08 |
| ENSSSCG00000015428 | 9 | 1.05E+08 | 1.05E+08 | Drip loss QTL (3806) | 9 | 55902727 | 1.2E+08 |
| ENSSSCG00000015428 | 9 | 1.05E+08 | 1.05E+08 | Meat color-L QTL (3807) | 9 | 55902727 | 1.2E+08 |
| ENSSSCG00000015428 | 9 | 1.05E+08 | 1.05E+08 | Meat color-L QTL (3817) | 9 | 55902727 | 1.2E+08 |
| ENSSSCG00000015428 | 9 | 1.05E+08 | 1.05E+08 | Average backfat thickness QTL (21258) | 9 | 56294340 | 1.31E+08 |
| ENSSSCG00000015428 | 9 | 1.05E+08 | 1.05E+08 | Diameter of type IIa muscle fibers QTL (7017) | 9 | 1.01E+08 | 1.4E+08 |
| ENSSSCG00000015428 | 9 | 1.05E+08 | 1.05E+08 | Potassium level QTL (6382) | 9 | 1.01E+08 | 1.36E+08 |
| ENSSSCG00000015428 | 9 | 1.05E+08 | 1.05E+08 | body weight (10 weeks) QTL (29704) | 9 | 1.01E+08 | 1.36E+08 |
| ENSSSCG00000015428 | 9 | 1.05E+08 | 1.05E+08 | Carcass weight (hot) QTL (29705) | 9 | 1.01E+08 | 1.36E+08 |
| ENSSSCG00000015428 | 9 | 1.05E+08 | 1.05E+08 | Carcass length QTL (29706) | 9 | 1.01E+08 | 1.36E+08 |
| ENSSSCG00000015428 | 9 | 1.05E+08 | 1.05E+08 | Capric acid content QTL (156944) | 9 | 1.01E+08 | 1.36E+08 |
| ENSSSCG00000015428 | 9 | 1.05E+08 | 1.05E+08 | Palmitoleic acid content QTL (156945) | 9 | 1.01E+08 | 1.36E+08 |
| ENSSSCG00000015428 | 9 | 1.05E+08 | 1.05E+08 | Palmitic acid content QTL (156883) | 9 | 1.01E+08 | 1.36E+08 |
| ENSSSCG00000015428 | 9 | 1.05E+08 | 1.05E+08 | Arachidic acid content QTL (156885) | 9 | 1.01E+08 | 1.36E+08 |
| ENSSSCG00000015428 | 9 | 1.05E+08 | 1.05E+08 | Saturated fatty acid content QTL (156886) | 9 | 1.01E+08 | 1.36E+08 |
| ENSSSCG00000015428 | 9 | 1.05E+08 | 1.05E+08 | Unsaturated fatty acid content QTL (156887) | 9 | 1.01E+08 | 1.36E+08 |
| ENSSSCG00000015569 | 9 | 1.26E+08 | 1.26E+08 | Distance from eye to corner of mouth QTL (153245) | 9 | 1.26E+08 | 1.26E+08 |
| ENSSSCG00000015569 | 9 | 1.26E+08 | 1.26E+08 | Distance from eye to corner of mouth QTL (153246) | 9 | 1.26E+08 | 1.26E+08 |
| ENSSSCG00000015569 | 9 | 1.26E+08 | 1.26E+08 | Meat color-b QTL (3275) | 9 | 93987072 | 1.27E+08 |
| ENSSSCG00000015569 | 9 | 1.26E+08 | 1.26E+08 | Carcass temperature (45 minutes post-mortem) QTL (3310) | 9 | 93987072 | 1.27E+08 |
| ENSSSCG00000015569 | 9 | 1.26E+08 | 1.26E+08 | backfat above muscle dorsi QTL (5205) | 9 | 3916128 | 1.36E+08 |
| ENSSSCG00000015569 | 9 | 1.26E+08 | 1.26E+08 | Muscle moisture percentage QTL (38097) | 9 | 6363708 | 1.36E+08 |
| ENSSSCG00000015569 | 9 | 1.26E+08 | 1.26E+08 | Meat color a* QTL (38098) | 9 | 6363708 | 1.36E+08 |
| ENSSSCG00000015569 | 9 | 1.26E+08 | 1.26E+08 | Meat color L* QTL (38099) | 9 | 6363708 | 1.36E+08 |
| ENSSSCG00000015569 | 9 | 1.26E+08 | 1.26E+08 | Backfat at first rib QTL (21261) | 9 | 55804824 | 1.3E+08 |
| ENSSSCG00000015569 | 9 | 1.26E+08 | 1.26E+08 | Average backfat thickness QTL (21258) | 9 | 56294340 | 1.31E+08 |
| ENSSSCG00000015569 | 9 | 1.26E+08 | 1.26E+08 | Diameter of type IIa muscle fibers QTL (7017) | 9 | 1.01E+08 | 1.4E+08 |
| ENSSSCG00000015569 | 9 | 1.26E+08 | 1.26E+08 | Potassium level QTL (6382) | 9 | 1.01E+08 | 1.36E+08 |
| ENSSSCG00000015569 | 9 | 1.26E+08 | 1.26E+08 | body weight (10 weeks) QTL (29704) | 9 | 1.01E+08 | 1.36E+08 |
| ENSSSCG00000015569 | 9 | 1.26E+08 | 1.26E+08 | Carcass weight (hot) QTL (29705) | 9 | 1.01E+08 | 1.36E+08 |
| ENSSSCG00000015569 | 9 | 1.26E+08 | 1.26E+08 | Carcass length QTL (29706) | 9 | 1.01E+08 | 1.36E+08 |
| ENSSSCG00000015569 | 9 | 1.26E+08 | 1.26E+08 | Capric acid content QTL (156944) | 9 | 1.01E+08 | 1.36E+08 |
| ENSSSCG00000015569 | 9 | 1.26E+08 | 1.26E+08 | Palmitoleic acid content QTL (156945) | 9 | 1.01E+08 | 1.36E+08 |
| ENSSSCG00000015569 | 9 | 1.26E+08 | 1.26E+08 | Palmitic acid content QTL (156883) | 9 | 1.01E+08 | 1.36E+08 |
| ENSSSCG00000015569 | 9 | 1.26E+08 | 1.26E+08 | Arachidic acid content QTL (156885) | 9 | 1.01E+08 | 1.36E+08 |
| ENSSSCG00000015569 | 9 | 1.26E+08 | 1.26E+08 | Saturated fatty acid content QTL (156886) | 9 | 1.01E+08 | 1.36E+08 |
| ENSSSCG00000015569 | 9 | 1.26E+08 | 1.26E+08 | Unsaturated fatty acid content QTL (156887) | 9 | 1.01E+08 | 1.36E+08 |
| ENSSSCG00000015569 | 9 | 1.26E+08 | 1.26E+08 | Interleukin-10 level QTL (12269) | 9 | 1.2E+08 | 1.36E+08 |
| ENSSSCG00000015569 | 9 | 1.26E+08 | 1.26E+08 | Creatine kinase level QTL (15110) | 9 | 1.22E+08 | 1.42E+08 |
| ENSSSCG00000015589 | 9 | 1.3E+08 | 1.31E+08 | backfat above muscle dorsi QTL (5205) | 9 | 3916128 | 1.36E+08 |
| ENSSSCG00000015589 | 9 | 1.3E+08 | 1.31E+08 | Muscle moisture percentage QTL (38097) | 9 | 6363708 | 1.36E+08 |
| ENSSSCG00000015589 | 9 | 1.3E+08 | 1.31E+08 | Meat color a* QTL (38098) | 9 | 6363708 | 1.36E+08 |
| ENSSSCG00000015589 | 9 | 1.3E+08 | 1.31E+08 | Meat color L* QTL (38099) | 9 | 6363708 | 1.36E+08 |
| ENSSSCG00000015589 | 9 | 1.3E+08 | 1.31E+08 | Average backfat thickness QTL (21258) | 9 | 56294340 | 1.31E+08 |
| ENSSSCG00000015589 | 9 | 1.3E+08 | 1.31E+08 | Diameter of type IIa muscle fibers QTL (7017) | 9 | 1.01E+08 | 1.4E+08 |
| ENSSSCG00000015589 | 9 | 1.3E+08 | 1.31E+08 | Potassium level QTL (6382) | 9 | 1.01E+08 | 1.36E+08 |
| ENSSSCG00000015589 | 9 | 1.3E+08 | 1.31E+08 | body weight (10 weeks) QTL (29704) | 9 | 1.01E+08 | 1.36E+08 |
| ENSSSCG00000015589 | 9 | 1.3E+08 | 1.31E+08 | Carcass weight (hot) QTL (29705) | 9 | 1.01E+08 | 1.36E+08 |
| ENSSSCG00000015589 | 9 | 1.3E+08 | 1.31E+08 | Carcass length QTL (29706) | 9 | 1.01E+08 | 1.36E+08 |
| ENSSSCG00000015589 | 9 | 1.3E+08 | 1.31E+08 | Capric acid content QTL (156944) | 9 | 1.01E+08 | 1.36E+08 |
| ENSSSCG00000015589 | 9 | 1.3E+08 | 1.31E+08 | Palmitoleic acid content QTL (156945) | 9 | 1.01E+08 | 1.36E+08 |
| ENSSSCG00000015589 | 9 | 1.3E+08 | 1.31E+08 | Palmitic acid content QTL (156883) | 9 | 1.01E+08 | 1.36E+08 |
| ENSSSCG00000015589 | 9 | 1.3E+08 | 1.31E+08 | Arachidic acid content QTL (156885) | 9 | 1.01E+08 | 1.36E+08 |
| ENSSSCG00000015589 | 9 | 1.3E+08 | 1.31E+08 | Saturated fatty acid content QTL (156886) | 9 | 1.01E+08 | 1.36E+08 |
| ENSSSCG00000015589 | 9 | 1.3E+08 | 1.31E+08 | Unsaturated fatty acid content QTL (156887) | 9 | 1.01E+08 | 1.36E+08 |
| ENSSSCG00000015589 | 9 | 1.3E+08 | 1.31E+08 | Interleukin-10 level QTL (12269) | 9 | 1.2E+08 | 1.36E+08 |
| ENSSSCG00000015589 | 9 | 1.3E+08 | 1.31E+08 | Creatine kinase level QTL (15110) | 9 | 1.22E+08 | 1.42E+08 |
| ENSSSCG00000028293 | 9 | 1.32E+08 | 1.32E+08 | backfat above muscle dorsi QTL (5205) | 9 | 3916128 | 1.36E+08 |
| ENSSSCG00000028293 | 9 | 1.32E+08 | 1.32E+08 | Muscle moisture percentage QTL (38097) | 9 | 6363708 | 1.36E+08 |
| ENSSSCG00000028293 | 9 | 1.32E+08 | 1.32E+08 | Meat color a* QTL (38098) | 9 | 6363708 | 1.36E+08 |
| ENSSSCG00000028293 | 9 | 1.32E+08 | 1.32E+08 | Meat color L* QTL (38099) | 9 | 6363708 | 1.36E+08 |
| ENSSSCG00000028293 | 9 | 1.32E+08 | 1.32E+08 | Diameter of type IIa muscle fibers QTL (7017) | 9 | 1.01E+08 | 1.4E+08 |
| ENSSSCG00000028293 | 9 | 1.32E+08 | 1.32E+08 | Potassium level QTL (6382) | 9 | 1.01E+08 | 1.36E+08 |
| ENSSSCG00000028293 | 9 | 1.32E+08 | 1.32E+08 | body weight (10 weeks) QTL (29704) | 9 | 1.01E+08 | 1.36E+08 |
| ENSSSCG00000028293 | 9 | 1.32E+08 | 1.32E+08 | Carcass weight (hot) QTL (29705) | 9 | 1.01E+08 | 1.36E+08 |
| ENSSSCG00000028293 | 9 | 1.32E+08 | 1.32E+08 | Carcass length QTL (29706) | 9 | 1.01E+08 | 1.36E+08 |
| ENSSSCG00000028293 | 9 | 1.32E+08 | 1.32E+08 | Capric acid content QTL (156944) | 9 | 1.01E+08 | 1.36E+08 |
| ENSSSCG00000028293 | 9 | 1.32E+08 | 1.32E+08 | Palmitoleic acid content QTL (156945) | 9 | 1.01E+08 | 1.36E+08 |
| ENSSSCG00000028293 | 9 | 1.32E+08 | 1.32E+08 | Palmitic acid content QTL (156883) | 9 | 1.01E+08 | 1.36E+08 |
| ENSSSCG00000028293 | 9 | 1.32E+08 | 1.32E+08 | Arachidic acid content QTL (156885) | 9 | 1.01E+08 | 1.36E+08 |
| ENSSSCG00000028293 | 9 | 1.32E+08 | 1.32E+08 | Saturated fatty acid content QTL (156886) | 9 | 1.01E+08 | 1.36E+08 |
| ENSSSCG00000028293 | 9 | 1.32E+08 | 1.32E+08 | Unsaturated fatty acid content QTL (156887) | 9 | 1.01E+08 | 1.36E+08 |
| ENSSSCG00000028293 | 9 | 1.32E+08 | 1.32E+08 | Interleukin-10 level QTL (12269) | 9 | 1.2E+08 | 1.36E+08 |
| ENSSSCG00000028293 | 9 | 1.32E+08 | 1.32E+08 | Creatine kinase level QTL (15110) | 9 | 1.22E+08 | 1.42E+08 |
| ENSSSCG00000022240 | X | 20756201 | 21102061 | Loin muscle depth QTL (400) | X | 3148485 | 48486669 |
| ENSSSCG00000022240 | X | 20756201 | 21102061 | Meat color b* QTL (3932) | X | 7493394 | 28840122 |
| ENSSSCG00000022240 | X | 20756201 | 21102061 | Carcass length QTL (1217) | X | 7493394 | 22165334 |
| ENSSSCG00000022240 | X | 20756201 | 21102061 | Average daily gain QTL (1218) | X | 7493394 | 22165334 |
| ENSSSCG00000022240 | X | 20756201 | 21102061 | Fat protein content QTL (12792) | X | 7493394 | 22165334 |
| ENSSSCG00000022240 | X | 20756201 | 21102061 | NADPH-generating enzyme activity QTL (12796) | X | 7493394 | 22165334 |
| ENSSSCG00000022240 | X | 20756201 | 21102061 | NADPH-generating enzyme activity QTL (12801) | X | 7493394 | 22165334 |
| ENSSSCG00000023759 | X | 42722785 | 42730199 | Backfat at last rib QTL (24300) | X | 42252668 | 48297759 |
| ENSSSCG00000023759 | X | 42722785 | 42730199 | Loin muscle depth QTL (400) | X | 3148485 | 48486669 |
| ENSSSCG00000023759 | X | 42722785 | 42730199 | Carcass length QTL (1207) | X | 22165334 | 46849456 |
| ENSSSCG00000023759 | X | 42722785 | 42730199 | Average backfat thickness QTL (212) | X | 34633335 | 46597578 |
| ENSSSCG00000023759 | X | 42722785 | 42730199 | Average backfat thickness QTL (213) | X | 34633335 | 46597578 |
| ENSSSCG00000023759 | X | 42722785 | 42730199 | Average backfat thickness QTL (214) | X | 34633335 | 46597578 |
| ENSSSCG00000023759 | X | 42722785 | 42730199 | Average backfat thickness QTL (215) | X | 34633335 | 46597578 |
| ENSSSCG00000023759 | X | 42722785 | 42730199 | Average backfat thickness QTL (216) | X | 34633335 | 46597578 |
| ENSSSCG00000023759 | X | 42722785 | 42730199 | Average backfat thickness QTL (142) | X | 34885213 | 55035517 |
| ENSSSCG00000023759 | X | 42722785 | 42730199 | Intramuscular fat content QTL (143) | X | 34885213 | 55035517 |
| ENSSSCG00000023759 | X | 42722785 | 42730199 | Backfat at last rib QTL (1239) | X | 34885213 | 55035517 |
| ENSSSCG00000023759 | X | 42722785 | 42730199 | Muscle moisture percentage QTL (17873) | X | 34885213 | 55035517 |
| ENSSSCG00000023759 | X | 42722785 | 42730199 | Intramuscular fat content QTL (17874) | X | 34885213 | 55035517 |
| ENSSSCG00000023759 | X | 42722785 | 42730199 | Average glycolytic potential QTL (17875) | X | 34885213 | 55035517 |
| ENSSSCG00000023759 | X | 42722785 | 42730199 | Average glycogen QTL (17876) | X | 34885213 | 55035517 |
| ENSSSCG00000023759 | X | 42722785 | 42730199 | Average backfat thickness QTL (355) | X | 34885213 | 48864487 |
| ENSSSCG00000023759 | X | 42722785 | 42730199 | Cooling loss QTL (1241) | X | 34885213 | 46849456 |
| ENSSSCG00000023759 | X | 42722785 | 42730199 | Shoulder subcutaneous fat thickness QTL (5511) | X | 35074122 | 54594729 |
| ENSSSCG00000023759 | X | 42722785 | 42730199 | Shoulder subcutaneous fat thickness QTL (24298) | X | 37844789 | 46093820 |
| ENSSSCG00000023759 | X | 42722785 | 42730199 | Intramuscular fat content QTL (24304) | X | 37844789 | 45527093 |
| ENSSSCG00000023759 | X | 42722785 | 42730199 | Backfat between 6th and 7th ribs QTL (24299) | X | 38411517 | 44960365 |
| ENSSSCG00000023759 | X | 42722785 | 42730199 | Average backfat thickness QTL (24302) | X | 38978244 | 43323153 |
| ENSSSCG00000023759 | X | 42722785 | 42730199 | Backfat at rump QTL (24317) | X | 40048729 | 44456608 |
| ENSSSCG00000023759 | X | 42722785 | 42730199 | Backfat at rump QTL (24301) | X | 40048729 | 43323153 |
| ENSSSCG00000023759 | X | 42722785 | 42730199 | Shoulder subcutaneous fat thickness QTL (21278) | X | 41497032 | 49557153 |
| ENSSSCG00000023759 | X | 42722785 | 42730199 | Loin muscle area QTL (24316) | X | 41685941 | 44960365 |
| ENSSSCG00000023759 | X | 42722785 | 42730199 | Loin muscle area QTL (24305) | X | 41685941 | 43323153 |
| ENSSSCG00000023759 | X | 42722785 | 42730199 | Loin muscle area QTL (24319) | X | 41685941 | 43323153 |
| ENSSSCG00000023759 | X | 42722785 | 42730199 | NADPH-generating enzyme activity QTL (12797) | X | 22165334 | 67881336 |
| ENSSSCG00000023759 | X | 42722785 | 42730199 | NADPH-generating enzyme activity QTL (12800) | X | 22165334 | 67881336 |
| ENSSSCG00000023759 | X | 42722785 | 42730199 | Carcass length QTL (12) | X | 22669092 | 86898186 |
| ENSSSCG00000023759 | X | 42722785 | 42730199 | Average backfat thickness QTL (38) | X | 41371092 | 86898186 |
| ENSSSCG00000023759 | X | 42722785 | 42730199 | Backfat at tenth rib QTL (26) | X | 41371092 | 80853094 |
| ENSSSCG00000023759 | X | 42722785 | 42730199 | Backfat at last rib QTL (29) | X | 41371092 | 80853094 |
| ENSSSCG00000024814 | X | 47759307 | 47859078 | Backfat at last rib QTL (24300) | X | 42252668 | 48297759 |
| ENSSSCG00000024814 | X | 47759307 | 47859078 | backfat between the last 3rd and 4th lumbar QTL (24306) | X | 43323153 | 49368244 |
| ENSSSCG00000024814 | X | 47759307 | 47859078 | Backfat at last rib QTL (24307) | X | 43323153 | 49368244 |
| ENSSSCG00000024814 | X | 47759307 | 47859078 | Average backfat thickness QTL (24308) | X | 43323153 | 49368244 |
| ENSSSCG00000024814 | X | 47759307 | 47859078 | Average backfat thickness QTL (24322) | X | 43889880 | 49934972 |
| ENSSSCG00000024814 | X | 47759307 | 47859078 | Average backfat thickness QTL (21274) | X | 45527093 | 48171820 |
| ENSSSCG00000024814 | X | 47759307 | 47859078 | Backfat at rump QTL (21276) | X | 45527093 | 48171820 |
| ENSSSCG00000024814 | X | 47759307 | 47859078 | Loin muscle depth QTL (400) | X | 3148485 | 48486669 |
| ENSSSCG00000024814 | X | 47759307 | 47859078 | Average backfat thickness QTL (142) | X | 34885213 | 55035517 |
| ENSSSCG00000024814 | X | 47759307 | 47859078 | Intramuscular fat content QTL (143) | X | 34885213 | 55035517 |
| ENSSSCG00000024814 | X | 47759307 | 47859078 | Backfat at last rib QTL (1239) | X | 34885213 | 55035517 |
| ENSSSCG00000024814 | X | 47759307 | 47859078 | Muscle moisture percentage QTL (17873) | X | 34885213 | 55035517 |
| ENSSSCG00000024814 | X | 47759307 | 47859078 | Intramuscular fat content QTL (17874) | X | 34885213 | 55035517 |
| ENSSSCG00000024814 | X | 47759307 | 47859078 | Average glycolytic potential QTL (17875) | X | 34885213 | 55035517 |
| ENSSSCG00000024814 | X | 47759307 | 47859078 | Average glycogen QTL (17876) | X | 34885213 | 55035517 |
| ENSSSCG00000024814 | X | 47759307 | 47859078 | Average backfat thickness QTL (355) | X | 34885213 | 48864487 |
| ENSSSCG00000024814 | X | 47759307 | 47859078 | Shoulder subcutaneous fat thickness QTL (5511) | X | 35074122 | 54594729 |
| ENSSSCG00000024814 | X | 47759307 | 47859078 | Shoulder subcutaneous fat thickness QTL (21278) | X | 41497032 | 49557153 |
| ENSSSCG00000024814 | X | 47759307 | 47859078 | Intramuscular fat content QTL (24315) | X | 43323153 | 50501699 |
| ENSSSCG00000024814 | X | 47759307 | 47859078 | Backfat thickness between 3rd and 4th rib QTL (430) | X | 43449093 | 53524245 |
| ENSSSCG00000024814 | X | 47759307 | 47859078 | Intramuscular fat content QTL (24310) | X | 44960365 | 50501699 |
| ENSSSCG00000024814 | X | 47759307 | 47859078 | Plasma FSH concentration QTL (647) | X | 45149274 | 55035517 |
| ENSSSCG00000024814 | X | 47759307 | 47859078 | Average backfat thickness QTL (804) | X | 46849456 | 55035517 |
| ENSSSCG00000024814 | X | 47759307 | 47859078 | Backfat at last rib QTL (1227) | X | 46849456 | 55035517 |
| ENSSSCG00000024814 | X | 47759307 | 47859078 | Empty body lipid content QTL (6456) | X | 46849456 | 55035517 |
| ENSSSCG00000024814 | X | 47759307 | 47859078 | Empty body protein content QTL (6457) | X | 46849456 | 55035517 |
| ENSSSCG00000024814 | X | 47759307 | 47859078 | Empty body protein content QTL (6458) | X | 46849456 | 55035517 |
| ENSSSCG00000024814 | X | 47759307 | 47859078 | Backfat at last rib QTL (21277) | X | 46849456 | 52201881 |
| ENSSSCG00000024814 | X | 47759307 | 47859078 | Backfat at last rib QTL (1206) | X | 47479153 | 53650184 |
| ENSSSCG00000024814 | X | 47759307 | 47859078 | Backfat at last rib QTL (1212) | X | 47479153 | 53650184 |
| ENSSSCG00000024814 | X | 47759307 | 47859078 | Intramuscular fat content QTL (24318) | X | 47731032 | 50501699 |
| ENSSSCG00000024814 | X | 47759307 | 47859078 | NADPH-generating enzyme activity QTL (12797) | X | 22165334 | 67881336 |
| ENSSSCG00000024814 | X | 47759307 | 47859078 | NADPH-generating enzyme activity QTL (12800) | X | 22165334 | 67881336 |
| ENSSSCG00000024814 | X | 47759307 | 47859078 | Carcass length QTL (12) | X | 22669092 | 86898186 |
| ENSSSCG00000024814 | X | 47759307 | 47859078 | Average backfat thickness QTL (38) | X | 41371092 | 86898186 |
| ENSSSCG00000024814 | X | 47759307 | 47859078 | Backfat at tenth rib QTL (26) | X | 41371092 | 80853094 |
| ENSSSCG00000024814 | X | 47759307 | 47859078 | Backfat at last rib QTL (29) | X | 41371092 | 80853094 |
| ENSSSCG00000024814 | X | 47759307 | 47859078 | Backfat at first rib QTL (22) | X | 46849456 | 80853094 |
| ENSSSCG00000025156 | X | 64241620 | 64403844 | NADPH-generating enzyme activity QTL (12797) | X | 22165334 | 67881336 |
| ENSSSCG00000025156 | X | 64241620 | 64403844 | NADPH-generating enzyme activity QTL (12800) | X | 22165334 | 67881336 |
| ENSSSCG00000025156 | X | 64241620 | 64403844 | Carcass length QTL (12) | X | 22669092 | 86898186 |
| ENSSSCG00000025156 | X | 64241620 | 64403844 | Average backfat thickness QTL (38) | X | 41371092 | 86898186 |
| ENSSSCG00000025156 | X | 64241620 | 64403844 | Backfat at tenth rib QTL (26) | X | 41371092 | 80853094 |
| ENSSSCG00000025156 | X | 64241620 | 64403844 | Backfat at last rib QTL (29) | X | 41371092 | 80853094 |
| ENSSSCG00000025156 | X | 64241620 | 64403844 | Backfat at first rib QTL (22) | X | 46849456 | 80853094 |
| ENSSSCG00000025156 | X | 64241620 | 64403844 | CO2 partial pressure QTL (6327) | X | 64166124 | 80853094 |
| ENSSSCG00000025156 | X | 64241620 | 64403844 | Muscle fat content QTL (3306) | X | 64166124 | 75122852 |
| ENSSSCG00000012459 | X | 68281840 | 68437243 | Carcass length QTL (12) | X | 22669092 | 86898186 |
| ENSSSCG00000012459 | X | 68281840 | 68437243 | Average backfat thickness QTL (38) | X | 41371092 | 86898186 |
| ENSSSCG00000012459 | X | 68281840 | 68437243 | Backfat at tenth rib QTL (26) | X | 41371092 | 80853094 |
| ENSSSCG00000012459 | X | 68281840 | 68437243 | Backfat at last rib QTL (29) | X | 41371092 | 80853094 |
| ENSSSCG00000012459 | X | 68281840 | 68437243 | Backfat at first rib QTL (22) | X | 46849456 | 80853094 |
| ENSSSCG00000012459 | X | 68281840 | 68437243 | CO2 partial pressure QTL (6327) | X | 64166124 | 80853094 |
| ENSSSCG00000012459 | X | 68281840 | 68437243 | Muscle fat content QTL (3306) | X | 64166124 | 75122852 |

Supplementary Table 10 of circRNAs

| gene_id | source_gene | chr | strand | genomic_start | genomic_end | spliced_length | annot_type |
| --- | --- | --- | --- | --- | --- | --- | --- |
| novel_circ_000001 | ENSSSCG00000003129 | 6 | + | 53671467 | 53671779 | 313 | one_exon |
| novel_circ_000002 | ENSSSCG00000026569 | 3 | - | 32116491 | 32121574 | 343 | annot_exons |
| novel_circ_000003 | ENSSSCG00000002528 | AEMK02000452.1 | + | 1186708 | 1187633 | 261 | annot_exons |
| novel_circ_000004 | ENSSSCG00000011136 | 10 | - | 65244719 | 65247076 | 597 | annot_exons |
| novel_circ_000005 | ENSSSCG00000027725 | 5 | + | 69276149 | 69281403 | 280 | annot_exons |
| novel_circ_000006 | ENSSSCG00000023738 | 11 | - | 21778941 | 21786602 | 560 | annot_exons |
| novel_circ_000007 | ENSSSCG00000020879 | 8 | + | 76607805 | 76609327 | 614 | annot_exons |
| novel_circ_000008 | ENSSSCG00000006196 | 4 | + | 65502957 | 65524693 | 516 | annot_exons |
| novel_circ_000009 | ENSSSCG00000044599 | X | - | 59282807 | 59286175 | 1955 | exon_intron |
| novel_circ_000010 | ENSSSCG00000033301 | 14 | - | 50591763 | 50592987 | 631 | annot_exons |
| novel_circ_000011 | ENSSSCG00000008335 | 3 | - | 72580460 | 72584762 | 222 | annot_exons |
| novel_circ_000012 | ENSSSCG00000004802 | 1 | + | 1.36E+08 | 1.36E+08 | 289 | annot_exons |
| novel_circ_000013 | ENSSSCG00000003700 | 6 | - | 1.07E+08 | 1.07E+08 | 2662 | annot_exons |
| novel_circ_000014 | ENSSSCG00000040887 | 6 | - | 34743079 | 34755027 | 642 | annot_exons |
| novel_circ_000015 | ENSSSCG00000024373 | 15 | - | 1.31E+08 | 1.31E+08 | 1491 | annot_exons |
| novel_circ_000016 | ENSSSCG00000029578 | 6 | + | 28711467 | 28714979 | 1298 | annot_exons |
| novel_circ_000017 | ENSSSCG00000009658 | 14 | + | 10325118 | 10340141 | 1458 | annot_exons |
| novel_circ_000018 | ENSSSCG00000016127 | 15 | - | 1.09E+08 | 1.09E+08 | 251 | annot_exons |
| novel_circ_000019 | ENSSSCG00000011236 | 13 | - | 19327525 | 19348052 | 549 | annot_exons |
| novel_circ_000020 | ENSSSCG00000015273 | 9 | + | 64407086 | 64431528 | 2850 | exon_intron |
| novel_circ_000021 | ENSSSCG00000014163 | 2 | + | 1.01E+08 | 1.01E+08 | 594 | annot_exons |
| novel_circ_000022 | ENSSSCG00000008689 | 8 | - | 1233477 | 1233849 | 373 | intronic |
| novel_circ_000023 | ENSSSCG00000010566 | 14 | - | 1.13E+08 | 1.13E+08 | 414 | annot_exons |
| novel_circ_000024 | ENSSSCG00000017310 | 12 | + | 17059589 | 17060678 | 306 | annot_exons |
| novel_circ_000025 | ENSSSCG00000027628 | 4 | - | 95352771 | 95359433 | 864 | annot_exons |
| novel_circ_000026 | ENSSSCG00000015976 | 15 | - | 81723793 | 81734144 | 3287 | annot_exons |
| novel_circ_000027 | ENSSSCG00000012434 | X | - | 61728504 | 61735982 | 405 | annot_exons |
| novel_circ_000028 | ENSSSCG00000046408 | X | - | 29578514 | 29594284 | 15771 | intronic |
| novel_circ_000029 | ENSSSCG00000031981 | 10 | - | 24161030 | 24161393 | 364 | antisense |
| novel_circ_000030 | ENSSSCG00000004535 | 1 | - | 1.05E+08 | 1.05E+08 | 373 | annot_exons |
| novel_circ_000031 | ENSSSCG00000024674 | 9 | - | 1.21E+08 | 1.21E+08 | 358 | annot_exons |
| novel_circ_000032 | ENSSSCG00000010652 | 14 | + | 1.25E+08 | 1.25E+08 | 1902 | annot_exons |
| novel_circ_000033 | ENSSSCG00000012686 | X | - | 1.11E+08 | 1.11E+08 | 1240 | intronic |
| novel_circ_000034 | ENSSSCG00000003461 | 6 | + | 74829110 | 74838414 | 364 | annot_exons |
| novel_circ_000035 | ENSSSCG00000008753 | 8 | - | 16726255 | 16743600 | 591 | annot_exons |
| novel_circ_000036 | ENSSSCG00000039896 | 1 | + | 2.47E+08 | 2.47E+08 | 430 | annot_exons |
| novel_circ_000037 | ENSSSCG00000003401 | 6 | + | 70613742 | 70624600 | 592 | annot_exons |
| novel_circ_000038 | ENSSSCG00000048856 | 2 | - | 6754805 | 6755333 | 529 | one_exon |
| novel_circ_000039 | ENSSSCG00000029602 | 10 | - | 68491183 | 68518239 | 1396 | annot_exons |
| novel_circ_000040 | ENSSSCG00000036132 | 6 | - | 51710219 | 51711185 | 288 | annot_exons |
| novel_circ_000041 | ENSSSCG00000011025 | 10 | + | 42063516 | 42147610 | 84095 | exon_intron |
| novel_circ_000042 | ENSSSCG00000016177 | 15 | + | 1.18E+08 | 1.18E+08 | 164 | annot_exons |
| novel_circ_000043 | ENSSSCG00000025120 | 11 | + | 45034287 | 45035369 | 342 | annot_exons |
| novel_circ_000044 | ENSSSCG00000010239 | 14 | - | 71521813 | 71524495 | 1926 | annot_exons |
| novel_circ_000045 | ENSSSCG00000004966 | 1 | + | 1.67E+08 | 1.67E+08 | 462 | annot_exons |
| novel_circ_000046 | ENSSSCG00000001068 | 7 | + | 13208015 | 13262713 | 4173 | exon_intron |
| novel_circ_000047 | ENSSSCG00000000553 | 5 | + | 46749902 | 46757889 | 3391 | exon_intron |
| novel_circ_000048 | ENSSSCG00000035196 | 4 | - | 1.25E+08 | 1.25E+08 | 703 | one_exon |
| novel_circ_000049 | ENSSSCG00000021793 | 8 | - | 30802791 | 30803058 | 268 | intronic |
| novel_circ_000050 | ENSSSCG00000015857 | 15 | - | 59162535 | 59165600 | 581 | annot_exons |
| novel_circ_000051 | ENSSSCG00000029538 | 9 | - | 39503890 | 39511891 | 625 | annot_exons |
| novel_circ_000052 | ENSSSCG00000015777 | 15 | + | 45033347 | 45035364 | 302 | annot_exons |
| novel_circ_000053 | ENSSSCG00000006024 | 4 | - | 27997979 | 27998967 | 238 | annot_exons |
| novel_circ_000054 | ENSSSCG00000028327 | 14 | - | 64335420 | 64345385 | 1250 | annot_exons |
| novel_circ_000055 | ENSSSCG00000014084 | 2 | - | 84709320 | 84730446 | 5352 | annot_exons |
| novel_circ_000056 | NA | 15 | + | 84310817 | 84311259 | 443 | intergenic |
| novel_circ_000057 | ENSSSCG00000014960 | 9 | + | 26904866 | 26962087 | 1951 | annot_exons |
| novel_circ_000058 | ENSSSCG00000026502 | 4 | + | 99399286 | 99400078 | 174 | annot_exons |
| novel_circ_000059 | ENSSSCG00000007839 | 3 | - | 23910594 | 23911080 | 324 | annot_exons |
| novel_circ_000060 | ENSSSCG00000004351 | 1 | - | 66420083 | 66425204 | 370 | annot_exons |
| novel_circ_000061 | ENSSSCG00000027865 | 7 | - | 1.1E+08 | 1.1E+08 | 332 | annot_exons |
| novel_circ_000062 | ENSSSCG00000027349 | 8 | - | 3918043 | 3918783 | 741 | one_exon |
| novel_circ_000063 | ENSSSCG00000023324 | 9 | + | 45784065 | 45785898 | 584 | annot_exons |
| novel_circ_000064 | ENSSSCG00000014868 | 9 | + | 10636306 | 10647784 | 806 | annot_exons |
| novel_circ_000065 | ENSSSCG00000007460 | 17 | + | 50688119 | 50695566 | 536 | annot_exons |
| novel_circ_000066 | ENSSSCG00000027403 | 8 | - | 55641841 | 55642319 | 396 | annot_exons |
| novel_circ_000067 | ENSSSCG00000044227 | 7 | - | 56259271 | 56261229 | 341 | annot_exons |
| novel_circ_000068 | ENSSSCG00000014430 | 2 | + | 1.5E+08 | 1.5E+08 | 660 | annot_exons |
| novel_circ_000069 | ENSSSCG00000021166 | 5 | - | 78055916 | 78056986 | 251 | annot_exons |
| novel_circ_000070 | ENSSSCG00000015823 | 15 | + | 48557959 | 48558336 | 378 | one_exon |
| novel_circ_000071 | ENSSSCG00000010190 | 14 | + | 60414244 | 60414636 | 271 | annot_exons |
| novel_circ_000072 | ENSSSCG00000040786 | 3 | + | 86623198 | 86623395 | 198 | antisense |
| novel_circ_000073 | ENSSSCG00000010211 | 14 | - | 63190562 | 63200604 | 419 | annot_exons |
| novel_circ_000074 | ENSSSCG00000025703 | 1 | - | 77263699 | 77267286 | 235 | annot_exons |
| novel_circ_000075 | ENSSSCG00000027722 | 8 | - | 1.39E+08 | 1.39E+08 | 987 | annot_exons |
| novel_circ_000076 | ENSSSCG00000029464 | 5 | - | 67686258 | 67688990 | 438 | annot_exons |
| novel_circ_000077 | ENSSSCG00000009334 | 11 | - | 7759074 | 7759263 | 53 | annot_exons |
| novel_circ_000078 | ENSSSCG00000008682 | 8 | + | 964857 | 968127 | 786 | annot_exons |
| novel_circ_000079 | ENSSSCG00000005282 | 1 | + | 2.3E+08 | 2.3E+08 | 299 | annot_exons |
| novel_circ_000080 | ENSSSCG00000000784 | 5 | + | 71874675 | 71877431 | 577 | annot_exons |
| novel_circ_000081 | ENSSSCG00000015360 | 9 | - | 85865687 | 85871670 | 260 | annot_exons |
| novel_circ_000082 | NA | 15 | - | 84284075 | 84284798 | 724 | intergenic |
| novel_circ_000083 | ENSSSCG00000000530 | 5 | - | 41684337 | 41686129 | 319 | annot_exons |
| novel_circ_000084 | ENSSSCG00000034122 | 8 | + | 6117161 | 6120917 | 301 | annot_exons |
| novel_circ_000085 | ENSSSCG00000017516 | 12 | - | 24174093 | 24180721 | 6629 | antisense |
| novel_circ_000086 | ENSSSCG00000014247 | 2 | - | 1.3E+08 | 1.3E+08 | 396 | annot_exons |
| novel_circ_000087 | ENSSSCG00000001487 | 7 | - | 27102625 | 27102839 | 215 | antisense |
| novel_circ_000088 | ENSSSCG00000004813 | 1 | - | 1.38E+08 | 1.38E+08 | 562 | annot_exons |
| novel_circ_000089 | ENSSSCG00000046809 | 6 | + | 47368797 | 47389662 | 20866 | exon_intron |
| novel_circ_000090 | ENSSSCG00000038708 | 7 | - | 48863003 | 48866128 | 3126 | exon_intron |
| novel_circ_000091 | ENSSSCG00000016118 | 15 | + | 1.07E+08 | 1.07E+08 | 304 | one_exon |
| novel_circ_000092 | NA | 15 | + | 84283252 | 84284781 | 1530 | intergenic |
| novel_circ_000093 | ENSSSCG00000037530 | 14 | + | 1.32E+08 | 1.32E+08 | 1258 | exon_intron |
| novel_circ_000094 | ENSSSCG00000036132 | 6 | + | 51704159 | 51704516 | 358 | antisense |
| novel_circ_000095 | ENSSSCG00000005247 | 1 | + | 2.23E+08 | 2.23E+08 | 258 | annot_exons |
| novel_circ_000096 | ENSSSCG00000021289 | 8 | - | 276025 | 280910 | 625 | annot_exons |
| novel_circ_000097 | ENSSSCG00000042402 | 9 | - | 1.02E+08 | 1.02E+08 | 4115 | exon_intron |
| novel_circ_000098 | ENSSSCG00000029760 | 7 | + | 1E+08 | 1E+08 | 292 | one_exon |
| novel_circ_000099 | ENSSSCG00000007058 | 17 | + | 18332208 | 18337608 | 473 | annot_exons |
| novel_circ_000100 | ENSSSCG00000011506 | 13 | - | 50487543 | 50500855 | 510 | annot_exons |
| novel_circ_000101 | ENSSSCG00000016720 | 18 | + | 48711071 | 48711251 | 181 | one_exon |
| novel_circ_000102 | ENSSSCG00000004701 | 1 | + | 1.28E+08 | 1.28E+08 | 1131 | annot_exons |
| novel_circ_000103 | ENSSSCG00000011765 | 13 | - | 1.18E+08 | 1.18E+08 | 1084 | antisense |
| novel_circ_000104 | ENSSSCG00000016409 | 18 | - | 1589912 | 1592709 | 424 | annot_exons |
| novel_circ_000105 | ENSSSCG00000001746 | 7 | - | 45663897 | 45686183 | 572 | annot_exons |
| novel_circ_000106 | ENSSSCG00000015410 | 9 | - | 1.02E+08 | 1.02E+08 | 331 | annot_exons |
| novel_circ_000107 | ENSSSCG00000003030 | 6 | - | 49647657 | 49647839 | 183 | one_exon |
| novel_circ_000108 | ENSSSCG00000038443 | 13 | + | 1.17E+08 | 1.17E+08 | 253 | annot_exons |
| novel_circ_000109 | ENSSSCG00000006231 | 4 | - | 72657070 | 72694320 | 2254 | annot_exons |
| novel_circ_000110 | ENSSSCG00000016834 | 16 | - | 21441787 | 21451895 | 885 | annot_exons |
| novel_circ_000111 | ENSSSCG00000014195 | 2 | + | 1.15E+08 | 1.15E+08 | 605 | annot_exons |
| novel_circ_000112 | ENSSSCG00000021977 | 13 | + | 18157143 | 18166692 | 490 | annot_exons |
| novel_circ_000113 | ENSSSCG00000018049 | 12 | - | 60370050 | 60376568 | 443 | annot_exons |
| novel_circ_000114 | ENSSSCG00000015313 | 9 | - | 72153385 | 72160471 | 407 | annot_exons |
| novel_circ_000115 | ENSSSCG00000015820 | 15 | + | 48184287 | 48192770 | 834 | annot_exons |
| novel_circ_000116 | ENSSSCG00000010092 | 14 | - | 50457842 | 50466908 | 300 | annot_exons |
| novel_circ_000117 | ENSSSCG00000010690 | 14 | + | 1.3E+08 | 1.3E+08 | 908 | exon_intron |
| novel_circ_000118 | ENSSSCG00000008513 | 3 | - | 1.07E+08 | 1.07E+08 | 380 | annot_exons |
| novel_circ_000119 | ENSSSCG00000014838 | 9 | - | 8707786 | 8716340 | 566 | annot_exons |
| novel_circ_000120 | ENSSSCG00000007757 | 3 | - | 17290672 | 17297683 | 955 | annot_exons |
| novel_circ_000121 | ENSSSCG00000010095 | 14 | + | 50541082 | 50541547 | 466 | one_exon |
| novel_circ_000122 | ENSSSCG00000025672 | 6 | - | 1.48E+08 | 1.48E+08 | 300 | annot_exons |
| novel_circ_000123 | ENSSSCG00000005269 | 1 | - | 2.28E+08 | 2.28E+08 | 234 | annot_exons |
| novel_circ_000124 | ENSSSCG00000016223 | 15 | + | 1.25E+08 | 1.25E+08 | 347 | annot_exons |
| novel_circ_000125 | ENSSSCG00000004934 | 1 | - | 1.63E+08 | 1.63E+08 | 471 | annot_exons |
| novel_circ_000126 | ENSSSCG00000007284 | 17 | - | 38164322 | 38173595 | 544 | annot_exons |
| novel_circ_000127 | ENSSSCG00000033356 | 3 | - | 7547774 | 7550124 | 262 | annot_exons |
| novel_circ_000128 | ENSSSCG00000014114 | 2 | - | 88457523 | 88458152 | 630 | antisense |
| novel_circ_000129 | ENSSSCG00000029606 | 15 | + | 1.04E+08 | 1.04E+08 | 297 | annot_exons |
| novel_circ_000130 | ENSSSCG00000008448 | 3 | + | 96154836 | 96156923 | 1317 | annot_exons |
| novel_circ_000131 | ENSSSCG00000003859 | 6 | + | 1.6E+08 | 1.6E+08 | 1093 | exon_intron |
| novel_circ_000132 | ENSSSCG00000021042 | 13 | - | 2.03E+08 | 2.03E+08 | 394 | annot_exons |
| novel_circ_000133 | ENSSSCG00000004387 | 1 | + | 74737037 | 74738472 | 1436 | one_exon |
| novel_circ_000134 | ENSSSCG00000014211 | 2 | + | 1.18E+08 | 1.18E+08 | 604 | annot_exons |
| novel_circ_000135 | ENSSSCG00000050244 | 12 | + | 59273100 | 59320524 | 915 | exon_intron |
| novel_circ_000136 | ENSSSCG00000009405 | 11 | + | 19629537 | 19630304 | 263 | annot_exons |
| novel_circ_000137 | ENSSSCG00000007034 | 17 | + | 13008092 | 13031589 | 538 | annot_exons |
| novel_circ_000138 | ENSSSCG00000008105 | 3 | + | 44522989 | 44535936 | 1108 | annot_exons |
| novel_circ_000139 | ENSSSCG00000029030 | 6 | - | 1.04E+08 | 1.04E+08 | 502 | annot_exons |
| novel_circ_000140 | ENSSSCG00000004814 | 1 | + | 1.38E+08 | 1.38E+08 | 239 | annot_exons |
| novel_circ_000141 | ENSSSCG00000023992 | 12 | - | 12397533 | 12402130 | 252 | annot_exons |
| novel_circ_000142 | ENSSSCG00000014416 | 2 | + | 1.48E+08 | 1.48E+08 | 511 | annot_exons |
| novel_circ_000143 | NA | 11 | + | 71224938 | 71243879 | 18942 | intergenic |
| novel_circ_000144 | ENSSSCG00000015358 | 9 | - | 85697922 | 85698198 | 277 | one_exon |
| novel_circ_000145 | ENSSSCG00000016877 | 16 | + | 28162460 | 28182377 | 1017 | annot_exons |
| novel_circ_000146 | ENSSSCG00000004351 | 1 | - | 66424606 | 66425204 | 239 | annot_exons |
| novel_circ_000147 | ENSSSCG00000004351 | 1 | - | 66398761 | 66399433 | 673 | one_exon |
| novel_circ_000148 | ENSSSCG00000006766 | 4 | + | 1.07E+08 | 1.07E+08 | 938 | annot_exons |
| novel_circ_000149 | ENSSSCG00000035824 | 13 | - | 34713489 | 34716678 | 304 | annot_exons |
| novel_circ_000150 | ENSSSCG00000034569 | 2 | + | 51442532 | 51442865 | 245 | annot_exons |
| novel_circ_000151 | ENSSSCG00000007805 | 3 | + | 18564982 | 18565214 | 233 | one_exon |
| novel_circ_000152 | ENSSSCG00000005706 | 1 | + | 2.71E+08 | 2.71E+08 | 1344 | annot_exons |
| novel_circ_000153 | ENSSSCG00000025675 | 2 | + | 74752347 | 74752520 | 174 | one_exon |
| novel_circ_000154 | ENSSSCG00000009007 | 8 | - | 75484513 | 75505017 | 1047 | annot_exons |
| novel_circ_000155 | ENSSSCG00000004524 | 1 | + | 1.01E+08 | 1.01E+08 | 854 | annot_exons |
| novel_circ_000156 | ENSSSCG00000008826 | 8 | - | 38421716 | 38425519 | 459 | annot_exons |
| novel_circ_000157 | ENSSSCG00000011045 | 10 | + | 46002429 | 46022353 | 340 | annot_exons |
| novel_circ_000158 | ENSSSCG00000000104 | 5 | + | 9493241 | 9494173 | 476 | annot_exons |
| novel_circ_000159 | ENSSSCG00000013919 | 2 | + | 58915719 | 58916432 | 367 | annot_exons |
| novel_circ_000160 | NA | 15 | - | 84276388 | 84276807 | 420 | intergenic |
| novel_circ_000161 | ENSSSCG00000001975 | 7 | + | 69696389 | 69717922 | 21534 | exon_intron |
| novel_circ_000162 | ENSSSCG00000004293 | 1 | - | 54528562 | 54529700 | 277 | annot_exons |
| novel_circ_000163 | ENSSSCG00000017255 | 12 | + | 11098684 | 11145774 | 4108 | annot_exons |
| novel_circ_000164 | ENSSSCG00000009864 | 14 | + | 36312060 | 36323544 | 798 | annot_exons |
| novel_circ_000165 | ENSSSCG00000004636 | 1 | + | 1.21E+08 | 1.21E+08 | 1302 | annot_exons |
| novel_circ_000166 | ENSSSCG00000036926 | 1 | + | 2.67E+08 | 2.67E+08 | 11686 | exon_intron |
| novel_circ_000167 | ENSSSCG00000042985 | 6 | + | 47424162 | 47428931 | 3061 | exon_intron |
| novel_circ_000168 | ENSSSCG00000032622 | 14 | + | 6754236 | 6771753 | 884 | annot_exons |
| novel_circ_000169 | ENSSSCG00000012434 | X | - | 61688015 | 61691522 | 432 | annot_exons |
| novel_circ_000170 | ENSSSCG00000027865 | 7 | - | 1.1E+08 | 1.1E+08 | 310 | annot_exons |
| novel_circ_000171 | ENSSSCG00000004717 | 1 | + | 1.28E+08 | 1.28E+08 | 229 | annot_exons |
| novel_circ_000172 | ENSSSCG00000005955 | 4 | - | 9102988 | 9104901 | 279 | annot_exons |
| novel_circ_000173 | ENSSSCG00000022112 | 11 | - | 67895181 | 67925050 | 606 | annot_exons |
| novel_circ_000174 | ENSSSCG00000035671 | 12 | - | 61283968 | 61286652 | 386 | annot_exons |
| novel_circ_000175 | ENSSSCG00000014401 | 2 | - | 1.45E+08 | 1.45E+08 | 878 | intronic |
| novel_circ_000176 | ENSSSCG00000049107 | 13 | + | 42749150 | 42749419 | 270 | antisense |
| novel_circ_000177 | ENSSSCG00000005065 | 1 | + | 1.86E+08 | 1.86E+08 | 345 | annot_exons |
| novel_circ_000178 | ENSSSCG00000032740 | 9 | + | 81849680 | 81855775 | 312 | annot_exons |
| novel_circ_000179 | ENSSSCG00000006282 | 4 | + | 80973985 | 80978012 | 361 | annot_exons |
| novel_circ_000180 | ENSSSCG00000027659 | 6 | - | 81641858 | 81647305 | 445 | annot_exons |
| novel_circ_000181 | ENSSSCG00000031608 | 17 | + | 10751925 | 10752084 | 160 | antisense |
| novel_circ_000182 | ENSSSCG00000009397 | 11 | + | 18313796 | 18315297 | 225 | annot_exons |
| novel_circ_000183 | ENSSSCG00000000466 | 5 | + | 28832466 | 28841053 | 344 | annot_exons |
| novel_circ_000184 | ENSSSCG00000024628 | 9 | - | 36101267 | 36107397 | 404 | annot_exons |
| novel_circ_000185 | ENSSSCG00000014127 | 2 | + | 89659827 | 89661673 | 365 | annot_exons |
| novel_circ_000186 | ENSSSCG00000002266 | 7 | - | 86044906 | 86087677 | 2127 | annot_exons |
| novel_circ_000187 | ENSSSCG00000016426 | 18 | - | 4697810 | 4703032 | 411 | annot_exons |
| novel_circ_000188 | ENSSSCG00000014269 | 2 | - | 1.34E+08 | 1.34E+08 | 438 | annot_exons |
| novel_circ_000189 | ENSSSCG00000005388 | 1 | + | 2.42E+08 | 2.42E+08 | 493 | annot_exons |
| novel_circ_000190 | ENSSSCG00000027865 | 7 | - | 1.1E+08 | 1.1E+08 | 412 | annot_exons |
| novel_circ_000191 | ENSSSCG00000010799 | 3 | + | 22765850 | 22796166 | 993 | annot_exons |
| novel_circ_000192 | ENSSSCG00000014213 | 2 | + | 1.19E+08 | 1.19E+08 | 2151 | intronic |
| novel_circ_000193 | NA | 15 | - | 44894098 | 44909102 | 15005 | intergenic |
| novel_circ_000194 | ENSSSCG00000003520 | 6 | + | 80068941 | 80082895 | 536 | annot_exons |
| novel_circ_000195 | ENSSSCG00000007799 | 3 | + | 17938133 | 17938265 | 133 | antisense |
| novel_circ_000196 | ENSSSCG00000033010 | 2 | + | 9195329 | 9196465 | 453 | annot_exons |
| novel_circ_000197 | ENSSSCG00000027778 | 7 | - | 34083499 | 34089247 | 841 | annot_exons |
| novel_circ_000198 | ENSSSCG00000027700 | 6 | - | 1.2E+08 | 1.2E+08 | 5246 | annot_exons |
| novel_circ_000199 | ENSSSCG00000008575 | 3 | + | 1.13E+08 | 1.13E+08 | 346 | annot_exons |
| novel_circ_000200 | ENSSSCG00000035400 | 12 | + | 35597421 | 35597734 | 314 | one_exon |
| novel_circ_000201 | ENSSSCG00000010290 | 14 | + | 75535724 | 75536555 | 166 | annot_exons |
| novel_circ_000202 | NA | 15 | - | 84302046 | 84302229 | 184 | intergenic |
| novel_circ_000203 | ENSSSCG00000002869 | 6 | + | 43879422 | 43894306 | 953 | annot_exons |
| novel_circ_000204 | ENSSSCG00000029785 | 6 | - | 55922141 | 55922585 | 445 | one_exon |
| novel_circ_000205 | ENSSSCG00000016027 | 15 | + | 91678147 | 91684121 | 352 | annot_exons |
| novel_circ_000206 | ENSSSCG00000008816 | 8 | - | 37826042 | 37836112 | 342 | annot_exons |
| novel_circ_000207 | ENSSSCG00000010569 | 14 | - | 1.13E+08 | 1.13E+08 | 690 | annot_exons |
| novel_circ_000208 | ENSSSCG00000009557 | 11 | - | 78543049 | 78543436 | 267 | exon_intron |
| novel_circ_000209 | ENSSSCG00000006301 | 4 | - | 82779059 | 82785563 | 412 | annot_exons |
| novel_circ_000210 | ENSSSCG00000000985 | 7 | - | 276260 | 307475 | 955 | annot_exons |
| novel_circ_000211 | ENSSSCG00000009714 | 14 | + | 20294336 | 20298983 | 277 | annot_exons |
| novel_circ_000212 | ENSSSCG00000001056 | 7 | - | 9980131 | 9997198 | 944 | annot_exons |
| novel_circ_000213 | ENSSSCG00000015432 | 9 | - | 1.06E+08 | 1.06E+08 | 480 | one_exon |
| novel_circ_000214 | ENSSSCG00000023738 | 11 | - | 21856562 | 21865590 | 676 | annot_exons |
| novel_circ_000215 | ENSSSCG00000009049 | 8 | - | 84588856 | 84590905 | 232 | annot_exons |
| novel_circ_000216 | ENSSSCG00000014949 | 9 | + | 26202581 | 26212510 | 732 | annot_exons |
| novel_circ_000217 | ENSSSCG00000021106 | X | - | 43058182 | 43062039 | 1881 | annot_exons |
| novel_circ_000218 | ENSSSCG00000001819 | 7 | + | 53514150 | 53517804 | 732 | annot_exons |
| novel_circ_000219 | ENSSSCG00000017164 | 12 | + | 3285215 | 3289714 | 1867 | exon_intron |
| novel_circ_000220 | ENSSSCG00000015747 | 15 | + | 33537061 | 33591650 | 7213 | annot_exons |
| novel_circ_000221 | ENSSSCG00000000766 | 5 | + | 69698164 | 69699221 | 265 | annot_exons |
| novel_circ_000222 | ENSSSCG00000015695 | 15 | - | 16792894 | 16814482 | 874 | annot_exons |
| novel_circ_000223 | ENSSSCG00000022032 | 4 | - | 1.29E+08 | 1.29E+08 | 325 | annot_exons |
| novel_circ_000224 | ENSSSCG00000001683 | 7 | + | 38597678 | 38597847 | 170 | one_exon |
| novel_circ_000225 | ENSSSCG00000014044 | 2 | - | 80688840 | 80704603 | 844 | annot_exons |
| novel_circ_000226 | ENSSSCG00000015880 | 15 | + | 66079760 | 66081091 | 318 | annot_exons |
| novel_circ_000227 | ENSSSCG00000001971 | 7 | + | 68406557 | 68410328 | 883 | annot_exons |
| novel_circ_000228 | ENSSSCG00000012434 | X | - | 61787632 | 61801965 | 14192 | exon_intron |
| novel_circ_000229 | ENSSSCG00000002523 | AEMK02000452.1 | - | 1690212 | 1699171 | 444 | annot_exons |
| novel_circ_000230 | ENSSSCG00000014114 | 2 | - | 88453428 | 88453765 | 338 | antisense |
| novel_circ_000231 | ENSSSCG00000026268 | 14 | - | 80250994 | 80269275 | 2068 | annot_exons |
| novel_circ_000232 | ENSSSCG00000013282 | 2 | - | 18225127 | 18232055 | 772 | annot_exons |
| novel_circ_000233 | ENSSSCG00000009050 | 8 | + | 85435253 | 85472740 | 922 | annot_exons |
| novel_circ_000234 | ENSSSCG00000013022 | 2 | + | 7417229 | 7417951 | 299 | annot_exons |
| novel_circ_000235 | ENSSSCG00000008991 | 8 | + | 73851349 | 73853347 | 253 | annot_exons |
| novel_circ_000236 | ENSSSCG00000032377 | 7 | + | 64253214 | 64287500 | 1371 | annot_exons |
| novel_circ_000237 | ENSSSCG00000000167 | 5 | - | 13506832 | 13516783 | 290 | annot_exons |
| novel_circ_000238 | ENSSSCG00000007839 | 3 | - | 23921028 | 23922152 | 411 | annot_exons |
| novel_circ_000239 | ENSSSCG00000010190 | 14 | - | 60414208 | 60414578 | 371 | antisense |
| novel_circ_000240 | ENSSSCG00000021072 | 1 | - | 95513003 | 95517739 | 717 | annot_exons |
| novel_circ_000241 | NA | 7 | + | 12209078 | 12218590 | 9513 | intergenic |
| novel_circ_000242 | ENSSSCG00000021464 | 9 | + | 1.21E+08 | 1.21E+08 | 235 | annot_exons |
| novel_circ_000243 | ENSSSCG00000026954 | 3 | - | 44298411 | 44303573 | 340 | annot_exons |
| novel_circ_000244 | ENSSSCG00000025284 | 6 | + | 19376842 | 19379246 | 302 | annot_exons |
| novel_circ_000245 | ENSSSCG00000030581 | 13 | - | 67738558 | 67744231 | 5674 | exon_intron |
| novel_circ_000246 | ENSSSCG00000022446 | 8 | - | 19544289 | 19545247 | 386 | exon_intron |
| novel_circ_000247 | NA | 15 | - | 84294149 | 84294536 | 388 | intergenic |
| novel_circ_000248 | ENSSSCG00000023593 | 8 | - | 1.32E+08 | 1.32E+08 | 350 | annot_exons |
| novel_circ_000249 | ENSSSCG00000000045 | 5 | + | 6565453 | 6566862 | 265 | annot_exons |
| novel_circ_000250 | ENSSSCG00000004589 | 1 | - | 1.13E+08 | 1.13E+08 | 579 | annot_exons |
| novel_circ_000251 | ENSSSCG00000008909 | 8 | - | 54816224 | 54820045 | 414 | annot_exons |
| novel_circ_000252 | ENSSSCG00000007895 | 3 | + | 31358570 | 31359294 | 298 | annot_exons |
| novel_circ_000253 | ENSSSCG00000008079 | 3 | + | 42881922 | 42901540 | 606 | annot_exons |
| novel_circ_000254 | ENSSSCG00000014321 | 2 | - | 1.4E+08 | 1.4E+08 | 15978 | exon_intron |
| novel_circ_000255 | ENSSSCG00000035045 | 14 | + | 1.37E+08 | 1.37E+08 | 644 | annot_exons |
| novel_circ_000256 | ENSSSCG00000036480 | 14 | - | 70012752 | 70024217 | 234 | annot_exons |
| novel_circ_000257 | ENSSSCG00000025323 | 6 | + | 13559898 | 13566223 | 422 | annot_exons |
| novel_circ_000258 | NA | 12 | - | 61231989 | 61233325 | 1337 | intergenic |
| novel_circ_000259 | ENSSSCG00000015789 | 15 | + | 46356116 | 46357860 | 314 | annot_exons |
| novel_circ_000260 | ENSSSCG00000023297 | 15 | + | 80378383 | 80385906 | 764 | annot_exons |
| novel_circ_000261 | ENSSSCG00000016138 | 5 | + | 16628232 | 16669538 | 671 | annot_exons |
| novel_circ_000262 | NA | 15 | - | 84258318 | 84259038 | 721 | intergenic |
| novel_circ_000263 | ENSSSCG00000015410 | 9 | - | 1.02E+08 | 1.02E+08 | 343 | annot_exons |
| novel_circ_000264 | ENSSSCG00000040317 | 1 | + | 7680057 | 7688786 | 543 | annot_exons |
| novel_circ_000265 | ENSSSCG00000038202 | AEMK02000328.1 | - | 334734 | 334857 | 124 | one_exon |
| novel_circ_000266 | ENSSSCG00000003334 | 6 | - | 63557493 | 63558219 | 323 | annot_exons |
| novel_circ_000267 | ENSSSCG00000011162 | 10 | + | 68645780 | 68671405 | 3193 | annot_exons |
| novel_circ_000268 | ENSSSCG00000044283 | 2 | + | 15012704 | 15013066 | 363 | antisense |
| novel_circ_000269 | ENSSSCG00000024030 | 12 | + | 59744649 | 59748725 | 738 | annot_exons |
| novel_circ_000270 | ENSSSCG00000017617 | 12 | + | 33289729 | 33290715 | 208 | annot_exons |
| novel_circ_000271 | ENSSSCG00000028902 | 4 | + | 77614097 | 77618720 | 928 | annot_exons |
| novel_circ_000272 | ENSSSCG00000014304 | 2 | + | 1.37E+08 | 1.37E+08 | 437 | annot_exons |
| novel_circ_000273 | ENSSSCG00000034484 | 6 | + | 75038901 | 75083806 | 2155 | annot_exons |
| novel_circ_000274 | ENSSSCG00000005305 | 1 | + | 2.36E+08 | 2.36E+08 | 342 | annot_exons |
| novel_circ_000275 | ENSSSCG00000013262 | 2 | + | 15967840 | 16021491 | 2640 | annot_exons |
| novel_circ_000276 | ENSSSCG00000006872 | 4 | - | 1.18E+08 | 1.18E+08 | 681 | annot_exons |
| novel_circ_000277 | ENSSSCG00000004425 | 1 | - | 78149283 | 78153884 | 442 | annot_exons |
| novel_circ_000278 | ENSSSCG00000009522 | 11 | + | 69105921 | 69131726 | 296 | annot_exons |
| novel_circ_000279 | ENSSSCG00000011022 | 10 | + | 41229017 | 41257277 | 200 | annot_exons |
| novel_circ_000280 | ENSSSCG00000026945 | 13 | - | 29484735 | 29487818 | 293 | annot_exons |
| novel_circ_000281 | ENSSSCG00000012055 | 13 | + | 2E+08 | 2E+08 | 431 | exon_intron |
| novel_circ_000282 | ENSSSCG00000012650 | X | + | 1.06E+08 | 1.06E+08 | 246 | annot_exons |
| novel_circ_000283 | ENSSSCG00000010187 | 14 | + | 60266108 | 60276493 | 975 | annot_exons |
| novel_circ_000284 | ENSSSCG00000011752 | 13 | + | 1.11E+08 | 1.11E+08 | 16115 | exon_intron |
| novel_circ_000285 | NA | 13 | + | 1.35E+08 | 1.35E+08 | 534 | intergenic |
| novel_circ_000286 | ENSSSCG00000013764 | 2 | + | 65201161 | 65201312 | 152 | one_exon |
| novel_circ_000287 | ENSSSCG00000032531 | 3 | + | 26701499 | 26705896 | 943 | annot_exons |
| novel_circ_000288 | ENSSSCG00000014149 | 2 | - | 96152766 | 96164977 | 373 | annot_exons |
| novel_circ_000289 | ENSSSCG00000009093 | 8 | + | 1.02E+08 | 1.02E+08 | 616 | annot_exons |
| novel_circ_000290 | ENSSSCG00000007454 | 17 | - | 49346520 | 49347809 | 1290 | exon_intron |
| novel_circ_000291 | ENSSSCG00000016613 | 18 | + | 24985702 | 25003037 | 1398 | annot_exons |
| novel_circ_000292 | ENSSSCG00000003612 | 6 | + | 88699037 | 88699401 | 278 | annot_exons |
| novel_circ_000293 | ENSSSCG00000028902 | 4 | + | 77612564 | 77618720 | 1048 | annot_exons |
| novel_circ_000294 | ENSSSCG00000035034 | 10 | + | 12698273 | 12701699 | 254 | annot_exons |
| novel_circ_000295 | ENSSSCG00000037674 | 1 | - | 92423751 | 92424204 | 454 | one_exon |
| novel_circ_000296 | ENSSSCG00000023520 | 1 | + | 2.29E+08 | 2.29E+08 | 483 | annot_exons |
| novel_circ_000297 | NA | 15 | + | 84260142 | 84260587 | 446 | intergenic |
| novel_circ_000298 | ENSSSCG00000033626 | 12 | + | 60743597 | 60744303 | 548 | annot_exons |
| novel_circ_000299 | ENSSSCG00000004070 | 1 | - | 12071470 | 12086591 | 576 | annot_exons |
| novel_circ_000300 | ENSSSCG00000016487 | 18 | - | 7797516 | 7801676 | 476 | annot_exons |
| novel_circ_000301 | ENSSSCG00000024578 | 9 | + | 45935665 | 45936067 | 247 | annot_exons |
| novel_circ_000302 | ENSSSCG00000013363 | 2 | - | 40768231 | 40787526 | 466 | annot_exons |
| novel_circ_000303 | ENSSSCG00000029578 | 6 | + | 28711467 | 28743372 | 1671 | annot_exons |
| novel_circ_000304 | ENSSSCG00000039157 | 15 | + | 29812672 | 29873913 | 928 | annot_exons |
| novel_circ_000305 | ENSSSCG00000050244 | 12 | + | 59273100 | 59296987 | 360 | exon_intron |
| novel_circ_000306 | ENSSSCG00000023738 | 11 | - | 21786478 | 21793730 | 535 | annot_exons |
| novel_circ_000307 | ENSSSCG00000003682 | 6 | - | 98733976 | 98756185 | 8943 | exon_intron |
| novel_circ_000308 | ENSSSCG00000015709 | 15 | - | 19942032 | 19954276 | 509 | annot_exons |
| novel_circ_000309 | ENSSSCG00000001201 | 7 | - | 21998542 | 21999393 | 852 | antisense |
| novel_circ_000310 | ENSSSCG00000002352 | 7 | + | 97232940 | 97243264 | 797 | annot_exons |
| novel_circ_000311 | ENSSSCG00000012456 | X | - | 67336836 | 67341231 | 243 | annot_exons |
| novel_circ_000312 | ENSSSCG00000008943 | 8 | + | 68000421 | 68061384 | 331 | annot_exons |
| novel_circ_000313 | ENSSSCG00000015894 | 15 | - | 68718241 | 68720212 | 372 | annot_exons |
| novel_circ_000314 | ENSSSCG00000016763 | 18 | + | 52625249 | 52628187 | 469 | annot_exons |
| novel_circ_000315 | ENSSSCG00000038561 | 15 | - | 84411871 | 84413356 | 1128 | annot_exons |
| novel_circ_000316 | ENSSSCG00000015588 | 9 | + | 1.3E+08 | 1.3E+08 | 326 | one_exon |
| novel_circ_000317 | ENSSSCG00000006785 | 4 | - | 1.09E+08 | 1.09E+08 | 495 | annot_exons |
| novel_circ_000318 | ENSSSCG00000008434 | 3 | - | 93551318 | 93555120 | 247 | annot_exons |
| novel_circ_000319 | ENSSSCG00000009298 | 11 | + | 4114333 | 4118265 | 252 | annot_exons |
| novel_circ_000320 | ENSSSCG00000031967 | 3 | + | 18168182 | 18168370 | 189 | one_exon |
| novel_circ_000321 | ENSSSCG00000014853 | 9 | - | 9669570 | 9680837 | 597 | annot_exons |
| novel_circ_000322 | ENSSSCG00000007576 | 3 | + | 3613549 | 3613833 | 285 | one_exon |
| novel_circ_000323 | ENSSSCG00000030947 | 4 | + | 7060626 | 7060877 | 252 | one_exon |
| novel_circ_000324 | ENSSSCG00000015917 | 15 | + | 73725660 | 73725895 | 236 | one_exon |
| novel_circ_000325 | ENSSSCG00000008247 | 3 | + | 60162503 | 60174324 | 696 | annot_exons |
| novel_circ_000326 | ENSSSCG00000000985 | 7 | - | 306094 | 311618 | 469 | annot_exons |
| novel_circ_000327 | ENSSSCG00000016397 | 15 | - | 654498 | 683576 | 29079 | antisense |
| novel_circ_000328 | ENSSSCG00000005983 | 4 | + | 16076090 | 16077751 | 263 | annot_exons |
| novel_circ_000329 | ENSSSCG00000039854 | 4 | - | 1.22E+08 | 1.22E+08 | 244 | annot_exons |
| novel_circ_000330 | ENSSSCG00000004951 | 1 | - | 1.65E+08 | 1.65E+08 | 174 | annot_exons |
| novel_circ_000331 | ENSSSCG00000035639 | 6 | - | 27532352 | 27533552 | 262 | annot_exons |
| novel_circ_000332 | ENSSSCG00000016948 | 16 | - | 44117112 | 44129690 | 813 | annot_exons |
| novel_circ_000333 | ENSSSCG00000015940 | 15 | + | 76330340 | 76353578 | 643 | annot_exons |
| novel_circ_000334 | ENSSSCG00000032917 | 14 | - | 41775937 | 41778345 | 240 | annot_exons |
| novel_circ_000335 | ENSSSCG00000031775 | 5 | - | 73473989 | 73481454 | 7373 | exon_intron |
| novel_circ_000336 | ENSSSCG00000007950 | 3 | + | 38262252 | 38288061 | 1135 | annot_exons |
| novel_circ_000337 | ENSSSCG00000016409 | 18 | - | 1580250 | 1604899 | 1150 | annot_exons |
| novel_circ_000338 | ENSSSCG00000024088 | 1 | - | 1.09E+08 | 1.09E+08 | 440 | annot_exons |
| novel_circ_000339 | ENSSSCG00000016115 | 15 | - | 1.06E+08 | 1.06E+08 | 242 | annot_exons |
| novel_circ_000340 | ENSSSCG00000006927 | 4 | - | 1.28E+08 | 1.28E+08 | 476 | annot_exons |
| novel_circ_000341 | ENSSSCG00000026276 | 6 | + | 80443721 | 80451792 | 7159 | exon_intron |
| novel_circ_000342 | ENSSSCG00000016494 | 18 | + | 9065917 | 9079520 | 608 | annot_exons |
| novel_circ_000343 | ENSSSCG00000028855 | 13 | + | 95766107 | 95775714 | 317 | annot_exons |
| novel_circ_000344 | NA | 15 | - | 84265779 | 84266025 | 247 | intergenic |
| novel_circ_000345 | ENSSSCG00000015247 | 9 | - | 56949123 | 56949658 | 536 | one_exon |
| novel_circ_000346 | ENSSSCG00000024761 | 9 | - | 87005342 | 87010419 | 532 | annot_exons |
| novel_circ_000347 | ENSSSCG00000039591 | 17 | - | 46907362 | 46918309 | 2033 | annot_exons |
| novel_circ_000348 | ENSSSCG00000025156 | X | - | 64310696 | 64319564 | 842 | annot_exons |
| novel_circ_000349 | ENSSSCG00000007023 | 17 | - | 11052778 | 11059120 | 307 | annot_exons |
| novel_circ_000350 | ENSSSCG00000015667 | 15 | - | 3697809 | 3700621 | 1235 | annot_exons |
| novel_circ_000351 | ENSSSCG00000002266 | 7 | - | 86072288 | 86075222 | 249 | annot_exons |
| novel_circ_000352 | ENSSSCG00000006071 | 4 | - | 37503204 | 37539512 | 386 | annot_exons |
| novel_circ_000353 | ENSSSCG00000014112 | 2 | + | 88057795 | 88062654 | 321 | annot_exons |
| novel_circ_000354 | NA | 11 | + | 77555112 | 77556299 | 1188 | intergenic |
| novel_circ_000355 | ENSSSCG00000039714 | 7 | - | 62685674 | 62697811 | 335 | annot_exons |
| novel_circ_000356 | ENSSSCG00000040325 | 2 | + | 94345474 | 94345627 | 154 | one_exon |
| novel_circ_000357 | NA | 15 | - | 84255224 | 84255523 | 300 | intergenic |
| novel_circ_000358 | ENSSSCG00000022830 | 15 | - | 1.13E+08 | 1.13E+08 | 479 | annot_exons |
| novel_circ_000359 | ENSSSCG00000037185 | 14 | - | 1.09E+08 | 1.09E+08 | 1228 | annot_exons |
| novel_circ_000360 | ENSSSCG00000011848 | 13 | - | 1.34E+08 | 1.34E+08 | 477 | antisense |
| novel_circ_000361 | ENSSSCG00000010625 | 14 | + | 1.21E+08 | 1.21E+08 | 554 | annot_exons |
| novel_circ_000362 | ENSSSCG00000010823 | 10 | + | 9702153 | 9713083 | 358 | annot_exons |
| novel_circ_000363 | ENSSSCG00000002285 | 7 | + | 90984394 | 90991180 | 6787 | intronic |
| novel_circ_000364 | NA | 15 | - | 84274585 | 84274801 | 217 | intergenic |
| novel_circ_000365 | ENSSSCG00000039539 | 10 | - | 59851943 | 59853584 | 465 | annot_exons |
| novel_circ_000366 | ENSSSCG00000005657 | 1 | + | 2.69E+08 | 2.69E+08 | 424 | annot_exons |
| novel_circ_000367 | ENSSSCG00000026617 | 17 | - | 37556106 | 37556638 | 533 | intronic |
| novel_circ_000368 | ENSSSCG00000001471 | 7 | - | 25161247 | 25161971 | 725 | antisense |
| novel_circ_000369 | ENSSSCG00000015695 | 15 | - | 16788284 | 16808289 | 851 | annot_exons |
| novel_circ_000370 | ENSSSCG00000003760 | 6 | + | 1.3E+08 | 1.3E+08 | 288 | annot_exons |
| novel_circ_000371 | ENSSSCG00000007206 | 17 | - | 34795182 | 34795577 | 293 | annot_exons |
| novel_circ_000372 | ENSSSCG00000009563 | 11 | + | 78715314 | 78717933 | 690 | annot_exons |
| novel_circ_000373 | NA | 15 | - | 84246039 | 84246541 | 503 | intergenic |
| novel_circ_000374 | ENSSSCG00000000939 | 5 | - | 1E+08 | 1E+08 | 469 | annot_exons |
| novel_circ_000375 | ENSSSCG00000049152 | 1 | + | 74766067 | 74788377 | 7398 | exon_intron |
| novel_circ_000376 | ENSSSCG00000011681 | 13 | + | 83469879 | 83474843 | 533 | annot_exons |
| novel_circ_000377 | ENSSSCG00000014891 | 9 | - | 12723325 | 12723625 | 301 | one_exon |
| novel_circ_000378 | ENSSSCG00000029251 | 13 | - | 30633074 | 30634417 | 1344 | one_exon |
| novel_circ_000379 | ENSSSCG00000015689 | 15 | + | 15866989 | 15872004 | 395 | annot_exons |
| novel_circ_000380 | ENSSSCG00000016033 | 15 | + | 93034378 | 93074570 | 34137 | exon_intron |
| novel_circ_000381 | ENSSSCG00000035673 | 9 | + | 1.07E+08 | 1.07E+08 | 1213 | annot_exons |
| novel_circ_000382 | ENSSSCG00000008444 | 3 | - | 94650240 | 94652277 | 2038 | intronic |
| novel_circ_000383 | ENSSSCG00000039843 | 10 | + | 24149673 | 24150271 | 599 | one_exon |
| novel_circ_000384 | ENSSSCG00000036926 | 1 | + | 2.67E+08 | 2.67E+08 | 350 | annot_exons |
| novel_circ_000385 | ENSSSCG00000006779 | 4 | + | 1.08E+08 | 1.08E+08 | 623 | annot_exons |
| novel_circ_000386 | ENSSSCG00000003194 | 6 | - | 54882110 | 54884400 | 689 | annot_exons |
| novel_circ_000387 | ENSSSCG00000012371 | X | + | 53675620 | 53702148 | 26529 | exon_intron |
| novel_circ_000388 | ENSSSCG00000017673 | 12 | + | 36450740 | 36461144 | 517 | annot_exons |
| novel_circ_000389 | ENSSSCG00000003771 | 6 | + | 1.35E+08 | 1.35E+08 | 204 | annot_exons |
| novel_circ_000390 | ENSSSCG00000031701 | 5 | - | 73372351 | 73399218 | 940 | annot_exons |
| novel_circ_000391 | ENSSSCG00000035956 | 10 | - | 25606345 | 25629631 | 650 | annot_exons |
| novel_circ_000392 | ENSSSCG00000024614 | 13 | + | 1.97E+08 | 1.97E+08 | 442 | annot_exons |
| novel_circ_000393 | ENSSSCG00000005252 | 1 | - | 2.23E+08 | 2.23E+08 | 342 | annot_exons |
| novel_circ_000394 | ENSSSCG00000038178 | AEMK02000697.1 | - | 40353 | 43374 | 2883 | annot_exons |
| novel_circ_000395 | ENSSSCG00000038776 | 2 | - | 66252542 | 66252936 | 306 | annot_exons |
| novel_circ_000396 | ENSSSCG00000022788 | 8 | + | 1.16E+08 | 1.16E+08 | 388 | annot_exons |
| novel_circ_000397 | ENSSSCG00000028227 | 17 | + | 40326208 | 40340166 | 466 | annot_exons |
| novel_circ_000398 | ENSSSCG00000049707 | 6 | - | 1.49E+08 | 1.49E+08 | 47168 | exon_intron |
| novel_circ_000399 | ENSSSCG00000004048 | 1 | + | 7593401 | 7598966 | 720 | annot_exons |
| novel_circ_000400 | ENSSSCG00000050244 | 12 | + | 59319172 | 59323398 | 600 | annot_exons |
| novel_circ_000401 | ENSSSCG00000012597 | X | + | 94971217 | 94971539 | 323 | one_exon |
| novel_circ_000402 | ENSSSCG00000029633 | 6 | + | 1.57E+08 | 1.57E+08 | 997 | annot_exons |
| novel_circ_000403 | ENSSSCG00000020878 | 3 | + | 1.02E+08 | 1.02E+08 | 6186 | exon_intron |
| novel_circ_000404 | NA | 15 | - | 84488230 | 84493916 | 5687 | intergenic |
| novel_circ_000405 | ENSSSCG00000033591 | 6 | - | 31962635 | 31980100 | 17466 | exon_intron |
| novel_circ_000406 | ENSSSCG00000029983 | 8 | + | 167697 | 171693 | 338 | annot_exons |
| novel_circ_000407 | ENSSSCG00000021023 | 13 | - | 1.95E+08 | 1.95E+08 | 433 | annot_exons |
| novel_circ_000408 | ENSSSCG00000009338 | 11 | + | 8663835 | 8670626 | 427 | annot_exons |
| novel_circ_000409 | ENSSSCG00000010357 | 14 | - | 87518138 | 87519470 | 402 | annot_exons |
| novel_circ_000410 | ENSSSCG00000009652 | 14 | - | 9478895 | 9498411 | 599 | annot_exons |
| novel_circ_000411 | ENSSSCG00000014113 | 2 | - | 88184110 | 88196830 | 233 | annot_exons |
| novel_circ_000412 | ENSSSCG00000015872 | 15 | + | 63658489 | 63661287 | 387 | annot_exons |
| novel_circ_000413 | ENSSSCG00000002311 | 7 | + | 93296032 | 93296234 | 203 | one_exon |
| novel_circ_000414 | ENSSSCG00000001078 | 7 | - | 15557725 | 15565867 | 488 | annot_exons |
| novel_circ_000415 | ENSSSCG00000001732 | 7 | - | 43616015 | 43618755 | 698 | annot_exons |
| novel_circ_000416 | ENSSSCG00000000766 | 5 | + | 69719912 | 69733724 | 584 | annot_exons |
| novel_circ_000417 | ENSSSCG00000035747 | 3 | - | 1.07E+08 | 1.07E+08 | 518 | annot_exons |
| novel_circ_000418 | ENSSSCG00000010360 | 14 | + | 87862520 | 87869921 | 300 | annot_exons |
| novel_circ_000419 | ENSSSCG00000008659 | 3 | - | 1.33E+08 | 1.33E+08 | 939 | annot_exons |
| novel_circ_000420 | ENSSSCG00000004089 | 1 | + | 14800957 | 14801447 | 491 | one_exon |
| novel_circ_000421 | ENSSSCG00000033272 | 18 | - | 1475203 | 1483960 | 603 | annot_exons |
| novel_circ_000422 | ENSSSCG00000013878 | 2 | - | 60179898 | 60180750 | 241 | annot_exons |
| novel_circ_000423 | ENSSSCG00000012000 | 13 | + | 1.74E+08 | 1.74E+08 | 353 | annot_exons |
| novel_circ_000424 | ENSSSCG00000025478 | 6 | + | 1.07E+08 | 1.07E+08 | 272 | annot_exons |
| novel_circ_000425 | ENSSSCG00000026754 | 2 | + | 74812588 | 74819252 | 6665 | antisense |
| novel_circ_000426 | ENSSSCG00000002032 | 7 | + | 75917037 | 75918564 | 328 | annot_exons |
| novel_circ_000427 | ENSSSCG00000017415 | 12 | - | 20776186 | 20776382 | 197 | one_exon |
| novel_circ_000428 | ENSSSCG00000006089 | 4 | - | 40314498 | 40330526 | 421 | annot_exons |
| novel_circ_000429 | ENSSSCG00000016695 | 18 | + | 44996263 | 45029514 | 9630 | exon_intron |
| novel_circ_000430 | NA | 18 | - | 47586086 | 47588459 | 2374 | intergenic |
| novel_circ_000431 | ENSSSCG00000015840 | 15 | - | 54062575 | 54063425 | 221 | annot_exons |
| novel_circ_000432 | ENSSSCG00000016948 | 16 | - | 44128660 | 44129690 | 462 | annot_exons |
| novel_circ_000433 | ENSSSCG00000015947 | 15 | + | 77587838 | 77591547 | 253 | annot_exons |
| novel_circ_000434 | ENSSSCG00000006757 | 4 | + | 1.06E+08 | 1.06E+08 | 558 | annot_exons |
| novel_circ_000435 | ENSSSCG00000008382 | 3 | + | 80483094 | 80503895 | 972 | annot_exons |
| novel_circ_000436 | ENSSSCG00000008495 | 3 | + | 1.03E+08 | 1.03E+08 | 441 | annot_exons |
| novel_circ_000437 | ENSSSCG00000005444 | 1 | - | 2.5E+08 | 2.5E+08 | 732 | annot_exons |
| novel_circ_000438 | ENSSSCG00000002285 | 7 | + | 90698518 | 90751496 | 300 | annot_exons |
| novel_circ_000439 | NA | 15 | + | 84275393 | 84277694 | 2302 | intergenic |
| novel_circ_000440 | ENSSSCG00000009370 | 11 | - | 15321048 | 15322402 | 1355 | one_exon |
| novel_circ_000441 | NA | 15 | - | 84335915 | 84337290 | 1376 | intergenic |
| novel_circ_000442 | ENSSSCG00000003775 | 6 | - | 1.38E+08 | 1.38E+08 | 306 | annot_exons |
| novel_circ_000443 | ENSSSCG00000015107 | 9 | + | 46289646 | 46293514 | 1039 | annot_exons |
| novel_circ_000444 | ENSSSCG00000013856 | 2 | - | 61364330 | 61366847 | 501 | annot_exons |
| novel_circ_000445 | ENSSSCG00000038561 | 15 | - | 84405128 | 84407996 | 1407 | annot_exons |
| novel_circ_000446 | ENSSSCG00000032820 | 5 | + | 776735 | 795087 | 2370 | annot_exons |
| novel_circ_000447 | ENSSSCG00000011849 | 13 | + | 1.34E+08 | 1.34E+08 | 431 | annot_exons |
| novel_circ_000448 | ENSSSCG00000000869 | 5 | - | 82823058 | 82823773 | 265 | annot_exons |
| novel_circ_000449 | ENSSSCG00000004382 | 1 | - | 74045955 | 74052367 | 215 | annot_exons |
| novel_circ_000450 | ENSSSCG00000009856 | 14 | + | 35201779 | 35202946 | 1168 | one_exon |
| novel_circ_000451 | ENSSSCG00000017046 | 16 | + | 64608442 | 64669823 | 482 | annot_exons |
| novel_circ_000452 | ENSSSCG00000008247 | 3 | + | 60152979 | 60174324 | 917 | annot_exons |
| novel_circ_000453 | ENSSSCG00000009319 | 11 | + | 5577247 | 5593953 | 1106 | annot_exons |
| novel_circ_000454 | ENSSSCG00000005366 | 1 | + | 2.39E+08 | 2.39E+08 | 308 | annot_exons |
| novel_circ_000455 | ENSSSCG00000009134 | 8 | - | 1.12E+08 | 1.12E+08 | 410 | annot_exons |
| novel_circ_000456 | NA | 15 | + | 84336992 | 84337236 | 245 | intergenic |
| novel_circ_000457 | ENSSSCG00000004334 | 1 | - | 58523530 | 58533984 | 650 | annot_exons |
| novel_circ_000458 | ENSSSCG00000016929 | 16 | - | 38089159 | 38125845 | 353 | annot_exons |
| novel_circ_000459 | ENSSSCG00000020785 | 15 | + | 1.21E+08 | 1.21E+08 | 240 | one_exon |
| novel_circ_000460 | ENSSSCG00000011454 | 13 | - | 35069291 | 35101407 | 490 | annot_exons |
| novel_circ_000461 | ENSSSCG00000017996 | 12 | - | 54651213 | 54655388 | 417 | annot_exons |
| novel_circ_000462 | ENSSSCG00000008079 | 3 | + | 42881922 | 42946710 | 1823 | annot_exons |
| novel_circ_000463 | ENSSSCG00000006970 | 17 | - | 1547525 | 1548672 | 1148 | one_exon |
| novel_circ_000464 | ENSSSCG00000002755 | 6 | - | 17352018 | 17362974 | 1116 | annot_exons |
| novel_circ_000465 | ENSSSCG00000004294 | 1 | - | 54576238 | 54576836 | 272 | annot_exons |
| novel_circ_000466 | ENSSSCG00000010144 | 14 | - | 54714524 | 54716478 | 322 | annot_exons |
| novel_circ_000467 | ENSSSCG00000002844 | 6 | - | 37077213 | 37093779 | 332 | annot_exons |
| novel_circ_000468 | ENSSSCG00000001543 | 7 | + | 31320130 | 31320430 | 301 | exon_intron |
| novel_circ_000469 | ENSSSCG00000013022 | 2 | + | 7409758 | 7411181 | 347 | annot_exons |
| novel_circ_000470 | ENSSSCG00000024486 | 17 | + | 11873945 | 11875595 | 343 | annot_exons |
| novel_circ_000471 | ENSSSCG00000022830 | 15 | - | 1.13E+08 | 1.13E+08 | 525 | annot_exons |
| novel_circ_000472 | ENSSSCG00000010300 | 14 | - | 76278036 | 76289229 | 928 | annot_exons |
| novel_circ_000473 | ENSSSCG00000006635 | 4 | + | 98119411 | 98119628 | 218 | one_exon |
| novel_circ_000474 | ENSSSCG00000003384 | 6 | - | 67476473 | 67477648 | 252 | annot_exons |
| novel_circ_000475 | NA | 15 | + | 84276269 | 84276513 | 245 | intergenic |
| novel_circ_000476 | ENSSSCG00000028925 | 12 | - | 14533441 | 14538259 | 723 | annot_exons |
| novel_circ_000477 | ENSSSCG00000010211 | 14 | - | 63220984 | 63237347 | 279 | annot_exons |
| novel_circ_000478 | ENSSSCG00000023974 | 2 | + | 16347499 | 16419290 | 30494 | exon_intron |
| novel_circ_000479 | ENSSSCG00000033528 | 12 | + | 14207097 | 14208324 | 254 | annot_exons |
| novel_circ_000480 | ENSSSCG00000016578 | 18 | - | 19768651 | 19768840 | 190 | one_exon |
| novel_circ_000481 | ENSSSCG00000001975 | 7 | + | 69814386 | 69828037 | 920 | annot_exons |
| novel_circ_000482 | ENSSSCG00000008647 | 3 | + | 1.29E+08 | 1.29E+08 | 317 | annot_exons |
| novel_circ_000483 | ENSSSCG00000025703 | 1 | - | 77324713 | 77334918 | 426 | annot_exons |
| novel_circ_000484 | ENSSSCG00000001769 | 7 | + | 48072181 | 48073231 | 347 | annot_exons |
| novel_circ_000485 | ENSSSCG00000024314 | 8 | + | 74626204 | 74627248 | 1045 | intronic |
| novel_circ_000486 | ENSSSCG00000011104 | 10 | - | 57719627 | 57721859 | 447 | annot_exons |
| novel_circ_000487 | ENSSSCG00000010928 | 10 | - | 24768866 | 24781769 | 1098 | annot_exons |
| novel_circ_000488 | ENSSSCG00000024761 | 9 | - | 87039402 | 87043305 | 315 | annot_exons |
| novel_circ_000489 | ENSSSCG00000003400 | 6 | + | 70298143 | 70300419 | 355 | annot_exons |
| novel_circ_000490 | ENSSSCG00000014114 | 2 | + | 88453374 | 88453784 | 411 | intronic |
| novel_circ_000491 | ENSSSCG00000009242 | 8 | + | 1.35E+08 | 1.35E+08 | 198 | one_exon |
| novel_circ_000492 | ENSSSCG00000030211 | 12 | - | 19773697 | 19784998 | 420 | annot_exons |
| novel_circ_000493 | ENSSSCG00000028735 | 14 | + | 23265995 | 23266318 | 216 | annot_exons |
| novel_circ_000494 | ENSSSCG00000018049 | 12 | - | 60361138 | 60376920 | 710 | annot_exons |
| novel_circ_000495 | ENSSSCG00000016543 | 18 | - | 15166982 | 15172587 | 339 | annot_exons |
| novel_circ_000496 | ENSSSCG00000051585 | 6 | - | 17899516 | 17911672 | 266 | annot_exons |
| novel_circ_000497 | ENSSSCG00000028902 | 4 | + | 77612564 | 77614563 | 418 | annot_exons |
| novel_circ_000498 | ENSSSCG00000032377 | 7 | + | 64253214 | 64257533 | 578 | annot_exons |
| novel_circ_000499 | ENSSSCG00000028097 | 6 | + | 1.66E+08 | 1.66E+08 | 482 | annot_exons |
| novel_circ_000500 | ENSSSCG00000030420 | 6 | + | 13123284 | 13127854 | 396 | annot_exons |
| novel_circ_000501 | ENSSSCG00000004522 | 1 | + | 1E+08 | 1E+08 | 254 | annot_exons |
| novel_circ_000502 | ENSSSCG00000028902 | 4 | + | 77612564 | 77621690 | 1219 | annot_exons |
| novel_circ_000503 | ENSSSCG00000000870 | 5 | + | 82803582 | 82804808 | 194 | annot_exons |
| novel_circ_000504 | ENSSSCG00000037561 | 11 | + | 78349882 | 78356609 | 408 | annot_exons |
| novel_circ_000505 | ENSSSCG00000003438 | 6 | + | 72268441 | 72268810 | 272 | annot_exons |
| novel_circ_000506 | ENSSSCG00000005934 | 4 | + | 3108315 | 3124561 | 9270 | exon_intron |
| novel_circ_000507 | ENSSSCG00000012730 | X | + | 1.21E+08 | 1.21E+08 | 967 | annot_exons |
| novel_circ_000508 | ENSSSCG00000003760 | 6 | + | 1.3E+08 | 1.3E+08 | 377 | annot_exons |
| novel_circ_000509 | ENSSSCG00000012459 | X | + | 68397834 | 68406594 | 389 | exon_intron |
| novel_circ_000510 | ENSSSCG00000039885 | 6 | - | 88429060 | 88432891 | 846 | annot_exons |
| novel_circ_000511 | ENSSSCG00000001065 | 7 | - | 12320895 | 12322989 | 2095 | one_exon |
| novel_circ_000512 | ENSSSCG00000000939 | 5 | - | 1E+08 | 1E+08 | 574 | annot_exons |
| novel_circ_000513 | ENSSSCG00000010238 | 14 | + | 71491530 | 71494146 | 662 | annot_exons |
| novel_circ_000514 | ENSSSCG00000017676 | 12 | - | 37178174 | 37269205 | 1303 | annot_exons |
| novel_circ_000515 | ENSSSCG00000015569 | 9 | + | 1.26E+08 | 1.26E+08 | 436 | exon_intron |
| novel_circ_000516 | ENSSSCG00000038437 | AEMK02000682.1 | - | 264782 | 267811 | 662 | annot_exons |
| novel_circ_000517 | ENSSSCG00000012878 | 2 | - | 4184685 | 4187133 | 2449 | exon_intron |
| novel_circ_000518 | ENSSSCG00000001499 | 7 | + | 29258310 | 29258981 | 327 | annot_exons |
| novel_circ_000519 | ENSSSCG00000016943 | 16 | - | 43984409 | 43997996 | 616 | annot_exons |
| novel_circ_000520 | ENSSSCG00000011163 | 10 | + | 68961015 | 69017027 | 615 | annot_exons |
| novel_circ_000521 | ENSSSCG00000003738 | 6 | + | 1.19E+08 | 1.19E+08 | 893 | exon_intron |
| novel_circ_000522 | ENSSSCG00000003512 | 6 | - | 79075061 | 79100041 | 753 | annot_exons |
| novel_circ_000523 | ENSSSCG00000036898 | 15 | + | 32176354 | 32176597 | 244 | antisense |
| novel_circ_000524 | ENSSSCG00000009276 | 11 | - | 1141224 | 1175446 | 856 | annot_exons |
| novel_circ_000525 | ENSSSCG00000037499 | 1 | + | 1.16E+08 | 1.16E+08 | 288 | exon_intron |
| novel_circ_000526 | ENSSSCG00000025478 | 6 | + | 1.07E+08 | 1.07E+08 | 1603 | intronic |
| novel_circ_000527 | ENSSSCG00000013257 | 2 | - | 15861767 | 15862791 | 310 | annot_exons |
| novel_circ_000528 | ENSSSCG00000025408 | 13 | - | 25706237 | 25715600 | 445 | annot_exons |
| novel_circ_000529 | ENSSSCG00000004729 | 1 | - | 1.29E+08 | 1.29E+08 | 1299 | annot_exons |
| novel_circ_000530 | ENSSSCG00000006508 | 4 | + | 94391005 | 94391746 | 742 | one_exon |
| novel_circ_000531 | ENSSSCG00000009021 | 8 | + | 78459588 | 78480241 | 450 | annot_exons |
| novel_circ_000532 | ENSSSCG00000016082 | 15 | - | 1.02E+08 | 1.02E+08 | 377 | annot_exons |
| novel_circ_000533 | ENSSSCG00000001971 | 7 | + | 68362207 | 68362706 | 310 | annot_exons |
| novel_circ_000534 | ENSSSCG00000039909 | 12 | + | 14961568 | 14962002 | 435 | one_exon |
| novel_circ_000535 | ENSSSCG00000031728 | 4 | + | 30712281 | 30712625 | 345 | one_exon |
| novel_circ_000536 | ENSSSCG00000008242 | 3 | - | 59631095 | 59654085 | 868 | annot_exons |
| novel_circ_000537 | ENSSSCG00000010235 | 14 | + | 71093567 | 71097952 | 512 | annot_exons |
| novel_circ_000538 | ENSSSCG00000015689 | 15 | + | 15878160 | 15886662 | 2061 | exon_intron |
| novel_circ_000539 | ENSSSCG00000014321 | 2 | - | 1.4E+08 | 1.4E+08 | 267 | annot_exons |
| novel_circ_000540 | ENSSSCG00000002664 | 6 | - | 3279069 | 3279294 | 226 | exon_intron |
| novel_circ_000541 | ENSSSCG00000040434 | 3 | + | 56983865 | 56986394 | 656 | annot_exons |
| novel_circ_000542 | ENSSSCG00000034493 | 13 | - | 1.6E+08 | 1.6E+08 | 392 | annot_exons |
| novel_circ_000543 | NA | 2 | + | 78813175 | 78813634 | 460 | intergenic |
| novel_circ_000544 | ENSSSCG00000003730 | 6 | + | 1.16E+08 | 1.16E+08 | 559 | annot_exons |
| novel_circ_000545 | ENSSSCG00000021393 | 9 | - | 1.26E+08 | 1.26E+08 | 988 | annot_exons |
| novel_circ_000546 | ENSSSCG00000006508 | 4 | + | 94446190 | 94446665 | 354 | annot_exons |
| novel_circ_000547 | ENSSSCG00000007454 | 17 | - | 49298226 | 49333789 | 1258 | annot_exons |
| novel_circ_000548 | ENSSSCG00000009172 | 8 | + | 1.2E+08 | 1.2E+08 | 398 | annot_exons |
| novel_circ_000549 | ENSSSCG00000042910 | 6 | + | 91974319 | 91997101 | 2159 | annot_exons |
| novel_circ_000550 | ENSSSCG00000009430 | 11 | - | 24729025 | 24739289 | 275 | annot_exons |
| novel_circ_000551 | ENSSSCG00000020858 | 7 | - | 13579269 | 13588465 | 610 | annot_exons |
| novel_circ_000552 | ENSSSCG00000003218 | 6 | + | 55285024 | 55297457 | 1450 | annot_exons |
| novel_circ_000553 | ENSSSCG00000008836 | 8 | - | 40092506 | 40093023 | 518 | exon_intron |
| novel_circ_000554 | ENSSSCG00000030373 | 3 | - | 23699476 | 23704670 | 401 | annot_exons |
| novel_circ_000555 | ENSSSCG00000011022 | 10 | + | 41430334 | 41436115 | 763 | annot_exons |
| novel_circ_000556 | ENSSSCG00000010947 | 10 | + | 27638465 | 27642090 | 256 | annot_exons |
| novel_circ_000557 | NA | 3 | - | 85570718 | 85584516 | 13799 | intergenic |
| novel_circ_000558 | ENSSSCG00000008624 | 3 | - | 1.25E+08 | 1.25E+08 | 363 | annot_exons |
| novel_circ_000559 | ENSSSCG00000012055 | 13 | + | 2E+08 | 2E+08 | 498 | annot_exons |
| novel_circ_000560 | ENSSSCG00000009320 | 11 | - | 5730927 | 5744988 | 1042 | annot_exons |
| novel_circ_000561 | ENSSSCG00000009676 | 14 | - | 12140805 | 12143946 | 710 | annot_exons |
| novel_circ_000562 | ENSSSCG00000006137 | 4 | - | 50402450 | 50403521 | 396 | annot_exons |
| novel_circ_000563 | ENSSSCG00000048439 | 18 | - | 54562129 | 54563773 | 362 | annot_exons |
| novel_circ_000564 | ENSSSCG00000023408 | 1 | + | 1.84E+08 | 1.84E+08 | 519 | one_exon |
| novel_circ_000565 | ENSSSCG00000008079 | 3 | - | 42968856 | 42969430 | 575 | antisense |
| novel_circ_000566 | NA | 15 | + | 84423429 | 84423554 | 126 | intergenic |
| novel_circ_000567 | ENSSSCG00000039157 | 15 | + | 29977370 | 30021973 | 2000 | annot_exons |
| novel_circ_000568 | ENSSSCG00000007757 | 3 | - | 17290706 | 17292922 | 435 | annot_exons |
| novel_circ_000569 | NA | 7 | - | 92673128 | 92680411 | 7284 | intergenic |
| novel_circ_000570 | ENSSSCG00000000795 | 5 | - | 74562717 | 74595084 | 9843 | exon_intron |
| novel_circ_000571 | ENSSSCG00000009568 | 11 | - | 603753 | 604571 | 581 | annot_exons |
| novel_circ_000572 | ENSSSCG00000029030 | 6 | - | 1.04E+08 | 1.04E+08 | 304 | annot_exons |
| novel_circ_000573 | ENSSSCG00000028228 | 3 | + | 80290983 | 80295248 | 621 | annot_exons |
| novel_circ_000574 | ENSSSCG00000008641 | 3 | + | 1.27E+08 | 1.27E+08 | 439 | annot_exons |
| novel_circ_000575 | NA | 8 | - | 1.14E+08 | 1.14E+08 | 2832 | intergenic |
| novel_circ_000576 | ENSSSCG00000030420 | 6 | + | 13145271 | 13149888 | 438 | annot_exons |
| novel_circ_000577 | ENSSSCG00000037670 | X | + | 90253910 | 90285436 | 389 | annot_exons |
| novel_circ_000578 | NA | 15 | + | 84267461 | 84267923 | 463 | intergenic |
| novel_circ_000579 | ENSSSCG00000024916 | 13 | - | 94319174 | 94320360 | 217 | annot_exons |
| novel_circ_000580 | ENSSSCG00000021042 | 13 | - | 2.03E+08 | 2.03E+08 | 403 | annot_exons |
| novel_circ_000581 | ENSSSCG00000027746 | 8 | - | 4120868 | 4121599 | 732 | one_exon |
| novel_circ_000582 | ENSSSCG00000006137 | 4 | - | 50395149 | 50408469 | 831 | annot_exons |
| novel_circ_000583 | ENSSSCG00000007027 | 17 | - | 11456345 | 11477126 | 559 | annot_exons |
| novel_circ_000584 | ENSSSCG00000017617 | 12 | + | 33277944 | 33289822 | 706 | annot_exons |
| novel_circ_000585 | ENSSSCG00000037305 | 8 | + | 33016128 | 33023577 | 1212 | exon_intron |
| novel_circ_000586 | ENSSSCG00000039103 | 13 | - | 1.25E+08 | 1.25E+08 | 403 | antisense |
| novel_circ_000587 | ENSSSCG00000011245 | 13 | + | 22318659 | 22335113 | 359 | annot_exons |
| novel_circ_000588 | ENSSSCG00000027074 | 2 | + | 1.14E+08 | 1.14E+08 | 595 | annot_exons |
| novel_circ_000589 | ENSSSCG00000008697 | 8 | + | 1861464 | 1862708 | 268 | annot_exons |
| novel_circ_000590 | ENSSSCG00000015499 | 9 | + | 1.16E+08 | 1.16E+08 | 401 | annot_exons |
| novel_circ_000591 | ENSSSCG00000016234 | 15 | - | 1.26E+08 | 1.26E+08 | 322 | annot_exons |
| novel_circ_000592 | ENSSSCG00000005201 | 1 | - | 2.16E+08 | 2.16E+08 | 16976 | exon_intron |
| novel_circ_000593 | ENSSSCG00000037424 | 1 | - | 76649382 | 76727151 | 688 | annot_exons |
| novel_circ_000594 | ENSSSCG00000036572 | 6 | - | 7029994 | 7037336 | 778 | annot_exons |
| novel_circ_000595 | ENSSSCG00000006100 | 4 | + | 42172655 | 42182843 | 421 | annot_exons |
| novel_circ_000596 | ENSSSCG00000007606 | 3 | + | 5912032 | 5926984 | 3027 | annot_exons |
| novel_circ_000597 | ENSSSCG00000012619 | X | - | 98060895 | 98064338 | 349 | annot_exons |
| novel_circ_000598 | ENSSSCG00000014057 | 2 | - | 81412625 | 81415989 | 494 | annot_exons |
| novel_circ_000599 | ENSSSCG00000009680 | 14 | + | 12451154 | 12455779 | 4626 | intronic |
| novel_circ_000600 | ENSSSCG00000001792 | 7 | - | 50941924 | 50946333 | 461 | annot_exons |
| novel_circ_000601 | ENSSSCG00000017513 | 12 | + | 23857979 | 23859066 | 270 | annot_exons |
| novel_circ_000602 | NA | 15 | - | 84265723 | 84266316 | 594 | intergenic |
| novel_circ_000603 | ENSSSCG00000020879 | 8 | + | 76509953 | 76521396 | 11444 | intronic |
| novel_circ_000604 | ENSSSCG00000005356 | 1 | + | 2.39E+08 | 2.39E+08 | 317 | annot_exons |
| novel_circ_000605 | ENSSSCG00000001641 | 7 | + | 37701803 | 37713084 | 651 | annot_exons |
| novel_circ_000606 | ENSSSCG00000016306 | 15 | - | 1.34E+08 | 1.34E+08 | 1283 | annot_exons |
| novel_circ_000607 | ENSSSCG00000015273 | 9 | + | 64407564 | 64431528 | 2372 | annot_exons |
| novel_circ_000608 | ENSSSCG00000016330 | 15 | + | 1.37E+08 | 1.37E+08 | 732 | annot_exons |
| novel_circ_000609 | ENSSSCG00000008624 | 3 | - | 1.25E+08 | 1.25E+08 | 601 | annot_exons |
| novel_circ_000610 | ENSSSCG00000005706 | 1 | + | 2.71E+08 | 2.71E+08 | 255 | annot_exons |
| novel_circ_000611 | ENSSSCG00000013400 | 2 | - | 47239763 | 47267743 | 412 | annot_exons |
| novel_circ_000612 | ENSSSCG00000000959 | AEMK02000361.1 | - | 621662 | 638070 | 16409 | exon_intron |
| novel_circ_000613 | ENSSSCG00000008242 | 3 | - | 59650317 | 59654085 | 308 | annot_exons |
| novel_circ_000614 | ENSSSCG00000026498 | 5 | - | 22670877 | 22689131 | 2883 | exon_intron |
| novel_circ_000615 | NA | 15 | - | 84309233 | 84309859 | 627 | intergenic |
| novel_circ_000616 | ENSSSCG00000004753 | 1 | + | 1.3E+08 | 1.3E+08 | 387 | annot_exons |
| novel_circ_000617 | ENSSSCG00000010288 | 14 | - | 75247422 | 75295452 | 419 | annot_exons |
| novel_circ_000618 | ENSSSCG00000004465 | 1 | - | 85893162 | 85893450 | 289 | intronic |
| novel_circ_000619 | ENSSSCG00000007514 | 17 | + | 58635283 | 58672300 | 29520 | exon_intron |
| novel_circ_000620 | ENSSSCG00000000970 | 5 | - | 302322 | 313850 | 898 | annot_exons |
| novel_circ_000621 | ENSSSCG00000013065 | 2 | - | 9340611 | 9343503 | 2308 | exon_intron |
| novel_circ_000622 | ENSSSCG00000010987 | 10 | + | 32859214 | 32870103 | 1060 | annot_exons |
| novel_circ_000623 | ENSSSCG00000011235 | 13 | - | 19227601 | 19234893 | 662 | annot_exons |
| novel_circ_000624 | ENSSSCG00000009229 | 8 | - | 1.33E+08 | 1.33E+08 | 757 | intronic |
| novel_circ_000625 | ENSSSCG00000008382 | 3 | + | 80551101 | 80551468 | 278 | exon_intron |
| novel_circ_000626 | ENSSSCG00000029697 | 1 | - | 128198 | 132692 | 519 | annot_exons |
| novel_circ_000627 | ENSSSCG00000004432 | 1 | - | 79885359 | 79886038 | 214 | annot_exons |
| novel_circ_000628 | ENSSSCG00000031775 | 5 | - | 73473989 | 73474253 | 172 | annot_exons |
| novel_circ_000629 | ENSSSCG00000010627 | 14 | + | 1.21E+08 | 1.21E+08 | 398 | annot_exons |
| novel_circ_000630 | ENSSSCG00000017094 | 16 | - | 72049998 | 72059836 | 676 | annot_exons |
| novel_circ_000631 | ENSSSCG00000024201 | 13 | - | 38536703 | 38545830 | 691 | annot_exons |
| novel_circ_000632 | ENSSSCG00000026536 | 11 | - | 78682007 | 78703726 | 2787 | annot_exons |
| novel_circ_000633 | ENSSSCG00000002357 | 7 | - | 97373565 | 97373867 | 303 | one_exon |
| novel_circ_000634 | ENSSSCG00000003512 | 6 | - | 79150170 | 79180903 | 1465 | annot_exons |
| novel_circ_000635 | ENSSSCG00000015857 | 15 | - | 59239097 | 59244748 | 452 | annot_exons |
| novel_circ_000636 | ENSSSCG00000000755 | 5 | + | 68310430 | 68329034 | 651 | annot_exons |
| novel_circ_000637 | ENSSSCG00000000039 | 5 | + | 6185346 | 6186572 | 478 | annot_exons |
| novel_circ_000638 | ENSSSCG00000010360 | 14 | + | 87806080 | 87822845 | 162 | annot_exons |
| novel_circ_000639 | ENSSSCG00000003870 | 6 | + | 1.6E+08 | 1.6E+08 | 221 | intronic |
| novel_circ_000640 | ENSSSCG00000001970 | 7 | - | 68233279 | 68257797 | 24519 | antisense |
| novel_circ_000641 | ENSSSCG00000009491 | 11 | - | 63701551 | 63706143 | 369 | annot_exons |
| novel_circ_000642 | ENSSSCG00000014148 | 2 | - | 95661294 | 95677798 | 380 | annot_exons |
| novel_circ_000643 | ENSSSCG00000015323 | 9 | + | 73684677 | 73684904 | 228 | intronic |
| novel_circ_000644 | ENSSSCG00000009134 | 8 | - | 1.12E+08 | 1.12E+08 | 860 | annot_exons |
| novel_circ_000645 | ENSSSCG00000005663 | 1 | - | 2.69E+08 | 2.69E+08 | 207 | annot_exons |
| novel_circ_000646 | ENSSSCG00000006116 | 4 | - | 45518253 | 45528300 | 498 | annot_exons |
| novel_circ_000647 | ENSSSCG00000010569 | 14 | - | 1.13E+08 | 1.13E+08 | 444 | annot_exons |
| novel_circ_000648 | ENSSSCG00000009042 | 8 | + | 83249031 | 83268011 | 1010 | annot_exons |
| novel_circ_000649 | ENSSSCG00000021042 | 13 | - | 2.03E+08 | 2.03E+08 | 1240 | annot_exons |
| novel_circ_000650 | ENSSSCG00000000064 | 5 | + | 7016400 | 7017797 | 1398 | antisense |
| novel_circ_000651 | ENSSSCG00000017672 | 12 | + | 36391790 | 36392716 | 503 | annot_exons |
| novel_circ_000652 | NA | 15 | - | 84282611 | 84283518 | 908 | intergenic |
| novel_circ_000653 | NA | 13 | + | 23870848 | 23871330 | 483 | intergenic |
| novel_circ_000654 | ENSSSCG00000040397 | 6 | - | 1.06E+08 | 1.06E+08 | 438 | antisense |
| novel_circ_000655 | ENSSSCG00000001808 | 7 | + | 52354140 | 52354328 | 189 | one_exon |
| novel_circ_000656 | ENSSSCG00000038397 | 4 | + | 2874096 | 2887088 | 6307 | exon_intron |
| novel_circ_000657 | ENSSSCG00000028662 | 5 | - | 52443950 | 52452918 | 599 | annot_exons |
| novel_circ_000658 | ENSSSCG00000033272 | 18 | - | 1475203 | 1481335 | 372 | annot_exons |
| novel_circ_000659 | ENSSSCG00000000549 | 5 | - | 46036612 | 46038107 | 438 | annot_exons |
| novel_circ_000660 | NA | 15 | + | 84292117 | 84294697 | 2581 | intergenic |
| novel_circ_000661 | ENSSSCG00000011752 | 13 | + | 1.11E+08 | 1.11E+08 | 823 | annot_exons |
| novel_circ_000662 | ENSSSCG00000005190 | 1 | + | 2.08E+08 | 2.08E+08 | 338 | annot_exons |
| novel_circ_000663 | ENSSSCG00000040855 | 16 | + | 26690558 | 26698399 | 1427 | exon_intron |
| novel_circ_000664 | ENSSSCG00000021466 | 18 | + | 32815047 | 32840017 | 313 | annot_exons |
| novel_circ_000665 | ENSSSCG00000014114 | 2 | + | 88496660 | 88498341 | 1682 | intronic |
| novel_circ_000666 | ENSSSCG00000016949 | 16 | + | 44156269 | 44166529 | 488 | annot_exons |
| novel_circ_000667 | ENSSSCG00000026153 | 9 | + | 36537603 | 36540781 | 361 | annot_exons |
| novel_circ_000668 | ENSSSCG00000026644 | 2 | + | 1.37E+08 | 1.37E+08 | 729 | annot_exons |
| novel_circ_000669 | ENSSSCG00000008466 | 3 | + | 97979211 | 97981059 | 1849 | one_exon |
| novel_circ_000670 | ENSSSCG00000014014 | 2 | + | 78558112 | 78608804 | 15652 | exon_intron |
| novel_circ_000671 | ENSSSCG00000009887 | 14 | + | 39651467 | 39661539 | 622 | annot_exons |
| novel_circ_000672 | ENSSSCG00000000877 | 5 | - | 83829027 | 83836287 | 669 | annot_exons |
| novel_circ_000673 | ENSSSCG00000008575 | 3 | + | 1.13E+08 | 1.13E+08 | 861 | annot_exons |
| novel_circ_000674 | ENSSSCG00000016872 | 16 | + | 27938485 | 27939068 | 584 | antisense |
| novel_circ_000675 | ENSSSCG00000026457 | 3 | + | 15123893 | 15181125 | 57233 | exon_intron |
| novel_circ_000676 | ENSSSCG00000003488 | 6 | - | 77683512 | 77683699 | 188 | one_exon |
| novel_circ_000677 | ENSSSCG00000011340 | 13 | - | 30472205 | 30473996 | 421 | annot_exons |
| novel_circ_000678 | ENSSSCG00000010465 | 14 | + | 1.03E+08 | 1.03E+08 | 612 | annot_exons |
| novel_circ_000679 | ENSSSCG00000010548 | 14 | - | 1.11E+08 | 1.11E+08 | 846 | annot_exons |
| novel_circ_000680 | ENSSSCG00000004358 | 1 | - | 67439729 | 67445988 | 884 | annot_exons |
| novel_circ_000681 | ENSSSCG00000005602 | 1 | + | 2.66E+08 | 2.66E+08 | 1474 | annot_exons |
| novel_circ_000682 | ENSSSCG00000006754 | 4 | + | 1.06E+08 | 1.06E+08 | 1538 | annot_exons |
| novel_circ_000683 | NA | 7 | - | 78551579 | 78551849 | 271 | intergenic |
| novel_circ_000684 | ENSSSCG00000021802 | 1 | - | 2.25E+08 | 2.25E+08 | 650 | annot_exons |
| novel_circ_000685 | ENSSSCG00000005607 | 1 | + | 2.68E+08 | 2.68E+08 | 227 | annot_exons |
| novel_circ_000686 | ENSSSCG00000005366 | 1 | + | 2.4E+08 | 2.4E+08 | 418 | annot_exons |
| novel_circ_000687 | ENSSSCG00000009524 | 11 | - | 69379816 | 69436664 | 1825 | annot_exons |
| novel_circ_000688 | ENSSSCG00000031705 | 16 | - | 18379579 | 18387297 | 247 | annot_exons |
| novel_circ_000689 | ENSSSCG00000010190 | 14 | + | 60415086 | 60415565 | 480 | one_exon |
| novel_circ_000690 | ENSSSCG00000007362 | 17 | + | 46122601 | 46122843 | 243 | one_exon |
| novel_circ_000691 | ENSSSCG00000026806 | 9 | + | 1.24E+08 | 1.24E+08 | 663 | annot_exons |
| novel_circ_000692 | ENSSSCG00000009142 | 8 | - | 1.13E+08 | 1.13E+08 | 698 | annot_exons |
| novel_circ_000693 | ENSSSCG00000010863 | 10 | + | 15051106 | 15085471 | 2670 | annot_exons |
| novel_circ_000694 | ENSSSCG00000011023 | 10 | - | 41724285 | 41730537 | 329 | annot_exons |
| novel_circ_000695 | ENSSSCG00000009281 | 11 | + | 2366496 | 2376189 | 317 | annot_exons |
| novel_circ_000696 | ENSSSCG00000036669 | 2 | - | 9213079 | 9213711 | 633 | antisense |
| novel_circ_000697 | ENSSSCG00000004306 | 1 | + | 56001862 | 56006189 | 312 | annot_exons |
| novel_circ_000698 | ENSSSCG00000034191 | 2 | + | 42947422 | 43023950 | 876 | annot_exons |
| novel_circ_000699 | NA | 15 | - | 84249776 | 84249992 | 217 | intergenic |
| novel_circ_000700 | ENSSSCG00000027374 | 6 | - | 1.69E+08 | 1.69E+08 | 219 | annot_exons |
| novel_circ_000701 | ENSSSCG00000036828 | 13 | + | 1.35E+08 | 1.35E+08 | 525 | annot_exons |
| novel_circ_000702 | ENSSSCG00000025221 | 18 | + | 3098530 | 3103597 | 694 | annot_exons |
| novel_circ_000703 | ENSSSCG00000031459 | 2 | + | 4743317 | 4752017 | 750 | annot_exons |
| novel_circ_000704 | ENSSSCG00000000784 | 5 | + | 71826801 | 71830059 | 245 | annot_exons |
| novel_circ_000705 | NA | 15 | + | 84273978 | 84275003 | 1026 | intergenic |
| novel_circ_000706 | ENSSSCG00000037979 | 12 | + | 19920599 | 19922487 | 619 | annot_exons |
| novel_circ_000707 | ENSSSCG00000003660 | 6 | - | 95393749 | 95395073 | 310 | annot_exons |
| novel_circ_000708 | ENSSSCG00000010358 | 14 | + | 87720164 | 87720504 | 341 | one_exon |
| novel_circ_000709 | ENSSSCG00000021118 | 3 | + | 31258831 | 31266844 | 608 | annot_exons |
| novel_circ_000710 | ENSSSCG00000023001 | 8 | - | 18861488 | 18879156 | 309 | annot_exons |
| novel_circ_000711 | ENSSSCG00000037811 | 14 | - | 31204459 | 31204662 | 204 | one_exon |
| novel_circ_000712 | ENSSSCG00000014269 | 2 | - | 1.34E+08 | 1.34E+08 | 262 | annot_exons |
| novel_circ_000713 | ENSSSCG00000029633 | 6 | + | 1.57E+08 | 1.57E+08 | 336 | annot_exons |
| novel_circ_000714 | ENSSSCG00000010928 | 10 | - | 24776756 | 24779098 | 341 | annot_exons |
| novel_circ_000715 | ENSSSCG00000004561 | 1 | + | 1.08E+08 | 1.08E+08 | 279 | annot_exons |
| novel_circ_000716 | ENSSSCG00000010957 | 10 | + | 29569850 | 29573233 | 338 | annot_exons |
| novel_circ_000717 | ENSSSCG00000006089 | 4 | - | 40327110 | 40339022 | 566 | annot_exons |
| novel_circ_000718 | ENSSSCG00000003591 | 6 | - | 87628833 | 87630082 | 288 | annot_exons |
| novel_circ_000719 | ENSSSCG00000006137 | 4 | - | 50433434 | 50433773 | 340 | one_exon |
| novel_circ_000720 | ENSSSCG00000010811 | 10 | + | 5788969 | 5797656 | 321 | annot_exons |
| novel_circ_000721 | ENSSSCG00000011653 | 13 | + | 77255763 | 77289518 | 473 | exon_intron |
| novel_circ_000722 | ENSSSCG00000002414 | 7 | - | 1.04E+08 | 1.04E+08 | 237 | annot_exons |
| novel_circ_000723 | ENSSSCG00000011632 | 13 | - | 73859561 | 73863238 | 657 | annot_exons |
| novel_circ_000724 | ENSSSCG00000010569 | 14 | - | 1.13E+08 | 1.13E+08 | 544 | annot_exons |
| novel_circ_000725 | ENSSSCG00000017752 | 12 | + | 43892243 | 43899845 | 3090 | annot_exons |
| novel_circ_000726 | ENSSSCG00000028585 | 9 | - | 1.07E+08 | 1.07E+08 | 9710 | antisense |
| novel_circ_000727 | ENSSSCG00000016618 | 18 | - | 25673846 | 25679887 | 558 | annot_exons |
| novel_circ_000728 | ENSSSCG00000010628 | 14 | + | 1.21E+08 | 1.21E+08 | 256 | annot_exons |
| novel_circ_000729 | ENSSSCG00000007280 | 17 | + | 37887802 | 37901251 | 750 | annot_exons |
| novel_circ_000730 | ENSSSCG00000028886 | 9 | - | 119541 | 122749 | 730 | annot_exons |
| novel_circ_000731 | ENSSSCG00000009069 | 8 | - | 95933769 | 95939551 | 494 | annot_exons |
| novel_circ_000732 | ENSSSCG00000025592 | 13 | + | 1.27E+08 | 1.27E+08 | 255 | one_exon |
| novel_circ_000733 | ENSSSCG00000002844 | 6 | - | 37091577 | 37093779 | 237 | annot_exons |
| novel_circ_000734 | ENSSSCG00000007280 | 17 | + | 37831734 | 37862678 | 1228 | annot_exons |
| novel_circ_000735 | ENSSSCG00000015668 | 15 | + | 4136904 | 4142592 | 2660 | exon_intron |
| novel_circ_000736 | ENSSSCG00000015428 | 9 | - | 1.05E+08 | 1.05E+08 | 359 | annot_exons |
| novel_circ_000737 | ENSSSCG00000015052 | 9 | - | 41462880 | 41464576 | 211 | annot_exons |
| novel_circ_000738 | ENSSSCG00000025349 | 13 | + | 1.36E+08 | 1.36E+08 | 196 | annot_exons |
| novel_circ_000739 | ENSSSCG00000006201 | 4 | + | 67537059 | 67538591 | 310 | annot_exons |
| novel_circ_000740 | ENSSSCG00000011794 | 13 | + | 1.23E+08 | 1.23E+08 | 364 | annot_exons |
| novel_circ_000741 | ENSSSCG00000009047 | 8 | - | 84198495 | 84200866 | 337 | annot_exons |
| novel_circ_000742 | ENSSSCG00000015746 | 15 | + | 33173052 | 33184938 | 1282 | annot_exons |
| novel_circ_000743 | ENSSSCG00000004979 | 1 | - | 1.69E+08 | 1.69E+08 | 341 | annot_exons |
| novel_circ_000744 | ENSSSCG00000010170 | 14 | - | 59030578 | 59033164 | 2587 | exon_intron |
| novel_circ_000745 | ENSSSCG00000035873 | 14 | + | 35715229 | 35718894 | 3101 | annot_exons |
| novel_circ_000746 | ENSSSCG00000016157 | 15 | - | 1.13E+08 | 1.13E+08 | 215 | one_exon |
| novel_circ_000747 | ENSSSCG00000001878 | 7 | + | 58112449 | 58140971 | 905 | annot_exons |
| novel_circ_000748 | ENSSSCG00000004624 | 1 | - | 1.2E+08 | 1.2E+08 | 1503 | annot_exons |
| novel_circ_000749 | ENSSSCG00000017904 | 12 | - | 51956216 | 51957996 | 534 | annot_exons |
| novel_circ_000750 | ENSSSCG00000013860 | 2 | + | 61073081 | 61074264 | 429 | annot_exons |
| novel_circ_000751 | ENSSSCG00000027293 | 16 | + | 49097121 | 49107340 | 3739 | annot_exons |
| novel_circ_000752 | ENSSSCG00000004857 | 1 | - | 1.46E+08 | 1.46E+08 | 9644 | exon_intron |
| novel_circ_000753 | ENSSSCG00000006115 | 4 | + | 44625779 | 44693355 | 1092 | annot_exons |
| novel_circ_000754 | ENSSSCG00000007805 | 3 | + | 18560272 | 18563733 | 456 | annot_exons |
| novel_circ_000755 | ENSSSCG00000009010 | 8 | - | 76211929 | 76237387 | 642 | annot_exons |
| novel_circ_000756 | ENSSSCG00000017311 | 12 | - | 17122167 | 17126300 | 453 | annot_exons |
| novel_circ_000757 | ENSSSCG00000017155 | 12 | + | 2288303 | 2292225 | 417 | annot_exons |
| novel_circ_000758 | ENSSSCG00000009142 | 8 | - | 1.13E+08 | 1.13E+08 | 599 | annot_exons |
| novel_circ_000759 | NA | 2 | - | 66607322 | 66607703 | 382 | intergenic |
| novel_circ_000760 | ENSSSCG00000030095 | 9 | + | 41724766 | 41745104 | 20339 | exon_intron |
| novel_circ_000761 | ENSSSCG00000034730 | 1 | - | 96515535 | 96516009 | 475 | one_exon |
| novel_circ_000762 | ENSSSCG00000008513 | 3 | - | 1.07E+08 | 1.07E+08 | 626 | annot_exons |
| novel_circ_000763 | ENSSSCG00000015943 | 15 | + | 77115558 | 77121805 | 679 | annot_exons |
| novel_circ_000764 | ENSSSCG00000016954 | 16 | + | 44484459 | 44506007 | 898 | annot_exons |
| novel_circ_000765 | ENSSSCG00000007253 | 17 | + | 36420497 | 36420831 | 335 | one_exon |
| novel_circ_000766 | ENSSSCG00000015695 | 15 | - | 16786136 | 16788456 | 600 | annot_exons |
| novel_circ_000767 | ENSSSCG00000015140 | 9 | - | 49983015 | 49983512 | 294 | annot_exons |
| novel_circ_000768 | ENSSSCG00000016794 | 16 | - | 5947961 | 5948292 | 265 | annot_exons |
| novel_circ_000769 | ENSSSCG00000017255 | 12 | + | 11098684 | 11106997 | 960 | annot_exons |
| novel_circ_000770 | ENSSSCG00000017806 | 12 | + | 47077502 | 47083715 | 6214 | antisense |
| novel_circ_000771 | ENSSSCG00000011599 | 13 | + | 70029821 | 70032723 | 452 | annot_exons |
| novel_circ_000772 | ENSSSCG00000004538 | 1 | + | 1.06E+08 | 1.06E+08 | 307 | annot_exons |
| novel_circ_000773 | ENSSSCG00000017513 | 12 | + | 23869313 | 23874195 | 495 | annot_exons |
| novel_circ_000774 | ENSSSCG00000004415 | 1 | + | 75285237 | 75304481 | 870 | annot_exons |
| novel_circ_000775 | ENSSSCG00000040887 | 6 | - | 34743079 | 34751712 | 529 | annot_exons |
| novel_circ_000776 | ENSSSCG00000009230 | 8 | + | 1.34E+08 | 1.34E+08 | 7845 | exon_intron |
| novel_circ_000777 | ENSSSCG00000046165 | 18 | - | 8084933 | 8086118 | 400 | annot_exons |
| novel_circ_000778 | ENSSSCG00000016349 | 15 | - | 1.39E+08 | 1.39E+08 | 980 | annot_exons |
| novel_circ_000779 | ENSSSCG00000023596 | 14 | - | 60980020 | 61016166 | 23339 | exon_intron |
| novel_circ_000780 | ENSSSCG00000017246 | 12 | - | 7709937 | 7712667 | 707 | exon_intron |
| novel_circ_000781 | ENSSSCG00000015940 | 15 | + | 76177104 | 76198564 | 621 | annot_exons |
| novel_circ_000782 | ENSSSCG00000016835 | 16 | + | 21480458 | 21485938 | 509 | annot_exons |
| novel_circ_000783 | ENSSSCG00000017378 | 12 | - | 19572244 | 19573449 | 437 | annot_exons |
| novel_circ_000784 | ENSSSCG00000004358 | 1 | - | 67540530 | 67550055 | 8909 | exon_intron |
| novel_circ_000785 | ENSSSCG00000004499 | 1 | - | 95648999 | 95685846 | 450 | annot_exons |
| novel_circ_000786 | ENSSSCG00000015743 | 15 | - | 31472458 | 31473618 | 1161 | exon_intron |
| novel_circ_000787 | ENSSSCG00000029920 | 8 | + | 87800823 | 87820356 | 405 | annot_exons |
| novel_circ_000788 | ENSSSCG00000006115 | 4 | + | 44625779 | 44711128 | 1631 | annot_exons |
| novel_circ_000789 | ENSSSCG00000011791 | 13 | + | 1.23E+08 | 1.23E+08 | 1095 | annot_exons |
| novel_circ_000790 | ENSSSCG00000009570 | 11 | + | 458635 | 468238 | 1306 | annot_exons |
| novel_circ_000791 | ENSSSCG00000031903 | 2 | + | 1253026 | 1253299 | 274 | intronic |
| novel_circ_000792 | ENSSSCG00000027403 | 8 | - | 55614208 | 55620179 | 966 | annot_exons |
| novel_circ_000793 | ENSSSCG00000008363 | 3 | - | 77378253 | 77378404 | 152 | one_exon |
| novel_circ_000794 | ENSSSCG00000007080 | 17 | - | 25124204 | 25125658 | 301 | annot_exons |
| novel_circ_000795 | ENSSSCG00000030467 | 13 | - | 1.09E+08 | 1.09E+08 | 562 | annot_exons |
| novel_circ_000796 | ENSSSCG00000024570 | 2 | - | 73841767 | 73858570 | 371 | annot_exons |
| novel_circ_000797 | ENSSSCG00000016395 | 15 | - | 793390 | 798569 | 365 | annot_exons |
| novel_circ_000798 | ENSSSCG00000006487 | 4 | + | 93738637 | 93752407 | 1326 | annot_exons |
| novel_circ_000799 | ENSSSCG00000008192 | 3 | + | 56396003 | 56398361 | 240 | annot_exons |
| novel_circ_000800 | ENSSSCG00000007874 | 3 | - | 28412294 | 28417579 | 311 | annot_exons |
| novel_circ_000801 | ENSSSCG00000016017 | 15 | + | 88235138 | 88235686 | 245 | annot_exons |
| novel_circ_000802 | ENSSSCG00000010849 | 10 | - | 12721730 | 12725565 | 316 | annot_exons |
| novel_circ_000803 | ENSSSCG00000031592 | 1 | + | 2.48E+08 | 2.48E+08 | 5768 | annot_exons |
| novel_circ_000804 | ENSSSCG00000021380 | 13 | + | 1.34E+08 | 1.34E+08 | 238 | annot_exons |
| novel_circ_000805 | ENSSSCG00000034493 | 13 | - | 1.6E+08 | 1.6E+08 | 746 | annot_exons |
| novel_circ_000806 | ENSSSCG00000015410 | 9 | - | 1.02E+08 | 1.02E+08 | 840 | annot_exons |
| novel_circ_000807 | NA | 13 | + | 12800954 | 12806862 | 5909 | intergenic |
| novel_circ_000808 | ENSSSCG00000034618 | 6 | - | 91256537 | 91266197 | 999 | annot_exons |
| novel_circ_000809 | ENSSSCG00000009029 | 8 | - | 80809383 | 80876761 | 689 | annot_exons |
| novel_circ_000810 | ENSSSCG00000006872 | 4 | - | 1.18E+08 | 1.18E+08 | 1019 | annot_exons |
| novel_circ_000811 | ENSSSCG00000028663 | 7 | + | 96423793 | 96429731 | 276 | annot_exons |
| novel_circ_000812 | ENSSSCG00000009652 | 14 | - | 9497312 | 9498411 | 285 | annot_exons |
| novel_circ_000813 | ENSSSCG00000013298 | 2 | - | 26039163 | 26060804 | 722 | annot_exons |
| novel_circ_000814 | ENSSSCG00000010483 | 14 | + | 1.06E+08 | 1.06E+08 | 317 | one_exon |
| novel_circ_000815 | ENSSSCG00000010679 | 14 | - | 1.29E+08 | 1.29E+08 | 402 | annot_exons |
| novel_circ_000816 | ENSSSCG00000009401 | 11 | - | 19260010 | 19270035 | 580 | annot_exons |
| novel_circ_000817 | ENSSSCG00000004367 | 1 | - | 71382432 | 71384471 | 832 | annot_exons |
| novel_circ_000818 | ENSSSCG00000023593 | 8 | - | 1.32E+08 | 1.32E+08 | 2405 | annot_exons |
| novel_circ_000819 | ENSSSCG00000009220 | 8 | - | 1.31E+08 | 1.31E+08 | 336 | annot_exons |
| novel_circ_000820 | ENSSSCG00000012474 | X | + | 78865408 | 78870311 | 355 | annot_exons |
| novel_circ_000821 | ENSSSCG00000015940 | 15 | + | 76135842 | 76170299 | 470 | annot_exons |
| novel_circ_000822 | ENSSSCG00000015743 | 15 | - | 31417943 | 31473618 | 692 | annot_exons |
| novel_circ_000823 | ENSSSCG00000009823 | 14 | - | 31861669 | 31861904 | 236 | one_exon |
| novel_circ_000824 | ENSSSCG00000008801 | 8 | + | 33098958 | 33102443 | 346 | annot_exons |
| novel_circ_000825 | ENSSSCG00000011301 | 13 | - | 28151062 | 28151391 | 330 | one_exon |
| novel_circ_000826 | ENSSSCG00000006762 | 4 | + | 1.07E+08 | 1.07E+08 | 2797 | annot_exons |
| novel_circ_000827 | ENSSSCG00000004207 | 1 | - | 33982907 | 34082131 | 1225 | annot_exons |
| novel_circ_000828 | ENSSSCG00000023738 | 11 | - | 21782009 | 21786602 | 336 | annot_exons |
| novel_circ_000829 | NA | 18 | - | 2648872 | 2654761 | 5890 | intergenic |
| novel_circ_000830 | ENSSSCG00000005934 | 4 | + | 3172175 | 3184987 | 656 | annot_exons |
| novel_circ_000831 | ENSSSCG00000030343 | 16 | - | 34798441 | 34800600 | 303 | annot_exons |
| novel_circ_000832 | ENSSSCG00000026466 | 17 | - | 13900084 | 13915744 | 374 | annot_exons |
| novel_circ_000833 | ENSSSCG00000011593 | 13 | - | 69061565 | 69062499 | 935 | one_exon |
| novel_circ_000834 | ENSSSCG00000012434 | X | - | 61645925 | 61651015 | 261 | annot_exons |
| novel_circ_000835 | ENSSSCG00000003872 | 6 | + | 1.61E+08 | 1.61E+08 | 652 | annot_exons |
| novel_circ_000836 | ENSSSCG00000021354 | 18 | + | 13918870 | 13923524 | 440 | annot_exons |
| novel_circ_000837 | ENSSSCG00000016568 | 18 | - | 19302353 | 19320896 | 408 | annot_exons |
| novel_circ_000838 | ENSSSCG00000006619 | 4 | - | 97566524 | 97595259 | 838 | annot_exons |
| novel_circ_000839 | ENSSSCG00000008753 | 8 | - | 16681471 | 16755944 | 2224 | annot_exons |
| novel_circ_000840 | ENSSSCG00000005180 | 1 | + | 2.06E+08 | 2.06E+08 | 1970 | one_exon |
| novel_circ_000841 | ENSSSCG00000034191 | 2 | + | 42933470 | 42947501 | 201 | annot_exons |
| novel_circ_000842 | ENSSSCG00000005015 | 1 | - | 1.8E+08 | 1.8E+08 | 771 | annot_exons |
| novel_circ_000843 | ENSSSCG00000002330 | 7 | + | 94553559 | 94558109 | 359 | annot_exons |
| novel_circ_000844 | ENSSSCG00000002755 | 6 | - | 17352018 | 17356850 | 557 | annot_exons |
| novel_circ_000845 | ENSSSCG00000000846 | 5 | - | 80400947 | 80407924 | 519 | annot_exons |
| novel_circ_000846 | ENSSSCG00000000784 | 5 | + | 71822699 | 71834961 | 475 | annot_exons |
| novel_circ_000847 | NA | 6 | - | 47386892 | 47388138 | 1247 | intergenic |
| novel_circ_000848 | ENSSSCG00000025179 | 5 | + | 63129448 | 63130844 | 375 | annot_exons |
| novel_circ_000849 | NA | 14 | - | 66079949 | 66082111 | 2163 | intergenic |
| novel_circ_000850 | ENSSSCG00000031249 | 13 | + | 2.07E+08 | 2.07E+08 | 331 | antisense |
| novel_circ_000851 | ENSSSCG00000032097 | 5 | + | 59877187 | 59935574 | 38740 | exon_intron |
| novel_circ_000852 | ENSSSCG00000007235 | 17 | + | 35461000 | 35462204 | 258 | annot_exons |
| novel_circ_000853 | ENSSSCG00000017735 | 12 | - | 42741473 | 42750757 | 419 | annot_exons |
| novel_circ_000854 | ENSSSCG00000023357 | 8 | + | 56059089 | 56059440 | 352 | one_exon |
| novel_circ_000855 | ENSSSCG00000017125 | 12 | + | 568688 | 569329 | 642 | antisense |
| novel_circ_000856 | NA | 16 | - | 74708626 | 74708823 | 198 | intergenic |
| novel_circ_000857 | ENSSSCG00000007280 | 17 | + | 37838591 | 37881223 | 1087 | annot_exons |
| novel_circ_000858 | ENSSSCG00000034418 | 5 | - | 59810017 | 59812114 | 343 | annot_exons |
| novel_circ_000859 | ENSSSCG00000017791 | 12 | - | 45870808 | 45887992 | 291 | annot_exons |
| novel_circ_000860 | ENSSSCG00000017549 | 12 | - | 25605643 | 25607747 | 277 | annot_exons |
| novel_circ_000861 | ENSSSCG00000003398 | 6 | + | 70437449 | 70453863 | 835 | annot_exons |
| novel_circ_000862 | ENSSSCG00000009335 | 11 | + | 7862263 | 7886903 | 524 | annot_exons |
| novel_circ_000863 | ENSSSCG00000015115 | 9 | + | 46486910 | 46488853 | 608 | annot_exons |
| novel_circ_000864 | ENSSSCG00000030478 | 5 | - | 89628594 | 89631789 | 319 | annot_exons |
| novel_circ_000865 | ENSSSCG00000008772 | 8 | - | 29136919 | 29151669 | 506 | annot_exons |
| novel_circ_000866 | ENSSSCG00000014304 | 2 | + | 1.37E+08 | 1.37E+08 | 350 | annot_exons |
| novel_circ_000867 | ENSSSCG00000028035 | 9 | + | 64522730 | 64523023 | 294 | intronic |
| novel_circ_000868 | ENSSSCG00000008382 | 3 | + | 80521466 | 80528430 | 422 | annot_exons |
| novel_circ_000869 | ENSSSCG00000024813 | 12 | - | 15269531 | 15274897 | 5367 | exon_intron |
| novel_circ_000870 | ENSSSCG00000011765 | 13 | + | 1.18E+08 | 1.18E+08 | 608 | one_exon |
| novel_circ_000871 | ENSSSCG00000009886 | 14 | - | 39560043 | 39562284 | 610 | annot_exons |
| novel_circ_000872 | ENSSSCG00000017513 | 12 | + | 23857979 | 23869452 | 645 | annot_exons |
| novel_circ_000873 | ENSSSCG00000006090 | 4 | + | 40379783 | 40393660 | 996 | annot_exons |
| novel_circ_000874 | ENSSSCG00000009655 | 14 | - | 9834835 | 9838951 | 519 | annot_exons |
| novel_circ_000875 | ENSSSCG00000003512 | 6 | - | 79089250 | 79100041 | 410 | exon_intron |
| novel_circ_000876 | ENSSSCG00000006083 | 4 | - | 38957345 | 38967502 | 392 | annot_exons |
| novel_circ_000877 | ENSSSCG00000012713 | X | - | 1.14E+08 | 1.14E+08 | 399 | annot_exons |
| novel_circ_000878 | ENSSSCG00000000496 | 5 | + | 33860701 | 33862629 | 505 | annot_exons |
| novel_circ_000879 | ENSSSCG00000022222 | 15 | + | 82069808 | 82126919 | 23881 | exon_intron |
| novel_circ_000880 | ENSSSCG00000024809 | X | + | 6996029 | 6997645 | 869 | annot_exons |
| novel_circ_000881 | ENSSSCG00000004552 | 1 | + | 1.08E+08 | 1.08E+08 | 1974 | antisense |
| novel_circ_000882 | ENSSSCG00000014578 | 9 | + | 295808 | 300977 | 834 | annot_exons |
| novel_circ_000883 | ENSSSCG00000022866 | 2 | + | 8947587 | 8949544 | 485 | annot_exons |
| novel_circ_000884 | ENSSSCG00000003728 | 6 | - | 1.16E+08 | 1.16E+08 | 282 | annot_exons |
| novel_circ_000885 | ENSSSCG00000010928 | 10 | - | 24768866 | 24770435 | 315 | annot_exons |
| novel_circ_000886 | ENSSSCG00000009268 | 11 | - | 864719 | 922044 | 776 | annot_exons |
| novel_circ_000887 | NA | 14 | - | 12913616 | 12917849 | 4234 | intergenic |
| novel_circ_000888 | ENSSSCG00000006388 | 4 | + | 90235723 | 90238988 | 1003 | annot_exons |
| novel_circ_000889 | ENSSSCG00000010092 | 14 | - | 50410556 | 50413833 | 498 | annot_exons |
| novel_circ_000890 | ENSSSCG00000035581 | 18 | - | 54149862 | 54192387 | 240 | annot_exons |
| novel_circ_000891 | ENSSSCG00000030396 | 8 | + | 87488517 | 87508914 | 604 | annot_exons |
| novel_circ_000892 | ENSSSCG00000026161 | 3 | + | 1.25E+08 | 1.25E+08 | 761 | exon_intron |
| novel_circ_000893 | ENSSSCG00000000555 | 5 | + | 47048736 | 47049858 | 330 | annot_exons |
| novel_circ_000894 | ENSSSCG00000002245 | 7 | + | 80343073 | 80346071 | 444 | annot_exons |
| novel_circ_000895 | ENSSSCG00000007331 | 17 | - | 40253732 | 40256075 | 404 | annot_exons |
| novel_circ_000896 | ENSSSCG00000038465 | 6 | + | 98212693 | 98213311 | 619 | antisense |
| novel_circ_000897 | ENSSSCG00000006346 | 4 | - | 88723352 | 88732517 | 397 | annot_exons |
| novel_circ_000898 | ENSSSCG00000006141 | 4 | + | 51228255 | 51234435 | 6181 | antisense |
| novel_circ_000899 | ENSSSCG00000017602 | 12 | + | 31358562 | 31388511 | 333 | annot_exons |
| novel_circ_000900 | ENSSSCG00000026960 | 1 | + | 1.15E+08 | 1.15E+08 | 281 | annot_exons |
| novel_circ_000901 | ENSSSCG00000003976 | 6 | - | 1.7E+08 | 1.7E+08 | 2617 | annot_exons |
| novel_circ_000902 | ENSSSCG00000028635 | 3 | - | 11850831 | 11851900 | 1070 | intronic |
| novel_circ_000903 | ENSSSCG00000011681 | 13 | + | 83468032 | 83470719 | 973 | exon_intron |
| novel_circ_000904 | ENSSSCG00000015402 | 9 | + | 98174812 | 98177186 | 129 | annot_exons |
| novel_circ_000905 | ENSSSCG00000004082 | 1 | + | 14087204 | 14093357 | 969 | annot_exons |
| novel_circ_000906 | ENSSSCG00000003700 | 6 | - | 1.07E+08 | 1.07E+08 | 2777 | annot_exons |
| novel_circ_000907 | NA | 3 | - | 85570718 | 85584548 | 13831 | intergenic |
| novel_circ_000908 | ENSSSCG00000009860 | 14 | - | 35484129 | 35487748 | 404 | annot_exons |
| novel_circ_000909 | ENSSSCG00000007805 | 3 | + | 18559247 | 18560385 | 324 | annot_exons |
| novel_circ_000910 | ENSSSCG00000004617 | 1 | + | 1.19E+08 | 1.19E+08 | 556 | exon_intron |
| novel_circ_000911 | ENSSSCG00000011765 | 13 | + | 1.18E+08 | 1.18E+08 | 879 | annot_exons |
| novel_circ_000912 | ENSSSCG00000008335 | 3 | - | 72605356 | 72606470 | 179 | annot_exons |
| novel_circ_000913 | ENSSSCG00000045565 | 9 | - | 1.02E+08 | 1.02E+08 | 4087 | exon_intron |
| novel_circ_000914 | ENSSSCG00000005249 | 1 | + | 2.23E+08 | 2.23E+08 | 604 | annot_exons |
| novel_circ_000915 | ENSSSCG00000015430 | 9 | + | 1.05E+08 | 1.05E+08 | 300 | annot_exons |
| novel_circ_000916 | ENSSSCG00000011117 | 10 | + | 59913525 | 59919743 | 613 | annot_exons |
| novel_circ_000917 | ENSSSCG00000013322 | 2 | + | 29279514 | 29285842 | 350 | annot_exons |
| novel_circ_000918 | ENSSSCG00000006305 | 4 | + | 83148304 | 83151377 | 406 | annot_exons |
| novel_circ_000919 | ENSSSCG00000017732 | 12 | - | 42592878 | 42596350 | 502 | annot_exons |
| novel_circ_000920 | ENSSSCG00000011845 | 13 | + | 1.34E+08 | 1.34E+08 | 491 | annot_exons |
| novel_circ_000921 | ENSSSCG00000035657 | AEMK02000328.1 | - | 269331 | 275679 | 465 | annot_exons |
| novel_circ_000922 | ENSSSCG00000003673 | 6 | - | 96686732 | 96688640 | 392 | annot_exons |
| novel_circ_000923 | ENSSSCG00000010627 | 14 | + | 1.21E+08 | 1.21E+08 | 677 | annot_exons |
| novel_circ_000924 | ENSSSCG00000011068 | 10 | - | 48781955 | 48788374 | 502 | annot_exons |
| novel_circ_000925 | NA | 15 | + | 84422891 | 84423153 | 263 | intergenic |
| novel_circ_000926 | ENSSSCG00000015842 | 15 | + | 54310541 | 54317753 | 755 | annot_exons |
| novel_circ_000927 | ENSSSCG00000006115 | 4 | + | 44710287 | 44727167 | 548 | annot_exons |
| novel_circ_000928 | ENSSSCG00000003976 | 6 | - | 1.7E+08 | 1.7E+08 | 1709 | annot_exons |
| novel_circ_000929 | ENSSSCG00000010654 | 14 | + | 1.26E+08 | 1.26E+08 | 479 | annot_exons |
| novel_circ_000930 | ENSSSCG00000010144 | 14 | - | 54704317 | 54716478 | 3869 | annot_exons |
| novel_circ_000931 | ENSSSCG00000014394 | 2 | + | 1.44E+08 | 1.44E+08 | 304 | annot_exons |
| novel_circ_000932 | ENSSSCG00000009713 | 14 | - | 20116799 | 20116974 | 176 | one_exon |
| novel_circ_000933 | ENSSSCG00000001878 | 7 | + | 58112449 | 58123585 | 465 | annot_exons |
| novel_circ_000934 | ENSSSCG00000001755 | 7 | - | 47467395 | 47469067 | 507 | annot_exons |
| novel_circ_000935 | ENSSSCG00000001004 | 7 | - | 2091526 | 2096730 | 259 | annot_exons |
| novel_circ_000936 | ENSSSCG00000031188 | 6 | - | 1.61E+08 | 1.61E+08 | 59952 | exon_intron |
| novel_circ_000937 | ENSSSCG00000028771 | 1 | + | 1.38E+08 | 1.38E+08 | 733 | annot_exons |
| novel_circ_000938 | ENSSSCG00000009435 | 11 | - | 25573447 | 25584052 | 409 | annot_exons |
| novel_circ_000939 | ENSSSCG00000038222 | 14 | - | 1.29E+08 | 1.29E+08 | 289 | annot_exons |
| novel_circ_000940 | NA | 12 | + | 45956104 | 45956346 | 243 | intergenic |
| novel_circ_000941 | ENSSSCG00000002412 | 7 | - | 1.04E+08 | 1.04E+08 | 1839 | one_exon |
| novel_circ_000942 | ENSSSCG00000010209 | 14 | - | 62667130 | 62701927 | 331 | annot_exons |
| novel_circ_000943 | NA | 15 | + | 84292130 | 84292241 | 112 | intergenic |
| novel_circ_000944 | ENSSSCG00000006089 | 4 | - | 40330385 | 40345193 | 578 | annot_exons |
| novel_circ_000945 | ENSSSCG00000035824 | 13 | - | 34813672 | 34816728 | 368 | annot_exons |
| novel_circ_000946 | ENSSSCG00000017046 | 16 | + | 64650293 | 64669823 | 258 | annot_exons |
| novel_circ_000947 | ENSSSCG00000001596 | 7 | - | 35293227 | 35297139 | 563 | annot_exons |
| novel_circ_000948 | ENSSSCG00000011678 | 13 | - | 83143347 | 83144616 | 277 | annot_exons |
| novel_circ_000949 | ENSSSCG00000031262 | 4 | + | 99348578 | 99348853 | 174 | annot_exons |
| novel_circ_000950 | ENSSSCG00000016157 | 15 | - | 1.13E+08 | 1.13E+08 | 396 | annot_exons |
| novel_circ_000951 | ENSSSCG00000005282 | 1 | + | 2.3E+08 | 2.3E+08 | 1015 | annot_exons |
| novel_circ_000952 | ENSSSCG00000016291 | 15 | + | 1.33E+08 | 1.33E+08 | 226 | annot_exons |
| novel_circ_000953 | ENSSSCG00000004082 | 1 | + | 14046058 | 14047923 | 1866 | exon_intron |
| novel_circ_000954 | ENSSSCG00000042541 | 4 | - | 1.3E+08 | 1.3E+08 | 2440 | intronic |
| novel_circ_000955 | ENSSSCG00000032969 | 6 | + | 58194555 | 58198645 | 684 | exon_intron |
| novel_circ_000956 | ENSSSCG00000010357 | 14 | - | 87507974 | 87516162 | 438 | annot_exons |
| novel_circ_000957 | ENSSSCG00000006864 | 4 | - | 1.18E+08 | 1.18E+08 | 458 | annot_exons |
| novel_circ_000958 | ENSSSCG00000013366 | 2 | - | 40819038 | 40820292 | 292 | annot_exons |
| novel_circ_000959 | ENSSSCG00000021038 | 13 | - | 1.8E+08 | 1.8E+08 | 2013 | exon_intron |
| novel_circ_000960 | ENSSSCG00000014578 | 9 | + | 311863 | 312050 | 188 | one_exon |
| novel_circ_000961 | ENSSSCG00000014057 | 2 | - | 81412625 | 81431978 | 1092 | annot_exons |
| novel_circ_000962 | ENSSSCG00000001958 | 7 | + | 64896440 | 64900770 | 340 | annot_exons |
| novel_circ_000963 | ENSSSCG00000028905 | 13 | - | 1.1E+08 | 1.1E+08 | 333 | annot_exons |
| novel_circ_000964 | ENSSSCG00000034191 | 2 | - | 42461532 | 42462129 | 598 | antisense |
| novel_circ_000965 | ENSSSCG00000013366 | 2 | - | 40817322 | 40820292 | 584 | annot_exons |
| novel_circ_000966 | ENSSSCG00000010579 | 14 | + | 1.13E+08 | 1.13E+08 | 173 | annot_exons |
| novel_circ_000967 | ENSSSCG00000012437 | X | + | 62075830 | 62082581 | 757 | annot_exons |
| novel_circ_000968 | ENSSSCG00000017101 | 16 | - | 74328311 | 74366126 | 681 | annot_exons |
| novel_circ_000969 | ENSSSCG00000011229 | 13 | + | 17586906 | 17587193 | 288 | one_exon |
| novel_circ_000970 | ENSSSCG00000023130 | 14 | - | 75008341 | 75025043 | 245 | annot_exons |
| novel_circ_000971 | ENSSSCG00000006504 | 4 | + | 94121443 | 94121930 | 266 | annot_exons |
| novel_circ_000972 | ENSSSCG00000010773 | 14 | - | 1.41E+08 | 1.41E+08 | 653 | annot_exons |
| novel_circ_000973 | ENSSSCG00000023354 | 13 | + | 71406117 | 71407910 | 287 | annot_exons |
| novel_circ_000974 | ENSSSCG00000022417 | 12 | - | 39310277 | 39311632 | 302 | annot_exons |
| novel_circ_000975 | ENSSSCG00000016618 | 18 | - | 25842699 | 25844414 | 302 | annot_exons |
| novel_circ_000976 | NA | 13 | + | 1.24E+08 | 1.24E+08 | 21381 | intergenic |
| novel_circ_000977 | ENSSSCG00000036948 | 10 | - | 23646010 | 23651322 | 5313 | antisense |
| novel_circ_000978 | ENSSSCG00000004524 | 1 | + | 1.01E+08 | 1.01E+08 | 641 | annot_exons |
| novel_circ_000979 | ENSSSCG00000006542 | 4 | + | 95077462 | 95079777 | 2316 | exon_intron |
| novel_circ_000980 | ENSSSCG00000037673 | 3 | - | 28599126 | 28600542 | 542 | annot_exons |
| novel_circ_000981 | NA | 15 | - | 84340166 | 84340610 | 445 | intergenic |
| novel_circ_000982 | ENSSSCG00000028516 | 5 | + | 16309169 | 16320268 | 514 | annot_exons |
| novel_circ_000983 | ENSSSCG00000013445 | 2 | - | 76582777 | 76583208 | 432 | one_exon |
| novel_circ_000984 | ENSSSCG00000009776 | 14 | + | 29595810 | 29597059 | 249 | annot_exons |
| novel_circ_000985 | ENSSSCG00000021042 | 13 | - | 2.03E+08 | 2.03E+08 | 234 | annot_exons |
| novel_circ_000986 | ENSSSCG00000005466 | 1 | - | 2.53E+08 | 2.53E+08 | 286 | annot_exons |
| novel_circ_000987 | ENSSSCG00000017186 | 12 | + | 5284618 | 5285574 | 345 | annot_exons |
| novel_circ_000988 | ENSSSCG00000010900 | 10 | - | 20469603 | 20492105 | 375 | annot_exons |
| novel_circ_000989 | ENSSSCG00000006752 | 4 | + | 1.06E+08 | 1.06E+08 | 301 | annot_exons |
| novel_circ_000990 | ENSSSCG00000025602 | 18 | + | 33845684 | 33847333 | 309 | annot_exons |
| novel_circ_000991 | ENSSSCG00000005467 | 1 | + | 2.53E+08 | 2.53E+08 | 281 | annot_exons |
| novel_circ_000992 | ENSSSCG00000007735 | 3 | - | 16320466 | 16341799 | 549 | annot_exons |
| novel_circ_000993 | ENSSSCG00000034290 | 7 | - | 94172818 | 94178740 | 581 | annot_exons |
| novel_circ_000994 | ENSSSCG00000009684 | 14 | + | 13379691 | 13384817 | 401 | exon_intron |
| novel_circ_000995 | ENSSSCG00000030480 | 13 | + | 2.01E+08 | 2.01E+08 | 941 | annot_exons |
| novel_circ_000996 | ENSSSCG00000015379 | 9 | + | 90682761 | 90695267 | 530 | exon_intron |
| novel_circ_000997 | ENSSSCG00000031271 | 15 | + | 1.05E+08 | 1.05E+08 | 368 | annot_exons |
| novel_circ_000998 | ENSSSCG00000016888 | 16 | - | 32442445 | 32442734 | 290 | one_exon |
| novel_circ_000999 | ENSSSCG00000015537 | 9 | + | 1.22E+08 | 1.22E+08 | 615 | annot_exons |
| novel_circ_001000 | ENSSSCG00000007462 | 17 | + | 51119840 | 51121095 | 1256 | antisense |
| novel_circ_001001 | ENSSSCG00000004958 | 1 | + | 1.66E+08 | 1.66E+08 | 970 | annot_exons |
| novel_circ_001002 | ENSSSCG00000008721 | 8 | + | 2984016 | 2998646 | 368 | annot_exons |
| novel_circ_001003 | ENSSSCG00000017668 | 12 | + | 35991800 | 35999542 | 307 | annot_exons |
| novel_circ_001004 | ENSSSCG00000011752 | 13 | + | 1.11E+08 | 1.11E+08 | 321 | annot_exons |
| novel_circ_001005 | ENSSSCG00000016828 | 16 | + | 20536858 | 20538180 | 454 | annot_exons |
| novel_circ_001006 | NA | 15 | + | 1.21E+08 | 1.21E+08 | 1436 | intergenic |
| novel_circ_001007 | ENSSSCG00000009887 | 14 | + | 39635800 | 39638619 | 283 | annot_exons |
| novel_circ_001008 | ENSSSCG00000000866 | 5 | - | 82571957 | 82585391 | 831 | annot_exons |
| novel_circ_001009 | ENSSSCG00000025406 | 2 | + | 42015142 | 42017066 | 333 | annot_exons |
| novel_circ_001010 | ENSSSCG00000009864 | 14 | + | 36366007 | 36366819 | 367 | annot_exons |
| novel_circ_001011 | ENSSSCG00000004110 | 1 | + | 16533944 | 16559800 | 25857 | antisense |
| novel_circ_001012 | ENSSSCG00000039157 | 15 | + | 29991565 | 29992784 | 223 | annot_exons |
| novel_circ_001013 | ENSSSCG00000009971 | 14 | + | 46180499 | 46194096 | 13598 | exon_intron |
| novel_circ_001014 | ENSSSCG00000004203 | 1 | - | 33271334 | 33278592 | 485 | annot_exons |
| novel_circ_001015 | ENSSSCG00000031273 | 13 | + | 1.58E+08 | 1.58E+08 | 494 | annot_exons |
| novel_circ_001016 | ENSSSCG00000007153 | 17 | - | 32142636 | 32159495 | 1241 | annot_exons |
| novel_circ_001017 | ENSSSCG00000015743 | 15 | - | 31421363 | 31422189 | 219 | annot_exons |
| novel_circ_001018 | ENSSSCG00000008168 | 3 | + | 53042582 | 53048509 | 403 | annot_exons |
| novel_circ_001019 | ENSSSCG00000035824 | 13 | - | 34726535 | 34729422 | 608 | annot_exons |
| novel_circ_001020 | ENSSSCG00000008942 | 8 | + | 67659787 | 67680849 | 482 | annot_exons |
| novel_circ_001021 | ENSSSCG00000006725 | 4 | + | 1.02E+08 | 1.02E+08 | 231 | annot_exons |
| novel_circ_001022 | ENSSSCG00000001680 | 7 | + | 38438110 | 38439128 | 385 | annot_exons |
| novel_circ_001023 | ENSSSCG00000045508 | 1 | + | 2.39E+08 | 2.39E+08 | 910 | annot_exons |
| novel_circ_001024 | ENSSSCG00000015949 | 15 | - | 77939396 | 77942749 | 380 | annot_exons |
| novel_circ_001025 | ENSSSCG00000016094 | 15 | + | 1.05E+08 | 1.05E+08 | 417 | annot_exons |
| novel_circ_001026 | ENSSSCG00000012743 | X | + | 1.22E+08 | 1.22E+08 | 823 | annot_exons |
| novel_circ_001027 | ENSSSCG00000006508 | 4 | + | 94436827 | 94438667 | 235 | annot_exons |
| novel_circ_001028 | ENSSSCG00000029544 | 7 | + | 77707934 | 77712077 | 288 | annot_exons |
| novel_circ_001029 | NA | 1 | + | 2.19E+08 | 2.19E+08 | 46437 | intergenic |
| novel_circ_001030 | ENSSSCG00000039598 | 1 | + | 21815401 | 21823434 | 647 | annot_exons |
| novel_circ_001031 | ENSSSCG00000009569 | 11 | - | 552302 | 555980 | 293 | annot_exons |
| novel_circ_001032 | ENSSSCG00000038036 | 1 | - | 2.62E+08 | 2.62E+08 | 785 | annot_exons |
| novel_circ_001033 | ENSSSCG00000001977 | 7 | + | 74351292 | 74366126 | 364 | exon_intron |
| novel_circ_001034 | ENSSSCG00000010640 | 14 | - | 1.24E+08 | 1.24E+08 | 183 | annot_exons |
| novel_circ_001035 | ENSSSCG00000004854 | 1 | - | 1.45E+08 | 1.45E+08 | 288 | annot_exons |
| novel_circ_001036 | ENSSSCG00000024030 | 12 | + | 59748171 | 59768376 | 1319 | annot_exons |
| novel_circ_001037 | ENSSSCG00000022177 | 15 | + | 1.33E+08 | 1.33E+08 | 13067 | intronic |
| novel_circ_001038 | ENSSSCG00000039338 | 11 | + | 25922303 | 25922501 | 199 | intronic |
| novel_circ_001039 | ENSSSCG00000034348 | 11 | + | 11160922 | 11209651 | 1045 | annot_exons |
| novel_circ_001040 | ENSSSCG00000015795 | 15 | - | 46518383 | 46520639 | 246 | annot_exons |
| novel_circ_001041 | ENSSSCG00000007109 | 17 | + | 29155959 | 29172437 | 687 | annot_exons |
| novel_circ_001042 | ENSSSCG00000011163 | 10 | + | 68970421 | 69017027 | 447 | annot_exons |
| novel_circ_001043 | ENSSSCG00000015050 | 9 | - | 41362858 | 41366657 | 523 | annot_exons |
| novel_circ_001044 | ENSSSCG00000009864 | 14 | + | 36079208 | 36101619 | 7696 | exon_intron |
| novel_circ_001045 | ENSSSCG00000014794 | 9 | + | 6367955 | 6382556 | 879 | annot_exons |
| novel_circ_001046 | ENSSSCG00000001950 | 7 | + | 64578330 | 64582878 | 4549 | antisense |
| novel_circ_001047 | ENSSSCG00000008261 | 3 | - | 68173390 | 68177952 | 440 | annot_exons |
| novel_circ_001048 | ENSSSCG00000008504 | 3 | - | 1.04E+08 | 1.04E+08 | 364 | annot_exons |
| novel_circ_001049 | ENSSSCG00000033591 | 6 | - | 31867238 | 31867964 | 222 | annot_exons |
| novel_circ_001050 | ENSSSCG00000015940 | 15 | + | 76166995 | 76170355 | 299 | annot_exons |
| novel_circ_001051 | ENSSSCG00000008857 | 8 | - | 43732762 | 43739096 | 562 | annot_exons |
| novel_circ_001052 | ENSSSCG00000008395 | 3 | - | 83444631 | 83468126 | 3479 | annot_exons |
| novel_circ_001053 | ENSSSCG00000017139 | 12 | - | 1771007 | 1783968 | 619 | annot_exons |
| novel_circ_001054 | ENSSSCG00000030685 | 5 | + | 76729220 | 76751177 | 1214 | annot_exons |
| novel_circ_001055 | ENSSSCG00000023112 | 8 | - | 31061651 | 31064029 | 266 | annot_exons |
| novel_circ_001056 | ENSSSCG00000034844 | 14 | - | 55825115 | 55831931 | 465 | annot_exons |
| novel_circ_001057 | ENSSSCG00000014139 | 2 | + | 1.02E+08 | 1.02E+08 | 455 | annot_exons |
| novel_circ_001058 | ENSSSCG00000033424 | 13 | - | 1.22E+08 | 1.22E+08 | 1533 | exon_intron |
| novel_circ_001059 | ENSSSCG00000009946 | 14 | + | 42253835 | 42256518 | 686 | annot_exons |
| novel_circ_001060 | ENSSSCG00000010465 | 14 | + | 1.03E+08 | 1.03E+08 | 312 | annot_exons |
| novel_circ_001061 | ENSSSCG00000000952 | 5 | - | 1.04E+08 | 1.04E+08 | 431 | annot_exons |
| novel_circ_001062 | ENSSSCG00000013295 | 2 | - | 25248313 | 25253299 | 437 | annot_exons |
| novel_circ_001063 | ENSSSCG00000004979 | 1 | - | 1.69E+08 | 1.69E+08 | 464 | annot_exons |
| novel_circ_001064 | ENSSSCG00000000571 | 5 | + | 51657471 | 51661763 | 319 | annot_exons |
| novel_circ_001065 | ENSSSCG00000016117 | 15 | + | 1.06E+08 | 1.06E+08 | 853 | annot_exons |
| novel_circ_001066 | ENSSSCG00000004508 | 1 | - | 98886959 | 98887554 | 596 | intronic |
| novel_circ_001067 | ENSSSCG00000012434 | X | - | 61619582 | 61651015 | 743 | annot_exons |
| novel_circ_001068 | ENSSSCG00000016988 | 16 | - | 51298744 | 51301436 | 224 | annot_exons |
| novel_circ_001069 | ENSSSCG00000016629 | 18 | - | 29135523 | 29142956 | 239 | annot_exons |
| novel_circ_001070 | ENSSSCG00000043604 | X | - | 59556112 | 59598975 | 5173 | annot_exons |
| novel_circ_001071 | ENSSSCG00000013938 | 2 | - | 58456862 | 58478376 | 376 | annot_exons |
| novel_circ_001072 | ENSSSCG00000026722 | 9 | + | 80731159 | 80762014 | 474 | annot_exons |
| novel_circ_001073 | ENSSSCG00000039085 | 13 | + | 2.07E+08 | 2.07E+08 | 339 | annot_exons |
| novel_circ_001074 | ENSSSCG00000016938 | 16 | + | 41216082 | 41228814 | 347 | annot_exons |
| novel_circ_001075 | ENSSSCG00000029633 | 6 | + | 1.57E+08 | 1.57E+08 | 519 | annot_exons |
| novel_circ_001076 | ENSSSCG00000008136 | 3 | - | 47570829 | 47571010 | 182 | one_exon |
| novel_circ_001077 | NA | 2 | + | 51521609 | 51522178 | 570 | intergenic |
| novel_circ_001078 | ENSSSCG00000006179 | 4 | + | 62178830 | 62197767 | 10237 | exon_intron |
| novel_circ_001079 | ENSSSCG00000028346 | 5 | - | 5051633 | 5059085 | 578 | annot_exons |
| novel_circ_001080 | ENSSSCG00000003976 | 6 | - | 1.7E+08 | 1.7E+08 | 543 | annot_exons |
| novel_circ_001081 | ENSSSCG00000003464 | 6 | + | 74739074 | 74741562 | 407 | annot_exons |
| novel_circ_001082 | ENSSSCG00000001852 | 7 | - | 55657733 | 55661779 | 760 | annot_exons |
| novel_circ_001083 | ENSSSCG00000012267 | X | + | 41652955 | 41678313 | 429 | annot_exons |
| novel_circ_001084 | ENSSSCG00000021208 | 16 | - | 27627809 | 27631128 | 1043 | annot_exons |
| novel_circ_001085 | ENSSSCG00000006283 | 4 | + | 81156524 | 81160440 | 293 | annot_exons |
| novel_circ_001086 | ENSSSCG00000021644 | 6 | + | 34508258 | 34515277 | 556 | annot_exons |
| novel_circ_001087 | ENSSSCG00000041238 | 6 | - | 62656329 | 62656597 | 269 | intronic |
| novel_circ_001088 | ENSSSCG00000030108 | 17 | - | 50949543 | 50951351 | 1809 | one_exon |
| novel_circ_001089 | NA | 15 | - | 84271967 | 84272228 | 262 | intergenic |
| novel_circ_001090 | ENSSSCG00000033303 | 13 | - | 68820770 | 68821056 | 210 | annot_exons |
| novel_circ_001091 | ENSSSCG00000008132 | 3 | + | 47004817 | 47007823 | 550 | annot_exons |
| novel_circ_001092 | ENSSSCG00000000583 | 5 | - | 51999867 | 52000834 | 234 | annot_exons |
| novel_circ_001093 | ENSSSCG00000015586 | 9 | - | 1.3E+08 | 1.3E+08 | 7017 | exon_intron |
| novel_circ_001094 | ENSSSCG00000008624 | 3 | - | 1.25E+08 | 1.25E+08 | 219 | annot_exons |
| novel_circ_001095 | ENSSSCG00000003971 | 6 | + | 1.7E+08 | 1.7E+08 | 555 | annot_exons |
| novel_circ_001096 | ENSSSCG00000041387 | 13 | + | 23867542 | 23867996 | 455 | exon_intron |
| novel_circ_001097 | ENSSSCG00000000939 | 5 | - | 1E+08 | 1E+08 | 605 | annot_exons |
| novel_circ_001098 | ENSSSCG00000001921 | 7 | - | 60079810 | 60084532 | 387 | annot_exons |
| novel_circ_001099 | ENSSSCG00000025953 | 6 | - | 29322627 | 29323574 | 243 | annot_exons |
| novel_circ_001100 | ENSSSCG00000050244 | 12 | + | 59273100 | 59342844 | 1924 | exon_intron |
| novel_circ_001101 | ENSSSCG00000003637 | 6 | + | 92312391 | 92313003 | 420 | annot_exons |
| novel_circ_001102 | ENSSSCG00000030095 | 9 | + | 41643139 | 41644483 | 1345 | one_exon |
| novel_circ_001103 | ENSSSCG00000009226 | 8 | + | 1.32E+08 | 1.32E+08 | 1095 | annot_exons |
| novel_circ_001104 | ENSSSCG00000029249 | 5 | - | 1.03E+08 | 1.03E+08 | 172 | intronic |
| novel_circ_001105 | ENSSSCG00000025087 | 4 | + | 71955069 | 71955315 | 247 | one_exon |
| novel_circ_001106 | ENSSSCG00000039609 | 16 | + | 196565 | 216679 | 271 | annot_exons |
| novel_circ_001107 | ENSSSCG00000010241 | 14 | + | 71765050 | 71773116 | 2496 | annot_exons |
| novel_circ_001108 | ENSSSCG00000035824 | 13 | - | 34769748 | 34780761 | 642 | annot_exons |
| novel_circ_001109 | ENSSSCG00000011064 | 10 | - | 48904200 | 48911562 | 484 | annot_exons |
| novel_circ_001110 | ENSSSCG00000006303 | 4 | - | 83002832 | 83034592 | 744 | annot_exons |
| novel_circ_001111 | ENSSSCG00000007805 | 3 | + | 18554299 | 18555003 | 384 | annot_exons |
| novel_circ_001112 | ENSSSCG00000016339 | 15 | - | 1.38E+08 | 1.38E+08 | 467 | annot_exons |
| novel_circ_001113 | ENSSSCG00000002283 | 7 | + | 89408734 | 89431122 | 22389 | exon_intron |
| novel_circ_001114 | ENSSSCG00000006071 | 4 | - | 37366367 | 37383210 | 475 | annot_exons |
| novel_circ_001115 | ENSSSCG00000015949 | 15 | - | 77898801 | 77953016 | 1510 | annot_exons |
| novel_circ_001116 | ENSSSCG00000011068 | 10 | - | 48816880 | 48819897 | 332 | annot_exons |
| novel_circ_001117 | ENSSSCG00000000870 | 5 | + | 82799606 | 82804808 | 332 | annot_exons |
| novel_circ_001118 | ENSSSCG00000009047 | 8 | - | 84206332 | 84211827 | 670 | annot_exons |
| novel_circ_001119 | ENSSSCG00000010172 | 14 | - | 59167649 | 59167874 | 226 | antisense |
| novel_circ_001120 | ENSSSCG00000012000 | 13 | + | 1.74E+08 | 1.74E+08 | 849 | annot_exons |
| novel_circ_001121 | ENSSSCG00000010190 | 14 | + | 60415377 | 60415597 | 221 | one_exon |
| novel_circ_001122 | NA | 15 | - | 84281285 | 84285405 | 4121 | intergenic |
| novel_circ_001123 | ENSSSCG00000009142 | 8 | - | 1.13E+08 | 1.13E+08 | 283 | annot_exons |
| novel_circ_001124 | ENSSSCG00000016717 | 18 | - | 47629573 | 47657002 | 656 | annot_exons |
| novel_circ_001125 | ENSSSCG00000008495 | 3 | + | 1.03E+08 | 1.03E+08 | 948 | one_exon |
| novel_circ_001126 | ENSSSCG00000000999 | 7 | + | 2951986 | 2953747 | 430 | annot_exons |
| novel_circ_001127 | ENSSSCG00000016397 | 15 | + | 712763 | 717839 | 525 | annot_exons |
| novel_circ_001128 | ENSSSCG00000015944 | 15 | - | 77226317 | 77283482 | 593 | annot_exons |
| novel_circ_001129 | ENSSSCG00000001499 | 7 | + | 29151456 | 29160814 | 7154 | exon_intron |
| novel_circ_001130 | ENSSSCG00000006785 | 4 | - | 1.09E+08 | 1.09E+08 | 411 | annot_exons |
| novel_circ_001131 | ENSSSCG00000003770 | 6 | - | 1.35E+08 | 1.35E+08 | 315 | annot_exons |
| novel_circ_001132 | ENSSSCG00000025133 | 13 | - | 2.08E+08 | 2.08E+08 | 322 | one_exon |
| novel_circ_001133 | NA | 18 | - | 14226810 | 14243823 | 17014 | intergenic |
| novel_circ_001134 | ENSSSCG00000036132 | 6 | - | 51709537 | 51711141 | 926 | exon_intron |
| novel_circ_001135 | ENSSSCG00000036431 | 1 | + | 1.1E+08 | 1.1E+08 | 995 | annot_exons |
| novel_circ_001136 | ENSSSCG00000002425 | 7 | - | 1.1E+08 | 1.1E+08 | 1445 | one_exon |
| novel_circ_001137 | ENSSSCG00000003531 | 6 | + | 80958369 | 80960170 | 321 | annot_exons |
| novel_circ_001138 | ENSSSCG00000001971 | 7 | + | 68406557 | 68408568 | 278 | annot_exons |
| novel_circ_001139 | ENSSSCG00000029837 | 11 | + | 25273271 | 25282303 | 690 | annot_exons |
| novel_circ_001140 | ENSSSCG00000030362 | 13 | - | 1.36E+08 | 1.36E+08 | 2141 | annot_exons |
| novel_circ_001141 | ENSSSCG00000010862 | 10 | - | 14786194 | 14792335 | 496 | annot_exons |
| novel_circ_001142 | ENSSSCG00000025305 | 3 | + | 1.31E+08 | 1.31E+08 | 265 | annot_exons |
| novel_circ_001143 | ENSSSCG00000008735 | 8 | - | 9237485 | 9240109 | 317 | annot_exons |
| novel_circ_001144 | ENSSSCG00000031175 | 8 | + | 47835194 | 47836168 | 975 | one_exon |
| novel_circ_001145 | ENSSSCG00000011917 | 13 | + | 1.46E+08 | 1.46E+08 | 321 | annot_exons |
| novel_circ_001146 | ENSSSCG00000001658 | 7 | + | 38116199 | 38116412 | 214 | antisense |
| novel_circ_001147 | ENSSSCG00000008068 | 3 | - | 41997364 | 42001550 | 297 | annot_exons |
| novel_circ_001148 | ENSSSCG00000037977 | 3 | - | 69382891 | 69386742 | 2384 | annot_exons |
| novel_circ_001149 | ENSSSCG00000025768 | 7 | - | 1.12E+08 | 1.12E+08 | 679 | antisense |
| novel_circ_001150 | ENSSSCG00000002378 | 7 | - | 98222032 | 98230257 | 599 | annot_exons |
| novel_circ_001151 | ENSSSCG00000027509 | 3 | + | 77886072 | 77887860 | 398 | annot_exons |
| novel_circ_001152 | ENSSSCG00000004082 | 1 | + | 14079141 | 14084086 | 423 | annot_exons |
| novel_circ_001153 | ENSSSCG00000011183 | 13 | - | 1464115 | 1467192 | 503 | annot_exons |
| novel_circ_001154 | NA | 15 | - | 84262569 | 84262928 | 360 | intergenic |
| novel_circ_001155 | ENSSSCG00000006995 | 17 | - | 5719251 | 5720151 | 137 | annot_exons |
| novel_circ_001156 | ENSSSCG00000018045 | 12 | + | 59821853 | 59822490 | 70 | annot_exons |
| novel_circ_001157 | ENSSSCG00000020663 | 18 | + | 5248722 | 5254144 | 1934 | annot_exons |
| novel_circ_001158 | NA | 7 | + | 68220757 | 68226624 | 5868 | intergenic |
| novel_circ_001159 | ENSSSCG00000014831 | 9 | + | 8286373 | 8321548 | 1110 | annot_exons |
| novel_circ_001160 | ENSSSCG00000009244 | 8 | + | 1.35E+08 | 1.35E+08 | 822 | annot_exons |
| novel_circ_001161 | ENSSSCG00000001964 | 7 | - | 66529998 | 66530404 | 296 | annot_exons |
| novel_circ_001162 | ENSSSCG00000015947 | 15 | + | 77565524 | 77579883 | 606 | annot_exons |
| novel_circ_001163 | ENSSSCG00000001499 | 7 | + | 29167275 | 29176480 | 844 | annot_exons |
| novel_circ_001164 | ENSSSCG00000011226 | 13 | + | 16822443 | 16826413 | 360 | annot_exons |
| novel_circ_001165 | ENSSSCG00000014170 | 2 | + | 1.03E+08 | 1.03E+08 | 330 | annot_exons |
| novel_circ_001166 | NA | 15 | - | 84438597 | 84438844 | 248 | intergenic |
| novel_circ_001167 | ENSSSCG00000017184 | 12 | + | 5159731 | 5172927 | 760 | annot_exons |
| novel_circ_001168 | ENSSSCG00000011025 | 10 | + | 42063516 | 42081674 | 18159 | intronic |
| novel_circ_001169 | ENSSSCG00000035488 | X | + | 96997114 | 96998433 | 259 | annot_exons |
| novel_circ_001170 | ENSSSCG00000010053 | 14 | + | 49399053 | 49424492 | 691 | annot_exons |
| novel_circ_001171 | ENSSSCG00000011332 | 13 | - | 29888031 | 29898615 | 333 | annot_exons |
| novel_circ_001172 | ENSSSCG00000010652 | 14 | + | 1.25E+08 | 1.25E+08 | 549 | annot_exons |
| novel_circ_001173 | ENSSSCG00000037755 | 4 | - | 14518852 | 14547742 | 261 | annot_exons |
| novel_circ_001174 | ENSSSCG00000029593 | 3 | - | 92299236 | 92299786 | 551 | one_exon |
| novel_circ_001175 | ENSSSCG00000040157 | 2 | + | 76445406 | 76447396 | 258 | annot_exons |
| novel_circ_001176 | ENSSSCG00000011025 | 10 | + | 42147410 | 42194913 | 735 | annot_exons |
| novel_circ_001177 | ENSSSCG00000014878 | 9 | - | 12093564 | 12097543 | 262 | annot_exons |
| novel_circ_001178 | ENSSSCG00000024623 | 13 | + | 1.81E+08 | 1.81E+08 | 465 | annot_exons |
| novel_circ_001179 | ENSSSCG00000004570 | 1 | - | 1.09E+08 | 1.09E+08 | 252 | annot_exons |
| novel_circ_001180 | ENSSSCG00000026382 | 1 | + | 1.95E+08 | 1.95E+08 | 1175 | exon_intron |
| novel_circ_001181 | ENSSSCG00000014296 | 2 | - | 1.36E+08 | 1.36E+08 | 434 | annot_exons |
| novel_circ_001182 | ENSSSCG00000008635 | 3 | + | 1.26E+08 | 1.26E+08 | 695 | annot_exons |
| novel_circ_001183 | ENSSSCG00000051610 | 18 | + | 17872046 | 17912006 | 17900 | exon_intron |
| novel_circ_001184 | ENSSSCG00000016922 | 16 | + | 36266989 | 36285749 | 1260 | annot_exons |
| novel_circ_001185 | ENSSSCG00000016954 | 16 | + | 44446274 | 44455050 | 441 | annot_exons |
| novel_circ_001186 | ENSSSCG00000017832 | 12 | - | 48532439 | 48543642 | 600 | annot_exons |
| novel_circ_001187 | ENSSSCG00000027967 | 3 | + | 23670086 | 23704655 | 26253 | exon_intron |
| novel_circ_001188 | ENSSSCG00000016960 | 16 | + | 47504607 | 47505681 | 342 | annot_exons |
| novel_circ_001189 | ENSSSCG00000009061 | 8 | - | 87653808 | 87659118 | 409 | annot_exons |
| novel_circ_001190 | ENSSSCG00000027778 | 7 | - | 34157535 | 34166958 | 518 | annot_exons |
| novel_circ_001191 | ENSSSCG00000007284 | 17 | - | 38156401 | 38173595 | 667 | annot_exons |
| novel_circ_001192 | ENSSSCG00000015602 | 9 | + | 1.31E+08 | 1.31E+08 | 323 | annot_exons |
| novel_circ_001193 | ENSSSCG00000017497 | 12 | - | 22606823 | 22607386 | 247 | annot_exons |
| novel_circ_001194 | ENSSSCG00000011599 | 13 | + | 70006253 | 70013088 | 6587 | exon_intron |
| novel_circ_001195 | ENSSSCG00000016291 | 15 | + | 1.33E+08 | 1.33E+08 | 675 | annot_exons |
| novel_circ_001196 | ENSSSCG00000034200 | 13 | - | 26094587 | 26096683 | 344 | annot_exons |
| novel_circ_001197 | ENSSSCG00000004220 | 1 | - | 36964151 | 36994867 | 2463 | annot_exons |
| novel_circ_001198 | ENSSSCG00000017513 | 12 | + | 23866934 | 23874195 | 559 | annot_exons |
| novel_circ_001199 | ENSSSCG00000015777 | 15 | + | 45014628 | 45018907 | 842 | annot_exons |
| novel_circ_001200 | ENSSSCG00000003828 | 6 | - | 1.53E+08 | 1.53E+08 | 263 | annot_exons |
| novel_circ_001201 | ENSSSCG00000002266 | 7 | - | 85990604 | 85999297 | 561 | annot_exons |
| novel_circ_001202 | ENSSSCG00000014088 | 2 | + | 85536348 | 85536506 | 159 | intronic |
| novel_circ_001203 | ENSSSCG00000025323 | 6 | + | 13527958 | 13534584 | 1772 | annot_exons |
| novel_circ_001204 | ENSSSCG00000000625 | 5 | + | 60269555 | 60270853 | 507 | annot_exons |
| novel_circ_001205 | ENSSSCG00000014010 | 2 | + | 78380148 | 78387048 | 2258 | annot_exons |
| novel_circ_001206 | ENSSSCG00000035686 | 5 | - | 21672280 | 21684161 | 927 | annot_exons |
| novel_circ_001207 | ENSSSCG00000010317 | 14 | - | 77625263 | 77629469 | 4207 | exon_intron |
| novel_circ_001208 | ENSSSCG00000003716 | 6 | - | 1.11E+08 | 1.11E+08 | 376 | annot_exons |
| novel_circ_001209 | ENSSSCG00000026705 | 10 | - | 12564815 | 12579241 | 936 | annot_exons |
| novel_circ_001210 | ENSSSCG00000004032 | 1 | + | 5857231 | 5859098 | 1868 | exon_intron |
| novel_circ_001211 | ENSSSCG00000029637 | 2 | - | 5285891 | 5290605 | 226 | annot_exons |
| novel_circ_001212 | ENSSSCG00000015052 | 9 | - | 41453661 | 41463012 | 399 | annot_exons |
| novel_circ_001213 | ENSSSCG00000007026 | 17 | + | 11420778 | 11423931 | 437 | annot_exons |
| novel_circ_001214 | ENSSSCG00000023171 | 6 | - | 1.28E+08 | 1.28E+08 | 2307 | exon_intron |
| novel_circ_001215 | ENSSSCG00000003673 | 6 | - | 96642188 | 96643977 | 445 | annot_exons |
| novel_circ_001216 | ENSSSCG00000008600 | 3 | + | 1.18E+08 | 1.18E+08 | 645 | annot_exons |
| novel_circ_001217 | ENSSSCG00000011760 | 13 | - | 1.15E+08 | 1.15E+08 | 217 | annot_exons |
| novel_circ_001218 | ENSSSCG00000008310 | 3 | + | 70023969 | 70040230 | 390 | annot_exons |
| novel_circ_001219 | ENSSSCG00000039285 | 13 | + | 1.17E+08 | 1.17E+08 | 540 | annot_exons |
| novel_circ_001220 | ENSSSCG00000022982 | 9 | + | 73124646 | 73149020 | 465 | annot_exons |
| novel_circ_001221 | ENSSSCG00000014376 | 2 | + | 1.42E+08 | 1.42E+08 | 488 | annot_exons |
| novel_circ_001222 | ENSSSCG00000008467 | 3 | - | 98047438 | 98064327 | 822 | annot_exons |
| novel_circ_001223 | ENSSSCG00000000952 | 5 | - | 1.04E+08 | 1.04E+08 | 499 | annot_exons |
| novel_circ_001224 | ENSSSCG00000008412 | 3 | + | 87305853 | 87307407 | 347 | annot_exons |
| novel_circ_001225 | ENSSSCG00000035639 | 6 | - | 27526548 | 27529866 | 329 | annot_exons |
| novel_circ_001226 | ENSSSCG00000028148 | X | + | 28189922 | 28190600 | 679 | antisense |
| novel_circ_001227 | ENSSSCG00000006785 | 4 | - | 1.09E+08 | 1.09E+08 | 589 | annot_exons |
| novel_circ_001228 | ENSSSCG00000027857 | 2 | + | 1.23E+08 | 1.23E+08 | 342 | annot_exons |
| novel_circ_001229 | ENSSSCG00000002822 | 6 | + | 29377388 | 29384324 | 411 | annot_exons |
| novel_circ_001230 | ENSSSCG00000011136 | 10 | - | 65235848 | 65238541 | 2409 | annot_exons |
| novel_circ_001231 | ENSSSCG00000049158 | 1 | + | 13986471 | 13986949 | 327 | annot_exons |
| novel_circ_001232 | ENSSSCG00000002505 | 7 | + | 1.18E+08 | 1.18E+08 | 725 | annot_exons |
| novel_circ_001233 | ENSSSCG00000016863 | 16 | - | 26626313 | 26646919 | 599 | annot_exons |
| novel_circ_001234 | ENSSSCG00000049447 | 3 | - | 51302945 | 51306765 | 774 | annot_exons |
| novel_circ_001235 | ENSSSCG00000016397 | 15 | + | 736701 | 742947 | 651 | annot_exons |
| novel_circ_001236 | ENSSSCG00000004070 | 1 | - | 12041132 | 12043885 | 1684 | annot_exons |
| novel_circ_001237 | ENSSSCG00000015097 | 9 | - | 46038901 | 46044924 | 611 | annot_exons |
| novel_circ_001238 | ENSSSCG00000008144 | 3 | - | 49086498 | 49087207 | 710 | one_exon |
| novel_circ_001239 | ENSSSCG00000000901 | 5 | + | 88177384 | 88182006 | 397 | annot_exons |
| novel_circ_001240 | ENSSSCG00000011045 | 10 | + | 45992309 | 46006364 | 636 | annot_exons |
| novel_circ_001241 | ENSSSCG00000008799 | 8 | + | 32683293 | 32692601 | 249 | annot_exons |
| novel_circ_001242 | ENSSSCG00000014121 | 2 | + | 89079718 | 89081961 | 504 | annot_exons |
| novel_circ_001243 | ENSSSCG00000028148 | X | - | 28366347 | 28367025 | 285 | annot_exons |
| novel_circ_001244 | ENSSSCG00000004716 | 1 | - | 1.28E+08 | 1.28E+08 | 546 | annot_exons |
| novel_circ_001245 | ENSSSCG00000011340 | 13 | - | 30516967 | 30524999 | 373 | annot_exons |
| novel_circ_001246 | ENSSSCG00000028178 | 10 | - | 12407369 | 12417836 | 1123 | annot_exons |
| novel_circ_001247 | ENSSSCG00000034795 | 2 | + | 87254645 | 87286479 | 717 | annot_exons |
| novel_circ_001248 | ENSSSCG00000017202 | 12 | + | 5633650 | 5634027 | 333 | annot_exons |
| novel_circ_001249 | ENSSSCG00000009528 | 11 | + | 70961245 | 70968258 | 521 | annot_exons |
| novel_circ_001250 | ENSSSCG00000009714 | 14 | + | 20194558 | 20203263 | 414 | annot_exons |
| novel_circ_001251 | ENSSSCG00000024864 | 5 | - | 3750906 | 3754014 | 256 | annot_exons |
| novel_circ_001252 | ENSSSCG00000034039 | AEMK02000258.1 | + | 364809 | 376062 | 4542 | annot_exons |
| novel_circ_001253 | ENSSSCG00000006987 | 17 | + | 5219405 | 5219793 | 389 | intronic |
| novel_circ_001254 | ENSSSCG00000009407 | 11 | + | 20661774 | 20664489 | 344 | annot_exons |
| novel_circ_001255 | ENSSSCG00000016938 | 16 | + | 41174350 | 41223347 | 1433 | annot_exons |
| novel_circ_001256 | ENSSSCG00000006100 | 4 | + | 42172655 | 42188530 | 544 | annot_exons |
| novel_circ_001257 | ENSSSCG00000010187 | 14 | + | 60259238 | 60276493 | 6065 | annot_exons |
| novel_circ_001258 | ENSSSCG00000014343 | 2 | + | 1.41E+08 | 1.41E+08 | 972 | annot_exons |
| novel_circ_001259 | ENSSSCG00000004636 | 1 | + | 1.21E+08 | 1.21E+08 | 1128 | annot_exons |
| novel_circ_001260 | ENSSSCG00000015960 | 15 | - | 79198010 | 79198269 | 260 | antisense |
| novel_circ_001261 | ENSSSCG00000020710 | 6 | + | 73202013 | 73206401 | 4387 | annot_exons |
| novel_circ_001262 | ENSSSCG00000011446 | 13 | - | 34882055 | 34882541 | 306 | annot_exons |
| novel_circ_001263 | ENSSSCG00000010833 | 10 | - | 11344911 | 11346876 | 279 | annot_exons |
| novel_circ_001264 | ENSSSCG00000008513 | 3 | - | 1.07E+08 | 1.07E+08 | 366 | annot_exons |
| novel_circ_001265 | ENSSSCG00000015273 | 9 | + | 64407564 | 64426983 | 2195 | annot_exons |
| novel_circ_001266 | ENSSSCG00000028033 | 14 | + | 927726 | 932501 | 411 | annot_exons |
| novel_circ_001267 | ENSSSCG00000011137 | 10 | + | 65179075 | 65191886 | 566 | annot_exons |
| novel_circ_001268 | ENSSSCG00000017890 | 12 | + | 50751715 | 50754749 | 490 | annot_exons |
| novel_circ_001269 | ENSSSCG00000025729 | 15 | - | 1.28E+08 | 1.28E+08 | 2427 | exon_intron |
| novel_circ_001270 | ENSSSCG00000035901 | 14 | + | 286861 | 287094 | 234 | one_exon |
| novel_circ_001271 | ENSSSCG00000013344 | 2 | - | 37193874 | 37201955 | 319 | annot_exons |
| novel_circ_001272 | ENSSSCG00000022343 | 6 | - | 69391257 | 69392466 | 427 | annot_exons |
| novel_circ_001273 | ENSSSCG00000037977 | 3 | - | 69382891 | 69401023 | 2706 | annot_exons |
| novel_circ_001274 | ENSSSCG00000002799 | 6 | - | 20304888 | 20309084 | 912 | annot_exons |
| novel_circ_001275 | ENSSSCG00000011233 | 13 | - | 18515099 | 18518523 | 217 | annot_exons |
| novel_circ_001276 | ENSSSCG00000029174 | 9 | + | 92384248 | 92397915 | 658 | annot_exons |
| novel_circ_001277 | ENSSSCG00000016853 | 16 | - | 24175667 | 24179590 | 297 | annot_exons |
| novel_circ_001278 | ENSSSCG00000004065 | 1 | - | 11656690 | 11658867 | 299 | annot_exons |
| novel_circ_001279 | ENSSSCG00000004739 | 1 | - | 1.3E+08 | 1.3E+08 | 1131 | one_exon |
| novel_circ_001280 | ENSSSCG00000021005 | 10 | + | 31295417 | 31297552 | 268 | annot_exons |
| novel_circ_001281 | ENSSSCG00000010794 | 3 | + | 22603929 | 22604675 | 279 | annot_exons |
| novel_circ_001282 | ENSSSCG00000010295 | 14 | - | 75976312 | 75980655 | 578 | annot_exons |
| novel_circ_001283 | ENSSSCG00000015867 | 15 | - | 60407130 | 60414442 | 802 | annot_exons |
| novel_circ_001284 | ENSSSCG00000023391 | 6 | - | 36409465 | 36416154 | 412 | annot_exons |
| novel_circ_001285 | ENSSSCG00000015743 | 15 | - | 31426460 | 31473618 | 385 | annot_exons |
| novel_circ_001286 | ENSSSCG00000004636 | 1 | + | 1.21E+08 | 1.21E+08 | 941 | annot_exons |
| novel_circ_001287 | ENSSSCG00000011542 | 13 | + | 65723023 | 65724436 | 366 | annot_exons |
| novel_circ_001288 | ENSSSCG00000016397 | 15 | - | 623412 | 626489 | 3078 | antisense |
| novel_circ_001289 | ENSSSCG00000006184 | 4 | - | 62811894 | 62816173 | 2686 | exon_intron |
| novel_circ_001290 | ENSSSCG00000027613 | 1 | + | 39257585 | 39280533 | 3616 | exon_intron |
| novel_circ_001291 | ENSSSCG00000015247 | 9 | - | 56957702 | 56973685 | 1406 | annot_exons |
| novel_circ_001292 | ENSSSCG00000004856 | 1 | - | 1.46E+08 | 1.46E+08 | 503 | annot_exons |
| novel_circ_001293 | ENSSSCG00000038977 | 9 | - | 1.02E+08 | 1.02E+08 | 449 | annot_exons |
| novel_circ_001294 | ENSSSCG00000023636 | 6 | + | 84559162 | 84559335 | 174 | one_exon |
| novel_circ_001295 | ENSSSCG00000035045 | 14 | + | 1.37E+08 | 1.37E+08 | 221 | annot_exons |
| novel_circ_001296 | ENSSSCG00000029811 | 13 | + | 4115487 | 4117973 | 2487 | one_exon |
| novel_circ_001297 | ENSSSCG00000029651 | 9 | + | 36233566 | 36233716 | 151 | antisense |
| novel_circ_001298 | ENSSSCG00000028771 | 1 | + | 1.38E+08 | 1.38E+08 | 550 | annot_exons |
| novel_circ_001299 | ENSSSCG00000025686 | 10 | + | 12471548 | 12479287 | 508 | annot_exons |
| novel_circ_001300 | NA | 7 | + | 4578332 | 4584896 | 6565 | intergenic |
| novel_circ_001301 | ENSSSCG00000040087 | 14 | + | 59176799 | 59177070 | 272 | antisense |
| novel_circ_001302 | ENSSSCG00000036926 | 1 | + | 2.67E+08 | 2.67E+08 | 458 | annot_exons |
| novel_circ_001303 | ENSSSCG00000051610 | 18 | + | 17859078 | 17912006 | 359 | annot_exons |
| novel_circ_001304 | ENSSSCG00000024784 | 10 | - | 23522207 | 23523319 | 440 | annot_exons |
| novel_circ_001305 | ENSSSCG00000016306 | 15 | - | 1.34E+08 | 1.34E+08 | 1142 | annot_exons |
| novel_circ_001306 | ENSSSCG00000016049 | 15 | - | 94830414 | 94839646 | 374 | annot_exons |
| novel_circ_001307 | ENSSSCG00000010872 | 10 | - | 16490536 | 16522506 | 555 | annot_exons |
| novel_circ_001308 | ENSSSCG00000001863 | 7 | + | 56364054 | 56367247 | 295 | annot_exons |
| novel_circ_001309 | ENSSSCG00000009994 | 14 | + | 46993155 | 46996102 | 211 | annot_exons |
| novel_circ_001310 | ENSSSCG00000015283 | 9 | - | 65101799 | 65106823 | 612 | annot_exons |
| novel_circ_001311 | ENSSSCG00000027314 | 10 | + | 23313239 | 23333461 | 421 | annot_exons |
| novel_circ_001312 | ENSSSCG00000008761 | 8 | + | 20273058 | 20308094 | 595 | annot_exons |
| novel_circ_001313 | ENSSSCG00000011068 | 10 | - | 48707018 | 48713706 | 337 | annot_exons |
| novel_circ_001314 | ENSSSCG00000029837 | 11 | + | 25318969 | 25343890 | 874 | annot_exons |
| novel_circ_001315 | ENSSSCG00000009974 | 14 | + | 46421193 | 46423590 | 355 | annot_exons |
| novel_circ_001316 | ENSSSCG00000017735 | 12 | - | 42750233 | 42758003 | 318 | annot_exons |
| novel_circ_001317 | ENSSSCG00000031077 | 13 | - | 1E+08 | 1E+08 | 438 | annot_exons |
| novel_circ_001318 | ENSSSCG00000011022 | 10 | + | 41434069 | 41436115 | 470 | annot_exons |
| novel_circ_001319 | ENSSSCG00000001934 | 7 | + | 61121465 | 61137643 | 766 | annot_exons |
| novel_circ_001320 | ENSSSCG00000005191 | 1 | + | 2.09E+08 | 2.09E+08 | 1110 | annot_exons |
| novel_circ_001321 | NA | 5 | - | 86923721 | 86932427 | 8707 | intergenic |
| novel_circ_001322 | NA | 13 | + | 46348179 | 46348397 | 219 | intergenic |
| novel_circ_001323 | ENSSSCG00000027738 | 13 | + | 21778794 | 21781212 | 285 | annot_exons |
| novel_circ_001324 | ENSSSCG00000032170 | 2 | + | 74767630 | 74777761 | 5272 | annot_exons |
| novel_circ_001325 | ENSSSCG00000016981 | 16 | - | 50437615 | 50446698 | 2096 | annot_exons |
| novel_circ_001326 | ENSSSCG00000030053 | 9 | + | 45877120 | 45880176 | 815 | annot_exons |
| novel_circ_001327 | NA | 15 | + | 84243647 | 84244044 | 398 | intergenic |
| novel_circ_001328 | ENSSSCG00000011765 | 13 | + | 1.18E+08 | 1.18E+08 | 465 | annot_exons |
| novel_circ_001329 | ENSSSCG00000010299 | 14 | - | 76216343 | 76217017 | 285 | annot_exons |
| novel_circ_001330 | ENSSSCG00000016773 | 18 | + | 55536901 | 55538825 | 610 | annot_exons |
| novel_circ_001331 | ENSSSCG00000008909 | 8 | - | 54816224 | 54817234 | 224 | annot_exons |
| novel_circ_001332 | ENSSSCG00000030528 | 13 | - | 1.92E+08 | 1.92E+08 | 492 | annot_exons |
| novel_circ_001333 | ENSSSCG00000009401 | 11 | - | 19196075 | 19201387 | 675 | annot_exons |
| novel_circ_001334 | ENSSSCG00000001621 | 7 | - | 36872569 | 36873684 | 224 | annot_exons |
| novel_circ_001335 | ENSSSCG00000003815 | 6 | - | 1.49E+08 | 1.49E+08 | 510 | annot_exons |
| novel_circ_001336 | ENSSSCG00000026466 | 17 | - | 13946021 | 13981293 | 388 | annot_exons |
| novel_circ_001337 | ENSSSCG00000009281 | 11 | + | 2349625 | 2357456 | 385 | annot_exons |
| novel_circ_001338 | ENSSSCG00000022790 | 2 | + | 1.26E+08 | 1.26E+08 | 297 | annot_exons |
| novel_circ_001339 | ENSSSCG00000031877 | 2 | + | 60385822 | 60386344 | 388 | annot_exons |
| novel_circ_001340 | ENSSSCG00000011295 | 13 | + | 26682837 | 26686462 | 751 | annot_exons |
| novel_circ_001341 | ENSSSCG00000033334 | 15 | + | 1.32E+08 | 1.32E+08 | 367 | annot_exons |
| novel_circ_001342 | ENSSSCG00000013155 | 2 | - | 12501212 | 12504173 | 215 | annot_exons |
| novel_circ_001343 | ENSSSCG00000002425 | 7 | - | 1.1E+08 | 1.1E+08 | 525 | annot_exons |
| novel_circ_001344 | ENSSSCG00000017529 | 12 | - | 24326459 | 24328063 | 450 | annot_exons |
| novel_circ_001345 | ENSSSCG00000005282 | 1 | + | 2.3E+08 | 2.3E+08 | 626 | annot_exons |
| novel_circ_001346 | ENSSSCG00000001080 | 7 | + | 15922699 | 15924948 | 288 | annot_exons |
| novel_circ_001347 | ENSSSCG00000007839 | 3 | - | 23892951 | 23913947 | 769 | annot_exons |
| novel_circ_001348 | ENSSSCG00000012148 | X | + | 13192814 | 13196240 | 214 | annot_exons |
| novel_circ_001349 | ENSSSCG00000015670 | 15 | + | 8022396 | 8044280 | 418 | annot_exons |
| novel_circ_001350 | ENSSSCG00000006037 | 4 | - | 30742591 | 30743646 | 279 | annot_exons |
| novel_circ_001351 | ENSSSCG00000024696 | 15 | + | 1.11E+08 | 1.11E+08 | 419 | annot_exons |
| novel_circ_001352 | ENSSSCG00000007056 | 17 | + | 17623991 | 17630911 | 418 | annot_exons |
| novel_circ_001353 | ENSSSCG00000004576 | 1 | + | 1.11E+08 | 1.11E+08 | 623 | intronic |
| novel_circ_001354 | ENSSSCG00000011025 | 10 | + | 42192452 | 42194913 | 309 | annot_exons |
| novel_circ_001355 | ENSSSCG00000002425 | 7 | - | 1.1E+08 | 1.1E+08 | 1716 | exon_intron |
| novel_circ_001356 | ENSSSCG00000004490 | 1 | + | 94661166 | 94661813 | 648 | one_exon |
| novel_circ_001357 | ENSSSCG00000000840 | 5 | - | 79458763 | 79461555 | 453 | annot_exons |
| novel_circ_001358 | ENSSSCG00000035249 | 14 | + | 1166639 | 1166852 | 214 | one_exon |
| novel_circ_001359 | ENSSSCG00000038826 | 6 | - | 98833466 | 98835565 | 279 | annot_exons |
| novel_circ_001360 | ENSSSCG00000002269 | 7 | + | 88517369 | 88518062 | 321 | annot_exons |
| novel_circ_001361 | ENSSSCG00000015853 | 15 | + | 56527520 | 56547111 | 2436 | annot_exons |
| novel_circ_001362 | ENSSSCG00000011131 | 10 | + | 64498378 | 64502263 | 338 | annot_exons |
| novel_circ_001363 | ENSSSCG00000007805 | 3 | - | 18560341 | 18565899 | 5559 | antisense |
| novel_circ_001364 | ENSSSCG00000001527 | 7 | - | 30582062 | 30619798 | 527 | annot_exons |
| novel_circ_001365 | ENSSSCG00000017301 | 12 | - | 16195632 | 16211961 | 450 | annot_exons |
| novel_circ_001366 | ENSSSCG00000034517 | 3 | - | 4466309 | 4467664 | 1356 | antisense |
| novel_circ_001367 | ENSSSCG00000008991 | 8 | + | 73942366 | 73946651 | 277 | annot_exons |
| novel_circ_001368 | ENSSSCG00000009551 | 11 | + | 77514922 | 77517517 | 409 | annot_exons |
| novel_circ_001369 | ENSSSCG00000004634 | 1 | - | 1.21E+08 | 1.21E+08 | 449 | annot_exons |
| novel_circ_001370 | ENSSSCG00000000274 | 5 | + | 18699011 | 18699271 | 261 | one_exon |
| novel_circ_001371 | ENSSSCG00000014917 | 9 | + | 20240367 | 20246726 | 476 | annot_exons |
| novel_circ_001372 | ENSSSCG00000004730 | 1 | + | 1.29E+08 | 1.29E+08 | 4461 | annot_exons |
| novel_circ_001373 | ENSSSCG00000011689 | 13 | - | 86452535 | 86459135 | 439 | annot_exons |
| novel_circ_001374 | ENSSSCG00000020962 | 12 | + | 34349303 | 34349533 | 231 | one_exon |
| novel_circ_001375 | ENSSSCG00000014878 | 9 | - | 12102961 | 12106836 | 521 | annot_exons |
| novel_circ_001376 | ENSSSCG00000003596 | 6 | - | 87863719 | 87874230 | 383 | annot_exons |
| novel_circ_001377 | ENSSSCG00000012000 | 13 | + | 1.74E+08 | 1.74E+08 | 563 | annot_exons |
| novel_circ_001378 | ENSSSCG00000025305 | 3 | + | 1.31E+08 | 1.31E+08 | 670 | exon_intron |
| novel_circ_001379 | ENSSSCG00000004394 | 1 | + | 77096918 | 77116270 | 19353 | exon_intron |
| novel_circ_001380 | ENSSSCG00000036553 | 4 | + | 20430576 | 20430893 | 318 | intronic |
| novel_circ_001381 | ENSSSCG00000006346 | 4 | - | 88731237 | 88732517 | 214 | annot_exons |
| novel_circ_001382 | ENSSSCG00000042985 | 6 | + | 47428745 | 47442978 | 14234 | exon_intron |
| novel_circ_001383 | ENSSSCG00000014913 | 9 | - | 19897760 | 19913189 | 416 | annot_exons |
| novel_circ_001384 | ENSSSCG00000013305 | 2 | - | 26847671 | 26851921 | 274 | annot_exons |
| novel_circ_001385 | ENSSSCG00000016845 | 16 | - | 22567969 | 22576391 | 810 | annot_exons |
| novel_circ_001386 | ENSSSCG00000016956 | 16 | + | 45500364 | 45505169 | 235 | annot_exons |
| novel_circ_001387 | ENSSSCG00000003512 | 6 | - | 79067443 | 79075866 | 717 | annot_exons |
| novel_circ_001388 | ENSSSCG00000009229 | 8 | - | 1.33E+08 | 1.33E+08 | 1186 | intronic |
| novel_circ_001389 | ENSSSCG00000036909 | 6 | + | 1.67E+08 | 1.67E+08 | 5389 | exon_intron |
| novel_circ_001390 | ENSSSCG00000025041 | 5 | + | 1.05E+08 | 1.05E+08 | 770 | exon_intron |
| novel_circ_001391 | ENSSSCG00000037556 | 9 | - | 1.07E+08 | 1.07E+08 | 297 | annot_exons |
| novel_circ_001392 | ENSSSCG00000000075 | 5 | + | 7960350 | 7961485 | 238 | annot_exons |
| novel_circ_001393 | ENSSSCG00000011136 | 10 | - | 65240555 | 65262220 | 5758 | exon_intron |
| novel_circ_001394 | ENSSSCG00000023243 | 6 | - | 1.51E+08 | 1.51E+08 | 141 | annot_exons |
| novel_circ_001395 | ENSSSCG00000011580 | 13 | + | 68518160 | 68519808 | 494 | exon_intron |
| novel_circ_001396 | ENSSSCG00000017688 | 12 | + | 38085680 | 38093378 | 593 | annot_exons |
| novel_circ_001397 | ENSSSCG00000030480 | 13 | + | 2.01E+08 | 2.01E+08 | 11016 | exon_intron |
| novel_circ_001398 | ENSSSCG00000012663 | X | - | 1.07E+08 | 1.07E+08 | 498 | annot_exons |
| novel_circ_001399 | ENSSSCG00000000037 | 5 | + | 6007304 | 6014815 | 377 | annot_exons |
| novel_circ_001400 | ENSSSCG00000023445 | 18 | - | 40675472 | 40680815 | 261 | annot_exons |
| novel_circ_001401 | ENSSSCG00000034569 | 2 | + | 51346346 | 51363384 | 1104 | annot_exons |
| novel_circ_001402 | ENSSSCG00000000625 | 5 | + | 60247516 | 60249461 | 569 | annot_exons |
| novel_circ_001403 | ENSSSCG00000034704 | 1 | - | 1.62E+08 | 1.62E+08 | 580 | annot_exons |
| novel_circ_001404 | ENSSSCG00000030547 | 4 | - | 98027350 | 98028904 | 453 | annot_exons |
| novel_circ_001405 | ENSSSCG00000008583 | 3 | + | 1.14E+08 | 1.14E+08 | 702 | annot_exons |
| novel_circ_001406 | ENSSSCG00000026596 | 12 | - | 39024324 | 39039068 | 1451 | annot_exons |
| novel_circ_001407 | ENSSSCG00000003512 | 6 | - | 79150170 | 79151009 | 840 | one_exon |
| novel_circ_001408 | ENSSSCG00000024570 | 2 | - | 73776864 | 73777286 | 423 | exon_intron |
| novel_circ_001409 | ENSSSCG00000003877 | 6 | + | 1.62E+08 | 1.62E+08 | 385 | annot_exons |
| novel_circ_001410 | ENSSSCG00000031940 | 2 | - | 36775369 | 36784833 | 206 | annot_exons |
| novel_circ_001411 | NA | 15 | - | 84462324 | 84463864 | 1541 | intergenic |
| novel_circ_001412 | ENSSSCG00000011102 | 10 | - | 56327575 | 56331045 | 301 | annot_exons |
| novel_circ_001413 | ENSSSCG00000027293 | 16 | + | 49101098 | 49107340 | 323 | annot_exons |
| novel_circ_001414 | ENSSSCG00000028148 | X | - | 28331459 | 28332363 | 309 | annot_exons |
| novel_circ_001415 | ENSSSCG00000004242 | 1 | + | 41076032 | 41083592 | 340 | annot_exons |
| novel_circ_001416 | ENSSSCG00000004522 | 1 | + | 1E+08 | 1E+08 | 273 | annot_exons |
| novel_circ_001417 | ENSSSCG00000012591 | X | - | 92629923 | 92632932 | 1082 | annot_exons |
| novel_circ_001418 | ENSSSCG00000042878 | 1 | - | 1.32E+08 | 1.32E+08 | 5310 | exon_intron |
| novel_circ_001419 | ENSSSCG00000016568 | 18 | - | 19302353 | 19399491 | 994 | annot_exons |
| novel_circ_001420 | ENSSSCG00000004281 | 1 | + | 52764469 | 52800752 | 218 | annot_exons |
| novel_circ_001421 | ENSSSCG00000039469 | 7 | + | 97035683 | 97036826 | 268 | annot_exons |
| novel_circ_001422 | ENSSSCG00000009480 | 11 | + | 51540591 | 51551593 | 286 | annot_exons |
| novel_circ_001423 | ENSSSCG00000036145 | 6 | - | 1.56E+08 | 1.56E+08 | 327 | annot_exons |
| novel_circ_001424 | ENSSSCG00000001869 | 7 | + | 57223552 | 57244084 | 20533 | antisense |
| novel_circ_001425 | ENSSSCG00000015462 | 9 | + | 1.13E+08 | 1.13E+08 | 239 | annot_exons |
| novel_circ_001426 | ENSSSCG00000022112 | 11 | - | 67895181 | 67898460 | 301 | annot_exons |
| novel_circ_001427 | ENSSSCG00000016850 | 16 | - | 23740869 | 23745763 | 3116 | annot_exons |
| novel_circ_001428 | ENSSSCG00000014132 | 2 | + | 90499141 | 90509714 | 256 | exon_intron |
| novel_circ_001429 | ENSSSCG00000011678 | 13 | - | 83143347 | 83147313 | 367 | annot_exons |
| novel_circ_001430 | ENSSSCG00000006947 | 4 | + | 1.31E+08 | 1.31E+08 | 684 | annot_exons |
| novel_circ_001431 | ENSSSCG00000006071 | 4 | - | 37491558 | 37539512 | 621 | annot_exons |
| novel_circ_001432 | ENSSSCG00000011942 | 13 | + | 1.51E+08 | 1.51E+08 | 3097 | exon_intron |
| novel_circ_001433 | ENSSSCG00000006754 | 4 | + | 1.06E+08 | 1.06E+08 | 1253 | annot_exons |
| novel_circ_001434 | NA | 15 | - | 84268633 | 84268908 | 276 | intergenic |
| novel_circ_001435 | ENSSSCG00000016690 | 18 | - | 43940468 | 43946581 | 238 | annot_exons |
| novel_circ_001436 | ENSSSCG00000034139 | 1 | - | 30377848 | 30378392 | 545 | intronic |
| novel_circ_001437 | ENSSSCG00000030053 | 9 | + | 45877120 | 45886672 | 981 | annot_exons |
| novel_circ_001438 | ENSSSCG00000015743 | 15 | - | 31421363 | 31473618 | 604 | annot_exons |
| novel_circ_001439 | ENSSSCG00000021363 | 12 | + | 53146111 | 53148151 | 567 | exon_intron |
| novel_circ_001440 | ENSSSCG00000000736 | 5 | - | 67130804 | 67131900 | 294 | annot_exons |
| novel_circ_001441 | ENSSSCG00000015255 | 9 | - | 60489006 | 60490676 | 1671 | one_exon |
| novel_circ_001442 | ENSSSCG00000034386 | 14 | + | 31727597 | 31736163 | 1002 | annot_exons |
| novel_circ_001443 | ENSSSCG00000030467 | 13 | - | 1.09E+08 | 1.09E+08 | 258 | annot_exons |
| novel_circ_001444 | ENSSSCG00000005076 | 1 | + | 1.88E+08 | 1.88E+08 | 2918 | exon_intron |
| novel_circ_001445 | ENSSSCG00000026389 | 7 | - | 93921496 | 93928855 | 375 | annot_exons |
| novel_circ_001446 | ENSSSCG00000026536 | 11 | - | 78701356 | 78703726 | 567 | annot_exons |
| novel_circ_001447 | ENSSSCG00000003111 | 6 | + | 52977944 | 52982531 | 417 | annot_exons |
| novel_circ_001448 | ENSSSCG00000001921 | 7 | - | 60087536 | 60125010 | 1983 | annot_exons |
| novel_circ_001449 | ENSSSCG00000003872 | 6 | + | 1.61E+08 | 1.61E+08 | 412 | annot_exons |
| novel_circ_001450 | ENSSSCG00000012435 | X | + | 61895576 | 61895803 | 228 | antisense |
| novel_circ_001451 | ENSSSCG00000015743 | 15 | - | 31471618 | 31473618 | 2001 | exon_intron |
| novel_circ_001452 | ENSSSCG00000011025 | 10 | + | 42077538 | 42079536 | 1999 | intronic |
| novel_circ_001453 | ENSSSCG00000021042 | 13 | - | 2.03E+08 | 2.03E+08 | 1623 | annot_exons |
| novel_circ_001454 | ENSSSCG00000024999 | 2 | + | 1.09E+08 | 1.09E+08 | 627 | annot_exons |
| novel_circ_001455 | ENSSSCG00000014148 | 2 | - | 95657634 | 95680452 | 3191 | exon_intron |
| novel_circ_001456 | ENSSSCG00000010483 | 14 | + | 1.05E+08 | 1.05E+08 | 1555 | exon_intron |
| novel_circ_001457 | ENSSSCG00000014440 | 2 | + | 1.51E+08 | 1.51E+08 | 747 | annot_exons |
| novel_circ_001458 | ENSSSCG00000015851 | 15 | - | 55523679 | 55528577 | 587 | annot_exons |
| novel_circ_001459 | ENSSSCG00000027314 | 10 | + | 23313239 | 23321391 | 246 | annot_exons |
| novel_circ_001460 | ENSSSCG00000016033 | 15 | + | 93065192 | 93074570 | 3323 | exon_intron |
| novel_circ_001461 | ENSSSCG00000028317 | 2 | + | 82982339 | 82987046 | 403 | annot_exons |
| novel_circ_001462 | ENSSSCG00000006054 | 4 | - | 34195603 | 34210618 | 15016 | intronic |
| novel_circ_001463 | ENSSSCG00000017791 | 12 | - | 45870808 | 45913679 | 25978 | exon_intron |
| novel_circ_001464 | ENSSSCG00000010900 | 10 | - | 20423359 | 20492142 | 2814 | exon_intron |
| novel_circ_001465 | ENSSSCG00000029649 | 9 | - | 45849114 | 45850697 | 318 | annot_exons |
| novel_circ_001466 | NA | 15 | - | 84272781 | 84273093 | 313 | intergenic |
| novel_circ_001467 | ENSSSCG00000011467 | 13 | - | 39471074 | 39486529 | 445 | annot_exons |
| novel_circ_001468 | ENSSSCG00000024517 | 7 | - | 66992744 | 66996176 | 372 | annot_exons |
| novel_circ_001469 | ENSSSCG00000000158 | 5 | + | 12596908 | 12601083 | 596 | annot_exons |
| novel_circ_001470 | ENSSSCG00000006622 | 4 | + | 97796454 | 97797983 | 284 | annot_exons |
| novel_circ_001471 | ENSSSCG00000024517 | 7 | - | 66960886 | 66961101 | 216 | one_exon |
| novel_circ_001472 | ENSSSCG00000004071 | 1 | + | 12382021 | 12388087 | 272 | annot_exons |
| novel_circ_001473 | ENSSSCG00000009090 | 8 | - | 1.02E+08 | 1.02E+08 | 363 | annot_exons |
| novel_circ_001474 | ENSSSCG00000016863 | 16 | - | 26576176 | 26589063 | 516 | annot_exons |
| novel_circ_001475 | ENSSSCG00000006231 | 4 | - | 72576154 | 72577564 | 363 | annot_exons |
| novel_circ_001476 | ENSSSCG00000004595 | 1 | + | 1.13E+08 | 1.13E+08 | 957 | annot_exons |
| novel_circ_001477 | ENSSSCG00000000645 | 5 | - | 61760916 | 61762192 | 198 | annot_exons |
| novel_circ_001478 | ENSSSCG00000034768 | 3 | + | 4985156 | 4994312 | 574 | annot_exons |
| novel_circ_001479 | ENSSSCG00000001002 | 7 | + | 2396476 | 2397674 | 449 | annot_exons |
| novel_circ_001480 | ENSSSCG00000014946 | 9 | + | 26137742 | 26143371 | 463 | annot_exons |
| novel_circ_001481 | ENSSSCG00000011394 | 13 | + | 32466924 | 32475162 | 4163 | annot_exons |
| novel_circ_001482 | ENSSSCG00000010925 | 10 | + | 24509454 | 24524402 | 883 | annot_exons |
| novel_circ_001483 | ENSSSCG00000039996 | 9 | - | 7858804 | 7868251 | 575 | annot_exons |
| novel_circ_001484 | ENSSSCG00000013405 | 2 | + | 48670248 | 48671743 | 454 | annot_exons |
| novel_circ_001485 | ENSSSCG00000038913 | 14 | - | 71179829 | 71229432 | 1463 | annot_exons |
| novel_circ_001486 | ENSSSCG00000022801 | 6 | + | 89127456 | 89131566 | 1146 | annot_exons |
| novel_circ_001487 | ENSSSCG00000033323 | 14 | - | 92304764 | 92309354 | 280 | annot_exons |
| novel_circ_001488 | ENSSSCG00000010508 | 14 | - | 1.08E+08 | 1.08E+08 | 196 | one_exon |
| novel_circ_001489 | ENSSSCG00000001621 | 7 | - | 36873609 | 36874486 | 254 | annot_exons |
| novel_circ_001490 | NA | 5 | - | 87035101 | 87055022 | 19922 | intergenic |
| novel_circ_001491 | ENSSSCG00000004293 | 1 | - | 54515845 | 54529700 | 491 | annot_exons |
| novel_circ_001492 | ENSSSCG00000034569 | 2 | + | 51346346 | 51380536 | 1380 | exon_intron |
| novel_circ_001493 | NA | 1 | - | 1.37E+08 | 1.37E+08 | 5126 | intergenic |
| novel_circ_001494 | ENSSSCG00000045716 | 8 | + | 66123998 | 66129631 | 5634 | exon_intron |
| novel_circ_001495 | ENSSSCG00000011022 | 10 | + | 41402999 | 41408711 | 256 | annot_exons |
| novel_circ_001496 | ENSSSCG00000004276 | 1 | + | 50832070 | 50838859 | 257 | annot_exons |
| novel_circ_001497 | ENSSSCG00000006074 | 4 | + | 38224332 | 38242684 | 369 | annot_exons |
| novel_circ_001498 | ENSSSCG00000015071 | 9 | - | 44436634 | 44449352 | 12719 | intronic |
| novel_circ_001499 | ENSSSCG00000023408 | 1 | + | 1.84E+08 | 1.84E+08 | 486 | intronic |
| novel_circ_001500 | ENSSSCG00000001878 | 7 | + | 58112449 | 58116041 | 234 | annot_exons |
| novel_circ_001501 | ENSSSCG00000001930 | 7 | + | 61022079 | 61022763 | 231 | annot_exons |
| novel_circ_001502 | NA | 15 | - | 84462271 | 84463973 | 1703 | intergenic |
| novel_circ_001503 | ENSSSCG00000007866 | 3 | + | 26325335 | 26329147 | 317 | annot_exons |
| novel_circ_001504 | ENSSSCG00000001493 | 7 | - | 28373900 | 28402891 | 327 | annot_exons |
| novel_circ_001505 | ENSSSCG00000011453 | 13 | - | 34928536 | 34929490 | 396 | annot_exons |
| novel_circ_001506 | ENSSSCG00000025606 | 9 | + | 36665959 | 36671678 | 715 | annot_exons |
| novel_circ_001507 | ENSSSCG00000017735 | 12 | - | 42740088 | 42750757 | 540 | annot_exons |
| novel_circ_001508 | ENSSSCG00000016397 | 15 | + | 665260 | 665571 | 312 | one_exon |
| novel_circ_001509 | ENSSSCG00000015743 | 15 | - | 31366888 | 31383831 | 368 | annot_exons |
| novel_circ_001510 | ENSSSCG00000029578 | 6 | + | 28711467 | 28754026 | 1995 | annot_exons |
| novel_circ_001511 | ENSSSCG00000004538 | 1 | + | 1.06E+08 | 1.06E+08 | 431 | annot_exons |
| novel_circ_001512 | ENSSSCG00000009283 | 11 | + | 2610396 | 2614989 | 4594 | intronic |
| novel_circ_001513 | NA | 15 | + | 84420412 | 84421132 | 721 | intergenic |
| novel_circ_001514 | ENSSSCG00000023596 | 14 | - | 60980020 | 61028624 | 24526 | exon_intron |
| novel_circ_001515 | ENSSSCG00000017836 | 12 | + | 48710747 | 48712536 | 367 | annot_exons |
| novel_circ_001516 | NA | 1 | + | 2.42E+08 | 2.43E+08 | 22437 | intergenic |
| novel_circ_001517 | ENSSSCG00000014838 | 9 | - | 8695489 | 8704110 | 548 | annot_exons |
| novel_circ_001518 | ENSSSCG00000029633 | 6 | + | 1.57E+08 | 1.57E+08 | 1098 | annot_exons |
| novel_circ_001519 | ENSSSCG00000016717 | 18 | - | 47629573 | 47656999 | 653 | annot_exons |
| novel_circ_001520 | ENSSSCG00000008105 | 3 | + | 44544594 | 44546528 | 361 | annot_exons |
| novel_circ_001521 | ENSSSCG00000027765 | 1 | + | 1.75E+08 | 1.75E+08 | 426 | annot_exons |
| novel_circ_001522 | ENSSSCG00000029633 | 6 | + | 1.57E+08 | 1.57E+08 | 485 | annot_exons |
| novel_circ_001523 | ENSSSCG00000017198 | 12 | + | 5469594 | 5470566 | 406 | annot_exons |
| novel_circ_001524 | ENSSSCG00000017401 | 12 | - | 20329572 | 20332068 | 306 | annot_exons |
| novel_circ_001525 | ENSSSCG00000009408 | 11 | - | 20710449 | 20719541 | 326 | annot_exons |
| novel_circ_001526 | ENSSSCG00000021837 | 3 | + | 10186105 | 10193809 | 357 | annot_exons |
| novel_circ_001527 | ENSSSCG00000027293 | 16 | + | 49087201 | 49090713 | 3513 | exon_intron |
| novel_circ_001528 | ENSSSCG00000030528 | 13 | - | 1.92E+08 | 1.92E+08 | 332 | annot_exons |
| novel_circ_001529 | ENSSSCG00000018049 | 12 | - | 60348042 | 60376568 | 982 | annot_exons |
| novel_circ_001530 | ENSSSCG00000038007 | 10 | - | 43872033 | 43918659 | 31631 | exon_intron |
| novel_circ_001531 | ENSSSCG00000014364 | 2 | + | 1.42E+08 | 1.42E+08 | 936 | annot_exons |
| novel_circ_001532 | ENSSSCG00000000509 | 5 | - | 35645986 | 35647472 | 410 | annot_exons |
| novel_circ_001533 | ENSSSCG00000014791 | 9 | - | 6040783 | 6042995 | 326 | annot_exons |
| novel_circ_001534 | ENSSSCG00000011684 | 13 | + | 83716747 | 83719105 | 231 | annot_exons |
| novel_circ_001535 | ENSSSCG00000012884 | 2 | - | 4454067 | 4479961 | 1117 | annot_exons |
| novel_circ_001536 | ENSSSCG00000006987 | 17 | + | 5246400 | 5247965 | 547 | annot_exons |
| novel_circ_001537 | ENSSSCG00000004951 | 1 | - | 1.65E+08 | 1.65E+08 | 271 | annot_exons |
| novel_circ_001538 | ENSSSCG00000029185 | 3 | - | 1.12E+08 | 1.12E+08 | 311 | annot_exons |
| novel_circ_001539 | ENSSSCG00000002523 | AEMK02000452.1 | - | 1677528 | 1679425 | 462 | annot_exons |
| novel_circ_001540 | ENSSSCG00000013284 | 2 | - | 18855295 | 18856176 | 414 | annot_exons |
| novel_circ_001541 | ENSSSCG00000025942 | 13 | - | 1.21E+08 | 1.21E+08 | 294 | annot_exons |
| novel_circ_001542 | ENSSSCG00000009856 | 14 | + | 35261305 | 35280536 | 874 | annot_exons |
| novel_circ_001543 | ENSSSCG00000009320 | 11 | - | 5766388 | 5767129 | 324 | annot_exons |
| novel_circ_001544 | ENSSSCG00000023133 | 15 | + | 84066275 | 84079703 | 570 | annot_exons |
| novel_circ_001545 | ENSSSCG00000022676 | X | - | 54286536 | 54307691 | 577 | annot_exons |
| novel_circ_001546 | ENSSSCG00000015326 | 9 | - | 74208494 | 74209795 | 1302 | antisense |
| novel_circ_001547 | ENSSSCG00000000583 | 5 | - | 51999867 | 52000836 | 236 | annot_exons |
| novel_circ_001548 | ENSSSCG00000015327 | 9 | + | 74337574 | 74342716 | 414 | annot_exons |
| novel_circ_001549 | ENSSSCG00000028431 | 2 | + | 1.23E+08 | 1.23E+08 | 240 | annot_exons |
| novel_circ_001550 | ENSSSCG00000011811 | 13 | + | 1.26E+08 | 1.26E+08 | 334 | intronic |
| novel_circ_001551 | ENSSSCG00000021106 | X | - | 43052047 | 43052598 | 309 | annot_exons |
| novel_circ_001552 | ENSSSCG00000036501 | 8 | - | 6434817 | 6436243 | 329 | annot_exons |
| novel_circ_001553 | ENSSSCG00000015283 | 9 | - | 65087405 | 65088680 | 437 | annot_exons |
| novel_circ_001554 | ENSSSCG00000011417 | 13 | - | 33727416 | 33738611 | 1071 | annot_exons |
| novel_circ_001555 | ENSSSCG00000004766 | 1 | - | 1.31E+08 | 1.31E+08 | 393 | annot_exons |
| novel_circ_001556 | ENSSSCG00000011278 | 13 | + | 25919411 | 25929968 | 2496 | annot_exons |
| novel_circ_001557 | ENSSSCG00000026354 | 10 | - | 48164054 | 48164543 | 490 | exon_intron |
| novel_circ_001558 | ENSSSCG00000034704 | 1 | - | 1.62E+08 | 1.62E+08 | 862 | annot_exons |
| novel_circ_001559 | ENSSSCG00000011845 | 13 | + | 1.34E+08 | 1.34E+08 | 777 | annot_exons |
| novel_circ_001560 | ENSSSCG00000016438 | 18 | - | 5906950 | 5908995 | 448 | annot_exons |
| novel_circ_001561 | ENSSSCG00000000784 | 5 | + | 71922092 | 71930617 | 919 | annot_exons |
| novel_circ_001562 | ENSSSCG00000012474 | X | + | 78878048 | 78885018 | 529 | annot_exons |
| novel_circ_001563 | ENSSSCG00000034395 | 15 | + | 1.11E+08 | 1.11E+08 | 439 | annot_exons |
| novel_circ_001564 | ENSSSCG00000038437 | AEMK02000682.1 | - | 262604 | 278294 | 1158 | annot_exons |
| novel_circ_001565 | ENSSSCG00000004415 | 1 | + | 75412186 | 75414546 | 372 | annot_exons |
| novel_circ_001566 | ENSSSCG00000014304 | 2 | + | 1.37E+08 | 1.37E+08 | 469 | annot_exons |
| novel_circ_001567 | ENSSSCG00000002502 | 7 | - | 1.18E+08 | 1.18E+08 | 1719 | annot_exons |
| novel_circ_001568 | ENSSSCG00000003872 | 6 | + | 1.61E+08 | 1.61E+08 | 759 | annot_exons |
| novel_circ_001569 | ENSSSCG00000034074 | 3 | + | 25241093 | 25241773 | 280 | annot_exons |
| novel_circ_001570 | ENSSSCG00000003660 | 6 | - | 95386908 | 95392519 | 950 | annot_exons |
| novel_circ_001571 | ENSSSCG00000035281 | 1 | + | 55666252 | 55669095 | 370 | annot_exons |
| novel_circ_001572 | ENSSSCG00000004089 | 1 | + | 14817548 | 14851934 | 891 | annot_exons |
| novel_circ_001573 | ENSSSCG00000017063 | 16 | + | 66802386 | 66803088 | 703 | antisense |
| novel_circ_001574 | ENSSSCG00000014955 | 9 | - | 26589400 | 26590851 | 304 | annot_exons |
| novel_circ_001575 | ENSSSCG00000036480 | 14 | - | 69354719 | 69383275 | 28557 | exon_intron |
| novel_circ_001576 | ENSSSCG00000016412 | 18 | + | 1875193 | 1899899 | 415 | annot_exons |
| novel_circ_001577 | ENSSSCG00000004927 | 1 | - | 1.63E+08 | 1.63E+08 | 544 | annot_exons |
| novel_circ_001578 | ENSSSCG00000000970 | 5 | - | 293303 | 303321 | 549 | annot_exons |
| novel_circ_001579 | ENSSSCG00000008452 | 3 | + | 96527643 | 96533576 | 827 | annot_exons |
| novel_circ_001580 | ENSSSCG00000006084 | 4 | - | 39069243 | 39076931 | 224 | annot_exons |
| novel_circ_001581 | ENSSSCG00000017895 | 12 | - | 51640771 | 51654962 | 596 | annot_exons |
| novel_circ_001582 | ENSSSCG00000001485 | 7 | - | 26689737 | 26690129 | 393 | one_exon |
| novel_circ_001583 | ENSSSCG00000029578 | 6 | + | 28711467 | 28748110 | 1812 | annot_exons |
| novel_circ_001584 | ENSSSCG00000009680 | 14 | + | 12530404 | 12546352 | 402 | annot_exons |
| novel_circ_001585 | ENSSSCG00000001863 | 7 | + | 56364029 | 56367247 | 320 | annot_exons |
| novel_circ_001586 | ENSSSCG00000027681 | 16 | - | 18601794 | 18606977 | 429 | annot_exons |
| novel_circ_001587 | ENSSSCG00000011243 | 13 | - | 22206116 | 22206383 | 268 | antisense |
| novel_circ_001588 | ENSSSCG00000001878 | 7 | + | 58112449 | 58117178 | 359 | annot_exons |
| novel_circ_001589 | ENSSSCG00000028148 | X | - | 29094935 | 29095226 | 292 | intronic |
| novel_circ_001590 | ENSSSCG00000016520 | 18 | + | 11609213 | 11631134 | 481 | annot_exons |
| novel_circ_001591 | ENSSSCG00000001076 | 7 | + | 14499880 | 14517025 | 17146 | intronic |
| novel_circ_001592 | ENSSSCG00000000214 | 5 | + | 16014092 | 16014393 | 302 | one_exon |
| novel_circ_001593 | ENSSSCG00000000278 | 5 | - | 18742363 | 18745284 | 525 | annot_exons |
| novel_circ_001594 | ENSSSCG00000007460 | 17 | + | 50689571 | 50695566 | 407 | annot_exons |
| novel_circ_001595 | ENSSSCG00000007815 | 3 | + | 19421276 | 19423629 | 338 | annot_exons |
| novel_circ_001596 | ENSSSCG00000018049 | 12 | - | 60376419 | 60376920 | 245 | annot_exons |
| novel_circ_001597 | ENSSSCG00000007188 | 17 | + | 33928517 | 33932965 | 278 | annot_exons |
| novel_circ_001598 | ENSSSCG00000029621 | 8 | - | 1.25E+08 | 1.25E+08 | 491 | annot_exons |
| novel_circ_001599 | ENSSSCG00000022895 | 13 | - | 1.62E+08 | 1.62E+08 | 549 | annot_exons |
| novel_circ_001600 | ENSSSCG00000011813 | 13 | - | 1.27E+08 | 1.27E+08 | 1479 | exon_intron |
| novel_circ_001601 | ENSSSCG00000001806 | 7 | + | 52175903 | 52183206 | 576 | annot_exons |
| novel_circ_001602 | NA | 3 | - | 17773612 | 17776837 | 3226 | intergenic |
| novel_circ_001603 | ENSSSCG00000005444 | 1 | - | 2.5E+08 | 2.5E+08 | 2006 | annot_exons |
| novel_circ_001604 | ENSSSCG00000016125 | 15 | - | 1.09E+08 | 1.09E+08 | 1198 | annot_exons |
| novel_circ_001605 | ENSSSCG00000018049 | 12 | - | 60366858 | 60370222 | 289 | annot_exons |
| novel_circ_001606 | ENSSSCG00000014901 | 9 | + | 17302044 | 17323832 | 6432 | annot_exons |
| novel_circ_001607 | ENSSSCG00000045048 | 14 | - | 41674858 | 41683814 | 8957 | antisense |
| novel_circ_001608 | ENSSSCG00000008692 | 8 | + | 1687981 | 1688323 | 243 | annot_exons |
| novel_circ_001609 | ENSSSCG00000005096 | 1 | + | 1.91E+08 | 1.91E+08 | 738 | annot_exons |
| novel_circ_001610 | ENSSSCG00000013314 | 2 | - | 27962402 | 27976384 | 1187 | annot_exons |
| novel_circ_001611 | ENSSSCG00000006137 | 4 | - | 50413105 | 50416871 | 237 | annot_exons |
| novel_circ_001612 | ENSSSCG00000001852 | 7 | - | 55655839 | 55658193 | 402 | annot_exons |
| novel_circ_001613 | ENSSSCG00000011918 | 13 | - | 1.46E+08 | 1.46E+08 | 153 | antisense |
| novel_circ_001614 | ENSSSCG00000025323 | 6 | + | 13530441 | 13538010 | 566 | annot_exons |
| novel_circ_001615 | ENSSSCG00000012817 | X | + | 1.25E+08 | 1.25E+08 | 227 | annot_exons |
| novel_circ_001616 | ENSSSCG00000025942 | 13 | - | 1.21E+08 | 1.21E+08 | 180 | annot_exons |
| novel_circ_001617 | ENSSSCG00000016397 | 15 | + | 712763 | 715420 | 210 | annot_exons |
| novel_circ_001618 | ENSSSCG00000011775 | 13 | + | 1.22E+08 | 1.22E+08 | 1543 | exon_intron |
| novel_circ_001619 | ENSSSCG00000032622 | 14 | + | 6766438 | 6770693 | 414 | annot_exons |
| novel_circ_001620 | ENSSSCG00000016434 | 18 | - | 5795914 | 5796502 | 589 | antisense |
| novel_circ_001621 | ENSSSCG00000039460 | 6 | + | 59556982 | 59557369 | 293 | annot_exons |
| novel_circ_001622 | ENSSSCG00000026940 | 15 | + | 1.05E+08 | 1.05E+08 | 735 | annot_exons |
| novel_circ_001623 | ENSSSCG00000004634 | 1 | - | 1.21E+08 | 1.21E+08 | 392 | annot_exons |
| novel_circ_001624 | ENSSSCG00000011616 | 13 | + | 71719658 | 71720414 | 320 | annot_exons |
| novel_circ_001625 | ENSSSCG00000027415 | 6 | - | 9366417 | 9464830 | 98414 | intronic |
| novel_circ_001626 | ENSSSCG00000016769 | 18 | - | 54336795 | 54349529 | 427 | annot_exons |
| novel_circ_001627 | ENSSSCG00000016017 | 15 | + | 88241812 | 88244842 | 492 | annot_exons |
| novel_circ_001628 | ENSSSCG00000023976 | 5 | - | 68936139 | 68938678 | 221 | annot_exons |
| novel_circ_001629 | ENSSSCG00000004844 | 1 | + | 1.44E+08 | 1.44E+08 | 3212 | exon_intron |
| novel_circ_001630 | ENSSSCG00000029633 | 6 | + | 1.57E+08 | 1.57E+08 | 392 | annot_exons |
| novel_circ_001631 | ENSSSCG00000004535 | 1 | - | 1.05E+08 | 1.05E+08 | 360 | annot_exons |
| novel_circ_001632 | ENSSSCG00000030435 | 3 | - | 59134007 | 59138942 | 561 | annot_exons |
| novel_circ_001633 | ENSSSCG00000014110 | 2 | - | 87745803 | 87760449 | 586 | annot_exons |
| novel_circ_001634 | ENSSSCG00000000900 | 5 | - | 88056782 | 88064420 | 562 | annot_exons |
| novel_circ_001635 | ENSSSCG00000036926 | 1 | + | 2.67E+08 | 2.67E+08 | 6467 | exon_intron |
| novel_circ_001636 | ENSSSCG00000029219 | 13 | + | 2.03E+08 | 2.03E+08 | 267 | annot_exons |
| novel_circ_001637 | ENSSSCG00000010247 | 14 | + | 72044646 | 72048880 | 559 | annot_exons |
| novel_circ_001638 | ENSSSCG00000033377 | 3 | + | 6981006 | 6993212 | 4025 | annot_exons |
| novel_circ_001639 | ENSSSCG00000016113 | 15 | + | 1.06E+08 | 1.06E+08 | 285 | annot_exons |
| novel_circ_001640 | ENSSSCG00000011045 | 10 | + | 45992309 | 46022353 | 707 | annot_exons |
| novel_circ_001641 | ENSSSCG00000000405 | 5 | - | 21986592 | 21987400 | 415 | annot_exons |
| novel_circ_001642 | ENSSSCG00000015355 | 9 | - | 84105161 | 84119222 | 295 | annot_exons |
| novel_circ_001643 | ENSSSCG00000014794 | 9 | + | 6374485 | 6376708 | 322 | annot_exons |
| novel_circ_001644 | ENSSSCG00000010640 | 14 | - | 1.24E+08 | 1.24E+08 | 1068 | annot_exons |
| novel_circ_001645 | ENSSSCG00000014267 | 2 | - | 1.34E+08 | 1.34E+08 | 17428 | exon_intron |
| novel_circ_001646 | ENSSSCG00000016578 | 18 | - | 19768651 | 19769074 | 347 | annot_exons |
| novel_circ_001647 | ENSSSCG00000020702 | 1 | - | 90220788 | 90255853 | 480 | annot_exons |
| novel_circ_001648 | ENSSSCG00000001960 | 7 | + | 65245623 | 65253820 | 510 | annot_exons |
| novel_circ_001649 | ENSSSCG00000003547 | 6 | + | 82220584 | 82222549 | 346 | annot_exons |
| novel_circ_001650 | ENSSSCG00000007284 | 17 | - | 38151936 | 38156523 | 4588 | exon_intron |
| novel_circ_001651 | ENSSSCG00000027057 | 2 | + | 7063694 | 7064327 | 544 | annot_exons |
| novel_circ_001652 | ENSSSCG00000016113 | 15 | + | 1.06E+08 | 1.06E+08 | 1280 | one_exon |
| novel_circ_001653 | ENSSSCG00000005720 | 1 | - | 2.72E+08 | 2.72E+08 | 225 | annot_exons |
| novel_circ_001654 | NA | 7 | + | 68200000 | 68212641 | 12642 | intergenic |
| novel_circ_001655 | ENSSSCG00000016958 | 16 | + | 46502180 | 46503607 | 414 | annot_exons |
| novel_circ_001656 | ENSSSCG00000008575 | 3 | + | 1.13E+08 | 1.13E+08 | 535 | annot_exons |
| novel_circ_001657 | ENSSSCG00000006619 | 4 | - | 97566524 | 97583160 | 6818 | exon_intron |
| novel_circ_001658 | ENSSSCG00000048856 | 2 | - | 6754669 | 6754973 | 305 | one_exon |
| novel_circ_001659 | ENSSSCG00000038867 | 8 | + | 1.31E+08 | 1.31E+08 | 311 | annot_exons |
| novel_circ_001660 | ENSSSCG00000004344 | 1 | - | 64395020 | 64445410 | 923 | annot_exons |
| novel_circ_001661 | ENSSSCG00000031271 | 15 | + | 1.05E+08 | 1.05E+08 | 468 | annot_exons |
| novel_circ_001662 | ENSSSCG00000023130 | 14 | - | 75008341 | 75032788 | 382 | annot_exons |
| novel_circ_001663 | ENSSSCG00000022830 | 15 | - | 1.13E+08 | 1.13E+08 | 462 | annot_exons |
| novel_circ_001664 | ENSSSCG00000015826 | 15 | - | 48628324 | 48629428 | 242 | annot_exons |
| novel_circ_001665 | ENSSSCG00000009994 | 14 | + | 46978000 | 46985309 | 283 | annot_exons |
| novel_circ_001666 | ENSSSCG00000004985 | 1 | + | 1.7E+08 | 1.7E+08 | 551 | annot_exons |
| novel_circ_001667 | ENSSSCG00000006066 | 4 | + | 36710955 | 36715733 | 423 | annot_exons |
| novel_circ_001668 | ENSSSCG00000008312 | 3 | - | 71155268 | 71183965 | 701 | annot_exons |
| novel_circ_001669 | ENSSSCG00000013106 | 2 | + | 10897114 | 10897382 | 269 | one_exon |
| novel_circ_001670 | ENSSSCG00000013366 | 2 | - | 40814681 | 40814886 | 206 | one_exon |
| novel_circ_001671 | ENSSSCG00000040887 | 6 | - | 34617617 | 34621968 | 395 | annot_exons |
| novel_circ_001672 | ENSSSCG00000008412 | 3 | + | 87298791 | 87301882 | 371 | annot_exons |
| novel_circ_001673 | ENSSSCG00000010952 | 10 | + | 29037750 | 29038934 | 370 | annot_exons |
| novel_circ_001674 | ENSSSCG00000004748 | 1 | + | 1.3E+08 | 1.3E+08 | 256 | annot_exons |
| novel_circ_001675 | ENSSSCG00000004979 | 1 | - | 1.7E+08 | 1.7E+08 | 1541 | annot_exons |
| novel_circ_001676 | ENSSSCG00000008803 | 8 | - | 33602279 | 33603766 | 215 | annot_exons |
| novel_circ_001677 | ENSSSCG00000014173 | 2 | + | 1.04E+08 | 1.04E+08 | 525 | annot_exons |
| novel_circ_001678 | ENSSSCG00000036123 | 8 | - | 1.36E+08 | 1.36E+08 | 429 | annot_exons |
| novel_circ_001679 | ENSSSCG00000001862 | 7 | - | 56448160 | 56448662 | 271 | exon_intron |
| novel_circ_001680 | ENSSSCG00000002266 | 7 | - | 86031558 | 86032780 | 299 | annot_exons |
| novel_circ_001681 | ENSSSCG00000009766 | 14 | - | 29281864 | 29290551 | 531 | annot_exons |
| novel_circ_001682 | NA | 15 | - | 84235019 | 84235183 | 165 | intergenic |
| novel_circ_001683 | ENSSSCG00000026733 | 18 | + | 10084528 | 10087363 | 272 | annot_exons |
| novel_circ_001684 | ENSSSCG00000015273 | 9 | + | 64376217 | 64376489 | 273 | intronic |
| novel_circ_001685 | ENSSSCG00000015670 | 15 | + | 7782677 | 7785694 | 268 | annot_exons |
| novel_circ_001686 | ENSSSCG00000014112 | 2 | + | 88043811 | 88057945 | 325 | annot_exons |
| novel_circ_001687 | ENSSSCG00000016397 | 15 | + | 606063 | 606269 | 207 | one_exon |
| novel_circ_001688 | NA | 6 | + | 47382889 | 47384422 | 1534 | intergenic |
| novel_circ_001689 | ENSSSCG00000037530 | 14 | - | 1.32E+08 | 1.32E+08 | 217 | antisense |
| novel_circ_001690 | ENSSSCG00000011243 | 13 | + | 22216776 | 22239729 | 471 | annot_exons |
| novel_circ_001691 | ENSSSCG00000015874 | 15 | - | 64759481 | 64788962 | 852 | annot_exons |
| novel_circ_001692 | ENSSSCG00000037556 | 9 | - | 1.07E+08 | 1.07E+08 | 174 | annot_exons |
| novel_circ_001693 | ENSSSCG00000023409 | 3 | + | 72268575 | 72270592 | 475 | annot_exons |
| novel_circ_001694 | ENSSSCG00000034518 | 1 | - | 2.7E+08 | 2.7E+08 | 304 | annot_exons |
| novel_circ_001695 | ENSSSCG00000011831 | 13 | - | 1.33E+08 | 1.33E+08 | 361 | annot_exons |
| novel_circ_001696 | ENSSSCG00000028228 | 3 | + | 80259117 | 80268798 | 2785 | annot_exons |
| novel_circ_001697 | ENSSSCG00000015428 | 9 | - | 1.05E+08 | 1.05E+08 | 451 | annot_exons |
| novel_circ_001698 | ENSSSCG00000000915 | 5 | + | 91713772 | 91713922 | 151 | one_exon |
| novel_circ_001699 | ENSSSCG00000016033 | 15 | + | 93166360 | 93166605 | 246 | intronic |
| novel_circ_001700 | ENSSSCG00000006179 | 4 | + | 62316217 | 62325616 | 9326 | exon_intron |
| novel_circ_001701 | ENSSSCG00000011578 | 13 | - | 67934546 | 67936021 | 266 | annot_exons |
| novel_circ_001702 | ENSSSCG00000038617 | 17 | + | 63033432 | 63044659 | 736 | annot_exons |
| novel_circ_001703 | ENSSSCG00000031954 | 15 | + | 99701720 | 99703874 | 359 | annot_exons |
| novel_circ_001704 | ENSSSCG00000028406 | 1 | - | 2.02E+08 | 2.02E+08 | 1487 | annot_exons |
| novel_circ_001705 | ENSSSCG00000035595 | 9 | + | 1.27E+08 | 1.27E+08 | 240 | annot_exons |
| novel_circ_001706 | ENSSSCG00000036132 | 6 | + | 51704350 | 51705156 | 807 | antisense |
| novel_circ_001707 | ENSSSCG00000003749 | 6 | + | 1.26E+08 | 1.26E+08 | 331 | annot_exons |
| novel_circ_001708 | ENSSSCG00000006277 | 4 | - | 80170780 | 80183572 | 764 | annot_exons |
| novel_circ_001709 | ENSSSCG00000010734 | 14 | - | 1.34E+08 | 1.34E+08 | 636 | annot_exons |
| novel_circ_001710 | ENSSSCG00000006903 | 4 | - | 1.25E+08 | 1.25E+08 | 369 | annot_exons |
| novel_circ_001711 | ENSSSCG00000007951 | 3 | + | 38470924 | 38472523 | 355 | annot_exons |
| novel_circ_001712 | ENSSSCG00000006872 | 4 | - | 1.18E+08 | 1.18E+08 | 532 | annot_exons |
| novel_circ_001713 | ENSSSCG00000017264 | 12 | - | 11892148 | 11892556 | 303 | annot_exons |
| novel_circ_001714 | ENSSSCG00000001499 | 7 | + | 29251685 | 29253972 | 276 | annot_exons |
| novel_circ_001715 | ENSSSCG00000036909 | 6 | + | 1.67E+08 | 1.67E+08 | 3851 | exon_intron |
| novel_circ_001716 | ENSSSCG00000013235 | 2 | - | 15273036 | 15274126 | 1091 | one_exon |
| novel_circ_001717 | ENSSSCG00000007097 | 17 | + | 26773532 | 26783795 | 441 | annot_exons |
| novel_circ_001718 | ENSSSCG00000045565 | 9 | - | 1.02E+08 | 1.02E+08 | 21873 | exon_intron |
| novel_circ_001719 | ENSSSCG00000018009 | 12 | + | 55430702 | 55432293 | 740 | annot_exons |
| novel_circ_001720 | NA | 15 | - | 84267670 | 84267885 | 216 | intergenic |
| novel_circ_001721 | ENSSSCG00000011241 | 13 | + | 22062201 | 22064050 | 1850 | one_exon |
| novel_circ_001722 | ENSSSCG00000016330 | 15 | + | 1.37E+08 | 1.37E+08 | 93 | annot_exons |
| novel_circ_001723 | ENSSSCG00000035657 | AEMK02000328.1 | - | 269331 | 289172 | 1234 | annot_exons |
| novel_circ_001724 | ENSSSCG00000012251 | X | + | 36818801 | 36835577 | 873 | annot_exons |
| novel_circ_001725 | ENSSSCG00000015747 | 15 | + | 33555033 | 33557679 | 304 | annot_exons |
| novel_circ_001726 | ENSSSCG00000015943 | 15 | + | 77115558 | 77127562 | 874 | annot_exons |
| novel_circ_001727 | NA | 15 | + | 84427540 | 84460250 | 32711 | intergenic |
| novel_circ_001728 | ENSSSCG00000004901 | 1 | + | 1.59E+08 | 1.59E+08 | 322 | annot_exons |
| novel_circ_001729 | ENSSSCG00000034191 | 2 | + | 42471337 | 42476652 | 5316 | exon_intron |
| novel_circ_001730 | ENSSSCG00000020879 | 8 | + | 76608764 | 76609327 | 564 | one_exon |
| novel_circ_001731 | ENSSSCG00000004064 | 1 | - | 9873358 | 9923857 | 514 | annot_exons |
| novel_circ_001732 | ENSSSCG00000015859 | 15 | + | 59325224 | 59328343 | 362 | annot_exons |
| novel_circ_001733 | ENSSSCG00000039506 | 5 | - | 21536633 | 21543230 | 6598 | antisense |
| novel_circ_001734 | ENSSSCG00000016397 | 15 | + | 707503 | 712867 | 426 | annot_exons |
| novel_circ_001735 | ENSSSCG00000023593 | 8 | - | 1.32E+08 | 1.32E+08 | 423 | one_exon |
| novel_circ_001736 | ENSSSCG00000006508 | 4 | + | 94405984 | 94446665 | 879 | annot_exons |
| novel_circ_001737 | ENSSSCG00000027389 | 18 | + | 38309526 | 38324769 | 720 | annot_exons |
| novel_circ_001738 | ENSSSCG00000006274 | 4 | + | 79834117 | 79836538 | 437 | annot_exons |
| novel_circ_001739 | ENSSSCG00000011022 | 10 | + | 41402999 | 41416627 | 404 | annot_exons |
| novel_circ_001740 | ENSSSCG00000029275 | 8 | - | 17865660 | 17867484 | 1142 | annot_exons |
| novel_circ_001741 | ENSSSCG00000017155 | 12 | + | 2288303 | 2307514 | 698 | annot_exons |
| novel_circ_001742 | ENSSSCG00000035639 | 6 | - | 27529757 | 27533552 | 372 | annot_exons |
| novel_circ_001743 | ENSSSCG00000002455 | 7 | + | 1.14E+08 | 1.14E+08 | 375 | annot_exons |
| novel_circ_001744 | ENSSSCG00000038561 | 15 | - | 84412322 | 84412891 | 425 | annot_exons |
| novel_circ_001745 | ENSSSCG00000013402 | 2 | - | 47514094 | 47519641 | 323 | annot_exons |
| novel_circ_001746 | ENSSSCG00000011593 | 13 | - | 69039990 | 69043142 | 555 | annot_exons |
| novel_circ_001747 | ENSSSCG00000016194 | 15 | - | 1.21E+08 | 1.21E+08 | 396 | annot_exons |
| novel_circ_001748 | ENSSSCG00000007142 | 17 | - | 31807232 | 31813630 | 681 | annot_exons |
| novel_circ_001749 | ENSSSCG00000004636 | 1 | + | 1.21E+08 | 1.21E+08 | 1012 | annot_exons |
| novel_circ_001750 | ENSSSCG00000037556 | 9 | - | 1.07E+08 | 1.07E+08 | 593 | annot_exons |
| novel_circ_001751 | ENSSSCG00000008079 | 3 | + | 42881922 | 42920029 | 1082 | annot_exons |
| novel_circ_001752 | ENSSSCG00000004753 | 1 | + | 1.3E+08 | 1.3E+08 | 336 | annot_exons |
| novel_circ_001753 | NA | 1 | + | 2.19E+08 | 2.19E+08 | 821 | intergenic |
| novel_circ_001754 | ENSSSCG00000001810 | 7 | - | 52458294 | 52485880 | 698 | annot_exons |
| novel_circ_001755 | ENSSSCG00000001865 | 7 | - | 56629386 | 56635081 | 388 | annot_exons |
| novel_circ_001756 | ENSSSCG00000011041 | 10 | - | 44884087 | 44890725 | 1021 | annot_exons |
| novel_circ_001757 | ENSSSCG00000014949 | 9 | + | 26202581 | 26207033 | 454 | annot_exons |
| novel_circ_001758 | ENSSSCG00000011045 | 10 | + | 45992309 | 46032747 | 5864 | exon_intron |
| novel_circ_001759 | ENSSSCG00000025751 | 3 | + | 78309126 | 78318136 | 9011 | antisense |
| novel_circ_001760 | ENSSSCG00000008068 | 3 | - | 41951780 | 41952939 | 297 | annot_exons |
| novel_circ_001761 | ENSSSCG00000013245 | 2 | + | 15399072 | 15399174 | 103 | intronic |
| novel_circ_001762 | ENSSSCG00000004328 | 1 | - | 57788272 | 57790359 | 276 | annot_exons |
| novel_circ_001763 | ENSSSCG00000015867 | 15 | - | 60412608 | 60414442 | 232 | annot_exons |
| novel_circ_001764 | ENSSSCG00000017995 | 12 | + | 54640778 | 54641183 | 406 | one_exon |
| novel_circ_001765 | ENSSSCG00000007074 | 17 | - | 22215079 | 22219999 | 4921 | exon_intron |
| novel_circ_001766 | ENSSSCG00000004739 | 1 | - | 1.3E+08 | 1.3E+08 | 956 | one_exon |
| novel_circ_001767 | ENSSSCG00000014146 | 2 | + | 94938475 | 94942578 | 189 | annot_exons |
| novel_circ_001768 | ENSSSCG00000009338 | 11 | + | 8670472 | 8686208 | 694 | annot_exons |
| novel_circ_001769 | ENSSSCG00000011752 | 13 | + | 1.11E+08 | 1.11E+08 | 244 | one_exon |
| novel_circ_001770 | ENSSSCG00000010627 | 14 | + | 1.21E+08 | 1.21E+08 | 563 | annot_exons |
| novel_circ_001771 | ENSSSCG00000017877 | 12 | - | 50266696 | 50270301 | 302 | annot_exons |
| novel_circ_001772 | NA | 15 | + | 84282997 | 84283411 | 415 | intergenic |
| novel_circ_001773 | ENSSSCG00000011226 | 13 | - | 16848022 | 16848288 | 267 | antisense |
| novel_circ_001774 | ENSSSCG00000011103 | 10 | - | 57250368 | 57317736 | 484 | annot_exons |
| novel_circ_001775 | ENSSSCG00000011103 | 10 | - | 57250368 | 57297790 | 308 | annot_exons |
| novel_circ_001776 | ENSSSCG00000003805 | 6 | - | 1.46E+08 | 1.46E+08 | 992 | intronic |
| novel_circ_001777 | ENSSSCG00000004901 | 1 | + | 1.59E+08 | 1.59E+08 | 717 | annot_exons |
| novel_circ_001778 | ENSSSCG00000014406 | 2 | - | 1.47E+08 | 1.47E+08 | 251 | intronic |
| novel_circ_001779 | ENSSSCG00000004729 | 1 | - | 1.29E+08 | 1.29E+08 | 875 | annot_exons |
| novel_circ_001780 | NA | 18 | - | 14244260 | 14244600 | 341 | intergenic |
| novel_circ_001781 | ENSSSCG00000029837 | 11 | + | 25318969 | 25321570 | 236 | annot_exons |
| novel_circ_001782 | ENSSSCG00000024784 | 10 | - | 23509173 | 23509335 | 163 | exon_intron |
| novel_circ_001783 | ENSSSCG00000002523 | AEMK02000452.1 | - | 1699023 | 1707701 | 329 | annot_exons |
| novel_circ_001784 | ENSSSCG00000006267 | 4 | + | 78124832 | 78125262 | 431 | one_exon |
| novel_circ_001785 | ENSSSCG00000045508 | 1 | + | 2.39E+08 | 2.39E+08 | 425 | annot_exons |
| novel_circ_001786 | ENSSSCG00000004524 | 1 | + | 1.01E+08 | 1.01E+08 | 1856 | annot_exons |
| novel_circ_001787 | ENSSSCG00000036926 | 1 | + | 2.67E+08 | 2.67E+08 | 3825 | annot_exons |
| novel_circ_001788 | ENSSSCG00000014165 | 2 | - | 1.02E+08 | 1.02E+08 | 318 | annot_exons |
| novel_circ_001789 | ENSSSCG00000016379 | 15 | - | 1.4E+08 | 1.4E+08 | 872 | annot_exons |
| novel_circ_001790 | ENSSSCG00000004620 | 1 | - | 1.19E+08 | 1.19E+08 | 461 | antisense |
| novel_circ_001791 | ENSSSCG00000001065 | 7 | - | 12556288 | 12616038 | 251 | annot_exons |
| novel_circ_001792 | ENSSSCG00000003486 | 6 | + | 76188097 | 76219804 | 793 | annot_exons |
| novel_circ_001793 | ENSSSCG00000004729 | 1 | - | 1.29E+08 | 1.29E+08 | 424 | annot_exons |
| novel_circ_001794 | ENSSSCG00000010190 | 14 | - | 60413808 | 60415661 | 1854 | antisense |
| novel_circ_001795 | ENSSSCG00000006870 | 4 | - | 1.18E+08 | 1.18E+08 | 807 | annot_exons |
| novel_circ_001796 | ENSSSCG00000011775 | 13 | + | 1.22E+08 | 1.22E+08 | 682 | annot_exons |
| novel_circ_001797 | ENSSSCG00000001499 | 7 | + | 29299227 | 29299947 | 721 | one_exon |
| novel_circ_001798 | ENSSSCG00000001076 | 7 | + | 14114293 | 14126171 | 3913 | annot_exons |
| novel_circ_001799 | ENSSSCG00000026004 | 5 | + | 29851336 | 29874465 | 15747 | exon_intron |
| novel_circ_001800 | ENSSSCG00000008127 | 3 | + | 46942944 | 46945219 | 579 | annot_exons |
| novel_circ_001801 | ENSSSCG00000008881 | 8 | + | 48158912 | 48159079 | 168 | one_exon |
| novel_circ_001802 | ENSSSCG00000016234 | 15 | - | 1.26E+08 | 1.26E+08 | 222 | annot_exons |
| novel_circ_001803 | ENSSSCG00000010485 | 14 | + | 1.06E+08 | 1.06E+08 | 478 | annot_exons |
| novel_circ_001804 | ENSSSCG00000011845 | 13 | + | 1.34E+08 | 1.34E+08 | 222 | annot_exons |
| novel_circ_001805 | ENSSSCG00000011214 | 13 | - | 12666411 | 12670563 | 361 | annot_exons |
| novel_circ_001806 | ENSSSCG00000000952 | 5 | - | 1.04E+08 | 1.04E+08 | 337 | annot_exons |
| novel_circ_001807 | ENSSSCG00000036145 | 6 | - | 1.56E+08 | 1.56E+08 | 727 | annot_exons |
| novel_circ_001808 | ENSSSCG00000015065 | 9 | - | 44101937 | 44145848 | 19417 | exon_intron |
| novel_circ_001809 | ENSSSCG00000006857 | 4 | + | 1.16E+08 | 1.16E+08 | 545 | annot_exons |
| novel_circ_001810 | ENSSSCG00000029920 | 8 | + | 87787563 | 87820356 | 657 | annot_exons |
| novel_circ_001811 | ENSSSCG00000012884 | 2 | - | 4431225 | 4442066 | 614 | annot_exons |
| novel_circ_001812 | ENSSSCG00000021005 | 10 | + | 31295417 | 31303258 | 690 | annot_exons |
| novel_circ_001813 | ENSSSCG00000034677 | 15 | + | 84406904 | 84407130 | 227 | antisense |
| novel_circ_001814 | ENSSSCG00000001756 | 7 | - | 47491693 | 47496150 | 660 | annot_exons |
| novel_circ_001815 | ENSSSCG00000042430 | 15 | + | 84424474 | 84424732 | 259 | antisense |
| novel_circ_001816 | ENSSSCG00000000939 | 5 | - | 1E+08 | 1E+08 | 470 | annot_exons |
| novel_circ_001817 | ENSSSCG00000024201 | 13 | - | 38540108 | 38545830 | 312 | annot_exons |
| novel_circ_001818 | ENSSSCG00000001971 | 7 | + | 68406557 | 68408974 | 461 | annot_exons |
| novel_circ_001819 | ENSSSCG00000004610 | 1 | + | 1.16E+08 | 1.16E+08 | 687 | annot_exons |
| novel_circ_001820 | ENSSSCG00000008513 | 3 | - | 1.07E+08 | 1.07E+08 | 604 | annot_exons |
| novel_circ_001821 | ENSSSCG00000011454 | 13 | - | 35056759 | 35058541 | 444 | annot_exons |
| novel_circ_001822 | NA | 8 | - | 85915786 | 85917156 | 1371 | intergenic |
| novel_circ_001823 | ENSSSCG00000005983 | 4 | + | 16054625 | 16056956 | 539 | annot_exons |
| novel_circ_001824 | ENSSSCG00000031462 | 6 | + | 12560718 | 12561616 | 899 | antisense |
| novel_circ_001825 | NA | 15 | + | 84269395 | 84269557 | 163 | intergenic |
| novel_circ_001826 | ENSSSCG00000001992 | 7 | + | 75024652 | 75026711 | 655 | annot_exons |
| novel_circ_001827 | ENSSSCG00000010212 | 14 | - | 63449090 | 63450254 | 276 | annot_exons |
| novel_circ_001828 | ENSSSCG00000025703 | 1 | - | 77268674 | 77270404 | 305 | annot_exons |
| novel_circ_001829 | ENSSSCG00000030486 | 13 | - | 1.59E+08 | 1.59E+08 | 399 | annot_exons |
| novel_circ_001830 | ENSSSCG00000012163 | X | - | 16498488 | 16513238 | 467 | annot_exons |
| novel_circ_001831 | ENSSSCG00000029805 | 2 | + | 1.02E+08 | 1.02E+08 | 494 | annot_exons |
| novel_circ_001832 | ENSSSCG00000008961 | 8 | + | 70206185 | 70224123 | 402 | annot_exons |
| novel_circ_001833 | ENSSSCG00000026092 | 5 | - | 6756793 | 6758430 | 614 | annot_exons |
| novel_circ_001834 | ENSSSCG00000010742 | 14 | - | 1.35E+08 | 1.35E+08 | 738 | annot_exons |
| novel_circ_001835 | ENSSSCG00000002759 | 6 | + | 17645723 | 17645980 | 258 | one_exon |
| novel_circ_001836 | ENSSSCG00000010687 | 14 | + | 1.3E+08 | 1.3E+08 | 650 | annot_exons |
| novel_circ_001837 | ENSSSCG00000006137 | 4 | - | 50408310 | 50433791 | 1295 | annot_exons |
| novel_circ_001838 | ENSSSCG00000035934 | 1 | - | 1.82E+08 | 1.82E+08 | 498 | annot_exons |
| novel_circ_001839 | ENSSSCG00000010190 | 14 | - | 60415050 | 60415334 | 285 | antisense |
| novel_circ_001840 | ENSSSCG00000011679 | 13 | - | 83257014 | 83269283 | 1369 | annot_exons |
| novel_circ_001841 | ENSSSCG00000044051 | 11 | - | 76902359 | 76905003 | 286 | annot_exons |
| novel_circ_001842 | ENSSSCG00000015795 | 15 | - | 46520506 | 46527370 | 1251 | exon_intron |
| novel_circ_001843 | ENSSSCG00000006000 | 4 | + | 19233621 | 19256590 | 935 | annot_exons |
| novel_circ_001844 | ENSSSCG00000004538 | 1 | + | 1.06E+08 | 1.06E+08 | 859 | annot_exons |
| novel_circ_001845 | ENSSSCG00000003872 | 6 | + | 1.61E+08 | 1.61E+08 | 378 | annot_exons |
| novel_circ_001846 | ENSSSCG00000011236 | 13 | - | 19324047 | 19331056 | 490 | annot_exons |
| novel_circ_001847 | ENSSSCG00000002743 | 6 | + | 14895772 | 14897526 | 581 | annot_exons |
| novel_circ_001848 | ENSSSCG00000005170 | 1 | - | 2.04E+08 | 2.04E+08 | 1179 | annot_exons |
| novel_circ_001849 | ENSSSCG00000006193 | 4 | + | 64790269 | 64792188 | 481 | annot_exons |
| novel_circ_001850 | ENSSSCG00000001934 | 7 | + | 61121465 | 61126663 | 607 | annot_exons |
| novel_circ_001851 | ENSSSCG00000009276 | 11 | - | 1180609 | 1186009 | 403 | annot_exons |
| novel_circ_001852 | ENSSSCG00000017336 | 12 | - | 18267991 | 18268393 | 403 | one_exon |
| novel_circ_001853 | ENSSSCG00000016975 | 16 | + | 48611821 | 48616188 | 248 | annot_exons |
| novel_circ_001854 | ENSSSCG00000011166 | 10 | - | 69201376 | 69235186 | 734 | annot_exons |
| novel_circ_001855 | ENSSSCG00000009834 | 14 | - | 32721927 | 32734205 | 276 | annot_exons |
| novel_circ_001856 | ENSSSCG00000037561 | 11 | + | 78349882 | 78356690 | 489 | exon_intron |
| novel_circ_001857 | ENSSSCG00000003630 | 6 | + | 92164899 | 92170345 | 442 | annot_exons |
| novel_circ_001858 | ENSSSCG00000000066 | 5 | + | 7267365 | 7271245 | 3881 | antisense |
| novel_circ_001859 | ENSSSCG00000021038 | 13 | + | 1.8E+08 | 1.8E+08 | 580 | antisense |
| novel_circ_001860 | NA | 15 | - | 84231488 | 84231683 | 196 | intergenic |
| novel_circ_001861 | ENSSSCG00000006508 | 4 | + | 94405984 | 94418263 | 275 | annot_exons |
| novel_circ_001862 | ENSSSCG00000004394 | 1 | + | 77080126 | 77087208 | 605 | annot_exons |
| novel_circ_001863 | ENSSSCG00000003693 | 6 | + | 1.04E+08 | 1.04E+08 | 342 | annot_exons |
| novel_circ_001864 | ENSSSCG00000002425 | 7 | - | 1.1E+08 | 1.1E+08 | 314 | annot_exons |
| novel_circ_001865 | ENSSSCG00000029251 | 13 | - | 30691654 | 30744517 | 430 | annot_exons |
| novel_circ_001866 | ENSSSCG00000016019 | 15 | - | 88482354 | 88486742 | 344 | annot_exons |
| novel_circ_001867 | ENSSSCG00000011765 | 13 | + | 1.18E+08 | 1.18E+08 | 550 | annot_exons |
| novel_circ_001868 | ENSSSCG00000007799 | 3 | - | 17938142 | 17940161 | 653 | annot_exons |
| novel_circ_001869 | ENSSSCG00000008444 | 3 | - | 94534268 | 94542205 | 411 | annot_exons |
| novel_circ_001870 | ENSSSCG00000027293 | 16 | + | 49101098 | 49112899 | 565 | annot_exons |
| novel_circ_001871 | ENSSSCG00000023126 | 6 | + | 71217834 | 71218359 | 305 | annot_exons |
| novel_circ_001872 | ENSSSCG00000007311 | 17 | + | 39197685 | 39215276 | 722 | annot_exons |
| novel_circ_001873 | ENSSSCG00000035618 | 14 | - | 22776543 | 22777086 | 349 | annot_exons |
| novel_circ_001874 | ENSSSCG00000018082 | MT | - | 10170 | 10324 | 155 | antisense |
| novel_circ_001875 | ENSSSCG00000025606 | 9 | + | 36676965 | 36685059 | 800 | annot_exons |
| novel_circ_001876 | ENSSSCG00000015602 | 9 | + | 1.31E+08 | 1.31E+08 | 285 | annot_exons |
| novel_circ_001877 | ENSSSCG00000003789 | 6 | - | 1.42E+08 | 1.42E+08 | 328 | annot_exons |
| novel_circ_001878 | ENSSSCG00000033424 | 13 | - | 1.22E+08 | 1.22E+08 | 337 | annot_exons |
| novel_circ_001879 | ENSSSCG00000048832 | 14 | - | 12935722 | 12943614 | 7893 | antisense |
| novel_circ_001880 | ENSSSCG00000006311 | 4 | - | 83873581 | 83873820 | 240 | one_exon |
| novel_circ_001881 | ENSSSCG00000040401 | 17 | - | 62522226 | 62523110 | 477 | annot_exons |
| novel_circ_001882 | ENSSSCG00000010548 | 14 | - | 1.11E+08 | 1.11E+08 | 916 | annot_exons |
| novel_circ_001883 | ENSSSCG00000000853 | 5 | + | 80673591 | 80680632 | 228 | annot_exons |
| novel_circ_001884 | ENSSSCG00000000755 | 5 | + | 68380565 | 68426856 | 429 | annot_exons |
| novel_circ_001885 | ENSSSCG00000025323 | 6 | + | 13564514 | 13566223 | 278 | annot_exons |
| novel_circ_001886 | ENSSSCG00000003775 | 6 | - | 1.37E+08 | 1.37E+08 | 264 | annot_exons |
| novel_circ_001887 | ENSSSCG00000010160 | 14 | + | 56675693 | 56688014 | 604 | annot_exons |
| novel_circ_001888 | ENSSSCG00000007805 | 3 | + | 18563264 | 18563733 | 342 | annot_exons |
| novel_circ_001889 | ENSSSCG00000010863 | 10 | + | 15051106 | 15053287 | 382 | annot_exons |
| novel_circ_001890 | ENSSSCG00000025703 | 1 | - | 77329375 | 77394243 | 59590 | exon_intron |
| novel_circ_001891 | NA | 15 | + | 84253813 | 84254302 | 490 | intergenic |
| novel_circ_001892 | NA | 15 | + | 1.21E+08 | 1.21E+08 | 163 | intergenic |
| novel_circ_001893 | ENSSSCG00000010151 | 14 | + | 55506083 | 55506854 | 294 | annot_exons |
| novel_circ_001894 | ENSSSCG00000003586 | 6 | + | 86013726 | 86022884 | 312 | annot_exons |
| novel_circ_001895 | ENSSSCG00000009528 | 11 | + | 71004041 | 71004248 | 208 | one_exon |
| novel_circ_001896 | ENSSSCG00000017419 | 12 | + | 20739319 | 20748017 | 749 | annot_exons |
| novel_circ_001897 | ENSSSCG00000008079 | 3 | + | 42881922 | 42902838 | 703 | annot_exons |
| novel_circ_001898 | ENSSSCG00000002799 | 6 | - | 20282522 | 20296786 | 2898 | exon_intron |
| novel_circ_001899 | ENSSSCG00000031652 | 6 | + | 31624571 | 31626095 | 234 | annot_exons |
| novel_circ_001900 | ENSSSCG00000009994 | 14 | + | 46957994 | 46996102 | 838 | annot_exons |
| novel_circ_001901 | ENSSSCG00000015558 | 9 | + | 1.25E+08 | 1.25E+08 | 350 | annot_exons |
| novel_circ_001902 | ENSSSCG00000016395 | 15 | - | 802987 | 812599 | 1152 | annot_exons |
| novel_circ_001903 | ENSSSCG00000008808 | 8 | - | 35137332 | 35142685 | 328 | annot_exons |
| novel_circ_001904 | NA | 10 | + | 56437592 | 56440959 | 3368 | intergenic |
| novel_circ_001905 | ENSSSCG00000012884 | 2 | - | 4437791 | 4449449 | 547 | annot_exons |
| novel_circ_001906 | ENSSSCG00000034200 | 13 | - | 26090644 | 26096683 | 529 | annot_exons |
| novel_circ_001907 | ENSSSCG00000017790 | 12 | + | 45731491 | 45733886 | 396 | annot_exons |
| novel_circ_001908 | ENSSSCG00000004934 | 1 | - | 1.63E+08 | 1.63E+08 | 741 | annot_exons |
| novel_circ_001909 | ENSSSCG00000002457 | 7 | - | 1.14E+08 | 1.14E+08 | 191 | annot_exons |
| novel_circ_001910 | ENSSSCG00000003859 | 6 | + | 1.6E+08 | 1.6E+08 | 557 | annot_exons |
| novel_circ_001911 | ENSSSCG00000040918 | 1 | - | 21426530 | 21428157 | 306 | annot_exons |
| novel_circ_001912 | ENSSSCG00000010604 | 14 | - | 1.15E+08 | 1.15E+08 | 330 | annot_exons |
| novel_circ_001913 | ENSSSCG00000014394 | 2 | + | 1.44E+08 | 1.44E+08 | 2402 | annot_exons |
| novel_circ_001914 | ENSSSCG00000031172 | 6 | - | 1.61E+08 | 1.61E+08 | 170 | one_exon |
| novel_circ_001915 | ENSSSCG00000002547 | AEMK02000452.1 | + | 2182611 | 2184607 | 493 | annot_exons |
| novel_circ_001916 | ENSSSCG00000007590 | 3 | + | 5123860 | 5131218 | 635 | annot_exons |
| novel_circ_001917 | ENSSSCG00000017791 | 12 | - | 45881323 | 45913679 | 25856 | exon_intron |
| novel_circ_001918 | ENSSSCG00000002001 | 7 | - | 75135635 | 75136922 | 414 | annot_exons |
| novel_circ_001919 | ENSSSCG00000010144 | 14 | - | 54696668 | 54700162 | 410 | annot_exons |
| novel_circ_001920 | ENSSSCG00000006840 | 4 | + | 1.11E+08 | 1.11E+08 | 618 | annot_exons |
| novel_circ_001921 | ENSSSCG00000014319 | 2 | + | 1.38E+08 | 1.38E+08 | 372 | annot_exons |
| novel_circ_001922 | ENSSSCG00000004943 | 1 | + | 1.64E+08 | 1.64E+08 | 358 | annot_exons |
| novel_circ_001923 | ENSSSCG00000022190 | 4 | - | 79631495 | 79637166 | 275 | annot_exons |
| novel_circ_001924 | ENSSSCG00000042985 | 6 | + | 47420963 | 47442978 | 16203 | exon_intron |
| novel_circ_001925 | NA | 15 | + | 84290583 | 84290779 | 197 | intergenic |
| novel_circ_001926 | ENSSSCG00000001878 | 7 | + | 58137959 | 58140971 | 440 | annot_exons |
| novel_circ_001927 | ENSSSCG00000012329 | X | - | 46725946 | 46726525 | 195 | annot_exons |
| novel_circ_001928 | ENSSSCG00000014838 | 9 | - | 8708444 | 8716340 | 468 | annot_exons |
| novel_circ_001929 | ENSSSCG00000009125 | 8 | - | 1.09E+08 | 1.09E+08 | 495 | annot_exons |
| novel_circ_001930 | ENSSSCG00000008534 | 3 | - | 1.1E+08 | 1.1E+08 | 567 | annot_exons |
| novel_circ_001931 | ENSSSCG00000033692 | 14 | + | 55770332 | 55815276 | 1415 | annot_exons |
| novel_circ_001932 | ENSSSCG00000009971 | 14 | + | 46180499 | 46181839 | 1341 | exon_intron |
| novel_circ_001933 | ENSSSCG00000004701 | 1 | + | 1.28E+08 | 1.28E+08 | 661 | annot_exons |
| novel_circ_001934 | ENSSSCG00000030291 | 4 | - | 98655267 | 98695000 | 1346 | annot_exons |
| novel_circ_001935 | ENSSSCG00000003776 | 6 | - | 1.38E+08 | 1.38E+08 | 237 | annot_exons |
| novel_circ_001936 | ENSSSCG00000004845 | 1 | - | 1.44E+08 | 1.44E+08 | 681 | intronic |
| novel_circ_001937 | ENSSSCG00000047432 | 2 | - | 6750520 | 6751696 | 1177 | antisense |
| novel_circ_001938 | ENSSSCG00000008404 | 3 | + | 85967534 | 85971244 | 478 | annot_exons |
| novel_circ_001939 | ENSSSCG00000024592 | 8 | - | 69443629 | 69451424 | 311 | annot_exons |
| novel_circ_001940 | ENSSSCG00000002755 | 6 | - | 17320031 | 17338519 | 551 | annot_exons |
| novel_circ_001941 | ENSSSCG00000008803 | 8 | - | 33445146 | 33445534 | 295 | annot_exons |
| novel_circ_001942 | ENSSSCG00000024623 | 13 | + | 1.81E+08 | 1.81E+08 | 1714 | annot_exons |
| novel_circ_001943 | ENSSSCG00000031775 | 5 | - | 73463796 | 73481454 | 17566 | exon_intron |
| novel_circ_001944 | ENSSSCG00000009657 | 14 | + | 10274690 | 10275948 | 364 | exon_intron |
| novel_circ_001945 | ENSSSCG00000017401 | 12 | - | 20307501 | 20332068 | 4369 | annot_exons |
| novel_circ_001946 | ENSSSCG00000013566 | 2 | + | 71919789 | 71922125 | 263 | annot_exons |
| novel_circ_001947 | ENSSSCG00000030480 | 13 | + | 2.01E+08 | 2.01E+08 | 262 | annot_exons |
| novel_circ_001948 | ENSSSCG00000017593 | 12 | + | 27540339 | 27548437 | 617 | annot_exons |
| novel_circ_001949 | ENSSSCG00000037305 | 8 | + | 33016128 | 33023054 | 689 | exon_intron |
| novel_circ_001950 | NA | 15 | + | 71259536 | 71263414 | 3879 | intergenic |
| novel_circ_001951 | ENSSSCG00000030528 | 13 | - | 1.92E+08 | 1.92E+08 | 313 | annot_exons |
| novel_circ_001952 | ENSSSCG00000024881 | 5 | - | 13958213 | 13970227 | 615 | annot_exons |
| novel_circ_001953 | ENSSSCG00000017165 | 12 | + | 3320813 | 3321727 | 214 | annot_exons |
| novel_circ_001954 | ENSSSCG00000016981 | 16 | + | 50426818 | 50429298 | 2481 | antisense |
| novel_circ_001955 | ENSSSCG00000027139 | 13 | + | 1.34E+08 | 1.34E+08 | 278 | annot_exons |
| novel_circ_001956 | ENSSSCG00000022128 | 14 | - | 1.21E+08 | 1.21E+08 | 228 | antisense |
| novel_circ_001957 | ENSSSCG00000000078 | 5 | - | 8104155 | 8109884 | 443 | annot_exons |
| novel_circ_001958 | ENSSSCG00000011760 | 13 | - | 1.15E+08 | 1.15E+08 | 192 | annot_exons |
| novel_circ_001959 | ENSSSCG00000031957 | 4 | - | 33087513 | 33153512 | 66000 | intronic |
| novel_circ_001960 | ENSSSCG00000015777 | 15 | + | 44992726 | 44996405 | 466 | annot_exons |
| novel_circ_001961 | ENSSSCG00000000167 | 5 | - | 13544236 | 13544844 | 609 | one_exon |
| novel_circ_001962 | ENSSSCG00000001499 | 7 | + | 29238238 | 29240758 | 447 | annot_exons |
| novel_circ_001963 | ENSSSCG00000035133 | 14 | + | 14490490 | 14492611 | 363 | annot_exons |
| novel_circ_001964 | ENSSSCG00000008624 | 3 | - | 1.25E+08 | 1.25E+08 | 4082 | annot_exons |
| novel_circ_001965 | ENSSSCG00000024453 | 13 | - | 68582517 | 68611602 | 1889 | annot_exons |
| novel_circ_001966 | ENSSSCG00000038561 | 15 | + | 84399027 | 84399291 | 265 | antisense |
| novel_circ_001967 | ENSSSCG00000011632 | 13 | - | 73861304 | 73863238 | 367 | annot_exons |
| novel_circ_001968 | ENSSSCG00000017877 | 12 | - | 50270123 | 50305019 | 22777 | exon_intron |
| novel_circ_001969 | ENSSSCG00000003204 | 6 | - | 54988309 | 55000884 | 803 | annot_exons |
| novel_circ_001970 | ENSSSCG00000010692 | 14 | + | 1.31E+08 | 1.31E+08 | 1201 | annot_exons |
| novel_circ_001971 | ENSSSCG00000013864 | 2 | - | 60933881 | 60938053 | 430 | annot_exons |
| novel_circ_001972 | ENSSSCG00000010460 | 14 | + | 1.03E+08 | 1.03E+08 | 550 | annot_exons |
| novel_circ_001973 | NA | 15 | + | 84298466 | 84298848 | 383 | intergenic |
| novel_circ_001974 | ENSSSCG00000010190 | 14 | - | 60414513 | 60415651 | 1139 | antisense |
| novel_circ_001975 | ENSSSCG00000034786 | 13 | + | 1.37E+08 | 1.37E+08 | 301 | annot_exons |
| novel_circ_001976 | ENSSSCG00000038329 | 6 | + | 1.36E+08 | 1.36E+08 | 735 | exon_intron |
| novel_circ_001977 | ENSSSCG00000004371 | 1 | + | 72712127 | 72717959 | 473 | annot_exons |
| novel_circ_001978 | ENSSSCG00000016850 | 16 | - | 23740869 | 23746765 | 3226 | annot_exons |
| novel_circ_001979 | ENSSSCG00000024201 | 13 | - | 38526031 | 38531676 | 308 | annot_exons |
| novel_circ_001980 | ENSSSCG00000024814 | X | - | 47795844 | 47826089 | 1001 | annot_exons |
| novel_circ_001981 | ENSSSCG00000004552 | 1 | - | 1.08E+08 | 1.08E+08 | 304 | annot_exons |
| novel_circ_001982 | ENSSSCG00000010628 | 14 | + | 1.21E+08 | 1.21E+08 | 443 | annot_exons |
| novel_circ_001983 | ENSSSCG00000017063 | 16 | - | 66813769 | 66821274 | 7506 | intronic |
| novel_circ_001984 | ENSSSCG00000037854 | 1 | - | 1.89E+08 | 1.89E+08 | 431 | annot_exons |
| novel_circ_001985 | ENSSSCG00000033185 | 13 | + | 77068767 | 77075075 | 296 | annot_exons |
| novel_circ_001986 | ENSSSCG00000030049 | 6 | + | 49302159 | 49302505 | 252 | annot_exons |
| novel_circ_001987 | ENSSSCG00000027257 | 1 | + | 27360 | 38757 | 427 | annot_exons |
| novel_circ_001988 | ENSSSCG00000012262 | X | + | 40128818 | 40132819 | 445 | annot_exons |
| novel_circ_001989 | NA | 15 | + | 84262896 | 84263105 | 210 | intergenic |
| novel_circ_001990 | ENSSSCG00000011803 | 13 | + | 1.25E+08 | 1.25E+08 | 228 | annot_exons |
| novel_circ_001991 | ENSSSCG00000014838 | 9 | - | 8637987 | 8646907 | 566 | annot_exons |
| novel_circ_001992 | ENSSSCG00000017255 | 12 | + | 11098684 | 11125448 | 2457 | annot_exons |
| novel_circ_001993 | ENSSSCG00000004138 | 1 | + | 22268062 | 22352249 | 13798 | exon_intron |
| novel_circ_001994 | ENSSSCG00000030343 | 16 | - | 34798441 | 34839397 | 1103 | annot_exons |
| novel_circ_001995 | ENSSSCG00000034156 | 11 | - | 22108700 | 22128793 | 8126 | exon_intron |
| novel_circ_001996 | ENSSSCG00000016487 | 18 | - | 7804595 | 7806657 | 434 | annot_exons |
| novel_circ_001997 | ENSSSCG00000009885 | 14 | + | 39492808 | 39497251 | 402 | annot_exons |
| novel_circ_001998 | ENSSSCG00000039701 | 7 | - | 92715574 | 92717520 | 388 | annot_exons |
| novel_circ_001999 | NA | 15 | - | 84269480 | 84269665 | 186 | intergenic |
| novel_circ_002000 | ENSSSCG00000029578 | 6 | + | 28766824 | 28767831 | 1008 | one_exon |
| novel_circ_002001 | ENSSSCG00000016866 | 16 | + | 27390021 | 27392591 | 352 | annot_exons |
| novel_circ_002002 | ENSSSCG00000028475 | 13 | + | 1.38E+08 | 1.38E+08 | 3971 | exon_intron |
| novel_circ_002003 | ENSSSCG00000013262 | 2 | + | 15967840 | 15974446 | 2191 | annot_exons |
| novel_circ_002004 | ENSSSCG00000005994 | 4 | + | 18444098 | 18444305 | 208 | one_exon |
| novel_circ_002005 | ENSSSCG00000003586 | 6 | + | 85922219 | 85922696 | 478 | one_exon |
| novel_circ_002006 | ENSSSCG00000000458 | 5 | + | 27173898 | 27175753 | 416 | annot_exons |
| novel_circ_002007 | ENSSSCG00000009029 | 8 | - | 80682788 | 80706483 | 405 | annot_exons |
| novel_circ_002008 | ENSSSCG00000025942 | 13 | - | 1.21E+08 | 1.21E+08 | 517 | annot_exons |
| novel_circ_002009 | ENSSSCG00000026978 | 1 | + | 44514881 | 44524153 | 601 | annot_exons |
| novel_circ_002010 | NA | 15 | - | 84314004 | 84314689 | 686 | intergenic |
| novel_circ_002011 | ENSSSCG00000001646 | 7 | + | 37853255 | 37904204 | 2026 | annot_exons |
| novel_circ_002012 | ENSSSCG00000039064 | 12 | + | 51534437 | 51541194 | 780 | annot_exons |
| novel_circ_002013 | ENSSSCG00000009276 | 11 | - | 1141224 | 1141776 | 333 | annot_exons |
| novel_circ_002014 | ENSSSCG00000004725 | 1 | + | 1.29E+08 | 1.29E+08 | 352 | one_exon |
| novel_circ_002015 | ENSSSCG00000004022 | 1 | + | 2486445 | 2498776 | 481 | annot_exons |
| novel_circ_002016 | ENSSSCG00000012251 | X | + | 36834742 | 36835577 | 308 | annot_exons |
| novel_circ_002017 | ENSSSCG00000021644 | 6 | + | 34513016 | 34515277 | 248 | annot_exons |
| novel_circ_002018 | ENSSSCG00000010029 | 14 | + | 48129173 | 48137265 | 416 | annot_exons |
| novel_circ_002019 | ENSSSCG00000008581 | 3 | - | 1.14E+08 | 1.14E+08 | 430 | annot_exons |
| novel_circ_002020 | ENSSSCG00000005636 | 1 | + | 2.69E+08 | 2.69E+08 | 363 | annot_exons |
| novel_circ_002021 | ENSSSCG00000000555 | 5 | + | 47308479 | 47319185 | 531 | annot_exons |
| novel_circ_002022 | ENSSSCG00000004415 | 1 | + | 75279281 | 75280655 | 349 | annot_exons |
| novel_circ_002023 | ENSSSCG00000035521 | 4 | - | 15793121 | 15793974 | 854 | antisense |
| novel_circ_002024 | NA | 15 | - | 84456200 | 84458469 | 2270 | intergenic |
| novel_circ_002025 | NA | 15 | - | 84290336 | 84294301 | 3966 | intergenic |
| novel_circ_002026 | ENSSSCG00000010816 | 10 | + | 8361319 | 8386109 | 740 | annot_exons |
| novel_circ_002027 | ENSSSCG00000006137 | 4 | - | 50447701 | 50486463 | 2407 | annot_exons |
| novel_circ_002028 | ENSSSCG00000033228 | 12 | - | 27298750 | 27329947 | 17671 | exon_intron |
| novel_circ_002029 | ENSSSCG00000008826 | 8 | - | 38531170 | 38535871 | 423 | annot_exons |
| novel_circ_002030 | ENSSSCG00000017608 | 12 | + | 32060088 | 32062771 | 276 | exon_intron |
| novel_circ_002031 | ENSSSCG00000033823 | 5 | - | 7549899 | 7569533 | 905 | annot_exons |
| novel_circ_002032 | ENSSSCG00000006771 | 4 | + | 1.08E+08 | 1.08E+08 | 405 | annot_exons |
| novel_circ_002033 | ENSSSCG00000007799 | 3 | - | 17939446 | 17940181 | 361 | annot_exons |
| novel_circ_002034 | ENSSSCG00000009652 | 14 | - | 9498315 | 9505715 | 263 | annot_exons |
| novel_circ_002035 | ENSSSCG00000008575 | 3 | + | 1.13E+08 | 1.13E+08 | 752 | annot_exons |
| novel_circ_002036 | ENSSSCG00000004626 | 1 | - | 1.2E+08 | 1.2E+08 | 357 | annot_exons |
| novel_circ_002037 | ENSSSCG00000002378 | 7 | - | 98227381 | 98230257 | 344 | annot_exons |
| novel_circ_002038 | ENSSSCG00000010688 | 14 | - | 1.3E+08 | 1.3E+08 | 886 | annot_exons |
| novel_circ_002039 | ENSSSCG00000002330 | 7 | + | 94527247 | 94533253 | 315 | annot_exons |
| novel_circ_002040 | ENSSSCG00000008105 | 3 | + | 44526875 | 44534535 | 616 | annot_exons |
| novel_circ_002041 | ENSSSCG00000004983 | 1 | + | 1.7E+08 | 1.7E+08 | 509 | annot_exons |
| novel_circ_002042 | ENSSSCG00000021739 | 2 | - | 18496995 | 18500836 | 3066 | exon_intron |
| novel_circ_002043 | ENSSSCG00000003673 | 6 | - | 96667496 | 96668320 | 275 | annot_exons |
| novel_circ_002044 | ENSSSCG00000017065 | 16 | - | 68241326 | 68250610 | 350 | annot_exons |
| novel_circ_002045 | ENSSSCG00000003218 | 6 | + | 55304022 | 55304287 | 266 | exon_intron |
| novel_circ_002046 | ENSSSCG00000016661 | 18 | - | 38110775 | 38132080 | 461 | annot_exons |
| novel_circ_002047 | ENSSSCG00000042162 | 2 | + | 41757562 | 41757888 | 327 | one_exon |
| novel_circ_002048 | ENSSSCG00000006194 | 4 | + | 65214828 | 65219019 | 343 | annot_exons |
| novel_circ_002049 | ENSSSCG00000004276 | 1 | + | 50832070 | 50857783 | 462 | annot_exons |
| novel_circ_002050 | ENSSSCG00000011199 | 13 | - | 4253500 | 4253663 | 164 | one_exon |
| novel_circ_002051 | ENSSSCG00000007454 | 17 | - | 49320124 | 49330179 | 431 | annot_exons |
| novel_circ_002052 | ENSSSCG00000015107 | 9 | + | 46287650 | 46288627 | 412 | annot_exons |
| novel_circ_002053 | ENSSSCG00000038178 | AEMK02000697.1 | - | 40353 | 62635 | 4104 | exon_intron |
| novel_circ_002054 | ENSSSCG00000007805 | 3 | + | 18559896 | 18560385 | 235 | annot_exons |
| novel_circ_002055 | ENSSSCG00000004018 | 1 | - | 1657903 | 1663793 | 476 | annot_exons |
| novel_circ_002056 | ENSSSCG00000020878 | 3 | + | 1.02E+08 | 1.02E+08 | 357 | annot_exons |
| novel_circ_002057 | ENSSSCG00000030326 | 2 | - | 27879731 | 27887282 | 612 | annot_exons |
| novel_circ_002058 | ENSSSCG00000003514 | 6 | - | 79875804 | 79876991 | 579 | annot_exons |
| novel_circ_002059 | ENSSSCG00000009569 | 11 | - | 477965 | 482662 | 4698 | exon_intron |
| novel_circ_002060 | ENSSSCG00000029464 | 5 | - | 67690123 | 67699579 | 333 | annot_exons |
| novel_circ_002061 | ENSSSCG00000007092 | 17 | + | 26515291 | 26517967 | 513 | annot_exons |
| novel_circ_002062 | ENSSSCG00000007022 | 17 | - | 10792387 | 10794759 | 364 | annot_exons |
| novel_circ_002063 | ENSSSCG00000005170 | 1 | - | 2.04E+08 | 2.04E+08 | 700 | annot_exons |
| novel_circ_002064 | ENSSSCG00000029251 | 13 | - | 30685269 | 30744517 | 544 | annot_exons |
| novel_circ_002065 | ENSSSCG00000011068 | 10 | - | 48735609 | 48739057 | 453 | annot_exons |
| novel_circ_002066 | ENSSSCG00000034730 | 1 | - | 96447977 | 96453655 | 353 | annot_exons |
| novel_circ_002067 | ENSSSCG00000002660 | 6 | + | 2573743 | 2574166 | 239 | annot_exons |
| novel_circ_002068 | ENSSSCG00000008820 | 8 | - | 38066081 | 38070725 | 269 | annot_exons |
| novel_circ_002069 | ENSSSCG00000015709 | 15 | - | 19949605 | 19961711 | 613 | annot_exons |
| novel_circ_002070 | ENSSSCG00000029485 | 3 | + | 92761995 | 92764582 | 488 | annot_exons |
| novel_circ_002071 | ENSSSCG00000028228 | 3 | + | 80265089 | 80268798 | 2653 | annot_exons |
| novel_circ_002072 | ENSSSCG00000002330 | 7 | + | 94500165 | 94515912 | 1949 | annot_exons |
| novel_circ_002073 | ENSSSCG00000017672 | 12 | + | 36384264 | 36385530 | 296 | annot_exons |
| novel_circ_002074 | ENSSSCG00000035671 | 12 | - | 61279324 | 61286652 | 926 | annot_exons |
| novel_circ_002075 | ENSSSCG00000006824 | 4 | - | 1.11E+08 | 1.11E+08 | 232 | one_exon |
| novel_circ_002076 | ENSSSCG00000029602 | 10 | - | 68509412 | 68518239 | 436 | annot_exons |
| novel_circ_002077 | ENSSSCG00000034378 | 13 | + | 1.97E+08 | 1.97E+08 | 318 | annot_exons |
| novel_circ_002078 | ENSSSCG00000009551 | 11 | + | 77498616 | 77499037 | 191 | annot_exons |
| novel_circ_002079 | NA | 15 | - | 84252941 | 84254775 | 1835 | intergenic |
| novel_circ_002080 | ENSSSCG00000024232 | 5 | - | 45400040 | 45403216 | 1519 | exon_intron |
| novel_circ_002081 | ENSSSCG00000004351 | 1 | - | 66401091 | 66412736 | 463 | annot_exons |
| novel_circ_002082 | ENSSSCG00000033591 | 6 | - | 31859873 | 31866445 | 321 | annot_exons |
| novel_circ_002083 | ENSSSCG00000011593 | 13 | - | 69223492 | 69228869 | 1266 | annot_exons |
| novel_circ_002084 | ENSSSCG00000002386 | 7 | + | 99342538 | 99363274 | 547 | annot_exons |
| novel_circ_002085 | ENSSSCG00000015427 | 9 | - | 1.04E+08 | 1.04E+08 | 327 | annot_exons |
| novel_circ_002086 | ENSSSCG00000037416 | 7 | - | 40727030 | 40742927 | 343 | annot_exons |
| novel_circ_002087 | ENSSSCG00000017506 | 12 | + | 22952938 | 22957391 | 206 | annot_exons |
| novel_circ_002088 | ENSSSCG00000011373 | 13 | - | 31682729 | 31683772 | 1044 | one_exon |
| novel_circ_002089 | ENSSSCG00000011642 | 13 | - | 75346690 | 75362989 | 411 | annot_exons |
| novel_circ_002090 | ENSSSCG00000018049 | 12 | - | 60366858 | 60376568 | 559 | annot_exons |
| novel_circ_002091 | ENSSSCG00000002277 | 7 | - | 88852496 | 88853252 | 757 | one_exon |
| novel_circ_002092 | ENSSSCG00000004402 | 1 | - | 76222929 | 76223610 | 682 | intronic |
| novel_circ_002093 | ENSSSCG00000003093 | 6 | - | 52157014 | 52157218 | 205 | one_exon |
| novel_circ_002094 | ENSSSCG00000011915 | 13 | - | 1.46E+08 | 1.46E+08 | 708 | annot_exons |
| novel_circ_002095 | ENSSSCG00000012328 | X | - | 46401350 | 46404171 | 360 | annot_exons |
| novel_circ_002096 | ENSSSCG00000009338 | 11 | + | 8441214 | 8455939 | 14726 | intronic |
| novel_circ_002097 | ENSSSCG00000020710 | 6 | + | 73204987 | 73206401 | 1415 | one_exon |
| novel_circ_002098 | ENSSSCG00000024598 | 16 | - | 25751226 | 25751950 | 614 | annot_exons |
| novel_circ_002099 | ENSSSCG00000010702 | 14 | + | 1.32E+08 | 1.32E+08 | 224 | annot_exons |
| novel_circ_002100 | ENSSSCG00000006508 | 4 | + | 94324418 | 94418263 | 6186 | exon_intron |
| novel_circ_002101 | ENSSSCG00000001497 | 7 | - | 28723460 | 28735707 | 547 | annot_exons |
| novel_circ_002102 | ENSSSCG00000028406 | 1 | - | 2.02E+08 | 2.02E+08 | 465 | annot_exons |
| novel_circ_002103 | ENSSSCG00000004814 | 1 | + | 1.38E+08 | 1.38E+08 | 415 | annot_exons |
| novel_circ_002104 | ENSSSCG00000006303 | 4 | - | 82951030 | 82958350 | 634 | annot_exons |
| novel_circ_002105 | ENSSSCG00000013022 | 2 | - | 7417844 | 7418565 | 722 | antisense |
| novel_circ_002106 | ENSSSCG00000031608 | 17 | - | 10759756 | 10761499 | 584 | exon_intron |
| novel_circ_002107 | ENSSSCG00000030413 | 13 | + | 1.17E+08 | 1.17E+08 | 1135 | annot_exons |
| novel_circ_002108 | ENSSSCG00000006754 | 4 | + | 1.06E+08 | 1.06E+08 | 412 | annot_exons |
| novel_circ_002109 | ENSSSCG00000010190 | 14 | - | 60414282 | 60414916 | 635 | antisense |
| novel_circ_002110 | ENSSSCG00000024864 | 5 | - | 3767999 | 3774893 | 275 | annot_exons |
| novel_circ_002111 | ENSSSCG00000006303 | 4 | - | 82951030 | 82951645 | 400 | annot_exons |
| novel_circ_002112 | ENSSSCG00000016291 | 15 | + | 1.33E+08 | 1.33E+08 | 594 | annot_exons |
| novel_circ_002113 | ENSSSCG00000014893 | 9 | - | 12903427 | 12905549 | 205 | annot_exons |
| novel_circ_002114 | ENSSSCG00000014324 | 2 | + | 1.4E+08 | 1.4E+08 | 183 | annot_exons |
| novel_circ_002115 | ENSSSCG00000008079 | 3 | + | 42929643 | 42946710 | 741 | annot_exons |
| novel_circ_002116 | ENSSSCG00000023548 | 8 | - | 1.16E+08 | 1.16E+08 | 720 | annot_exons |
| novel_circ_002117 | ENSSSCG00000004626 | 1 | - | 1.2E+08 | 1.2E+08 | 2147 | annot_exons |
| novel_circ_002118 | ENSSSCG00000003627 | 6 | + | 91559499 | 91561270 | 691 | annot_exons |
| novel_circ_002119 | ENSSSCG00000033692 | 14 | + | 55777475 | 55799718 | 502 | annot_exons |
| novel_circ_002120 | ENSSSCG00000009298 | 11 | + | 4122184 | 4143040 | 18491 | exon_intron |
| novel_circ_002121 | ENSSSCG00000017877 | 12 | - | 50266696 | 50273912 | 400 | annot_exons |
| novel_circ_002122 | ENSSSCG00000021118 | 3 | + | 31265467 | 31266844 | 433 | annot_exons |
| novel_circ_002123 | ENSSSCG00000029029 | 3 | - | 16964040 | 16964840 | 220 | annot_exons |
| novel_circ_002124 | ENSSSCG00000028293 | 9 | - | 1.32E+08 | 1.32E+08 | 355 | annot_exons |
| novel_circ_002125 | ENSSSCG00000030067 | 1 | - | 16368499 | 16374414 | 600 | annot_exons |
| novel_circ_002126 | ENSSSCG00000010467 | 14 | - | 1.04E+08 | 1.04E+08 | 256 | antisense |
| novel_circ_002127 | ENSSSCG00000037499 | 1 | + | 1.16E+08 | 1.16E+08 | 578 | exon_intron |
| novel_circ_002128 | ENSSSCG00000010987 | 10 | + | 32800584 | 32819154 | 329 | annot_exons |
| novel_circ_002129 | ENSSSCG00000030118 | 14 | - | 16632802 | 16661066 | 28265 | exon_intron |
| novel_circ_002130 | ENSSSCG00000017593 | 12 | + | 27540339 | 27540737 | 301 | annot_exons |
| novel_circ_002131 | ENSSSCG00000011720 | 13 | + | 92579336 | 92580621 | 1286 | intronic |
| novel_circ_002132 | NA | 15 | - | 84428309 | 84428499 | 191 | intergenic |
| novel_circ_002133 | ENSSSCG00000008600 | 3 | + | 1.18E+08 | 1.18E+08 | 1105 | annot_exons |
| novel_circ_002134 | ENSSSCG00000010738 | 14 | + | 1.34E+08 | 1.34E+08 | 851 | one_exon |
| novel_circ_002135 | ENSSSCG00000004364 | 1 | - | 71053397 | 71055262 | 306 | annot_exons |
| novel_circ_002136 | ENSSSCG00000014195 | 2 | + | 1.15E+08 | 1.15E+08 | 276 | annot_exons |
| novel_circ_002137 | ENSSSCG00000010360 | 14 | + | 87822731 | 87862622 | 607 | annot_exons |
| novel_circ_002138 | ENSSSCG00000033148 | 12 | - | 1108963 | 1109268 | 306 | intronic |
| novel_circ_002139 | ENSSSCG00000011603 | 13 | - | 70406477 | 70408175 | 380 | annot_exons |
| novel_circ_002140 | ENSSSCG00000031775 | 5 | - | 73463796 | 73474253 | 10365 | exon_intron |
| novel_circ_002141 | ENSSSCG00000000853 | 5 | + | 80658988 | 80659577 | 131 | annot_exons |
| novel_circ_002142 | ENSSSCG00000014917 | 9 | + | 20216032 | 20222611 | 271 | annot_exons |
| novel_circ_002143 | ENSSSCG00000004082 | 1 | + | 14108477 | 14167020 | 6337 | annot_exons |
| novel_circ_002144 | ENSSSCG00000031639 | 6 | + | 87911284 | 87926357 | 440 | annot_exons |
| novel_circ_002145 | ENSSSCG00000017260 | 12 | + | 11721129 | 11728401 | 350 | annot_exons |
| novel_circ_002146 | ENSSSCG00000002690 | 6 | - | 6946245 | 6958719 | 1335 | annot_exons |
| novel_circ_002147 | ENSSSCG00000011394 | 13 | + | 32557718 | 32560025 | 354 | annot_exons |
| novel_circ_002148 | ENSSSCG00000016017 | 15 | + | 88244695 | 88252360 | 268 | annot_exons |
| novel_circ_002149 | NA | 14 | - | 41643306 | 41672257 | 28952 | intergenic |
| novel_circ_002150 | ENSSSCG00000005751 | 1 | - | 2.74E+08 | 2.74E+08 | 508 | antisense |
| novel_circ_002151 | ENSSSCG00000042430 | 15 | + | 84423891 | 84424210 | 320 | antisense |
| novel_circ_002152 | ENSSSCG00000005975 | 4 | + | 15084665 | 15088270 | 364 | annot_exons |
| novel_circ_002153 | ENSSSCG00000010210 | 14 | - | 63049348 | 63070159 | 376 | annot_exons |
| novel_circ_002154 | ENSSSCG00000005969 | 4 | + | 14650499 | 14659096 | 802 | annot_exons |
| novel_circ_002155 | ENSSSCG00000041513 | 1 | + | 2.42E+08 | 2.42E+08 | 94206 | exon_intron |
| novel_circ_002156 | ENSSSCG00000032556 | 3 | + | 18259593 | 18259756 | 164 | one_exon |
| novel_circ_002157 | ENSSSCG00000001918 | 7 | + | 59809999 | 59837616 | 3198 | annot_exons |
| novel_circ_002158 | ENSSSCG00000004070 | 1 | - | 11887417 | 11888352 | 936 | intronic |
| novel_circ_002159 | ENSSSCG00000014114 | 2 | + | 88334351 | 88338133 | 502 | annot_exons |
| novel_circ_002160 | ENSSSCG00000007799 | 3 | - | 17940227 | 17940974 | 748 | exon_intron |
| novel_circ_002161 | ENSSSCG00000015818 | 15 | - | 48271060 | 48273310 | 593 | annot_exons |
| novel_circ_002162 | ENSSSCG00000006981 | 17 | + | 4802543 | 4823695 | 705 | annot_exons |
| novel_circ_002163 | ENSSSCG00000017668 | 12 | + | 35952997 | 35960199 | 440 | annot_exons |
| novel_circ_002164 | ENSSSCG00000011788 | 13 | + | 1.23E+08 | 1.23E+08 | 535 | exon_intron |
| novel_circ_002165 | ENSSSCG00000031636 | 8 | + | 40180318 | 40215963 | 607 | annot_exons |
| novel_circ_002166 | NA | 15 | + | 84267063 | 84267260 | 198 | intergenic |
| novel_circ_002167 | ENSSSCG00000004717 | 1 | + | 1.28E+08 | 1.28E+08 | 258 | annot_exons |
| novel_circ_002168 | ENSSSCG00000023334 | X | - | 16263068 | 16268724 | 370 | annot_exons |
| novel_circ_002169 | ENSSSCG00000010243 | 14 | + | 71852747 | 71857332 | 600 | annot_exons |
| novel_circ_002170 | ENSSSCG00000021354 | 18 | + | 13893889 | 13894154 | 266 | one_exon |
| novel_circ_002171 | ENSSSCG00000022559 | 6 | - | 1.06E+08 | 1.06E+08 | 288 | annot_exons |
| novel_circ_002172 | ENSSSCG00000009844 | 14 | + | 33626558 | 33627055 | 498 | antisense |
| novel_circ_002173 | ENSSSCG00000003828 | 6 | - | 1.53E+08 | 1.53E+08 | 698 | annot_exons |
| novel_circ_002174 | ENSSSCG00000009480 | 11 | + | 51511828 | 51531654 | 19827 | exon_intron |
| novel_circ_002175 | ENSSSCG00000010246 | 14 | + | 72021326 | 72030857 | 898 | annot_exons |
| novel_circ_002176 | ENSSSCG00000026996 | 11 | - | 64260970 | 64261366 | 397 | exon_intron |
| novel_circ_002177 | ENSSSCG00000009142 | 8 | - | 1.13E+08 | 1.13E+08 | 1264 | annot_exons |
| novel_circ_002178 | ENSSSCG00000015940 | 15 | + | 76299506 | 76301485 | 338 | annot_exons |
| novel_circ_002179 | ENSSSCG00000010306 | 14 | + | 76475822 | 76477894 | 336 | annot_exons |
| novel_circ_002180 | ENSSSCG00000003917 | 6 | + | 1.66E+08 | 1.66E+08 | 486 | annot_exons |
| novel_circ_002181 | ENSSSCG00000017513 | 12 | + | 23857979 | 23859799 | 331 | annot_exons |
| novel_circ_002182 | ENSSSCG00000006101 | 4 | + | 42344803 | 42361848 | 994 | annot_exons |
| novel_circ_002183 | ENSSSCG00000035133 | 14 | + | 14486402 | 14492611 | 743 | annot_exons |
| novel_circ_002184 | NA | 3 | + | 7960804 | 7961264 | 461 | intergenic |
| novel_circ_002185 | ENSSSCG00000014304 | 2 | + | 1.37E+08 | 1.37E+08 | 787 | annot_exons |
| novel_circ_002186 | ENSSSCG00000032804 | 5 | - | 60035320 | 60069981 | 34662 | exon_intron |
| novel_circ_002187 | ENSSSCG00000022963 | 3 | + | 40446251 | 40457300 | 521 | annot_exons |
| novel_circ_002188 | ENSSSCG00000017593 | 12 | + | 27531355 | 27538247 | 256 | annot_exons |
| novel_circ_002189 | ENSSSCG00000033323 | 14 | + | 92297840 | 92298036 | 197 | antisense |
| novel_circ_002190 | ENSSSCG00000017311 | 12 | - | 17146073 | 17173710 | 23174 | exon_intron |
| novel_circ_002191 | ENSSSCG00000000078 | 5 | - | 8073497 | 8078016 | 558 | annot_exons |
| novel_circ_002192 | ENSSSCG00000014304 | 2 | + | 1.37E+08 | 1.37E+08 | 564 | annot_exons |
| novel_circ_002193 | NA | 15 | - | 84463740 | 84463946 | 207 | intergenic |
| novel_circ_002194 | ENSSSCG00000016845 | 16 | - | 22534285 | 22535679 | 414 | annot_exons |
| novel_circ_002195 | ENSSSCG00000023112 | 8 | - | 31030063 | 31031646 | 444 | annot_exons |
| novel_circ_002196 | ENSSSCG00000000214 | 5 | - | 16013908 | 16014268 | 361 | antisense |
| novel_circ_002197 | ENSSSCG00000028558 | 1 | - | 1.94E+08 | 1.94E+08 | 403 | antisense |
| novel_circ_002198 | ENSSSCG00000006857 | 4 | + | 1.16E+08 | 1.16E+08 | 379 | annot_exons |
| novel_circ_002199 | ENSSSCG00000000753 | 5 | + | 68055984 | 68075752 | 480 | annot_exons |
| novel_circ_002200 | ENSSSCG00000003700 | 6 | - | 1.07E+08 | 1.07E+08 | 657 | annot_exons |
| novel_circ_002201 | ENSSSCG00000016578 | 18 | + | 19768297 | 19768781 | 485 | antisense |
| novel_circ_002202 | ENSSSCG00000039368 | 10 | + | 25760691 | 25773920 | 405 | annot_exons |
| novel_circ_002203 | ENSSSCG00000017783 | 12 | - | 45375951 | 45377258 | 310 | annot_exons |
| novel_circ_002204 | ENSSSCG00000007592 | 3 | + | 5089147 | 5090490 | 181 | annot_exons |
| novel_circ_002205 | ENSSSCG00000030291 | 4 | - | 98669435 | 98670214 | 780 | exon_intron |
| novel_circ_002206 | ENSSSCG00000022915 | 17 | + | 50914046 | 50914419 | 242 | annot_exons |
| novel_circ_002207 | ENSSSCG00000004311 | 1 | - | 57030072 | 57037382 | 453 | annot_exons |
| novel_circ_002208 | ENSSSCG00000008637 | 3 | - | 1.27E+08 | 1.27E+08 | 282 | annot_exons |
| novel_circ_002209 | ENSSSCG00000038561 | 15 | - | 84405614 | 84406551 | 431 | annot_exons |
| novel_circ_002210 | ENSSSCG00000007345 | 17 | + | 41522913 | 41523117 | 205 | exon_intron |
| novel_circ_002211 | NA | 2 | - | 1.34E+08 | 1.34E+08 | 1127 | intergenic |
| novel_circ_002212 | ENSSSCG00000005777 | 6 | - | 1.28E+08 | 1.28E+08 | 1862 | exon_intron |
| novel_circ_002213 | ENSSSCG00000003627 | 6 | + | 91554750 | 91605991 | 3128 | annot_exons |
| novel_circ_002214 | ENSSSCG00000022126 | 9 | + | 1.39E+08 | 1.39E+08 | 471 | annot_exons |
| novel_circ_002215 | ENSSSCG00000024570 | 2 | - | 73829182 | 73837640 | 1429 | annot_exons |
| novel_circ_002216 | ENSSSCG00000039701 | 7 | - | 92743360 | 92781367 | 551 | annot_exons |
| novel_circ_002217 | NA | 3 | + | 97013524 | 97048096 | 34573 | intergenic |
| novel_circ_002218 | ENSSSCG00000000555 | 5 | + | 47226827 | 47238901 | 443 | annot_exons |
| novel_circ_002219 | ENSSSCG00000011237 | 13 | + | 19630228 | 19640780 | 807 | annot_exons |
| novel_circ_002220 | ENSSSCG00000024623 | 13 | + | 1.81E+08 | 1.81E+08 | 350 | annot_exons |
| novel_circ_002221 | ENSSSCG00000008201 | 3 | - | 56798010 | 56800085 | 1248 | annot_exons |
| novel_circ_002222 | ENSSSCG00000002266 | 7 | - | 86024175 | 86024350 | 176 | one_exon |
| novel_circ_002223 | ENSSSCG00000007477 | 17 | - | 52807264 | 52872517 | 1692 | annot_exons |
| novel_circ_002224 | ENSSSCG00000026665 | 12 | - | 37496922 | 37509574 | 316 | annot_exons |
| novel_circ_002225 | ENSSSCG00000003828 | 6 | - | 1.53E+08 | 1.53E+08 | 353 | annot_exons |
| novel_circ_002226 | ENSSSCG00000005191 | 1 | + | 2.09E+08 | 2.09E+08 | 711 | annot_exons |
| novel_circ_002227 | ENSSSCG00000008412 | 3 | + | 87297672 | 87299574 | 268 | annot_exons |
| novel_circ_002228 | ENSSSCG00000027898 | 5 | + | 93044095 | 93049026 | 909 | annot_exons |
| novel_circ_002229 | ENSSSCG00000042401 | 6 | - | 1.57E+08 | 1.57E+08 | 7675 | exon_intron |
| novel_circ_002230 | ENSSSCG00000039885 | 6 | - | 88423227 | 88432891 | 1085 | annot_exons |
| novel_circ_002231 | ENSSSCG00000004576 | 1 | + | 1.11E+08 | 1.11E+08 | 6970 | intronic |
| novel_circ_002232 | ENSSSCG00000000693 | 5 | + | 64155666 | 64155845 | 180 | antisense |
| novel_circ_002233 | ENSSSCG00000002820 | 6 | + | 19168552 | 19184536 | 1316 | annot_exons |
| novel_circ_002234 | ENSSSCG00000036669 | 2 | - | 9213502 | 9213798 | 297 | antisense |
| novel_circ_002235 | ENSSSCG00000016317 | 15 | + | 1.36E+08 | 1.36E+08 | 377 | annot_exons |
| novel_circ_002236 | ENSSSCG00000008445 | 3 | + | 95104014 | 95109324 | 467 | annot_exons |
| novel_circ_002237 | ENSSSCG00000033185 | 13 | + | 77065148 | 77068923 | 244 | annot_exons |
| novel_circ_002238 | ENSSSCG00000016509 | 18 | - | 10292639 | 10316777 | 449 | annot_exons |
| novel_circ_002239 | ENSSSCG00000001068 | 7 | + | 13311326 | 13318444 | 336 | annot_exons |
| novel_circ_002240 | ENSSSCG00000020663 | 18 | + | 5206287 | 5208471 | 249 | annot_exons |
| novel_circ_002241 | NA | 8 | - | 99149758 | 99150184 | 427 | intergenic |
| novel_circ_002242 | ENSSSCG00000025107 | 13 | + | 1.38E+08 | 1.38E+08 | 273 | annot_exons |
| novel_circ_002243 | ENSSSCG00000039763 | 16 | + | 72191516 | 72194195 | 342 | annot_exons |
| novel_circ_002244 | NA | 15 | - | 84236300 | 84236582 | 283 | intergenic |
| novel_circ_002245 | ENSSSCG00000034484 | 6 | + | 75042635 | 75043060 | 426 | one_exon |
| novel_circ_002246 | ENSSSCG00000000970 | 5 | - | 304707 | 308205 | 431 | annot_exons |
| novel_circ_002247 | ENSSSCG00000011961 | 13 | - | 1.58E+08 | 1.58E+08 | 309 | annot_exons |
| novel_circ_002248 | ENSSSCG00000038973 | 5 | + | 1.02E+08 | 1.02E+08 | 410 | annot_exons |
| novel_circ_002249 | ENSSSCG00000017191 | 12 | + | 5374046 | 5383722 | 727 | annot_exons |
| novel_circ_002250 | ENSSSCG00000002370 | 7 | - | 97906851 | 97910353 | 507 | annot_exons |
| novel_circ_002251 | ENSSSCG00000014878 | 9 | - | 11836758 | 11841300 | 435 | annot_exons |
| novel_circ_002252 | ENSSSCG00000001563 | 7 | - | 32218356 | 32225760 | 320 | annot_exons |
| novel_circ_002253 | ENSSSCG00000009793 | 14 | + | 30371329 | 30389491 | 349 | annot_exons |
| novel_circ_002254 | ENSSSCG00000004636 | 1 | + | 1.21E+08 | 1.21E+08 | 532 | annot_exons |
| novel_circ_002255 | ENSSSCG00000017095 | 16 | + | 72985021 | 72985217 | 197 | one_exon |
| novel_circ_002256 | ENSSSCG00000009885 | 14 | + | 39454991 | 39455635 | 265 | annot_exons |
| novel_circ_002257 | ENSSSCG00000011166 | 10 | - | 69212794 | 69235186 | 527 | annot_exons |
| novel_circ_002258 | ENSSSCG00000011972 | 13 | + | 1.59E+08 | 1.59E+08 | 498 | intronic |
| novel_circ_002259 | ENSSSCG00000010799 | 3 | + | 22796026 | 22810630 | 340 | annot_exons |
| novel_circ_002260 | ENSSSCG00000009029 | 8 | - | 80800577 | 80815987 | 222 | annot_exons |
| novel_circ_002261 | ENSSSCG00000021118 | 3 | + | 31255432 | 31256666 | 477 | annot_exons |
| novel_circ_002262 | ENSSSCG00000005016 | 1 | - | 1.8E+08 | 1.8E+08 | 458 | annot_exons |
| novel_circ_002263 | ENSSSCG00000011085 | 10 | - | 53169687 | 53200168 | 286 | annot_exons |
| novel_circ_002264 | ENSSSCG00000022534 | 4 | + | 1.26E+08 | 1.26E+08 | 3705 | annot_exons |
| novel_circ_002265 | ENSSSCG00000035581 | 18 | - | 54260019 | 54269615 | 332 | annot_exons |
| novel_circ_002266 | ENSSSCG00000030354 | 15 | + | 25128581 | 25143159 | 329 | annot_exons |
| novel_circ_002267 | ENSSSCG00000005975 | 4 | + | 15092277 | 15095858 | 307 | annot_exons |
| novel_circ_002268 | ENSSSCG00000006283 | 4 | + | 81156524 | 81169492 | 790 | annot_exons |
| novel_circ_002269 | ENSSSCG00000014835 | 9 | - | 8471324 | 8483952 | 865 | annot_exons |
| novel_circ_002270 | ENSSSCG00000029602 | 10 | - | 68512778 | 68518239 | 275 | annot_exons |
| novel_circ_002271 | ENSSSCG00000017346 | 12 | + | 18492047 | 18497749 | 375 | annot_exons |
| novel_circ_002272 | ENSSSCG00000003700 | 6 | - | 1.07E+08 | 1.07E+08 | 2354 | annot_exons |
| novel_circ_002273 | ENSSSCG00000015393 | 9 | - | 93451048 | 93452747 | 237 | annot_exons |
| novel_circ_002274 | ENSSSCG00000001805 | 7 | - | 52122189 | 52123931 | 1743 | exon_intron |
| novel_circ_002275 | ENSSSCG00000015840 | 15 | - | 53982371 | 53984110 | 247 | annot_exons |
| novel_circ_002276 | ENSSSCG00000006622 | 4 | + | 97819782 | 97820670 | 493 | annot_exons |
| novel_circ_002277 | ENSSSCG00000039373 | 2 | + | 1.26E+08 | 1.26E+08 | 4530 | exon_intron |
| novel_circ_002278 | ENSSSCG00000005382 | 1 | + | 2.41E+08 | 2.41E+08 | 888 | annot_exons |
| novel_circ_002279 | ENSSSCG00000006831 | 4 | + | 1.11E+08 | 1.11E+08 | 463 | annot_exons |
| novel_circ_002280 | ENSSSCG00000002467 | 7 | - | 1.15E+08 | 1.15E+08 | 418 | annot_exons |
| novel_circ_002281 | ENSSSCG00000006872 | 4 | - | 1.18E+08 | 1.18E+08 | 775 | annot_exons |
| novel_circ_002282 | ENSSSCG00000046937 | 6 | + | 68529830 | 68544564 | 429 | exon_intron |
| novel_circ_002283 | ENSSSCG00000024296 | 13 | - | 10700223 | 10710044 | 458 | annot_exons |
| novel_circ_002284 | ENSSSCG00000008540 | 3 | - | 1.1E+08 | 1.1E+08 | 827 | annot_exons |
| novel_circ_002285 | ENSSSCG00000017688 | 12 | + | 38083733 | 38093378 | 674 | annot_exons |
| novel_circ_002286 | ENSSSCG00000008103 | 3 | - | 44375005 | 44381705 | 356 | annot_exons |
| novel_circ_002287 | ENSSSCG00000015428 | 9 | - | 1.05E+08 | 1.05E+08 | 462 | annot_exons |
| novel_circ_002288 | ENSSSCG00000035555 | AEMK02000452.1 | + | 3333362 | 3333778 | 417 | one_exon |
| novel_circ_002289 | ENSSSCG00000010640 | 14 | - | 1.24E+08 | 1.24E+08 | 645 | annot_exons |
| novel_circ_002290 | ENSSSCG00000005366 | 1 | + | 2.39E+08 | 2.39E+08 | 473 | annot_exons |
| novel_circ_002291 | ENSSSCG00000030268 | 1 | - | 2.26E+08 | 2.26E+08 | 272 | annot_exons |
| novel_circ_002292 | ENSSSCG00000005459 | 1 | - | 2.52E+08 | 2.52E+08 | 507 | annot_exons |
| novel_circ_002293 | ENSSSCG00000021749 | 13 | - | 1.21E+08 | 1.21E+08 | 372 | annot_exons |
| novel_circ_002294 | ENSSSCG00000038001 | 3 | + | 1.11E+08 | 1.11E+08 | 281 | annot_exons |
| novel_circ_002295 | ENSSSCG00000007930 | 3 | - | 37706321 | 37710535 | 403 | annot_exons |
| novel_circ_002296 | ENSSSCG00000017676 | 12 | + | 37029818 | 37030721 | 904 | antisense |
| novel_circ_002297 | ENSSSCG00000015545 | 9 | - | 1.24E+08 | 1.24E+08 | 904 | annot_exons |
| novel_circ_002298 | ENSSSCG00000010723 | 14 | + | 1.33E+08 | 1.33E+08 | 258 | annot_exons |
| novel_circ_002299 | ENSSSCG00000023269 | 8 | + | 55194966 | 55211996 | 1105 | annot_exons |
| novel_circ_002300 | ENSSSCG00000014411 | 2 | - | 1.47E+08 | 1.47E+08 | 312 | annot_exons |
| novel_circ_002301 | ENSSSCG00000005916 | 4 | - | 594402 | 594860 | 380 | annot_exons |
| novel_circ_002302 | ENSSSCG00000005388 | 1 | + | 2.42E+08 | 2.42E+08 | 775 | annot_exons |
| novel_circ_002303 | ENSSSCG00000002461 | 7 | - | 1.15E+08 | 1.15E+08 | 1359 | annot_exons |
| novel_circ_002304 | ENSSSCG00000015940 | 15 | + | 76166995 | 76177857 | 493 | annot_exons |
| novel_circ_002305 | ENSSSCG00000010640 | 14 | - | 1.24E+08 | 1.24E+08 | 531 | annot_exons |
| novel_circ_002306 | ENSSSCG00000039416 | 15 | - | 15666332 | 15666579 | 248 | antisense |
| novel_circ_002307 | ENSSSCG00000033527 | 6 | + | 37255641 | 37317710 | 412 | annot_exons |
| novel_circ_002308 | ENSSSCG00000033786 | 2 | + | 44943647 | 44945046 | 300 | annot_exons |
| novel_circ_002309 | ENSSSCG00000038128 | 6 | + | 7552153 | 7603038 | 7308 | annot_exons |
| novel_circ_002310 | ENSSSCG00000007275 | 17 | + | 37488036 | 37488303 | 268 | intronic |
| novel_circ_002311 | ENSSSCG00000007951 | 3 | + | 38413987 | 38414699 | 713 | one_exon |
| novel_circ_002312 | ENSSSCG00000031170 | 9 | - | 33091637 | 33092047 | 411 | one_exon |
| novel_circ_002313 | ENSSSCG00000024814 | X | - | 47765553 | 47767213 | 418 | annot_exons |
| novel_circ_002314 | ENSSSCG00000006101 | 4 | + | 42327946 | 42361848 | 2202 | annot_exons |
| novel_circ_002315 | ENSSSCG00000026753 | 13 | + | 1.35E+08 | 1.35E+08 | 337 | annot_exons |
| novel_circ_002316 | ENSSSCG00000008697 | 8 | + | 1829671 | 1832303 | 309 | annot_exons |
| novel_circ_002317 | ENSSSCG00000014292 | 2 | + | 1.35E+08 | 1.35E+08 | 387 | annot_exons |
| novel_circ_002318 | ENSSSCG00000026569 | 3 | - | 31988381 | 32085104 | 1383 | annot_exons |
| novel_circ_002319 | ENSSSCG00000015923 | 15 | + | 75334315 | 75335592 | 331 | annot_exons |
| novel_circ_002320 | ENSSSCG00000024784 | 10 | - | 23525570 | 23536387 | 1776 | annot_exons |
| novel_circ_002321 | ENSSSCG00000021203 | 13 | - | 79490411 | 79496843 | 531 | annot_exons |
| novel_circ_002322 | ENSSSCG00000024614 | 13 | + | 1.97E+08 | 1.97E+08 | 254 | annot_exons |
| novel_circ_002323 | ENSSSCG00000025315 | 8 | - | 19125340 | 19130013 | 257 | annot_exons |
| novel_circ_002324 | ENSSSCG00000012403 | X | + | 57556824 | 57557656 | 833 | one_exon |
| novel_circ_002325 | ENSSSCG00000010327 | 14 | - | 80431028 | 80432888 | 285 | annot_exons |
| novel_circ_002326 | ENSSSCG00000012250 | X | + | 36355880 | 36356120 | 241 | antisense |
| novel_circ_002327 | ENSSSCG00000000068 | 5 | - | 7341106 | 7348222 | 713 | annot_exons |
| novel_circ_002328 | ENSSSCG00000009338 | 11 | + | 8608726 | 8619754 | 715 | annot_exons |
| novel_circ_002329 | ENSSSCG00000033753 | 3 | - | 1.15E+08 | 1.15E+08 | 297 | annot_exons |
| novel_circ_002330 | ENSSSCG00000009435 | 11 | - | 25578332 | 25587428 | 552 | annot_exons |
| novel_circ_002331 | ENSSSCG00000013402 | 2 | - | 47491558 | 47494398 | 417 | annot_exons |
| novel_circ_002332 | NA | 15 | - | 84265250 | 84265519 | 270 | intergenic |
| novel_circ_002333 | ENSSSCG00000007073 | 17 | + | 22003313 | 22023699 | 649 | annot_exons |
| novel_circ_002334 | ENSSSCG00000000901 | 5 | + | 88211724 | 88212454 | 250 | annot_exons |
| novel_circ_002335 | ENSSSCG00000009864 | 14 | + | 36225903 | 36243162 | 169 | annot_exons |
| novel_circ_002336 | ENSSSCG00000027722 | 8 | - | 1.39E+08 | 1.39E+08 | 705 | annot_exons |
| novel_circ_002337 | ENSSSCG00000010690 | 14 | + | 1.3E+08 | 1.3E+08 | 259 | annot_exons |
| novel_circ_002338 | ENSSSCG00000014343 | 2 | + | 1.41E+08 | 1.41E+08 | 30703 | exon_intron |
| novel_circ_002339 | ENSSSCG00000002430 | 7 | - | 1.12E+08 | 1.12E+08 | 679 | exon_intron |
| novel_circ_002340 | ENSSSCG00000015335 | 9 | + | 75628054 | 75646985 | 479 | annot_exons |
| novel_circ_002341 | ENSSSCG00000015052 | 9 | - | 41434661 | 41464576 | 1433 | annot_exons |
| novel_circ_002342 | ENSSSCG00000034164 | 10 | - | 11762419 | 11766465 | 275 | annot_exons |
| novel_circ_002343 | ENSSSCG00000016836 | 16 | - | 21524717 | 21531355 | 300 | annot_exons |
| novel_circ_002344 | ENSSSCG00000004140 | 1 | - | 22891664 | 22898956 | 313 | annot_exons |
| novel_circ_002345 | ENSSSCG00000012875 | 2 | - | 4099610 | 4100223 | 255 | annot_exons |
| novel_circ_002346 | ENSSSCG00000034386 | 14 | + | 31715996 | 31720137 | 632 | annot_exons |
| novel_circ_002347 | ENSSSCG00000005288 | 1 | + | 2.32E+08 | 2.32E+08 | 731 | annot_exons |
| novel_circ_002348 | ENSSSCG00000015777 | 15 | + | 45021868 | 45026211 | 465 | annot_exons |
| novel_circ_002349 | ENSSSCG00000003718 | 6 | + | 1.11E+08 | 1.11E+08 | 1504 | annot_exons |
| novel_circ_002350 | ENSSSCG00000010834 | 10 | + | 11425122 | 11426929 | 1808 | exon_intron |
| novel_circ_002351 | ENSSSCG00000002923 | 6 | + | 45463249 | 45467918 | 403 | annot_exons |
| novel_circ_002352 | ENSSSCG00000012884 | 2 | - | 4407216 | 4408798 | 261 | annot_exons |
| novel_circ_002353 | ENSSSCG00000014269 | 2 | - | 1.34E+08 | 1.34E+08 | 1900 | annot_exons |
| novel_circ_002354 | ENSSSCG00000029227 | 8 | - | 11712865 | 11724812 | 380 | annot_exons |
| novel_circ_002355 | ENSSSCG00000023974 | 2 | + | 16347499 | 16389509 | 713 | annot_exons |
| novel_circ_002356 | ENSSSCG00000007317 | 17 | + | 39739371 | 39773039 | 750 | annot_exons |
| novel_circ_002357 | ENSSSCG00000049098 | 4 | + | 3031809 | 3032045 | 237 | one_exon |
| novel_circ_002358 | ENSSSCG00000003218 | 6 | + | 55302098 | 55302784 | 585 | annot_exons |
| novel_circ_002359 | ENSSSCG00000017983 | 12 | - | 53366715 | 53367213 | 308 | annot_exons |
| novel_circ_002360 | ENSSSCG00000007280 | 17 | + | 37862504 | 37881223 | 459 | annot_exons |
| novel_circ_002361 | ENSSSCG00000007917 | 3 | - | 35059400 | 35088585 | 406 | annot_exons |
| novel_circ_002362 | ENSSSCG00000029311 | 14 | + | 71370337 | 71370814 | 478 | exon_intron |
| novel_circ_002363 | ENSSSCG00000005388 | 1 | + | 2.42E+08 | 2.42E+08 | 3045 | exon_intron |
| novel_circ_002364 | ENSSSCG00000011166 | 10 | - | 69224981 | 69235186 | 10206 | exon_intron |
| novel_circ_002365 | ENSSSCG00000016956 | 16 | + | 45500364 | 45511908 | 1134 | annot_exons |
| novel_circ_002366 | NA | 15 | - | 84317384 | 84317616 | 233 | intergenic |
| novel_circ_002367 | ENSSSCG00000016223 | 15 | + | 1.25E+08 | 1.25E+08 | 414 | annot_exons |
| novel_circ_002368 | ENSSSCG00000024486 | 17 | + | 11904677 | 11905517 | 177 | annot_exons |
| novel_circ_002369 | ENSSSCG00000007109 | 17 | + | 29132369 | 29150556 | 1124 | annot_exons |
| novel_circ_002370 | ENSSSCG00000003218 | 6 | + | 55296593 | 55297457 | 277 | annot_exons |
| novel_circ_002371 | ENSSSCG00000005602 | 1 | + | 2.66E+08 | 2.66E+08 | 1062 | annot_exons |
| novel_circ_002372 | ENSSSCG00000026268 | 14 | - | 80286341 | 80294935 | 884 | annot_exons |
| novel_circ_002373 | ENSSSCG00000021784 | 8 | + | 1.16E+08 | 1.16E+08 | 334 | annot_exons |
| novel_circ_002374 | ENSSSCG00000039793 | 6 | - | 1.51E+08 | 1.51E+08 | 278 | annot_exons |
| novel_circ_002375 | ENSSSCG00000004140 | 1 | - | 22891664 | 22901667 | 408 | annot_exons |
| novel_circ_002376 | ENSSSCG00000007878 | 3 | + | 28754389 | 28790148 | 384 | annot_exons |
| novel_circ_002377 | ENSSSCG00000008016 | 3 | + | 40194210 | 40194413 | 204 | one_exon |
| novel_circ_002378 | ENSSSCG00000005367 | 1 | - | 2.39E+08 | 2.39E+08 | 1212 | exon_intron |
| novel_circ_002379 | ENSSSCG00000021060 | 11 | + | 18114767 | 18133037 | 271 | annot_exons |
| novel_circ_002380 | ENSSSCG00000028117 | 1 | - | 2.61E+08 | 2.61E+08 | 288 | annot_exons |
| novel_circ_002381 | ENSSSCG00000009345 | 11 | + | 9166634 | 9190868 | 613 | annot_exons |
| novel_circ_002382 | ENSSSCG00000015360 | 9 | - | 85859910 | 85871670 | 611 | annot_exons |
| novel_circ_002383 | ENSSSCG00000009021 | 8 | + | 78282065 | 78381727 | 576 | annot_exons |
| novel_circ_002384 | ENSSSCG00000009072 | 8 | + | 96703653 | 96707124 | 3472 | exon_intron |
| novel_circ_002385 | ENSSSCG00000006202 | 4 | - | 67679793 | 67689279 | 591 | exon_intron |
| novel_circ_002386 | ENSSSCG00000010190 | 14 | - | 60414213 | 60414590 | 378 | antisense |
| novel_circ_002387 | ENSSSCG00000007264 | 17 | - | 36877687 | 36881256 | 277 | annot_exons |
| novel_circ_002388 | ENSSSCG00000015015 | 9 | - | 38501090 | 38565438 | 859 | annot_exons |
| novel_circ_002389 | ENSSSCG00000004207 | 1 | - | 33815062 | 33816042 | 308 | annot_exons |
| novel_circ_002390 | ENSSSCG00000029633 | 6 | + | 1.57E+08 | 1.57E+08 | 244 | annot_exons |
| novel_circ_002391 | ENSSSCG00000003559 | 6 | + | 84105757 | 84106350 | 331 | annot_exons |
| novel_circ_002392 | ENSSSCG00000004457 | 1 | - | 83042489 | 83044702 | 552 | annot_exons |
| novel_circ_002393 | ENSSSCG00000027293 | 16 | + | 49110894 | 49112899 | 242 | annot_exons |
| novel_circ_002394 | ENSSSCG00000012063 | 13 | - | 2.01E+08 | 2.01E+08 | 462 | annot_exons |
| novel_circ_002395 | ENSSSCG00000026744 | 11 | - | 1335980 | 1371291 | 799 | annot_exons |
| novel_circ_002396 | ENSSSCG00000015565 | 9 | + | 1.26E+08 | 1.26E+08 | 2341 | exon_intron |
| novel_circ_002397 | ENSSSCG00000024614 | 13 | + | 1.97E+08 | 1.97E+08 | 378 | annot_exons |
| novel_circ_002398 | ENSSSCG00000009787 | 14 | - | 30059979 | 30063150 | 360 | annot_exons |
| novel_circ_002399 | ENSSSCG00000004394 | 1 | + | 77086749 | 77087208 | 460 | one_exon |
| novel_circ_002400 | ENSSSCG00000036855 | 13 | + | 81772003 | 81784515 | 5835 | annot_exons |
| novel_circ_002401 | ENSSSCG00000008376 | 3 | - | 79857162 | 79857443 | 282 | one_exon |
| novel_circ_002402 | ENSSSCG00000038397 | 4 | + | 2858804 | 2868663 | 469 | annot_exons |
| novel_circ_002403 | ENSSSCG00000025768 | 7 | - | 1.12E+08 | 1.12E+08 | 254 | antisense |
| novel_circ_002404 | ENSSSCG00000027860 | 2 | + | 1.04E+08 | 1.04E+08 | 264 | annot_exons |
| novel_circ_002405 | ENSSSCG00000010798 | 3 | + | 22715279 | 22716037 | 219 | annot_exons |
| novel_circ_002406 | ENSSSCG00000021292 | 6 | - | 1.6E+08 | 1.6E+08 | 588 | annot_exons |
| novel_circ_002407 | ENSSSCG00000039285 | 13 | + | 1.17E+08 | 1.17E+08 | 288 | annot_exons |
| novel_circ_002408 | ENSSSCG00000016549 | 18 | - | 17598680 | 17629822 | 605 | annot_exons |
| novel_circ_002409 | ENSSSCG00000016067 | 15 | + | 1E+08 | 1E+08 | 220 | antisense |
| novel_circ_002410 | ENSSSCG00000007477 | 17 | - | 52866612 | 52872517 | 1175 | annot_exons |
| novel_circ_002411 | ENSSSCG00000029030 | 6 | - | 1.04E+08 | 1.04E+08 | 238 | annot_exons |
| novel_circ_002412 | ENSSSCG00000004048 | 1 | + | 7598843 | 7599773 | 481 | annot_exons |
| novel_circ_002413 | ENSSSCG00000005987 | 4 | - | 16277323 | 16281983 | 263 | annot_exons |
| novel_circ_002414 | ENSSSCG00000029945 | 5 | - | 10032632 | 10034248 | 557 | annot_exons |
| novel_circ_002415 | ENSSSCG00000016248 | 15 | + | 1.29E+08 | 1.29E+08 | 756 | annot_exons |
| novel_circ_002416 | ENSSSCG00000014832 | 9 | + | 8351833 | 8353265 | 228 | annot_exons |
| novel_circ_002417 | ENSSSCG00000017184 | 12 | + | 5152130 | 5159903 | 240 | annot_exons |
| novel_circ_002418 | ENSSSCG00000007951 | 3 | + | 38483180 | 38484897 | 396 | annot_exons |
| novel_circ_002419 | ENSSSCG00000009338 | 11 | + | 8660882 | 8662015 | 265 | annot_exons |
| novel_circ_002420 | ENSSSCG00000004814 | 1 | + | 1.38E+08 | 1.38E+08 | 217 | annot_exons |
| novel_circ_002421 | ENSSSCG00000006728 | 4 | + | 1.03E+08 | 1.03E+08 | 382 | annot_exons |
| novel_circ_002422 | ENSSSCG00000005288 | 1 | - | 2.32E+08 | 2.32E+08 | 1417 | antisense |
| novel_circ_002423 | ENSSSCG00000020663 | 18 | + | 5029807 | 5032752 | 2946 | intronic |
| novel_circ_002424 | ENSSSCG00000002259 | 7 | + | 88126639 | 88165789 | 1056 | annot_exons |
| novel_circ_002425 | ENSSSCG00000032007 | 3 | - | 86251390 | 86251927 | 538 | antisense |
| novel_circ_002426 | ENSSSCG00000015582 | 9 | + | 1.29E+08 | 1.29E+08 | 495 | annot_exons |
| novel_circ_002427 | ENSSSCG00000011295 | 13 | + | 26685774 | 26686462 | 689 | one_exon |
| novel_circ_002428 | NA | 9 | + | 1.22E+08 | 1.22E+08 | 12417 | intergenic |
| novel_circ_002429 | ENSSSCG00000012713 | X | - | 1.14E+08 | 1.14E+08 | 318 | annot_exons |
| novel_circ_002430 | ENSSSCG00000033228 | 12 | - | 27239024 | 27250993 | 1441 | annot_exons |
| novel_circ_002431 | ENSSSCG00000032377 | 7 | + | 64253214 | 64267051 | 763 | annot_exons |
| novel_circ_002432 | ENSSSCG00000015379 | 9 | + | 90712543 | 90715612 | 511 | annot_exons |
| novel_circ_002433 | ENSSSCG00000016409 | 18 | - | 1599270 | 1604899 | 478 | annot_exons |
| novel_circ_002434 | ENSSSCG00000015669 | 15 | - | 4207516 | 4211249 | 318 | annot_exons |
| novel_circ_002435 | ENSSSCG00000027403 | 8 | - | 55611873 | 55612616 | 255 | annot_exons |
| novel_circ_002436 | ENSSSCG00000018049 | 12 | - | 60361138 | 60376568 | 615 | annot_exons |
| novel_circ_002437 | ENSSSCG00000031459 | 2 | + | 4748455 | 4749110 | 276 | annot_exons |
| novel_circ_002438 | ENSSSCG00000008600 | 3 | + | 1.18E+08 | 1.18E+08 | 666 | annot_exons |
| novel_circ_002439 | ENSSSCG00000005465 | 1 | - | 2.53E+08 | 2.53E+08 | 270 | annot_exons |
| novel_circ_002440 | ENSSSCG00000006865 | 4 | - | 1.18E+08 | 1.18E+08 | 5030 | exon_intron |
| novel_circ_002441 | NA | 7 | - | 78551520 | 78551856 | 337 | intergenic |
| novel_circ_002442 | ENSSSCG00000012007 | 13 | - | 1.79E+08 | 1.79E+08 | 382 | annot_exons |
| novel_circ_002443 | ENSSSCG00000013347 | 2 | - | 38950389 | 38963167 | 354 | annot_exons |
| novel_circ_002444 | ENSSSCG00000010579 | 14 | + | 1.13E+08 | 1.13E+08 | 47841 | exon_intron |
| novel_circ_002445 | ENSSSCG00000023371 | 6 | + | 53431186 | 53431598 | 413 | one_exon |
| novel_circ_002446 | ENSSSCG00000008943 | 8 | + | 68022433 | 68042773 | 20341 | exon_intron |
| novel_circ_002447 | NA | 15 | - | 84472690 | 84475342 | 2653 | intergenic |
| novel_circ_002448 | ENSSSCG00000023533 | 1 | - | 1.48E+08 | 1.48E+08 | 254 | annot_exons |
| novel_circ_002449 | ENSSSCG00000010114 | 14 | - | 50953777 | 50954138 | 271 | annot_exons |
| novel_circ_002450 | ENSSSCG00000011235 | 13 | - | 19234817 | 19251022 | 229 | annot_exons |
| novel_circ_002451 | ENSSSCG00000017694 | 12 | - | 38796303 | 38800438 | 386 | annot_exons |
| novel_circ_002452 | ENSSSCG00000004845 | 1 | - | 1.44E+08 | 1.44E+08 | 363 | exon_intron |
| novel_circ_002453 | ENSSSCG00000004293 | 1 | - | 54472561 | 54494313 | 629 | annot_exons |
| novel_circ_002454 | ENSSSCG00000010467 | 14 | + | 1.04E+08 | 1.04E+08 | 694 | annot_exons |
| novel_circ_002455 | ENSSSCG00000010579 | 14 | + | 1.13E+08 | 1.13E+08 | 305 | annot_exons |
| novel_circ_002456 | ENSSSCG00000027667 | 7 | + | 1.21E+08 | 1.21E+08 | 326 | annot_exons |
| novel_circ_002457 | ENSSSCG00000034191 | 2 | + | 42984787 | 42988525 | 414 | annot_exons |
| novel_circ_002458 | ENSSSCG00000011811 | 13 | + | 1.26E+08 | 1.26E+08 | 12431 | exon_intron |
| novel_circ_002459 | ENSSSCG00000016758 | 18 | - | 51340815 | 51349397 | 358 | annot_exons |
| novel_circ_002460 | ENSSSCG00000027070 | 17 | + | 30918813 | 30920113 | 220 | annot_exons |
| novel_circ_002461 | ENSSSCG00000024088 | 1 | - | 1.09E+08 | 1.09E+08 | 231 | annot_exons |
| novel_circ_002462 | ENSSSCG00000034545 | 2 | - | 75711485 | 75714291 | 308 | annot_exons |
| novel_circ_002463 | ENSSSCG00000000502 | 5 | + | 34470911 | 34502196 | 651 | annot_exons |
| novel_circ_002464 | ENSSSCG00000016863 | 16 | - | 26631927 | 26646919 | 486 | annot_exons |
| novel_circ_002465 | ENSSSCG00000025592 | 13 | + | 1.27E+08 | 1.27E+08 | 14035 | exon_intron |
| novel_circ_002466 | ENSSSCG00000004082 | 1 | + | 14087204 | 14089226 | 306 | annot_exons |
| novel_circ_002467 | ENSSSCG00000025478 | 6 | + | 1.07E+08 | 1.07E+08 | 377 | annot_exons |
| novel_circ_002468 | ENSSSCG00000005170 | 1 | - | 2.04E+08 | 2.04E+08 | 1501 | annot_exons |
| novel_circ_002469 | ENSSSCG00000004303 | 1 | - | 55940567 | 55954186 | 425 | annot_exons |
| novel_circ_002470 | ENSSSCG00000038708 | 7 | - | 48821286 | 48866128 | 456 | annot_exons |
| novel_circ_002471 | NA | 15 | - | 84244360 | 84246210 | 1851 | intergenic |
| novel_circ_002472 | ENSSSCG00000014902 | 9 | + | 17351072 | 17373225 | 547 | annot_exons |
| novel_circ_002473 | ENSSSCG00000030467 | 13 | - | 1.09E+08 | 1.09E+08 | 400 | annot_exons |
| novel_circ_002474 | ENSSSCG00000034164 | 10 | - | 11818651 | 11823816 | 338 | annot_exons |
| novel_circ_002475 | ENSSSCG00000029050 | 2 | - | 51241937 | 51243482 | 422 | exon_intron |
| novel_circ_002476 | ENSSSCG00000020743 | 1 | + | 2.64E+08 | 2.64E+08 | 451 | annot_exons |
| novel_circ_002477 | ENSSSCG00000024486 | 17 | + | 11825820 | 11855091 | 456 | annot_exons |
| novel_circ_002478 | ENSSSCG00000003218 | 6 | + | 55304148 | 55306393 | 437 | annot_exons |
| novel_circ_002479 | ENSSSCG00000039802 | 13 | + | 19147167 | 19170984 | 375 | annot_exons |
| novel_circ_002480 | ENSSSCG00000007541 | 3 | - | 313709 | 315231 | 293 | annot_exons |
| novel_circ_002481 | ENSSSCG00000006866 | 4 | + | 1.18E+08 | 1.18E+08 | 339 | annot_exons |
| novel_circ_002482 | ENSSSCG00000010863 | 10 | + | 15051106 | 15056946 | 563 | annot_exons |
| novel_circ_002483 | ENSSSCG00000022636 | 5 | + | 42740180 | 42755389 | 844 | annot_exons |
| novel_circ_002484 | ENSSSCG00000010107 | 14 | - | 50721468 | 50724385 | 295 | annot_exons |
| novel_circ_002485 | ENSSSCG00000039332 | 9 | - | 1.2E+08 | 1.2E+08 | 622 | annot_exons |
| novel_circ_002486 | ENSSSCG00000029533 | 14 | + | 1.12E+08 | 1.12E+08 | 243 | one_exon |
| novel_circ_002487 | ENSSSCG00000021761 | 10 | - | 32569928 | 32576571 | 1049 | annot_exons |
| novel_circ_002488 | ENSSSCG00000009410 | 11 | + | 21020402 | 21022536 | 279 | annot_exons |
| novel_circ_002489 | ENSSSCG00000015886 | 15 | - | 67050872 | 67057687 | 287 | annot_exons |
| novel_circ_002490 | ENSSSCG00000014411 | 2 | - | 1.47E+08 | 1.47E+08 | 393 | annot_exons |
| novel_circ_002491 | ENSSSCG00000002259 | 7 | + | 88148989 | 88165789 | 2342 | exon_intron |
| novel_circ_002492 | ENSSSCG00000015238 | 9 | - | 55976991 | 56013767 | 601 | annot_exons |
| novel_circ_002493 | ENSSSCG00000004942 | 1 | - | 1.64E+08 | 1.64E+08 | 394 | annot_exons |
| novel_circ_002494 | ENSSSCG00000004729 | 1 | - | 1.29E+08 | 1.29E+08 | 183 | one_exon |
| novel_circ_002495 | ENSSSCG00000036909 | 6 | + | 1.67E+08 | 1.67E+08 | 3692 | annot_exons |
| novel_circ_002496 | ENSSSCG00000002547 | AEMK02000452.1 | + | 2196760 | 2208500 | 520 | annot_exons |
| novel_circ_002497 | ENSSSCG00000027669 | 15 | - | 1.2E+08 | 1.2E+08 | 1235 | annot_exons |
| novel_circ_002498 | ENSSSCG00000009326 | 11 | - | 7025645 | 7047232 | 633 | annot_exons |
| novel_circ_002499 | ENSSSCG00000008512 | 3 | - | 1.07E+08 | 1.07E+08 | 318 | annot_exons |
| novel_circ_002500 | ENSSSCG00000016863 | 16 | - | 26644381 | 26646919 | 200 | annot_exons |
| novel_circ_002501 | ENSSSCG00000034484 | 6 | + | 75076438 | 75083806 | 514 | annot_exons |
| novel_circ_002502 | ENSSSCG00000001732 | 7 | - | 43610581 | 43617387 | 691 | annot_exons |
| novel_circ_002503 | ENSSSCG00000028886 | 9 | - | 139769 | 142536 | 2768 | exon_intron |
| novel_circ_002504 | ENSSSCG00000009302 | 11 | - | 4639863 | 4652306 | 243 | annot_exons |
| novel_circ_002505 | ENSSSCG00000006386 | 4 | + | 90188160 | 90190282 | 792 | annot_exons |
| novel_circ_002506 | ENSSSCG00000006995 | 17 | - | 5722312 | 5732265 | 432 | annot_exons |
| novel_circ_002507 | NA | 15 | - | 84300946 | 84301464 | 519 | intergenic |
| novel_circ_002508 | ENSSSCG00000005934 | 4 | + | 3108315 | 3115539 | 248 | annot_exons |
| novel_circ_002509 | ENSSSCG00000009834 | 14 | - | 32705988 | 32710060 | 560 | annot_exons |
| novel_circ_002510 | ENSSSCG00000016185 | 15 | + | 1.2E+08 | 1.2E+08 | 329 | annot_exons |
| novel_circ_002511 | ENSSSCG00000020963 | 9 | + | 1.08E+08 | 1.08E+08 | 402 | annot_exons |
| novel_circ_002512 | ENSSSCG00000017754 | 12 | + | 44136404 | 44140669 | 4266 | exon_intron |
| novel_circ_002513 | ENSSSCG00000003488 | 6 | - | 77590737 | 77592790 | 585 | annot_exons |
| novel_circ_002514 | ENSSSCG00000011963 | 13 | + | 1.59E+08 | 1.59E+08 | 301 | annot_exons |
| novel_circ_002515 | ENSSSCG00000010548 | 14 | - | 1.11E+08 | 1.11E+08 | 379 | annot_exons |
| novel_circ_002516 | ENSSSCG00000002381 | 7 | - | 98303462 | 98312065 | 552 | annot_exons |
| novel_circ_002517 | ENSSSCG00000000522 | 5 | - | 39348509 | 39350101 | 271 | annot_exons |
| novel_circ_002518 | ENSSSCG00000001924 | 7 | - | 60731692 | 60732585 | 174 | annot_exons |
| novel_circ_002519 | ENSSSCG00000010239 | 14 | - | 71521813 | 71526357 | 2094 | annot_exons |
| novel_circ_002520 | ENSSSCG00000003989 | 6 | + | 62375379 | 62376393 | 1015 | one_exon |
| novel_circ_002521 | ENSSSCG00000010900 | 10 | - | 20438111 | 20481282 | 421 | annot_exons |
| novel_circ_002522 | ENSSSCG00000005465 | 1 | - | 2.53E+08 | 2.53E+08 | 1736 | annot_exons |
| novel_circ_002523 | ENSSSCG00000026346 | 1 | + | 83165723 | 83180536 | 642 | annot_exons |
| novel_circ_002524 | ENSSSCG00000016922 | 16 | + | 36258140 | 36268756 | 535 | annot_exons |
| novel_circ_002525 | ENSSSCG00000015558 | 9 | + | 1.25E+08 | 1.25E+08 | 320 | annot_exons |
| novel_circ_002526 | ENSSSCG00000024777 | 15 | - | 1.39E+08 | 1.39E+08 | 1412 | exon_intron |
| novel_circ_002527 | ENSSSCG00000031332 | 1 | + | 1.87E+08 | 1.87E+08 | 313 | one_exon |
| novel_circ_002528 | ENSSSCG00000000955 | 5 | - | 2688838 | 2692132 | 192 | annot_exons |
| novel_circ_002529 | ENSSSCG00000005994 | 4 | + | 18397410 | 18444305 | 428 | annot_exons |
| novel_circ_002530 | ENSSSCG00000008312 | 3 | - | 71024711 | 71026357 | 258 | annot_exons |
| novel_circ_002531 | ENSSSCG00000030343 | 16 | - | 34791656 | 34817575 | 1054 | annot_exons |
| novel_circ_002532 | ENSSSCG00000004602 | 1 | - | 1.16E+08 | 1.16E+08 | 436 | annot_exons |
| novel_circ_002533 | ENSSSCG00000006308 | 4 | - | 83468286 | 83468587 | 302 | antisense |
| novel_circ_002534 | ENSSSCG00000000492 | 5 | + | 33533423 | 33548592 | 1599 | annot_exons |
| novel_circ_002535 | ENSSSCG00000010732 | 14 | - | 1.34E+08 | 1.34E+08 | 779 | one_exon |
| novel_circ_002536 | NA | 1 | + | 22436989 | 22438414 | 1426 | intergenic |
| novel_circ_002537 | ENSSSCG00000024125 | 11 | + | 18007133 | 18020404 | 700 | annot_exons |
| novel_circ_002538 | ENSSSCG00000014838 | 9 | - | 8646177 | 8646907 | 341 | annot_exons |
| novel_circ_002539 | ENSSSCG00000003512 | 6 | - | 79099871 | 79108480 | 425 | annot_exons |
| novel_circ_002540 | ENSSSCG00000009889 | 14 | - | 39918840 | 39924474 | 438 | annot_exons |
| novel_circ_002541 | ENSSSCG00000025478 | 6 | + | 1.07E+08 | 1.07E+08 | 679 | annot_exons |
| novel_circ_002542 | ENSSSCG00000034191 | 2 | + | 42572532 | 42573733 | 1202 | exon_intron |
| novel_circ_002543 | ENSSSCG00000027410 | 17 | + | 37114409 | 37127180 | 514 | annot_exons |
| novel_circ_002544 | ENSSSCG00000017578 | 12 | + | 26250942 | 26257412 | 1111 | annot_exons |
| novel_circ_002545 | ENSSSCG00000031940 | 2 | - | 36828617 | 36840192 | 287 | annot_exons |
| novel_circ_002546 | ENSSSCG00000011103 | 10 | - | 57126906 | 57128875 | 1970 | exon_intron |
| novel_circ_002547 | ENSSSCG00000010485 | 14 | + | 1.06E+08 | 1.06E+08 | 277 | annot_exons |
| novel_circ_002548 | ENSSSCG00000002467 | 7 | - | 1.15E+08 | 1.15E+08 | 737 | annot_exons |
| novel_circ_002549 | NA | 15 | + | 84262105 | 84262464 | 360 | intergenic |
| novel_circ_002550 | ENSSSCG00000011332 | 13 | - | 29944690 | 29945901 | 255 | annot_exons |
| novel_circ_002551 | ENSSSCG00000011085 | 10 | - | 53089612 | 53104147 | 370 | annot_exons |
| novel_circ_002552 | ENSSSCG00000015488 | 9 | - | 1.16E+08 | 1.16E+08 | 413 | annot_exons |
| novel_circ_002553 | ENSSSCG00000016976 | 16 | - | 48847946 | 48849496 | 255 | annot_exons |
| novel_circ_002554 | ENSSSCG00000003805 | 6 | - | 1.46E+08 | 1.46E+08 | 5466 | exon_intron |
| novel_circ_002555 | ENSSSCG00000004918 | 1 | + | 1.62E+08 | 1.62E+08 | 3482 | annot_exons |
| novel_circ_002556 | ENSSSCG00000048636 | 2 | + | 1.03E+08 | 1.03E+08 | 6992 | exon_intron |
| novel_circ_002557 | NA | 15 | + | 84253764 | 84253974 | 211 | intergenic |
| novel_circ_002558 | ENSSSCG00000002535 | AEMK02000452.1 | + | 917174 | 917616 | 443 | antisense |
| novel_circ_002559 | ENSSSCG00000004037 | 1 | - | 6896014 | 6899204 | 491 | annot_exons |
| novel_circ_002560 | ENSSSCG00000004293 | 1 | - | 54520912 | 54529700 | 406 | annot_exons |
| novel_circ_002561 | ENSSSCG00000016922 | 16 | + | 36258140 | 36269888 | 595 | annot_exons |
| novel_circ_002562 | ENSSSCG00000006388 | 4 | + | 90231216 | 90244818 | 1232 | exon_intron |
| novel_circ_002563 | ENSSSCG00000016033 | 15 | + | 93239180 | 93243693 | 234 | annot_exons |
| novel_circ_002564 | ENSSSCG00000004544 | 1 | + | 1.07E+08 | 1.07E+08 | 363 | annot_exons |
| novel_circ_002565 | ENSSSCG00000009924 | 14 | - | 40973122 | 40979005 | 315 | annot_exons |
| novel_circ_002566 | ENSSSCG00000023141 | 14 | - | 1E+08 | 1E+08 | 328 | annot_exons |
| novel_circ_002567 | NA | 15 | - | 84238794 | 84318950 | 80157 | intergenic |
| novel_circ_002568 | ENSSSCG00000034139 | 1 | - | 30306043 | 30317807 | 405 | annot_exons |
| novel_circ_002569 | ENSSSCG00000027935 | 6 | + | 1.2E+08 | 1.2E+08 | 642 | annot_exons |
| novel_circ_002570 | ENSSSCG00000009230 | 8 | + | 1.34E+08 | 1.34E+08 | 9857 | exon_intron |
| novel_circ_002571 | ENSSSCG00000008799 | 8 | + | 32773630 | 32776267 | 374 | annot_exons |
| novel_circ_002572 | ENSSSCG00000033591 | 6 | - | 31835306 | 31849452 | 765 | annot_exons |
| novel_circ_002573 | ENSSSCG00000014304 | 2 | + | 1.37E+08 | 1.37E+08 | 654 | annot_exons |
| novel_circ_002574 | ENSSSCG00000015545 | 9 | - | 1.24E+08 | 1.24E+08 | 357 | one_exon |
| novel_circ_002575 | ENSSSCG00000011833 | 13 | + | 1.33E+08 | 1.33E+08 | 1358 | annot_exons |
| novel_circ_002576 | ENSSSCG00000011494 | 13 | - | 45521734 | 45522605 | 475 | annot_exons |
| novel_circ_002577 | ENSSSCG00000011103 | 10 | - | 57126906 | 57137646 | 351 | annot_exons |
| novel_circ_002578 | NA | 7 | + | 68160992 | 68189993 | 29002 | intergenic |
| novel_circ_002579 | ENSSSCG00000045164 | 13 | - | 81453088 | 81457263 | 2306 | annot_exons |
| novel_circ_002580 | ENSSSCG00000047868 | AEMK02000489.1 | + | 42224 | 42498 | 275 | intronic |
| novel_circ_002581 | ENSSSCG00000012440 | X | - | 62189339 | 62190338 | 1000 | antisense |
| novel_circ_002582 | ENSSSCG00000012411 | X | - | 58498370 | 58501518 | 326 | annot_exons |
| novel_circ_002583 | ENSSSCG00000033272 | 18 | - | 1463489 | 1466144 | 345 | annot_exons |
| novel_circ_002584 | ENSSSCG00000002315 | 7 | - | 93773489 | 93775334 | 1846 | one_exon |
| novel_circ_002585 | ENSSSCG00000008971 | 8 | + | 71436665 | 71437764 | 412 | annot_exons |
| novel_circ_002586 | ENSSSCG00000017139 | 12 | - | 1853295 | 1922863 | 492 | annot_exons |
| novel_circ_002587 | ENSSSCG00000010358 | 14 | - | 87661150 | 87682623 | 21474 | antisense |
| novel_circ_002588 | ENSSSCG00000016850 | 16 | - | 23729762 | 23745763 | 3455 | annot_exons |
| novel_circ_002589 | ENSSSCG00000004242 | 1 | + | 41180826 | 41207401 | 307 | annot_exons |
| novel_circ_002590 | ENSSSCG00000008853 | 8 | - | 42908218 | 42941575 | 607 | annot_exons |
| novel_circ_002591 | ENSSSCG00000003627 | 6 | + | 91554750 | 91561270 | 1079 | annot_exons |
| novel_circ_002592 | ENSSSCG00000016156 | 15 | - | 1.13E+08 | 1.13E+08 | 459 | annot_exons |
| novel_circ_002593 | ENSSSCG00000015097 | 9 | - | 46032547 | 46044924 | 777 | annot_exons |
| novel_circ_002594 | ENSSSCG00000011477 | 13 | - | 40239099 | 40245179 | 640 | annot_exons |
| novel_circ_002595 | ENSSSCG00000008940 | 8 | - | 67590094 | 67590336 | 243 | one_exon |
| novel_circ_002596 | ENSSSCG00000021784 | 8 | + | 1.16E+08 | 1.16E+08 | 715 | annot_exons |
| novel_circ_002597 | ENSSSCG00000008909 | 8 | - | 54821981 | 54826397 | 354 | annot_exons |
| novel_circ_002598 | ENSSSCG00000023585 | 6 | + | 88003870 | 88006722 | 571 | annot_exons |
| novel_circ_002599 | ENSSSCG00000003591 | 6 | - | 87566204 | 87570047 | 527 | annot_exons |
| novel_circ_002600 | ENSSSCG00000022895 | 13 | - | 1.62E+08 | 1.62E+08 | 543 | annot_exons |
| novel_circ_002601 | ENSSSCG00000009957 | 14 | - | 43685015 | 43685256 | 242 | antisense |
| novel_circ_002602 | ENSSSCG00000021208 | 16 | - | 27585782 | 27631128 | 41070 | exon_intron |
| novel_circ_002603 | ENSSSCG00000032377 | 7 | + | 64279287 | 64287500 | 388 | annot_exons |
| novel_circ_002604 | NA | 15 | + | 84290821 | 84293413 | 2593 | intergenic |
| novel_circ_002605 | ENSSSCG00000003439 | 6 | - | 72559637 | 72565387 | 485 | annot_exons |
| novel_circ_002606 | ENSSSCG00000007896 | 3 | + | 31385093 | 31394045 | 445 | annot_exons |
| novel_circ_002607 | ENSSSCG00000016986 | 16 | - | 51181073 | 51183322 | 330 | exon_intron |
| novel_circ_002608 | ENSSSCG00000013223 | 2 | + | 14885418 | 14887371 | 268 | annot_exons |
| novel_circ_002609 | ENSSSCG00000006841 | 4 | + | 1.11E+08 | 1.11E+08 | 363 | annot_exons |
| novel_circ_002610 | ENSSSCG00000008068 | 3 | - | 41961399 | 41962459 | 273 | annot_exons |
| novel_circ_002611 | ENSSSCG00000032029 | AEMK02000569.1 | - | 146622 | 147613 | 992 | exon_intron |
| novel_circ_002612 | ENSSSCG00000005382 | 1 | + | 2.41E+08 | 2.41E+08 | 6569 | exon_intron |
| novel_circ_002613 | ENSSSCG00000035571 | 2 | + | 96395591 | 96402476 | 6886 | intronic |
| novel_circ_002614 | ENSSSCG00000034386 | 14 | + | 31685025 | 31693023 | 244 | annot_exons |
| novel_circ_002615 | ENSSSCG00000016836 | 16 | - | 21524717 | 21540786 | 560 | annot_exons |
| novel_circ_002616 | NA | 15 | - | 84362812 | 84363099 | 288 | intergenic |
| novel_circ_002617 | ENSSSCG00000006071 | 4 | - | 37426857 | 37443006 | 1017 | annot_exons |
| novel_circ_002618 | ENSSSCG00000008600 | 3 | + | 1.18E+08 | 1.18E+08 | 2170 | annot_exons |
| novel_circ_002619 | ENSSSCG00000025406 | 2 | + | 42003958 | 42006969 | 267 | annot_exons |
| novel_circ_002620 | ENSSSCG00000008821 | 8 | + | 38282480 | 38287382 | 660 | annot_exons |
| novel_circ_002621 | ENSSSCG00000003870 | 6 | + | 1.6E+08 | 1.6E+08 | 1072 | exon_intron |
| novel_circ_002622 | ENSSSCG00000006981 | 17 | + | 4765508 | 4787975 | 396 | annot_exons |
| novel_circ_002623 | ENSSSCG00000027444 | 17 | - | 50811425 | 50862418 | 2068 | annot_exons |
| novel_circ_002624 | ENSSSCG00000006760 | 4 | - | 1.07E+08 | 1.07E+08 | 555 | annot_exons |
| novel_circ_002625 | ENSSSCG00000042710 | 4 | + | 89111117 | 89111486 | 370 | one_exon |
| novel_circ_002626 | ENSSSCG00000008444 | 3 | - | 94711378 | 94711717 | 340 | intronic |
| novel_circ_002627 | ENSSSCG00000010956 | 10 | - | 29227447 | 29235132 | 648 | annot_exons |
| novel_circ_002628 | ENSSSCG00000005590 | 1 | + | 2.65E+08 | 2.65E+08 | 12339 | antisense |
| novel_circ_002629 | ENSSSCG00000009464 | 11 | - | 47549072 | 47558805 | 507 | annot_exons |
| novel_circ_002630 | ENSSSCG00000036790 | 1 | - | 32316914 | 32354628 | 570 | annot_exons |
| novel_circ_002631 | ENSSSCG00000010258 | 14 | - | 73064383 | 73065278 | 213 | annot_exons |
| novel_circ_002632 | ENSSSCG00000015886 | 15 | + | 67173512 | 67174025 | 514 | antisense |
| novel_circ_002633 | ENSSSCG00000010190 | 14 | + | 60415362 | 60415663 | 302 | one_exon |
| novel_circ_002634 | ENSSSCG00000016301 | 15 | + | 1.34E+08 | 1.34E+08 | 510 | annot_exons |
| novel_circ_002635 | ENSSSCG00000051610 | 18 | + | 17855522 | 17912006 | 501 | annot_exons |
| novel_circ_002636 | ENSSSCG00000013895 | 2 | - | 59680628 | 59681693 | 627 | annot_exons |
| novel_circ_002637 | ENSSSCG00000022616 | 13 | - | 35399558 | 35402476 | 819 | annot_exons |
| novel_circ_002638 | ENSSSCG00000016272 | 15 | + | 1.32E+08 | 1.32E+08 | 1507 | annot_exons |
| novel_circ_002639 | ENSSSCG00000009208 | 8 | - | 1.3E+08 | 1.3E+08 | 392 | annot_exons |
| novel_circ_002640 | ENSSSCG00000009652 | 14 | - | 9478895 | 9480356 | 314 | annot_exons |
| novel_circ_002641 | ENSSSCG00000003770 | 6 | - | 1.35E+08 | 1.35E+08 | 700 | annot_exons |
| novel_circ_002642 | ENSSSCG00000026960 | 1 | + | 1.15E+08 | 1.15E+08 | 605 | annot_exons |
| novel_circ_002643 | ENSSSCG00000020879 | 8 | + | 76509953 | 76521376 | 11424 | intronic |
| novel_circ_002644 | ENSSSCG00000007478 | 17 | - | 52996471 | 52998000 | 256 | annot_exons |
| novel_circ_002645 | ENSSSCG00000026264 | 6 | + | 1.58E+08 | 1.58E+08 | 4681 | annot_exons |
| novel_circ_002646 | ENSSSCG00000032048 | 14 | + | 16635007 | 16636099 | 1093 | intronic |
| novel_circ_002647 | ENSSSCG00000035619 | 9 | + | 20809498 | 20812474 | 221 | annot_exons |
| novel_circ_002648 | NA | 15 | - | 84269116 | 84269597 | 482 | intergenic |
| novel_circ_002649 | ENSSSCG00000002032 | 7 | + | 75907142 | 75910611 | 382 | annot_exons |
| novel_circ_002650 | ENSSSCG00000005444 | 1 | - | 2.5E+08 | 2.5E+08 | 471 | annot_exons |
| novel_circ_002651 | ENSSSCG00000030653 | 2 | + | 9104339 | 9104824 | 237 | annot_exons |
| novel_circ_002652 | ENSSSCG00000027613 | 1 | + | 39431308 | 39431480 | 55 | annot_exons |
| novel_circ_002653 | ENSSSCG00000011501 | 13 | - | 48930998 | 48960285 | 836 | annot_exons |
| novel_circ_002654 | ENSSSCG00000015857 | 15 | - | 59244574 | 59250682 | 419 | annot_exons |
| novel_circ_002655 | ENSSSCG00000003627 | 6 | + | 91537803 | 91605991 | 4227 | annot_exons |
| novel_circ_002656 | ENSSSCG00000038825 | 6 | + | 56533770 | 56551065 | 4319 | annot_exons |
| novel_circ_002657 | ENSSSCG00000010151 | 14 | + | 55549471 | 55553080 | 348 | annot_exons |
| novel_circ_002658 | ENSSSCG00000005085 | 1 | - | 1.89E+08 | 1.89E+08 | 291 | annot_exons |
| novel_circ_002659 | ENSSSCG00000003971 | 6 | + | 1.7E+08 | 1.7E+08 | 360 | annot_exons |
| novel_circ_002660 | ENSSSCG00000036363 | 2 | + | 59592470 | 59595767 | 278 | annot_exons |
| novel_circ_002661 | ENSSSCG00000005090 | 1 | + | 1.9E+08 | 1.9E+08 | 389 | annot_exons |
| novel_circ_002662 | ENSSSCG00000046408 | X | - | 29492001 | 29495890 | 3890 | intronic |
| novel_circ_002663 | ENSSSCG00000016834 | 16 | - | 21446906 | 21451895 | 599 | annot_exons |
| novel_circ_002664 | ENSSSCG00000036948 | 10 | + | 23646107 | 23646381 | 275 | intronic |
| novel_circ_002665 | ENSSSCG00000028748 | 6 | + | 1.58E+08 | 1.58E+08 | 423 | annot_exons |
| novel_circ_002666 | ENSSSCG00000013630 | 2 | + | 69564515 | 69565336 | 284 | annot_exons |
| novel_circ_002667 | ENSSSCG00000012591 | X | - | 92616983 | 92623219 | 761 | annot_exons |
| novel_circ_002668 | ENSSSCG00000014399 | 2 | + | 1.45E+08 | 1.45E+08 | 450 | annot_exons |
| novel_circ_002669 | ENSSSCG00000028724 | 13 | + | 1.92E+08 | 1.92E+08 | 262 | annot_exons |
| novel_circ_002670 | ENSSSCG00000008581 | 3 | - | 1.14E+08 | 1.14E+08 | 693 | annot_exons |
| novel_circ_002671 | ENSSSCG00000008943 | 8 | + | 68022433 | 68025325 | 2893 | exon_intron |
| novel_circ_002672 | ENSSSCG00000013054 | 2 | + | 8388248 | 8390219 | 289 | annot_exons |
| novel_circ_002673 | ENSSSCG00000020858 | 7 | - | 13562827 | 13564499 | 246 | annot_exons |
| novel_circ_002674 | ENSSSCG00000000510 | 5 | + | 35771132 | 35780762 | 316 | annot_exons |
| novel_circ_002675 | NA | 15 | + | 84267623 | 84267815 | 193 | intergenic |
| novel_circ_002676 | ENSSSCG00000013858 | 2 | + | 61110209 | 61134150 | 990 | annot_exons |
| novel_circ_002677 | ENSSSCG00000017094 | 16 | - | 72057304 | 72059836 | 317 | annot_exons |
| novel_circ_002678 | ENSSSCG00000026644 | 2 | + | 1.37E+08 | 1.37E+08 | 522 | annot_exons |
| novel_circ_002679 | ENSSSCG00000008989 | 8 | - | 73258405 | 73262907 | 395 | annot_exons |
| novel_circ_002680 | ENSSSCG00000035495 | 5 | + | 94082004 | 94084942 | 337 | annot_exons |
| novel_circ_002681 | ENSSSCG00000023955 | 12 | + | 34980903 | 34998939 | 433 | annot_exons |
| novel_circ_002682 | ENSSSCG00000002259 | 7 | + | 88150859 | 88172966 | 647 | annot_exons |
| novel_circ_002683 | ENSSSCG00000026596 | 12 | - | 38992252 | 38998122 | 464 | annot_exons |
| novel_circ_002684 | ENSSSCG00000007034 | 17 | + | 13008092 | 13012850 | 448 | annot_exons |
| novel_circ_002685 | ENSSSCG00000006074 | 4 | + | 38110196 | 38111049 | 333 | annot_exons |
| novel_circ_002686 | ENSSSCG00000009408 | 11 | - | 20713782 | 20719541 | 248 | annot_exons |
| novel_circ_002687 | ENSSSCG00000016549 | 18 | - | 17612336 | 17613850 | 232 | annot_exons |
| novel_circ_002688 | ENSSSCG00000038469 | 9 | + | 91931590 | 91944532 | 494 | annot_exons |
| novel_circ_002689 | ENSSSCG00000005366 | 1 | + | 2.39E+08 | 2.39E+08 | 1046 | annot_exons |
| novel_circ_002690 | ENSSSCG00000004558 | 1 | + | 1.08E+08 | 1.08E+08 | 401 | one_exon |
| novel_circ_002691 | ENSSSCG00000042814 | 1 | + | 1.8E+08 | 1.8E+08 | 293 | annot_exons |
| novel_circ_002692 | ENSSSCG00000030396 | 8 | + | 87502698 | 87502887 | 190 | one_exon |
| novel_circ_002693 | ENSSSCG00000028148 | X | - | 28437952 | 28461112 | 795 | annot_exons |
| novel_circ_002694 | ENSSSCG00000012411 | X | + | 58457241 | 58457421 | 181 | antisense |
| novel_circ_002695 | ENSSSCG00000016961 | 16 | + | 47423320 | 47435032 | 1461 | annot_exons |
| novel_circ_002696 | ENSSSCG00000036835 | 17 | + | 61668651 | 61672194 | 416 | annot_exons |
| novel_circ_002697 | ENSSSCG00000015391 | 9 | - | 93284772 | 93287079 | 322 | annot_exons |
| novel_circ_002698 | ENSSSCG00000009828 | 14 | - | 32073134 | 32079986 | 1033 | annot_exons |
| novel_circ_002699 | ENSSSCG00000030095 | 9 | + | 41724766 | 41738352 | 13587 | exon_intron |
| novel_circ_002700 | ENSSSCG00000006137 | 4 | - | 50402450 | 50416871 | 793 | annot_exons |
| novel_circ_002701 | ENSSSCG00000029633 | 6 | + | 1.57E+08 | 1.57E+08 | 1758 | annot_exons |
| novel_circ_002702 | ENSSSCG00000002779 | 6 | + | 27992241 | 28037996 | 2909 | annot_exons |
| novel_circ_002703 | ENSSSCG00000004082 | 1 | + | 14186645 | 14188834 | 342 | annot_exons |
| novel_circ_002704 | ENSSSCG00000006865 | 4 | - | 1.18E+08 | 1.18E+08 | 279 | annot_exons |
| novel_circ_002705 | ENSSSCG00000003877 | 6 | + | 1.61E+08 | 1.61E+08 | 253 | annot_exons |
| novel_circ_002706 | ENSSSCG00000016053 | 15 | - | 95122041 | 95124354 | 399 | annot_exons |
| novel_circ_002707 | ENSSSCG00000016510 | 18 | - | 10416117 | 10420695 | 732 | annot_exons |
| novel_circ_002708 | ENSSSCG00000008808 | 8 | - | 35142540 | 35142685 | 146 | one_exon |
| novel_circ_002709 | ENSSSCG00000008079 | 3 | + | 42881922 | 42882168 | 247 | one_exon |
| novel_circ_002710 | ENSSSCG00000010325 | 14 | + | 80096815 | 80103314 | 6500 | antisense |
| novel_circ_002711 | ENSSSCG00000002523 | AEMK02000452.1 | - | 1705711 | 1713483 | 272 | annot_exons |
| novel_circ_002712 | ENSSSCG00000033763 | 1 | - | 2.31E+08 | 2.31E+08 | 259 | annot_exons |
| novel_circ_002713 | ENSSSCG00000003520 | 6 | + | 80068941 | 80087974 | 1961 | annot_exons |
| novel_circ_002714 | ENSSSCG00000001641 | 7 | + | 37692015 | 37701933 | 324 | annot_exons |
| novel_circ_002715 | ENSSSCG00000010470 | 14 | - | 1.04E+08 | 1.04E+08 | 226 | annot_exons |
| novel_circ_002716 | NA | 15 | - | 84312684 | 84312880 | 197 | intergenic |
| novel_circ_002717 | ENSSSCG00000015631 | 9 | - | 1.37E+08 | 1.37E+08 | 340 | annot_exons |
| novel_circ_002718 | ENSSSCG00000047432 | 2 | + | 6750385 | 6751423 | 974 | exon_intron |
| novel_circ_002719 | ENSSSCG00000027074 | 2 | + | 1.14E+08 | 1.14E+08 | 420 | annot_exons |
| novel_circ_002720 | ENSSSCG00000026596 | 12 | - | 39037825 | 39039068 | 237 | annot_exons |
| novel_circ_002721 | ENSSSCG00000015714 | 15 | - | 23576644 | 23588674 | 477 | annot_exons |
| novel_circ_002722 | ENSSSCG00000011013 | 10 | + | 39935261 | 39935456 | 196 | one_exon |
| novel_circ_002723 | ENSSSCG00000006303 | 4 | - | 82993955 | 83008862 | 679 | annot_exons |
| novel_circ_002724 | ENSSSCG00000016510 | 18 | - | 10402043 | 10402935 | 233 | annot_exons |
| novel_circ_002725 | ENSSSCG00000016769 | 18 | - | 54324875 | 54325859 | 249 | annot_exons |
| novel_circ_002726 | ENSSSCG00000006779 | 4 | + | 1.08E+08 | 1.08E+08 | 410 | annot_exons |
| novel_circ_002727 | ENSSSCG00000023276 | 6 | - | 1.19E+08 | 1.19E+08 | 249 | one_exon |
| novel_circ_002728 | ENSSSCG00000003591 | 6 | - | 87558922 | 87570047 | 919 | annot_exons |
| novel_circ_002729 | ENSSSCG00000006136 | 4 | + | 50362201 | 50370969 | 386 | annot_exons |
| novel_circ_002730 | ENSSSCG00000005170 | 1 | - | 2.04E+08 | 2.04E+08 | 322 | one_exon |
| novel_circ_002731 | ENSSSCG00000000035 | 5 | + | 5808982 | 5812316 | 525 | annot_exons |
| novel_circ_002732 | ENSSSCG00000008980 | 8 | - | 71887808 | 71887965 | 158 | one_exon |
| novel_circ_002733 | ENSSSCG00000016501 | 18 | + | 9651790 | 9652810 | 247 | annot_exons |
| novel_circ_002734 | ENSSSCG00000017593 | 12 | + | 27528864 | 27538247 | 369 | annot_exons |
| novel_circ_002735 | ENSSSCG00000016606 | 18 | + | 23852444 | 23853454 | 1011 | antisense |
| novel_circ_002736 | ENSSSCG00000015499 | 9 | + | 1.17E+08 | 1.17E+08 | 245 | annot_exons |
| novel_circ_002737 | ENSSSCG00000028148 | X | - | 27723964 | 27821717 | 609 | annot_exons |
| novel_circ_002738 | ENSSSCG00000010190 | 14 | - | 60415050 | 60415661 | 612 | antisense |
| novel_circ_002739 | ENSSSCG00000031981 | 10 | - | 24161051 | 24161381 | 331 | antisense |
| novel_circ_002740 | ENSSSCG00000014949 | 9 | + | 26197373 | 26200095 | 595 | annot_exons |
| novel_circ_002741 | ENSSSCG00000009215 | 8 | + | 1.31E+08 | 1.31E+08 | 361 | one_exon |
| novel_circ_002742 | ENSSSCG00000012251 | X | + | 36862369 | 36863881 | 1513 | exon_intron |
| novel_circ_002743 | ENSSSCG00000001971 | 7 | + | 68389520 | 68402796 | 1817 | annot_exons |
| novel_circ_002744 | ENSSSCG00000017043 | 16 | + | 64300483 | 64307672 | 331 | annot_exons |
| novel_circ_002745 | ENSSSCG00000001880 | 7 | + | 58196071 | 58208812 | 1131 | annot_exons |
| novel_circ_002746 | ENSSSCG00000014044 | 2 | - | 80702172 | 80715319 | 845 | annot_exons |
| novel_circ_002747 | ENSSSCG00000024481 | 4 | - | 1.01E+08 | 1.01E+08 | 468 | annot_exons |
| novel_circ_002748 | ENSSSCG00000015692 | 15 | - | 16241111 | 16244435 | 274 | annot_exons |
| novel_circ_002749 | ENSSSCG00000038505 | 12 | + | 33965871 | 33966079 | 209 | one_exon |
| novel_circ_002750 | ENSSSCG00000005996 | 4 | + | 18633885 | 18641189 | 209 | annot_exons |
| novel_circ_002751 | ENSSSCG00000028148 | X | - | 27410214 | 27410482 | 269 | intronic |
| novel_circ_002752 | ENSSSCG00000006135 | 4 | - | 50283248 | 50285126 | 371 | annot_exons |
| novel_circ_002753 | ENSSSCG00000001076 | 7 | + | 14126030 | 14206695 | 39034 | exon_intron |
| novel_circ_002754 | ENSSSCG00000037355 | 4 | + | 99522014 | 99522219 | 206 | intronic |
| novel_circ_002755 | ENSSSCG00000002333 | 7 | - | 96042175 | 96061879 | 267 | annot_exons |
| novel_circ_002756 | ENSSSCG00000040887 | 6 | - | 34725091 | 34747797 | 977 | annot_exons |
| novel_circ_002757 | ENSSSCG00000027738 | 13 | + | 21697536 | 21697679 | 144 | one_exon |
| novel_circ_002758 | ENSSSCG00000007576 | 3 | + | 3610346 | 3614777 | 1256 | annot_exons |
| novel_circ_002759 | ENSSSCG00000010563 | 14 | + | 1.13E+08 | 1.13E+08 | 257 | annot_exons |
| novel_circ_002760 | ENSSSCG00000047585 | 13 | + | 46314093 | 46314698 | 606 | antisense |
| novel_circ_002761 | ENSSSCG00000015842 | 15 | + | 54310541 | 54312703 | 384 | annot_exons |
| novel_circ_002762 | ENSSSCG00000001074 | 7 | + | 13873685 | 13886691 | 1007 | annot_exons |
| novel_circ_002763 | ENSSSCG00000010548 | 14 | - | 1.11E+08 | 1.11E+08 | 828 | annot_exons |
| novel_circ_002764 | ENSSSCG00000003790 | 6 | + | 1.43E+08 | 1.43E+08 | 2198 | exon_intron |
| novel_circ_002765 | ENSSSCG00000014794 | 9 | + | 6409665 | 6414461 | 509 | annot_exons |
| novel_circ_002766 | ENSSSCG00000026761 | 13 | - | 1.92E+08 | 1.92E+08 | 357 | annot_exons |
| novel_circ_002767 | ENSSSCG00000001850 | 7 | - | 55438549 | 55458259 | 276 | annot_exons |
| novel_circ_002768 | NA | 15 | + | 84234711 | 84234929 | 219 | intergenic |
| novel_circ_002769 | ENSSSCG00000014114 | 2 | + | 88358554 | 88372391 | 457 | annot_exons |
| novel_circ_002770 | ENSSSCG00000046809 | 6 | + | 47348521 | 47369005 | 20313 | exon_intron |
| novel_circ_002771 | ENSSSCG00000040887 | 6 | - | 34714334 | 34725179 | 410 | annot_exons |
| novel_circ_002772 | ENSSSCG00000004394 | 1 | + | 76967217 | 76980603 | 444 | annot_exons |
| novel_circ_002773 | ENSSSCG00000029441 | 12 | - | 55276792 | 55277482 | 229 | annot_exons |
| novel_circ_002774 | ENSSSCG00000006274 | 4 | + | 79784917 | 79788607 | 548 | annot_exons |
| novel_circ_002775 | ENSSSCG00000021440 | 4 | - | 1.11E+08 | 1.11E+08 | 375 | annot_exons |
| novel_circ_002776 | ENSSSCG00000022190 | 4 | - | 79424581 | 79425276 | 443 | annot_exons |
| novel_circ_002777 | ENSSSCG00000005719 | 1 | - | 2.72E+08 | 2.72E+08 | 336 | annot_exons |
| novel_circ_002778 | ENSSSCG00000015976 | 15 | - | 81796259 | 81799621 | 319 | annot_exons |
| novel_circ_002779 | ENSSSCG00000048207 | 13 | + | 1.81E+08 | 1.81E+08 | 246 | annot_exons |
| novel_circ_002780 | ENSSSCG00000023247 | 10 | - | 48549518 | 48551873 | 360 | annot_exons |
| novel_circ_002781 | ENSSSCG00000007264 | 17 | - | 36894455 | 36899810 | 563 | annot_exons |
| novel_circ_002782 | ENSSSCG00000002869 | 6 | + | 43867657 | 43887734 | 660 | annot_exons |
| novel_circ_002783 | ENSSSCG00000004293 | 1 | - | 54494181 | 54502778 | 500 | annot_exons |
| novel_circ_002784 | ENSSSCG00000002872 | 6 | + | 44006567 | 44008365 | 558 | annot_exons |
| novel_circ_002785 | ENSSSCG00000016192 | 15 | - | 1.21E+08 | 1.21E+08 | 33832 | antisense |
| novel_circ_002786 | NA | 14 | - | 41634910 | 41636993 | 2084 | intergenic |
| novel_circ_002787 | ENSSSCG00000011714 | 13 | + | 91368437 | 91378095 | 756 | annot_exons |
| novel_circ_002788 | ENSSSCG00000010811 | 10 | + | 5761746 | 5777849 | 637 | annot_exons |
| novel_circ_002789 | ENSSSCG00000013082 | 2 | - | 10075620 | 10082057 | 401 | annot_exons |
| novel_circ_002790 | ENSSSCG00000003401 | 6 | + | 70511058 | 70511227 | 170 | one_exon |
| novel_circ_002791 | ENSSSCG00000005135 | 1 | - | 2.01E+08 | 2.01E+08 | 314 | annot_exons |
| novel_circ_002792 | ENSSSCG00000008881 | 8 | + | 48237988 | 48239158 | 296 | annot_exons |
| novel_circ_002793 | NA | 15 | + | 84456259 | 84456441 | 183 | intergenic |
| novel_circ_002794 | ENSSSCG00000035934 | 1 | - | 1.82E+08 | 1.82E+08 | 229 | annot_exons |
| novel_circ_002795 | ENSSSCG00000026665 | 12 | - | 37534103 | 37534314 | 212 | intronic |
| novel_circ_002796 | ENSSSCG00000023728 | 9 | - | 48282059 | 48282998 | 940 | antisense |
| novel_circ_002797 | ENSSSCG00000010862 | 10 | - | 14786194 | 14794270 | 697 | annot_exons |
| novel_circ_002798 | ENSSSCG00000009015 | 8 | + | 77265267 | 77286040 | 1048 | annot_exons |
| novel_circ_002799 | ENSSSCG00000002298 | 7 | - | 91547494 | 91548524 | 346 | annot_exons |
| novel_circ_002800 | ENSSSCG00000023993 | 13 | - | 1.48E+08 | 1.48E+08 | 544 | annot_exons |
| novel_circ_002801 | ENSSSCG00000029464 | 5 | - | 67653816 | 67655863 | 342 | annot_exons |
| novel_circ_002802 | ENSSSCG00000006508 | 4 | + | 94391005 | 94406163 | 922 | annot_exons |
| novel_circ_002803 | ENSSSCG00000004595 | 1 | + | 1.13E+08 | 1.13E+08 | 519 | annot_exons |
| novel_circ_002804 | ENSSSCG00000008499 | 3 | + | 1.03E+08 | 1.03E+08 | 615 | annot_exons |
| novel_circ_002805 | ENSSSCG00000004499 | 1 | + | 95725367 | 95726393 | 1027 | antisense |
| novel_circ_002806 | ENSSSCG00000009007 | 8 | - | 75503374 | 75507182 | 1146 | annot_exons |
| novel_circ_002807 | ENSSSCG00000029198 | 9 | + | 36475680 | 36481531 | 398 | annot_exons |
| novel_circ_002808 | ENSSSCG00000029805 | 2 | + | 1.02E+08 | 1.02E+08 | 366 | one_exon |
| novel_circ_002809 | ENSSSCG00000006845 | 4 | - | 1.11E+08 | 1.11E+08 | 1102 | annot_exons |
| novel_circ_002810 | NA | 15 | - | 84276567 | 84276840 | 274 | intergenic |
| novel_circ_002811 | ENSSSCG00000001056 | 7 | - | 9980131 | 10010669 | 1373 | annot_exons |
| novel_circ_002812 | ENSSSCG00000015743 | 15 | - | 31467433 | 31473618 | 6186 | exon_intron |
| novel_circ_002813 | ENSSSCG00000007280 | 17 | + | 37890389 | 37912170 | 831 | annot_exons |
| novel_circ_002814 | ENSSSCG00000016501 | 18 | + | 9647842 | 9652810 | 737 | annot_exons |
| novel_circ_002815 | NA | 8 | + | 9287995 | 9305525 | 17531 | intergenic |
| novel_circ_002816 | ENSSSCG00000014304 | 2 | + | 1.37E+08 | 1.37E+08 | 686 | annot_exons |
| novel_circ_002817 | ENSSSCG00000005979 | 4 | - | 15730234 | 15734771 | 4538 | antisense |
| novel_circ_002818 | ENSSSCG00000010987 | 10 | + | 32862723 | 32868451 | 662 | annot_exons |
| novel_circ_002819 | ENSSSCG00000001499 | 7 | + | 29151456 | 29159867 | 6207 | annot_exons |
| novel_circ_002820 | ENSSSCG00000014057 | 2 | - | 81414130 | 81431978 | 948 | annot_exons |
| novel_circ_002821 | NA | 2 | - | 1.34E+08 | 1.34E+08 | 1623 | intergenic |
| novel_circ_002822 | ENSSSCG00000006841 | 4 | + | 1.11E+08 | 1.11E+08 | 711 | annot_exons |
| novel_circ_002823 | ENSSSCG00000017248 | 12 | - | 7756469 | 7757319 | 598 | annot_exons |
| novel_circ_002824 | ENSSSCG00000007342 | 17 | + | 41119108 | 41127499 | 416 | annot_exons |
| novel_circ_002825 | ENSSSCG00000012836 | 2 | + | 594391 | 600366 | 391 | annot_exons |
| novel_circ_002826 | ENSSSCG00000051610 | 18 | + | 17883391 | 17889795 | 6405 | exon_intron |
| novel_circ_002827 | ENSSSCG00000016397 | 15 | - | 765831 | 766121 | 291 | antisense |
| novel_circ_002828 | ENSSSCG00000016725 | 18 | - | 49121618 | 49121842 | 225 | antisense |
| novel_circ_002829 | ENSSSCG00000045213 | 11 | + | 17760988 | 17781703 | 12978 | exon_intron |
| novel_circ_002830 | ENSSSCG00000016618 | 18 | - | 25873363 | 25884224 | 396 | annot_exons |
| novel_circ_002831 | ENSSSCG00000041410 | 7 | - | 23022971 | 23026682 | 3712 | exon_intron |
| novel_circ_002832 | ENSSSCG00000004281 | 1 | + | 52753551 | 52800752 | 11136 | exon_intron |
| novel_circ_002833 | ENSSSCG00000050072 | 5 | + | 89882333 | 89884309 | 1977 | exon_intron |
| novel_circ_002834 | ENSSSCG00000010605 | 14 | - | 1.15E+08 | 1.15E+08 | 454 | annot_exons |
| novel_circ_002835 | ENSSSCG00000001485 | 7 | - | 26689737 | 26719989 | 20619 | exon_intron |
| novel_circ_002836 | ENSSSCG00000004951 | 1 | - | 1.65E+08 | 1.65E+08 | 540 | annot_exons |
| novel_circ_002837 | ENSSSCG00000008498 | 3 | + | 1.03E+08 | 1.03E+08 | 1068 | annot_exons |
| novel_circ_002838 | ENSSSCG00000005036 | 1 | + | 1.82E+08 | 1.82E+08 | 273 | annot_exons |
| novel_circ_002839 | ENSSSCG00000003863 | 6 | - | 1.6E+08 | 1.6E+08 | 847 | annot_exons |
| novel_circ_002840 | ENSSSCG00000006760 | 4 | - | 1.07E+08 | 1.07E+08 | 326 | annot_exons |
| novel_circ_002841 | ENSSSCG00000029482 | 14 | - | 1.33E+08 | 1.34E+08 | 2082 | annot_exons |
| novel_circ_002842 | NA | 15 | - | 84238664 | 84238957 | 294 | intergenic |
| novel_circ_002843 | ENSSSCG00000014113 | 2 | - | 88184110 | 88201577 | 365 | annot_exons |
| novel_circ_002844 | NA | 15 | - | 84466784 | 84467730 | 947 | intergenic |
| novel_circ_002845 | ENSSSCG00000009480 | 11 | + | 51531489 | 51554792 | 3785 | annot_exons |
| novel_circ_002846 | ENSSSCG00000037372 | 1 | - | 14814 | 18696 | 608 | annot_exons |
| novel_circ_002847 | ENSSSCG00000021380 | 13 | + | 1.34E+08 | 1.34E+08 | 366 | annot_exons |
| novel_circ_002848 | ENSSSCG00000003591 | 6 | - | 87628833 | 87630079 | 285 | annot_exons |
| novel_circ_002849 | ENSSSCG00000012437 | X | + | 62057443 | 62062319 | 371 | annot_exons |
| novel_circ_002850 | ENSSSCG00000026996 | 11 | - | 64126186 | 64133950 | 348 | annot_exons |
| novel_circ_002851 | ENSSSCG00000009319 | 11 | + | 5577247 | 5588249 | 849 | annot_exons |
| novel_circ_002852 | ENSSSCG00000025281 | 3 | + | 26724542 | 26726624 | 378 | annot_exons |
| novel_circ_002853 | ENSSSCG00000036812 | 12 | - | 61151270 | 61153644 | 144 | annot_exons |
| novel_circ_002854 | ENSSSCG00000036909 | 6 | + | 1.67E+08 | 1.67E+08 | 269 | annot_exons |
| novel_circ_002855 | ENSSSCG00000007815 | 3 | + | 19421276 | 19427247 | 495 | annot_exons |
| novel_circ_002856 | ENSSSCG00000017094 | 16 | - | 72065603 | 72067344 | 343 | annot_exons |
| novel_circ_002857 | ENSSSCG00000027738 | 13 | - | 21735003 | 21735287 | 285 | antisense |
| novel_circ_002858 | ENSSSCG00000037663 | 13 | + | 2.06E+08 | 2.06E+08 | 1174 | annot_exons |
| novel_circ_002859 | NA | 15 | - | 84253685 | 84253883 | 199 | intergenic |
| novel_circ_002860 | ENSSSCG00000008486 | 3 | - | 1.02E+08 | 1.02E+08 | 1225 | annot_exons |
| novel_circ_002861 | ENSSSCG00000010741 | 14 | + | 1.35E+08 | 1.35E+08 | 270 | annot_exons |
| novel_circ_002862 | ENSSSCG00000018049 | 12 | - | 60348042 | 60370222 | 712 | annot_exons |
| novel_circ_002863 | ENSSSCG00000016194 | 15 | - | 1.21E+08 | 1.21E+08 | 460 | annot_exons |
| novel_circ_002864 | ENSSSCG00000006872 | 4 | - | 1.18E+08 | 1.18E+08 | 1751 | annot_exons |
| novel_circ_002865 | ENSSSCG00000005607 | 1 | + | 2.68E+08 | 2.68E+08 | 278 | annot_exons |
| novel_circ_002866 | ENSSSCG00000016045 | 15 | + | 94445128 | 94446961 | 1133 | annot_exons |
| novel_circ_002867 | ENSSSCG00000015886 | 15 | - | 67107399 | 67116547 | 618 | annot_exons |
| novel_circ_002868 | ENSSSCG00000029967 | 9 | - | 72411512 | 72424220 | 913 | annot_exons |
| novel_circ_002869 | ENSSSCG00000005373 | 1 | + | 2.4E+08 | 2.4E+08 | 471 | annot_exons |
| novel_circ_002870 | ENSSSCG00000013308 | 2 | + | 27150884 | 27163942 | 450 | annot_exons |
| novel_circ_002871 | ENSSSCG00000006071 | 4 | - | 37460268 | 37539512 | 1046 | annot_exons |
| novel_circ_002872 | ENSSSCG00000007311 | 17 | + | 39197685 | 39213719 | 543 | annot_exons |
| novel_circ_002873 | ENSSSCG00000015523 | 9 | + | 1.21E+08 | 1.21E+08 | 301 | annot_exons |
| novel_circ_002874 | ENSSSCG00000015052 | 9 | - | 41427170 | 41427364 | 195 | one_exon |
| novel_circ_002875 | ENSSSCG00000027288 | 3 | + | 4755564 | 4757075 | 399 | annot_exons |
| novel_circ_002876 | ENSSSCG00000004802 | 1 | + | 1.36E+08 | 1.36E+08 | 325 | annot_exons |
| novel_circ_002877 | ENSSSCG00000023409 | 3 | + | 72250326 | 72259464 | 491 | annot_exons |
| novel_circ_002878 | ENSSSCG00000026498 | 5 | - | 22655523 | 22689131 | 309 | exon_intron |
| novel_circ_002879 | NA | 8 | - | 29995959 | 29996320 | 362 | intergenic |
| novel_circ_002880 | ENSSSCG00000016291 | 15 | + | 1.33E+08 | 1.33E+08 | 609 | annot_exons |
| novel_circ_002881 | ENSSSCG00000004938 | 1 | - | 1.64E+08 | 1.64E+08 | 411 | annot_exons |
| novel_circ_002882 | ENSSSCG00000009298 | 11 | + | 4140485 | 4143040 | 190 | annot_exons |
| novel_circ_002883 | ENSSSCG00000040595 | 14 | - | 32805427 | 32817850 | 443 | annot_exons |
| novel_circ_002884 | ENSSSCG00000021072 | 1 | - | 95571669 | 95582063 | 686 | annot_exons |
| novel_circ_002885 | ENSSSCG00000006661 | 4 | - | 98970827 | 98995745 | 938 | annot_exons |
| novel_circ_002886 | ENSSSCG00000017745 | 12 | + | 43096806 | 43097800 | 453 | annot_exons |
| novel_circ_002887 | ENSSSCG00000016545 | 18 | + | 16298400 | 16313908 | 15509 | exon_intron |
| novel_circ_002888 | ENSSSCG00000031949 | 1 | + | 1.28E+08 | 1.28E+08 | 637 | annot_exons |
| novel_circ_002889 | ENSSSCG00000000475 | 5 | + | 30554965 | 30572528 | 754 | annot_exons |
| novel_circ_002890 | ENSSSCG00000037594 | 1 | - | 66356093 | 66364270 | 601 | annot_exons |
| novel_circ_002891 | ENSSSCG00000012874 | 2 | + | 3608379 | 3609192 | 814 | exon_intron |
| novel_circ_002892 | ENSSSCG00000008098 | 3 | - | 43960849 | 43962443 | 344 | annot_exons |
| novel_circ_002893 | NA | 15 | + | 84232569 | 84232960 | 392 | intergenic |
| novel_circ_002894 | ENSSSCG00000016094 | 15 | + | 1.05E+08 | 1.05E+08 | 5200 | intronic |
| novel_circ_002895 | ENSSSCG00000030289 | 14 | - | 39273153 | 39292398 | 733 | exon_intron |
| novel_circ_002896 | ENSSSCG00000009714 | 14 | + | 20281913 | 20294491 | 345 | annot_exons |
| novel_circ_002897 | ENSSSCG00000004666 | 1 | - | 1.26E+08 | 1.26E+08 | 431 | annot_exons |
| novel_circ_002898 | ENSSSCG00000004893 | 1 | + | 1.58E+08 | 1.58E+08 | 608 | annot_exons |
| novel_circ_002899 | ENSSSCG00000034191 | 2 | + | 42672803 | 42715717 | 567 | annot_exons |
| novel_circ_002900 | ENSSSCG00000014339 | 2 | + | 1.41E+08 | 1.41E+08 | 353 | annot_exons |
| novel_circ_002901 | ENSSSCG00000010826 | 10 | + | 10169065 | 10182130 | 438 | annot_exons |
| novel_circ_002902 | ENSSSCG00000025910 | 18 | - | 33327163 | 33329926 | 244 | annot_exons |
| novel_circ_002903 | ENSSSCG00000011858 | 13 | + | 1.35E+08 | 1.35E+08 | 217 | annot_exons |
| novel_circ_002904 | ENSSSCG00000007735 | 3 | - | 16314182 | 16328043 | 734 | exon_intron |
| novel_circ_002905 | ENSSSCG00000006090 | 4 | + | 40379783 | 40390649 | 776 | annot_exons |
| novel_circ_002906 | ENSSSCG00000004415 | 1 | + | 75429568 | 75431499 | 250 | annot_exons |
| novel_circ_002907 | ENSSSCG00000033367 | 15 | - | 47648592 | 47649115 | 216 | annot_exons |
| novel_circ_002908 | ENSSSCG00000016194 | 15 | - | 1.21E+08 | 1.21E+08 | 388 | annot_exons |
| novel_circ_002909 | ENSSSCG00000007280 | 17 | + | 37831734 | 37854328 | 957 | annot_exons |
| novel_circ_002910 | ENSSSCG00000010821 | 10 | - | 9613070 | 9614246 | 345 | annot_exons |
| novel_circ_002911 | ENSSSCG00000016409 | 18 | - | 1589912 | 1604899 | 902 | annot_exons |
| novel_circ_002912 | ENSSSCG00000009828 | 14 | - | 32078738 | 32079986 | 363 | annot_exons |
| novel_circ_002913 | ENSSSCG00000034730 | 1 | - | 96483722 | 96516009 | 837 | annot_exons |
| novel_circ_002914 | ENSSSCG00000011074 | 10 | + | 50663344 | 50665836 | 542 | annot_exons |
| novel_circ_002915 | ENSSSCG00000009029 | 8 | - | 80800577 | 80827917 | 12152 | exon_intron |
| novel_circ_002916 | ENSSSCG00000014255 | 2 | + | 1.31E+08 | 1.31E+08 | 292 | annot_exons |
| novel_circ_002917 | ENSSSCG00000038437 | AEMK02000682.1 | - | 264782 | 272452 | 767 | annot_exons |
| novel_circ_002918 | ENSSSCG00000006179 | 4 | + | 62187691 | 62197767 | 10077 | exon_intron |
| novel_circ_002919 | ENSSSCG00000033497 | 6 | - | 69210845 | 69211313 | 469 | one_exon |
| novel_circ_002920 | ENSSSCG00000034290 | 7 | - | 94174729 | 94176980 | 305 | annot_exons |
| novel_circ_002921 | ENSSSCG00000002245 | 7 | + | 80344261 | 80346071 | 325 | annot_exons |
| novel_circ_002922 | ENSSSCG00000010484 | 14 | - | 1.06E+08 | 1.06E+08 | 182 | annot_exons |
| novel_circ_002923 | ENSSSCG00000030480 | 13 | + | 2.01E+08 | 2.01E+08 | 557 | one_exon |
| novel_circ_002924 | ENSSSCG00000007004 | 17 | - | 12034427 | 12041282 | 1560 | annot_exons |
| novel_circ_002925 | NA | 15 | + | 6706213 | 6711395 | 5183 | intergenic |
| novel_circ_002926 | ENSSSCG00000035676 | 16 | - | 71253682 | 71255339 | 453 | annot_exons |
| novel_circ_002927 | ENSSSCG00000014838 | 9 | - | 8695489 | 8716340 | 1295 | annot_exons |
| novel_circ_002928 | ENSSSCG00000010956 | 10 | - | 29223951 | 29230143 | 550 | annot_exons |
| novel_circ_002929 | ENSSSCG00000043503 | 13 | + | 1.21E+08 | 1.21E+08 | 572 | annot_exons |
| novel_circ_002930 | ENSSSCG00000037358 | 2 | + | 40921354 | 40925620 | 572 | annot_exons |
| novel_circ_002931 | ENSSSCG00000006661 | 4 | - | 98970827 | 98999220 | 1256 | annot_exons |
| novel_circ_002932 | ENSSSCG00000029311 | 14 | + | 71301776 | 71311455 | 1395 | annot_exons |
| novel_circ_002933 | ENSSSCG00000029837 | 11 | + | 25081807 | 25122908 | 488 | annot_exons |
| novel_circ_002934 | ENSSSCG00000037424 | 1 | - | 76649382 | 76723195 | 526 | annot_exons |
| novel_circ_002935 | ENSSSCG00000029413 | 3 | + | 1.14E+08 | 1.14E+08 | 462 | annot_exons |
| novel_circ_002936 | ENSSSCG00000028033 | 14 | + | 919880 | 920515 | 398 | annot_exons |
| novel_circ_002937 | ENSSSCG00000036537 | 2 | + | 75193549 | 75194077 | 529 | antisense |
| novel_circ_002938 | ENSSSCG00000029621 | 8 | - | 1.25E+08 | 1.25E+08 | 3740 | exon_intron |
| novel_circ_002939 | ENSSSCG00000029030 | 6 | - | 1.04E+08 | 1.04E+08 | 452 | annot_exons |
| novel_circ_002940 | ENSSSCG00000026569 | 3 | - | 32047943 | 32085104 | 861 | annot_exons |
| novel_circ_002941 | ENSSSCG00000028943 | 6 | - | 47637796 | 47638219 | 291 | annot_exons |
| novel_circ_002942 | ENSSSCG00000035818 | 13 | - | 42153536 | 42154862 | 1327 | intronic |
| novel_circ_002943 | ENSSSCG00000036052 | 15 | + | 84227691 | 84228006 | 316 | antisense |
| novel_circ_002944 | ENSSSCG00000031077 | 13 | - | 1E+08 | 1E+08 | 269 | annot_exons |
| novel_circ_002945 | ENSSSCG00000003591 | 6 | - | 87558922 | 87568427 | 651 | annot_exons |
| novel_circ_002946 | ENSSSCG00000011340 | 13 | - | 30522328 | 30524999 | 248 | annot_exons |
| novel_circ_002947 | ENSSSCG00000014394 | 2 | + | 1.44E+08 | 1.44E+08 | 343 | annot_exons |
| novel_circ_002948 | ENSSSCG00000006871 | 4 | - | 1.18E+08 | 1.18E+08 | 700 | annot_exons |
| novel_circ_002949 | ENSSSCG00000015368 | 9 | + | 87806993 | 87822303 | 668 | annot_exons |
| novel_circ_002950 | ENSSSCG00000011236 | 13 | - | 19401276 | 19415099 | 491 | annot_exons |
| novel_circ_002951 | ENSSSCG00000012642 | X | + | 1.02E+08 | 1.02E+08 | 792 | annot_exons |
| novel_circ_002952 | ENSSSCG00000012328 | X | - | 46314295 | 46315147 | 395 | annot_exons |
| novel_circ_002953 | ENSSSCG00000008943 | 8 | + | 68200357 | 68245290 | 6036 | exon_intron |
| novel_circ_002954 | ENSSSCG00000015566 | 9 | - | 1.26E+08 | 1.26E+08 | 508 | annot_exons |
| novel_circ_002955 | ENSSSCG00000014411 | 2 | - | 1.48E+08 | 1.48E+08 | 1059 | annot_exons |
| novel_circ_002956 | ENSSSCG00000002447 | 7 | + | 1.14E+08 | 1.14E+08 | 379 | annot_exons |
| novel_circ_002957 | ENSSSCG00000005777 | 6 | - | 1.28E+08 | 1.28E+08 | 174 | annot_exons |
| novel_circ_002958 | ENSSSCG00000014960 | 9 | + | 26904866 | 26923854 | 1229 | annot_exons |
| novel_circ_002959 | ENSSSCG00000034521 | 6 | + | 29344146 | 29357287 | 546 | annot_exons |
| novel_circ_002960 | ENSSSCG00000007637 | 3 | - | 7829708 | 7831227 | 327 | annot_exons |
| novel_circ_002961 | ENSSSCG00000031206 | AEMK02000258.1 | + | 266698 | 278445 | 839 | annot_exons |
| novel_circ_002962 | ENSSSCG00000011454 | 13 | - | 35035479 | 35058541 | 1453 | annot_exons |
| novel_circ_002963 | ENSSSCG00000036052 | 15 | - | 84229793 | 84230007 | 215 | one_exon |
| novel_circ_002964 | ENSSSCG00000030486 | 13 | - | 1.59E+08 | 1.59E+08 | 423 | annot_exons |
| novel_circ_002965 | ENSSSCG00000031775 | 5 | - | 73463796 | 73475069 | 11181 | exon_intron |
| novel_circ_002966 | ENSSSCG00000006100 | 4 | + | 42203898 | 42209472 | 1400 | annot_exons |
| novel_circ_002967 | ENSSSCG00000010863 | 10 | + | 15083385 | 15085471 | 516 | annot_exons |
| novel_circ_002968 | ENSSSCG00000039701 | 7 | + | 92719325 | 92719672 | 348 | antisense |
| novel_circ_002969 | ENSSSCG00000029578 | 6 | + | 28711467 | 28783794 | 6468 | annot_exons |
| novel_circ_002970 | ENSSSCG00000008215 | 3 | - | 57885445 | 57898889 | 751 | annot_exons |
| novel_circ_002971 | ENSSSCG00000016069 | 15 | + | 1.01E+08 | 1.01E+08 | 736 | annot_exons |
| novel_circ_002972 | ENSSSCG00000031627 | 11 | - | 25758593 | 25761445 | 291 | annot_exons |
| novel_circ_002973 | ENSSSCG00000026959 | 9 | - | 36581948 | 36587073 | 400 | annot_exons |
| novel_circ_002974 | ENSSSCG00000005992 | 4 | + | 17596492 | 17597118 | 627 | one_exon |
| novel_circ_002975 | ENSSSCG00000021478 | 12 | + | 23310823 | 23314040 | 261 | annot_exons |
| novel_circ_002976 | ENSSSCG00000035676 | 16 | - | 71253682 | 71254824 | 351 | annot_exons |
| novel_circ_002977 | ENSSSCG00000002690 | 6 | - | 6955954 | 6958719 | 466 | annot_exons |
| novel_circ_002978 | ENSSSCG00000006386 | 4 | + | 90154266 | 90165184 | 539 | annot_exons |
| novel_circ_002979 | ENSSSCG00000008079 | 3 | + | 42919426 | 42920029 | 376 | annot_exons |
| novel_circ_002980 | ENSSSCG00000033692 | 14 | + | 55770332 | 55799718 | 650 | annot_exons |
| novel_circ_002981 | ENSSSCG00000011042 | 10 | + | 45139215 | 45142435 | 317 | annot_exons |
| novel_circ_002982 | ENSSSCG00000036669 | 2 | - | 9214669 | 9215059 | 391 | antisense |
| novel_circ_002983 | NA | 2 | - | 6751798 | 6752074 | 277 | intergenic |
| novel_circ_002984 | ENSSSCG00000006386 | 4 | + | 90154266 | 90171499 | 757 | annot_exons |
| novel_circ_002985 | ENSSSCG00000006074 | 4 | + | 38110196 | 38160794 | 50078 | exon_intron |
| novel_circ_002986 | ENSSSCG00000029539 | 7 | + | 28639544 | 28653086 | 3617 | exon_intron |
| novel_circ_002987 | ENSSSCG00000004701 | 1 | + | 1.28E+08 | 1.28E+08 | 221 | one_exon |
| novel_circ_002988 | ENSSSCG00000032522 | 15 | + | 1.21E+08 | 1.21E+08 | 327 | annot_exons |
| novel_circ_002989 | ENSSSCG00000004716 | 1 | - | 1.28E+08 | 1.28E+08 | 390 | annot_exons |
| novel_circ_002990 | ENSSSCG00000014207 | 2 | - | 1.17E+08 | 1.17E+08 | 574 | annot_exons |
| novel_circ_002991 | ENSSSCG00000014339 | 2 | + | 1.41E+08 | 1.41E+08 | 303 | annot_exons |
| novel_circ_002992 | NA | 15 | + | 84238715 | 84238978 | 264 | intergenic |
| novel_circ_002993 | ENSSSCG00000005983 | 4 | + | 16054625 | 16055697 | 406 | annot_exons |
| novel_circ_002994 | NA | 8 | - | 3992101 | 4001482 | 9382 | intergenic |
| novel_circ_002995 | ENSSSCG00000009244 | 8 | + | 1.35E+08 | 1.35E+08 | 315 | annot_exons |
| novel_circ_002996 | ENSSSCG00000005997 | 4 | - | 19018199 | 19027950 | 497 | annot_exons |
| novel_circ_002997 | ENSSSCG00000008583 | 3 | + | 1.14E+08 | 1.14E+08 | 457 | annot_exons |
| novel_circ_002998 | ENSSSCG00000012000 | 13 | + | 1.74E+08 | 1.74E+08 | 1663 | annot_exons |
| novel_circ_002999 | ENSSSCG00000008970 | 8 | - | 71251151 | 71257962 | 573 | annot_exons |
| novel_circ_003000 | NA | X | - | 59293851 | 59294382 | 532 | intergenic |
| novel_circ_003001 | ENSSSCG00000034677 | 15 | + | 84408231 | 84409999 | 1769 | antisense |
| novel_circ_003002 | ENSSSCG00000009435 | 11 | - | 25573447 | 25589582 | 757 | annot_exons |
| novel_circ_003003 | ENSSSCG00000003790 | 6 | + | 1.43E+08 | 1.43E+08 | 344 | annot_exons |
| novel_circ_003004 | ENSSSCG00000026996 | 11 | - | 64133759 | 64138070 | 675 | annot_exons |
| novel_circ_003005 | ENSSSCG00000023738 | 11 | - | 21778941 | 21793730 | 970 | annot_exons |
| novel_circ_003006 | ENSSSCG00000008385 | 3 | + | 80795078 | 80800559 | 724 | exon_intron |
| novel_circ_003007 | ENSSSCG00000005613 | 1 | + | 2.68E+08 | 2.68E+08 | 2681 | exon_intron |
| novel_circ_003008 | ENSSSCG00000027741 | 6 | + | 57008834 | 57011490 | 2657 | exon_intron |
| novel_circ_003009 | ENSSSCG00000001806 | 7 | + | 52185502 | 52190846 | 310 | annot_exons |
| novel_circ_003010 | ENSSSCG00000010298 | 14 | - | 76140586 | 76146519 | 387 | annot_exons |
| novel_circ_003011 | ENSSSCG00000016888 | 16 | - | 32446070 | 32448182 | 403 | annot_exons |
| novel_circ_003012 | ENSSSCG00000001087 | 7 | + | 19265556 | 19273195 | 629 | annot_exons |
| novel_circ_003013 | ENSSSCG00000033937 | 9 | + | 33729669 | 33732436 | 497 | annot_exons |
| novel_circ_003014 | ENSSSCG00000005460 | 1 | + | 2.52E+08 | 2.52E+08 | 4324 | intronic |
| novel_circ_003015 | ENSSSCG00000006889 | 4 | + | 1.23E+08 | 1.23E+08 | 816 | annot_exons |
| novel_circ_003016 | ENSSSCG00000030947 | 4 | + | 7096282 | 7116661 | 413 | annot_exons |
| novel_circ_003017 | ENSSSCG00000004814 | 1 | + | 1.38E+08 | 1.38E+08 | 334 | annot_exons |
| novel_circ_003018 | ENSSSCG00000038558 | 4 | + | 98500781 | 98502211 | 247 | annot_exons |
| novel_circ_003019 | ENSSSCG00000025942 | 13 | - | 1.21E+08 | 1.21E+08 | 246 | annot_exons |
| novel_circ_003020 | ENSSSCG00000021287 | 3 | + | 67593397 | 67593744 | 348 | exon_intron |
| novel_circ_003021 | ENSSSCG00000029633 | 6 | + | 1.57E+08 | 1.57E+08 | 378 | annot_exons |
| novel_circ_003022 | ENSSSCG00000012698 | X | + | 1.11E+08 | 1.11E+08 | 300 | annot_exons |
| novel_circ_003023 | ENSSSCG00000037372 | 1 | - | 10906 | 18696 | 4516 | exon_intron |
| novel_circ_003024 | ENSSSCG00000015284 | 9 | + | 65213903 | 65217479 | 3386 | annot_exons |
| novel_circ_003025 | ENSSSCG00000010849 | 10 | - | 12738033 | 12744135 | 394 | annot_exons |
| novel_circ_003026 | ENSSSCG00000042430 | 15 | - | 84423696 | 84423974 | 279 | intronic |
| novel_circ_003027 | ENSSSCG00000001497 | 7 | - | 28729531 | 28735707 | 238 | annot_exons |
| novel_circ_003028 | ENSSSCG00000015747 | 15 | + | 33554894 | 33557679 | 443 | exon_intron |
| novel_circ_003029 | ENSSSCG00000016981 | 16 | - | 50437615 | 50478728 | 2265 | annot_exons |
| novel_circ_003030 | ENSSSCG00000010999 | 10 | + | 33421630 | 33423785 | 321 | annot_exons |
| novel_circ_003031 | ENSSSCG00000003630 | 6 | + | 92121987 | 92130264 | 467 | annot_exons |
| novel_circ_003032 | NA | 18 | - | 2648872 | 2651581 | 2710 | intergenic |
| novel_circ_003033 | ENSSSCG00000011192 | 13 | - | 2799779 | 2802701 | 454 | annot_exons |
| novel_circ_003034 | ENSSSCG00000004570 | 1 | - | 1.09E+08 | 1.09E+08 | 4032 | annot_exons |
| novel_circ_003035 | ENSSSCG00000005060 | 1 | + | 1.85E+08 | 1.85E+08 | 419 | annot_exons |
| novel_circ_003036 | ENSSSCG00000004856 | 1 | - | 1.46E+08 | 1.46E+08 | 1098 | one_exon |
| novel_circ_003037 | ENSSSCG00000037556 | 9 | - | 1.07E+08 | 1.07E+08 | 467 | annot_exons |
| novel_circ_003038 | ENSSSCG00000015874 | 15 | - | 64783591 | 64795113 | 723 | annot_exons |
| novel_circ_003039 | ENSSSCG00000017904 | 12 | - | 51956216 | 51956609 | 303 | annot_exons |
| novel_circ_003040 | ENSSSCG00000034484 | 6 | + | 75042627 | 75043103 | 477 | one_exon |
| novel_circ_003041 | ENSSSCG00000002029 | 7 | + | 75653484 | 75658286 | 1559 | annot_exons |
| novel_circ_003042 | NA | 15 | - | 84312453 | 84313315 | 863 | intergenic |
| novel_circ_003043 | ENSSSCG00000037236 | 13 | + | 24074311 | 24075912 | 285 | annot_exons |
| novel_circ_003044 | ENSSSCG00000008799 | 8 | + | 32489104 | 32573367 | 84264 | exon_intron |
| novel_circ_003045 | ENSSSCG00000005436 | 1 | + | 2.48E+08 | 2.48E+08 | 899 | annot_exons |
| novel_circ_003046 | ENSSSCG00000005706 | 1 | + | 2.71E+08 | 2.71E+08 | 1191 | annot_exons |
| novel_circ_003047 | NA | 15 | + | 84458321 | 84459948 | 1628 | intergenic |
| novel_circ_003048 | ENSSSCG00000014114 | 2 | + | 88496660 | 88500881 | 4222 | intronic |
| novel_circ_003049 | ENSSSCG00000006174 | 4 | - | 61694607 | 61694922 | 316 | antisense |
| novel_circ_003050 | ENSSSCG00000008495 | 3 | + | 1.03E+08 | 1.03E+08 | 355 | annot_exons |
| novel_circ_003051 | ENSSSCG00000023112 | 8 | - | 31024321 | 31041185 | 1347 | annot_exons |
| novel_circ_003052 | ENSSSCG00000003438 | 6 | + | 72363394 | 72390656 | 2012 | annot_exons |
| novel_circ_003053 | ENSSSCG00000037159 | 12 | - | 48472937 | 48480255 | 581 | annot_exons |
| novel_circ_003054 | ENSSSCG00000011858 | 13 | + | 1.35E+08 | 1.35E+08 | 686 | annot_exons |
| novel_circ_003055 | ENSSSCG00000011287 | 13 | - | 26208740 | 26211358 | 2051 | exon_intron |
| novel_circ_003056 | ENSSSCG00000016127 | 15 | - | 1.09E+08 | 1.09E+08 | 566 | annot_exons |
| novel_circ_003057 | ENSSSCG00000012884 | 2 | - | 4437791 | 4459058 | 864 | annot_exons |
| novel_circ_003058 | ENSSSCG00000033692 | 14 | + | 55770332 | 55789427 | 539 | annot_exons |
| novel_circ_003059 | ENSSSCG00000012165 | X | + | 18037310 | 18038865 | 300 | annot_exons |
| novel_circ_003060 | ENSSSCG00000006766 | 4 | + | 1.07E+08 | 1.07E+08 | 219 | annot_exons |
| novel_circ_003061 | ENSSSCG00000037655 | 13 | + | 1.89E+08 | 1.89E+08 | 3121 | annot_exons |
| novel_circ_003062 | ENSSSCG00000010053 | 14 | + | 49380314 | 49380521 | 208 | one_exon |
| novel_circ_003063 | ENSSSCG00000033142 | 5 | + | 78073759 | 78075966 | 2208 | antisense |
| novel_circ_003064 | ENSSSCG00000031705 | 16 | - | 18387166 | 18392618 | 5453 | exon_intron |
| novel_circ_003065 | ENSSSCG00000008332 | 3 | + | 72300370 | 72306113 | 321 | annot_exons |
| novel_circ_003066 | ENSSSCG00000030343 | 16 | - | 34791656 | 34803686 | 470 | annot_exons |
| novel_circ_003067 | ENSSSCG00000011776 | 13 | + | 1.22E+08 | 1.22E+08 | 291 | annot_exons |
| novel_circ_003068 | ENSSSCG00000011811 | 13 | + | 1.26E+08 | 1.26E+08 | 201 | one_exon |
| novel_circ_003069 | ENSSSCG00000016863 | 16 | - | 26631927 | 26658361 | 11749 | exon_intron |
| novel_circ_003070 | ENSSSCG00000001955 | 7 | - | 64841730 | 64842287 | 183 | annot_exons |
| novel_circ_003071 | ENSSSCG00000024481 | 4 | - | 1.01E+08 | 1.01E+08 | 1946 | annot_exons |
| novel_circ_003072 | ENSSSCG00000007166 | 17 | + | 32779392 | 32779645 | 254 | antisense |
| novel_circ_003073 | ENSSSCG00000009300 | 11 | + | 4338231 | 4345913 | 407 | annot_exons |
| novel_circ_003074 | ENSSSCG00000004917 | 1 | - | 1.62E+08 | 1.62E+08 | 276 | annot_exons |
| novel_circ_003075 | ENSSSCG00000004505 | 1 | - | 97447508 | 97451864 | 509 | annot_exons |
| novel_circ_003076 | ENSSSCG00000005436 | 1 | + | 2.48E+08 | 2.48E+08 | 405 | annot_exons |
| novel_circ_003077 | ENSSSCG00000001002 | 7 | - | 2373349 | 2373676 | 328 | antisense |
| novel_circ_003078 | NA | 18 | - | 2648872 | 2661452 | 12581 | intergenic |
| novel_circ_003079 | ENSSSCG00000021588 | 1 | - | 1.08E+08 | 1.08E+08 | 2498 | antisense |
| novel_circ_003080 | ENSSSCG00000011258 | 13 | + | 23282794 | 23293830 | 553 | annot_exons |
| novel_circ_003081 | ENSSSCG00000030396 | 8 | + | 87525170 | 87525542 | 373 | one_exon |
| novel_circ_003082 | ENSSSCG00000000571 | 5 | + | 51563352 | 51584647 | 699 | annot_exons |
| novel_circ_003083 | ENSSSCG00000034569 | 2 | + | 51346346 | 51351104 | 552 | annot_exons |
| novel_circ_003084 | ENSSSCG00000043851 | 14 | + | 89913666 | 89914248 | 583 | one_exon |
| novel_circ_003085 | ENSSSCG00000001641 | 7 | + | 37759054 | 37768836 | 506 | annot_exons |
| novel_circ_003086 | ENSSSCG00000009464 | 11 | - | 47571129 | 47577956 | 530 | annot_exons |
| novel_circ_003087 | ENSSSCG00000013054 | 2 | + | 8377390 | 8389221 | 360 | annot_exons |
| novel_circ_003088 | ENSSSCG00000024108 | 12 | - | 47824734 | 47828717 | 341 | annot_exons |
| novel_circ_003089 | ENSSSCG00000035230 | 6 | + | 946547 | 947855 | 305 | annot_exons |
| novel_circ_003090 | ENSSSCG00000030396 | 8 | + | 87488517 | 87498301 | 332 | annot_exons |
| novel_circ_003091 | ENSSSCG00000006388 | 4 | + | 90259590 | 90260459 | 237 | annot_exons |
| novel_circ_003092 | ENSSSCG00000009505 | 11 | + | 66594891 | 66605238 | 673 | annot_exons |
| novel_circ_003093 | ENSSSCG00000010928 | 10 | - | 24760509 | 24761243 | 307 | annot_exons |
| novel_circ_003094 | ENSSSCG00000011013 | 10 | + | 39984146 | 39996009 | 645 | annot_exons |
| novel_circ_003095 | ENSSSCG00000026465 | 3 | - | 1.11E+08 | 1.11E+08 | 443 | annot_exons |
| novel_circ_003096 | ENSSSCG00000024377 | 7 | - | 1.13E+08 | 1.13E+08 | 412 | annot_exons |
| novel_circ_003097 | ENSSSCG00000016069 | 15 | + | 1.01E+08 | 1.01E+08 | 810 | annot_exons |
| novel_circ_003098 | ENSSSCG00000011198 | 13 | - | 3611031 | 3611427 | 397 | intronic |
| novel_circ_003099 | NA | 7 | + | 68200000 | 68215565 | 15566 | intergenic |
| novel_circ_003100 | ENSSSCG00000010569 | 14 | - | 1.13E+08 | 1.13E+08 | 543 | annot_exons |
| novel_circ_003101 | ENSSSCG00000009134 | 8 | - | 1.12E+08 | 1.12E+08 | 864 | annot_exons |
| novel_circ_003102 | ENSSSCG00000026239 | 6 | + | 37955596 | 37962774 | 1257 | annot_exons |
| novel_circ_003103 | ENSSSCG00000023738 | 11 | - | 21804834 | 21808388 | 460 | annot_exons |
| novel_circ_003104 | ENSSSCG00000007337 | 17 | + | 40809934 | 40843085 | 655 | annot_exons |
| novel_circ_003105 | ENSSSCG00000011532 | 13 | - | 61018842 | 61022479 | 249 | annot_exons |
| novel_circ_003106 | ENSSSCG00000004454 | 1 | + | 82953759 | 82955701 | 212 | annot_exons |
| novel_circ_003107 | ENSSSCG00000029920 | 8 | + | 87799383 | 87801061 | 327 | annot_exons |
| novel_circ_003108 | ENSSSCG00000007203 | 17 | + | 34741781 | 34743068 | 277 | annot_exons |
| novel_circ_003109 | ENSSSCG00000029547 | 15 | + | 95259699 | 95260536 | 838 | one_exon |
| novel_circ_003110 | ENSSSCG00000015097 | 9 | - | 46038901 | 46046498 | 741 | annot_exons |
| novel_circ_003111 | ENSSSCG00000002544 | AEMK02000452.1 | - | 2281177 | 2295643 | 345 | annot_exons |
| novel_circ_003112 | ENSSSCG00000010947 | 10 | + | 27648836 | 27651062 | 279 | annot_exons |
| novel_circ_003113 | ENSSSCG00000002508 | 7 | - | 1.2E+08 | 1.2E+08 | 572 | annot_exons |
| novel_circ_003114 | ENSSSCG00000039885 | 6 | - | 88432187 | 88432891 | 705 | one_exon |
| novel_circ_003115 | ENSSSCG00000016094 | 15 | + | 1.05E+08 | 1.05E+08 | 578 | annot_exons |
| novel_circ_003116 | ENSSSCG00000009528 | 11 | + | 70995656 | 70999816 | 380 | annot_exons |
| novel_circ_003117 | ENSSSCG00000017869 | 12 | - | 49900022 | 49900829 | 206 | annot_exons |
| novel_circ_003118 | ENSSSCG00000007574 | 3 | + | 3095556 | 3096513 | 303 | annot_exons |
| novel_circ_003119 | NA | 15 | + | 84262877 | 84263224 | 348 | intergenic |
| novel_circ_003120 | ENSSSCG00000017102 | 16 | - | 75225387 | 75232858 | 743 | annot_exons |
| novel_circ_003121 | ENSSSCG00000015368 | 9 | + | 87818565 | 87822303 | 552 | annot_exons |
| novel_circ_003122 | ENSSSCG00000021644 | 6 | + | 34513016 | 34518902 | 708 | annot_exons |
| novel_circ_003123 | ENSSSCG00000024754 | 18 | - | 40140465 | 40161356 | 422 | annot_exons |
| novel_circ_003124 | ENSSSCG00000007284 | 17 | - | 38125800 | 38138374 | 3251 | annot_exons |
| novel_circ_003125 | ENSSSCG00000004460 | 1 | + | 84020523 | 84036212 | 727 | annot_exons |
| novel_circ_003126 | ENSSSCG00000040317 | 1 | + | 7689994 | 7690545 | 552 | one_exon |
| novel_circ_003127 | ENSSSCG00000007424 | 17 | + | 48025586 | 48026549 | 964 | antisense |
| novel_circ_003128 | ENSSSCG00000024784 | 10 | - | 23525570 | 23526296 | 352 | annot_exons |
| novel_circ_003129 | ENSSSCG00000002337 | 7 | - | 96334164 | 96353198 | 827 | annot_exons |
| novel_circ_003130 | ENSSSCG00000004082 | 1 | + | 14090110 | 14095458 | 996 | annot_exons |
| novel_circ_003131 | ENSSSCG00000014081 | 2 | - | 84452944 | 84457932 | 251 | exon_intron |
| novel_circ_003132 | ENSSSCG00000009111 | 8 | + | 1.05E+08 | 1.05E+08 | 609 | antisense |
| novel_circ_003133 | ENSSSCG00000000064 | 5 | - | 7029907 | 7040783 | 489 | annot_exons |
| novel_circ_003134 | ENSSSCG00000029251 | 13 | - | 30681584 | 30691776 | 372 | annot_exons |
| novel_circ_003135 | ENSSSCG00000029196 | 5 | + | 16536102 | 16540801 | 437 | annot_exons |
| novel_circ_003136 | ENSSSCG00000017836 | 12 | + | 48687199 | 48712536 | 587 | annot_exons |
| novel_circ_003137 | ENSSSCG00000003700 | 6 | - | 1.07E+08 | 1.07E+08 | 481 | annot_exons |
| novel_circ_003138 | ENSSSCG00000015480 | 9 | + | 1.14E+08 | 1.14E+08 | 1939 | annot_exons |
| novel_circ_003139 | ENSSSCG00000003520 | 6 | + | 80068941 | 80072086 | 228 | annot_exons |
| novel_circ_003140 | ENSSSCG00000007930 | 3 | - | 37730065 | 37733938 | 473 | annot_exons |
| novel_circ_003141 | ENSSSCG00000006646 | 4 | + | 98369584 | 98372553 | 405 | annot_exons |
| novel_circ_003142 | ENSSSCG00000000890 | 5 | - | 86731715 | 86735350 | 441 | annot_exons |
| novel_circ_003143 | ENSSSCG00000004753 | 1 | + | 1.3E+08 | 1.3E+08 | 464 | annot_exons |
| novel_circ_003144 | ENSSSCG00000004090 | 1 | + | 14885681 | 14886144 | 464 | exon_intron |
| novel_circ_003145 | ENSSSCG00000006646 | 4 | + | 98369520 | 98375331 | 681 | annot_exons |
| novel_circ_003146 | ENSSSCG00000024377 | 7 | - | 1.13E+08 | 1.13E+08 | 224 | annot_exons |
| novel_circ_003147 | ENSSSCG00000037371 | 13 | - | 18973813 | 18974491 | 679 | one_exon |
| novel_circ_003148 | ENSSSCG00000034191 | 2 | + | 42481218 | 42573733 | 72751 | exon_intron |
| novel_circ_003149 | ENSSSCG00000003775 | 6 | - | 1.37E+08 | 1.37E+08 | 241 | annot_exons |
| novel_circ_003150 | ENSSSCG00000038323 | 2 | - | 5862053 | 5862461 | 315 | annot_exons |
| novel_circ_003151 | ENSSSCG00000040989 | 12 | - | 6704681 | 6705766 | 1086 | one_exon |
| novel_circ_003152 | ENSSSCG00000035119 | 11 | - | 3200458 | 3203716 | 492 | annot_exons |
| novel_circ_003153 | ENSSSCG00000015434 | 9 | - | 1.06E+08 | 1.06E+08 | 235 | one_exon |
| novel_circ_003154 | ENSSSCG00000046809 | 6 | + | 47368460 | 47383155 | 14524 | exon_intron |
| novel_circ_003155 | ENSSSCG00000004008 | 1 | + | 641934 | 653683 | 300 | annot_exons |
| novel_circ_003156 | ENSSSCG00000010190 | 14 | - | 60415413 | 60415661 | 249 | antisense |
| novel_circ_003157 | ENSSSCG00000036132 | 6 | + | 51707036 | 51712341 | 5306 | antisense |
| novel_circ_003158 | NA | 15 | + | 84250543 | 84251864 | 1322 | intergenic |
| novel_circ_003159 | ENSSSCG00000038007 | 10 | - | 43907525 | 43918659 | 527 | annot_exons |
| novel_circ_003160 | ENSSSCG00000008162 | 3 | - | 52234070 | 52239994 | 404 | annot_exons |
| novel_circ_003161 | ENSSSCG00000002931 | 6 | - | 45553891 | 45567303 | 389 | annot_exons |
| novel_circ_003162 | ENSSSCG00000016976 | 16 | - | 48849361 | 48899780 | 299 | exon_intron |
| novel_circ_003163 | ENSSSCG00000004456 | 1 | + | 83020400 | 83025603 | 420 | annot_exons |
| novel_circ_003164 | ENSSSCG00000009244 | 8 | + | 1.35E+08 | 1.35E+08 | 896 | annot_exons |
| novel_circ_003165 | ENSSSCG00000002315 | 7 | - | 93766303 | 93775334 | 9032 | exon_intron |
| novel_circ_003166 | ENSSSCG00000015689 | 15 | + | 15866989 | 15880950 | 666 | annot_exons |
| novel_circ_003167 | ENSSSCG00000011873 | 13 | + | 1.38E+08 | 1.38E+08 | 1274 | one_exon |
| novel_circ_003168 | ENSSSCG00000000985 | 7 | - | 259291 | 284670 | 782 | annot_exons |
| novel_circ_003169 | ENSSSCG00000010690 | 14 | + | 1.3E+08 | 1.3E+08 | 457 | exon_intron |
| novel_circ_003170 | ENSSSCG00000009359 | 11 | - | 12854807 | 12862293 | 385 | annot_exons |
| novel_circ_003171 | NA | 2 | - | 6810490 | 6811177 | 688 | intergenic |
| novel_circ_003172 | ENSSSCG00000011022 | 10 | + | 41416480 | 41424754 | 503 | annot_exons |
| novel_circ_003173 | ENSSSCG00000016543 | 18 | - | 15811892 | 15812135 | 244 | one_exon |
| novel_circ_003174 | ENSSSCG00000027403 | 8 | - | 55614208 | 55623888 | 1448 | annot_exons |
| novel_circ_003175 | ENSSSCG00000000492 | 5 | + | 33533423 | 33545826 | 1409 | annot_exons |
| novel_circ_003176 | ENSSSCG00000025941 | 1 | - | 45179113 | 45179394 | 282 | antisense |
| novel_circ_003177 | ENSSSCG00000020898 | 6 | + | 1.2E+08 | 1.2E+08 | 449 | annot_exons |
| novel_circ_003178 | ENSSSCG00000014819 | 9 | + | 7353804 | 7354666 | 432 | annot_exons |
| novel_circ_003179 | NA | 5 | - | 415443 | 416533 | 1091 | intergenic |
| novel_circ_003180 | ENSSSCG00000016911 | 16 | + | 34555976 | 34590178 | 919 | annot_exons |
| novel_circ_003181 | ENSSSCG00000001805 | 7 | - | 52112041 | 52122358 | 1182 | annot_exons |
| novel_circ_003182 | ENSSSCG00000023445 | 18 | - | 40685239 | 40687168 | 355 | annot_exons |
| novel_circ_003183 | NA | 15 | - | 84262522 | 84262712 | 191 | intergenic |
| novel_circ_003184 | ENSSSCG00000006135 | 4 | - | 50296155 | 50296891 | 194 | annot_exons |
| novel_circ_003185 | ENSSSCG00000004958 | 1 | + | 1.66E+08 | 1.66E+08 | 332 | annot_exons |
| novel_circ_003186 | ENSSSCG00000022430 | 16 | + | 47292545 | 47296893 | 1124 | exon_intron |
| novel_circ_003187 | ENSSSCG00000004138 | 1 | + | 22285744 | 22352249 | 192 | exon_intron |
| novel_circ_003188 | ENSSSCG00000008368 | 3 | - | 78013714 | 78014790 | 236 | annot_exons |
| novel_circ_003189 | ENSSSCG00000017678 | 12 | + | 37636936 | 37641401 | 241 | annot_exons |
| novel_circ_003190 | ENSSSCG00000033700 | 14 | + | 90489536 | 90489914 | 179 | annot_exons |
| novel_circ_003191 | ENSSSCG00000006930 | 4 | + | 1.29E+08 | 1.29E+08 | 342 | antisense |
| novel_circ_003192 | ENSSSCG00000004432 | 1 | - | 79873989 | 79874476 | 250 | annot_exons |
| novel_circ_003193 | ENSSSCG00000009968 | 14 | - | 45490289 | 45490796 | 508 | one_exon |
| novel_circ_003194 | ENSSSCG00000008169 | 3 | + | 53237773 | 53238013 | 241 | one_exon |
| novel_circ_003195 | ENSSSCG00000015747 | 15 | + | 33569805 | 33577863 | 2342 | annot_exons |
| novel_circ_003196 | ENSSSCG00000009007 | 8 | - | 75494755 | 75507182 | 1295 | annot_exons |
| novel_circ_003197 | ENSSSCG00000003738 | 6 | + | 1.19E+08 | 1.19E+08 | 360 | annot_exons |
| novel_circ_003198 | ENSSSCG00000006709 | 4 | + | 1.01E+08 | 1.01E+08 | 1208 | annot_exons |
| novel_circ_003199 | ENSSSCG00000023915 | 12 | + | 52632848 | 52634240 | 629 | annot_exons |
| novel_circ_003200 | ENSSSCG00000009077 | 8 | - | 97050978 | 97053136 | 237 | annot_exons |
| novel_circ_003201 | ENSSSCG00000001501 | 7 | - | 29634004 | 29635978 | 773 | annot_exons |
| novel_circ_003202 | NA | 2 | + | 6796798 | 6797142 | 345 | intergenic |
| novel_circ_003203 | ENSSSCG00000005582 | 1 | - | 2.64E+08 | 2.64E+08 | 1214 | annot_exons |
| novel_circ_003204 | NA | 15 | - | 84270843 | 84271091 | 249 | intergenic |
| novel_circ_003205 | ENSSSCG00000027613 | 1 | + | 39358698 | 39359115 | 418 | intronic |
| novel_circ_003206 | ENSSSCG00000014298 | 2 | - | 1.37E+08 | 1.37E+08 | 755 | annot_exons |
| novel_circ_003207 | ENSSSCG00000010470 | 14 | - | 1.04E+08 | 1.04E+08 | 393 | annot_exons |
| novel_circ_003208 | ENSSSCG00000004857 | 1 | - | 1.46E+08 | 1.46E+08 | 2955 | annot_exons |
| novel_circ_003209 | ENSSSCG00000000502 | 5 | + | 34492041 | 34513084 | 575 | annot_exons |
| novel_circ_003210 | ENSSSCG00000002799 | 6 | - | 20317779 | 20326998 | 1173 | annot_exons |
| novel_circ_003211 | ENSSSCG00000000728 | 5 | + | 66469779 | 66470061 | 204 | annot_exons |
| novel_circ_003212 | ENSSSCG00000004366 | 1 | - | 71349075 | 71350243 | 297 | annot_exons |
| novel_circ_003213 | ENSSSCG00000004032 | 1 | - | 5643275 | 5643488 | 214 | antisense |
| novel_circ_003214 | ENSSSCG00000014110 | 2 | - | 87694641 | 87694884 | 244 | one_exon |
| novel_circ_003215 | ENSSSCG00000015582 | 9 | + | 1.29E+08 | 1.29E+08 | 1478 | one_exon |
| novel_circ_003216 | ENSSSCG00000005090 | 1 | + | 1.9E+08 | 1.9E+08 | 331 | annot_exons |
| novel_circ_003217 | ENSSSCG00000002266 | 7 | - | 85996423 | 86006435 | 628 | annot_exons |
| novel_circ_003218 | ENSSSCG00000008498 | 3 | + | 1.03E+08 | 1.03E+08 | 643 | annot_exons |
| novel_circ_003219 | ENSSSCG00000009551 | 11 | + | 77489152 | 77503607 | 403 | annot_exons |
| novel_circ_003220 | ENSSSCG00000016842 | 16 | + | 22257342 | 22258377 | 252 | annot_exons |
| novel_circ_003221 | ENSSSCG00000026536 | 11 | - | 78703329 | 78703726 | 398 | one_exon |
| novel_circ_003222 | ENSSSCG00000000877 | 5 | - | 83824883 | 83831997 | 489 | annot_exons |
| novel_circ_003223 | ENSSSCG00000003700 | 6 | - | 1.07E+08 | 1.07E+08 | 513 | annot_exons |
| novel_circ_003224 | ENSSSCG00000030996 | 14 | + | 46012122 | 46012338 | 217 | antisense |
| novel_circ_003225 | ENSSSCG00000004433 | 1 | - | 80078290 | 80136352 | 304 | annot_exons |
| novel_circ_003226 | ENSSSCG00000016095 | 15 | - | 1.05E+08 | 1.05E+08 | 592 | annot_exons |
| novel_circ_003227 | ENSSSCG00000029944 | 12 | + | 925348 | 925685 | 251 | annot_exons |
| novel_circ_003228 | ENSSSCG00000003761 | 6 | - | 1.32E+08 | 1.32E+08 | 1155 | annot_exons |
| novel_circ_003229 | ENSSSCG00000006308 | 4 | + | 83463212 | 83465136 | 305 | annot_exons |
| novel_circ_003230 | ENSSSCG00000032097 | 5 | + | 59915033 | 59916786 | 730 | annot_exons |
| novel_circ_003231 | ENSSSCG00000002467 | 7 | + | 1.15E+08 | 1.15E+08 | 188 | antisense |
| novel_circ_003232 | ENSSSCG00000009839 | 14 | + | 33115205 | 33122872 | 498 | annot_exons |
| novel_circ_003233 | ENSSSCG00000014828 | 9 | - | 8155799 | 8158002 | 324 | annot_exons |
| novel_circ_003234 | ENSSSCG00000008445 | 3 | + | 94934784 | 94939823 | 424 | annot_exons |
| novel_circ_003235 | ENSSSCG00000030386 | 18 | - | 13766233 | 13768583 | 317 | annot_exons |
| novel_circ_003236 | ENSSSCG00000023409 | 3 | + | 72257086 | 72270592 | 1342 | annot_exons |
| novel_circ_003237 | ENSSSCG00000009229 | 8 | - | 1.33E+08 | 1.33E+08 | 1416 | annot_exons |
| novel_circ_003238 | ENSSSCG00000000866 | 5 | - | 82571957 | 82572152 | 196 | one_exon |
| novel_circ_003239 | ENSSSCG00000013223 | 2 | + | 14884459 | 14887371 | 370 | annot_exons |
| novel_circ_003240 | ENSSSCG00000007153 | 17 | - | 32173028 | 32184644 | 618 | annot_exons |
| novel_circ_003241 | ENSSSCG00000010190 | 14 | - | 60414178 | 60414590 | 413 | antisense |
| novel_circ_003242 | ENSSSCG00000022331 | X | - | 1.14E+08 | 1.14E+08 | 2797 | intronic |
| novel_circ_003243 | ENSSSCG00000011114 | 10 | - | 59810368 | 59813097 | 145 | annot_exons |
| novel_circ_003244 | ENSSSCG00000006388 | 4 | + | 90238894 | 90253801 | 445 | annot_exons |
| novel_circ_003245 | ENSSSCG00000023709 | 2 | - | 14599740 | 14602393 | 426 | annot_exons |
| novel_circ_003246 | ENSSSCG00000002740 | 6 | - | 14729467 | 14739512 | 771 | annot_exons |
| novel_circ_003247 | ENSSSCG00000000512 | 5 | + | 35866143 | 35877522 | 641 | annot_exons |
| novel_circ_003248 | ENSSSCG00000006283 | 4 | + | 81151255 | 81153340 | 300 | annot_exons |
| novel_circ_003249 | ENSSSCG00000009010 | 8 | - | 76204104 | 76212150 | 380 | annot_exons |
| novel_circ_003250 | ENSSSCG00000006766 | 4 | + | 1.07E+08 | 1.07E+08 | 1672 | annot_exons |
| novel_circ_003251 | ENSSSCG00000005711 | 1 | + | 2.71E+08 | 2.71E+08 | 1829 | annot_exons |
| novel_circ_003252 | ENSSSCG00000001862 | 7 | - | 56515845 | 56519340 | 382 | annot_exons |
| novel_circ_003253 | ENSSSCG00000014387 | 2 | + | 1.43E+08 | 1.43E+08 | 681 | antisense |
| novel_circ_003254 | ENSSSCG00000001779 | 7 | + | 49019208 | 49021872 | 441 | annot_exons |
| novel_circ_003255 | ENSSSCG00000006872 | 4 | - | 1.18E+08 | 1.18E+08 | 150 | one_exon |
| novel_circ_003256 | NA | 8 | + | 3960207 | 3960790 | 584 | intergenic |
| novel_circ_003257 | ENSSSCG00000027738 | 13 | + | 21724153 | 21724779 | 627 | intronic |
| novel_circ_003258 | ENSSSCG00000005015 | 1 | - | 1.8E+08 | 1.8E+08 | 1712 | annot_exons |
| novel_circ_003259 | ENSSSCG00000011775 | 13 | + | 1.22E+08 | 1.22E+08 | 1348 | annot_exons |
| novel_circ_003260 | ENSSSCG00000000466 | 5 | + | 28891382 | 28894911 | 312 | annot_exons |
| novel_circ_003261 | ENSSSCG00000002461 | 7 | - | 1.15E+08 | 1.15E+08 | 446 | annot_exons |
| novel_circ_003262 | ENSSSCG00000004064 | 1 | - | 10131566 | 10175846 | 44281 | exon_intron |
| novel_circ_003263 | ENSSSCG00000009464 | 11 | - | 47733496 | 47733979 | 484 | intronic |
| novel_circ_003264 | ENSSSCG00000014946 | 9 | + | 26125750 | 26130163 | 419 | annot_exons |
| novel_circ_003265 | ENSSSCG00000015435 | 9 | - | 1.06E+08 | 1.06E+08 | 390 | annot_exons |
| novel_circ_003266 | ENSSSCG00000000071 | 5 | + | 7603617 | 7607294 | 299 | annot_exons |
| novel_circ_003267 | ENSSSCG00000028512 | 2 | + | 69853256 | 69853745 | 400 | annot_exons |
| novel_circ_003268 | ENSSSCG00000025316 | 13 | - | 95696483 | 95703536 | 719 | annot_exons |
| novel_circ_003269 | ENSSSCG00000011843 | 13 | - | 1.33E+08 | 1.33E+08 | 528 | annot_exons |
| novel_circ_003270 | ENSSSCG00000009007 | 8 | - | 75516109 | 75517258 | 183 | annot_exons |
| novel_circ_003271 | ENSSSCG00000017268 | 12 | + | 13183766 | 13185579 | 389 | annot_exons |
| novel_circ_003272 | NA | 15 | - | 84462160 | 84465728 | 3569 | intergenic |
| novel_circ_003273 | ENSSSCG00000002340 | 7 | + | 96517745 | 96521028 | 396 | annot_exons |
| novel_circ_003274 | ENSSSCG00000000077 | 5 | - | 8029917 | 8030829 | 267 | annot_exons |
| novel_circ_003275 | ENSSSCG00000003728 | 6 | - | 1.16E+08 | 1.16E+08 | 488 | annot_exons |
| novel_circ_003276 | ENSSSCG00000010380 | 14 | + | 89064375 | 89069006 | 463 | annot_exons |
| novel_circ_003277 | ENSSSCG00000015774 | 15 | + | 44194787 | 44195338 | 552 | intronic |
| novel_circ_003278 | ENSSSCG00000007477 | 17 | - | 52821785 | 52872012 | 1046 | annot_exons |
| novel_circ_003279 | ENSSSCG00000044227 | 7 | - | 56259271 | 56302617 | 445 | annot_exons |
| novel_circ_003280 | ENSSSCG00000009856 | 14 | + | 35261305 | 35265974 | 292 | annot_exons |
| novel_circ_003281 | ENSSSCG00000009781 | 14 | + | 29864677 | 29865475 | 405 | annot_exons |
| novel_circ_003282 | ENSSSCG00000030113 | 11 | + | 3806678 | 3807254 | 577 | antisense |
| novel_circ_003283 | ENSSSCG00000003438 | 6 | + | 72328044 | 72328256 | 213 | one_exon |
| novel_circ_003284 | ENSSSCG00000048702 | 4 | + | 6935279 | 6939470 | 4192 | intronic |
| novel_circ_003285 | ENSSSCG00000023266 | 6 | + | 1.69E+08 | 1.69E+08 | 1117 | annot_exons |
| novel_circ_003286 | ENSSSCG00000014414 | 2 | + | 1.48E+08 | 1.48E+08 | 261 | annot_exons |
| novel_circ_003287 | ENSSSCG00000013883 | 2 | - | 60149063 | 60152648 | 460 | annot_exons |
| novel_circ_003288 | ENSSSCG00000000496 | 5 | + | 34018844 | 34033773 | 631 | annot_exons |
| novel_circ_003289 | ENSSSCG00000009015 | 8 | + | 77285851 | 77301994 | 403 | annot_exons |
| novel_circ_003290 | ENSSSCG00000003511 | 6 | - | 78986846 | 78994000 | 471 | annot_exons |
| novel_circ_003291 | ENSSSCG00000003679 | 6 | + | 98481331 | 98492705 | 482 | annot_exons |
| novel_circ_003292 | ENSSSCG00000012102 | X | + | 6058185 | 6058797 | 613 | intronic |
| novel_circ_003293 | ENSSSCG00000024570 | 2 | - | 73836406 | 73852681 | 1498 | annot_exons |
| novel_circ_003294 | ENSSSCG00000008965 | 8 | - | 70656157 | 70661554 | 265 | annot_exons |
| novel_circ_003295 | ENSSSCG00000030547 | 4 | - | 98027350 | 98027649 | 300 | one_exon |
| novel_circ_003296 | ENSSSCG00000011022 | 10 | + | 41253387 | 41257277 | 3891 | exon_intron |
| novel_circ_003297 | ENSSSCG00000002245 | 7 | + | 80336806 | 80346071 | 896 | annot_exons |
| novel_circ_003298 | ENSSSCG00000032861 | 5 | + | 14119354 | 14134704 | 15351 | exon_intron |
| novel_circ_003299 | ENSSSCG00000009464 | 11 | - | 47592255 | 47605644 | 790 | annot_exons |
| novel_circ_003300 | ENSSSCG00000015427 | 9 | - | 1.04E+08 | 1.04E+08 | 580 | annot_exons |
| novel_circ_003301 | ENSSSCG00000005366 | 1 | + | 2.39E+08 | 2.39E+08 | 618 | annot_exons |
| novel_circ_003302 | ENSSSCG00000021203 | 13 | - | 79521818 | 79571122 | 802 | annot_exons |
| novel_circ_003303 | ENSSSCG00000007478 | 17 | - | 53037004 | 53048324 | 5347 | exon_intron |
| novel_circ_003304 | ENSSSCG00000008826 | 8 | - | 38531170 | 38531719 | 262 | annot_exons |
| novel_circ_003305 | ENSSSCG00000032377 | 7 | + | 64253903 | 64263326 | 510 | annot_exons |
| novel_circ_003306 | ENSSSCG00000021060 | 11 | + | 18161884 | 18165256 | 428 | annot_exons |
| novel_circ_003307 | ENSSSCG00000025606 | 9 | + | 36687166 | 36689855 | 268 | annot_exons |
| novel_circ_003308 | ENSSSCG00000002799 | 6 | - | 20329305 | 20340324 | 977 | annot_exons |
| novel_circ_003309 | ENSSSCG00000005934 | 4 | + | 3163791 | 3172320 | 270 | annot_exons |
| novel_circ_003310 | ENSSSCG00000007253 | 17 | + | 36469353 | 36470700 | 347 | exon_intron |
| novel_circ_003311 | ENSSSCG00000008068 | 3 | - | 42011359 | 42012685 | 403 | annot_exons |
| novel_circ_003312 | ENSSSCG00000011025 | 10 | + | 42063616 | 42147610 | 83995 | exon_intron |
| novel_circ_003313 | ENSSSCG00000024088 | 1 | - | 1.09E+08 | 1.09E+08 | 596 | annot_exons |
| novel_circ_003314 | ENSSSCG00000009717 | 14 | + | 20624916 | 20627939 | 272 | annot_exons |
| novel_circ_003315 | ENSSSCG00000037671 | AEMK02000452.1 | + | 3414060 | 3415726 | 216 | annot_exons |
| novel_circ_003316 | ENSSSCG00000022618 | 13 | + | 18341600 | 18350091 | 430 | annot_exons |
| novel_circ_003317 | ENSSSCG00000006661 | 4 | - | 98994482 | 98995745 | 417 | annot_exons |
| novel_circ_003318 | ENSSSCG00000022636 | 5 | + | 42865804 | 42867248 | 1445 | one_exon |
| novel_circ_003319 | ENSSSCG00000003816 | 6 | - | 1.5E+08 | 1.5E+08 | 402 | annot_exons |
| novel_circ_003320 | ENSSSCG00000002311 | 7 | + | 93293216 | 93296234 | 3019 | exon_intron |
| novel_circ_003321 | ENSSSCG00000009007 | 8 | - | 75554892 | 75644000 | 308 | annot_exons |
| novel_circ_003322 | ENSSSCG00000003699 | 6 | + | 1.07E+08 | 1.07E+08 | 943 | annot_exons |
| novel_circ_003323 | ENSSSCG00000006648 | 4 | + | 98422323 | 98423402 | 292 | annot_exons |
| novel_circ_003324 | ENSSSCG00000008831 | 8 | + | 39008497 | 39032833 | 481 | annot_exons |
| novel_circ_003325 | ENSSSCG00000010175 | 14 | - | 59216478 | 59220580 | 258 | annot_exons |
| novel_circ_003326 | ENSSSCG00000015670 | 15 | + | 7986395 | 8044280 | 751 | annot_exons |
| novel_circ_003327 | ENSSSCG00000017310 | 12 | + | 17044270 | 17060678 | 411 | annot_exons |
| novel_circ_003328 | ENSSSCG00000011788 | 13 | + | 1.23E+08 | 1.23E+08 | 475 | annot_exons |
| novel_circ_003329 | ENSSSCG00000001934 | 7 | + | 61121465 | 61165713 | 1678 | annot_exons |
| novel_circ_003330 | ENSSSCG00000012730 | X | + | 1.21E+08 | 1.21E+08 | 846 | one_exon |
| novel_circ_003331 | ENSSSCG00000029602 | 10 | - | 68491183 | 68507352 | 960 | annot_exons |
| novel_circ_003332 | ENSSSCG00000015857 | 15 | - | 59162535 | 59181694 | 1563 | annot_exons |
| novel_circ_003333 | ENSSSCG00000027417 | 6 | - | 96516466 | 96526660 | 10195 | exon_intron |
| novel_circ_003334 | ENSSSCG00000038383 | 3 | - | 96679882 | 96701311 | 5969 | annot_exons |
| novel_circ_003335 | ENSSSCG00000004082 | 1 | + | 14017929 | 14038852 | 11490 | exon_intron |
| novel_circ_003336 | ENSSSCG00000004576 | 1 | + | 1.11E+08 | 1.11E+08 | 1272 | intronic |
| novel_circ_003337 | ENSSSCG00000012434 | X | - | 61588766 | 61591534 | 501 | annot_exons |
| novel_circ_003338 | ENSSSCG00000012699 | X | - | 1.11E+08 | 1.11E+08 | 328 | antisense |
| novel_circ_003339 | ENSSSCG00000051585 | 6 | - | 18004570 | 18004761 | 192 | one_exon |
| novel_circ_003340 | ENSSSCG00000006762 | 4 | + | 1.07E+08 | 1.07E+08 | 992 | annot_exons |
| novel_circ_003341 | ENSSSCG00000015310 | 9 | + | 72008287 | 72009983 | 337 | exon_intron |
| novel_circ_003342 | ENSSSCG00000016605 | 18 | - | 23804005 | 23804324 | 320 | one_exon |
| novel_circ_003343 | ENSSSCG00000000939 | 5 | - | 1E+08 | 1E+08 | 205 | annot_exons |
| novel_circ_003344 | ENSSSCG00000003760 | 6 | + | 1.3E+08 | 1.3E+08 | 313 | annot_exons |
| novel_circ_003345 | ENSSSCG00000022430 | 16 | + | 47314813 | 47318175 | 558 | annot_exons |
| novel_circ_003346 | ENSSSCG00000030326 | 2 | - | 27885606 | 27899766 | 441 | annot_exons |
| novel_circ_003347 | ENSSSCG00000008772 | 8 | - | 29151598 | 29155524 | 297 | annot_exons |
| novel_circ_003348 | ENSSSCG00000011514 | 13 | + | 51386726 | 51412393 | 825 | annot_exons |
| novel_circ_003349 | ENSSSCG00000017357 | 12 | + | 18820583 | 18832945 | 299 | annot_exons |
| novel_circ_003350 | ENSSSCG00000015586 | 9 | - | 1.3E+08 | 1.3E+08 | 222 | one_exon |
| novel_circ_003351 | ENSSSCG00000012119 | X | + | 9651374 | 9651595 | 222 | one_exon |
| novel_circ_003352 | ENSSSCG00000026152 | 6 | - | 52584896 | 52585202 | 232 | annot_exons |
| novel_circ_003353 | ENSSSCG00000021746 | 3 | - | 58565462 | 58566706 | 335 | annot_exons |
| novel_circ_003354 | ENSSSCG00000013082 | 2 | - | 10081948 | 10082649 | 334 | annot_exons |
| novel_circ_003355 | ENSSSCG00000012181 | X | + | 20600203 | 20610903 | 9126 | exon_intron |
| novel_circ_003356 | ENSSSCG00000010941 | 10 | - | 26180874 | 26212860 | 1253 | annot_exons |
| novel_circ_003357 | ENSSSCG00000016976 | 16 | - | 48837089 | 48849496 | 11112 | exon_intron |
| novel_circ_003358 | ENSSSCG00000017546 | 12 | - | 25503482 | 25504734 | 409 | annot_exons |
| novel_circ_003359 | ENSSSCG00000004065 | 1 | - | 11798732 | 11799068 | 337 | exon_intron |
| novel_circ_003360 | ENSSSCG00000015247 | 9 | - | 56957485 | 56973685 | 1623 | annot_exons |
| novel_circ_003361 | ENSSSCG00000003642 | 6 | - | 93891053 | 93895849 | 513 | annot_exons |
| novel_circ_003362 | ENSSSCG00000033228 | 12 | - | 27298750 | 27305281 | 246 | annot_exons |
| novel_circ_003363 | ENSSSCG00000031901 | 8 | + | 1.14E+08 | 1.14E+08 | 494 | annot_exons |
| novel_circ_003364 | ENSSSCG00000020663 | 18 | + | 5183032 | 5190667 | 444 | annot_exons |
| novel_circ_003365 | ENSSSCG00000036948 | 10 | + | 23644902 | 23645053 | 152 | intronic |
| novel_circ_003366 | ENSSSCG00000016406 | 18 | + | 612442 | 620550 | 616 | annot_exons |
| novel_circ_003367 | ENSSSCG00000023660 | 10 | - | 14217434 | 14218735 | 395 | annot_exons |
| novel_circ_003368 | ENSSSCG00000010698 | 14 | - | 1.32E+08 | 1.32E+08 | 12764 | intronic |
| novel_circ_003369 | ENSSSCG00000004318 | 1 | - | 57390866 | 57393736 | 458 | annot_exons |
| novel_circ_003370 | ENSSSCG00000017043 | 16 | + | 64266577 | 64300665 | 937 | annot_exons |
| novel_circ_003371 | ENSSSCG00000008179 | 3 | + | 54783404 | 54816635 | 725 | annot_exons |
| novel_circ_003372 | ENSSSCG00000011848 | 13 | + | 1.34E+08 | 1.34E+08 | 278 | annot_exons |
| novel_circ_003373 | ENSSSCG00000001806 | 7 | + | 52165917 | 52167154 | 817 | exon_intron |
| novel_circ_003374 | ENSSSCG00000023273 | 3 | + | 1.33E+08 | 1.33E+08 | 739 | annot_exons |
| novel_circ_003375 | ENSSSCG00000032390 | 18 | - | 9133515 | 9140760 | 2258 | annot_exons |
| novel_circ_003376 | ENSSSCG00000008487 | 3 | + | 1.02E+08 | 1.02E+08 | 245 | one_exon |
| novel_circ_003377 | ENSSSCG00000039843 | 10 | + | 24129789 | 24133305 | 407 | annot_exons |
| novel_circ_003378 | ENSSSCG00000036115 | 3 | + | 74066316 | 74067423 | 1108 | one_exon |
| novel_circ_003379 | ENSSSCG00000011540 | 13 | + | 65815438 | 65824773 | 1773 | annot_exons |
| novel_circ_003380 | ENSSSCG00000008621 | 3 | + | 1.22E+08 | 1.22E+08 | 238 | annot_exons |
| novel_circ_003381 | ENSSSCG00000010485 | 14 | + | 1.06E+08 | 1.06E+08 | 380 | annot_exons |
| novel_circ_003382 | ENSSSCG00000004653 | 1 | - | 1.23E+08 | 1.23E+08 | 278 | antisense |
| novel_circ_003383 | ENSSSCG00000008581 | 3 | - | 1.14E+08 | 1.14E+08 | 35222 | exon_intron |
| novel_circ_003384 | ENSSSCG00000009150 | 8 | - | 1.14E+08 | 1.14E+08 | 601 | annot_exons |
| novel_circ_003385 | ENSSSCG00000012463 | X | - | 68890168 | 68896588 | 260 | annot_exons |
| novel_circ_003386 | ENSSSCG00000032357 | 13 | + | 39101860 | 39108410 | 336 | annot_exons |
| novel_circ_003387 | ENSSSCG00000031775 | 5 | + | 73423661 | 73424027 | 367 | antisense |
| novel_circ_003388 | ENSSSCG00000015844 | 15 | + | 54405977 | 54416820 | 422 | annot_exons |
| novel_circ_003389 | ENSSSCG00000015097 | 9 | - | 46041865 | 46044924 | 365 | annot_exons |
| novel_circ_003390 | ENSSSCG00000002041 | 7 | + | 76235901 | 76236181 | 281 | one_exon |
| novel_circ_003391 | ENSSSCG00000015874 | 15 | - | 64833118 | 64834650 | 245 | annot_exons |
| novel_circ_003392 | ENSSSCG00000015792 | 15 | - | 46425015 | 46430362 | 512 | annot_exons |
| novel_circ_003393 | ENSSSCG00000008132 | 3 | + | 47006767 | 47007039 | 194 | annot_exons |
| novel_circ_003394 | ENSSSCG00000038870 | AEMK02000510.1 | - | 631195 | 631791 | 597 | exon_intron |
| novel_circ_003395 | ENSSSCG00000025942 | 13 | - | 1.21E+08 | 1.21E+08 | 184 | annot_exons |
| novel_circ_003396 | ENSSSCG00000006646 | 4 | + | 98369520 | 98372553 | 469 | annot_exons |
| novel_circ_003397 | ENSSSCG00000015667 | 15 | - | 3913056 | 3943647 | 312 | annot_exons |
| novel_circ_003398 | NA | 3 | - | 85583284 | 85584548 | 1265 | intergenic |
| novel_circ_003399 | ENSSSCG00000014915 | 9 | + | 20136920 | 20144395 | 500 | annot_exons |
| novel_circ_003400 | ENSSSCG00000009522 | 11 | + | 68934898 | 68943733 | 195 | annot_exons |
| novel_circ_003401 | ENSSSCG00000009172 | 8 | + | 1.2E+08 | 1.2E+08 | 2484 | exon_intron |
| novel_circ_003402 | ENSSSCG00000003591 | 6 | - | 87615275 | 87630082 | 726 | annot_exons |
| novel_circ_003403 | ENSSSCG00000016113 | 15 | + | 1.06E+08 | 1.06E+08 | 434 | annot_exons |
| novel_circ_003404 | ENSSSCG00000024088 | 1 | - | 1.09E+08 | 1.09E+08 | 420 | annot_exons |
| novel_circ_003405 | ENSSSCG00000000579 | 5 | - | 51945167 | 51947065 | 421 | annot_exons |
| novel_circ_003406 | ENSSSCG00000006100 | 4 | + | 42182675 | 42189817 | 565 | annot_exons |
| novel_circ_003407 | ENSSSCG00000022112 | 11 | - | 67900649 | 67925050 | 305 | annot_exons |
| novel_circ_003408 | ENSSSCG00000015443 | 9 | + | 1.08E+08 | 1.08E+08 | 608 | annot_exons |
| novel_circ_003409 | ENSSSCG00000011025 | 10 | + | 42063522 | 42079536 | 16015 | intronic |
| novel_circ_003410 | ENSSSCG00000006174 | 4 | + | 61715333 | 61715686 | 354 | one_exon |
| novel_circ_003411 | ENSSSCG00000017516 | 12 | - | 24174093 | 24180724 | 6632 | antisense |
| novel_circ_003412 | ENSSSCG00000016639 | 18 | - | 31365101 | 31387218 | 1228 | annot_exons |
| novel_circ_003413 | ENSSSCG00000033692 | 14 | + | 55783529 | 55810812 | 790 | annot_exons |
| novel_circ_003414 | ENSSSCG00000042910 | 6 | + | 91974319 | 91985640 | 829 | annot_exons |
| novel_circ_003415 | ENSSSCG00000031382 | 1 | - | 2.28E+08 | 2.28E+08 | 2747 | exon_intron |
| novel_circ_003416 | ENSSSCG00000037977 | 3 | - | 69377498 | 69401023 | 2866 | annot_exons |
| novel_circ_003417 | ENSSSCG00000007080 | 17 | - | 24916807 | 24962373 | 297 | annot_exons |
| novel_circ_003418 | ENSSSCG00000040366 | 1 | - | 2.04E+08 | 2.04E+08 | 572 | annot_exons |
| novel_circ_003419 | ENSSSCG00000005459 | 1 | - | 2.52E+08 | 2.52E+08 | 195 | annot_exons |
| novel_circ_003420 | ENSSSCG00000030467 | 13 | - | 1.09E+08 | 1.09E+08 | 411 | annot_exons |
| novel_circ_003421 | ENSSSCG00000007845 | 3 | - | 24201531 | 24201807 | 277 | intronic |
| novel_circ_003422 | ENSSSCG00000029169 | 1 | - | 2.66E+08 | 2.66E+08 | 750 | annot_exons |
| novel_circ_003423 | ENSSSCG00000022982 | 9 | + | 73146003 | 73156356 | 419 | annot_exons |
| novel_circ_003424 | ENSSSCG00000011497 | 13 | - | 46987049 | 47002023 | 389 | annot_exons |
| novel_circ_003425 | ENSSSCG00000038187 | 9 | + | 77536361 | 77537028 | 668 | one_exon |
| novel_circ_003426 | ENSSSCG00000015266 | 9 | + | 63913243 | 63915598 | 329 | annot_exons |
| novel_circ_003427 | ENSSSCG00000005459 | 1 | - | 2.52E+08 | 2.52E+08 | 356 | annot_exons |
| novel_circ_003428 | ENSSSCG00000010209 | 14 | - | 62644195 | 62645991 | 350 | annot_exons |
| novel_circ_003429 | ENSSSCG00000003917 | 6 | + | 1.66E+08 | 1.66E+08 | 318 | annot_exons |
| novel_circ_003430 | ENSSSCG00000039157 | 15 | + | 29959468 | 29963908 | 303 | annot_exons |
| novel_circ_003431 | ENSSSCG00000014199 | 2 | + | 1.16E+08 | 1.16E+08 | 432 | annot_exons |
| novel_circ_003432 | ENSSSCG00000028771 | 1 | + | 1.38E+08 | 1.38E+08 | 2287 | exon_intron |
| novel_circ_003433 | ENSSSCG00000002333 | 7 | - | 96061742 | 96083316 | 217 | annot_exons |
| novel_circ_003434 | ENSSSCG00000016234 | 15 | - | 1.26E+08 | 1.26E+08 | 357 | annot_exons |
| novel_circ_003435 | ENSSSCG00000011121 | 10 | + | 60703495 | 60705742 | 2248 | antisense |
| novel_circ_003436 | ENSSSCG00000011720 | 13 | + | 92652259 | 92662887 | 483 | annot_exons |
| novel_circ_003437 | ENSSSCG00000025478 | 6 | + | 1.07E+08 | 1.07E+08 | 1078 | annot_exons |
| novel_circ_003438 | ENSSSCG00000020663 | 18 | + | 5103837 | 5109435 | 544 | annot_exons |
| novel_circ_003439 | ENSSSCG00000009479 | 11 | - | 51393621 | 51394378 | 256 | annot_exons |
| novel_circ_003440 | ENSSSCG00000012642 | X | + | 1.02E+08 | 1.02E+08 | 523 | annot_exons |
| novel_circ_003441 | ENSSSCG00000037334 | AEMK02000452.1 | + | 3015167 | 3021595 | 881 | annot_exons |
| novel_circ_003442 | ENSSSCG00000026665 | 12 | - | 37490006 | 37517080 | 788 | exon_intron |
| novel_circ_003443 | ENSSSCG00000021393 | 9 | - | 1.26E+08 | 1.26E+08 | 654 | annot_exons |
| novel_circ_003444 | ENSSSCG00000002523 | AEMK02000452.1 | - | 1657853 | 1658513 | 224 | annot_exons |
| novel_circ_003445 | ENSSSCG00000029119 | 17 | + | 62830429 | 62837542 | 658 | annot_exons |
| novel_circ_003446 | ENSSSCG00000021749 | 13 | - | 1.21E+08 | 1.21E+08 | 543 | annot_exons |
| novel_circ_003447 | ENSSSCG00000033791 | 4 | + | 54948974 | 54956465 | 391 | annot_exons |
| novel_circ_003448 | ENSSSCG00000017812 | 12 | + | 47197656 | 47204903 | 508 | annot_exons |
| novel_circ_003449 | ENSSSCG00000015931 | 15 | + | 75855393 | 75858997 | 327 | annot_exons |
| novel_circ_003450 | ENSSSCG00000014410 | 2 | + | 1.47E+08 | 1.47E+08 | 366 | annot_exons |
| novel_circ_003451 | ENSSSCG00000000078 | 5 | - | 8104155 | 8113408 | 602 | annot_exons |
| novel_circ_003452 | ENSSSCG00000016948 | 16 | - | 44122165 | 44136109 | 800 | annot_exons |
| novel_circ_003453 | ENSSSCG00000028805 | 6 | - | 99218645 | 99229294 | 8372 | exon_intron |
| novel_circ_003454 | ENSSSCG00000001532 | 7 | + | 30790168 | 30793853 | 414 | annot_exons |
| novel_circ_003455 | ENSSSCG00000012174 | X | - | 19987739 | 19995816 | 419 | annot_exons |
| novel_circ_003456 | NA | 6 | + | 47384311 | 47386320 | 2010 | intergenic |
| novel_circ_003457 | ENSSSCG00000015743 | 15 | - | 31364330 | 31387416 | 591 | annot_exons |
| novel_circ_003458 | ENSSSCG00000010571 | 14 | - | 1.13E+08 | 1.13E+08 | 657 | annot_exons |
| novel_circ_003459 | ENSSSCG00000015498 | 9 | - | 1.16E+08 | 1.16E+08 | 539 | annot_exons |
| novel_circ_003460 | ENSSSCG00000004071 | 1 | + | 12381442 | 12388087 | 341 | annot_exons |
| novel_circ_003461 | ENSSSCG00000008513 | 3 | - | 1.07E+08 | 1.07E+08 | 1093 | annot_exons |
| novel_circ_003462 | ENSSSCG00000003506 | 6 | + | 78877002 | 78882041 | 813 | annot_exons |
| novel_circ_003463 | ENSSSCG00000004929 | 1 | - | 1.63E+08 | 1.63E+08 | 345 | annot_exons |
| novel_circ_003464 | ENSSSCG00000029228 | 6 | - | 300690 | 301971 | 294 | annot_exons |
| novel_circ_003465 | ENSSSCG00000010148 | 14 | + | 55065406 | 55070918 | 204 | annot_exons |
| novel_circ_003466 | ENSSSCG00000014835 | 9 | - | 8466879 | 8474887 | 737 | annot_exons |
| novel_circ_003467 | ENSSSCG00000010313 | 14 | + | 76768345 | 76783205 | 736 | annot_exons |
| novel_circ_003468 | ENSSSCG00000014114 | 2 | + | 88354417 | 88372391 | 743 | annot_exons |
| novel_circ_003469 | ENSSSCG00000010278 | 14 | + | 74692246 | 74692411 | 166 | intronic |
| novel_circ_003470 | ENSSSCG00000011162 | 10 | + | 68671265 | 68688441 | 461 | annot_exons |
| novel_circ_003471 | ENSSSCG00000014838 | 9 | - | 8641102 | 8646907 | 406 | annot_exons |
| novel_circ_003472 | ENSSSCG00000018049 | 12 | - | 60370050 | 60376920 | 538 | annot_exons |
| novel_circ_003473 | ENSSSCG00000008591 | 3 | + | 1.15E+08 | 1.15E+08 | 578 | annot_exons |
| novel_circ_003474 | ENSSSCG00000000466 | 5 | + | 28907687 | 28917531 | 709 | annot_exons |
| novel_circ_003475 | NA | 15 | - | 84468377 | 84468499 | 123 | intergenic |
| novel_circ_003476 | ENSSSCG00000003660 | 6 | - | 95386908 | 95387875 | 481 | annot_exons |
| novel_circ_003477 | ENSSSCG00000002330 | 7 | + | 94567390 | 94608753 | 2746 | annot_exons |
| novel_circ_003478 | ENSSSCG00000016223 | 15 | + | 1.25E+08 | 1.25E+08 | 388 | annot_exons |
| novel_circ_003479 | ENSSSCG00000005191 | 1 | + | 2.09E+08 | 2.09E+08 | 343 | annot_exons |
| novel_circ_003480 | ENSSSCG00000022486 | 13 | + | 1.54E+08 | 1.54E+08 | 532 | annot_exons |
| novel_circ_003481 | ENSSSCG00000020663 | 18 | + | 5199558 | 5208471 | 462 | annot_exons |
| novel_circ_003482 | ENSSSCG00000016494 | 18 | + | 9058146 | 9066076 | 429 | annot_exons |
| novel_circ_003483 | ENSSSCG00000007454 | 17 | - | 49333807 | 49334286 | 480 | intronic |
| novel_circ_003484 | ENSSSCG00000004592 | 1 | + | 1.13E+08 | 1.13E+08 | 621 | annot_exons |
| novel_circ_003485 | ENSSSCG00000001880 | 7 | + | 58196071 | 58200499 | 399 | annot_exons |
| novel_circ_003486 | ENSSSCG00000006871 | 4 | - | 1.18E+08 | 1.18E+08 | 8690 | exon_intron |
| novel_circ_003487 | ENSSSCG00000033497 | 6 | - | 69071403 | 69124265 | 582 | annot_exons |
| novel_circ_003488 | ENSSSCG00000001065 | 7 | + | 12366352 | 12366698 | 347 | antisense |
| novel_circ_003489 | ENSSSCG00000010470 | 14 | - | 1.04E+08 | 1.04E+08 | 563 | annot_exons |
| novel_circ_003490 | ENSSSCG00000002528 | AEMK02000452.1 | + | 1174519 | 1174823 | 305 | one_exon |
| novel_circ_003491 | ENSSSCG00000002016 | 7 | - | 75550243 | 75552161 | 640 | annot_exons |
| novel_circ_003492 | ENSSSCG00000008753 | 8 | - | 16704056 | 16718458 | 520 | annot_exons |
| novel_circ_003493 | ENSSSCG00000016111 | 15 | - | 1.06E+08 | 1.06E+08 | 201 | antisense |
| novel_circ_003494 | ENSSSCG00000013893 | 2 | + | 59772500 | 59774513 | 2014 | antisense |
| novel_circ_003495 | ENSSSCG00000002505 | 7 | + | 1.18E+08 | 1.18E+08 | 465 | annot_exons |
| novel_circ_003496 | NA | 17 | - | 40625165 | 40625283 | 119 | intergenic |
| novel_circ_003497 | ENSSSCG00000034261 | 6 | + | 1.58E+08 | 1.58E+08 | 669 | annot_exons |
| novel_circ_003498 | ENSSSCG00000051610 | 18 | + | 17855522 | 17889795 | 351 | annot_exons |
| novel_circ_003499 | ENSSSCG00000010470 | 14 | - | 1.04E+08 | 1.04E+08 | 865 | exon_intron |
| novel_circ_003500 | ENSSSCG00000012112 | X | - | 7964438 | 8003493 | 685 | annot_exons |
| novel_circ_003501 | ENSSSCG00000003770 | 6 | - | 1.35E+08 | 1.35E+08 | 442 | annot_exons |
| novel_circ_003502 | ENSSSCG00000010485 | 14 | + | 1.06E+08 | 1.06E+08 | 605 | annot_exons |
| novel_circ_003503 | ENSSSCG00000009050 | 8 | + | 85468772 | 85472740 | 268 | annot_exons |
| novel_circ_003504 | ENSSSCG00000040881 | 6 | - | 20372142 | 20422382 | 39915 | exon_intron |
| novel_circ_003505 | ENSSSCG00000002461 | 7 | - | 1.15E+08 | 1.15E+08 | 434 | intronic |
| novel_circ_003506 | NA | 2 | - | 6751232 | 6751679 | 448 | intergenic |
| novel_circ_003507 | ENSSSCG00000014893 | 9 | - | 12905483 | 12918332 | 337 | annot_exons |
| novel_circ_003508 | ENSSSCG00000001780 | 7 | - | 49084251 | 49086368 | 2118 | antisense |
| novel_circ_003509 | ENSSSCG00000006927 | 4 | - | 1.28E+08 | 1.28E+08 | 633 | annot_exons |
| novel_circ_003510 | NA | 15 | + | 84236229 | 84236609 | 381 | intergenic |
| novel_circ_003511 | ENSSSCG00000010358 | 14 | - | 87661198 | 87682623 | 21426 | antisense |
| novel_circ_003512 | ENSSSCG00000010877 | 10 | - | 17478592 | 17481300 | 2142 | annot_exons |
| novel_circ_003513 | ENSSSCG00000025602 | 18 | + | 33663765 | 33673693 | 365 | annot_exons |
| novel_circ_003514 | ENSSSCG00000023793 | 9 | + | 91983343 | 91986364 | 330 | annot_exons |
| novel_circ_003515 | ENSSSCG00000008583 | 3 | - | 1.14E+08 | 1.14E+08 | 958 | antisense |
| novel_circ_003516 | ENSSSCG00000011843 | 13 | - | 1.33E+08 | 1.33E+08 | 577 | annot_exons |
| novel_circ_003517 | ENSSSCG00000016549 | 18 | - | 17554494 | 17558649 | 352 | annot_exons |
| novel_circ_003518 | ENSSSCG00000004349 | 1 | - | 66321461 | 66339064 | 975 | annot_exons |
| novel_circ_003519 | ENSSSCG00000017668 | 12 | + | 35952997 | 35999542 | 747 | annot_exons |
| novel_circ_003520 | ENSSSCG00000015747 | 15 | + | 33584669 | 33591650 | 2865 | annot_exons |
| novel_circ_003521 | ENSSSCG00000017274 | 12 | + | 14049645 | 14050487 | 213 | annot_exons |
| novel_circ_003522 | ENSSSCG00000049707 | 6 | - | 1.49E+08 | 1.49E+08 | 7732 | exon_intron |
| novel_circ_003523 | ENSSSCG00000010295 | 14 | - | 75970870 | 75975028 | 344 | annot_exons |
| novel_circ_003524 | ENSSSCG00000022989 | 4 | + | 56000649 | 56003370 | 1241 | annot_exons |
| novel_circ_003525 | ENSSSCG00000010212 | 14 | - | 63670134 | 63681591 | 399 | annot_exons |
| novel_circ_003526 | ENSSSCG00000030211 | 12 | - | 19760214 | 19768678 | 1714 | annot_exons |
| novel_circ_003527 | ENSSSCG00000016125 | 15 | - | 1.09E+08 | 1.09E+08 | 462 | annot_exons |
| novel_circ_003528 | ENSSSCG00000003465 | 6 | + | 74953243 | 74955605 | 297 | annot_exons |
| novel_circ_003529 | ENSSSCG00000015071 | 9 | - | 44436634 | 44441486 | 4853 | intronic |
| novel_circ_003530 | ENSSSCG00000024361 | 1 | + | 11584817 | 11596113 | 381 | annot_exons |
| novel_circ_003531 | ENSSSCG00000033334 | 15 | + | 1.32E+08 | 1.32E+08 | 570 | annot_exons |
| novel_circ_003532 | ENSSSCG00000015690 | 15 | - | 16184227 | 16197963 | 523 | annot_exons |
| novel_circ_003533 | ENSSSCG00000032007 | 3 | + | 86235066 | 86239754 | 347 | annot_exons |
| novel_circ_003534 | ENSSSCG00000000920 | 5 | + | 93158505 | 93187362 | 841 | annot_exons |
| novel_circ_003535 | ENSSSCG00000017678 | 12 | + | 37669216 | 37671584 | 357 | annot_exons |
| novel_circ_003536 | ENSSSCG00000020702 | 1 | - | 90238341 | 90255853 | 331 | annot_exons |
| novel_circ_003537 | ENSSSCG00000033217 | 3 | + | 4008731 | 4009628 | 773 | annot_exons |
| novel_circ_003538 | ENSSSCG00000040786 | 3 | - | 86594330 | 86595389 | 1060 | exon_intron |
| novel_circ_003539 | ENSSSCG00000009106 | 8 | + | 1.04E+08 | 1.04E+08 | 309 | annot_exons |
| novel_circ_003540 | ENSSSCG00000006391 | 4 | - | 90349228 | 90349460 | 233 | exon_intron |
| novel_circ_003541 | ENSSSCG00000027935 | 6 | + | 1.21E+08 | 1.21E+08 | 1178 | annot_exons |
| novel_circ_003542 | ENSSSCG00000022464 | 18 | + | 40616158 | 40634324 | 1578 | annot_exons |
| novel_circ_003543 | ENSSSCG00000029482 | 14 | - | 1.33E+08 | 1.33E+08 | 1674 | annot_exons |
| novel_circ_003544 | ENSSSCG00000006231 | 4 | - | 72630918 | 72643259 | 402 | annot_exons |
| novel_circ_003545 | ENSSSCG00000015115 | 9 | + | 46486910 | 46487508 | 513 | annot_exons |
| novel_circ_003546 | ENSSSCG00000035581 | 18 | - | 54260019 | 54266233 | 258 | annot_exons |
| novel_circ_003547 | ENSSSCG00000031441 | 5 | - | 2906978 | 2911166 | 332 | annot_exons |
| novel_circ_003548 | ENSSSCG00000012018 | 13 | + | 1.83E+08 | 1.83E+08 | 945 | annot_exons |
| novel_circ_003549 | ENSSSCG00000024180 | 10 | - | 14032327 | 14032661 | 335 | one_exon |
| novel_circ_003550 | ENSSSCG00000011213 | 13 | - | 12542442 | 12548099 | 627 | annot_exons |
| novel_circ_003551 | ENSSSCG00000001056 | 7 | - | 9980131 | 9992700 | 531 | annot_exons |
| novel_circ_003552 | ENSSSCG00000046809 | 6 | + | 47368460 | 47378974 | 10343 | exon_intron |
| novel_circ_003553 | ENSSSCG00000037561 | 11 | + | 78351385 | 78356609 | 318 | annot_exons |
| novel_circ_003554 | ENSSSCG00000011085 | 10 | - | 53197803 | 53202893 | 294 | annot_exons |
| novel_circ_003555 | ENSSSCG00000015311 | 9 | - | 72068514 | 72076914 | 578 | annot_exons |
| novel_circ_003556 | ENSSSCG00000033185 | 13 | + | 77065148 | 77075075 | 383 | annot_exons |
| novel_circ_003557 | ENSSSCG00000008499 | 3 | + | 1.03E+08 | 1.03E+08 | 346 | annot_exons |
| novel_circ_003558 | ENSSSCG00000004029 | 1 | + | 4739924 | 4740223 | 300 | antisense |
| novel_circ_003559 | ENSSSCG00000015670 | 15 | + | 7751278 | 7759736 | 6811 | exon_intron |
| novel_circ_003560 | ENSSSCG00000002670 | 6 | - | 4003874 | 4015029 | 1192 | annot_exons |
| novel_circ_003561 | ENSSSCG00000005467 | 1 | + | 2.53E+08 | 2.53E+08 | 318 | annot_exons |
| novel_circ_003562 | ENSSSCG00000016045 | 15 | + | 94439886 | 94440125 | 240 | intronic |
| novel_circ_003563 | ENSSSCG00000022168 | 8 | - | 31935344 | 31937724 | 203 | annot_exons |
| novel_circ_003564 | ENSSSCG00000022895 | 13 | + | 1.62E+08 | 1.62E+08 | 210 | antisense |
| novel_circ_003565 | ENSSSCG00000006779 | 4 | + | 1.08E+08 | 1.08E+08 | 368 | annot_exons |
| novel_circ_003566 | ENSSSCG00000024754 | 18 | - | 40140465 | 40143799 | 326 | annot_exons |
| novel_circ_003567 | ENSSSCG00000023738 | 11 | - | 21856562 | 21860092 | 307 | annot_exons |
| novel_circ_003568 | ENSSSCG00000009021 | 8 | + | 78370924 | 78381727 | 409 | annot_exons |
| novel_circ_003569 | ENSSSCG00000006192 | 4 | + | 64724977 | 64741174 | 306 | annot_exons |
| novel_circ_003570 | ENSSSCG00000016157 | 15 | - | 1.13E+08 | 1.13E+08 | 309 | one_exon |
| novel_circ_003571 | ENSSSCG00000009305 | 11 | + | 4867556 | 4867966 | 411 | one_exon |
| novel_circ_003572 | ENSSSCG00000015242 | 9 | - | 56641051 | 56641890 | 277 | annot_exons |
| novel_circ_003573 | ENSSSCG00000013315 | 2 | - | 28260377 | 28265877 | 410 | annot_exons |
| novel_circ_003574 | ENSSSCG00000031808 | 11 | + | 68458898 | 68463198 | 424 | annot_exons |
| novel_circ_003575 | ENSSSCG00000013366 | 2 | - | 40817322 | 40818287 | 292 | annot_exons |
| novel_circ_003576 | ENSSSCG00000009994 | 14 | + | 46886930 | 46973910 | 458 | annot_exons |
| novel_circ_003577 | ENSSSCG00000002535 | AEMK02000452.1 | + | 917253 | 917583 | 331 | antisense |
| novel_circ_003578 | ENSSSCG00000000039 | 5 | + | 6191582 | 6195200 | 388 | annot_exons |
| novel_circ_003579 | ENSSSCG00000017125 | 12 | - | 580548 | 585916 | 490 | annot_exons |
| novel_circ_003580 | ENSSSCG00000034191 | 2 | + | 42552692 | 42572613 | 157 | annot_exons |
| novel_circ_003581 | ENSSSCG00000004524 | 1 | + | 1.01E+08 | 1.01E+08 | 796 | annot_exons |
| novel_circ_003582 | ENSSSCG00000008097 | 3 | - | 44008062 | 44018329 | 312 | annot_exons |
| novel_circ_003583 | ENSSSCG00000008009 | 3 | + | 40901263 | 40917549 | 392 | annot_exons |
| novel_circ_003584 | ENSSSCG00000034191 | 2 | + | 42688581 | 42715717 | 449 | annot_exons |
| novel_circ_003585 | NA | 15 | + | 84271791 | 84271996 | 206 | intergenic |
| novel_circ_003586 | ENSSSCG00000008829 | 8 | + | 38752932 | 38765908 | 12977 | antisense |
| novel_circ_003587 | ENSSSCG00000027669 | 15 | - | 1.2E+08 | 1.2E+08 | 628 | annot_exons |
| novel_circ_003588 | ENSSSCG00000001499 | 7 | + | 29178698 | 29184512 | 694 | annot_exons |
| novel_circ_003589 | ENSSSCG00000023329 | 2 | + | 77014673 | 77016142 | 1470 | antisense |
| novel_circ_003590 | ENSSSCG00000015213 | 9 | + | 52743673 | 52750537 | 598 | annot_exons |
| novel_circ_003591 | ENSSSCG00000008395 | 3 | - | 83499674 | 83534429 | 261 | annot_exons |
| novel_circ_003592 | ENSSSCG00000030467 | 13 | - | 1.09E+08 | 1.09E+08 | 402 | annot_exons |
| novel_circ_003593 | ENSSSCG00000008961 | 8 | + | 70193530 | 70204678 | 460 | annot_exons |
| novel_circ_003594 | ENSSSCG00000022672 | 6 | + | 54358003 | 54358636 | 468 | annot_exons |
| novel_circ_003595 | ENSSSCG00000036480 | 14 | - | 70489004 | 70489895 | 438 | annot_exons |
| novel_circ_003596 | ENSSSCG00000024453 | 13 | - | 68595006 | 68624887 | 1313 | annot_exons |
| novel_circ_003597 | ENSSSCG00000003812 | 6 | - | 1.49E+08 | 1.49E+08 | 407 | annot_exons |
| novel_circ_003598 | ENSSSCG00000015844 | 15 | + | 54402949 | 54416820 | 511 | annot_exons |
| novel_circ_003599 | ENSSSCG00000024663 | 13 | + | 1.46E+08 | 1.46E+08 | 501 | annot_exons |
| novel_circ_003600 | ENSSSCG00000014269 | 2 | - | 1.34E+08 | 1.34E+08 | 338 | annot_exons |
| novel_circ_003601 | ENSSSCG00000017260 | 12 | + | 11721129 | 11723627 | 253 | annot_exons |
| novel_circ_003602 | ENSSSCG00000006508 | 4 | - | 94351645 | 94351875 | 231 | antisense |
| novel_circ_003603 | NA | 15 | - | 84275200 | 84275418 | 219 | intergenic |
| novel_circ_003604 | ENSSSCG00000010877 | 10 | - | 17491117 | 17491728 | 213 | annot_exons |
| novel_circ_003605 | ENSSSCG00000015795 | 15 | - | 46524570 | 46525060 | 491 | intronic |
| novel_circ_003606 | ENSSSCG00000014838 | 9 | - | 8703167 | 8709627 | 1077 | annot_exons |
| novel_circ_003607 | ENSSSCG00000026996 | 11 | - | 64228871 | 64233867 | 384 | annot_exons |
| novel_circ_003608 | ENSSSCG00000016118 | 15 | + | 1.07E+08 | 1.07E+08 | 443 | annot_exons |
| novel_circ_003609 | ENSSSCG00000040603 | 16 | - | 44194905 | 44214957 | 379 | annot_exons |
| novel_circ_003610 | ENSSSCG00000039793 | 6 | - | 1.5E+08 | 1.5E+08 | 381 | annot_exons |
| novel_circ_003611 | ENSSSCG00000015566 | 9 | - | 1.26E+08 | 1.26E+08 | 395 | annot_exons |
| novel_circ_003612 | NA | X | - | 29401315 | 29405271 | 3957 | intergenic |
| novel_circ_003613 | ENSSSCG00000015247 | 9 | - | 56972613 | 56973685 | 1073 | one_exon |
| novel_circ_003614 | ENSSSCG00000026130 | 13 | - | 1.67E+08 | 1.67E+08 | 661 | one_exon |
| novel_circ_003615 | ENSSSCG00000032556 | 3 | + | 18259909 | 18260099 | 191 | one_exon |
| novel_circ_003616 | ENSSSCG00000015599 | 9 | - | 1.31E+08 | 1.31E+08 | 746 | annot_exons |
| novel_circ_003617 | ENSSSCG00000042910 | 6 | + | 91991606 | 91997101 | 614 | annot_exons |
| novel_circ_003618 | ENSSSCG00000008991 | 8 | + | 73830830 | 73842173 | 450 | annot_exons |
| novel_circ_003619 | ENSSSCG00000003805 | 6 | - | 1.46E+08 | 1.46E+08 | 1075 | annot_exons |
| novel_circ_003620 | ENSSSCG00000025703 | 1 | - | 77267171 | 77270404 | 421 | annot_exons |
| novel_circ_003621 | ENSSSCG00000015747 | 15 | + | 33541562 | 33555194 | 881 | annot_exons |
| novel_circ_003622 | ENSSSCG00000005689 | 1 | - | 2.7E+08 | 2.7E+08 | 198 | one_exon |
| novel_circ_003623 | ENSSSCG00000016085 | 15 | - | 1.03E+08 | 1.03E+08 | 249 | annot_exons |
| novel_circ_003624 | NA | 15 | - | 84466334 | 84468614 | 2281 | intergenic |
| novel_circ_003625 | ENSSSCG00000033591 | 6 | - | 31978481 | 31980100 | 1620 | one_exon |
| novel_circ_003626 | ENSSSCG00000001523 | 7 | - | 30190812 | 30195429 | 1274 | annot_exons |
| novel_circ_003627 | ENSSSCG00000011258 | 13 | + | 23289447 | 23293830 | 263 | annot_exons |
| novel_circ_003628 | ENSSSCG00000012856 | 2 | - | 2076978 | 2089434 | 633 | annot_exons |
| novel_circ_003629 | ENSSSCG00000020663 | 18 | + | 5229294 | 5235676 | 2489 | annot_exons |
| novel_circ_003630 | ENSSSCG00000038870 | AEMK02000510.1 | - | 631357 | 663972 | 545 | annot_exons |
| novel_circ_003631 | ENSSSCG00000017759 | 12 | - | 44810012 | 44811503 | 312 | annot_exons |
| novel_circ_003632 | ENSSSCG00000002285 | 7 | + | 90751454 | 90794467 | 201 | annot_exons |
| novel_circ_003633 | ENSSSCG00000038947 | 7 | + | 77950751 | 77956163 | 441 | annot_exons |
| novel_circ_003634 | ENSSSCG00000020717 | 8 | - | 77351300 | 77351598 | 299 | intronic |
| novel_circ_003635 | ENSSSCG00000014794 | 9 | + | 6361824 | 6376708 | 556 | annot_exons |
| novel_circ_003636 | NA | 2 | - | 6751232 | 6751703 | 472 | intergenic |
| novel_circ_003637 | ENSSSCG00000017717 | 12 | - | 40021670 | 40029267 | 870 | annot_exons |
| novel_circ_003638 | ENSSSCG00000024570 | 2 | - | 73841767 | 73852681 | 263 | annot_exons |
| novel_circ_003639 | ENSSSCG00000011639 | 13 | + | 74991933 | 74998162 | 192 | annot_exons |
| novel_circ_003640 | ENSSSCG00000015498 | 9 | - | 1.16E+08 | 1.16E+08 | 497 | annot_exons |
| novel_circ_003641 | ENSSSCG00000025463 | 13 | + | 1.66E+08 | 1.66E+08 | 462 | annot_exons |
| novel_circ_003642 | ENSSSCG00000022112 | 11 | - | 67767430 | 67782441 | 945 | annot_exons |
| novel_circ_003643 | ENSSSCG00000007046 | 17 | - | 14782131 | 14782968 | 445 | annot_exons |
| novel_circ_003644 | ENSSSCG00000002841 | 6 | + | 36163931 | 36168591 | 336 | annot_exons |
| novel_circ_003645 | ENSSSCG00000007681 | 3 | - | 9343180 | 9344558 | 1379 | antisense |
| novel_circ_003646 | ENSSSCG00000001819 | 7 | + | 53513689 | 53516514 | 515 | annot_exons |
| novel_circ_003647 | ENSSSCG00000036428 | 3 | - | 28896463 | 28904794 | 564 | annot_exons |
| novel_circ_003648 | ENSSSCG00000035686 | 5 | - | 21672280 | 21673645 | 432 | annot_exons |
| novel_circ_003649 | ENSSSCG00000004535 | 1 | - | 1.05E+08 | 1.05E+08 | 1042 | annot_exons |
| novel_circ_003650 | ENSSSCG00000010358 | 14 | + | 87661152 | 87699150 | 877 | annot_exons |
| novel_circ_003651 | ENSSSCG00000007153 | 17 | - | 32142636 | 32143743 | 261 | annot_exons |
| novel_circ_003652 | ENSSSCG00000038607 | 13 | - | 17001137 | 17025521 | 5633 | annot_exons |
| novel_circ_003653 | ENSSSCG00000023434 | 13 | - | 1.01E+08 | 1.01E+08 | 1076 | antisense |
| novel_circ_003654 | ENSSSCG00000003511 | 6 | - | 78981505 | 78994000 | 631 | annot_exons |
| novel_circ_003655 | ENSSSCG00000033958 | 7 | - | 80770348 | 80773427 | 391 | annot_exons |
| novel_circ_003656 | ENSSSCG00000016845 | 16 | - | 22546397 | 22556122 | 584 | annot_exons |
| novel_circ_003657 | ENSSSCG00000032877 | 2 | + | 1.3E+08 | 1.3E+08 | 257 | annot_exons |
| novel_circ_003658 | ENSSSCG00000003559 | 6 | + | 84105757 | 84108248 | 812 | annot_exons |
| novel_circ_003659 | ENSSSCG00000010450 | 14 | - | 1.01E+08 | 1.01E+08 | 1459 | annot_exons |
| novel_circ_003660 | NA | 15 | - | 84288986 | 84289190 | 205 | intergenic |
| novel_circ_003661 | ENSSSCG00000036877 | 15 | - | 37953657 | 37954814 | 1158 | exon_intron |
| novel_circ_003662 | ENSSSCG00000017672 | 12 | + | 36415178 | 36415809 | 416 | annot_exons |
| novel_circ_003663 | ENSSSCG00000030108 | 17 | - | 50934085 | 50939846 | 661 | annot_exons |
| novel_circ_003664 | ENSSSCG00000028148 | X | - | 28332184 | 28352815 | 351 | annot_exons |
| novel_circ_003665 | ENSSSCG00000014406 | 2 | - | 1.47E+08 | 1.47E+08 | 423 | annot_exons |
| novel_circ_003666 | ENSSSCG00000011025 | 10 | + | 42061873 | 42147610 | 85738 | exon_intron |
| novel_circ_003667 | ENSSSCG00000015247 | 9 | + | 56949161 | 56949424 | 264 | antisense |
| novel_circ_003668 | ENSSSCG00000003194 | 6 | + | 54881829 | 54881997 | 169 | antisense |
| novel_circ_003669 | ENSSSCG00000022227 | 2 | + | 62312200 | 62313757 | 274 | annot_exons |
| novel_circ_003670 | ENSSSCG00000008105 | 3 | + | 44522989 | 44526970 | 527 | annot_exons |
| novel_circ_003671 | ENSSSCG00000004368 | 1 | - | 71546115 | 71556619 | 494 | annot_exons |
| novel_circ_003672 | ENSSSCG00000016125 | 15 | - | 1.09E+08 | 1.09E+08 | 1089 | annot_exons |
| novel_circ_003673 | ENSSSCG00000039541 | 6 | + | 586614 | 589023 | 389 | annot_exons |
| novel_circ_003674 | ENSSSCG00000004985 | 1 | + | 1.7E+08 | 1.7E+08 | 1084 | annot_exons |
| novel_circ_003675 | ENSSSCG00000012713 | X | - | 1.14E+08 | 1.14E+08 | 279 | annot_exons |
| novel_circ_003676 | ENSSSCG00000013742 | 2 | - | 66028179 | 66031860 | 2739 | exon_intron |
| novel_circ_003677 | ENSSSCG00000024131 | 1 | - | 1.13E+08 | 1.13E+08 | 246 | annot_exons |
| novel_circ_003678 | ENSSSCG00000020710 | 6 | + | 73196842 | 73206401 | 4498 | annot_exons |
| novel_circ_003679 | ENSSSCG00000015286 | 9 | - | 65844093 | 65847110 | 548 | annot_exons |
| novel_circ_003680 | ENSSSCG00000028249 | 11 | - | 1317565 | 1317882 | 228 | annot_exons |
| novel_circ_003681 | ENSSSCG00000000549 | 5 | - | 46042145 | 46043770 | 352 | annot_exons |
| novel_circ_003682 | ENSSSCG00000004490 | 1 | + | 94314527 | 94315177 | 651 | one_exon |
| novel_circ_003683 | ENSSSCG00000001697 | 7 | - | 39145040 | 39145327 | 288 | antisense |
| novel_circ_003684 | ENSSSCG00000006956 | 4 | + | 1038217 | 1067334 | 833 | annot_exons |
| novel_circ_003685 | ENSSSCG00000004538 | 1 | + | 1.06E+08 | 1.06E+08 | 1612 | annot_exons |
| novel_circ_003686 | ENSSSCG00000030460 | X | - | 1.12E+08 | 1.12E+08 | 412 | annot_exons |
| novel_circ_003687 | ENSSSCG00000010984 | 10 | + | 32511793 | 32520542 | 284 | annot_exons |
| novel_circ_003688 | ENSSSCG00000012435 | X | - | 61915168 | 61935991 | 660 | annot_exons |
| novel_circ_003689 | ENSSSCG00000009338 | 11 | + | 8627782 | 8630518 | 300 | annot_exons |
| novel_circ_003690 | ENSSSCG00000001710 | 7 | + | 40215305 | 40222732 | 262 | annot_exons |
| novel_circ_003691 | ENSSSCG00000040220 | 6 | + | 1.36E+08 | 1.36E+08 | 402 | annot_exons |
| novel_circ_003692 | ENSSSCG00000040997 | 14 | - | 94061884 | 94073749 | 1630 | annot_exons |
| novel_circ_003693 | ENSSSCG00000035581 | 18 | - | 54260019 | 54270584 | 384 | annot_exons |
| novel_circ_003694 | ENSSSCG00000028516 | 5 | + | 16309169 | 16328852 | 783 | annot_exons |
| novel_circ_003695 | ENSSSCG00000032067 | 6 | - | 89300591 | 89302018 | 269 | annot_exons |
| novel_circ_003696 | ENSSSCG00000035320 | 13 | - | 39134918 | 39138659 | 418 | annot_exons |
| novel_circ_003697 | ENSSSCG00000004524 | 1 | + | 1.01E+08 | 1.01E+08 | 1437 | annot_exons |
| novel_circ_003698 | ENSSSCG00000000901 | 5 | + | 88211724 | 88215871 | 481 | annot_exons |
| novel_circ_003699 | ENSSSCG00000016553 | 18 | + | 18274179 | 18276491 | 257 | annot_exons |
| novel_circ_003700 | ENSSSCG00000004012 | 1 | + | 874627 | 874861 | 235 | one_exon |
| novel_circ_003701 | ENSSSCG00000030396 | 8 | + | 87488517 | 87516224 | 880 | annot_exons |
| novel_circ_003702 | ENSSSCG00000029251 | 13 | - | 30685269 | 30691776 | 237 | annot_exons |
| novel_circ_003703 | ENSSSCG00000039157 | 15 | + | 29977370 | 30033227 | 2324 | annot_exons |
| novel_circ_003704 | ENSSSCG00000006496 | 4 | - | 93914749 | 93914941 | 193 | intronic |
| novel_circ_003705 | ENSSSCG00000010679 | 14 | - | 1.29E+08 | 1.29E+08 | 321 | annot_exons |
| novel_circ_003706 | ENSSSCG00000015755 | 15 | + | 37807312 | 37823387 | 7028 | exon_intron |
| novel_circ_003707 | ENSSSCG00000004684 | 1 | + | 1.27E+08 | 1.27E+08 | 524 | annot_exons |
| novel_circ_003708 | ENSSSCG00000003654 | 6 | + | 95171603 | 95174915 | 192 | annot_exons |
| novel_circ_003709 | NA | 15 | - | 84293265 | 84293567 | 303 | intergenic |
| novel_circ_003710 | ENSSSCG00000000708 | 5 | - | 64319080 | 64319313 | 234 | antisense |
| novel_circ_003711 | ENSSSCG00000007805 | 3 | + | 18555760 | 18555926 | 167 | one_exon |
| novel_circ_003712 | ENSSSCG00000000458 | 5 | + | 27184128 | 27202298 | 1396 | annot_exons |
| novel_circ_003713 | ENSSSCG00000016958 | 16 | + | 46446240 | 46446956 | 717 | one_exon |
| novel_circ_003714 | ENSSSCG00000006845 | 4 | - | 1.11E+08 | 1.11E+08 | 1281 | annot_exons |
| novel_circ_003715 | ENSSSCG00000015931 | 15 | + | 75876154 | 75876430 | 277 | one_exon |
| novel_circ_003716 | ENSSSCG00000028327 | 14 | - | 64367918 | 64368587 | 306 | annot_exons |
| novel_circ_003717 | ENSSSCG00000006196 | 4 | + | 65462158 | 65508255 | 41150 | exon_intron |
| novel_circ_003718 | ENSSSCG00000017504 | 12 | - | 22812395 | 22813863 | 317 | annot_exons |
| novel_circ_003719 | ENSSSCG00000007721 | 3 | + | 11771884 | 11794655 | 2622 | annot_exons |
| novel_circ_003720 | ENSSSCG00000004587 | 1 | + | 1.13E+08 | 1.13E+08 | 765 | annot_exons |
| novel_circ_003721 | ENSSSCG00000015023 | 9 | - | 39556377 | 39590095 | 539 | annot_exons |
| novel_circ_003722 | ENSSSCG00000029198 | 9 | + | 36477400 | 36477883 | 239 | annot_exons |
| novel_circ_003723 | ENSSSCG00000005170 | 1 | - | 2.03E+08 | 2.04E+08 | 469 | annot_exons |
| novel_circ_003724 | ENSSSCG00000032531 | 3 | + | 26683193 | 26697117 | 1328 | annot_exons |
| novel_circ_003725 | ENSSSCG00000003872 | 6 | + | 1.61E+08 | 1.61E+08 | 310 | annot_exons |
| novel_circ_003726 | ENSSSCG00000031903 | 2 | + | 1313053 | 1313162 | 110 | one_exon |
| novel_circ_003727 | ENSSSCG00000008215 | 3 | - | 57885445 | 57895613 | 574 | annot_exons |
| novel_circ_003728 | ENSSSCG00000036828 | 13 | + | 1.35E+08 | 1.35E+08 | 408 | annot_exons |
| novel_circ_003729 | NA | 15 | - | 84460823 | 84460982 | 160 | intergenic |
| novel_circ_003730 | ENSSSCG00000000167 | 5 | - | 13522123 | 13523181 | 325 | annot_exons |
| novel_circ_003731 | ENSSSCG00000006510 | 4 | + | 94216682 | 94222905 | 754 | annot_exons |
| novel_circ_003732 | ENSSSCG00000008510 | 3 | - | 1.06E+08 | 1.06E+08 | 1731 | annot_exons |
| novel_circ_003733 | ENSSSCG00000014411 | 2 | - | 1.47E+08 | 1.47E+08 | 334 | annot_exons |
| novel_circ_003734 | ENSSSCG00000023324 | 9 | + | 45784065 | 45786789 | 698 | annot_exons |
| novel_circ_003735 | ENSSSCG00000021333 | 6 | + | 47902555 | 47904173 | 330 | annot_exons |
| novel_circ_003736 | ENSSSCG00000026034 | 7 | - | 13404112 | 13412723 | 1710 | annot_exons |
| novel_circ_003737 | ENSSSCG00000006303 | 4 | - | 82990344 | 82995062 | 463 | annot_exons |
| novel_circ_003738 | ENSSSCG00000011760 | 13 | - | 1.15E+08 | 1.15E+08 | 264 | annot_exons |
| novel_circ_003739 | ENSSSCG00000003434 | 6 | + | 72032455 | 72037434 | 315 | annot_exons |
| novel_circ_003740 | ENSSSCG00000023445 | 18 | + | 40667328 | 40668272 | 945 | antisense |
| novel_circ_003741 | ENSSSCG00000004634 | 1 | - | 1.21E+08 | 1.21E+08 | 3193 | annot_exons |
| novel_circ_003742 | ENSSSCG00000005465 | 1 | - | 2.53E+08 | 2.53E+08 | 582 | annot_exons |
| novel_circ_003743 | NA | 6 | + | 97208865 | 97209241 | 377 | intergenic |
| novel_circ_003744 | ENSSSCG00000004854 | 1 | - | 1.45E+08 | 1.45E+08 | 610 | annot_exons |
| novel_circ_003745 | ENSSSCG00000015747 | 15 | + | 33579433 | 33581091 | 379 | annot_exons |
| novel_circ_003746 | ENSSSCG00000011229 | 13 | + | 17612080 | 17630364 | 1548 | annot_exons |
| novel_circ_003747 | ENSSSCG00000039673 | 12 | + | 19036291 | 19036424 | 134 | antisense |
| novel_circ_003748 | NA | 2 | - | 6810742 | 6810929 | 188 | intergenic |
| novel_circ_003749 | ENSSSCG00000012649 | X | - | 1.06E+08 | 1.06E+08 | 647 | annot_exons |
| novel_circ_003750 | ENSSSCG00000029251 | 13 | - | 30678033 | 30744517 | 1912 | annot_exons |
| novel_circ_003751 | ENSSSCG00000025941 | 1 | + | 45173527 | 45175843 | 771 | annot_exons |
| novel_circ_003752 | ENSSSCG00000011765 | 13 | + | 1.18E+08 | 1.18E+08 | 904 | annot_exons |
| novel_circ_003753 | ENSSSCG00000006873 | 4 | + | 1.18E+08 | 1.18E+08 | 247 | annot_exons |
| novel_circ_003754 | ENSSSCG00000004022 | 1 | + | 2497080 | 2498776 | 257 | annot_exons |
| novel_circ_003755 | ENSSSCG00000003913 | 6 | - | 1.65E+08 | 1.65E+08 | 6478 | intronic |
| novel_circ_003756 | ENSSSCG00000038128 | 6 | + | 7552153 | 7552741 | 589 | one_exon |
| novel_circ_003757 | ENSSSCG00000002536 | AEMK02000452.1 | - | 892857 | 894041 | 1185 | exon_intron |
| novel_circ_003758 | ENSSSCG00000034386 | 14 | + | 31718305 | 31736163 | 1553 | annot_exons |
| novel_circ_003759 | ENSSSCG00000004739 | 1 | - | 1.3E+08 | 1.3E+08 | 502 | annot_exons |
| novel_circ_003760 | ENSSSCG00000000512 | 5 | + | 35878817 | 35880247 | 199 | annot_exons |
| novel_circ_003761 | ENSSSCG00000026819 | 14 | + | 55341575 | 55343743 | 599 | annot_exons |
| novel_circ_003762 | ENSSSCG00000038397 | 4 | + | 2738960 | 2793098 | 899 | annot_exons |
| novel_circ_003763 | ENSSSCG00000016564 | 18 | + | 18903934 | 18923062 | 222 | annot_exons |
| novel_circ_003764 | ENSSSCG00000001646 | 7 | + | 37922045 | 37924159 | 241 | annot_exons |
| novel_circ_003765 | ENSSSCG00000008498 | 3 | + | 1.03E+08 | 1.03E+08 | 946 | annot_exons |
| novel_circ_003766 | ENSSSCG00000013050 | 2 | - | 8161577 | 8162069 | 277 | annot_exons |
| novel_circ_003767 | ENSSSCG00000037803 | 1 | - | 79793662 | 79794000 | 339 | antisense |
| novel_circ_003768 | ENSSSCG00000010947 | 10 | + | 27641998 | 27651062 | 372 | annot_exons |
| novel_circ_003769 | ENSSSCG00000025156 | X | - | 64344586 | 64346816 | 383 | annot_exons |
| novel_circ_003770 | ENSSSCG00000016338 | 15 | - | 1.38E+08 | 1.38E+08 | 218 | annot_exons |
| novel_circ_003771 | ENSSSCG00000003680 | 6 | - | 98564833 | 98566623 | 294 | annot_exons |
| novel_circ_003772 | ENSSSCG00000009464 | 11 | - | 47603604 | 47605644 | 323 | annot_exons |
| novel_circ_003773 | ENSSSCG00000039673 | 12 | - | 19038434 | 19038886 | 453 | exon_intron |
| novel_circ_003774 | NA | 6 | + | 47372669 | 47384422 | 11754 | intergenic |
| novel_circ_003775 | ENSSSCG00000016821 | 16 | - | 20018930 | 20020259 | 305 | annot_exons |
| novel_circ_003776 | ENSSSCG00000007231 | 17 | + | 35504077 | 35506063 | 452 | annot_exons |
| novel_circ_003777 | ENSSSCG00000012264 | X | + | 41096054 | 41116058 | 367 | annot_exons |
| novel_circ_003778 | ENSSSCG00000011068 | 10 | - | 48820847 | 48824559 | 377 | annot_exons |
| novel_circ_003779 | ENSSSCG00000023304 | 3 | - | 39288629 | 39289189 | 561 | antisense |
| novel_circ_003780 | ENSSSCG00000032556 | 3 | - | 18259293 | 18259621 | 329 | antisense |
| novel_circ_003781 | ENSSSCG00000002547 | AEMK02000452.1 | + | 2193313 | 2208500 | 794 | annot_exons |
| novel_circ_003782 | ENSSSCG00000039658 | 10 | + | 46979179 | 46984113 | 4935 | exon_intron |
| novel_circ_003783 | ENSSSCG00000017694 | 12 | - | 38741143 | 38744279 | 315 | annot_exons |
| novel_circ_003784 | ENSSSCG00000000862 | 5 | + | 82403730 | 82422198 | 568 | annot_exons |
| novel_circ_003785 | ENSSSCG00000022145 | 6 | - | 1.29E+08 | 1.29E+08 | 5869 | annot_exons |
| novel_circ_003786 | ENSSSCG00000015940 | 15 | + | 76197130 | 76198564 | 360 | annot_exons |
| novel_circ_003787 | ENSSSCG00000036236 | 8 | + | 1.12E+08 | 1.12E+08 | 530 | annot_exons |
| novel_circ_003788 | ENSSSCG00000035196 | 4 | - | 1.25E+08 | 1.25E+08 | 1005 | annot_exons |
| novel_circ_003789 | ENSSSCG00000012203 | X | - | 26646459 | 26656356 | 280 | annot_exons |
| novel_circ_003790 | ENSSSCG00000009693 | 14 | - | 14279606 | 14284893 | 5288 | intronic |
| novel_circ_003791 | ENSSSCG00000048791 | 15 | - | 20060255 | 20071789 | 1165 | annot_exons |
| novel_circ_003792 | ENSSSCG00000006694 | 4 | - | 99674928 | 99678366 | 1235 | annot_exons |
| novel_circ_003793 | ENSSSCG00000003465 | 6 | + | 74948584 | 74953421 | 349 | annot_exons |
| novel_circ_003794 | ENSSSCG00000035658 | 11 | - | 15711288 | 15726712 | 678 | annot_exons |
| novel_circ_003795 | ENSSSCG00000003976 | 6 | - | 1.7E+08 | 1.7E+08 | 2777 | annot_exons |
| novel_circ_003796 | ENSSSCG00000004071 | 1 | + | 12376070 | 12388087 | 401 | annot_exons |
| novel_circ_003797 | ENSSSCG00000006889 | 4 | + | 1.23E+08 | 1.23E+08 | 551 | annot_exons |
| novel_circ_003798 | ENSSSCG00000035581 | 18 | - | 54192244 | 54260139 | 357 | annot_exons |
| novel_circ_003799 | ENSSSCG00000012642 | X | + | 1.02E+08 | 1.02E+08 | 426 | annot_exons |
| novel_circ_003800 | ENSSSCG00000000167 | 5 | + | 13439840 | 13488450 | 48611 | antisense |
| novel_circ_003801 | ENSSSCG00000008569 | 3 | - | 1.13E+08 | 1.13E+08 | 245 | annot_exons |
| novel_circ_003802 | ENSSSCG00000000466 | 5 | + | 28832466 | 28858296 | 2695 | annot_exons |
| novel_circ_003803 | ENSSSCG00000033741 | 6 | - | 17642766 | 17644074 | 1103 | annot_exons |
| novel_circ_003804 | ENSSSCG00000016769 | 18 | - | 54393586 | 54404485 | 1142 | annot_exons |
| novel_circ_003805 | ENSSSCG00000005343 | 1 | - | 2.37E+08 | 2.37E+08 | 269 | annot_exons |
| novel_circ_003806 | ENSSSCG00000032673 | 6 | + | 45994478 | 46002431 | 313 | annot_exons |
| novel_circ_003807 | ENSSSCG00000029464 | 5 | - | 67679713 | 67690214 | 875 | annot_exons |
| novel_circ_003808 | ENSSSCG00000009910 | 14 | + | 40502326 | 40502723 | 322 | annot_exons |
| novel_circ_003809 | ENSSSCG00000004725 | 1 | + | 1.29E+08 | 1.29E+08 | 3641 | annot_exons |
| novel_circ_003810 | ENSSSCG00000009134 | 8 | - | 1.12E+08 | 1.12E+08 | 363 | annot_exons |
| novel_circ_003811 | ENSSSCG00000017895 | 12 | - | 51651445 | 51654962 | 281 | annot_exons |
| novel_circ_003812 | ENSSSCG00000016981 | 16 | - | 50478647 | 50487975 | 9329 | exon_intron |
| novel_circ_003813 | ENSSSCG00000033954 | 7 | + | 55863609 | 55864157 | 295 | annot_exons |
| novel_circ_003814 | ENSSSCG00000000037 | 5 | + | 6010064 | 6011187 | 216 | annot_exons |
| novel_circ_003815 | ENSSSCG00000008072 | 3 | - | 42175984 | 42183873 | 711 | annot_exons |
| novel_circ_003816 | ENSSSCG00000015345 | 9 | + | 78246576 | 78312958 | 720 | annot_exons |
| novel_circ_003817 | ENSSSCG00000006219 | 4 | - | 68947011 | 68958236 | 1092 | annot_exons |
| novel_circ_003818 | ENSSSCG00000011499 | 13 | - | 47933287 | 47945326 | 810 | annot_exons |
| novel_circ_003819 | ENSSSCG00000010811 | 10 | + | 5777659 | 5797656 | 512 | annot_exons |
| novel_circ_003820 | ENSSSCG00000022190 | 4 | - | 79575474 | 79593731 | 18258 | exon_intron |
| novel_circ_003821 | ENSSSCG00000011363 | 13 | - | 31427503 | 31448147 | 956 | annot_exons |
| novel_circ_003822 | ENSSSCG00000036083 | 14 | + | 1.03E+08 | 1.03E+08 | 362 | annot_exons |
| novel_circ_003823 | ENSSSCG00000031616 | 6 | - | 51838945 | 51839695 | 751 | antisense |
| novel_circ_003824 | ENSSSCG00000006303 | 4 | - | 82993955 | 82995062 | 214 | annot_exons |
| novel_circ_003825 | ENSSSCG00000016708 | 18 | + | 45647051 | 45654745 | 237 | annot_exons |
| novel_circ_003826 | ENSSSCG00000000806 | 5 | - | 76839521 | 76841927 | 2407 | one_exon |
| novel_circ_003827 | ENSSSCG00000027403 | 8 | - | 55633660 | 55642319 | 5322 | annot_exons |
| novel_circ_003828 | ENSSSCG00000017513 | 12 | + | 23857979 | 23862768 | 441 | annot_exons |
| novel_circ_003829 | ENSSSCG00000004602 | 1 | - | 1.16E+08 | 1.16E+08 | 571 | annot_exons |
| novel_circ_003830 | NA | 15 | - | 84365150 | 84376623 | 11474 | intergenic |
| novel_circ_003831 | ENSSSCG00000014868 | 9 | + | 10688009 | 10701055 | 694 | annot_exons |
| novel_circ_003832 | ENSSSCG00000033106 | 3 | - | 92045274 | 92046871 | 351 | annot_exons |
| novel_circ_003833 | ENSSSCG00000039793 | 6 | - | 1.5E+08 | 1.5E+08 | 414 | annot_exons |
| novel_circ_003834 | ENSSSCG00000027529 | 9 | + | 33043520 | 33045820 | 1185 | annot_exons |
| novel_circ_003835 | ENSSSCG00000013362 | 2 | + | 40633194 | 40653859 | 867 | annot_exons |
| novel_circ_003836 | ENSSSCG00000020743 | 1 | + | 2.64E+08 | 2.64E+08 | 640 | annot_exons |
| novel_circ_003837 | ENSSSCG00000005507 | 1 | - | 2.61E+08 | 2.61E+08 | 335 | annot_exons |
| novel_circ_003838 | ENSSSCG00000016922 | 16 | + | 36266989 | 36267460 | 348 | annot_exons |
| novel_circ_003839 | ENSSSCG00000037451 | 2 | - | 3081630 | 3084833 | 450 | annot_exons |
| novel_circ_003840 | ENSSSCG00000016127 | 15 | - | 1.09E+08 | 1.09E+08 | 390 | annot_exons |
| novel_circ_003841 | ENSSSCG00000002799 | 6 | + | 20300809 | 20304994 | 4186 | antisense |
| novel_circ_003842 | ENSSSCG00000025703 | 1 | - | 77334729 | 77389841 | 55113 | exon_intron |
| novel_circ_003843 | ENSSSCG00000002266 | 7 | - | 86044906 | 86061957 | 812 | annot_exons |
| novel_circ_003844 | ENSSSCG00000010243 | 14 | + | 71844195 | 71861362 | 1271 | annot_exons |
| novel_circ_003845 | ENSSSCG00000015569 | 9 | + | 1.26E+08 | 1.26E+08 | 421 | annot_exons |
| novel_circ_003846 | ENSSSCG00000008339 | 3 | + | 73046115 | 73061319 | 786 | annot_exons |
| novel_circ_003847 | ENSSSCG00000016367 | 15 | - | 1.4E+08 | 1.4E+08 | 1762 | antisense |
| novel_circ_003848 | ENSSSCG00000015940 | 15 | + | 76356916 | 76357837 | 350 | annot_exons |
| novel_circ_003849 | ENSSSCG00000003642 | 6 | - | 93897569 | 93901963 | 357 | annot_exons |
| novel_circ_003850 | ENSSSCG00000004653 | 1 | + | 1.23E+08 | 1.23E+08 | 1011 | annot_exons |
| novel_circ_003851 | NA | 15 | - | 84267369 | 84267734 | 366 | intergenic |
| novel_circ_003852 | ENSSSCG00000012000 | 13 | + | 1.74E+08 | 1.74E+08 | 2894 | annot_exons |
| novel_circ_003853 | ENSSSCG00000005997 | 4 | + | 18980735 | 18980936 | 202 | antisense |
| novel_circ_003854 | ENSSSCG00000014304 | 2 | + | 1.37E+08 | 1.37E+08 | 340 | annot_exons |
| novel_circ_003855 | ENSSSCG00000005459 | 1 | - | 2.52E+08 | 2.52E+08 | 555 | annot_exons |
| novel_circ_003856 | ENSSSCG00000002429 | 7 | - | 1.11E+08 | 1.11E+08 | 439 | intronic |
| novel_circ_003857 | ENSSSCG00000003218 | 6 | - | 55302748 | 55308688 | 5941 | antisense |
| novel_circ_003858 | NA | 15 | - | 84259921 | 84260081 | 161 | intergenic |
| novel_circ_003859 | ENSSSCG00000005582 | 1 | - | 2.64E+08 | 2.64E+08 | 1892 | exon_intron |
| novel_circ_003860 | ENSSSCG00000002032 | 7 | - | 75920773 | 75920942 | 170 | antisense |
| novel_circ_003861 | ENSSSCG00000002142 | 7 | + | 78497590 | 78498065 | 283 | annot_exons |
| novel_circ_003862 | ENSSSCG00000009401 | 11 | - | 19256521 | 19264448 | 414 | annot_exons |
| novel_circ_003863 | ENSSSCG00000002267 | 7 | + | 85958397 | 85958911 | 515 | one_exon |
| novel_circ_003864 | ENSSSCG00000009535 | 11 | - | 74186742 | 74186897 | 156 | one_exon |
| novel_circ_003865 | ENSSSCG00000011933 | 13 | - | 1.49E+08 | 1.49E+08 | 909 | annot_exons |
| novel_circ_003866 | ENSSSCG00000022830 | 15 | - | 1.13E+08 | 1.13E+08 | 274 | annot_exons |
| novel_circ_003867 | ENSSSCG00000000298 | 5 | - | 19763019 | 19763170 | 152 | one_exon |
| novel_circ_003868 | ENSSSCG00000029538 | 9 | - | 39502835 | 39504434 | 380 | annot_exons |
| novel_circ_003869 | ENSSSCG00000004645 | 1 | + | 1.21E+08 | 1.21E+08 | 733 | annot_exons |
| novel_circ_003870 | ENSSSCG00000002724 | 6 | + | 13214838 | 13216077 | 396 | annot_exons |
| novel_circ_003871 | ENSSSCG00000011353 | 13 | - | 31250577 | 31250933 | 215 | annot_exons |
| novel_circ_003872 | ENSSSCG00000015288 | 9 | - | 65800961 | 65809568 | 1352 | annot_exons |
| novel_circ_003873 | ENSSSCG00000040510 | 13 | - | 30047562 | 30052780 | 498 | annot_exons |
| novel_circ_003874 | ENSSSCG00000021170 | 2 | - | 45527878 | 45543990 | 4021 | exon_intron |
| novel_circ_003875 | ENSSSCG00000014578 | 9 | + | 272910 | 312050 | 23920 | exon_intron |
| novel_circ_003876 | ENSSSCG00000027085 | 6 | + | 39368826 | 39369601 | 776 | antisense |
| novel_circ_003877 | ENSSSCG00000039658 | 10 | + | 46981022 | 46984113 | 3092 | exon_intron |
| novel_circ_003878 | ENSSSCG00000033763 | 1 | - | 2.31E+08 | 2.31E+08 | 184 | intronic |
| novel_circ_003879 | ENSSSCG00000023269 | 8 | + | 55194966 | 55210730 | 862 | annot_exons |
| novel_circ_003880 | ENSSSCG00000002455 | 7 | + | 1.14E+08 | 1.14E+08 | 448 | annot_exons |
| novel_circ_003881 | ENSSSCG00000015345 | 9 | + | 78300728 | 78312958 | 478 | annot_exons |
| novel_circ_003882 | ENSSSCG00000035564 | 15 | + | 15928996 | 15953002 | 1810 | annot_exons |
| novel_circ_003883 | ENSSSCG00000034191 | 2 | + | 42827361 | 42864865 | 34636 | exon_intron |
| novel_circ_003884 | ENSSSCG00000022464 | 18 | + | 40616158 | 40616672 | 515 | one_exon |
| novel_circ_003885 | ENSSSCG00000037439 | 9 | + | 46244047 | 46245174 | 231 | annot_exons |
| novel_circ_003886 | ENSSSCG00000009319 | 11 | + | 5587554 | 5602241 | 970 | annot_exons |
| novel_circ_003887 | ENSSSCG00000012591 | X | - | 92597675 | 92623219 | 1248 | annot_exons |
| novel_circ_003888 | ENSSSCG00000000908 | 5 | + | 89706497 | 89707045 | 382 | annot_exons |
| novel_circ_003889 | ENSSSCG00000008517 | 3 | - | 1.07E+08 | 1.07E+08 | 312 | annot_exons |
| novel_circ_003890 | ENSSSCG00000012266 | X | - | 41275981 | 41299010 | 936 | annot_exons |
| novel_circ_003891 | ENSSSCG00000027016 | 6 | - | 48627040 | 48629020 | 291 | annot_exons |
| novel_circ_003892 | ENSSSCG00000027765 | 1 | + | 1.75E+08 | 1.75E+08 | 529 | annot_exons |
| novel_circ_003893 | ENSSSCG00000037530 | 14 | + | 1.32E+08 | 1.32E+08 | 1862 | intronic |
| novel_circ_003894 | ENSSSCG00000005221 | 1 | + | 2.17E+08 | 2.17E+08 | 983 | annot_exons |
| novel_circ_003895 | ENSSSCG00000013017 | 2 | + | 7321147 | 7321487 | 341 | one_exon |
| novel_circ_003896 | ENSSSCG00000021811 | 3 | - | 18286224 | 18286443 | 112 | annot_exons |
| novel_circ_003897 | ENSSSCG00000016101 | 15 | + | 1.05E+08 | 1.05E+08 | 1023 | annot_exons |
| novel_circ_003898 | ENSSSCG00000008753 | 8 | - | 16716774 | 16743600 | 1114 | annot_exons |
| novel_circ_003899 | NA | 15 | - | 84471802 | 84471940 | 139 | intergenic |
| novel_circ_003900 | NA | 9 | + | 1.18E+08 | 1.18E+08 | 83494 | intergenic |
| novel_circ_003901 | ENSSSCG00000014343 | 2 | + | 1.41E+08 | 1.41E+08 | 552 | annot_exons |
| novel_circ_003902 | ENSSSCG00000004829 | 6 | - | 1.48E+08 | 1.48E+08 | 489 | annot_exons |
| novel_circ_003903 | ENSSSCG00000012035 | 13 | - | 1.96E+08 | 1.96E+08 | 334 | annot_exons |
| novel_circ_003904 | ENSSSCG00000016834 | 16 | - | 21437009 | 21437210 | 202 | intronic |
| novel_circ_003905 | ENSSSCG00000009464 | 11 | - | 47605553 | 47609973 | 4421 | exon_intron |
| novel_circ_003906 | ENSSSCG00000009917 | 14 | - | 40712179 | 40720760 | 401 | annot_exons |
| novel_circ_003907 | ENSSSCG00000024351 | 18 | - | 29473289 | 29474508 | 306 | annot_exons |
| novel_circ_003908 | ENSSSCG00000008961 | 8 | + | 70193530 | 70224123 | 1382 | annot_exons |
| novel_circ_003909 | ENSSSCG00000006033 | 4 | + | 29296187 | 29301149 | 266 | annot_exons |
| novel_circ_003910 | ENSSSCG00000036428 | 3 | - | 28903672 | 28904794 | 378 | annot_exons |
| novel_circ_003911 | ENSSSCG00000000784 | 5 | + | 71872463 | 71877431 | 759 | annot_exons |
| novel_circ_003912 | ENSSSCG00000014168 | 2 | - | 1.03E+08 | 1.03E+08 | 473 | annot_exons |
| novel_circ_003913 | ENSSSCG00000009569 | 11 | - | 534135 | 562934 | 862 | annot_exons |
| novel_circ_003914 | ENSSSCG00000039506 | 5 | - | 21543057 | 21543230 | 174 | antisense |
| novel_circ_003915 | ENSSSCG00000000862 | 5 | + | 82416126 | 82436342 | 568 | annot_exons |
| novel_circ_003916 | ENSSSCG00000005423 | 1 | - | 2.46E+08 | 2.46E+08 | 355 | annot_exons |
| novel_circ_003917 | ENSSSCG00000026744 | 11 | - | 1364578 | 1371291 | 225 | annot_exons |
| novel_circ_003918 | ENSSSCG00000011013 | 10 | + | 40010114 | 40017055 | 578 | annot_exons |
| novel_circ_003919 | ENSSSCG00000006866 | 4 | + | 1.18E+08 | 1.18E+08 | 437 | annot_exons |
| novel_circ_003920 | ENSSSCG00000009050 | 8 | + | 85167482 | 85200429 | 493 | annot_exons |
| novel_circ_003921 | ENSSSCG00000032097 | 5 | + | 59915033 | 59935574 | 894 | annot_exons |
| novel_circ_003922 | ENSSSCG00000017732 | 12 | - | 42587230 | 42590132 | 542 | annot_exons |
| novel_circ_003923 | ENSSSCG00000028148 | X | - | 28674023 | 28675535 | 314 | annot_exons |
| novel_circ_003924 | ENSSSCG00000031077 | 13 | - | 1E+08 | 1E+08 | 258 | annot_exons |
| novel_circ_003925 | ENSSSCG00000000037 | 5 | + | 6010064 | 6014815 | 304 | annot_exons |
| novel_circ_003926 | ENSSSCG00000003728 | 6 | - | 1.16E+08 | 1.16E+08 | 524 | exon_intron |
| novel_circ_003927 | ENSSSCG00000007187 | 17 | + | 33872308 | 33872531 | 224 | exon_intron |
| novel_circ_003928 | ENSSSCG00000010190 | 14 | + | 60415380 | 60415681 | 302 | one_exon |
| novel_circ_003929 | NA | 11 | - | 11000012 | 11001848 | 1837 | intergenic |
| novel_circ_003930 | ENSSSCG00000005997 | 4 | - | 18795587 | 18796063 | 268 | annot_exons |
| novel_circ_003931 | ENSSSCG00000030460 | X | - | 1.12E+08 | 1.12E+08 | 314 | annot_exons |
| novel_circ_003932 | ENSSSCG00000005997 | 4 | - | 19025344 | 19027950 | 321 | annot_exons |
| novel_circ_003933 | ENSSSCG00000027778 | 7 | - | 34085093 | 34089247 | 576 | annot_exons |
| novel_circ_003934 | ENSSSCG00000028724 | 13 | + | 1.92E+08 | 1.92E+08 | 680 | annot_exons |
| novel_circ_003935 | ENSSSCG00000005460 | 1 | + | 2.52E+08 | 2.52E+08 | 870 | intronic |
| novel_circ_003936 | ENSSSCG00000005126 | 1 | + | 1.96E+08 | 1.96E+08 | 791 | annot_exons |
| novel_circ_003937 | ENSSSCG00000005599 | 1 | + | 2.66E+08 | 2.66E+08 | 311 | annot_exons |
| novel_circ_003938 | ENSSSCG00000008513 | 3 | - | 1.07E+08 | 1.07E+08 | 3342 | annot_exons |
| novel_circ_003939 | ENSSSCG00000022479 | 4 | + | 34758248 | 34758471 | 224 | one_exon |
| novel_circ_003940 | NA | 4 | - | 28030432 | 28031622 | 1191 | intergenic |
| novel_circ_003941 | ENSSSCG00000004919 | 1 | - | 1.62E+08 | 1.62E+08 | 423 | annot_exons |
| novel_circ_003942 | ENSSSCG00000035564 | 15 | + | 15931074 | 15935249 | 673 | annot_exons |
| novel_circ_003943 | ENSSSCG00000006310 | 4 | - | 83624752 | 83637988 | 538 | annot_exons |
| novel_circ_003944 | ENSSSCG00000007056 | 17 | + | 17591384 | 17596265 | 307 | annot_exons |
| novel_circ_003945 | ENSSSCG00000035824 | 13 | - | 34779561 | 34780761 | 188 | annot_exons |
| novel_circ_003946 | ENSSSCG00000025478 | 6 | + | 1.07E+08 | 1.07E+08 | 1250 | annot_exons |
| novel_circ_003947 | ENSSSCG00000017877 | 12 | - | 50226122 | 50233618 | 621 | annot_exons |
| novel_circ_003948 | ENSSSCG00000006872 | 4 | - | 1.18E+08 | 1.18E+08 | 577 | annot_exons |
| novel_circ_003949 | ENSSSCG00000015692 | 15 | - | 16285931 | 16292039 | 551 | annot_exons |
| novel_circ_003950 | ENSSSCG00000017694 | 12 | - | 38762049 | 38763971 | 181 | annot_exons |
| novel_circ_003951 | ENSSSCG00000010702 | 14 | + | 1.32E+08 | 1.32E+08 | 368 | annot_exons |
| novel_circ_003952 | ENSSSCG00000011684 | 13 | + | 83714726 | 83721691 | 594 | annot_exons |
| novel_circ_003953 | ENSSSCG00000021309 | 10 | - | 43443984 | 43446589 | 402 | annot_exons |
| novel_circ_003954 | ENSSSCG00000006066 | 4 | + | 36710955 | 36716577 | 585 | annot_exons |
| novel_circ_003955 | ENSSSCG00000010313 | 14 | + | 76776802 | 76777877 | 239 | annot_exons |
| novel_circ_003956 | ENSSSCG00000015569 | 9 | + | 1.26E+08 | 1.26E+08 | 469 | annot_exons |
| novel_circ_003957 | ENSSSCG00000037029 | AEMK02000258.1 | + | 305028 | 306730 | 497 | annot_exons |
| novel_circ_003958 | ENSSSCG00000012591 | X | - | 92613898 | 92623219 | 907 | annot_exons |
| novel_circ_003959 | ENSSSCG00000037766 | 8 | + | 80596816 | 80598527 | 496 | annot_exons |
| novel_circ_003960 | ENSSSCG00000028431 | 2 | + | 1.23E+08 | 1.23E+08 | 660 | annot_exons |
| novel_circ_003961 | ENSSSCG00000011022 | 10 | + | 41355865 | 41356191 | 218 | annot_exons |
| novel_circ_003962 | ENSSSCG00000025901 | 3 | - | 1.09E+08 | 1.09E+08 | 5299 | exon_intron |
| novel_circ_003963 | ENSSSCG00000003218 | 6 | + | 55302398 | 55304287 | 425 | annot_exons |
| novel_circ_003964 | ENSSSCG00000034879 | 9 | - | 28120503 | 28122017 | 1515 | one_exon |
| novel_circ_003965 | ENSSSCG00000033987 | 15 | + | 86556414 | 86561911 | 606 | annot_exons |
| novel_circ_003966 | ENSSSCG00000045919 | 9 | + | 1.22E+08 | 1.22E+08 | 623 | exon_intron |
| novel_circ_003967 | ENSSSCG00000011803 | 13 | + | 1.25E+08 | 1.25E+08 | 170 | annot_exons |
| novel_circ_003968 | ENSSSCG00000003971 | 6 | + | 1.7E+08 | 1.7E+08 | 2751 | exon_intron |
| novel_circ_003969 | ENSSSCG00000004979 | 1 | - | 1.7E+08 | 1.7E+08 | 540 | annot_exons |
| novel_circ_003970 | ENSSSCG00000001004 | 7 | - | 2004297 | 2013361 | 645 | annot_exons |
| novel_circ_003971 | ENSSSCG00000010456 | 14 | - | 1.01E+08 | 1.01E+08 | 353 | one_exon |
| novel_circ_003972 | ENSSSCG00000027017 | 13 | + | 10767701 | 10778469 | 1257 | annot_exons |
| novel_circ_003973 | ENSSSCG00000006754 | 4 | + | 1.06E+08 | 1.06E+08 | 285 | annot_exons |
| novel_circ_003974 | ENSSSCG00000008498 | 3 | + | 1.03E+08 | 1.03E+08 | 297 | annot_exons |
| novel_circ_003975 | ENSSSCG00000029311 | 14 | + | 71310553 | 71311455 | 903 | one_exon |
| novel_circ_003976 | ENSSSCG00000007142 | 17 | - | 31807232 | 31817495 | 1034 | annot_exons |
| novel_circ_003977 | ENSSSCG00000015908 | 15 | - | 72431133 | 72450009 | 1018 | annot_exons |
| novel_circ_003978 | NA | 15 | + | 84246906 | 84247203 | 298 | intergenic |
| novel_circ_003979 | ENSSSCG00000000504 | 5 | - | 34739515 | 34743083 | 534 | annot_exons |
| novel_circ_003980 | ENSSSCG00000016234 | 15 | - | 1.26E+08 | 1.26E+08 | 419 | annot_exons |
| novel_circ_003981 | ENSSSCG00000006872 | 4 | - | 1.18E+08 | 1.18E+08 | 1392 | annot_exons |
| novel_circ_003982 | ENSSSCG00000023873 | 4 | + | 34250050 | 34251517 | 317 | annot_exons |
| novel_circ_003983 | ENSSSCG00000020799 | 3 | - | 1.27E+08 | 1.27E+08 | 2251 | exon_intron |
| novel_circ_003984 | ENSSSCG00000000486 | 5 | + | 32998512 | 33000625 | 249 | annot_exons |
| novel_circ_003985 | ENSSSCG00000009030 | 8 | + | 81082507 | 81083424 | 405 | annot_exons |
| novel_circ_003986 | ENSSSCG00000015586 | 9 | - | 1.3E+08 | 1.3E+08 | 666 | annot_exons |
| novel_circ_003987 | ENSSSCG00000015857 | 15 | - | 59180557 | 59185795 | 279 | annot_exons |
| novel_circ_003988 | ENSSSCG00000008629 | 3 | + | 1.25E+08 | 1.25E+08 | 1877 | exon_intron |
| novel_circ_003989 | ENSSSCG00000040731 | 4 | + | 15278145 | 15303961 | 398 | annot_exons |
| novel_circ_003990 | ENSSSCG00000037639 | 13 | - | 1.41E+08 | 1.41E+08 | 583 | annot_exons |
| novel_circ_003991 | ENSSSCG00000000509 | 5 | - | 35635564 | 35638764 | 491 | annot_exons |
| novel_circ_003992 | ENSSSCG00000011340 | 13 | - | 30524874 | 30547685 | 435 | annot_exons |
| novel_circ_003993 | ENSSSCG00000009334 | 11 | - | 7750444 | 7753831 | 364 | annot_exons |
| novel_circ_003994 | ENSSSCG00000004952 | 1 | + | 1.65E+08 | 1.65E+08 | 351 | annot_exons |
| novel_circ_003995 | ENSSSCG00000026499 | 10 | + | 46706438 | 46717523 | 477 | annot_exons |
| novel_circ_003996 | ENSSSCG00000016853 | 16 | - | 24213700 | 24221062 | 7117 | exon_intron |
| novel_circ_003997 | ENSSSCG00000009029 | 8 | - | 80928441 | 80928671 | 158 | annot_exons |
| novel_circ_003998 | ENSSSCG00000012743 | X | + | 1.22E+08 | 1.22E+08 | 294 | annot_exons |
| novel_circ_003999 | ENSSSCG00000017748 | 12 | - | 43623021 | 43624080 | 347 | annot_exons |
| novel_circ_004000 | ENSSSCG00000010706 | 6 | + | 48037867 | 48038991 | 429 | annot_exons |
| novel_circ_004001 | ENSSSCG00000004979 | 1 | - | 1.69E+08 | 1.69E+08 | 383 | annot_exons |
| novel_circ_004002 | ENSSSCG00000010241 | 14 | + | 71765050 | 71767362 | 2311 | annot_exons |
| novel_circ_004003 | ENSSSCG00000003917 | 6 | + | 1.66E+08 | 1.66E+08 | 486 | exon_intron |
| novel_circ_004004 | ENSSSCG00000003642 | 6 | - | 93940578 | 93940843 | 128 | annot_exons |
| novel_circ_004005 | ENSSSCG00000004620 | 1 | + | 1.19E+08 | 1.19E+08 | 1001 | annot_exons |
| novel_circ_004006 | ENSSSCG00000017904 | 12 | - | 51956275 | 51956652 | 287 | annot_exons |
| novel_circ_004007 | ENSSSCG00000012885 | 2 | - | 4608364 | 4623769 | 924 | annot_exons |
| novel_circ_004008 | ENSSSCG00000034191 | 2 | + | 42534759 | 42573733 | 19210 | exon_intron |
| novel_circ_004009 | ENSSSCG00000023974 | 2 | + | 16491731 | 16505030 | 837 | annot_exons |
| novel_circ_004010 | NA | 1 | + | 1.3E+08 | 1.3E+08 | 135 | intergenic |
| novel_circ_004011 | ENSSSCG00000010190 | 14 | - | 60414250 | 60415173 | 924 | antisense |
| novel_circ_004012 | ENSSSCG00000017301 | 12 | - | 16209070 | 16211961 | 282 | annot_exons |
| novel_circ_004013 | ENSSSCG00000003693 | 6 | + | 1.04E+08 | 1.04E+08 | 1343 | annot_exons |
| novel_circ_004014 | ENSSSCG00000016157 | 15 | - | 1.13E+08 | 1.13E+08 | 318 | annot_exons |
| novel_circ_004015 | ENSSSCG00000017231 | 12 | + | 6460664 | 6462407 | 340 | annot_exons |
| novel_circ_004016 | ENSSSCG00000008513 | 3 | - | 1.07E+08 | 1.07E+08 | 1884 | annot_exons |
| novel_circ_004017 | ENSSSCG00000050406 | 4 | + | 1.28E+08 | 1.28E+08 | 24611 | exon_intron |
| novel_circ_004018 | ENSSSCG00000008877 | 8 | + | 47644977 | 47654033 | 1006 | annot_exons |
| novel_circ_004019 | ENSSSCG00000005366 | 1 | + | 2.4E+08 | 2.4E+08 | 408 | annot_exons |
| novel_circ_004020 | ENSSSCG00000007153 | 17 | - | 32111605 | 32143743 | 1709 | annot_exons |
| novel_circ_004021 | ENSSSCG00000008412 | 3 | + | 87260603 | 87262951 | 195 | annot_exons |
| novel_circ_004022 | ENSSSCG00000014948 | 9 | + | 26178130 | 26186408 | 659 | annot_exons |
| novel_circ_004023 | NA | 15 | - | 84264654 | 84264827 | 174 | intergenic |
| novel_circ_004024 | ENSSSCG00000025221 | 18 | + | 3096932 | 3103597 | 928 | annot_exons |
| novel_circ_004025 | ENSSSCG00000016943 | 16 | - | 44051153 | 44051899 | 309 | annot_exons |
| novel_circ_004026 | ENSSSCG00000005444 | 1 | - | 2.5E+08 | 2.5E+08 | 562 | annot_exons |
| novel_circ_004027 | ENSSSCG00000011068 | 10 | - | 48815859 | 48818387 | 421 | annot_exons |
| novel_circ_004028 | ENSSSCG00000011068 | 10 | - | 48831898 | 48849331 | 504 | annot_exons |
| novel_circ_004029 | ENSSSCG00000004725 | 1 | + | 1.29E+08 | 1.29E+08 | 834 | annot_exons |
| novel_circ_004030 | ENSSSCG00000032176 | 12 | + | 14458188 | 14461336 | 288 | annot_exons |
| novel_circ_004031 | ENSSSCG00000026945 | 13 | - | 29491538 | 29498836 | 294 | annot_exons |
| novel_circ_004032 | ENSSSCG00000012859 | 2 | - | 2365031 | 2365589 | 185 | annot_exons |
| novel_circ_004033 | ENSSSCG00000022396 | 4 | - | 81610979 | 81616189 | 549 | annot_exons |
| novel_circ_004034 | ENSSSCG00000006487 | 4 | + | 93738637 | 93749340 | 767 | annot_exons |
| novel_circ_004035 | ENSSSCG00000004370 | 1 | - | 72422396 | 72454659 | 213 | annot_exons |
| novel_circ_004036 | ENSSSCG00000015283 | 9 | - | 65103719 | 65106823 | 423 | annot_exons |
| novel_circ_004037 | ENSSSCG00000011025 | 10 | + | 42063542 | 42079536 | 15995 | intronic |
| novel_circ_004038 | ENSSSCG00000040713 | X | + | 13266919 | 13270698 | 396 | annot_exons |
| novel_circ_004039 | ENSSSCG00000005386 | 1 | + | 2.42E+08 | 2.42E+08 | 479 | annot_exons |
| novel_circ_004040 | ENSSSCG00000050244 | 12 | + | 59351567 | 59360421 | 6857 | exon_intron |
| novel_circ_004041 | ENSSSCG00000000840 | 5 | - | 79462271 | 79470833 | 2223 | annot_exons |
| novel_circ_004042 | ENSSSCG00000005719 | 1 | - | 2.72E+08 | 2.72E+08 | 590 | annot_exons |
| novel_circ_004043 | ENSSSCG00000028694 | 14 | + | 4305018 | 4308988 | 615 | annot_exons |
| novel_circ_004044 | ENSSSCG00000008540 | 3 | - | 1.1E+08 | 1.1E+08 | 359 | annot_exons |
| novel_circ_004045 | NA | 15 | - | 84247664 | 84248193 | 530 | intergenic |
| novel_circ_004046 | ENSSSCG00000016564 | 18 | + | 18888234 | 18888929 | 152 | annot_exons |
| novel_circ_004047 | ENSSSCG00000023533 | 1 | - | 1.48E+08 | 1.48E+08 | 477 | annot_exons |
| novel_circ_004048 | ENSSSCG00000000039 | 5 | + | 6189355 | 6195200 | 481 | annot_exons |
| novel_circ_004049 | ENSSSCG00000035045 | 14 | + | 1.37E+08 | 1.37E+08 | 883 | annot_exons |
| novel_circ_004050 | ENSSSCG00000025870 | 9 | - | 73054956 | 73059640 | 636 | annot_exons |
| novel_circ_004051 | ENSSSCG00000038958 | 9 | + | 1.14E+08 | 1.14E+08 | 354 | annot_exons |
| novel_circ_004052 | ENSSSCG00000011689 | 13 | - | 86452535 | 86474319 | 723 | annot_exons |
| novel_circ_004053 | ENSSSCG00000033120 | 1 | + | 2.51E+08 | 2.51E+08 | 696 | one_exon |
| novel_circ_004054 | ENSSSCG00000023434 | 13 | + | 1.01E+08 | 1.01E+08 | 20168 | exon_intron |
| novel_circ_004055 | ENSSSCG00000039157 | 15 | + | 29959468 | 29969822 | 411 | annot_exons |
| novel_circ_004056 | ENSSSCG00000011833 | 13 | + | 1.33E+08 | 1.33E+08 | 381 | annot_exons |
| novel_circ_004057 | ENSSSCG00000016397 | 15 | + | 720411 | 720728 | 318 | intronic |
| novel_circ_004058 | ENSSSCG00000003401 | 6 | + | 70545569 | 70550321 | 369 | annot_exons |
| novel_circ_004059 | ENSSSCG00000011194 | 13 | - | 2960603 | 2964480 | 262 | annot_exons |
| novel_circ_004060 | ENSSSCG00000009319 | 11 | + | 5577247 | 5602241 | 1441 | annot_exons |
| novel_circ_004061 | ENSSSCG00000017759 | 12 | - | 44814894 | 44815535 | 351 | annot_exons |
| novel_circ_004062 | ENSSSCG00000006864 | 4 | - | 1.18E+08 | 1.18E+08 | 668 | annot_exons |
| novel_circ_004063 | ENSSSCG00000014440 | 2 | + | 1.51E+08 | 1.51E+08 | 430 | annot_exons |
| novel_circ_004064 | ENSSSCG00000014835 | 9 | - | 8471324 | 8481227 | 717 | annot_exons |
| novel_circ_004065 | ENSSSCG00000012474 | X | + | 78867668 | 78885018 | 816 | annot_exons |
| novel_circ_004066 | ENSSSCG00000007839 | 3 | - | 23910594 | 23913947 | 465 | annot_exons |
| novel_circ_004067 | NA | 15 | + | 84475894 | 84477034 | 1141 | intergenic |
| novel_circ_004068 | ENSSSCG00000017672 | 12 | + | 36346394 | 36372744 | 1663 | annot_exons |
| novel_circ_004069 | ENSSSCG00000009534 | 11 | + | 71125629 | 71135931 | 613 | annot_exons |
| novel_circ_004070 | ENSSSCG00000012982 | 2 | - | 6614277 | 6614664 | 305 | annot_exons |
| novel_circ_004071 | ENSSSCG00000009090 | 8 | - | 1.02E+08 | 1.02E+08 | 797 | annot_exons |
| novel_circ_004072 | ENSSSCG00000020720 | 2 | + | 48822509 | 48823373 | 865 | exon_intron |
| novel_circ_004073 | ENSSSCG00000016877 | 16 | + | 28167355 | 28182377 | 813 | annot_exons |
| novel_circ_004074 | ENSSSCG00000008575 | 3 | + | 1.13E+08 | 1.13E+08 | 1082 | annot_exons |
| novel_circ_004075 | ENSSSCG00000024296 | 13 | - | 10700223 | 10700464 | 242 | one_exon |
| novel_circ_004076 | ENSSSCG00000006740 | 4 | - | 1.04E+08 | 1.04E+08 | 971 | annot_exons |
| novel_circ_004077 | ENSSSCG00000015718 | 15 | - | 24866918 | 24868712 | 977 | annot_exons |
| novel_circ_004078 | ENSSSCG00000020663 | 18 | + | 5029807 | 5075381 | 45575 | exon_intron |
| novel_circ_004079 | NA | 15 | + | 33443400 | 33444270 | 871 | intergenic |
| novel_circ_004080 | ENSSSCG00000016300 | 15 | + | 1.34E+08 | 1.34E+08 | 249 | annot_exons |
| novel_circ_004081 | ENSSSCG00000013744 | 2 | + | 65947278 | 65948953 | 439 | annot_exons |
| novel_circ_004082 | ENSSSCG00000004857 | 1 | - | 1.46E+08 | 1.46E+08 | 220 | annot_exons |
| novel_circ_004083 | ENSSSCG00000026232 | 8 | - | 12643329 | 12655717 | 854 | annot_exons |
| novel_circ_004084 | ENSSSCG00000016845 | 16 | - | 22546397 | 22572494 | 1585 | annot_exons |
| novel_circ_004085 | ENSSSCG00000036363 | 2 | + | 59587338 | 59590434 | 823 | annot_exons |
| novel_circ_004086 | ENSSSCG00000025672 | 6 | - | 1.48E+08 | 1.48E+08 | 818 | annot_exons |
| novel_circ_004087 | ENSSSCG00000000955 | 5 | - | 2675422 | 2692132 | 307 | annot_exons |
| novel_circ_004088 | ENSSSCG00000026744 | 11 | - | 1355228 | 1364698 | 345 | annot_exons |
| novel_circ_004089 | ENSSSCG00000024453 | 13 | - | 68601960 | 68611602 | 706 | annot_exons |
| novel_circ_004090 | ENSSSCG00000015861 | 15 | + | 59562524 | 59570339 | 2435 | annot_exons |
| novel_circ_004091 | ENSSSCG00000008150 | 3 | - | 51451796 | 51452194 | 399 | intronic |
| novel_circ_004092 | ENSSSCG00000025794 | 3 | - | 17733251 | 17733646 | 292 | annot_exons |
| novel_circ_004093 | ENSSSCG00000008499 | 3 | + | 1.03E+08 | 1.03E+08 | 479 | annot_exons |
| novel_circ_004094 | ENSSSCG00000006179 | 4 | + | 62167471 | 62187826 | 429 | annot_exons |
| novel_circ_004095 | NA | 15 | + | 84266720 | 84268138 | 1419 | intergenic |
| novel_circ_004096 | ENSSSCG00000011843 | 13 | - | 1.33E+08 | 1.33E+08 | 610 | annot_exons |
| novel_circ_004097 | ENSSSCG00000010211 | 14 | - | 63190562 | 63221112 | 652 | annot_exons |
| novel_circ_004098 | ENSSSCG00000023738 | 11 | - | 21804834 | 21808357 | 429 | annot_exons |
| novel_circ_004099 | ENSSSCG00000000840 | 5 | - | 79458763 | 79460620 | 349 | annot_exons |
| novel_circ_004100 | ENSSSCG00000032049 | 12 | - | 50056028 | 50056931 | 293 | annot_exons |
| novel_circ_004101 | ENSSSCG00000012160 | X | - | 15892843 | 15927085 | 674 | annot_exons |
| novel_circ_004102 | ENSSSCG00000008498 | 3 | + | 1.03E+08 | 1.03E+08 | 493 | annot_exons |
| novel_circ_004103 | ENSSSCG00000034191 | 2 | + | 42469592 | 42471447 | 1856 | exon_intron |
| novel_circ_004104 | ENSSSCG00000002333 | 7 | - | 96061742 | 96140732 | 710 | annot_exons |
| novel_circ_004105 | ENSSSCG00000016412 | 18 | + | 1819614 | 1830021 | 253 | annot_exons |
| novel_circ_004106 | ENSSSCG00000014338 | 2 | - | 1.41E+08 | 1.41E+08 | 917 | annot_exons |
| novel_circ_004107 | ENSSSCG00000021118 | 3 | + | 31255432 | 31258924 | 571 | annot_exons |
| novel_circ_004108 | ENSSSCG00000026063 | 2 | + | 74138526 | 74142784 | 371 | annot_exons |
| novel_circ_004109 | ENSSSCG00000034493 | 13 | - | 1.6E+08 | 1.6E+08 | 578 | annot_exons |
| novel_circ_004110 | ENSSSCG00000016147 | 15 | + | 1.11E+08 | 1.11E+08 | 409 | annot_exons |
| novel_circ_004111 | ENSSSCG00000016842 | 16 | + | 22312692 | 22315638 | 234 | annot_exons |
| novel_circ_004112 | ENSSSCG00000027667 | 7 | + | 1.21E+08 | 1.21E+08 | 319 | annot_exons |
| novel_circ_004113 | ENSSSCG00000037355 | 4 | + | 99537775 | 99537986 | 212 | one_exon |
| novel_circ_004114 | ENSSSCG00000007034 | 17 | + | 13008092 | 13008471 | 276 | annot_exons |
| novel_circ_004115 | ENSSSCG00000008575 | 3 | + | 1.13E+08 | 1.13E+08 | 651 | annot_exons |
| novel_circ_004116 | ENSSSCG00000040013 | 17 | - | 5338883 | 5340400 | 276 | annot_exons |
| novel_circ_004117 | ENSSSCG00000030222 | 14 | - | 31775545 | 31783989 | 573 | annot_exons |
| novel_circ_004118 | ENSSSCG00000029808 | 6 | + | 1.66E+08 | 1.66E+08 | 3139 | annot_exons |
| novel_circ_004119 | ENSSSCG00000016397 | 15 | + | 622250 | 623446 | 213 | annot_exons |
| novel_circ_004120 | ENSSSCG00000014835 | 9 | - | 8495639 | 8496682 | 469 | annot_exons |
| novel_circ_004121 | ENSSSCG00000013022 | 2 | - | 7414793 | 7415212 | 420 | antisense |
| novel_circ_004122 | ENSSSCG00000029420 | 6 | - | 46371479 | 46385814 | 7866 | annot_exons |
| novel_circ_004123 | ENSSSCG00000035379 | 8 | - | 67440125 | 67445040 | 471 | annot_exons |
| novel_circ_004124 | ENSSSCG00000004979 | 1 | - | 1.69E+08 | 1.69E+08 | 622 | annot_exons |
| novel_circ_004125 | ENSSSCG00000050627 | 3 | - | 59423230 | 59425872 | 998 | annot_exons |
| novel_circ_004126 | ENSSSCG00000029174 | 9 | + | 92397525 | 92397915 | 391 | one_exon |
| novel_circ_004127 | ENSSSCG00000008803 | 8 | - | 33488207 | 33508847 | 344 | annot_exons |
| novel_circ_004128 | ENSSSCG00000010313 | 14 | + | 76786741 | 76788678 | 391 | annot_exons |
| novel_circ_004129 | ENSSSCG00000011720 | 13 | + | 92652259 | 92677963 | 949 | annot_exons |
| novel_circ_004130 | ENSSSCG00000016117 | 15 | + | 1.06E+08 | 1.06E+08 | 564 | annot_exons |
| novel_circ_004131 | ENSSSCG00000005170 | 1 | - | 2.04E+08 | 2.04E+08 | 320 | annot_exons |
| novel_circ_004132 | ENSSSCG00000031459 | 2 | + | 4738245 | 4752017 | 898 | annot_exons |
| novel_circ_004133 | ENSSSCG00000029169 | 1 | - | 2.66E+08 | 2.66E+08 | 531 | annot_exons |
| novel_circ_004134 | ENSSSCG00000005444 | 1 | - | 2.5E+08 | 2.5E+08 | 1535 | annot_exons |
| novel_circ_004135 | ENSSSCG00000011317 | 13 | - | 29125718 | 29132223 | 377 | annot_exons |
| novel_circ_004136 | ENSSSCG00000011022 | 10 | + | 41430334 | 41434472 | 587 | annot_exons |
| novel_circ_004137 | ENSSSCG00000005198 | 1 | - | 2.15E+08 | 2.15E+08 | 6819 | exon_intron |
| novel_circ_004138 | ENSSSCG00000004666 | 1 | - | 1.26E+08 | 1.26E+08 | 405 | annot_exons |
| novel_circ_004139 | ENSSSCG00000015603 | 9 | + | 1.31E+08 | 1.31E+08 | 616 | annot_exons |
| novel_circ_004140 | ENSSSCG00000022982 | 9 | + | 73100284 | 73124744 | 379 | annot_exons |
| novel_circ_004141 | ENSSSCG00000030095 | 9 | - | 41668655 | 41668971 | 317 | antisense |
| novel_circ_004142 | ENSSSCG00000011843 | 13 | - | 1.33E+08 | 1.33E+08 | 356 | annot_exons |
| novel_circ_004143 | ENSSSCG00000004394 | 1 | + | 77116127 | 77124674 | 373 | annot_exons |
| novel_circ_004144 | ENSSSCG00000006840 | 4 | + | 1.11E+08 | 1.11E+08 | 1187 | annot_exons |
| novel_circ_004145 | ENSSSCG00000008581 | 3 | - | 1.14E+08 | 1.14E+08 | 1288 | annot_exons |
| novel_circ_004146 | ENSSSCG00000023738 | 11 | - | 21807483 | 21813745 | 450 | annot_exons |
| novel_circ_004147 | ENSSSCG00000003816 | 6 | + | 1.5E+08 | 1.5E+08 | 199 | antisense |
| novel_circ_004148 | ENSSSCG00000014114 | 2 | + | 88358554 | 88360045 | 276 | annot_exons |
| novel_circ_004149 | ENSSSCG00000017067 | 16 | - | 68339891 | 68342147 | 376 | annot_exons |
| novel_circ_004150 | ENSSSCG00000015233 | 9 | - | 53298150 | 53298348 | 199 | one_exon |
| novel_circ_004151 | ENSSSCG00000004729 | 1 | - | 1.29E+08 | 1.29E+08 | 1032 | annot_exons |
| novel_circ_004152 | ENSSSCG00000010862 | 10 | - | 14826467 | 14850824 | 329 | annot_exons |
| novel_circ_004153 | ENSSSCG00000008753 | 8 | - | 16690863 | 16704217 | 789 | annot_exons |
| novel_circ_004154 | ENSSSCG00000050244 | 12 | + | 59320434 | 59323398 | 427 | annot_exons |
| novel_circ_004155 | ENSSSCG00000004814 | 1 | + | 1.38E+08 | 1.38E+08 | 670 | annot_exons |
| novel_circ_004156 | ENSSSCG00000003712 | 6 | - | 1.09E+08 | 1.09E+08 | 241 | annot_exons |
| novel_circ_004157 | ENSSSCG00000005575 | 1 | - | 2.64E+08 | 2.64E+08 | 453 | annot_exons |
| novel_circ_004158 | ENSSSCG00000016513 | 18 | + | 10794878 | 10806244 | 899 | annot_exons |
| novel_circ_004159 | ENSSSCG00000012250 | X | - | 36388349 | 36405619 | 1035 | annot_exons |
| novel_circ_004160 | ENSSSCG00000028585 | 9 | + | 1.07E+08 | 1.07E+08 | 427 | annot_exons |
| novel_circ_004161 | NA | 15 | - | 84232436 | 84232672 | 237 | intergenic |
| novel_circ_004162 | ENSSSCG00000031831 | 9 | - | 42795737 | 42797854 | 2118 | intronic |
| novel_circ_004163 | ENSSSCG00000008041 | 3 | + | 39897303 | 39897437 | 135 | one_exon |
| novel_circ_004164 | ENSSSCG00000007034 | 17 | + | 13234008 | 13259559 | 2490 | annot_exons |
| novel_circ_004165 | ENSSSCG00000010872 | 10 | - | 16499157 | 16522506 | 467 | annot_exons |
| novel_circ_004166 | ENSSSCG00000010548 | 14 | - | 1.11E+08 | 1.11E+08 | 551 | annot_exons |
| novel_circ_004167 | ENSSSCG00000009090 | 8 | - | 1.02E+08 | 1.02E+08 | 569 | annot_exons |
| novel_circ_004168 | ENSSSCG00000014126 | 2 | + | 89360817 | 89377048 | 459 | annot_exons |
| novel_circ_004169 | ENSSSCG00000022982 | 9 | + | 73094595 | 73095910 | 195 | annot_exons |
| novel_circ_004170 | ENSSSCG00000026457 | 3 | + | 15180942 | 15220796 | 351 | annot_exons |
| novel_circ_004171 | ENSSSCG00000015558 | 9 | + | 1.25E+08 | 1.25E+08 | 637 | annot_exons |
| novel_circ_004172 | ENSSSCG00000001641 | 7 | + | 37725682 | 37728630 | 313 | annot_exons |
| novel_circ_004173 | NA | 15 | - | 84468093 | 84468289 | 197 | intergenic |
| novel_circ_004174 | ENSSSCG00000021072 | 1 | - | 95513003 | 95514049 | 355 | annot_exons |
| novel_circ_004175 | ENSSSCG00000039258 | 5 | - | 28659669 | 28660480 | 812 | one_exon |
| novel_circ_004176 | ENSSSCG00000003512 | 6 | - | 79073283 | 79075866 | 489 | annot_exons |
| novel_circ_004177 | ENSSSCG00000016518 | 18 | - | 11105572 | 11116379 | 400 | annot_exons |
| novel_circ_004178 | ENSSSCG00000001533 | 7 | - | 30802688 | 30803230 | 185 | annot_exons |
| novel_circ_004179 | ENSSSCG00000004082 | 1 | + | 14156376 | 14167020 | 502 | annot_exons |
| novel_circ_004180 | ENSSSCG00000010243 | 14 | + | 71844195 | 71853159 | 753 | annot_exons |
| novel_circ_004181 | ENSSSCG00000044189 | 2 | - | 1.52E+08 | 1.52E+08 | 262 | annot_exons |
| novel_circ_004182 | ENSSSCG00000038437 | AEMK02000682.1 | - | 264782 | 274132 | 869 | annot_exons |
| novel_circ_004183 | ENSSSCG00000029464 | 5 | - | 67674585 | 67688990 | 1098 | annot_exons |
| novel_circ_004184 | NA | 15 | + | 84460796 | 84461676 | 881 | intergenic |
| novel_circ_004185 | ENSSSCG00000016842 | 16 | + | 22305279 | 22306742 | 334 | annot_exons |
| novel_circ_004186 | ENSSSCG00000029633 | 6 | + | 1.57E+08 | 1.57E+08 | 311 | annot_exons |
| novel_circ_004187 | ENSSSCG00000010742 | 14 | - | 1.35E+08 | 1.35E+08 | 846 | annot_exons |
| novel_circ_004188 | ENSSSCG00000008226 | 3 | + | 58676460 | 58678177 | 355 | annot_exons |
| novel_circ_004189 | ENSSSCG00000000846 | 5 | - | 80375027 | 80384669 | 1066 | annot_exons |
| novel_circ_004190 | NA | 5 | - | 34887478 | 34887740 | 263 | intergenic |
| novel_circ_004191 | ENSSSCG00000011769 | 13 | + | 1.19E+08 | 1.19E+08 | 432 | annot_exons |
| novel_circ_004192 | ENSSSCG00000000019 | 5 | - | 4210660 | 4212802 | 2143 | exon_intron |
| novel_circ_004193 | ENSSSCG00000016379 | 15 | - | 1.4E+08 | 1.4E+08 | 3032 | annot_exons |
| novel_circ_004194 | ENSSSCG00000026960 | 1 | + | 1.15E+08 | 1.15E+08 | 353 | annot_exons |
| novel_circ_004195 | ENSSSCG00000016019 | 15 | - | 88476598 | 88477180 | 204 | annot_exons |
| novel_circ_004196 | ENSSSCG00000004636 | 1 | + | 1.21E+08 | 1.21E+08 | 445 | annot_exons |
| novel_circ_004197 | ENSSSCG00000004465 | 1 | - | 85926481 | 85935975 | 209 | annot_exons |
| novel_circ_004198 | ENSSSCG00000016835 | 16 | + | 21480496 | 21482495 | 475 | exon_intron |
| novel_circ_004199 | ENSSSCG00000009522 | 11 | + | 69067104 | 69080842 | 428 | annot_exons |
| novel_circ_004200 | ENSSSCG00000024621 | 13 | + | 7021801 | 7053012 | 740 | annot_exons |
| novel_circ_004201 | ENSSSCG00000011518 | 13 | - | 54171984 | 54173527 | 284 | annot_exons |
| novel_circ_004202 | ENSSSCG00000007311 | 17 | - | 39240226 | 39240431 | 206 | antisense |
| novel_circ_004203 | NA | 15 | + | 84273420 | 84273695 | 276 | intergenic |
| novel_circ_004204 | ENSSSCG00000016223 | 15 | + | 1.25E+08 | 1.25E+08 | 440 | annot_exons |
| novel_circ_004205 | ENSSSCG00000029094 | 13 | + | 1744416 | 1752159 | 1074 | annot_exons |
| novel_circ_004206 | ENSSSCG00000015586 | 9 | - | 1.3E+08 | 1.3E+08 | 390 | annot_exons |
| novel_circ_004207 | ENSSSCG00000028148 | X | - | 28460957 | 28468255 | 629 | annot_exons |
| novel_circ_004208 | ENSSSCG00000002547 | AEMK02000452.1 | + | 2189903 | 2193727 | 464 | annot_exons |
| novel_circ_004209 | ENSSSCG00000003700 | 6 | - | 1.07E+08 | 1.07E+08 | 1087 | exon_intron |
| novel_circ_004210 | ENSSSCG00000039157 | 15 | + | 29932361 | 29939015 | 345 | annot_exons |
| novel_circ_004211 | ENSSSCG00000009502 | 11 | - | 65315580 | 65329042 | 1121 | annot_exons |
| novel_circ_004212 | ENSSSCG00000008591 | 3 | + | 1.15E+08 | 1.15E+08 | 568 | annot_exons |
| novel_circ_004213 | ENSSSCG00000022636 | 5 | + | 42848099 | 42851573 | 268 | annot_exons |
| novel_circ_004214 | ENSSSCG00000006912 | 4 | + | 1.26E+08 | 1.26E+08 | 970 | annot_exons |
| novel_circ_004215 | ENSSSCG00000023674 | 13 | + | 1.97E+08 | 1.97E+08 | 2067 | annot_exons |
| novel_circ_004216 | ENSSSCG00000038036 | 1 | - | 2.62E+08 | 2.62E+08 | 807 | annot_exons |
| novel_circ_004217 | ENSSSCG00000035824 | 13 | - | 34799994 | 34802554 | 292 | annot_exons |
| novel_circ_004218 | ENSSSCG00000038760 | 12 | - | 26987759 | 26989423 | 635 | annot_exons |
| novel_circ_004219 | ENSSSCG00000022708 | 3 | - | 71332900 | 71334071 | 270 | annot_exons |
| novel_circ_004220 | ENSSSCG00000009856 | 14 | + | 35261305 | 35286318 | 1295 | annot_exons |
| novel_circ_004221 | ENSSSCG00000012484 | X | - | 82403059 | 82405276 | 608 | annot_exons |
| novel_circ_004222 | ENSSSCG00000001852 | 7 | - | 55654510 | 55664446 | 1338 | annot_exons |
| novel_circ_004223 | ENSSSCG00000003401 | 6 | + | 70647883 | 70655830 | 891 | annot_exons |
| novel_circ_004224 | ENSSSCG00000014913 | 9 | - | 19897760 | 19918888 | 6115 | exon_intron |
| novel_circ_004225 | ENSSSCG00000008575 | 3 | + | 1.13E+08 | 1.13E+08 | 608 | annot_exons |
| novel_circ_004226 | ENSSSCG00000006642 | 4 | - | 98219019 | 98219206 | 188 | antisense |
| novel_circ_004227 | ENSSSCG00000039763 | 16 | + | 72198246 | 72204314 | 572 | annot_exons |
| novel_circ_004228 | ENSSSCG00000023738 | 11 | - | 21797314 | 21805764 | 471 | annot_exons |
| novel_circ_004229 | ENSSSCG00000002988 | 6 | + | 48424532 | 48426035 | 223 | annot_exons |
| novel_circ_004230 | ENSSSCG00000024361 | 1 | + | 11584833 | 11596113 | 365 | annot_exons |
| novel_circ_004231 | ENSSSCG00000016784 | 16 | - | 4159083 | 4175644 | 420 | annot_exons |
| novel_circ_004232 | ENSSSCG00000000900 | 5 | - | 88040453 | 88050924 | 835 | annot_exons |
| novel_circ_004233 | NA | 15 | - | 84279863 | 84280064 | 202 | intergenic |
| novel_circ_004234 | ENSSSCG00000006137 | 4 | - | 50402450 | 50408469 | 556 | annot_exons |
| novel_circ_004235 | ENSSSCG00000010894 | 10 | - | 19846605 | 19848818 | 277 | annot_exons |
| novel_circ_004236 | ENSSSCG00000034521 | 6 | + | 29344146 | 29350957 | 355 | annot_exons |
| novel_circ_004237 | ENSSSCG00000010571 | 14 | - | 1.13E+08 | 1.13E+08 | 360 | annot_exons |
| novel_circ_004238 | ENSSSCG00000015789 | 15 | + | 46294098 | 46307723 | 301 | annot_exons |
| novel_circ_004239 | ENSSSCG00000003712 | 6 | - | 1.09E+08 | 1.09E+08 | 810 | annot_exons |
| novel_circ_004240 | ENSSSCG00000010467 | 14 | + | 1.04E+08 | 1.04E+08 | 834 | exon_intron |
| novel_circ_004241 | ENSSSCG00000010164 | 14 | - | 57438586 | 57463544 | 870 | annot_exons |
| novel_circ_004242 | ENSSSCG00000043604 | X | - | 59553515 | 59571888 | 3161 | exon_intron |
| novel_circ_004243 | ENSSSCG00000029920 | 8 | + | 87799383 | 87820356 | 493 | annot_exons |
| novel_circ_004244 | ENSSSCG00000003789 | 6 | - | 1.42E+08 | 1.42E+08 | 275 | annot_exons |
| novel_circ_004245 | ENSSSCG00000008980 | 8 | - | 71874944 | 71887965 | 495 | annot_exons |
| novel_circ_004246 | ENSSSCG00000017052 | 16 | + | 65763592 | 65769629 | 331 | annot_exons |
| novel_circ_004247 | ENSSSCG00000017260 | 12 | + | 11745679 | 11748619 | 493 | annot_exons |
| novel_circ_004248 | ENSSSCG00000016912 | 16 | - | 34633974 | 34666354 | 588 | annot_exons |
| novel_circ_004249 | ENSSSCG00000037358 | 2 | + | 40929361 | 40931409 | 511 | annot_exons |
| novel_circ_004250 | ENSSSCG00000015434 | 9 | - | 1.06E+08 | 1.06E+08 | 5180 | exon_intron |
| novel_circ_004251 | ENSSSCG00000015462 | 9 | + | 1.13E+08 | 1.13E+08 | 246 | annot_exons |
| novel_circ_004252 | ENSSSCG00000010823 | 10 | + | 9687882 | 9689261 | 283 | annot_exons |
| novel_circ_004253 | ENSSSCG00000035671 | 12 | + | 61274041 | 61274215 | 175 | antisense |
| novel_circ_004254 | ENSSSCG00000023771 | 11 | - | 3959346 | 3960189 | 240 | annot_exons |
| novel_circ_004255 | ENSSSCG00000036790 | 1 | - | 32324753 | 32354628 | 409 | annot_exons |
| novel_circ_004256 | ENSSSCG00000030291 | 4 | - | 98655267 | 98656925 | 544 | annot_exons |
| novel_circ_004257 | ENSSSCG00000007736 | 3 | - | 16260678 | 16264205 | 463 | annot_exons |
| novel_circ_004258 | ENSSSCG00000023307 | 16 | + | 52275057 | 52277655 | 2599 | intronic |
| novel_circ_004259 | NA | 15 | - | 84273211 | 84273692 | 482 | intergenic |
| novel_circ_004260 | ENSSSCG00000022676 | X | - | 54286536 | 54297759 | 343 | annot_exons |
| novel_circ_004261 | ENSSSCG00000009134 | 8 | - | 1.12E+08 | 1.12E+08 | 2244 | annot_exons |
| novel_circ_004262 | ENSSSCG00000015940 | 15 | + | 76208643 | 76226908 | 377 | annot_exons |
| novel_circ_004263 | ENSSSCG00000013022 | 2 | + | 7416124 | 7417345 | 365 | annot_exons |
| novel_circ_004264 | ENSSSCG00000016583 | 18 | - | 19926060 | 19929506 | 548 | annot_exons |
| novel_circ_004265 | ENSSSCG00000007951 | 3 | + | 38413987 | 38450234 | 890 | annot_exons |
| novel_circ_004266 | ENSSSCG00000011672 | 13 | + | 82256891 | 82285635 | 394 | annot_exons |
| novel_circ_004267 | ENSSSCG00000015857 | 15 | - | 59226488 | 59244748 | 976 | annot_exons |
| novel_circ_004268 | ENSSSCG00000012117 | X | + | 9518317 | 9527875 | 558 | annot_exons |
| novel_circ_004269 | ENSSSCG00000002441 | 7 | - | 1.13E+08 | 1.13E+08 | 334 | annot_exons |
| novel_circ_004270 | ENSSSCG00000029464 | 5 | - | 67687658 | 67688990 | 279 | annot_exons |
| novel_circ_004271 | ENSSSCG00000033591 | 6 | - | 31851972 | 31853898 | 1073 | annot_exons |
| novel_circ_004272 | NA | 15 | + | 84266437 | 84266718 | 282 | intergenic |
| novel_circ_004273 | ENSSSCG00000036870 | 6 | + | 51920282 | 51921166 | 885 | antisense |
| novel_circ_004274 | ENSSSCG00000005103 | 1 | + | 1.91E+08 | 1.91E+08 | 354 | one_exon |
| novel_circ_004275 | ENSSSCG00000029837 | 11 | + | 25178811 | 25240454 | 1001 | annot_exons |
| novel_circ_004276 | ENSSSCG00000009142 | 8 | - | 1.13E+08 | 1.13E+08 | 830 | annot_exons |
| novel_circ_004277 | ENSSSCG00000009007 | 8 | - | 75520077 | 75530004 | 917 | annot_exons |
| novel_circ_004278 | ENSSSCG00000034493 | 13 | - | 1.6E+08 | 1.6E+08 | 1167 | annot_exons |
| novel_circ_004279 | ENSSSCG00000031147 | 13 | - | 1.32E+08 | 1.32E+08 | 393 | annot_exons |
| novel_circ_004280 | ENSSSCG00000030291 | 4 | - | 98636787 | 98669610 | 11289 | exon_intron |
| novel_circ_004281 | ENSSSCG00000003885 | 6 | - | 1.64E+08 | 1.64E+08 | 643 | annot_exons |
| novel_circ_004282 | ENSSSCG00000029538 | 9 | - | 39494895 | 39503008 | 533 | annot_exons |
| novel_circ_004283 | ENSSSCG00000007280 | 17 | + | 37900616 | 37905127 | 323 | annot_exons |
| novel_circ_004284 | ENSSSCG00000003971 | 6 | + | 1.7E+08 | 1.7E+08 | 1064 | exon_intron |
| novel_circ_004285 | ENSSSCG00000010681 | 14 | - | 1.29E+08 | 1.29E+08 | 2399 | exon_intron |
| novel_circ_004286 | ENSSSCG00000011163 | 10 | + | 68959424 | 68970531 | 423 | annot_exons |
| novel_circ_004287 | ENSSSCG00000002684 | 6 | - | 5544365 | 5545178 | 814 | intronic |
| novel_circ_004288 | ENSSSCG00000021023 | 13 | - | 1.95E+08 | 1.95E+08 | 569 | annot_exons |
| novel_circ_004289 | ENSSSCG00000010594 | 14 | - | 1.14E+08 | 1.14E+08 | 17065 | exon_intron |
| novel_circ_004290 | ENSSSCG00000041176 | 9 | - | 1.35E+08 | 1.35E+08 | 282 | annot_exons |
| novel_circ_004291 | ENSSSCG00000050914 | 16 | - | 25613034 | 25618464 | 4729 | exon_intron |
| novel_circ_004292 | ENSSSCG00000038534 | 12 | - | 48409933 | 48411383 | 407 | annot_exons |
| novel_circ_004293 | ENSSSCG00000014213 | 2 | + | 1.19E+08 | 1.19E+08 | 419 | one_exon |
| novel_circ_004294 | ENSSSCG00000024197 | 6 | - | 79787142 | 79790525 | 451 | annot_exons |
| novel_circ_004295 | ENSSSCG00000011000 | 10 | - | 33434993 | 33435619 | 217 | annot_exons |
| novel_circ_004296 | ENSSSCG00000006872 | 4 | - | 1.18E+08 | 1.18E+08 | 373 | annot_exons |
| novel_circ_004297 | ENSSSCG00000005076 | 1 | + | 1.88E+08 | 1.88E+08 | 638 | annot_exons |
| novel_circ_004298 | ENSSSCG00000033577 | 4 | - | 21207952 | 21210136 | 264 | annot_exons |
| novel_circ_004299 | ENSSSCG00000015931 | 15 | + | 75876164 | 75876972 | 809 | one_exon |
| novel_circ_004300 | ENSSSCG00000011117 | 10 | + | 59913525 | 59914874 | 359 | annot_exons |
| novel_circ_004301 | ENSSSCG00000030435 | 3 | - | 59143569 | 59145324 | 290 | annot_exons |
| novel_circ_004302 | ENSSSCG00000011446 | 13 | - | 34876368 | 34882541 | 461 | annot_exons |
| novel_circ_004303 | ENSSSCG00000008971 | 8 | + | 71441778 | 71443049 | 303 | annot_exons |
| novel_circ_004304 | ENSSSCG00000009345 | 11 | + | 9170758 | 9187549 | 365 | annot_exons |
| novel_circ_004305 | ENSSSCG00000026705 | 10 | - | 12607745 | 12652349 | 1128 | annot_exons |
| novel_circ_004306 | ENSSSCG00000009040 | 8 | - | 82919677 | 82927163 | 597 | annot_exons |
| novel_circ_004307 | ENSSSCG00000015498 | 9 | - | 1.16E+08 | 1.16E+08 | 725 | annot_exons |
| novel_circ_004308 | ENSSSCG00000007799 | 3 | - | 17938142 | 17939550 | 417 | annot_exons |
| novel_circ_004309 | ENSSSCG00000013302 | 2 | - | 26494977 | 26502165 | 2060 | annot_exons |
| novel_circ_004310 | ENSSSCG00000005959 | 4 | + | 10609731 | 10620799 | 311 | annot_exons |
| novel_circ_004311 | ENSSSCG00000008132 | 3 | + | 47004817 | 47005413 | 256 | annot_exons |
| novel_circ_004312 | ENSSSCG00000024748 | 3 | - | 25294057 | 25294658 | 602 | antisense |
| novel_circ_004313 | ENSSSCG00000032861 | 5 | + | 14134584 | 14156724 | 339 | annot_exons |
| novel_circ_004314 | ENSSSCG00000031925 | 5 | - | 52032 | 59673 | 2470 | annot_exons |
| novel_circ_004315 | ENSSSCG00000006174 | 4 | + | 61635533 | 61693247 | 879 | annot_exons |
| novel_circ_004316 | ENSSSCG00000009492 | 11 | + | 63732198 | 63744217 | 590 | annot_exons |
| novel_circ_004317 | ENSSSCG00000037655 | 13 | + | 1.89E+08 | 1.89E+08 | 583 | annot_exons |
| novel_circ_004318 | ENSSSCG00000038561 | 15 | + | 84405620 | 84406444 | 825 | antisense |
| novel_circ_004319 | ENSSSCG00000036350 | 18 | + | 46208495 | 46213267 | 1042 | annot_exons |
| novel_circ_004320 | ENSSSCG00000017256 | 12 | + | 11258622 | 11268222 | 1016 | annot_exons |
| novel_circ_004321 | ENSSSCG00000029082 | 9 | + | 1.05E+08 | 1.05E+08 | 530 | annot_exons |
| novel_circ_004322 | ENSSSCG00000017732 | 12 | - | 42587230 | 42596350 | 1076 | annot_exons |
| novel_circ_004323 | ENSSSCG00000002844 | 6 | - | 37091577 | 37094925 | 295 | annot_exons |
| novel_circ_004324 | ENSSSCG00000004608 | 1 | + | 1.16E+08 | 1.16E+08 | 410 | annot_exons |
| novel_circ_004325 | ENSSSCG00000009244 | 8 | + | 1.35E+08 | 1.35E+08 | 406 | annot_exons |
| novel_circ_004326 | ENSSSCG00000007360 | 17 | - | 44164326 | 44166273 | 298 | annot_exons |
| novel_circ_004327 | ENSSSCG00000031392 | 13 | + | 10289379 | 10332426 | 281 | annot_exons |
| novel_circ_004328 | ENSSSCG00000011511 | 13 | - | 50553856 | 50554072 | 217 | one_exon |
| novel_circ_004329 | ENSSSCG00000039373 | 2 | + | 1.26E+08 | 1.26E+08 | 575 | intronic |
| novel_circ_004330 | ENSSSCG00000029837 | 11 | + | 25209180 | 25224424 | 402 | annot_exons |
| novel_circ_004331 | ENSSSCG00000015336 | 9 | - | 75936930 | 75949857 | 408 | annot_exons |
| novel_circ_004332 | ENSSSCG00000003775 | 6 | - | 1.38E+08 | 1.38E+08 | 192 | annot_exons |
| novel_circ_004333 | ENSSSCG00000005038 | 1 | + | 1.82E+08 | 1.82E+08 | 274 | annot_exons |
| novel_circ_004334 | ENSSSCG00000040887 | 6 | - | 34609187 | 34609517 | 231 | annot_exons |
| novel_circ_004335 | ENSSSCG00000041410 | 7 | - | 23022971 | 23023608 | 638 | one_exon |
| novel_circ_004336 | ENSSSCG00000016564 | 18 | + | 18897278 | 18923062 | 6878 | exon_intron |
| novel_circ_004337 | NA | 15 | - | 84364491 | 84367087 | 2597 | intergenic |
| novel_circ_004338 | ENSSSCG00000001723 | 7 | - | 41506141 | 41507096 | 267 | annot_exons |
| novel_circ_004339 | ENSSSCG00000014296 | 2 | - | 1.36E+08 | 1.36E+08 | 601 | annot_exons |
| novel_circ_004340 | ENSSSCG00000002426 | 7 | + | 1.1E+08 | 1.11E+08 | 1319 | annot_exons |
| novel_circ_004341 | ENSSSCG00000005388 | 1 | + | 2.42E+08 | 2.42E+08 | 1169 | annot_exons |
| novel_circ_004342 | ENSSSCG00000001921 | 7 | - | 60079176 | 60080048 | 332 | annot_exons |
| novel_circ_004343 | ENSSSCG00000004237 | 1 | + | 40116745 | 40119904 | 332 | annot_exons |
| novel_circ_004344 | ENSSSCG00000028748 | 6 | + | 1.58E+08 | 1.58E+08 | 786 | annot_exons |
| novel_circ_004345 | ENSSSCG00000016938 | 16 | + | 41238917 | 41274722 | 570 | annot_exons |
| novel_circ_004346 | ENSSSCG00000004936 | 1 | - | 1.64E+08 | 1.64E+08 | 296 | annot_exons |
| novel_circ_004347 | ENSSSCG00000015509 | 9 | - | 1.18E+08 | 1.18E+08 | 446 | annot_exons |
| novel_circ_004348 | ENSSSCG00000010360 | 14 | + | 87806050 | 87854827 | 418 | annot_exons |
| novel_circ_004349 | ENSSSCG00000009192 | 8 | + | 1.25E+08 | 1.25E+08 | 516 | antisense |
| novel_circ_004350 | ENSSSCG00000016850 | 16 | - | 23743903 | 23745763 | 270 | annot_exons |
| novel_circ_004351 | NA | 15 | + | 84285579 | 84285771 | 193 | intergenic |
| novel_circ_004352 | ENSSSCG00000001485 | 7 | - | 26620967 | 26633973 | 219 | annot_exons |
| novel_circ_004353 | ENSSSCG00000002547 | AEMK02000452.1 | + | 2186063 | 2190692 | 495 | annot_exons |
| novel_circ_004354 | ENSSSCG00000004123 | 1 | - | 20549777 | 20567142 | 574 | annot_exons |
| novel_circ_004355 | ENSSSCG00000022081 | 13 | - | 1.48E+08 | 1.48E+08 | 282 | annot_exons |
| novel_circ_004356 | ENSSSCG00000016518 | 18 | - | 11116261 | 11133851 | 17591 | exon_intron |
| novel_circ_004357 | ENSSSCG00000021042 | 13 | - | 2.03E+08 | 2.03E+08 | 562 | annot_exons |
| novel_circ_004358 | ENSSSCG00000007034 | 17 | + | 13115684 | 13146266 | 215 | annot_exons |
| novel_circ_004359 | ENSSSCG00000024001 | 13 | - | 73091513 | 73094623 | 1428 | annot_exons |
| novel_circ_004360 | ENSSSCG00000004499 | 1 | - | 95742426 | 95742917 | 377 | annot_exons |
| novel_circ_004361 | ENSSSCG00000000754 | 5 | - | 68157711 | 68158502 | 264 | annot_exons |
| novel_circ_004362 | ENSSSCG00000006779 | 4 | + | 1.08E+08 | 1.08E+08 | 544 | annot_exons |
| novel_circ_004363 | ENSSSCG00000009217 | 8 | - | 1.31E+08 | 1.31E+08 | 366 | annot_exons |
| novel_circ_004364 | ENSSSCG00000039061 | 1 | - | 1.31E+08 | 1.31E+08 | 182 | annot_exons |
| novel_circ_004365 | ENSSSCG00000045048 | 14 | - | 41681832 | 41686858 | 5027 | antisense |
| novel_circ_004366 | ENSSSCG00000010456 | 14 | - | 1.01E+08 | 1.01E+08 | 607 | annot_exons |
| novel_circ_004367 | ENSSSCG00000005444 | 1 | - | 2.5E+08 | 2.5E+08 | 401 | annot_exons |
| novel_circ_004368 | NA | 15 | - | 84264337 | 84264530 | 194 | intergenic |
| novel_circ_004369 | ENSSSCG00000004143 | 1 | - | 25232015 | 25232203 | 189 | antisense |
| novel_circ_004370 | ENSSSCG00000014117 | 2 | + | 88764474 | 88772689 | 739 | annot_exons |
| novel_circ_004371 | ENSSSCG00000011521 | 13 | - | 54675580 | 54678797 | 336 | annot_exons |
| novel_circ_004372 | ENSSSCG00000014070 | 2 | - | 82708191 | 82710619 | 225 | annot_exons |
| novel_circ_004373 | ENSSSCG00000008942 | 8 | + | 67674329 | 67680849 | 261 | annot_exons |
| novel_circ_004374 | ENSSSCG00000020663 | 18 | + | 5266215 | 5271994 | 1636 | annot_exons |
| novel_circ_004375 | ENSSSCG00000002426 | 7 | + | 1.1E+08 | 1.1E+08 | 257 | annot_exons |
| novel_circ_004376 | ENSSSCG00000010360 | 14 | + | 87822731 | 87854827 | 341 | annot_exons |
| novel_circ_004377 | ENSSSCG00000015499 | 9 | + | 1.17E+08 | 1.17E+08 | 270 | annot_exons |
| novel_circ_004378 | NA | 15 | - | 84262528 | 84263008 | 481 | intergenic |
| novel_circ_004379 | ENSSSCG00000000161 | 5 | - | 12707999 | 12710312 | 271 | annot_exons |
| novel_circ_004380 | ENSSSCG00000034704 | 1 | - | 1.62E+08 | 1.62E+08 | 445 | annot_exons |
| novel_circ_004381 | ENSSSCG00000009551 | 11 | + | 77489152 | 77510259 | 836 | annot_exons |
| novel_circ_004382 | ENSSSCG00000030343 | 16 | - | 34798441 | 34803686 | 363 | annot_exons |
| novel_circ_004383 | ENSSSCG00000016033 | 15 | + | 93074445 | 93159114 | 1153 | annot_exons |
| novel_circ_004384 | ENSSSCG00000000784 | 5 | + | 71844424 | 71847150 | 522 | annot_exons |
| novel_circ_004385 | ENSSSCG00000004558 | 1 | - | 1.08E+08 | 1.08E+08 | 514 | antisense |
| novel_circ_004386 | ENSSSCG00000032549 | 3 | - | 2053313 | 2054665 | 1353 | intronic |
| novel_circ_004387 | ENSSSCG00000017401 | 12 | - | 20312323 | 20313397 | 363 | annot_exons |
| novel_circ_004388 | ENSSSCG00000009029 | 8 | - | 80870172 | 80876761 | 332 | annot_exons |
| novel_circ_004389 | NA | 18 | - | 8954742 | 8956187 | 1446 | intergenic |
| novel_circ_004390 | ENSSSCG00000011772 | 13 | + | 1.21E+08 | 1.21E+08 | 377 | annot_exons |
| novel_circ_004391 | ENSSSCG00000022758 | 15 | - | 1.18E+08 | 1.18E+08 | 345 | annot_exons |
| novel_circ_004392 | ENSSSCG00000007805 | 3 | + | 18553400 | 18560385 | 956 | annot_exons |
| novel_circ_004393 | ENSSSCG00000010640 | 14 | - | 1.24E+08 | 1.24E+08 | 556 | exon_intron |
| novel_circ_004394 | ENSSSCG00000021200 | X | - | 58351585 | 58384217 | 2547 | annot_exons |
| novel_circ_004395 | ENSSSCG00000000078 | 5 | - | 8086844 | 8087658 | 412 | annot_exons |
| novel_circ_004396 | ENSSSCG00000007599 | 3 | + | 5347407 | 5368914 | 551 | annot_exons |
| novel_circ_004397 | ENSSSCG00000015599 | 9 | - | 1.31E+08 | 1.31E+08 | 392 | annot_exons |
| novel_circ_004398 | ENSSSCG00000004394 | 1 | + | 77124446 | 77124674 | 229 | one_exon |
| novel_circ_004399 | ENSSSCG00000016572 | 18 | + | 19651436 | 19653128 | 252 | annot_exons |
| novel_circ_004400 | ENSSSCG00000003589 | 6 | - | 86095842 | 86096363 | 522 | one_exon |
| novel_circ_004401 | ENSSSCG00000024784 | 10 | - | 23548827 | 23549237 | 296 | annot_exons |
| novel_circ_004402 | ENSSSCG00000037305 | 8 | + | 33016128 | 33026134 | 773 | exon_intron |
| novel_circ_004403 | ENSSSCG00000013539 | 2 | + | 72688897 | 72690859 | 267 | annot_exons |
| novel_circ_004404 | ENSSSCG00000003772 | 6 | + | 1.36E+08 | 1.36E+08 | 1842 | annot_exons |
| novel_circ_004405 | ENSSSCG00000008192 | 3 | + | 56435331 | 56436620 | 482 | annot_exons |
| novel_circ_004406 | ENSSSCG00000023784 | 9 | + | 99368230 | 99385468 | 816 | annot_exons |
| novel_circ_004407 | ENSSSCG00000036659 | 3 | - | 16916706 | 16925579 | 268 | annot_exons |
| novel_circ_004408 | ENSSSCG00000011652 | 13 | - | 77750384 | 77758619 | 528 | annot_exons |
| novel_circ_004409 | ENSSSCG00000009112 | 8 | + | 1.05E+08 | 1.05E+08 | 433 | annot_exons |
| novel_circ_004410 | ENSSSCG00000002540 | AEMK02000452.1 | + | 764826 | 773915 | 558 | annot_exons |
| novel_circ_004411 | ENSSSCG00000017136 | 12 | - | 413917 | 435701 | 789 | annot_exons |
| novel_circ_004412 | ENSSSCG00000024694 | 17 | - | 61492556 | 61493585 | 243 | annot_exons |
| novel_circ_004413 | ENSSSCG00000002799 | 6 | - | 20317779 | 20319063 | 395 | annot_exons |
| novel_circ_004414 | ENSSSCG00000025534 | 17 | + | 50789950 | 50794980 | 1927 | annot_exons |
| novel_circ_004415 | ENSSSCG00000009384 | 11 | + | 16455084 | 16457143 | 502 | annot_exons |
| novel_circ_004416 | ENSSSCG00000011332 | 13 | - | 29898476 | 29925370 | 1175 | annot_exons |
| novel_circ_004417 | ENSSSCG00000011678 | 13 | - | 83151533 | 83156879 | 546 | annot_exons |
| novel_circ_004418 | ENSSSCG00000008201 | 3 | - | 56797432 | 56798148 | 717 | one_exon |
| novel_circ_004419 | ENSSSCG00000006032 | 4 | - | 29014534 | 29047618 | 9641 | annot_exons |
| novel_circ_004420 | ENSSSCG00000023324 | 9 | + | 45796974 | 45797447 | 185 | annot_exons |
| novel_circ_004421 | ENSSSCG00000001532 | 7 | + | 30763473 | 30765053 | 294 | annot_exons |
| novel_circ_004422 | ENSSSCG00000011516 | 13 | - | 53090589 | 53106944 | 452 | annot_exons |
| novel_circ_004423 | ENSSSCG00000005343 | 1 | - | 2.37E+08 | 2.37E+08 | 262 | one_exon |
| novel_circ_004424 | ENSSSCG00000010513 | 14 | + | 1.08E+08 | 1.08E+08 | 515 | annot_exons |
| novel_circ_004425 | ENSSSCG00000016118 | 15 | + | 1.07E+08 | 1.07E+08 | 1014 | annot_exons |
| novel_circ_004426 | ENSSSCG00000042492 | 18 | + | 14923086 | 14926945 | 3860 | antisense |
| novel_circ_004427 | ENSSSCG00000005038 | 1 | + | 1.82E+08 | 1.82E+08 | 186 | annot_exons |
| novel_circ_004428 | ENSSSCG00000017592 | 12 | - | 27474893 | 27475842 | 459 | annot_exons |
| novel_circ_004429 | NA | 1 | - | 1.8E+08 | 1.8E+08 | 12593 | intergenic |
| novel_circ_004430 | ENSSSCG00000014304 | 2 | + | 1.37E+08 | 1.37E+08 | 906 | annot_exons |
| novel_circ_004431 | ENSSSCG00000010175 | 14 | - | 59220451 | 59230895 | 490 | annot_exons |
| novel_circ_004432 | ENSSSCG00000035934 | 1 | - | 1.82E+08 | 1.82E+08 | 510 | annot_exons |
| novel_circ_004433 | ENSSSCG00000013392 | 2 | + | 44767177 | 44773133 | 746 | annot_exons |
| novel_circ_004434 | NA | 2 | + | 77005328 | 77005621 | 294 | intergenic |
| novel_circ_004435 | ENSSSCG00000020868 | 8 | + | 43969450 | 43971598 | 412 | annot_exons |
| novel_circ_004436 | ENSSSCG00000011498 | 13 | + | 47760569 | 47764526 | 267 | annot_exons |
| novel_circ_004437 | ENSSSCG00000005366 | 1 | - | 2.39E+08 | 2.39E+08 | 28705 | antisense |
| novel_circ_004438 | ENSSSCG00000029857 | X | + | 20162284 | 20165966 | 359 | annot_exons |
| novel_circ_004439 | ENSSSCG00000024618 | 10 | + | 19949148 | 19952852 | 249 | annot_exons |
| novel_circ_004440 | ENSSSCG00000011646 | 13 | - | 75744118 | 75755826 | 566 | exon_intron |
| novel_circ_004441 | ENSSSCG00000008604 | 3 | + | 1.18E+08 | 1.18E+08 | 422 | annot_exons |
| novel_circ_004442 | ENSSSCG00000021184 | 4 | - | 46892870 | 46905852 | 576 | annot_exons |
| novel_circ_004443 | ENSSSCG00000009018 | 8 | + | 77823704 | 77839713 | 1516 | annot_exons |
| novel_circ_004444 | ENSSSCG00000021620 | 2 | - | 7912378 | 7919742 | 1056 | annot_exons |
| novel_circ_004445 | ENSSSCG00000035934 | 1 | - | 1.82E+08 | 1.82E+08 | 647 | annot_exons |
| novel_circ_004446 | ENSSSCG00000015247 | 9 | + | 56951048 | 56951531 | 484 | antisense |
| novel_circ_004447 | ENSSSCG00000010987 | 10 | + | 32855276 | 32866152 | 744 | annot_exons |
| novel_circ_004448 | ENSSSCG00000005423 | 1 | - | 2.46E+08 | 2.46E+08 | 523 | annot_exons |
| novel_circ_004449 | ENSSSCG00000000902 | 5 | + | 88263304 | 88271508 | 464 | annot_exons |
| novel_circ_004450 | NA | 7 | + | 51925087 | 51925239 | 153 | intergenic |
| novel_circ_004451 | ENSSSCG00000032984 | 10 | + | 48206021 | 48225744 | 918 | annot_exons |
| novel_circ_004452 | NA | 15 | + | 84241340 | 84242222 | 883 | intergenic |
| novel_circ_004453 | ENSSSCG00000017617 | 12 | + | 33277944 | 33290715 | 820 | annot_exons |
| novel_circ_004454 | ENSSSCG00000040634 | 1 | - | 2.42E+08 | 2.42E+08 | 301 | annot_exons |
| novel_circ_004455 | ENSSSCG00000001779 | 7 | + | 49003745 | 49021872 | 724 | annot_exons |
| novel_circ_004456 | ENSSSCG00000026044 | 14 | + | 14989270 | 14992789 | 321 | annot_exons |
| novel_circ_004457 | ENSSSCG00000016157 | 15 | - | 1.13E+08 | 1.13E+08 | 355 | intronic |
| novel_circ_004458 | ENSSSCG00000017277 | 12 | + | 14588252 | 14598384 | 711 | annot_exons |
| novel_circ_004459 | ENSSSCG00000004070 | 1 | - | 12050189 | 12058925 | 700 | annot_exons |
| novel_circ_004460 | ENSSSCG00000015692 | 15 | - | 16262628 | 16286227 | 576 | annot_exons |
| novel_circ_004461 | ENSSSCG00000000877 | 5 | - | 83838720 | 83862466 | 658 | annot_exons |
| novel_circ_004462 | ENSSSCG00000006274 | 4 | + | 79784917 | 79814317 | 3224 | annot_exons |
| novel_circ_004463 | ENSSSCG00000009047 | 8 | - | 84211727 | 84214973 | 268 | annot_exons |
| novel_circ_004464 | ENSSSCG00000025703 | 1 | - | 77278779 | 77279692 | 914 | one_exon |
| novel_circ_004465 | ENSSSCG00000034516 | 1 | - | 1.8E+08 | 1.8E+08 | 527 | annot_exons |
| novel_circ_004466 | ENSSSCG00000008799 | 8 | + | 32773630 | 32780660 | 570 | annot_exons |
| novel_circ_004467 | ENSSSCG00000003859 | 6 | + | 1.6E+08 | 1.6E+08 | 448 | one_exon |
| novel_circ_004468 | ENSSSCG00000014427 | 2 | + | 1.5E+08 | 1.5E+08 | 1326 | annot_exons |
| novel_circ_004469 | ENSSSCG00000007458 | 17 | + | 49650743 | 49653096 | 557 | annot_exons |
| novel_circ_004470 | NA | 18 | - | 2578336 | 2580821 | 2486 | intergenic |
| novel_circ_004471 | ENSSSCG00000004535 | 1 | - | 1.05E+08 | 1.05E+08 | 177 | annot_exons |
| novel_circ_004472 | ENSSSCG00000034677 | 15 | - | 84407065 | 84407963 | 344 | annot_exons |
| novel_circ_004473 | ENSSSCG00000014291 | 2 | - | 1.35E+08 | 1.35E+08 | 336 | annot_exons |
| novel_circ_004474 | ENSSSCG00000032622 | 14 | - | 6687136 | 6688472 | 1337 | antisense |
| novel_circ_004475 | ENSSSCG00000014838 | 9 | - | 8706351 | 8709627 | 663 | annot_exons |
| novel_circ_004476 | ENSSSCG00000004508 | 1 | - | 98882666 | 98885389 | 305 | annot_exons |
| novel_circ_004477 | ENSSSCG00000016192 | 15 | + | 1.21E+08 | 1.21E+08 | 258 | annot_exons |
| novel_circ_004478 | ENSSSCG00000008415 | 3 | + | 87419739 | 87449950 | 795 | annot_exons |
| novel_circ_004479 | ENSSSCG00000014304 | 2 | + | 1.37E+08 | 1.37E+08 | 483 | one_exon |
| novel_circ_004480 | ENSSSCG00000016968 | 16 | + | 47810330 | 47814614 | 323 | annot_exons |
| novel_circ_004481 | ENSSSCG00000016114 | 15 | + | 1.06E+08 | 1.06E+08 | 359 | annot_exons |
| novel_circ_004482 | ENSSSCG00000001732 | 7 | - | 43609181 | 43620359 | 1603 | annot_exons |
| novel_circ_004483 | ENSSSCG00000016629 | 18 | - | 29115878 | 29142960 | 812 | annot_exons |
| novel_circ_004484 | ENSSSCG00000004207 | 1 | - | 34079218 | 34096613 | 476 | annot_exons |
| novel_circ_004485 | ENSSSCG00000017073 | 16 | + | 69125276 | 69126691 | 438 | annot_exons |
| novel_circ_004486 | ENSSSCG00000046003 | 1 | - | 2.73E+08 | 2.73E+08 | 598 | annot_exons |
| novel_circ_004487 | ENSSSCG00000004600 | 1 | + | 1.15E+08 | 1.15E+08 | 179 | antisense |
| novel_circ_004488 | ENSSSCG00000032556 | 3 | + | 18258631 | 18260169 | 950 | annot_exons |
| novel_circ_004489 | ENSSSCG00000014177 | 2 | - | 1.05E+08 | 1.05E+08 | 2539 | annot_exons |
| novel_circ_004490 | ENSSSCG00000012035 | 13 | + | 1.96E+08 | 1.96E+08 | 259 | antisense |
| novel_circ_004491 | ENSSSCG00000011194 | 13 | - | 2958044 | 2964480 | 1048 | exon_intron |
| novel_circ_004492 | ENSSSCG00000003798 | 6 | + | 1.45E+08 | 1.45E+08 | 205 | annot_exons |
| novel_circ_004493 | ENSSSCG00000003398 | 6 | + | 70429225 | 70436457 | 775 | annot_exons |
| novel_circ_004494 | ENSSSCG00000025777 | 1 | - | 14456953 | 14457143 | 191 | one_exon |
| novel_circ_004495 | ENSSSCG00000023001 | 8 | - | 18834654 | 18840153 | 476 | annot_exons |
| novel_circ_004496 | ENSSSCG00000006779 | 4 | + | 1.08E+08 | 1.08E+08 | 350 | annot_exons |
| novel_circ_004497 | ENSSSCG00000028225 | 7 | - | 1.16E+08 | 1.16E+08 | 266 | annot_exons |
| novel_circ_004498 | ENSSSCG00000008048 | 3 | + | 39742506 | 39744782 | 639 | annot_exons |
| novel_circ_004499 | ENSSSCG00000011833 | 13 | + | 1.33E+08 | 1.33E+08 | 404 | annot_exons |
| novel_circ_004500 | ENSSSCG00000023761 | 6 | - | 81208179 | 81219203 | 669 | annot_exons |
| novel_circ_004501 | NA | 2 | - | 6808791 | 6809191 | 401 | intergenic |
| novel_circ_004502 | NA | 14 | - | 41639323 | 41643575 | 4253 | intergenic |
| novel_circ_004503 | ENSSSCG00000011238 | 13 | - | 21079030 | 21079241 | 212 | antisense |
| novel_circ_004504 | ENSSSCG00000027314 | 10 | + | 23350909 | 23351129 | 221 | one_exon |
| novel_circ_004505 | ENSSSCG00000012743 | X | + | 1.22E+08 | 1.22E+08 | 444 | annot_exons |
| novel_circ_004506 | ENSSSCG00000024621 | 13 | + | 7021801 | 7038479 | 273 | annot_exons |
| novel_circ_004507 | ENSSSCG00000015940 | 15 | + | 76207329 | 76232137 | 1130 | annot_exons |
| novel_circ_004508 | ENSSSCG00000018058 | 12 | - | 23625258 | 23625967 | 326 | annot_exons |
| novel_circ_004509 | ENSSSCG00000005935 | 4 | + | 2986971 | 2989523 | 434 | annot_exons |
| novel_circ_004510 | ENSSSCG00000003673 | 6 | - | 96699486 | 96703322 | 464 | annot_exons |
| novel_circ_004511 | ENSSSCG00000014215 | 2 | - | 1.19E+08 | 1.19E+08 | 446 | one_exon |
| novel_circ_004512 | ENSSSCG00000012059 | 13 | - | 2.01E+08 | 2.01E+08 | 272 | one_exon |
| novel_circ_004513 | ENSSSCG00000038397 | 4 | + | 2868532 | 2887088 | 342 | annot_exons |
| novel_circ_004514 | ENSSSCG00000016874 | 16 | - | 28111895 | 28113952 | 238 | annot_exons |
| novel_circ_004515 | ENSSSCG00000004103 | 1 | - | 16213409 | 16220396 | 424 | annot_exons |
| novel_circ_004516 | ENSSSCG00000006386 | 4 | + | 90161231 | 90163552 | 346 | annot_exons |
| novel_circ_004517 | ENSSSCG00000003627 | 6 | + | 91593753 | 91596635 | 420 | annot_exons |
| novel_circ_004518 | ENSSSCG00000026733 | 18 | + | 10082517 | 10087363 | 516 | annot_exons |
| novel_circ_004519 | ENSSSCG00000010862 | 10 | - | 14666448 | 14667955 | 242 | annot_exons |
| novel_circ_004520 | ENSSSCG00000009085 | 8 | + | 1.01E+08 | 1.01E+08 | 648 | annot_exons |
| novel_circ_004521 | ENSSSCG00000008621 | 3 | + | 1.22E+08 | 1.22E+08 | 385 | exon_intron |
| novel_circ_004522 | ENSSSCG00000026819 | 14 | + | 55301204 | 55308756 | 399 | annot_exons |
| novel_circ_004523 | ENSSSCG00000008412 | 3 | + | 87251850 | 87262951 | 453 | annot_exons |
| novel_circ_004524 | ENSSSCG00000006197 | 4 | - | 65613993 | 65620622 | 698 | annot_exons |
| novel_circ_004525 | ENSSSCG00000006089 | 4 | - | 40335139 | 40339022 | 311 | annot_exons |
| novel_circ_004526 | ENSSSCG00000002844 | 6 | - | 37077213 | 37166517 | 1631 | annot_exons |
| novel_circ_004527 | NA | 15 | - | 84366974 | 84374289 | 7316 | intergenic |
| novel_circ_004528 | ENSSSCG00000011197 | 13 | + | 3404652 | 3405113 | 182 | annot_exons |
| novel_circ_004529 | ENSSSCG00000020702 | 1 | - | 90172372 | 90174082 | 193 | annot_exons |
| novel_circ_004530 | ENSSSCG00000038708 | 7 | - | 48865867 | 48866128 | 262 | one_exon |
| novel_circ_004531 | NA | 15 | + | 84370126 | 84370326 | 201 | intergenic |
| novel_circ_004532 | ENSSSCG00000016532 | 18 | + | 14113730 | 14118790 | 486 | annot_exons |
| novel_circ_004533 | ENSSSCG00000015690 | 15 | - | 16192020 | 16197963 | 429 | annot_exons |
| novel_circ_004534 | ENSSSCG00000035914 | 6 | - | 91823115 | 91823349 | 235 | intronic |
| novel_circ_004535 | ENSSSCG00000017082 | 16 | + | 71363867 | 71364041 | 175 | intronic |
| novel_circ_004536 | ENSSSCG00000023984 | 3 | + | 31283103 | 31288541 | 744 | annot_exons |
| novel_circ_004537 | ENSSSCG00000011953 | 13 | + | 1.57E+08 | 1.57E+08 | 509 | annot_exons |
| novel_circ_004538 | ENSSSCG00000036052 | 15 | - | 84228006 | 84229361 | 700 | annot_exons |
| novel_circ_004539 | ENSSSCG00000013022 | 2 | - | 7416140 | 7417316 | 1177 | antisense |
| novel_circ_004540 | ENSSSCG00000015853 | 15 | + | 56479282 | 56493813 | 1994 | annot_exons |
| novel_circ_004541 | ENSSSCG00000031882 | 3 | + | 85614722 | 85622090 | 211 | annot_exons |
| novel_circ_004542 | ENSSSCG00000036926 | 1 | + | 2.67E+08 | 2.67E+08 | 19276 | exon_intron |
| novel_circ_004543 | ENSSSCG00000016194 | 15 | - | 1.21E+08 | 1.21E+08 | 447 | annot_exons |
| novel_circ_004544 | ENSSSCG00000034357 | 13 | + | 32059508 | 32070917 | 858 | annot_exons |
| novel_circ_004545 | ENSSSCG00000021440 | 4 | - | 1.11E+08 | 1.11E+08 | 303 | one_exon |
| novel_circ_004546 | ENSSSCG00000010240 | 14 | - | 71612798 | 71612986 | 189 | intronic |
| novel_circ_004547 | ENSSSCG00000004071 | 1 | + | 12353964 | 12373696 | 617 | annot_exons |
| novel_circ_004548 | ENSSSCG00000003872 | 6 | + | 1.61E+08 | 1.61E+08 | 593 | annot_exons |
| novel_circ_004549 | ENSSSCG00000038401 | 7 | - | 46340798 | 46341480 | 287 | annot_exons |
| novel_circ_004550 | ENSSSCG00000009142 | 8 | - | 1.13E+08 | 1.13E+08 | 1015 | annot_exons |
| novel_circ_004551 | ENSSSCG00000030685 | 5 | + | 76729220 | 76738914 | 421 | annot_exons |
| novel_circ_004552 | ENSSSCG00000011652 | 13 | - | 77417167 | 77431049 | 341 | annot_exons |
| novel_circ_004553 | NA | 18 | - | 36419020 | 36435503 | 16484 | intergenic |
| novel_circ_004554 | ENSSSCG00000011236 | 13 | - | 19411774 | 19415099 | 282 | annot_exons |
| novel_circ_004555 | ENSSSCG00000034816 | 6 | + | 1.04E+08 | 1.04E+08 | 457 | annot_exons |
| novel_circ_004556 | ENSSSCG00000006324 | 4 | + | 85041504 | 85042510 | 338 | annot_exons |
| novel_circ_004557 | ENSSSCG00000010956 | 10 | - | 29300932 | 29304243 | 358 | annot_exons |
| novel_circ_004558 | ENSSSCG00000029805 | 2 | + | 1.02E+08 | 1.02E+08 | 728 | annot_exons |
| novel_circ_004559 | ENSSSCG00000017672 | 12 | + | 36394021 | 36394937 | 917 | one_exon |
| novel_circ_004560 | ENSSSCG00000009090 | 8 | - | 1.02E+08 | 1.02E+08 | 401 | annot_exons |
| novel_circ_004561 | ENSSSCG00000014252 | 2 | + | 1.3E+08 | 1.3E+08 | 1079 | annot_exons |
| novel_circ_004562 | ENSSSCG00000029464 | 5 | - | 67674585 | 67681947 | 660 | annot_exons |
| novel_circ_004563 | ENSSSCG00000014146 | 2 | + | 94936432 | 94950343 | 725 | annot_exons |
| novel_circ_004564 | ENSSSCG00000028475 | 13 | + | 1.38E+08 | 1.38E+08 | 564 | annot_exons |
| novel_circ_004565 | ENSSSCG00000008989 | 8 | - | 73238320 | 73262907 | 485 | annot_exons |
| novel_circ_004566 | ENSSSCG00000003776 | 6 | - | 1.38E+08 | 1.38E+08 | 477 | annot_exons |
| novel_circ_004567 | ENSSSCG00000036885 | 13 | - | 1.68E+08 | 1.68E+08 | 707 | annot_exons |
| novel_circ_004568 | ENSSSCG00000038561 | 15 | - | 84412678 | 84413903 | 843 | annot_exons |
| novel_circ_004569 | ENSSSCG00000018016 | 12 | + | 56464310 | 56473522 | 295 | annot_exons |
| novel_circ_004570 | ENSSSCG00000004318 | 1 | - | 57385549 | 57393736 | 607 | annot_exons |
| novel_circ_004571 | ENSSSCG00000011103 | 10 | - | 57297022 | 57351952 | 1207 | exon_intron |
| novel_circ_004572 | ENSSSCG00000020663 | 18 | + | 5252444 | 5254144 | 1701 | one_exon |
| novel_circ_004573 | ENSSSCG00000008772 | 8 | - | 29141456 | 29155524 | 592 | annot_exons |
| novel_circ_004574 | ENSSSCG00000013367 | 2 | - | 40884644 | 40885927 | 359 | annot_exons |
| novel_circ_004575 | ENSSSCG00000013744 | 2 | + | 65943005 | 65943316 | 229 | annot_exons |
| novel_circ_004576 | ENSSSCG00000003566 | 6 | + | 84249236 | 84249534 | 219 | annot_exons |
| novel_circ_004577 | ENSSSCG00000005073 | 1 | + | 1.87E+08 | 1.87E+08 | 268 | annot_exons |
| novel_circ_004578 | ENSSSCG00000010928 | 10 | - | 24788311 | 24799565 | 507 | annot_exons |
| novel_circ_004579 | ENSSSCG00000004717 | 1 | + | 1.28E+08 | 1.28E+08 | 316 | annot_exons |
| novel_circ_004580 | ENSSSCG00000037424 | 1 | - | 76722000 | 76727151 | 1358 | exon_intron |
| novel_circ_004581 | ENSSSCG00000004403 | 1 | + | 76222893 | 76223628 | 250 | annot_exons |
| novel_circ_004582 | ENSSSCG00000030291 | 4 | + | 98646177 | 98646371 | 195 | antisense |
| novel_circ_004583 | ENSSSCG00000016863 | 16 | - | 26587566 | 26658501 | 12286 | exon_intron |
| novel_circ_004584 | ENSSSCG00000036537 | 2 | - | 75202144 | 75202291 | 148 | one_exon |
| novel_circ_004585 | ENSSSCG00000004739 | 1 | - | 1.3E+08 | 1.3E+08 | 1115 | annot_exons |
| novel_circ_004586 | ENSSSCG00000014252 | 2 | + | 1.3E+08 | 1.3E+08 | 532 | annot_exons |
| novel_circ_004587 | ENSSSCG00000000972 | 5 | + | 448264 | 450376 | 375 | annot_exons |
| novel_circ_004588 | ENSSSCG00000009276 | 11 | - | 1141224 | 1186009 | 1259 | annot_exons |
| novel_circ_004589 | ENSSSCG00000015097 | 9 | - | 46038901 | 46043475 | 464 | annot_exons |
| novel_circ_004590 | ENSSSCG00000017256 | 12 | + | 11258622 | 11261616 | 482 | annot_exons |
| novel_circ_004591 | ENSSSCG00000010654 | 14 | + | 1.26E+08 | 1.26E+08 | 385 | annot_exons |
| novel_circ_004592 | ENSSSCG00000007805 | 3 | + | 18565742 | 18565962 | 221 | one_exon |
| novel_circ_004593 | ENSSSCG00000015238 | 9 | - | 56044142 | 56068731 | 495 | annot_exons |
| novel_circ_004594 | ENSSSCG00000020743 | 1 | + | 2.64E+08 | 2.64E+08 | 261 | annot_exons |
| novel_circ_004595 | ENSSSCG00000006840 | 4 | + | 1.11E+08 | 1.11E+08 | 479 | annot_exons |
| novel_circ_004596 | ENSSSCG00000016845 | 16 | - | 22567969 | 22578996 | 881 | annot_exons |
| novel_circ_004597 | ENSSSCG00000006864 | 4 | - | 1.18E+08 | 1.18E+08 | 588 | annot_exons |
| novel_circ_004598 | ENSSSCG00000024486 | 17 | + | 11848370 | 11850285 | 184 | annot_exons |
| novel_circ_004599 | ENSSSCG00000014347 | 2 | + | 1.41E+08 | 1.41E+08 | 404 | antisense |
| novel_circ_004600 | ENSSSCG00000016834 | 16 | - | 21449905 | 21451895 | 431 | annot_exons |
| novel_circ_004601 | ENSSSCG00000016629 | 18 | - | 29135523 | 29142960 | 243 | annot_exons |
| novel_circ_004602 | ENSSSCG00000010169 | 14 | + | 58280692 | 58291727 | 1010 | annot_exons |
| novel_circ_004603 | ENSSSCG00000004541 | 1 | - | 1.07E+08 | 1.07E+08 | 341 | annot_exons |
| novel_circ_004604 | ENSSSCG00000007544 | 3 | + | 501359 | 507810 | 657 | annot_exons |
| novel_circ_004605 | ENSSSCG00000033958 | 7 | - | 80919227 | 80956041 | 1142 | annot_exons |
| novel_circ_004606 | ENSSSCG00000029029 | 3 | - | 16964040 | 16971275 | 739 | annot_exons |
| novel_circ_004607 | ENSSSCG00000000107 | 5 | + | 9607844 | 9608237 | 260 | annot_exons |
| novel_circ_004608 | ENSSSCG00000007981 | 3 | + | 41532734 | 41533739 | 295 | annot_exons |
| novel_circ_004609 | ENSSSCG00000013302 | 2 | - | 26516504 | 26518029 | 242 | annot_exons |
| novel_circ_004610 | ENSSSCG00000011237 | 13 | + | 19630228 | 19635663 | 525 | annot_exons |
| novel_circ_004611 | ENSSSCG00000000068 | 5 | - | 7341106 | 7341743 | 188 | annot_exons |
| novel_circ_004612 | ENSSSCG00000014098 | 2 | - | 86329278 | 86340231 | 1001 | annot_exons |
| novel_circ_004613 | ENSSSCG00000015211 | 9 | + | 52406720 | 52419957 | 13238 | exon_intron |
| novel_circ_004614 | ENSSSCG00000000458 | 5 | + | 27173898 | 27181752 | 737 | annot_exons |
| novel_circ_004615 | ENSSSCG00000011467 | 13 | - | 39482886 | 39486529 | 314 | annot_exons |
| novel_circ_004616 | ENSSSCG00000007805 | 3 | + | 18559247 | 18563733 | 666 | annot_exons |
| novel_circ_004617 | ENSSSCG00000017024 | 16 | + | 60350373 | 60355331 | 4959 | antisense |
| novel_circ_004618 | ENSSSCG00000022032 | 4 | - | 1.29E+08 | 1.29E+08 | 1116 | annot_exons |
| novel_circ_004619 | ENSSSCG00000017672 | 12 | + | 36362691 | 36367060 | 813 | annot_exons |
| novel_circ_004620 | ENSSSCG00000012108 | X | + | 6859159 | 6871558 | 471 | annot_exons |
| novel_circ_004621 | ENSSSCG00000000496 | 5 | + | 34018844 | 34020069 | 535 | annot_exons |
| novel_circ_004622 | ENSSSCG00000011776 | 13 | + | 1.22E+08 | 1.22E+08 | 278 | annot_exons |
| novel_circ_004623 | ENSSSCG00000004535 | 1 | - | 1.05E+08 | 1.05E+08 | 1175 | annot_exons |
| novel_circ_004624 | ENSSSCG00000010144 | 14 | - | 54678768 | 54684046 | 645 | annot_exons |
| novel_circ_004625 | ENSSSCG00000004739 | 1 | - | 1.3E+08 | 1.3E+08 | 662 | one_exon |
| novel_circ_004626 | ENSSSCG00000017861 | 12 | + | 49617557 | 49619336 | 290 | annot_exons |
| novel_circ_004627 | ENSSSCG00000026113 | 13 | + | 1.45E+08 | 1.45E+08 | 16520 | exon_intron |
| novel_circ_004628 | ENSSSCG00000005052 | 1 | - | 1.84E+08 | 1.84E+08 | 259 | annot_exons |
| novel_circ_004629 | ENSSSCG00000038870 | AEMK02000510.1 | - | 611865 | 615228 | 342 | annot_exons |
| novel_circ_004630 | ENSSSCG00000027530 | 6 | - | 85627585 | 85628633 | 369 | annot_exons |
| novel_circ_004631 | ENSSSCG00000033971 | 1 | - | 1.49E+08 | 1.49E+08 | 372 | annot_exons |
| novel_circ_004632 | ENSSSCG00000015071 | 9 | - | 44278271 | 44332021 | 4856 | annot_exons |
| novel_circ_004633 | ENSSSCG00000040728 | 4 | + | 64184610 | 64201174 | 432 | annot_exons |
| novel_circ_004634 | ENSSSCG00000021408 | 13 | - | 35363068 | 35363185 | 118 | one_exon |
| novel_circ_004635 | ENSSSCG00000002869 | 6 | + | 43867657 | 43867820 | 164 | one_exon |
| novel_circ_004636 | ENSSSCG00000005317 | 1 | - | 2.36E+08 | 2.36E+08 | 2008 | annot_exons |
| novel_circ_004637 | ENSSSCG00000005724 | 1 | - | 2.72E+08 | 2.72E+08 | 504 | annot_exons |
| novel_circ_004638 | ENSSSCG00000004856 | 1 | - | 1.46E+08 | 1.46E+08 | 573 | annot_exons |
| novel_circ_004639 | ENSSSCG00000004725 | 1 | - | 1.29E+08 | 1.29E+08 | 167 | antisense |
| novel_circ_004640 | ENSSSCG00000016409 | 18 | - | 1589912 | 1599809 | 815 | annot_exons |
| novel_circ_004641 | ENSSSCG00000004620 | 1 | + | 1.19E+08 | 1.19E+08 | 428 | annot_exons |
| novel_circ_004642 | ENSSSCG00000008540 | 3 | - | 1.1E+08 | 1.1E+08 | 540 | annot_exons |
| novel_circ_004643 | ENSSSCG00000009230 | 8 | + | 1.34E+08 | 1.34E+08 | 293 | annot_exons |
| novel_circ_004644 | ENSSSCG00000015590 | 9 | - | 1.31E+08 | 1.31E+08 | 407 | annot_exons |
| novel_circ_004645 | ENSSSCG00000001924 | 7 | - | 60748007 | 60757895 | 294 | annot_exons |
| novel_circ_004646 | ENSSSCG00000022145 | 6 | - | 1.29E+08 | 1.29E+08 | 4459 | annot_exons |
| novel_circ_004647 | ENSSSCG00000028035 | 9 | + | 64529138 | 64531386 | 325 | annot_exons |
| novel_circ_004648 | ENSSSCG00000016243 | 15 | + | 1.28E+08 | 1.28E+08 | 641 | annot_exons |
| novel_circ_004649 | ENSSSCG00000009480 | 11 | + | 51531489 | 51531654 | 166 | one_exon |
| novel_circ_004650 | ENSSSCG00000028097 | 6 | + | 1.66E+08 | 1.66E+08 | 331 | one_exon |
| novel_circ_004651 | ENSSSCG00000009554 | 11 | - | 78622155 | 78622340 | 186 | one_exon |
| novel_circ_004652 | ENSSSCG00000001862 | 7 | - | 56462719 | 56526919 | 1794 | annot_exons |
| novel_circ_004653 | ENSSSCG00000010987 | 10 | + | 32840622 | 32843863 | 308 | annot_exons |
| novel_circ_004654 | ENSSSCG00000016769 | 18 | - | 54336795 | 54404485 | 1569 | annot_exons |
| novel_circ_004655 | ENSSSCG00000011772 | 13 | + | 1.21E+08 | 1.21E+08 | 616 | annot_exons |
| novel_circ_004656 | ENSSSCG00000001862 | 7 | - | 56509179 | 56519340 | 465 | annot_exons |
| novel_circ_004657 | ENSSSCG00000024373 | 15 | - | 1.31E+08 | 1.31E+08 | 245 | annot_exons |
| novel_circ_004658 | ENSSSCG00000015818 | 15 | - | 48273692 | 48277942 | 625 | annot_exons |
| novel_circ_004659 | NA | 15 | + | 84246536 | 84246987 | 452 | intergenic |
| novel_circ_004660 | ENSSSCG00000032024 | 13 | - | 1.96E+08 | 1.96E+08 | 4586 | annot_exons |
| novel_circ_004661 | ENSSSCG00000022486 | 13 | + | 1.54E+08 | 1.54E+08 | 647 | annot_exons |
| novel_circ_004662 | ENSSSCG00000041457 | 1 | - | 1.94E+08 | 1.94E+08 | 250 | annot_exons |
| novel_circ_004663 | ENSSSCG00000008772 | 8 | - | 29136919 | 29155524 | 731 | annot_exons |
| novel_circ_004664 | ENSSSCG00000007246 | 17 | + | 35798731 | 35803202 | 681 | annot_exons |
| novel_circ_004665 | ENSSSCG00000025281 | 3 | + | 26726402 | 26730173 | 700 | annot_exons |
| novel_circ_004666 | ENSSSCG00000005388 | 1 | + | 2.42E+08 | 2.42E+08 | 287 | one_exon |
| novel_circ_004667 | ENSSSCG00000016597 | 18 | + | 22896628 | 22913514 | 366 | annot_exons |
| novel_circ_004668 | ENSSSCG00000012163 | X | - | 16498488 | 16500039 | 268 | annot_exons |
| novel_circ_004669 | ENSSSCG00000021072 | 1 | - | 95571669 | 95572715 | 380 | annot_exons |
| novel_circ_004670 | ENSSSCG00000000941 | 5 | - | 99261643 | 99288129 | 2714 | annot_exons |
| novel_circ_004671 | ENSSSCG00000027410 | 17 | + | 37109691 | 37129133 | 912 | annot_exons |
| novel_circ_004672 | ENSSSCG00000001921 | 7 | - | 60160838 | 60162330 | 276 | annot_exons |
| novel_circ_004673 | ENSSSCG00000009526 | 11 | + | 70151355 | 70219859 | 264 | annot_exons |
| novel_circ_004674 | ENSSSCG00000012650 | X | + | 1.06E+08 | 1.06E+08 | 420 | annot_exons |
| novel_circ_004675 | ENSSSCG00000004729 | 1 | - | 1.29E+08 | 1.29E+08 | 728 | annot_exons |
| novel_circ_004676 | ENSSSCG00000007355 | 17 | + | 43775146 | 43777223 | 311 | annot_exons |
| novel_circ_004677 | ENSSSCG00000005078 | 1 | + | 1.88E+08 | 1.88E+08 | 2850 | intronic |
| novel_circ_004678 | ENSSSCG00000012287 | X | - | 43101228 | 43101456 | 229 | one_exon |
| novel_circ_004679 | ENSSSCG00000015857 | 15 | - | 59164161 | 59185795 | 1422 | annot_exons |
| novel_circ_004680 | ENSSSCG00000029482 | 14 | - | 1.33E+08 | 1.33E+08 | 487 | annot_exons |
| novel_circ_004681 | ENSSSCG00000004281 | 1 | + | 52447350 | 52467058 | 19709 | intronic |
| novel_circ_004682 | ENSSSCG00000013570 | 2 | - | 71609783 | 71610202 | 334 | annot_exons |
| novel_circ_004683 | ENSSSCG00000015539 | 9 | - | 1.23E+08 | 1.23E+08 | 656 | annot_exons |
| novel_circ_004684 | ENSSSCG00000004729 | 1 | - | 1.29E+08 | 1.29E+08 | 432 | annot_exons |
| novel_circ_004685 | ENSSSCG00000020743 | 1 | + | 2.64E+08 | 2.64E+08 | 1599 | annot_exons |
| novel_circ_004686 | ENSSSCG00000002410 | 7 | - | 1.04E+08 | 1.04E+08 | 531 | annot_exons |
| novel_circ_004687 | ENSSSCG00000035478 | 3 | - | 58044300 | 58048808 | 424 | annot_exons |
| novel_circ_004688 | ENSSSCG00000017268 | 12 | + | 13214453 | 13216025 | 312 | annot_exons |
| novel_circ_004689 | ENSSSCG00000036145 | 6 | - | 1.56E+08 | 1.56E+08 | 1326 | annot_exons |
| novel_circ_004690 | ENSSSCG00000028431 | 2 | + | 1.23E+08 | 1.23E+08 | 531 | annot_exons |
| novel_circ_004691 | ENSSSCG00000011193 | 13 | + | 2875544 | 2888086 | 673 | annot_exons |
| novel_circ_004692 | ENSSSCG00000037594 | 1 | - | 66367794 | 66370053 | 308 | annot_exons |
| novel_circ_004693 | ENSSSCG00000005785 | 1 | + | 1.46E+08 | 1.46E+08 | 326 | annot_exons |
| novel_circ_004694 | ENSSSCG00000023445 | 18 | - | 40705091 | 40710541 | 279 | annot_exons |
| novel_circ_004695 | ENSSSCG00000022155 | 8 | + | 20154184 | 20154599 | 251 | annot_exons |
| novel_circ_004696 | ENSSSCG00000015145 | 9 | - | 50516564 | 50520175 | 365 | annot_exons |
| novel_circ_004697 | ENSSSCG00000003857 | 6 | - | 1.59E+08 | 1.59E+08 | 2133 | annot_exons |
| novel_circ_004698 | ENSSSCG00000003589 | 6 | - | 86065117 | 86071240 | 1437 | annot_exons |
| novel_circ_004699 | ENSSSCG00000038359 | 12 | - | 52664160 | 52664445 | 286 | antisense |
| novel_circ_004700 | NA | 5 | - | 86859290 | 86868083 | 8794 | intergenic |
| novel_circ_004701 | ENSSSCG00000025953 | 6 | - | 29315536 | 29315744 | 209 | one_exon |
| novel_circ_004702 | ENSSSCG00000001499 | 7 | + | 29018969 | 29058245 | 422 | annot_exons |
| novel_circ_004703 | NA | 6 | + | 47356477 | 47356997 | 521 | intergenic |
| novel_circ_004704 | ENSSSCG00000011213 | 13 | - | 12542442 | 12542763 | 240 | annot_exons |
| novel_circ_004705 | ENSSSCG00000000274 | 5 | + | 18676552 | 18678182 | 367 | annot_exons |
| novel_circ_004706 | NA | 15 | - | 84262986 | 84263337 | 352 | intergenic |
| novel_circ_004707 | ENSSSCG00000015603 | 9 | + | 1.32E+08 | 1.32E+08 | 299 | one_exon |
| novel_circ_004708 | ENSSSCG00000031882 | 3 | + | 85601526 | 85604159 | 276 | annot_exons |
| novel_circ_004709 | ENSSSCG00000012884 | 2 | - | 4456428 | 4459058 | 204 | annot_exons |
| novel_circ_004710 | ENSSSCG00000008641 | 3 | + | 1.27E+08 | 1.27E+08 | 457 | annot_exons |
| novel_circ_004711 | ENSSSCG00000008372 | 3 | - | 78952890 | 78958596 | 495 | annot_exons |
| novel_circ_004712 | ENSSSCG00000000488 | 5 | + | 33124742 | 33132527 | 395 | annot_exons |
| novel_circ_004713 | ENSSSCG00000005466 | 1 | - | 2.53E+08 | 2.53E+08 | 3751 | annot_exons |
| novel_circ_004714 | ENSSSCG00000012642 | X | + | 1.02E+08 | 1.02E+08 | 275 | annot_exons |
| novel_circ_004715 | ENSSSCG00000007280 | 17 | + | 37900616 | 37901251 | 226 | annot_exons |
| novel_circ_004716 | ENSSSCG00000005288 | 1 | + | 2.32E+08 | 2.32E+08 | 557 | intronic |
| novel_circ_004717 | ENSSSCG00000010698 | 14 | - | 1.31E+08 | 1.31E+08 | 43847 | exon_intron |
| novel_circ_004718 | ENSSSCG00000038007 | 10 | - | 43903313 | 43918659 | 627 | annot_exons |
| novel_circ_004719 | ENSSSCG00000008642 | 3 | - | 1.27E+08 | 1.27E+08 | 659 | annot_exons |
| novel_circ_004720 | ENSSSCG00000033591 | 6 | - | 31859873 | 31867964 | 543 | annot_exons |
| novel_circ_004721 | ENSSSCG00000009142 | 8 | - | 1.13E+08 | 1.13E+08 | 314 | annot_exons |
| novel_circ_004722 | NA | 7 | + | 17350577 | 17350829 | 253 | intergenic |
| novel_circ_004723 | ENSSSCG00000011720 | 13 | - | 92532457 | 92532800 | 344 | antisense |
| novel_circ_004724 | ENSSSCG00000014364 | 2 | + | 1.42E+08 | 1.42E+08 | 463 | annot_exons |
| novel_circ_004725 | ENSSSCG00000010289 | 14 | - | 75379211 | 75390545 | 338 | annot_exons |
| novel_circ_004726 | ENSSSCG00000001000 | 7 | - | 2434078 | 2434861 | 224 | annot_exons |
| novel_circ_004727 | ENSSSCG00000026044 | 14 | + | 15009909 | 15010083 | 175 | one_exon |
| novel_circ_004728 | ENSSSCG00000010314 | 14 | + | 76813742 | 76814017 | 276 | antisense |
| novel_circ_004729 | ENSSSCG00000014267 | 2 | - | 1.34E+08 | 1.34E+08 | 336 | annot_exons |
| novel_circ_004730 | ENSSSCG00000010288 | 14 | - | 75233246 | 75295452 | 528 | annot_exons |
| novel_circ_004731 | ENSSSCG00000014023 | 2 | - | 79002338 | 79010277 | 288 | annot_exons |
| novel_circ_004732 | ENSSSCG00000015537 | 9 | + | 1.22E+08 | 1.22E+08 | 307 | annot_exons |
| novel_circ_004733 | ENSSSCG00000039393 | 1 | - | 1.33E+08 | 1.33E+08 | 394 | annot_exons |
| novel_circ_004734 | ENSSSCG00000029249 | 5 | - | 1.03E+08 | 1.03E+08 | 404 | annot_exons |
| novel_circ_004735 | ENSSSCG00000011949 | 13 | - | 1.57E+08 | 1.57E+08 | 542 | annot_exons |
| novel_circ_004736 | ENSSSCG00000017668 | 12 | + | 35955224 | 35960199 | 338 | annot_exons |
| novel_circ_004737 | ENSSSCG00000006619 | 4 | - | 97571565 | 97576948 | 363 | annot_exons |
| novel_circ_004738 | ENSSSCG00000010799 | 3 | + | 22740544 | 22740753 | 210 | one_exon |
| novel_circ_004739 | ENSSSCG00000025777 | 1 | - | 14408861 | 14457143 | 7788 | exon_intron |
| novel_circ_004740 | ENSSSCG00000031382 | 1 | - | 2.28E+08 | 2.28E+08 | 35693 | exon_intron |
| novel_circ_004741 | ENSSSCG00000006066 | 4 | + | 36710955 | 36720759 | 799 | annot_exons |
| novel_circ_004742 | ENSSSCG00000004387 | 1 | - | 74737540 | 74737788 | 249 | antisense |
| novel_circ_004743 | ENSSSCG00000017346 | 12 | + | 18507559 | 18508149 | 253 | annot_exons |
| novel_circ_004744 | NA | 15 | - | 84252842 | 84253677 | 836 | intergenic |
| novel_circ_004745 | ENSSSCG00000009134 | 8 | - | 1.12E+08 | 1.12E+08 | 631 | annot_exons |
| novel_circ_004746 | ENSSSCG00000008448 | 3 | + | 96136594 | 96137597 | 403 | annot_exons |
| novel_circ_004747 | ENSSSCG00000017024 | 16 | - | 60351743 | 60351937 | 195 | one_exon |
| novel_circ_004748 | ENSSSCG00000009889 | 14 | - | 39914895 | 39915739 | 288 | annot_exons |
| novel_circ_004749 | ENSSSCG00000033295 | 7 | + | 91104405 | 91105338 | 242 | annot_exons |
| novel_circ_004750 | ENSSSCG00000038397 | 4 | + | 2678086 | 2678726 | 641 | exon_intron |
| novel_circ_004751 | ENSSSCG00000016397 | 15 | + | 658177 | 659897 | 420 | annot_exons |
| novel_circ_004752 | ENSSSCG00000038561 | 15 | - | 84405258 | 84405721 | 354 | annot_exons |
| novel_circ_004753 | ENSSSCG00000021322 | X | + | 20243402 | 20247157 | 713 | annot_exons |
| novel_circ_004754 | ENSSSCG00000029547 | 15 | + | 95273050 | 95288509 | 556 | annot_exons |
| novel_circ_004755 | ENSSSCG00000030467 | 13 | - | 1.09E+08 | 1.09E+08 | 1377 | annot_exons |
| novel_circ_004756 | ENSSSCG00000010303 | 14 | - | 76456198 | 76457265 | 1068 | one_exon |
| novel_circ_004757 | ENSSSCG00000029119 | 17 | + | 62817354 | 62822006 | 505 | annot_exons |
| novel_circ_004758 | ENSSSCG00000005305 | 1 | + | 2.36E+08 | 2.36E+08 | 8669 | annot_exons |
| novel_circ_004759 | ENSSSCG00000028157 | 15 | + | 1.05E+08 | 1.05E+08 | 245 | annot_exons |
| novel_circ_004760 | ENSSSCG00000038465 | 6 | - | 98229606 | 98240807 | 8082 | exon_intron |
| novel_circ_004761 | ENSSSCG00000036062 | AEMK02000697.1 | + | 163763 | 163931 | 169 | antisense |
| novel_circ_004762 | ENSSSCG00000012885 | 2 | - | 4615482 | 4623769 | 595 | annot_exons |
| novel_circ_004763 | ENSSSCG00000008135 | 3 | + | 47474368 | 47484827 | 1529 | exon_intron |
| novel_circ_004764 | ENSSSCG00000004745 | 1 | + | 1.3E+08 | 1.3E+08 | 343 | annot_exons |
| novel_circ_004765 | ENSSSCG00000030480 | 13 | + | 2.01E+08 | 2.01E+08 | 432 | annot_exons |
| novel_circ_004766 | ENSSSCG00000011803 | 13 | + | 1.25E+08 | 1.25E+08 | 343 | one_exon |
| novel_circ_004767 | ENSSSCG00000021042 | 13 | - | 2.03E+08 | 2.03E+08 | 527 | annot_exons |
| novel_circ_004768 | ENSSSCG00000001097 | 7 | - | 19666315 | 19667273 | 235 | annot_exons |
| novel_circ_004769 | NA | 15 | + | 84246016 | 84246422 | 407 | intergenic |
| novel_circ_004770 | ENSSSCG00000010690 | 14 | + | 1.3E+08 | 1.3E+08 | 1118 | annot_exons |
| novel_circ_004771 | ENSSSCG00000011001 | 10 | + | 33610154 | 33613235 | 264 | annot_exons |
| novel_circ_004772 | ENSSSCG00000005135 | 1 | - | 2.01E+08 | 2.01E+08 | 657 | annot_exons |
| novel_circ_004773 | ENSSSCG00000030149 | 6 | + | 45515047 | 45515523 | 265 | annot_exons |
| novel_circ_004774 | ENSSSCG00000008372 | 3 | - | 78952890 | 78971884 | 751 | annot_exons |
| novel_circ_004775 | ENSSSCG00000033377 | 3 | + | 6981006 | 6988833 | 420 | annot_exons |
| novel_circ_004776 | ENSSSCG00000030395 | 15 | + | 38711933 | 38712146 | 214 | antisense |
| novel_circ_004777 | ENSSSCG00000001974 | 7 | - | 68889311 | 68898544 | 591 | annot_exons |
| novel_circ_004778 | ENSSSCG00000012742 | X | + | 1.22E+08 | 1.22E+08 | 1608 | exon_intron |
| novel_circ_004779 | ENSSSCG00000024784 | 10 | - | 23546471 | 23551591 | 875 | annot_exons |
| novel_circ_004780 | ENSSSCG00000027389 | 18 | + | 38309526 | 38320787 | 278 | annot_exons |
| novel_circ_004781 | ENSSSCG00000015857 | 15 | - | 59179085 | 59185795 | 469 | annot_exons |
| novel_circ_004782 | ENSSSCG00000012055 | 13 | + | 2E+08 | 2E+08 | 401 | annot_exons |
| novel_circ_004783 | ENSSSCG00000009781 | 14 | + | 29764024 | 29769272 | 5249 | exon_intron |
| novel_circ_004784 | ENSSSCG00000005935 | 4 | + | 2966794 | 2982196 | 317 | annot_exons |
| novel_circ_004785 | ENSSSCG00000006870 | 4 | - | 1.18E+08 | 1.18E+08 | 780 | annot_exons |
| novel_circ_004786 | ENSSSCG00000034677 | 15 | - | 84411030 | 84411308 | 279 | intronic |
| novel_circ_004787 | ENSSSCG00000011901 | 13 | + | 1.41E+08 | 1.41E+08 | 444 | exon_intron |
| novel_circ_004788 | ENSSSCG00000026239 | 6 | + | 37957475 | 37961977 | 481 | annot_exons |
| novel_circ_004789 | ENSSSCG00000017419 | 12 | + | 20730842 | 20741653 | 8043 | exon_intron |
| novel_circ_004790 | ENSSSCG00000015940 | 15 | + | 76166995 | 76198564 | 920 | annot_exons |
| novel_circ_004791 | ENSSSCG00000010882 | 10 | + | 19098554 | 19109001 | 439 | annot_exons |
| novel_circ_004792 | ENSSSCG00000006231 | 4 | - | 72636561 | 72643259 | 346 | annot_exons |
| novel_circ_004793 | ENSSSCG00000017311 | 12 | - | 17102077 | 17126300 | 949 | annot_exons |
| novel_circ_004794 | ENSSSCG00000011843 | 13 | - | 1.33E+08 | 1.33E+08 | 315 | annot_exons |
| novel_circ_004795 | ENSSSCG00000005388 | 1 | + | 2.42E+08 | 2.42E+08 | 956 | annot_exons |
| novel_circ_004796 | ENSSSCG00000002660 | 6 | + | 2564305 | 2574166 | 662 | annot_exons |
| novel_circ_004797 | ENSSSCG00000004951 | 1 | - | 1.65E+08 | 1.65E+08 | 462 | annot_exons |
| novel_circ_004798 | ENSSSCG00000013257 | 2 | - | 15860882 | 15870484 | 880 | annot_exons |
| novel_circ_004799 | ENSSSCG00000027074 | 2 | + | 1.14E+08 | 1.14E+08 | 411 | annot_exons |
| novel_circ_004800 | ENSSSCG00000009511 | 11 | - | 67524508 | 67538300 | 13771 | exon_intron |
| novel_circ_004801 | ENSSSCG00000009395 | 11 | - | 18256532 | 18265450 | 609 | annot_exons |
| novel_circ_004802 | ENSSSCG00000024674 | 9 | - | 1.21E+08 | 1.21E+08 | 243 | annot_exons |
| novel_circ_004803 | ENSSSCG00000011117 | 10 | + | 59913525 | 59918554 | 509 | annot_exons |
| novel_circ_004804 | ENSSSCG00000001641 | 7 | + | 37701803 | 37725810 | 6106 | annot_exons |
| novel_circ_004805 | ENSSSCG00000035702 | 15 | - | 66203239 | 66208789 | 321 | annot_exons |
| novel_circ_004806 | ENSSSCG00000008167 | 3 | - | 53073338 | 53077591 | 318 | annot_exons |
| novel_circ_004807 | ENSSSCG00000000492 | 5 | + | 33533423 | 33544765 | 1255 | annot_exons |
| novel_circ_004808 | ENSSSCG00000026110 | 9 | - | 1.05E+08 | 1.05E+08 | 283 | annot_exons |
| novel_circ_004809 | ENSSSCG00000026996 | 11 | - | 64205858 | 64233867 | 1147 | annot_exons |
| novel_circ_004810 | ENSSSCG00000017741 | 12 | + | 42866516 | 42866976 | 461 | one_exon |
| novel_circ_004811 | ENSSSCG00000003712 | 6 | - | 1.09E+08 | 1.09E+08 | 343 | annot_exons |
| novel_circ_004812 | ENSSSCG00000007839 | 3 | - | 23918119 | 23921155 | 398 | annot_exons |
| novel_circ_004813 | ENSSSCG00000001009 | 7 | + | 1857207 | 1864887 | 893 | annot_exons |
| novel_circ_004814 | ENSSSCG00000011474 | 13 | + | 40101738 | 40112945 | 286 | annot_exons |
| novel_circ_004815 | ENSSSCG00000009844 | 14 | - | 33626964 | 33627135 | 172 | one_exon |
| novel_circ_004816 | ENSSSCG00000022903 | 9 | - | 1.03E+08 | 1.03E+08 | 18301 | exon_intron |
| novel_circ_004817 | ENSSSCG00000031593 | 7 | - | 30345147 | 30346563 | 1417 | one_exon |
| novel_circ_004818 | ENSSSCG00000007153 | 17 | - | 32071470 | 32113789 | 2454 | annot_exons |
| novel_circ_004819 | ENSSSCG00000014195 | 2 | + | 1.15E+08 | 1.15E+08 | 403 | annot_exons |
| novel_circ_004820 | ENSSSCG00000006303 | 4 | - | 82951030 | 82990592 | 4819 | annot_exons |
| novel_circ_004821 | ENSSSCG00000037556 | 9 | - | 1.07E+08 | 1.07E+08 | 262 | annot_exons |
| novel_circ_004822 | ENSSSCG00000035442 | 16 | - | 71554919 | 71555215 | 297 | intronic |
| novel_circ_004823 | ENSSSCG00000009281 | 11 | + | 2365747 | 2366615 | 869 | exon_intron |
| novel_circ_004824 | ENSSSCG00000010327 | 14 | - | 80424301 | 80431137 | 487 | annot_exons |
| novel_circ_004825 | ENSSSCG00000029030 | 6 | - | 1.04E+08 | 1.04E+08 | 364 | annot_exons |
| novel_circ_004826 | ENSSSCG00000014364 | 2 | + | 1.42E+08 | 1.42E+08 | 1055 | annot_exons |
| novel_circ_004827 | ENSSSCG00000027389 | 18 | + | 38307155 | 38320787 | 392 | annot_exons |
| novel_circ_004828 | NA | 16 | - | 74621778 | 74708823 | 87046 | intergenic |
| novel_circ_004829 | ENSSSCG00000016961 | 16 | + | 47423320 | 47425696 | 330 | annot_exons |
| novel_circ_004830 | ENSSSCG00000008831 | 8 | + | 39008497 | 39040610 | 590 | annot_exons |
| novel_circ_004831 | ENSSSCG00000000571 | 5 | + | 51657471 | 51668553 | 620 | annot_exons |
| novel_circ_004832 | ENSSSCG00000002875 | 6 | + | 44073938 | 44085673 | 1190 | exon_intron |
| novel_circ_004833 | ENSSSCG00000015555 | 9 | - | 1.24E+08 | 1.24E+08 | 1376 | antisense |
| novel_circ_004834 | ENSSSCG00000004508 | 1 | - | 98860451 | 98885389 | 514 | annot_exons |
| novel_circ_004835 | ENSSSCG00000012328 | X | - | 46390703 | 46401502 | 216 | annot_exons |
| novel_circ_004836 | ENSSSCG00000004425 | 1 | - | 78113146 | 78119226 | 356 | annot_exons |
| novel_circ_004837 | ENSSSCG00000028097 | 6 | + | 1.66E+08 | 1.66E+08 | 267 | annot_exons |
| novel_circ_004838 | ENSSSCG00000015537 | 9 | + | 1.22E+08 | 1.22E+08 | 371 | annot_exons |
| novel_circ_004839 | ENSSSCG00000035852 | 12 | + | 789119 | 789942 | 312 | annot_exons |
| novel_circ_004840 | ENSSSCG00000008591 | 3 | + | 1.15E+08 | 1.15E+08 | 323 | annot_exons |
| novel_circ_004841 | ENSSSCG00000008629 | 3 | + | 1.25E+08 | 1.25E+08 | 321 | annot_exons |
| novel_circ_004842 | ENSSSCG00000017272 | 12 | - | 13527304 | 13534202 | 1409 | annot_exons |
| novel_circ_004843 | ENSSSCG00000035595 | 9 | + | 1.27E+08 | 1.27E+08 | 258 | annot_exons |
| novel_circ_004844 | ENSSSCG00000037530 | 14 | + | 1.32E+08 | 1.32E+08 | 952 | exon_intron |
| novel_circ_004845 | ENSSSCG00000036139 | 4 | - | 34857361 | 34857555 | 195 | antisense |
| novel_circ_004846 | ENSSSCG00000030682 | 6 | + | 82950268 | 82956638 | 393 | annot_exons |
| novel_circ_004847 | ENSSSCG00000015577 | 9 | + | 1.28E+08 | 1.28E+08 | 297 | annot_exons |
| novel_circ_004848 | ENSSSCG00000015872 | 15 | + | 63626162 | 63645378 | 408 | annot_exons |
| novel_circ_004849 | ENSSSCG00000029507 | 8 | + | 1.37E+08 | 1.37E+08 | 444 | annot_exons |
| novel_circ_004850 | ENSSSCG00000008600 | 3 | + | 1.18E+08 | 1.18E+08 | 386 | annot_exons |
| novel_circ_004851 | ENSSSCG00000009010 | 8 | - | 76195576 | 76253449 | 1055 | annot_exons |
| novel_circ_004852 | ENSSSCG00000009957 | 14 | + | 43654545 | 43665205 | 314 | annot_exons |
| novel_circ_004853 | ENSSSCG00000011510 | 13 | - | 50449895 | 50462859 | 1328 | annot_exons |
| novel_circ_004854 | ENSSSCG00000010604 | 14 | - | 1.15E+08 | 1.15E+08 | 170 | annot_exons |
| novel_circ_004855 | ENSSSCG00000001641 | 7 | + | 37732753 | 37739970 | 534 | annot_exons |
| novel_circ_004856 | ENSSSCG00000032531 | 3 | + | 26698722 | 26703165 | 784 | annot_exons |
| novel_circ_004857 | ENSSSCG00000007080 | 17 | - | 25124204 | 25132019 | 652 | annot_exons |
| novel_circ_004858 | ENSSSCG00000009142 | 8 | - | 1.13E+08 | 1.13E+08 | 382 | annot_exons |
| novel_circ_004859 | ENSSSCG00000015509 | 9 | - | 1.18E+08 | 1.18E+08 | 251 | annot_exons |
| novel_circ_004860 | ENSSSCG00000040184 | 11 | + | 48089175 | 48097079 | 1003 | annot_exons |
| novel_circ_004861 | ENSSSCG00000006303 | 4 | - | 83028286 | 83034592 | 279 | annot_exons |
| novel_circ_004862 | ENSSSCG00000030857 | 6 | - | 58865552 | 58866056 | 331 | annot_exons |
| novel_circ_004863 | ENSSSCG00000005021 | 1 | - | 1.8E+08 | 1.8E+08 | 444 | one_exon |
| novel_circ_004864 | ENSSSCG00000024621 | 13 | + | 7071777 | 7073598 | 336 | annot_exons |
| novel_circ_004865 | ENSSSCG00000014831 | 9 | + | 8311788 | 8321548 | 486 | annot_exons |
| novel_circ_004866 | ENSSSCG00000007282 | 17 | - | 37980007 | 38012430 | 665 | annot_exons |
| novel_circ_004867 | ENSSSCG00000011229 | 13 | + | 17630200 | 17633588 | 381 | annot_exons |
| novel_circ_004868 | ENSSSCG00000007307 | 17 | - | 39005450 | 39006264 | 182 | exon_intron |
| novel_circ_004869 | ENSSSCG00000026177 | 9 | + | 92497947 | 92501670 | 288 | annot_exons |
| novel_circ_004870 | ENSSSCG00000028148 | X | - | 28949135 | 28949624 | 490 | intronic |
| novel_circ_004871 | ENSSSCG00000003630 | 6 | + | 92128391 | 92139991 | 10073 | exon_intron |
| novel_circ_004872 | ENSSSCG00000015670 | 15 | + | 7736973 | 7785694 | 686 | annot_exons |
| novel_circ_004873 | ENSSSCG00000000458 | 5 | + | 27216072 | 27229297 | 1560 | annot_exons |
| novel_circ_004874 | ENSSSCG00000013347 | 2 | - | 38993497 | 39012138 | 288 | annot_exons |
| novel_circ_004875 | ENSSSCG00000000633 | 5 | - | 61360834 | 61361253 | 420 | antisense |
| novel_circ_004876 | ENSSSCG00000007574 | 3 | + | 2814416 | 2831893 | 415 | annot_exons |
| novel_circ_004877 | ENSSSCG00000000864 | 5 | - | 82524286 | 82524867 | 290 | annot_exons |
| novel_circ_004878 | ENSSSCG00000016971 | 16 | + | 47957476 | 47989504 | 1071 | annot_exons |
| novel_circ_004879 | ENSSSCG00000014207 | 2 | - | 1.17E+08 | 1.17E+08 | 396 | annot_exons |
| novel_circ_004880 | ENSSSCG00000031701 | 5 | - | 73380414 | 73399218 | 643 | annot_exons |
| novel_circ_004881 | ENSSSCG00000026473 | 12 | + | 11406491 | 11418177 | 717 | annot_exons |
| novel_circ_004882 | ENSSSCG00000003812 | 6 | - | 1.49E+08 | 1.49E+08 | 1432 | exon_intron |
| novel_circ_004883 | ENSSSCG00000015586 | 9 | - | 1.3E+08 | 1.3E+08 | 694 | annot_exons |
| novel_circ_004884 | ENSSSCG00000031169 | 6 | + | 44129896 | 44138702 | 703 | annot_exons |
| novel_circ_004885 | ENSSSCG00000009397 | 11 | + | 18350469 | 18387865 | 865 | annot_exons |
| novel_circ_004886 | ENSSSCG00000016330 | 15 | + | 1.37E+08 | 1.37E+08 | 309 | annot_exons |
| novel_circ_004887 | ENSSSCG00000040912 | 7 | + | 65198472 | 65198799 | 246 | annot_exons |
| novel_circ_004888 | ENSSSCG00000016272 | 15 | + | 1.32E+08 | 1.32E+08 | 327 | annot_exons |
| novel_circ_004889 | ENSSSCG00000016397 | 15 | + | 721936 | 729378 | 930 | annot_exons |
| novel_circ_004890 | ENSSSCG00000010239 | 14 | - | 71537902 | 71544709 | 394 | annot_exons |
| novel_circ_004891 | ENSSSCG00000027675 | 13 | - | 52841867 | 52876892 | 35026 | exon_intron |
| novel_circ_004892 | ENSSSCG00000040286 | 6 | - | 34107266 | 34112759 | 1034 | annot_exons |
| novel_circ_004893 | ENSSSCG00000015560 | 9 | - | 1.25E+08 | 1.25E+08 | 603 | one_exon |
| novel_circ_004894 | ENSSSCG00000016082 | 15 | - | 1.02E+08 | 1.02E+08 | 367 | annot_exons |
| novel_circ_004895 | ENSSSCG00000005480 | 1 | + | 2.54E+08 | 2.54E+08 | 240 | exon_intron |
| novel_circ_004896 | ENSSSCG00000009302 | 11 | - | 4620688 | 4630378 | 589 | annot_exons |
| novel_circ_004897 | ENSSSCG00000004927 | 1 | - | 1.63E+08 | 1.63E+08 | 279 | annot_exons |
| novel_circ_004898 | ENSSSCG00000034280 | 8 | - | 96772487 | 96794459 | 443 | annot_exons |
| novel_circ_004899 | ENSSSCG00000001971 | 7 | + | 68378508 | 68379774 | 242 | annot_exons |
| novel_circ_004900 | ENSSSCG00000000492 | 5 | + | 33544650 | 33548592 | 460 | annot_exons |
| novel_circ_004901 | ENSSSCG00000007478 | 17 | - | 52987380 | 52998000 | 469 | annot_exons |
| novel_circ_004902 | ENSSSCG00000039473 | 12 | + | 37856184 | 37858472 | 240 | annot_exons |
| novel_circ_004903 | ENSSSCG00000036431 | 1 | + | 1.1E+08 | 1.1E+08 | 478 | annot_exons |
| novel_circ_004904 | ENSSSCG00000008018 | 3 | - | 40207742 | 40208661 | 30 | annot_exons |
| novel_circ_004905 | ENSSSCG00000006074 | 4 | + | 38060333 | 38086563 | 325 | annot_exons |
| novel_circ_004906 | ENSSSCG00000008991 | 8 | + | 73940646 | 73942525 | 268 | annot_exons |
| novel_circ_004907 | ENSSSCG00000009551 | 11 | + | 77475172 | 77489240 | 507 | annot_exons |
| novel_circ_004908 | ENSSSCG00000027190 | 5 | - | 70164849 | 70165146 | 298 | antisense |
| novel_circ_004909 | ENSSSCG00000008877 | 8 | + | 47644977 | 47648280 | 432 | annot_exons |
| novel_circ_004910 | ENSSSCG00000006779 | 4 | + | 1.08E+08 | 1.08E+08 | 447 | annot_exons |
| novel_circ_004911 | ENSSSCG00000029578 | 6 | + | 28753900 | 28767831 | 1135 | annot_exons |
| novel_circ_004912 | ENSSSCG00000007786 | 3 | - | 17627422 | 17629123 | 1157 | annot_exons |
| novel_circ_004913 | ENSSSCG00000004718 | 1 | + | 1.29E+08 | 1.29E+08 | 284 | annot_exons |
| novel_circ_004914 | ENSSSCG00000017791 | 12 | - | 45881323 | 45887992 | 169 | annot_exons |
| novel_circ_004915 | ENSSSCG00000004802 | 1 | + | 1.36E+08 | 1.36E+08 | 653 | annot_exons |
| novel_circ_004916 | ENSSSCG00000030685 | 5 | + | 76771226 | 76791074 | 590 | annot_exons |
| novel_circ_004917 | ENSSSCG00000012163 | X | - | 16469577 | 16483070 | 919 | annot_exons |
| novel_circ_004918 | ENSSSCG00000017101 | 16 | - | 74360017 | 74366126 | 290 | annot_exons |
| novel_circ_004919 | ENSSSCG00000021640 | 2 | - | 79449275 | 79449951 | 223 | annot_exons |
| novel_circ_004920 | ENSSSCG00000022536 | 9 | - | 52263331 | 52264888 | 1558 | antisense |
| novel_circ_004921 | ENSSSCG00000047083 | 4 | - | 99170884 | 99171190 | 307 | antisense |
| novel_circ_004922 | ENSSSCG00000031857 | 17 | + | 11556986 | 11560635 | 263 | annot_exons |
| novel_circ_004923 | ENSSSCG00000010738 | 14 | + | 1.34E+08 | 1.34E+08 | 1026 | exon_intron |
| novel_circ_004924 | ENSSSCG00000004196 | 1 | + | 31980772 | 31991763 | 1774 | annot_exons |
| novel_circ_004925 | ENSSSCG00000000816 | 5 | - | 78442065 | 78443399 | 288 | annot_exons |
| novel_circ_004926 | ENSSSCG00000004358 | 1 | - | 67444343 | 67461889 | 1385 | annot_exons |
| novel_circ_004927 | ENSSSCG00000004029 | 1 | - | 4820891 | 4843415 | 1741 | exon_intron |
| novel_circ_004928 | ENSSSCG00000014343 | 2 | + | 1.41E+08 | 1.41E+08 | 420 | annot_exons |
| novel_circ_004929 | ENSSSCG00000028949 | 15 | + | 88595230 | 88614213 | 567 | annot_exons |
| novel_circ_004930 | ENSSSCG00000038254 | 15 | - | 84145377 | 84148920 | 292 | annot_exons |
| novel_circ_004931 | ENSSSCG00000029578 | 6 | + | 28688383 | 28714979 | 24382 | exon_intron |
| novel_circ_004932 | ENSSSCG00000003828 | 6 | - | 1.53E+08 | 1.53E+08 | 402 | annot_exons |
| novel_circ_004933 | ENSSSCG00000013467 | 2 | + | 75886222 | 75887941 | 370 | annot_exons |
| novel_circ_004934 | ENSSSCG00000000753 | 5 | + | 68095263 | 68096836 | 554 | annot_exons |
| novel_circ_004935 | ENSSSCG00000022112 | 11 | - | 67895181 | 67904523 | 474 | annot_exons |
| novel_circ_004936 | ENSSSCG00000011933 | 13 | - | 1.48E+08 | 1.49E+08 | 5090 | annot_exons |
| novel_circ_004937 | ENSSSCG00000026382 | 1 | + | 1.95E+08 | 1.95E+08 | 392 | annot_exons |
| novel_circ_004938 | ENSSSCG00000016695 | 18 | + | 44996263 | 45015134 | 756 | annot_exons |
| novel_circ_004939 | ENSSSCG00000011332 | 13 | - | 29901475 | 29916530 | 802 | annot_exons |
| novel_circ_004940 | ENSSSCG00000006277 | 4 | - | 80177541 | 80183572 | 397 | annot_exons |
| novel_circ_004941 | ENSSSCG00000001862 | 7 | - | 56524083 | 56526919 | 312 | annot_exons |
| novel_circ_004942 | ENSSSCG00000049630 | 4 | - | 19018199 | 19060921 | 676 | annot_exons |
| novel_circ_004943 | ENSSSCG00000016306 | 15 | - | 1.34E+08 | 1.34E+08 | 516 | annot_exons |
| novel_circ_004944 | ENSSSCG00000004415 | 1 | + | 75429568 | 75429695 | 128 | one_exon |
| novel_circ_004945 | ENSSSCG00000026536 | 11 | - | 78703337 | 78703726 | 390 | one_exon |
| novel_circ_004946 | ENSSSCG00000014149 | 2 | - | 96155707 | 96165180 | 469 | annot_exons |
| novel_circ_004947 | ENSSSCG00000011332 | 13 | - | 29898476 | 29901609 | 275 | annot_exons |
| novel_circ_004948 | ENSSSCG00000003812 | 6 | - | 1.49E+08 | 1.49E+08 | 249 | exon_intron |
| novel_circ_004949 | ENSSSCG00000037977 | 3 | - | 69308675 | 69317475 | 539 | annot_exons |
| novel_circ_004950 | ENSSSCG00000029593 | 3 | - | 92287276 | 92299786 | 676 | annot_exons |
| novel_circ_004951 | ENSSSCG00000007585 | 3 | - | 4087598 | 4088597 | 772 | annot_exons |
| novel_circ_004952 | ENSSSCG00000038873 | 13 | + | 39701921 | 39710141 | 686 | annot_exons |
| novel_circ_004953 | ENSSSCG00000027857 | 2 | + | 1.23E+08 | 1.23E+08 | 436 | annot_exons |
| novel_circ_004954 | ENSSSCG00000008150 | 3 | - | 51469322 | 51475834 | 6513 | intronic |
| novel_circ_004955 | ENSSSCG00000007896 | 3 | + | 31432565 | 31432718 | 154 | intronic |
| novel_circ_004956 | ENSSSCG00000009134 | 8 | - | 1.12E+08 | 1.12E+08 | 813 | annot_exons |
| novel_circ_004957 | ENSSSCG00000005282 | 1 | + | 2.3E+08 | 2.3E+08 | 363 | annot_exons |
| novel_circ_004958 | ENSSSCG00000011236 | 13 | - | 19445235 | 19483077 | 918 | annot_exons |
| novel_circ_004959 | ENSSSCG00000015987 | 15 | - | 82974214 | 82977955 | 1744 | annot_exons |
| novel_circ_004960 | ENSSSCG00000013940 | 2 | - | 56892733 | 56897960 | 4476 | annot_exons |
| novel_circ_004961 | ENSSSCG00000005466 | 1 | - | 2.53E+08 | 2.53E+08 | 3831 | annot_exons |
| novel_circ_004962 | ENSSSCG00000023533 | 1 | - | 1.48E+08 | 1.48E+08 | 403 | annot_exons |
| novel_circ_004963 | ENSSSCG00000003863 | 6 | - | 1.6E+08 | 1.6E+08 | 930 | exon_intron |
| novel_circ_004964 | ENSSSCG00000010289 | 14 | - | 75379211 | 75402610 | 503 | annot_exons |
| novel_circ_004965 | ENSSSCG00000036431 | 1 | + | 1.1E+08 | 1.1E+08 | 308 | annot_exons |
| novel_circ_004966 | ENSSSCG00000012642 | X | + | 1.02E+08 | 1.02E+08 | 251 | annot_exons |
| novel_circ_004967 | ENSSSCG00000006089 | 4 | - | 40338864 | 40345193 | 284 | annot_exons |
| novel_circ_004968 | ENSSSCG00000014917 | 9 | + | 20252049 | 20253739 | 237 | annot_exons |
| novel_circ_004969 | NA | 6 | + | 47386174 | 47387064 | 891 | intergenic |
| novel_circ_004970 | ENSSSCG00000010190 | 14 | - | 60415135 | 60415589 | 455 | antisense |
| novel_circ_004971 | ENSSSCG00000003460 | 6 | - | 74710923 | 74711823 | 285 | annot_exons |
| novel_circ_004972 | ENSSSCG00000002381 | 7 | - | 98311934 | 98318944 | 543 | annot_exons |
| novel_circ_004973 | ENSSSCG00000006872 | 4 | - | 1.18E+08 | 1.18E+08 | 188 | one_exon |
| novel_circ_004974 | ENSSSCG00000003199 | 6 | + | 54801799 | 54802488 | 406 | annot_exons |
| novel_circ_004975 | ENSSSCG00000037670 | X | - | 90288670 | 90288909 | 240 | antisense |
| novel_circ_004976 | ENSSSCG00000004814 | 1 | + | 1.38E+08 | 1.38E+08 | 336 | annot_exons |
| novel_circ_004977 | ENSSSCG00000017504 | 12 | - | 22811222 | 22813863 | 488 | annot_exons |
| novel_circ_004978 | ENSSSCG00000005436 | 1 | + | 2.48E+08 | 2.48E+08 | 1198 | annot_exons |
| novel_circ_004979 | ENSSSCG00000011652 | 13 | - | 77417167 | 77463301 | 788 | annot_exons |
| novel_circ_004980 | ENSSSCG00000015565 | 9 | + | 1.26E+08 | 1.26E+08 | 286 | annot_exons |
| novel_circ_004981 | ENSSSCG00000000795 | 5 | - | 74586270 | 74614932 | 1476 | annot_exons |
| novel_circ_004982 | ENSSSCG00000030165 | 5 | - | 9694704 | 9694966 | 263 | intronic |
| novel_circ_004983 | ENSSSCG00000020878 | 3 | + | 1.02E+08 | 1.02E+08 | 618 | annot_exons |
| novel_circ_004984 | ENSSSCG00000008540 | 3 | - | 1.1E+08 | 1.1E+08 | 461 | one_exon |
| novel_circ_004985 | ENSSSCG00000007917 | 3 | - | 34966209 | 35008697 | 334 | annot_exons |
| novel_circ_004986 | ENSSSCG00000008761 | 8 | + | 20285480 | 20308094 | 519 | annot_exons |
| novel_circ_004987 | ENSSSCG00000020743 | 1 | + | 2.64E+08 | 2.64E+08 | 245 | annot_exons |
| novel_circ_004988 | NA | 15 | - | 84419839 | 84420120 | 282 | intergenic |
| novel_circ_004989 | ENSSSCG00000010172 | 14 | + | 59156916 | 59163188 | 235 | annot_exons |
| novel_circ_004990 | ENSSSCG00000023727 | 12 | - | 35290360 | 35291326 | 319 | annot_exons |
| novel_circ_004991 | ENSSSCG00000015283 | 9 | - | 65111378 | 65114982 | 500 | annot_exons |
| novel_circ_004992 | ENSSSCG00000049630 | 4 | - | 19094805 | 19096580 | 303 | annot_exons |
| novel_circ_004993 | ENSSSCG00000016981 | 16 | - | 50458293 | 50483222 | 4627 | exon_intron |
| novel_circ_004994 | ENSSSCG00000035038 | 1 | - | 1.15E+08 | 1.15E+08 | 349 | annot_exons |
| novel_circ_004995 | ENSSSCG00000016843 | 16 | - | 22444229 | 22446274 | 1136 | exon_intron |
| novel_circ_004996 | ENSSSCG00000000843 | 5 | - | 80183834 | 80187058 | 204 | annot_exons |
| novel_circ_004997 | ENSSSCG00000034768 | 3 | + | 4974527 | 4985274 | 334 | annot_exons |
| novel_circ_004998 | ENSSSCG00000003772 | 6 | + | 1.36E+08 | 1.36E+08 | 1857 | exon_intron |
| novel_circ_004999 | ENSSSCG00000011036 | 10 | + | 43967033 | 43994747 | 688 | annot_exons |
| novel_circ_005000 | ENSSSCG00000015743 | 15 | - | 31324933 | 31336505 | 753 | annot_exons |
| novel_circ_005001 | ENSSSCG00000039896 | 1 | + | 2.47E+08 | 2.47E+08 | 548 | annot_exons |
| novel_circ_005002 | ENSSSCG00000029539 | 7 | + | 28649149 | 28653086 | 3327 | exon_intron |
| novel_circ_005003 | ENSSSCG00000003630 | 6 | + | 92115566 | 92130264 | 6888 | exon_intron |
| novel_circ_005004 | ENSSSCG00000030108 | 17 | - | 50931699 | 50933570 | 257 | annot_exons |
| novel_circ_005005 | ENSSSCG00000010190 | 14 | + | 60414528 | 60415663 | 1042 | annot_exons |
| novel_circ_005006 | ENSSSCG00000004196 | 1 | + | 31970093 | 31982095 | 964 | annot_exons |
| novel_circ_005007 | NA | 15 | - | 84269667 | 84269840 | 174 | intergenic |
| novel_circ_005008 | ENSSSCG00000016912 | 16 | - | 34613687 | 34666354 | 646 | annot_exons |
| novel_circ_005009 | ENSSSCG00000027608 | 2 | - | 1.01E+08 | 1.01E+08 | 480 | annot_exons |
| novel_circ_005010 | ENSSSCG00000011772 | 13 | + | 1.21E+08 | 1.21E+08 | 718 | annot_exons |
| novel_circ_005011 | ENSSSCG00000023639 | 7 | + | 23225339 | 23225510 | 172 | antisense |
| novel_circ_005012 | ENSSSCG00000005388 | 1 | + | 2.42E+08 | 2.42E+08 | 463 | annot_exons |
| novel_circ_005013 | ENSSSCG00000013645 | 2 | + | 69322286 | 69324457 | 596 | annot_exons |
| novel_circ_005014 | ENSSSCG00000021814 | 11 | + | 675689 | 700356 | 1789 | annot_exons |
| novel_circ_005015 | ENSSSCG00000013022 | 2 | + | 7411080 | 7412033 | 438 | annot_exons |
| novel_circ_005016 | ENSSSCG00000008512 | 3 | - | 1.07E+08 | 1.07E+08 | 299 | annot_exons |
| novel_circ_005017 | ENSSSCG00000039473 | 12 | + | 37927186 | 37928284 | 208 | annot_exons |
| novel_circ_005018 | ENSSSCG00000000492 | 5 | + | 33539476 | 33548592 | 1285 | annot_exons |
| novel_circ_005019 | ENSSSCG00000020878 | 3 | + | 1.02E+08 | 1.02E+08 | 1030 | annot_exons |
| novel_circ_005020 | ENSSSCG00000041461 | AEMK02000489.1 | - | 40306 | 45181 | 4340 | exon_intron |
| novel_circ_005021 | ENSSSCG00000010799 | 3 | + | 22805323 | 22810630 | 199 | annot_exons |
| novel_circ_005022 | ENSSSCG00000008624 | 3 | - | 1.25E+08 | 1.25E+08 | 735 | annot_exons |
| novel_circ_005023 | ENSSSCG00000002505 | 7 | + | 1.18E+08 | 1.18E+08 | 265 | annot_exons |
| novel_circ_005024 | ENSSSCG00000021744 | 4 | + | 16145129 | 16150040 | 579 | annot_exons |
| novel_circ_005025 | ENSSSCG00000015695 | 15 | - | 16792894 | 16796655 | 337 | annot_exons |
| novel_circ_005026 | ENSSSCG00000002426 | 7 | + | 1.1E+08 | 1.1E+08 | 7140 | exon_intron |
| novel_circ_005027 | ENSSSCG00000011672 | 13 | + | 82256891 | 82261474 | 222 | annot_exons |
| novel_circ_005028 | ENSSSCG00000027417 | 6 | - | 96514931 | 96526660 | 11730 | exon_intron |
| novel_circ_005029 | ENSSSCG00000015883 | 15 | + | 66695651 | 66696058 | 408 | exon_intron |
| novel_circ_005030 | ENSSSCG00000012463 | X | - | 68998325 | 69001290 | 198 | annot_exons |
| novel_circ_005031 | ENSSSCG00000039285 | 13 | + | 1.17E+08 | 1.17E+08 | 715 | annot_exons |
| novel_circ_005032 | ENSSSCG00000001064 | 7 | + | 12261370 | 12280446 | 567 | annot_exons |
| novel_circ_005033 | ENSSSCG00000009256 | 8 | + | 1.38E+08 | 1.38E+08 | 340 | annot_exons |
| novel_circ_005034 | ENSSSCG00000003460 | 6 | - | 74710923 | 74711828 | 290 | annot_exons |
| novel_circ_005035 | ENSSSCG00000008513 | 3 | - | 1.07E+08 | 1.07E+08 | 474 | annot_exons |
| novel_circ_005036 | ENSSSCG00000004103 | 1 | - | 16218321 | 16224015 | 312 | annot_exons |
| novel_circ_005037 | ENSSSCG00000008337 | 3 | - | 72961924 | 72962360 | 437 | antisense |
| novel_circ_005038 | ENSSSCG00000011467 | 13 | - | 39468507 | 39468774 | 164 | annot_exons |
| novel_circ_005039 | ENSSSCG00000005110 | 1 | - | 1.94E+08 | 1.94E+08 | 364 | annot_exons |
| novel_circ_005040 | ENSSSCG00000032556 | 3 | - | 18257011 | 18258701 | 1691 | antisense |
| novel_circ_005041 | ENSSSCG00000024761 | 9 | - | 87020706 | 87021020 | 224 | annot_exons |
| novel_circ_005042 | ENSSSCG00000009516 | 11 | + | 68255109 | 68279709 | 418 | annot_exons |
| novel_circ_005043 | ENSSSCG00000009029 | 8 | - | 80800577 | 80809470 | 147 | annot_exons |
| novel_circ_005044 | ENSSSCG00000010952 | 10 | + | 29028959 | 29035802 | 501 | annot_exons |
| novel_circ_005045 | ENSSSCG00000017732 | 12 | - | 42589975 | 42596350 | 692 | annot_exons |
| novel_circ_005046 | ENSSSCG00000001487 | 7 | + | 27102674 | 27103050 | 377 | one_exon |
| novel_circ_005047 | ENSSSCG00000033753 | 3 | - | 1.15E+08 | 1.15E+08 | 246 | annot_exons |
| novel_circ_005048 | ENSSSCG00000038977 | 9 | - | 1.02E+08 | 1.02E+08 | 311 | annot_exons |
| novel_circ_005049 | ENSSSCG00000023738 | 11 | - | 21807483 | 21808357 | 210 | annot_exons |
| novel_circ_005050 | NA | 7 | - | 78055271 | 78056399 | 1129 | intergenic |
| novel_circ_005051 | ENSSSCG00000031903 | 2 | + | 1304189 | 1305604 | 1248 | annot_exons |
| novel_circ_005052 | ENSSSCG00000007289 | 17 | - | 38417789 | 38434388 | 697 | annot_exons |
| novel_circ_005053 | ENSSSCG00000006779 | 4 | + | 1.08E+08 | 1.08E+08 | 215 | annot_exons |
| novel_circ_005054 | ENSSSCG00000010360 | 14 | + | 87860941 | 87862622 | 266 | annot_exons |
| novel_circ_005055 | ENSSSCG00000036885 | 13 | - | 1.68E+08 | 1.68E+08 | 228 | exon_intron |
| novel_circ_005056 | ENSSSCG00000014913 | 9 | - | 19883798 | 19913189 | 677 | annot_exons |
| novel_circ_005057 | ENSSSCG00000039061 | 1 | - | 1.31E+08 | 1.31E+08 | 599 | annot_exons |
| novel_circ_005058 | ENSSSCG00000000753 | 5 | + | 68072367 | 68075752 | 379 | annot_exons |
| novel_circ_005059 | ENSSSCG00000038646 | 10 | - | 13491755 | 13527498 | 498 | annot_exons |
| novel_circ_005060 | NA | 14 | - | 81431285 | 81431439 | 155 | intergenic |
| novel_circ_005061 | ENSSSCG00000009401 | 11 | - | 19253377 | 19264448 | 531 | annot_exons |
| novel_circ_005062 | ENSSSCG00000016409 | 18 | - | 1565896 | 1599809 | 1276 | annot_exons |
| novel_circ_005063 | ENSSSCG00000005201 | 1 | - | 2.16E+08 | 2.16E+08 | 232 | annot_exons |
| novel_circ_005064 | ENSSSCG00000008080 | 3 | + | 43040518 | 43051814 | 4056 | exon_intron |
| novel_circ_005065 | ENSSSCG00000016618 | 18 | - | 25804045 | 25807266 | 440 | annot_exons |
| novel_circ_005066 | ENSSSCG00000029464 | 5 | - | 67690123 | 67712468 | 1584 | annot_exons |
| novel_circ_005067 | ENSSSCG00000002266 | 7 | - | 85996423 | 86001623 | 493 | annot_exons |
| novel_circ_005068 | ENSSSCG00000038973 | 5 | + | 1.02E+08 | 1.02E+08 | 286 | annot_exons |
| novel_circ_005069 | ENSSSCG00000001732 | 7 | - | 43609181 | 43618755 | 1175 | annot_exons |
| novel_circ_005070 | ENSSSCG00000004901 | 1 | + | 1.59E+08 | 1.59E+08 | 498 | annot_exons |
| novel_circ_005071 | ENSSSCG00000023784 | 9 | + | 99345879 | 99357301 | 344 | annot_exons |
| novel_circ_005072 | ENSSSCG00000044456 | 14 | - | 60924849 | 60925564 | 223 | annot_exons |
| novel_circ_005073 | ENSSSCG00000044396 | 10 | + | 14479215 | 14479432 | 218 | one_exon |
| novel_circ_005074 | ENSSSCG00000015097 | 9 | - | 46041865 | 46043475 | 218 | annot_exons |
| novel_circ_005075 | ENSSSCG00000003488 | 6 | - | 77604094 | 77605337 | 398 | annot_exons |
| novel_circ_005076 | ENSSSCG00000004927 | 1 | - | 1.63E+08 | 1.63E+08 | 342 | annot_exons |
| novel_circ_005077 | ENSSSCG00000030480 | 13 | + | 2.01E+08 | 2.01E+08 | 3083 | exon_intron |
| novel_circ_005078 | ENSSSCG00000007280 | 17 | + | 37837321 | 37854328 | 657 | annot_exons |
| novel_circ_005079 | ENSSSCG00000011394 | 13 | + | 32466924 | 32478114 | 4233 | annot_exons |
| novel_circ_005080 | ENSSSCG00000033682 | 2 | + | 81811037 | 81815641 | 4605 | antisense |
| novel_circ_005081 | ENSSSCG00000038870 | AEMK02000510.1 | - | 600438 | 605665 | 688 | annot_exons |
| novel_circ_005082 | ENSSSCG00000035533 | 3 | + | 56779478 | 56780136 | 574 | annot_exons |
| novel_circ_005083 | ENSSSCG00000004592 | 1 | + | 1.13E+08 | 1.13E+08 | 460 | annot_exons |
| novel_circ_005084 | ENSSSCG00000039793 | 6 | - | 1.5E+08 | 1.5E+08 | 289 | annot_exons |
| novel_circ_005085 | ENSSSCG00000000770 | 5 | - | 70036296 | 70036890 | 595 | intronic |
| novel_circ_005086 | ENSSSCG00000001097 | 7 | - | 19626374 | 19636149 | 626 | annot_exons |
| novel_circ_005087 | ENSSSCG00000009345 | 11 | + | 9132180 | 9149581 | 654 | annot_exons |
| novel_circ_005088 | NA | 15 | - | 84445381 | 84452028 | 6648 | intergenic |
| novel_circ_005089 | ENSSSCG00000004979 | 1 | - | 1.7E+08 | 1.7E+08 | 567 | annot_exons |
| novel_circ_005090 | ENSSSCG00000031831 | 9 | - | 42795737 | 42797896 | 2160 | intronic |
| novel_circ_005091 | ENSSSCG00000003218 | 6 | + | 55295717 | 55299982 | 1032 | annot_exons |
| novel_circ_005092 | ENSSSCG00000040984 | 7 | - | 32541481 | 32544575 | 543 | annot_exons |
| novel_circ_005093 | ENSSSCG00000011594 | 13 | - | 69469788 | 69490526 | 3236 | annot_exons |
| novel_circ_005094 | ENSSSCG00000028724 | 13 | + | 1.92E+08 | 1.92E+08 | 178 | annot_exons |
| novel_circ_005095 | ENSSSCG00000015492 | 9 | + | 1.16E+08 | 1.16E+08 | 489 | annot_exons |
| novel_circ_005096 | ENSSSCG00000028097 | 6 | + | 1.66E+08 | 1.66E+08 | 1510 | annot_exons |
| novel_circ_005097 | ENSSSCG00000007080 | 17 | - | 25114368 | 25122516 | 242 | annot_exons |
| novel_circ_005098 | ENSSSCG00000037647 | 13 | - | 82802690 | 82893436 | 697 | annot_exons |
| novel_circ_005099 | ENSSSCG00000008227 | 3 | + | 58905881 | 58913632 | 690 | annot_exons |
| novel_circ_005100 | ENSSSCG00000026506 | 17 | - | 28557861 | 28559563 | 449 | annot_exons |
| novel_circ_005101 | ENSSSCG00000015611 | 9 | - | 1.33E+08 | 1.33E+08 | 260 | annot_exons |
| novel_circ_005102 | ENSSSCG00000013366 | 2 | + | 40818120 | 40818189 | 70 | antisense |
| novel_circ_005103 | NA | 15 | - | 84354745 | 84356302 | 1558 | intergenic |
| novel_circ_005104 | ENSSSCG00000003976 | 6 | - | 1.7E+08 | 1.7E+08 | 395 | annot_exons |
| novel_circ_005105 | ENSSSCG00000016019 | 15 | - | 88475213 | 88476704 | 240 | annot_exons |
| novel_circ_005106 | ENSSSCG00000021285 | 3 | - | 4238841 | 4279580 | 16098 | exon_intron |
| novel_circ_005107 | ENSSSCG00000038178 | AEMK02000697.1 | - | 40353 | 62329 | 3798 | annot_exons |
| novel_circ_005108 | ENSSSCG00000040286 | 6 | - | 34107266 | 34112786 | 1061 | annot_exons |
| novel_circ_005109 | ENSSSCG00000000840 | 5 | - | 79468294 | 79470833 | 376 | annot_exons |
| novel_circ_005110 | ENSSSCG00000033213 | 2 | + | 1.34E+08 | 1.34E+08 | 325 | one_exon |
| novel_circ_005111 | ENSSSCG00000033010 | 2 | + | 9207332 | 9207648 | 317 | intronic |
| novel_circ_005112 | ENSSSCG00000006508 | 4 | + | 94446190 | 94463950 | 909 | annot_exons |
| novel_circ_005113 | ENSSSCG00000009134 | 8 | - | 1.12E+08 | 1.12E+08 | 1185 | annot_exons |
| novel_circ_005114 | ENSSSCG00000006090 | 4 | + | 40393080 | 40395305 | 382 | annot_exons |
| novel_circ_005115 | ENSSSCG00000012035 | 13 | - | 1.96E+08 | 1.96E+08 | 252 | annot_exons |
| novel_circ_005116 | ENSSSCG00000016863 | 16 | - | 26541231 | 26589063 | 606 | annot_exons |
| novel_circ_005117 | ENSSSCG00000016954 | 16 | + | 44484459 | 44510642 | 1013 | annot_exons |
| novel_circ_005118 | ENSSSCG00000015712 | 15 | + | 23441826 | 23442179 | 354 | one_exon |
| novel_circ_005119 | ENSSSCG00000016618 | 18 | - | 25804045 | 25844414 | 958 | annot_exons |
| novel_circ_005120 | ENSSSCG00000044793 | 12 | - | 1049914 | 1050649 | 736 | exon_intron |
| novel_circ_005121 | ENSSSCG00000002799 | 6 | - | 20301313 | 20329473 | 2564 | annot_exons |
| novel_circ_005122 | ENSSSCG00000015286 | 9 | + | 65834182 | 65834542 | 361 | antisense |
| novel_circ_005123 | ENSSSCG00000021042 | 13 | - | 2.03E+08 | 2.03E+08 | 383 | annot_exons |
| novel_circ_005124 | ENSSSCG00000017311 | 12 | - | 17140057 | 17150743 | 381 | annot_exons |
| novel_circ_005125 | ENSSSCG00000023738 | 11 | - | 21765631 | 21766882 | 227 | annot_exons |
| novel_circ_005126 | ENSSSCG00000017672 | 12 | + | 36366596 | 36367060 | 274 | annot_exons |
| novel_circ_005127 | ENSSSCG00000007757 | 3 | + | 17285631 | 17287078 | 1448 | antisense |
| novel_circ_005128 | ENSSSCG00000003438 | 6 | + | 72282918 | 72283966 | 207 | annot_exons |
| novel_circ_005129 | ENSSSCG00000006642 | 4 | + | 98223601 | 98224793 | 869 | annot_exons |
| novel_circ_005130 | ENSSSCG00000004082 | 1 | + | 14143615 | 14167020 | 4399 | annot_exons |
| novel_circ_005131 | ENSSSCG00000022952 | 13 | + | 1.01E+08 | 1.01E+08 | 533 | annot_exons |
| novel_circ_005132 | ENSSSCG00000002337 | 7 | - | 96347125 | 96353198 | 720 | annot_exons |
| novel_circ_005133 | ENSSSCG00000014113 | 2 | - | 88196738 | 88206327 | 382 | annot_exons |
| novel_circ_005134 | ENSSSCG00000035676 | 16 | - | 71257300 | 71266608 | 756 | annot_exons |
| novel_circ_005135 | ENSSSCG00000030049 | 6 | + | 49286824 | 49289394 | 231 | exon_intron |
| novel_circ_005136 | ENSSSCG00000002517 | 7 | + | 1.21E+08 | 1.21E+08 | 279 | annot_exons |
| novel_circ_005137 | ENSSSCG00000014868 | 9 | + | 10684937 | 10690581 | 510 | annot_exons |
| novel_circ_005138 | ENSSSCG00000016613 | 18 | + | 24985702 | 25000371 | 1163 | annot_exons |
| novel_circ_005139 | ENSSSCG00000004570 | 1 | - | 1.09E+08 | 1.09E+08 | 203 | one_exon |
| novel_circ_005140 | ENSSSCG00000005465 | 1 | - | 2.53E+08 | 2.53E+08 | 303 | annot_exons |
| novel_circ_005141 | ENSSSCG00000009029 | 8 | - | 80787216 | 80809470 | 413 | annot_exons |
| novel_circ_005142 | ENSSSCG00000015480 | 9 | + | 1.14E+08 | 1.14E+08 | 1305 | annot_exons |
| novel_circ_005143 | ENSSSCG00000014113 | 2 | - | 88184110 | 88206327 | 522 | annot_exons |
| novel_circ_005144 | ENSSSCG00000007153 | 17 | - | 32135396 | 32159495 | 1838 | annot_exons |
| novel_circ_005145 | ENSSSCG00000027457 | 7 | + | 56097518 | 56122384 | 730 | annot_exons |
| novel_circ_005146 | ENSSSCG00000014199 | 2 | + | 1.16E+08 | 1.16E+08 | 822 | annot_exons |
| novel_circ_005147 | ENSSSCG00000015411 | 9 | - | 1.03E+08 | 1.03E+08 | 275 | annot_exons |
| novel_circ_005148 | NA | 15 | + | 84277839 | 84308405 | 30567 | intergenic |
| novel_circ_005149 | ENSSSCG00000028047 | 6 | + | 1.19E+08 | 1.19E+08 | 261 | annot_exons |
| novel_circ_005150 | NA | 15 | + | 13637175 | 13639951 | 2777 | intergenic |
| novel_circ_005151 | ENSSSCG00000004307 | 1 | - | 56049753 | 56052896 | 294 | annot_exons |
| novel_circ_005152 | ENSSSCG00000025602 | 18 | + | 33630566 | 33638712 | 237 | annot_exons |
| novel_circ_005153 | ENSSSCG00000034191 | 2 | + | 42861755 | 42947501 | 476 | annot_exons |
| novel_circ_005154 | ENSSSCG00000011395 | 13 | + | 32610663 | 32610968 | 228 | annot_exons |
| novel_circ_005155 | ENSSSCG00000008363 | 3 | - | 77375906 | 77378404 | 336 | annot_exons |
| novel_circ_005156 | ENSSSCG00000035027 | 1 | - | 9492460 | 9492750 | 291 | intronic |
| novel_circ_005157 | ENSSSCG00000009243 | 8 | - | 1.35E+08 | 1.35E+08 | 500 | annot_exons |
| novel_circ_005158 | ENSSSCG00000013314 | 2 | - | 27989714 | 28020514 | 4066 | annot_exons |
| novel_circ_005159 | ENSSSCG00000015818 | 15 | - | 48271060 | 48277942 | 1599 | annot_exons |
| novel_circ_005160 | ENSSSCG00000015857 | 15 | - | 59181592 | 59185795 | 199 | annot_exons |
| novel_circ_005161 | ENSSSCG00000006841 | 4 | + | 1.11E+08 | 1.11E+08 | 1050 | annot_exons |
| novel_circ_005162 | ENSSSCG00000032556 | 3 | - | 18256677 | 18259732 | 3056 | antisense |
| novel_circ_005163 | ENSSSCG00000027722 | 8 | - | 1.39E+08 | 1.39E+08 | 345 | annot_exons |
| novel_circ_005164 | ENSSSCG00000017207 | 12 | - | 6073486 | 6074110 | 254 | annot_exons |
| novel_circ_005165 | ENSSSCG00000009222 | 8 | + | 1.31E+08 | 1.31E+08 | 407 | annot_exons |
| novel_circ_005166 | ENSSSCG00000025902 | 8 | + | 19331232 | 19335172 | 192 | annot_exons |
| novel_circ_005167 | ENSSSCG00000017678 | 12 | + | 37649097 | 37657745 | 293 | annot_exons |
| novel_circ_005168 | ENSSSCG00000016605 | 18 | + | 23804022 | 23804366 | 345 | antisense |
| novel_circ_005169 | ENSSSCG00000007074 | 17 | - | 22333520 | 22347295 | 7454 | exon_intron |
| novel_circ_005170 | ENSSSCG00000001852 | 7 | - | 55659526 | 55661779 | 471 | annot_exons |
| novel_circ_005171 | ENSSSCG00000027765 | 1 | + | 1.75E+08 | 1.75E+08 | 891 | annot_exons |
| novel_circ_005172 | ENSSSCG00000003591 | 6 | - | 87568298 | 87570047 | 398 | annot_exons |
| novel_circ_005173 | ENSSSCG00000031791 | 13 | - | 39412846 | 39414598 | 198 | annot_exons |
| novel_circ_005174 | ENSSSCG00000007805 | 3 | + | 18555760 | 18559335 | 256 | annot_exons |
| novel_circ_005175 | ENSSSCG00000034386 | 14 | + | 31715996 | 31736163 | 1634 | annot_exons |
| novel_circ_005176 | ENSSSCG00000023593 | 8 | - | 1.32E+08 | 1.32E+08 | 503 | annot_exons |
| novel_circ_005177 | ENSSSCG00000011340 | 13 | - | 30538624 | 30547685 | 233 | annot_exons |
| novel_circ_005178 | ENSSSCG00000034677 | 15 | - | 84408236 | 84410512 | 858 | annot_exons |
| novel_circ_005179 | ENSSSCG00000040397 | 6 | + | 1.06E+08 | 1.06E+08 | 1269 | annot_exons |
| novel_circ_005180 | ENSSSCG00000004263 | 1 | + | 44992332 | 45003147 | 10641 | exon_intron |
| novel_circ_005181 | ENSSSCG00000038617 | 17 | + | 63033432 | 63050763 | 4534 | exon_intron |
| novel_circ_005182 | ENSSSCG00000022636 | 5 | + | 42796938 | 42843975 | 3716 | annot_exons |
| novel_circ_005183 | ENSSSCG00000010211 | 14 | - | 63190562 | 63237347 | 802 | annot_exons |
| novel_circ_005184 | ENSSSCG00000012171 | X | + | 19807991 | 19809733 | 240 | annot_exons |
| novel_circ_005185 | ENSSSCG00000015283 | 9 | - | 65082590 | 65085907 | 445 | annot_exons |
| novel_circ_005186 | ENSSSCG00000001499 | 7 | + | 29053170 | 29058245 | 360 | annot_exons |
| novel_circ_005187 | ENSSSCG00000008169 | 3 | + | 53270127 | 53270926 | 307 | annot_exons |
| novel_circ_005188 | ENSSSCG00000016430 | 18 | - | 5306642 | 5308460 | 271 | annot_exons |
| novel_circ_005189 | ENSSSCG00000033763 | 1 | - | 2.31E+08 | 2.31E+08 | 185 | one_exon |
| novel_circ_005190 | ENSSSCG00000007721 | 3 | + | 11771884 | 11782001 | 492 | annot_exons |
| novel_circ_005191 | ENSSSCG00000027556 | 13 | + | 97912254 | 97913066 | 322 | annot_exons |
| novel_circ_005192 | ENSSSCG00000026597 | 5 | - | 77436970 | 77447245 | 4760 | annot_exons |
| novel_circ_005193 | ENSSSCG00000016850 | 16 | - | 23735576 | 23745763 | 3280 | annot_exons |
| novel_circ_005194 | ENSSSCG00000011112 | 10 | - | 59745682 | 59752288 | 294 | annot_exons |
| novel_circ_005195 | ENSSSCG00000031775 | 5 | - | 73473989 | 73474314 | 233 | exon_intron |
| novel_circ_005196 | ENSSSCG00000017518 | 12 | - | 24065298 | 24065813 | 368 | annot_exons |
| novel_circ_005197 | ENSSSCG00000014364 | 2 | + | 1.42E+08 | 1.42E+08 | 593 | annot_exons |
| novel_circ_005198 | ENSSSCG00000035318 | 11 | + | 3245493 | 3256021 | 4796 | annot_exons |
| novel_circ_005199 | ENSSSCG00000017748 | 12 | - | 43684482 | 43691261 | 419 | annot_exons |
| novel_circ_005200 | ENSSSCG00000011833 | 13 | + | 1.33E+08 | 1.33E+08 | 326 | annot_exons |
| novel_circ_005201 | ENSSSCG00000001930 | 7 | + | 61022675 | 61025520 | 506 | annot_exons |
| novel_circ_005202 | ENSSSCG00000007839 | 3 | - | 23931447 | 23956449 | 411 | exon_intron |
| novel_circ_005203 | ENSSSCG00000015311 | 9 | - | 72070655 | 72076914 | 403 | annot_exons |
| novel_circ_005204 | ENSSSCG00000000859 | 5 | - | 82055444 | 82056857 | 326 | annot_exons |
| novel_circ_005205 | ENSSSCG00000004717 | 1 | + | 1.28E+08 | 1.28E+08 | 378 | annot_exons |
| novel_circ_005206 | ENSSSCG00000007765 | 3 | + | 17395282 | 17395526 | 245 | one_exon |
| novel_circ_005207 | ENSSSCG00000001499 | 7 | - | 29299204 | 29299747 | 544 | antisense |
| novel_circ_005208 | ENSSSCG00000002869 | 6 | + | 43879422 | 43887734 | 366 | annot_exons |
| novel_circ_005209 | ENSSSCG00000002127 | 7 | + | 77769110 | 77771399 | 310 | annot_exons |
| novel_circ_005210 | ENSSSCG00000011000 | 10 | - | 33434993 | 33437160 | 332 | annot_exons |
| novel_circ_005211 | ENSSSCG00000007331 | 17 | - | 40205226 | 40206991 | 314 | annot_exons |
| novel_circ_005212 | ENSSSCG00000015423 | 9 | - | 1.03E+08 | 1.03E+08 | 568 | annot_exons |
| novel_circ_005213 | ENSSSCG00000032498 | 7 | - | 47766885 | 47767680 | 322 | annot_exons |
| novel_circ_005214 | ENSSSCG00000003511 | 6 | - | 78979609 | 78981757 | 365 | exon_intron |
| novel_circ_005215 | ENSSSCG00000043604 | X | - | 59570212 | 59571888 | 435 | exon_intron |
| novel_circ_005216 | ENSSSCG00000009551 | 11 | + | 77489152 | 77512780 | 1055 | annot_exons |
| novel_circ_005217 | ENSSSCG00000023994 | 15 | + | 1.32E+08 | 1.32E+08 | 439 | annot_exons |
| novel_circ_005218 | ENSSSCG00000014149 | 2 | - | 96152766 | 96165180 | 517 | annot_exons |
| novel_circ_005219 | ENSSSCG00000008943 | 8 | + | 68260211 | 68266603 | 6393 | intronic |
| novel_circ_005220 | ENSSSCG00000011163 | 10 | + | 69070327 | 69079106 | 994 | annot_exons |
| novel_circ_005221 | ENSSSCG00000004570 | 1 | - | 1.09E+08 | 1.09E+08 | 2280 | one_exon |
| novel_circ_005222 | ENSSSCG00000004934 | 1 | - | 1.63E+08 | 1.63E+08 | 581 | annot_exons |
| novel_circ_005223 | ENSSSCG00000015480 | 9 | + | 1.14E+08 | 1.14E+08 | 441 | annot_exons |
| novel_circ_005224 | ENSSSCG00000015743 | 15 | - | 31467437 | 31473618 | 6182 | exon_intron |
| novel_circ_005225 | ENSSSCG00000011454 | 13 | - | 35054802 | 35058541 | 595 | annot_exons |
| novel_circ_005226 | ENSSSCG00000003776 | 6 | - | 1.38E+08 | 1.38E+08 | 346 | annot_exons |
| novel_circ_005227 | ENSSSCG00000017608 | 12 | + | 32060088 | 32067455 | 494 | annot_exons |
| novel_circ_005228 | ENSSSCG00000027293 | 16 | + | 49107259 | 49143198 | 2136 | annot_exons |
| novel_circ_005229 | ENSSSCG00000000869 | 5 | - | 82859915 | 82865192 | 353 | annot_exons |
| novel_circ_005230 | NA | 15 | + | 84273978 | 84274797 | 820 | intergenic |
| novel_circ_005231 | ENSSSCG00000043503 | 13 | + | 1.21E+08 | 1.21E+08 | 576 | annot_exons |
| novel_circ_005232 | ENSSSCG00000001499 | 7 | + | 29053170 | 29055757 | 267 | annot_exons |
| novel_circ_005233 | ENSSSCG00000014132 | 2 | + | 90580651 | 90594124 | 237 | annot_exons |
| novel_circ_005234 | ENSSSCG00000032146 | 7 | + | 7782168 | 7782370 | 203 | one_exon |
| novel_circ_005235 | ENSSSCG00000011775 | 13 | + | 1.22E+08 | 1.22E+08 | 1652 | annot_exons |
| novel_circ_005236 | ENSSSCG00000008167 | 3 | - | 53073334 | 53077591 | 322 | exon_intron |
| novel_circ_005237 | ENSSSCG00000014913 | 9 | - | 19875266 | 19913189 | 1128 | annot_exons |
| novel_circ_005238 | NA | 15 | - | 84354981 | 84367087 | 12107 | intergenic |
| novel_circ_005239 | ENSSSCG00000009858 | 14 | + | 35326610 | 35342568 | 936 | annot_exons |
| novel_circ_005240 | ENSSSCG00000009522 | 11 | + | 69121334 | 69131726 | 220 | annot_exons |
| novel_circ_005241 | ENSSSCG00000003684 | 6 | - | 99103458 | 99104726 | 1269 | one_exon |
| novel_circ_005242 | ENSSSCG00000001971 | 7 | + | 68385921 | 68386742 | 275 | annot_exons |
| novel_circ_005243 | ENSSSCG00000009298 | 11 | + | 4118125 | 4143040 | 331 | annot_exons |
| novel_circ_005244 | ENSSSCG00000004373 | 1 | - | 72775619 | 72809371 | 890 | annot_exons |
| novel_circ_005245 | ENSSSCG00000040887 | 6 | - | 34751603 | 34756041 | 311 | annot_exons |
| novel_circ_005246 | ENSSSCG00000011340 | 13 | - | 30516967 | 30564659 | 970 | annot_exons |
| novel_circ_005247 | ENSSSCG00000006090 | 4 | + | 40393080 | 40393660 | 220 | annot_exons |
| novel_circ_005248 | ENSSSCG00000004103 | 1 | - | 16213409 | 16224015 | 529 | annot_exons |
| novel_circ_005249 | ENSSSCG00000026041 | 1 | + | 27473761 | 27478260 | 447 | annot_exons |
| novel_circ_005250 | ENSSSCG00000012642 | X | + | 1.02E+08 | 1.02E+08 | 849 | annot_exons |
| novel_circ_005251 | ENSSSCG00000003812 | 6 | - | 1.49E+08 | 1.49E+08 | 1218 | annot_exons |
| novel_circ_005252 | ENSSSCG00000007979 | 3 | + | 41460222 | 41461373 | 255 | annot_exons |
| novel_circ_005253 | ENSSSCG00000011394 | 13 | + | 32548617 | 32551325 | 492 | annot_exons |
| novel_circ_005254 | ENSSSCG00000005073 | 1 | + | 1.87E+08 | 1.87E+08 | 269 | annot_exons |
| novel_circ_005255 | ENSSSCG00000000927 | 5 | - | 94368661 | 94375527 | 536 | annot_exons |
| novel_circ_005256 | ENSSSCG00000028148 | X | - | 28087912 | 28088376 | 465 | intronic |
| novel_circ_005257 | ENSSSCG00000014127 | 2 | + | 89692984 | 89696375 | 216 | annot_exons |
| novel_circ_005258 | NA | 15 | - | 84282611 | 84283069 | 459 | intergenic |
| novel_circ_005259 | NA | 15 | + | 84271980 | 84272321 | 342 | intergenic |
| novel_circ_005260 | NA | 4 | - | 82358445 | 82363319 | 4875 | intergenic |
| novel_circ_005261 | ENSSSCG00000002690 | 6 | - | 6946245 | 6949483 | 529 | annot_exons |
| novel_circ_005262 | ENSSSCG00000042703 | 1 | - | 1.08E+08 | 1.08E+08 | 297 | annot_exons |
| novel_circ_005263 | ENSSSCG00000015795 | 15 | - | 46516328 | 46535584 | 1684 | exon_intron |
| novel_circ_005264 | ENSSSCG00000004082 | 1 | + | 14038312 | 14038852 | 330 | annot_exons |
| novel_circ_005265 | ENSSSCG00000008445 | 3 | + | 94934784 | 94958933 | 761 | annot_exons |
| novel_circ_005266 | ENSSSCG00000035161 | 15 | - | 1.05E+08 | 1.05E+08 | 282 | annot_exons |
| novel_circ_005267 | ENSSSCG00000007950 | 3 | + | 38262252 | 38277042 | 514 | annot_exons |
| novel_circ_005268 | ENSSSCG00000024623 | 13 | + | 1.81E+08 | 1.81E+08 | 431 | annot_exons |
| novel_circ_005269 | ENSSSCG00000015820 | 15 | + | 48227848 | 48228856 | 316 | annot_exons |
| novel_circ_005270 | ENSSSCG00000016911 | 16 | + | 34486719 | 34493412 | 381 | annot_exons |
| novel_circ_005271 | ENSSSCG00000014882 | 9 | - | 12195434 | 12227414 | 391 | annot_exons |
| novel_circ_005272 | ENSSSCG00000014913 | 9 | - | 19897760 | 19929369 | 16596 | exon_intron |
| novel_circ_005273 | NA | 14 | + | 77846849 | 77862429 | 15581 | intergenic |
| novel_circ_005274 | ENSSSCG00000009551 | 11 | + | 77498616 | 77503607 | 314 | annot_exons |
| novel_circ_005275 | ENSSSCG00000024481 | 4 | - | 1.01E+08 | 1.01E+08 | 495 | annot_exons |
| novel_circ_005276 | ENSSSCG00000032275 | 16 | + | 29160649 | 29163072 | 116 | annot_exons |
| novel_circ_005277 | ENSSSCG00000024927 | 6 | + | 84934413 | 84936156 | 170 | annot_exons |
| novel_circ_005278 | ENSSSCG00000017735 | 12 | - | 42741473 | 42758003 | 526 | annot_exons |
| novel_circ_005279 | ENSSSCG00000004529 | 1 | + | 1.04E+08 | 1.04E+08 | 734 | annot_exons |
| novel_circ_005280 | ENSSSCG00000031274 | 2 | + | 80535124 | 80536441 | 305 | annot_exons |
| novel_circ_005281 | ENSSSCG00000006126 | 4 | - | 46733885 | 46735800 | 220 | annot_exons |
| novel_circ_005282 | ENSSSCG00000015050 | 9 | - | 41365582 | 41368124 | 433 | annot_exons |
| novel_circ_005283 | ENSSSCG00000038248 | 16 | - | 65592396 | 65595584 | 563 | annot_exons |
| novel_circ_005284 | ENSSSCG00000031271 | 15 | + | 1.05E+08 | 1.05E+08 | 893 | annot_exons |
| novel_circ_005285 | ENSSSCG00000028327 | 14 | - | 64354863 | 64368587 | 13361 | exon_intron |
| novel_circ_005286 | ENSSSCG00000010897 | 10 | - | 20060944 | 20073030 | 528 | annot_exons |
| novel_circ_005287 | NA | 14 | - | 41634910 | 41639999 | 5090 | intergenic |
| novel_circ_005288 | ENSSSCG00000008068 | 3 | - | 41951780 | 41955342 | 423 | annot_exons |
| novel_circ_005289 | ENSSSCG00000001076 | 7 | + | 14161464 | 14168049 | 246 | annot_exons |
| novel_circ_005290 | ENSSSCG00000008247 | 3 | + | 60152979 | 60158332 | 221 | annot_exons |
| novel_circ_005291 | ENSSSCG00000022240 | X | + | 20910055 | 20912062 | 311 | annot_exons |
| novel_circ_005292 | ENSSSCG00000012055 | 13 | + | 2E+08 | 2E+08 | 665 | exon_intron |
| novel_circ_005293 | ENSSSCG00000007231 | 17 | + | 35505036 | 35506063 | 346 | annot_exons |
| novel_circ_005294 | ENSSSCG00000010652 | 14 | + | 1.25E+08 | 1.25E+08 | 235 | annot_exons |
| novel_circ_005295 | ENSSSCG00000018045 | 12 | + | 59834074 | 59839936 | 312 | annot_exons |
| novel_circ_005296 | ENSSSCG00000004934 | 1 | - | 1.63E+08 | 1.63E+08 | 409 | annot_exons |
| novel_circ_005297 | ENSSSCG00000029602 | 10 | - | 68509412 | 68512874 | 258 | annot_exons |
| novel_circ_005298 | ENSSSCG00000000612 | 5 | - | 58032630 | 58036502 | 339 | annot_exons |
| novel_circ_005299 | ENSSSCG00000028771 | 1 | + | 1.38E+08 | 1.38E+08 | 738 | annot_exons |
| novel_circ_005300 | ENSSSCG00000015583 | 9 | - | 1.29E+08 | 1.29E+08 | 297 | annot_exons |
| novel_circ_005301 | ENSSSCG00000012818 | X | - | 1.25E+08 | 1.25E+08 | 326 | annot_exons |
| novel_circ_005302 | ENSSSCG00000013538 | 2 | + | 72664994 | 72665377 | 306 | annot_exons |
| novel_circ_005303 | ENSSSCG00000016125 | 15 | - | 1.09E+08 | 1.09E+08 | 376 | annot_exons |
| novel_circ_005304 | ENSSSCG00000004979 | 1 | - | 1.69E+08 | 1.69E+08 | 438 | annot_exons |
| novel_circ_005305 | ENSSSCG00000008143 | 3 | + | 48893039 | 48909045 | 378 | annot_exons |
| novel_circ_005306 | ENSSSCG00000000869 | 5 | - | 82852270 | 82859067 | 879 | annot_exons |
| novel_circ_005307 | ENSSSCG00000029920 | 8 | + | 87789294 | 87820356 | 608 | annot_exons |
| novel_circ_005308 | ENSSSCG00000032622 | 14 | + | 6763066 | 6803080 | 1820 | annot_exons |
| novel_circ_005309 | ENSSSCG00000033196 | 10 | - | 32418838 | 32419274 | 437 | antisense |
| novel_circ_005310 | ENSSSCG00000017277 | 12 | + | 14588252 | 14593304 | 610 | annot_exons |
| novel_circ_005311 | ENSSSCG00000032198 | 15 | - | 13156175 | 13157038 | 410 | annot_exons |
| novel_circ_005312 | ENSSSCG00000011553 | 13 | + | 66114472 | 66116302 | 680 | annot_exons |
| novel_circ_005313 | ENSSSCG00000010742 | 14 | - | 1.35E+08 | 1.35E+08 | 580 | annot_exons |
| novel_circ_005314 | ENSSSCG00000007840 | 3 | - | 23867538 | 23869023 | 420 | annot_exons |
| novel_circ_005315 | ENSSSCG00000023520 | 1 | + | 2.29E+08 | 2.29E+08 | 221 | annot_exons |
| novel_circ_005316 | ENSSSCG00000026382 | 1 | + | 1.95E+08 | 1.95E+08 | 879 | intronic |
| novel_circ_005317 | NA | 9 | + | 1.22E+08 | 1.22E+08 | 13407 | intergenic |
| novel_circ_005318 | ENSSSCG00000006090 | 4 | + | 40379783 | 40380122 | 340 | one_exon |
| novel_circ_005319 | ENSSSCG00000022830 | 15 | - | 1.13E+08 | 1.13E+08 | 340 | annot_exons |
| novel_circ_005320 | ENSSSCG00000007951 | 3 | + | 38490819 | 38494815 | 787 | annot_exons |
| novel_circ_005321 | ENSSSCG00000003630 | 6 | + | 92102426 | 92130264 | 639 | annot_exons |
| novel_circ_005322 | ENSSSCG00000003976 | 6 | - | 1.7E+08 | 1.7E+08 | 1741 | annot_exons |
| novel_circ_005323 | ENSSSCG00000008635 | 3 | + | 1.26E+08 | 1.26E+08 | 818 | annot_exons |
| novel_circ_005324 | ENSSSCG00000009460 | 11 | + | 45046357 | 45046921 | 565 | antisense |
| novel_circ_005325 | ENSSSCG00000023974 | 2 | + | 16418775 | 16420440 | 1666 | intronic |
| novel_circ_005326 | ENSSSCG00000024621 | 13 | + | 7059116 | 7073598 | 706 | annot_exons |
| novel_circ_005327 | ENSSSCG00000010134 | 14 | + | 51455314 | 51458739 | 1936 | annot_exons |
| novel_circ_005328 | ENSSSCG00000021285 | 3 | - | 4288138 | 4308978 | 1421 | annot_exons |
| novel_circ_005329 | ENSSSCG00000013638 | 2 | + | 69427148 | 69427731 | 408 | annot_exons |
| novel_circ_005330 | ENSSSCG00000027902 | 6 | + | 96011445 | 96037109 | 831 | annot_exons |
| novel_circ_005331 | ENSSSCG00000011136 | 10 | - | 65215038 | 65220864 | 1544 | annot_exons |
| novel_circ_005332 | ENSSSCG00000015247 | 9 | - | 56959726 | 56963085 | 249 | annot_exons |
| novel_circ_005333 | ENSSSCG00000006196 | 4 | + | 65502957 | 65557837 | 715 | annot_exons |
| novel_circ_005334 | ENSSSCG00000013988 | 2 | - | 51286946 | 51287318 | 373 | antisense |
| novel_circ_005335 | ENSSSCG00000022895 | 13 | + | 1.62E+08 | 1.62E+08 | 511 | antisense |
| novel_circ_005336 | ENSSSCG00000011045 | 10 | + | 45992309 | 46027671 | 788 | annot_exons |
| novel_circ_005337 | ENSSSCG00000010209 | 14 | - | 62683404 | 62701927 | 183 | annot_exons |
| novel_circ_005338 | ENSSSCG00000030396 | 8 | + | 87502698 | 87512770 | 390 | annot_exons |
| novel_circ_005339 | ENSSSCG00000016147 | 15 | + | 1.11E+08 | 1.11E+08 | 589 | annot_exons |
| novel_circ_005340 | ENSSSCG00000015784 | 15 | - | 45877613 | 45882359 | 462 | annot_exons |
| novel_circ_005341 | ENSSSCG00000014902 | 9 | + | 17351072 | 17355850 | 421 | annot_exons |
| novel_circ_005342 | ENSSSCG00000020717 | 8 | - | 77416163 | 77429247 | 1210 | annot_exons |
| novel_circ_005343 | ENSSSCG00000014802 | 9 | + | 6630152 | 6630874 | 723 | antisense |
| novel_circ_005344 | ENSSSCG00000008991 | 8 | + | 73924333 | 73926488 | 389 | annot_exons |
| novel_circ_005345 | ENSSSCG00000036078 | 15 | + | 62274924 | 62275247 | 324 | exon_intron |
| novel_circ_005346 | ENSSSCG00000004829 | 6 | - | 1.48E+08 | 1.48E+08 | 6668 | exon_intron |
| novel_circ_005347 | ENSSSCG00000011678 | 13 | - | 83064769 | 83067048 | 2280 | exon_intron |
| novel_circ_005348 | ENSSSCG00000024623 | 13 | + | 1.81E+08 | 1.81E+08 | 287 | annot_exons |
| novel_circ_005349 | ENSSSCG00000027459 | 13 | + | 90108498 | 90137838 | 291 | annot_exons |
| novel_circ_005350 | ENSSSCG00000008644 | 3 | + | 1.27E+08 | 1.27E+08 | 313 | annot_exons |
| novel_circ_005351 | ENSSSCG00000007839 | 3 | - | 23892951 | 23911080 | 628 | annot_exons |
| novel_circ_005352 | ENSSSCG00000036145 | 6 | - | 1.56E+08 | 1.56E+08 | 857 | annot_exons |
| novel_circ_005353 | ENSSSCG00000007098 | 17 | + | 26816057 | 26817853 | 327 | annot_exons |
| novel_circ_005354 | ENSSSCG00000029602 | 10 | - | 68491183 | 68512874 | 1218 | annot_exons |
| novel_circ_005355 | ENSSSCG00000029485 | 3 | + | 92781215 | 92784772 | 390 | annot_exons |
| novel_circ_005356 | ENSSSCG00000010151 | 14 | + | 55583796 | 55599982 | 981 | annot_exons |
| novel_circ_005357 | ENSSSCG00000024596 | 8 | - | 87886825 | 87889383 | 1061 | annot_exons |
| novel_circ_005358 | ENSSSCG00000003398 | 6 | + | 70428067 | 70430862 | 529 | annot_exons |
| novel_circ_005359 | NA | 15 | - | 84271295 | 84271773 | 479 | intergenic |
| novel_circ_005360 | ENSSSCG00000016943 | 16 | - | 43832056 | 43840787 | 426 | annot_exons |
| novel_circ_005361 | ENSSSCG00000006267 | 4 | + | 78139386 | 78156274 | 3918 | annot_exons |
| novel_circ_005362 | ENSSSCG00000012000 | 13 | + | 1.74E+08 | 1.74E+08 | 286 | annot_exons |
| novel_circ_005363 | ENSSSCG00000024813 | 12 | - | 15255206 | 15269630 | 335 | annot_exons |
| novel_circ_005364 | ENSSSCG00000017676 | 12 | - | 37178174 | 37187452 | 396 | annot_exons |
| novel_circ_005365 | ENSSSCG00000016954 | 16 | + | 44454933 | 44473184 | 408 | annot_exons |
| novel_circ_005366 | ENSSSCG00000009345 | 11 | + | 9145564 | 9170916 | 550 | annot_exons |
| novel_circ_005367 | ENSSSCG00000036431 | 1 | + | 1.1E+08 | 1.1E+08 | 421 | annot_exons |
| novel_circ_005368 | ENSSSCG00000003673 | 6 | - | 96686732 | 96690762 | 513 | annot_exons |
| novel_circ_005369 | ENSSSCG00000017668 | 12 | + | 35952997 | 35956744 | 329 | annot_exons |
| novel_circ_005370 | ENSSSCG00000004254 | 1 | - | 44323601 | 44350272 | 473 | annot_exons |
| novel_circ_005371 | ENSSSCG00000028225 | 7 | - | 1.16E+08 | 1.16E+08 | 531 | annot_exons |
| novel_circ_005372 | ENSSSCG00000006866 | 4 | + | 1.18E+08 | 1.18E+08 | 270 | annot_exons |
| novel_circ_005373 | ENSSSCG00000008831 | 8 | + | 39008497 | 39021487 | 318 | annot_exons |
| novel_circ_005374 | ENSSSCG00000025606 | 9 | + | 36707534 | 36708547 | 341 | annot_exons |
| novel_circ_005375 | ENSSSCG00000010987 | 10 | + | 32859214 | 32868451 | 846 | annot_exons |
| novel_circ_005376 | ENSSSCG00000008812 | 8 | + | 37441277 | 37445470 | 522 | annot_exons |
| novel_circ_005377 | ENSSSCG00000014902 | 9 | + | 17346054 | 17351207 | 364 | annot_exons |
| novel_circ_005378 | ENSSSCG00000018049 | 12 | - | 60369555 | 60376920 | 1033 | exon_intron |
| novel_circ_005379 | ENSSSCG00000010470 | 14 | - | 1.04E+08 | 1.04E+08 | 686 | annot_exons |
| novel_circ_005380 | ENSSSCG00000016695 | 18 | + | 45008376 | 45020744 | 982 | annot_exons |
| novel_circ_005381 | ENSSSCG00000039085 | 13 | + | 2.07E+08 | 2.07E+08 | 262 | annot_exons |
| novel_circ_005382 | ENSSSCG00000004587 | 1 | + | 1.13E+08 | 1.13E+08 | 1031 | annot_exons |
| novel_circ_005383 | ENSSSCG00000017543 | 12 | + | 25066506 | 25069215 | 285 | annot_exons |
| novel_circ_005384 | ENSSSCG00000008637 | 3 | - | 1.27E+08 | 1.27E+08 | 436 | annot_exons |
| novel_circ_005385 | ENSSSCG00000006730 | 4 | - | 1.03E+08 | 1.03E+08 | 625 | annot_exons |
| novel_circ_005386 | ENSSSCG00000041409 | 1 | + | 77162324 | 77164387 | 286 | annot_exons |
| novel_circ_005387 | ENSSSCG00000008697 | 8 | + | 1820703 | 1824798 | 205 | annot_exons |
| novel_circ_005388 | ENSSSCG00000009716 | 14 | + | 20521354 | 20522546 | 372 | annot_exons |
| novel_circ_005389 | ENSSSCG00000008515 | 3 | + | 1.08E+08 | 1.08E+08 | 225 | annot_exons |
| novel_circ_005390 | ENSSSCG00000002755 | 6 | - | 17316576 | 17320176 | 2646 | annot_exons |
| novel_circ_005391 | ENSSSCG00000007246 | 17 | + | 35798687 | 35803202 | 725 | annot_exons |
| novel_circ_005392 | ENSSSCG00000030413 | 13 | + | 1.17E+08 | 1.17E+08 | 638 | annot_exons |
| novel_circ_005393 | ENSSSCG00000028855 | 13 | + | 95786312 | 95787213 | 377 | annot_exons |
| novel_circ_005394 | ENSSSCG00000037775 | AEMK02000452.1 | + | 3764266 | 3764560 | 295 | antisense |
| novel_circ_005395 | ENSSSCG00000038437 | AEMK02000682.1 | - | 277825 | 278294 | 470 | exon_intron |
| novel_circ_005396 | ENSSSCG00000014258 | 2 | + | 1.32E+08 | 1.32E+08 | 246 | one_exon |
| novel_circ_005397 | ENSSSCG00000010313 | 14 | + | 76763060 | 76763499 | 279 | annot_exons |
| novel_circ_005398 | ENSSSCG00000014057 | 2 | - | 81418818 | 81425202 | 437 | annot_exons |
| novel_circ_005399 | ENSSSCG00000000853 | 5 | + | 80664889 | 80673677 | 796 | annot_exons |
| novel_circ_005400 | ENSSSCG00000000795 | 5 | - | 74573058 | 74573624 | 487 | annot_exons |
| novel_circ_005401 | ENSSSCG00000015427 | 9 | - | 1.04E+08 | 1.04E+08 | 224 | annot_exons |
| novel_circ_005402 | ENSSSCG00000000770 | 5 | - | 69972153 | 69978167 | 602 | annot_exons |
| novel_circ_005403 | ENSSSCG00000013284 | 2 | - | 18867494 | 18885581 | 358 | annot_exons |
| novel_circ_005404 | ENSSSCG00000020878 | 3 | + | 1.02E+08 | 1.02E+08 | 1389 | annot_exons |
| novel_circ_005405 | ENSSSCG00000037663 | 13 | + | 2.06E+08 | 2.06E+08 | 924 | annot_exons |
| novel_circ_005406 | ENSSSCG00000003798 | 6 | - | 1.45E+08 | 1.45E+08 | 307 | antisense |
| novel_circ_005407 | ENSSSCG00000008388 | 3 | - | 80899164 | 80901790 | 292 | annot_exons |
| novel_circ_005408 | ENSSSCG00000038807 | 6 | - | 54697207 | 54697397 | 191 | antisense |
| novel_circ_005409 | ENSSSCG00000038428 | AEMK02000682.1 | - | 876253 | 876955 | 398 | annot_exons |
| novel_circ_005410 | ENSSSCG00000009480 | 11 | + | 51547073 | 51554792 | 3391 | annot_exons |
| novel_circ_005411 | ENSSSCG00000016888 | 16 | - | 32448055 | 32459786 | 273 | annot_exons |
| novel_circ_005412 | ENSSSCG00000004718 | 1 | + | 1.29E+08 | 1.29E+08 | 246 | annot_exons |
| novel_circ_005413 | ENSSSCG00000001732 | 7 | - | 43609181 | 43617387 | 807 | annot_exons |
| novel_circ_005414 | ENSSSCG00000010241 | 14 | + | 71799479 | 71806756 | 466 | annot_exons |
| novel_circ_005415 | ENSSSCG00000006911 | 4 | + | 1.25E+08 | 1.25E+08 | 1590 | annot_exons |
| novel_circ_005416 | ENSSSCG00000017513 | 12 | + | 23869313 | 23872399 | 275 | annot_exons |
| novel_circ_005417 | ENSSSCG00000014828 | 9 | - | 8155799 | 8157830 | 218 | annot_exons |
| novel_circ_005418 | ENSSSCG00000023866 | 6 | - | 684771 | 692304 | 411 | annot_exons |
| novel_circ_005419 | ENSSSCG00000000970 | 5 | - | 304707 | 305202 | 227 | annot_exons |
| novel_circ_005420 | ENSSSCG00000047496 | 1 | - | 1.46E+08 | 1.46E+08 | 285 | annot_exons |
| novel_circ_005421 | ENSSSCG00000003401 | 6 | + | 70609103 | 70655830 | 2831 | annot_exons |
| novel_circ_005422 | ENSSSCG00000035901 | 14 | + | 281357 | 296713 | 4131 | annot_exons |
| novel_circ_005423 | ENSSSCG00000012006 | 13 | - | 1.79E+08 | 1.79E+08 | 339 | antisense |
| novel_circ_005424 | ENSSSCG00000010190 | 14 | - | 60414289 | 60415589 | 1301 | antisense |
| novel_circ_005425 | ENSSSCG00000004358 | 1 | - | 67363177 | 67400887 | 472 | annot_exons |
| novel_circ_005426 | ENSSSCG00000011782 | 13 | - | 1.22E+08 | 1.22E+08 | 350 | annot_exons |
| novel_circ_005427 | ENSSSCG00000013262 | 2 | + | 15967840 | 15970677 | 670 | annot_exons |
| novel_circ_005428 | ENSSSCG00000007280 | 17 | + | 37836857 | 37841896 | 451 | annot_exons |
| novel_circ_005429 | ENSSSCG00000006889 | 4 | + | 1.23E+08 | 1.23E+08 | 372 | annot_exons |
| novel_circ_005430 | ENSSSCG00000007799 | 3 | - | 17939272 | 17940181 | 440 | annot_exons |
| novel_circ_005431 | ENSSSCG00000004508 | 1 | - | 98725588 | 98725770 | 183 | one_exon |
| novel_circ_005432 | ENSSSCG00000022112 | 11 | - | 67826919 | 67854350 | 1646 | annot_exons |
| novel_circ_005433 | ENSSSCG00000006100 | 4 | + | 42205209 | 42216935 | 840 | annot_exons |
| novel_circ_005434 | ENSSSCG00000004415 | 1 | + | 75326041 | 75333864 | 451 | annot_exons |
| novel_circ_005435 | ENSSSCG00000004561 | 1 | + | 1.08E+08 | 1.08E+08 | 1016 | annot_exons |
| novel_circ_005436 | ENSSSCG00000011045 | 10 | + | 46027591 | 46059990 | 1700 | exon_intron |
| novel_circ_005437 | ENSSSCG00000011036 | 10 | + | 43994555 | 43996556 | 288 | annot_exons |
| novel_circ_005438 | ENSSSCG00000040184 | 11 | + | 48078638 | 48116048 | 2577 | annot_exons |
| novel_circ_005439 | ENSSSCG00000004576 | 1 | + | 1.11E+08 | 1.11E+08 | 33946 | intronic |
| novel_circ_005440 | ENSSSCG00000002266 | 7 | - | 86024175 | 86056387 | 1694 | annot_exons |
| novel_circ_005441 | ENSSSCG00000012434 | X | - | 61698880 | 61702090 | 577 | annot_exons |
| novel_circ_005442 | ENSSSCG00000038872 | 6 | + | 85664602 | 85677583 | 500 | annot_exons |
| novel_circ_005443 | ENSSSCG00000003630 | 6 | + | 92161587 | 92170345 | 562 | annot_exons |
| novel_circ_005444 | ENSSSCG00000025417 | 6 | - | 18553565 | 18555525 | 457 | annot_exons |
| novel_circ_005445 | ENSSSCG00000033185 | 13 | - | 77076265 | 77076592 | 328 | antisense |
| novel_circ_005446 | ENSSSCG00000030095 | 9 | + | 41738255 | 41768627 | 185 | annot_exons |
| novel_circ_005447 | ENSSSCG00000003379 | 6 | + | 67422702 | 67434056 | 11355 | antisense |
| novel_circ_005448 | NA | 7 | + | 68191893 | 68204994 | 13102 | intergenic |
| novel_circ_005449 | ENSSSCG00000017155 | 12 | + | 2308469 | 2311186 | 352 | annot_exons |
| novel_circ_005450 | ENSSSCG00000000475 | 5 | + | 30594364 | 30599293 | 1285 | annot_exons |
| novel_circ_005451 | ENSSSCG00000006038 | 4 | - | 31964422 | 31996226 | 8048 | exon_intron |
| novel_circ_005452 | ENSSSCG00000039060 | 5 | + | 29104851 | 29107579 | 348 | annot_exons |
| novel_circ_005453 | ENSSSCG00000008943 | 8 | + | 67987917 | 68022509 | 353 | annot_exons |
| novel_circ_005454 | NA | 15 | - | 84418028 | 84418182 | 155 | intergenic |
| novel_circ_005455 | ENSSSCG00000042363 | 1 | + | 1.7E+08 | 1.7E+08 | 36351 | exon_intron |
| novel_circ_005456 | ENSSSCG00000023112 | 8 | - | 31017611 | 31019705 | 255 | annot_exons |
| novel_circ_005457 | ENSSSCG00000006865 | 4 | - | 1.18E+08 | 1.18E+08 | 325 | annot_exons |
| novel_circ_005458 | ENSSSCG00000011563 | 13 | + | 66283421 | 66284493 | 217 | annot_exons |
| novel_circ_005459 | ENSSSCG00000005015 | 1 | - | 1.8E+08 | 1.8E+08 | 981 | annot_exons |
| novel_circ_005460 | ENSSSCG00000011376 | 13 | + | 31809498 | 31812007 | 339 | annot_exons |
| novel_circ_005461 | ENSSSCG00000011782 | 13 | - | 1.22E+08 | 1.22E+08 | 283 | annot_exons |
| novel_circ_005462 | ENSSSCG00000039119 | 3 | + | 7486289 | 7491522 | 406 | annot_exons |
| novel_circ_005463 | ENSSSCG00000014331 | 2 | + | 1.4E+08 | 1.4E+08 | 773 | annot_exons |
| novel_circ_005464 | ENSSSCG00000040738 | AEMK02000452.1 | + | 879297 | 879605 | 215 | annot_exons |
| novel_circ_005465 | ENSSSCG00000008382 | 3 | + | 80483094 | 80500119 | 690 | annot_exons |
| novel_circ_005466 | ENSSSCG00000006619 | 4 | - | 97566524 | 97576948 | 606 | annot_exons |
| novel_circ_005467 | NA | 15 | - | 84471405 | 84471993 | 589 | intergenic |
| novel_circ_005468 | ENSSSCG00000001068 | 7 | + | 13217738 | 13237201 | 13068 | exon_intron |
| novel_circ_005469 | NA | 15 | - | 84254261 | 84254931 | 671 | intergenic |
| novel_circ_005470 | ENSSSCG00000011752 | 13 | + | 1.11E+08 | 1.11E+08 | 295 | annot_exons |
| novel_circ_005471 | ENSSSCG00000024814 | X | - | 47764058 | 47767213 | 895 | annot_exons |
| novel_circ_005472 | ENSSSCG00000003630 | 6 | + | 92155928 | 92165946 | 748 | annot_exons |
| novel_circ_005473 | ENSSSCG00000014913 | 9 | - | 19892116 | 19913189 | 528 | annot_exons |
| novel_circ_005474 | ENSSSCG00000007727 | 3 | + | 13854403 | 13854615 | 213 | one_exon |
| novel_circ_005475 | ENSSSCG00000029082 | 9 | + | 1.05E+08 | 1.05E+08 | 2962 | exon_intron |
| novel_circ_005476 | ENSSSCG00000036052 | 15 | + | 84227393 | 84227642 | 250 | antisense |
| novel_circ_005477 | ENSSSCG00000015859 | 15 | + | 59336611 | 59341772 | 486 | annot_exons |
| novel_circ_005478 | ENSSSCG00000003784 | 6 | + | 1.39E+08 | 1.39E+08 | 895 | exon_intron |
| novel_circ_005479 | ENSSSCG00000026705 | 10 | - | 12607745 | 12631297 | 331 | annot_exons |
| novel_circ_005480 | ENSSSCG00000029198 | 9 | + | 36475680 | 36493066 | 663 | annot_exons |
| novel_circ_005481 | ENSSSCG00000011376 | 13 | + | 31778304 | 31779227 | 492 | annot_exons |
| novel_circ_005482 | ENSSSCG00000016510 | 18 | - | 10392308 | 10406199 | 2152 | annot_exons |
| novel_circ_005483 | ENSSSCG00000010548 | 14 | - | 1.11E+08 | 1.11E+08 | 414 | exon_intron |
| novel_circ_005484 | ENSSSCG00000021354 | 18 | + | 13893889 | 13911007 | 464 | annot_exons |
| novel_circ_005485 | ENSSSCG00000023297 | 15 | + | 80385694 | 80410643 | 557 | annot_exons |
| novel_circ_005486 | ENSSSCG00000007585 | 3 | + | 4087561 | 4087646 | 86 | antisense |
| novel_circ_005487 | ENSSSCG00000004071 | 1 | + | 12387963 | 12396662 | 1698 | exon_intron |
| novel_circ_005488 | ENSSSCG00000015695 | 15 | - | 16798554 | 16814482 | 537 | annot_exons |
| novel_circ_005489 | NA | 1 | - | 1.29E+08 | 1.29E+08 | 560 | intergenic |
| novel_circ_005490 | ENSSSCG00000002547 | AEMK02000452.1 | + | 2201474 | 2208500 | 293 | annot_exons |
| novel_circ_005491 | ENSSSCG00000002352 | 7 | - | 97226813 | 97227040 | 228 | antisense |
| novel_circ_005492 | ENSSSCG00000040887 | 6 | - | 34743079 | 34747797 | 419 | annot_exons |
| novel_circ_005493 | ENSSSCG00000004624 | 1 | - | 1.2E+08 | 1.2E+08 | 1338 | annot_exons |
| novel_circ_005494 | ENSSSCG00000027176 | 13 | + | 28871682 | 28873176 | 242 | annot_exons |
| novel_circ_005495 | ENSSSCG00000022790 | 2 | + | 1.26E+08 | 1.26E+08 | 363 | annot_exons |
| novel_circ_005496 | ENSSSCG00000006016 | 4 | + | 22038996 | 22053930 | 2538 | annot_exons |
| novel_circ_005497 | ENSSSCG00000009674 | 14 | + | 11880512 | 11883637 | 289 | annot_exons |
| novel_circ_005498 | ENSSSCG00000005232 | 1 | - | 2.2E+08 | 2.2E+08 | 195 | annot_exons |
| novel_circ_005499 | ENSSSCG00000009246 | 8 | - | 1.36E+08 | 1.36E+08 | 653 | annot_exons |
| novel_circ_005500 | ENSSSCG00000004169 | 1 | + | 29018351 | 29027965 | 656 | annot_exons |
| novel_circ_005501 | ENSSSCG00000006755 | 4 | + | 1.06E+08 | 1.06E+08 | 914 | annot_exons |
| novel_circ_005502 | ENSSSCG00000005090 | 1 | + | 1.9E+08 | 1.9E+08 | 227 | annot_exons |
| novel_circ_005503 | ENSSSCG00000003512 | 6 | - | 79150170 | 79249227 | 1858 | annot_exons |
| novel_circ_005504 | ENSSSCG00000004205 | 1 | + | 33672928 | 33681370 | 336 | annot_exons |
| novel_circ_005505 | ENSSSCG00000008987 | 8 | - | 72695065 | 72698949 | 576 | annot_exons |
| novel_circ_005506 | ENSSSCG00000036948 | 10 | + | 23636516 | 23637783 | 263 | annot_exons |
| novel_circ_005507 | ENSSSCG00000001852 | 7 | + | 55654471 | 55654672 | 202 | antisense |
| novel_circ_005508 | ENSSSCG00000024636 | 3 | + | 49493943 | 49496419 | 350 | annot_exons |
| novel_circ_005509 | ENSSSCG00000017274 | 12 | + | 14038571 | 14040563 | 320 | annot_exons |
| novel_circ_005510 | ENSSSCG00000011894 | 13 | + | 1.41E+08 | 1.41E+08 | 790 | annot_exons |
| novel_circ_005511 | ENSSSCG00000016045 | 15 | + | 94387078 | 94414945 | 1567 | exon_intron |
| novel_circ_005512 | ENSSSCG00000004802 | 1 | + | 1.36E+08 | 1.36E+08 | 1233 | annot_exons |
| novel_circ_005513 | ENSSSCG00000009018 | 8 | - | 77690008 | 77690319 | 312 | antisense |
| novel_circ_005514 | ENSSSCG00000014195 | 2 | + | 1.15E+08 | 1.15E+08 | 569 | annot_exons |
| novel_circ_005515 | ENSSSCG00000017489 | 12 | - | 22350995 | 22351458 | 295 | annot_exons |
| novel_circ_005516 | ENSSSCG00000015286 | 9 | - | 65839293 | 65853464 | 1813 | annot_exons |
| novel_circ_005517 | NA | 14 | - | 41669450 | 41672257 | 2808 | intergenic |
| novel_circ_005518 | ENSSSCG00000001078 | 7 | - | 15577056 | 15585059 | 320 | annot_exons |
| novel_circ_005519 | ENSSSCG00000000509 | 5 | - | 35635564 | 35640848 | 847 | annot_exons |
| novel_circ_005520 | ENSSSCG00000006032 | 4 | - | 29038357 | 29047618 | 455 | annot_exons |
| novel_circ_005521 | ENSSSCG00000000847 | 5 | - | 80479558 | 80480419 | 314 | annot_exons |
| novel_circ_005522 | ENSSSCG00000037267 | 7 | + | 89143637 | 89143830 | 194 | antisense |
| novel_circ_005523 | ENSSSCG00000000612 | 5 | - | 58036347 | 58038309 | 235 | annot_exons |
| novel_circ_005524 | ENSSSCG00000037355 | 4 | + | 99533807 | 99537986 | 4180 | exon_intron |
| novel_circ_005525 | ENSSSCG00000004032 | 1 | + | 5605263 | 5698673 | 73957 | exon_intron |
| novel_circ_005526 | ENSSSCG00000036924 | 7 | - | 47442793 | 47444085 | 344 | annot_exons |
| novel_circ_005527 | ENSSSCG00000003412 | 6 | - | 71274645 | 71275042 | 187 | annot_exons |
| novel_circ_005528 | ENSSSCG00000003743 | 6 | + | 1.19E+08 | 1.19E+08 | 5722 | exon_intron |
| novel_circ_005529 | ENSSSCG00000006177 | 4 | + | 62046570 | 62056605 | 195 | annot_exons |
| novel_circ_005530 | ENSSSCG00000016543 | 18 | - | 15347146 | 15347365 | 220 | one_exon |
| novel_circ_005531 | ENSSSCG00000017308 | 12 | + | 16822210 | 16829262 | 350 | annot_exons |
| novel_circ_005532 | ENSSSCG00000022430 | 16 | + | 47301579 | 47306223 | 337 | annot_exons |
| novel_circ_005533 | ENSSSCG00000029082 | 9 | + | 1.05E+08 | 1.05E+08 | 6859 | exon_intron |
| novel_circ_005534 | ENSSSCG00000016306 | 15 | - | 1.34E+08 | 1.34E+08 | 730 | annot_exons |
| novel_circ_005535 | ENSSSCG00000034730 | 1 | - | 96472570 | 96483856 | 476 | annot_exons |
| novel_circ_005536 | ENSSSCG00000013262 | 2 | + | 15969547 | 15970677 | 357 | annot_exons |
| novel_circ_005537 | ENSSSCG00000008591 | 3 | + | 1.15E+08 | 1.15E+08 | 448 | annot_exons |
| novel_circ_005538 | ENSSSCG00000005386 | 1 | + | 2.42E+08 | 2.42E+08 | 253 | annot_exons |
| novel_circ_005539 | ENSSSCG00000008974 | 8 | - | 71638232 | 71643022 | 315 | annot_exons |
| novel_circ_005540 | ENSSSCG00000005320 | 1 | + | 2.36E+08 | 2.36E+08 | 509 | annot_exons |
| novel_circ_005541 | ENSSSCG00000003111 | 6 | + | 52992807 | 53015810 | 324 | annot_exons |
| novel_circ_005542 | ENSSSCG00000035712 | 8 | - | 34963963 | 34987988 | 24026 | intronic |
| novel_circ_005543 | ENSSSCG00000004138 | 1 | + | 22268062 | 22274619 | 6558 | intronic |
| novel_circ_005544 | ENSSSCG00000004576 | 1 | + | 1.11E+08 | 1.11E+08 | 624 | annot_exons |
| novel_circ_005545 | ENSSSCG00000006136 | 4 | + | 50356925 | 50364239 | 414 | annot_exons |
| novel_circ_005546 | ENSSSCG00000009125 | 8 | - | 1.09E+08 | 1.09E+08 | 352 | annot_exons |
| novel_circ_005547 | ENSSSCG00000034017 | 6 | + | 88645545 | 88646110 | 254 | annot_exons |
| novel_circ_005548 | ENSSSCG00000016027 | 15 | + | 91663090 | 91684121 | 1055 | annot_exons |
| novel_circ_005549 | ENSSSCG00000011794 | 13 | + | 1.23E+08 | 1.23E+08 | 273 | annot_exons |
| novel_circ_005550 | ENSSSCG00000029621 | 8 | - | 1.25E+08 | 1.25E+08 | 727 | annot_exons |
| novel_circ_005551 | ENSSSCG00000017094 | 16 | - | 72099772 | 72105944 | 315 | annot_exons |
| novel_circ_005552 | ENSSSCG00000009511 | 11 | - | 67470911 | 67524738 | 741 | annot_exons |
| novel_circ_005553 | ENSSSCG00000020720 | 2 | + | 48822509 | 48835611 | 1023 | exon_intron |
| novel_circ_005554 | ENSSSCG00000036885 | 13 | - | 1.68E+08 | 1.68E+08 | 479 | annot_exons |
| novel_circ_005555 | ENSSSCG00000008831 | 8 | + | 39018212 | 39040610 | 479 | annot_exons |
| novel_circ_005556 | ENSSSCG00000033228 | 12 | - | 27261000 | 27274847 | 307 | annot_exons |
| novel_circ_005557 | ENSSSCG00000001732 | 7 | - | 43613690 | 43616186 | 421 | annot_exons |
| novel_circ_005558 | ENSSSCG00000010396 | 14 | - | 90386719 | 90405784 | 365 | annot_exons |
| novel_circ_005559 | ENSSSCG00000001080 | 7 | + | 16671133 | 16674261 | 374 | annot_exons |
| novel_circ_005560 | ENSSSCG00000003693 | 6 | - | 1.04E+08 | 1.04E+08 | 479 | antisense |
| novel_circ_005561 | ENSSSCG00000009776 | 14 | + | 29612352 | 29614352 | 271 | annot_exons |
| novel_circ_005562 | ENSSSCG00000002502 | 7 | - | 1.18E+08 | 1.18E+08 | 153 | annot_exons |
| novel_circ_005563 | ENSSSCG00000010863 | 10 | + | 15051106 | 15063364 | 888 | annot_exons |
| novel_circ_005564 | ENSSSCG00000016464 | 18 | - | 6914973 | 6918871 | 529 | annot_exons |
| novel_circ_005565 | ENSSSCG00000007023 | 17 | - | 11047513 | 11047829 | 317 | one_exon |
| novel_circ_005566 | ENSSSCG00000014838 | 9 | - | 8695489 | 8707883 | 827 | annot_exons |
| novel_circ_005567 | ENSSSCG00000010295 | 14 | - | 75958945 | 75961262 | 426 | annot_exons |
| novel_circ_005568 | ENSSSCG00000029633 | 6 | + | 1.57E+08 | 1.57E+08 | 516 | annot_exons |
| novel_circ_005569 | ENSSSCG00000005015 | 1 | - | 1.8E+08 | 1.8E+08 | 995 | annot_exons |
| novel_circ_005570 | ENSSSCG00000003630 | 6 | + | 92155928 | 92170345 | 933 | annot_exons |
| novel_circ_005571 | ENSSSCG00000008068 | 3 | - | 41980348 | 41993675 | 1122 | annot_exons |
| novel_circ_005572 | ENSSSCG00000028979 | 9 | + | 10116127 | 10139416 | 3570 | annot_exons |
| novel_circ_005573 | ENSSSCG00000026569 | 3 | - | 32081242 | 32085104 | 231 | annot_exons |
| novel_circ_005574 | ENSSSCG00000005524 | 1 | + | 2.62E+08 | 2.62E+08 | 253 | annot_exons |
| novel_circ_005575 | ENSSSCG00000004008 | 1 | + | 600486 | 611340 | 802 | annot_exons |
| novel_circ_005576 | ENSSSCG00000004349 | 1 | - | 66332071 | 66339064 | 380 | annot_exons |
| novel_circ_005577 | ENSSSCG00000004718 | 1 | + | 1.29E+08 | 1.29E+08 | 285 | annot_exons |
| novel_circ_005578 | ENSSSCG00000037719 | 3 | - | 39445692 | 39454645 | 781 | annot_exons |
| novel_circ_005579 | NA | 15 | + | 84263770 | 84264106 | 337 | intergenic |
| novel_circ_005580 | ENSSSCG00000017601 | 12 | + | 31220402 | 31223236 | 345 | annot_exons |
| novel_circ_005581 | ENSSSCG00000051618 | 14 | - | 30867352 | 30869146 | 1712 | exon_intron |
| novel_circ_005582 | ENSSSCG00000010900 | 10 | - | 20508015 | 20536074 | 546 | annot_exons |
| novel_circ_005583 | ENSSSCG00000006174 | 4 | + | 61635533 | 61636292 | 760 | one_exon |
| novel_circ_005584 | ENSSSCG00000011763 | 13 | - | 1.18E+08 | 1.18E+08 | 310 | annot_exons |
| novel_circ_005585 | ENSSSCG00000008377 | 3 | + | 79929760 | 79941956 | 1996 | annot_exons |
| novel_circ_005586 | ENSSSCG00000004123 | 1 | - | 20908952 | 20910141 | 277 | annot_exons |
| novel_circ_005587 | ENSSSCG00000026996 | 11 | - | 64113353 | 64133950 | 611 | annot_exons |
| novel_circ_005588 | ENSSSCG00000002460 | 7 | + | 1.15E+08 | 1.15E+08 | 1033 | exon_intron |
| novel_circ_005589 | ENSSSCG00000001056 | 7 | - | 9995687 | 10000624 | 407 | annot_exons |
| novel_circ_005590 | ENSSSCG00000006510 | 4 | + | 94216682 | 94220967 | 603 | annot_exons |
| novel_circ_005591 | ENSSSCG00000001708 | 7 | + | 39403319 | 39407636 | 477 | annot_exons |
| novel_circ_005592 | NA | 15 | + | 84468286 | 84468796 | 511 | intergenic |
| novel_circ_005593 | ENSSSCG00000030119 | 4 | - | 98764286 | 98766490 | 374 | annot_exons |
| novel_circ_005594 | ENSSSCG00000004938 | 1 | - | 1.64E+08 | 1.64E+08 | 4603 | exon_intron |
| novel_circ_005595 | ENSSSCG00000017139 | 12 | - | 1746369 | 1752772 | 892 | annot_exons |
| novel_circ_005596 | ENSSSCG00000016968 | 16 | + | 47868004 | 47870677 | 781 | annot_exons |
| novel_circ_005597 | ENSSSCG00000011889 | 13 | + | 1.4E+08 | 1.4E+08 | 286 | annot_exons |
| novel_circ_005598 | ENSSSCG00000032522 | 15 | + | 1.21E+08 | 1.21E+08 | 768 | annot_exons |
| novel_circ_005599 | ENSSSCG00000028855 | 13 | + | 95760812 | 95775714 | 499 | annot_exons |
| novel_circ_005600 | ENSSSCG00000014800 | 9 | + | 6555049 | 6561930 | 384 | annot_exons |
| novel_circ_005601 | ENSSSCG00000026239 | 6 | + | 37957475 | 37962774 | 591 | annot_exons |
| novel_circ_005602 | ENSSSCG00000014960 | 9 | + | 26904866 | 26905802 | 937 | one_exon |
| novel_circ_005603 | ENSSSCG00000027477 | 7 | + | 47864212 | 47874345 | 320 | annot_exons |
| novel_circ_005604 | ENSSSCG00000011833 | 13 | + | 1.33E+08 | 1.33E+08 | 309 | exon_intron |
| novel_circ_005605 | ENSSSCG00000015379 | 9 | + | 90693713 | 90695267 | 359 | annot_exons |
| novel_circ_005606 | ENSSSCG00000016747 | 18 | + | 50771848 | 50772282 | 435 | exon_intron |
| novel_circ_005607 | ENSSSCG00000013511 | 2 | - | 74350301 | 74350628 | 328 | intronic |
| novel_circ_005608 | NA | 15 | + | 84253354 | 84253974 | 621 | intergenic |
| novel_circ_005609 | ENSSSCG00000027128 | 2 | + | 73269742 | 73271691 | 349 | annot_exons |
| novel_circ_005610 | ENSSSCG00000020663 | 18 | + | 5075293 | 5109435 | 4432 | annot_exons |
| novel_circ_005611 | ENSSSCG00000000189 | 5 | - | 15128142 | 15128884 | 231 | annot_exons |
| novel_circ_005612 | ENSSSCG00000005207 | 1 | + | 2.16E+08 | 2.16E+08 | 319 | annot_exons |
| novel_circ_005613 | ENSSSCG00000011765 | 13 | + | 1.18E+08 | 1.18E+08 | 265 | annot_exons |
| novel_circ_005614 | ENSSSCG00000001485 | 7 | - | 26664530 | 26676922 | 1701 | annot_exons |
| novel_circ_005615 | ENSSSCG00000022177 | 15 | + | 1.33E+08 | 1.33E+08 | 307 | annot_exons |
| novel_circ_005616 | ENSSSCG00000033527 | 6 | + | 37201734 | 37204316 | 279 | annot_exons |
| novel_circ_005617 | ENSSSCG00000014061 | 2 | + | 81665842 | 81666223 | 263 | annot_exons |
| novel_circ_005618 | ENSSSCG00000013344 | 2 | + | 37180345 | 37182133 | 1789 | antisense |
| novel_circ_005619 | ENSSSCG00000022534 | 4 | + | 1.26E+08 | 1.26E+08 | 2178 | exon_intron |
| novel_circ_005620 | ENSSSCG00000006136 | 4 | + | 50356925 | 50370969 | 504 | annot_exons |
| novel_circ_005621 | ENSSSCG00000011075 | 10 | + | 50855365 | 50855624 | 260 | antisense |
| novel_circ_005622 | ENSSSCG00000003559 | 6 | + | 84075836 | 84080165 | 783 | annot_exons |
| novel_circ_005623 | ENSSSCG00000023873 | 4 | + | 34250050 | 34254131 | 648 | annot_exons |
| novel_circ_005624 | ENSSSCG00000025287 | 2 | - | 1.09E+08 | 1.09E+08 | 286 | one_exon |
| novel_circ_005625 | NA | 15 | - | 84305531 | 84308301 | 2771 | intergenic |
| novel_circ_005626 | ENSSSCG00000029578 | 6 | + | 28766824 | 28783794 | 4473 | annot_exons |
| novel_circ_005627 | ENSSSCG00000032220 | 13 | + | 18670725 | 18676623 | 465 | annot_exons |
| novel_circ_005628 | ENSSSCG00000012995 | 2 | - | 6880349 | 6881507 | 433 | annot_exons |
| novel_circ_005629 | ENSSSCG00000005997 | 4 | - | 19027765 | 19027950 | 186 | one_exon |
| novel_circ_005630 | ENSSSCG00000009298 | 11 | + | 4150278 | 4153663 | 287 | annot_exons |
| novel_circ_005631 | ENSSSCG00000011652 | 13 | - | 77351950 | 77384654 | 895 | annot_exons |
| novel_circ_005632 | ENSSSCG00000033626 | 12 | + | 60742768 | 60744004 | 758 | annot_exons |
| novel_circ_005633 | ENSSSCG00000039701 | 7 | - | 92778443 | 92785790 | 451 | annot_exons |
| novel_circ_005634 | ENSSSCG00000030378 | 3 | + | 11274006 | 11275965 | 223 | annot_exons |
| novel_circ_005635 | ENSSSCG00000017791 | 12 | - | 45856452 | 45887992 | 14548 | exon_intron |
| novel_circ_005636 | ENSSSCG00000001806 | 7 | + | 52165917 | 52166637 | 721 | exon_intron |
| novel_circ_005637 | ENSSSCG00000008404 | 3 | + | 85966091 | 85971244 | 650 | annot_exons |
| novel_circ_005638 | ENSSSCG00000003465 | 6 | + | 74955488 | 74960740 | 256 | annot_exons |
| novel_circ_005639 | ENSSSCG00000009994 | 14 | + | 46977920 | 46996102 | 574 | exon_intron |
| novel_circ_005640 | ENSSSCG00000026346 | 1 | + | 83295333 | 83295734 | 402 | one_exon |
| novel_circ_005641 | ENSSSCG00000014960 | 9 | + | 26899161 | 26905802 | 1087 | annot_exons |
| novel_circ_005642 | ENSSSCG00000031903 | 2 | + | 1253032 | 1253867 | 836 | intronic |
| novel_circ_005643 | ENSSSCG00000025941 | 1 | + | 45173524 | 45175843 | 774 | exon_intron |
| novel_circ_005644 | ENSSSCG00000003738 | 6 | + | 1.19E+08 | 1.19E+08 | 2030 | annot_exons |
| novel_circ_005645 | ENSSSCG00000011394 | 13 | + | 32466924 | 32506478 | 4307 | annot_exons |
| novel_circ_005646 | ENSSSCG00000004138 | 1 | + | 22281591 | 22352249 | 269 | annot_exons |
| novel_circ_005647 | ENSSSCG00000013566 | 2 | + | 71814867 | 71815418 | 552 | one_exon |
| novel_circ_005648 | NA | 15 | + | 1.21E+08 | 1.21E+08 | 3107 | intergenic |
| novel_circ_005649 | ENSSSCG00000008753 | 8 | - | 16700782 | 16718458 | 724 | annot_exons |
| novel_circ_005650 | ENSSSCG00000004254 | 1 | - | 44316951 | 44329819 | 394 | annot_exons |
| novel_circ_005651 | ENSSSCG00000003776 | 6 | - | 1.38E+08 | 1.38E+08 | 764 | annot_exons |
| novel_circ_005652 | ENSSSCG00000018031 | 12 | + | 59042473 | 59043128 | 214 | annot_exons |
| novel_circ_005653 | ENSSSCG00000008312 | 3 | - | 71177121 | 71179376 | 336 | exon_intron |
| novel_circ_005654 | ENSSSCG00000007023 | 17 | - | 11044273 | 11059120 | 879 | annot_exons |
| novel_circ_005655 | ENSSSCG00000006137 | 4 | - | 50388301 | 50388572 | 272 | one_exon |
| novel_circ_005656 | ENSSSCG00000001076 | 7 | + | 14052463 | 14052674 | 212 | one_exon |
| novel_circ_005657 | ENSSSCG00000010101 | 14 | + | 50628370 | 50629322 | 506 | annot_exons |
| novel_circ_005658 | ENSSSCG00000034795 | 2 | + | 87254645 | 87264182 | 497 | annot_exons |
| novel_circ_005659 | ENSSSCG00000034816 | 6 | + | 1.04E+08 | 1.04E+08 | 897 | annot_exons |
| novel_circ_005660 | ENSSSCG00000013367 | 2 | - | 40858690 | 40871492 | 801 | annot_exons |
| novel_circ_005661 | ENSSSCG00000026744 | 11 | - | 1355228 | 1371291 | 449 | annot_exons |
| novel_circ_005662 | ENSSSCG00000032357 | 13 | + | 39114289 | 39115035 | 747 | exon_intron |
| novel_circ_005663 | ENSSSCG00000011695 | 13 | - | 88979552 | 88979781 | 230 | antisense |
| novel_circ_005664 | ENSSSCG00000034730 | 1 | - | 96453516 | 96472730 | 607 | annot_exons |
| novel_circ_005665 | ENSSSCG00000036952 | 13 | - | 1.47E+08 | 1.47E+08 | 231 | annot_exons |
| novel_circ_005666 | ENSSSCG00000001700 | 7 | + | 39227689 | 39228358 | 446 | annot_exons |
| novel_circ_005667 | NA | 15 | - | 84421279 | 84422294 | 1016 | intergenic |
| novel_circ_005668 | ENSSSCG00000025179 | 5 | + | 63129448 | 63130832 | 363 | annot_exons |
| novel_circ_005669 | ENSSSCG00000009567 | 11 | - | 78957410 | 78962174 | 651 | annot_exons |
| novel_circ_005670 | ENSSSCG00000010143 | 14 | - | 54611059 | 54620212 | 588 | annot_exons |
| novel_circ_005671 | ENSSSCG00000014239 | 2 | - | 1.27E+08 | 1.27E+08 | 291 | annot_exons |
| novel_circ_005672 | ENSSSCG00000040184 | 11 | + | 48077009 | 48108814 | 3662 | annot_exons |
| novel_circ_005673 | ENSSSCG00000038323 | 2 | + | 5860528 | 5860843 | 316 | antisense |
| novel_circ_005674 | ENSSSCG00000005227 | 1 | - | 2.19E+08 | 2.19E+08 | 308 | antisense |
| novel_circ_005675 | ENSSSCG00000010190 | 14 | + | 60414154 | 60415477 | 1108 | annot_exons |
| novel_circ_005676 | ENSSSCG00000011395 | 13 | + | 32601373 | 32602572 | 337 | annot_exons |
| novel_circ_005677 | ENSSSCG00000010300 | 14 | - | 76250831 | 76251952 | 1122 | intronic |
| novel_circ_005678 | NA | 15 | - | 84466334 | 84466579 | 246 | intergenic |
| novel_circ_005679 | ENSSSCG00000038561 | 15 | - | 84415728 | 84417172 | 1355 | exon_intron |
| novel_circ_005680 | ENSSSCG00000001641 | 7 | + | 37704695 | 37712125 | 431 | annot_exons |
| novel_circ_005681 | ENSSSCG00000007544 | 3 | + | 504690 | 506331 | 347 | annot_exons |
| novel_circ_005682 | NA | 5 | + | 76899173 | 76899352 | 180 | intergenic |
| novel_circ_005683 | NA | 15 | - | 84268843 | 84269592 | 750 | intergenic |
| novel_circ_005684 | ENSSSCG00000034386 | 14 | + | 31719725 | 31719951 | 227 | one_exon |
| novel_circ_005685 | ENSSSCG00000007438 | 17 | - | 48239458 | 48241671 | 482 | annot_exons |
| novel_circ_005686 | ENSSSCG00000007478 | 17 | - | 52987380 | 53000592 | 630 | annot_exons |
| novel_circ_005687 | ENSSSCG00000017877 | 12 | - | 50185929 | 50187531 | 485 | annot_exons |
| novel_circ_005688 | ENSSSCG00000009473 | 11 | - | 49322689 | 49323027 | 254 | annot_exons |
| novel_circ_005689 | ENSSSCG00000002703 | 6 | - | 12014251 | 12017409 | 3159 | exon_intron |
| novel_circ_005690 | ENSSSCG00000011772 | 13 | + | 1.21E+08 | 1.21E+08 | 422 | annot_exons |
| novel_circ_005691 | ENSSSCG00000000458 | 5 | + | 27173898 | 27195997 | 1938 | annot_exons |
| novel_circ_005692 | ENSSSCG00000013278 | 2 | - | 17467170 | 17479643 | 1874 | annot_exons |
| novel_circ_005693 | ENSSSCG00000014794 | 9 | + | 6345348 | 6352989 | 591 | annot_exons |
| novel_circ_005694 | ENSSSCG00000014915 | 9 | + | 20133607 | 20139090 | 367 | annot_exons |
| novel_circ_005695 | ENSSSCG00000026239 | 6 | + | 37957475 | 37968385 | 1201 | annot_exons |
| novel_circ_005696 | ENSSSCG00000029662 | 14 | - | 91112603 | 91115019 | 393 | annot_exons |
| novel_circ_005697 | ENSSSCG00000005208 | 1 | - | 2.16E+08 | 2.16E+08 | 194 | annot_exons |
| novel_circ_005698 | ENSSSCG00000003743 | 6 | + | 1.19E+08 | 1.19E+08 | 5897 | exon_intron |
| novel_circ_005699 | ENSSSCG00000024663 | 13 | + | 1.46E+08 | 1.46E+08 | 354 | annot_exons |
| novel_circ_005700 | ENSSSCG00000006137 | 4 | - | 50421199 | 50422806 | 271 | annot_exons |
| novel_circ_005701 | ENSSSCG00000009172 | 8 | + | 1.2E+08 | 1.2E+08 | 719 | intronic |
| novel_circ_005702 | ENSSSCG00000022479 | 4 | + | 34707238 | 34743081 | 2922 | annot_exons |
| novel_circ_005703 | ENSSSCG00000016845 | 16 | - | 22567969 | 22572494 | 523 | annot_exons |
| novel_circ_005704 | ENSSSCG00000018049 | 12 | - | 60348042 | 60376920 | 1077 | annot_exons |
| novel_circ_005705 | NA | 15 | - | 84253469 | 84253697 | 229 | intergenic |
| novel_circ_005706 | ENSSSCG00000036052 | 15 | - | 84229858 | 84230103 | 246 | exon_intron |
| novel_circ_005707 | ENSSSCG00000023974 | 2 | + | 16347499 | 16348851 | 1353 | exon_intron |
| novel_circ_005708 | ENSSSCG00000028148 | X | - | 28468110 | 28486118 | 569 | annot_exons |
| novel_circ_005709 | ENSSSCG00000023924 | 15 | - | 87681624 | 87715704 | 692 | annot_exons |
| novel_circ_005710 | ENSSSCG00000010053 | 14 | + | 49359963 | 49380521 | 2186 | annot_exons |
| novel_circ_005711 | ENSSSCG00000010250 | 14 | + | 72229179 | 72231451 | 272 | annot_exons |
| novel_circ_005712 | ENSSSCG00000003627 | 6 | + | 91554750 | 91555248 | 388 | annot_exons |
| novel_circ_005713 | ENSSSCG00000008079 | 3 | + | 42881838 | 42902838 | 787 | exon_intron |
| novel_circ_005714 | NA | 15 | - | 84233200 | 84233762 | 563 | intergenic |
| novel_circ_005715 | NA | 15 | + | 84263724 | 84263963 | 240 | intergenic |
| novel_circ_005716 | ENSSSCG00000006179 | 4 | + | 62146811 | 62187826 | 693 | annot_exons |
| novel_circ_005717 | ENSSSCG00000001497 | 7 | - | 28695782 | 28735707 | 6016 | annot_exons |
| novel_circ_005718 | ENSSSCG00000017877 | 12 | - | 50194815 | 50197791 | 474 | annot_exons |
| novel_circ_005719 | ENSSSCG00000008614 | 3 | + | 1.2E+08 | 1.2E+08 | 481 | annot_exons |
| novel_circ_005720 | ENSSSCG00000000939 | 5 | - | 1E+08 | 1E+08 | 318 | annot_exons |
| novel_circ_005721 | ENSSSCG00000006885 | 4 | + | 1.22E+08 | 1.22E+08 | 305 | exon_intron |
| novel_circ_005722 | ENSSSCG00000009061 | 8 | - | 87643220 | 87644581 | 282 | annot_exons |
| novel_circ_005723 | ENSSSCG00000009142 | 8 | - | 1.13E+08 | 1.13E+08 | 409 | annot_exons |
| novel_circ_005724 | ENSSSCG00000004065 | 1 | - | 11731352 | 11735531 | 405 | annot_exons |
| novel_circ_005725 | ENSSSCG00000015892 | 15 | + | 68163251 | 68167528 | 414 | annot_exons |
| novel_circ_005726 | ENSSSCG00000033673 | AEMK02000569.1 | + | 793120 | 793325 | 206 | antisense |
| novel_circ_005727 | ENSSSCG00000011022 | 10 | + | 41202411 | 41257277 | 26806 | exon_intron |
| novel_circ_005728 | NA | 15 | - | 84387944 | 84388260 | 317 | intergenic |
| novel_circ_005729 | ENSSSCG00000015861 | 15 | - | 59583567 | 59583942 | 376 | antisense |
| novel_circ_005730 | NA | 15 | - | 84426382 | 84426633 | 252 | intergenic |
| novel_circ_005731 | ENSSSCG00000011833 | 13 | + | 1.33E+08 | 1.33E+08 | 11820 | exon_intron |
| novel_circ_005732 | ENSSSCG00000014048 | 2 | + | 80985215 | 80985664 | 450 | exon_intron |
| novel_circ_005733 | ENSSSCG00000015462 | 9 | + | 1.13E+08 | 1.13E+08 | 316 | annot_exons |
| novel_circ_005734 | ENSSSCG00000001862 | 7 | - | 56518062 | 56519340 | 211 | annot_exons |
| novel_circ_005735 | ENSSSCG00000021042 | 13 | - | 2.03E+08 | 2.03E+08 | 633 | annot_exons |
| novel_circ_005736 | ENSSSCG00000011676 | 13 | + | 82698276 | 82713285 | 1867 | exon_intron |
| novel_circ_005737 | ENSSSCG00000009061 | 8 | - | 87645971 | 87651892 | 446 | annot_exons |
| novel_circ_005738 | ENSSSCG00000005459 | 1 | - | 2.52E+08 | 2.52E+08 | 897 | annot_exons |
| novel_circ_005739 | ENSSSCG00000026665 | 12 | - | 37509450 | 37517080 | 354 | exon_intron |
| novel_circ_005740 | NA | 15 | - | 84493675 | 84494934 | 1260 | intergenic |
| novel_circ_005741 | ENSSSCG00000048856 | 2 | - | 6754918 | 6755278 | 361 | one_exon |
| novel_circ_005742 | ENSSSCG00000011394 | 13 | + | 32466924 | 32541480 | 4382 | annot_exons |
| novel_circ_005743 | NA | 15 | + | 84259928 | 84260542 | 615 | intergenic |
| novel_circ_005744 | ENSSSCG00000039867 | 4 | - | 84860816 | 84861880 | 257 | annot_exons |
| novel_circ_005745 | ENSSSCG00000024232 | 5 | - | 45342547 | 45403216 | 548 | annot_exons |
| novel_circ_005746 | ENSSSCG00000001065 | 7 | - | 12653261 | 12653449 | 189 | exon_intron |
| novel_circ_005747 | ENSSSCG00000008513 | 3 | - | 1.07E+08 | 1.07E+08 | 401 | annot_exons |
| novel_circ_005748 | ENSSSCG00000030291 | 4 | - | 98669435 | 98669610 | 176 | one_exon |
| novel_circ_005749 | ENSSSCG00000004958 | 1 | + | 1.66E+08 | 1.66E+08 | 312 | annot_exons |
| novel_circ_005750 | ENSSSCG00000006873 | 4 | + | 1.18E+08 | 1.18E+08 | 692 | annot_exons |
| novel_circ_005751 | ENSSSCG00000010289 | 14 | - | 75288977 | 75349065 | 458 | annot_exons |
| novel_circ_005752 | ENSSSCG00000015402 | 9 | + | 98019785 | 98070939 | 232 | annot_exons |
| novel_circ_005753 | ENSSSCG00000016911 | 16 | + | 34555976 | 34575367 | 593 | annot_exons |
| novel_circ_005754 | ENSSSCG00000027039 | 18 | - | 8057485 | 8086118 | 28634 | exon_intron |
| novel_circ_005755 | ENSSSCG00000032223 | X | + | 16433010 | 16433260 | 251 | antisense |
| novel_circ_005756 | ENSSSCG00000027935 | 6 | + | 1.2E+08 | 1.2E+08 | 401 | annot_exons |
| novel_circ_005757 | ENSSSCG00000026153 | 9 | + | 36537603 | 36538671 | 247 | annot_exons |
| novel_circ_005758 | ENSSSCG00000004589 | 1 | - | 1.13E+08 | 1.13E+08 | 896 | one_exon |
| novel_circ_005759 | ENSSSCG00000008829 | 8 | + | 38761016 | 38765908 | 4893 | antisense |
| novel_circ_005760 | NA | 15 | + | 84310488 | 84310833 | 346 | intergenic |
| novel_circ_005761 | ENSSSCG00000011845 | 13 | + | 1.34E+08 | 1.34E+08 | 374 | annot_exons |
| novel_circ_005762 | ENSSSCG00000006303 | 4 | - | 82990344 | 83008862 | 928 | annot_exons |
| novel_circ_005763 | ENSSSCG00000011194 | 13 | - | 2931438 | 2933716 | 484 | annot_exons |
| novel_circ_005764 | ENSSSCG00000000612 | 5 | - | 58050540 | 58054445 | 802 | annot_exons |
| novel_circ_005765 | ENSSSCG00000025164 | 3 | - | 8391173 | 8393713 | 562 | annot_exons |
| novel_circ_005766 | ENSSSCG00000007812 | 3 | + | 18826737 | 18830614 | 581 | annot_exons |
| novel_circ_005767 | ENSSSCG00000009956 | 14 | + | 43436685 | 43437447 | 763 | exon_intron |
| novel_circ_005768 | ENSSSCG00000010603 | 14 | + | 1.14E+08 | 1.14E+08 | 564 | annot_exons |
| novel_circ_005769 | ENSSSCG00000001064 | 7 | + | 12261370 | 12266794 | 378 | annot_exons |
| novel_circ_005770 | ENSSSCG00000006137 | 4 | - | 50395149 | 50403299 | 537 | annot_exons |
| novel_circ_005771 | ENSSSCG00000006508 | 4 | + | 94324418 | 94324938 | 521 | exon_intron |
| novel_circ_005772 | ENSSSCG00000025602 | 18 | + | 33520610 | 33520821 | 212 | intronic |
| novel_circ_005773 | ENSSSCG00000025260 | 5 | + | 10302842 | 10303033 | 192 | one_exon |
| novel_circ_005774 | NA | 2 | - | 1.13E+08 | 1.13E+08 | 3622 | intergenic |
| novel_circ_005775 | ENSSSCG00000002684 | 6 | - | 5544365 | 5551572 | 7208 | intronic |
| novel_circ_005776 | ENSSSCG00000016157 | 15 | - | 1.13E+08 | 1.13E+08 | 252 | annot_exons |
| novel_circ_005777 | ENSSSCG00000031954 | 15 | + | 99680180 | 99703874 | 567 | annot_exons |
| novel_circ_005778 | ENSSSCG00000010029 | 14 | + | 48134655 | 48143955 | 662 | annot_exons |
| novel_circ_005779 | ENSSSCG00000003776 | 6 | - | 1.38E+08 | 1.38E+08 | 659 | annot_exons |
| novel_circ_005780 | ENSSSCG00000015917 | 15 | + | 73728614 | 73728837 | 224 | one_exon |
| novel_circ_005781 | ENSSSCG00000024814 | X | - | 47820725 | 47826089 | 362 | annot_exons |
| novel_circ_005782 | ENSSSCG00000042985 | 6 | + | 47424162 | 47424687 | 526 | intronic |
| novel_circ_005783 | ENSSSCG00000016848 | 16 | + | 23489309 | 23490835 | 254 | annot_exons |
| novel_circ_005784 | ENSSSCG00000006864 | 4 | - | 1.18E+08 | 1.18E+08 | 3902 | exon_intron |
| novel_circ_005785 | ENSSSCG00000006648 | 4 | + | 98417883 | 98429133 | 1042 | exon_intron |
| novel_circ_005786 | ENSSSCG00000008641 | 3 | + | 1.27E+08 | 1.27E+08 | 210 | annot_exons |
| novel_circ_005787 | ENSSSCG00000031710 | AEMK02000452.1 | - | 3325019 | 3327814 | 504 | annot_exons |
| novel_circ_005788 | ENSSSCG00000002266 | 7 | - | 86064996 | 86070457 | 685 | annot_exons |
| novel_circ_005789 | ENSSSCG00000032531 | 3 | + | 26691921 | 26693748 | 272 | annot_exons |
| novel_circ_005790 | ENSSSCG00000038607 | 13 | - | 17012278 | 17025247 | 5206 | annot_exons |
| novel_circ_005791 | ENSSSCG00000003218 | 6 | + | 55302098 | 55304287 | 725 | annot_exons |
| novel_circ_005792 | ENSSSCG00000022895 | 13 | + | 1.62E+08 | 1.62E+08 | 296 | antisense |
| novel_circ_005793 | ENSSSCG00000009473 | 11 | - | 49203468 | 49216889 | 941 | annot_exons |
| novel_circ_005794 | ENSSSCG00000016109 | 15 | - | 1.05E+08 | 1.05E+08 | 444 | annot_exons |
| novel_circ_005795 | ENSSSCG00000035119 | 11 | - | 3193839 | 3199769 | 636 | annot_exons |
| novel_circ_005796 | ENSSSCG00000022788 | 8 | + | 1.16E+08 | 1.16E+08 | 214 | annot_exons |
| novel_circ_005797 | ENSSSCG00000006284 | 4 | - | 81196423 | 81203653 | 285 | annot_exons |
| novel_circ_005798 | ENSSSCG00000017826 | 12 | - | 48369268 | 48369964 | 324 | annot_exons |
| novel_circ_005799 | ENSSSCG00000036145 | 6 | - | 1.56E+08 | 1.56E+08 | 632 | annot_exons |
| novel_circ_005800 | ENSSSCG00000017272 | 12 | - | 13501057 | 13501309 | 166 | annot_exons |
| novel_circ_005801 | ENSSSCG00000035901 | 14 | + | 249923 | 287094 | 888 | annot_exons |
| novel_circ_005802 | ENSSSCG00000016834 | 16 | + | 21416103 | 21416361 | 259 | antisense |
| novel_circ_005803 | ENSSSCG00000003512 | 6 | - | 79106734 | 79108480 | 254 | annot_exons |
| novel_circ_005804 | ENSSSCG00000005660 | 1 | + | 2.69E+08 | 2.69E+08 | 439 | annot_exons |
| novel_circ_005805 | ENSSSCG00000015052 | 9 | - | 41453661 | 41464576 | 477 | annot_exons |
| novel_circ_005806 | NA | 8 | - | 3964585 | 3976580 | 11996 | intergenic |
| novel_circ_005807 | ENSSSCG00000028035 | 9 | - | 64522414 | 64522602 | 189 | antisense |
| novel_circ_005808 | ENSSSCG00000007512 | 17 | - | 58454182 | 58468153 | 1030 | exon_intron |
| novel_circ_005809 | ENSSSCG00000013460 | 2 | - | 76294269 | 76294972 | 319 | annot_exons |
| novel_circ_005810 | ENSSSCG00000006987 | 17 | + | 5251578 | 5253074 | 300 | annot_exons |
| novel_circ_005811 | ENSSSCG00000004138 | 1 | + | 22274446 | 22352249 | 7414 | exon_intron |
| novel_circ_005812 | NA | 6 | + | 47452513 | 47455183 | 2671 | intergenic |
| novel_circ_005813 | NA | 14 | - | 12913616 | 12935855 | 22240 | intergenic |
| novel_circ_005814 | ENSSSCG00000010295 | 14 | - | 75974900 | 75978154 | 589 | annot_exons |
| novel_circ_005815 | ENSSSCG00000000612 | 5 | - | 58032630 | 58038309 | 418 | annot_exons |
| novel_circ_005816 | ENSSSCG00000003493 | 6 | - | 77853446 | 77853943 | 498 | exon_intron |
| novel_circ_005817 | ENSSSCG00000041187 | 10 | + | 12547679 | 12551049 | 979 | annot_exons |
| novel_circ_005818 | ENSSSCG00000006553 | 4 | - | 95503981 | 95515576 | 767 | annot_exons |
| novel_circ_005819 | ENSSSCG00000009134 | 8 | - | 1.12E+08 | 1.12E+08 | 268 | annot_exons |
| novel_circ_005820 | ENSSSCG00000015592 | 9 | - | 1.31E+08 | 1.31E+08 | 642 | annot_exons |
| novel_circ_005821 | ENSSSCG00000030386 | 18 | - | 13756151 | 13761629 | 341 | annot_exons |
| novel_circ_005822 | ENSSSCG00000015795 | 15 | - | 46516897 | 46518469 | 323 | exon_intron |
| novel_circ_005823 | ENSSSCG00000004303 | 1 | - | 55937066 | 55954186 | 502 | annot_exons |
| novel_circ_005824 | ENSSSCG00000015599 | 9 | - | 1.31E+08 | 1.31E+08 | 299 | annot_exons |
| novel_circ_005825 | ENSSSCG00000047420 | 17 | - | 38986014 | 38991291 | 1902 | annot_exons |
| novel_circ_005826 | ENSSSCG00000024373 | 15 | - | 1.31E+08 | 1.31E+08 | 1354 | annot_exons |
| novel_circ_005827 | ENSSSCG00000009631 | 14 | - | 7085604 | 7165819 | 73493 | exon_intron |
| novel_circ_005828 | ENSSSCG00000008136 | 3 | - | 47549284 | 47550135 | 264 | annot_exons |
| novel_circ_005829 | ENSSSCG00000008591 | 3 | + | 1.15E+08 | 1.15E+08 | 366 | annot_exons |
| novel_circ_005830 | ENSSSCG00000010811 | 10 | + | 5788969 | 5806269 | 648 | annot_exons |
| novel_circ_005831 | ENSSSCG00000012251 | X | + | 36792047 | 36805365 | 3254 | annot_exons |
| novel_circ_005832 | ENSSSCG00000015558 | 9 | + | 1.25E+08 | 1.25E+08 | 607 | annot_exons |
| novel_circ_005833 | ENSSSCG00000016114 | 15 | + | 1.06E+08 | 1.06E+08 | 491 | annot_exons |
| novel_circ_005834 | ENSSSCG00000029920 | 8 | + | 87799386 | 87828391 | 8525 | exon_intron |
| novel_circ_005835 | ENSSSCG00000012742 | X | + | 1.22E+08 | 1.22E+08 | 454 | annot_exons |
| novel_circ_005836 | ENSSSCG00000025876 | 14 | - | 71443510 | 71451475 | 3266 | annot_exons |
| novel_circ_005837 | ENSSSCG00000000866 | 5 | - | 82561533 | 82561858 | 326 | one_exon |
| novel_circ_005838 | ENSSSCG00000010298 | 14 | - | 76106931 | 76107894 | 275 | annot_exons |
| novel_circ_005839 | ENSSSCG00000016125 | 15 | - | 1.09E+08 | 1.09E+08 | 1667 | annot_exons |
| novel_circ_005840 | ENSSSCG00000010952 | 10 | + | 29033187 | 29035802 | 162 | annot_exons |
| novel_circ_005841 | ENSSSCG00000001646 | 7 | + | 37902522 | 37904204 | 1683 | one_exon |
| novel_circ_005842 | ENSSSCG00000025298 | 9 | - | 88762439 | 88764377 | 633 | annot_exons |
| novel_circ_005843 | ENSSSCG00000014887 | 9 | - | 12514001 | 12516453 | 294 | annot_exons |
| novel_circ_005844 | ENSSSCG00000013978 | 2 | - | 52301793 | 52308258 | 4986 | exon_intron |
| novel_circ_005845 | ENSSSCG00000008832 | 8 | + | 39128332 | 39129054 | 723 | antisense |
| novel_circ_005846 | ENSSSCG00000016127 | 15 | - | 1.09E+08 | 1.09E+08 | 1215 | annot_exons |
| novel_circ_005847 | ENSSSCG00000033692 | 14 | + | 55783529 | 55807478 | 664 | annot_exons |
| novel_circ_005848 | ENSSSCG00000011213 | 13 | - | 12528540 | 12529463 | 336 | annot_exons |
| novel_circ_005849 | ENSSSCG00000004717 | 1 | + | 1.28E+08 | 1.28E+08 | 944 | annot_exons |
| novel_circ_005850 | ENSSSCG00000016083 | 15 | + | 1.02E+08 | 1.02E+08 | 7684 | exon_intron |
| novel_circ_005851 | ENSSSCG00000016117 | 15 | + | 1.06E+08 | 1.06E+08 | 685 | annot_exons |
| novel_circ_005852 | ENSSSCG00000026714 | X | - | 62209903 | 62210204 | 302 | intronic |
| novel_circ_005853 | ENSSSCG00000011163 | 10 | + | 69084072 | 69120803 | 1036 | annot_exons |
| novel_circ_005854 | ENSSSCG00000016005 | 15 | - | 84806578 | 84812231 | 366 | annot_exons |
| novel_circ_005855 | ENSSSCG00000011612 | 13 | + | 71897446 | 71897752 | 307 | one_exon |
| novel_circ_005856 | ENSSSCG00000017717 | 12 | - | 40021670 | 40022545 | 337 | annot_exons |
| novel_circ_005857 | ENSSSCG00000014126 | 2 | + | 89270175 | 89278885 | 550 | annot_exons |
| novel_circ_005858 | ENSSSCG00000004029 | 1 | - | 4775755 | 4777265 | 1511 | exon_intron |
| novel_circ_005859 | ENSSSCG00000009994 | 14 | + | 46957994 | 46985309 | 627 | annot_exons |
| novel_circ_005860 | ENSSSCG00000013410 | 2 | - | 49745189 | 49747348 | 375 | annot_exons |
| novel_circ_005861 | ENSSSCG00000014207 | 2 | - | 1.17E+08 | 1.17E+08 | 963 | annot_exons |
| novel_circ_005862 | ENSSSCG00000002869 | 6 | + | 43887219 | 43887734 | 243 | annot_exons |
| novel_circ_005863 | ENSSSCG00000016640 | 18 | - | 32035277 | 32035769 | 493 | antisense |
| novel_circ_005864 | ENSSSCG00000015569 | 9 | + | 1.26E+08 | 1.26E+08 | 749 | annot_exons |
| novel_circ_005865 | NA | 14 | - | 41639323 | 41672257 | 32935 | intergenic |
| novel_circ_005866 | ENSSSCG00000003218 | 6 | + | 55302315 | 55304287 | 508 | annot_exons |
| novel_circ_005867 | ENSSSCG00000034357 | 13 | + | 32070527 | 32070917 | 391 | one_exon |
| novel_circ_005868 | ENSSSCG00000016291 | 15 | + | 1.33E+08 | 1.33E+08 | 301 | annot_exons |
| novel_circ_005869 | ENSSSCG00000030528 | 13 | - | 1.92E+08 | 1.92E+08 | 527 | annot_exons |
| novel_circ_005870 | ENSSSCG00000001925 | 7 | + | 60592097 | 60602546 | 289 | annot_exons |
| novel_circ_005871 | ENSSSCG00000007737 | 3 | - | 16454327 | 16487913 | 1145 | annot_exons |
| novel_circ_005872 | ENSSSCG00000008452 | 3 | + | 96497331 | 96506776 | 751 | annot_exons |
| novel_circ_005873 | ENSSSCG00000006831 | 4 | + | 1.11E+08 | 1.11E+08 | 498 | annot_exons |
| novel_circ_005874 | ENSSSCG00000000939 | 5 | - | 1E+08 | 1E+08 | 621 | annot_exons |
| novel_circ_005875 | ENSSSCG00000030480 | 13 | + | 2.01E+08 | 2.01E+08 | 282 | annot_exons |
| novel_circ_005876 | ENSSSCG00000014331 | 2 | + | 1.4E+08 | 1.4E+08 | 437 | annot_exons |
| novel_circ_005877 | ENSSSCG00000017069 | 16 | - | 68406583 | 68407045 | 345 | annot_exons |
| novel_circ_005878 | ENSSSCG00000000694 | 5 | - | 64129708 | 64129952 | 245 | one_exon |
| novel_circ_005879 | ENSSSCG00000006274 | 4 | + | 79750564 | 79753708 | 389 | annot_exons |
| novel_circ_005880 | ENSSSCG00000016008 | 15 | - | 85583415 | 85588329 | 203 | annot_exons |
| novel_circ_005881 | ENSSSCG00000040388 | 17 | - | 62677113 | 62688878 | 1252 | annot_exons |
| novel_circ_005882 | ENSSSCG00000001970 | 7 | + | 68239408 | 68242211 | 472 | exon_intron |
| novel_circ_005883 | ENSSSCG00000007606 | 3 | + | 5928738 | 5934175 | 592 | annot_exons |
| novel_circ_005884 | ENSSSCG00000010579 | 14 | - | 1.13E+08 | 1.13E+08 | 16122 | antisense |
| novel_circ_005885 | ENSSSCG00000011629 | 13 | - | 73736299 | 73737630 | 227 | annot_exons |
| novel_circ_005886 | ENSSSCG00000006000 | 4 | + | 19228775 | 19232531 | 446 | annot_exons |
| novel_circ_005887 | ENSSSCG00000015509 | 9 | - | 1.18E+08 | 1.18E+08 | 870 | annot_exons |
| novel_circ_005888 | ENSSSCG00000010703 | 14 | + | 1.32E+08 | 1.32E+08 | 343 | annot_exons |
| novel_circ_005889 | ENSSSCG00000029030 | 6 | - | 1.04E+08 | 1.04E+08 | 343 | annot_exons |
| novel_circ_005890 | ENSSSCG00000016397 | 15 | + | 622250 | 626964 | 729 | annot_exons |
| novel_circ_005891 | ENSSSCG00000014331 | 2 | + | 1.4E+08 | 1.4E+08 | 220 | annot_exons |
| novel_circ_005892 | ENSSSCG00000022159 | 11 | - | 18594295 | 18604222 | 714 | annot_exons |
| novel_circ_005893 | ENSSSCG00000016192 | 15 | + | 1.21E+08 | 1.21E+08 | 23913 | exon_intron |
| novel_circ_005894 | ENSSSCG00000011194 | 13 | - | 2970768 | 2983349 | 645 | annot_exons |
| novel_circ_005895 | ENSSSCG00000030343 | 16 | - | 34791656 | 34810715 | 641 | annot_exons |
| novel_circ_005896 | ENSSSCG00000002505 | 7 | + | 1.18E+08 | 1.18E+08 | 587 | annot_exons |
| novel_circ_005897 | ENSSSCG00000002436 | 7 | - | 1.12E+08 | 1.12E+08 | 410 | annot_exons |
| novel_circ_005898 | ENSSSCG00000000160 | 5 | + | 12654723 | 12656405 | 269 | annot_exons |
| novel_circ_005899 | ENSSSCG00000009957 | 14 | + | 43569251 | 43569748 | 235 | annot_exons |
| novel_circ_005900 | NA | 15 | + | 84273632 | 84273795 | 164 | intergenic |
| novel_circ_005901 | ENSSSCG00000039793 | 6 | - | 1.51E+08 | 1.51E+08 | 465 | annot_exons |
| novel_circ_005902 | ENSSSCG00000002306 | 7 | + | 93032720 | 93033643 | 265 | annot_exons |
| novel_circ_005903 | ENSSSCG00000038913 | 14 | - | 71145355 | 71166358 | 2453 | annot_exons |
| novel_circ_005904 | ENSSSCG00000034484 | 6 | + | 75086627 | 75092311 | 455 | annot_exons |
| novel_circ_005905 | ENSSSCG00000021814 | 11 | + | 685277 | 686516 | 379 | annot_exons |
| novel_circ_005906 | ENSSSCG00000014868 | 9 | + | 10684937 | 10699154 | 675 | annot_exons |
| novel_circ_005907 | ENSSSCG00000004538 | 1 | + | 1.06E+08 | 1.06E+08 | 351 | annot_exons |
| novel_circ_005908 | ENSSSCG00000011162 | 10 | + | 68690561 | 68692165 | 375 | annot_exons |
| novel_circ_005909 | ENSSSCG00000001871 | 7 | + | 57548768 | 57554593 | 494 | annot_exons |
| novel_circ_005910 | ENSSSCG00000000806 | 5 | - | 76844512 | 76857881 | 503 | annot_exons |
| novel_circ_005911 | ENSSSCG00000009793 | 14 | + | 30407230 | 30408174 | 945 | one_exon |
| novel_circ_005912 | ENSSSCG00000005465 | 1 | - | 2.53E+08 | 2.53E+08 | 472 | annot_exons |
| novel_circ_005913 | ENSSSCG00000015861 | 15 | + | 59564532 | 59567946 | 270 | annot_exons |
| novel_circ_005914 | ENSSSCG00000012699 | X | + | 1.11E+08 | 1.11E+08 | 697 | one_exon |
| novel_circ_005915 | ENSSSCG00000008787 | 8 | + | 30396357 | 30401155 | 517 | annot_exons |
| novel_circ_005916 | ENSSSCG00000007454 | 17 | - | 49312897 | 49313377 | 479 | annot_exons |
| novel_circ_005917 | ENSSSCG00000026705 | 10 | - | 12595562 | 12631297 | 4996 | annot_exons |
| novel_circ_005918 | ENSSSCG00000013402 | 2 | - | 47525392 | 47530845 | 457 | annot_exons |
| novel_circ_005919 | ENSSSCG00000000864 | 5 | - | 82507118 | 82515818 | 502 | annot_exons |
| novel_circ_005920 | ENSSSCG00000016501 | 18 | + | 9651790 | 9653711 | 456 | annot_exons |
| novel_circ_005921 | ENSSSCG00000017551 | 12 | - | 25899204 | 25902053 | 448 | annot_exons |
| novel_circ_005922 | NA | 13 | - | 61462025 | 61488274 | 26250 | intergenic |
| novel_circ_005923 | ENSSSCG00000005667 | 1 | + | 2.69E+08 | 2.69E+08 | 376 | annot_exons |
| novel_circ_005924 | ENSSSCG00000029723 | 2 | + | 343415 | 344651 | 1237 | exon_intron |
| novel_circ_005925 | ENSSSCG00000014126 | 2 | + | 89312694 | 89350868 | 556 | annot_exons |
| novel_circ_005926 | ENSSSCG00000008624 | 3 | - | 1.25E+08 | 1.25E+08 | 2624 | annot_exons |
| novel_circ_005927 | ENSSSCG00000032266 | 18 | + | 9400686 | 9421492 | 690 | annot_exons |
| novel_circ_005928 | NA | 9 | + | 1.22E+08 | 1.22E+08 | 3149 | intergenic |
| novel_circ_005929 | ENSSSCG00000001080 | 7 | + | 15922699 | 16013622 | 668 | annot_exons |
| novel_circ_005930 | ENSSSCG00000051039 | 17 | + | 8466081 | 8469366 | 3286 | antisense |
| novel_circ_005931 | ENSSSCG00000000233 | 5 | + | 17338840 | 17341509 | 450 | annot_exons |
| novel_circ_005932 | ENSSSCG00000039896 | 1 | + | 2.47E+08 | 2.47E+08 | 391 | annot_exons |
| novel_circ_005933 | ENSSSCG00000004626 | 1 | - | 1.2E+08 | 1.2E+08 | 741 | annot_exons |
| novel_circ_005934 | NA | 7 | + | 68160992 | 68185517 | 24526 | intergenic |
| novel_circ_005935 | ENSSSCG00000013399 | 2 | - | 46647129 | 46647372 | 244 | intronic |
| novel_circ_005936 | ENSSSCG00000000571 | 5 | + | 51607457 | 51612041 | 268 | annot_exons |
| novel_circ_005937 | ENSSSCG00000016976 | 16 | - | 48847946 | 48876101 | 26860 | exon_intron |
| novel_circ_005938 | ENSSSCG00000005240 | 1 | - | 2.21E+08 | 2.21E+08 | 1324 | annot_exons |
| novel_circ_005939 | ENSSSCG00000022164 | 13 | - | 2.07E+08 | 2.07E+08 | 231 | annot_exons |
| novel_circ_005940 | ENSSSCG00000029578 | 6 | + | 28736919 | 28767831 | 1705 | annot_exons |
| novel_circ_005941 | ENSSSCG00000008068 | 3 | - | 41952796 | 41962459 | 649 | annot_exons |
| novel_circ_005942 | ENSSSCG00000023112 | 8 | - | 31020866 | 31025401 | 316 | annot_exons |
| novel_circ_005943 | ENSSSCG00000014902 | 9 | + | 17351072 | 17398149 | 1098 | annot_exons |
| novel_circ_005944 | ENSSSCG00000017139 | 12 | - | 1838275 | 1922863 | 668 | annot_exons |
| novel_circ_005945 | ENSSSCG00000006179 | 4 | + | 62243622 | 62259943 | 644 | annot_exons |
| novel_circ_005946 | ENSSSCG00000004165 | 1 | - | 27998868 | 28008324 | 545 | annot_exons |
| novel_circ_005947 | ENSSSCG00000006194 | 4 | + | 65233835 | 65235030 | 809 | annot_exons |
| novel_circ_005948 | ENSSSCG00000016127 | 15 | - | 1.09E+08 | 1.09E+08 | 208 | annot_exons |
| novel_circ_005949 | ENSSSCG00000016330 | 15 | + | 1.37E+08 | 1.37E+08 | 198 | annot_exons |
| novel_circ_005950 | ENSSSCG00000011013 | 10 | + | 40015293 | 40018911 | 434 | annot_exons |
| novel_circ_005951 | ENSSSCG00000012742 | X | + | 1.22E+08 | 1.22E+08 | 213 | annot_exons |
| novel_circ_005952 | ENSSSCG00000013883 | 2 | - | 60151406 | 60152648 | 340 | annot_exons |
| novel_circ_005953 | ENSSSCG00000038619 | 6 | - | 83434350 | 83434927 | 211 | annot_exons |
| novel_circ_005954 | ENSSSCG00000002029 | 7 | + | 75666325 | 75695781 | 8122 | annot_exons |
| novel_circ_005955 | ENSSSCG00000006231 | 4 | - | 72630918 | 72637790 | 260 | annot_exons |
| novel_circ_005956 | ENSSSCG00000035824 | 13 | - | 34726535 | 34727649 | 422 | annot_exons |
| novel_circ_005957 | ENSSSCG00000009972 | 14 | + | 46300103 | 46304260 | 279 | annot_exons |
| novel_circ_005958 | ENSSSCG00000006762 | 4 | + | 1.07E+08 | 1.07E+08 | 490 | annot_exons |
| novel_circ_005959 | ENSSSCG00000001868 | 7 | - | 57171597 | 57173633 | 369 | annot_exons |
| novel_circ_005960 | ENSSSCG00000026110 | 9 | - | 1.05E+08 | 1.05E+08 | 692 | annot_exons |
| novel_circ_005961 | ENSSSCG00000011085 | 10 | - | 53169687 | 53202893 | 390 | annot_exons |
| novel_circ_005962 | NA | 15 | + | 84466054 | 84466939 | 886 | intergenic |
| novel_circ_005963 | ENSSSCG00000002285 | 7 | + | 90811957 | 90848269 | 604 | annot_exons |
| novel_circ_005964 | ENSSSCG00000028506 | 6 | + | 1.58E+08 | 1.58E+08 | 467 | annot_exons |
| novel_circ_005965 | ENSSSCG00000021731 | 15 | + | 44768145 | 44769374 | 298 | annot_exons |
| novel_circ_005966 | ENSSSCG00000021941 | 3 | + | 7928957 | 7932649 | 3450 | exon_intron |
| novel_circ_005967 | ENSSSCG00000000078 | 5 | - | 8070405 | 8073592 | 321 | annot_exons |
| novel_circ_005968 | ENSSSCG00000030947 | 4 | + | 7094328 | 7116661 | 501 | annot_exons |
| novel_circ_005969 | ENSSSCG00000002540 | AEMK02000452.1 | + | 745432 | 773915 | 758 | annot_exons |
| novel_circ_005970 | ENSSSCG00000028720 | 12 | + | 47069504 | 47071451 | 305 | annot_exons |
| novel_circ_005971 | ENSSSCG00000029803 | 14 | + | 31091335 | 31091801 | 286 | annot_exons |
| novel_circ_005972 | ENSSSCG00000005170 | 1 | - | 2.04E+08 | 2.04E+08 | 219 | one_exon |
| novel_circ_005973 | ENSSSCG00000017104 | 16 | + | 75354076 | 75355507 | 206 | annot_exons |
| novel_circ_005974 | ENSSSCG00000014960 | 9 | + | 26931084 | 26962087 | 722 | annot_exons |
| novel_circ_005975 | ENSSSCG00000009758 | 14 | + | 28214230 | 28219484 | 597 | annot_exons |
| novel_circ_005976 | ENSSSCG00000005095 | 1 | + | 1.9E+08 | 1.9E+08 | 601 | annot_exons |
| novel_circ_005977 | ENSSSCG00000026697 | 5 | + | 84007999 | 84015236 | 1954 | annot_exons |
| novel_circ_005978 | ENSSSCG00000027403 | 8 | - | 55619378 | 55620179 | 802 | one_exon |
| novel_circ_005979 | NA | 2 | - | 1.31E+08 | 1.31E+08 | 26706 | intergenic |
| novel_circ_005980 | ENSSSCG00000009472 | 11 | - | 49142293 | 49145731 | 475 | annot_exons |
| novel_circ_005981 | ENSSSCG00000042225 | 8 | - | 1.21E+08 | 1.21E+08 | 245 | antisense |
| novel_circ_005982 | ENSSSCG00000009793 | 14 | + | 30407230 | 30413591 | 1120 | annot_exons |
| novel_circ_005983 | ENSSSCG00000015940 | 15 | + | 76192264 | 76198564 | 427 | annot_exons |
| novel_circ_005984 | ENSSSCG00000005969 | 4 | + | 14614916 | 14629761 | 567 | annot_exons |
| novel_circ_005985 | ENSSSCG00000015931 | 15 | + | 75893150 | 75893273 | 124 | one_exon |
| novel_circ_005986 | ENSSSCG00000024623 | 13 | - | 1.81E+08 | 1.81E+08 | 7885 | antisense |
| novel_circ_005987 | ENSSSCG00000006267 | 4 | + | 78124832 | 78139488 | 534 | annot_exons |
| novel_circ_005988 | ENSSSCG00000016842 | 16 | + | 22318006 | 22319938 | 564 | annot_exons |
| novel_circ_005989 | ENSSSCG00000023596 | 14 | - | 61002771 | 61016166 | 588 | annot_exons |
| novel_circ_005990 | ENSSSCG00000009071 | 8 | - | 96682956 | 96687491 | 3919 | exon_intron |
| novel_circ_005991 | ENSSSCG00000006037 | 4 | - | 30808728 | 30818482 | 305 | annot_exons |
| novel_circ_005992 | ENSSSCG00000015140 | 9 | - | 49984887 | 49985330 | 226 | annot_exons |
| novel_circ_005993 | ENSSSCG00000017091 | 16 | + | 71947633 | 71952290 | 474 | annot_exons |
| novel_circ_005994 | ENSSSCG00000028113 | 18 | - | 29316100 | 29316509 | 410 | one_exon |
| novel_circ_005995 | ENSSSCG00000004199 | 1 | + | 32520013 | 32522430 | 698 | annot_exons |
| novel_circ_005996 | NA | 15 | - | 84310231 | 84310470 | 240 | intergenic |
| novel_circ_005997 | ENSSSCG00000002820 | 6 | + | 19168552 | 19175212 | 671 | annot_exons |
| novel_circ_005998 | ENSSSCG00000009994 | 14 | + | 46982871 | 46996102 | 461 | annot_exons |
| novel_circ_005999 | ENSSSCG00000023738 | 11 | - | 21857828 | 21864250 | 425 | annot_exons |
| novel_circ_006000 | ENSSSCG00000005599 | 1 | + | 2.66E+08 | 2.66E+08 | 473 | annot_exons |
| novel_circ_006001 | ENSSSCG00000038036 | 1 | - | 2.62E+08 | 2.62E+08 | 231 | annot_exons |
| novel_circ_006002 | ENSSSCG00000032556 | 3 | + | 18259350 | 18260106 | 608 | annot_exons |
| novel_circ_006003 | ENSSSCG00000043791 | 17 | + | 8568461 | 8570675 | 2215 | antisense |
| novel_circ_006004 | ENSSSCG00000004979 | 1 | - | 1.7E+08 | 1.7E+08 | 1451 | annot_exons |
| novel_circ_006005 | ENSSSCG00000002528 | AEMK02000452.1 | + | 1174519 | 1187633 | 566 | annot_exons |
| novel_circ_006006 | ENSSSCG00000031592 | 1 | + | 2.48E+08 | 2.48E+08 | 6433 | annot_exons |
| novel_circ_006007 | ENSSSCG00000016578 | 18 | + | 19774142 | 19775069 | 928 | antisense |
| novel_circ_006008 | ENSSSCG00000006872 | 4 | - | 1.18E+08 | 1.18E+08 | 617 | annot_exons |
| novel_circ_006009 | ENSSSCG00000026268 | 14 | - | 80294676 | 80294935 | 260 | one_exon |
| novel_circ_006010 | ENSSSCG00000015940 | 15 | + | 76287103 | 76301485 | 583 | annot_exons |
| novel_circ_006011 | ENSSSCG00000006508 | 4 | + | 94346183 | 94346958 | 776 | intronic |
| novel_circ_006012 | ENSSSCG00000008398 | 3 | + | 85663515 | 85672247 | 723 | annot_exons |
| novel_circ_006013 | ENSSSCG00000016836 | 16 | - | 21537442 | 21540786 | 260 | annot_exons |
| novel_circ_006014 | ENSSSCG00000003901 | 6 | - | 1.65E+08 | 1.65E+08 | 251 | annot_exons |
| novel_circ_006015 | ENSSSCG00000014376 | 2 | + | 1.42E+08 | 1.42E+08 | 517 | exon_intron |
| novel_circ_006016 | ENSSSCG00000010190 | 14 | + | 60413781 | 60413921 | 141 | one_exon |
| novel_circ_006017 | ENSSSCG00000036132 | 6 | + | 51711037 | 51712383 | 1347 | antisense |
| novel_circ_006018 | ENSSSCG00000004639 | 1 | - | 1.21E+08 | 1.21E+08 | 231 | annot_exons |
| novel_circ_006019 | ENSSSCG00000031639 | 6 | + | 87888787 | 87913534 | 464 | annot_exons |
| novel_circ_006020 | ENSSSCG00000004710 | 1 | - | 1.28E+08 | 1.28E+08 | 290 | annot_exons |
| novel_circ_006021 | ENSSSCG00000005038 | 1 | + | 1.82E+08 | 1.82E+08 | 274 | annot_exons |
| novel_circ_006022 | ENSSSCG00000016061 | 15 | + | 95981215 | 96007318 | 686 | annot_exons |
| novel_circ_006023 | ENSSSCG00000042794 | 18 | - | 4529231 | 4529878 | 615 | exon_intron |
| novel_circ_006024 | ENSSSCG00000000104 | 5 | + | 9494756 | 9495051 | 173 | annot_exons |
| novel_circ_006025 | ENSSSCG00000017260 | 12 | + | 11741197 | 11748619 | 601 | annot_exons |
| novel_circ_006026 | ENSSSCG00000008103 | 3 | - | 44352110 | 44352326 | 217 | one_exon |
| novel_circ_006027 | ENSSSCG00000005224 | 1 | + | 2.18E+08 | 2.18E+08 | 1328 | annot_exons |
| novel_circ_006028 | ENSSSCG00000016019 | 15 | - | 88461715 | 88463763 | 279 | annot_exons |
| novel_circ_006029 | ENSSSCG00000009037 | 8 | + | 82654479 | 82654959 | 481 | one_exon |
| novel_circ_006030 | ENSSSCG00000009464 | 11 | - | 47567123 | 47571287 | 318 | annot_exons |
| novel_circ_006031 | ENSSSCG00000025703 | 1 | - | 77373802 | 77394243 | 20442 | intronic |
| novel_circ_006032 | ENSSSCG00000033142 | 5 | - | 78079879 | 78080050 | 172 | one_exon |
| novel_circ_006033 | ENSSSCG00000007592 | 3 | + | 5089147 | 5091217 | 281 | annot_exons |
| novel_circ_006034 | ENSSSCG00000020926 | 6 | - | 75647810 | 75651824 | 2034 | exon_intron |
| novel_circ_006035 | ENSSSCG00000035676 | 16 | - | 71254368 | 71255339 | 343 | annot_exons |
| novel_circ_006036 | ENSSSCG00000008738 | 8 | + | 10889018 | 10894216 | 258 | annot_exons |
| novel_circ_006037 | ENSSSCG00000004938 | 1 | - | 1.64E+08 | 1.64E+08 | 323 | annot_exons |
| novel_circ_006038 | ENSSSCG00000006084 | 4 | - | 39059673 | 39069367 | 374 | annot_exons |
| novel_circ_006039 | ENSSSCG00000005994 | 4 | - | 18307901 | 18310422 | 2522 | antisense |
| novel_circ_006040 | ENSSSCG00000007438 | 17 | - | 48237859 | 48251334 | 693 | annot_exons |
| novel_circ_006041 | ENSSSCG00000012650 | X | + | 1.06E+08 | 1.06E+08 | 1874 | annot_exons |
| novel_circ_006042 | ENSSSCG00000001068 | 7 | + | 13232916 | 13237201 | 223 | annot_exons |
| novel_circ_006043 | ENSSSCG00000016738 | 18 | + | 50538799 | 50542127 | 192 | annot_exons |
| novel_circ_006044 | ENSSSCG00000013402 | 2 | - | 47511193 | 47530845 | 930 | annot_exons |
| novel_circ_006045 | NA | 15 | + | 84243897 | 84244075 | 179 | intergenic |
| novel_circ_006046 | ENSSSCG00000000024 | 5 | - | 4943920 | 4959257 | 264 | annot_exons |
| novel_circ_006047 | ENSSSCG00000003111 | 6 | + | 52977944 | 52980682 | 317 | annot_exons |
| novel_circ_006048 | ENSSSCG00000027667 | 7 | + | 1.21E+08 | 1.21E+08 | 390 | annot_exons |
| novel_circ_006049 | ENSSSCG00000037670 | X | + | 90177247 | 90207986 | 205 | annot_exons |
| novel_circ_006050 | ENSSSCG00000006267 | 4 | + | 78123402 | 78139488 | 1964 | exon_intron |
| novel_circ_006051 | ENSSSCG00000014062 | 2 | - | 81583157 | 81584662 | 1506 | one_exon |
| novel_circ_006052 | ENSSSCG00000032648 | 6 | - | 31774038 | 31774827 | 277 | annot_exons |
| novel_circ_006053 | ENSSSCG00000011027 | 10 | - | 42669134 | 42669632 | 269 | annot_exons |
| novel_circ_006054 | ENSSSCG00000021203 | 13 | - | 79518730 | 79522041 | 404 | annot_exons |
| novel_circ_006055 | ENSSSCG00000034569 | 2 | + | 51393324 | 51393511 | 188 | intronic |
| novel_circ_006056 | ENSSSCG00000009853 | 14 | + | 34622192 | 34622720 | 377 | annot_exons |
| novel_circ_006057 | ENSSSCG00000026945 | 13 | - | 29504071 | 29509375 | 352 | annot_exons |
| novel_circ_006058 | ENSSSCG00000012266 | X | - | 41298898 | 41311804 | 468 | annot_exons |
| novel_circ_006059 | ENSSSCG00000012250 | X | - | 36364281 | 36378721 | 991 | annot_exons |
| novel_circ_006060 | ENSSSCG00000007034 | 17 | + | 13115684 | 13116300 | 617 | exon_intron |
| novel_circ_006061 | ENSSSCG00000012181 | X | + | 20600203 | 20610931 | 9154 | exon_intron |
| novel_circ_006062 | ENSSSCG00000004897 | 1 | - | 1.59E+08 | 1.59E+08 | 350 | annot_exons |
| novel_circ_006063 | ENSSSCG00000014800 | 9 | + | 6584546 | 6584647 | 102 | one_exon |
| novel_circ_006064 | ENSSSCG00000006093 | 4 | - | 41631217 | 41645866 | 619 | annot_exons |
| novel_circ_006065 | ENSSSCG00000007289 | 17 | - | 38417789 | 38421102 | 3314 | exon_intron |
| novel_circ_006066 | ENSSSCG00000007280 | 17 | + | 37890389 | 37901251 | 661 | annot_exons |
| novel_circ_006067 | NA | 15 | - | 84250291 | 84252752 | 2462 | intergenic |
| novel_circ_006068 | ENSSSCG00000032986 | AEMK02000569.1 | + | 897757 | 899546 | 927 | annot_exons |
| novel_circ_006069 | ENSSSCG00000029311 | 14 | + | 71327584 | 71335961 | 415 | annot_exons |
| novel_circ_006070 | ENSSSCG00000010798 | 3 | + | 22709563 | 22713415 | 327 | annot_exons |
| novel_circ_006071 | ENSSSCG00000016692 | 18 | - | 44864663 | 44864848 | 186 | one_exon |
| novel_circ_006072 | ENSSSCG00000012463 | X | - | 68975772 | 68978519 | 464 | annot_exons |
| novel_circ_006073 | ENSSSCG00000008192 | 3 | + | 56427942 | 56428793 | 316 | annot_exons |
| novel_circ_006074 | ENSSSCG00000000985 | 7 | - | 229160 | 264071 | 1148 | annot_exons |
| novel_circ_006075 | ENSSSCG00000027257 | 1 | + | 27360 | 33429 | 320 | annot_exons |
| novel_circ_006076 | ENSSSCG00000016426 | 18 | - | 4697810 | 4707261 | 615 | annot_exons |
| novel_circ_006077 | ENSSSCG00000009192 | 8 | - | 1.25E+08 | 1.25E+08 | 2217 | annot_exons |
| novel_circ_006078 | ENSSSCG00000032531 | 3 | + | 26691921 | 26699741 | 869 | annot_exons |
| novel_circ_006079 | ENSSSCG00000023759 | X | + | 42725835 | 42726193 | 241 | annot_exons |
| novel_circ_006080 | ENSSSCG00000026346 | 1 | + | 83174465 | 83180536 | 343 | annot_exons |
| novel_circ_006081 | ENSSSCG00000012328 | X | - | 46339032 | 46341599 | 406 | annot_exons |
| novel_circ_006082 | ENSSSCG00000026744 | 11 | - | 1341589 | 1355348 | 381 | annot_exons |
| novel_circ_006083 | ENSSSCG00000025606 | 9 | + | 36729254 | 36731849 | 341 | annot_exons |
| novel_circ_006084 | ENSSSCG00000038752 | 7 | - | 19516117 | 19519186 | 258 | annot_exons |
| novel_circ_006085 | NA | 15 | + | 84303867 | 84304929 | 1063 | intergenic |
| novel_circ_006086 | ENSSSCG00000044913 | 2 | - | 6801336 | 6802304 | 969 | exon_intron |
| novel_circ_006087 | ENSSSCG00000004979 | 1 | - | 1.7E+08 | 1.7E+08 | 382 | annot_exons |
| novel_circ_006088 | ENSSSCG00000050094 | 5 | + | 64129689 | 64131401 | 1713 | one_exon |
| novel_circ_006089 | ENSSSCG00000036909 | 6 | + | 1.67E+08 | 1.67E+08 | 3819 | annot_exons |
| novel_circ_006090 | ENSSSCG00000003384 | 6 | - | 67470674 | 67471765 | 393 | annot_exons |
| novel_circ_006091 | ENSSSCG00000002682 | 6 | + | 4516231 | 4517729 | 742 | annot_exons |
| novel_circ_006092 | ENSSSCG00000006752 | 4 | + | 1.06E+08 | 1.06E+08 | 306 | annot_exons |
| novel_circ_006093 | ENSSSCG00000033377 | 3 | + | 6981006 | 6983946 | 283 | annot_exons |
| novel_circ_006094 | ENSSSCG00000017289 | 12 | - | 15122130 | 15123727 | 268 | annot_exons |
| novel_circ_006095 | ENSSSCG00000011485 | 13 | + | 43548996 | 43563837 | 354 | annot_exons |
| novel_circ_006096 | ENSSSCG00000012055 | 13 | + | 2E+08 | 2E+08 | 400 | annot_exons |
| novel_circ_006097 | ENSSSCG00000017308 | 12 | + | 16864075 | 16868735 | 247 | annot_exons |
| novel_circ_006098 | ENSSSCG00000006872 | 4 | - | 1.18E+08 | 1.18E+08 | 16513 | exon_intron |
| novel_circ_006099 | ENSSSCG00000021005 | 10 | + | 31303100 | 31303258 | 159 | one_exon |
| novel_circ_006100 | ENSSSCG00000037451 | 2 | - | 3096937 | 3098647 | 419 | annot_exons |
| novel_circ_006101 | ENSSSCG00000004651 | 1 | - | 1.22E+08 | 1.22E+08 | 238 | annot_exons |
| novel_circ_006102 | ENSSSCG00000038397 | 4 | + | 2738960 | 2760683 | 482 | annot_exons |
| novel_circ_006103 | ENSSSCG00000004829 | 6 | - | 1.48E+08 | 1.48E+08 | 561 | annot_exons |
| novel_circ_006104 | ENSSSCG00000036537 | 2 | - | 75205185 | 75217004 | 522 | annot_exons |
| novel_circ_006105 | ENSSSCG00000032220 | 13 | + | 18665942 | 18670875 | 313 | annot_exons |
| novel_circ_006106 | ENSSSCG00000023738 | 11 | - | 21856562 | 21857923 | 221 | annot_exons |
| novel_circ_006107 | ENSSSCG00000039991 | 8 | + | 21470 | 30272 | 1363 | annot_exons |
| novel_circ_006108 | ENSSSCG00000009134 | 8 | - | 1.12E+08 | 1.12E+08 | 610 | annot_exons |
| novel_circ_006109 | ENSSSCG00000010357 | 14 | - | 87502986 | 87508558 | 579 | annot_exons |
| novel_circ_006110 | ENSSSCG00000009890 | 14 | - | 39870224 | 39874266 | 393 | annot_exons |
| novel_circ_006111 | ENSSSCG00000002386 | 7 | + | 99337966 | 99363274 | 612 | annot_exons |
| novel_circ_006112 | ENSSSCG00000003809 | 6 | + | 1.48E+08 | 1.48E+08 | 318 | annot_exons |
| novel_circ_006113 | ENSSSCG00000002869 | 6 | + | 43870383 | 43887734 | 496 | annot_exons |
| novel_circ_006114 | ENSSSCG00000004385 | 1 | - | 74308772 | 74314994 | 221 | annot_exons |
| novel_circ_006115 | ENSSSCG00000012251 | X | + | 36761086 | 36761370 | 285 | one_exon |
| novel_circ_006116 | ENSSSCG00000001596 | 7 | - | 35311739 | 35313400 | 292 | annot_exons |
| novel_circ_006117 | ENSSSCG00000034624 | 13 | - | 1.97E+08 | 1.97E+08 | 416 | annot_exons |
| novel_circ_006118 | ENSSSCG00000017626 | 12 | - | 34276461 | 34279927 | 3391 | annot_exons |
| novel_circ_006119 | ENSSSCG00000010148 | 14 | + | 55105293 | 55105756 | 340 | annot_exons |
| novel_circ_006120 | ENSSSCG00000010569 | 14 | - | 1.13E+08 | 1.13E+08 | 303 | annot_exons |
| novel_circ_006121 | ENSSSCG00000031808 | 11 | + | 68469325 | 68469785 | 461 | one_exon |
| novel_circ_006122 | ENSSSCG00000024373 | 15 | + | 1.31E+08 | 1.31E+08 | 412 | antisense |
| novel_circ_006123 | ENSSSCG00000000866 | 5 | - | 82571957 | 82579563 | 453 | annot_exons |
| novel_circ_006124 | ENSSSCG00000011632 | 13 | - | 73859561 | 73864547 | 785 | annot_exons |
| novel_circ_006125 | ENSSSCG00000003627 | 6 | + | 91593753 | 91605991 | 781 | annot_exons |
| novel_circ_006126 | ENSSSCG00000006090 | 4 | + | 40379783 | 40395305 | 1158 | annot_exons |
| novel_circ_006127 | ENSSSCG00000014126 | 2 | + | 89323270 | 89413823 | 1245 | annot_exons |
| novel_circ_006128 | ENSSSCG00000013399 | 2 | - | 46435587 | 46455592 | 543 | annot_exons |
| novel_circ_006129 | ENSSSCG00000022830 | 15 | - | 1.13E+08 | 1.13E+08 | 1579 | annot_exons |
| novel_circ_006130 | ENSSSCG00000002436 | 7 | - | 1.12E+08 | 1.12E+08 | 252 | annot_exons |
| novel_circ_006131 | ENSSSCG00000017042 | 16 | - | 64195119 | 64198609 | 236 | annot_exons |
| novel_circ_006132 | ENSSSCG00000001485 | 7 | - | 26620967 | 26676922 | 2175 | annot_exons |
| novel_circ_006133 | ENSSSCG00000008130 | 3 | - | 46961408 | 46962480 | 1073 | one_exon |
| novel_circ_006134 | ENSSSCG00000012328 | X | - | 46365334 | 46367314 | 482 | annot_exons |
| novel_circ_006135 | ENSSSCG00000026004 | 5 | + | 29727194 | 29727942 | 749 | intronic |
| novel_circ_006136 | ENSSSCG00000010631 | 14 | - | 1.23E+08 | 1.23E+08 | 526 | annot_exons |
| novel_circ_006137 | ENSSSCG00000015940 | 15 | + | 76228677 | 76232137 | 573 | annot_exons |
| novel_circ_006138 | ENSSSCG00000014792 | 9 | - | 6246942 | 6248057 | 1116 | intronic |
| novel_circ_006139 | ENSSSCG00000006305 | 4 | + | 83148304 | 83154780 | 535 | annot_exons |
| novel_circ_006140 | ENSSSCG00000034395 | 15 | + | 1.11E+08 | 1.11E+08 | 406 | annot_exons |
| novel_circ_006141 | ENSSSCG00000003081 | 6 | + | 51226510 | 51226836 | 327 | one_exon |
| novel_circ_006142 | ENSSSCG00000021060 | 11 | + | 18114767 | 18151301 | 592 | annot_exons |
| novel_circ_006143 | NA | 15 | - | 84262141 | 84262436 | 296 | intergenic |
| novel_circ_006144 | ENSSSCG00000000801 | 5 | + | 75167350 | 75234212 | 198 | annot_exons |
| novel_circ_006145 | ENSSSCG00000050094 | 5 | + | 64129689 | 64129867 | 179 | one_exon |
| novel_circ_006146 | ENSSSCG00000016956 | 16 | + | 45542516 | 45542745 | 230 | one_exon |
| novel_circ_006147 | ENSSSCG00000033591 | 6 | - | 31842360 | 31849452 | 531 | annot_exons |
| novel_circ_006148 | ENSSSCG00000001806 | 7 | + | 52175903 | 52178590 | 291 | annot_exons |
| novel_circ_006149 | ENSSSCG00000027131 | 18 | - | 18676123 | 18684684 | 726 | annot_exons |
| novel_circ_006150 | ENSSSCG00000030610 | 2 | + | 14915160 | 14922158 | 985 | annot_exons |
| novel_circ_006151 | ENSSSCG00000020663 | 18 | + | 5258888 | 5261844 | 668 | annot_exons |
| novel_circ_006152 | ENSSSCG00000015790 | 15 | - | 46197571 | 46197933 | 363 | antisense |
| novel_circ_006153 | ENSSSCG00000016816 | 16 | - | 19906267 | 19906628 | 362 | one_exon |
| novel_circ_006154 | ENSSSCG00000011867 | 13 | + | 1.37E+08 | 1.37E+08 | 967 | annot_exons |
| novel_circ_006155 | ENSSSCG00000000970 | 5 | - | 313664 | 341527 | 707 | annot_exons |
| novel_circ_006156 | ENSSSCG00000022830 | 15 | - | 1.13E+08 | 1.13E+08 | 799 | annot_exons |
| novel_circ_006157 | ENSSSCG00000017273 | 12 | - | 13713600 | 13713973 | 285 | annot_exons |
| novel_circ_006158 | ENSSSCG00000003833 | 6 | + | 1.55E+08 | 1.55E+08 | 203 | one_exon |
| novel_circ_006159 | ENSSSCG00000036948 | 10 | + | 23645814 | 23646068 | 255 | intronic |
| novel_circ_006160 | ENSSSCG00000011628 | 13 | + | 73686496 | 73689649 | 288 | annot_exons |
| novel_circ_006161 | ENSSSCG00000034677 | 15 | - | 84407848 | 84410040 | 677 | annot_exons |
| novel_circ_006162 | ENSSSCG00000001053 | 7 | - | 9695480 | 9700142 | 326 | annot_exons |
| novel_circ_006163 | ENSSSCG00000036812 | 12 | - | 61081538 | 61083056 | 249 | annot_exons |
| novel_circ_006164 | ENSSSCG00000031990 | 16 | - | 41104364 | 41104716 | 217 | annot_exons |
| novel_circ_006165 | ENSSSCG00000009123 | 8 | + | 1.09E+08 | 1.09E+08 | 294 | annot_exons |
| novel_circ_006166 | ENSSSCG00000010956 | 10 | - | 29228248 | 29230143 | 346 | annot_exons |
| novel_circ_006167 | ENSSSCG00000010863 | 10 | + | 15095068 | 15095653 | 259 | annot_exons |
| novel_circ_006168 | NA | 2 | + | 6808349 | 6808778 | 430 | intergenic |
| novel_circ_006169 | ENSSSCG00000027857 | 2 | + | 1.23E+08 | 1.23E+08 | 670 | annot_exons |
| novel_circ_006170 | ENSSSCG00000025602 | 18 | + | 33732361 | 33736560 | 354 | annot_exons |
| novel_circ_006171 | ENSSSCG00000005602 | 1 | + | 2.66E+08 | 2.66E+08 | 325 | annot_exons |
| novel_circ_006172 | ENSSSCG00000011953 | 13 | + | 1.57E+08 | 1.57E+08 | 567 | annot_exons |
| novel_circ_006173 | ENSSSCG00000026724 | 5 | + | 58018273 | 58019205 | 293 | annot_exons |
| novel_circ_006174 | ENSSSCG00000038628 | 2 | - | 1.36E+08 | 1.36E+08 | 315 | annot_exons |
| novel_circ_006175 | ENSSSCG00000017024 | 16 | + | 60349430 | 60349669 | 240 | antisense |
| novel_circ_006176 | ENSSSCG00000017339 | 12 | - | 18321661 | 18326321 | 256 | annot_exons |
| novel_circ_006177 | ENSSSCG00000024814 | X | - | 47764058 | 47803092 | 8753 | exon_intron |
| novel_circ_006178 | ENSSSCG00000022940 | 8 | - | 71274536 | 71275152 | 617 | one_exon |
| novel_circ_006179 | ENSSSCG00000030608 | 5 | - | 87898605 | 87899774 | 344 | annot_exons |
| novel_circ_006180 | ENSSSCG00000034704 | 1 | - | 1.62E+08 | 1.62E+08 | 282 | one_exon |
| novel_circ_006181 | ENSSSCG00000025130 | 11 | - | 46069180 | 46155680 | 746 | annot_exons |
| novel_circ_006182 | ENSSSCG00000016848 | 16 | + | 23489309 | 23509656 | 420 | annot_exons |
| novel_circ_006183 | ENSSSCG00000006096 | 4 | - | 41871105 | 41873396 | 265 | annot_exons |
| novel_circ_006184 | ENSSSCG00000012955 | 2 | - | 6114129 | 6114797 | 265 | annot_exons |
| novel_circ_006185 | ENSSSCG00000015940 | 15 | + | 76166995 | 76192330 | 560 | annot_exons |
| novel_circ_006186 | ENSSSCG00000034569 | 2 | + | 51363109 | 51370259 | 7151 | exon_intron |
| novel_circ_006187 | ENSSSCG00000005477 | 1 | - | 2.54E+08 | 2.54E+08 | 479 | annot_exons |
| novel_circ_006188 | ENSSSCG00000004281 | 1 | + | 52441583 | 52467058 | 25476 | intronic |
| novel_circ_006189 | ENSSSCG00000005719 | 1 | - | 2.72E+08 | 2.72E+08 | 1584 | annot_exons |
| novel_circ_006190 | ENSSSCG00000015001 | 9 | - | 36303459 | 36306494 | 304 | annot_exons |
| novel_circ_006191 | ENSSSCG00000011103 | 10 | - | 56976187 | 56986705 | 243 | annot_exons |
| novel_circ_006192 | ENSSSCG00000022659 | 12 | + | 35882708 | 35884162 | 399 | annot_exons |
| novel_circ_006193 | NA | 15 | - | 84428248 | 84428625 | 378 | intergenic |
| novel_circ_006194 | ENSSSCG00000018049 | 12 | - | 60361138 | 60370222 | 345 | annot_exons |
| novel_circ_006195 | ENSSSCG00000022534 | 4 | + | 1.26E+08 | 1.26E+08 | 4228 | annot_exons |
| novel_circ_006196 | ENSSSCG00000008753 | 8 | + | 16750409 | 16751510 | 1102 | antisense |
| novel_circ_006197 | ENSSSCG00000020663 | 18 | + | 5245060 | 5254144 | 2077 | annot_exons |
| novel_circ_006198 | ENSSSCG00000014291 | 2 | - | 1.35E+08 | 1.35E+08 | 1165 | annot_exons |
| novel_circ_006199 | ENSSSCG00000000806 | 5 | - | 76839521 | 76855626 | 2826 | annot_exons |
| novel_circ_006200 | NA | 11 | - | 13137264 | 13137476 | 213 | intergenic |
| novel_circ_006201 | ENSSSCG00000011103 | 10 | - | 57126906 | 57127145 | 240 | one_exon |
| novel_circ_006202 | ENSSSCG00000007097 | 17 | + | 26776910 | 26783795 | 325 | annot_exons |
| novel_circ_006203 | NA | 15 | + | 84428668 | 84430863 | 2196 | intergenic |
| novel_circ_006204 | ENSSSCG00000009971 | 14 | + | 46180499 | 46182509 | 2011 | exon_intron |
| novel_circ_006205 | ENSSSCG00000015862 | 15 | + | 59691831 | 59705245 | 498 | annot_exons |
| novel_circ_006206 | ENSSSCG00000012742 | X | + | 1.22E+08 | 1.22E+08 | 423 | annot_exons |
| novel_circ_006207 | ENSSSCG00000006388 | 4 | - | 90202165 | 90202383 | 219 | antisense |
| novel_circ_006208 | ENSSSCG00000015747 | 15 | + | 33565996 | 33572812 | 609 | annot_exons |
| novel_circ_006209 | ENSSSCG00000006927 | 4 | - | 1.28E+08 | 1.28E+08 | 167 | annot_exons |
| novel_circ_006210 | ENSSSCG00000035196 | 4 | - | 1.25E+08 | 1.25E+08 | 578 | annot_exons |
| novel_circ_006211 | ENSSSCG00000024814 | X | - | 47818210 | 47822652 | 431 | exon_intron |
| novel_circ_006212 | ENSSSCG00000023408 | 1 | + | 1.84E+08 | 1.84E+08 | 883 | annot_exons |
| novel_circ_006213 | ENSSSCG00000020743 | 1 | + | 2.64E+08 | 2.64E+08 | 630 | annot_exons |
| novel_circ_006214 | ENSSSCG00000026153 | 9 | + | 36536798 | 36540781 | 505 | annot_exons |
| novel_circ_006215 | ENSSSCG00000032390 | 18 | - | 9133515 | 9140672 | 2170 | annot_exons |
| novel_circ_006216 | ENSSSCG00000028979 | 9 | + | 10137495 | 10139416 | 1695 | annot_exons |
| novel_circ_006217 | ENSSSCG00000005672 | 1 | + | 2.69E+08 | 2.69E+08 | 713 | exon_intron |
| novel_circ_006218 | ENSSSCG00000009464 | 11 | - | 47604849 | 47614315 | 336 | annot_exons |
| novel_circ_006219 | ENSSSCG00000000571 | 5 | + | 51561006 | 51561815 | 309 | annot_exons |
| novel_circ_006220 | ENSSSCG00000012642 | X | + | 1.02E+08 | 1.02E+08 | 437 | annot_exons |
| novel_circ_006221 | ENSSSCG00000002003 | 7 | - | 75145650 | 75146187 | 363 | annot_exons |
| novel_circ_006222 | ENSSSCG00000025406 | 2 | + | 41990793 | 41996912 | 754 | annot_exons |
| novel_circ_006223 | ENSSSCG00000008337 | 3 | + | 72926172 | 72929955 | 496 | exon_intron |
| novel_circ_006224 | ENSSSCG00000024030 | 12 | + | 59744649 | 59773763 | 1612 | annot_exons |
| novel_circ_006225 | ENSSSCG00000023264 | 15 | - | 1.11E+08 | 1.11E+08 | 398 | annot_exons |
| novel_circ_006226 | ENSSSCG00000029169 | 1 | - | 2.66E+08 | 2.66E+08 | 601 | annot_exons |
| novel_circ_006227 | ENSSSCG00000001903 | 7 | + | 58850345 | 58868520 | 885 | exon_intron |
| novel_circ_006228 | ENSSSCG00000002269 | 7 | + | 88526920 | 88527712 | 261 | annot_exons |
| novel_circ_006229 | ENSSSCG00000012585 | X | - | 91433021 | 91442301 | 727 | annot_exons |
| novel_circ_006230 | ENSSSCG00000005715 | 1 | + | 2.71E+08 | 2.71E+08 | 503 | annot_exons |
| novel_circ_006231 | ENSSSCG00000011214 | 13 | - | 12631992 | 12633500 | 276 | annot_exons |
| novel_circ_006232 | ENSSSCG00000036145 | 6 | - | 1.56E+08 | 1.56E+08 | 233 | annot_exons |
| novel_circ_006233 | ENSSSCG00000029464 | 5 | - | 67643269 | 67653977 | 1239 | annot_exons |
| novel_circ_006234 | ENSSSCG00000039025 | 1 | + | 2.08E+08 | 2.08E+08 | 477 | annot_exons |
| novel_circ_006235 | ENSSSCG00000016513 | 18 | + | 10794878 | 10800526 | 581 | annot_exons |
| novel_circ_006236 | ENSSSCG00000039701 | 7 | - | 92743360 | 92759618 | 281 | annot_exons |
| novel_circ_006237 | ENSSSCG00000001800 | 7 | - | 51824231 | 51839443 | 829 | annot_exons |
| novel_circ_006238 | ENSSSCG00000009714 | 14 | + | 20178343 | 20189932 | 337 | annot_exons |
| novel_circ_006239 | ENSSSCG00000004739 | 1 | - | 1.3E+08 | 1.3E+08 | 913 | annot_exons |
| novel_circ_006240 | ENSSSCG00000016291 | 15 | + | 1.33E+08 | 1.33E+08 | 1981 | annot_exons |
| novel_circ_006241 | ENSSSCG00000012884 | 2 | - | 4431225 | 4449449 | 728 | annot_exons |
| novel_circ_006242 | ENSSSCG00000009682 | 14 | + | 12826402 | 12837511 | 265 | annot_exons |
| novel_circ_006243 | ENSSSCG00000024486 | 17 | + | 11825820 | 11850285 | 388 | annot_exons |
| novel_circ_006244 | ENSSSCG00000004934 | 1 | - | 1.63E+08 | 1.63E+08 | 280 | annot_exons |
| novel_circ_006245 | ENSSSCG00000002259 | 7 | + | 88142523 | 88165789 | 843 | annot_exons |
| novel_circ_006246 | ENSSSCG00000003700 | 6 | - | 1.07E+08 | 1.07E+08 | 423 | annot_exons |
| novel_circ_006247 | ENSSSCG00000008079 | 3 | + | 42946461 | 42962746 | 897 | annot_exons |
| novel_circ_006248 | ENSSSCG00000013984 | 2 | + | 50877353 | 50886317 | 616 | annot_exons |
| novel_circ_006249 | ENSSSCG00000007805 | 3 | + | 18553400 | 18555926 | 632 | annot_exons |
| novel_circ_006250 | ENSSSCG00000016518 | 18 | - | 11062517 | 11063935 | 462 | annot_exons |
| novel_circ_006251 | ENSSSCG00000007309 | 17 | - | 39049738 | 39052455 | 409 | annot_exons |
| novel_circ_006252 | ENSSSCG00000020743 | 1 | + | 2.64E+08 | 2.64E+08 | 973 | annot_exons |
| novel_circ_006253 | ENSSSCG00000013645 | 2 | + | 69326466 | 69326895 | 276 | annot_exons |
| novel_circ_006254 | ENSSSCG00000001865 | 7 | - | 56751130 | 56759939 | 8810 | exon_intron |
| novel_circ_006255 | ENSSSCG00000002245 | 7 | + | 80343058 | 80346071 | 459 | exon_intron |
| novel_circ_006256 | ENSSSCG00000007077 | 17 | - | 22463411 | 22465786 | 268 | annot_exons |
| novel_circ_006257 | ENSSSCG00000006873 | 4 | + | 1.18E+08 | 1.18E+08 | 55730 | exon_intron |
| novel_circ_006258 | ENSSSCG00000050244 | 12 | + | 59273100 | 59291841 | 18742 | exon_intron |
| novel_circ_006259 | ENSSSCG00000004032 | 1 | + | 6442170 | 6525252 | 69324 | exon_intron |
| novel_circ_006260 | ENSSSCG00000007542 | 3 | - | 403295 | 404907 | 263 | annot_exons |
| novel_circ_006261 | ENSSSCG00000016397 | 15 | + | 635616 | 636572 | 516 | annot_exons |
| novel_circ_006262 | ENSSSCG00000006487 | 4 | + | 93738637 | 93742580 | 215 | annot_exons |
| novel_circ_006263 | ENSSSCG00000022225 | 15 | + | 1.06E+08 | 1.06E+08 | 1774 | exon_intron |
| novel_circ_006264 | ENSSSCG00000017672 | 12 | + | 36384264 | 36388515 | 418 | annot_exons |
| novel_circ_006265 | ENSSSCG00000036948 | 10 | + | 23648926 | 23652219 | 1045 | annot_exons |
| novel_circ_006266 | ENSSSCG00000012000 | 13 | + | 1.74E+08 | 1.74E+08 | 626 | annot_exons |
| novel_circ_006267 | ENSSSCG00000016113 | 15 | - | 1.06E+08 | 1.06E+08 | 797 | antisense |
| novel_circ_006268 | ENSSSCG00000031701 | 5 | - | 73383231 | 73399218 | 391 | annot_exons |
| novel_circ_006269 | NA | 15 | + | 84462336 | 84466583 | 4248 | intergenic |
| novel_circ_006270 | ENSSSCG00000014794 | 9 | + | 6336548 | 6339661 | 380 | annot_exons |
| novel_circ_006271 | ENSSSCG00000013566 | 2 | + | 71919789 | 71923513 | 331 | annot_exons |
| novel_circ_006272 | ENSSSCG00000010679 | 14 | - | 1.29E+08 | 1.29E+08 | 376 | annot_exons |
| novel_circ_006273 | ENSSSCG00000025206 | 6 | - | 89236865 | 89237070 | 206 | one_exon |
| novel_circ_006274 | ENSSSCG00000016948 | 16 | - | 44108616 | 44117246 | 501 | annot_exons |
| novel_circ_006275 | NA | 15 | - | 44894098 | 44894655 | 558 | intergenic |
| novel_circ_006276 | ENSSSCG00000029441 | 12 | - | 55232453 | 55232571 | 119 | one_exon |
| novel_circ_006277 | ENSSSCG00000006983 | 17 | - | 4939669 | 4945899 | 501 | annot_exons |
| novel_circ_006278 | ENSSSCG00000008591 | 3 | + | 1.15E+08 | 1.15E+08 | 635 | annot_exons |
| novel_circ_006279 | ENSSSCG00000005444 | 1 | - | 2.5E+08 | 2.5E+08 | 2671 | exon_intron |
| novel_circ_006280 | NA | 15 | - | 84468158 | 84468400 | 243 | intergenic |
| novel_circ_006281 | ENSSSCG00000000599 | 5 | - | 56657427 | 56664010 | 514 | annot_exons |
| novel_circ_006282 | ENSSSCG00000021285 | 3 | - | 4303785 | 4308978 | 305 | annot_exons |
| novel_circ_006283 | ENSSSCG00000011068 | 10 | - | 48814823 | 48816010 | 279 | annot_exons |
| novel_circ_006284 | ENSSSCG00000011854 | 13 | + | 1.34E+08 | 1.34E+08 | 441 | annot_exons |
| novel_circ_006285 | ENSSSCG00000002872 | 6 | + | 44019030 | 44021236 | 1160 | annot_exons |
| novel_circ_006286 | ENSSSCG00000025514 | 8 | + | 67550217 | 67551581 | 292 | annot_exons |
| novel_circ_006287 | ENSSSCG00000009591 | 14 | - | 2478793 | 2485932 | 4879 | exon_intron |
| novel_circ_006288 | ENSSSCG00000004999 | 1 | - | 1.75E+08 | 1.75E+08 | 310 | annot_exons |
| novel_circ_006289 | ENSSSCG00000015714 | 15 | - | 23546608 | 23559784 | 3074 | annot_exons |
| novel_circ_006290 | ENSSSCG00000005436 | 1 | + | 2.48E+08 | 2.48E+08 | 638 | annot_exons |
| novel_circ_006291 | ENSSSCG00000012382 | X | + | 56182839 | 56188670 | 389 | annot_exons |
| novel_circ_006292 | ENSSSCG00000016618 | 18 | - | 25791450 | 25809926 | 1137 | annot_exons |
| novel_circ_006293 | ENSSSCG00000010360 | 14 | + | 87854602 | 87876505 | 834 | annot_exons |
| novel_circ_006294 | ENSSSCG00000027898 | 5 | + | 92992680 | 92993106 | 427 | one_exon |
| novel_circ_006295 | ENSSSCG00000004029 | 1 | - | 4745308 | 4843415 | 831 | annot_exons |
| novel_circ_006296 | ENSSSCG00000028724 | 13 | + | 1.92E+08 | 1.92E+08 | 175 | annot_exons |
| novel_circ_006297 | ENSSSCG00000007280 | 17 | + | 37836857 | 37854328 | 799 | annot_exons |
| novel_circ_006298 | ENSSSCG00000009430 | 11 | - | 24726868 | 24739289 | 410 | annot_exons |
| novel_circ_006299 | ENSSSCG00000002285 | 7 | + | 90811957 | 90831658 | 482 | annot_exons |
| novel_circ_006300 | ENSSSCG00000013344 | 2 | - | 37201691 | 37215750 | 532 | annot_exons |
| novel_circ_006301 | ENSSSCG00000000985 | 7 | - | 229160 | 246213 | 529 | annot_exons |
| novel_circ_006302 | ENSSSCG00000034139 | 1 | - | 30459213 | 30502065 | 42853 | exon_intron |
| novel_circ_006303 | ENSSSCG00000006065 | 4 | + | 36435871 | 36437697 | 497 | annot_exons |
| novel_circ_006304 | ENSSSCG00000002628 | 7 | - | 46696413 | 46701207 | 319 | annot_exons |
| novel_circ_006305 | ENSSSCG00000006510 | 4 | + | 94218669 | 94220967 | 317 | annot_exons |
| novel_circ_006306 | ENSSSCG00000002779 | 6 | + | 28013753 | 28037996 | 1629 | annot_exons |
| novel_circ_006307 | ENSSSCG00000037653 | 13 | + | 6908887 | 6909142 | 256 | one_exon |
| novel_circ_006308 | ENSSSCG00000010739 | 14 | - | 1.34E+08 | 1.34E+08 | 264 | annot_exons |
| novel_circ_006309 | ENSSSCG00000002502 | 7 | - | 1.18E+08 | 1.18E+08 | 377 | annot_exons |
| novel_circ_006310 | ENSSSCG00000039094 | 4 | - | 752988 | 753404 | 417 | antisense |
| novel_circ_006311 | ENSSSCG00000029637 | 2 | - | 5285891 | 5292935 | 444 | annot_exons |
| novel_circ_006312 | ENSSSCG00000008575 | 3 | + | 1.13E+08 | 1.13E+08 | 1472 | annot_exons |
| novel_circ_006313 | ENSSSCG00000029311 | 14 | + | 71327584 | 71342956 | 557 | annot_exons |
| novel_circ_006314 | ENSSSCG00000011033 | 10 | - | 43517750 | 43523213 | 5464 | antisense |
| novel_circ_006315 | ENSSSCG00000020803 | 7 | + | 31958113 | 31962520 | 253 | annot_exons |
| novel_circ_006316 | ENSSSCG00000011340 | 13 | - | 30431233 | 30445612 | 397 | annot_exons |
| novel_circ_006317 | ENSSSCG00000023873 | 4 | + | 34249440 | 34251517 | 452 | annot_exons |
| novel_circ_006318 | ENSSSCG00000007544 | 3 | + | 501359 | 506331 | 444 | annot_exons |
| novel_circ_006319 | ENSSSCG00000009714 | 14 | + | 20270674 | 20282065 | 645 | annot_exons |
| novel_circ_006320 | ENSSSCG00000029464 | 5 | - | 67674585 | 67699579 | 1431 | annot_exons |
| novel_circ_006321 | ENSSSCG00000023992 | 12 | - | 12801758 | 12807169 | 478 | annot_exons |
| novel_circ_006322 | ENSSSCG00000000842 | 5 | - | 79294042 | 79296502 | 2461 | exon_intron |
| novel_circ_006323 | ENSSSCG00000004082 | 1 | + | 14069211 | 14074352 | 655 | annot_exons |
| novel_circ_006324 | ENSSSCG00000002950 | 6 | + | 47062616 | 47070506 | 852 | annot_exons |
| novel_circ_006325 | ENSSSCG00000015039 | 9 | + | 39891300 | 39902452 | 925 | annot_exons |
| novel_circ_006326 | ENSSSCG00000024088 | 1 | - | 1.09E+08 | 1.1E+08 | 45289 | exon_intron |
| novel_circ_006327 | ENSSSCG00000001930 | 7 | - | 61032640 | 61032736 | 97 | antisense |
| novel_circ_006328 | ENSSSCG00000026196 | 8 | + | 1.3E+08 | 1.3E+08 | 400 | annot_exons |
| novel_circ_006329 | ENSSSCG00000032357 | 13 | - | 39120076 | 39121645 | 1570 | antisense |
| novel_circ_006330 | ENSSSCG00000001918 | 7 | + | 59804978 | 59837616 | 3546 | annot_exons |
| novel_circ_006331 | NA | 3 | + | 1.02E+08 | 1.02E+08 | 4753 | intergenic |
| novel_circ_006332 | ENSSSCG00000002505 | 7 | + | 1.18E+08 | 1.18E+08 | 650 | annot_exons |
| novel_circ_006333 | ENSSSCG00000016027 | 15 | + | 91666223 | 91684121 | 901 | annot_exons |
| novel_circ_006334 | ENSSSCG00000005178 | 1 | - | 2.06E+08 | 2.06E+08 | 545 | annot_exons |
| novel_circ_006335 | ENSSSCG00000013984 | 2 | + | 50831116 | 50831660 | 545 | one_exon |
| novel_circ_006336 | ENSSSCG00000015273 | 9 | + | 64402332 | 64403424 | 456 | annot_exons |
| novel_circ_006337 | ENSSSCG00000023266 | 6 | + | 1.69E+08 | 1.69E+08 | 1015 | annot_exons |
| novel_circ_006338 | ENSSSCG00000011373 | 13 | - | 31673546 | 31676127 | 448 | annot_exons |
| novel_circ_006339 | ENSSSCG00000050244 | 12 | + | 59273100 | 59283672 | 10573 | exon_intron |
| novel_circ_006340 | ENSSSCG00000004979 | 1 | - | 1.69E+08 | 1.69E+08 | 306 | annot_exons |
| novel_circ_006341 | ENSSSCG00000026113 | 13 | - | 1.46E+08 | 1.46E+08 | 12286 | antisense |
| novel_circ_006342 | ENSSSCG00000028902 | 4 | + | 77616124 | 77618720 | 630 | annot_exons |
| novel_circ_006343 | ENSSSCG00000010329 | 14 | + | 81455080 | 81477281 | 226 | exon_intron |
| novel_circ_006344 | ENSSSCG00000015509 | 9 | - | 1.18E+08 | 1.18E+08 | 364 | annot_exons |
| novel_circ_006345 | ENSSSCG00000036669 | 2 | + | 9212611 | 9212919 | 309 | intronic |
| novel_circ_006346 | ENSSSCG00000004603 | 1 | + | 1.16E+08 | 1.16E+08 | 7369 | exon_intron |
| novel_circ_006347 | ENSSSCG00000016426 | 18 | - | 4718566 | 4730946 | 292 | annot_exons |
| novel_circ_006348 | ENSSSCG00000004390 | 1 | - | 75145585 | 75153476 | 7892 | intronic |
| novel_circ_006349 | ENSSSCG00000045677 | 11 | - | 20685964 | 20686626 | 663 | intronic |
| novel_circ_006350 | NA | 15 | + | 84292636 | 84293048 | 413 | intergenic |
| novel_circ_006351 | ENSSSCG00000016069 | 15 | + | 1.01E+08 | 1.01E+08 | 198 | annot_exons |
| novel_circ_006352 | ENSSSCG00000033690 | 4 | + | 16323825 | 16335370 | 464 | annot_exons |
| novel_circ_006353 | ENSSSCG00000009885 | 14 | + | 39484811 | 39485240 | 311 | annot_exons |
| novel_circ_006354 | ENSSSCG00000024513 | 13 | + | 30110846 | 30113591 | 272 | annot_exons |
| novel_circ_006355 | ENSSSCG00000029578 | 6 | + | 28714817 | 28714979 | 163 | one_exon |
| novel_circ_006356 | ENSSSCG00000001727 | 7 | - | 42064668 | 42065162 | 495 | one_exon |
| novel_circ_006357 | ENSSSCG00000003301 | 6 | - | 59437305 | 59437513 | 209 | intronic |
| novel_circ_006358 | ENSSSCG00000015908 | 15 | - | 72447550 | 72450009 | 358 | annot_exons |
| novel_circ_006359 | ENSSSCG00000031459 | 2 | + | 4730666 | 4749110 | 726 | annot_exons |
| novel_circ_006360 | ENSSSCG00000008512 | 3 | - | 1.07E+08 | 1.07E+08 | 412 | annot_exons |
| novel_circ_006361 | ENSSSCG00000001903 | 7 | + | 58868185 | 58868520 | 336 | one_exon |
| novel_circ_006362 | ENSSSCG00000021170 | 2 | - | 45560493 | 45577171 | 552 | annot_exons |
| novel_circ_006363 | ENSSSCG00000006725 | 4 | + | 1.02E+08 | 1.02E+08 | 403 | annot_exons |
| novel_circ_006364 | ENSSSCG00000026996 | 11 | - | 64111450 | 64138070 | 1200 | annot_exons |
| novel_circ_006365 | ENSSSCG00000009917 | 14 | - | 40712179 | 40715025 | 312 | annot_exons |
| novel_circ_006366 | ENSSSCG00000011192 | 13 | - | 2810084 | 2828036 | 355 | annot_exons |
| novel_circ_006367 | ENSSSCG00000028148 | X | - | 28352645 | 28383695 | 774 | annot_exons |
| novel_circ_006368 | ENSSSCG00000005366 | 1 | + | 2.39E+08 | 2.39E+08 | 9092 | exon_intron |
| novel_circ_006369 | ENSSSCG00000001852 | 7 | - | 55659526 | 55659913 | 305 | annot_exons |
| novel_circ_006370 | ENSSSCG00000027973 | 6 | - | 13517890 | 13522471 | 373 | annot_exons |
| novel_circ_006371 | ENSSSCG00000007721 | 3 | + | 11771861 | 11777751 | 384 | exon_intron |
| novel_circ_006372 | ENSSSCG00000001064 | 7 | + | 12209078 | 12266794 | 46081 | exon_intron |
| novel_circ_006373 | ENSSSCG00000015196 | 9 | - | 51880421 | 51880698 | 278 | one_exon |
| novel_circ_006374 | ENSSSCG00000020858 | 7 | - | 13567187 | 13567849 | 325 | annot_exons |
| novel_circ_006375 | ENSSSCG00000009134 | 8 | - | 1.12E+08 | 1.12E+08 | 246 | annot_exons |
| novel_circ_006376 | ENSSSCG00000014199 | 2 | + | 1.16E+08 | 1.16E+08 | 352 | annot_exons |
| novel_circ_006377 | ENSSSCG00000011765 | 13 | + | 1.18E+08 | 1.18E+08 | 725 | annot_exons |
| novel_circ_006378 | ENSSSCG00000000119 | 5 | - | 9975420 | 9980383 | 996 | annot_exons |
| novel_circ_006379 | ENSSSCG00000010317 | 14 | - | 77601802 | 77603556 | 1755 | exon_intron |
| novel_circ_006380 | ENSSSCG00000014913 | 9 | - | 19852498 | 19861801 | 560 | annot_exons |
| novel_circ_006381 | ENSSSCG00000004626 | 1 | - | 1.2E+08 | 1.2E+08 | 239 | annot_exons |
| novel_circ_006382 | ENSSSCG00000032176 | 12 | + | 14478611 | 14483274 | 438 | annot_exons |
| novel_circ_006383 | ENSSSCG00000015428 | 9 | - | 1.05E+08 | 1.05E+08 | 452 | annot_exons |
| novel_circ_006384 | ENSSSCG00000033971 | 1 | - | 1.49E+08 | 1.49E+08 | 551 | annot_exons |
| novel_circ_006385 | ENSSSCG00000031639 | 6 | + | 87924214 | 87926357 | 247 | annot_exons |
| novel_circ_006386 | ENSSSCG00000032668 | 6 | + | 54662913 | 54664571 | 730 | annot_exons |
| novel_circ_006387 | ENSSSCG00000032711 | 6 | - | 49408762 | 49413731 | 3728 | exon_intron |
| novel_circ_006388 | ENSSSCG00000037065 | 3 | + | 32478194 | 32478840 | 647 | intronic |
| novel_circ_006389 | ENSSSCG00000006274 | 4 | + | 79784917 | 79825948 | 3574 | annot_exons |
| novel_circ_006390 | ENSSSCG00000039171 | 3 | - | 28988595 | 28991023 | 1158 | annot_exons |
| novel_circ_006391 | ENSSSCG00000029227 | 8 | - | 11712865 | 11716325 | 207 | annot_exons |
| novel_circ_006392 | ENSSSCG00000024486 | 17 | + | 11903520 | 11904781 | 206 | annot_exons |
| novel_circ_006393 | ENSSSCG00000002540 | AEMK02000452.1 | + | 780716 | 785341 | 474 | annot_exons |
| novel_circ_006394 | ENSSSCG00000009994 | 14 | + | 46956297 | 46973910 | 2041 | exon_intron |
| novel_circ_006395 | ENSSSCG00000028148 | X | - | 27740014 | 27743008 | 2995 | intronic |
| novel_circ_006396 | ENSSSCG00000015859 | 15 | + | 59387358 | 59393060 | 734 | annot_exons |
| novel_circ_006397 | ENSSSCG00000000890 | 5 | - | 86711988 | 86735350 | 1327 | annot_exons |
| novel_circ_006398 | ENSSSCG00000003917 | 6 | + | 1.66E+08 | 1.66E+08 | 633 | annot_exons |
| novel_circ_006399 | ENSSSCG00000004281 | 1 | + | 52645934 | 52646411 | 478 | intronic |
| novel_circ_006400 | ENSSSCG00000016568 | 18 | - | 19291571 | 19294817 | 198 | annot_exons |
| novel_circ_006401 | ENSSSCG00000015917 | 15 | + | 73732427 | 73733239 | 813 | one_exon |
| novel_circ_006402 | ENSSSCG00000031462 | 6 | - | 12557721 | 12559346 | 1626 | one_exon |
| novel_circ_006403 | ENSSSCG00000032674 | 6 | - | 81277209 | 81280378 | 376 | annot_exons |
| novel_circ_006404 | ENSSSCG00000039336 | 12 | - | 11925775 | 11926287 | 513 | one_exon |
| novel_circ_006405 | ENSSSCG00000025565 | 3 | - | 28226496 | 28227963 | 269 | annot_exons |
| novel_circ_006406 | ENSSSCG00000001499 | 7 | + | 29133161 | 29165288 | 7669 | annot_exons |
| novel_circ_006407 | ENSSSCG00000013405 | 2 | - | 48678643 | 48679022 | 380 | antisense |
| novel_circ_006408 | ENSSSCG00000016842 | 16 | + | 22246390 | 22248632 | 309 | annot_exons |
| novel_circ_006409 | ENSSSCG00000035196 | 4 | - | 1.25E+08 | 1.25E+08 | 545 | annot_exons |
| novel_circ_006410 | ENSSSCG00000024784 | 10 | - | 23533949 | 23534645 | 72 | annot_exons |
| novel_circ_006411 | ENSSSCG00000009050 | 8 | + | 85357610 | 85358219 | 236 | annot_exons |
| novel_circ_006412 | ENSSSCG00000016157 | 15 | - | 1.13E+08 | 1.13E+08 | 2898 | annot_exons |
| novel_circ_006413 | ENSSSCG00000012911 | 2 | - | 5097538 | 5097730 | 193 | one_exon |
| novel_circ_006414 | ENSSSCG00000006083 | 4 | - | 38961991 | 38967502 | 296 | annot_exons |
| novel_circ_006415 | ENSSSCG00000015743 | 15 | - | 31417943 | 31435861 | 537 | annot_exons |
| novel_circ_006416 | ENSSSCG00000006078 | 4 | - | 38627995 | 38629395 | 433 | annot_exons |
| novel_circ_006417 | ENSSSCG00000012084 | 13 | - | 2.05E+08 | 2.05E+08 | 349 | annot_exons |
| novel_circ_006418 | ENSSSCG00000009528 | 11 | + | 70961245 | 70976889 | 1014 | annot_exons |
| novel_circ_006419 | ENSSSCG00000011022 | 10 | + | 41410146 | 41410634 | 489 | intronic |
| novel_circ_006420 | ENSSSCG00000026753 | 13 | + | 1.35E+08 | 1.35E+08 | 256 | annot_exons |
| novel_circ_006421 | ENSSSCG00000005356 | 1 | + | 2.39E+08 | 2.39E+08 | 515 | annot_exons |
| novel_circ_006422 | ENSSSCG00000028148 | X | + | 28133063 | 28133325 | 263 | antisense |
| novel_circ_006423 | ENSSSCG00000009021 | 8 | + | 78090611 | 78097811 | 601 | annot_exons |
| novel_circ_006424 | ENSSSCG00000001641 | 7 | + | 37769606 | 37778393 | 637 | annot_exons |
| novel_circ_006425 | ENSSSCG00000004318 | 1 | - | 57392229 | 57393736 | 315 | annot_exons |
| novel_circ_006426 | ENSSSCG00000005243 | 1 | + | 2.22E+08 | 2.22E+08 | 346 | annot_exons |
| novel_circ_006427 | ENSSSCG00000024813 | 12 | - | 15263850 | 15269630 | 214 | annot_exons |
| novel_circ_006428 | NA | 15 | + | 84298817 | 84299236 | 420 | intergenic |
| novel_circ_006429 | ENSSSCG00000006508 | 4 | + | 94405984 | 94438667 | 525 | annot_exons |
| novel_circ_006430 | ENSSSCG00000017268 | 12 | - | 13221806 | 13262433 | 40628 | antisense |
| novel_circ_006431 | ENSSSCG00000008068 | 3 | - | 41973352 | 41978180 | 291 | annot_exons |
| novel_circ_006432 | ENSSSCG00000015777 | 15 | + | 45021868 | 45022649 | 242 | annot_exons |
| novel_circ_006433 | ENSSSCG00000005257 | 1 | - | 2.24E+08 | 2.24E+08 | 484 | annot_exons |
| novel_circ_006434 | ENSSSCG00000009994 | 14 | + | 46965261 | 46985309 | 576 | annot_exons |
| novel_circ_006435 | ENSSSCG00000009839 | 14 | + | 33115205 | 33121520 | 323 | annot_exons |
| novel_circ_006436 | ENSSSCG00000032622 | 14 | + | 6671194 | 6673572 | 465 | annot_exons |
| novel_circ_006437 | ENSSSCG00000006098 | 4 | - | 41968917 | 41969658 | 359 | annot_exons |
| novel_circ_006438 | ENSSSCG00000033527 | 6 | - | 37286137 | 37286381 | 245 | antisense |
| novel_circ_006439 | ENSSSCG00000014113 | 2 | - | 88144038 | 88147594 | 349 | annot_exons |
| novel_circ_006440 | ENSSSCG00000010465 | 14 | + | 1.03E+08 | 1.03E+08 | 1084 | annot_exons |
| novel_circ_006441 | ENSSSCG00000015958 | 15 | + | 78610675 | 78620652 | 797 | annot_exons |
| novel_circ_006442 | ENSSSCG00000032049 | 12 | - | 50136982 | 50139045 | 367 | annot_exons |
| novel_circ_006443 | NA | 4 | + | 7380004 | 7381369 | 1366 | intergenic |
| novel_circ_006444 | ENSSSCG00000026940 | 15 | + | 1.05E+08 | 1.05E+08 | 246 | annot_exons |
| novel_circ_006445 | ENSSSCG00000001065 | 7 | - | 12457322 | 12460723 | 3402 | exon_intron |
| novel_circ_006446 | ENSSSCG00000006346 | 4 | - | 88758198 | 88759728 | 278 | annot_exons |
| novel_circ_006447 | ENSSSCG00000038711 | 2 | - | 8982261 | 8983263 | 1003 | antisense |
| novel_circ_006448 | ENSSSCG00000021129 | 5 | + | 78512681 | 78513245 | 356 | annot_exons |
| novel_circ_006449 | ENSSSCG00000009298 | 11 | + | 4104218 | 4118265 | 328 | annot_exons |
| novel_circ_006450 | ENSSSCG00000014116 | 2 | - | 88676022 | 88677083 | 418 | annot_exons |
| novel_circ_006451 | ENSSSCG00000017395 | 12 | + | 20233514 | 20238667 | 488 | annot_exons |
| novel_circ_006452 | ENSSSCG00000034844 | 14 | - | 55825115 | 55837033 | 651 | annot_exons |
| novel_circ_006453 | ENSSSCG00000016082 | 15 | - | 1.02E+08 | 1.02E+08 | 744 | annot_exons |
| novel_circ_006454 | ENSSSCG00000001534 | 7 | - | 30956952 | 30960485 | 3534 | antisense |
| novel_circ_006455 | ENSSSCG00000017159 | 12 | + | 2401755 | 2405707 | 834 | annot_exons |
| novel_circ_006456 | ENSSSCG00000025565 | 3 | - | 28242597 | 28243686 | 291 | annot_exons |
| novel_circ_006457 | NA | 15 | - | 84271967 | 84272172 | 206 | intergenic |
| novel_circ_006458 | ENSSSCG00000014285 | 2 | + | 1.35E+08 | 1.35E+08 | 1216 | antisense |
| novel_circ_006459 | ENSSSCG00000011025 | 10 | + | 42063616 | 42079536 | 15921 | intronic |
| novel_circ_006460 | ENSSSCG00000014012 | 2 | + | 78314628 | 78322594 | 716 | annot_exons |
| novel_circ_006461 | ENSSSCG00000010303 | 14 | + | 76458256 | 76458477 | 222 | antisense |
| novel_circ_006462 | NA | 4 | + | 95746863 | 95747066 | 204 | intergenic |
| novel_circ_006463 | ENSSSCG00000015689 | 15 | + | 15859697 | 15867100 | 356 | annot_exons |
| novel_circ_006464 | ENSSSCG00000007721 | 3 | + | 11771884 | 11777751 | 361 | annot_exons |
| novel_circ_006465 | ENSSSCG00000016397 | 15 | + | 743708 | 745925 | 291 | annot_exons |
| novel_circ_006466 | ENSSSCG00000000488 | 5 | + | 33129958 | 33132527 | 234 | annot_exons |
| novel_circ_006467 | ENSSSCG00000028033 | 14 | + | 932379 | 935525 | 346 | annot_exons |
| novel_circ_006468 | ENSSSCG00000035676 | 16 | - | 71262565 | 71263268 | 256 | annot_exons |
| novel_circ_006469 | ENSSSCG00000011074 | 10 | + | 50665596 | 50665836 | 164 | annot_exons |
| novel_circ_006470 | ENSSSCG00000005314 | 1 | + | 2.36E+08 | 2.36E+08 | 168 | antisense |
| novel_circ_006471 | ENSSSCG00000009042 | 8 | + | 83278177 | 83279596 | 220 | annot_exons |
| novel_circ_006472 | ENSSSCG00000014304 | 2 | + | 1.37E+08 | 1.37E+08 | 674 | annot_exons |
| novel_circ_006473 | ENSSSCG00000013865 | 2 | - | 60894107 | 60906050 | 491 | annot_exons |
| novel_circ_006474 | ENSSSCG00000017063 | 16 | - | 66756032 | 66756987 | 956 | intronic |
| novel_circ_006475 | ENSSSCG00000004064 | 1 | - | 10070672 | 10099544 | 261 | annot_exons |
| novel_circ_006476 | ENSSSCG00000038220 | 1 | + | 2.74E+08 | 2.74E+08 | 597 | annot_exons |
| novel_circ_006477 | ENSSSCG00000020663 | 18 | + | 5236417 | 5241617 | 2228 | annot_exons |
| novel_circ_006478 | ENSSSCG00000007336 | 17 | - | 40625165 | 40629608 | 4444 | antisense |
| novel_circ_006479 | ENSSSCG00000013022 | 2 | + | 7414294 | 7420437 | 1634 | annot_exons |
| novel_circ_006480 | ENSSSCG00000003488 | 6 | - | 77681952 | 77682317 | 246 | annot_exons |
| novel_circ_006481 | ENSSSCG00000024481 | 4 | - | 1.01E+08 | 1.01E+08 | 250 | annot_exons |
| novel_circ_006482 | ENSSSCG00000036139 | 4 | + | 34835874 | 34850285 | 855 | annot_exons |
| novel_circ_006483 | ENSSSCG00000004358 | 1 | - | 67602244 | 67602480 | 237 | intronic |
| novel_circ_006484 | ENSSSCG00000041457 | 1 | - | 1.94E+08 | 1.94E+08 | 31389 | exon_intron |
| novel_circ_006485 | ENSSSCG00000036659 | 3 | - | 16925439 | 16930751 | 493 | annot_exons |
| novel_circ_006486 | ENSSSCG00000011013 | 10 | + | 39984146 | 39991160 | 336 | annot_exons |
| novel_circ_006487 | ENSSSCG00000004478 | 1 | - | 90046152 | 90053440 | 498 | annot_exons |
| novel_circ_006488 | ENSSSCG00000023112 | 8 | - | 31024321 | 31025401 | 253 | annot_exons |
| novel_circ_006489 | ENSSSCG00000010928 | 10 | - | 24774960 | 24781769 | 783 | annot_exons |
| novel_circ_006490 | ENSSSCG00000001700 | 7 | + | 39230659 | 39231103 | 198 | annot_exons |
| novel_circ_006491 | ENSSSCG00000010144 | 14 | - | 54696668 | 54709634 | 4043 | annot_exons |
| novel_circ_006492 | ENSSSCG00000005582 | 1 | - | 2.64E+08 | 2.64E+08 | 435 | annot_exons |
| novel_circ_006493 | ENSSSCG00000003218 | 6 | + | 55302098 | 55302788 | 589 | exon_intron |
| novel_circ_006494 | ENSSSCG00000005073 | 1 | + | 1.87E+08 | 1.87E+08 | 446 | annot_exons |
| novel_circ_006495 | ENSSSCG00000010190 | 14 | + | 60414092 | 60414612 | 399 | annot_exons |
| novel_circ_006496 | ENSSSCG00000004318 | 1 | - | 57376845 | 57391008 | 1457 | annot_exons |
| novel_circ_006497 | NA | 7 | + | 13138271 | 13138507 | 237 | intergenic |
| novel_circ_006498 | ENSSSCG00000007872 | 3 | - | 27292824 | 27293334 | 511 | one_exon |
| novel_circ_006499 | ENSSSCG00000009134 | 8 | - | 1.12E+08 | 1.12E+08 | 286 | annot_exons |
| novel_circ_006500 | ENSSSCG00000037530 | 14 | + | 1.32E+08 | 1.32E+08 | 445 | annot_exons |
| novel_circ_006501 | ENSSSCG00000005725 | 1 | - | 2.72E+08 | 2.72E+08 | 1776 | annot_exons |
| novel_circ_006502 | ENSSSCG00000000900 | 5 | - | 88042137 | 88046593 | 295 | annot_exons |
| novel_circ_006503 | ENSSSCG00000006196 | 4 | + | 65508038 | 65557837 | 582 | annot_exons |
| novel_circ_006504 | ENSSSCG00000012613 | X | - | 98262075 | 98263484 | 336 | annot_exons |
| novel_circ_006505 | ENSSSCG00000033692 | 14 | + | 55777475 | 55802841 | 581 | annot_exons |
| novel_circ_006506 | NA | 15 | - | 84251742 | 84252516 | 775 | intergenic |
| novel_circ_006507 | ENSSSCG00000004328 | 1 | - | 57706687 | 57708581 | 672 | annot_exons |
| novel_circ_006508 | ENSSSCG00000015908 | 15 | - | 72456954 | 72465336 | 489 | annot_exons |
| novel_circ_006509 | ENSSSCG00000003718 | 6 | + | 1.11E+08 | 1.11E+08 | 1250 | annot_exons |
| novel_circ_006510 | ENSSSCG00000038913 | 14 | - | 71219663 | 71239477 | 796 | annot_exons |
| novel_circ_006511 | ENSSSCG00000022982 | 9 | + | 73100284 | 73102060 | 237 | annot_exons |
| novel_circ_006512 | ENSSSCG00000017506 | 12 | + | 22948544 | 22950945 | 223 | annot_exons |
| novel_circ_006513 | ENSSSCG00000010358 | 14 | + | 87706284 | 87707473 | 302 | annot_exons |
| novel_circ_006514 | ENSSSCG00000002296 | 7 | - | 91436717 | 91438716 | 261 | annot_exons |
| novel_circ_006515 | ENSSSCG00000045048 | 14 | - | 41684734 | 41691763 | 7030 | antisense |
| novel_circ_006516 | ENSSSCG00000007917 | 3 | - | 34985848 | 35008697 | 281 | annot_exons |
| novel_circ_006517 | ENSSSCG00000022982 | 9 | + | 73155846 | 73168561 | 599 | annot_exons |
| novel_circ_006518 | ENSSSCG00000005959 | 4 | + | 10616624 | 10620799 | 228 | annot_exons |
| novel_circ_006519 | ENSSSCG00000011137 | 10 | + | 65187800 | 65188643 | 331 | annot_exons |
| novel_circ_006520 | ENSSSCG00000034569 | 2 | + | 51346346 | 51370259 | 7979 | exon_intron |
| novel_circ_006521 | ENSSSCG00000017186 | 12 | + | 5288901 | 5289968 | 348 | annot_exons |
| novel_circ_006522 | ENSSSCG00000000801 | 5 | + | 75401851 | 75402550 | 700 | one_exon |
| novel_circ_006523 | ENSSSCG00000004725 | 1 | + | 1.29E+08 | 1.29E+08 | 1232 | annot_exons |
| novel_circ_006524 | ENSSSCG00000009134 | 8 | - | 1.12E+08 | 1.12E+08 | 412 | annot_exons |
| novel_circ_006525 | ENSSSCG00000002340 | 7 | + | 96490724 | 96491038 | 222 | annot_exons |
| novel_circ_006526 | ENSSSCG00000032622 | 14 | + | 6766438 | 6771753 | 486 | annot_exons |
| novel_circ_006527 | ENSSSCG00000030608 | 5 | - | 87896929 | 87899774 | 536 | annot_exons |
| novel_circ_006528 | ENSSSCG00000039338 | 11 | + | 25937034 | 25941525 | 273 | annot_exons |
| novel_circ_006529 | ENSSSCG00000009142 | 8 | - | 1.13E+08 | 1.13E+08 | 6554 | exon_intron |
| novel_circ_006530 | ENSSSCG00000011532 | 13 | - | 60992625 | 60993620 | 238 | annot_exons |
| novel_circ_006531 | ENSSSCG00000027857 | 2 | + | 1.23E+08 | 1.23E+08 | 801 | annot_exons |
| novel_circ_006532 | ENSSSCG00000009018 | 8 | - | 77853741 | 77865869 | 12129 | antisense |
| novel_circ_006533 | ENSSSCG00000009910 | 14 | + | 40481309 | 40502476 | 1154 | annot_exons |
| novel_circ_006534 | ENSSSCG00000009134 | 8 | - | 1.12E+08 | 1.12E+08 | 438 | annot_exons |
| novel_circ_006535 | ENSSSCG00000006872 | 4 | - | 1.18E+08 | 1.18E+08 | 653 | annot_exons |
| novel_circ_006536 | ENSSSCG00000032043 | 6 | + | 1.46E+08 | 1.46E+08 | 196 | annot_exons |
| novel_circ_006537 | ENSSSCG00000016412 | 18 | + | 1829882 | 1881411 | 578 | annot_exons |
| novel_circ_006538 | ENSSSCG00000004551 | 1 | - | 1.08E+08 | 1.08E+08 | 874 | one_exon |
| novel_circ_006539 | ENSSSCG00000017791 | 12 | - | 45860862 | 45887992 | 10138 | exon_intron |
| novel_circ_006540 | ENSSSCG00000024481 | 4 | - | 1.01E+08 | 1.01E+08 | 1950 | annot_exons |
| novel_circ_006541 | ENSSSCG00000010144 | 14 | - | 54704317 | 54709634 | 3547 | annot_exons |
| novel_circ_006542 | ENSSSCG00000027973 | 6 | - | 13496003 | 13505999 | 632 | annot_exons |
| novel_circ_006543 | ENSSSCG00000012435 | X | - | 61915993 | 61935991 | 570 | annot_exons |
| novel_circ_006544 | ENSSSCG00000016541 | 18 | + | 14904679 | 14906161 | 186 | annot_exons |
| novel_circ_006545 | ENSSSCG00000002628 | 7 | - | 46690001 | 46701207 | 521 | annot_exons |
| novel_circ_006546 | ENSSSCG00000016026 | 15 | + | 91512801 | 91514202 | 373 | annot_exons |
| novel_circ_006547 | ENSSSCG00000001825 | 7 | - | 53717427 | 53729722 | 640 | annot_exons |
| novel_circ_006548 | ENSSSCG00000005785 | 1 | + | 1.46E+08 | 1.46E+08 | 421 | annot_exons |
| novel_circ_006549 | ENSSSCG00000004064 | 1 | - | 10099395 | 10175846 | 345 | annot_exons |
| novel_circ_006550 | ENSSSCG00000011340 | 13 | - | 30544508 | 30564659 | 451 | annot_exons |
| novel_circ_006551 | ENSSSCG00000003776 | 6 | - | 1.38E+08 | 1.38E+08 | 563 | annot_exons |
| novel_circ_006552 | ENSSSCG00000021184 | 4 | - | 46892870 | 46897315 | 421 | annot_exons |
| novel_circ_006553 | ENSSSCG00000004927 | 1 | - | 1.63E+08 | 1.63E+08 | 315 | annot_exons |
| novel_circ_006554 | ENSSSCG00000022830 | 15 | - | 1.13E+08 | 1.13E+08 | 1259 | annot_exons |
| novel_circ_006555 | ENSSSCG00000011394 | 13 | + | 32554728 | 32560025 | 587 | annot_exons |
| novel_circ_006556 | ENSSSCG00000016960 | 16 | + | 47522393 | 47526925 | 329 | annot_exons |
| novel_circ_006557 | ENSSSCG00000011394 | 13 | + | 32471840 | 32478114 | 1439 | annot_exons |
| novel_circ_006558 | ENSSSCG00000040220 | 6 | + | 1.36E+08 | 1.36E+08 | 249 | annot_exons |
| novel_circ_006559 | ENSSSCG00000008513 | 3 | - | 1.07E+08 | 1.07E+08 | 1857 | annot_exons |
| novel_circ_006560 | ENSSSCG00000000955 | 5 | - | 2675422 | 2701290 | 378 | annot_exons |
| novel_circ_006561 | ENSSSCG00000013879 | 2 | - | 60208144 | 60209136 | 312 | annot_exons |
| novel_circ_006562 | ENSSSCG00000011107 | 10 | + | 58021216 | 58042335 | 425 | annot_exons |
| novel_circ_006563 | ENSSSCG00000003093 | 6 | - | 52157014 | 52159344 | 511 | annot_exons |
| novel_circ_006564 | ENSSSCG00000011453 | 13 | - | 34937219 | 34943897 | 1345 | annot_exons |
| novel_circ_006565 | ENSSSCG00000003983 | 6 | - | 1.71E+08 | 1.71E+08 | 279 | annot_exons |
| novel_circ_006566 | ENSSSCG00000010238 | 14 | + | 71491530 | 71493586 | 546 | annot_exons |
| novel_circ_006567 | ENSSSCG00000015883 | 15 | + | 66664434 | 66683291 | 1792 | annot_exons |
| novel_circ_006568 | ENSSSCG00000011312 | 13 | + | 28557260 | 28621153 | 655 | annot_exons |
| novel_circ_006569 | ENSSSCG00000007907 | 3 | + | 33785887 | 33787969 | 338 | annot_exons |
| novel_circ_006570 | ENSSSCG00000021166 | 5 | - | 78034615 | 78040700 | 464 | annot_exons |
| novel_circ_006571 | ENSSSCG00000009978 | 14 | + | 46469748 | 46470460 | 713 | antisense |
| novel_circ_006572 | ENSSSCG00000016005 | 15 | - | 84830996 | 84870609 | 5055 | annot_exons |
| novel_circ_006573 | NA | 1 | - | 54180177 | 54226241 | 46065 | intergenic |
| novel_circ_006574 | ENSSSCG00000004576 | 1 | + | 1.11E+08 | 1.11E+08 | 32643 | intronic |
| novel_circ_006575 | ENSSSCG00000039591 | 17 | - | 46910525 | 46911614 | 275 | annot_exons |
| novel_circ_006576 | ENSSSCG00000010143 | 14 | - | 54586423 | 54630518 | 1694 | annot_exons |
| novel_circ_006577 | ENSSSCG00000003766 | 6 | + | 1.35E+08 | 1.35E+08 | 258 | antisense |
| novel_circ_006578 | ENSSSCG00000011678 | 13 | - | 83151533 | 83152060 | 206 | annot_exons |
| novel_circ_006579 | ENSSSCG00000023084 | 13 | - | 66540552 | 66543247 | 503 | annot_exons |
| novel_circ_006580 | ENSSSCG00000006775 | 4 | - | 1.08E+08 | 1.08E+08 | 271 | annot_exons |
| novel_circ_006581 | ENSSSCG00000015948 | 15 | + | 77818746 | 77831758 | 4179 | exon_intron |
| novel_circ_006582 | ENSSSCG00000015050 | 9 | - | 41365582 | 41366657 | 263 | annot_exons |
| novel_circ_006583 | ENSSSCG00000001971 | 7 | + | 68401378 | 68406779 | 503 | annot_exons |
| novel_circ_006584 | NA | 13 | - | 43400493 | 43401148 | 656 | intergenic |
| novel_circ_006585 | ENSSSCG00000000570 | 5 | + | 51097637 | 51112176 | 910 | annot_exons |
| novel_circ_006586 | ENSSSCG00000011394 | 13 | + | 32471840 | 32506478 | 1513 | annot_exons |
| novel_circ_006587 | ENSSSCG00000005466 | 1 | - | 2.53E+08 | 2.53E+08 | 4117 | annot_exons |
| novel_circ_006588 | ENSSSCG00000021072 | 1 | - | 95602074 | 95603018 | 945 | one_exon |
| novel_circ_006589 | ENSSSCG00000004901 | 1 | + | 1.59E+08 | 1.59E+08 | 367 | annot_exons |
| novel_circ_006590 | ENSSSCG00000026004 | 5 | + | 29751201 | 29779626 | 26792 | exon_intron |
| novel_circ_006591 | ENSSSCG00000002003 | 7 | - | 75151742 | 75151919 | 178 | one_exon |
| novel_circ_006592 | ENSSSCG00000006995 | 17 | - | 5732179 | 5733109 | 178 | annot_exons |
| novel_circ_006593 | ENSSSCG00000011332 | 13 | - | 29953610 | 29960782 | 427 | annot_exons |
| novel_circ_006594 | ENSSSCG00000030560 | 1 | - | 1.38E+08 | 1.38E+08 | 546 | one_exon |
| novel_circ_006595 | ENSSSCG00000004032 | 1 | + | 5748740 | 5750270 | 1531 | intronic |
| novel_circ_006596 | ENSSSCG00000006872 | 4 | - | 1.18E+08 | 1.18E+08 | 1054 | annot_exons |
| novel_circ_006597 | ENSSSCG00000012884 | 2 | - | 4428979 | 4449449 | 814 | annot_exons |
| novel_circ_006598 | ENSSSCG00000024696 | 15 | + | 1.11E+08 | 1.11E+08 | 211 | annot_exons |
| novel_circ_006599 | NA | 15 | - | 84479026 | 84479562 | 537 | intergenic |
| novel_circ_006600 | ENSSSCG00000008513 | 3 | - | 1.07E+08 | 1.07E+08 | 647 | annot_exons |
| novel_circ_006601 | ENSSSCG00000040631 | 14 | + | 4113663 | 4122496 | 462 | annot_exons |
| novel_circ_006602 | ENSSSCG00000000806 | 5 | - | 76855469 | 76857881 | 242 | annot_exons |
| novel_circ_006603 | ENSSSCG00000035747 | 3 | - | 1.07E+08 | 1.07E+08 | 404 | annot_exons |
| novel_circ_006604 | ENSSSCG00000006101 | 4 | + | 42349369 | 42360992 | 505 | annot_exons |
| novel_circ_006605 | ENSSSCG00000001479 | 7 | + | 25658626 | 25679321 | 498 | annot_exons |
| novel_circ_006606 | ENSSSCG00000040887 | 6 | - | 34747657 | 34756041 | 452 | annot_exons |
| novel_circ_006607 | ENSSSCG00000016033 | 15 | + | 93222405 | 93243693 | 750 | annot_exons |
| novel_circ_006608 | ENSSSCG00000008803 | 8 | - | 33422988 | 33432916 | 306 | annot_exons |
| novel_circ_006609 | ENSSSCG00000014364 | 2 | + | 1.42E+08 | 1.42E+08 | 782 | annot_exons |
| novel_circ_006610 | ENSSSCG00000020717 | 8 | - | 77359535 | 77365218 | 248 | annot_exons |
| novel_circ_006611 | ENSSSCG00000002410 | 7 | + | 1.03E+08 | 1.03E+08 | 2048 | antisense |
| novel_circ_006612 | ENSSSCG00000009479 | 11 | - | 51389930 | 51394378 | 345 | annot_exons |
| novel_circ_006613 | ENSSSCG00000017082 | 16 | + | 71369950 | 71374075 | 385 | annot_exons |
| novel_circ_006614 | ENSSSCG00000027278 | 13 | - | 1.63E+08 | 1.63E+08 | 371 | annot_exons |
| novel_circ_006615 | ENSSSCG00000040457 | 12 | - | 60182783 | 60184718 | 381 | annot_exons |
| novel_circ_006616 | ENSSSCG00000023710 | 3 | + | 58544737 | 58544914 | 178 | one_exon |
| novel_circ_006617 | ENSSSCG00000010987 | 10 | + | 32850884 | 32859397 | 305 | annot_exons |
| novel_circ_006618 | ENSSSCG00000015859 | 15 | + | 59336611 | 59371599 | 1194 | annot_exons |
| novel_circ_006619 | ENSSSCG00000000366 | 5 | - | 21267344 | 21267784 | 388 | annot_exons |
| novel_circ_006620 | ENSSSCG00000030679 | 1 | + | 1.75E+08 | 1.75E+08 | 750 | annot_exons |
| novel_circ_006621 | ENSSSCG00000031631 | 6 | - | 93632261 | 93633202 | 942 | intronic |
| novel_circ_006622 | ENSSSCG00000038973 | 5 | + | 1.02E+08 | 1.02E+08 | 555 | annot_exons |
| novel_circ_006623 | ENSSSCG00000007805 | 3 | + | 18553400 | 18555003 | 465 | annot_exons |
| novel_circ_006624 | ENSSSCG00000035493 | 11 | + | 2393249 | 2393374 | 126 | antisense |
| novel_circ_006625 | ENSSSCG00000006850 | 4 | - | 1.11E+08 | 1.11E+08 | 315 | annot_exons |
| novel_circ_006626 | ENSSSCG00000007979 | 3 | + | 41460222 | 41468073 | 525 | annot_exons |
| novel_circ_006627 | ENSSSCG00000030480 | 13 | + | 2.01E+08 | 2.01E+08 | 439 | annot_exons |
| novel_circ_006628 | ENSSSCG00000008504 | 3 | - | 1.04E+08 | 1.04E+08 | 381 | annot_exons |
| novel_circ_006629 | ENSSSCG00000012659 | X | - | 1.07E+08 | 1.07E+08 | 350 | annot_exons |
| novel_circ_006630 | ENSSSCG00000032312 | 1 | + | 1.64E+08 | 1.64E+08 | 390 | annot_exons |
| novel_circ_006631 | NA | 8 | - | 76103989 | 76104298 | 310 | intergenic |
| novel_circ_006632 | ENSSSCG00000017904 | 12 | + | 51955607 | 51959342 | 3736 | antisense |
| novel_circ_006633 | ENSSSCG00000005030 | 1 | - | 1.82E+08 | 1.82E+08 | 828 | annot_exons |
| novel_circ_006634 | ENSSSCG00000022240 | X | + | 20809488 | 20820112 | 748 | annot_exons |
| novel_circ_006635 | NA | 7 | + | 68168858 | 68220926 | 52069 | intergenic |
| novel_circ_006636 | ENSSSCG00000015569 | 9 | + | 1.26E+08 | 1.26E+08 | 776 | annot_exons |
| novel_circ_006637 | ENSSSCG00000008312 | 3 | - | 71165102 | 71179271 | 427 | annot_exons |
| novel_circ_006638 | ENSSSCG00000010190 | 14 | + | 60415377 | 60415663 | 287 | one_exon |
| novel_circ_006639 | ENSSSCG00000021250 | 18 | - | 40507148 | 40517359 | 818 | annot_exons |
| novel_circ_006640 | ENSSSCG00000032531 | 3 | + | 26685470 | 26697117 | 1172 | annot_exons |
| novel_circ_006641 | ENSSSCG00000003107 | 6 | + | 52754997 | 52774396 | 4016 | annot_exons |
| novel_circ_006642 | ENSSSCG00000010253 | 14 | + | 72426644 | 72427752 | 380 | annot_exons |
| novel_circ_006643 | ENSSSCG00000015789 | 15 | + | 46356116 | 46363704 | 752 | annot_exons |
| novel_circ_006644 | ENSSSCG00000021712 | 8 | - | 1.31E+08 | 1.31E+08 | 377 | annot_exons |
| novel_circ_006645 | ENSSSCG00000022034 | 10 | + | 18699626 | 18699917 | 292 | antisense |
| novel_circ_006646 | ENSSSCG00000028148 | X | - | 27821553 | 27821717 | 165 | one_exon |
| novel_circ_006647 | NA | 15 | + | 84293285 | 84293882 | 598 | intergenic |
| novel_circ_006648 | ENSSSCG00000004460 | 1 | + | 84036049 | 84038578 | 401 | annot_exons |
| novel_circ_006649 | ENSSSCG00000010358 | 14 | + | 87682482 | 87714147 | 1368 | annot_exons |
| novel_circ_006650 | ENSSSCG00000004106 | 1 | + | 16307508 | 16307993 | 290 | annot_exons |
| novel_circ_006651 | ENSSSCG00000016157 | 15 | - | 1.13E+08 | 1.13E+08 | 297 | one_exon |
| novel_circ_006652 | ENSSSCG00000009244 | 8 | + | 1.35E+08 | 1.35E+08 | 273 | annot_exons |
| novel_circ_006653 | ENSSSCG00000009994 | 14 | + | 46965261 | 46973910 | 293 | annot_exons |
| novel_circ_006654 | ENSSSCG00000035956 | 10 | - | 25600746 | 25606516 | 358 | annot_exons |
| novel_circ_006655 | ENSSSCG00000021042 | 13 | - | 2.03E+08 | 2.03E+08 | 694 | annot_exons |
| novel_circ_006656 | ENSSSCG00000027331 | 15 | + | 1.37E+08 | 1.37E+08 | 442 | antisense |
| novel_circ_006657 | NA | 15 | + | 84250240 | 84252632 | 2393 | intergenic |
| novel_circ_006658 | ENSSSCG00000011218 | 13 | - | 13987176 | 14004211 | 718 | annot_exons |
| novel_circ_006659 | ENSSSCG00000004985 | 1 | + | 1.7E+08 | 1.7E+08 | 492 | annot_exons |
| novel_circ_006660 | ENSSSCG00000016845 | 16 | - | 22524457 | 22528282 | 483 | annot_exons |
| novel_circ_006661 | ENSSSCG00000008943 | 8 | + | 68022433 | 68061384 | 235 | annot_exons |
| novel_circ_006662 | ENSSSCG00000040728 | 4 | + | 64223154 | 64252140 | 494 | annot_exons |
| novel_circ_006663 | ENSSSCG00000011162 | 10 | + | 68684614 | 68688441 | 320 | annot_exons |
| novel_circ_006664 | ENSSSCG00000001732 | 7 | - | 43610581 | 43618755 | 1059 | annot_exons |
| novel_circ_006665 | ENSSSCG00000022636 | 5 | + | 42814872 | 42843975 | 575 | annot_exons |
| novel_circ_006666 | ENSSSCG00000008389 | 3 | - | 81002969 | 81003518 | 206 | annot_exons |
| novel_circ_006667 | ENSSSCG00000010170 | 14 | - | 58800592 | 58813278 | 347 | annot_exons |
| novel_circ_006668 | ENSSSCG00000004570 | 1 | - | 1.09E+08 | 1.09E+08 | 806 | intronic |
| novel_circ_006669 | NA | 5 | - | 8736204 | 8736471 | 268 | intergenic |
| novel_circ_006670 | ENSSSCG00000010685 | 14 | - | 1.29E+08 | 1.29E+08 | 2012 | annot_exons |
| novel_circ_006671 | ENSSSCG00000033272 | 18 | - | 1463489 | 1466141 | 342 | annot_exons |
| novel_circ_006672 | ENSSSCG00000011104 | 10 | - | 57704928 | 57707394 | 217 | annot_exons |
| novel_circ_006673 | ENSSSCG00000005232 | 1 | - | 2.2E+08 | 2.2E+08 | 409 | annot_exons |
| novel_circ_006674 | ENSSSCG00000003398 | 6 | + | 70443884 | 70453863 | 599 | annot_exons |
| novel_circ_006675 | ENSSSCG00000039793 | 6 | - | 1.51E+08 | 1.51E+08 | 269 | annot_exons |
| novel_circ_006676 | ENSSSCG00000004958 | 1 | + | 1.66E+08 | 1.66E+08 | 226 | annot_exons |
| novel_circ_006677 | ENSSSCG00000013522 | 2 | - | 73322629 | 73322835 | 207 | one_exon |
| novel_circ_006678 | ENSSSCG00000002535 | AEMK02000452.1 | - | 917299 | 917530 | 232 | one_exon |
| novel_circ_006679 | ENSSSCG00000014139 | 2 | + | 1.02E+08 | 1.02E+08 | 283 | one_exon |
| novel_circ_006680 | ENSSSCG00000007162 | 17 | + | 32530053 | 32546879 | 447 | annot_exons |
| novel_circ_006681 | ENSSSCG00000007512 | 17 | - | 58467490 | 58468153 | 664 | exon_intron |
| novel_circ_006682 | ENSSSCG00000000158 | 5 | + | 12596908 | 12598060 | 365 | annot_exons |
| novel_circ_006683 | ENSSSCG00000011236 | 13 | - | 19310574 | 19311005 | 324 | annot_exons |
| novel_circ_006684 | ENSSSCG00000011261 | 13 | + | 23754735 | 23763627 | 505 | annot_exons |
| novel_circ_006685 | ENSSSCG00000029311 | 14 | + | 71327584 | 71358688 | 1801 | annot_exons |
| novel_circ_006686 | ENSSSCG00000015940 | 15 | + | 76207329 | 76235600 | 1227 | annot_exons |
| novel_circ_006687 | ENSSSCG00000023728 | 9 | + | 48152108 | 48168468 | 973 | annot_exons |
| novel_circ_006688 | ENSSSCG00000007805 | 3 | + | 18559896 | 18563733 | 577 | annot_exons |
| novel_circ_006689 | ENSSSCG00000042363 | 1 | + | 1.7E+08 | 1.7E+08 | 3182 | exon_intron |
| novel_circ_006690 | ENSSSCG00000015288 | 9 | - | 65797472 | 65797794 | 226 | annot_exons |
| novel_circ_006691 | ENSSSCG00000045919 | 9 | + | 1.22E+08 | 1.22E+08 | 375 | annot_exons |
| novel_circ_006692 | ENSSSCG00000030067 | 1 | - | 16366075 | 16374414 | 764 | annot_exons |
| novel_circ_006693 | ENSSSCG00000034624 | 13 | - | 1.97E+08 | 1.97E+08 | 323 | annot_exons |
| novel_circ_006694 | ENSSSCG00000010190 | 14 | + | 60414028 | 60414636 | 487 | annot_exons |
| novel_circ_006695 | ENSSSCG00000011235 | 13 | - | 19230686 | 19251022 | 442 | annot_exons |
| novel_circ_006696 | ENSSSCG00000007280 | 17 | + | 37837321 | 37838728 | 263 | annot_exons |
| novel_circ_006697 | ENSSSCG00000016836 | 16 | - | 21519866 | 21540786 | 722 | annot_exons |
| novel_circ_006698 | ENSSSCG00000027865 | 7 | - | 1.1E+08 | 1.1E+08 | 1392 | annot_exons |
| novel_circ_006699 | ENSSSCG00000003885 | 6 | - | 1.64E+08 | 1.64E+08 | 300 | annot_exons |
| novel_circ_006700 | ENSSSCG00000029343 | 12 | - | 4792015 | 4796795 | 489 | annot_exons |
| novel_circ_006701 | ENSSSCG00000001080 | 7 | + | 15922704 | 15924948 | 283 | annot_exons |
| novel_circ_006702 | ENSSSCG00000003851 | 6 | - | 1.59E+08 | 1.59E+08 | 1305 | one_exon |
| novel_circ_006703 | ENSSSCG00000010732 | 14 | - | 1.34E+08 | 1.34E+08 | 340 | annot_exons |
| novel_circ_006704 | ENSSSCG00000012262 | X | + | 40131728 | 40148508 | 457 | annot_exons |
| novel_circ_006705 | ENSSSCG00000011022 | 10 | + | 41343313 | 41360718 | 935 | annot_exons |
| novel_circ_006706 | ENSSSCG00000014304 | 2 | + | 1.37E+08 | 1.37E+08 | 1701 | annot_exons |
| novel_circ_006707 | ENSSSCG00000011171 | 10 | + | 25205440 | 25206443 | 284 | annot_exons |
| novel_circ_006708 | ENSSSCG00000004725 | 1 | + | 1.29E+08 | 1.29E+08 | 281 | annot_exons |
| novel_circ_006709 | ENSSSCG00000025194 | 16 | + | 40231897 | 40253338 | 657 | annot_exons |
| novel_circ_006710 | ENSSSCG00000009372 | 11 | - | 15461776 | 15466506 | 433 | annot_exons |
| novel_circ_006711 | ENSSSCG00000015747 | 15 | - | 33569871 | 33570907 | 1037 | antisense |
| novel_circ_006712 | ENSSSCG00000028033 | 14 | + | 929381 | 935525 | 552 | annot_exons |
| novel_circ_006713 | NA | 15 | + | 84282560 | 84282995 | 436 | intergenic |
| novel_circ_006714 | ENSSSCG00000009007 | 8 | - | 75506602 | 75507182 | 373 | annot_exons |
| novel_circ_006715 | ENSSSCG00000024481 | 4 | + | 1.01E+08 | 1.01E+08 | 2376 | antisense |
| novel_circ_006716 | ENSSSCG00000028345 | 14 | - | 60900305 | 60901112 | 241 | annot_exons |
| novel_circ_006717 | NA | 15 | - | 84380600 | 84385036 | 4437 | intergenic |
| novel_circ_006718 | ENSSSCG00000015857 | 15 | - | 59171238 | 59180636 | 600 | annot_exons |
| novel_circ_006719 | ENSSSCG00000008218 | 3 | + | 58183450 | 58183688 | 239 | one_exon |
| novel_circ_006720 | ENSSSCG00000009933 | 14 | - | 41428540 | 41438049 | 2076 | annot_exons |
| novel_circ_006721 | ENSSSCG00000034280 | 8 | - | 96772487 | 96773545 | 319 | annot_exons |
| novel_circ_006722 | ENSSSCG00000016991 | 16 | - | 51460768 | 51461086 | 319 | antisense |
| novel_circ_006723 | ENSSSCG00000000189 | 5 | - | 15128142 | 15131765 | 490 | annot_exons |
| novel_circ_006724 | ENSSSCG00000008591 | 3 | + | 1.15E+08 | 1.15E+08 | 373 | annot_exons |
| novel_circ_006725 | ENSSSCG00000017748 | 12 | - | 43508452 | 43509462 | 352 | annot_exons |
| novel_circ_006726 | ENSSSCG00000002337 | 7 | - | 96377645 | 96378576 | 932 | one_exon |
| novel_circ_006727 | ENSSSCG00000032357 | 13 | + | 39110393 | 39115874 | 448 | annot_exons |
| novel_circ_006728 | ENSSSCG00000016934 | 16 | - | 39725960 | 39731561 | 293 | annot_exons |
| novel_circ_006729 | ENSSSCG00000004929 | 1 | - | 1.63E+08 | 1.63E+08 | 393 | annot_exons |
| novel_circ_006730 | ENSSSCG00000038646 | 10 | - | 13516188 | 13527498 | 344 | annot_exons |
| novel_circ_006731 | NA | 15 | - | 84418147 | 84420078 | 1932 | intergenic |
| novel_circ_006732 | ENSSSCG00000033175 | 1 | - | 17372568 | 17382520 | 215 | annot_exons |
| novel_circ_006733 | ENSSSCG00000012642 | X | + | 1.01E+08 | 1.01E+08 | 560 | intronic |
| novel_circ_006734 | ENSSSCG00000008048 | 3 | + | 39742506 | 39743093 | 344 | annot_exons |
| novel_circ_006735 | ENSSSCG00000036828 | 13 | + | 1.35E+08 | 1.35E+08 | 647 | annot_exons |
| novel_circ_006736 | ENSSSCG00000004935 | 1 | + | 1.64E+08 | 1.64E+08 | 241 | annot_exons |
| novel_circ_006737 | ENSSSCG00000017754 | 12 | + | 44136413 | 44140669 | 4257 | exon_intron |
| novel_circ_006738 | ENSSSCG00000032377 | 7 | + | 64263201 | 64287500 | 793 | annot_exons |
| novel_circ_006739 | ENSSSCG00000005436 | 1 | + | 2.48E+08 | 2.48E+08 | 261 | annot_exons |
| novel_circ_006740 | ENSSSCG00000017094 | 16 | - | 72053549 | 72057438 | 225 | annot_exons |
| novel_circ_006741 | ENSSSCG00000011394 | 13 | + | 32460816 | 32478114 | 4343 | annot_exons |
| novel_circ_006742 | ENSSSCG00000033228 | 12 | - | 27247382 | 27249877 | 439 | annot_exons |
| novel_circ_006743 | ENSSSCG00000008943 | 8 | + | 67899038 | 67899217 | 180 | one_exon |
| novel_circ_006744 | ENSSSCG00000050344 | 2 | - | 1.31E+08 | 1.31E+08 | 40880 | exon_intron |
| novel_circ_006745 | ENSSSCG00000009535 | 11 | - | 74209709 | 74209992 | 284 | one_exon |
| novel_circ_006746 | ENSSSCG00000013362 | 2 | + | 40652441 | 40653859 | 345 | annot_exons |
| novel_circ_006747 | ENSSSCG00000035479 | 10 | + | 11693303 | 11694808 | 350 | annot_exons |
| novel_circ_006748 | ENSSSCG00000017255 | 12 | + | 11098684 | 11110769 | 1188 | annot_exons |
| novel_circ_006749 | ENSSSCG00000026731 | 6 | + | 83677287 | 83679028 | 423 | annot_exons |
| novel_circ_006750 | ENSSSCG00000023324 | 9 | + | 45808593 | 45812247 | 317 | annot_exons |
| novel_circ_006751 | ENSSSCG00000013930 | 2 | + | 58270838 | 58271233 | 281 | annot_exons |
| novel_circ_006752 | ENSSSCG00000029311 | 14 | - | 71372446 | 71372969 | 524 | antisense |
| novel_circ_006753 | ENSSSCG00000007142 | 17 | - | 31807232 | 31820827 | 1141 | annot_exons |
| novel_circ_006754 | ENSSSCG00000034364 | 12 | - | 59541641 | 59546736 | 461 | annot_exons |
| novel_circ_006755 | NA | 15 | + | 84247963 | 84248449 | 487 | intergenic |
| novel_circ_006756 | ENSSSCG00000048263 | 13 | + | 43166523 | 43167805 | 1283 | intronic |
| novel_circ_006757 | ENSSSCG00000021644 | 6 | + | 34513016 | 34516600 | 305 | annot_exons |
| novel_circ_006758 | ENSSSCG00000010351 | 14 | + | 85602428 | 85620168 | 558 | annot_exons |
| novel_circ_006759 | ENSSSCG00000008573 | 3 | - | 1.13E+08 | 1.13E+08 | 192 | annot_exons |
| novel_circ_006760 | ENSSSCG00000037424 | 1 | - | 76723120 | 76727151 | 238 | annot_exons |
| novel_circ_006761 | ENSSSCG00000000840 | 5 | - | 79426603 | 79437245 | 546 | annot_exons |
| novel_circ_006762 | ENSSSCG00000002516 | 7 | - | 1.21E+08 | 1.21E+08 | 323 | annot_exons |
| novel_circ_006763 | ENSSSCG00000023391 | 6 | - | 36387420 | 36413963 | 404 | annot_exons |
| novel_circ_006764 | ENSSSCG00000010719 | 14 | + | 1.32E+08 | 1.32E+08 | 283 | annot_exons |
| novel_circ_006765 | ENSSSCG00000008179 | 3 | + | 54799381 | 54816635 | 357 | annot_exons |
| novel_circ_006766 | ENSSSCG00000008337 | 3 | + | 72871099 | 72888556 | 371 | annot_exons |
| novel_circ_006767 | ENSSSCG00000000406 | 5 | - | 22005744 | 22009750 | 373 | annot_exons |
| novel_circ_006768 | ENSSSCG00000006725 | 4 | + | 1.02E+08 | 1.02E+08 | 11951 | intronic |
| novel_circ_006769 | ENSSSCG00000036909 | 6 | + | 1.67E+08 | 1.67E+08 | 503 | annot_exons |
| novel_circ_006770 | ENSSSCG00000016846 | 16 | + | 22841618 | 22846449 | 324 | annot_exons |
| novel_circ_006771 | ENSSSCG00000039009 | 1 | + | 2.3E+08 | 2.3E+08 | 319 | antisense |
| novel_circ_006772 | ENSSSCG00000010358 | 14 | + | 87660995 | 87677816 | 7059 | exon_intron |
| novel_circ_006773 | ENSSSCG00000022479 | 4 | + | 34707238 | 34710992 | 694 | annot_exons |
| novel_circ_006774 | ENSSSCG00000006391 | 4 | - | 90365515 | 90365882 | 165 | annot_exons |
| novel_circ_006775 | ENSSSCG00000023434 | 13 | + | 1.01E+08 | 1.01E+08 | 44482 | exon_intron |
| novel_circ_006776 | ENSSSCG00000039309 | 2 | - | 1.46E+08 | 1.46E+08 | 319 | annot_exons |
| novel_circ_006777 | ENSSSCG00000003693 | 6 | + | 1.03E+08 | 1.03E+08 | 164 | one_exon |
| novel_circ_006778 | ENSSSCG00000000169 | 5 | - | 13857975 | 13864346 | 302 | annot_exons |
| novel_circ_006779 | ENSSSCG00000010190 | 14 | - | 60412946 | 60414137 | 1192 | antisense |
| novel_circ_006780 | ENSSSCG00000036145 | 6 | - | 1.56E+08 | 1.56E+08 | 4037 | intronic |
| novel_circ_006781 | ENSSSCG00000008512 | 3 | - | 1.07E+08 | 1.07E+08 | 447 | annot_exons |
| novel_circ_006782 | ENSSSCG00000026087 | 10 | + | 31215191 | 31224915 | 315 | annot_exons |
| novel_circ_006783 | ENSSSCG00000025316 | 13 | - | 95702963 | 95703536 | 303 | annot_exons |
| novel_circ_006784 | ENSSSCG00000017670 | 12 | + | 36150268 | 36154292 | 221 | annot_exons |
| novel_circ_006785 | ENSSSCG00000008991 | 8 | + | 73837182 | 73853347 | 549 | annot_exons |
| novel_circ_006786 | ENSSSCG00000026596 | 12 | - | 39027517 | 39029701 | 275 | annot_exons |
| novel_circ_006787 | ENSSSCG00000002844 | 6 | - | 37127249 | 37166517 | 883 | annot_exons |
| novel_circ_006788 | ENSSSCG00000003728 | 6 | - | 1.16E+08 | 1.16E+08 | 1410 | annot_exons |
| novel_circ_006789 | ENSSSCG00000008581 | 3 | - | 1.14E+08 | 1.14E+08 | 2773 | annot_exons |
| novel_circ_006790 | ENSSSCG00000011332 | 13 | - | 29915862 | 29916530 | 667 | annot_exons |
| novel_circ_006791 | ENSSSCG00000007203 | 17 | + | 34722015 | 34724622 | 322 | annot_exons |
| novel_circ_006792 | NA | 15 | - | 84279821 | 84280641 | 821 | intergenic |
| novel_circ_006793 | ENSSSCG00000015462 | 9 | + | 1.13E+08 | 1.13E+08 | 386 | annot_exons |
| novel_circ_006794 | ENSSSCG00000011296 | 13 | - | 26892208 | 26899656 | 321 | annot_exons |
| novel_circ_006795 | ENSSSCG00000018009 | 12 | + | 55430883 | 55432293 | 559 | annot_exons |
| novel_circ_006796 | ENSSSCG00000023710 | 3 | + | 58497758 | 58523990 | 400 | annot_exons |
| novel_circ_006797 | ENSSSCG00000009050 | 8 | + | 85198193 | 85200429 | 236 | annot_exons |
| novel_circ_006798 | ENSSSCG00000038708 | 7 | - | 48865699 | 48866128 | 430 | exon_intron |
| novel_circ_006799 | ENSSSCG00000010465 | 14 | + | 1.03E+08 | 1.03E+08 | 938 | annot_exons |
| novel_circ_006800 | ENSSSCG00000032220 | 13 | + | 18687083 | 18688121 | 353 | annot_exons |
| novel_circ_006801 | ENSSSCG00000009554 | 11 | - | 78629279 | 78632495 | 284 | annot_exons |
| novel_circ_006802 | ENSSSCG00000011714 | 13 | + | 91310020 | 91314586 | 352 | annot_exons |
| novel_circ_006803 | ENSSSCG00000000784 | 5 | + | 71826791 | 71834961 | 368 | annot_exons |
| novel_circ_006804 | ENSSSCG00000015284 | 9 | + | 65195717 | 65202098 | 4309 | annot_exons |
| novel_circ_006805 | ENSSSCG00000006873 | 4 | + | 1.18E+08 | 1.18E+08 | 759 | annot_exons |
| novel_circ_006806 | ENSSSCG00000007521 | 17 | + | 59133156 | 59136271 | 597 | annot_exons |
| novel_circ_006807 | ENSSSCG00000017923 | 12 | - | 52373102 | 52376066 | 2965 | antisense |
| novel_circ_006808 | ENSSSCG00000010825 | 10 | + | 10038504 | 10066131 | 501 | annot_exons |
| novel_circ_006809 | ENSSSCG00000010900 | 10 | - | 20455784 | 20492105 | 477 | annot_exons |
| novel_circ_006810 | ENSSSCG00000030420 | 6 | + | 13138872 | 13141246 | 225 | annot_exons |
| novel_circ_006811 | ENSSSCG00000022126 | 9 | + | 1.39E+08 | 1.39E+08 | 333 | annot_exons |
| novel_circ_006812 | ENSSSCG00000008575 | 3 | + | 1.13E+08 | 1.13E+08 | 390 | annot_exons |
| novel_circ_006813 | ENSSSCG00000016968 | 16 | + | 47814481 | 47817069 | 229 | annot_exons |
| novel_circ_006814 | ENSSSCG00000039157 | 15 | + | 29963741 | 29963908 | 168 | one_exon |
| novel_circ_006815 | ENSSSCG00000000795 | 5 | - | 74586270 | 74595084 | 976 | annot_exons |
| novel_circ_006816 | ENSSSCG00000015494 | 9 | + | 1.16E+08 | 1.16E+08 | 1057 | annot_exons |
| novel_circ_006817 | NA | 15 | + | 84292117 | 84292280 | 164 | intergenic |
| novel_circ_006818 | ENSSSCG00000016945 | 16 | + | 44097581 | 44099411 | 265 | annot_exons |
| novel_circ_006819 | ENSSSCG00000010468 | 14 | - | 1.04E+08 | 1.04E+08 | 674 | annot_exons |
| novel_circ_006820 | ENSSSCG00000002245 | 7 | + | 80336806 | 80341076 | 452 | annot_exons |
| novel_circ_006821 | ENSSSCG00000006199 | 4 | - | 66781891 | 66801230 | 362 | annot_exons |
| novel_circ_006822 | ENSSSCG00000011183 | 13 | - | 1466116 | 1467192 | 265 | annot_exons |
| novel_circ_006823 | ENSSSCG00000010987 | 10 | + | 32862723 | 32866152 | 492 | annot_exons |
| novel_circ_006824 | ENSSSCG00000024481 | 4 | - | 1.01E+08 | 1.01E+08 | 306 | annot_exons |
| novel_circ_006825 | ENSSSCG00000039542 | 2 | - | 77379617 | 77380927 | 327 | annot_exons |
| novel_circ_006826 | ENSSSCG00000004487 | 1 | - | 92459864 | 92460262 | 304 | annot_exons |
| novel_circ_006827 | ENSSSCG00000004589 | 1 | - | 1.13E+08 | 1.13E+08 | 442 | annot_exons |
| novel_circ_006828 | ENSSSCG00000028516 | 5 | + | 16320132 | 16322891 | 241 | annot_exons |
| novel_circ_006829 | ENSSSCG00000003757 | 6 | + | 1.29E+08 | 1.29E+08 | 311 | annot_exons |
| novel_circ_006830 | ENSSSCG00000051585 | 6 | - | 18032138 | 18041648 | 383 | annot_exons |
| novel_circ_006831 | ENSSSCG00000016971 | 16 | + | 47980992 | 47986842 | 460 | annot_exons |
| novel_circ_006832 | ENSSSCG00000024373 | 15 | - | 1.31E+08 | 1.31E+08 | 932 | annot_exons |
| novel_circ_006833 | ENSSSCG00000002267 | 7 | + | 85941709 | 85958911 | 631 | annot_exons |
| novel_circ_006834 | ENSSSCG00000030378 | 3 | + | 11268400 | 11268784 | 273 | annot_exons |
| novel_circ_006835 | ENSSSCG00000014114 | 2 | + | 88462019 | 88470418 | 8400 | intronic |
| novel_circ_006836 | ENSSSCG00000005282 | 1 | + | 2.3E+08 | 2.3E+08 | 536 | annot_exons |
| novel_circ_006837 | ENSSSCG00000034569 | 2 | + | 51369984 | 51370259 | 276 | intronic |
| novel_circ_006838 | ENSSSCG00000036553 | 4 | + | 20327035 | 20381384 | 271 | exon_intron |
| novel_circ_006839 | ENSSSCG00000009390 | 11 | + | 17854849 | 17864720 | 387 | annot_exons |
| novel_circ_006840 | ENSSSCG00000010688 | 14 | - | 1.3E+08 | 1.3E+08 | 1104 | annot_exons |
| novel_circ_006841 | ENSSSCG00000006755 | 4 | + | 1.06E+08 | 1.06E+08 | 298 | annot_exons |
| novel_circ_006842 | ENSSSCG00000003648 | 6 | - | 94792018 | 94796340 | 407 | annot_exons |
| novel_circ_006843 | ENSSSCG00000036145 | 6 | + | 1.56E+08 | 1.56E+08 | 398 | antisense |
| novel_circ_006844 | ENSSSCG00000004318 | 1 | - | 57385549 | 57392343 | 407 | annot_exons |
| novel_circ_006845 | ENSSSCG00000022479 | 4 | + | 34707238 | 34708526 | 500 | annot_exons |
| novel_circ_006846 | ENSSSCG00000004347 | 1 | - | 65894300 | 65911662 | 805 | annot_exons |
| novel_circ_006847 | ENSSSCG00000004242 | 1 | + | 41144282 | 41156428 | 463 | annot_exons |
| novel_circ_006848 | ENSSSCG00000039591 | 17 | - | 46913078 | 46914246 | 388 | annot_exons |
| novel_circ_006849 | ENSSSCG00000035730 | 1 | + | 2.08E+08 | 2.08E+08 | 679 | annot_exons |
| novel_circ_006850 | ENSSSCG00000015266 | 9 | + | 63924125 | 63924477 | 353 | one_exon |
| novel_circ_006851 | ENSSSCG00000038220 | 1 | + | 2.74E+08 | 2.74E+08 | 767 | annot_exons |
| novel_circ_006852 | ENSSSCG00000014927 | 9 | - | 22666473 | 22690654 | 143 | annot_exons |
| novel_circ_006853 | ENSSSCG00000003809 | 6 | + | 1.48E+08 | 1.48E+08 | 419 | one_exon |
| novel_circ_006854 | ENSSSCG00000027509 | 3 | + | 77936961 | 77946126 | 766 | annot_exons |
| novel_circ_006855 | NA | 11 | + | 71243696 | 71252357 | 8662 | intergenic |
| novel_circ_006856 | ENSSSCG00000030049 | 6 | + | 49286824 | 49291518 | 371 | exon_intron |
| novel_circ_006857 | ENSSSCG00000029547 | 15 | + | 95259699 | 95275846 | 1024 | annot_exons |
| novel_circ_006858 | ENSSSCG00000016922 | 16 | + | 36266989 | 36268756 | 415 | annot_exons |
| novel_circ_006859 | ENSSSCG00000006137 | 4 | - | 50473240 | 50486463 | 262 | annot_exons |
| novel_circ_006860 | ENSSSCG00000032377 | 7 | + | 64253214 | 64270922 | 872 | annot_exons |
| novel_circ_006861 | ENSSSCG00000017256 | 12 | + | 11253178 | 11268222 | 1273 | annot_exons |
| novel_circ_006862 | ENSSSCG00000042491 | 8 | - | 69311850 | 69316398 | 4324 | exon_intron |
| novel_circ_006863 | ENSSSCG00000004600 | 1 | - | 1.15E+08 | 1.15E+08 | 432 | annot_exons |
| novel_circ_006864 | ENSSSCG00000004608 | 1 | + | 1.16E+08 | 1.16E+08 | 778 | exon_intron |
| novel_circ_006865 | ENSSSCG00000002296 | 7 | - | 91432352 | 91438716 | 661 | annot_exons |
| novel_circ_006866 | ENSSSCG00000035967 | 6 | - | 1051422 | 1057079 | 787 | annot_exons |
| novel_circ_006867 | ENSSSCG00000020808 | 3 | - | 16847447 | 16848143 | 282 | annot_exons |
| novel_circ_006868 | ENSSSCG00000002340 | 7 | + | 96520887 | 96536557 | 485 | annot_exons |
| novel_circ_006869 | ENSSSCG00000006346 | 4 | - | 88723352 | 88740487 | 529 | annot_exons |
| novel_circ_006870 | ENSSSCG00000011775 | 13 | + | 1.22E+08 | 1.22E+08 | 2030 | annot_exons |
| novel_circ_006871 | ENSSSCG00000030460 | X | - | 1.12E+08 | 1.12E+08 | 385 | annot_exons |
| novel_circ_006872 | ENSSSCG00000028983 | 8 | + | 29513969 | 29527178 | 13210 | exon_intron |
| novel_circ_006873 | ENSSSCG00000016272 | 15 | + | 1.32E+08 | 1.32E+08 | 832 | annot_exons |
| novel_circ_006874 | ENSSSCG00000031304 | 13 | + | 27673147 | 27688847 | 530 | annot_exons |
| novel_circ_006875 | ENSSSCG00000002502 | 7 | - | 1.18E+08 | 1.18E+08 | 1142 | annot_exons |
| novel_circ_006876 | ENSSSCG00000045722 | 7 | + | 97388452 | 97395211 | 264 | annot_exons |
| novel_circ_006877 | ENSSSCG00000014128 | 2 | + | 89814129 | 89817432 | 412 | annot_exons |
| novel_circ_006878 | ENSSSCG00000023771 | 11 | - | 3959346 | 3968427 | 310 | annot_exons |
| novel_circ_006879 | ENSSSCG00000000553 | 5 | + | 46752694 | 46757889 | 599 | annot_exons |
| novel_circ_006880 | ENSSSCG00000031413 | AEMK02000682.1 | - | 1309271 | 1311249 | 258 | annot_exons |
| novel_circ_006881 | NA | 2 | - | 6750991 | 6751177 | 187 | intergenic |
| novel_circ_006882 | ENSSSCG00000011068 | 10 | - | 48814823 | 48820979 | 744 | annot_exons |
| novel_circ_006883 | NA | 15 | - | 84351996 | 84355067 | 3072 | intergenic |
| novel_circ_006884 | ENSSSCG00000015987 | 15 | + | 82976042 | 82977937 | 1896 | antisense |
| novel_circ_006885 | ENSSSCG00000005436 | 1 | + | 2.48E+08 | 2.48E+08 | 616 | annot_exons |
| novel_circ_006886 | ENSSSCG00000029637 | 2 | - | 5251035 | 5251976 | 402 | annot_exons |
| novel_circ_006887 | ENSSSCG00000012613 | X | - | 98245814 | 98263484 | 2361 | annot_exons |
| novel_circ_006888 | ENSSSCG00000033823 | 5 | - | 7557452 | 7557859 | 408 | one_exon |
| novel_circ_006889 | ENSSSCG00000035421 | 11 | + | 3636057 | 3664233 | 504 | annot_exons |
| novel_circ_006890 | ENSSSCG00000025768 | 7 | - | 1.12E+08 | 1.12E+08 | 461 | antisense |
| novel_circ_006891 | ENSSSCG00000004242 | 1 | + | 41144282 | 41149254 | 346 | annot_exons |
| novel_circ_006892 | ENSSSCG00000005441 | 1 | + | 2.5E+08 | 2.5E+08 | 283 | annot_exons |
| novel_circ_006893 | ENSSSCG00000000510 | 5 | + | 35787170 | 35787588 | 419 | one_exon |
| novel_circ_006894 | ENSSSCG00000015792 | 15 | - | 46394953 | 46405221 | 1770 | annot_exons |
| novel_circ_006895 | ENSSSCG00000008470 | 3 | + | 1.01E+08 | 1.01E+08 | 275 | annot_exons |
| novel_circ_006896 | ENSSSCG00000008887 | 8 | - | 51974842 | 51981264 | 316 | annot_exons |
| novel_circ_006897 | ENSSSCG00000005198 | 1 | - | 2.15E+08 | 2.15E+08 | 292 | annot_exons |
| novel_circ_006898 | ENSSSCG00000009801 | 14 | - | 30671954 | 30681039 | 290 | annot_exons |
| novel_circ_006899 | ENSSSCG00000025323 | 6 | + | 13566069 | 13568869 | 330 | annot_exons |
| novel_circ_006900 | ENSSSCG00000010298 | 14 | - | 76097808 | 76107894 | 680 | annot_exons |
| novel_circ_006901 | ENSSSCG00000005016 | 1 | - | 1.8E+08 | 1.8E+08 | 5376 | exon_intron |
| novel_circ_006902 | ENSSSCG00000032556 | 3 | + | 18259248 | 18259422 | 175 | one_exon |
| novel_circ_006903 | ENSSSCG00000035901 | 14 | + | 266888 | 287094 | 677 | annot_exons |
| novel_circ_006904 | ENSSSCG00000002506 | 7 | + | 1.18E+08 | 1.18E+08 | 694 | annot_exons |
| novel_circ_006905 | ENSSSCG00000016509 | 18 | - | 10292639 | 10300806 | 354 | annot_exons |
| novel_circ_006906 | ENSSSCG00000003712 | 6 | - | 1.09E+08 | 1.09E+08 | 429 | annot_exons |
| novel_circ_006907 | ENSSSCG00000039368 | 10 | + | 25760691 | 25779015 | 473 | annot_exons |
| novel_circ_006908 | ENSSSCG00000007264 | 17 | - | 36921253 | 36921733 | 339 | annot_exons |
| novel_circ_006909 | ENSSSCG00000010652 | 14 | + | 1.25E+08 | 1.25E+08 | 477 | annot_exons |
| novel_circ_006910 | ENSSSCG00000040056 | 5 | + | 89715551 | 89715817 | 267 | antisense |
| novel_circ_006911 | ENSSSCG00000008821 | 8 | + | 38279848 | 38287382 | 825 | annot_exons |
| novel_circ_006912 | ENSSSCG00000037530 | 14 | + | 1.32E+08 | 1.32E+08 | 261 | annot_exons |
| novel_circ_006913 | ENSSSCG00000008753 | 8 | - | 16681471 | 16691496 | 672 | annot_exons |
| novel_circ_006914 | ENSSSCG00000028646 | 15 | - | 2499410 | 2499497 | 88 | antisense |
| novel_circ_006915 | ENSSSCG00000004358 | 1 | - | 67576479 | 67584208 | 597 | annot_exons |
| novel_circ_006916 | ENSSSCG00000021384 | 12 | + | 36548266 | 36551241 | 422 | annot_exons |
| novel_circ_006917 | NA | 15 | - | 84274829 | 84275257 | 429 | intergenic |
| novel_circ_006918 | ENSSSCG00000003021 | 6 | - | 49608535 | 49612523 | 1326 | annot_exons |
| novel_circ_006919 | ENSSSCG00000017877 | 12 | - | 50206420 | 50226240 | 828 | annot_exons |
| novel_circ_006920 | ENSSSCG00000004457 | 1 | - | 83108433 | 83112794 | 373 | annot_exons |
| novel_circ_006921 | ENSSSCG00000007369 | 17 | - | 46637291 | 46638070 | 780 | one_exon |
| novel_circ_006922 | ENSSSCG00000030362 | 13 | - | 1.36E+08 | 1.36E+08 | 389 | annot_exons |
| novel_circ_006923 | ENSSSCG00000015627 | 9 | - | 1.36E+08 | 1.36E+08 | 495 | annot_exons |
| novel_circ_006924 | ENSSSCG00000025478 | 6 | + | 1.07E+08 | 1.07E+08 | 1142 | annot_exons |
| novel_circ_006925 | ENSSSCG00000005602 | 1 | + | 2.66E+08 | 2.66E+08 | 412 | annot_exons |
| novel_circ_006926 | ENSSSCG00000029578 | 6 | + | 28736919 | 28743372 | 373 | annot_exons |
| novel_circ_006927 | ENSSSCG00000014119 | 2 | - | 88863298 | 88868668 | 435 | annot_exons |
| novel_circ_006928 | ENSSSCG00000010467 | 14 | + | 1.04E+08 | 1.04E+08 | 311 | annot_exons |
| novel_circ_006929 | ENSSSCG00000023784 | 9 | + | 99373604 | 99376050 | 215 | annot_exons |
| novel_circ_006930 | ENSSSCG00000035533 | 3 | + | 56781048 | 56781323 | 276 | intronic |
| novel_circ_006931 | ENSSSCG00000014010 | 2 | + | 78380148 | 78385328 | 2133 | annot_exons |
| novel_circ_006932 | ENSSSCG00000008513 | 3 | - | 1.07E+08 | 1.07E+08 | 405 | annot_exons |
| novel_circ_006933 | ENSSSCG00000022994 | 1 | + | 53152426 | 53157947 | 308 | annot_exons |
| novel_circ_006934 | ENSSSCG00000013399 | 2 | - | 46469819 | 46472486 | 191 | annot_exons |
| novel_circ_006935 | ENSSSCG00000000625 | 5 | + | 60241617 | 60249461 | 898 | annot_exons |
| novel_circ_006936 | ENSSSCG00000030182 | 6 | + | 49764937 | 49803399 | 336 | annot_exons |
| novel_circ_006937 | ENSSSCG00000013233 | 2 | + | 15132030 | 15136640 | 455 | annot_exons |
| novel_circ_006938 | ENSSSCG00000015443 | 9 | + | 1.08E+08 | 1.08E+08 | 293 | annot_exons |
| novel_circ_006939 | ENSSSCG00000017832 | 12 | - | 48503094 | 48503900 | 687 | annot_exons |
| novel_circ_006940 | ENSSSCG00000005243 | 1 | + | 2.22E+08 | 2.22E+08 | 436 | annot_exons |
| novel_circ_006941 | ENSSSCG00000031903 | 2 | - | 1303712 | 1303910 | 199 | antisense |
| novel_circ_006942 | ENSSSCG00000017735 | 12 | - | 42750233 | 42762928 | 577 | annot_exons |
| novel_circ_006943 | ENSSSCG00000010290 | 14 | + | 75535724 | 75573886 | 37497 | exon_intron |
| novel_circ_006944 | ENSSSCG00000004524 | 1 | + | 1.01E+08 | 1.01E+08 | 1902 | annot_exons |
| novel_circ_006945 | ENSSSCG00000020663 | 18 | + | 5153662 | 5155852 | 287 | annot_exons |
| novel_circ_006946 | ENSSSCG00000029576 | 14 | + | 92200168 | 92201131 | 964 | one_exon |
| novel_circ_006947 | ENSSSCG00000028293 | 9 | - | 1.32E+08 | 1.32E+08 | 366 | annot_exons |
| novel_circ_006948 | ENSSSCG00000013322 | 2 | + | 29248988 | 29285842 | 463 | annot_exons |
| novel_circ_006949 | ENSSSCG00000001779 | 7 | - | 49027735 | 49028028 | 294 | antisense |
| novel_circ_006950 | ENSSSCG00000010862 | 10 | - | 14790087 | 14794270 | 530 | annot_exons |
| novel_circ_006951 | ENSSSCG00000020663 | 18 | + | 5245060 | 5248954 | 376 | annot_exons |
| novel_circ_006952 | ENSSSCG00000021814 | 11 | + | 713800 | 714610 | 273 | annot_exons |
| novel_circ_006953 | ENSSSCG00000039793 | 6 | - | 1.5E+08 | 1.5E+08 | 425 | annot_exons |
| novel_circ_006954 | ENSSSCG00000020879 | 8 | + | 76686440 | 76688711 | 261 | annot_exons |
| novel_circ_006955 | NA | 15 | + | 84233182 | 84233663 | 482 | intergenic |
| novel_circ_006956 | ENSSSCG00000017817 | 12 | - | 47863875 | 47864637 | 382 | annot_exons |
| novel_circ_006957 | ENSSSCG00000038539 | 10 | - | 8602491 | 8612217 | 316 | annot_exons |
| novel_circ_006958 | ENSSSCG00000011312 | 13 | + | 28634101 | 28648156 | 742 | annot_exons |
| novel_circ_006959 | ENSSSCG00000027293 | 16 | + | 49093476 | 49112899 | 4057 | annot_exons |
| novel_circ_006960 | ENSSSCG00000007478 | 17 | - | 53037004 | 53043217 | 368 | annot_exons |
| novel_circ_006961 | ENSSSCG00000017877 | 12 | - | 50273815 | 50282622 | 201 | annot_exons |
| novel_circ_006962 | ENSSSCG00000023434 | 13 | - | 1.01E+08 | 1.01E+08 | 249 | antisense |
| novel_circ_006963 | ENSSSCG00000023585 | 6 | + | 88003870 | 88015088 | 1190 | annot_exons |
| novel_circ_006964 | ENSSSCG00000017712 | 12 | - | 39840857 | 39847346 | 276 | annot_exons |
| novel_circ_006965 | ENSSSCG00000009319 | 11 | + | 5503065 | 5507316 | 260 | annot_exons |
| novel_circ_006966 | ENSSSCG00000021814 | 11 | + | 675689 | 686516 | 1522 | annot_exons |
| novel_circ_006967 | ENSSSCG00000025005 | 6 | - | 1.16E+08 | 1.16E+08 | 356 | annot_exons |
| novel_circ_006968 | ENSSSCG00000044899 | 17 | - | 15046186 | 15052085 | 5900 | exon_intron |
| novel_circ_006969 | ENSSSCG00000010238 | 14 | + | 71491530 | 71495730 | 798 | annot_exons |
| novel_circ_006970 | ENSSSCG00000024904 | 13 | - | 1.37E+08 | 1.37E+08 | 560 | annot_exons |
| novel_circ_006971 | ENSSSCG00000036669 | 2 | + | 9238931 | 9244178 | 5248 | exon_intron |
| novel_circ_006972 | ENSSSCG00000016763 | 18 | + | 52569239 | 52609441 | 661 | annot_exons |
| novel_circ_006973 | ENSSSCG00000011521 | 13 | - | 54881268 | 54887738 | 195 | annot_exons |
| novel_circ_006974 | ENSSSCG00000010994 | 10 | - | 33171737 | 33173745 | 209 | annot_exons |
| novel_circ_006975 | ENSSSCG00000026655 | 8 | - | 1.32E+08 | 1.32E+08 | 1088 | annot_exons |
| novel_circ_006976 | ENSSSCG00000015882 | 15 | - | 66291710 | 66293017 | 208 | annot_exons |
| novel_circ_006977 | ENSSSCG00000016564 | 18 | + | 18897278 | 18905375 | 6749 | exon_intron |
| novel_circ_006978 | ENSSSCG00000002714 | 6 | + | 12704998 | 12713457 | 411 | annot_exons |
| novel_circ_006979 | ENSSSCG00000003401 | 6 | + | 70609103 | 70612317 | 422 | annot_exons |
| novel_circ_006980 | NA | 15 | + | 84289303 | 84289501 | 199 | intergenic |
| novel_circ_006981 | ENSSSCG00000014998 | 9 | + | 35906006 | 35942709 | 582 | annot_exons |
| novel_circ_006982 | ENSSSCG00000008298 | 3 | - | 69216876 | 69218285 | 1410 | antisense |
| novel_circ_006983 | ENSSSCG00000014177 | 2 | - | 1.05E+08 | 1.05E+08 | 887 | annot_exons |
| novel_circ_006984 | ENSSSCG00000003772 | 6 | + | 1.36E+08 | 1.36E+08 | 286 | annot_exons |
| novel_circ_006985 | ENSSSCG00000004146 | 1 | + | 25526210 | 25528664 | 507 | annot_exons |
| novel_circ_006986 | ENSSSCG00000006872 | 4 | - | 1.18E+08 | 1.18E+08 | 320 | annot_exons |
| novel_circ_006987 | ENSSSCG00000016291 | 15 | + | 1.33E+08 | 1.33E+08 | 1107 | annot_exons |
| novel_circ_006988 | ENSSSCG00000011243 | 13 | + | 22226107 | 22239729 | 267 | annot_exons |
| novel_circ_006989 | NA | 13 | - | 38938050 | 38939005 | 956 | intergenic |
| novel_circ_006990 | ENSSSCG00000030535 | 1 | - | 9191141 | 9195425 | 2939 | annot_exons |
| novel_circ_006991 | ENSSSCG00000011332 | 13 | - | 29945767 | 29960782 | 562 | annot_exons |
| novel_circ_006992 | ENSSSCG00000006136 | 4 | + | 50356925 | 50377213 | 711 | annot_exons |
| novel_circ_006993 | ENSSSCG00000001534 | 7 | + | 30898715 | 30902633 | 774 | annot_exons |
| novel_circ_006994 | ENSSSCG00000010299 | 14 | - | 76216343 | 76222620 | 509 | annot_exons |
| novel_circ_006995 | ENSSSCG00000008404 | 3 | + | 85958484 | 85959178 | 338 | annot_exons |
| novel_circ_006996 | ENSSSCG00000011833 | 13 | + | 1.33E+08 | 1.33E+08 | 307 | annot_exons |
| novel_circ_006997 | ENSSSCG00000023133 | 15 | + | 84092401 | 84094790 | 290 | annot_exons |
| novel_circ_006998 | ENSSSCG00000003973 | 6 | + | 1.7E+08 | 1.7E+08 | 440 | antisense |
| novel_circ_006999 | ENSSSCG00000008398 | 3 | + | 85677811 | 85679444 | 234 | annot_exons |
| novel_circ_007000 | ENSSSCG00000015988 | 15 | + | 82968312 | 82968539 | 228 | one_exon |
| novel_circ_007001 | ENSSSCG00000008508 | 3 | - | 1.06E+08 | 1.06E+08 | 214 | antisense |
| novel_circ_007002 | ENSSSCG00000010468 | 14 | - | 1.04E+08 | 1.04E+08 | 258 | annot_exons |
| novel_circ_007003 | ENSSSCG00000009714 | 14 | + | 20168415 | 20187416 | 427 | annot_exons |
| novel_circ_007004 | ENSSSCG00000007280 | 17 | + | 37862504 | 37875412 | 330 | annot_exons |
| novel_circ_007005 | ENSSSCG00000016695 | 18 | + | 44996263 | 45011080 | 567 | annot_exons |
| novel_circ_007006 | ENSSSCG00000036236 | 8 | + | 1.12E+08 | 1.12E+08 | 12293 | exon_intron |
| novel_circ_007007 | ENSSSCG00000033528 | 12 | - | 14133607 | 14135379 | 1773 | antisense |
| novel_circ_007008 | ENSSSCG00000009511 | 11 | - | 67470911 | 67477541 | 453 | annot_exons |
| novel_circ_007009 | ENSSSCG00000023548 | 8 | - | 1.16E+08 | 1.16E+08 | 1254 | annot_exons |
| novel_circ_007010 | ENSSSCG00000040220 | 6 | + | 1.36E+08 | 1.36E+08 | 227 | annot_exons |
| novel_circ_007011 | ENSSSCG00000001479 | 7 | + | 25639784 | 25679321 | 687 | annot_exons |
| novel_circ_007012 | ENSSSCG00000012108 | X | + | 6859159 | 6862999 | 314 | annot_exons |
| novel_circ_007013 | ENSSSCG00000004603 | 1 | + | 1.16E+08 | 1.16E+08 | 403 | annot_exons |
| novel_circ_007014 | ENSSSCG00000028148 | X | - | 28617320 | 28674133 | 264 | annot_exons |
| novel_circ_007015 | ENSSSCG00000014364 | 2 | + | 1.42E+08 | 1.42E+08 | 1313 | annot_exons |
| novel_circ_007016 | ENSSSCG00000009419 | 11 | - | 22249417 | 22255527 | 406 | annot_exons |
| novel_circ_007017 | ENSSSCG00000015129 | 9 | + | 47561176 | 47567772 | 216 | annot_exons |
| novel_circ_007018 | ENSSSCG00000006202 | 4 | - | 67679793 | 67689204 | 516 | annot_exons |
| novel_circ_007019 | ENSSSCG00000010190 | 14 | - | 60415450 | 60415669 | 220 | antisense |
| novel_circ_007020 | ENSSSCG00000004529 | 1 | + | 1.04E+08 | 1.04E+08 | 438 | annot_exons |
| novel_circ_007021 | ENSSSCG00000040178 | 7 | + | 15857181 | 15858761 | 368 | annot_exons |
| novel_circ_007022 | ENSSSCG00000003881 | 6 | + | 1.63E+08 | 1.63E+08 | 370 | annot_exons |
| novel_circ_007023 | ENSSSCG00000003512 | 6 | - | 79045304 | 79063127 | 776 | annot_exons |
| novel_circ_007024 | ENSSSCG00000025323 | 6 | + | 13546447 | 13551862 | 335 | annot_exons |
| novel_circ_007025 | ENSSSCG00000017668 | 12 | + | 35952253 | 35960199 | 509 | annot_exons |
| novel_circ_007026 | ENSSSCG00000015428 | 9 | - | 1.05E+08 | 1.05E+08 | 645 | exon_intron |
| novel_circ_007027 | ENSSSCG00000012479 | X | - | 82070406 | 82145774 | 560 | annot_exons |
| novel_circ_007028 | ENSSSCG00000009276 | 11 | - | 1141224 | 1163643 | 717 | annot_exons |
| novel_circ_007029 | NA | 7 | + | 4574943 | 4608689 | 33747 | intergenic |
| novel_circ_007030 | ENSSSCG00000015747 | 15 | + | 33541562 | 33541987 | 321 | annot_exons |
| novel_circ_007031 | ENSSSCG00000014113 | 2 | - | 88196738 | 88201577 | 225 | annot_exons |
| novel_circ_007032 | ENSSSCG00000000551 | 5 | - | 46402704 | 46435727 | 17422 | exon_intron |
| novel_circ_007033 | ENSSSCG00000000509 | 5 | - | 35627860 | 35633470 | 827 | annot_exons |
| novel_circ_007034 | ENSSSCG00000027459 | 13 | + | 90092395 | 90127343 | 2240 | annot_exons |
| novel_circ_007035 | ENSSSCG00000016082 | 15 | - | 1.02E+08 | 1.02E+08 | 3751 | annot_exons |
| novel_circ_007036 | ENSSSCG00000008428 | 3 | - | 93097254 | 93099206 | 275 | annot_exons |
| novel_circ_007037 | ENSSSCG00000039393 | 1 | - | 1.33E+08 | 1.33E+08 | 547 | annot_exons |
| novel_circ_007038 | ENSSSCG00000008961 | 8 | + | 70193530 | 70266960 | 1508 | annot_exons |
| novel_circ_007039 | ENSSSCG00000008591 | 3 | + | 1.15E+08 | 1.15E+08 | 403 | annot_exons |
| novel_circ_007040 | ENSSSCG00000003627 | 6 | + | 91603895 | 91605991 | 361 | annot_exons |
| novel_circ_007041 | ENSSSCG00000039887 | 14 | + | 1.04E+08 | 1.04E+08 | 287 | annot_exons |
| novel_circ_007042 | ENSSSCG00000028516 | 5 | + | 16315891 | 16322891 | 317 | annot_exons |
| novel_circ_007043 | ENSSSCG00000003648 | 6 | - | 94792018 | 94800529 | 820 | annot_exons |
| novel_circ_007044 | ENSSSCG00000016291 | 15 | + | 1.33E+08 | 1.33E+08 | 468 | annot_exons |
| novel_circ_007045 | ENSSSCG00000024189 | 2 | - | 1.41E+08 | 1.41E+08 | 535 | annot_exons |
| novel_circ_007046 | ENSSSCG00000007955 | 3 | - | 38734411 | 38737226 | 276 | annot_exons |
| novel_circ_007047 | ENSSSCG00000003264 | 6 | - | 55975852 | 55977469 | 1618 | one_exon |
| novel_circ_007048 | ENSSSCG00000026092 | 5 | - | 6768078 | 6768882 | 805 | intronic |
| novel_circ_007049 | ENSSSCG00000039094 | 4 | + | 734531 | 734650 | 120 | one_exon |
| novel_circ_007050 | ENSSSCG00000009473 | 11 | - | 49203468 | 49210487 | 526 | annot_exons |
| novel_circ_007051 | ENSSSCG00000002013 | 7 | - | 75248396 | 75253859 | 198 | annot_exons |
| novel_circ_007052 | ENSSSCG00000041050 | 2 | + | 6755164 | 6755445 | 282 | one_exon |
| novel_circ_007053 | ENSSSCG00000004104 | 1 | + | 16354443 | 16357982 | 527 | annot_exons |
| novel_circ_007054 | NA | 14 | - | 41629766 | 41636993 | 7228 | intergenic |
| novel_circ_007055 | ENSSSCG00000021669 | AEMK02000452.1 | + | 1966581 | 1984156 | 246 | annot_exons |
| novel_circ_007056 | NA | 5 | - | 86915633 | 86932427 | 16795 | intergenic |
| novel_circ_007057 | ENSSSCG00000002690 | 6 | - | 6934248 | 6958719 | 1735 | annot_exons |
| novel_circ_007058 | ENSSSCG00000022659 | 12 | + | 35873785 | 35874776 | 431 | annot_exons |
| novel_circ_007059 | ENSSSCG00000006878 | 4 | + | 1.2E+08 | 1.2E+08 | 367 | annot_exons |
| novel_circ_007060 | ENSSSCG00000004166 | 1 | + | 28608494 | 28610424 | 280 | annot_exons |
| novel_circ_007061 | ENSSSCG00000004753 | 1 | + | 1.3E+08 | 1.3E+08 | 1047 | annot_exons |
| novel_circ_007062 | ENSSSCG00000011022 | 10 | + | 41381255 | 41416627 | 1958 | annot_exons |
| novel_circ_007063 | ENSSSCG00000025430 | 3 | - | 9908545 | 9912753 | 993 | annot_exons |
| novel_circ_007064 | ENSSSCG00000040199 | 1 | + | 74428874 | 74440246 | 333 | annot_exons |
| novel_circ_007065 | ENSSSCG00000010243 | 14 | + | 71852747 | 71853159 | 308 | annot_exons |
| novel_circ_007066 | ENSSSCG00000027722 | 8 | - | 1.39E+08 | 1.39E+08 | 1050 | annot_exons |
| novel_circ_007067 | ENSSSCG00000011424 | 13 | + | 33862062 | 33864970 | 741 | annot_exons |
| novel_circ_007068 | ENSSSCG00000010928 | 10 | - | 24781611 | 24784588 | 366 | annot_exons |
| novel_circ_007069 | ENSSSCG00000035822 | X | - | 46871978 | 46878238 | 459 | annot_exons |
| novel_circ_007070 | ENSSSCG00000000901 | 5 | + | 88111103 | 88113521 | 2419 | one_exon |
| novel_circ_007071 | ENSSSCG00000010300 | 14 | - | 76267807 | 76285566 | 680 | annot_exons |
| novel_circ_007072 | ENSSSCG00000010303 | 14 | + | 76458876 | 76459065 | 190 | antisense |
| novel_circ_007073 | ENSSSCG00000017907 | 12 | + | 51962709 | 51962890 | 182 | one_exon |
| novel_circ_007074 | ENSSSCG00000011332 | 13 | - | 29915862 | 29925370 | 900 | annot_exons |
| novel_circ_007075 | ENSSSCG00000013630 | 2 | + | 69554862 | 69571197 | 1432 | annot_exons |
| novel_circ_007076 | ENSSSCG00000006303 | 4 | - | 82951030 | 82995062 | 5033 | annot_exons |
| novel_circ_007077 | ENSSSCG00000017260 | 12 | + | 11737198 | 11748619 | 863 | annot_exons |
| novel_circ_007078 | ENSSSCG00000003400 | 6 | + | 70303836 | 70306728 | 1581 | annot_exons |
| novel_circ_007079 | ENSSSCG00000036145 | 6 | - | 1.56E+08 | 1.56E+08 | 1594 | intronic |
| novel_circ_007080 | NA | 7 | + | 99378201 | 99379862 | 1662 | intergenic |
| novel_circ_007081 | ENSSSCG00000039244 | 10 | + | 48409498 | 48410391 | 280 | annot_exons |
| novel_circ_007082 | ENSSSCG00000035595 | 9 | + | 1.27E+08 | 1.27E+08 | 497 | annot_exons |
| novel_circ_007083 | ENSSSCG00000014114 | 2 | + | 88483138 | 88498341 | 15204 | intronic |
| novel_circ_007084 | ENSSSCG00000039085 | 13 | + | 2.07E+08 | 2.07E+08 | 685 | annot_exons |
| novel_circ_007085 | ENSSSCG00000011720 | 13 | + | 92547506 | 92580621 | 33116 | exon_intron |
| novel_circ_007086 | ENSSSCG00000015789 | 15 | + | 46313775 | 46315302 | 360 | annot_exons |
| novel_circ_007087 | ENSSSCG00000028475 | 13 | + | 1.38E+08 | 1.38E+08 | 195 | annot_exons |
| novel_circ_007088 | ENSSSCG00000004272 | 1 | - | 49919213 | 49941923 | 357 | annot_exons |
| novel_circ_007089 | ENSSSCG00000033099 | 3 | + | 72412374 | 72488470 | 551 | annot_exons |
| novel_circ_007090 | ENSSSCG00000027139 | 13 | + | 1.34E+08 | 1.34E+08 | 262 | annot_exons |
| novel_circ_007091 | ENSSSCG00000017712 | 12 | - | 39840857 | 39850021 | 465 | annot_exons |
| novel_circ_007092 | ENSSSCG00000009709 | 14 | + | 20026944 | 20033837 | 654 | annot_exons |
| novel_circ_007093 | ENSSSCG00000013392 | 2 | + | 44769840 | 44773133 | 346 | annot_exons |
| novel_circ_007094 | ENSSSCG00000010062 | 14 | - | 49766538 | 49771912 | 748 | annot_exons |
| novel_circ_007095 | ENSSSCG00000016572 | 18 | + | 19602979 | 19606144 | 432 | annot_exons |
| novel_circ_007096 | ENSSSCG00000009040 | 8 | - | 82957079 | 82967294 | 640 | annot_exons |
| novel_circ_007097 | ENSSSCG00000036052 | 15 | + | 84227236 | 84227547 | 312 | antisense |
| novel_circ_007098 | ENSSSCG00000000152 | 5 | + | 11842157 | 11842381 | 225 | one_exon |
| novel_circ_007099 | ENSSSCG00000043851 | 14 | + | 89915882 | 89916041 | 160 | one_exon |
| novel_circ_007100 | ENSSSCG00000011045 | 10 | + | 46002429 | 46047270 | 494 | annot_exons |
| novel_circ_007101 | ENSSSCG00000010144 | 14 | - | 54678768 | 54680774 | 462 | annot_exons |
| novel_circ_007102 | ENSSSCG00000004896 | 1 | - | 1.59E+08 | 1.59E+08 | 323 | annot_exons |
| novel_circ_007103 | ENSSSCG00000028148 | X | + | 27029369 | 27029777 | 409 | antisense |
| novel_circ_007104 | ENSSSCG00000028771 | 1 | + | 1.38E+08 | 1.38E+08 | 2285 | exon_intron |
| novel_circ_007105 | ENSSSCG00000026034 | 7 | - | 13420160 | 13426402 | 448 | annot_exons |
| novel_circ_007106 | ENSSSCG00000034655 | 3 | + | 26369233 | 26371179 | 294 | annot_exons |
| novel_circ_007107 | ENSSSCG00000016397 | 15 | + | 654564 | 655979 | 337 | annot_exons |
| novel_circ_007108 | ENSSSCG00000029593 | 3 | - | 92283614 | 92299786 | 741 | annot_exons |
| novel_circ_007109 | ENSSSCG00000003630 | 6 | + | 92102426 | 92122107 | 293 | annot_exons |
| novel_circ_007110 | ENSSSCG00000003738 | 6 | + | 1.19E+08 | 1.19E+08 | 488 | annot_exons |
| novel_circ_007111 | ENSSSCG00000006635 | 4 | + | 98112898 | 98118419 | 1737 | annot_exons |
| novel_circ_007112 | ENSSSCG00000006752 | 4 | + | 1.06E+08 | 1.06E+08 | 823 | annot_exons |
| novel_circ_007113 | ENSSSCG00000008445 | 3 | + | 94944703 | 94974100 | 603 | annot_exons |
| novel_circ_007114 | ENSSSCG00000009482 | 11 | - | 52153962 | 52154237 | 276 | one_exon |
| novel_circ_007115 | ENSSSCG00000006074 | 4 | + | 38096090 | 38111049 | 439 | exon_intron |
| novel_circ_007116 | ENSSSCG00000007931 | 3 | + | 37566454 | 37567367 | 382 | annot_exons |
| novel_circ_007117 | ENSSSCG00000016395 | 15 | - | 798470 | 812599 | 1252 | annot_exons |
| novel_circ_007118 | ENSSSCG00000032023 | 6 | - | 57390887 | 57392360 | 1474 | one_exon |
| novel_circ_007119 | ENSSSCG00000010190 | 14 | + | 60415086 | 60415673 | 588 | one_exon |
| novel_circ_007120 | ENSSSCG00000017272 | 12 | - | 13565013 | 13571493 | 592 | annot_exons |
| novel_circ_007121 | ENSSSCG00000010485 | 14 | + | 1.06E+08 | 1.06E+08 | 424 | annot_exons |
| novel_circ_007122 | ENSSSCG00000005625 | 1 | - | 2.68E+08 | 2.68E+08 | 459 | annot_exons |
| novel_circ_007123 | ENSSSCG00000025401 | 6 | - | 337524 | 338484 | 879 | annot_exons |
| novel_circ_007124 | ENSSSCG00000006032 | 4 | - | 29058486 | 29068902 | 323 | annot_exons |
| novel_circ_007125 | NA | 18 | - | 2633093 | 2661452 | 28360 | intergenic |
| novel_circ_007126 | ENSSSCG00000012941 | 2 | + | 6032715 | 6034356 | 628 | annot_exons |
| novel_circ_007127 | ENSSSCG00000014917 | 9 | + | 20184956 | 20185193 | 238 | one_exon |
| novel_circ_007128 | ENSSSCG00000015283 | 9 | - | 65077225 | 65079972 | 236 | annot_exons |
| novel_circ_007129 | ENSSSCG00000033909 | 18 | - | 6381891 | 6382097 | 207 | one_exon |
| novel_circ_007130 | ENSSSCG00000000862 | 5 | + | 82439058 | 82444539 | 1681 | annot_exons |
| novel_circ_007131 | ENSSSCG00000012474 | X | + | 78864544 | 78885018 | 1813 | exon_intron |
| novel_circ_007132 | ENSSSCG00000005078 | 1 | + | 1.89E+08 | 1.89E+08 | 184 | one_exon |
| novel_circ_007133 | ENSSSCG00000013864 | 2 | - | 60931358 | 60938053 | 548 | annot_exons |
| novel_circ_007134 | ENSSSCG00000006872 | 4 | - | 1.18E+08 | 1.18E+08 | 341 | annot_exons |
| novel_circ_007135 | ENSSSCG00000023391 | 6 | - | 36440012 | 36457415 | 1008 | annot_exons |
| novel_circ_007136 | ENSSSCG00000004551 | 1 | - | 1.07E+08 | 1.07E+08 | 226 | one_exon |
| novel_circ_007137 | ENSSSCG00000017836 | 12 | + | 48687199 | 48687418 | 220 | one_exon |
| novel_circ_007138 | ENSSSCG00000013299 | 2 | + | 26103380 | 26109922 | 268 | annot_exons |
| novel_circ_007139 | ENSSSCG00000024916 | 13 | - | 94310914 | 94312044 | 345 | annot_exons |
| novel_circ_007140 | ENSSSCG00000000734 | 5 | + | 66868994 | 66886003 | 369 | annot_exons |
| novel_circ_007141 | ENSSSCG00000037766 | 8 | + | 80630974 | 80638316 | 289 | annot_exons |
| novel_circ_007142 | ENSSSCG00000015670 | 15 | + | 7757935 | 7785694 | 588 | annot_exons |
| novel_circ_007143 | ENSSSCG00000014038 | 2 | + | 80418481 | 80423636 | 5156 | exon_intron |
| novel_circ_007144 | ENSSSCG00000016082 | 15 | - | 1.02E+08 | 1.02E+08 | 351 | annot_exons |
| novel_circ_007145 | ENSSSCG00000010928 | 10 | - | 24776756 | 24781769 | 620 | annot_exons |
| novel_circ_007146 | ENSSSCG00000003743 | 6 | + | 1.19E+08 | 1.19E+08 | 417 | annot_exons |
| novel_circ_007147 | ENSSSCG00000007322 | 17 | - | 39934929 | 39945863 | 529 | annot_exons |
| novel_circ_007148 | ENSSSCG00000016845 | 16 | - | 22550953 | 22578996 | 1845 | annot_exons |
| novel_circ_007149 | ENSSSCG00000009040 | 8 | - | 82913302 | 82927163 | 854 | annot_exons |
| novel_circ_007150 | NA | 15 | - | 1.21E+08 | 1.21E+08 | 306 | intergenic |
| novel_circ_007151 | ENSSSCG00000034630 | 15 | + | 1.07E+08 | 1.07E+08 | 420 | annot_exons |
| novel_circ_007152 | ENSSSCG00000020878 | 3 | + | 1.02E+08 | 1.02E+08 | 20337 | exon_intron |
| novel_circ_007153 | ENSSSCG00000007592 | 3 | + | 5090410 | 5091217 | 181 | annot_exons |
| novel_circ_007154 | ENSSSCG00000029507 | 8 | + | 1.37E+08 | 1.37E+08 | 4732 | annot_exons |
| novel_circ_007155 | ENSSSCG00000004700 | 1 | - | 1.28E+08 | 1.28E+08 | 197 | annot_exons |
| novel_circ_007156 | ENSSSCG00000002306 | 7 | + | 93001502 | 93018302 | 783 | annot_exons |
| novel_circ_007157 | ENSSSCG00000000466 | 5 | + | 28846156 | 28847621 | 339 | annot_exons |
| novel_circ_007158 | ENSSSCG00000023738 | 11 | - | 21789455 | 21805764 | 973 | annot_exons |
| novel_circ_007159 | ENSSSCG00000002752 | 6 | - | 15720673 | 15721144 | 335 | annot_exons |
| novel_circ_007160 | ENSSSCG00000008179 | 3 | + | 54799381 | 54801088 | 188 | annot_exons |
| novel_circ_007161 | ENSSSCG00000022479 | 4 | + | 34768971 | 34777538 | 1277 | annot_exons |
| novel_circ_007162 | ENSSSCG00000025942 | 13 | - | 1.21E+08 | 1.21E+08 | 894 | annot_exons |
| novel_circ_007163 | ENSSSCG00000013861 | 2 | + | 61041207 | 61043020 | 264 | annot_exons |
| novel_circ_007164 | ENSSSCG00000016692 | 18 | - | 44873297 | 44873626 | 240 | annot_exons |
| novel_circ_007165 | ENSSSCG00000003812 | 6 | - | 1.49E+08 | 1.49E+08 | 724 | annot_exons |
| novel_circ_007166 | ENSSSCG00000000952 | 5 | - | 1.04E+08 | 1.04E+08 | 2329 | exon_intron |
| novel_circ_007167 | ENSSSCG00000028840 | 9 | + | 1.09E+08 | 1.09E+08 | 783 | annot_exons |
| novel_circ_007168 | ENSSSCG00000008591 | 3 | + | 1.15E+08 | 1.15E+08 | 788 | annot_exons |
| novel_circ_007169 | ENSSSCG00000009885 | 14 | + | 39429510 | 39430520 | 255 | annot_exons |
| novel_circ_007170 | ENSSSCG00000015874 | 15 | - | 64788863 | 64795113 | 576 | annot_exons |
| novel_circ_007171 | ENSSSCG00000015288 | 9 | - | 65796721 | 65809568 | 1926 | annot_exons |
| novel_circ_007172 | ENSSSCG00000026898 | 14 | + | 76018396 | 76019552 | 260 | annot_exons |
| novel_circ_007173 | ENSSSCG00000016430 | 18 | - | 5314027 | 5326209 | 5503 | annot_exons |
| novel_circ_007174 | ENSSSCG00000017139 | 12 | - | 1783066 | 1853441 | 2112 | annot_exons |
| novel_circ_007175 | ENSSSCG00000007027 | 17 | - | 11449989 | 11470388 | 515 | annot_exons |
| novel_circ_007176 | ENSSSCG00000033591 | 6 | - | 31978481 | 31984987 | 6507 | exon_intron |
| novel_circ_007177 | ENSSSCG00000001973 | 7 | - | 68828865 | 68837531 | 392 | annot_exons |
| novel_circ_007178 | ENSSSCG00000026596 | 12 | - | 39010331 | 39029701 | 1788 | annot_exons |
| novel_circ_007179 | ENSSSCG00000011394 | 13 | + | 32559768 | 32563493 | 431 | annot_exons |
| novel_circ_007180 | ENSSSCG00000010299 | 14 | - | 76216343 | 76222147 | 396 | annot_exons |
| novel_circ_007181 | ENSSSCG00000016117 | 15 | + | 1.06E+08 | 1.06E+08 | 1043 | annot_exons |
| novel_circ_007182 | ENSSSCG00000015115 | 9 | + | 46471871 | 46475122 | 304 | annot_exons |
| novel_circ_007183 | NA | 15 | + | 84321054 | 84321499 | 446 | intergenic |
| novel_circ_007184 | ENSSSCG00000009074 | 8 | - | 96900997 | 96903458 | 331 | annot_exons |
| novel_circ_007185 | ENSSSCG00000007280 | 17 | + | 37890389 | 37890962 | 294 | annot_exons |
| novel_circ_007186 | ENSSSCG00000004979 | 1 | - | 1.69E+08 | 1.69E+08 | 530 | annot_exons |
| novel_circ_007187 | ENSSSCG00000015790 | 15 | - | 46195224 | 46197560 | 2337 | antisense |
| novel_circ_007188 | ENSSSCG00000009946 | 14 | + | 42234964 | 42241850 | 594 | annot_exons |
| novel_circ_007189 | ENSSSCG00000023948 | 16 | + | 51961036 | 51961272 | 237 | one_exon |
| novel_circ_007190 | ENSSSCG00000004249 | 1 | + | 43407280 | 43441648 | 950 | annot_exons |
| novel_circ_007191 | ENSSSCG00000009817 | 14 | - | 31436767 | 31444026 | 348 | annot_exons |
| novel_circ_007192 | ENSSSCG00000016954 | 16 | + | 44469297 | 44482325 | 341 | annot_exons |
| novel_circ_007193 | ENSSSCG00000030632 | 17 | + | 51630867 | 51631263 | 397 | antisense |
| novel_circ_007194 | ENSSSCG00000002330 | 7 | + | 94536685 | 94547874 | 8902 | exon_intron |
| novel_circ_007195 | ENSSSCG00000027613 | 1 | - | 39505812 | 39506144 | 333 | antisense |
| novel_circ_007196 | ENSSSCG00000018057 | 12 | - | 23707875 | 23711016 | 507 | annot_exons |
| novel_circ_007197 | ENSSSCG00000020785 | 15 | + | 1.21E+08 | 1.21E+08 | 306 | annot_exons |
| novel_circ_007198 | ENSSSCG00000007153 | 17 | - | 32154934 | 32157958 | 583 | annot_exons |
| novel_circ_007199 | ENSSSCG00000026457 | 3 | + | 15339539 | 15351975 | 12437 | exon_intron |
| novel_circ_007200 | ENSSSCG00000015729 | 15 | - | 29707009 | 29708177 | 310 | annot_exons |
| novel_circ_007201 | ENSSSCG00000008079 | 3 | + | 42886966 | 42902838 | 456 | annot_exons |
| novel_circ_007202 | ENSSSCG00000012836 | 2 | + | 603367 | 606966 | 467 | annot_exons |
| novel_circ_007203 | ENSSSCG00000024754 | 18 | - | 40259679 | 40285405 | 574 | annot_exons |
| novel_circ_007204 | ENSSSCG00000035196 | 4 | - | 1.25E+08 | 1.25E+08 | 716 | annot_exons |
| novel_circ_007205 | ENSSSCG00000029331 | 14 | - | 20810348 | 20812608 | 2261 | intronic |
| novel_circ_007206 | ENSSSCG00000004561 | 1 | + | 1.08E+08 | 1.08E+08 | 956 | one_exon |
| novel_circ_007207 | ENSSSCG00000010360 | 14 | + | 87854602 | 87882941 | 1325 | annot_exons |
| novel_circ_007208 | ENSSSCG00000017063 | 16 | + | 66453201 | 66454209 | 1009 | antisense |
| novel_circ_007209 | ENSSSCG00000016397 | 15 | + | 706151 | 712867 | 735 | annot_exons |
| novel_circ_007210 | ENSSSCG00000031701 | 5 | - | 73380414 | 73387814 | 550 | annot_exons |
| novel_circ_007211 | ENSSSCG00000004934 | 1 | - | 1.63E+08 | 1.63E+08 | 282 | annot_exons |
| novel_circ_007212 | ENSSSCG00000009029 | 8 | - | 80682788 | 80742382 | 556 | annot_exons |
| novel_circ_007213 | ENSSSCG00000034677 | 15 | - | 84410651 | 84411189 | 539 | exon_intron |
| novel_circ_007214 | ENSSSCG00000036948 | 10 | + | 23646069 | 23656907 | 3042 | exon_intron |
| novel_circ_007215 | ENSSSCG00000009283 | 11 | + | 2626410 | 2627094 | 276 | annot_exons |
| novel_circ_007216 | ENSSSCG00000015379 | 9 | + | 90608975 | 90613564 | 474 | annot_exons |
| novel_circ_007217 | ENSSSCG00000020796 | 12 | - | 25803228 | 25803405 | 178 | one_exon |
| novel_circ_007218 | ENSSSCG00000004602 | 1 | - | 1.16E+08 | 1.16E+08 | 4908 | annot_exons |
| novel_circ_007219 | ENSSSCG00000036546 | 2 | + | 48999102 | 49002684 | 309 | annot_exons |
| novel_circ_007220 | ENSSSCG00000009071 | 8 | - | 96648481 | 96687491 | 406 | annot_exons |
| novel_circ_007221 | ENSSSCG00000013554 | 2 | - | 72411885 | 72413244 | 444 | annot_exons |
| novel_circ_007222 | ENSSSCG00000034348 | 11 | + | 11156485 | 11161359 | 2555 | annot_exons |
| novel_circ_007223 | ENSSSCG00000024784 | 10 | - | 23525570 | 23526843 | 499 | annot_exons |
| novel_circ_007224 | ENSSSCG00000005252 | 1 | - | 2.23E+08 | 2.23E+08 | 237 | annot_exons |
| novel_circ_007225 | ENSSSCG00000015402 | 9 | + | 98233585 | 98235872 | 392 | annot_exons |
| novel_circ_007226 | ENSSSCG00000009814 | 14 | + | 31264466 | 31278187 | 362 | annot_exons |
| novel_circ_007227 | ENSSSCG00000031392 | 13 | + | 10232425 | 10289511 | 57087 | exon_intron |
| novel_circ_007228 | ENSSSCG00000038886 | 7 | - | 4717996 | 4724210 | 450 | annot_exons |
| novel_circ_007229 | ENSSSCG00000004739 | 1 | - | 1.3E+08 | 1.3E+08 | 730 | annot_exons |
| novel_circ_007230 | ENSSSCG00000017125 | 12 | - | 573171 | 575827 | 874 | annot_exons |
| novel_circ_007231 | ENSSSCG00000004717 | 1 | + | 1.28E+08 | 1.28E+08 | 615 | annot_exons |
| novel_circ_007232 | ENSSSCG00000024201 | 13 | - | 38538687 | 38545830 | 566 | annot_exons |
| novel_circ_007233 | ENSSSCG00000007516 | 17 | + | 58876594 | 58877473 | 357 | annot_exons |
| novel_circ_007234 | ENSSSCG00000009480 | 11 | + | 51540591 | 51554792 | 3485 | annot_exons |
| novel_circ_007235 | ENSSSCG00000009281 | 11 | + | 2349625 | 2366615 | 505 | annot_exons |
| novel_circ_007236 | ENSSSCG00000028516 | 5 | + | 16309169 | 16322891 | 618 | annot_exons |
| novel_circ_007237 | ENSSSCG00000047585 | 13 | + | 46313896 | 46314698 | 803 | antisense |
| novel_circ_007238 | ENSSSCG00000013409 | 2 | + | 49450553 | 49480738 | 679 | exon_intron |
| novel_circ_007239 | ENSSSCG00000012884 | 2 | - | 4439896 | 4442066 | 283 | annot_exons |
| novel_circ_007240 | ENSSSCG00000038178 | AEMK02000697.1 | - | 102899 | 105608 | 645 | annot_exons |
| novel_circ_007241 | ENSSSCG00000004958 | 1 | + | 1.66E+08 | 1.66E+08 | 445 | one_exon |
| novel_circ_007242 | ENSSSCG00000003218 | 6 | + | 55308568 | 55308753 | 186 | one_exon |
| novel_circ_007243 | ENSSSCG00000007203 | 17 | + | 34722015 | 34733655 | 628 | annot_exons |
| novel_circ_007244 | ENSSSCG00000003493 | 6 | - | 77853446 | 77867331 | 540 | annot_exons |
| novel_circ_007245 | ENSSSCG00000015695 | 15 | - | 16804269 | 16814482 | 468 | annot_exons |
| novel_circ_007246 | ENSSSCG00000008079 | 3 | + | 42881922 | 42887048 | 330 | annot_exons |
| novel_circ_007247 | ENSSSCG00000025478 | 6 | + | 1.07E+08 | 1.07E+08 | 2164 | annot_exons |
| novel_circ_007248 | NA | 15 | + | 84231803 | 84232370 | 568 | intergenic |
| novel_circ_007249 | ENSSSCG00000039025 | 1 | + | 2.08E+08 | 2.08E+08 | 1050 | annot_exons |
| novel_circ_007250 | ENSSSCG00000015743 | 15 | - | 31346993 | 31387416 | 751 | annot_exons |
| novel_circ_007251 | ENSSSCG00000009075 | 8 | + | 96951728 | 96953154 | 355 | annot_exons |
| novel_circ_007252 | ENSSSCG00000028949 | 15 | + | 88591136 | 88593310 | 299 | annot_exons |
| novel_circ_007253 | ENSSSCG00000011026 | 10 | - | 42468077 | 42490463 | 571 | annot_exons |
| novel_circ_007254 | ENSSSCG00000026722 | 9 | + | 80731159 | 80741082 | 301 | annot_exons |
| novel_circ_007255 | ENSSSCG00000016164 | 15 | + | 1.16E+08 | 1.16E+08 | 248 | antisense |
| novel_circ_007256 | ENSSSCG00000024814 | X | - | 47764058 | 47765831 | 756 | annot_exons |
| novel_circ_007257 | ENSSSCG00000010189 | 14 | + | 60395043 | 60400195 | 354 | annot_exons |
| novel_circ_007258 | ENSSSCG00000027410 | 17 | + | 37109691 | 37114650 | 386 | annot_exons |
| novel_circ_007259 | ENSSSCG00000016033 | 15 | + | 93239180 | 93252479 | 5519 | annot_exons |
| novel_circ_007260 | NA | 7 | + | 68160992 | 68169856 | 8865 | intergenic |
| novel_circ_007261 | ENSSSCG00000014126 | 2 | + | 89359613 | 89370588 | 539 | annot_exons |
| novel_circ_007262 | ENSSSCG00000012250 | X | - | 36388349 | 36400978 | 772 | annot_exons |
| novel_circ_007263 | ENSSSCG00000000902 | 5 | + | 88253163 | 88273584 | 1160 | annot_exons |
| novel_circ_007264 | NA | AEMK02000489.1 | - | 51048 | 51316 | 269 | intergenic |
| novel_circ_007265 | ENSSSCG00000004979 | 1 | - | 1.69E+08 | 1.69E+08 | 260 | annot_exons |
| novel_circ_007266 | ENSSSCG00000007737 | 3 | - | 16486968 | 16487913 | 946 | one_exon |
| novel_circ_007267 | ENSSSCG00000039332 | 9 | - | 1.2E+08 | 1.2E+08 | 479 | annot_exons |
| novel_circ_007268 | ENSSSCG00000007839 | 3 | - | 23910594 | 23928030 | 1417 | annot_exons |
| novel_circ_007269 | ENSSSCG00000026152 | 6 | - | 52568184 | 52572803 | 261 | annot_exons |
| novel_circ_007270 | ENSSSCG00000014794 | 9 | + | 6361824 | 6374618 | 368 | annot_exons |
| novel_circ_007271 | ENSSSCG00000004934 | 1 | - | 1.63E+08 | 1.64E+08 | 456 | annot_exons |
| novel_circ_007272 | ENSSSCG00000004465 | 1 | - | 85961743 | 85964180 | 399 | annot_exons |
| novel_circ_007273 | ENSSSCG00000015314 | 9 | + | 72230096 | 72242063 | 580 | annot_exons |
| novel_circ_007274 | ENSSSCG00000027403 | 8 | - | 55621938 | 55623888 | 482 | annot_exons |
| novel_circ_007275 | ENSSSCG00000009864 | 14 | + | 36128175 | 36128520 | 346 | intronic |
| novel_circ_007276 | ENSSSCG00000015509 | 9 | - | 1.18E+08 | 1.18E+08 | 309 | annot_exons |
| novel_circ_007277 | ENSSSCG00000000492 | 5 | + | 33541409 | 33548592 | 965 | annot_exons |
| novel_circ_007278 | ENSSSCG00000009729 | 14 | - | 22572451 | 22577570 | 424 | annot_exons |
| novel_circ_007279 | ENSSSCG00000039085 | 13 | + | 2.07E+08 | 2.07E+08 | 415 | annot_exons |
| novel_circ_007280 | ENSSSCG00000006179 | 4 | + | 62162191 | 62187826 | 627 | annot_exons |
| novel_circ_007281 | ENSSSCG00000004666 | 1 | - | 1.26E+08 | 1.26E+08 | 422 | annot_exons |
| novel_circ_007282 | ENSSSCG00000015940 | 15 | + | 76177104 | 76177857 | 194 | annot_exons |
| novel_circ_007283 | NA | 18 | - | 14212354 | 14226917 | 14564 | intergenic |
| novel_circ_007284 | NA | 6 | + | 1.49E+08 | 1.49E+08 | 610 | intergenic |
| novel_circ_007285 | ENSSSCG00000011183 | 13 | - | 1491239 | 1493431 | 284 | annot_exons |
| novel_circ_007286 | ENSSSCG00000048198 | 1 | - | 2.72E+08 | 2.72E+08 | 954 | antisense |
| novel_circ_007287 | ENSSSCG00000000571 | 5 | + | 51630416 | 51643707 | 573 | annot_exons |
| novel_circ_007288 | ENSSSCG00000005466 | 1 | - | 2.53E+08 | 2.53E+08 | 3666 | annot_exons |
| novel_circ_007289 | ENSSSCG00000004032 | 1 | - | 5739509 | 5739889 | 381 | antisense |
| novel_circ_007290 | ENSSSCG00000039885 | 6 | - | 88424922 | 88432891 | 1010 | annot_exons |
| novel_circ_007291 | ENSSSCG00000048856 | 2 | - | 6755664 | 6756045 | 382 | one_exon |
| novel_circ_007292 | ENSSSCG00000009042 | 8 | + | 83256967 | 83258814 | 215 | annot_exons |
| novel_circ_007293 | ENSSSCG00000016853 | 16 | - | 24151895 | 24153311 | 463 | annot_exons |
| novel_circ_007294 | ENSSSCG00000033424 | 13 | - | 1.22E+08 | 1.22E+08 | 386 | annot_exons |
| novel_circ_007295 | ENSSSCG00000006981 | 17 | + | 4802543 | 4820201 | 478 | annot_exons |
| novel_circ_007296 | ENSSSCG00000030682 | 6 | + | 82950268 | 82952280 | 269 | annot_exons |
| novel_circ_007297 | ENSSSCG00000015931 | 15 | + | 75864199 | 75868240 | 294 | annot_exons |
| novel_circ_007298 | ENSSSCG00000024694 | 17 | - | 61482067 | 61497286 | 1733 | annot_exons |
| novel_circ_007299 | ENSSSCG00000021072 | 1 | - | 95527140 | 95536688 | 1057 | annot_exons |
| novel_circ_007300 | ENSSSCG00000033356 | 3 | - | 7549994 | 7553955 | 420 | annot_exons |
| novel_circ_007301 | ENSSSCG00000011672 | 13 | + | 82304411 | 82306675 | 179 | annot_exons |
| novel_circ_007302 | ENSSSCG00000006824 | 4 | - | 1.11E+08 | 1.11E+08 | 284 | annot_exons |
| novel_circ_007303 | ENSSSCG00000048856 | 2 | - | 6754914 | 6755236 | 323 | one_exon |
| novel_circ_007304 | ENSSSCG00000016695 | 18 | + | 44996263 | 45020744 | 1143 | annot_exons |
| novel_circ_007305 | ENSSSCG00000004293 | 1 | - | 54474921 | 54494313 | 517 | annot_exons |
| novel_circ_007306 | ENSSSCG00000009369 | 11 | + | 14608535 | 14623293 | 510 | annot_exons |
| novel_circ_007307 | ENSSSCG00000010654 | 14 | + | 1.25E+08 | 1.25E+08 | 338 | annot_exons |
| novel_circ_007308 | ENSSSCG00000004899 | 1 | - | 1.59E+08 | 1.59E+08 | 370 | annot_exons |
| novel_circ_007309 | ENSSSCG00000001903 | 7 | + | 58850363 | 58868520 | 867 | annot_exons |
| novel_circ_007310 | ENSSSCG00000002330 | 7 | + | 94527244 | 94533253 | 318 | annot_exons |
| novel_circ_007311 | ENSSSCG00000000531 | 5 | - | 41937707 | 41942985 | 664 | annot_exons |
| novel_circ_007312 | ENSSSCG00000042703 | 1 | - | 1.08E+08 | 1.08E+08 | 297 | annot_exons |
| novel_circ_007313 | ENSSSCG00000015857 | 15 | - | 59179085 | 59181694 | 373 | annot_exons |
| novel_circ_007314 | ENSSSCG00000003812 | 6 | - | 1.49E+08 | 1.49E+08 | 1625 | annot_exons |
| novel_circ_007315 | ENSSSCG00000028406 | 1 | - | 2.02E+08 | 2.02E+08 | 319 | annot_exons |
| novel_circ_007316 | ENSSSCG00000004820 | 1 | - | 1.39E+08 | 1.39E+08 | 591 | one_exon |
| novel_circ_007317 | ENSSSCG00000023324 | 9 | + | 45789727 | 45795661 | 837 | annot_exons |
| novel_circ_007318 | ENSSSCG00000005575 | 1 | - | 2.64E+08 | 2.64E+08 | 826 | annot_exons |
| novel_circ_007319 | ENSSSCG00000012123 | X | + | 10325909 | 10328276 | 496 | annot_exons |
| novel_circ_007320 | NA | 14 | - | 41636873 | 41643575 | 6703 | intergenic |
| novel_circ_007321 | ENSSSCG00000008799 | 8 | + | 32773630 | 32783783 | 673 | annot_exons |
| novel_circ_007322 | ENSSSCG00000033298 | 2 | - | 65096942 | 65097643 | 702 | antisense |
| novel_circ_007323 | ENSSSCG00000004207 | 1 | - | 34166378 | 34213181 | 527 | annot_exons |
| novel_circ_007324 | NA | 15 | - | 84253685 | 84253876 | 192 | intergenic |
| novel_circ_007325 | ENSSSCG00000009680 | 14 | + | 12530404 | 12540502 | 273 | annot_exons |
| novel_circ_007326 | ENSSSCG00000025653 | 6 | + | 1.06E+08 | 1.06E+08 | 194 | annot_exons |
| novel_circ_007327 | ENSSSCG00000006508 | 4 | + | 94369484 | 94391746 | 844 | annot_exons |
| novel_circ_007328 | ENSSSCG00000037639 | 13 | - | 1.41E+08 | 1.41E+08 | 426 | annot_exons |
| novel_circ_007329 | ENSSSCG00000026596 | 12 | - | 39010331 | 39016435 | 1130 | annot_exons |
| novel_circ_007330 | ENSSSCG00000016430 | 18 | - | 5300933 | 5326209 | 6098 | annot_exons |
| novel_circ_007331 | ENSSSCG00000016397 | 15 | + | 633526 | 634297 | 417 | annot_exons |
| novel_circ_007332 | ENSSSCG00000027857 | 2 | + | 1.23E+08 | 1.23E+08 | 600 | annot_exons |
| novel_circ_007333 | ENSSSCG00000004646 | 1 | + | 1.22E+08 | 1.22E+08 | 330 | annot_exons |
| novel_circ_007334 | ENSSSCG00000033692 | 14 | + | 55783529 | 55789427 | 290 | annot_exons |
| novel_circ_007335 | ENSSSCG00000024453 | 13 | - | 68595006 | 68599780 | 488 | annot_exons |
| novel_circ_007336 | ENSSSCG00000017904 | 12 | - | 51954622 | 51955769 | 480 | annot_exons |
| novel_circ_007337 | NA | 15 | - | 84418404 | 84421411 | 3008 | intergenic |
| novel_circ_007338 | ENSSSCG00000014913 | 9 | - | 19885865 | 19892227 | 219 | annot_exons |
| novel_circ_007339 | ENSSSCG00000040317 | 1 | + | 7690018 | 7690221 | 204 | one_exon |
| novel_circ_007340 | ENSSSCG00000011194 | 13 | - | 2940058 | 2964480 | 1128 | annot_exons |
| novel_circ_007341 | ENSSSCG00000014884 | 9 | - | 12383553 | 12395636 | 697 | annot_exons |
| novel_circ_007342 | ENSSSCG00000011775 | 13 | + | 1.22E+08 | 1.22E+08 | 218 | one_exon |
| novel_circ_007343 | ENSSSCG00000011013 | 10 | + | 39991048 | 40011985 | 964 | annot_exons |
| novel_circ_007344 | ENSSSCG00000021634 | 6 | + | 84975876 | 84979732 | 520 | annot_exons |
| novel_circ_007345 | ENSSSCG00000011752 | 13 | + | 1.11E+08 | 1.11E+08 | 8678 | exon_intron |
| novel_circ_007346 | ENSSSCG00000014114 | 2 | + | 88356925 | 88372391 | 2086 | exon_intron |
| novel_circ_007347 | ENSSSCG00000024864 | 5 | - | 3717600 | 3720056 | 279 | annot_exons |
| novel_circ_007348 | ENSSSCG00000009015 | 8 | + | 77301300 | 77301994 | 695 | exon_intron |
| novel_circ_007349 | ENSSSCG00000015940 | 15 | + | 76226667 | 76228805 | 285 | annot_exons |
| novel_circ_007350 | ENSSSCG00000006143 | 4 | - | 51408732 | 51423953 | 5886 | annot_exons |
| novel_circ_007351 | ENSSSCG00000022240 | X | + | 20811537 | 20813374 | 225 | annot_exons |
| novel_circ_007352 | ENSSSCG00000008192 | 3 | + | 56352120 | 56378046 | 1206 | exon_intron |
| novel_circ_007353 | ENSSSCG00000003154 | 6 | - | 54244674 | 54245209 | 335 | annot_exons |
| novel_circ_007354 | ENSSSCG00000002461 | 7 | - | 1.15E+08 | 1.15E+08 | 1118 | annot_exons |
| novel_circ_007355 | ENSSSCG00000003772 | 6 | + | 1.36E+08 | 1.36E+08 | 577 | annot_exons |
| novel_circ_007356 | ENSSSCG00000024999 | 2 | + | 1.09E+08 | 1.09E+08 | 343 | annot_exons |
| novel_circ_007357 | ENSSSCG00000003693 | 6 | + | 1.03E+08 | 1.04E+08 | 821 | annot_exons |
| novel_circ_007358 | ENSSSCG00000016726 | 18 | - | 50106628 | 50125659 | 381 | annot_exons |
| novel_circ_007359 | ENSSSCG00000023996 | 1 | - | 1.27E+08 | 1.27E+08 | 270 | annot_exons |
| novel_circ_007360 | ENSSSCG00000017672 | 12 | + | 36418549 | 36419324 | 488 | annot_exons |
| novel_circ_007361 | ENSSSCG00000008644 | 3 | + | 1.27E+08 | 1.27E+08 | 1914 | annot_exons |
| novel_circ_007362 | ENSSSCG00000035757 | 2 | + | 90121042 | 90121892 | 851 | antisense |
| novel_circ_007363 | ENSSSCG00000023738 | 11 | - | 21791604 | 21805764 | 864 | annot_exons |
| novel_circ_007364 | ENSSSCG00000033291 | 12 | - | 23343835 | 23344201 | 367 | antisense |
| novel_circ_007365 | ENSSSCG00000007355 | 17 | + | 43773574 | 43777223 | 433 | annot_exons |
| novel_circ_007366 | ENSSSCG00000040373 | 5 | + | 69830432 | 69838356 | 214 | annot_exons |
| novel_circ_007367 | ENSSSCG00000008339 | 3 | + | 72986138 | 72990291 | 237 | annot_exons |
| novel_circ_007368 | ENSSSCG00000027342 | 3 | - | 10814283 | 10829380 | 667 | annot_exons |
| novel_circ_007369 | ENSSSCG00000036075 | 5 | + | 82264638 | 82285715 | 1237 | annot_exons |
| novel_circ_007370 | ENSSSCG00000013284 | 2 | - | 18900210 | 18904237 | 732 | annot_exons |
| novel_circ_007371 | ENSSSCG00000021292 | 6 | - | 1.6E+08 | 1.6E+08 | 265 | annot_exons |
| novel_circ_007372 | ENSSSCG00000000146 | 3 | + | 7073431 | 7088481 | 2065 | annot_exons |
| novel_circ_007373 | ENSSSCG00000035824 | 13 | - | 34694854 | 34707449 | 1206 | annot_exons |
| novel_circ_007374 | ENSSSCG00000033356 | 3 | - | 7553137 | 7561037 | 522 | annot_exons |
| novel_circ_007375 | ENSSSCG00000036145 | 6 | - | 1.56E+08 | 1.56E+08 | 469 | annot_exons |
| novel_circ_007376 | ENSSSCG00000015784 | 15 | - | 45877613 | 45879911 | 306 | annot_exons |
| novel_circ_007377 | ENSSSCG00000026724 | 5 | + | 57977723 | 57987136 | 509 | annot_exons |
| novel_circ_007378 | ENSSSCG00000000571 | 5 | + | 51630416 | 51630869 | 219 | annot_exons |
| novel_circ_007379 | ENSSSCG00000005191 | 1 | + | 2.09E+08 | 2.09E+08 | 353 | annot_exons |
| novel_circ_007380 | ENSSSCG00000007478 | 17 | - | 52996471 | 53014574 | 651 | annot_exons |
| novel_circ_007381 | ENSSSCG00000016898 | 16 | - | 34084658 | 34090473 | 5816 | exon_intron |
| novel_circ_007382 | ENSSSCG00000026153 | 9 | + | 36544676 | 36545443 | 339 | annot_exons |
| novel_circ_007383 | ENSSSCG00000016397 | 15 | + | 740414 | 742947 | 210 | annot_exons |
| novel_circ_007384 | ENSSSCG00000016243 | 15 | + | 1.28E+08 | 1.28E+08 | 931 | annot_exons |
| novel_circ_007385 | ENSSSCG00000004718 | 1 | + | 1.29E+08 | 1.29E+08 | 605 | annot_exons |
| novel_circ_007386 | ENSSSCG00000021203 | 13 | - | 79514825 | 79518909 | 351 | annot_exons |
| novel_circ_007387 | ENSSSCG00000004595 | 1 | + | 1.13E+08 | 1.13E+08 | 427 | annot_exons |
| novel_circ_007388 | ENSSSCG00000011765 | 13 | + | 1.18E+08 | 1.18E+08 | 637 | annot_exons |
| novel_circ_007389 | ENSSSCG00000010357 | 14 | - | 87502986 | 87503536 | 292 | annot_exons |
| novel_circ_007390 | ENSSSCG00000012362 | X | - | 50273395 | 50289228 | 15834 | exon_intron |
| novel_circ_007391 | ENSSSCG00000015882 | 15 | - | 66291710 | 66299872 | 452 | annot_exons |
| novel_circ_007392 | ENSSSCG00000009401 | 11 | - | 19250142 | 19253493 | 538 | annot_exons |
| novel_circ_007393 | ENSSSCG00000050727 | 1 | + | 2.43E+08 | 2.43E+08 | 475 | annot_exons |
| novel_circ_007394 | ENSSSCG00000015555 | 9 | + | 1.24E+08 | 1.24E+08 | 435 | annot_exons |
| novel_circ_007395 | ENSSSCG00000035533 | 3 | + | 56778383 | 56779603 | 255 | annot_exons |
| novel_circ_007396 | ENSSSCG00000030467 | 13 | - | 1.09E+08 | 1.09E+08 | 1576 | annot_exons |
| novel_circ_007397 | ENSSSCG00000035934 | 1 | - | 1.82E+08 | 1.82E+08 | 415 | annot_exons |
| novel_circ_007398 | ENSSSCG00000046809 | 6 | + | 47358549 | 47375028 | 16308 | exon_intron |
| novel_circ_007399 | ENSSSCG00000038561 | 15 | - | 84412284 | 84412512 | 229 | one_exon |
| novel_circ_007400 | ENSSSCG00000015944 | 15 | - | 77251049 | 77283482 | 191 | annot_exons |
| novel_circ_007401 | ENSSSCG00000050590 | 14 | - | 41672920 | 41697708 | 24576 | exon_intron |
| novel_circ_007402 | ENSSSCG00000027381 | 3 | + | 59210066 | 59210610 | 398 | annot_exons |
| novel_circ_007403 | ENSSSCG00000000152 | 5 | + | 11842157 | 11865412 | 426 | annot_exons |
| novel_circ_007404 | ENSSSCG00000006487 | 4 | + | 93738637 | 93747422 | 552 | annot_exons |
| novel_circ_007405 | ENSSSCG00000021890 | 6 | - | 63879633 | 63893464 | 513 | annot_exons |
| novel_circ_007406 | ENSSSCG00000024864 | 5 | - | 3739281 | 3754014 | 337 | annot_exons |
| novel_circ_007407 | ENSSSCG00000015565 | 9 | + | 1.26E+08 | 1.26E+08 | 315 | annot_exons |
| novel_circ_007408 | ENSSSCG00000003731 | 6 | - | 1.16E+08 | 1.16E+08 | 1307 | exon_intron |
| novel_circ_007409 | ENSSSCG00000012251 | X | + | 36881418 | 36882414 | 466 | annot_exons |
| novel_circ_007410 | ENSSSCG00000030420 | 6 | + | 13105853 | 13123467 | 763 | annot_exons |
| novel_circ_007411 | ENSSSCG00000007881 | 3 | - | 29282185 | 29287417 | 875 | annot_exons |
| novel_circ_007412 | NA | 15 | - | 84378055 | 84381041 | 2987 | intergenic |
| novel_circ_007413 | ENSSSCG00000017790 | 12 | + | 45694158 | 45710855 | 640 | annot_exons |
| novel_circ_007414 | ENSSSCG00000007100 | 17 | + | 27538642 | 27558311 | 19670 | exon_intron |
| novel_circ_007415 | ENSSSCG00000016578 | 18 | - | 19760225 | 19760866 | 534 | annot_exons |
| novel_circ_007416 | ENSSSCG00000010243 | 14 | + | 71844195 | 71856260 | 883 | annot_exons |
| novel_circ_007417 | ENSSSCG00000003580 | 6 | - | 85115009 | 85123684 | 373 | annot_exons |
| novel_circ_007418 | ENSSSCG00000010734 | 14 | - | 1.34E+08 | 1.34E+08 | 388 | annot_exons |
| novel_circ_007419 | ENSSSCG00000002245 | 7 | + | 80345062 | 80346071 | 184 | annot_exons |
| novel_circ_007420 | ENSSSCG00000012440 | X | + | 62193796 | 62198727 | 352 | annot_exons |
| novel_circ_007421 | ENSSSCG00000017904 | 12 | + | 51954652 | 51955727 | 1076 | antisense |
| novel_circ_007422 | ENSSSCG00000011752 | 13 | + | 1.11E+08 | 1.11E+08 | 351 | annot_exons |
| novel_circ_007423 | NA | 12 | - | 48495840 | 48496173 | 334 | intergenic |
| novel_circ_007424 | ENSSSCG00000002869 | 6 | + | 43867657 | 43879544 | 417 | annot_exons |
| novel_circ_007425 | ENSSSCG00000009283 | 11 | + | 2609301 | 2610503 | 1203 | intronic |
| novel_circ_007426 | ENSSSCG00000001865 | 7 | - | 56727497 | 56759939 | 8934 | exon_intron |
| novel_circ_007427 | ENSSSCG00000004082 | 1 | + | 14090110 | 14093357 | 663 | annot_exons |
| novel_circ_007428 | ENSSSCG00000043136 | 7 | + | 4798004 | 4808408 | 10405 | antisense |
| novel_circ_007429 | ENSSSCG00000014792 | 9 | - | 6075865 | 6088259 | 861 | annot_exons |
| novel_circ_007430 | ENSSSCG00000003398 | 6 | + | 70429221 | 70430862 | 313 | annot_exons |
| novel_circ_007431 | ENSSSCG00000033528 | 12 | + | 14184080 | 14185034 | 488 | annot_exons |
| novel_circ_007432 | ENSSSCG00000014182 | 2 | - | 1.08E+08 | 1.08E+08 | 548 | annot_exons |
| novel_circ_007433 | ENSSSCG00000009887 | 14 | + | 39657028 | 39661539 | 522 | annot_exons |
| novel_circ_007434 | ENSSSCG00000012328 | X | - | 46345680 | 46354249 | 475 | annot_exons |
| novel_circ_007435 | ENSSSCG00000012148 | X | + | 13188717 | 13201433 | 430 | annot_exons |
| novel_circ_007436 | ENSSSCG00000006157 | 4 | - | 94269034 | 94269368 | 202 | annot_exons |
| novel_circ_007437 | ENSSSCG00000030467 | 13 | - | 1.09E+08 | 1.09E+08 | 2423 | annot_exons |
| novel_circ_007438 | ENSSSCG00000004457 | 1 | - | 83098177 | 83112794 | 734 | annot_exons |
| novel_circ_007439 | ENSSSCG00000010799 | 3 | + | 22810516 | 22822228 | 259 | annot_exons |
| novel_circ_007440 | ENSSSCG00000018045 | 12 | + | 59827745 | 59839936 | 556 | annot_exons |
| novel_circ_007441 | ENSSSCG00000010144 | 14 | - | 54742492 | 54742753 | 262 | one_exon |
| novel_circ_007442 | ENSSSCG00000027331 | 15 | - | 1.37E+08 | 1.37E+08 | 199 | one_exon |
| novel_circ_007443 | ENSSSCG00000008513 | 3 | - | 1.07E+08 | 1.07E+08 | 579 | annot_exons |
| novel_circ_007444 | ENSSSCG00000010144 | 14 | - | 54674554 | 54675695 | 225 | annot_exons |
| novel_circ_007445 | ENSSSCG00000028327 | 14 | - | 64342539 | 64349498 | 1285 | annot_exons |
| novel_circ_007446 | ENSSSCG00000011382 | 13 | - | 31891050 | 31894444 | 466 | annot_exons |
| novel_circ_007447 | ENSSSCG00000033692 | 14 | + | 55770332 | 55783722 | 443 | annot_exons |
| novel_circ_007448 | ENSSSCG00000026232 | 8 | - | 12643329 | 12645144 | 326 | annot_exons |
| novel_circ_007449 | ENSSSCG00000008753 | 8 | - | 16728875 | 16732088 | 233 | annot_exons |
| novel_circ_007450 | ENSSSCG00000006857 | 4 | + | 1.16E+08 | 1.16E+08 | 261 | annot_exons |
| novel_circ_007451 | ENSSSCG00000009364 | 11 | + | 14132780 | 14151667 | 18888 | exon_intron |
| novel_circ_007452 | ENSSSCG00000048439 | 18 | - | 54558710 | 54563773 | 537 | annot_exons |
| novel_circ_007453 | ENSSSCG00000010947 | 10 | + | 27638465 | 27651062 | 535 | annot_exons |
| novel_circ_007454 | ENSSSCG00000005366 | 1 | + | 2.39E+08 | 2.39E+08 | 428 | annot_exons |
| novel_circ_007455 | ENSSSCG00000015027 | 9 | + | 39641668 | 39641826 | 159 | intronic |
| novel_circ_007456 | ENSSSCG00000010698 | 14 | - | 1.31E+08 | 1.32E+08 | 81347 | intronic |
| novel_circ_007457 | ENSSSCG00000030108 | 17 | - | 50934085 | 50935350 | 295 | annot_exons |
| novel_circ_007458 | ENSSSCG00000011047 | 10 | + | 46538292 | 46569174 | 657 | annot_exons |
| novel_circ_007459 | ENSSSCG00000034348 | 11 | + | 11248377 | 11249395 | 241 | annot_exons |
| novel_circ_007460 | NA | 15 | - | 84264018 | 84264910 | 893 | intergenic |
| novel_circ_007461 | ENSSSCG00000028805 | 6 | - | 99495382 | 99505669 | 454 | annot_exons |
| novel_circ_007462 | ENSSSCG00000013366 | 2 | - | 40817322 | 40819211 | 466 | annot_exons |
| novel_circ_007463 | ENSSSCG00000011163 | 10 | + | 69028579 | 69030169 | 318 | annot_exons |
| novel_circ_007464 | NA | 15 | - | 1E+08 | 1E+08 | 1585 | intergenic |
| novel_circ_007465 | ENSSSCG00000011572 | 13 | - | 66493801 | 66495580 | 405 | annot_exons |
| novel_circ_007466 | ENSSSCG00000005366 | 1 | + | 2.39E+08 | 2.39E+08 | 606 | annot_exons |
| novel_circ_007467 | ENSSSCG00000001921 | 7 | - | 60112737 | 60124950 | 464 | annot_exons |
| novel_circ_007468 | ENSSSCG00000016033 | 15 | + | 93222405 | 93223292 | 291 | annot_exons |
| novel_circ_007469 | ENSSSCG00000002266 | 7 | - | 86064996 | 86068321 | 551 | annot_exons |
| novel_circ_007470 | ENSSSCG00000029464 | 5 | - | 67679713 | 67688990 | 783 | annot_exons |
| novel_circ_007471 | ENSSSCG00000004196 | 1 | + | 31980772 | 31982095 | 392 | annot_exons |
| novel_circ_007472 | ENSSSCG00000004897 | 1 | - | 1.59E+08 | 1.59E+08 | 292 | annot_exons |
| novel_circ_007473 | ENSSSCG00000020657 | 6 | + | 51302717 | 51303698 | 417 | annot_exons |
| novel_circ_007474 | ENSSSCG00000011022 | 10 | + | 41408040 | 41408711 | 126 | annot_exons |
| novel_circ_007475 | ENSSSCG00000000587 | 5 | - | 53623267 | 53659626 | 602 | annot_exons |
| novel_circ_007476 | ENSSSCG00000015247 | 9 | - | 56946957 | 56973685 | 8900 | exon_intron |
| novel_circ_007477 | ENSSSCG00000005444 | 1 | - | 2.5E+08 | 2.5E+08 | 1356 | exon_intron |
| novel_circ_007478 | ENSSSCG00000011733 | 13 | - | 99987011 | 1E+08 | 741 | annot_exons |
| novel_circ_007479 | ENSSSCG00000016036 | 15 | + | 93992834 | 94001331 | 562 | annot_exons |
| novel_circ_007480 | ENSSSCG00000007280 | 17 | + | 37856179 | 37862678 | 271 | annot_exons |
| novel_circ_007481 | ENSSSCG00000015820 | 15 | + | 48195070 | 48206163 | 405 | annot_exons |
| novel_circ_007482 | ENSSSCG00000030467 | 13 | - | 1.09E+08 | 1.09E+08 | 322 | annot_exons |
| novel_circ_007483 | ENSSSCG00000015940 | 15 | + | 76266532 | 76292128 | 484 | annot_exons |
| novel_circ_007484 | ENSSSCG00000026161 | 3 | + | 1.25E+08 | 1.25E+08 | 636 | annot_exons |
| novel_circ_007485 | ENSSSCG00000036828 | 13 | + | 1.35E+08 | 1.35E+08 | 286 | annot_exons |
| novel_circ_007486 | ENSSSCG00000011025 | 10 | + | 42079398 | 42082530 | 3133 | intronic |
| novel_circ_007487 | ENSSSCG00000001499 | 7 | + | 29108214 | 29108528 | 220 | annot_exons |
| novel_circ_007488 | ENSSSCG00000006508 | 4 | + | 94476170 | 94476592 | 312 | annot_exons |
| novel_circ_007489 | ENSSSCG00000017748 | 12 | - | 43520344 | 43526438 | 370 | annot_exons |
| novel_circ_007490 | ENSSSCG00000013409 | 2 | + | 49499793 | 49507542 | 548 | annot_exons |
| novel_circ_007491 | ENSSSCG00000005467 | 1 | + | 2.53E+08 | 2.53E+08 | 263 | annot_exons |
| novel_circ_007492 | ENSSSCG00000027806 | 17 | - | 40144101 | 40145366 | 302 | annot_exons |
| novel_circ_007493 | ENSSSCG00000004919 | 1 | - | 1.62E+08 | 1.62E+08 | 519 | annot_exons |
| novel_circ_007494 | ENSSSCG00000033177 | 1 | - | 2.54E+08 | 2.54E+08 | 204 | annot_exons |
| novel_circ_007495 | ENSSSCG00000021814 | 11 | + | 714454 | 716256 | 283 | annot_exons |
| novel_circ_007496 | ENSSSCG00000006867 | 4 | + | 1.18E+08 | 1.18E+08 | 299 | annot_exons |
| novel_circ_007497 | ENSSSCG00000024399 | 8 | + | 4959300 | 4959624 | 325 | one_exon |
| novel_circ_007498 | ENSSSCG00000021620 | 2 | - | 7917525 | 7918215 | 351 | annot_exons |
| novel_circ_007499 | ENSSSCG00000012642 | X | + | 1.02E+08 | 1.02E+08 | 220 | annot_exons |
| novel_circ_007500 | ENSSSCG00000025606 | 9 | + | 36625370 | 36627646 | 361 | annot_exons |
| novel_circ_007501 | ENSSSCG00000006766 | 4 | + | 1.07E+08 | 1.07E+08 | 1894 | annot_exons |
| novel_circ_007502 | ENSSSCG00000035827 | 1 | - | 1.27E+08 | 1.27E+08 | 289 | annot_exons |
| novel_circ_007503 | ENSSSCG00000026087 | 10 | + | 31153774 | 31157554 | 397 | annot_exons |
| novel_circ_007504 | ENSSSCG00000031273 | 13 | + | 1.58E+08 | 1.58E+08 | 905 | annot_exons |
| novel_circ_007505 | ENSSSCG00000025942 | 13 | - | 1.21E+08 | 1.21E+08 | 598 | annot_exons |
| novel_circ_007506 | ENSSSCG00000003776 | 6 | - | 1.38E+08 | 1.38E+08 | 352 | annot_exons |
| novel_circ_007507 | ENSSSCG00000017694 | 12 | - | 38711800 | 38716290 | 437 | annot_exons |
| novel_circ_007508 | ENSSSCG00000008192 | 3 | + | 56405641 | 56406910 | 270 | annot_exons |
| novel_circ_007509 | ENSSSCG00000003761 | 6 | - | 1.32E+08 | 1.32E+08 | 19894 | exon_intron |
| novel_circ_007510 | ENSSSCG00000004220 | 1 | - | 36985761 | 36994867 | 607 | annot_exons |
| novel_circ_007511 | ENSSSCG00000024373 | 15 | - | 1.31E+08 | 1.31E+08 | 243 | annot_exons |
| novel_circ_007512 | ENSSSCG00000017190 | 12 | + | 5349486 | 5349744 | 259 | intronic |
| novel_circ_007513 | ENSSSCG00000005282 | 1 | + | 2.3E+08 | 2.3E+08 | 615 | exon_intron |
| novel_circ_007514 | ENSSSCG00000013566 | 2 | + | 71923446 | 71929053 | 584 | annot_exons |
| novel_circ_007515 | ENSSSCG00000026161 | 3 | + | 1.25E+08 | 1.25E+08 | 544 | exon_intron |
| novel_circ_007516 | ENSSSCG00000008581 | 3 | - | 1.14E+08 | 1.14E+08 | 263 | annot_exons |
| novel_circ_007517 | ENSSSCG00000006100 | 4 | + | 42205209 | 42206174 | 489 | annot_exons |
| novel_circ_007518 | ENSSSCG00000038973 | 5 | + | 1.02E+08 | 1.02E+08 | 279 | annot_exons |
| novel_circ_007519 | ENSSSCG00000013598 | 2 | - | 70951849 | 70953147 | 1299 | antisense |
| novel_circ_007520 | ENSSSCG00000012934 | 2 | - | 5825410 | 5826569 | 377 | annot_exons |
| novel_circ_007521 | ENSSSCG00000017136 | 12 | - | 439695 | 443923 | 451 | annot_exons |
| novel_circ_007522 | ENSSSCG00000025408 | 13 | - | 25565162 | 25610799 | 785 | annot_exons |
| novel_circ_007523 | ENSSSCG00000006927 | 4 | - | 1.28E+08 | 1.28E+08 | 301 | one_exon |
| novel_circ_007524 | ENSSSCG00000016542 | 18 | - | 14963829 | 14989256 | 498 | exon_intron |
| novel_circ_007525 | ENSSSCG00000038526 | 3 | - | 4068812 | 4073745 | 1760 | annot_exons |
| novel_circ_007526 | ENSSSCG00000008942 | 8 | + | 67674329 | 67688705 | 395 | annot_exons |
| novel_circ_007527 | ENSSSCG00000003627 | 6 | + | 91596484 | 91604006 | 264 | annot_exons |
| novel_circ_007528 | ENSSSCG00000026004 | 5 | + | 29732023 | 29753021 | 314 | annot_exons |
| novel_circ_007529 | ENSSSCG00000011397 | 13 | + | 32693818 | 32694930 | 365 | annot_exons |
| novel_circ_007530 | ENSSSCG00000004110 | 1 | - | 16558808 | 16566086 | 1689 | annot_exons |
| novel_circ_007531 | ENSSSCG00000021285 | 3 | - | 4264224 | 4279580 | 523 | annot_exons |
| novel_circ_007532 | ENSSSCG00000017015 | 16 | - | 55385796 | 55397890 | 524 | annot_exons |
| novel_circ_007533 | ENSSSCG00000034569 | 2 | - | 51518254 | 51519211 | 958 | antisense |
| novel_circ_007534 | ENSSSCG00000015211 | 9 | + | 52562682 | 52575753 | 361 | annot_exons |
| novel_circ_007535 | ENSSSCG00000010360 | 14 | + | 87854602 | 87862622 | 492 | annot_exons |
| novel_circ_007536 | ENSSSCG00000007789 | 3 | - | 17688479 | 17691346 | 824 | annot_exons |
| novel_circ_007537 | ENSSSCG00000002740 | 6 | - | 14736586 | 14740749 | 715 | annot_exons |
| novel_circ_007538 | ENSSSCG00000007735 | 3 | - | 16314182 | 16328040 | 731 | annot_exons |
| novel_circ_007539 | ENSSSCG00000009898 | 14 | - | 40179862 | 40180345 | 318 | annot_exons |
| novel_circ_007540 | ENSSSCG00000013298 | 2 | - | 26015759 | 26049587 | 1050 | annot_exons |
| novel_circ_007541 | ENSSSCG00000011103 | 10 | - | 57227399 | 57252743 | 958 | annot_exons |
| novel_circ_007542 | ENSSSCG00000025870 | 9 | - | 73042697 | 73059640 | 933 | annot_exons |
| novel_circ_007543 | ENSSSCG00000010734 | 14 | - | 1.34E+08 | 1.34E+08 | 764 | annot_exons |
| novel_circ_007544 | ENSSSCG00000011843 | 13 | - | 1.33E+08 | 1.33E+08 | 492 | annot_exons |
| novel_circ_007545 | ENSSSCG00000003859 | 6 | + | 1.6E+08 | 1.6E+08 | 889 | annot_exons |
| novel_circ_007546 | ENSSSCG00000014794 | 9 | + | 6407824 | 6414461 | 667 | annot_exons |
| novel_circ_007547 | ENSSSCG00000029198 | 9 | + | 36456127 | 36456435 | 227 | annot_exons |
| novel_circ_007548 | ENSSSCG00000016541 | 18 | + | 14891785 | 14904788 | 520 | annot_exons |
| novel_circ_007549 | ENSSSCG00000000160 | 5 | + | 12656287 | 12658795 | 333 | annot_exons |
| novel_circ_007550 | ENSSSCG00000009142 | 8 | - | 1.13E+08 | 1.13E+08 | 350 | annot_exons |
| novel_circ_007551 | ENSSSCG00000008575 | 3 | + | 1.13E+08 | 1.13E+08 | 554 | annot_exons |
| novel_circ_007552 | ENSSSCG00000027331 | 15 | - | 1.37E+08 | 1.37E+08 | 202 | one_exon |
| novel_circ_007553 | ENSSSCG00000006390 | 4 | + | 90276877 | 90277070 | 194 | antisense |
| novel_circ_007554 | ENSSSCG00000011752 | 13 | + | 1.11E+08 | 1.11E+08 | 215 | annot_exons |
| novel_circ_007555 | ENSSSCG00000011373 | 13 | - | 31675253 | 31676127 | 333 | annot_exons |
| novel_circ_007556 | ENSSSCG00000000866 | 5 | - | 82583909 | 82585391 | 324 | annot_exons |
| novel_circ_007557 | ENSSSCG00000030460 | X | - | 1.12E+08 | 1.12E+08 | 797 | annot_exons |
| novel_circ_007558 | ENSSSCG00000003971 | 6 | + | 1.7E+08 | 1.7E+08 | 1068 | exon_intron |
| novel_circ_007559 | ENSSSCG00000011720 | 13 | + | 92675456 | 92677963 | 466 | annot_exons |
| novel_circ_007560 | ENSSSCG00000001892 | 7 | + | 58689009 | 58689789 | 247 | annot_exons |
| novel_circ_007561 | ENSSSCG00000030478 | 5 | - | 89644928 | 89656169 | 313 | annot_exons |
| novel_circ_007562 | ENSSSCG00000016717 | 18 | - | 47642052 | 47657002 | 275 | annot_exons |
| novel_circ_007563 | ENSSSCG00000004537 | 1 | - | 1.06E+08 | 1.06E+08 | 464 | annot_exons |
| novel_circ_007564 | ENSSSCG00000029637 | 2 | - | 5275864 | 5292935 | 915 | annot_exons |
| novel_circ_007565 | ENSSSCG00000012164 | X | + | 17625328 | 17639067 | 291 | annot_exons |
| novel_circ_007566 | ENSSSCG00000036610 | 13 | + | 79018336 | 79029345 | 415 | annot_exons |
| novel_circ_007567 | ENSSSCG00000010797 | 3 | + | 22660735 | 22666364 | 346 | annot_exons |
| novel_circ_007568 | ENSSSCG00000050244 | 12 | + | 59273100 | 59308104 | 474 | exon_intron |
| novel_circ_007569 | ENSSSCG00000008498 | 3 | + | 1.03E+08 | 1.03E+08 | 2535 | annot_exons |
| novel_circ_007570 | ENSSSCG00000026960 | 1 | + | 1.15E+08 | 1.15E+08 | 499 | annot_exons |
| novel_circ_007571 | ENSSSCG00000010928 | 10 | - | 24795546 | 24799565 | 372 | annot_exons |
| novel_circ_007572 | ENSSSCG00000006018 | 4 | + | 22989684 | 22990287 | 604 | one_exon |
| novel_circ_007573 | ENSSSCG00000015462 | 9 | + | 1.13E+08 | 1.13E+08 | 527 | annot_exons |
| novel_circ_007574 | ENSSSCG00000037561 | 11 | + | 78343010 | 78352808 | 402 | annot_exons |
| novel_circ_007575 | ENSSSCG00000030113 | 11 | - | 3806304 | 3806962 | 659 | one_exon |
| novel_circ_007576 | ENSSSCG00000034730 | 1 | - | 96447977 | 96465249 | 659 | annot_exons |
| novel_circ_007577 | ENSSSCG00000006267 | 4 | + | 78123402 | 78125262 | 1861 | exon_intron |
| novel_circ_007578 | ENSSSCG00000014020 | 2 | - | 78894699 | 78896781 | 499 | annot_exons |
| novel_circ_007579 | ENSSSCG00000005170 | 1 | - | 2.03E+08 | 2.03E+08 | 515 | annot_exons |
| novel_circ_007580 | ENSSSCG00000024517 | 7 | - | 67124423 | 67125796 | 261 | annot_exons |
| novel_circ_007581 | ENSSSCG00000028182 | 5 | + | 87262973 | 87276568 | 765 | annot_exons |
| novel_circ_007582 | ENSSSCG00000038607 | 13 | - | 16940725 | 17038336 | 18937 | exon_intron |
| novel_circ_007583 | ENSSSCG00000037670 | X | + | 90177247 | 90250748 | 42967 | exon_intron |
| novel_circ_007584 | ENSSSCG00000031121 | 9 | + | 1.25E+08 | 1.25E+08 | 367 | annot_exons |
| novel_circ_007585 | ENSSSCG00000013399 | 2 | - | 46455458 | 46472486 | 338 | annot_exons |
| novel_circ_007586 | ENSSSCG00000028979 | 9 | + | 10120704 | 10139416 | 1848 | annot_exons |
| novel_circ_007587 | ENSSSCG00000015589 | 9 | - | 1.3E+08 | 1.3E+08 | 132 | annot_exons |
| novel_circ_007588 | ENSSSCG00000038872 | 6 | + | 85664602 | 85681933 | 658 | annot_exons |
| novel_circ_007589 | ENSSSCG00000024570 | 2 | - | 73829182 | 73858570 | 1800 | annot_exons |
| novel_circ_007590 | ENSSSCG00000030095 | 9 | + | 41738255 | 41745104 | 6850 | exon_intron |
| novel_circ_007591 | ENSSSCG00000004399 | 1 | - | 76432534 | 76436834 | 338 | annot_exons |
| novel_circ_007592 | ENSSSCG00000000625 | 5 | + | 60269555 | 60276758 | 1246 | annot_exons |
| novel_circ_007593 | ENSSSCG00000011367 | 13 | + | 31573092 | 31575948 | 408 | annot_exons |
| novel_circ_007594 | ENSSSCG00000017617 | 12 | + | 33288728 | 33290715 | 443 | annot_exons |
| novel_circ_007595 | ENSSSCG00000045919 | 9 | + | 1.22E+08 | 1.22E+08 | 4251 | exon_intron |
| novel_circ_007596 | ENSSSCG00000027857 | 2 | + | 1.23E+08 | 1.23E+08 | 377 | annot_exons |
| novel_circ_007597 | ENSSSCG00000010987 | 10 | + | 32868282 | 32870103 | 384 | annot_exons |
| novel_circ_007598 | ENSSSCG00000034191 | 2 | + | 42933470 | 42961389 | 12586 | exon_intron |
| novel_circ_007599 | ENSSSCG00000016629 | 18 | - | 29084364 | 29142960 | 927 | annot_exons |
| novel_circ_007600 | ENSSSCG00000003872 | 6 | + | 1.61E+08 | 1.61E+08 | 845 | annot_exons |
| novel_circ_007601 | ENSSSCG00000002628 | 7 | - | 46700944 | 46701207 | 264 | one_exon |
| novel_circ_007602 | ENSSSCG00000010358 | 14 | - | 87720563 | 87720712 | 150 | antisense |
| novel_circ_007603 | ENSSSCG00000002330 | 7 | + | 94561632 | 94568013 | 308 | annot_exons |
| novel_circ_007604 | ENSSSCG00000008412 | 3 | + | 87262802 | 87262951 | 150 | one_exon |
| novel_circ_007605 | NA | 15 | - | 84250685 | 84252543 | 1859 | intergenic |
| novel_circ_007606 | ENSSSCG00000039393 | 1 | - | 1.33E+08 | 1.33E+08 | 372 | annot_exons |
| novel_circ_007607 | ENSSSCG00000004064 | 1 | - | 9893445 | 9923857 | 334 | annot_exons |
| novel_circ_007608 | ENSSSCG00000000477 | 5 | + | 30649554 | 30659750 | 1366 | annot_exons |
| novel_circ_007609 | ENSSSCG00000003488 | 6 | - | 77608299 | 77611297 | 645 | annot_exons |
| novel_circ_007610 | ENSSSCG00000023409 | 3 | + | 72257086 | 72262713 | 625 | annot_exons |
| novel_circ_007611 | ENSSSCG00000011632 | 13 | - | 73862998 | 73863238 | 241 | one_exon |
| novel_circ_007612 | ENSSSCG00000013022 | 2 | + | 7416124 | 7418610 | 774 | annot_exons |
| novel_circ_007613 | ENSSSCG00000022080 | 13 | - | 31923711 | 31929502 | 279 | annot_exons |
| novel_circ_007614 | ENSSSCG00000025796 | 12 | + | 40037309 | 40038184 | 298 | annot_exons |
| novel_circ_007615 | ENSSSCG00000003844 | 6 | - | 1.58E+08 | 1.58E+08 | 203 | annot_exons |
| novel_circ_007616 | ENSSSCG00000011791 | 13 | + | 1.23E+08 | 1.23E+08 | 560 | one_exon |
| novel_circ_007617 | ENSSSCG00000037424 | 1 | - | 76620009 | 76651641 | 442 | annot_exons |
| novel_circ_007618 | ENSSSCG00000007895 | 3 | + | 31357057 | 31359294 | 460 | annot_exons |
| novel_circ_007619 | ENSSSCG00000003863 | 6 | - | 1.6E+08 | 1.6E+08 | 878 | annot_exons |
| novel_circ_007620 | ENSSSCG00000017403 | 12 | + | 20440837 | 20443556 | 395 | annot_exons |
| novel_circ_007621 | ENSSSCG00000004071 | 1 | + | 12373577 | 12381510 | 249 | annot_exons |
| novel_circ_007622 | ENSSSCG00000041461 | AEMK02000489.1 | - | 41191 | 41347 | 157 | one_exon |
| novel_circ_007623 | ENSSSCG00000011131 | 10 | + | 64498378 | 64531678 | 1971 | annot_exons |
| novel_circ_007624 | ENSSSCG00000016397 | 15 | + | 654489 | 656083 | 516 | annot_exons |
| novel_circ_007625 | ENSSSCG00000015584 | 9 | - | 1.3E+08 | 1.3E+08 | 2095 | annot_exons |
| novel_circ_007626 | ENSSSCG00000024663 | 13 | + | 1.46E+08 | 1.46E+08 | 291 | annot_exons |
| novel_circ_007627 | ENSSSCG00000007874 | 3 | - | 28428101 | 28428304 | 204 | one_exon |
| novel_circ_007628 | ENSSSCG00000014219 | 2 | - | 1.2E+08 | 1.2E+08 | 234 | annot_exons |
| novel_circ_007629 | ENSSSCG00000011382 | 13 | - | 31891050 | 31891384 | 208 | annot_exons |
| novel_circ_007630 | ENSSSCG00000003976 | 6 | - | 1.7E+08 | 1.7E+08 | 282 | annot_exons |
| novel_circ_007631 | ENSSSCG00000000233 | 5 | + | 17338840 | 17345187 | 581 | annot_exons |
| novel_circ_007632 | ENSSSCG00000010799 | 3 | + | 22745957 | 22746522 | 266 | annot_exons |
| novel_circ_007633 | ENSSSCG00000029082 | 9 | + | 1.05E+08 | 1.05E+08 | 658 | annot_exons |
| novel_circ_007634 | ENSSSCG00000024794 | 6 | - | 75410959 | 75415003 | 384 | annot_exons |
| novel_circ_007635 | ENSSSCG00000002505 | 7 | + | 1.18E+08 | 1.18E+08 | 323 | annot_exons |
| novel_circ_007636 | ENSSSCG00000005060 | 1 | + | 1.85E+08 | 1.85E+08 | 248 | annot_exons |
| novel_circ_007637 | NA | 9 | + | 1.22E+08 | 1.22E+08 | 1406 | intergenic |
| novel_circ_007638 | ENSSSCG00000016841 | 16 | + | 21943061 | 21954312 | 970 | annot_exons |
| novel_circ_007639 | ENSSSCG00000014044 | 2 | - | 80711394 | 80713361 | 381 | annot_exons |
| novel_circ_007640 | NA | 15 | - | 84354981 | 84367753 | 12773 | intergenic |
| novel_circ_007641 | ENSSSCG00000004753 | 1 | + | 1.3E+08 | 1.3E+08 | 1311 | annot_exons |
| novel_circ_007642 | ENSSSCG00000008821 | 8 | + | 38273589 | 38296131 | 1052 | annot_exons |
| novel_circ_007643 | ENSSSCG00000009319 | 11 | + | 5503065 | 5524214 | 422 | annot_exons |
| novel_circ_007644 | ENSSSCG00000013054 | 2 | + | 8389129 | 8390219 | 232 | annot_exons |
| novel_circ_007645 | ENSSSCG00000043503 | 13 | + | 1.21E+08 | 1.21E+08 | 418 | annot_exons |
| novel_circ_007646 | NA | 15 | + | 84252409 | 84253057 | 649 | intergenic |
| novel_circ_007647 | ENSSSCG00000000842 | 5 | + | 79293489 | 79293928 | 440 | antisense |
| novel_circ_007648 | ENSSSCG00000011686 | 13 | - | 84459152 | 84486328 | 409 | annot_exons |
| novel_circ_007649 | ENSSSCG00000009097 | 8 | + | 1.02E+08 | 1.02E+08 | 532 | annot_exons |
| novel_circ_007650 | ENSSSCG00000014893 | 9 | - | 12903427 | 12918332 | 475 | annot_exons |
| novel_circ_007651 | NA | 15 | - | 84300946 | 84301245 | 300 | intergenic |
| novel_circ_007652 | ENSSSCG00000017604 | 12 | + | 31634181 | 31639609 | 545 | annot_exons |
| novel_circ_007653 | NA | 15 | + | 84272147 | 84272904 | 758 | intergenic |
| novel_circ_007654 | ENSSSCG00000008192 | 3 | + | 56352095 | 56378046 | 1231 | exon_intron |
| novel_circ_007655 | ENSSSCG00000002032 | 7 | + | 75855617 | 75856174 | 558 | exon_intron |
| novel_circ_007656 | ENSSSCG00000002509 | 7 | + | 1.21E+08 | 1.21E+08 | 981 | exon_intron |
| novel_circ_007657 | NA | 15 | + | 84289927 | 84290777 | 851 | intergenic |
| novel_circ_007658 | NA | 15 | - | 84262100 | 84262471 | 372 | intergenic |
| novel_circ_007659 | ENSSSCG00000010571 | 14 | - | 1.13E+08 | 1.13E+08 | 255 | annot_exons |
| novel_circ_007660 | NA | 3 | - | 9761614 | 9766498 | 4885 | intergenic |
| novel_circ_007661 | ENSSSCG00000010640 | 14 | + | 1.24E+08 | 1.24E+08 | 5209 | antisense |
| novel_circ_007662 | ENSSSCG00000015326 | 9 | - | 74174651 | 74174886 | 236 | antisense |
| novel_circ_007663 | NA | 15 | - | 84244441 | 84249244 | 4804 | intergenic |
| novel_circ_007664 | ENSSSCG00000017894 | 12 | - | 51182192 | 51182821 | 300 | annot_exons |
| novel_circ_007665 | ENSSSCG00000015631 | 9 | - | 1.37E+08 | 1.37E+08 | 249 | annot_exons |
| novel_circ_007666 | ENSSSCG00000029251 | 13 | - | 30681584 | 30685382 | 249 | annot_exons |
| novel_circ_007667 | ENSSSCG00000009395 | 11 | - | 18284009 | 18290494 | 441 | annot_exons |
| novel_circ_007668 | ENSSSCG00000004639 | 1 | - | 1.21E+08 | 1.21E+08 | 674 | annot_exons |
| novel_circ_007669 | ENSSSCG00000021814 | 11 | + | 713800 | 724858 | 927 | annot_exons |
| novel_circ_007670 | ENSSSCG00000010399 | 14 | - | 90510793 | 90512701 | 346 | annot_exons |
| novel_circ_007671 | ENSSSCG00000012693 | X | - | 1.11E+08 | 1.11E+08 | 73019 | exon_intron |
| novel_circ_007672 | ENSSSCG00000035142 | 8 | + | 53757977 | 53781139 | 23163 | exon_intron |
| novel_circ_007673 | ENSSSCG00000009957 | 14 | + | 43618687 | 43624430 | 639 | annot_exons |
| novel_circ_007674 | ENSSSCG00000011192 | 13 | - | 2799779 | 2800988 | 295 | annot_exons |
| novel_circ_007675 | ENSSSCG00000001792 | 7 | - | 50928383 | 50946333 | 995 | annot_exons |
| novel_circ_007676 | ENSSSCG00000005065 | 1 | + | 1.86E+08 | 1.86E+08 | 442 | annot_exons |
| novel_circ_007677 | ENSSSCG00000004854 | 1 | - | 1.45E+08 | 1.45E+08 | 506 | annot_exons |
| novel_circ_007678 | ENSSSCG00000008167 | 3 | - | 53073338 | 53077544 | 271 | annot_exons |
| novel_circ_007679 | ENSSSCG00000005467 | 1 | + | 2.53E+08 | 2.53E+08 | 431 | annot_exons |
| novel_circ_007680 | ENSSSCG00000007812 | 3 | + | 18826737 | 18836800 | 691 | annot_exons |
| novel_circ_007681 | ENSSSCG00000017626 | 12 | - | 34278523 | 34279927 | 1405 | one_exon |
| novel_circ_007682 | ENSSSCG00000007478 | 17 | - | 53012907 | 53043217 | 731 | annot_exons |
| novel_circ_007683 | ENSSSCG00000000145 | 5 | - | 11418872 | 11419397 | 526 | antisense |
| novel_circ_007684 | ENSSSCG00000003218 | 6 | - | 55305939 | 55306355 | 417 | antisense |
| novel_circ_007685 | ENSSSCG00000029082 | 9 | + | 1.05E+08 | 1.05E+08 | 311 | annot_exons |
| novel_circ_007686 | ENSSSCG00000010882 | 10 | + | 19074731 | 19075159 | 429 | one_exon |
| novel_circ_007687 | ENSSSCG00000035414 | 13 | - | 1.32E+08 | 1.32E+08 | 2566 | intronic |
| novel_circ_007688 | ENSSSCG00000003857 | 6 | - | 1.59E+08 | 1.59E+08 | 559 | annot_exons |
| novel_circ_007689 | ENSSSCG00000001732 | 7 | - | 43609181 | 43616186 | 649 | annot_exons |
| novel_circ_007690 | ENSSSCG00000039157 | 15 | + | 29932361 | 29939777 | 513 | annot_exons |
| novel_circ_007691 | ENSSSCG00000003776 | 6 | - | 1.38E+08 | 1.38E+08 | 313 | annot_exons |
| novel_circ_007692 | ENSSSCG00000009808 | 14 | - | 30933737 | 30936150 | 236 | annot_exons |
| novel_circ_007693 | ENSSSCG00000026959 | 9 | - | 36576888 | 36582172 | 575 | annot_exons |
| novel_circ_007694 | ENSSSCG00000034191 | 2 | + | 42977934 | 42988525 | 561 | annot_exons |
| novel_circ_007695 | ENSSSCG00000009569 | 11 | - | 562633 | 562934 | 302 | one_exon |
| novel_circ_007696 | ENSSSCG00000031271 | 15 | + | 1.05E+08 | 1.05E+08 | 246 | annot_exons |
| novel_circ_007697 | ENSSSCG00000011752 | 13 | + | 1.11E+08 | 1.11E+08 | 212 | annot_exons |
| novel_circ_007698 | ENSSSCG00000000973 | 5 | - | 474119 | 475405 | 385 | annot_exons |
| novel_circ_007699 | ENSSSCG00000032531 | 3 | + | 26689980 | 26705896 | 2077 | annot_exons |
| novel_circ_007700 | ENSSSCG00000026276 | 6 | + | 80443721 | 80451778 | 7145 | exon_intron |
| novel_circ_007701 | ENSSSCG00000022479 | 4 | + | 34731292 | 34731956 | 353 | annot_exons |
| novel_circ_007702 | ENSSSCG00000012062 | 13 | + | 2.01E+08 | 2.01E+08 | 1593 | annot_exons |
| novel_circ_007703 | ENSSSCG00000003812 | 6 | - | 1.49E+08 | 1.49E+08 | 727 | annot_exons |
| novel_circ_007704 | ENSSSCG00000042430 | 15 | - | 84424863 | 84425097 | 235 | one_exon |
| novel_circ_007705 | ENSSSCG00000008363 | 3 | - | 77371816 | 77378404 | 459 | annot_exons |
| novel_circ_007706 | ENSSSCG00000012000 | 13 | + | 1.74E+08 | 1.74E+08 | 262 | annot_exons |
| novel_circ_007707 | ENSSSCG00000039894 | Y | - | 5979493 | 5979666 | 174 | antisense |
| novel_circ_007708 | ENSSSCG00000029196 | 5 | + | 16548828 | 16551131 | 329 | annot_exons |
| novel_circ_007709 | ENSSSCG00000013235 | 2 | - | 15322375 | 15323114 | 489 | annot_exons |
| novel_circ_007710 | ENSSSCG00000009823 | 14 | - | 31861669 | 31864826 | 428 | annot_exons |
| novel_circ_007711 | ENSSSCG00000032834 | 3 | + | 43535845 | 43540475 | 3835 | exon_intron |
| novel_circ_007712 | ENSSSCG00000032517 | 1 | + | 1.2E+08 | 1.2E+08 | 1096 | annot_exons |
| novel_circ_007713 | ENSSSCG00000001779 | 7 | + | 49003745 | 49010575 | 283 | annot_exons |
| novel_circ_007714 | ENSSSCG00000025174 | 15 | - | 1.26E+08 | 1.26E+08 | 411 | annot_exons |
| novel_circ_007715 | ENSSSCG00000016430 | 18 | - | 5319586 | 5326209 | 624 | annot_exons |
| novel_circ_007716 | ENSSSCG00000014948 | 9 | + | 26170413 | 26184794 | 872 | annot_exons |
| novel_circ_007717 | ENSSSCG00000006870 | 4 | - | 1.18E+08 | 1.18E+08 | 462 | annot_exons |
| novel_circ_007718 | NA | 6 | + | 47350552 | 47350986 | 435 | intergenic |
| novel_circ_007719 | ENSSSCG00000002340 | 7 | + | 96543074 | 96555754 | 357 | annot_exons |
| novel_circ_007720 | ENSSSCG00000041063 | 2 | + | 40680514 | 40688349 | 5183 | exon_intron |
| novel_circ_007721 | ENSSSCG00000025673 | 17 | + | 40432052 | 40432260 | 209 | one_exon |
| novel_circ_007722 | ENSSSCG00000004718 | 1 | + | 1.29E+08 | 1.29E+08 | 7356 | exon_intron |
| novel_circ_007723 | ENSSSCG00000003895 | 6 | + | 1.65E+08 | 1.65E+08 | 524 | exon_intron |
| novel_circ_007724 | ENSSSCG00000026940 | 15 | + | 1.05E+08 | 1.05E+08 | 962 | annot_exons |
| novel_circ_007725 | ENSSSCG00000013445 | 2 | - | 76599998 | 76602870 | 2873 | intronic |
| novel_circ_007726 | ENSSSCG00000016397 | 15 | + | 687282 | 692199 | 1050 | annot_exons |
| novel_circ_007727 | ENSSSCG00000001806 | 7 | + | 52173078 | 52183206 | 698 | annot_exons |
| novel_circ_007728 | ENSSSCG00000013566 | 2 | + | 71922023 | 71932721 | 952 | annot_exons |
| novel_circ_007729 | ENSSSCG00000014269 | 2 | - | 1.34E+08 | 1.34E+08 | 317 | annot_exons |
| novel_circ_007730 | ENSSSCG00000028148 | X | - | 27387829 | 27388838 | 390 | annot_exons |
| novel_circ_007731 | ENSSSCG00000006074 | 4 | + | 38086449 | 38111049 | 514 | annot_exons |
| novel_circ_007732 | ENSSSCG00000032531 | 3 | + | 26689980 | 26690578 | 265 | annot_exons |
| novel_circ_007733 | ENSSSCG00000023873 | 4 | + | 34251358 | 34254131 | 491 | annot_exons |
| novel_circ_007734 | ENSSSCG00000032731 | 2 | + | 1.52E+08 | 1.52E+08 | 221 | annot_exons |
| novel_circ_007735 | ENSSSCG00000003407 | 6 | - | 70966643 | 70968053 | 1411 | intronic |
| novel_circ_007736 | ENSSSCG00000006016 | 4 | + | 22038996 | 22052064 | 672 | annot_exons |
| novel_circ_007737 | ENSSSCG00000012885 | 2 | - | 4569845 | 4571734 | 412 | annot_exons |
| novel_circ_007738 | ENSSSCG00000004857 | 1 | - | 1.46E+08 | 1.46E+08 | 387 | intronic |
| novel_circ_007739 | ENSSSCG00000022044 | 1 | - | 2.36E+08 | 2.36E+08 | 3107 | exon_intron |
| novel_circ_007740 | ENSSSCG00000025408 | 13 | - | 25579844 | 25610799 | 588 | annot_exons |
| novel_circ_007741 | ENSSSCG00000014960 | 9 | + | 26899161 | 26932473 | 1614 | annot_exons |
| novel_circ_007742 | NA | 11 | + | 17878811 | 17880117 | 1307 | intergenic |
| novel_circ_007743 | ENSSSCG00000017790 | 12 | + | 45688138 | 45693388 | 359 | annot_exons |
| novel_circ_007744 | ENSSSCG00000010741 | 14 | + | 1.35E+08 | 1.35E+08 | 224 | annot_exons |
| novel_circ_007745 | ENSSSCG00000011068 | 10 | - | 48815859 | 48817035 | 308 | annot_exons |
| novel_circ_007746 | ENSSSCG00000023112 | 8 | - | 31031523 | 31041185 | 774 | annot_exons |
| novel_circ_007747 | ENSSSCG00000036948 | 10 | - | 23658340 | 23659304 | 965 | antisense |
| novel_circ_007748 | ENSSSCG00000005934 | 4 | + | 3108315 | 3137676 | 609 | annot_exons |
| novel_circ_007749 | ENSSSCG00000003693 | 6 | + | 1.04E+08 | 1.04E+08 | 624 | annot_exons |
| novel_circ_007750 | ENSSSCG00000011284 | 13 | + | 26160701 | 26161417 | 234 | annot_exons |
| novel_circ_007751 | ENSSSCG00000020879 | 8 | + | 76509953 | 76609327 | 66482 | exon_intron |
| novel_circ_007752 | ENSSSCG00000036480 | 14 | + | 70743516 | 70744222 | 707 | antisense |
| novel_circ_007753 | ENSSSCG00000003486 | 6 | + | 76170648 | 76174166 | 426 | annot_exons |
| novel_circ_007754 | ENSSSCG00000026110 | 9 | - | 1.05E+08 | 1.05E+08 | 566 | annot_exons |
| novel_circ_007755 | ENSSSCG00000034407 | 5 | + | 42538537 | 42543247 | 553 | annot_exons |
| novel_circ_007756 | ENSSSCG00000017592 | 12 | - | 27474893 | 27499620 | 1229 | annot_exons |
| novel_circ_007757 | ENSSSCG00000040731 | 4 | + | 15375890 | 15385422 | 9533 | exon_intron |
| novel_circ_007758 | ENSSSCG00000003461 | 6 | + | 74817822 | 74824581 | 494 | annot_exons |
| novel_circ_007759 | ENSSSCG00000004207 | 1 | - | 33775136 | 33790484 | 808 | annot_exons |
| novel_circ_007760 | ENSSSCG00000007280 | 17 | + | 37852424 | 37862678 | 619 | annot_exons |
| novel_circ_007761 | ENSSSCG00000051274 | 7 | + | 1.22E+08 | 1.22E+08 | 394 | one_exon |
| novel_circ_007762 | ENSSSCG00000040507 | 2 | - | 80460282 | 80460551 | 270 | antisense |
| novel_circ_007763 | ENSSSCG00000021310 | 10 | - | 14145729 | 14154272 | 284 | annot_exons |
| novel_circ_007764 | ENSSSCG00000044913 | 2 | - | 6798445 | 6800220 | 319 | annot_exons |
| novel_circ_007765 | ENSSSCG00000016397 | 15 | + | 739174 | 743812 | 540 | annot_exons |
| novel_circ_007766 | ENSSSCG00000022545 | 6 | - | 52514851 | 52515359 | 509 | antisense |
| novel_circ_007767 | ENSSSCG00000013235 | 2 | - | 15284635 | 15289402 | 446 | annot_exons |
| novel_circ_007768 | ENSSSCG00000006115 | 4 | + | 44681766 | 44693355 | 652 | annot_exons |
| novel_circ_007769 | ENSSSCG00000028346 | 5 | - | 5056715 | 5059085 | 348 | annot_exons |
| novel_circ_007770 | ENSSSCG00000013299 | 2 | + | 26109805 | 26111727 | 254 | annot_exons |
| novel_circ_007771 | ENSSSCG00000011775 | 13 | + | 1.22E+08 | 1.22E+08 | 367 | annot_exons |
| novel_circ_007772 | ENSSSCG00000032352 | 6 | + | 62633610 | 62635354 | 1743 | exon_intron |
| novel_circ_007773 | ENSSSCG00000001641 | 7 | + | 37665474 | 37705389 | 786 | annot_exons |
| novel_circ_007774 | ENSSSCG00000014304 | 2 | + | 1.37E+08 | 1.37E+08 | 1438 | annot_exons |
| novel_circ_007775 | ENSSSCG00000008412 | 3 | + | 87316814 | 87330087 | 712 | annot_exons |
| novel_circ_007776 | ENSSSCG00000029837 | 11 | + | 25103746 | 25122908 | 410 | annot_exons |
| novel_circ_007777 | ENSSSCG00000018049 | 12 | - | 60366858 | 60376920 | 654 | annot_exons |
| novel_circ_007778 | ENSSSCG00000034730 | 1 | - | 96440774 | 96453655 | 4957 | exon_intron |
| novel_circ_007779 | ENSSSCG00000024373 | 15 | - | 1.31E+08 | 1.31E+08 | 423 | annot_exons |
| novel_circ_007780 | ENSSSCG00000008821 | 8 | + | 38273589 | 38287382 | 974 | annot_exons |
| novel_circ_007781 | ENSSSCG00000017678 | 12 | + | 37636936 | 37669406 | 1110 | annot_exons |
| novel_circ_007782 | ENSSSCG00000028097 | 6 | + | 1.66E+08 | 1.66E+08 | 261 | annot_exons |
| novel_circ_007783 | ENSSSCG00000024267 | 6 | + | 82867016 | 82869332 | 181 | annot_exons |
| novel_circ_007784 | ENSSSCG00000009021 | 8 | + | 78139995 | 78140442 | 448 | one_exon |
| novel_circ_007785 | ENSSSCG00000015668 | 15 | + | 4136904 | 4159229 | 1011 | annot_exons |
| novel_circ_007786 | ENSSSCG00000032377 | 7 | + | 64263201 | 64279355 | 474 | annot_exons |
| novel_circ_007787 | ENSSSCG00000009345 | 11 | + | 9145564 | 9149581 | 267 | annot_exons |
| novel_circ_007788 | ENSSSCG00000016127 | 15 | - | 1.09E+08 | 1.09E+08 | 924 | annot_exons |
| novel_circ_007789 | ENSSSCG00000002311 | 7 | + | 93296032 | 93308785 | 12754 | exon_intron |
| novel_circ_007790 | ENSSSCG00000007592 | 3 | + | 5094441 | 5097362 | 213 | annot_exons |
| novel_circ_007791 | ENSSSCG00000030424 | 3 | + | 23099435 | 23102487 | 346 | annot_exons |
| novel_circ_007792 | ENSSSCG00000028516 | 5 | + | 16322788 | 16333246 | 589 | annot_exons |
| novel_circ_007793 | ENSSSCG00000008372 | 3 | - | 79091804 | 79110848 | 779 | annot_exons |
| novel_circ_007794 | ENSSSCG00000016961 | 16 | + | 47423320 | 47424396 | 230 | annot_exons |
| novel_circ_007795 | ENSSSCG00000021393 | 9 | - | 1.26E+08 | 1.26E+08 | 858 | annot_exons |
| novel_circ_007796 | ENSSSCG00000007431 | 17 | + | 48086245 | 48086724 | 311 | annot_exons |
| novel_circ_007797 | ENSSSCG00000006303 | 4 | - | 82972304 | 82994074 | 725 | annot_exons |
| novel_circ_007798 | ENSSSCG00000006179 | 4 | + | 62243622 | 62268457 | 776 | annot_exons |
| novel_circ_007799 | ENSSSCG00000017255 | 12 | + | 11098684 | 11103750 | 623 | annot_exons |
| novel_circ_007800 | ENSSSCG00000010734 | 14 | - | 1.34E+08 | 1.34E+08 | 449 | annot_exons |
| novel_circ_007801 | ENSSSCG00000012884 | 2 | - | 4407216 | 4412623 | 383 | annot_exons |
| novel_circ_007802 | ENSSSCG00000028327 | 14 | - | 64342539 | 64345385 | 1099 | annot_exons |
| novel_circ_007803 | ENSSSCG00000029251 | 13 | - | 30645234 | 30652373 | 878 | annot_exons |
| novel_circ_007804 | ENSSSCG00000009994 | 14 | + | 46985143 | 46996102 | 378 | annot_exons |
| novel_circ_007805 | ENSSSCG00000024373 | 15 | - | 1.31E+08 | 1.31E+08 | 1248 | annot_exons |
| novel_circ_007806 | ENSSSCG00000010863 | 10 | + | 15083385 | 15094383 | 944 | annot_exons |
| novel_circ_007807 | ENSSSCG00000029621 | 8 | - | 1.25E+08 | 1.25E+08 | 366 | annot_exons |
| novel_circ_007808 | ENSSSCG00000006137 | 4 | - | 50433434 | 50462793 | 1012 | annot_exons |
| novel_circ_007809 | ENSSSCG00000000037 | 5 | + | 6010064 | 6020984 | 364 | annot_exons |
| novel_circ_007810 | ENSSSCG00000020796 | 12 | - | 25799184 | 25814104 | 827 | annot_exons |
| novel_circ_007811 | ENSSSCG00000039701 | 7 | - | 92728724 | 92759618 | 409 | annot_exons |
| novel_circ_007812 | ENSSSCG00000003218 | 6 | + | 55295717 | 55296699 | 273 | annot_exons |
| novel_circ_007813 | ENSSSCG00000003760 | 6 | + | 1.3E+08 | 1.3E+08 | 358 | annot_exons |
| novel_circ_007814 | ENSSSCG00000014820 | 9 | - | 7373041 | 7417219 | 1122 | annot_exons |
| novel_circ_007815 | ENSSSCG00000016509 | 18 | - | 10276122 | 10321515 | 5678 | annot_exons |
| novel_circ_007816 | ENSSSCG00000009010 | 8 | - | 76215987 | 76253449 | 500 | annot_exons |
| novel_circ_007817 | ENSSSCG00000024484 | 10 | + | 12564139 | 12564881 | 743 | one_exon |
| novel_circ_007818 | ENSSSCG00000014927 | 9 | - | 22640189 | 22690654 | 542 | annot_exons |
| novel_circ_007819 | ENSSSCG00000039085 | 13 | - | 2.07E+08 | 2.07E+08 | 1012 | antisense |
| novel_circ_007820 | ENSSSCG00000013588 | 2 | - | 71300458 | 71303012 | 1418 | annot_exons |
| novel_circ_007821 | ENSSSCG00000002752 | 6 | - | 15798085 | 15863316 | 3509 | annot_exons |
| novel_circ_007822 | ENSSSCG00000008357 | 3 | + | 76615866 | 76627539 | 412 | annot_exons |
| novel_circ_007823 | ENSSSCG00000011243 | 13 | + | 22216776 | 22234431 | 384 | annot_exons |
| novel_circ_007824 | ENSSSCG00000005997 | 4 | - | 18819735 | 18827315 | 403 | annot_exons |
| novel_circ_007825 | ENSSSCG00000014364 | 2 | + | 1.42E+08 | 1.42E+08 | 625 | annot_exons |
| novel_circ_007826 | ENSSSCG00000016052 | 15 | + | 95042537 | 95044148 | 1612 | one_exon |
| novel_circ_007827 | ENSSSCG00000006864 | 4 | - | 1.18E+08 | 1.18E+08 | 3626 | exon_intron |
| novel_circ_007828 | ENSSSCG00000005221 | 1 | + | 2.17E+08 | 2.17E+08 | 653 | annot_exons |
| novel_circ_007829 | ENSSSCG00000050772 | 9 | - | 49202381 | 49221301 | 322 | annot_exons |
| novel_circ_007830 | ENSSSCG00000029196 | 5 | + | 16539988 | 16544407 | 337 | annot_exons |
| novel_circ_007831 | ENSSSCG00000009431 | 11 | - | 24857303 | 24858237 | 233 | annot_exons |
| novel_circ_007832 | ENSSSCG00000011849 | 13 | + | 1.34E+08 | 1.34E+08 | 4908 | annot_exons |
| novel_circ_007833 | ENSSSCG00000004293 | 1 | - | 54523093 | 54529700 | 321 | annot_exons |
| novel_circ_007834 | ENSSSCG00000003707 | 6 | - | 1.08E+08 | 1.08E+08 | 550 | annot_exons |
| novel_circ_007835 | ENSSSCG00000012424 | X | - | 60240445 | 60257143 | 285 | annot_exons |
| novel_circ_007836 | ENSSSCG00000051588 | 10 | - | 7247065 | 7247588 | 524 | intronic |
| novel_circ_007837 | ENSSSCG00000009345 | 11 | + | 9166634 | 9170916 | 283 | annot_exons |
| novel_circ_007838 | ENSSSCG00000010034 | 14 | + | 48456454 | 48458261 | 271 | annot_exons |
| novel_circ_007839 | ENSSSCG00000033272 | 18 | - | 1463489 | 1481335 | 5931 | annot_exons |
| novel_circ_007840 | ENSSSCG00000002266 | 7 | - | 86031558 | 86056387 | 1157 | annot_exons |
| novel_circ_007841 | ENSSSCG00000030535 | 1 | - | 9139951 | 9148944 | 259 | annot_exons |
| novel_circ_007842 | ENSSSCG00000027190 | 5 | + | 70169631 | 70170311 | 681 | one_exon |
| novel_circ_007843 | ENSSSCG00000011653 | 13 | + | 77261334 | 77289518 | 327 | annot_exons |
| novel_circ_007844 | ENSSSCG00000037594 | 1 | - | 66358763 | 66360969 | 329 | annot_exons |
| novel_circ_007845 | ENSSSCG00000021285 | 3 | - | 4288138 | 4290175 | 1116 | annot_exons |
| novel_circ_007846 | ENSSSCG00000002819 | 6 | + | 19208372 | 19210655 | 169 | annot_exons |
| novel_circ_007847 | ENSSSCG00000015402 | 9 | + | 97866871 | 97883761 | 199 | annot_exons |
| novel_circ_007848 | ENSSSCG00000009516 | 11 | + | 68173040 | 68181097 | 358 | annot_exons |
| novel_circ_007849 | ENSSSCG00000029311 | 14 | + | 71348983 | 71349948 | 490 | annot_exons |
| novel_circ_007850 | ENSSSCG00000024232 | 5 | - | 45401089 | 45403216 | 470 | annot_exons |
| novel_circ_007851 | ENSSSCG00000016397 | 15 | - | 756585 | 757137 | 553 | antisense |
| novel_circ_007852 | ENSSSCG00000000152 | 5 | + | 11842157 | 11862117 | 372 | annot_exons |
| novel_circ_007853 | ENSSSCG00000009233 | 8 | - | 1.35E+08 | 1.35E+08 | 580 | annot_exons |
| novel_circ_007854 | ENSSSCG00000004121 | 1 | + | 19764591 | 19766628 | 2038 | one_exon |
| novel_circ_007855 | ENSSSCG00000009172 | 8 | + | 1.2E+08 | 1.2E+08 | 201 | annot_exons |
| novel_circ_007856 | ENSSSCG00000014148 | 2 | - | 95657661 | 95677798 | 510 | annot_exons |
| novel_circ_007857 | ENSSSCG00000035730 | 1 | + | 2.08E+08 | 2.08E+08 | 350 | annot_exons |
| novel_circ_007858 | ENSSSCG00000003700 | 6 | - | 1.07E+08 | 1.07E+08 | 291 | annot_exons |
| novel_circ_007859 | ENSSSCG00000015402 | 9 | + | 98232344 | 98235872 | 464 | annot_exons |
| novel_circ_007860 | ENSSSCG00000004138 | 1 | + | 22285747 | 22352249 | 189 | annot_exons |
| novel_circ_007861 | ENSSSCG00000008445 | 3 | + | 94934784 | 94946499 | 657 | annot_exons |
| novel_circ_007862 | ENSSSCG00000016397 | 15 | + | 678037 | 683382 | 210 | annot_exons |
| novel_circ_007863 | ENSSSCG00000009758 | 14 | + | 28218126 | 28219484 | 242 | annot_exons |
| novel_circ_007864 | ENSSSCG00000002859 | 6 | - | 42537042 | 42539106 | 379 | annot_exons |
| novel_circ_007865 | ENSSSCG00000006179 | 4 | + | 62259806 | 62283092 | 331 | annot_exons |
| novel_circ_007866 | ENSSSCG00000004934 | 1 | - | 1.63E+08 | 1.63E+08 | 341 | annot_exons |
| novel_circ_007867 | ENSSSCG00000004114 | 1 | - | 18987938 | 19005183 | 831 | annot_exons |
| novel_circ_007868 | ENSSSCG00000003218 | 6 | + | 55302098 | 55306393 | 1022 | annot_exons |
| novel_circ_007869 | ENSSSCG00000013522 | 2 | - | 73322629 | 73330844 | 357 | annot_exons |
| novel_circ_007870 | ENSSSCG00000046387 | 13 | + | 1.22E+08 | 1.22E+08 | 1182 | annot_exons |
| novel_circ_007871 | ENSSSCG00000020717 | 8 | - | 77359535 | 77370419 | 5449 | exon_intron |
| novel_circ_007872 | ENSSSCG00000000152 | 5 | + | 11842157 | 11878928 | 580 | annot_exons |
| novel_circ_007873 | ENSSSCG00000023865 | 14 | - | 1.33E+08 | 1.33E+08 | 3130 | annot_exons |
| novel_circ_007874 | ENSSSCG00000001805 | 7 | + | 52109083 | 52112345 | 3263 | antisense |
| novel_circ_007875 | ENSSSCG00000002306 | 7 | + | 93001495 | 93018302 | 790 | annot_exons |
| novel_circ_007876 | ENSSSCG00000008591 | 3 | + | 1.15E+08 | 1.15E+08 | 509 | annot_exons |
| novel_circ_007877 | ENSSSCG00000021200 | X | - | 58378203 | 58450493 | 964 | annot_exons |
| novel_circ_007878 | ENSSSCG00000036132 | 6 | + | 51704143 | 51705169 | 1027 | antisense |
| novel_circ_007879 | ENSSSCG00000007805 | 3 | + | 18559247 | 18565263 | 1002 | annot_exons |
| novel_circ_007880 | ENSSSCG00000022479 | 4 | + | 34777295 | 34777538 | 244 | one_exon |
| novel_circ_007881 | ENSSSCG00000000509 | 5 | - | 35632406 | 35633470 | 427 | annot_exons |
| novel_circ_007882 | ENSSSCG00000004576 | 1 | + | 1.11E+08 | 1.11E+08 | 4665 | intronic |
| novel_circ_007883 | ENSSSCG00000004351 | 1 | - | 66395163 | 66397121 | 185 | annot_exons |
| novel_circ_007884 | ENSSSCG00000026116 | 6 | - | 27873867 | 27874335 | 207 | annot_exons |
| novel_circ_007885 | ENSSSCG00000009359 | 11 | - | 12843297 | 12862293 | 1086 | annot_exons |
| novel_circ_007886 | ENSSSCG00000011022 | 10 | + | 41381255 | 41403128 | 1684 | annot_exons |
| novel_circ_007887 | ENSSSCG00000005347 | 1 | + | 2.38E+08 | 2.38E+08 | 267 | annot_exons |
| novel_circ_007888 | ENSSSCG00000001725 | 7 | - | 41679782 | 41687212 | 657 | annot_exons |
| novel_circ_007889 | ENSSSCG00000014168 | 2 | - | 1.03E+08 | 1.03E+08 | 807 | annot_exons |
| novel_circ_007890 | ENSSSCG00000027293 | 16 | + | 49134995 | 49139284 | 371 | annot_exons |
| novel_circ_007891 | ENSSSCG00000010190 | 14 | - | 60414282 | 60415669 | 1388 | antisense |
| novel_circ_007892 | NA | 15 | + | 84267623 | 84267887 | 265 | intergenic |
| novel_circ_007893 | ENSSSCG00000017745 | 12 | + | 43070860 | 43074243 | 361 | annot_exons |
| novel_circ_007894 | ENSSSCG00000025941 | 1 | + | 45173524 | 45177436 | 849 | exon_intron |
| novel_circ_007895 | ENSSSCG00000004983 | 1 | + | 1.7E+08 | 1.7E+08 | 562 | annot_exons |
| novel_circ_007896 | ENSSSCG00000008808 | 8 | - | 35211477 | 35216206 | 264 | annot_exons |
| novel_circ_007897 | ENSSSCG00000000941 | 5 | - | 99261643 | 99299445 | 10596 | annot_exons |
| novel_circ_007898 | ENSSSCG00000010174 | 14 | + | 59194074 | 59194741 | 668 | one_exon |
| novel_circ_007899 | ENSSSCG00000024019 | 1 | - | 8917288 | 8917913 | 626 | one_exon |
| novel_circ_007900 | ENSSSCG00000010190 | 14 | + | 60415019 | 60415200 | 182 | one_exon |
| novel_circ_007901 | ENSSSCG00000002799 | 6 | - | 20298796 | 20321216 | 2179 | annot_exons |
| novel_circ_007902 | ENSSSCG00000011765 | 13 | + | 1.18E+08 | 1.18E+08 | 1608 | annot_exons |
| novel_circ_007903 | ENSSSCG00000017259 | 12 | - | 11669306 | 11672541 | 531 | annot_exons |
| novel_circ_007904 | ENSSSCG00000011235 | 13 | - | 19230686 | 19234893 | 290 | annot_exons |
| novel_circ_007905 | ENSSSCG00000025478 | 6 | + | 1.07E+08 | 1.07E+08 | 1428 | annot_exons |
| novel_circ_007906 | ENSSSCG00000003909 | 6 | + | 1.65E+08 | 1.65E+08 | 566 | annot_exons |
| novel_circ_007907 | ENSSSCG00000001924 | 7 | - | 60731692 | 60757895 | 468 | annot_exons |
| novel_circ_007908 | ENSSSCG00000012149 | X | - | 13305101 | 13306625 | 290 | annot_exons |
| novel_circ_007909 | ENSSSCG00000038561 | 15 | - | 84412739 | 84412884 | 146 | one_exon |
| novel_circ_007910 | ENSSSCG00000015695 | 15 | - | 16786136 | 16790385 | 690 | annot_exons |
| novel_circ_007911 | ENSSSCG00000012619 | X | - | 98054302 | 98064338 | 446 | annot_exons |
| novel_circ_007912 | ENSSSCG00000008581 | 3 | - | 1.14E+08 | 1.14E+08 | 528 | annot_exons |
| novel_circ_007913 | NA | 7 | + | 1.22E+08 | 1.22E+08 | 4121 | intergenic |
| novel_circ_007914 | ENSSSCG00000010300 | 14 | - | 76278036 | 76285566 | 602 | annot_exons |
| novel_circ_007915 | ENSSSCG00000037384 | 1 | - | 1.26E+08 | 1.26E+08 | 372 | annot_exons |
| novel_circ_007916 | ENSSSCG00000006872 | 4 | - | 1.18E+08 | 1.18E+08 | 504 | annot_exons |
| novel_circ_007917 | ENSSSCG00000034191 | 2 | + | 42861755 | 42949127 | 599 | annot_exons |
| novel_circ_007918 | ENSSSCG00000016291 | 15 | + | 1.33E+08 | 1.33E+08 | 200 | annot_exons |
| novel_circ_007919 | ENSSSCG00000016464 | 18 | - | 6934952 | 6936925 | 644 | annot_exons |
| novel_circ_007920 | ENSSSCG00000028805 | 6 | - | 99933264 | 99936447 | 3184 | intronic |
| novel_circ_007921 | ENSSSCG00000010468 | 14 | - | 1.04E+08 | 1.04E+08 | 205 | annot_exons |
| novel_circ_007922 | ENSSSCG00000013410 | 2 | - | 49740211 | 49759956 | 840 | annot_exons |
| novel_circ_007923 | ENSSSCG00000021644 | 6 | + | 34506591 | 34515277 | 741 | annot_exons |
| novel_circ_007924 | ENSSSCG00000004454 | 1 | + | 82875117 | 82891579 | 342 | annot_exons |
| novel_circ_007925 | ENSSSCG00000011042 | 10 | + | 45130708 | 45142435 | 438 | annot_exons |
| novel_circ_007926 | ENSSSCG00000010987 | 10 | + | 32855276 | 32862936 | 466 | annot_exons |
| novel_circ_007927 | ENSSSCG00000015310 | 9 | + | 72040304 | 72041563 | 371 | annot_exons |
| novel_circ_007928 | ENSSSCG00000029082 | 9 | + | 1.05E+08 | 1.05E+08 | 611 | annot_exons |
| novel_circ_007929 | ENSSSCG00000008513 | 3 | - | 1.07E+08 | 1.07E+08 | 240 | annot_exons |
| novel_circ_007930 | ENSSSCG00000001065 | 7 | - | 12556288 | 12557058 | 771 | exon_intron |
| novel_circ_007931 | ENSSSCG00000027765 | 1 | + | 1.75E+08 | 1.75E+08 | 334 | annot_exons |
| novel_circ_007932 | ENSSSCG00000038006 | 6 | + | 69949133 | 69963755 | 426 | annot_exons |
| novel_circ_007933 | ENSSSCG00000038187 | 9 | + | 77529970 | 77532203 | 337 | annot_exons |
| novel_circ_007934 | ENSSSCG00000028148 | X | - | 28616574 | 28675535 | 685 | annot_exons |
| novel_circ_007935 | ENSSSCG00000002869 | 6 | + | 43867657 | 43870512 | 294 | annot_exons |
| novel_circ_007936 | ENSSSCG00000012108 | X | + | 6883577 | 6884579 | 1003 | exon_intron |
| novel_circ_007937 | ENSSSCG00000022758 | 15 | - | 1.18E+08 | 1.18E+08 | 382 | annot_exons |
| novel_circ_007938 | ENSSSCG00000010687 | 14 | + | 1.3E+08 | 1.3E+08 | 247 | annot_exons |
| novel_circ_007939 | ENSSSCG00000028225 | 7 | - | 1.16E+08 | 1.16E+08 | 534 | annot_exons |
| novel_circ_007940 | ENSSSCG00000015335 | 9 | + | 75733606 | 75737973 | 267 | annot_exons |
| novel_circ_007941 | ENSSSCG00000030197 | 7 | + | 42298465 | 42321213 | 1006 | annot_exons |
| novel_circ_007942 | ENSSSCG00000028454 | 3 | - | 33641767 | 33642042 | 276 | one_exon |
| novel_circ_007943 | ENSSSCG00000032531 | 3 | + | 26689980 | 26703165 | 1546 | annot_exons |
| novel_circ_007944 | ENSSSCG00000033010 | 2 | + | 9207602 | 9207753 | 152 | intronic |
| novel_circ_007945 | ENSSSCG00000008515 | 3 | + | 1.08E+08 | 1.08E+08 | 4931 | annot_exons |
| novel_circ_007946 | ENSSSCG00000011424 | 13 | + | 33880624 | 33882569 | 183 | annot_exons |
| novel_circ_007947 | ENSSSCG00000015211 | 9 | + | 52419293 | 52419957 | 665 | exon_intron |
| novel_circ_007948 | ENSSSCG00000025344 | 6 | - | 89604373 | 89605240 | 597 | annot_exons |
| novel_circ_007949 | ENSSSCG00000012743 | X | + | 1.22E+08 | 1.22E+08 | 918 | annot_exons |
| novel_circ_007950 | ENSSSCG00000011317 | 13 | - | 29117004 | 29121316 | 2681 | annot_exons |
| novel_circ_007951 | ENSSSCG00000011538 | 13 | + | 64959581 | 64960117 | 537 | intronic |
| novel_circ_007952 | ENSSSCG00000021353 | 17 | - | 16845395 | 16869145 | 439 | annot_exons |
| novel_circ_007953 | ENSSSCG00000008991 | 8 | + | 73884248 | 73893587 | 446 | annot_exons |
| novel_circ_007954 | NA | 7 | + | 68168858 | 68176204 | 7347 | intergenic |
| novel_circ_007955 | ENSSSCG00000010190 | 14 | - | 60415352 | 60415651 | 300 | antisense |
| novel_circ_007956 | ENSSSCG00000004328 | 1 | - | 57788272 | 57800994 | 870 | annot_exons |
| novel_circ_007957 | ENSSSCG00000005126 | 1 | + | 1.96E+08 | 1.96E+08 | 362 | annot_exons |
| novel_circ_007958 | ENSSSCG00000014304 | 2 | + | 1.37E+08 | 1.37E+08 | 1623 | annot_exons |
| novel_circ_007959 | ENSSSCG00000011825 | 13 | - | 1.31E+08 | 1.31E+08 | 1776 | annot_exons |
| novel_circ_007960 | ENSSSCG00000037647 | 13 | - | 82881417 | 82893436 | 3364 | exon_intron |
| novel_circ_007961 | ENSSSCG00000032170 | 2 | + | 74776555 | 74777761 | 1207 | one_exon |
| novel_circ_007962 | ENSSSCG00000007058 | 17 | + | 18318476 | 18337608 | 877 | annot_exons |
| novel_circ_007963 | ENSSSCG00000002266 | 7 | - | 86024175 | 86032780 | 836 | annot_exons |
| novel_circ_007964 | ENSSSCG00000015492 | 9 | + | 1.16E+08 | 1.16E+08 | 893 | annot_exons |
| novel_circ_007965 | ENSSSCG00000006274 | 4 | + | 79718560 | 79719089 | 280 | annot_exons |
| novel_circ_007966 | ENSSSCG00000005602 | 1 | - | 2.66E+08 | 2.66E+08 | 537 | antisense |
| novel_circ_007967 | ENSSSCG00000004114 | 1 | - | 18940256 | 18944020 | 245 | annot_exons |
| novel_circ_007968 | ENSSSCG00000009436 | 11 | + | 25614345 | 25618056 | 282 | annot_exons |
| novel_circ_007969 | ENSSSCG00000010802 | 10 | + | 759106 | 761696 | 261 | annot_exons |
| novel_circ_007970 | ENSSSCG00000032916 | 1 | + | 2552419 | 2552777 | 359 | one_exon |
| novel_circ_007971 | ENSSSCG00000012884 | 2 | - | 4431225 | 4459058 | 1045 | annot_exons |
| novel_circ_007972 | ENSSSCG00000048791 | 15 | - | 20022022 | 20084083 | 8736 | annot_exons |
| novel_circ_007973 | NA | 15 | - | 84265057 | 84265302 | 246 | intergenic |
| novel_circ_007974 | ENSSSCG00000005110 | 1 | - | 1.94E+08 | 1.94E+08 | 492 | annot_exons |
| novel_circ_007975 | ENSSSCG00000011261 | 13 | + | 23747155 | 23751782 | 433 | annot_exons |
| novel_circ_007976 | ENSSSCG00000014121 | 2 | + | 89083911 | 89085932 | 336 | annot_exons |
| novel_circ_007977 | ENSSSCG00000003512 | 6 | - | 79075061 | 79075866 | 384 | annot_exons |
| novel_circ_007978 | ENSSSCG00000006785 | 4 | - | 1.09E+08 | 1.09E+08 | 214 | annot_exons |
| novel_circ_007979 | ENSSSCG00000010304 | 14 | + | 76445541 | 76447197 | 1657 | antisense |
| novel_circ_007980 | NA | 15 | - | 84365150 | 84371281 | 6132 | intergenic |
| novel_circ_007981 | ENSSSCG00000015857 | 15 | - | 59241718 | 59244748 | 351 | annot_exons |
| novel_circ_007982 | ENSSSCG00000015844 | 15 | + | 54405977 | 54411041 | 218 | annot_exons |
| novel_circ_007983 | ENSSSCG00000005599 | 1 | + | 2.66E+08 | 2.66E+08 | 312 | annot_exons |
| novel_circ_007984 | NA | 15 | + | 84276753 | 84277559 | 807 | intergenic |
| novel_circ_007985 | ENSSSCG00000011841 | 13 | - | 1.33E+08 | 1.33E+08 | 371 | annot_exons |
| novel_circ_007986 | ENSSSCG00000004207 | 1 | - | 33789904 | 33799804 | 675 | annot_exons |
| novel_circ_007987 | ENSSSCG00000014934 | 9 | - | 23192217 | 23199299 | 460 | annot_exons |
| novel_circ_007988 | ENSSSCG00000021042 | 13 | - | 2.03E+08 | 2.03E+08 | 286 | annot_exons |
| novel_circ_007989 | ENSSSCG00000016853 | 16 | - | 24151895 | 24158878 | 879 | annot_exons |
| novel_circ_007990 | ENSSSCG00000016640 | 18 | - | 32035237 | 32035879 | 643 | antisense |
| novel_circ_007991 | ENSSSCG00000009230 | 8 | + | 1.34E+08 | 1.34E+08 | 303 | annot_exons |
| novel_circ_007992 | ENSSSCG00000004854 | 1 | - | 1.45E+08 | 1.45E+08 | 501 | annot_exons |
| novel_circ_007993 | ENSSSCG00000010190 | 14 | - | 60414564 | 60414845 | 282 | antisense |
| novel_circ_007994 | ENSSSCG00000008803 | 8 | - | 33422988 | 33437049 | 504 | annot_exons |
| novel_circ_007995 | ENSSSCG00000003917 | 6 | + | 1.66E+08 | 1.66E+08 | 437 | annot_exons |
| novel_circ_007996 | ENSSSCG00000032531 | 3 | + | 26701499 | 26703165 | 412 | annot_exons |
| novel_circ_007997 | ENSSSCG00000010586 | 14 | + | 1.14E+08 | 1.14E+08 | 568 | annot_exons |
| novel_circ_007998 | ENSSSCG00000005356 | 1 | + | 2.39E+08 | 2.39E+08 | 312 | annot_exons |
| novel_circ_007999 | ENSSSCG00000007955 | 3 | - | 38741343 | 38745384 | 360 | annot_exons |
| novel_circ_008000 | ENSSSCG00000008222 | 3 | + | 58236910 | 58248575 | 241 | annot_exons |
| novel_circ_008001 | ENSSSCG00000040286 | 6 | - | 34107266 | 34110051 | 409 | annot_exons |
| novel_circ_008002 | ENSSSCG00000030095 | 9 | + | 41645870 | 41646122 | 253 | intronic |
| novel_circ_008003 | ENSSSCG00000033753 | 3 | - | 1.15E+08 | 1.15E+08 | 543 | annot_exons |
| novel_circ_008004 | ENSSSCG00000022240 | X | + | 20809488 | 20813374 | 475 | annot_exons |
| novel_circ_008005 | ENSSSCG00000016395 | 15 | - | 809993 | 812599 | 555 | annot_exons |
| novel_circ_008006 | ENSSSCG00000038766 | 3 | - | 1277701 | 1300791 | 701 | annot_exons |
| novel_circ_008007 | ENSSSCG00000003580 | 6 | - | 85107261 | 85118705 | 631 | annot_exons |
| novel_circ_008008 | ENSSSCG00000030486 | 13 | - | 1.59E+08 | 1.59E+08 | 171 | one_exon |
| novel_circ_008009 | ENSSSCG00000015140 | 9 | + | 49984504 | 49984781 | 278 | antisense |
| novel_circ_008010 | ENSSSCG00000010652 | 14 | + | 1.25E+08 | 1.25E+08 | 968 | annot_exons |
| novel_circ_008011 | ENSSSCG00000023761 | 6 | - | 81230087 | 81233688 | 341 | annot_exons |
| novel_circ_008012 | ENSSSCG00000032622 | 14 | + | 6745390 | 6746054 | 323 | annot_exons |
| novel_circ_008013 | ENSSSCG00000015857 | 15 | - | 59173726 | 59181694 | 532 | annot_exons |
| novel_circ_008014 | ENSSSCG00000016850 | 16 | - | 23736824 | 23745763 | 7161 | exon_intron |
| novel_circ_008015 | ENSSSCG00000035488 | X | + | 96997114 | 97002248 | 463 | annot_exons |
| novel_circ_008016 | ENSSSCG00000031206 | AEMK02000258.1 | + | 275347 | 277808 | 371 | annot_exons |
| novel_circ_008017 | ENSSSCG00000003398 | 6 | + | 70383718 | 70398243 | 415 | annot_exons |
| novel_circ_008018 | ENSSSCG00000008132 | 3 | + | 47004817 | 47007039 | 450 | annot_exons |
| novel_circ_008019 | ENSSSCG00000015692 | 15 | - | 16258946 | 16277686 | 431 | annot_exons |
| novel_circ_008020 | ENSSSCG00000036537 | 2 | - | 75212487 | 75214231 | 324 | annot_exons |
| novel_circ_008021 | ENSSSCG00000000469 | 5 | + | 29661935 | 29663091 | 384 | annot_exons |
| novel_circ_008022 | ENSSSCG00000005582 | 1 | - | 2.64E+08 | 2.64E+08 | 821 | annot_exons |
| novel_circ_008023 | ENSSSCG00000014902 | 9 | + | 17351072 | 17386234 | 826 | annot_exons |
| novel_circ_008024 | NA | 15 | - | 84372770 | 84374005 | 1236 | intergenic |
| novel_circ_008025 | NA | 15 | - | 84466708 | 84466950 | 243 | intergenic |
| novel_circ_008026 | ENSSSCG00000004570 | 1 | + | 1.09E+08 | 1.09E+08 | 2330 | antisense |
| novel_circ_008027 | ENSSSCG00000024754 | 18 | - | 40175333 | 40184681 | 364 | annot_exons |
| novel_circ_008028 | ENSSSCG00000010931 | 10 | - | 24908903 | 24912841 | 741 | annot_exons |
| novel_circ_008029 | NA | 15 | - | 84354981 | 84355914 | 934 | intergenic |
| novel_circ_008030 | ENSSSCG00000002397 | 7 | - | 1E+08 | 1E+08 | 569 | annot_exons |
| novel_circ_008031 | ENSSSCG00000011454 | 13 | - | 35084780 | 35101407 | 249 | annot_exons |
| novel_circ_008032 | ENSSSCG00000012328 | X | - | 46307819 | 46308992 | 402 | annot_exons |
| novel_circ_008033 | ENSSSCG00000011047 | 10 | + | 46538292 | 46563430 | 480 | annot_exons |
| novel_circ_008034 | ENSSSCG00000035045 | 14 | + | 1.36E+08 | 1.36E+08 | 399 | annot_exons |
| novel_circ_008035 | ENSSSCG00000011027 | 10 | - | 42646861 | 42647382 | 238 | annot_exons |
| novel_circ_008036 | ENSSSCG00000010358 | 14 | + | 87695612 | 87699150 | 326 | annot_exons |
| novel_circ_008037 | ENSSSCG00000032556 | 3 | - | 18259622 | 18260127 | 506 | antisense |
| novel_circ_008038 | ENSSSCG00000010485 | 14 | + | 1.06E+08 | 1.06E+08 | 297 | annot_exons |
| novel_circ_008039 | ENSSSCG00000004065 | 1 | - | 11750859 | 11753865 | 398 | annot_exons |
| novel_circ_008040 | ENSSSCG00000011653 | 13 | + | 77255763 | 77263360 | 349 | exon_intron |
| novel_circ_008041 | ENSSSCG00000011022 | 10 | + | 41339780 | 41360718 | 993 | annot_exons |
| novel_circ_008042 | ENSSSCG00000000571 | 5 | + | 51558424 | 51561815 | 504 | annot_exons |
| novel_circ_008043 | ENSSSCG00000009172 | 8 | + | 1.2E+08 | 1.2E+08 | 897 | annot_exons |
| novel_circ_008044 | ENSSSCG00000014376 | 2 | + | 1.42E+08 | 1.42E+08 | 371 | annot_exons |
| novel_circ_008045 | ENSSSCG00000023548 | 8 | - | 1.16E+08 | 1.16E+08 | 1160 | annot_exons |
| novel_circ_008046 | ENSSSCG00000023445 | 18 | - | 40685239 | 40710541 | 1480 | annot_exons |
| novel_circ_008047 | ENSSSCG00000025228 | 15 | + | 1.38E+08 | 1.38E+08 | 428 | annot_exons |
| novel_circ_008048 | ENSSSCG00000000755 | 5 | + | 68354014 | 68362580 | 335 | annot_exons |
| novel_circ_008049 | ENSSSCG00000025703 | 1 | - | 77203141 | 77206654 | 642 | annot_exons |
| novel_circ_008050 | ENSSSCG00000036480 | 14 | - | 70489004 | 70502038 | 12581 | exon_intron |
| novel_circ_008051 | ENSSSCG00000010544 | 14 | - | 1.11E+08 | 1.11E+08 | 294 | annot_exons |
| novel_circ_008052 | ENSSSCG00000016922 | 16 | + | 36231146 | 36231510 | 365 | intronic |
| novel_circ_008053 | ENSSSCG00000038437 | AEMK02000682.1 | - | 287165 | 300872 | 1013 | annot_exons |
| novel_circ_008054 | ENSSSCG00000015840 | 15 | - | 54027500 | 54037592 | 472 | annot_exons |
| novel_circ_008055 | ENSSSCG00000009825 | 14 | - | 31911160 | 31916240 | 453 | annot_exons |
| novel_circ_008056 | NA | 15 | + | 84467953 | 84469535 | 1583 | intergenic |
| novel_circ_008057 | ENSSSCG00000014331 | 2 | + | 1.4E+08 | 1.4E+08 | 1438 | annot_exons |
| novel_circ_008058 | ENSSSCG00000000066 | 5 | - | 7266047 | 7272170 | 680 | annot_exons |
| novel_circ_008059 | ENSSSCG00000038913 | 14 | - | 71144383 | 71145521 | 234 | annot_exons |
| novel_circ_008060 | ENSSSCG00000015792 | 15 | - | 46394953 | 46405687 | 1913 | annot_exons |
| novel_circ_008061 | ENSSSCG00000009492 | 11 | + | 63740098 | 63751684 | 983 | annot_exons |
| novel_circ_008062 | ENSSSCG00000007264 | 17 | - | 36889868 | 36896286 | 637 | annot_exons |
| novel_circ_008063 | ENSSSCG00000028572 | 6 | + | 68500550 | 68502648 | 222 | annot_exons |
| novel_circ_008064 | ENSSSCG00000017272 | 12 | - | 13566706 | 13571493 | 444 | annot_exons |
| novel_circ_008065 | ENSSSCG00000007364 | 17 | + | 46213341 | 46218205 | 491 | annot_exons |
| novel_circ_008066 | ENSSSCG00000051610 | 18 | + | 17855522 | 17947161 | 564 | annot_exons |
| novel_circ_008067 | ENSSSCG00000024486 | 17 | + | 11844341 | 11850285 | 302 | annot_exons |
| novel_circ_008068 | ENSSSCG00000000625 | 5 | + | 60221275 | 60249461 | 1093 | annot_exons |
| novel_circ_008069 | ENSSSCG00000008179 | 3 | + | 54832393 | 54850266 | 959 | annot_exons |
| novel_circ_008070 | ENSSSCG00000013858 | 2 | + | 61110209 | 61110412 | 204 | one_exon |
| novel_circ_008071 | ENSSSCG00000017669 | 12 | - | 36103170 | 36105578 | 449 | annot_exons |
| novel_circ_008072 | ENSSSCG00000004935 | 1 | + | 1.64E+08 | 1.64E+08 | 239 | annot_exons |
| novel_circ_008073 | ENSSSCG00000035564 | 15 | + | 15948817 | 15953002 | 447 | annot_exons |
| novel_circ_008074 | ENSSSCG00000024794 | 6 | - | 75371765 | 75375460 | 419 | annot_exons |
| novel_circ_008075 | ENSSSCG00000031175 | 8 | + | 47793837 | 47801426 | 332 | annot_exons |
| novel_circ_008076 | ENSSSCG00000009836 | 14 | + | 32871222 | 32879031 | 872 | annot_exons |
| novel_circ_008077 | ENSSSCG00000011075 | 10 | - | 50814869 | 50819992 | 293 | annot_exons |
| novel_circ_008078 | ENSSSCG00000004070 | 1 | - | 12056480 | 12061089 | 540 | annot_exons |
| novel_circ_008079 | ENSSSCG00000008368 | 3 | - | 77993506 | 78014790 | 20399 | exon_intron |
| novel_circ_008080 | ENSSSCG00000004528 | 1 | - | 1.04E+08 | 1.04E+08 | 756 | annot_exons |
| novel_circ_008081 | ENSSSCG00000029571 | 5 | - | 23118132 | 23121185 | 3054 | exon_intron |
| novel_circ_008082 | ENSSSCG00000033624 | 13 | - | 65374802 | 65376965 | 2164 | exon_intron |
| novel_circ_008083 | ENSSSCG00000000952 | 5 | - | 1.04E+08 | 1.04E+08 | 1669 | annot_exons |
| novel_circ_008084 | ENSSSCG00000026004 | 5 | + | 29732023 | 29741425 | 9403 | exon_intron |
| novel_circ_008085 | ENSSSCG00000040434 | 3 | + | 56975159 | 56975594 | 277 | annot_exons |
| novel_circ_008086 | ENSSSCG00000015859 | 15 | + | 59336611 | 59353857 | 608 | annot_exons |
| novel_circ_008087 | ENSSSCG00000010212 | 14 | - | 63495089 | 63502884 | 487 | annot_exons |
| novel_circ_008088 | ENSSSCG00000005221 | 1 | + | 2.17E+08 | 2.17E+08 | 1108 | annot_exons |
| novel_circ_008089 | ENSSSCG00000001878 | 7 | + | 58137959 | 58146958 | 534 | annot_exons |
| novel_circ_008090 | ENSSSCG00000010190 | 14 | - | 60412993 | 60414137 | 1145 | antisense |
| novel_circ_008091 | ENSSSCG00000004936 | 1 | - | 1.64E+08 | 1.64E+08 | 1384 | annot_exons |
| novel_circ_008092 | ENSSSCG00000011854 | 13 | + | 1.34E+08 | 1.34E+08 | 378 | annot_exons |
| novel_circ_008093 | ENSSSCG00000020912 | 14 | + | 1.03E+08 | 1.03E+08 | 332 | annot_exons |
| novel_circ_008094 | ENSSSCG00000015853 | 15 | + | 56517881 | 56520557 | 527 | annot_exons |
| novel_circ_008095 | ENSSSCG00000003438 | 6 | + | 72284692 | 72292011 | 2649 | annot_exons |
| novel_circ_008096 | NA | 15 | - | 84300539 | 84300856 | 318 | intergenic |
| novel_circ_008097 | ENSSSCG00000051274 | 7 | + | 1.22E+08 | 1.22E+08 | 4732 | one_exon |
| novel_circ_008098 | ENSSSCG00000004070 | 1 | - | 12053595 | 12061089 | 654 | annot_exons |
| novel_circ_008099 | ENSSSCG00000011163 | 10 | + | 69028579 | 69037404 | 410 | annot_exons |
| novel_circ_008100 | ENSSSCG00000002740 | 6 | - | 14754391 | 14755554 | 276 | annot_exons |
| novel_circ_008101 | ENSSSCG00000011025 | 10 | + | 42063516 | 42117737 | 54222 | intronic |
| novel_circ_008102 | ENSSSCG00000000720 | 5 | + | 65804711 | 65807023 | 2313 | one_exon |
| novel_circ_008103 | ENSSSCG00000015969 | 15 | - | 80680234 | 80681161 | 434 | annot_exons |
| novel_circ_008104 | ENSSSCG00000008575 | 3 | + | 1.13E+08 | 1.13E+08 | 1943 | annot_exons |
| novel_circ_008105 | ENSSSCG00000010240 | 14 | - | 71615773 | 71625769 | 257 | annot_exons |
| novel_circ_008106 | ENSSSCG00000006841 | 4 | + | 1.11E+08 | 1.11E+08 | 457 | annot_exons |
| novel_circ_008107 | ENSSSCG00000015940 | 15 | + | 76207329 | 76226908 | 557 | annot_exons |
| novel_circ_008108 | ENSSSCG00000000555 | 5 | + | 47178540 | 47178894 | 216 | annot_exons |
| novel_circ_008109 | ENSSSCG00000008179 | 3 | + | 54832393 | 54838629 | 334 | annot_exons |
| novel_circ_008110 | ENSSSCG00000021292 | 6 | - | 1.6E+08 | 1.6E+08 | 353 | annot_exons |
| novel_circ_008111 | ENSSSCG00000046212 | 1 | + | 1.23E+08 | 1.23E+08 | 5690 | exon_intron |
| novel_circ_008112 | ENSSSCG00000017101 | 16 | - | 74328311 | 74343745 | 391 | annot_exons |
| novel_circ_008113 | ENSSSCG00000009043 | 8 | - | 83320683 | 83322381 | 354 | annot_exons |
| novel_circ_008114 | ENSSSCG00000010190 | 14 | - | 60414148 | 60414827 | 680 | antisense |
| novel_circ_008115 | ENSSSCG00000029113 | 11 | - | 50762620 | 50773425 | 940 | annot_exons |
| novel_circ_008116 | ENSSSCG00000026196 | 8 | + | 1.3E+08 | 1.3E+08 | 367 | annot_exons |
| novel_circ_008117 | ENSSSCG00000026851 | 6 | + | 1.08E+08 | 1.08E+08 | 245 | annot_exons |
| novel_circ_008118 | ENSSSCG00000003976 | 6 | - | 1.7E+08 | 1.7E+08 | 299 | annot_exons |
| novel_circ_008119 | ENSSSCG00000008388 | 3 | - | 80880275 | 80880696 | 318 | annot_exons |
| novel_circ_008120 | ENSSSCG00000040401 | 17 | + | 62522365 | 62522991 | 627 | antisense |
| novel_circ_008121 | ENSSSCG00000001971 | 7 | + | 68401378 | 68402796 | 280 | annot_exons |
| novel_circ_008122 | NA | 15 | + | 84232734 | 84232960 | 227 | intergenic |
| novel_circ_008123 | ENSSSCG00000048853 | 13 | + | 12699049 | 12732176 | 32802 | exon_intron |
| novel_circ_008124 | ENSSSCG00000010470 | 14 | - | 1.04E+08 | 1.04E+08 | 204 | annot_exons |
| novel_circ_008125 | ENSSSCG00000004997 | 1 | + | 1.75E+08 | 1.75E+08 | 442 | annot_exons |
| novel_circ_008126 | ENSSSCG00000024088 | 1 | - | 1.09E+08 | 1.09E+08 | 824 | annot_exons |
| novel_circ_008127 | ENSSSCG00000036614 | 3 | - | 39777436 | 39777725 | 290 | antisense |
| novel_circ_008128 | ENSSSCG00000012459 | X | + | 68403938 | 68406594 | 283 | annot_exons |
| novel_circ_008129 | ENSSSCG00000015566 | 9 | - | 1.26E+08 | 1.26E+08 | 792 | annot_exons |
| novel_circ_008130 | ENSSSCG00000017125 | 12 | - | 573171 | 573877 | 504 | annot_exons |
| novel_circ_008131 | ENSSSCG00000021814 | 11 | + | 713800 | 719582 | 660 | annot_exons |
| novel_circ_008132 | ENSSSCG00000015386 | 9 | - | 91752357 | 91770742 | 2095 | annot_exons |
| novel_circ_008133 | ENSSSCG00000014022 | 2 | + | 78964727 | 78968273 | 804 | annot_exons |
| novel_circ_008134 | ENSSSCG00000016692 | 18 | - | 44837492 | 44838430 | 324 | annot_exons |
| novel_circ_008135 | ENSSSCG00000008504 | 3 | - | 1.04E+08 | 1.04E+08 | 538 | annot_exons |
| novel_circ_008136 | ENSSSCG00000014399 | 2 | + | 1.45E+08 | 1.45E+08 | 253 | annot_exons |
| novel_circ_008137 | ENSSSCG00000006927 | 4 | - | 1.28E+08 | 1.28E+08 | 1477 | annot_exons |
| novel_circ_008138 | ENSSSCG00000014794 | 9 | + | 6386178 | 6388462 | 431 | annot_exons |
| novel_circ_008139 | ENSSSCG00000007454 | 17 | - | 49278762 | 49281294 | 563 | annot_exons |
| novel_circ_008140 | ENSSSCG00000018056 | 12 | + | 23561803 | 23569557 | 1090 | annot_exons |
| novel_circ_008141 | NA | 15 | + | 84231533 | 84231949 | 417 | intergenic |
| novel_circ_008142 | NA | 2 | - | 6750704 | 6751233 | 530 | intergenic |
| novel_circ_008143 | ENSSSCG00000034639 | 18 | - | 7933889 | 7939015 | 647 | annot_exons |
| novel_circ_008144 | ENSSSCG00000021042 | 13 | - | 2.03E+08 | 2.03E+08 | 300 | annot_exons |
| novel_circ_008145 | NA | 15 | + | 84282595 | 84282995 | 401 | intergenic |
| novel_circ_008146 | ENSSSCG00000004717 | 1 | + | 1.28E+08 | 1.28E+08 | 791 | annot_exons |
| novel_circ_008147 | ENSSSCG00000002441 | 7 | - | 1.13E+08 | 1.13E+08 | 3823 | annot_exons |
| novel_circ_008148 | ENSSSCG00000011769 | 13 | + | 1.19E+08 | 1.19E+08 | 503 | annot_exons |
| novel_circ_008149 | ENSSSCG00000016036 | 15 | + | 94016853 | 94019208 | 421 | annot_exons |
| novel_circ_008150 | ENSSSCG00000028877 | 7 | + | 91622714 | 91636049 | 441 | annot_exons |
| novel_circ_008151 | ENSSSCG00000038913 | 14 | - | 71145355 | 71162086 | 2302 | annot_exons |
| novel_circ_008152 | ENSSSCG00000037594 | 1 | - | 66360779 | 66372342 | 976 | annot_exons |
| novel_circ_008153 | ENSSSCG00000007311 | 17 | + | 39115371 | 39144798 | 1224 | annot_exons |
| novel_circ_008154 | ENSSSCG00000029082 | 9 | + | 1.05E+08 | 1.05E+08 | 426 | annot_exons |
| novel_circ_008155 | ENSSSCG00000022534 | 4 | + | 1.26E+08 | 1.26E+08 | 218 | annot_exons |
| novel_circ_008156 | ENSSSCG00000014917 | 9 | + | 20260938 | 20261456 | 300 | annot_exons |
| novel_circ_008157 | ENSSSCG00000008721 | 8 | + | 3012369 | 3014564 | 301 | annot_exons |
| novel_circ_008158 | ENSSSCG00000007203 | 17 | + | 34722015 | 34730464 | 484 | annot_exons |
| novel_circ_008159 | ENSSSCG00000016397 | 15 | + | 633999 | 634297 | 213 | annot_exons |
| novel_circ_008160 | ENSSSCG00000027898 | 5 | + | 93006195 | 93013228 | 579 | annot_exons |
| novel_circ_008161 | ENSSSCG00000016778 | 16 | + | 44259999 | 44265302 | 409 | annot_exons |
| novel_circ_008162 | ENSSSCG00000035730 | 1 | + | 2.08E+08 | 2.08E+08 | 251 | annot_exons |
| novel_circ_008163 | ENSSSCG00000043604 | X | - | 59556112 | 59571884 | 560 | annot_exons |
| novel_circ_008164 | ENSSSCG00000035136 | 10 | + | 16345992 | 16348572 | 237 | annot_exons |
| novel_circ_008165 | ENSSSCG00000014416 | 2 | - | 1.48E+08 | 1.48E+08 | 212 | antisense |
| novel_circ_008166 | ENSSSCG00000032804 | 5 | - | 59999638 | 60007176 | 7537 | exon_intron |
| novel_circ_008167 | ENSSSCG00000015008 | 9 | - | 36769325 | 36777264 | 399 | exon_intron |
| novel_circ_008168 | ENSSSCG00000008143 | 3 | + | 48894234 | 48896705 | 1172 | exon_intron |
| novel_circ_008169 | ENSSSCG00000000753 | 5 | + | 68106936 | 68113748 | 234 | annot_exons |
| novel_circ_008170 | ENSSSCG00000032266 | 18 | + | 9400686 | 9409053 | 363 | annot_exons |
| novel_circ_008171 | ENSSSCG00000008332 | 3 | + | 72304432 | 72309811 | 318 | annot_exons |
| novel_circ_008172 | NA | 15 | - | 84451840 | 84458469 | 6630 | intergenic |
| novel_circ_008173 | ENSSSCG00000034709 | AEMK02000452.1 | + | 1908088 | 1908734 | 300 | annot_exons |
| novel_circ_008174 | ENSSSCG00000026724 | 5 | + | 57844541 | 57854757 | 452 | annot_exons |
| novel_circ_008175 | ENSSSCG00000022177 | 15 | + | 1.33E+08 | 1.33E+08 | 234 | one_exon |
| novel_circ_008176 | ENSSSCG00000016109 | 15 | - | 1.05E+08 | 1.05E+08 | 1124 | annot_exons |
| novel_circ_008177 | ENSSSCG00000016542 | 18 | - | 15063255 | 15065687 | 190 | annot_exons |
| novel_circ_008178 | ENSSSCG00000015689 | 15 | + | 15839856 | 15864971 | 347 | annot_exons |
| novel_circ_008179 | ENSSSCG00000024623 | 13 | + | 1.81E+08 | 1.81E+08 | 374 | annot_exons |
| novel_circ_008180 | ENSSSCG00000046408 | X | - | 29578514 | 29580636 | 2123 | intronic |
| novel_circ_008181 | ENSSSCG00000005215 | 1 | - | 2.17E+08 | 2.17E+08 | 676 | annot_exons |
| novel_circ_008182 | ENSSSCG00000025478 | 6 | + | 1.07E+08 | 1.07E+08 | 776 | annot_exons |
| novel_circ_008183 | ENSSSCG00000022331 | X | + | 1.14E+08 | 1.14E+08 | 1234 | antisense |
| novel_circ_008184 | NA | 15 | + | 84250293 | 84252016 | 1724 | intergenic |
| novel_circ_008185 | ENSSSCG00000029633 | 6 | + | 1.57E+08 | 1.57E+08 | 223 | annot_exons |
| novel_circ_008186 | ENSSSCG00000032275 | 16 | - | 29112120 | 29112433 | 314 | antisense |
| novel_circ_008187 | ENSSSCG00000006911 | 4 | + | 1.25E+08 | 1.25E+08 | 180 | one_exon |
| novel_circ_008188 | ENSSSCG00000011103 | 10 | - | 57137536 | 57168112 | 469 | annot_exons |
| novel_circ_008189 | ENSSSCG00000035148 | 14 | + | 22386974 | 22387454 | 214 | annot_exons |
| novel_circ_008190 | ENSSSCG00000007805 | 3 | + | 18554778 | 18559960 | 547 | annot_exons |
| novel_circ_008191 | ENSSSCG00000000006 | 5 | - | 3310248 | 3313241 | 506 | annot_exons |
| novel_circ_008192 | ENSSSCG00000011629 | 13 | - | 73800008 | 73803519 | 370 | annot_exons |
| novel_circ_008193 | ENSSSCG00000034156 | 11 | - | 22004762 | 22059413 | 4337 | annot_exons |
| novel_circ_008194 | ENSSSCG00000008515 | 3 | + | 1.08E+08 | 1.08E+08 | 255 | annot_exons |
| novel_circ_008195 | ENSSSCG00000016125 | 15 | - | 1.09E+08 | 1.09E+08 | 469 | annot_exons |
| novel_circ_008196 | ENSSSCG00000009040 | 8 | - | 82957079 | 82957656 | 578 | one_exon |
| novel_circ_008197 | ENSSSCG00000005243 | 1 | + | 2.22E+08 | 2.22E+08 | 782 | annot_exons |
| novel_circ_008198 | ENSSSCG00000048856 | 2 | - | 6754918 | 6755333 | 416 | one_exon |
| novel_circ_008199 | ENSSSCG00000010485 | 14 | + | 1.06E+08 | 1.06E+08 | 481 | annot_exons |
| novel_circ_008200 | ENSSSCG00000015499 | 9 | + | 1.16E+08 | 1.16E+08 | 269 | annot_exons |
| novel_circ_008201 | ENSSSCG00000032134 | 7 | + | 1.18E+08 | 1.18E+08 | 259 | one_exon |
| novel_circ_008202 | ENSSSCG00000006136 | 4 | + | 50356925 | 50362297 | 215 | annot_exons |
| novel_circ_008203 | ENSSSCG00000018045 | 12 | + | 59854566 | 59876473 | 2436 | annot_exons |
| novel_circ_008204 | ENSSSCG00000002020 | 7 | - | 75623444 | 75624649 | 569 | annot_exons |
| novel_circ_008205 | ENSSSCG00000002703 | 6 | - | 12012556 | 12014334 | 1779 | exon_intron |
| novel_circ_008206 | ENSSSCG00000015976 | 15 | - | 81730214 | 81742512 | 1019 | annot_exons |
| novel_circ_008207 | ENSSSCG00000006995 | 17 | - | 5719251 | 5733109 | 660 | annot_exons |
| novel_circ_008208 | ENSSSCG00000006864 | 4 | - | 1.18E+08 | 1.18E+08 | 303 | annot_exons |
| novel_circ_008209 | ENSSSCG00000010196 | 14 | + | 60941315 | 60963130 | 402 | annot_exons |
| novel_circ_008210 | ENSSSCG00000006137 | 4 | - | 50461881 | 50462793 | 264 | annot_exons |
| novel_circ_008211 | ENSSSCG00000038561 | 15 | - | 84402264 | 84408453 | 2876 | annot_exons |
| novel_circ_008212 | ENSSSCG00000041715 | 3 | - | 40980515 | 40983377 | 2010 | exon_intron |
| novel_circ_008213 | ENSSSCG00000033528 | 12 | + | 14178158 | 14185034 | 865 | annot_exons |
| novel_circ_008214 | ENSSSCG00000017125 | 12 | - | 573171 | 585916 | 1364 | annot_exons |
| novel_circ_008215 | ENSSSCG00000026569 | 3 | - | 31988381 | 31994331 | 522 | annot_exons |
| novel_circ_008216 | ENSSSCG00000008412 | 3 | + | 87311544 | 87312575 | 196 | annot_exons |
| novel_circ_008217 | ENSSSCG00000036705 | 12 | + | 47400191 | 47401710 | 1520 | exon_intron |
| novel_circ_008218 | ENSSSCG00000017065 | 16 | - | 68243726 | 68250610 | 280 | annot_exons |
| novel_circ_008219 | ENSSSCG00000004366 | 1 | - | 71339696 | 71350243 | 607 | annot_exons |
| novel_circ_008220 | ENSSSCG00000038465 | 6 | - | 98230656 | 98233063 | 257 | annot_exons |
| novel_circ_008221 | ENSSSCG00000004415 | 1 | + | 75285237 | 75294266 | 417 | annot_exons |
| novel_circ_008222 | ENSSSCG00000003070 | 6 | + | 50746659 | 50754132 | 506 | annot_exons |
| novel_circ_008223 | ENSSSCG00000020878 | 3 | + | 1.02E+08 | 1.02E+08 | 690 | annot_exons |
| novel_circ_008224 | ENSSSCG00000016549 | 18 | - | 17554494 | 17565509 | 565 | annot_exons |
| novel_circ_008225 | ENSSSCG00000027503 | 5 | - | 39663677 | 39666548 | 224 | annot_exons |
| novel_circ_008226 | ENSSSCG00000006487 | 4 | + | 93738637 | 93750841 | 948 | annot_exons |
| novel_circ_008227 | ENSSSCG00000008098 | 3 | - | 43957662 | 43959180 | 416 | annot_exons |
| novel_circ_008228 | ENSSSCG00000036001 | 17 | - | 11785783 | 11798831 | 370 | annot_exons |
| novel_circ_008229 | ENSSSCG00000020785 | 15 | + | 1.21E+08 | 1.21E+08 | 162 | one_exon |
| novel_circ_008230 | ENSSSCG00000005205 | 1 | + | 2.16E+08 | 2.16E+08 | 438 | intronic |
| novel_circ_008231 | ENSSSCG00000010467 | 14 | + | 1.04E+08 | 1.04E+08 | 448 | annot_exons |
| novel_circ_008232 | ENSSSCG00000031271 | 15 | + | 1.05E+08 | 1.05E+08 | 645 | annot_exons |
| novel_circ_008233 | ENSSSCG00000008452 | 3 | + | 96497331 | 96497963 | 272 | annot_exons |
| novel_circ_008234 | ENSSSCG00000015815 | 15 | + | 48094739 | 48096210 | 315 | annot_exons |
| novel_circ_008235 | ENSSSCG00000009172 | 8 | + | 1.2E+08 | 1.2E+08 | 696 | annot_exons |
| novel_circ_008236 | ENSSSCG00000010468 | 14 | - | 1.04E+08 | 1.04E+08 | 377 | annot_exons |
| novel_circ_008237 | ENSSSCG00000034200 | 13 | - | 26086741 | 26109530 | 2457 | exon_intron |
| novel_circ_008238 | ENSSSCG00000009401 | 11 | - | 19250142 | 19256608 | 626 | annot_exons |
| novel_circ_008239 | ENSSSCG00000016090 | 15 | + | 1.04E+08 | 1.04E+08 | 343 | annot_exons |
| novel_circ_008240 | ENSSSCG00000025703 | 1 | - | 77386870 | 77394243 | 7374 | intronic |
| novel_circ_008241 | ENSSSCG00000010651 | 14 | - | 1.25E+08 | 1.25E+08 | 515 | annot_exons |
| novel_circ_008242 | ENSSSCG00000035379 | 8 | - | 67480358 | 67481001 | 644 | intronic |
| novel_circ_008243 | ENSSSCG00000000469 | 5 | + | 29660599 | 29663091 | 610 | annot_exons |
| novel_circ_008244 | ENSSSCG00000029920 | 8 | + | 87799386 | 87801061 | 324 | annot_exons |
| novel_circ_008245 | ENSSSCG00000007280 | 17 | + | 37838591 | 37854328 | 532 | annot_exons |
| novel_circ_008246 | ENSSSCG00000003798 | 6 | - | 1.45E+08 | 1.45E+08 | 364 | antisense |
| novel_circ_008247 | ENSSSCG00000010029 | 14 | + | 48137096 | 48143955 | 592 | annot_exons |
| novel_circ_008248 | ENSSSCG00000029485 | 3 | + | 92761995 | 92762903 | 358 | annot_exons |
| novel_circ_008249 | NA | 15 | - | 84468090 | 84468289 | 200 | intergenic |
| novel_circ_008250 | ENSSSCG00000004146 | 1 | + | 25523036 | 25528664 | 982 | annot_exons |
| novel_circ_008251 | ENSSSCG00000004856 | 1 | - | 1.46E+08 | 1.46E+08 | 376 | annot_exons |
| novel_circ_008252 | ENSSSCG00000006870 | 4 | - | 1.18E+08 | 1.18E+08 | 287 | annot_exons |
| novel_circ_008253 | ENSSSCG00000028902 | 4 | + | 77614097 | 77614563 | 298 | annot_exons |
| novel_circ_008254 | NA | 6 | + | 47401837 | 47402264 | 428 | intergenic |
| novel_circ_008255 | ENSSSCG00000015880 | 15 | + | 66069856 | 66097861 | 687 | annot_exons |
| novel_circ_008256 | ENSSSCG00000036033 | 13 | + | 11341939 | 11342187 | 249 | antisense |
| novel_circ_008257 | NA | 15 | - | 84266644 | 84267089 | 446 | intergenic |
| novel_circ_008258 | ENSSSCG00000001400 | 7 | - | 23661897 | 23664343 | 1101 | annot_exons |
| novel_circ_008259 | ENSSSCG00000016005 | 15 | - | 84871348 | 84871958 | 611 | intronic |
| novel_circ_008260 | ENSSSCG00000004388 | 1 | + | 75059888 | 75066482 | 6595 | intronic |
| novel_circ_008261 | ENSSSCG00000015958 | 15 | + | 78613464 | 78620652 | 655 | annot_exons |
| novel_circ_008262 | ENSSSCG00000014416 | 2 | + | 1.48E+08 | 1.48E+08 | 709 | annot_exons |
| novel_circ_008263 | ENSSSCG00000029571 | 5 | - | 23098507 | 23104780 | 958 | annot_exons |
| novel_circ_008264 | ENSSSCG00000010680 | 14 | + | 1.29E+08 | 1.29E+08 | 203 | annot_exons |
| novel_circ_008265 | ENSSSCG00000008136 | 3 | - | 47572235 | 47574992 | 295 | annot_exons |
| novel_circ_008266 | ENSSSCG00000004123 | 1 | - | 20821002 | 20826456 | 480 | annot_exons |
| novel_circ_008267 | NA | 15 | + | 84488988 | 84493705 | 4718 | intergenic |
| novel_circ_008268 | ENSSSCG00000016842 | 16 | + | 22318006 | 22322380 | 699 | annot_exons |
| novel_circ_008269 | ENSSSCG00000029633 | 6 | + | 1.57E+08 | 1.57E+08 | 950 | exon_intron |
| novel_circ_008270 | ENSSSCG00000036480 | 14 | - | 70408467 | 70409532 | 1066 | intronic |
| novel_circ_008271 | ENSSSCG00000008600 | 3 | + | 1.18E+08 | 1.18E+08 | 248 | annot_exons |
| novel_circ_008272 | ENSSSCG00000010190 | 14 | + | 60412967 | 60415673 | 1640 | annot_exons |
| novel_circ_008273 | ENSSSCG00000005389 | 1 | - | 2.42E+08 | 2.42E+08 | 957 | annot_exons |
| novel_circ_008274 | ENSSSCG00000000893 | 5 | - | 87553022 | 87554273 | 252 | annot_exons |
| novel_circ_008275 | ENSSSCG00000021845 | 3 | + | 18512814 | 18513266 | 284 | annot_exons |
| novel_circ_008276 | ENSSSCG00000003771 | 6 | + | 1.35E+08 | 1.35E+08 | 146 | one_exon |
| novel_circ_008277 | NA | 8 | - | 99199685 | 99228789 | 29105 | intergenic |
| novel_circ_008278 | ENSSSCG00000040912 | 7 | + | 65168585 | 65173711 | 596 | annot_exons |
| novel_circ_008279 | ENSSSCG00000004729 | 1 | - | 1.29E+08 | 1.29E+08 | 1886 | annot_exons |
| novel_circ_008280 | ENSSSCG00000004802 | 1 | + | 1.36E+08 | 1.36E+08 | 673 | annot_exons |
| novel_circ_008281 | ENSSSCG00000036669 | 2 | - | 9215617 | 9215983 | 367 | antisense |
| novel_circ_008282 | ENSSSCG00000034348 | 11 | + | 11156485 | 11169711 | 2677 | annot_exons |
| novel_circ_008283 | ENSSSCG00000016567 | 18 | - | 19214132 | 19215271 | 310 | annot_exons |
| novel_circ_008284 | ENSSSCG00000021791 | 13 | + | 1.58E+08 | 1.58E+08 | 393 | annot_exons |
| novel_circ_008285 | ENSSSCG00000034357 | 13 | + | 32059508 | 32059882 | 375 | one_exon |
| novel_circ_008286 | ENSSSCG00000017198 | 12 | + | 5471981 | 5473179 | 354 | annot_exons |
| novel_circ_008287 | ENSSSCG00000006137 | 4 | - | 50408310 | 50418702 | 611 | annot_exons |
| novel_circ_008288 | ENSSSCG00000016397 | 15 | + | 590180 | 590377 | 198 | one_exon |
| novel_circ_008289 | ENSSSCG00000005356 | 1 | + | 2.39E+08 | 2.39E+08 | 7619 | exon_intron |
| novel_circ_008290 | NA | 8 | - | 29996203 | 30000238 | 4036 | intergenic |
| novel_circ_008291 | ENSSSCG00000011340 | 13 | - | 30516967 | 30547685 | 682 | annot_exons |
| novel_circ_008292 | ENSSSCG00000010900 | 10 | - | 20455784 | 20481282 | 330 | annot_exons |
| novel_circ_008293 | NA | 4 | - | 90275728 | 90276418 | 691 | intergenic |
| novel_circ_008294 | ENSSSCG00000008009 | 3 | + | 40901263 | 40928175 | 914 | annot_exons |
| novel_circ_008295 | ENSSSCG00000016049 | 15 | - | 94907084 | 94922554 | 307 | annot_exons |
| novel_circ_008296 | ENSSSCG00000001921 | 7 | - | 60092932 | 60095037 | 247 | annot_exons |
| novel_circ_008297 | ENSSSCG00000012163 | X | - | 16491482 | 16491878 | 214 | annot_exons |
| novel_circ_008298 | ENSSSCG00000025777 | 1 | - | 14387851 | 14416451 | 28601 | exon_intron |
| novel_circ_008299 | ENSSSCG00000033528 | 12 | + | 14216841 | 14218889 | 670 | annot_exons |
| novel_circ_008300 | ENSSSCG00000020663 | 18 | + | 5153662 | 5168717 | 457 | annot_exons |
| novel_circ_008301 | ENSSSCG00000003749 | 6 | + | 1.26E+08 | 1.26E+08 | 619 | annot_exons |
| novel_circ_008302 | ENSSSCG00000015145 | 9 | - | 50583590 | 50594565 | 7090 | exon_intron |
| novel_circ_008303 | ENSSSCG00000010947 | 10 | + | 27641998 | 27648976 | 234 | annot_exons |
| novel_circ_008304 | ENSSSCG00000008515 | 3 | + | 1.08E+08 | 1.08E+08 | 368 | annot_exons |
| novel_circ_008305 | ENSSSCG00000006872 | 4 | - | 1.18E+08 | 1.18E+08 | 897 | annot_exons |
| novel_circ_008306 | ENSSSCG00000021393 | 9 | - | 1.26E+08 | 1.26E+08 | 682 | annot_exons |
| novel_circ_008307 | ENSSSCG00000021784 | 8 | + | 1.16E+08 | 1.16E+08 | 3688 | annot_exons |
| novel_circ_008308 | ENSSSCG00000030396 | 8 | + | 87526447 | 87526911 | 465 | one_exon |
| novel_circ_008309 | ENSSSCG00000012322 | X | - | 45971065 | 45971247 | 183 | one_exon |
| novel_circ_008310 | ENSSSCG00000011765 | 13 | + | 1.18E+08 | 1.18E+08 | 569 | intronic |
| novel_circ_008311 | ENSSSCG00000016846 | 16 | + | 22868833 | 22875446 | 461 | annot_exons |
| novel_circ_008312 | ENSSSCG00000009029 | 8 | - | 80872584 | 80876761 | 237 | annot_exons |
| novel_circ_008313 | ENSSSCG00000037015 | 9 | - | 27208689 | 27210541 | 1853 | one_exon |
| novel_circ_008314 | ENSSSCG00000008143 | 3 | + | 48887031 | 48909045 | 6386 | exon_intron |
| novel_circ_008315 | ENSSSCG00000026596 | 12 | - | 39036725 | 39039068 | 349 | annot_exons |
| novel_circ_008316 | ENSSSCG00000001963 | 7 | - | 65831881 | 65832793 | 913 | antisense |
| novel_circ_008317 | ENSSSCG00000004524 | 1 | + | 1.01E+08 | 1.01E+08 | 1576 | annot_exons |
| novel_circ_008318 | ENSSSCG00000006388 | 4 | + | 90238894 | 90244818 | 206 | annot_exons |
| novel_circ_008319 | ENSSSCG00000017693 | 12 | + | 38501975 | 38506507 | 572 | annot_exons |
| novel_circ_008320 | ENSSSCG00000014364 | 2 | + | 1.42E+08 | 1.42E+08 | 357 | annot_exons |
| novel_circ_008321 | ENSSSCG00000007284 | 17 | - | 38122670 | 38128908 | 255 | annot_exons |
| novel_circ_008322 | ENSSSCG00000015576 | 9 | - | 1.28E+08 | 1.28E+08 | 600 | annot_exons |
| novel_circ_008323 | ENSSSCG00000031147 | 13 | - | 1.32E+08 | 1.32E+08 | 243 | annot_exons |
| novel_circ_008324 | ENSSSCG00000009994 | 14 | + | 46985143 | 46993251 | 264 | annot_exons |
| novel_circ_008325 | ENSSSCG00000005459 | 1 | - | 2.52E+08 | 2.52E+08 | 342 | annot_exons |
| novel_circ_008326 | ENSSSCG00000011788 | 13 | + | 1.23E+08 | 1.23E+08 | 444 | annot_exons |
| novel_circ_008327 | ENSSSCG00000004653 | 1 | + | 1.23E+08 | 1.23E+08 | 504 | annot_exons |
| novel_circ_008328 | NA | 18 | - | 2651505 | 2661452 | 9948 | intergenic |
| novel_circ_008329 | ENSSSCG00000029745 | 8 | + | 1472469 | 1491356 | 1168 | annot_exons |
| novel_circ_008330 | ENSSSCG00000022004 | 8 | + | 1614969 | 1615143 | 175 | one_exon |
| novel_circ_008331 | ENSSSCG00000031808 | 11 | + | 68450305 | 68454998 | 311 | annot_exons |
| novel_circ_008332 | ENSSSCG00000037670 | X | + | 90177247 | 90253988 | 284 | annot_exons |
| novel_circ_008333 | ENSSSCG00000039978 | 6 | - | 61938916 | 61939181 | 266 | antisense |
| novel_circ_008334 | ENSSSCG00000032622 | 14 | + | 6745390 | 6754347 | 435 | annot_exons |
| novel_circ_008335 | ENSSSCG00000003660 | 6 | - | 95394880 | 95395073 | 194 | one_exon |
| novel_circ_008336 | ENSSSCG00000008513 | 3 | - | 1.07E+08 | 1.07E+08 | 709 | annot_exons |
| novel_circ_008337 | ENSSSCG00000040434 | 3 | + | 56992246 | 56995376 | 875 | annot_exons |
| novel_circ_008338 | ENSSSCG00000027224 | 3 | + | 72378974 | 72379228 | 255 | antisense |
| novel_circ_008339 | ENSSSCG00000024481 | 4 | - | 1.01E+08 | 1.01E+08 | 588 | annot_exons |
| novel_circ_008340 | ENSSSCG00000012885 | 2 | - | 4575749 | 4578640 | 507 | annot_exons |
| novel_circ_008341 | ENSSSCG00000009096 | 8 | - | 1.02E+08 | 1.02E+08 | 577 | annot_exons |
| novel_circ_008342 | ENSSSCG00000030343 | 16 | - | 34798441 | 34810715 | 534 | annot_exons |
| novel_circ_008343 | ENSSSCG00000016543 | 18 | - | 15347146 | 15392839 | 45694 | exon_intron |
| novel_circ_008344 | ENSSSCG00000016954 | 16 | + | 44513270 | 44525240 | 1997 | annot_exons |
| novel_circ_008345 | ENSSSCG00000004500 | 1 | + | 95846715 | 95847499 | 785 | one_exon |
| novel_circ_008346 | ENSSSCG00000031077 | 13 | - | 1E+08 | 1E+08 | 355 | annot_exons |
| novel_circ_008347 | ENSSSCG00000027675 | 13 | - | 52503349 | 52590151 | 926 | annot_exons |
| novel_circ_008348 | ENSSSCG00000004328 | 1 | - | 57778013 | 57778882 | 316 | annot_exons |
| novel_circ_008349 | ENSSSCG00000004857 | 1 | - | 1.46E+08 | 1.46E+08 | 3483 | annot_exons |
| novel_circ_008350 | ENSSSCG00000015944 | 15 | - | 77234646 | 77283482 | 267 | annot_exons |
| novel_circ_008351 | ENSSSCG00000009359 | 11 | - | 12842165 | 12854933 | 986 | annot_exons |
| novel_circ_008352 | ENSSSCG00000000801 | 5 | + | 74926273 | 74927178 | 906 | exon_intron |
| novel_circ_008353 | ENSSSCG00000026761 | 13 | - | 1.92E+08 | 1.92E+08 | 450 | annot_exons |
| novel_circ_008354 | ENSSSCG00000017260 | 12 | + | 11747975 | 11748619 | 220 | annot_exons |
| novel_circ_008355 | ENSSSCG00000001485 | 7 | - | 26689737 | 26731237 | 31867 | exon_intron |
| novel_circ_008356 | ENSSSCG00000003461 | 6 | + | 74824455 | 74838414 | 3328 | annot_exons |
| novel_circ_008357 | ENSSSCG00000038870 | AEMK02000510.1 | - | 631357 | 631791 | 435 | one_exon |
| novel_circ_008358 | ENSSSCG00000009319 | 11 | + | 5587551 | 5608636 | 1112 | annot_exons |
| novel_circ_008359 | ENSSSCG00000003761 | 6 | - | 1.32E+08 | 1.32E+08 | 1720 | annot_exons |
| novel_circ_008360 | ENSSSCG00000003512 | 6 | - | 79057470 | 79063127 | 477 | annot_exons |
| novel_circ_008361 | ENSSSCG00000007839 | 3 | - | 23956138 | 23956449 | 310 | exon_intron |
| novel_circ_008362 | ENSSSCG00000015965 | 15 | - | 80444887 | 80448241 | 722 | annot_exons |
| novel_circ_008363 | ENSSSCG00000002259 | 7 | + | 88163866 | 88172966 | 351 | annot_exons |
| novel_circ_008364 | ENSSSCG00000010292 | 14 | - | 75890549 | 75897301 | 495 | annot_exons |
| novel_circ_008365 | ENSSSCG00000006307 | 4 | - | 83324170 | 83325042 | 873 | one_exon |
| novel_circ_008366 | ENSSSCG00000005110 | 1 | - | 1.94E+08 | 1.94E+08 | 663 | annot_exons |
| novel_circ_008367 | ENSSSCG00000003035 | 6 | + | 49693927 | 49694293 | 195 | annot_exons |
| novel_circ_008368 | ENSSSCG00000023865 | 14 | - | 1.33E+08 | 1.33E+08 | 1569 | exon_intron |
| novel_circ_008369 | ENSSSCG00000011238 | 13 | + | 20977292 | 20988377 | 4802 | annot_exons |
| novel_circ_008370 | ENSSSCG00000002298 | 7 | - | 91555150 | 91557998 | 943 | annot_exons |
| novel_circ_008371 | ENSSSCG00000000625 | 5 | + | 60302547 | 60309182 | 577 | annot_exons |
| novel_circ_008372 | ENSSSCG00000002414 | 7 | - | 1.04E+08 | 1.04E+08 | 529 | annot_exons |
| novel_circ_008373 | ENSSSCG00000017268 | 12 | + | 12959478 | 12961036 | 1559 | intronic |
| novel_circ_008374 | ENSSSCG00000025194 | 16 | + | 40251241 | 40253338 | 300 | annot_exons |
| novel_circ_008375 | ENSSSCG00000039332 | 9 | - | 1.2E+08 | 1.2E+08 | 200 | annot_exons |
| novel_circ_008376 | ENSSSCG00000006860 | 4 | - | 1.17E+08 | 1.17E+08 | 702 | annot_exons |
| novel_circ_008377 | ENSSSCG00000011183 | 13 | - | 1466116 | 1480617 | 821 | annot_exons |
| novel_circ_008378 | ENSSSCG00000005466 | 1 | - | 2.53E+08 | 2.53E+08 | 568 | annot_exons |
| novel_circ_008379 | ENSSSCG00000004570 | 1 | - | 1.09E+08 | 1.09E+08 | 608 | annot_exons |
| novel_circ_008380 | ENSSSCG00000011582 | 13 | + | 68773839 | 68776092 | 967 | annot_exons |
| novel_circ_008381 | ENSSSCG00000018045 | 12 | + | 59824456 | 59834568 | 635 | annot_exons |
| novel_circ_008382 | ENSSSCG00000016916 | 16 | - | 35113222 | 35116352 | 573 | annot_exons |
| novel_circ_008383 | ENSSSCG00000030362 | 13 | - | 1.36E+08 | 1.36E+08 | 616 | annot_exons |
| novel_circ_008384 | ENSSSCG00000010826 | 10 | + | 10169065 | 10187763 | 3186 | annot_exons |
| novel_circ_008385 | ENSSSCG00000005227 | 1 | + | 2.19E+08 | 2.19E+08 | 735 | annot_exons |
| novel_circ_008386 | ENSSSCG00000006110 | 4 | + | 42952659 | 42954553 | 1895 | one_exon |
| novel_circ_008387 | ENSSSCG00000033690 | 4 | + | 16328959 | 16335370 | 352 | annot_exons |
| novel_circ_008388 | NA | 9 | + | 1.22E+08 | 1.22E+08 | 8750 | intergenic |
| novel_circ_008389 | ENSSSCG00000015940 | 15 | + | 76337793 | 76343935 | 359 | annot_exons |
| novel_circ_008390 | ENSSSCG00000029578 | 6 | + | 28743200 | 28748110 | 314 | annot_exons |
| novel_circ_008391 | ENSSSCG00000010029 | 14 | + | 48129173 | 48143955 | 838 | annot_exons |
| novel_circ_008392 | ENSSSCG00000001709 | 7 | - | 39887235 | 39962564 | 403 | annot_exons |
| novel_circ_008393 | ENSSSCG00000008991 | 8 | + | 73899672 | 73908411 | 841 | annot_exons |
| novel_circ_008394 | ENSSSCG00000038222 | 14 | - | 1.29E+08 | 1.29E+08 | 230 | annot_exons |
| novel_circ_008395 | ENSSSCG00000028225 | 7 | - | 1.16E+08 | 1.16E+08 | 590 | annot_exons |
| novel_circ_008396 | ENSSSCG00000017785 | 12 | + | 45481605 | 45482027 | 423 | antisense |
| novel_circ_008397 | ENSSSCG00000001878 | 7 | + | 58140643 | 58146958 | 423 | annot_exons |
| novel_circ_008398 | ENSSSCG00000021200 | X | - | 58444426 | 58450493 | 664 | annot_exons |
| novel_circ_008399 | ENSSSCG00000028512 | 2 | + | 69840324 | 69841770 | 1065 | annot_exons |
| novel_circ_008400 | ENSSSCG00000027166 | 3 | - | 42440890 | 42441272 | 383 | intronic |
| novel_circ_008401 | ENSSSCG00000008512 | 3 | - | 1.07E+08 | 1.07E+08 | 27462 | exon_intron |
| novel_circ_008402 | ENSSSCG00000004358 | 1 | - | 67576479 | 67578266 | 476 | annot_exons |
| novel_circ_008403 | ENSSSCG00000004542 | 1 | - | 1.07E+08 | 1.07E+08 | 514 | annot_exons |
| novel_circ_008404 | ENSSSCG00000014149 | 2 | + | 96126827 | 96133406 | 6580 | antisense |
| novel_circ_008405 | ENSSSCG00000026724 | 5 | + | 57883430 | 57887138 | 251 | annot_exons |
| novel_circ_008406 | ENSSSCG00000008697 | 8 | + | 1876953 | 1882271 | 446 | annot_exons |
| novel_circ_008407 | ENSSSCG00000009864 | 14 | + | 36101382 | 36101619 | 238 | one_exon |
| novel_circ_008408 | ENSSSCG00000013314 | 2 | - | 27975024 | 27976384 | 446 | annot_exons |
| novel_circ_008409 | ENSSSCG00000007360 | 17 | - | 44164326 | 44180350 | 819 | annot_exons |
| novel_circ_008410 | ENSSSCG00000015874 | 15 | - | 64759481 | 64777189 | 605 | annot_exons |
| novel_circ_008411 | ENSSSCG00000005057 | 1 | + | 1.85E+08 | 1.85E+08 | 281 | one_exon |
| novel_circ_008412 | ENSSSCG00000030354 | 15 | + | 25157152 | 25157483 | 161 | annot_exons |
| novel_circ_008413 | ENSSSCG00000037670 | X | + | 90155567 | 90207986 | 21885 | exon_intron |
| novel_circ_008414 | ENSSSCG00000032220 | 13 | + | 18670725 | 18680602 | 583 | annot_exons |
| novel_circ_008415 | ENSSSCG00000000108 | 5 | + | 9657411 | 9659528 | 414 | annot_exons |
| novel_circ_008416 | ENSSSCG00000008412 | 3 | + | 87328900 | 87330087 | 285 | annot_exons |
| novel_circ_008417 | ENSSSCG00000010151 | 14 | + | 55622992 | 55623498 | 238 | annot_exons |
| novel_circ_008418 | ENSSSCG00000007247 | 17 | + | 35991572 | 35994261 | 192 | exon_intron |
| novel_circ_008419 | ENSSSCG00000004275 | 1 | + | 50655824 | 50663384 | 284 | annot_exons |
| novel_circ_008420 | ENSSSCG00000006840 | 4 | + | 1.11E+08 | 1.11E+08 | 382 | annot_exons |
| novel_circ_008421 | ENSSSCG00000010687 | 14 | + | 1.3E+08 | 1.3E+08 | 433 | annot_exons |
| novel_circ_008422 | ENSSSCG00000008749 | 8 | - | 14858994 | 14859811 | 818 | antisense |
| novel_circ_008423 | ENSSSCG00000038760 | 12 | - | 26981583 | 26989423 | 817 | annot_exons |
| novel_circ_008424 | ENSSSCG00000038913 | 14 | - | 71135574 | 71145521 | 501 | annot_exons |
| novel_circ_008425 | ENSSSCG00000008068 | 3 | - | 41951780 | 41962459 | 802 | annot_exons |
| novel_circ_008426 | ENSSSCG00000009464 | 11 | - | 47603604 | 47609973 | 4652 | exon_intron |
| novel_circ_008427 | ENSSSCG00000010811 | 10 | + | 5770011 | 5777849 | 563 | annot_exons |
| novel_circ_008428 | ENSSSCG00000035824 | 13 | - | 34813672 | 34814973 | 251 | annot_exons |
| novel_circ_008429 | ENSSSCG00000024201 | 13 | - | 38532801 | 38545830 | 829 | annot_exons |
| novel_circ_008430 | ENSSSCG00000008488 | 3 | - | 1.02E+08 | 1.02E+08 | 727 | annot_exons |
| novel_circ_008431 | ENSSSCG00000008961 | 8 | + | 70193530 | 70208061 | 1289 | annot_exons |
| novel_circ_008432 | ENSSSCG00000009844 | 14 | + | 33626310 | 33627119 | 810 | antisense |
| novel_circ_008433 | ENSSSCG00000037719 | 3 | - | 39466946 | 39478565 | 691 | annot_exons |
| novel_circ_008434 | ENSSSCG00000009852 | 14 | + | 34588351 | 34592636 | 564 | annot_exons |
| novel_circ_008435 | ENSSSCG00000006766 | 4 | + | 1.07E+08 | 1.07E+08 | 3781 | annot_exons |
| novel_circ_008436 | ENSSSCG00000006487 | 4 | + | 93736189 | 93738733 | 273 | annot_exons |
| novel_circ_008437 | ENSSSCG00000014211 | 2 | + | 1.18E+08 | 1.18E+08 | 302 | intronic |
| novel_circ_008438 | ENSSSCG00000010928 | 10 | - | 24760509 | 24779098 | 1126 | annot_exons |
| novel_circ_008439 | ENSSSCG00000013865 | 2 | - | 60894107 | 60906053 | 494 | annot_exons |
| novel_circ_008440 | ENSSSCG00000036083 | 14 | + | 1.03E+08 | 1.03E+08 | 407 | annot_exons |
| novel_circ_008441 | ENSSSCG00000003777 | 6 | + | 1.38E+08 | 1.38E+08 | 245 | annot_exons |
| novel_circ_008442 | ENSSSCG00000013326 | 2 | + | 29733143 | 29739086 | 514 | annot_exons |
| novel_circ_008443 | ENSSSCG00000030480 | 13 | + | 2.01E+08 | 2.01E+08 | 6390 | exon_intron |
| novel_circ_008444 | ENSSSCG00000022032 | 4 | - | 1.29E+08 | 1.29E+08 | 464 | annot_exons |
| novel_circ_008445 | ENSSSCG00000011752 | 13 | + | 1.11E+08 | 1.11E+08 | 612 | annot_exons |
| novel_circ_008446 | ENSSSCG00000024754 | 18 | - | 40140465 | 40200881 | 1099 | annot_exons |
| novel_circ_008447 | ENSSSCG00000003828 | 6 | - | 1.53E+08 | 1.53E+08 | 264 | annot_exons |
| novel_circ_008448 | ENSSSCG00000006903 | 4 | - | 1.25E+08 | 1.25E+08 | 1206 | annot_exons |
| novel_circ_008449 | ENSSSCG00000023409 | 3 | + | 72250326 | 72262713 | 722 | annot_exons |
| novel_circ_008450 | ENSSSCG00000016464 | 18 | - | 6914973 | 6915565 | 348 | annot_exons |
| novel_circ_008451 | ENSSSCG00000004725 | 1 | + | 1.29E+08 | 1.29E+08 | 376 | annot_exons |
| novel_circ_008452 | ENSSSCG00000001806 | 7 | + | 52169611 | 52173199 | 376 | annot_exons |
| novel_circ_008453 | ENSSSCG00000036948 | 10 | + | 23641900 | 23642921 | 264 | exon_intron |
| novel_circ_008454 | ENSSSCG00000037251 | 9 | + | 12285388 | 12285926 | 539 | exon_intron |
| novel_circ_008455 | ENSSSCG00000009302 | 11 | - | 4639811 | 4652306 | 295 | annot_exons |
| novel_circ_008456 | ENSSSCG00000014324 | 2 | + | 1.4E+08 | 1.4E+08 | 847 | annot_exons |
| novel_circ_008457 | ENSSSCG00000029196 | 5 | + | 16526820 | 16531299 | 500 | annot_exons |
| novel_circ_008458 | ENSSSCG00000045254 | 12 | + | 58237298 | 58243430 | 6133 | antisense |
| novel_circ_008459 | ENSSSCG00000011794 | 13 | + | 1.23E+08 | 1.23E+08 | 201 | annot_exons |
| novel_circ_008460 | NA | X | + | 49742406 | 49742743 | 338 | intergenic |
| novel_circ_008461 | ENSSSCG00000015402 | 9 | + | 97835162 | 97883761 | 31908 | exon_intron |
| novel_circ_008462 | ENSSSCG00000035824 | 13 | - | 34799994 | 34814970 | 540 | annot_exons |
| novel_circ_008463 | ENSSSCG00000003772 | 6 | + | 1.36E+08 | 1.36E+08 | 961 | annot_exons |
| novel_circ_008464 | ENSSSCG00000009338 | 11 | + | 8661843 | 8670626 | 600 | annot_exons |
| novel_circ_008465 | ENSSSCG00000015872 | 15 | + | 63626162 | 63661287 | 1035 | annot_exons |
| novel_circ_008466 | ENSSSCG00000016082 | 15 | - | 1.02E+08 | 1.02E+08 | 3384 | annot_exons |
| novel_circ_008467 | ENSSSCG00000013276 | 2 | - | 17225982 | 17227421 | 492 | annot_exons |
| novel_circ_008468 | ENSSSCG00000002656 | 6 | + | 1904952 | 1906179 | 260 | annot_exons |
| novel_circ_008469 | ENSSSCG00000010470 | 14 | - | 1.04E+08 | 1.04E+08 | 185 | annot_exons |
| novel_circ_008470 | ENSSSCG00000038374 | 4 | - | 98809463 | 98814550 | 325 | annot_exons |
| novel_circ_008471 | ENSSSCG00000017272 | 12 | - | 13541404 | 13552091 | 656 | annot_exons |
| novel_circ_008472 | ENSSSCG00000000866 | 5 | - | 82594554 | 82597385 | 437 | annot_exons |
| novel_circ_008473 | ENSSSCG00000017015 | 16 | - | 55401528 | 55406130 | 642 | annot_exons |
| novel_circ_008474 | ENSSSCG00000008414 | 3 | - | 87369571 | 87371772 | 347 | annot_exons |
| novel_circ_008475 | ENSSSCG00000010401 | 14 | + | 90616436 | 90618550 | 299 | annot_exons |
| novel_circ_008476 | ENSSSCG00000032984 | 10 | + | 48206021 | 48209078 | 426 | annot_exons |
| novel_circ_008477 | ENSSSCG00000049158 | 1 | + | 13984334 | 13986949 | 645 | annot_exons |
| novel_circ_008478 | ENSSSCG00000006653 | 4 | + | 98563278 | 98563525 | 248 | one_exon |
| novel_circ_008479 | ENSSSCG00000016816 | 16 | - | 19651518 | 19689722 | 1103 | annot_exons |
| novel_circ_008480 | ENSSSCG00000017672 | 12 | + | 36410960 | 36413830 | 683 | annot_exons |
| novel_circ_008481 | NA | X | + | 59299801 | 59300460 | 660 | intergenic |
| novel_circ_008482 | ENSSSCG00000015795 | 15 | - | 46516539 | 46516841 | 303 | intronic |
| novel_circ_008483 | ENSSSCG00000026004 | 5 | + | 29732023 | 29733036 | 1014 | exon_intron |
| novel_circ_008484 | ENSSSCG00000006346 | 4 | - | 88791783 | 88797757 | 272 | annot_exons |
| novel_circ_008485 | ENSSSCG00000011022 | 10 | + | 41416480 | 41436115 | 1439 | annot_exons |
| novel_circ_008486 | ENSSSCG00000010802 | 10 | + | 734757 | 761696 | 1101 | annot_exons |
| novel_circ_008487 | ENSSSCG00000045919 | 9 | + | 1.22E+08 | 1.22E+08 | 737 | exon_intron |
| novel_circ_008488 | ENSSSCG00000006174 | 4 | + | 61715202 | 61716039 | 838 | one_exon |
| novel_circ_008489 | ENSSSCG00000009134 | 8 | - | 1.12E+08 | 1.12E+08 | 386 | annot_exons |
| novel_circ_008490 | ENSSSCG00000026833 | 9 | - | 35862033 | 35863342 | 156 | annot_exons |
| novel_circ_008491 | ENSSSCG00000006982 | 17 | + | 4926027 | 4926340 | 314 | one_exon |
| novel_circ_008492 | ENSSSCG00000017190 | 12 | + | 5349486 | 5349780 | 295 | intronic |
| novel_circ_008493 | ENSSSCG00000042373 | 16 | + | 23176613 | 23186873 | 10261 | exon_intron |
| novel_circ_008494 | ENSSSCG00000005366 | 1 | + | 2.39E+08 | 2.39E+08 | 397 | annot_exons |
| novel_circ_008495 | ENSSSCG00000024864 | 5 | - | 3717600 | 3729566 | 445 | annot_exons |
| novel_circ_008496 | ENSSSCG00000039996 | 9 | - | 7868126 | 7926560 | 332 | annot_exons |
| novel_circ_008497 | ENSSSCG00000006927 | 4 | - | 1.28E+08 | 1.28E+08 | 774 | annot_exons |
| novel_circ_008498 | ENSSSCG00000016397 | 15 | + | 760123 | 761023 | 279 | annot_exons |
| novel_circ_008499 | ENSSSCG00000015379 | 9 | + | 90618099 | 90618704 | 356 | annot_exons |
| novel_circ_008500 | ENSSSCG00000040944 | 6 | + | 64236415 | 64240458 | 1859 | annot_exons |
| novel_circ_008501 | ENSSSCG00000006201 | 4 | + | 67521653 | 67527275 | 457 | annot_exons |
| novel_circ_008502 | ENSSSCG00000032531 | 3 | + | 26701499 | 26701952 | 293 | annot_exons |
| novel_circ_008503 | ENSSSCG00000005286 | 1 | + | 2.31E+08 | 2.31E+08 | 279 | annot_exons |
| novel_circ_008504 | ENSSSCG00000028979 | 9 | + | 10120704 | 10212507 | 2040 | annot_exons |
| novel_circ_008505 | ENSSSCG00000013392 | 2 | + | 44796916 | 44798820 | 345 | annot_exons |
| novel_circ_008506 | ENSSSCG00000032531 | 3 | + | 26683193 | 26690578 | 831 | annot_exons |
| novel_circ_008507 | ENSSSCG00000025106 | 3 | - | 57813373 | 57818865 | 637 | annot_exons |
| novel_circ_008508 | ENSSSCG00000009916 | 14 | + | 40627730 | 40632773 | 2021 | annot_exons |
| novel_circ_008509 | ENSSSCG00000004729 | 1 | - | 1.29E+08 | 1.29E+08 | 706 | annot_exons |
| novel_circ_008510 | ENSSSCG00000037065 | 3 | + | 32482765 | 32488028 | 374 | annot_exons |
| novel_circ_008511 | ENSSSCG00000007805 | 3 | + | 18563276 | 18563726 | 323 | annot_exons |
| novel_circ_008512 | ENSSSCG00000004589 | 1 | - | 1.13E+08 | 1.13E+08 | 679 | annot_exons |
| novel_circ_008513 | ENSSSCG00000012021 | 13 | - | 1.89E+08 | 1.89E+08 | 168 | annot_exons |
| novel_circ_008514 | ENSSSCG00000012314 | 1 | - | 8998426 | 9003215 | 4790 | antisense |
| novel_circ_008515 | ENSSSCG00000029464 | 5 | - | 67679713 | 67681947 | 345 | annot_exons |
| novel_circ_008516 | ENSSSCG00000028925 | 12 | - | 14564246 | 14569779 | 345 | annot_exons |
| novel_circ_008517 | ENSSSCG00000025187 | 9 | + | 19578839 | 19582636 | 325 | annot_exons |
| novel_circ_008518 | ENSSSCG00000025703 | 1 | - | 77318498 | 77394243 | 59851 | exon_intron |
| novel_circ_008519 | ENSSSCG00000006194 | 4 | + | 65229453 | 65235030 | 5099 | annot_exons |
| novel_circ_008520 | ENSSSCG00000020678 | 6 | - | 1446263 | 1451841 | 416 | annot_exons |
| novel_circ_008521 | ENSSSCG00000027115 | X | + | 57425172 | 57425431 | 178 | annot_exons |
| novel_circ_008522 | ENSSSCG00000003512 | 6 | - | 79043260 | 79063127 | 858 | annot_exons |
| novel_circ_008523 | ENSSSCG00000011261 | 13 | + | 23774623 | 23778566 | 290 | annot_exons |
| novel_circ_008524 | ENSSSCG00000010319 | 14 | + | 77706066 | 77706658 | 593 | one_exon |
| novel_circ_008525 | ENSSSCG00000039710 | 14 | - | 32206161 | 32206297 | 137 | one_exon |
| novel_circ_008526 | ENSSSCG00000004156 | 1 | + | 27046256 | 27051886 | 645 | annot_exons |
| novel_circ_008527 | ENSSSCG00000022676 | X | - | 54121488 | 54185322 | 558 | annot_exons |
| novel_circ_008528 | ENSSSCG00000002872 | 6 | + | 44001516 | 44006707 | 363 | annot_exons |
| novel_circ_008529 | ENSSSCG00000051610 | 18 | + | 17889683 | 17947161 | 326 | annot_exons |
| novel_circ_008530 | ENSSSCG00000038036 | 1 | - | 2.62E+08 | 2.62E+08 | 39545 | exon_intron |
| novel_circ_008531 | ENSSSCG00000026196 | 8 | + | 1.3E+08 | 1.3E+08 | 577 | annot_exons |
| novel_circ_008532 | ENSSSCG00000009042 | 8 | + | 83276933 | 83281507 | 702 | annot_exons |
| novel_circ_008533 | ENSSSCG00000003531 | 6 | + | 80956390 | 80960170 | 412 | annot_exons |
| novel_circ_008534 | ENSSSCG00000015590 | 9 | - | 1.31E+08 | 1.31E+08 | 579 | annot_exons |
| novel_circ_008535 | ENSSSCG00000001715 | 7 | - | 41003826 | 41008498 | 346 | annot_exons |
| novel_circ_008536 | ENSSSCG00000016958 | 16 | + | 46495563 | 46503607 | 582 | annot_exons |
| novel_circ_008537 | ENSSSCG00000010295 | 14 | - | 75972129 | 75975028 | 258 | annot_exons |
| novel_circ_008538 | ENSSSCG00000032198 | 15 | - | 13144278 | 13146702 | 357 | annot_exons |
| novel_circ_008539 | NA | 15 | + | 84260773 | 84260871 | 99 | intergenic |
| novel_circ_008540 | ENSSSCG00000013409 | 2 | + | 49499793 | 49502933 | 356 | annot_exons |
| novel_circ_008541 | ENSSSCG00000027105 | 15 | + | 32034803 | 32038391 | 1168 | annot_exons |
| novel_circ_008542 | ENSSSCG00000016077 | 15 | - | 1.01E+08 | 1.01E+08 | 442 | annot_exons |
| novel_circ_008543 | ENSSSCG00000039854 | 4 | - | 1.22E+08 | 1.22E+08 | 484 | annot_exons |
| novel_circ_008544 | ENSSSCG00000013125 | 2 | + | 11882218 | 11885704 | 460 | annot_exons |
| novel_circ_008545 | ENSSSCG00000029920 | 8 | + | 87799383 | 87828391 | 8528 | exon_intron |
| novel_circ_008546 | ENSSSCG00000014913 | 9 | - | 20102422 | 20104632 | 238 | annot_exons |
| novel_circ_008547 | ENSSSCG00000029633 | 6 | + | 1.57E+08 | 1.57E+08 | 394 | annot_exons |
| novel_circ_008548 | ENSSSCG00000028420 | 8 | + | 1.21E+08 | 1.21E+08 | 274 | annot_exons |
| novel_circ_008549 | ENSSSCG00000033368 | 5 | - | 18873193 | 18874212 | 275 | annot_exons |
| novel_circ_008550 | ENSSSCG00000028117 | 1 | - | 2.61E+08 | 2.61E+08 | 188 | annot_exons |
| novel_circ_008551 | ENSSSCG00000013285 | 2 | - | 18983695 | 18985054 | 256 | annot_exons |
| novel_circ_008552 | ENSSSCG00000009864 | 14 | + | 36321195 | 36323544 | 271 | annot_exons |
| novel_circ_008553 | ENSSSCG00000009714 | 14 | + | 20221955 | 20238937 | 267 | annot_exons |
| novel_circ_008554 | ENSSSCG00000010251 | 14 | + | 72251531 | 72255440 | 396 | annot_exons |
| novel_circ_008555 | ENSSSCG00000005316 | 1 | - | 2.36E+08 | 2.36E+08 | 382 | intronic |
| novel_circ_008556 | ENSSSCG00000002266 | 7 | - | 86055233 | 86056387 | 281 | annot_exons |
| novel_circ_008557 | ENSSSCG00000017401 | 12 | - | 20315162 | 20317766 | 364 | annot_exons |
| novel_circ_008558 | ENSSSCG00000027063 | 18 | + | 38826704 | 38857874 | 543 | annot_exons |
| novel_circ_008559 | ENSSSCG00000006179 | 4 | + | 62245322 | 62247682 | 321 | annot_exons |
| novel_circ_008560 | ENSSSCG00000007477 | 17 | - | 52788058 | 52791943 | 873 | annot_exons |
| novel_circ_008561 | ENSSSCG00000008498 | 3 | + | 1.03E+08 | 1.03E+08 | 253 | annot_exons |
| novel_circ_008562 | ENSSSCG00000045919 | 9 | + | 1.22E+08 | 1.22E+08 | 5752 | exon_intron |
| novel_circ_008563 | NA | 15 | - | 84259904 | 84260281 | 378 | intergenic |
| novel_circ_008564 | ENSSSCG00000005229 | 1 | - | 2.19E+08 | 2.19E+08 | 366 | annot_exons |
| novel_circ_008565 | ENSSSCG00000021166 | 5 | - | 78038560 | 78040700 | 386 | annot_exons |
| novel_circ_008566 | ENSSSCG00000008947 | 8 | - | 69348407 | 69350836 | 397 | annot_exons |
| novel_circ_008567 | ENSSSCG00000000755 | 5 | + | 68354014 | 68380700 | 510 | annot_exons |
| novel_circ_008568 | ENSSSCG00000029920 | 8 | + | 87866307 | 87876502 | 844 | annot_exons |
| novel_circ_008569 | ENSSSCG00000021562 | 18 | + | 16498987 | 16499291 | 305 | one_exon |
| novel_circ_008570 | ENSSSCG00000016692 | 18 | - | 44890421 | 44903054 | 460 | annot_exons |
| novel_circ_008571 | ENSSSCG00000003895 | 6 | + | 1.65E+08 | 1.65E+08 | 343 | annot_exons |
| novel_circ_008572 | ENSSSCG00000004652 | 1 | + | 1.23E+08 | 1.23E+08 | 232 | annot_exons |
| novel_circ_008573 | ENSSSCG00000006872 | 4 | - | 1.18E+08 | 1.18E+08 | 279 | annot_exons |
| novel_circ_008574 | ENSSSCG00000016943 | 16 | - | 44066493 | 44071188 | 281 | annot_exons |
| novel_circ_008575 | ENSSSCG00000000555 | 5 | + | 47388932 | 47416672 | 2310 | exon_intron |
| novel_circ_008576 | ENSSSCG00000008339 | 3 | + | 73046115 | 73059202 | 622 | annot_exons |
| novel_circ_008577 | ENSSSCG00000017505 | 12 | + | 22853900 | 22856035 | 239 | annot_exons |
| novel_circ_008578 | ENSSSCG00000004473 | 1 | + | 87134227 | 87153004 | 411 | annot_exons |
| novel_circ_008579 | ENSSSCG00000025606 | 9 | + | 36656682 | 36663203 | 818 | annot_exons |
| novel_circ_008580 | ENSSSCG00000023434 | 13 | - | 1.01E+08 | 1.01E+08 | 745 | antisense |
| novel_circ_008581 | ENSSSCG00000009230 | 8 | + | 1.34E+08 | 1.34E+08 | 947 | annot_exons |
| novel_circ_008582 | ENSSSCG00000036418 | 3 | + | 99897136 | 99898967 | 1831 | exon_intron |
| novel_circ_008583 | ENSSSCG00000037204 | 14 | + | 61055671 | 61056168 | 223 | annot_exons |
| novel_circ_008584 | ENSSSCG00000038607 | 13 | - | 17017302 | 17020482 | 218 | annot_exons |
| novel_circ_008585 | ENSSSCG00000016938 | 16 | + | 41145224 | 41194153 | 1449 | annot_exons |
| novel_circ_008586 | ENSSSCG00000007478 | 17 | - | 53037004 | 53050028 | 7051 | exon_intron |
| novel_circ_008587 | ENSSSCG00000012534 | X | - | 84554467 | 84554949 | 483 | one_exon |
| novel_circ_008588 | ENSSSCG00000001076 | 7 | + | 14126030 | 14168049 | 388 | annot_exons |
| novel_circ_008589 | ENSSSCG00000016138 | 5 | + | 16628232 | 16657139 | 200 | annot_exons |
| novel_circ_008590 | ENSSSCG00000004985 | 1 | + | 1.7E+08 | 1.7E+08 | 150 | annot_exons |
| novel_circ_008591 | ENSSSCG00000016397 | 15 | + | 674840 | 677690 | 516 | annot_exons |
| novel_circ_008592 | ENSSSCG00000014269 | 2 | - | 1.34E+08 | 1.34E+08 | 560 | annot_exons |
| novel_circ_008593 | ENSSSCG00000014292 | 2 | + | 1.35E+08 | 1.35E+08 | 322 | annot_exons |
| novel_circ_008594 | ENSSSCG00000022725 | 7 | - | 23262630 | 23263295 | 369 | annot_exons |
| novel_circ_008595 | ENSSSCG00000024761 | 9 | - | 87018358 | 87039523 | 519 | annot_exons |
| novel_circ_008596 | ENSSSCG00000037153 | 2 | + | 74042922 | 74044333 | 1412 | antisense |
| novel_circ_008597 | ENSSSCG00000021588 | 1 | + | 1.08E+08 | 1.08E+08 | 281 | one_exon |
| novel_circ_008598 | ENSSSCG00000021837 | 3 | + | 10171702 | 10193809 | 707 | annot_exons |
| novel_circ_008599 | ENSSSCG00000029837 | 11 | + | 25130960 | 25200474 | 1296 | annot_exons |
| novel_circ_008600 | ENSSSCG00000005382 | 1 | + | 2.41E+08 | 2.41E+08 | 720 | annot_exons |
| novel_circ_008601 | ENSSSCG00000014960 | 9 | + | 26899161 | 26923854 | 1379 | annot_exons |
| novel_circ_008602 | ENSSSCG00000007478 | 17 | - | 52974200 | 53014574 | 1026 | annot_exons |
| novel_circ_008603 | ENSSSCG00000011235 | 13 | - | 19228140 | 19234893 | 554 | annot_exons |
| novel_circ_008604 | ENSSSCG00000029602 | 10 | - | 68491183 | 68493795 | 1986 | exon_intron |
| novel_circ_008605 | ENSSSCG00000006089 | 4 | - | 40330385 | 40339022 | 453 | annot_exons |
| novel_circ_008606 | ENSSSCG00000010494 | 14 | - | 1.07E+08 | 1.07E+08 | 487 | annot_exons |
| novel_circ_008607 | ENSSSCG00000005969 | 4 | + | 14607025 | 14629761 | 1092 | annot_exons |
| novel_circ_008608 | ENSSSCG00000033649 | 9 | - | 53005823 | 53006033 | 211 | one_exon |
| novel_circ_008609 | ENSSSCG00000003630 | 6 | + | 92121987 | 92128599 | 330 | annot_exons |
| novel_circ_008610 | ENSSSCG00000004089 | 1 | + | 14817548 | 14851175 | 709 | annot_exons |
| novel_circ_008611 | ENSSSCG00000006388 | 4 | + | 90235723 | 90244818 | 1114 | annot_exons |
| novel_circ_008612 | ENSSSCG00000004639 | 1 | - | 1.21E+08 | 1.21E+08 | 424 | annot_exons |
| novel_circ_008613 | ENSSSCG00000009967 | 14 | - | 45227613 | 45230916 | 267 | annot_exons |
| novel_circ_008614 | ENSSSCG00000000488 | 5 | + | 33124742 | 33130113 | 317 | annot_exons |
| novel_circ_008615 | ENSSSCG00000018016 | 12 | + | 56438413 | 56473522 | 478 | annot_exons |
| novel_circ_008616 | ENSSSCG00000040749 | 6 | + | 27779975 | 27780426 | 175 | annot_exons |
| novel_circ_008617 | NA | 15 | - | 84428248 | 84431003 | 2756 | intergenic |
| novel_circ_008618 | ENSSSCG00000006740 | 4 | - | 1.04E+08 | 1.04E+08 | 393 | annot_exons |
| novel_circ_008619 | ENSSSCG00000016498 | 18 | + | 9342606 | 9352442 | 586 | annot_exons |
| novel_circ_008620 | ENSSSCG00000021891 | 2 | + | 5876316 | 5876593 | 278 | one_exon |
| novel_circ_008621 | ENSSSCG00000002555 | AEMK02000452.1 | - | 3269241 | 3270506 | 483 | annot_exons |
| novel_circ_008622 | ENSSSCG00000024750 | 6 | - | 28311909 | 28312248 | 340 | one_exon |
| novel_circ_008623 | ENSSSCG00000015558 | 9 | + | 1.25E+08 | 1.25E+08 | 287 | annot_exons |
| novel_circ_008624 | ENSSSCG00000015488 | 9 | - | 1.16E+08 | 1.16E+08 | 603 | annot_exons |
| novel_circ_008625 | ENSSSCG00000009172 | 8 | + | 1.2E+08 | 1.2E+08 | 39062 | exon_intron |
| novel_circ_008626 | ENSSSCG00000034952 | 17 | - | 52363300 | 52373971 | 333 | annot_exons |
| novel_circ_008627 | ENSSSCG00000015857 | 15 | - | 59226488 | 59230432 | 363 | annot_exons |
| novel_circ_008628 | ENSSSCG00000001926 | 7 | - | 60884414 | 60899561 | 15148 | exon_intron |
| novel_circ_008629 | ENSSSCG00000015940 | 15 | + | 76177104 | 76192330 | 261 | annot_exons |
| novel_circ_008630 | ENSSSCG00000014800 | 9 | + | 6555095 | 6561930 | 338 | annot_exons |
| novel_circ_008631 | ENSSSCG00000010698 | 14 | - | 1.32E+08 | 1.32E+08 | 16538 | intronic |
| novel_circ_008632 | ENSSSCG00000014913 | 9 | - | 19885865 | 19913189 | 635 | annot_exons |
| novel_circ_008633 | ENSSSCG00000014913 | 9 | - | 19897760 | 19909216 | 273 | annot_exons |
| novel_circ_008634 | ENSSSCG00000010928 | 10 | - | 24795546 | 24796546 | 294 | annot_exons |
| novel_circ_008635 | ENSSSCG00000004576 | 1 | + | 1.11E+08 | 1.11E+08 | 23298 | intronic |
| novel_circ_008636 | ENSSSCG00000017688 | 12 | + | 38103106 | 38107692 | 518 | annot_exons |
| novel_circ_008637 | ENSSSCG00000038469 | 9 | + | 91944390 | 91961419 | 384 | annot_exons |
| novel_circ_008638 | ENSSSCG00000000952 | 5 | - | 1.04E+08 | 1.04E+08 | 577 | annot_exons |
| novel_circ_008639 | ENSSSCG00000032171 | 5 | + | 6484365 | 6490077 | 5713 | one_exon |
| novel_circ_008640 | ENSSSCG00000008496 | 3 | + | 1.03E+08 | 1.03E+08 | 450 | annot_exons |
| novel_circ_008641 | ENSSSCG00000006872 | 4 | - | 1.18E+08 | 1.18E+08 | 1070 | annot_exons |
| novel_circ_008642 | ENSSSCG00000016948 | 16 | - | 44117112 | 44136109 | 935 | annot_exons |
| novel_circ_008643 | ENSSSCG00000034993 | 2 | - | 1.18E+08 | 1.18E+08 | 210 | antisense |
| novel_circ_008644 | ENSSSCG00000038682 | 9 | - | 26156756 | 26157263 | 102 | annot_exons |
| novel_circ_008645 | ENSSSCG00000012062 | 13 | + | 2.01E+08 | 2.01E+08 | 1312 | annot_exons |
| novel_circ_008646 | ENSSSCG00000017063 | 16 | - | 66609509 | 66700676 | 310 | annot_exons |
| novel_circ_008647 | ENSSSCG00000001080 | 7 | + | 16297379 | 16340574 | 1748 | annot_exons |
| novel_circ_008648 | ENSSSCG00000015747 | 15 | + | 33569805 | 33574804 | 669 | annot_exons |
| novel_circ_008649 | ENSSSCG00000016661 | 18 | - | 38115728 | 38132080 | 343 | annot_exons |
| novel_circ_008650 | ENSSSCG00000008297 | 3 | + | 69241158 | 69242579 | 300 | annot_exons |
| novel_circ_008651 | ENSSSCG00000031271 | 15 | + | 1.05E+08 | 1.05E+08 | 771 | annot_exons |
| novel_circ_008652 | ENSSSCG00000007280 | 17 | + | 37905031 | 37915774 | 391 | annot_exons |
| novel_circ_008653 | ENSSSCG00000028228 | 3 | + | 80295054 | 80297962 | 869 | annot_exons |
| novel_circ_008654 | NA | 15 | - | 84307352 | 84307645 | 294 | intergenic |
| novel_circ_008655 | ENSSSCG00000001950 | 7 | - | 64622583 | 64626089 | 345 | annot_exons |
| novel_circ_008656 | ENSSSCG00000006090 | 4 | + | 40379783 | 40393227 | 924 | annot_exons |
| novel_circ_008657 | ENSSSCG00000000770 | 5 | - | 69952831 | 69955947 | 403 | annot_exons |
| novel_circ_008658 | ENSSSCG00000035639 | 6 | - | 27526548 | 27533552 | 591 | annot_exons |
| novel_circ_008659 | ENSSSCG00000030677 | 13 | - | 1.97E+08 | 1.97E+08 | 214 | annot_exons |
| novel_circ_008660 | ENSSSCG00000009338 | 11 | + | 8561600 | 8561799 | 200 | one_exon |
| novel_circ_008661 | ENSSSCG00000029331 | 14 | - | 20707527 | 20711059 | 177 | annot_exons |
| novel_circ_008662 | ENSSSCG00000021739 | 2 | - | 18499951 | 18500836 | 110 | annot_exons |
| novel_circ_008663 | ENSSSCG00000007280 | 17 | + | 37862504 | 37891423 | 1128 | annot_exons |
| novel_circ_008664 | ENSSSCG00000006090 | 4 | + | 40379783 | 40382549 | 493 | annot_exons |
| novel_circ_008665 | ENSSSCG00000011022 | 10 | + | 41381255 | 41384739 | 504 | annot_exons |
| novel_circ_008666 | ENSSSCG00000021814 | 11 | + | 675689 | 683082 | 1143 | annot_exons |
| novel_circ_008667 | ENSSSCG00000006701 | 4 | - | 1E+08 | 1E+08 | 1211 | annot_exons |
| novel_circ_008668 | ENSSSCG00000029035 | 2 | - | 70592974 | 70594737 | 345 | annot_exons |
| novel_circ_008669 | ENSSSCG00000000753 | 5 | + | 68118449 | 68119569 | 475 | annot_exons |
| novel_circ_008670 | ENSSSCG00000030660 | 6 | + | 83545878 | 83546179 | 302 | one_exon |
| novel_circ_008671 | ENSSSCG00000015959 | 15 | + | 78994268 | 78994727 | 161 | annot_exons |
| novel_circ_008672 | ENSSSCG00000026281 | 13 | - | 30263943 | 30297674 | 1162 | annot_exons |
| novel_circ_008673 | ENSSSCG00000026153 | 9 | + | 36532608 | 36533977 | 315 | annot_exons |
| novel_circ_008674 | ENSSSCG00000003630 | 6 | + | 92160465 | 92161706 | 356 | annot_exons |
| novel_circ_008675 | ENSSSCG00000008832 | 8 | - | 39111148 | 39116257 | 360 | annot_exons |
| novel_circ_008676 | ENSSSCG00000025675 | 2 | - | 74754409 | 74754588 | 180 | antisense |
| novel_circ_008677 | ENSSSCG00000010739 | 14 | - | 1.34E+08 | 1.34E+08 | 6608 | exon_intron |
| novel_circ_008678 | ENSSSCG00000014062 | 2 | - | 81566651 | 81578018 | 507 | annot_exons |
| novel_circ_008679 | ENSSSCG00000004919 | 1 | - | 1.62E+08 | 1.62E+08 | 352 | annot_exons |
| novel_circ_008680 | ENSSSCG00000003747 | 6 | + | 1.21E+08 | 1.21E+08 | 412 | exon_intron |
| novel_circ_008681 | ENSSSCG00000011496 | 13 | - | 46075637 | 46110421 | 681 | annot_exons |
| novel_circ_008682 | ENSSSCG00000004551 | 1 | - | 1.08E+08 | 1.08E+08 | 18077 | exon_intron |
| novel_circ_008683 | ENSSSCG00000004064 | 1 | - | 9923614 | 9976196 | 286 | annot_exons |
| novel_circ_008684 | ENSSSCG00000004473 | 1 | + | 87177832 | 87178553 | 289 | annot_exons |
| novel_circ_008685 | ENSSSCG00000016194 | 15 | - | 1.21E+08 | 1.21E+08 | 373 | annot_exons |
| novel_circ_008686 | ENSSSCG00000021270 | 5 | - | 35683735 | 35685136 | 1402 | antisense |
| novel_circ_008687 | ENSSSCG00000008445 | 3 | + | 94934784 | 94969558 | 869 | annot_exons |
| novel_circ_008688 | ENSSSCG00000017826 | 12 | - | 48377612 | 48381478 | 218 | annot_exons |
| novel_circ_008689 | ENSSSCG00000017678 | 12 | + | 37657577 | 37662196 | 4620 | exon_intron |
| novel_circ_008690 | ENSSSCG00000034364 | 12 | - | 59556291 | 59557816 | 1526 | one_exon |
| novel_circ_008691 | ENSSSCG00000031903 | 2 | + | 1306684 | 1308059 | 1376 | one_exon |
| novel_circ_008692 | ENSSSCG00000008448 | 3 | + | 96116880 | 96119457 | 397 | annot_exons |
| novel_circ_008693 | ENSSSCG00000007228 | 17 | + | 35256740 | 35257315 | 228 | annot_exons |
| novel_circ_008694 | NA | 9 | - | 45444793 | 45447394 | 2602 | intergenic |
| novel_circ_008695 | ENSSSCG00000011183 | 13 | - | 1510570 | 1513988 | 301 | annot_exons |
| novel_circ_008696 | ENSSSCG00000038913 | 14 | - | 71179829 | 71198359 | 293 | annot_exons |
| novel_circ_008697 | ENSSSCG00000035676 | 16 | - | 71255238 | 71262738 | 663 | annot_exons |
| novel_circ_008698 | ENSSSCG00000016397 | 15 | + | 715316 | 715678 | 210 | annot_exons |
| novel_circ_008699 | ENSSSCG00000003760 | 6 | + | 1.3E+08 | 1.3E+08 | 222 | annot_exons |
| novel_circ_008700 | ENSSSCG00000015213 | 9 | + | 52743673 | 52745743 | 409 | annot_exons |
| novel_circ_008701 | ENSSSCG00000015857 | 15 | - | 59218157 | 59259639 | 1793 | annot_exons |
| novel_circ_008702 | ENSSSCG00000010627 | 14 | + | 1.21E+08 | 1.21E+08 | 512 | annot_exons |
| novel_circ_008703 | ENSSSCG00000004209 | 1 | + | 35131947 | 35216500 | 1416 | annot_exons |
| novel_circ_008704 | ENSSSCG00000004134 | 1 | - | 21779719 | 21781094 | 416 | annot_exons |
| novel_circ_008705 | ENSSSCG00000001577 | 7 | + | 32894555 | 32897792 | 512 | annot_exons |
| novel_circ_008706 | ENSSSCG00000017752 | 12 | + | 43895415 | 43899845 | 2652 | annot_exons |
| novel_circ_008707 | ENSSSCG00000012251 | X | + | 36792047 | 36801183 | 2919 | annot_exons |
| novel_circ_008708 | ENSSSCG00000013402 | 2 | - | 47525392 | 47537458 | 1668 | annot_exons |
| novel_circ_008709 | ENSSSCG00000015872 | 15 | + | 63710083 | 63711307 | 443 | annot_exons |
| novel_circ_008710 | ENSSSCG00000008513 | 3 | - | 1.07E+08 | 1.07E+08 | 972 | annot_exons |
| novel_circ_008711 | ENSSSCG00000030679 | 1 | + | 1.75E+08 | 1.75E+08 | 525 | one_exon |
| novel_circ_008712 | ENSSSCG00000021203 | 13 | - | 79518730 | 79532151 | 817 | annot_exons |
| novel_circ_008713 | ENSSSCG00000009042 | 8 | + | 83267616 | 83268011 | 288 | annot_exons |
| novel_circ_008714 | ENSSSCG00000022331 | X | - | 1.14E+08 | 1.14E+08 | 1090 | intronic |
| novel_circ_008715 | ENSSSCG00000037766 | 8 | + | 80312232 | 80313990 | 1759 | one_exon |
| novel_circ_008716 | ENSSSCG00000034491 | 5 | - | 73712022 | 73712885 | 864 | one_exon |
| novel_circ_008717 | ENSSSCG00000039157 | 15 | + | 29977370 | 29980632 | 373 | annot_exons |
| novel_circ_008718 | ENSSSCG00000015931 | 15 | - | 75875928 | 75876309 | 382 | antisense |
| novel_circ_008719 | NA | 14 | - | 3916866 | 3919973 | 3108 | intergenic |
| novel_circ_008720 | ENSSSCG00000038561 | 15 | - | 84412319 | 84412897 | 434 | annot_exons |
| novel_circ_008721 | ENSSSCG00000038561 | 15 | - | 84413078 | 84413903 | 561 | annot_exons |
| novel_circ_008722 | ENSSSCG00000011068 | 10 | - | 48820847 | 48827379 | 460 | annot_exons |
| novel_circ_008723 | ENSSSCG00000015071 | 9 | - | 44226341 | 44227691 | 304 | annot_exons |
| novel_circ_008724 | ENSSSCG00000012362 | X | - | 50389422 | 50425041 | 372 | annot_exons |
| novel_circ_008725 | ENSSSCG00000037105 | 7 | + | 1.21E+08 | 1.21E+08 | 347 | annot_exons |
| novel_circ_008726 | ENSSSCG00000009090 | 8 | - | 1.02E+08 | 1.02E+08 | 308 | annot_exons |
| novel_circ_008727 | ENSSSCG00000010826 | 10 | + | 10169065 | 10170555 | 304 | annot_exons |
| novel_circ_008728 | ENSSSCG00000011803 | 13 | + | 1.25E+08 | 1.25E+08 | 569 | one_exon |
| novel_circ_008729 | ENSSSCG00000023297 | 15 | + | 80385694 | 80399628 | 13935 | exon_intron |
| novel_circ_008730 | ENSSSCG00000003612 | 6 | + | 88702706 | 88703422 | 275 | annot_exons |
| novel_circ_008731 | ENSSSCG00000009319 | 11 | + | 5577247 | 5581004 | 468 | annot_exons |
| novel_circ_008732 | ENSSSCG00000022430 | 16 | + | 47293393 | 47296893 | 276 | annot_exons |
| novel_circ_008733 | ENSSSCG00000008475 | 3 | + | 1.01E+08 | 1.01E+08 | 273 | annot_exons |
| novel_circ_008734 | ENSSSCG00000023078 | 13 | - | 2.06E+08 | 2.06E+08 | 331 | annot_exons |
| novel_circ_008735 | ENSSSCG00000009779 | 14 | + | 29689597 | 29693488 | 244 | annot_exons |
| novel_circ_008736 | ENSSSCG00000015314 | 9 | + | 72230096 | 72230374 | 279 | one_exon |
| novel_circ_008737 | ENSSSCG00000004371 | 1 | + | 72737792 | 72738156 | 273 | annot_exons |
| novel_circ_008738 | NA | 15 | + | 84290209 | 84290547 | 339 | intergenic |
| novel_circ_008739 | ENSSSCG00000039094 | 4 | - | 752988 | 753311 | 324 | antisense |
| novel_circ_008740 | ENSSSCG00000034395 | 15 | + | 1.11E+08 | 1.11E+08 | 269 | annot_exons |
| novel_circ_008741 | ENSSSCG00000011194 | 13 | - | 2981756 | 2983349 | 289 | annot_exons |
| novel_circ_008742 | ENSSSCG00000004454 | 1 | + | 82878091 | 82891579 | 266 | annot_exons |
| novel_circ_008743 | ENSSSCG00000005090 | 1 | + | 1.9E+08 | 1.9E+08 | 598 | annot_exons |
| novel_circ_008744 | ENSSSCG00000012884 | 2 | - | 4412502 | 4442066 | 1238 | annot_exons |
| novel_circ_008745 | ENSSSCG00000000806 | 5 | - | 76839521 | 76844589 | 2485 | annot_exons |
| novel_circ_008746 | ENSSSCG00000021802 | 1 | - | 2.25E+08 | 2.25E+08 | 470 | one_exon |
| novel_circ_008747 | ENSSSCG00000006179 | 4 | + | 62146811 | 62178989 | 557 | annot_exons |
| novel_circ_008748 | ENSSSCG00000025478 | 6 | + | 1.07E+08 | 1.07E+08 | 1600 | annot_exons |
| novel_circ_008749 | ENSSSCG00000015462 | 9 | + | 1.13E+08 | 1.13E+08 | 36308 | exon_intron |
| novel_circ_008750 | ENSSSCG00000015567 | 9 | - | 1.26E+08 | 1.26E+08 | 546 | annot_exons |
| novel_circ_008751 | ENSSSCG00000007043 | 17 | - | 14445562 | 14446776 | 351 | annot_exons |
| novel_circ_008752 | ENSSSCG00000013865 | 2 | - | 60858802 | 60877586 | 1914 | annot_exons |
| novel_circ_008753 | NA | 15 | - | 84287800 | 84288177 | 378 | intergenic |
| novel_circ_008754 | ENSSSCG00000014915 | 9 | + | 20130905 | 20138153 | 438 | annot_exons |
| novel_circ_008755 | ENSSSCG00000022083 | 5 | + | 85065913 | 85124635 | 801 | annot_exons |
| novel_circ_008756 | ENSSSCG00000005343 | 1 | - | 2.37E+08 | 2.37E+08 | 408 | annot_exons |
| novel_circ_008757 | ENSSSCG00000030697 | 13 | + | 2471206 | 2474387 | 307 | annot_exons |
| novel_circ_008758 | ENSSSCG00000016272 | 15 | + | 1.32E+08 | 1.32E+08 | 335 | annot_exons |
| novel_circ_008759 | ENSSSCG00000037673 | 3 | - | 28600206 | 28600542 | 337 | one_exon |
| novel_circ_008760 | ENSSSCG00000000757 | 5 | + | 68782717 | 68792945 | 373 | annot_exons |
| novel_circ_008761 | ENSSSCG00000007280 | 17 | + | 37887802 | 37891423 | 524 | annot_exons |
| novel_circ_008762 | ENSSSCG00000014127 | 2 | + | 89683592 | 89696375 | 857 | annot_exons |
| novel_circ_008763 | ENSSSCG00000006089 | 4 | - | 40335139 | 40345193 | 436 | annot_exons |
| novel_circ_008764 | ENSSSCG00000006619 | 4 | - | 97566524 | 97576926 | 584 | annot_exons |
| novel_circ_008765 | ENSSSCG00000009916 | 14 | + | 40632358 | 40632773 | 288 | annot_exons |
| novel_circ_008766 | ENSSSCG00000008495 | 3 | + | 1.03E+08 | 1.03E+08 | 308 | annot_exons |
| novel_circ_008767 | ENSSSCG00000017688 | 12 | + | 38083733 | 38085933 | 335 | annot_exons |
| novel_circ_008768 | ENSSSCG00000039516 | 6 | - | 65266429 | 65268757 | 2329 | intronic |
| novel_circ_008769 | ENSSSCG00000005444 | 1 | - | 2.5E+08 | 2.5E+08 | 269 | annot_exons |
| novel_circ_008770 | ENSSSCG00000025592 | 13 | + | 1.27E+08 | 1.27E+08 | 65947 | exon_intron |
| novel_circ_008771 | ENSSSCG00000011720 | 13 | + | 92685016 | 92685264 | 249 | intronic |
| novel_circ_008772 | ENSSSCG00000012163 | X | - | 16491482 | 16492148 | 252 | annot_exons |
| novel_circ_008773 | ENSSSCG00000009569 | 11 | - | 543480 | 562934 | 756 | annot_exons |
| novel_circ_008774 | ENSSSCG00000009061 | 8 | - | 87660369 | 87663752 | 263 | annot_exons |
| novel_circ_008775 | ENSSSCG00000015097 | 9 | - | 46044839 | 46046455 | 173 | annot_exons |
| novel_circ_008776 | NA | 15 | - | 84231488 | 84232434 | 947 | intergenic |
| novel_circ_008777 | ENSSSCG00000016101 | 15 | + | 1.05E+08 | 1.05E+08 | 511 | one_exon |
| novel_circ_008778 | ENSSSCG00000007839 | 3 | - | 23921028 | 23921795 | 261 | annot_exons |
| novel_circ_008779 | ENSSSCG00000012328 | X | - | 46334487 | 46354249 | 1692 | annot_exons |
| novel_circ_008780 | ENSSSCG00000002937 | 6 | - | 46506704 | 46508460 | 216 | annot_exons |
| novel_circ_008781 | ENSSSCG00000007477 | 17 | - | 52866612 | 52872012 | 670 | annot_exons |
| novel_circ_008782 | ENSSSCG00000009134 | 8 | - | 1.12E+08 | 1.12E+08 | 1926 | annot_exons |
| novel_circ_008783 | ENSSSCG00000022296 | 15 | + | 1.05E+08 | 1.05E+08 | 245 | annot_exons |
| novel_circ_008784 | ENSSSCG00000001927 | 7 | + | 60906695 | 60909340 | 616 | annot_exons |
| novel_circ_008785 | ENSSSCG00000005943 | 4 | + | 7824345 | 7835519 | 11175 | exon_intron |
| novel_circ_008786 | ENSSSCG00000011041 | 10 | - | 44928914 | 44935556 | 346 | annot_exons |
| novel_circ_008787 | ENSSSCG00000008569 | 3 | + | 1.13E+08 | 1.13E+08 | 264 | antisense |
| novel_circ_008788 | ENSSSCG00000030343 | 16 | - | 34798441 | 34813720 | 814 | annot_exons |
| novel_circ_008789 | ENSSSCG00000030467 | 13 | - | 1.09E+08 | 1.09E+08 | 505 | annot_exons |
| novel_circ_008790 | ENSSSCG00000040373 | 5 | + | 69825738 | 69838356 | 412 | annot_exons |
| novel_circ_008791 | ENSSSCG00000012456 | X | - | 67336836 | 67356454 | 360 | annot_exons |
| novel_circ_008792 | ENSSSCG00000006522 | 4 | + | 94601795 | 94602258 | 339 | annot_exons |
| novel_circ_008793 | ENSSSCG00000003660 | 6 | - | 95393610 | 95395073 | 449 | exon_intron |
| novel_circ_008794 | ENSSSCG00000034704 | 1 | - | 1.62E+08 | 1.62E+08 | 417 | annot_exons |
| novel_circ_008795 | ENSSSCG00000005096 | 1 | + | 1.91E+08 | 1.91E+08 | 422 | annot_exons |
| novel_circ_008796 | ENSSSCG00000010742 | 14 | - | 1.35E+08 | 1.35E+08 | 416 | annot_exons |
| novel_circ_008797 | ENSSSCG00000005043 | 1 | - | 1.83E+08 | 1.83E+08 | 574 | annot_exons |
| novel_circ_008798 | ENSSSCG00000009473 | 11 | - | 49203468 | 49205360 | 358 | annot_exons |
| novel_circ_008799 | ENSSSCG00000014917 | 9 | + | 20257548 | 20259537 | 425 | annot_exons |
| novel_circ_008800 | ENSSSCG00000021791 | 13 | + | 1.58E+08 | 1.58E+08 | 343 | one_exon |
| novel_circ_008801 | ENSSSCG00000008787 | 8 | + | 30396357 | 30407357 | 730 | annot_exons |
| novel_circ_008802 | ENSSSCG00000014267 | 2 | - | 1.34E+08 | 1.34E+08 | 449 | annot_exons |
| novel_circ_008803 | ENSSSCG00000033228 | 12 | - | 27268443 | 27275982 | 430 | annot_exons |
| novel_circ_008804 | ENSSSCG00000004729 | 1 | - | 1.29E+08 | 1.29E+08 | 667 | annot_exons |
| novel_circ_008805 | ENSSSCG00000042878 | 1 | + | 1.32E+08 | 1.32E+08 | 2647 | antisense |
| novel_circ_008806 | ENSSSCG00000025592 | 13 | + | 1.27E+08 | 1.27E+08 | 262 | annot_exons |
| novel_circ_008807 | ENSSSCG00000041094 | 4 | + | 1.28E+08 | 1.28E+08 | 466 | intronic |
| novel_circ_008808 | ENSSSCG00000038607 | 13 | - | 17012278 | 17020482 | 441 | annot_exons |
| novel_circ_008809 | ENSSSCG00000003712 | 6 | - | 1.09E+08 | 1.09E+08 | 1790 | exon_intron |
| novel_circ_008810 | ENSSSCG00000028446 | 13 | - | 34091316 | 34091814 | 290 | annot_exons |
| novel_circ_008811 | ENSSSCG00000010351 | 14 | + | 85602428 | 85611075 | 254 | annot_exons |
| novel_circ_008812 | ENSSSCG00000024316 | 7 | + | 58631041 | 58643977 | 480 | annot_exons |
| novel_circ_008813 | ENSSSCG00000008099 | 3 | + | 44179926 | 44181936 | 345 | annot_exons |
| novel_circ_008814 | ENSSSCG00000028004 | 17 | + | 28045027 | 28047838 | 438 | annot_exons |
| novel_circ_008815 | ENSSSCG00000036546 | 2 | + | 49015139 | 49020698 | 293 | annot_exons |
| novel_circ_008816 | ENSSSCG00000012434 | X | - | 61765511 | 61787744 | 347 | annot_exons |
| novel_circ_008817 | ENSSSCG00000027293 | 16 | + | 49131828 | 49135093 | 187 | annot_exons |
| novel_circ_008818 | ENSSSCG00000010290 | 14 | + | 75700988 | 75702683 | 276 | annot_exons |
| novel_circ_008819 | ENSSSCG00000002269 | 7 | + | 88506227 | 88512410 | 470 | annot_exons |
| novel_circ_008820 | ENSSSCG00000023996 | 1 | - | 1.27E+08 | 1.27E+08 | 226 | annot_exons |
| novel_circ_008821 | ENSSSCG00000009480 | 11 | + | 51531489 | 51540684 | 394 | annot_exons |
| novel_circ_008822 | ENSSSCG00000000891 | 5 | - | 87516546 | 87516925 | 380 | antisense |
| novel_circ_008823 | ENSSSCG00000001683 | 7 | + | 38592559 | 38593402 | 244 | annot_exons |
| novel_circ_008824 | ENSSSCG00000016553 | 18 | + | 18251378 | 18276491 | 636 | annot_exons |
| novel_circ_008825 | ENSSSCG00000033185 | 13 | + | 77013571 | 77017976 | 225 | annot_exons |
| novel_circ_008826 | ENSSSCG00000043557 | 13 | - | 29688999 | 29700873 | 8407 | exon_intron |
| novel_circ_008827 | ENSSSCG00000030547 | 4 | + | 98033312 | 98034793 | 1482 | antisense |
| novel_circ_008828 | ENSSSCG00000001877 | 7 | + | 58054321 | 58054733 | 202 | annot_exons |
| novel_circ_008829 | ENSSSCG00000038873 | 13 | + | 39633254 | 39635437 | 2184 | exon_intron |
| novel_circ_008830 | ENSSSCG00000006324 | 4 | + | 85039073 | 85042510 | 603 | annot_exons |
| novel_circ_008831 | ENSSSCG00000043604 | X | - | 59548427 | 59571888 | 907 | exon_intron |
| novel_circ_008832 | ENSSSCG00000037954 | 1 | - | 2.42E+08 | 2.42E+08 | 241 | antisense |
| novel_circ_008833 | ENSSSCG00000003670 | 6 | - | 95949680 | 95950107 | 428 | antisense |
| novel_circ_008834 | ENSSSCG00000014044 | 2 | - | 80748379 | 80807643 | 3016 | annot_exons |
| novel_circ_008835 | ENSSSCG00000038861 | 6 | + | 1.51E+08 | 1.51E+08 | 940 | annot_exons |
| novel_circ_008836 | ENSSSCG00000004242 | 1 | + | 41178748 | 41247966 | 1087 | annot_exons |
| novel_circ_008837 | ENSSSCG00000017791 | 12 | - | 45870808 | 45904373 | 16672 | exon_intron |
| novel_circ_008838 | ENSSSCG00000011163 | 10 | + | 69070327 | 69076370 | 803 | annot_exons |
| novel_circ_008839 | ENSSSCG00000026744 | 11 | - | 1335980 | 1364698 | 695 | annot_exons |
| novel_circ_008840 | ENSSSCG00000004390 | 1 | - | 75122222 | 75153476 | 31255 | intronic |
| novel_circ_008841 | ENSSSCG00000000842 | 5 | - | 79297641 | 79298171 | 331 | annot_exons |
| novel_circ_008842 | ENSSSCG00000002872 | 6 | + | 44006567 | 44011694 | 656 | annot_exons |
| novel_circ_008843 | ENSSSCG00000004064 | 1 | - | 9797465 | 9801736 | 1012 | annot_exons |
| novel_circ_008844 | ENSSSCG00000015853 | 15 | + | 56527505 | 56547111 | 2451 | annot_exons |
| novel_circ_008845 | ENSSSCG00000003559 | 6 | + | 84077910 | 84080165 | 570 | annot_exons |
| novel_circ_008846 | ENSSSCG00000016853 | 16 | - | 24193110 | 24213797 | 295 | annot_exons |
| novel_circ_008847 | ENSSSCG00000015743 | 15 | - | 31473464 | 31473618 | 155 | one_exon |
| novel_circ_008848 | ENSSSCG00000010298 | 14 | - | 76140586 | 76141020 | 252 | annot_exons |
| novel_circ_008849 | ENSSSCG00000024088 | 1 | - | 1.09E+08 | 1.09E+08 | 587 | exon_intron |
| novel_circ_008850 | ENSSSCG00000022222 | 15 | + | 82069808 | 82094002 | 23228 | exon_intron |
| novel_circ_008851 | NA | 7 | + | 68220757 | 68230272 | 9516 | intergenic |
| novel_circ_008852 | ENSSSCG00000011652 | 13 | - | 77340728 | 77342010 | 278 | annot_exons |
| novel_circ_008853 | ENSSSCG00000013522 | 2 | - | 73334106 | 73334305 | 200 | one_exon |
| novel_circ_008854 | ENSSSCG00000009390 | 11 | + | 17870879 | 17878988 | 8110 | exon_intron |
| novel_circ_008855 | ENSSSCG00000037355 | 4 | + | 99515003 | 99537986 | 314 | annot_exons |
| novel_circ_008856 | ENSSSCG00000014794 | 9 | + | 6355158 | 6374618 | 507 | annot_exons |
| novel_circ_008857 | ENSSSCG00000001921 | 7 | - | 60218700 | 60218975 | 276 | one_exon |
| novel_circ_008858 | ENSSSCG00000024938 | 13 | - | 2552854 | 2554975 | 2122 | exon_intron |
| novel_circ_008859 | ENSSSCG00000016156 | 15 | - | 1.13E+08 | 1.13E+08 | 907 | annot_exons |
| novel_circ_008860 | ENSSSCG00000015795 | 15 | - | 46516595 | 46516795 | 201 | intronic |
| novel_circ_008861 | ENSSSCG00000023266 | 6 | + | 1.69E+08 | 1.69E+08 | 823 | annot_exons |
| novel_circ_008862 | ENSSSCG00000011684 | 13 | + | 83716747 | 83721691 | 480 | annot_exons |
| novel_circ_008863 | ENSSSCG00000008513 | 3 | - | 1.07E+08 | 1.07E+08 | 671 | annot_exons |
| novel_circ_008864 | NA | 9 | - | 52281824 | 52283387 | 1564 | intergenic |
| novel_circ_008865 | ENSSSCG00000000886 | 5 | - | 85285642 | 85287681 | 535 | annot_exons |
| novel_circ_008866 | ENSSSCG00000015697 | 15 | + | 16974817 | 16975165 | 349 | antisense |
| novel_circ_008867 | ENSSSCG00000034139 | 1 | - | 30501967 | 30516606 | 14640 | exon_intron |
| novel_circ_008868 | ENSSSCG00000027767 | 14 | + | 88079260 | 88079759 | 330 | annot_exons |
| novel_circ_008869 | ENSSSCG00000005640 | 1 | - | 2.69E+08 | 2.69E+08 | 302 | exon_intron |
| novel_circ_008870 | ENSSSCG00000010467 | 14 | + | 1.04E+08 | 1.04E+08 | 426 | annot_exons |
| novel_circ_008871 | NA | 2 | - | 6808317 | 6808487 | 171 | intergenic |
| novel_circ_008872 | ENSSSCG00000024916 | 13 | - | 94336123 | 94338366 | 251 | annot_exons |
| novel_circ_008873 | ENSSSCG00000011511 | 13 | - | 50714324 | 50742186 | 339 | annot_exons |
| novel_circ_008874 | ENSSSCG00000026754 | 2 | + | 74812588 | 74831409 | 18822 | antisense |
| novel_circ_008875 | ENSSSCG00000009359 | 11 | - | 12858353 | 12862293 | 258 | annot_exons |
| novel_circ_008876 | NA | 15 | - | 84333461 | 84333658 | 198 | intergenic |
| novel_circ_008877 | ENSSSCG00000008753 | 8 | - | 16681471 | 16704217 | 1038 | annot_exons |
| novel_circ_008878 | ENSSSCG00000010863 | 10 | + | 15051106 | 15066662 | 1127 | annot_exons |
| novel_circ_008879 | ENSSSCG00000010470 | 14 | - | 1.04E+08 | 1.04E+08 | 695 | exon_intron |
| novel_circ_008880 | ENSSSCG00000033176 | 16 | - | 68700535 | 68700769 | 235 | exon_intron |
| novel_circ_008881 | ENSSSCG00000027299 | 6 | - | 64401351 | 64401532 | 182 | one_exon |
| novel_circ_008882 | ENSSSCG00000003693 | 6 | + | 1.04E+08 | 1.04E+08 | 966 | annot_exons |
| novel_circ_008883 | ENSSSCG00000010288 | 14 | - | 75288977 | 75309994 | 14823 | exon_intron |
| novel_circ_008884 | ENSSSCG00000033421 | 12 | - | 16295480 | 16299646 | 247 | annot_exons |
| novel_circ_008885 | ENSSSCG00000007713 | 3 | + | 10962194 | 10965931 | 373 | annot_exons |
| novel_circ_008886 | ENSSSCG00000010823 | 10 | + | 9690786 | 9691439 | 400 | annot_exons |
| novel_circ_008887 | ENSSSCG00000008304 | 3 | + | 69549911 | 69550517 | 211 | annot_exons |
| novel_circ_008888 | ENSSSCG00000016916 | 16 | - | 35120233 | 35123287 | 545 | annot_exons |
| novel_circ_008889 | ENSSSCG00000000793 | 5 | + | 73631382 | 73642776 | 257 | annot_exons |
| novel_circ_008890 | ENSSSCG00000006461 | 4 | + | 93115622 | 93116210 | 589 | exon_intron |
| novel_circ_008891 | ENSSSCG00000005582 | 1 | - | 2.64E+08 | 2.64E+08 | 452 | annot_exons |
| novel_circ_008892 | ENSSSCG00000008499 | 3 | + | 1.03E+08 | 1.03E+08 | 309 | annot_exons |
| novel_circ_008893 | ENSSSCG00000011389 | 13 | - | 32250956 | 32253622 | 2135 | exon_intron |
| novel_circ_008894 | ENSSSCG00000011340 | 13 | - | 30544508 | 30547685 | 163 | annot_exons |
| novel_circ_008895 | ENSSSCG00000028117 | 1 | - | 2.61E+08 | 2.61E+08 | 312 | annot_exons |
| novel_circ_008896 | ENSSSCG00000004958 | 1 | + | 1.66E+08 | 1.66E+08 | 547 | annot_exons |
| novel_circ_008897 | ENSSSCG00000013865 | 2 | - | 60811442 | 60877586 | 3240 | annot_exons |
| novel_circ_008898 | ENSSSCG00000017895 | 12 | - | 51657943 | 51680596 | 507 | annot_exons |
| novel_circ_008899 | ENSSSCG00000009994 | 14 | + | 46973303 | 46973910 | 207 | annot_exons |
| novel_circ_008900 | ENSSSCG00000002654 | 6 | - | 1964698 | 1964960 | 263 | antisense |
| novel_circ_008901 | ENSSSCG00000014255 | 2 | + | 1.31E+08 | 1.31E+08 | 322 | annot_exons |
| novel_circ_008902 | NA | 6 | - | 1.1E+08 | 1.1E+08 | 185 | intergenic |
| novel_circ_008903 | ENSSSCG00000004856 | 1 | - | 1.46E+08 | 1.46E+08 | 517 | annot_exons |
| novel_circ_008904 | ENSSSCG00000003693 | 6 | + | 1.04E+08 | 1.04E+08 | 504 | annot_exons |
| novel_circ_008905 | ENSSSCG00000016566 | 18 | - | 19068807 | 19087736 | 615 | annot_exons |
| novel_circ_008906 | ENSSSCG00000016177 | 15 | + | 1.18E+08 | 1.18E+08 | 559 | annot_exons |
| novel_circ_008907 | ENSSSCG00000011858 | 13 | + | 1.35E+08 | 1.35E+08 | 605 | annot_exons |
| novel_circ_008908 | ENSSSCG00000032176 | 12 | + | 14465466 | 14471485 | 355 | annot_exons |
| novel_circ_008909 | ENSSSCG00000016397 | 15 | + | 562477 | 571179 | 435 | annot_exons |
| novel_circ_008910 | ENSSSCG00000026161 | 3 | + | 1.25E+08 | 1.25E+08 | 419 | annot_exons |
| novel_circ_008911 | ENSSSCG00000021285 | 3 | - | 4269795 | 4279580 | 383 | annot_exons |
| novel_circ_008912 | ENSSSCG00000016618 | 18 | - | 25673846 | 25692875 | 734 | annot_exons |
| novel_circ_008913 | ENSSSCG00000003790 | 6 | + | 1.43E+08 | 1.43E+08 | 621 | annot_exons |
| novel_circ_008914 | ENSSSCG00000008858 | 8 | - | 43750083 | 43753643 | 748 | annot_exons |
| novel_circ_008915 | ENSSSCG00000020686 | 16 | - | 79847060 | 79848602 | 294 | annot_exons |
| novel_circ_008916 | ENSSSCG00000003859 | 6 | + | 1.6E+08 | 1.6E+08 | 607 | annot_exons |
| novel_circ_008917 | ENSSSCG00000042373 | 16 | + | 23186708 | 23186873 | 166 | one_exon |
| novel_circ_008918 | NA | 15 | - | 84421671 | 84421919 | 249 | intergenic |
| novel_circ_008919 | ENSSSCG00000020799 | 3 | - | 1.27E+08 | 1.27E+08 | 948 | annot_exons |
| novel_circ_008920 | ENSSSCG00000021427 | 2 | - | 1.52E+08 | 1.52E+08 | 261 | annot_exons |
| novel_circ_008921 | ENSSSCG00000000068 | 5 | - | 7344899 | 7348222 | 525 | annot_exons |
| novel_circ_008922 | ENSSSCG00000008513 | 3 | - | 1.07E+08 | 1.07E+08 | 1320 | annot_exons |
| novel_circ_008923 | ENSSSCG00000031775 | 5 | - | 73473989 | 73474309 | 228 | exon_intron |
| novel_circ_008924 | ENSSSCG00000009834 | 14 | - | 32699847 | 32701663 | 366 | annot_exons |
| novel_circ_008925 | ENSSSCG00000010900 | 10 | - | 20438111 | 20492105 | 568 | annot_exons |
| novel_circ_008926 | ENSSSCG00000011022 | 10 | + | 41430334 | 41431903 | 293 | annot_exons |
| novel_circ_008927 | ENSSSCG00000027139 | 13 | + | 1.34E+08 | 1.34E+08 | 623 | annot_exons |
| novel_circ_008928 | ENSSSCG00000030150 | 8 | - | 30738034 | 30752751 | 818 | annot_exons |
| novel_circ_008929 | ENSSSCG00000033823 | 5 | - | 7557452 | 7569533 | 525 | annot_exons |
| novel_circ_008930 | ENSSSCG00000001100 | 7 | + | 20283341 | 20289763 | 414 | annot_exons |
| novel_circ_008931 | ENSSSCG00000029538 | 9 | - | 39494895 | 39504434 | 739 | annot_exons |
| novel_circ_008932 | ENSSSCG00000015358 | 9 | - | 85418943 | 85419174 | 232 | one_exon |
| novel_circ_008933 | ENSSSCG00000016317 | 15 | + | 1.36E+08 | 1.36E+08 | 321 | annot_exons |
| novel_circ_008934 | ENSSSCG00000005724 | 1 | - | 2.72E+08 | 2.72E+08 | 5922 | exon_intron |
| novel_circ_008935 | ENSSSCG00000009042 | 8 | + | 83276933 | 83280342 | 661 | annot_exons |
| novel_circ_008936 | ENSSSCG00000006878 | 4 | + | 1.2E+08 | 1.2E+08 | 278 | annot_exons |
| novel_circ_008937 | ENSSSCG00000014044 | 2 | - | 80673386 | 80677047 | 319 | annot_exons |
| novel_circ_008938 | ENSSSCG00000028406 | 1 | - | 2.02E+08 | 2.02E+08 | 844 | annot_exons |
| novel_circ_008939 | ENSSSCG00000034395 | 15 | + | 1.11E+08 | 1.11E+08 | 549 | annot_exons |
| novel_circ_008940 | ENSSSCG00000015908 | 15 | - | 72487205 | 72488421 | 492 | annot_exons |
| novel_circ_008941 | ENSSSCG00000036610 | 13 | + | 79018336 | 79023396 | 334 | annot_exons |
| novel_circ_008942 | ENSSSCG00000003987 | 6 | + | 62322528 | 62323022 | 495 | exon_intron |
| novel_circ_008943 | ENSSSCG00000007345 | 17 | + | 41581192 | 41598567 | 795 | annot_exons |
| novel_circ_008944 | ENSSSCG00000015247 | 9 | - | 56951778 | 56951965 | 188 | one_exon |
| novel_circ_008945 | ENSSSCG00000003110 | 6 | - | 52904381 | 52906996 | 2616 | intronic |
| novel_circ_008946 | ENSSSCG00000034191 | 2 | + | 42861755 | 42864865 | 242 | annot_exons |
| novel_circ_008947 | ENSSSCG00000004624 | 1 | - | 1.2E+08 | 1.2E+08 | 1193 | one_exon |
| novel_circ_008948 | ENSSSCG00000017382 | 12 | + | 20126112 | 20129339 | 273 | annot_exons |
| novel_circ_008949 | ENSSSCG00000026536 | 11 | - | 78673497 | 78703726 | 10575 | exon_intron |
| novel_circ_008950 | ENSSSCG00000010034 | 14 | + | 48486297 | 48490935 | 631 | annot_exons |
| novel_circ_008951 | NA | 2 | - | 6750711 | 6751430 | 720 | intergenic |
| novel_circ_008952 | ENSSSCG00000004814 | 1 | + | 1.38E+08 | 1.38E+08 | 510 | annot_exons |
| novel_circ_008953 | ENSSSCG00000004857 | 1 | - | 1.46E+08 | 1.46E+08 | 3657 | annot_exons |
| novel_circ_008954 | ENSSSCG00000012249 | X | - | 36333293 | 36344150 | 613 | annot_exons |
| novel_circ_008955 | ENSSSCG00000038323 | 2 | + | 5862092 | 5862409 | 318 | antisense |
| novel_circ_008956 | ENSSSCG00000033323 | 14 | - | 92300012 | 92309354 | 374 | annot_exons |
| novel_circ_008957 | ENSSSCG00000009903 | 14 | - | 40470474 | 40473338 | 372 | annot_exons |
| novel_circ_008958 | ENSSSCG00000040383 | 1 | + | 1.28E+08 | 1.28E+08 | 538 | annot_exons |
| novel_circ_008959 | ENSSSCG00000011652 | 13 | - | 77444936 | 77463301 | 303 | annot_exons |
| novel_circ_008960 | ENSSSCG00000011013 | 10 | + | 39990396 | 39991160 | 229 | annot_exons |
| novel_circ_008961 | ENSSSCG00000011794 | 13 | + | 1.23E+08 | 1.23E+08 | 206 | exon_intron |
| novel_circ_008962 | ENSSSCG00000006872 | 4 | + | 1.18E+08 | 1.18E+08 | 173 | antisense |
| novel_circ_008963 | ENSSSCG00000016430 | 18 | - | 5319586 | 5321088 | 291 | annot_exons |
| novel_circ_008964 | ENSSSCG00000028748 | 6 | + | 1.58E+08 | 1.58E+08 | 314 | annot_exons |
| novel_circ_008965 | ENSSSCG00000029967 | 9 | - | 72395044 | 72395639 | 326 | annot_exons |
| novel_circ_008966 | ENSSSCG00000030480 | 13 | + | 2.01E+08 | 2.01E+08 | 801 | intronic |
| novel_circ_008967 | ENSSSCG00000028720 | 12 | + | 47051002 | 47054774 | 353 | annot_exons |
| novel_circ_008968 | ENSSSCG00000000466 | 5 | + | 28907687 | 28911827 | 528 | annot_exons |
| novel_circ_008969 | ENSSSCG00000008312 | 3 | - | 71177121 | 71179271 | 231 | annot_exons |
| novel_circ_008970 | ENSSSCG00000027128 | 2 | + | 73280306 | 73281077 | 328 | annot_exons |
| novel_circ_008971 | ENSSSCG00000008621 | 3 | + | 1.22E+08 | 1.22E+08 | 1201 | annot_exons |
| novel_circ_008972 | ENSSSCG00000009524 | 11 | - | 69425725 | 69436664 | 637 | annot_exons |
| novel_circ_008973 | ENSSSCG00000004856 | 1 | - | 1.46E+08 | 1.46E+08 | 184 | exon_intron |
| novel_circ_008974 | ENSSSCG00000015857 | 15 | - | 59239097 | 59250682 | 696 | annot_exons |
| novel_circ_008975 | ENSSSCG00000026465 | 3 | - | 1.11E+08 | 1.11E+08 | 254 | annot_exons |
| novel_circ_008976 | ENSSSCG00000013720 | 2 | + | 66331348 | 66334518 | 764 | annot_exons |
| novel_circ_008977 | ENSSSCG00000006981 | 17 | + | 4765508 | 4777830 | 265 | annot_exons |
| novel_circ_008978 | ENSSSCG00000009435 | 11 | - | 25545251 | 25570452 | 446 | annot_exons |
| novel_circ_008979 | ENSSSCG00000016954 | 16 | + | 44484459 | 44486773 | 316 | annot_exons |
| novel_circ_008980 | NA | 15 | - | 84463740 | 84466974 | 3235 | intergenic |
| novel_circ_008981 | ENSSSCG00000022112 | 11 | - | 67868220 | 67876147 | 749 | annot_exons |
| novel_circ_008982 | ENSSSCG00000002259 | 7 | + | 88148989 | 88154611 | 2166 | exon_intron |
| novel_circ_008983 | ENSSSCG00000003870 | 6 | + | 1.6E+08 | 1.6E+08 | 3174 | annot_exons |
| novel_circ_008984 | ENSSSCG00000016894 | 16 | - | 33420653 | 33420773 | 121 | intronic |
| novel_circ_008985 | ENSSSCG00000009113 | 8 | - | 1.05E+08 | 1.05E+08 | 346 | annot_exons |
| novel_circ_008986 | ENSSSCG00000012131 | X | + | 11454561 | 11455528 | 290 | annot_exons |
| novel_circ_008987 | NA | 10 | - | 17467676 | 17468635 | 960 | intergenic |
| novel_circ_008988 | ENSSSCG00000009405 | 11 | + | 19623259 | 19645083 | 712 | annot_exons |
| novel_circ_008989 | ENSSSCG00000027444 | 17 | - | 50821910 | 50844309 | 404 | annot_exons |
| novel_circ_008990 | ENSSSCG00000008821 | 8 | + | 38273589 | 38280012 | 314 | annot_exons |
| novel_circ_008991 | ENSSSCG00000016409 | 18 | - | 1560744 | 1561079 | 256 | annot_exons |
| novel_circ_008992 | ENSSSCG00000005423 | 1 | - | 2.46E+08 | 2.46E+08 | 524 | annot_exons |
| novel_circ_008993 | ENSSSCG00000035676 | 16 | - | 71262565 | 71266608 | 369 | annot_exons |
| novel_circ_008994 | ENSSSCG00000039085 | 13 | - | 2.07E+08 | 2.07E+08 | 228 | antisense |
| novel_circ_008995 | ENSSSCG00000010460 | 14 | + | 1.03E+08 | 1.03E+08 | 553 | annot_exons |
| novel_circ_008996 | ENSSSCG00000016512 | 18 | + | 10562054 | 10565214 | 223 | annot_exons |
| novel_circ_008997 | ENSSSCG00000003667 | 6 | - | 95652504 | 95657337 | 388 | annot_exons |
| novel_circ_008998 | ENSSSCG00000008478 | 3 | + | 1.01E+08 | 1.01E+08 | 861 | annot_exons |
| novel_circ_008999 | ENSSSCG00000033497 | 6 | - | 69106984 | 69124265 | 308 | annot_exons |
| novel_circ_009000 | ENSSSCG00000017277 | 12 | + | 14588252 | 14590571 | 554 | annot_exons |
| novel_circ_009001 | ENSSSCG00000025941 | 1 | + | 45173524 | 45175282 | 643 | exon_intron |
| novel_circ_009002 | ENSSSCG00000002629 | 7 | + | 46735628 | 46736108 | 481 | intronic |
| novel_circ_009003 | ENSSSCG00000016763 | 18 | + | 52599764 | 52628187 | 1024 | annot_exons |
| novel_circ_009004 | ENSSSCG00000000466 | 5 | + | 28864431 | 28864854 | 233 | annot_exons |
| novel_circ_009005 | ENSSSCG00000004275 | 1 | + | 50650950 | 50691798 | 667 | annot_exons |
| novel_circ_009006 | ENSSSCG00000008310 | 3 | + | 70184898 | 70205790 | 246 | annot_exons |
| novel_circ_009007 | ENSSSCG00000008153 | 3 | - | 51563791 | 51564671 | 421 | annot_exons |
| novel_circ_009008 | ENSSSCG00000013094 | 2 | + | 10186250 | 10186530 | 185 | annot_exons |
| novel_circ_009009 | ENSSSCG00000011163 | 10 | + | 68959424 | 68961182 | 312 | annot_exons |
| novel_circ_009010 | ENSSSCG00000027293 | 16 | + | 49139185 | 49143198 | 318 | annot_exons |
| novel_circ_009011 | ENSSSCG00000003895 | 6 | + | 1.65E+08 | 1.65E+08 | 238 | annot_exons |
| novel_circ_009012 | ENSSSCG00000014946 | 9 | + | 26143219 | 26148093 | 360 | annot_exons |
| novel_circ_009013 | ENSSSCG00000011137 | 10 | + | 65174467 | 65179209 | 436 | annot_exons |
| novel_circ_009014 | ENSSSCG00000014838 | 9 | - | 8707786 | 8709627 | 482 | annot_exons |
| novel_circ_009015 | ENSSSCG00000021941 | 3 | + | 7928957 | 7929871 | 672 | annot_exons |
| novel_circ_009016 | ENSSSCG00000015537 | 9 | + | 1.22E+08 | 1.22E+08 | 234 | annot_exons |
| novel_circ_009017 | ENSSSCG00000017382 | 12 | + | 20126112 | 20134694 | 782 | annot_exons |
| novel_circ_009018 | ENSSSCG00000015368 | 9 | + | 87829337 | 87840517 | 11181 | exon_intron |
| novel_circ_009019 | ENSSSCG00000016948 | 16 | - | 44117112 | 44139462 | 1098 | annot_exons |
| novel_circ_009020 | ENSSSCG00000032165 | 14 | - | 1.14E+08 | 1.14E+08 | 189 | annot_exons |
| novel_circ_009021 | ENSSSCG00000027459 | 13 | + | 90098805 | 90137838 | 4940 | exon_intron |
| novel_circ_009022 | ENSSSCG00000007280 | 17 | + | 37900616 | 37912170 | 396 | annot_exons |
| novel_circ_009023 | ENSSSCG00000043799 | 8 | + | 40780414 | 40790792 | 10379 | exon_intron |
| novel_circ_009024 | ENSSSCG00000003812 | 6 | - | 1.49E+08 | 1.49E+08 | 898 | annot_exons |
| novel_circ_009025 | ENSSSCG00000003627 | 6 | + | 91566041 | 91570358 | 264 | annot_exons |
| novel_circ_009026 | NA | 15 | + | 84272652 | 84273183 | 532 | intergenic |
| novel_circ_009027 | ENSSSCG00000032176 | 12 | + | 14475484 | 14479879 | 432 | annot_exons |
| novel_circ_009028 | ENSSSCG00000015592 | 9 | - | 1.31E+08 | 1.31E+08 | 194 | annot_exons |
| novel_circ_009029 | NA | 7 | + | 68218968 | 68230272 | 11305 | intergenic |
| novel_circ_009030 | ENSSSCG00000004207 | 1 | - | 34023744 | 34096613 | 793 | annot_exons |
| novel_circ_009031 | ENSSSCG00000004508 | 1 | - | 99000897 | 99001088 | 192 | one_exon |
| novel_circ_009032 | ENSSSCG00000011064 | 10 | - | 48911448 | 48916899 | 309 | annot_exons |
| novel_circ_009033 | ENSSSCG00000014136 | 2 | + | 91384391 | 91391360 | 325 | annot_exons |
| novel_circ_009034 | ENSSSCG00000011342 | 13 | + | 30624651 | 30624952 | 302 | one_exon |
| novel_circ_009035 | ENSSSCG00000003511 | 6 | - | 78990898 | 78994000 | 380 | annot_exons |
| novel_circ_009036 | ENSSSCG00000039085 | 13 | + | 2.07E+08 | 2.07E+08 | 346 | annot_exons |
| novel_circ_009037 | NA | 9 | + | 713575 | 737189 | 23615 | intergenic |
| novel_circ_009038 | ENSSSCG00000012699 | X | - | 1.11E+08 | 1.11E+08 | 296 | antisense |
| novel_circ_009039 | ENSSSCG00000010239 | 14 | - | 71507008 | 71513016 | 274 | annot_exons |
| novel_circ_009040 | ENSSSCG00000000488 | 5 | + | 33164482 | 33173496 | 980 | annot_exons |
| novel_circ_009041 | ENSSSCG00000015023 | 9 | - | 39547691 | 39556507 | 8817 | exon_intron |
| novel_circ_009042 | ENSSSCG00000038801 | 8 | - | 1.16E+08 | 1.16E+08 | 1093 | exon_intron |
| novel_circ_009043 | ENSSSCG00000008357 | 3 | + | 76615866 | 76625140 | 347 | annot_exons |
| novel_circ_009044 | ENSSSCG00000008826 | 8 | - | 38512887 | 38521829 | 447 | annot_exons |
| novel_circ_009045 | ENSSSCG00000005787 | 1 | - | 1.46E+08 | 1.46E+08 | 409 | annot_exons |
| novel_circ_009046 | ENSSSCG00000042430 | 15 | - | 84424099 | 84424324 | 226 | one_exon |
| novel_circ_009047 | ENSSSCG00000009101 | 8 | + | 1.03E+08 | 1.03E+08 | 370 | annot_exons |
| novel_circ_009048 | ENSSSCG00000043353 | 6 | - | 11998869 | 12014334 | 15315 | exon_intron |
| novel_circ_009049 | ENSSSCG00000007874 | 3 | - | 28415454 | 28417579 | 235 | annot_exons |
| novel_circ_009050 | ENSSSCG00000035136 | 10 | + | 16222141 | 16239910 | 395 | annot_exons |
| novel_circ_009051 | ENSSSCG00000006024 | 4 | - | 27991818 | 28001951 | 537 | annot_exons |
| novel_circ_009052 | ENSSSCG00000012771 | X | - | 1.24E+08 | 1.24E+08 | 381 | antisense |
| novel_circ_009053 | ENSSSCG00000013400 | 2 | - | 47170673 | 47171374 | 307 | annot_exons |
| novel_circ_009054 | ENSSSCG00000030460 | X | - | 1.12E+08 | 1.12E+08 | 346 | annot_exons |
| novel_circ_009055 | ENSSSCG00000015492 | 9 | + | 1.16E+08 | 1.16E+08 | 198 | annot_exons |
| novel_circ_009056 | ENSSSCG00000011970 | 13 | - | 1.59E+08 | 1.59E+08 | 246 | exon_intron |
| novel_circ_009057 | ENSSSCG00000005041 | 1 | - | 1.83E+08 | 1.83E+08 | 310 | annot_exons |
| novel_circ_009058 | ENSSSCG00000014399 | 2 | + | 1.44E+08 | 1.44E+08 | 431 | annot_exons |
| novel_circ_009059 | ENSSSCG00000017191 | 12 | + | 5374046 | 5381452 | 586 | annot_exons |
| novel_circ_009060 | ENSSSCG00000011023 | 10 | - | 41675568 | 41730537 | 407 | annot_exons |
| novel_circ_009061 | ENSSSCG00000022955 | 8 | + | 56244215 | 56246887 | 484 | annot_exons |
| novel_circ_009062 | ENSSSCG00000034569 | 2 | + | 51480990 | 51482241 | 255 | annot_exons |
| novel_circ_009063 | NA | 10 | + | 13202491 | 13227709 | 25219 | intergenic |
| novel_circ_009064 | ENSSSCG00000026034 | 7 | - | 13440411 | 13444653 | 246 | annot_exons |
| novel_circ_009065 | ENSSSCG00000014989 | 9 | - | 33683434 | 33683734 | 214 | annot_exons |
| novel_circ_009066 | ENSSSCG00000040977 | 3 | + | 1591745 | 1597198 | 5454 | antisense |
| novel_circ_009067 | ENSSSCG00000004934 | 1 | - | 1.63E+08 | 1.63E+08 | 501 | annot_exons |
| novel_circ_009068 | ENSSSCG00000013554 | 2 | + | 72408934 | 72409483 | 550 | antisense |
| novel_circ_009069 | ENSSSCG00000017282 | 12 | - | 15045792 | 15046265 | 474 | antisense |
| novel_circ_009070 | ENSSSCG00000000075 | 5 | + | 7808693 | 7853188 | 44496 | exon_intron |
| novel_circ_009071 | ENSSSCG00000004293 | 1 | - | 54465940 | 54469544 | 409 | annot_exons |
| novel_circ_009072 | ENSSSCG00000015310 | 9 | - | 72001214 | 72001696 | 483 | antisense |
| novel_circ_009073 | ENSSSCG00000001641 | 7 | + | 37725682 | 37730969 | 486 | annot_exons |
| novel_circ_009074 | ENSSSCG00000010246 | 14 | + | 72019478 | 72021445 | 282 | annot_exons |
| novel_circ_009075 | ENSSSCG00000015790 | 15 | - | 46197493 | 46197842 | 350 | antisense |
| novel_circ_009076 | ENSSSCG00000013992 | 2 | - | 51236405 | 51236973 | 569 | antisense |
| novel_circ_009077 | ENSSSCG00000017764 | 12 | - | 44858687 | 44859112 | 332 | annot_exons |
| novel_circ_009078 | ENSSSCG00000005524 | 1 | + | 2.62E+08 | 2.62E+08 | 149 | one_exon |
| novel_circ_009079 | ENSSSCG00000026113 | 13 | + | 1.46E+08 | 1.46E+08 | 1605 | one_exon |
| novel_circ_009080 | ENSSSCG00000050344 | 2 | - | 1.31E+08 | 1.31E+08 | 35209 | exon_intron |
| novel_circ_009081 | ENSSSCG00000004636 | 1 | + | 1.21E+08 | 1.21E+08 | 544 | annot_exons |
| novel_circ_009082 | ENSSSCG00000021200 | X | - | 58378203 | 58384217 | 300 | annot_exons |
| novel_circ_009083 | ENSSSCG00000013402 | 2 | - | 47475559 | 47475766 | 208 | one_exon |
| novel_circ_009084 | ENSSSCG00000004856 | 1 | - | 1.46E+08 | 1.46E+08 | 706 | annot_exons |
| novel_circ_009085 | ENSSSCG00000036705 | 12 | + | 47467180 | 47470959 | 3780 | exon_intron |
| novel_circ_009086 | ENSSSCG00000012063 | 13 | - | 2.01E+08 | 2.01E+08 | 612 | annot_exons |
| novel_circ_009087 | ENSSSCG00000006016 | 4 | + | 22038996 | 22042122 | 418 | annot_exons |
| novel_circ_009088 | NA | 15 | - | 84269116 | 84269665 | 550 | intergenic |
| novel_circ_009089 | ENSSSCG00000015940 | 15 | + | 76248140 | 76266620 | 1004 | annot_exons |
| novel_circ_009090 | ENSSSCG00000010151 | 14 | + | 55583796 | 55588339 | 398 | annot_exons |
| novel_circ_009091 | NA | 15 | - | 84354981 | 84356302 | 1322 | intergenic |
| novel_circ_009092 | NA | 15 | - | 84268729 | 84269459 | 731 | intergenic |
| novel_circ_009093 | ENSSSCG00000017624 | 12 | - | 34167390 | 34167959 | 570 | one_exon |
| novel_circ_009094 | ENSSSCG00000009029 | 8 | - | 80706324 | 80742382 | 311 | annot_exons |
| novel_circ_009095 | ENSSSCG00000004729 | 1 | - | 1.29E+08 | 1.29E+08 | 311 | annot_exons |
| novel_circ_009096 | ENSSSCG00000012059 | 13 | - | 2.01E+08 | 2.01E+08 | 3242 | exon_intron |
| novel_circ_009097 | ENSSSCG00000010652 | 14 | + | 1.25E+08 | 1.25E+08 | 249 | annot_exons |
| novel_circ_009098 | ENSSSCG00000005425 | 1 | + | 2.47E+08 | 2.47E+08 | 407 | annot_exons |
| novel_circ_009099 | ENSSSCG00000014170 | 2 | + | 1.03E+08 | 1.03E+08 | 300 | exon_intron |
| novel_circ_009100 | ENSSSCG00000005082 | 1 | + | 1.89E+08 | 1.89E+08 | 1224 | annot_exons |
| novel_circ_009101 | ENSSSCG00000012181 | X | + | 20588035 | 20601957 | 7137 | annot_exons |
| novel_circ_009102 | ENSSSCG00000011858 | 13 | + | 1.35E+08 | 1.35E+08 | 349 | exon_intron |
| novel_circ_009103 | ENSSSCG00000010190 | 14 | - | 60415441 | 60415651 | 211 | antisense |
| novel_circ_009104 | ENSSSCG00000038008 | 2 | - | 5782769 | 5784337 | 616 | annot_exons |
| novel_circ_009105 | ENSSSCG00000015694 | 15 | + | 16443887 | 16447152 | 297 | annot_exons |
| novel_circ_009106 | ENSSSCG00000005126 | 1 | + | 1.96E+08 | 1.96E+08 | 692 | annot_exons |
| novel_circ_009107 | ENSSSCG00000031302 | 8 | - | 479744 | 479950 | 207 | one_exon |
| novel_circ_009108 | ENSSSCG00000017748 | 12 | - | 43595390 | 43611935 | 861 | annot_exons |
| novel_circ_009109 | ENSSSCG00000027667 | 7 | + | 1.21E+08 | 1.21E+08 | 511 | annot_exons |
| novel_circ_009110 | ENSSSCG00000016954 | 16 | + | 44454933 | 44466793 | 287 | annot_exons |
| novel_circ_009111 | ENSSSCG00000000064 | 5 | - | 7034375 | 7040783 | 396 | annot_exons |
| novel_circ_009112 | ENSSSCG00000003712 | 6 | - | 1.09E+08 | 1.09E+08 | 674 | annot_exons |
| novel_circ_009113 | ENSSSCG00000013262 | 2 | + | 15968765 | 15970677 | 416 | annot_exons |
| novel_circ_009114 | ENSSSCG00000040595 | 14 | - | 32837395 | 32839410 | 361 | annot_exons |
| novel_circ_009115 | ENSSSCG00000016397 | 15 | + | 608519 | 610257 | 213 | annot_exons |
| novel_circ_009116 | ENSSSCG00000008225 | 3 | - | 58646042 | 58647018 | 196 | annot_exons |
| novel_circ_009117 | ENSSSCG00000022559 | 6 | + | 1.06E+08 | 1.06E+08 | 467 | antisense |
| novel_circ_009118 | ENSSSCG00000001485 | 7 | + | 26689928 | 26690106 | 179 | antisense |
| novel_circ_009119 | ENSSSCG00000010325 | 14 | - | 79362504 | 79364310 | 1807 | exon_intron |
| novel_circ_009120 | ENSSSCG00000011619 | 13 | - | 72116572 | 72128165 | 470 | annot_exons |
| novel_circ_009121 | ENSSSCG00000011672 | 13 | + | 82318869 | 82322181 | 306 | annot_exons |
| novel_circ_009122 | ENSSSCG00000036855 | 13 | + | 81767591 | 81784515 | 6000 | annot_exons |
| novel_circ_009123 | ENSSSCG00000004479 | 1 | + | 90521051 | 90570316 | 640 | annot_exons |
| novel_circ_009124 | ENSSSCG00000005015 | 1 | - | 1.8E+08 | 1.8E+08 | 223 | one_exon |
| novel_circ_009125 | ENSSSCG00000030480 | 13 | + | 2.01E+08 | 2.01E+08 | 11102 | exon_intron |
| novel_circ_009126 | ENSSSCG00000008919 | 8 | - | 63369134 | 63384107 | 391 | annot_exons |
| novel_circ_009127 | ENSSSCG00000008332 | 3 | + | 72300370 | 72304536 | 216 | annot_exons |
| novel_circ_009128 | ENSSSCG00000034191 | 2 | + | 42498291 | 42573733 | 55678 | exon_intron |
| novel_circ_009129 | ENSSSCG00000011772 | 13 | + | 1.21E+08 | 1.21E+08 | 336 | annot_exons |
| novel_circ_009130 | ENSSSCG00000014044 | 2 | - | 80698194 | 80703369 | 387 | annot_exons |
| novel_circ_009131 | ENSSSCG00000011720 | 13 | + | 92562600 | 92563197 | 598 | intronic |
| novel_circ_009132 | ENSSSCG00000029311 | 14 | - | 71346414 | 71346886 | 473 | antisense |
| novel_circ_009133 | ENSSSCG00000009946 | 14 | + | 42234964 | 42239167 | 417 | annot_exons |
| novel_circ_009134 | ENSSSCG00000028925 | 12 | - | 14567916 | 14569779 | 237 | annot_exons |
| novel_circ_009135 | ENSSSCG00000012062 | 13 | + | 2.01E+08 | 2.01E+08 | 414 | annot_exons |
| novel_circ_009136 | ENSSSCG00000013602 | 2 | - | 70823669 | 70825581 | 1913 | intronic |
| novel_circ_009137 | ENSSSCG00000028201 | 9 | - | 36014214 | 36016257 | 187 | annot_exons |
| novel_circ_009138 | ENSSSCG00000014304 | 2 | + | 1.37E+08 | 1.37E+08 | 249 | annot_exons |
| novel_circ_009139 | ENSSSCG00000015747 | 15 | + | 33497474 | 33498920 | 1447 | intronic |
| novel_circ_009140 | ENSSSCG00000000078 | 5 | - | 8120823 | 8121164 | 342 | one_exon |
| novel_circ_009141 | ENSSSCG00000022288 | 5 | - | 46515940 | 46521211 | 200 | annot_exons |
| novel_circ_009142 | ENSSSCG00000017678 | 12 | + | 37636936 | 37657745 | 534 | annot_exons |
| novel_circ_009143 | ENSSSCG00000021606 | 7 | + | 98745664 | 98748964 | 283 | annot_exons |
| novel_circ_009144 | ENSSSCG00000039473 | 12 | + | 37856184 | 37892283 | 2642 | annot_exons |
| novel_circ_009145 | ENSSSCG00000004110 | 1 | - | 16576987 | 16578141 | 1155 | intronic |
| novel_circ_009146 | ENSSSCG00000014878 | 9 | - | 12097454 | 12106836 | 611 | annot_exons |
| novel_circ_009147 | ENSSSCG00000015859 | 15 | + | 59341631 | 59361531 | 745 | annot_exons |
| novel_circ_009148 | ENSSSCG00000011373 | 13 | - | 31699086 | 31701284 | 410 | annot_exons |
| novel_circ_009149 | ENSSSCG00000015283 | 9 | - | 65111378 | 65112705 | 319 | annot_exons |
| novel_circ_009150 | ENSSSCG00000021285 | 3 | - | 4264224 | 4277544 | 420 | annot_exons |
| novel_circ_009151 | ENSSSCG00000016397 | 15 | + | 621185 | 632268 | 1282 | annot_exons |
| novel_circ_009152 | ENSSSCG00000037381 | 13 | + | 2.03E+08 | 2.03E+08 | 349 | annot_exons |
| novel_circ_009153 | ENSSSCG00000009390 | 11 | + | 17870039 | 17870267 | 229 | one_exon |
| novel_circ_009154 | ENSSSCG00000003747 | 6 | + | 1.21E+08 | 1.21E+08 | 674 | one_exon |
| novel_circ_009155 | ENSSSCG00000011025 | 10 | + | 42147410 | 42147610 | 201 | one_exon |
| novel_circ_009156 | ENSSSCG00000008300 | 3 | - | 69158854 | 69186281 | 1481 | annot_exons |
| novel_circ_009157 | ENSSSCG00000009584 | 14 | - | 967932 | 971780 | 2782 | annot_exons |
| novel_circ_009158 | ENSSSCG00000039714 | 7 | - | 62587195 | 62603890 | 16696 | exon_intron |
| novel_circ_009159 | ENSSSCG00000010640 | 14 | - | 1.24E+08 | 1.24E+08 | 317 | intronic |
| novel_circ_009160 | ENSSSCG00000001499 | 7 | + | 29238238 | 29238641 | 228 | annot_exons |
| novel_circ_009161 | NA | 15 | - | 84354981 | 84365218 | 10238 | intergenic |
| novel_circ_009162 | ENSSSCG00000021170 | 2 | - | 45538589 | 45577171 | 1564 | annot_exons |
| novel_circ_009163 | ENSSSCG00000038397 | 4 | + | 2771954 | 2774992 | 143 | annot_exons |
| novel_circ_009164 | ENSSSCG00000007345 | 17 | + | 41535240 | 41539729 | 483 | annot_exons |
| novel_circ_009165 | ENSSSCG00000035478 | 3 | - | 58048643 | 58049988 | 333 | annot_exons |
| novel_circ_009166 | ENSSSCG00000014440 | 2 | + | 1.51E+08 | 1.51E+08 | 679 | annot_exons |
| novel_circ_009167 | ENSSSCG00000010288 | 14 | - | 75288977 | 75295452 | 281 | annot_exons |
| novel_circ_009168 | ENSSSCG00000004318 | 1 | - | 57392229 | 57417726 | 721 | annot_exons |
| novel_circ_009169 | ENSSSCG00000026806 | 9 | + | 1.24E+08 | 1.24E+08 | 1166 | annot_exons |
| novel_circ_009170 | ENSSSCG00000040013 | 17 | - | 5368469 | 5410340 | 791 | annot_exons |
| novel_circ_009171 | ENSSSCG00000025606 | 9 | + | 36707534 | 36715744 | 1083 | annot_exons |
| novel_circ_009172 | NA | 15 | - | 84271692 | 84272093 | 402 | intergenic |
| novel_circ_009173 | ENSSSCG00000008368 | 3 | - | 78003035 | 78014790 | 10870 | exon_intron |
| novel_circ_009174 | ENSSSCG00000011194 | 13 | - | 2958761 | 2964480 | 331 | annot_exons |
| novel_circ_009175 | ENSSSCG00000006274 | 4 | + | 79822366 | 79845802 | 2962 | annot_exons |
| novel_circ_009176 | ENSSSCG00000049463 | 3 | - | 1.01E+08 | 1.01E+08 | 3779 | antisense |
| novel_circ_009177 | ENSSSCG00000009096 | 8 | - | 1.02E+08 | 1.02E+08 | 361 | annot_exons |
| novel_circ_009178 | ENSSSCG00000003712 | 6 | - | 1.09E+08 | 1.09E+08 | 1017 | annot_exons |
| novel_circ_009179 | ENSSSCG00000037238 | 12 | - | 18622295 | 18625068 | 341 | annot_exons |
| novel_circ_009180 | ENSSSCG00000001641 | 7 | + | 37709643 | 37712125 | 229 | annot_exons |
| novel_circ_009181 | ENSSSCG00000002822 | 6 | + | 29382431 | 29384324 | 318 | annot_exons |
| novel_circ_009182 | ENSSSCG00000008150 | 3 | - | 51456411 | 51492113 | 337 | annot_exons |
| novel_circ_009183 | ENSSSCG00000003809 | 6 | - | 1.48E+08 | 1.48E+08 | 293 | antisense |
| novel_circ_009184 | ENSSSCG00000025156 | X | - | 64330107 | 64346816 | 956 | annot_exons |
| novel_circ_009185 | ENSSSCG00000026914 | 2 | + | 8427300 | 8430413 | 372 | annot_exons |
| novel_circ_009186 | ENSSSCG00000008111 | 3 | - | 46333828 | 46341322 | 446 | annot_exons |
| novel_circ_009187 | ENSSSCG00000030535 | 1 | - | 9167471 | 9169598 | 317 | annot_exons |
| novel_circ_009188 | ENSSSCG00000002440 | 7 | - | 1.13E+08 | 1.13E+08 | 478 | annot_exons |
| novel_circ_009189 | ENSSSCG00000017505 | 12 | + | 22854805 | 22856035 | 164 | annot_exons |
| novel_circ_009190 | NA | 2 | - | 1.34E+08 | 1.34E+08 | 1582 | intergenic |
| novel_circ_009191 | ENSSSCG00000004123 | 1 | - | 20782263 | 20784622 | 333 | annot_exons |
| novel_circ_009192 | ENSSSCG00000006390 | 4 | - | 90276419 | 90278977 | 1040 | exon_intron |
| novel_circ_009193 | ENSSSCG00000029125 | 2 | - | 1.4E+08 | 1.4E+08 | 574 | annot_exons |
| novel_circ_009194 | ENSSSCG00000023771 | 11 | - | 3953585 | 3960189 | 413 | annot_exons |
| novel_circ_009195 | ENSSSCG00000016845 | 16 | - | 22557193 | 22576391 | 1288 | annot_exons |
| novel_circ_009196 | ENSSSCG00000000884 | 5 | - | 85218615 | 85236388 | 1223 | annot_exons |
| novel_circ_009197 | ENSSSCG00000005105 | 1 | + | 1.91E+08 | 1.91E+08 | 187 | one_exon |
| novel_circ_009198 | ENSSSCG00000002844 | 6 | - | 37151567 | 37166517 | 518 | annot_exons |
| novel_circ_009199 | ENSSSCG00000013249 | 2 | + | 15752898 | 15756374 | 558 | annot_exons |
| novel_circ_009200 | ENSSSCG00000004169 | 1 | + | 29027848 | 29042644 | 874 | annot_exons |
| novel_circ_009201 | ENSSSCG00000003693 | 6 | + | 1.04E+08 | 1.04E+08 | 304 | annot_exons |
| novel_circ_009202 | ENSSSCG00000013905 | 2 | + | 59261733 | 59262716 | 391 | annot_exons |
| novel_circ_009203 | ENSSSCG00000004499 | 1 | - | 95743460 | 95748901 | 739 | annot_exons |
| novel_circ_009204 | ENSSSCG00000010494 | 14 | - | 1.07E+08 | 1.07E+08 | 1031 | annot_exons |
| novel_circ_009205 | ENSSSCG00000029331 | 14 | - | 20794797 | 20802914 | 8118 | intronic |
| novel_circ_009206 | ENSSSCG00000035410 | 15 | - | 59875540 | 59875827 | 288 | antisense |
| novel_circ_009207 | ENSSSCG00000025316 | 13 | - | 95702963 | 95709483 | 494 | annot_exons |
| novel_circ_009208 | ENSSSCG00000022331 | X | - | 1.14E+08 | 1.14E+08 | 187 | intronic |
| novel_circ_009209 | ENSSSCG00000017791 | 12 | - | 45856452 | 45913679 | 40235 | exon_intron |
| novel_circ_009210 | ENSSSCG00000010190 | 14 | - | 60414513 | 60414916 | 404 | antisense |
| novel_circ_009211 | ENSSSCG00000050244 | 12 | + | 59333970 | 59335643 | 1674 | exon_intron |
| novel_circ_009212 | ENSSSCG00000029169 | 1 | - | 2.66E+08 | 2.66E+08 | 680 | annot_exons |
| novel_circ_009213 | ENSSSCG00000010360 | 14 | + | 87822731 | 87861103 | 504 | annot_exons |
| novel_circ_009214 | ENSSSCG00000010399 | 14 | - | 90510793 | 90511831 | 237 | annot_exons |
| novel_circ_009215 | ENSSSCG00000014014 | 2 | + | 78620475 | 78622364 | 302 | annot_exons |
| novel_circ_009216 | ENSSSCG00000030467 | 13 | - | 1.09E+08 | 1.09E+08 | 1482 | annot_exons |
| novel_circ_009217 | ENSSSCG00000026559 | 3 | - | 86297988 | 86298417 | 329 | annot_exons |
| novel_circ_009218 | ENSSSCG00000001903 | 7 | + | 58850363 | 58853955 | 531 | annot_exons |
| novel_circ_009219 | ENSSSCG00000011854 | 13 | + | 1.35E+08 | 1.35E+08 | 257 | annot_exons |
| novel_circ_009220 | ENSSSCG00000000910 | 5 | - | 89285477 | 89301915 | 590 | annot_exons |
| novel_circ_009221 | ENSSSCG00000002844 | 6 | - | 37077213 | 37099195 | 684 | annot_exons |
| novel_circ_009222 | ENSSSCG00000005367 | 1 | - | 2.39E+08 | 2.39E+08 | 242 | one_exon |
| novel_circ_009223 | ENSSSCG00000014014 | 2 | + | 78573441 | 78573691 | 251 | one_exon |
| novel_circ_009224 | ENSSSCG00000007043 | 17 | - | 14446212 | 14449910 | 528 | annot_exons |
| novel_circ_009225 | ENSSSCG00000004979 | 1 | - | 1.69E+08 | 1.69E+08 | 480 | annot_exons |
| novel_circ_009226 | ENSSSCG00000030420 | 6 | + | 13132378 | 13134026 | 224 | annot_exons |
| novel_circ_009227 | ENSSSCG00000022895 | 13 | - | 1.62E+08 | 1.62E+08 | 1092 | annot_exons |
| novel_circ_009228 | ENSSSCG00000025682 | 5 | - | 83923357 | 83925966 | 324 | annot_exons |
| novel_circ_009229 | ENSSSCG00000035045 | 14 | + | 1.36E+08 | 1.36E+08 | 1523 | intronic |
| novel_circ_009230 | ENSSSCG00000010429 | 14 | - | 98567541 | 98567872 | 332 | intronic |
| novel_circ_009231 | ENSSSCG00000026239 | 6 | + | 37967643 | 37968385 | 364 | annot_exons |
| novel_circ_009232 | NA | 2 | - | 1.31E+08 | 1.31E+08 | 11669 | intergenic |
| novel_circ_009233 | ENSSSCG00000007917 | 3 | - | 35008565 | 35070439 | 329 | annot_exons |
| novel_circ_009234 | ENSSSCG00000016720 | 18 | + | 48710846 | 48711251 | 295 | annot_exons |
| novel_circ_009235 | ENSSSCG00000031627 | 11 | - | 25755742 | 25761445 | 485 | annot_exons |
| novel_circ_009236 | ENSSSCG00000011101 | 10 | - | 56098270 | 56111566 | 547 | annot_exons |
| novel_circ_009237 | ENSSSCG00000009466 | 11 | + | 47845080 | 47851849 | 420 | annot_exons |
| novel_circ_009238 | ENSSSCG00000006490 | 4 | + | 93811039 | 93812799 | 1761 | intronic |
| novel_circ_009239 | ENSSSCG00000009029 | 8 | - | 80742232 | 80776468 | 34237 | exon_intron |
| novel_circ_009240 | ENSSSCG00000008641 | 3 | + | 1.27E+08 | 1.27E+08 | 741 | annot_exons |
| novel_circ_009241 | ENSSSCG00000044227 | 7 | - | 56256735 | 56261229 | 2877 | exon_intron |
| novel_circ_009242 | NA | 18 | - | 2578336 | 2580824 | 2489 | intergenic |
| novel_circ_009243 | ENSSSCG00000003731 | 6 | - | 1.16E+08 | 1.16E+08 | 24222 | exon_intron |
| novel_circ_009244 | ENSSSCG00000046928 | 11 | - | 22478085 | 22489969 | 11885 | antisense |
| novel_circ_009245 | ENSSSCG00000029464 | 5 | - | 67681766 | 67690214 | 712 | annot_exons |
| novel_circ_009246 | ENSSSCG00000011332 | 13 | - | 29944690 | 29960782 | 682 | annot_exons |
| novel_circ_009247 | ENSSSCG00000000795 | 5 | - | 74569862 | 74595084 | 2698 | annot_exons |
| novel_circ_009248 | ENSSSCG00000013276 | 2 | + | 17223490 | 17223690 | 201 | antisense |
| novel_circ_009249 | ENSSSCG00000003627 | 6 | + | 91537803 | 91540596 | 758 | annot_exons |
| novel_circ_009250 | ENSSSCG00000009887 | 14 | + | 39617312 | 39623804 | 344 | annot_exons |
| novel_circ_009251 | ENSSSCG00000006328 | 4 | + | 85338214 | 85349852 | 802 | annot_exons |
| novel_circ_009252 | ENSSSCG00000004983 | 1 | + | 1.7E+08 | 1.7E+08 | 138 | annot_exons |
| novel_circ_009253 | ENSSSCG00000023956 | 7 | - | 8027684 | 8028289 | 606 | exon_intron |
| novel_circ_009254 | ENSSSCG00000006779 | 4 | + | 1.08E+08 | 1.08E+08 | 331 | annot_exons |
| novel_circ_009255 | ENSSSCG00000033228 | 12 | - | 27274748 | 27275982 | 308 | annot_exons |
| novel_circ_009256 | ENSSSCG00000016518 | 18 | - | 11076502 | 11099194 | 940 | annot_exons |
| novel_circ_009257 | ENSSSCG00000011236 | 13 | - | 19324136 | 19327640 | 256 | annot_exons |
| novel_circ_009258 | ENSSSCG00000036339 | 15 | - | 1.05E+08 | 1.05E+08 | 377 | annot_exons |
| novel_circ_009259 | ENSSSCG00000011041 | 10 | - | 44934036 | 44935556 | 264 | annot_exons |
| novel_circ_009260 | ENSSSCG00000015247 | 9 | + | 56953732 | 56953956 | 225 | antisense |
| novel_circ_009261 | ENSSSCG00000029602 | 10 | - | 68491925 | 68507352 | 845 | annot_exons |
| novel_circ_009262 | NA | 15 | + | 84310239 | 84310538 | 300 | intergenic |
| novel_circ_009263 | ENSSSCG00000000862 | 5 | + | 82416126 | 82422198 | 406 | annot_exons |
| novel_circ_009264 | ENSSSCG00000011811 | 13 | - | 1.26E+08 | 1.26E+08 | 419 | antisense |
| novel_circ_009265 | ENSSSCG00000005602 | 1 | + | 2.66E+08 | 2.66E+08 | 1341 | annot_exons |
| novel_circ_009266 | ENSSSCG00000007153 | 17 | - | 32134467 | 32159495 | 2072 | annot_exons |
| novel_circ_009267 | ENSSSCG00000028516 | 5 | + | 16315891 | 16333246 | 802 | annot_exons |
| novel_circ_009268 | ENSSSCG00000016692 | 18 | - | 44885414 | 44890608 | 347 | annot_exons |
| novel_circ_009269 | ENSSSCG00000029602 | 10 | - | 68491183 | 68499831 | 630 | annot_exons |
| novel_circ_009270 | ENSSSCG00000016916 | 16 | - | 35113222 | 35117931 | 867 | annot_exons |
| novel_circ_009271 | ENSSSCG00000025942 | 13 | - | 1.21E+08 | 1.21E+08 | 300 | annot_exons |
| novel_circ_009272 | ENSSSCG00000005232 | 1 | - | 2.2E+08 | 2.2E+08 | 363 | exon_intron |
| novel_circ_009273 | ENSSSCG00000003154 | 6 | - | 54245024 | 54245209 | 186 | one_exon |
| novel_circ_009274 | ENSSSCG00000007590 | 3 | + | 5123860 | 5128740 | 550 | annot_exons |
| novel_circ_009275 | ENSSSCG00000017673 | 12 | + | 36450740 | 36456220 | 313 | annot_exons |
| novel_circ_009276 | ENSSSCG00000003401 | 6 | + | 70630562 | 70631434 | 175 | annot_exons |
| novel_circ_009277 | ENSSSCG00000006252 | 4 | + | 75979721 | 75990474 | 4186 | annot_exons |
| novel_circ_009278 | ENSSSCG00000025221 | 18 | + | 3101571 | 3102902 | 174 | annot_exons |
| novel_circ_009279 | ENSSSCG00000010569 | 14 | - | 1.13E+08 | 1.13E+08 | 849 | annot_exons |
| novel_circ_009280 | ENSSSCG00000000458 | 5 | + | 27184128 | 27193623 | 740 | annot_exons |
| novel_circ_009281 | ENSSSCG00000040494 | 5 | + | 8925251 | 8926054 | 305 | annot_exons |
| novel_circ_009282 | ENSSSCG00000002374 | 7 | + | 98105737 | 98108568 | 457 | annot_exons |
| novel_circ_009283 | ENSSSCG00000016568 | 18 | - | 19302353 | 19303003 | 245 | annot_exons |
| novel_circ_009284 | ENSSSCG00000028979 | 9 | + | 10212402 | 10258500 | 633 | annot_exons |
| novel_circ_009285 | ENSSSCG00000001056 | 7 | - | 9995687 | 10010669 | 651 | annot_exons |
| novel_circ_009286 | ENSSSCG00000010928 | 10 | - | 24760509 | 24781769 | 1405 | annot_exons |
| novel_circ_009287 | ENSSSCG00000023307 | 16 | - | 52333339 | 52333667 | 329 | antisense |
| novel_circ_009288 | ENSSSCG00000003461 | 6 | + | 74817822 | 74829237 | 3459 | annot_exons |
| novel_circ_009289 | ENSSSCG00000017101 | 16 | - | 74418373 | 74428142 | 426 | annot_exons |
| novel_circ_009290 | ENSSSCG00000003627 | 6 | + | 91566041 | 91575552 | 670 | annot_exons |
| novel_circ_009291 | ENSSSCG00000008844 | 8 | - | 41821502 | 41821857 | 235 | annot_exons |
| novel_circ_009292 | ENSSSCG00000003512 | 6 | - | 79172204 | 79180903 | 516 | annot_exons |
| novel_circ_009293 | ENSSSCG00000030480 | 13 | + | 2.01E+08 | 2.01E+08 | 857 | annot_exons |
| novel_circ_009294 | ENSSSCG00000005426 | 1 | + | 2.47E+08 | 2.47E+08 | 264 | annot_exons |
| novel_circ_009295 | NA | 7 | - | 32614984 | 32615795 | 812 | intergenic |
| novel_circ_009296 | ENSSSCG00000040139 | 6 | - | 27792227 | 27792392 | 166 | one_exon |
| novel_circ_009297 | ENSSSCG00000008943 | 8 | + | 68150867 | 68152269 | 252 | annot_exons |
| novel_circ_009298 | ENSSSCG00000001975 | 7 | + | 69716529 | 69717922 | 1394 | exon_intron |
| novel_circ_009299 | ENSSSCG00000004524 | 1 | + | 1.01E+08 | 1.01E+08 | 418 | annot_exons |
| novel_circ_009300 | ENSSSCG00000002032 | 7 | + | 75876558 | 75878458 | 357 | annot_exons |
| novel_circ_009301 | ENSSSCG00000011233 | 13 | - | 18526605 | 18529971 | 518 | annot_exons |
| novel_circ_009302 | ENSSSCG00000005743 | 1 | - | 2.73E+08 | 2.73E+08 | 387 | annot_exons |
| novel_circ_009303 | ENSSSCG00000013630 | 2 | + | 69538354 | 69565336 | 1306 | annot_exons |
| novel_circ_009304 | ENSSSCG00000003743 | 6 | + | 1.19E+08 | 1.19E+08 | 472 | annot_exons |
| novel_circ_009305 | ENSSSCG00000022830 | 15 | - | 1.13E+08 | 1.13E+08 | 320 | annot_exons |
| novel_circ_009306 | ENSSSCG00000012699 | X | + | 1.11E+08 | 1.11E+08 | 987 | annot_exons |
| novel_circ_009307 | ENSSSCG00000010811 | 10 | + | 5777659 | 5806269 | 839 | annot_exons |
| novel_circ_009308 | ENSSSCG00000035581 | 18 | - | 54227235 | 54266233 | 350 | annot_exons |
| novel_circ_009309 | ENSSSCG00000004432 | 1 | - | 79882324 | 79886038 | 356 | annot_exons |
| novel_circ_009310 | ENSSSCG00000004121 | 1 | + | 19842578 | 19882686 | 417 | annot_exons |
| novel_circ_009311 | ENSSSCG00000034290 | 7 | - | 94161418 | 94176980 | 576 | annot_exons |
| novel_circ_009312 | NA | 10 | + | 15175030 | 15179109 | 4080 | intergenic |
| novel_circ_009313 | ENSSSCG00000004207 | 1 | - | 34205898 | 34213181 | 284 | annot_exons |
| novel_circ_009314 | ENSSSCG00000000466 | 5 | + | 28850613 | 28858296 | 1049 | annot_exons |
| novel_circ_009315 | ENSSSCG00000009435 | 11 | - | 25563087 | 25584052 | 639 | annot_exons |
| novel_circ_009316 | ENSSSCG00000039580 | 5 | + | 63069068 | 63069414 | 347 | one_exon |
| novel_circ_009317 | ENSSSCG00000000536 | 5 | + | 44342290 | 44347400 | 296 | annot_exons |
| novel_circ_009318 | ENSSSCG00000027613 | 1 | + | 39428343 | 39428858 | 516 | intronic |
| novel_circ_009319 | ENSSSCG00000012403 | X | + | 57525285 | 57531143 | 397 | annot_exons |
| novel_circ_009320 | ENSSSCG00000039009 | 1 | - | 2.3E+08 | 2.3E+08 | 472 | annot_exons |
| novel_circ_009321 | ENSSSCG00000008682 | 8 | + | 986798 | 992003 | 471 | annot_exons |
| novel_circ_009322 | ENSSSCG00000014792 | 9 | - | 6083176 | 6088259 | 356 | annot_exons |
| novel_circ_009323 | ENSSSCG00000047188 | 11 | + | 13246347 | 13262673 | 362 | annot_exons |
| novel_circ_009324 | ENSSSCG00000006239 | 4 | - | 74433795 | 74434458 | 664 | intronic |
| novel_circ_009325 | NA | 9 | + | 1.22E+08 | 1.22E+08 | 2975 | intergenic |
| novel_circ_009326 | ENSSSCG00000022636 | 5 | + | 42853124 | 42860919 | 517 | annot_exons |
| novel_circ_009327 | ENSSSCG00000011198 | 13 | - | 3503213 | 3514690 | 320 | annot_exons |
| novel_circ_009328 | ENSSSCG00000017094 | 16 | - | 72086154 | 72105944 | 809 | annot_exons |
| novel_circ_009329 | ENSSSCG00000012250 | X | - | 36398908 | 36409625 | 1142 | annot_exons |
| novel_circ_009330 | ENSSSCG00000002266 | 7 | - | 86055233 | 86058691 | 498 | annot_exons |
| novel_circ_009331 | ENSSSCG00000049158 | 1 | + | 14023435 | 14024828 | 572 | exon_intron |
| novel_circ_009332 | ENSSSCG00000017877 | 12 | - | 50207857 | 50213931 | 410 | annot_exons |
| novel_circ_009333 | ENSSSCG00000017835 | 12 | - | 48753502 | 48755352 | 375 | annot_exons |
| novel_circ_009334 | ENSSSCG00000004919 | 1 | - | 1.62E+08 | 1.62E+08 | 246 | annot_exons |
| novel_circ_009335 | ENSSSCG00000029578 | 6 | + | 28711467 | 28737118 | 1498 | annot_exons |
| novel_circ_009336 | ENSSSCG00000017063 | 16 | - | 66830975 | 66844232 | 13258 | intronic |
| novel_circ_009337 | NA | 6 | + | 47372669 | 47375741 | 3073 | intergenic |
| novel_circ_009338 | ENSSSCG00000005009 | 1 | + | 1.8E+08 | 1.8E+08 | 314 | annot_exons |
| novel_circ_009339 | ENSSSCG00000004844 | 1 | + | 1.44E+08 | 1.44E+08 | 353 | annot_exons |
| novel_circ_009340 | ENSSSCG00000005934 | 4 | + | 3131460 | 3137676 | 361 | annot_exons |
| novel_circ_009341 | ENSSSCG00000008854 | 8 | - | 43600697 | 43602937 | 365 | annot_exons |
| novel_circ_009342 | ENSSSCG00000005300 | 1 | - | 2.36E+08 | 2.36E+08 | 414 | annot_exons |
| novel_circ_009343 | ENSSSCG00000029837 | 11 | + | 25328680 | 25343890 | 483 | annot_exons |
| novel_circ_009344 | ENSSSCG00000030420 | 6 | + | 13123284 | 13136177 | 774 | annot_exons |
| novel_circ_009345 | ENSSSCG00000016769 | 18 | + | 54312446 | 54312622 | 177 | antisense |
| novel_circ_009346 | ENSSSCG00000021041 | 9 | - | 66523998 | 66541260 | 678 | annot_exons |
| novel_circ_009347 | ENSSSCG00000032622 | 14 | + | 6745390 | 6766696 | 894 | annot_exons |
| novel_circ_009348 | ENSSSCG00000031382 | 1 | - | 2.28E+08 | 2.28E+08 | 376 | annot_exons |
| novel_circ_009349 | ENSSSCG00000033334 | 15 | + | 1.32E+08 | 1.32E+08 | 278 | annot_exons |
| novel_circ_009350 | ENSSSCG00000006779 | 4 | + | 1.08E+08 | 1.08E+08 | 774 | annot_exons |
| novel_circ_009351 | ENSSSCG00000028840 | 9 | + | 1.09E+08 | 1.09E+08 | 549 | annot_exons |
| novel_circ_009352 | ENSSSCG00000027342 | 3 | - | 10814283 | 10816987 | 320 | annot_exons |
| novel_circ_009353 | ENSSSCG00000012251 | X | + | 36873570 | 36876572 | 316 | annot_exons |
| novel_circ_009354 | ENSSSCG00000032531 | 3 | + | 26691921 | 26697117 | 497 | annot_exons |
| novel_circ_009355 | ENSSSCG00000033323 | 14 | - | 92296680 | 92296896 | 217 | one_exon |
| novel_circ_009356 | ENSSSCG00000005249 | 1 | + | 2.23E+08 | 2.23E+08 | 1871 | exon_intron |
| novel_circ_009357 | ENSSSCG00000006231 | 4 | - | 72576154 | 72581516 | 807 | annot_exons |
| novel_circ_009358 | ENSSSCG00000004576 | 1 | - | 1.11E+08 | 1.11E+08 | 1960 | antisense |
| novel_circ_009359 | ENSSSCG00000010801 | 10 | - | 585594 | 591729 | 351 | annot_exons |
| novel_circ_009360 | ENSSSCG00000035673 | 9 | + | 1.07E+08 | 1.07E+08 | 1397 | annot_exons |
| novel_circ_009361 | ENSSSCG00000003872 | 6 | + | 1.61E+08 | 1.61E+08 | 3443 | annot_exons |
| novel_circ_009362 | ENSSSCG00000008535 | 3 | - | 1.1E+08 | 1.1E+08 | 1411 | annot_exons |
| novel_circ_009363 | ENSSSCG00000000078 | 5 | - | 8160308 | 8162357 | 2050 | intronic |
| novel_circ_009364 | NA | 7 | + | 68179437 | 68204994 | 25558 | intergenic |
| novel_circ_009365 | ENSSSCG00000011085 | 10 | - | 53169687 | 53227401 | 551 | annot_exons |
| novel_circ_009366 | ENSSSCG00000006487 | 4 | + | 93738637 | 93745088 | 402 | annot_exons |
| novel_circ_009367 | ENSSSCG00000009047 | 8 | - | 84188605 | 84189357 | 332 | annot_exons |
| novel_circ_009368 | ENSSSCG00000011192 | 13 | - | 2810084 | 2832193 | 643 | annot_exons |
| novel_circ_009369 | ENSSSCG00000006071 | 4 | - | 37491558 | 37503331 | 363 | annot_exons |
| novel_circ_009370 | ENSSSCG00000003917 | 6 | + | 1.66E+08 | 1.66E+08 | 315 | one_exon |
| novel_circ_009371 | ENSSSCG00000011106 | 10 | + | 57862461 | 57866461 | 388 | annot_exons |
| novel_circ_009372 | ENSSSCG00000010470 | 14 | - | 1.04E+08 | 1.04E+08 | 1035 | annot_exons |
| novel_circ_009373 | ENSSSCG00000003673 | 6 | - | 96718173 | 96719247 | 240 | annot_exons |
| novel_circ_009374 | ENSSSCG00000012328 | X | - | 46307819 | 46308305 | 303 | annot_exons |
| novel_circ_009375 | ENSSSCG00000040184 | 11 | + | 48096771 | 48097079 | 309 | one_exon |
| novel_circ_009376 | ENSSSCG00000037238 | 12 | - | 18606802 | 18611552 | 351 | annot_exons |
| novel_circ_009377 | ENSSSCG00000006324 | 4 | + | 85032415 | 85040050 | 411 | annot_exons |
| novel_circ_009378 | ENSSSCG00000001970 | 7 | - | 68257577 | 68262211 | 4635 | antisense |
| novel_circ_009379 | ENSSSCG00000011873 | 13 | + | 1.38E+08 | 1.38E+08 | 368 | annot_exons |
| novel_circ_009380 | ENSSSCG00000016045 | 15 | + | 94387078 | 94446961 | 1909 | annot_exons |
| novel_circ_009381 | ENSSSCG00000035686 | 5 | - | 21672280 | 21672613 | 232 | annot_exons |
| novel_circ_009382 | ENSSSCG00000007668 | 3 | - | 8588462 | 8589011 | 325 | annot_exons |
| novel_circ_009383 | ENSSSCG00000002508 | 7 | - | 1.2E+08 | 1.2E+08 | 682 | annot_exons |
| novel_circ_009384 | ENSSSCG00000003596 | 6 | - | 87872214 | 87874230 | 260 | annot_exons |
| novel_circ_009385 | ENSSSCG00000012054 | 13 | + | 2E+08 | 2E+08 | 225 | annot_exons |
| novel_circ_009386 | ENSSSCG00000016248 | 15 | - | 1.29E+08 | 1.29E+08 | 2133 | antisense |
| novel_circ_009387 | ENSSSCG00000005449 | 1 | - | 2.5E+08 | 2.5E+08 | 653 | annot_exons |
| novel_circ_009388 | ENSSSCG00000006873 | 4 | + | 1.18E+08 | 1.18E+08 | 563 | annot_exons |
| novel_circ_009389 | ENSSSCG00000004528 | 1 | - | 1.04E+08 | 1.04E+08 | 1887 | exon_intron |
| novel_circ_009390 | ENSSSCG00000006179 | 4 | + | 62154577 | 62187826 | 8241 | exon_intron |
| novel_circ_009391 | ENSSSCG00000008337 | 3 | + | 72899063 | 72901158 | 337 | annot_exons |
| novel_circ_009392 | ENSSSCG00000014902 | 9 | + | 17394781 | 17406791 | 1081 | annot_exons |
| novel_circ_009393 | ENSSSCG00000025109 | 6 | + | 48660682 | 48662289 | 450 | annot_exons |
| novel_circ_009394 | ENSSSCG00000023533 | 1 | - | 1.48E+08 | 1.48E+08 | 308 | annot_exons |
| novel_circ_009395 | ENSSSCG00000011236 | 13 | - | 19324047 | 19324275 | 229 | one_exon |
| novel_circ_009396 | ENSSSCG00000016618 | 18 | - | 25798075 | 25809926 | 778 | annot_exons |
| novel_circ_009397 | ENSSSCG00000012713 | X | - | 1.14E+08 | 1.14E+08 | 528 | annot_exons |
| novel_circ_009398 | ENSSSCG00000017277 | 12 | + | 14685735 | 14690514 | 804 | annot_exons |
| novel_circ_009399 | ENSSSCG00000036569 | 4 | + | 2345568 | 2345737 | 170 | antisense |
| novel_circ_009400 | ENSSSCG00000003872 | 6 | + | 1.61E+08 | 1.61E+08 | 433 | annot_exons |
| novel_circ_009401 | ENSSSCG00000003812 | 6 | - | 1.49E+08 | 1.49E+08 | 436 | annot_exons |
| novel_circ_009402 | ENSSSCG00000017735 | 12 | - | 42750233 | 42750757 | 211 | annot_exons |
| novel_circ_009403 | ENSSSCG00000004022 | 1 | + | 2506525 | 2509260 | 412 | annot_exons |
| novel_circ_009404 | ENSSSCG00000029920 | 8 | + | 87799383 | 87866480 | 2917 | annot_exons |
| novel_circ_009405 | ENSSSCG00000024756 | 6 | - | 417780 | 428196 | 949 | annot_exons |
| novel_circ_009406 | ENSSSCG00000009142 | 8 | - | 1.13E+08 | 1.13E+08 | 490 | annot_exons |
| novel_circ_009407 | ENSSSCG00000028327 | 14 | - | 64332280 | 64335570 | 240 | annot_exons |
| novel_circ_009408 | ENSSSCG00000034569 | 2 | + | 51440668 | 51442957 | 1062 | annot_exons |
| novel_circ_009409 | ENSSSCG00000030053 | 9 | + | 45878428 | 45880176 | 551 | annot_exons |
| novel_circ_009410 | ENSSSCG00000007109 | 17 | + | 29125460 | 29128722 | 224 | annot_exons |
| novel_circ_009411 | ENSSSCG00000047420 | 17 | - | 38987688 | 38991291 | 228 | annot_exons |
| novel_circ_009412 | ENSSSCG00000014240 | 2 | + | 1.27E+08 | 1.27E+08 | 537 | annot_exons |
| novel_circ_009413 | ENSSSCG00000016845 | 16 | - | 22567969 | 22568699 | 251 | annot_exons |
| novel_circ_009414 | NA | 15 | + | 84275187 | 84275372 | 186 | intergenic |
| novel_circ_009415 | ENSSSCG00000023264 | 15 | - | 1.11E+08 | 1.11E+08 | 430 | annot_exons |
| novel_circ_009416 | ENSSSCG00000001924 | 7 | - | 60731692 | 60748628 | 323 | annot_exons |
| novel_circ_009417 | ENSSSCG00000016395 | 15 | - | 789988 | 793654 | 611 | annot_exons |
| novel_circ_009418 | ENSSSCG00000017401 | 12 | - | 20307501 | 20323786 | 1670 | annot_exons |
| novel_circ_009419 | ENSSSCG00000009134 | 8 | - | 1.12E+08 | 1.12E+08 | 784 | annot_exons |
| novel_circ_009420 | ENSSSCG00000032220 | 13 | + | 18697128 | 18701877 | 380 | annot_exons |
| novel_circ_009421 | ENSSSCG00000015238 | 9 | - | 56044142 | 56045898 | 386 | annot_exons |
| novel_circ_009422 | ENSSSCG00000021038 | 13 | - | 1.8E+08 | 1.8E+08 | 400 | annot_exons |
| novel_circ_009423 | NA | 15 | + | 84277153 | 84277775 | 623 | intergenic |
| novel_circ_009424 | ENSSSCG00000014998 | 9 | + | 35933885 | 35942709 | 356 | annot_exons |
| novel_circ_009425 | ENSSSCG00000008293 | 3 | - | 69067545 | 69067732 | 188 | one_exon |
| novel_circ_009426 | ENSSSCG00000031903 | 2 | + | 1188601 | 1189142 | 542 | intronic |
| novel_circ_009427 | ENSSSCG00000016543 | 18 | - | 15347146 | 15348948 | 1803 | exon_intron |
| novel_circ_009428 | ENSSSCG00000016845 | 16 | - | 22550953 | 22556122 | 486 | annot_exons |
| novel_circ_009429 | ENSSSCG00000007896 | 3 | + | 31385093 | 31389328 | 315 | annot_exons |
| novel_circ_009430 | ENSSSCG00000000795 | 5 | - | 74575842 | 74577090 | 285 | annot_exons |
| novel_circ_009431 | ENSSSCG00000015747 | 15 | + | 33555033 | 33561243 | 558 | annot_exons |
| novel_circ_009432 | NA | 15 | - | 84463747 | 84464000 | 254 | intergenic |
| novel_circ_009433 | ENSSSCG00000004587 | 1 | + | 1.13E+08 | 1.13E+08 | 329 | annot_exons |
| novel_circ_009434 | NA | 15 | - | 84307874 | 84307973 | 100 | intergenic |
| novel_circ_009435 | ENSSSCG00000029441 | 12 | - | 55227783 | 55228755 | 498 | annot_exons |
| novel_circ_009436 | ENSSSCG00000011114 | 10 | - | 59798568 | 59805130 | 755 | annot_exons |
| novel_circ_009437 | ENSSSCG00000014126 | 2 | + | 89323270 | 89350868 | 328 | annot_exons |
| novel_circ_009438 | ENSSSCG00000015488 | 9 | - | 1.16E+08 | 1.16E+08 | 17056 | exon_intron |
| novel_circ_009439 | ENSSSCG00000025130 | 11 | - | 46117913 | 46155680 | 683 | annot_exons |
| novel_circ_009440 | ENSSSCG00000004979 | 1 | - | 1.69E+08 | 1.69E+08 | 698 | annot_exons |
| novel_circ_009441 | ENSSSCG00000006987 | 17 | + | 5193681 | 5194748 | 1068 | intronic |
| novel_circ_009442 | ENSSSCG00000013362 | 2 | + | 40640776 | 40644331 | 358 | annot_exons |
| novel_circ_009443 | ENSSSCG00000015931 | 15 | + | 75881879 | 75888366 | 599 | annot_exons |
| novel_circ_009444 | ENSSSCG00000016938 | 16 | + | 41245256 | 41246111 | 161 | annot_exons |
| novel_circ_009445 | ENSSSCG00000032531 | 3 | + | 26713005 | 26714133 | 258 | annot_exons |
| novel_circ_009446 | ENSSSCG00000006033 | 4 | + | 29324181 | 29326839 | 339 | annot_exons |
| novel_circ_009447 | ENSSSCG00000025406 | 2 | + | 42003958 | 42012054 | 704 | annot_exons |
| novel_circ_009448 | ENSSSCG00000011933 | 13 | - | 1.49E+08 | 1.49E+08 | 757 | annot_exons |
| novel_circ_009449 | ENSSSCG00000045846 | 11 | - | 6647851 | 6651029 | 279 | annot_exons |
| novel_circ_009450 | ENSSSCG00000007340 | 17 | - | 41048462 | 41049147 | 686 | one_exon |
| novel_circ_009451 | ENSSSCG00000013233 | 2 | + | 15090472 | 15091423 | 952 | exon_intron |
| novel_circ_009452 | ENSSSCG00000000068 | 5 | - | 7338822 | 7345954 | 619 | annot_exons |
| novel_circ_009453 | ENSSSCG00000016157 | 15 | - | 1.13E+08 | 1.13E+08 | 3268 | annot_exons |
| novel_circ_009454 | ENSSSCG00000016520 | 18 | + | 11636575 | 11642613 | 460 | annot_exons |
| novel_circ_009455 | ENSSSCG00000013876 | 2 | - | 60466633 | 60468872 | 447 | annot_exons |
| novel_circ_009456 | ENSSSCG00000036418 | 3 | + | 1E+08 | 1E+08 | 458 | annot_exons |
| novel_circ_009457 | ENSSSCG00000006903 | 4 | - | 1.25E+08 | 1.25E+08 | 233 | annot_exons |
| novel_circ_009458 | ENSSSCG00000026344 | AEMK02000328.1 | + | 481686 | 482730 | 467 | annot_exons |
| novel_circ_009459 | ENSSSCG00000001862 | 7 | - | 56509179 | 56526919 | 777 | annot_exons |
| novel_circ_009460 | ENSSSCG00000021822 | 9 | + | 9103677 | 9149572 | 340 | annot_exons |
| novel_circ_009461 | ENSSSCG00000001097 | 7 | - | 19635981 | 19636149 | 169 | one_exon |
| novel_circ_009462 | ENSSSCG00000006996 | 17 | - | 5790827 | 5792792 | 299 | annot_exons |
| novel_circ_009463 | ENSSSCG00000023533 | 1 | - | 1.48E+08 | 1.48E+08 | 1833 | annot_exons |
| novel_circ_009464 | ENSSSCG00000035949 | 6 | - | 31388854 | 31427802 | 695 | annot_exons |
| novel_circ_009465 | NA | 15 | - | 84419923 | 84421411 | 1489 | intergenic |
| novel_circ_009466 | ENSSSCG00000031304 | 13 | + | 27669986 | 27676831 | 576 | annot_exons |
| novel_circ_009467 | ENSSSCG00000038437 | AEMK02000682.1 | - | 272348 | 278294 | 330 | annot_exons |
| novel_circ_009468 | ENSSSCG00000016916 | 16 | - | 35120233 | 35127635 | 666 | annot_exons |
| novel_circ_009469 | ENSSSCG00000003682 | 6 | - | 98754169 | 98756185 | 344 | annot_exons |
| novel_circ_009470 | NA | 18 | - | 14212354 | 14243823 | 31470 | intergenic |
| novel_circ_009471 | ENSSSCG00000003218 | 6 | + | 55293317 | 55295882 | 339 | annot_exons |
| novel_circ_009472 | ENSSSCG00000006840 | 4 | + | 1.11E+08 | 1.11E+08 | 171 | one_exon |
| novel_circ_009473 | ENSSSCG00000009021 | 8 | + | 78149537 | 78175633 | 409 | annot_exons |
| novel_circ_009474 | ENSSSCG00000011454 | 13 | - | 35056759 | 35101407 | 1023 | annot_exons |
| novel_circ_009475 | ENSSSCG00000008363 | 3 | - | 77389964 | 77390473 | 510 | one_exon |
| novel_circ_009476 | ENSSSCG00000005715 | 1 | + | 2.71E+08 | 2.71E+08 | 730 | annot_exons |
| novel_circ_009477 | ENSSSCG00000002266 | 7 | - | 85990604 | 85990850 | 247 | one_exon |
| novel_circ_009478 | ENSSSCG00000006219 | 4 | - | 68935650 | 68947962 | 1068 | annot_exons |
| novel_circ_009479 | ENSSSCG00000016027 | 15 | + | 91683984 | 91701447 | 891 | annot_exons |
| novel_circ_009480 | ENSSSCG00000002311 | 7 | + | 93282452 | 93296234 | 13783 | exon_intron |
| novel_circ_009481 | ENSSSCG00000017094 | 16 | - | 72049998 | 72057438 | 494 | annot_exons |
| novel_circ_009482 | ENSSSCG00000046261 | 11 | + | 7578380 | 7587679 | 662 | annot_exons |
| novel_circ_009483 | ENSSSCG00000025286 | 2 | - | 1.02E+08 | 1.02E+08 | 503 | annot_exons |
| novel_circ_009484 | ENSSSCG00000041050 | 2 | - | 6793378 | 6794056 | 679 | antisense |
| novel_circ_009485 | ENSSSCG00000011045 | 10 | + | 45992309 | 46002543 | 482 | annot_exons |
| novel_circ_009486 | ENSSSCG00000001554 | 7 | - | 31665249 | 31669318 | 826 | annot_exons |
| novel_circ_009487 | ENSSSCG00000014269 | 2 | - | 1.34E+08 | 1.34E+08 | 268 | annot_exons |
| novel_circ_009488 | ENSSSCG00000003630 | 6 | + | 92193860 | 92197354 | 432 | annot_exons |
| novel_circ_009489 | ENSSSCG00000004651 | 1 | - | 1.22E+08 | 1.23E+08 | 451 | annot_exons |
| novel_circ_009490 | ENSSSCG00000017406 | 12 | + | 20545146 | 20548566 | 295 | annot_exons |
| novel_circ_009491 | ENSSSCG00000003192 | 6 | + | 54922047 | 54922280 | 234 | antisense |
| novel_circ_009492 | ENSSSCG00000026153 | 9 | + | 36537603 | 36545443 | 765 | annot_exons |
| novel_circ_009493 | ENSSSCG00000013857 | 2 | + | 61207155 | 61209996 | 312 | annot_exons |
| novel_circ_009494 | ENSSSCG00000016397 | 15 | + | 662410 | 683794 | 2915 | annot_exons |
| novel_circ_009495 | ENSSSCG00000004997 | 1 | + | 1.75E+08 | 1.75E+08 | 292 | annot_exons |
| novel_circ_009496 | ENSSSCG00000028124 | AEMK02000602.1 | - | 109451 | 110148 | 292 | annot_exons |
| novel_circ_009497 | ENSSSCG00000029518 | 6 | + | 1.08E+08 | 1.08E+08 | 358 | annot_exons |
| novel_circ_009498 | ENSSSCG00000017139 | 12 | - | 1733693 | 1752772 | 1410 | annot_exons |
| novel_circ_009499 | ENSSSCG00000003682 | 6 | - | 98742427 | 98766305 | 708 | annot_exons |
| novel_circ_009500 | ENSSSCG00000040642 | 12 | - | 23240963 | 23259162 | 288 | annot_exons |
| novel_circ_009501 | ENSSSCG00000040657 | 7 | + | 68532381 | 68533985 | 272 | annot_exons |
| novel_circ_009502 | ENSSSCG00000010290 | 14 | + | 75678941 | 75702683 | 346 | annot_exons |
| novel_circ_009503 | ENSSSCG00000010235 | 14 | + | 71095689 | 71097952 | 395 | annot_exons |
| novel_circ_009504 | ENSSSCG00000004351 | 1 | - | 66412649 | 66425204 | 483 | annot_exons |
| novel_circ_009505 | NA | 15 | + | 84257796 | 84259071 | 1276 | intergenic |
| novel_circ_009506 | ENSSSCG00000014364 | 2 | + | 1.42E+08 | 1.42E+08 | 296 | annot_exons |
| novel_circ_009507 | ENSSSCG00000015213 | 9 | + | 52729720 | 52731041 | 1322 | exon_intron |
| novel_circ_009508 | NA | 15 | - | 84325168 | 84326089 | 922 | intergenic |
| novel_circ_009509 | ENSSSCG00000024621 | 13 | + | 7052821 | 7073598 | 898 | annot_exons |
| novel_circ_009510 | ENSSSCG00000037530 | 14 | + | 1.32E+08 | 1.32E+08 | 5962 | one_exon |
| novel_circ_009511 | ENSSSCG00000038505 | 12 | + | 33965925 | 33966079 | 155 | one_exon |
| novel_circ_009512 | ENSSSCG00000030947 | 4 | + | 7142133 | 7152497 | 377 | annot_exons |
| novel_circ_009513 | ENSSSCG00000005232 | 1 | - | 2.2E+08 | 2.2E+08 | 525 | annot_exons |
| novel_circ_009514 | ENSSSCG00000009236 | 8 | - | 1.35E+08 | 1.35E+08 | 1979 | antisense |
| novel_circ_009515 | ENSSSCG00000000866 | 5 | - | 82579396 | 82585391 | 546 | annot_exons |
| novel_circ_009516 | ENSSSCG00000026087 | 10 | + | 31201782 | 31215292 | 255 | annot_exons |
| novel_circ_009517 | ENSSSCG00000047585 | 13 | + | 46314096 | 46314694 | 599 | antisense |
| novel_circ_009518 | ENSSSCG00000011199 | 13 | - | 4540501 | 4590112 | 49612 | exon_intron |
| novel_circ_009519 | ENSSSCG00000016816 | 16 | - | 19672793 | 19713057 | 1254 | annot_exons |
| novel_circ_009520 | ENSSSCG00000017694 | 12 | - | 38678062 | 38680726 | 360 | annot_exons |
| novel_circ_009521 | ENSSSCG00000028572 | 6 | + | 68518310 | 68519409 | 418 | annot_exons |
| novel_circ_009522 | ENSSSCG00000032369 | 3 | + | 17460508 | 17460724 | 217 | antisense |
| novel_circ_009523 | ENSSSCG00000011917 | 13 | + | 1.46E+08 | 1.46E+08 | 324 | annot_exons |
| novel_circ_009524 | ENSSSCG00000005249 | 1 | + | 2.23E+08 | 2.23E+08 | 362 | annot_exons |
| novel_circ_009525 | ENSSSCG00000009818 | 14 | + | 31522527 | 31527471 | 544 | annot_exons |
| novel_circ_009526 | ENSSSCG00000026996 | 11 | - | 64100226 | 64133950 | 852 | annot_exons |
| novel_circ_009527 | ENSSSCG00000006779 | 4 | + | 1.08E+08 | 1.08E+08 | 213 | annot_exons |
| novel_circ_009528 | ENSSSCG00000023434 | 13 | + | 1.01E+08 | 1.01E+08 | 41452 | exon_intron |
| novel_circ_009529 | ENSSSCG00000011858 | 13 | + | 1.35E+08 | 1.35E+08 | 1020 | annot_exons |
| novel_circ_009530 | ENSSSCG00000014022 | 2 | + | 78967472 | 78967998 | 385 | annot_exons |
| novel_circ_009531 | ENSSSCG00000004961 | 1 | - | 1.66E+08 | 1.66E+08 | 645 | annot_exons |
| novel_circ_009532 | ENSSSCG00000006137 | 4 | - | 50418357 | 50418702 | 214 | annot_exons |
| novel_circ_009533 | ENSSSCG00000036537 | 2 | - | 75212487 | 75217004 | 396 | annot_exons |
| novel_circ_009534 | ENSSSCG00000029652 | 5 | - | 15691067 | 15692660 | 1594 | antisense |
| novel_circ_009535 | ENSSSCG00000027669 | 15 | - | 1.2E+08 | 1.2E+08 | 1785 | annot_exons |
| novel_circ_009536 | ENSSSCG00000010190 | 14 | - | 60415093 | 60415637 | 545 | antisense |
| novel_circ_009537 | ENSSSCG00000034280 | 8 | - | 96810097 | 96813424 | 539 | annot_exons |
| novel_circ_009538 | ENSSSCG00000009814 | 14 | + | 31289213 | 31290986 | 318 | annot_exons |
| novel_circ_009539 | NA | 15 | - | 84463945 | 84466538 | 2594 | intergenic |
| novel_circ_009540 | ENSSSCG00000002265 | 7 | + | 86303130 | 86319194 | 16065 | exon_intron |
| novel_circ_009541 | ENSSSCG00000010190 | 14 | - | 60413808 | 60414590 | 783 | antisense |
| novel_circ_009542 | ENSSSCG00000033937 | 9 | + | 33731308 | 33735453 | 739 | annot_exons |
| novel_circ_009543 | ENSSSCG00000010638 | 14 | + | 1.23E+08 | 1.23E+08 | 23398 | exon_intron |
| novel_circ_009544 | ENSSSCG00000030222 | 14 | - | 31770513 | 31776086 | 724 | annot_exons |
| novel_circ_009545 | ENSSSCG00000008479 | 3 | + | 1.02E+08 | 1.02E+08 | 549 | annot_exons |
| novel_circ_009546 | ENSSSCG00000015859 | 15 | + | 59336611 | 59360503 | 911 | annot_exons |
| novel_circ_009547 | ENSSSCG00000015565 | 9 | + | 1.26E+08 | 1.26E+08 | 220 | one_exon |
| novel_circ_009548 | ENSSSCG00000016082 | 15 | - | 1.02E+08 | 1.02E+08 | 495 | annot_exons |
| novel_circ_009549 | NA | 15 | + | 84309797 | 84310982 | 1186 | intergenic |
| novel_circ_009550 | ENSSSCG00000011765 | 13 | + | 1.18E+08 | 1.18E+08 | 1325 | annot_exons |
| novel_circ_009551 | ENSSSCG00000000078 | 5 | - | 8236426 | 8242601 | 165 | annot_exons |
| novel_circ_009552 | ENSSSCG00000010274 | 14 | + | 73752038 | 73759656 | 431 | annot_exons |
| novel_circ_009553 | ENSSSCG00000006752 | 4 | + | 1.06E+08 | 1.06E+08 | 309 | annot_exons |
| novel_circ_009554 | ENSSSCG00000002243 | 7 | + | 80250621 | 80252396 | 393 | annot_exons |
| novel_circ_009555 | ENSSSCG00000029533 | 14 | + | 1.12E+08 | 1.12E+08 | 394 | annot_exons |
| novel_circ_009556 | ENSSSCG00000020743 | 1 | + | 2.64E+08 | 2.64E+08 | 1424 | annot_exons |
| novel_circ_009557 | ENSSSCG00000004753 | 1 | + | 1.3E+08 | 1.3E+08 | 226 | one_exon |
| novel_circ_009558 | ENSSSCG00000021784 | 8 | + | 1.15E+08 | 1.16E+08 | 651 | exon_intron |
| novel_circ_009559 | ENSSSCG00000038561 | 15 | - | 84405200 | 84407873 | 1212 | annot_exons |
| novel_circ_009560 | ENSSSCG00000017877 | 12 | - | 50282520 | 50305019 | 22500 | exon_intron |
| novel_circ_009561 | NA | 15 | - | 84269116 | 84269459 | 344 | intergenic |
| novel_circ_009562 | ENSSSCG00000022830 | 15 | - | 1.13E+08 | 1.13E+08 | 667 | annot_exons |
| novel_circ_009563 | ENSSSCG00000009061 | 8 | - | 87653808 | 87664522 | 757 | annot_exons |
| novel_circ_009564 | ENSSSCG00000028148 | X | - | 28549891 | 28571196 | 566 | annot_exons |
| novel_circ_009565 | ENSSSCG00000017672 | 12 | + | 36372064 | 36372744 | 681 | one_exon |
| novel_circ_009566 | ENSSSCG00000015743 | 15 | - | 31398277 | 31473618 | 4887 | exon_intron |
| novel_circ_009567 | ENSSSCG00000012772 | X | - | 1.24E+08 | 1.24E+08 | 361 | annot_exons |
| novel_circ_009568 | ENSSSCG00000009449 | 11 | + | 33313986 | 33348677 | 454 | annot_exons |
| novel_circ_009569 | ENSSSCG00000010470 | 14 | - | 1.04E+08 | 1.04E+08 | 325 | annot_exons |
| novel_circ_009570 | ENSSSCG00000041485 | 6 | + | 47433760 | 47434061 | 302 | antisense |
| novel_circ_009571 | ENSSSCG00000014113 | 2 | - | 88201446 | 88253793 | 1536 | annot_exons |
| novel_circ_009572 | ENSSSCG00000004979 | 1 | - | 1.7E+08 | 1.7E+08 | 1169 | annot_exons |
| novel_circ_009573 | ENSSSCG00000004074 | 1 | + | 12545815 | 12546166 | 352 | antisense |
| novel_circ_009574 | ENSSSCG00000003693 | 6 | + | 1.04E+08 | 1.04E+08 | 427 | annot_exons |
| novel_circ_009575 | ENSSSCG00000021885 | 18 | - | 31065678 | 31102892 | 396 | annot_exons |
| novel_circ_009576 | NA | 2 | - | 70501959 | 70505522 | 3564 | intergenic |
| novel_circ_009577 | ENSSSCG00000007153 | 17 | - | 32156795 | 32157958 | 428 | annot_exons |
| novel_circ_009578 | ENSSSCG00000010251 | 14 | + | 72250195 | 72255440 | 504 | annot_exons |
| novel_circ_009579 | ENSSSCG00000011645 | 13 | + | 75652554 | 75675474 | 626 | annot_exons |
| novel_circ_009580 | ENSSSCG00000007789 | 3 | - | 17693009 | 17693900 | 693 | annot_exons |
| novel_circ_009581 | ENSSSCG00000010493 | 14 | - | 1.07E+08 | 1.07E+08 | 285 | annot_exons |
| novel_circ_009582 | ENSSSCG00000016845 | 16 | - | 22571093 | 22576391 | 559 | annot_exons |
| novel_circ_009583 | ENSSSCG00000011889 | 13 | + | 1.4E+08 | 1.4E+08 | 242 | annot_exons |
| novel_circ_009584 | ENSSSCG00000034122 | 8 | + | 6120759 | 6122767 | 268 | annot_exons |
| novel_circ_009585 | ENSSSCG00000011858 | 13 | + | 1.35E+08 | 1.35E+08 | 334 | annot_exons |
| novel_circ_009586 | ENSSSCG00000009244 | 8 | + | 1.35E+08 | 1.35E+08 | 508 | annot_exons |
| novel_circ_009587 | ENSSSCG00000007733 | 3 | - | 16113887 | 16135103 | 290 | annot_exons |
| novel_circ_009588 | ENSSSCG00000015577 | 9 | - | 1.28E+08 | 1.28E+08 | 239 | antisense |
| novel_circ_009589 | ENSSSCG00000010996 | 10 | + | 33229551 | 33231237 | 212 | annot_exons |
| novel_circ_009590 | ENSSSCG00000004979 | 1 | - | 1.7E+08 | 1.7E+08 | 264 | annot_exons |
| novel_circ_009591 | ENSSSCG00000031899 | 2 | - | 76419157 | 76420030 | 263 | annot_exons |
| novel_circ_009592 | ENSSSCG00000009071 | 8 | - | 96247185 | 96251607 | 4423 | exon_intron |
| novel_circ_009593 | ENSSSCG00000022982 | 9 | + | 73094595 | 73102060 | 557 | annot_exons |
| novel_circ_009594 | ENSSSCG00000009369 | 11 | + | 14603381 | 14622572 | 407 | annot_exons |
| novel_circ_009595 | ENSSSCG00000028278 | 12 | + | 52789307 | 52789708 | 311 | annot_exons |
| novel_circ_009596 | ENSSSCG00000002425 | 7 | - | 1.1E+08 | 1.1E+08 | 752 | annot_exons |
| novel_circ_009597 | ENSSSCG00000004980 | 1 | + | 1.69E+08 | 1.69E+08 | 551 | annot_exons |
| novel_circ_009598 | ENSSSCG00000033842 | 6 | + | 12234387 | 12236356 | 338 | annot_exons |
| novel_circ_009599 | ENSSSCG00000007153 | 17 | - | 32173028 | 32188492 | 702 | annot_exons |
| novel_circ_009600 | ENSSSCG00000014921 | 9 | + | 20622403 | 20622882 | 480 | one_exon |
| novel_circ_009601 | ENSSSCG00000016853 | 16 | - | 24145032 | 24147171 | 288 | annot_exons |
| novel_circ_009602 | ENSSSCG00000015313 | 9 | - | 72161163 | 72170034 | 566 | annot_exons |
| novel_circ_009603 | ENSSSCG00000015709 | 15 | - | 19952049 | 19960548 | 426 | annot_exons |
| novel_circ_009604 | ENSSSCG00000010399 | 14 | - | 90510793 | 90515501 | 731 | annot_exons |
| novel_circ_009605 | ENSSSCG00000014119 | 2 | - | 88878455 | 88900788 | 430 | annot_exons |
| novel_circ_009606 | ENSSSCG00000002258 | 7 | - | 88262901 | 88264702 | 1767 | exon_intron |
| novel_circ_009607 | ENSSSCG00000036039 | 14 | - | 60482991 | 60504383 | 1439 | annot_exons |
| novel_circ_009608 | ENSSSCG00000011278 | 13 | + | 25900980 | 25904944 | 394 | annot_exons |
| novel_circ_009609 | ENSSSCG00000008621 | 3 | + | 1.22E+08 | 1.22E+08 | 367 | annot_exons |
| novel_circ_009610 | ENSSSCG00000016397 | 15 | + | 621441 | 623446 | 420 | annot_exons |
| novel_circ_009611 | ENSSSCG00000022989 | 4 | + | 55789994 | 55791945 | 1952 | exon_intron |
| novel_circ_009612 | ENSSSCG00000011064 | 10 | - | 48903085 | 48907143 | 443 | annot_exons |
| novel_circ_009613 | ENSSSCG00000027278 | 13 | - | 1.63E+08 | 1.63E+08 | 507 | annot_exons |
| novel_circ_009614 | ENSSSCG00000015940 | 15 | + | 76207329 | 76228805 | 686 | annot_exons |
| novel_circ_009615 | ENSSSCG00000006872 | 4 | - | 1.18E+08 | 1.18E+08 | 287 | annot_exons |
| novel_circ_009616 | ENSSSCG00000008242 | 3 | - | 59631095 | 59636902 | 560 | annot_exons |
| novel_circ_009617 | ENSSSCG00000005190 | 1 | + | 2.08E+08 | 2.08E+08 | 363 | annot_exons |
| novel_circ_009618 | ENSSSCG00000001732 | 7 | - | 43609181 | 43610692 | 228 | annot_exons |
| novel_circ_009619 | ENSSSCG00000038760 | 12 | - | 26987759 | 26988244 | 486 | one_exon |
| novel_circ_009620 | ENSSSCG00000014835 | 9 | - | 8443074 | 8452840 | 1304 | annot_exons |
| novel_circ_009621 | ENSSSCG00000036855 | 13 | + | 81783159 | 81784515 | 357 | annot_exons |
| novel_circ_009622 | ENSSSCG00000021822 | 9 | + | 9083657 | 9104799 | 21143 | exon_intron |
| novel_circ_009623 | ENSSSCG00000035934 | 1 | - | 1.82E+08 | 1.82E+08 | 364 | annot_exons |
| novel_circ_009624 | ENSSSCG00000038854 | 17 | - | 34158485 | 34158637 | 153 | one_exon |
| novel_circ_009625 | ENSSSCG00000006137 | 4 | - | 50402450 | 50413193 | 645 | annot_exons |
| novel_circ_009626 | ENSSSCG00000014040 | 2 | + | 80602222 | 80606275 | 677 | annot_exons |
| novel_circ_009627 | ENSSSCG00000016745 | 18 | + | 50709051 | 50710061 | 335 | annot_exons |
| novel_circ_009628 | NA | 14 | - | 41639879 | 41641756 | 1878 | intergenic |
| novel_circ_009629 | ENSSSCG00000032622 | 14 | + | 6763062 | 6771753 | 626 | annot_exons |
| novel_circ_009630 | ENSSSCG00000044338 | 7 | - | 58508115 | 58522781 | 552 | annot_exons |
| novel_circ_009631 | NA | 15 | - | 84246753 | 84247824 | 1072 | intergenic |
| novel_circ_009632 | NA | 6 | + | 47455049 | 47457834 | 2786 | intergenic |
| novel_circ_009633 | ENSSSCG00000004634 | 1 | - | 1.21E+08 | 1.21E+08 | 282 | annot_exons |
| novel_circ_009634 | ENSSSCG00000036948 | 10 | + | 23640531 | 23641800 | 256 | annot_exons |
| novel_circ_009635 | ENSSSCG00000026596 | 12 | - | 39000635 | 39016435 | 1724 | annot_exons |
| novel_circ_009636 | ENSSSCG00000009917 | 14 | - | 40712179 | 40713364 | 192 | annot_exons |
| novel_circ_009637 | ENSSSCG00000010702 | 14 | + | 1.32E+08 | 1.32E+08 | 437 | annot_exons |
| novel_circ_009638 | ENSSSCG00000009844 | 14 | + | 33626252 | 33626470 | 219 | antisense |
| novel_circ_009639 | ENSSSCG00000016116 | 15 | - | 1.06E+08 | 1.06E+08 | 297 | annot_exons |
| novel_circ_009640 | ENSSSCG00000015387 | 9 | - | 92303886 | 92308742 | 471 | annot_exons |
| novel_circ_009641 | ENSSSCG00000024373 | 15 | - | 1.31E+08 | 1.31E+08 | 2346 | annot_exons |
| novel_circ_009642 | ENSSSCG00000011752 | 13 | - | 1.11E+08 | 1.11E+08 | 600 | antisense |
| novel_circ_009643 | ENSSSCG00000008103 | 3 | - | 44434020 | 44438793 | 275 | annot_exons |
| novel_circ_009644 | ENSSSCG00000011045 | 10 | + | 46002429 | 46006364 | 269 | annot_exons |
| novel_circ_009645 | ENSSSCG00000004856 | 1 | - | 1.46E+08 | 1.46E+08 | 678 | one_exon |
| novel_circ_009646 | ENSSSCG00000005041 | 1 | - | 1.83E+08 | 1.83E+08 | 418 | annot_exons |
| novel_circ_009647 | ENSSSCG00000009319 | 11 | + | 5587551 | 5602241 | 973 | annot_exons |
| novel_circ_009648 | ENSSSCG00000017669 | 12 | - | 36105362 | 36110086 | 581 | annot_exons |
| novel_circ_009649 | NA | 6 | + | 47442637 | 47442978 | 342 | intergenic |
| novel_circ_009650 | ENSSSCG00000031949 | 1 | + | 1.28E+08 | 1.28E+08 | 484 | one_exon |
| novel_circ_009651 | NA | 15 | + | 84261357 | 84261745 | 389 | intergenic |
| novel_circ_009652 | ENSSSCG00000008961 | 8 | + | 70193530 | 70194427 | 308 | annot_exons |
| novel_circ_009653 | ENSSSCG00000030369 | 6 | - | 89088637 | 89088855 | 219 | one_exon |
| novel_circ_009654 | ENSSSCG00000015795 | 15 | + | 46533834 | 46534069 | 236 | antisense |
| novel_circ_009655 | ENSSSCG00000036909 | 6 | + | 1.67E+08 | 1.67E+08 | 13585 | exon_intron |
| novel_circ_009656 | ENSSSCG00000014994 | 9 | - | 34461542 | 34466418 | 368 | annot_exons |
| novel_circ_009657 | ENSSSCG00000014319 | 2 | + | 1.38E+08 | 1.38E+08 | 202 | one_exon |
| novel_circ_009658 | ENSSSCG00000038452 | 1 | - | 1.39E+08 | 1.39E+08 | 512 | intronic |
| novel_circ_009659 | ENSSSCG00000007541 | 3 | - | 313709 | 319969 | 390 | annot_exons |
| novel_circ_009660 | ENSSSCG00000011615 | 13 | - | 71782276 | 71794920 | 475 | annot_exons |
| novel_circ_009661 | ENSSSCG00000006206 | 4 | - | 67923230 | 67930972 | 196 | annot_exons |
| novel_circ_009662 | ENSSSCG00000016043 | 15 | - | 94340048 | 94341891 | 311 | annot_exons |
| novel_circ_009663 | ENSSSCG00000011652 | 13 | - | 77334366 | 77342010 | 484 | annot_exons |
| novel_circ_009664 | ENSSSCG00000008097 | 3 | - | 44008062 | 44009470 | 1409 | exon_intron |
| novel_circ_009665 | NA | 15 | - | 84269109 | 84269459 | 351 | intergenic |
| novel_circ_009666 | ENSSSCG00000005170 | 1 | - | 2.04E+08 | 2.04E+08 | 380 | annot_exons |
| novel_circ_009667 | ENSSSCG00000027613 | 1 | + | 39257585 | 39277175 | 258 | annot_exons |
| novel_circ_009668 | ENSSSCG00000038886 | 7 | - | 4746340 | 4746851 | 512 | one_exon |
| novel_circ_009669 | ENSSSCG00000024168 | 8 | - | 1.15E+08 | 1.15E+08 | 248 | annot_exons |
| novel_circ_009670 | ENSSSCG00000000696 | 5 | + | 64115305 | 64115506 | 198 | exon_intron |
| novel_circ_009671 | ENSSSCG00000037507 | 7 | - | 33773170 | 33773436 | 267 | intronic |
| novel_circ_009672 | ENSSSCG00000017895 | 12 | - | 51680468 | 51703106 | 22639 | exon_intron |
| novel_circ_009673 | ENSSSCG00000011854 | 13 | + | 1.35E+08 | 1.35E+08 | 335 | annot_exons |
| novel_circ_009674 | ENSSSCG00000004405 | 1 | + | 75718076 | 75731720 | 431 | annot_exons |
| novel_circ_009675 | ENSSSCG00000000694 | 5 | - | 64129687 | 64129952 | 266 | one_exon |
| novel_circ_009676 | ENSSSCG00000002508 | 7 | - | 1.2E+08 | 1.2E+08 | 330 | annot_exons |
| novel_circ_009677 | ENSSSCG00000027293 | 16 | + | 49093476 | 49103950 | 3733 | annot_exons |
| novel_circ_009678 | ENSSSCG00000014598 | 9 | - | 2038535 | 2055979 | 333 | annot_exons |
| novel_circ_009679 | ENSSSCG00000015492 | 9 | + | 1.16E+08 | 1.16E+08 | 322 | annot_exons |
| novel_circ_009680 | ENSSSCG00000006757 | 4 | + | 1.06E+08 | 1.06E+08 | 908 | annot_exons |
| novel_circ_009681 | ENSSSCG00000010566 | 14 | - | 1.13E+08 | 1.13E+08 | 415 | annot_exons |
| novel_circ_009682 | ENSSSCG00000039324 | 12 | + | 60486294 | 60486614 | 321 | one_exon |
| novel_circ_009683 | ENSSSCG00000006328 | 4 | + | 85347056 | 85349852 | 331 | annot_exons |
| novel_circ_009684 | ENSSSCG00000039309 | 2 | - | 1.46E+08 | 1.46E+08 | 269 | annot_exons |
| novel_circ_009685 | ENSSSCG00000004082 | 1 | + | 14028894 | 14038852 | 525 | annot_exons |
| novel_circ_009686 | ENSSSCG00000011833 | 13 | + | 1.33E+08 | 1.33E+08 | 278 | annot_exons |
| novel_circ_009687 | ENSSSCG00000024623 | 13 | + | 1.81E+08 | 1.81E+08 | 258 | annot_exons |
| novel_circ_009688 | ENSSSCG00000002381 | 7 | - | 98298456 | 98301129 | 549 | annot_exons |
| novel_circ_009689 | ENSSSCG00000038714 | 3 | + | 51518756 | 51522890 | 1854 | exon_intron |
| novel_circ_009690 | ENSSSCG00000026180 | 3 | + | 90338816 | 90381579 | 1017 | annot_exons |
| novel_circ_009691 | ENSSSCG00000006872 | 4 | - | 1.18E+08 | 1.18E+08 | 577 | annot_exons |
| novel_circ_009692 | NA | 14 | - | 41643306 | 41643575 | 270 | intergenic |
| novel_circ_009693 | ENSSSCG00000003553 | 6 | + | 83268936 | 83275871 | 1071 | annot_exons |
| novel_circ_009694 | ENSSSCG00000016430 | 18 | - | 5300933 | 5308460 | 595 | annot_exons |
| novel_circ_009695 | ENSSSCG00000038397 | 4 | + | 2748388 | 2793098 | 672 | annot_exons |
| novel_circ_009696 | ENSSSCG00000000466 | 5 | + | 28836818 | 28844046 | 783 | annot_exons |
| novel_circ_009697 | ENSSSCG00000030359 | 13 | - | 38883502 | 38890816 | 1785 | exon_intron |
| novel_circ_009698 | ENSSSCG00000001064 | 7 | + | 12261370 | 12276255 | 460 | annot_exons |
| novel_circ_009699 | ENSSSCG00000026536 | 11 | - | 78681976 | 78703726 | 2818 | annot_exons |
| novel_circ_009700 | ENSSSCG00000016430 | 18 | - | 5300933 | 5303104 | 324 | annot_exons |
| novel_circ_009701 | NA | 15 | - | 84261373 | 84262084 | 712 | intergenic |
| novel_circ_009702 | ENSSSCG00000033692 | 14 | + | 55770332 | 55802841 | 729 | annot_exons |
| novel_circ_009703 | ENSSSCG00000039542 | 2 | - | 77377826 | 77378193 | 264 | annot_exons |
| novel_circ_009704 | ENSSSCG00000015368 | 9 | + | 87924389 | 87943328 | 305 | annot_exons |
| novel_circ_009705 | ENSSSCG00000016898 | 16 | - | 34070748 | 34090473 | 681 | annot_exons |
| novel_circ_009706 | ENSSSCG00000035479 | 10 | + | 11603222 | 11654794 | 965 | annot_exons |
| novel_circ_009707 | ENSSSCG00000032357 | 13 | + | 39108264 | 39111421 | 578 | annot_exons |
| novel_circ_009708 | ENSSSCG00000006930 | 4 | - | 1.29E+08 | 1.29E+08 | 658 | annot_exons |
| novel_circ_009709 | ENSSSCG00000006865 | 4 | - | 1.18E+08 | 1.18E+08 | 267 | annot_exons |
| novel_circ_009710 | ENSSSCG00000007606 | 3 | + | 5981927 | 5996608 | 2043 | annot_exons |
| novel_circ_009711 | ENSSSCG00000015497 | 9 | + | 1.16E+08 | 1.16E+08 | 316 | annot_exons |
| novel_circ_009712 | ENSSSCG00000014195 | 2 | + | 1.15E+08 | 1.15E+08 | 514 | annot_exons |
| novel_circ_009713 | ENSSSCG00000008225 | 3 | - | 58651892 | 58655607 | 336 | annot_exons |
| novel_circ_009714 | ENSSSCG00000038607 | 13 | - | 16954535 | 16960769 | 252 | annot_exons |
| novel_circ_009715 | ENSSSCG00000012713 | X | - | 1.14E+08 | 1.15E+08 | 39105 | exon_intron |
| novel_circ_009716 | ENSSSCG00000004425 | 1 | - | 78109183 | 78113262 | 437 | annot_exons |
| novel_circ_009717 | ENSSSCG00000034191 | 2 | + | 42552692 | 42573733 | 1277 | exon_intron |
| novel_circ_009718 | ENSSSCG00000005436 | 1 | + | 2.48E+08 | 2.48E+08 | 560 | annot_exons |
| novel_circ_009719 | ENSSSCG00000012150 | X | + | 13558768 | 13636590 | 535 | annot_exons |
| novel_circ_009720 | ENSSSCG00000005715 | 1 | + | 2.71E+08 | 2.71E+08 | 1175 | annot_exons |
| novel_circ_009721 | ENSSSCG00000028979 | 9 | + | 10247446 | 10258500 | 400 | annot_exons |
| novel_circ_009722 | ENSSSCG00000024168 | 8 | - | 1.15E+08 | 1.15E+08 | 794 | annot_exons |
| novel_circ_009723 | ENSSSCG00000007034 | 17 | + | 13177338 | 13177531 | 194 | one_exon |
| novel_circ_009724 | ENSSSCG00000002844 | 6 | - | 37024201 | 37033529 | 750 | annot_exons |
| novel_circ_009725 | ENSSSCG00000007004 | 17 | - | 12061716 | 12084564 | 1338 | annot_exons |
| novel_circ_009726 | ENSSSCG00000001994 | 7 | + | 75058530 | 75062170 | 3252 | annot_exons |
| novel_circ_009727 | ENSSSCG00000003111 | 6 | + | 52977944 | 52992912 | 523 | annot_exons |
| novel_circ_009728 | ENSSSCG00000016747 | 18 | + | 50771848 | 50773498 | 1651 | exon_intron |
| novel_circ_009729 | ENSSSCG00000008748 | 8 | - | 12907135 | 12920387 | 311 | annot_exons |
| novel_circ_009730 | ENSSSCG00000010925 | 10 | + | 24507191 | 24509572 | 2382 | exon_intron |
| novel_circ_009731 | ENSSSCG00000009397 | 11 | + | 18350469 | 18355949 | 307 | annot_exons |
| novel_circ_009732 | ENSSSCG00000007514 | 17 | + | 58635283 | 58643708 | 1010 | annot_exons |
| novel_circ_009733 | ENSSSCG00000001850 | 7 | - | 55469774 | 55471587 | 520 | annot_exons |
| novel_circ_009734 | ENSSSCG00000009963 | 14 | - | 44129749 | 44134022 | 250 | annot_exons |
| novel_circ_009735 | ENSSSCG00000006930 | 4 | - | 1.29E+08 | 1.29E+08 | 498 | annot_exons |
| novel_circ_009736 | ENSSSCG00000008909 | 8 | - | 54804170 | 54806366 | 569 | annot_exons |
| novel_circ_009737 | ENSSSCG00000004958 | 1 | + | 1.66E+08 | 1.66E+08 | 419 | annot_exons |
| novel_circ_009738 | ENSSSCG00000022955 | 8 | + | 56252493 | 56253933 | 504 | annot_exons |
| novel_circ_009739 | ENSSSCG00000031903 | 2 | - | 1299355 | 1316463 | 17109 | antisense |
| novel_circ_009740 | ENSSSCG00000006911 | 4 | + | 1.25E+08 | 1.25E+08 | 421 | annot_exons |
| novel_circ_009741 | ENSSSCG00000015402 | 9 | + | 98070810 | 98126619 | 483 | annot_exons |
| novel_circ_009742 | ENSSSCG00000015056 | 9 | + | 41982369 | 41983179 | 190 | annot_exons |
| novel_circ_009743 | ENSSSCG00000030386 | 18 | - | 13764759 | 13765569 | 389 | annot_exons |
| novel_circ_009744 | ENSSSCG00000015052 | 9 | - | 41453661 | 41454859 | 266 | annot_exons |
| novel_circ_009745 | ENSSSCG00000008600 | 3 | + | 1.18E+08 | 1.18E+08 | 11027 | exon_intron |
| novel_circ_009746 | ENSSSCG00000032968 | 14 | + | 1.23E+08 | 1.23E+08 | 271 | intronic |
| novel_circ_009747 | ENSSSCG00000004454 | 1 | + | 82875117 | 82878252 | 238 | annot_exons |
| novel_circ_009748 | ENSSSCG00000015362 | 9 | + | 86021853 | 86025117 | 477 | annot_exons |
| novel_circ_009749 | ENSSSCG00000013276 | 2 | - | 17225982 | 17227416 | 487 | annot_exons |
| novel_circ_009750 | ENSSSCG00000025478 | 6 | + | 1.07E+08 | 1.07E+08 | 691 | annot_exons |
| novel_circ_009751 | ENSSSCG00000039658 | 10 | + | 46980932 | 46984113 | 3182 | exon_intron |
| novel_circ_009752 | ENSSSCG00000008870 | 8 | + | 44560554 | 44562777 | 242 | annot_exons |
| novel_circ_009753 | ENSSSCG00000001092 | 7 | - | 19493174 | 19498333 | 556 | annot_exons |
| novel_circ_009754 | ENSSSCG00000027608 | 2 | - | 1.01E+08 | 1.01E+08 | 57638 | exon_intron |
| novel_circ_009755 | ENSSSCG00000015867 | 15 | - | 60405553 | 60414442 | 1032 | annot_exons |
| novel_circ_009756 | ENSSSCG00000009007 | 8 | - | 75509863 | 75517258 | 454 | annot_exons |
| novel_circ_009757 | ENSSSCG00000038437 | AEMK02000682.1 | - | 315438 | 318832 | 489 | annot_exons |
| novel_circ_009758 | ENSSSCG00000034139 | 1 | - | 30317624 | 30326130 | 618 | annot_exons |
| novel_circ_009759 | ENSSSCG00000003693 | 6 | + | 1.04E+08 | 1.04E+08 | 607 | annot_exons |
| novel_circ_009760 | ENSSSCG00000006169 | 4 | - | 59548289 | 59552301 | 3061 | annot_exons |
| novel_circ_009761 | ENSSSCG00000030560 | 1 | - | 1.37E+08 | 1.37E+08 | 612 | annot_exons |
| novel_circ_009762 | ENSSSCG00000027550 | 13 | - | 22853981 | 22854350 | 229 | annot_exons |
| novel_circ_009763 | ENSSSCG00000014113 | 2 | - | 88201446 | 88206327 | 289 | annot_exons |
| novel_circ_009764 | ENSSSCG00000016501 | 18 | + | 9624147 | 9626706 | 490 | annot_exons |
| novel_circ_009765 | ENSSSCG00000006467 | 4 | - | 93308380 | 93311203 | 615 | annot_exons |
| novel_circ_009766 | ENSSSCG00000008498 | 3 | + | 1.03E+08 | 1.03E+08 | 434 | annot_exons |
| novel_circ_009767 | ENSSSCG00000035824 | 13 | - | 34802407 | 34816728 | 516 | annot_exons |
| novel_circ_009768 | ENSSSCG00000026753 | 13 | + | 1.35E+08 | 1.35E+08 | 433 | annot_exons |
| novel_circ_009769 | ENSSSCG00000006179 | 4 | + | 62178830 | 62187826 | 296 | annot_exons |
| novel_circ_009770 | ENSSSCG00000008451 | 3 | - | 96249368 | 96249919 | 185 | annot_exons |
| novel_circ_009771 | ENSSSCG00000009569 | 11 | - | 552302 | 562934 | 595 | annot_exons |
| novel_circ_009772 | ENSSSCG00000022957 | 11 | + | 376042 | 384347 | 813 | annot_exons |
| novel_circ_009773 | ENSSSCG00000005208 | 1 | - | 2.16E+08 | 2.16E+08 | 388 | annot_exons |
| novel_circ_009774 | ENSSSCG00000010358 | 14 | + | 87682482 | 87699150 | 533 | annot_exons |
| novel_circ_009775 | ENSSSCG00000027741 | 6 | + | 57007809 | 57008959 | 1151 | exon_intron |
| novel_circ_009776 | ENSSSCG00000000094 | 5 | + | 9279888 | 9284306 | 293 | annot_exons |
| novel_circ_009777 | ENSSSCG00000011881 | 13 | + | 1.39E+08 | 1.39E+08 | 404 | annot_exons |
| novel_circ_009778 | ENSSSCG00000039085 | 13 | + | 2.07E+08 | 2.07E+08 | 218 | annot_exons |
| novel_circ_009779 | ENSSSCG00000000755 | 5 | + | 68397012 | 68426856 | 293 | annot_exons |
| novel_circ_009780 | ENSSSCG00000016430 | 18 | - | 5314027 | 5316439 | 1844 | annot_exons |
| novel_circ_009781 | ENSSSCG00000012818 | X | - | 1.25E+08 | 1.25E+08 | 460 | annot_exons |
| novel_circ_009782 | ENSSSCG00000017601 | 12 | + | 31219376 | 31223236 | 495 | annot_exons |
| novel_circ_009783 | ENSSSCG00000013235 | 2 | - | 15293721 | 15295047 | 344 | annot_exons |
| novel_circ_009784 | NA | 9 | + | 1.22E+08 | 1.22E+08 | 6226 | intergenic |
| novel_circ_009785 | ENSSSCG00000020858 | 7 | - | 13533473 | 13535825 | 326 | annot_exons |
| novel_circ_009786 | ENSSSCG00000016976 | 16 | - | 48824712 | 48849496 | 23489 | exon_intron |
| novel_circ_009787 | ENSSSCG00000004717 | 1 | - | 1.28E+08 | 1.28E+08 | 571 | antisense |
| novel_circ_009788 | ENSSSCG00000022317 | 12 | + | 1492107 | 1494267 | 505 | annot_exons |
| novel_circ_009789 | ENSSSCG00000011557 | 13 | + | 66134702 | 66144903 | 10202 | antisense |
| novel_circ_009790 | ENSSSCG00000016272 | 15 | + | 1.32E+08 | 1.32E+08 | 497 | annot_exons |
| novel_circ_009791 | ENSSSCG00000002142 | 7 | + | 78493469 | 78495461 | 534 | annot_exons |
| novel_circ_009792 | ENSSSCG00000005122 | 1 | - | 1.96E+08 | 1.96E+08 | 273 | annot_exons |
| novel_circ_009793 | ENSSSCG00000011511 | 13 | - | 50549472 | 50549753 | 282 | one_exon |
| novel_circ_009794 | ENSSSCG00000004092 | 1 | - | 15310676 | 15313904 | 366 | annot_exons |
| novel_circ_009795 | ENSSSCG00000010304 | 14 | - | 76450226 | 76451547 | 270 | annot_exons |
| novel_circ_009796 | ENSSSCG00000017310 | 12 | + | 16953323 | 16954694 | 1372 | one_exon |
| novel_circ_009797 | ENSSSCG00000005582 | 1 | - | 2.64E+08 | 2.64E+08 | 499 | annot_exons |
| novel_circ_009798 | NA | 1 | - | 1.26E+08 | 1.26E+08 | 8787 | intergenic |
| novel_circ_009799 | ENSSSCG00000034704 | 1 | - | 1.62E+08 | 1.62E+08 | 622 | annot_exons |
| novel_circ_009800 | ENSSSCG00000016763 | 18 | + | 52417988 | 52495626 | 955 | annot_exons |
| novel_circ_009801 | ENSSSCG00000003805 | 6 | - | 1.46E+08 | 1.46E+08 | 305 | intronic |
| novel_circ_009802 | ENSSSCG00000046165 | 18 | - | 8084933 | 8092541 | 542 | annot_exons |
| novel_circ_009803 | ENSSSCG00000014211 | 2 | + | 1.18E+08 | 1.18E+08 | 349 | annot_exons |
| novel_circ_009804 | ENSSSCG00000009403 | 11 | - | 19352012 | 19356078 | 598 | annot_exons |
| novel_circ_009805 | ENSSSCG00000016157 | 15 | - | 1.13E+08 | 1.13E+08 | 3320 | annot_exons |
| novel_circ_009806 | ENSSSCG00000004064 | 1 | - | 10175652 | 10175846 | 195 | one_exon |
| novel_circ_009807 | ENSSSCG00000035901 | 14 | + | 281357 | 287094 | 488 | annot_exons |
| novel_circ_009808 | ENSSSCG00000005060 | 1 | + | 1.85E+08 | 1.85E+08 | 993 | annot_exons |
| novel_circ_009809 | ENSSSCG00000012746 | X | - | 1.23E+08 | 1.23E+08 | 364 | antisense |
| novel_circ_009810 | ENSSSCG00000015670 | 15 | + | 8038896 | 8044280 | 308 | annot_exons |
| novel_circ_009811 | ENSSSCG00000008215 | 3 | - | 57892680 | 57898889 | 522 | annot_exons |
| novel_circ_009812 | ENSSSCG00000007142 | 17 | - | 31812189 | 31820827 | 891 | annot_exons |
| novel_circ_009813 | ENSSSCG00000011163 | 10 | + | 69026408 | 69030169 | 453 | annot_exons |
| novel_circ_009814 | ENSSSCG00000000743 | 5 | - | 67313874 | 67314092 | 219 | one_exon |
| novel_circ_009815 | ENSSSCG00000040366 | 1 | - | 2.04E+08 | 2.04E+08 | 543 | annot_exons |
| novel_circ_009816 | ENSSSCG00000016070 | 15 | - | 1.01E+08 | 1.01E+08 | 482 | annot_exons |
| novel_circ_009817 | ENSSSCG00000007331 | 17 | - | 40253732 | 40259523 | 759 | annot_exons |
| novel_circ_009818 | NA | 15 | - | 84247195 | 84248193 | 999 | intergenic |
| novel_circ_009819 | ENSSSCG00000021784 | 8 | + | 1.16E+08 | 1.16E+08 | 1467 | annot_exons |
| novel_circ_009820 | ENSSSCG00000022830 | 15 | - | 1.13E+08 | 1.13E+08 | 518 | annot_exons |
| novel_circ_009821 | NA | 4 | - | 28058743 | 28060026 | 1284 | intergenic |
| novel_circ_009822 | ENSSSCG00000008168 | 3 | + | 53042582 | 53055099 | 693 | annot_exons |
| novel_circ_009823 | ENSSSCG00000045659 | 16 | + | 45570683 | 45604874 | 33807 | exon_intron |
| novel_circ_009824 | ENSSSCG00000011068 | 10 | - | 48841821 | 48849331 | 295 | annot_exons |
| novel_circ_009825 | ENSSSCG00000011236 | 13 | - | 19411774 | 19425923 | 458 | annot_exons |
| novel_circ_009826 | ENSSSCG00000034714 | 16 | + | 4088994 | 4091913 | 500 | annot_exons |
| novel_circ_009827 | ENSSSCG00000039701 | 7 | - | 92728724 | 92781367 | 679 | annot_exons |
| novel_circ_009828 | ENSSSCG00000011811 | 13 | + | 1.26E+08 | 1.26E+08 | 265 | annot_exons |
| novel_circ_009829 | ENSSSCG00000035658 | 11 | - | 15711288 | 15728105 | 2071 | exon_intron |
| novel_circ_009830 | ENSSSCG00000002266 | 7 | - | 86024175 | 86025429 | 347 | annot_exons |
| novel_circ_009831 | ENSSSCG00000015283 | 9 | - | 65077225 | 65080622 | 318 | annot_exons |
| novel_circ_009832 | ENSSSCG00000036926 | 1 | + | 2.67E+08 | 2.67E+08 | 227 | annot_exons |
| novel_circ_009833 | NA | 3 | + | 97047928 | 97056433 | 8506 | intergenic |
| novel_circ_009834 | ENSSSCG00000024235 | 12 | + | 1263901 | 1271332 | 590 | annot_exons |
| novel_circ_009835 | ENSSSCG00000004207 | 1 | - | 33884806 | 33901143 | 354 | annot_exons |
| novel_circ_009836 | ENSSSCG00000022636 | 5 | + | 42851428 | 42853242 | 265 | annot_exons |
| novel_circ_009837 | ENSSSCG00000024592 | 8 | - | 69374301 | 69374498 | 198 | one_exon |
| novel_circ_009838 | ENSSSCG00000041478 | 15 | - | 81466047 | 81483873 | 17371 | exon_intron |
| novel_circ_009839 | ENSSSCG00000007721 | 3 | + | 11781135 | 11787399 | 1812 | annot_exons |
| novel_circ_009840 | ENSSSCG00000035822 | X | - | 46832331 | 46832580 | 250 | one_exon |
| novel_circ_009841 | ENSSSCG00000008799 | 8 | + | 32692491 | 32700141 | 846 | annot_exons |
| novel_circ_009842 | ENSSSCG00000002436 | 7 | - | 1.12E+08 | 1.12E+08 | 375 | annot_exons |
| novel_circ_009843 | ENSSSCG00000038994 | 6 | + | 82442094 | 82462831 | 477 | annot_exons |
| novel_circ_009844 | ENSSSCG00000002547 | AEMK02000452.1 | + | 2189903 | 2208500 | 984 | annot_exons |
| novel_circ_009845 | ENSSSCG00000010164 | 14 | - | 57445922 | 57463544 | 747 | annot_exons |
| novel_circ_009846 | ENSSSCG00000025534 | 17 | + | 50789950 | 50791634 | 1548 | annot_exons |
| novel_circ_009847 | NA | 15 | - | 84249776 | 84250210 | 435 | intergenic |
| novel_circ_009848 | ENSSSCG00000038607 | 13 | - | 17001137 | 17025247 | 5458 | annot_exons |
| novel_circ_009849 | ENSSSCG00000006872 | 4 | - | 1.18E+08 | 1.18E+08 | 2289 | annot_exons |
| novel_circ_009850 | ENSSSCG00000014173 | 2 | + | 1.04E+08 | 1.04E+08 | 271 | annot_exons |
| novel_circ_009851 | ENSSSCG00000036201 | 16 | + | 18853042 | 18873158 | 290 | annot_exons |
| novel_circ_009852 | ENSSSCG00000004029 | 1 | - | 4822372 | 4843415 | 260 | annot_exons |
| novel_circ_009853 | ENSSSCG00000011025 | 10 | + | 42061873 | 42079536 | 17664 | intronic |
| novel_circ_009854 | ENSSSCG00000008583 | 3 | + | 1.14E+08 | 1.14E+08 | 423 | annot_exons |
| novel_circ_009855 | ENSSSCG00000006490 | 4 | + | 93804507 | 93805245 | 639 | exon_intron |
| novel_circ_009856 | ENSSSCG00000024513 | 13 | + | 30123418 | 30129943 | 833 | exon_intron |
| novel_circ_009857 | ENSSSCG00000011373 | 13 | - | 31673546 | 31683772 | 1492 | annot_exons |
| novel_circ_009858 | ENSSSCG00000031719 | 7 | + | 55786399 | 55787153 | 378 | annot_exons |
| novel_circ_009859 | ENSSSCG00000032046 | 11 | + | 71147531 | 71151608 | 440 | annot_exons |
| novel_circ_009860 | ENSSSCG00000009473 | 11 | - | 49307682 | 49309007 | 497 | annot_exons |
| novel_circ_009861 | ENSSSCG00000026644 | 2 | + | 1.37E+08 | 1.37E+08 | 607 | annot_exons |
| novel_circ_009862 | ENSSSCG00000009142 | 8 | - | 1.13E+08 | 1.13E+08 | 1325 | annot_exons |
| novel_circ_009863 | ENSSSCG00000024481 | 4 | - | 1.01E+08 | 1.01E+08 | 342 | annot_exons |
| novel_circ_009864 | ENSSSCG00000023118 | 9 | + | 85927697 | 85937522 | 564 | annot_exons |
| novel_circ_009865 | ENSSSCG00000029251 | 13 | - | 30650759 | 30652373 | 323 | annot_exons |
| novel_circ_009866 | ENSSSCG00000032083 | 6 | - | 1.58E+08 | 1.58E+08 | 344 | annot_exons |
| novel_circ_009867 | ENSSSCG00000011848 | 13 | + | 1.34E+08 | 1.34E+08 | 517 | annot_exons |
| novel_circ_009868 | ENSSSCG00000010651 | 14 | - | 1.25E+08 | 1.25E+08 | 650 | annot_exons |
| novel_circ_009869 | ENSSSCG00000040631 | 14 | + | 4122385 | 4124280 | 346 | annot_exons |
| novel_circ_009870 | ENSSSCG00000016968 | 16 | + | 47891502 | 47893035 | 386 | annot_exons |
| novel_circ_009871 | ENSSSCG00000004435 | 1 | + | 81854722 | 81857192 | 273 | annot_exons |
| novel_circ_009872 | ENSSSCG00000002799 | 6 | - | 20295439 | 20296786 | 827 | exon_intron |
| novel_circ_009873 | ENSSSCG00000015008 | 9 | - | 36769325 | 36790554 | 785 | exon_intron |
| novel_circ_009874 | ENSSSCG00000002752 | 6 | - | 15853429 | 15853925 | 497 | one_exon |
| novel_circ_009875 | ENSSSCG00000015747 | 15 | + | 33554894 | 33566123 | 825 | exon_intron |
| novel_circ_009876 | ENSSSCG00000009432 | 15 | + | 1.34E+08 | 1.34E+08 | 192 | annot_exons |
| novel_circ_009877 | ENSSSCG00000014364 | 2 | + | 1.42E+08 | 1.42E+08 | 1100 | annot_exons |
| novel_circ_009878 | ENSSSCG00000036132 | 6 | + | 51705157 | 51705763 | 607 | antisense |
| novel_circ_009879 | ENSSSCG00000028182 | 5 | + | 87271453 | 87276568 | 368 | annot_exons |
| novel_circ_009880 | ENSSSCG00000004979 | 1 | - | 1.69E+08 | 1.7E+08 | 2554 | annot_exons |
| novel_circ_009881 | ENSSSCG00000021688 | 2 | - | 40277505 | 40292505 | 834 | annot_exons |
| novel_circ_009882 | ENSSSCG00000029920 | 8 | + | 87799386 | 87820356 | 490 | annot_exons |
| novel_circ_009883 | ENSSSCG00000015244 | 9 | + | 56834108 | 56845084 | 411 | annot_exons |
| novel_circ_009884 | ENSSSCG00000026960 | 1 | + | 1.15E+08 | 1.15E+08 | 259 | annot_exons |
| novel_circ_009885 | ENSSSCG00000011679 | 13 | - | 83270062 | 83271995 | 537 | annot_exons |
| novel_circ_009886 | ENSSSCG00000017207 | 12 | - | 6097638 | 6101624 | 291 | annot_exons |
| novel_circ_009887 | ENSSSCG00000003630 | 6 | + | 92111761 | 92122107 | 10347 | exon_intron |
| novel_circ_009888 | ENSSSCG00000005015 | 1 | - | 1.8E+08 | 1.8E+08 | 513 | annot_exons |
| novel_circ_009889 | NA | 6 | - | 69261240 | 69262364 | 1125 | intergenic |
| novel_circ_009890 | ENSSSCG00000024904 | 13 | - | 1.37E+08 | 1.37E+08 | 541 | annot_exons |
| novel_circ_009891 | ENSSSCG00000015721 | 15 | + | 25200881 | 25204823 | 388 | annot_exons |
| novel_circ_009892 | ENSSSCG00000014117 | 2 | + | 88735851 | 88736054 | 204 | one_exon |
| novel_circ_009893 | ENSSSCG00000007031 | 17 | + | 11360919 | 11367099 | 251 | annot_exons |
| novel_circ_009894 | ENSSSCG00000007454 | 17 | - | 49312897 | 49313313 | 415 | annot_exons |
| novel_circ_009895 | ENSSSCG00000007109 | 17 | + | 29125250 | 29125571 | 240 | annot_exons |
| novel_circ_009896 | ENSSSCG00000010954 | 10 | + | 29079187 | 29080757 | 346 | annot_exons |
| novel_circ_009897 | ENSSSCG00000004332 | 1 | - | 58191656 | 58192463 | 313 | annot_exons |
| novel_circ_009898 | ENSSSCG00000009021 | 8 | + | 78149537 | 78199258 | 474 | annot_exons |
| novel_circ_009899 | ENSSSCG00000040257 | 18 | + | 55851216 | 55855793 | 960 | annot_exons |
| novel_circ_009900 | ENSSSCG00000002869 | 6 | + | 43870383 | 43879544 | 253 | annot_exons |
| novel_circ_009901 | ENSSSCG00000010817 | 10 | + | 8950586 | 8967563 | 270 | annot_exons |
| novel_circ_009902 | ENSSSCG00000012663 | X | - | 1.07E+08 | 1.07E+08 | 84357 | exon_intron |
| novel_circ_009903 | ENSSSCG00000009551 | 11 | + | 77449564 | 77449772 | 209 | intronic |
| novel_circ_009904 | ENSSSCG00000032556 | 3 | - | 18258604 | 18260127 | 1524 | antisense |
| novel_circ_009905 | ENSSSCG00000008635 | 3 | + | 1.26E+08 | 1.26E+08 | 270 | annot_exons |
| novel_circ_009906 | ENSSSCG00000000625 | 5 | + | 60168451 | 60168844 | 394 | one_exon |
| novel_circ_009907 | ENSSSCG00000010440 | 14 | + | 99992348 | 99994629 | 300 | annot_exons |
| novel_circ_009908 | ENSSSCG00000011332 | 13 | - | 29959012 | 29960782 | 202 | annot_exons |
| novel_circ_009909 | ENSSSCG00000039157 | 15 | + | 30013559 | 30021973 | 718 | annot_exons |
| novel_circ_009910 | ENSSSCG00000015940 | 15 | + | 76257845 | 76308992 | 1369 | annot_exons |
| novel_circ_009911 | ENSSSCG00000024813 | 12 | - | 15287153 | 15288986 | 215 | annot_exons |
| novel_circ_009912 | ENSSSCG00000001924 | 7 | - | 60723646 | 60732585 | 420 | annot_exons |
| novel_circ_009913 | ENSSSCG00000011843 | 13 | - | 1.33E+08 | 1.33E+08 | 305 | annot_exons |
| novel_circ_009914 | ENSSSCG00000024761 | 9 | - | 87018358 | 87021020 | 397 | annot_exons |
| novel_circ_009915 | ENSSSCG00000030480 | 13 | + | 2.01E+08 | 2.01E+08 | 11194 | exon_intron |
| novel_circ_009916 | ENSSSCG00000037530 | 14 | - | 1.32E+08 | 1.32E+08 | 257 | antisense |
| novel_circ_009917 | ENSSSCG00000006983 | 17 | - | 4936705 | 4945899 | 612 | annot_exons |
| novel_circ_009918 | ENSSSCG00000028148 | X | - | 28483294 | 28486118 | 423 | annot_exons |
| novel_circ_009919 | ENSSSCG00000012055 | 13 | + | 2E+08 | 2E+08 | 496 | annot_exons |
| novel_circ_009920 | ENSSSCG00000033493 | AEMK02000682.1 | - | 172050 | 176997 | 370 | annot_exons |
| novel_circ_009921 | ENSSSCG00000034484 | 6 | + | 75038901 | 75043103 | 1641 | annot_exons |
| novel_circ_009922 | ENSSSCG00000012884 | 2 | - | 4439896 | 4459058 | 714 | annot_exons |
| novel_circ_009923 | ENSSSCG00000022982 | 9 | + | 73164446 | 73172031 | 494 | annot_exons |
| novel_circ_009924 | ENSSSCG00000007744 | 3 | + | 16824498 | 16825745 | 349 | annot_exons |
| novel_circ_009925 | ENSSSCG00000013256 | 2 | + | 15838942 | 15839476 | 278 | annot_exons |
| novel_circ_009926 | ENSSSCG00000008504 | 3 | - | 1.04E+08 | 1.04E+08 | 174 | one_exon |
| novel_circ_009927 | ENSSSCG00000004522 | 1 | + | 1E+08 | 1E+08 | 588 | annot_exons |
| novel_circ_009928 | ENSSSCG00000014339 | 2 | + | 1.41E+08 | 1.41E+08 | 557 | annot_exons |
| novel_circ_009929 | ENSSSCG00000017832 | 12 | - | 48532439 | 48539716 | 400 | annot_exons |
| novel_circ_009930 | ENSSSCG00000007907 | 3 | + | 33789131 | 33791943 | 329 | annot_exons |
| novel_circ_009931 | ENSSSCG00000021761 | 10 | - | 32576447 | 32588655 | 166 | annot_exons |
| novel_circ_009932 | ENSSSCG00000021040 | 12 | - | 2230413 | 2231065 | 249 | annot_exons |
| novel_circ_009933 | ENSSSCG00000005436 | 1 | + | 2.48E+08 | 2.48E+08 | 582 | annot_exons |
| novel_circ_009934 | ENSSSCG00000004901 | 1 | + | 1.59E+08 | 1.59E+08 | 285 | annot_exons |
| novel_circ_009935 | ENSSSCG00000007454 | 17 | - | 49312897 | 49324971 | 822 | annot_exons |
| novel_circ_009936 | ENSSSCG00000010802 | 10 | + | 754493 | 761696 | 478 | annot_exons |
| novel_circ_009937 | ENSSSCG00000008137 | 3 | - | 47619650 | 47620978 | 338 | annot_exons |
| novel_circ_009938 | ENSSSCG00000010226 | 14 | - | 66663935 | 66666186 | 638 | annot_exons |
| novel_circ_009939 | ENSSSCG00000009186 | 8 | - | 1.21E+08 | 1.21E+08 | 481 | annot_exons |
| novel_circ_009940 | ENSSSCG00000002441 | 7 | + | 1.13E+08 | 1.13E+08 | 9884 | antisense |
| novel_circ_009941 | ENSSSCG00000031392 | 13 | + | 10239300 | 10332426 | 50360 | exon_intron |
| novel_circ_009942 | ENSSSCG00000024814 | X | - | 47820221 | 47826089 | 419 | annot_exons |
| novel_circ_009943 | ENSSSCG00000016005 | 15 | - | 84845471 | 84870609 | 4551 | annot_exons |
| novel_circ_009944 | ENSSSCG00000009293 | 11 | - | 2848788 | 2851028 | 511 | annot_exons |
| novel_circ_009945 | ENSSSCG00000047585 | 13 | + | 46314093 | 46314694 | 602 | antisense |
| novel_circ_009946 | ENSSSCG00000015555 | 9 | + | 1.24E+08 | 1.24E+08 | 459 | annot_exons |
| novel_circ_009947 | ENSSSCG00000024784 | 10 | - | 23565997 | 23567776 | 237 | annot_exons |
| novel_circ_009948 | ENSSSCG00000036363 | 2 | + | 59589980 | 59590434 | 263 | annot_exons |
| novel_circ_009949 | ENSSSCG00000030643 | 3 | - | 1.13E+08 | 1.13E+08 | 783 | annot_exons |
| novel_circ_009950 | ENSSSCG00000025281 | 3 | + | 26731232 | 26732627 | 717 | annot_exons |
| novel_circ_009951 | ENSSSCG00000004854 | 1 | - | 1.45E+08 | 1.45E+08 | 726 | annot_exons |
| novel_circ_009952 | ENSSSCG00000001877 | 7 | + | 58054321 | 58060785 | 378 | annot_exons |
| novel_circ_009953 | ENSSSCG00000006115 | 4 | + | 44693174 | 44711128 | 721 | annot_exons |
| novel_circ_009954 | ENSSSCG00000034954 | 2 | + | 1.37E+08 | 1.37E+08 | 270 | one_exon |
| novel_circ_009955 | ENSSSCG00000037594 | 1 | - | 66367794 | 66372342 | 389 | annot_exons |
| novel_circ_009956 | ENSSSCG00000034704 | 1 | - | 1.62E+08 | 1.62E+08 | 2363 | one_exon |
| novel_circ_009957 | ENSSSCG00000004729 | 1 | - | 1.29E+08 | 1.29E+08 | 856 | annot_exons |
| novel_circ_009958 | ENSSSCG00000005444 | 1 | - | 2.5E+08 | 2.5E+08 | 188 | annot_exons |
| novel_circ_009959 | ENSSSCG00000015689 | 15 | + | 15817690 | 15859799 | 299 | annot_exons |
| novel_circ_009960 | ENSSSCG00000029251 | 13 | - | 30678033 | 30681718 | 1368 | annot_exons |
| novel_circ_009961 | ENSSSCG00000030053 | 9 | + | 45886507 | 45890219 | 423 | annot_exons |
| novel_circ_009962 | NA | 15 | - | 84289471 | 84289724 | 254 | intergenic |
| novel_circ_009963 | ENSSSCG00000024232 | 5 | - | 45440622 | 45443134 | 121 | annot_exons |
| novel_circ_009964 | ENSSSCG00000010811 | 10 | + | 5806114 | 5809073 | 253 | annot_exons |
| novel_circ_009965 | ENSSSCG00000015960 | 15 | + | 79268340 | 79290039 | 423 | annot_exons |
| novel_circ_009966 | ENSSSCG00000032556 | 3 | + | 18256669 | 18257091 | 339 | annot_exons |
| novel_circ_009967 | ENSSSCG00000009480 | 11 | + | 51511828 | 51534900 | 19961 | exon_intron |
| novel_circ_009968 | ENSSSCG00000024623 | 13 | + | 1.81E+08 | 1.81E+08 | 512 | annot_exons |
| novel_circ_009969 | ENSSSCG00000037121 | 6 | - | 91654006 | 91673840 | 1087 | annot_exons |
| novel_circ_009970 | ENSSSCG00000014436 | 2 | + | 1.51E+08 | 1.51E+08 | 441 | annot_exons |
| novel_circ_009971 | ENSSSCG00000020796 | 12 | - | 25803228 | 25811484 | 458 | annot_exons |
| novel_circ_009972 | ENSSSCG00000013249 | 2 | + | 15720398 | 15733317 | 832 | annot_exons |
| novel_circ_009973 | ENSSSCG00000041976 | 17 | + | 20071813 | 20097430 | 25618 | antisense |
| novel_circ_009974 | ENSSSCG00000037647 | 13 | - | 82809605 | 82893436 | 527 | annot_exons |
| novel_circ_009975 | ENSSSCG00000002799 | 6 | - | 20340052 | 20340324 | 273 | one_exon |
| novel_circ_009976 | ENSSSCG00000005126 | 1 | + | 1.96E+08 | 1.96E+08 | 474 | annot_exons |
| novel_circ_009977 | ENSSSCG00000004725 | 1 | + | 1.29E+08 | 1.29E+08 | 398 | annot_exons |
| novel_circ_009978 | ENSSSCG00000021612 | 6 | - | 1.01E+08 | 1.01E+08 | 358 | annot_exons |
| novel_circ_009979 | ENSSSCG00000016874 | 16 | - | 28106178 | 28112020 | 406 | annot_exons |
| novel_circ_009980 | ENSSSCG00000002266 | 7 | - | 86058475 | 86068321 | 893 | annot_exons |
| novel_circ_009981 | ENSSSCG00000009150 | 8 | - | 1.14E+08 | 1.14E+08 | 472 | annot_exons |
| novel_circ_009982 | ENSSSCG00000014256 | 2 | - | 1.31E+08 | 1.31E+08 | 291 | annot_exons |
| novel_circ_009983 | ENSSSCG00000001950 | 7 | - | 64586117 | 64586778 | 177 | annot_exons |
| novel_circ_009984 | ENSSSCG00000005366 | 1 | + | 2.39E+08 | 2.39E+08 | 593 | annot_exons |
| novel_circ_009985 | ENSSSCG00000009464 | 11 | - | 47621690 | 47628837 | 783 | annot_exons |
| novel_circ_009986 | ENSSSCG00000026153 | 9 | + | 36531618 | 36545443 | 1323 | annot_exons |
| novel_circ_009987 | ENSSSCG00000024513 | 13 | + | 30123619 | 30129943 | 632 | annot_exons |
| novel_circ_009988 | ENSSSCG00000002755 | 6 | - | 17320031 | 17356850 | 1108 | annot_exons |
| novel_circ_009989 | ENSSSCG00000017712 | 12 | - | 39808610 | 39817016 | 463 | annot_exons |
| novel_circ_009990 | ENSSSCG00000013742 | 2 | + | 65957190 | 65957792 | 603 | antisense |
| novel_circ_009991 | ENSSSCG00000016045 | 15 | + | 94387078 | 94400911 | 438 | annot_exons |
| novel_circ_009992 | ENSSSCG00000026733 | 18 | + | 10129596 | 10129840 | 245 | one_exon |
| novel_circ_009993 | ENSSSCG00000017783 | 12 | - | 45378734 | 45378910 | 177 | one_exon |
| novel_circ_009994 | ENSSSCG00000034624 | 13 | - | 1.97E+08 | 1.97E+08 | 434 | annot_exons |
| novel_circ_009995 | ENSSSCG00000021644 | 6 | + | 34493843 | 34498255 | 333 | annot_exons |
[truncated: 367,484 more chars]
